# Supplementary figures and images for: Remote sensing image analysis and prediction based on improved Pix2Pix model for water environment protection of smart cities (part 1 of 6)
Source: PeerJ Comput Sci. 2023 Apr 26;9:e1292. doi: 10.7717/peerj-cs.1292 (PMC10280440; doi:10.7717/peerj-cs.1292)

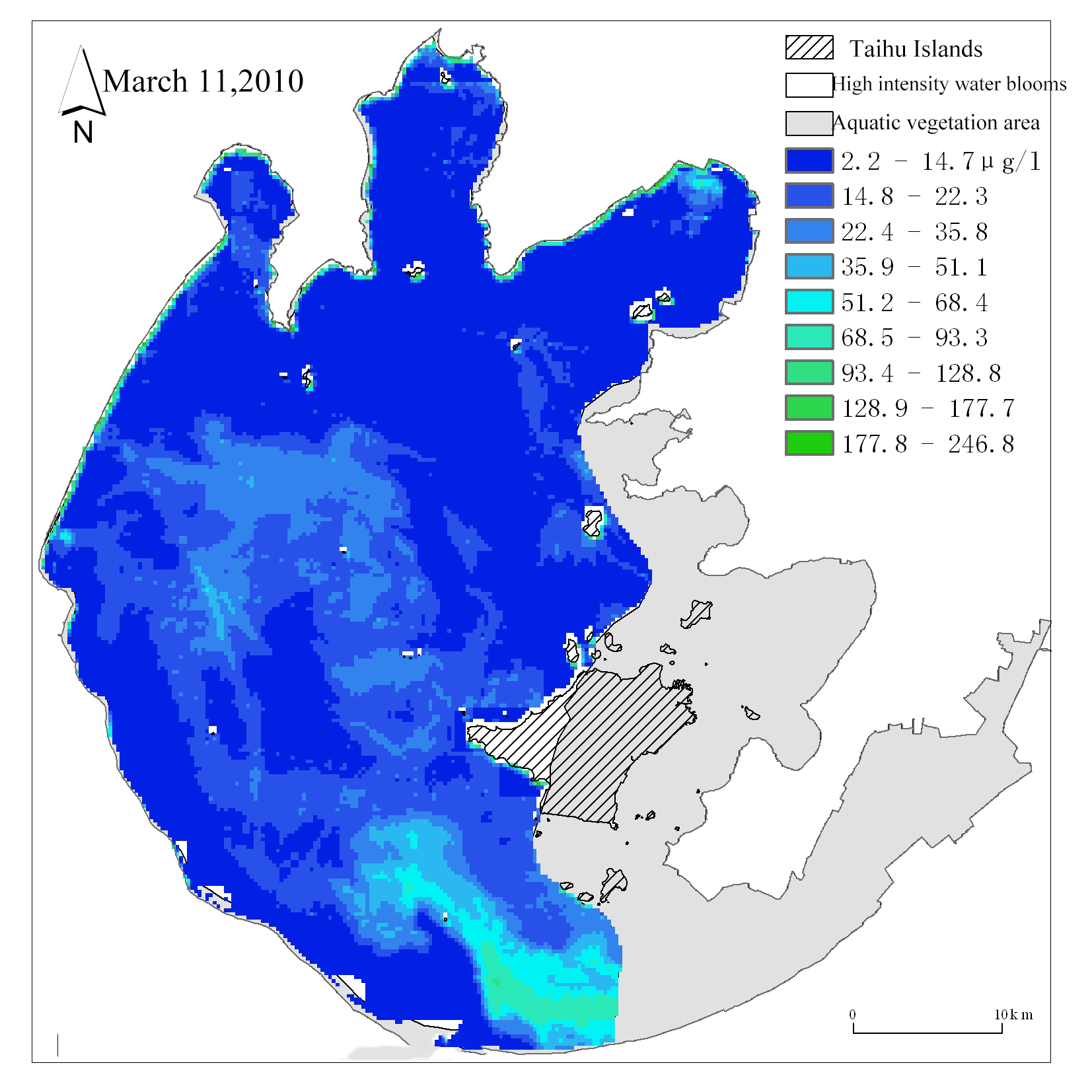

Supplement: Supplemental Information 2 — The data were obtained from the remote sensing image data of chlorophyll a concentration from the Lake-Watershed Science SubCenter, National Earth System Science Data Center, National Science & Technology Infrastructure of China, which had inconsistent data scales, data anomalies and different sampling intervals, and the chlorophyll a concentration unit was µg/L. [file peerj-cs-09-1292-s002.zip › 201003110215_taihu_chla.jpg]

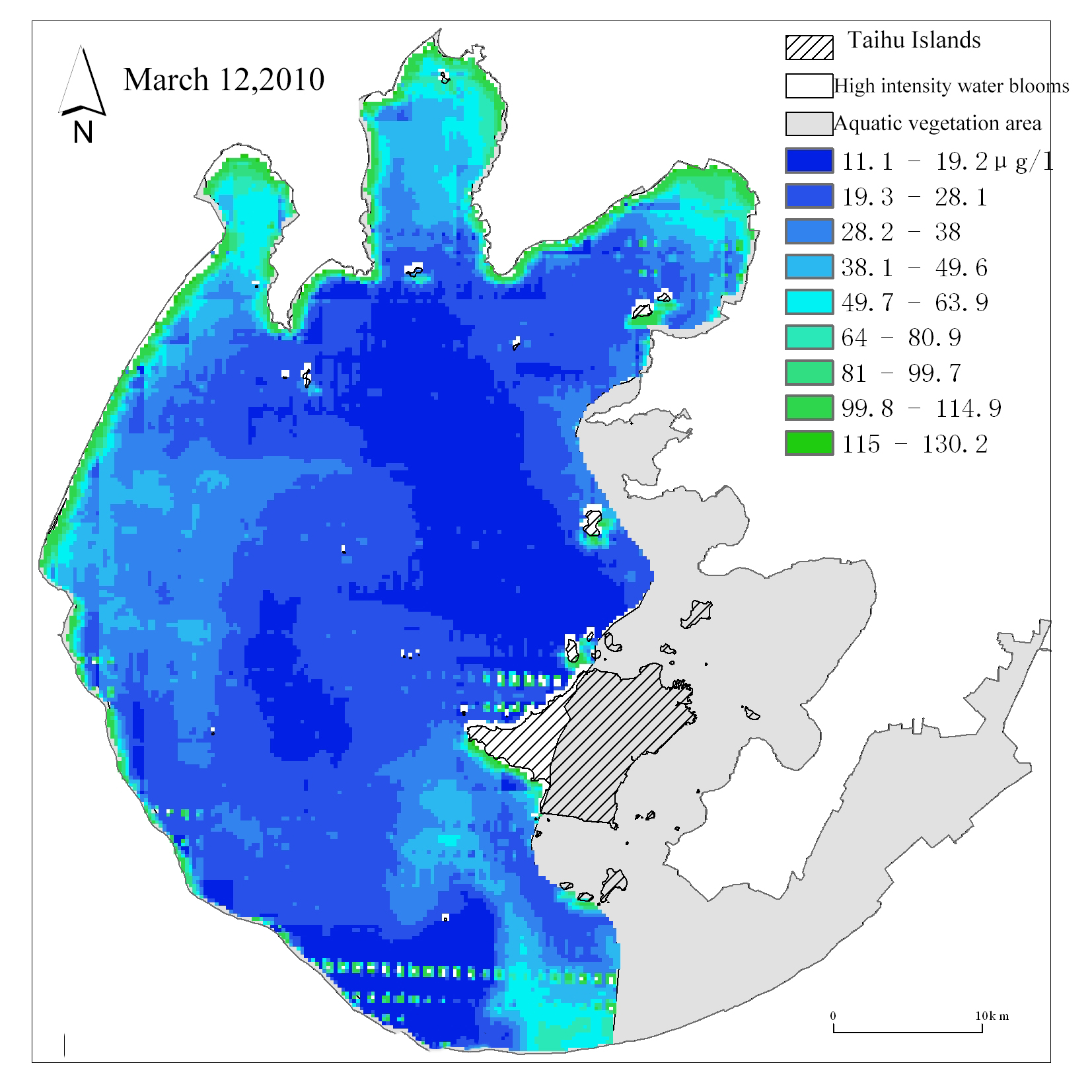

Supplement: Supplemental Information 2 — The data were obtained from the remote sensing image data of chlorophyll a concentration from the Lake-Watershed Science SubCenter, National Earth System Science Data Center, National Science & Technology Infrastructure of China, which had inconsistent data scales, data anomalies and different sampling intervals, and the chlorophyll a concentration unit was µg/L. [file peerj-cs-09-1292-s002.zip › 201003120203_taihu_chla.jpg]

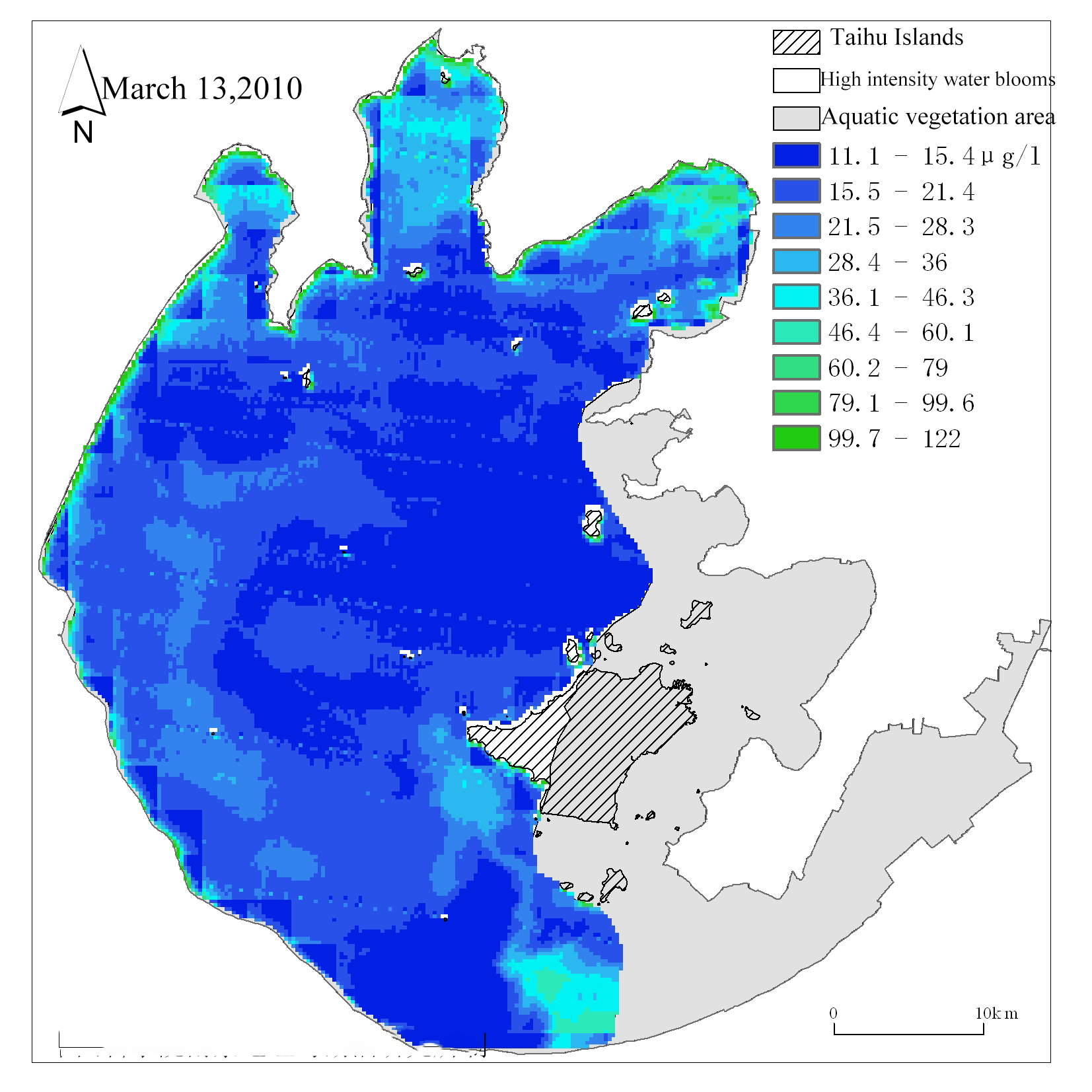

Supplement: Supplemental Information 2 — The data were obtained from the remote sensing image data of chlorophyll a concentration from the Lake-Watershed Science SubCenter, National Earth System Science Data Center, National Science & Technology Infrastructure of China, which had inconsistent data scales, data anomalies and different sampling intervals, and the chlorophyll a concentration unit was µg/L. [file peerj-cs-09-1292-s002.zip › 201003130245_taihu_chla.jpg]

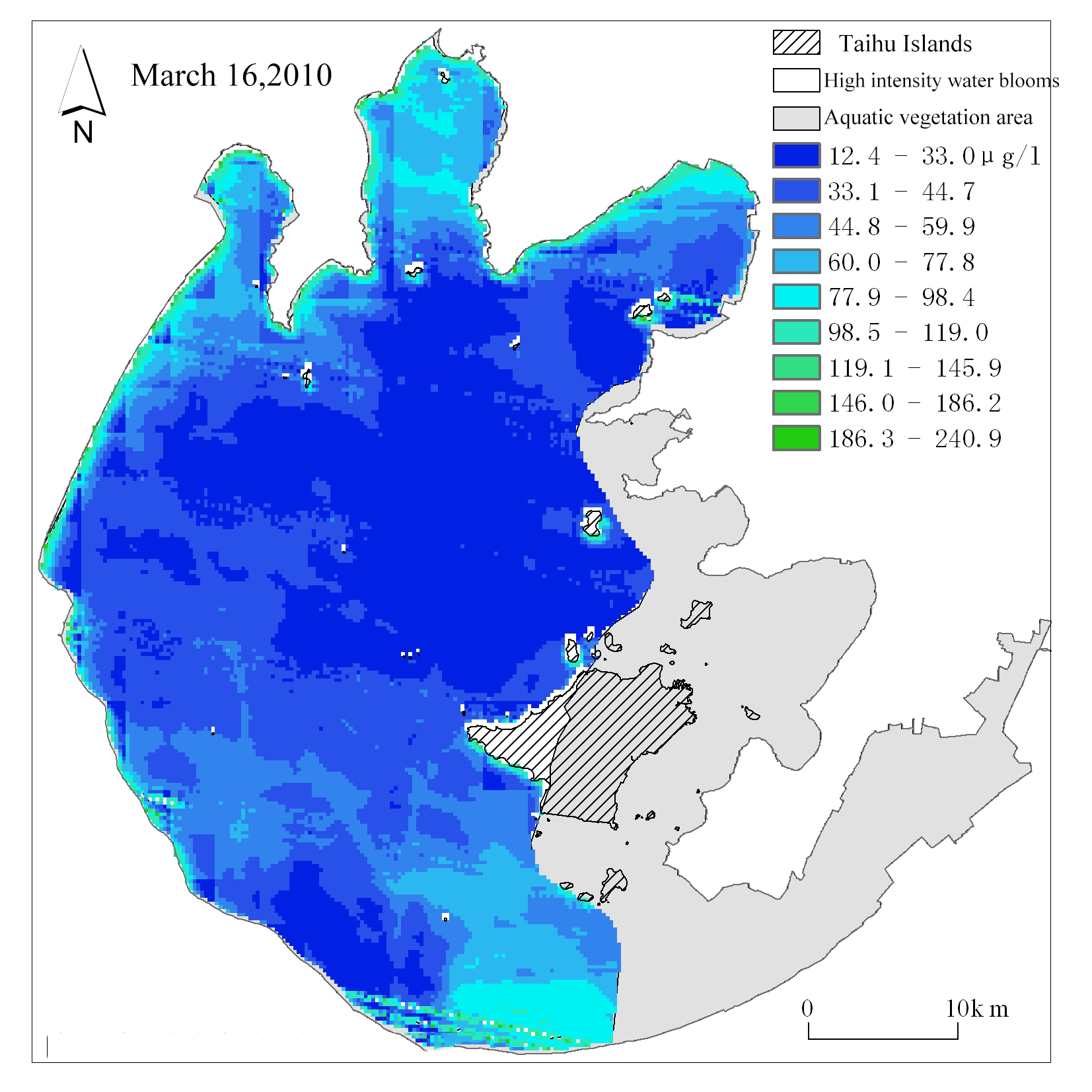

Supplement: Supplemental Information 2 — The data were obtained from the remote sensing image data of chlorophyll a concentration from the Lake-Watershed Science SubCenter, National Earth System Science Data Center, National Science & Technology Infrastructure of China, which had inconsistent data scales, data anomalies and different sampling intervals, and the chlorophyll a concentration unit was µg/L. [file peerj-cs-09-1292-s002.zip › 201003161111_taihu_chla.jpg]

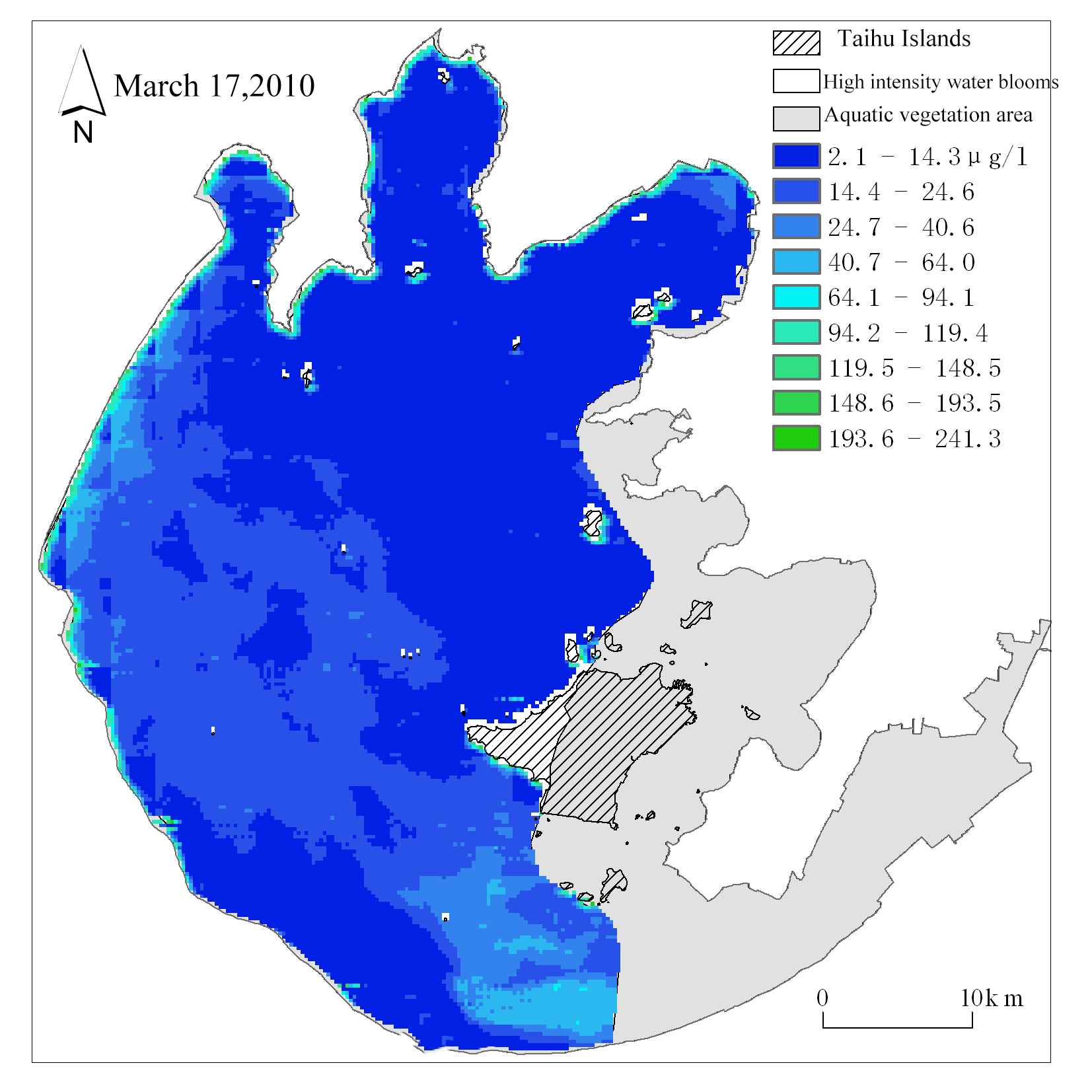

Supplement: Supplemental Information 2 — The data were obtained from the remote sensing image data of chlorophyll a concentration from the Lake-Watershed Science SubCenter, National Earth System Science Data Center, National Science & Technology Infrastructure of China, which had inconsistent data scales, data anomalies and different sampling intervals, and the chlorophyll a concentration unit was µg/L. [file peerj-cs-09-1292-s002.zip › 201003171111_taihu_chla.jpg]

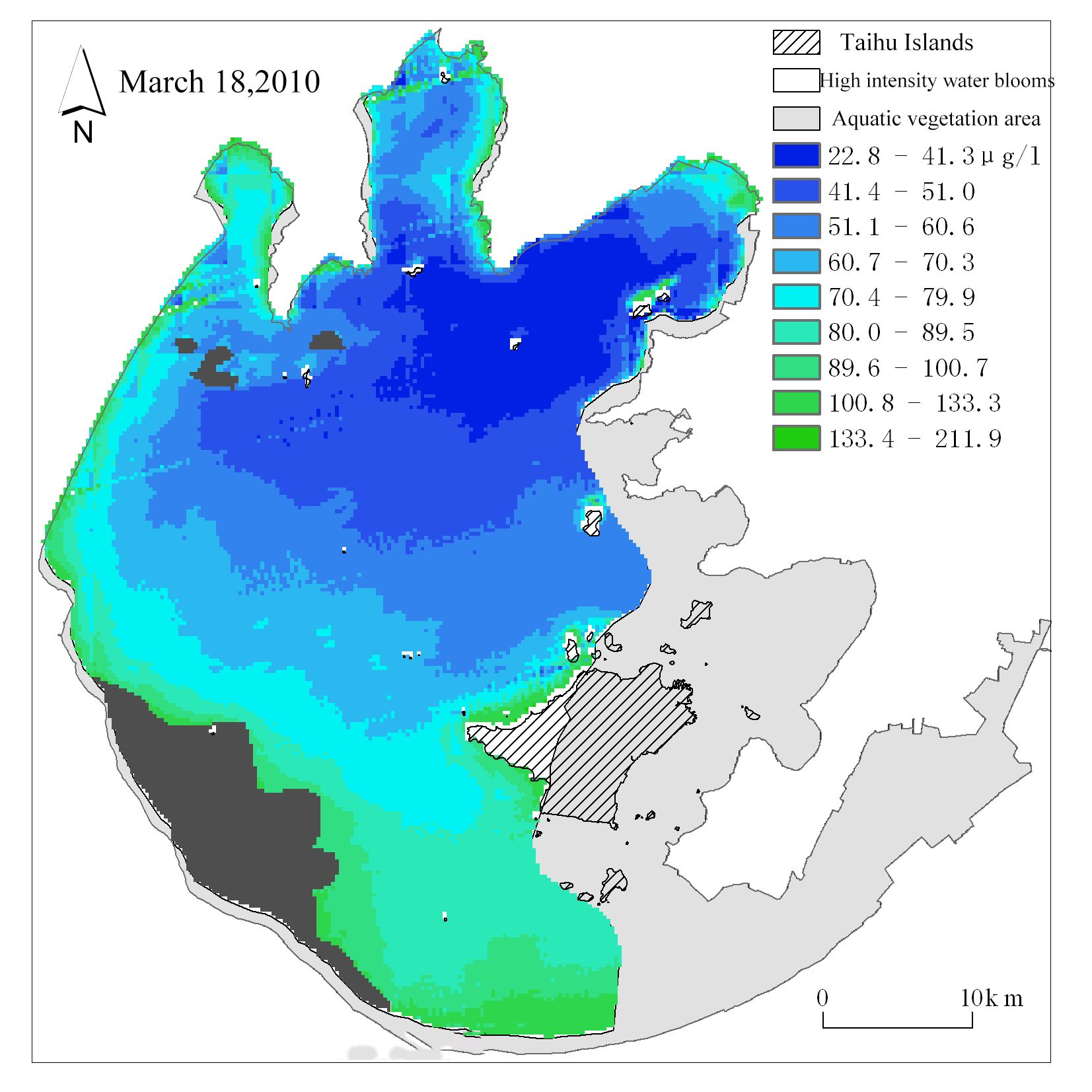

Supplement: Supplemental Information 2 — The data were obtained from the remote sensing image data of chlorophyll a concentration from the Lake-Watershed Science SubCenter, National Earth System Science Data Center, National Science & Technology Infrastructure of China, which had inconsistent data scales, data anomalies and different sampling intervals, and the chlorophyll a concentration unit was µg/L. [file peerj-cs-09-1292-s002.zip › 201003180438_taihu_chla.jpg]

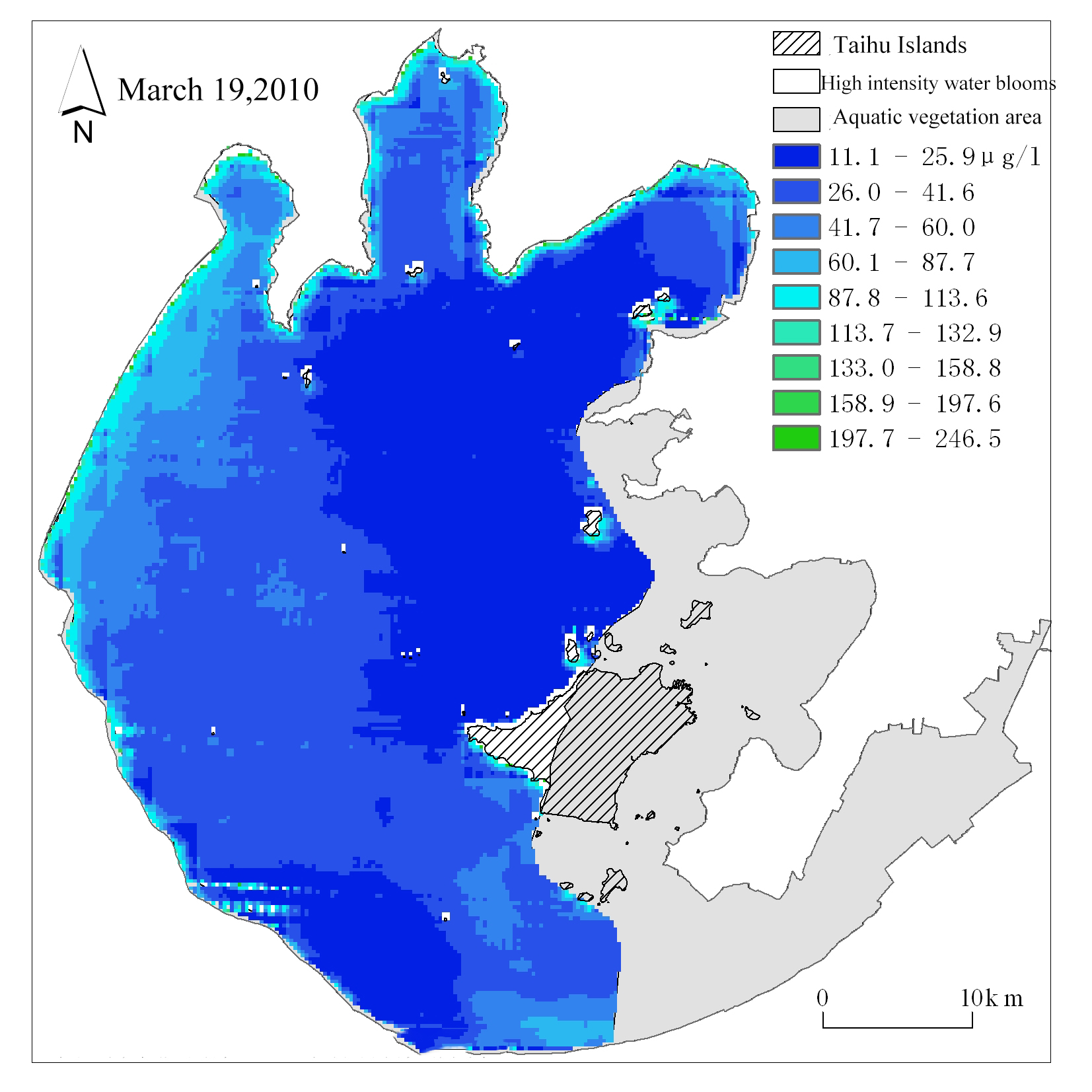

Supplement: Supplemental Information 2 — The data were obtained from the remote sensing image data of chlorophyll a concentration from the Lake-Watershed Science SubCenter, National Earth System Science Data Center, National Science & Technology Infrastructure of China, which had inconsistent data scales, data anomalies and different sampling intervals, and the chlorophyll a concentration unit was µg/L. [file peerj-cs-09-1292-s002.zip › 201003191111_taihu_chla.jpg]

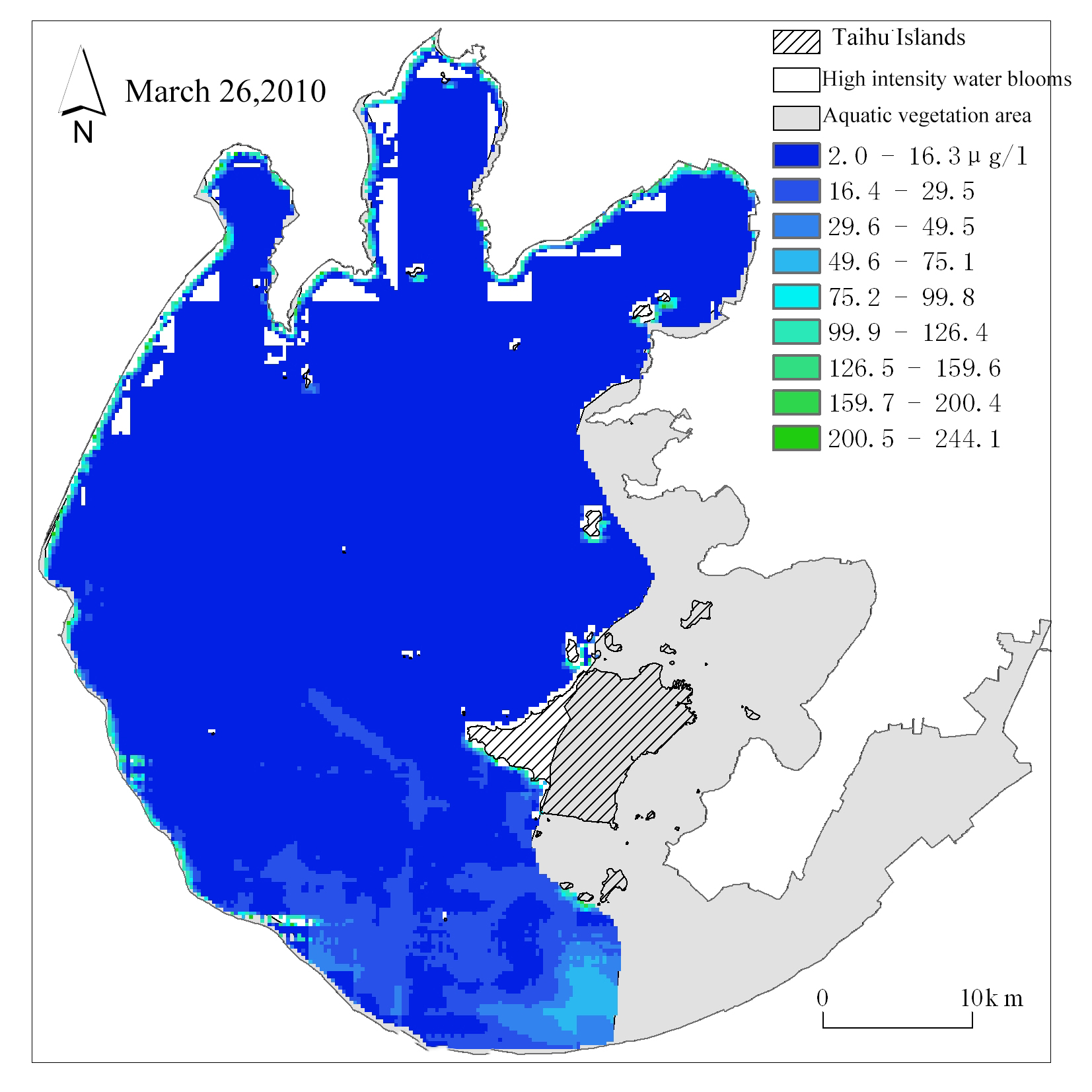

Supplement: Supplemental Information 2 — The data were obtained from the remote sensing image data of chlorophyll a concentration from the Lake-Watershed Science SubCenter, National Earth System Science Data Center, National Science & Technology Infrastructure of China, which had inconsistent data scales, data anomalies and different sampling intervals, and the chlorophyll a concentration unit was µg/L. [file peerj-cs-09-1292-s002.zip › 201003260215_taihu_chla.jpg]

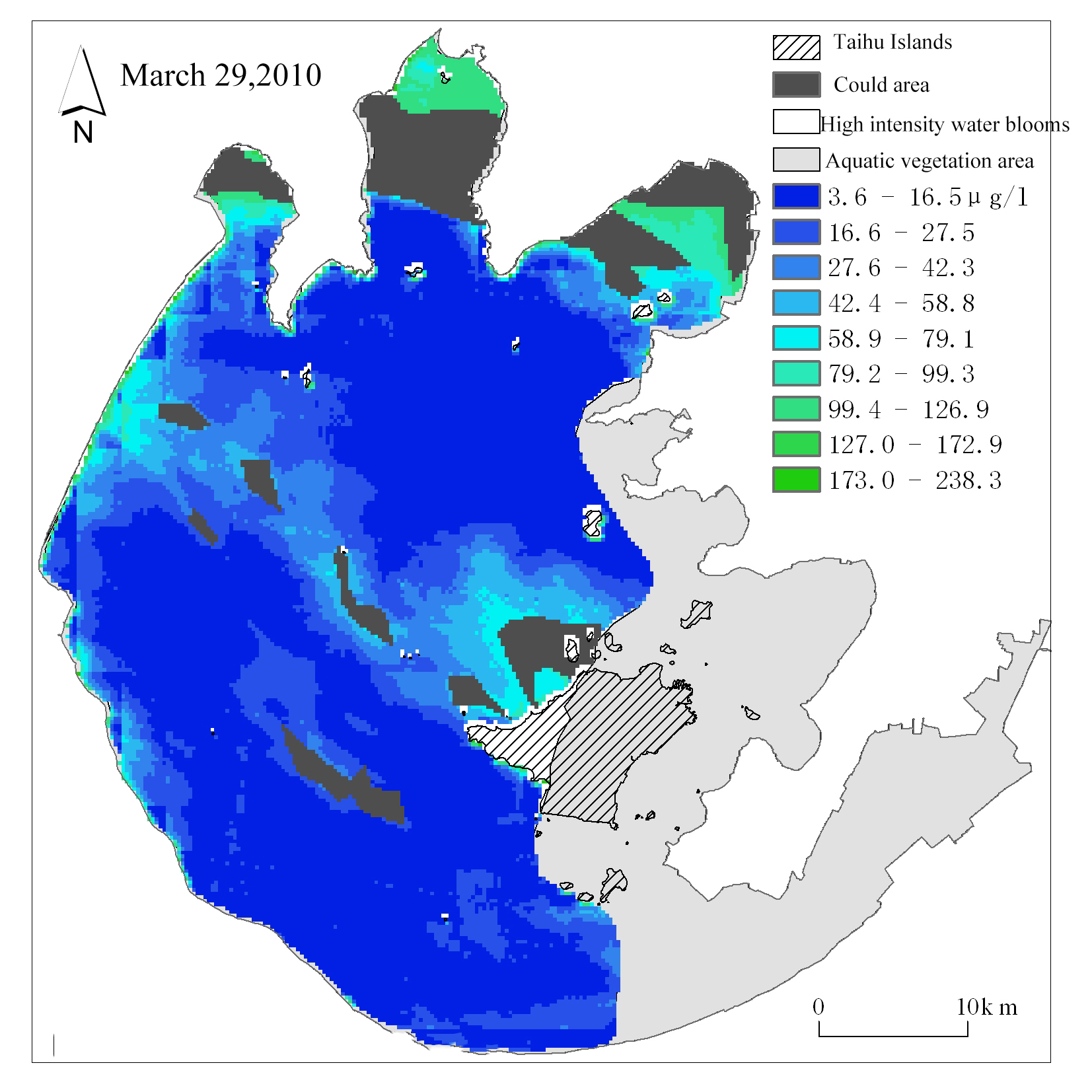

Supplement: Supplemental Information 2 — The data were obtained from the remote sensing image data of chlorophyll a concentration from the Lake-Watershed Science SubCenter, National Earth System Science Data Center, National Science & Technology Infrastructure of China, which had inconsistent data scales, data anomalies and different sampling intervals, and the chlorophyll a concentration unit was µg/L. [file peerj-cs-09-1292-s002.zip › 201003290245_taihu_chla.jpg]

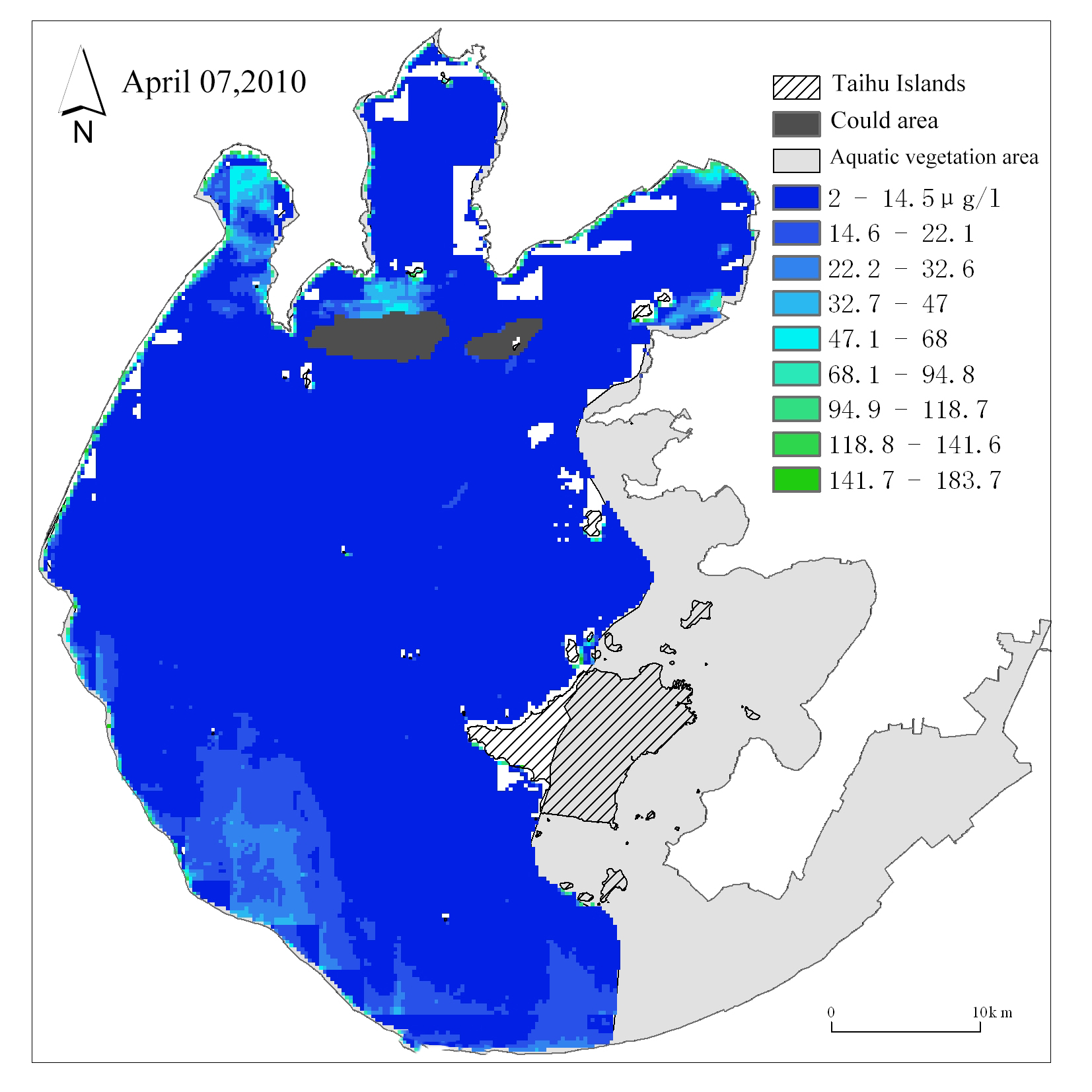

Supplement: Supplemental Information 2 — The data were obtained from the remote sensing image data of chlorophyll a concentration from the Lake-Watershed Science SubCenter, National Earth System Science Data Center, National Science & Technology Infrastructure of China, which had inconsistent data scales, data anomalies and different sampling intervals, and the chlorophyll a concentration unit was µg/L. [file peerj-cs-09-1292-s002.zip › 201004070239_taihu_chla.jpg]

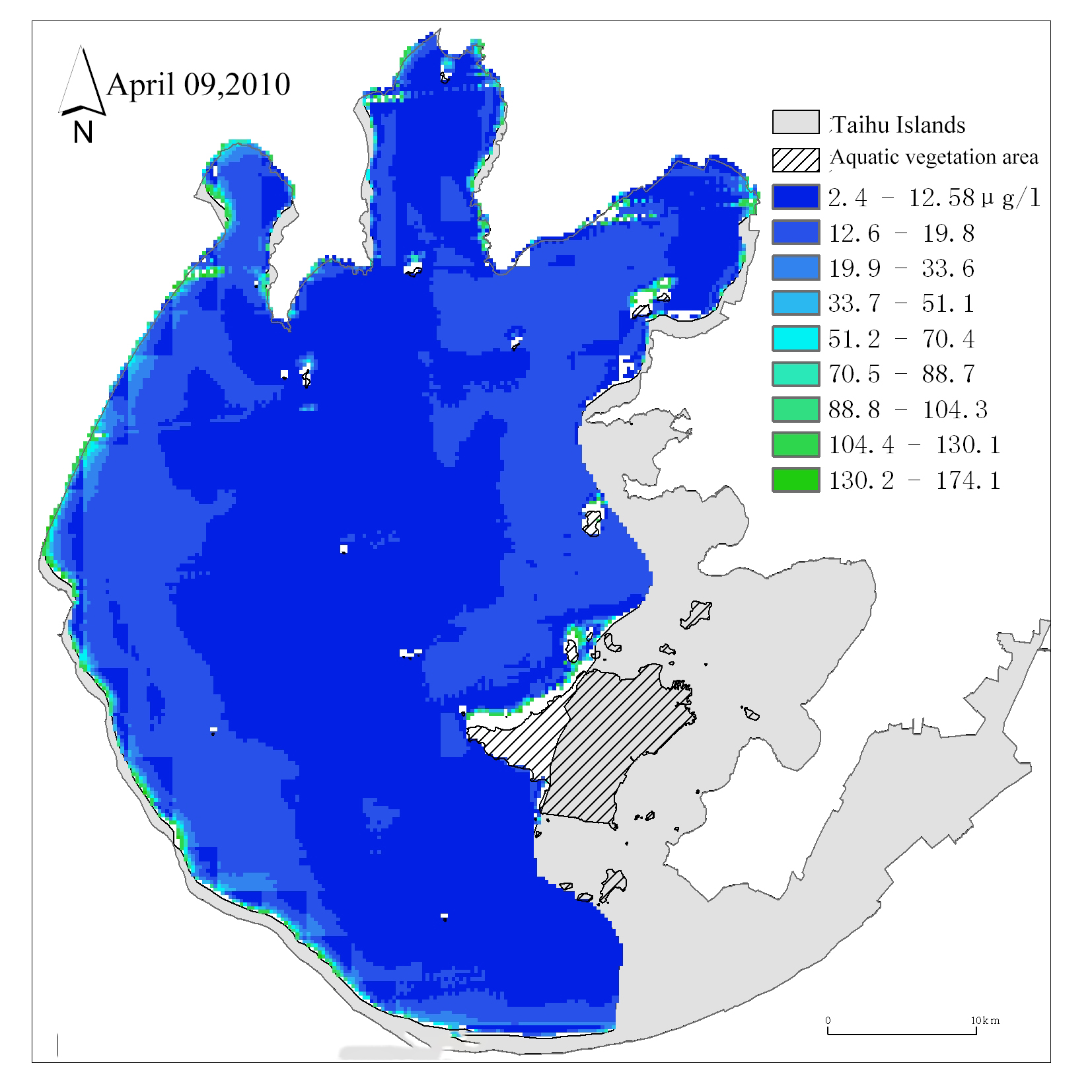

Supplement: Supplemental Information 2 — The data were obtained from the remote sensing image data of chlorophyll a concentration from the Lake-Watershed Science SubCenter, National Earth System Science Data Center, National Science & Technology Infrastructure of China, which had inconsistent data scales, data anomalies and different sampling intervals, and the chlorophyll a concentration unit was µg/L. [file peerj-cs-09-1292-s002.zip › 201004090537_taihu_chla.jpg]

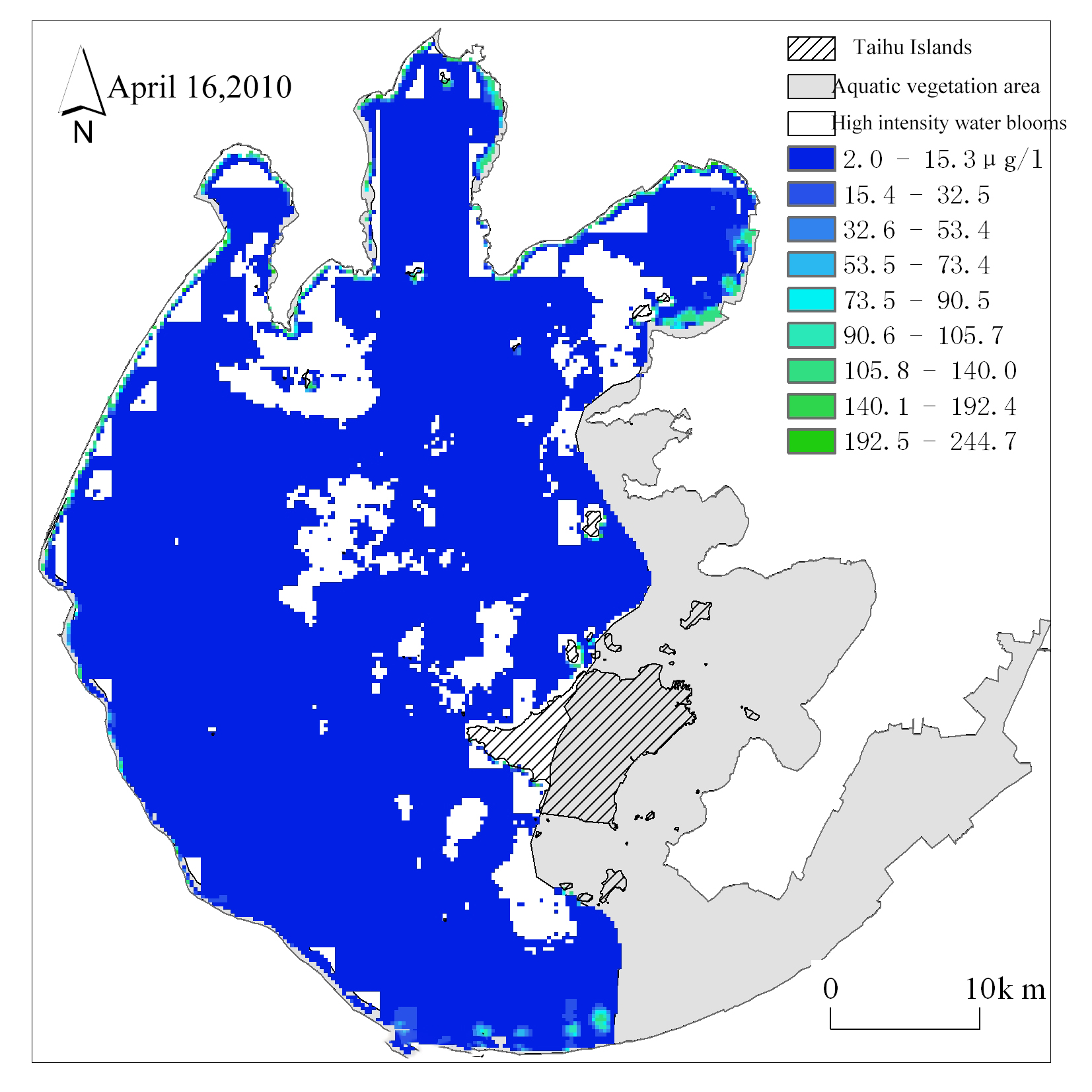

Supplement: Supplemental Information 2 — The data were obtained from the remote sensing image data of chlorophyll a concentration from the Lake-Watershed Science SubCenter, National Earth System Science Data Center, National Science & Technology Infrastructure of China, which had inconsistent data scales, data anomalies and different sampling intervals, and the chlorophyll a concentration unit was µg/L. [file peerj-cs-09-1292-s002.zip › 201004160234_taihu_chla.jpg]

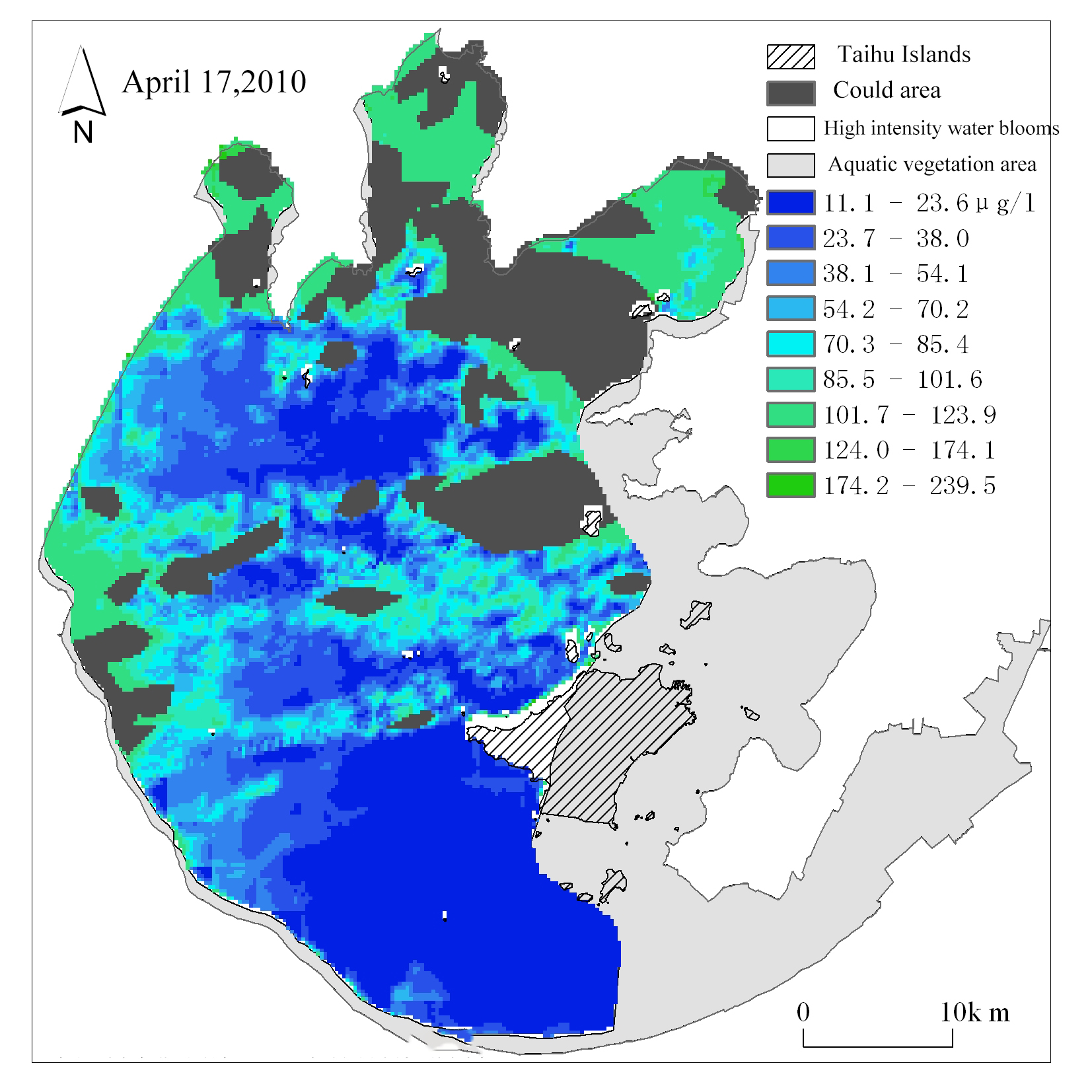

Supplement: Supplemental Information 2 — The data were obtained from the remote sensing image data of chlorophyll a concentration from the Lake-Watershed Science SubCenter, National Earth System Science Data Center, National Science & Technology Infrastructure of China, which had inconsistent data scales, data anomalies and different sampling intervals, and the chlorophyll a concentration unit was µg/L. [file peerj-cs-09-1292-s002.zip › 201004170446_taihu_chla.jpg]

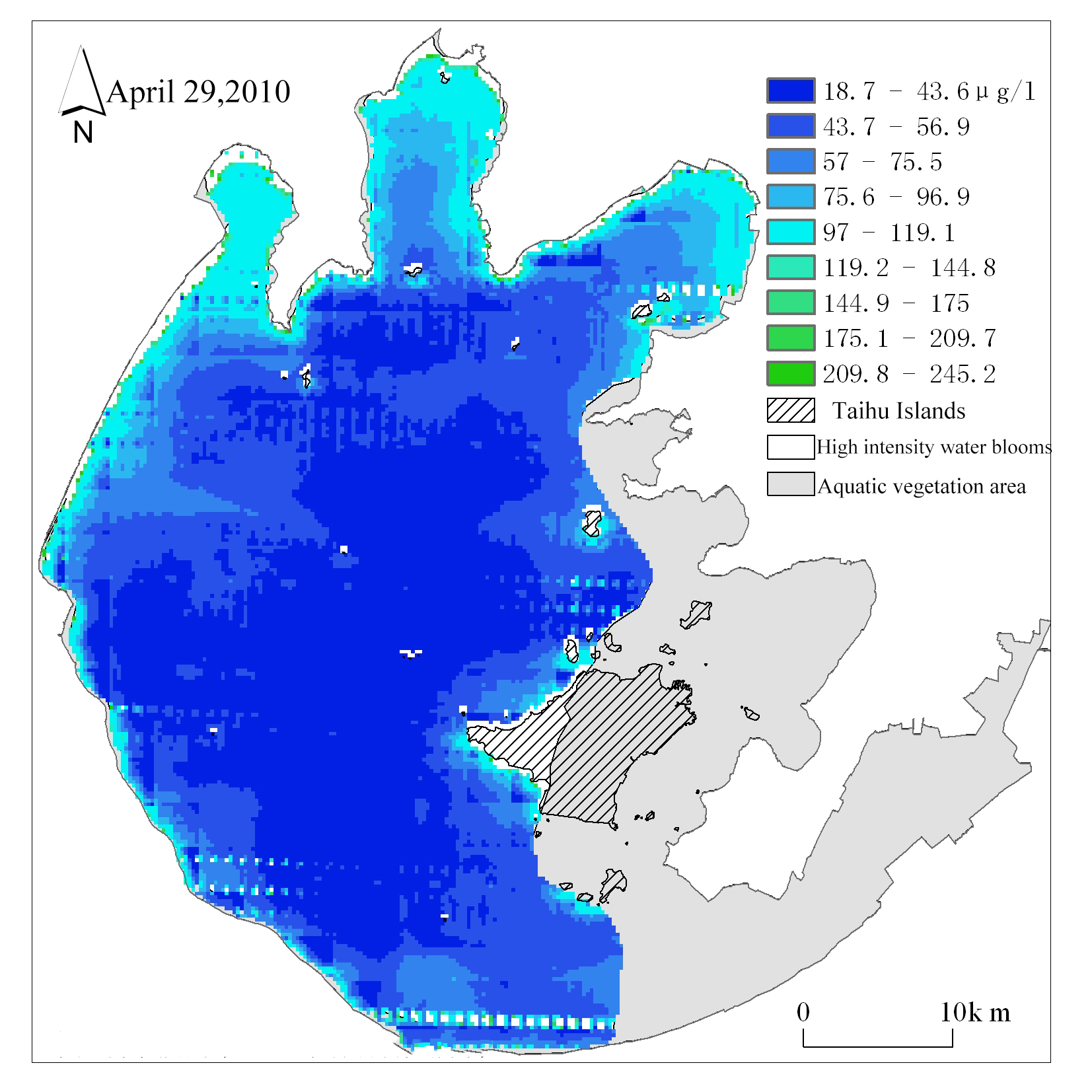

Supplement: Supplemental Information 2 — The data were obtained from the remote sensing image data of chlorophyll a concentration from the Lake-Watershed Science SubCenter, National Earth System Science Data Center, National Science & Technology Infrastructure of China, which had inconsistent data scales, data anomalies and different sampling intervals, and the chlorophyll a concentration unit was µg/L. [file peerj-cs-09-1292-s002.zip › 201004290203_chla.jpg]

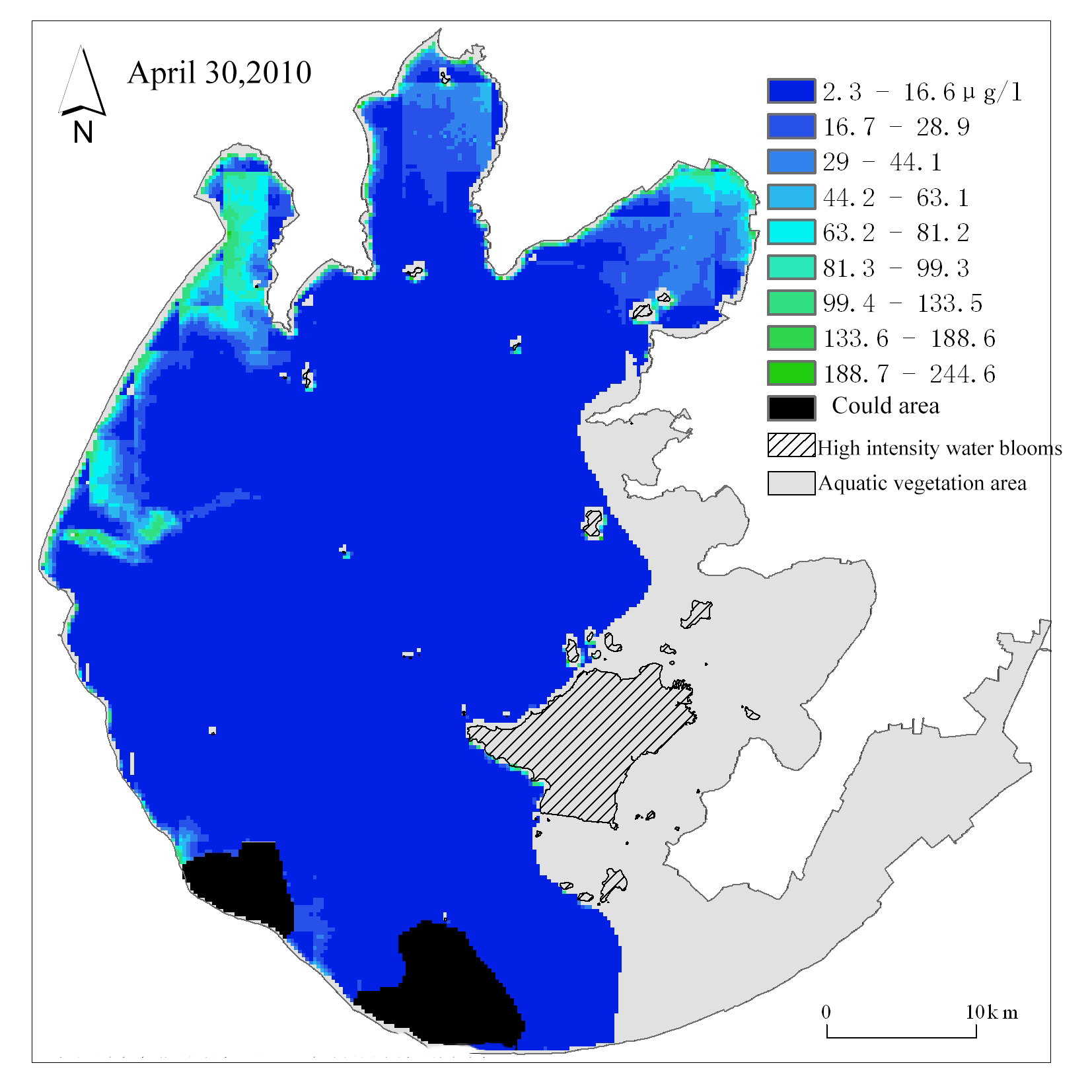

Supplement: Supplemental Information 2 — The data were obtained from the remote sensing image data of chlorophyll a concentration from the Lake-Watershed Science SubCenter, National Earth System Science Data Center, National Science & Technology Infrastructure of China, which had inconsistent data scales, data anomalies and different sampling intervals, and the chlorophyll a concentration unit was µg/L. [file peerj-cs-09-1292-s002.zip › 201004300245taihu_chla.jpg]

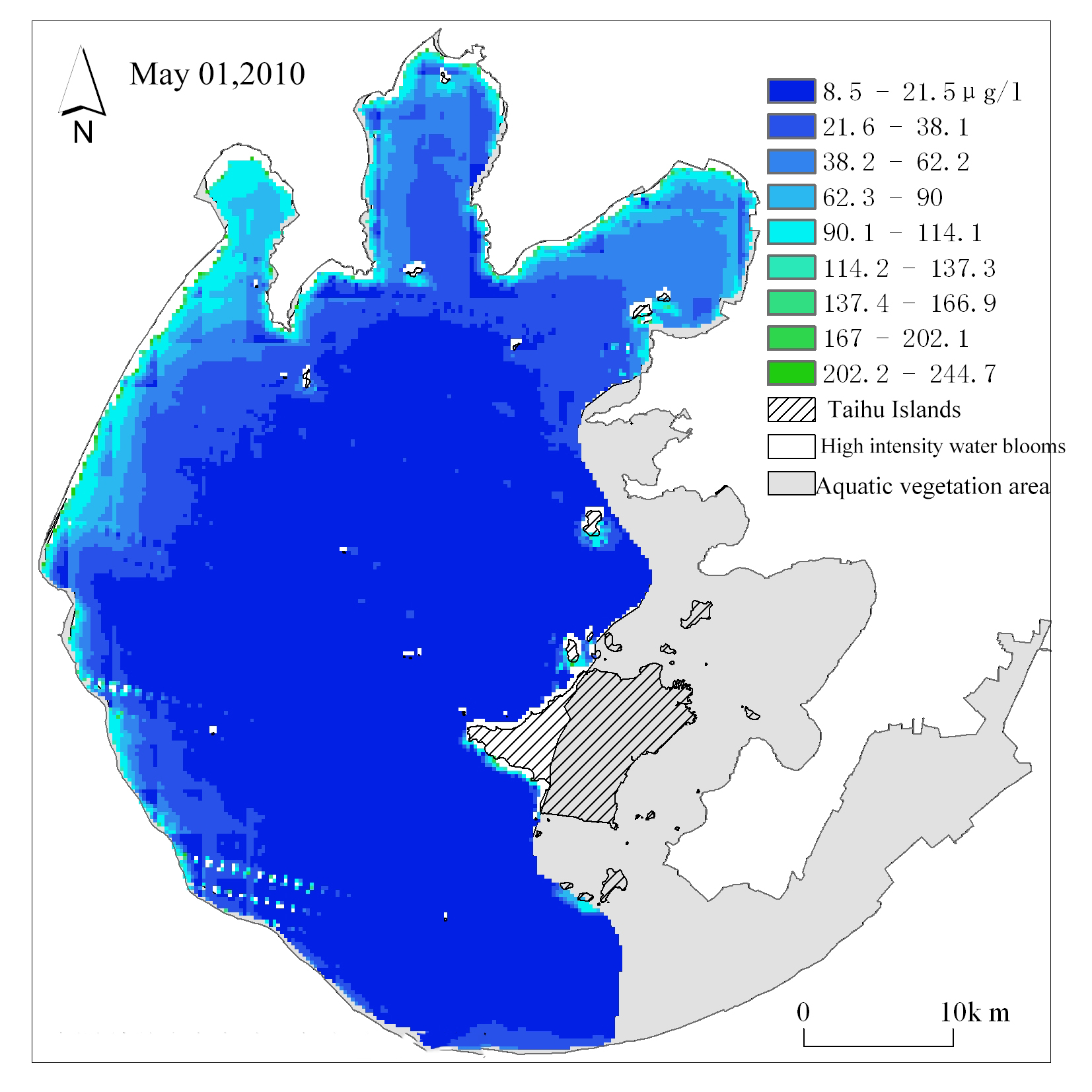

Supplement: Supplemental Information 2 — The data were obtained from the remote sensing image data of chlorophyll a concentration from the Lake-Watershed Science SubCenter, National Earth System Science Data Center, National Science & Technology Infrastructure of China, which had inconsistent data scales, data anomalies and different sampling intervals, and the chlorophyll a concentration unit was µg/L. [file peerj-cs-09-1292-s002.zip › 201005010327taihu_chla.jpg]

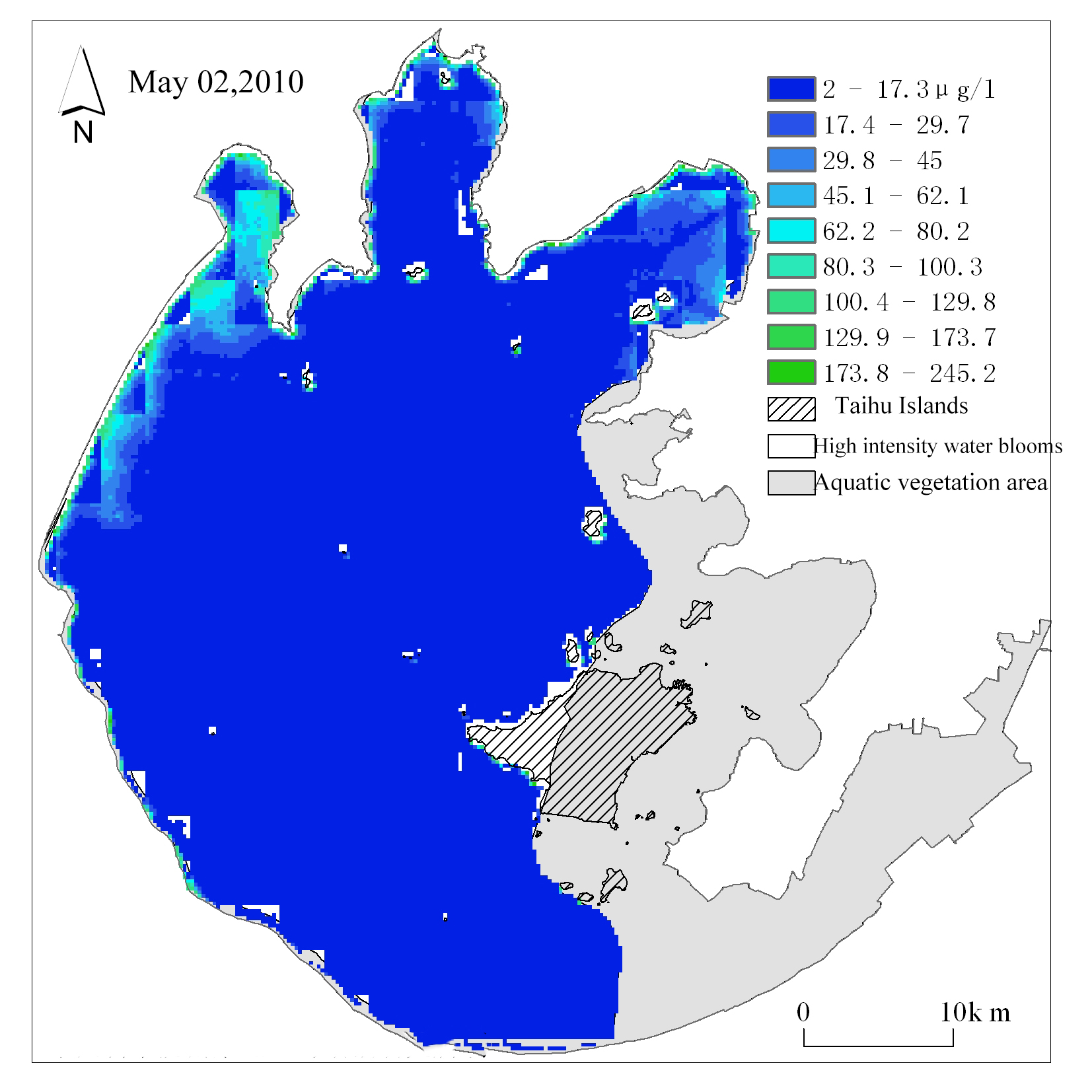

Supplement: Supplemental Information 2 — The data were obtained from the remote sensing image data of chlorophyll a concentration from the Lake-Watershed Science SubCenter, National Earth System Science Data Center, National Science & Technology Infrastructure of China, which had inconsistent data scales, data anomalies and different sampling intervals, and the chlorophyll a concentration unit was µg/L. [file peerj-cs-09-1292-s002.zip › 201005020232taihu_chla.jpg]

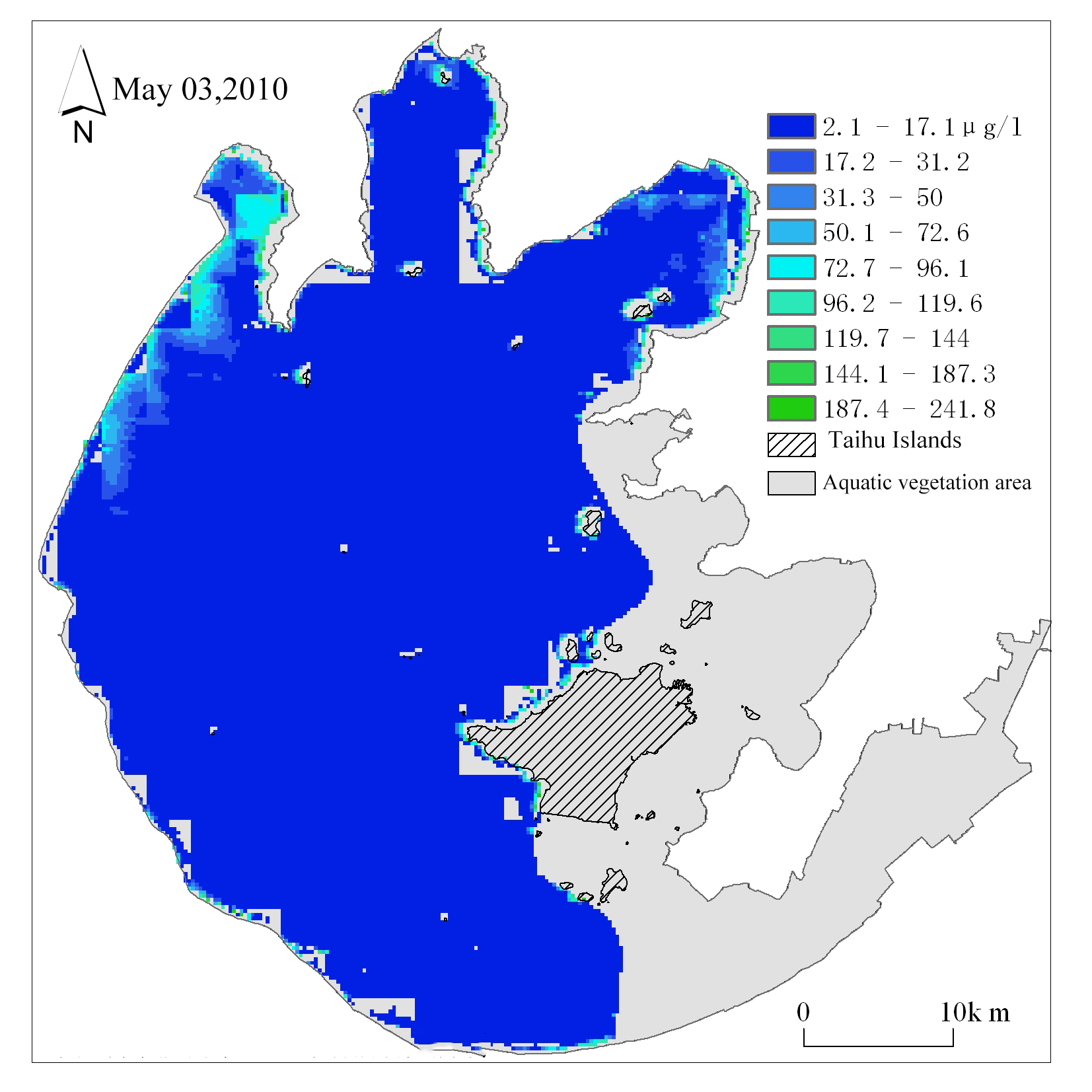

Supplement: Supplemental Information 2 — The data were obtained from the remote sensing image data of chlorophyll a concentration from the Lake-Watershed Science SubCenter, National Earth System Science Data Center, National Science & Technology Infrastructure of China, which had inconsistent data scales, data anomalies and different sampling intervals, and the chlorophyll a concentration unit was µg/L. [file peerj-cs-09-1292-s002.zip › 201005030315taihu_chla.jpg]

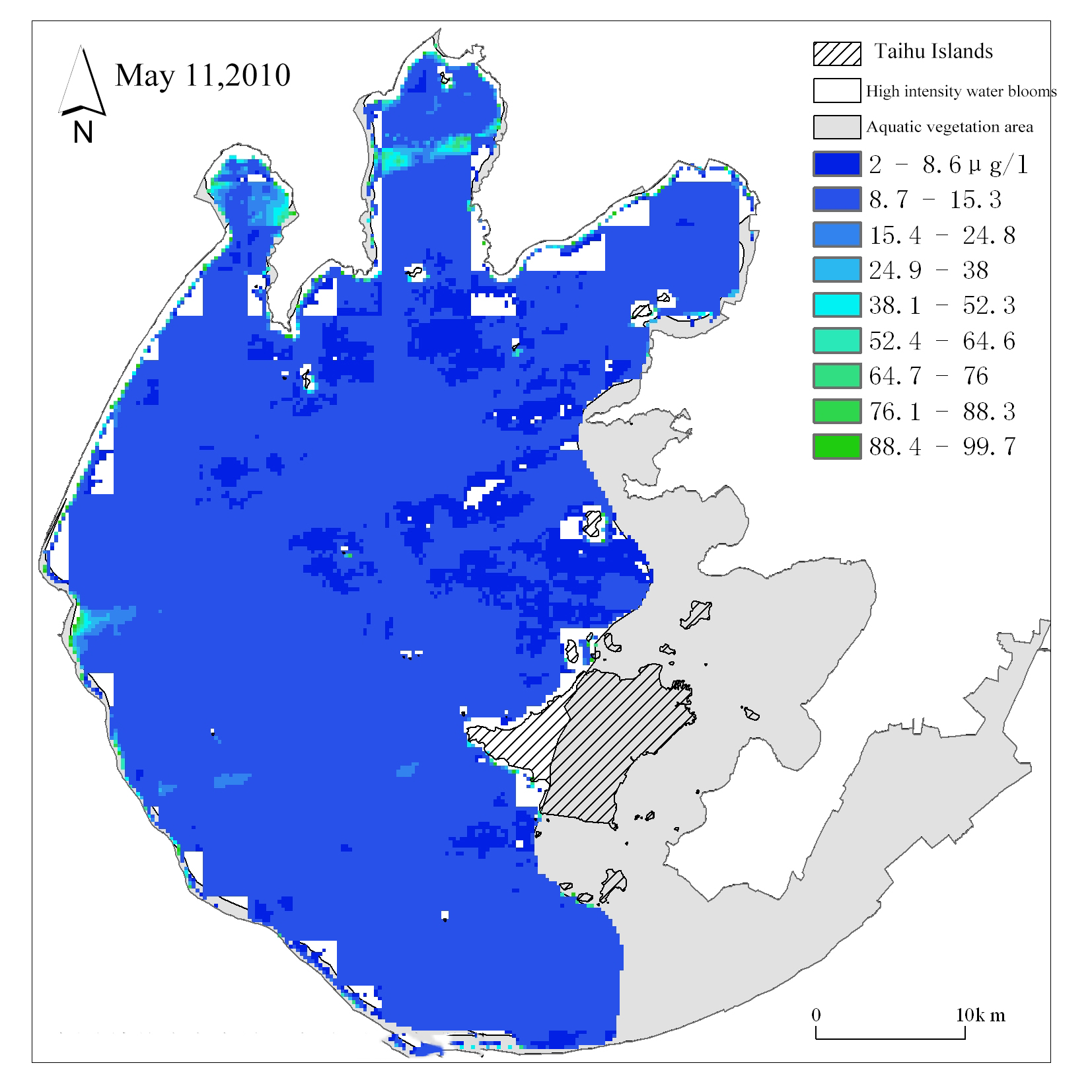

Supplement: Supplemental Information 2 — The data were obtained from the remote sensing image data of chlorophyll a concentration from the Lake-Watershed Science SubCenter, National Earth System Science Data Center, National Science & Technology Infrastructure of China, which had inconsistent data scales, data anomalies and different sampling intervals, and the chlorophyll a concentration unit was µg/L. [file peerj-cs-09-1292-s002.zip › 201005110227_taihu_chla.jpg]

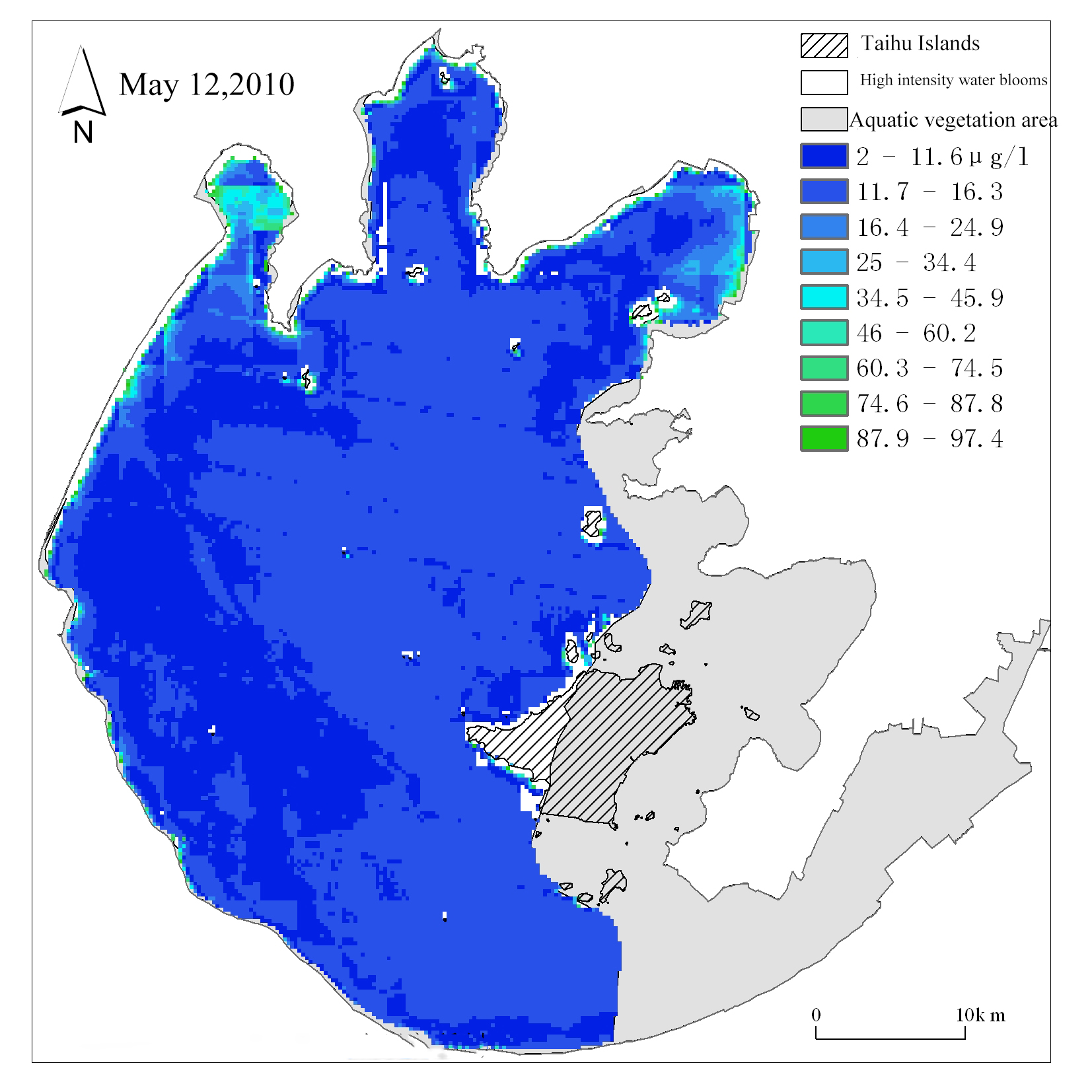

Supplement: Supplemental Information 2 — The data were obtained from the remote sensing image data of chlorophyll a concentration from the Lake-Watershed Science SubCenter, National Earth System Science Data Center, National Science & Technology Infrastructure of China, which had inconsistent data scales, data anomalies and different sampling intervals, and the chlorophyll a concentration unit was µg/L. [file peerj-cs-09-1292-s002.zip › 201005120309_taihu_chla.jpg]

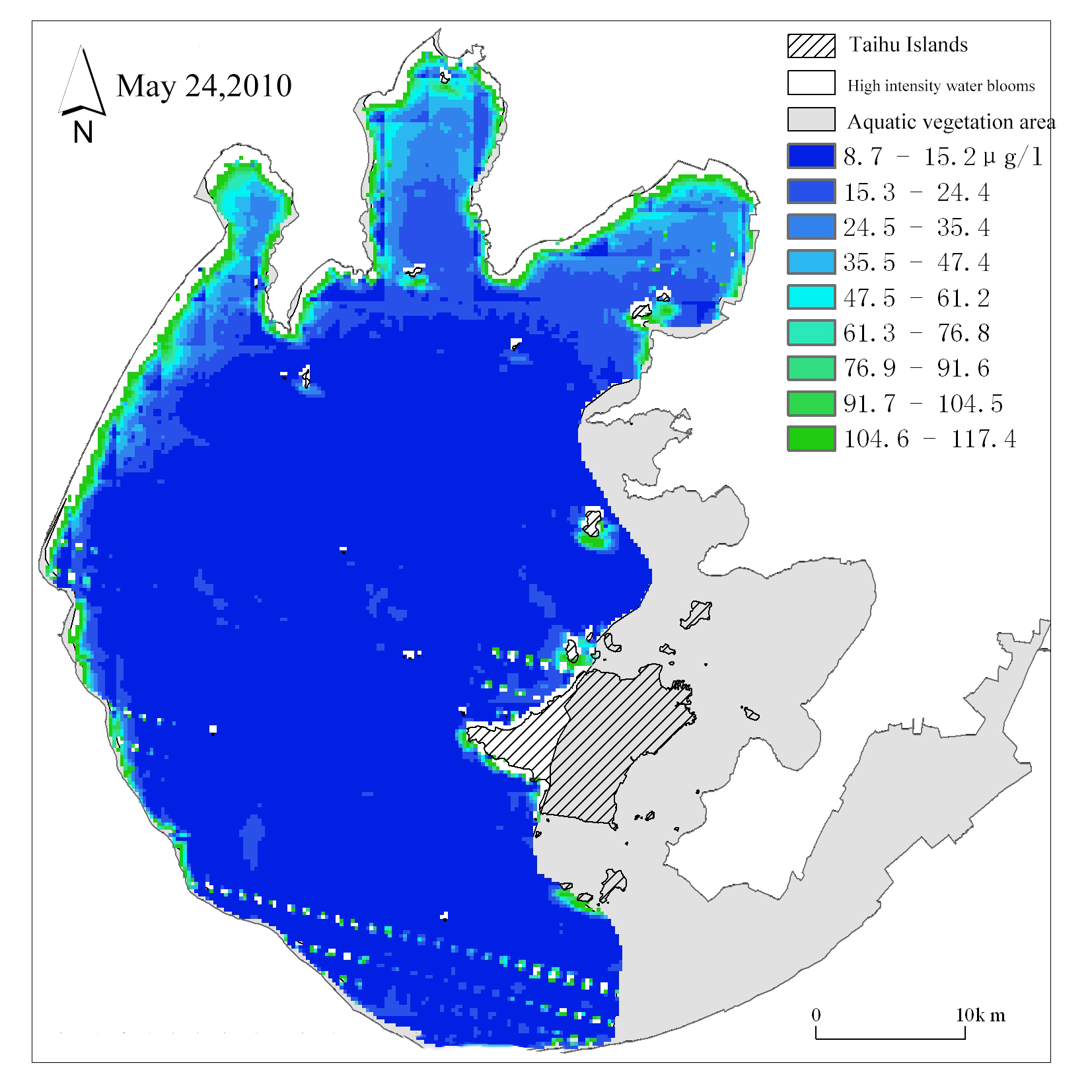

Supplement: Supplemental Information 2 — The data were obtained from the remote sensing image data of chlorophyll a concentration from the Lake-Watershed Science SubCenter, National Earth System Science Data Center, National Science & Technology Infrastructure of China, which had inconsistent data scales, data anomalies and different sampling intervals, and the chlorophyll a concentration unit was µg/L. [file peerj-cs-09-1292-s002.zip › 201005240334_taihu_chla.jpg]

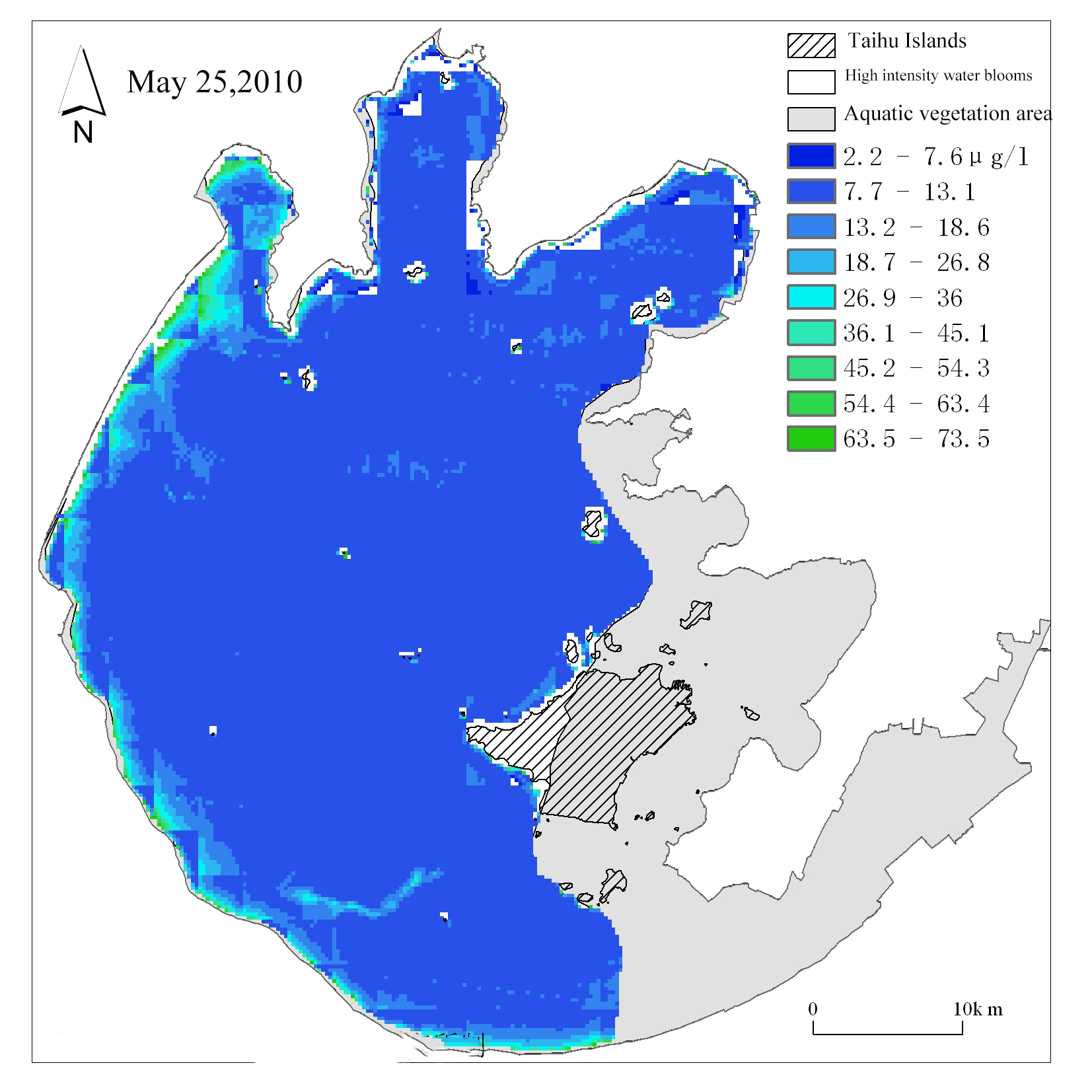

Supplement: Supplemental Information 2 — The data were obtained from the remote sensing image data of chlorophyll a concentration from the Lake-Watershed Science SubCenter, National Earth System Science Data Center, National Science & Technology Infrastructure of China, which had inconsistent data scales, data anomalies and different sampling intervals, and the chlorophyll a concentration unit was µg/L. [file peerj-cs-09-1292-s002.zip › 201005250239_taihu_chla.jpg]

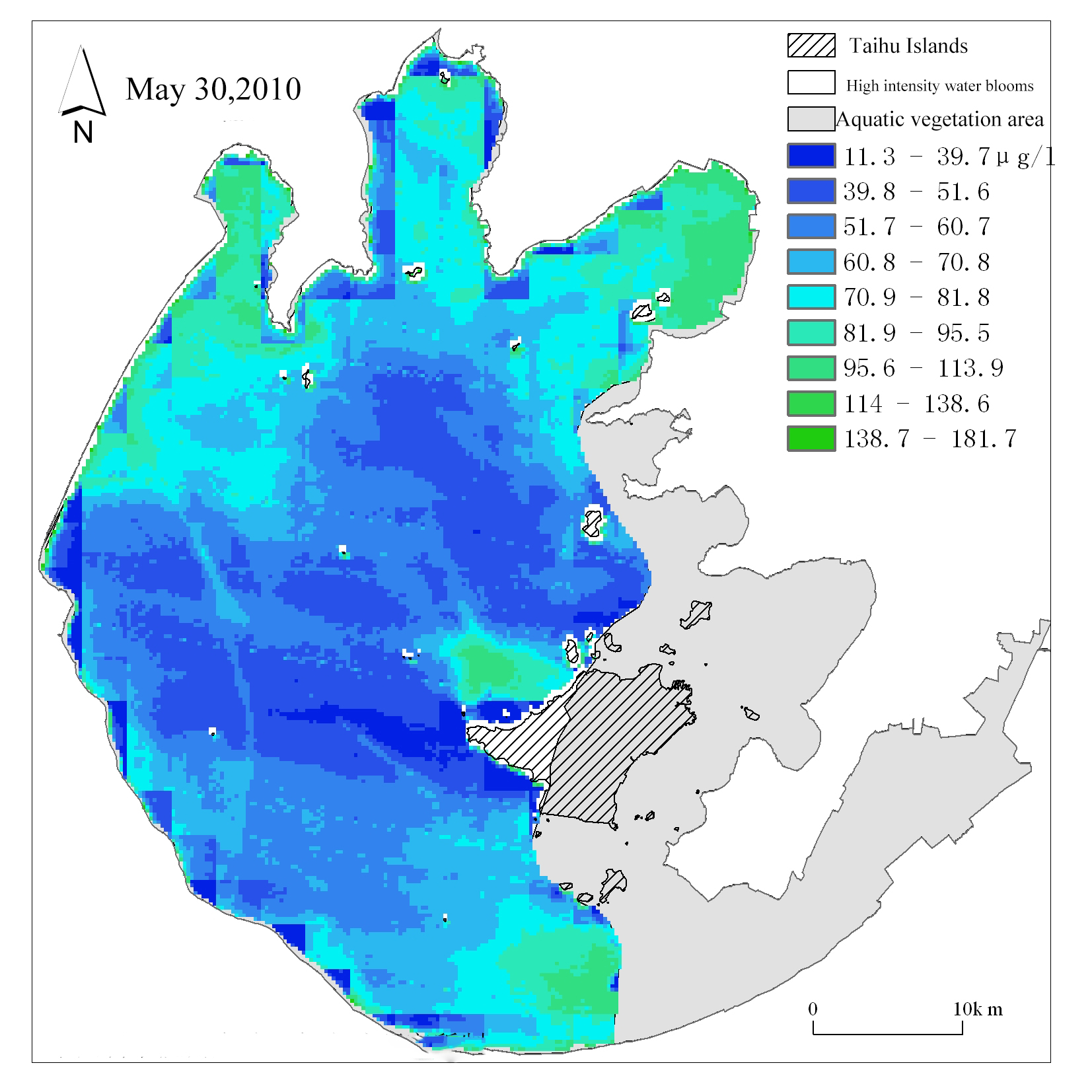

Supplement: Supplemental Information 2 — The data were obtained from the remote sensing image data of chlorophyll a concentration from the Lake-Watershed Science SubCenter, National Earth System Science Data Center, National Science & Technology Infrastructure of China, which had inconsistent data scales, data anomalies and different sampling intervals, and the chlorophyll a concentration unit was µg/L. [file peerj-cs-09-1292-s002.zip › 201005300227_taihu_chla.jpg]

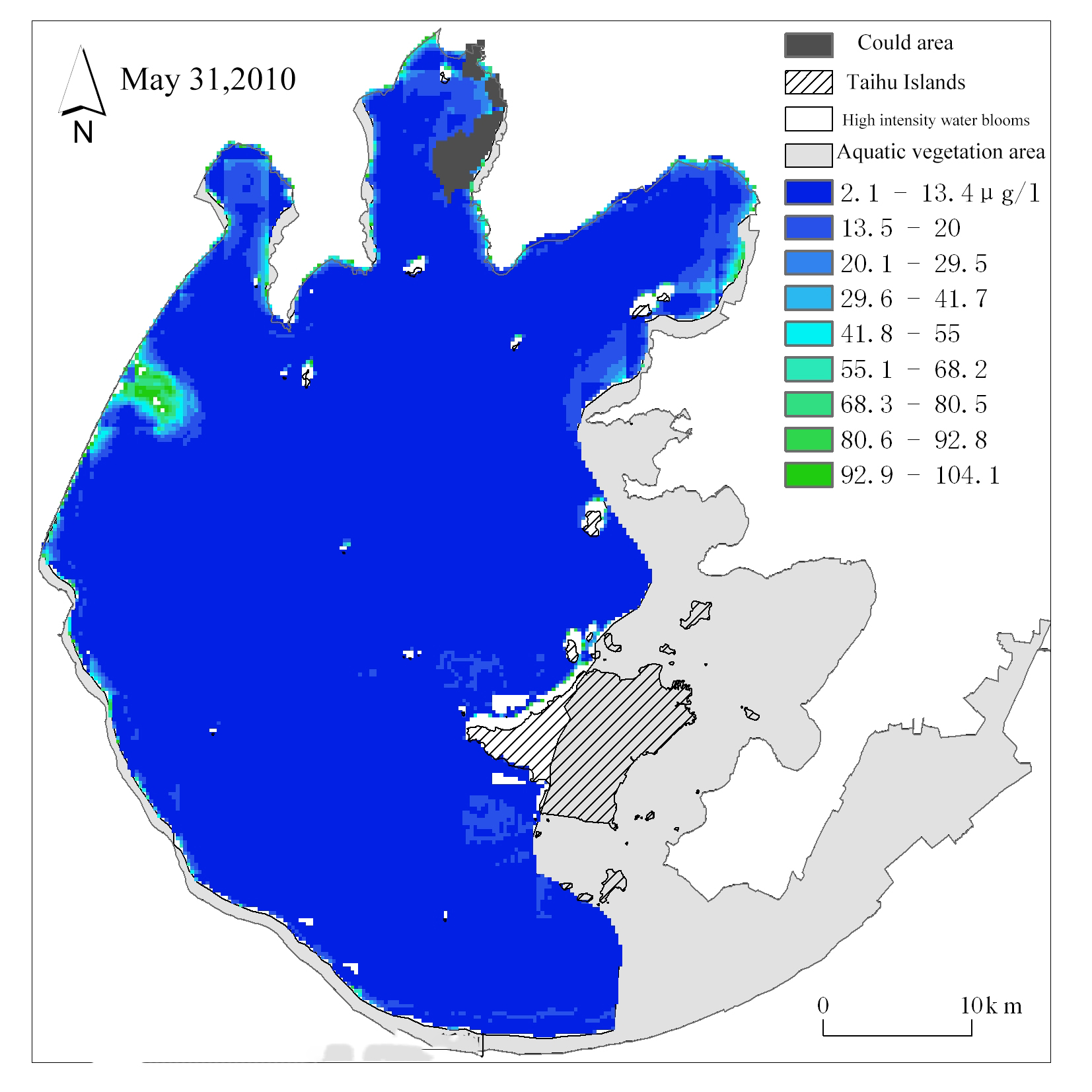

Supplement: Supplemental Information 2 — The data were obtained from the remote sensing image data of chlorophyll a concentration from the Lake-Watershed Science SubCenter, National Earth System Science Data Center, National Science & Technology Infrastructure of China, which had inconsistent data scales, data anomalies and different sampling intervals, and the chlorophyll a concentration unit was µg/L. [file peerj-cs-09-1292-s002.zip › 201005310512_taihu_chla.jpg]

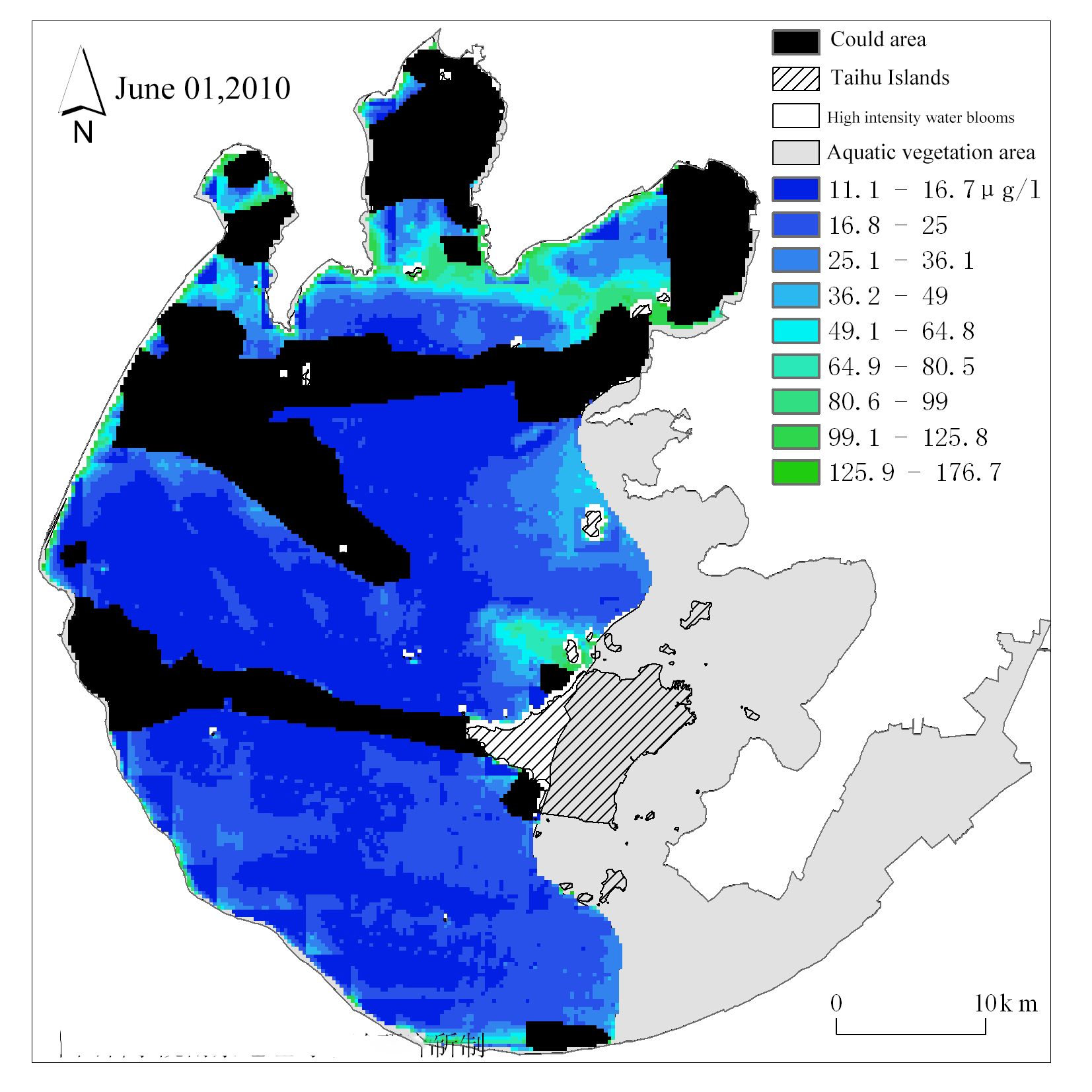

Supplement: Supplemental Information 2 — The data were obtained from the remote sensing image data of chlorophyll a concentration from the Lake-Watershed Science SubCenter, National Earth System Science Data Center, National Science & Technology Infrastructure of China, which had inconsistent data scales, data anomalies and different sampling intervals, and the chlorophyll a concentration unit was µg/L. [file peerj-cs-09-1292-s002.zip › 201006010245_taihu_chla.jpg]

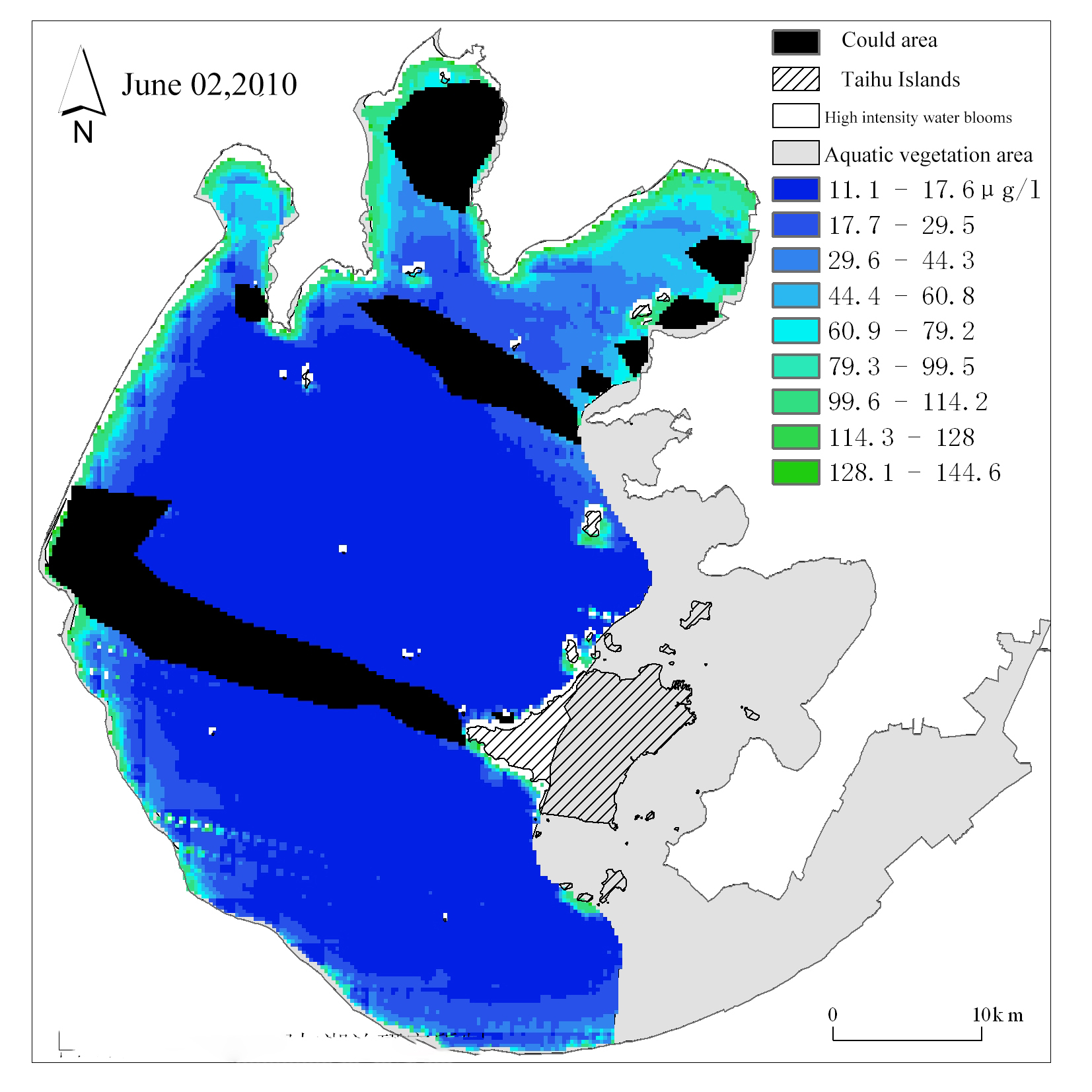

Supplement: Supplemental Information 2 — The data were obtained from the remote sensing image data of chlorophyll a concentration from the Lake-Watershed Science SubCenter, National Earth System Science Data Center, National Science & Technology Infrastructure of China, which had inconsistent data scales, data anomalies and different sampling intervals, and the chlorophyll a concentration unit was µg/L. [file peerj-cs-09-1292-s002.zip › 201006020337_taihu_chla.jpg]

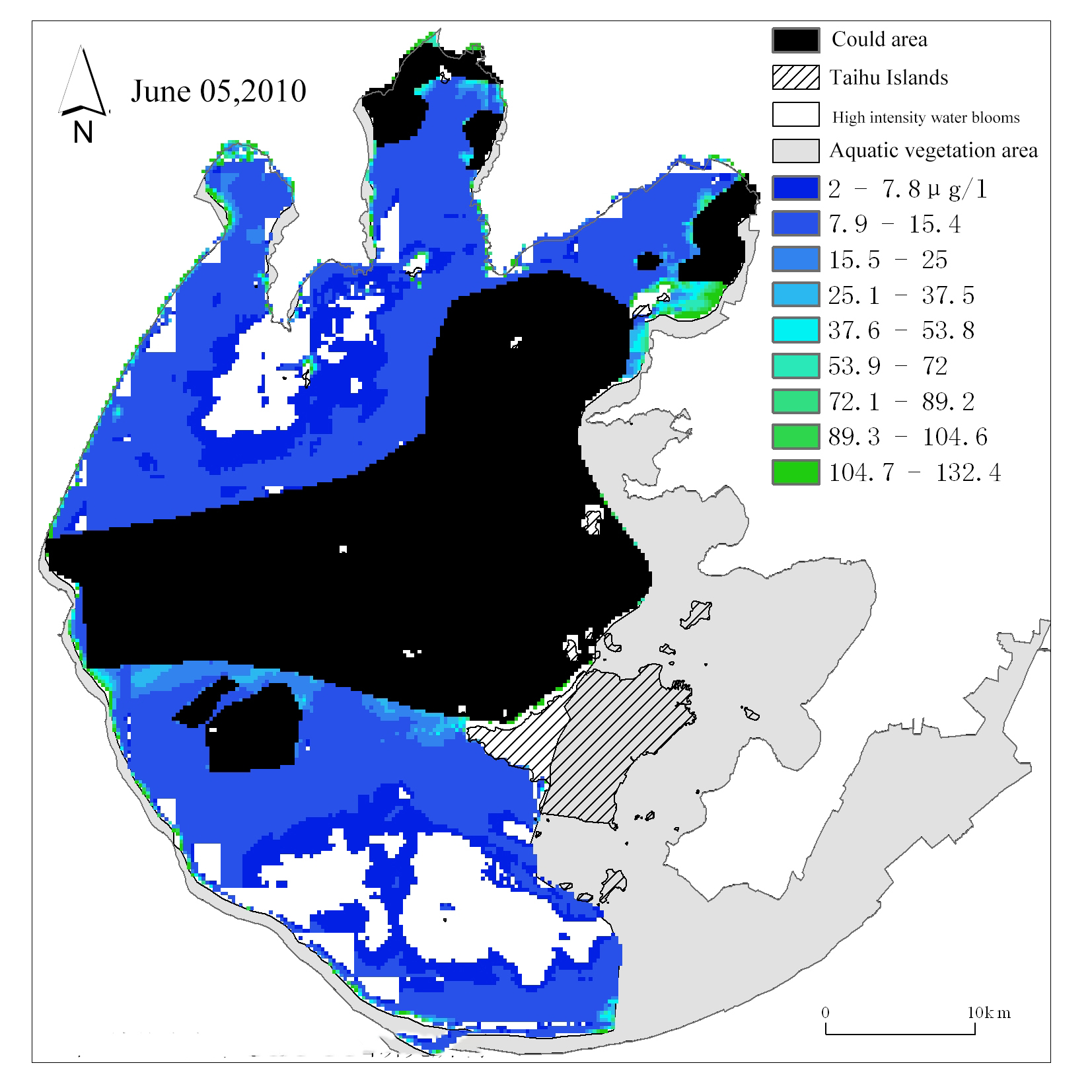

Supplement: Supplemental Information 2 — The data were obtained from the remote sensing image data of chlorophyll a concentration from the Lake-Watershed Science SubCenter, National Earth System Science Data Center, National Science & Technology Infrastructure of China, which had inconsistent data scales, data anomalies and different sampling intervals, and the chlorophyll a concentration unit was µg/L. [file peerj-cs-09-1292-s002.zip › 201006050530_taihu_chla.jpg]

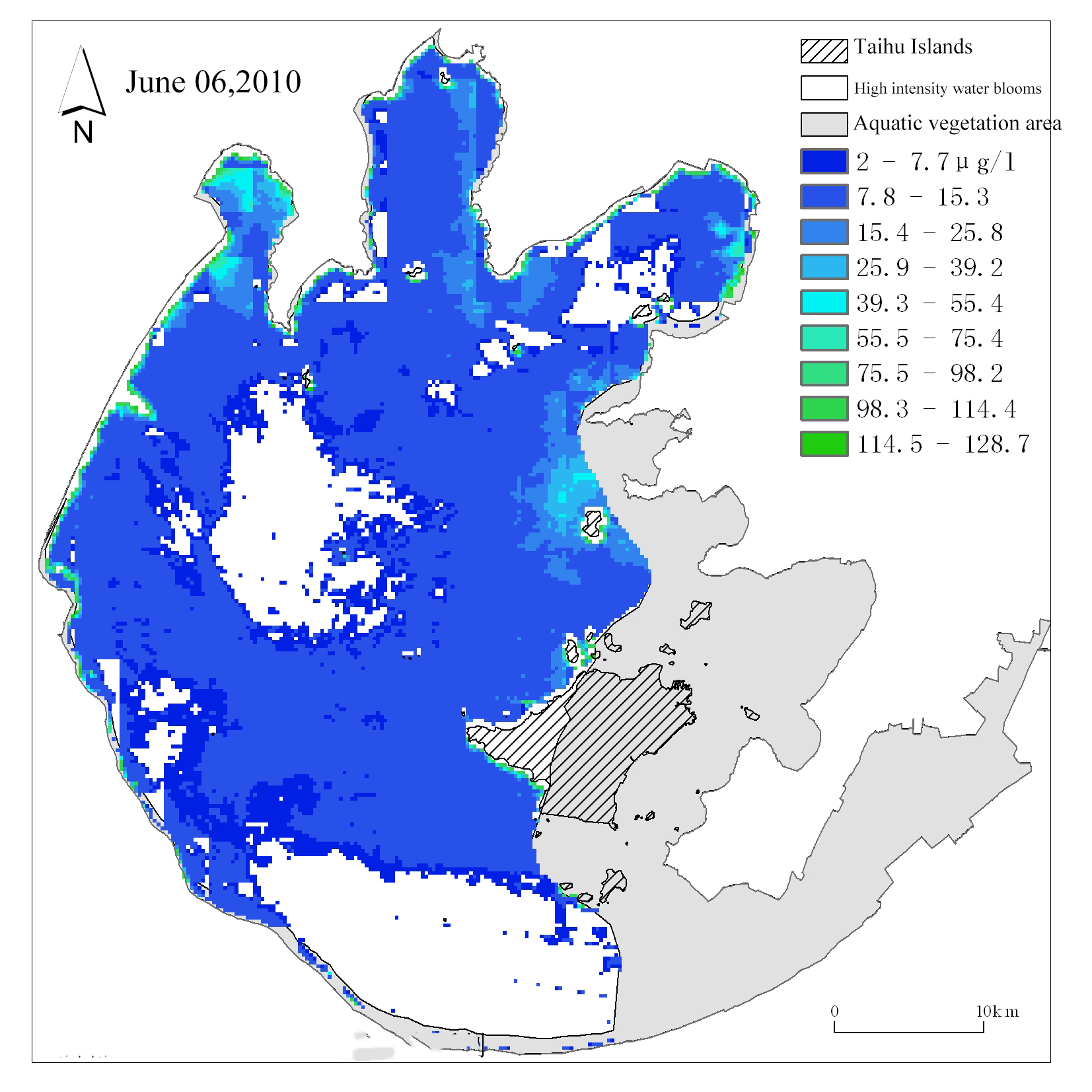

Supplement: Supplemental Information 2 — The data were obtained from the remote sensing image data of chlorophyll a concentration from the Lake-Watershed Science SubCenter, National Earth System Science Data Center, National Science & Technology Infrastructure of China, which had inconsistent data scales, data anomalies and different sampling intervals, and the chlorophyll a concentration unit was µg/L. [file peerj-cs-09-1292-s002.zip › 201006060303_taihu_chla.jpg]

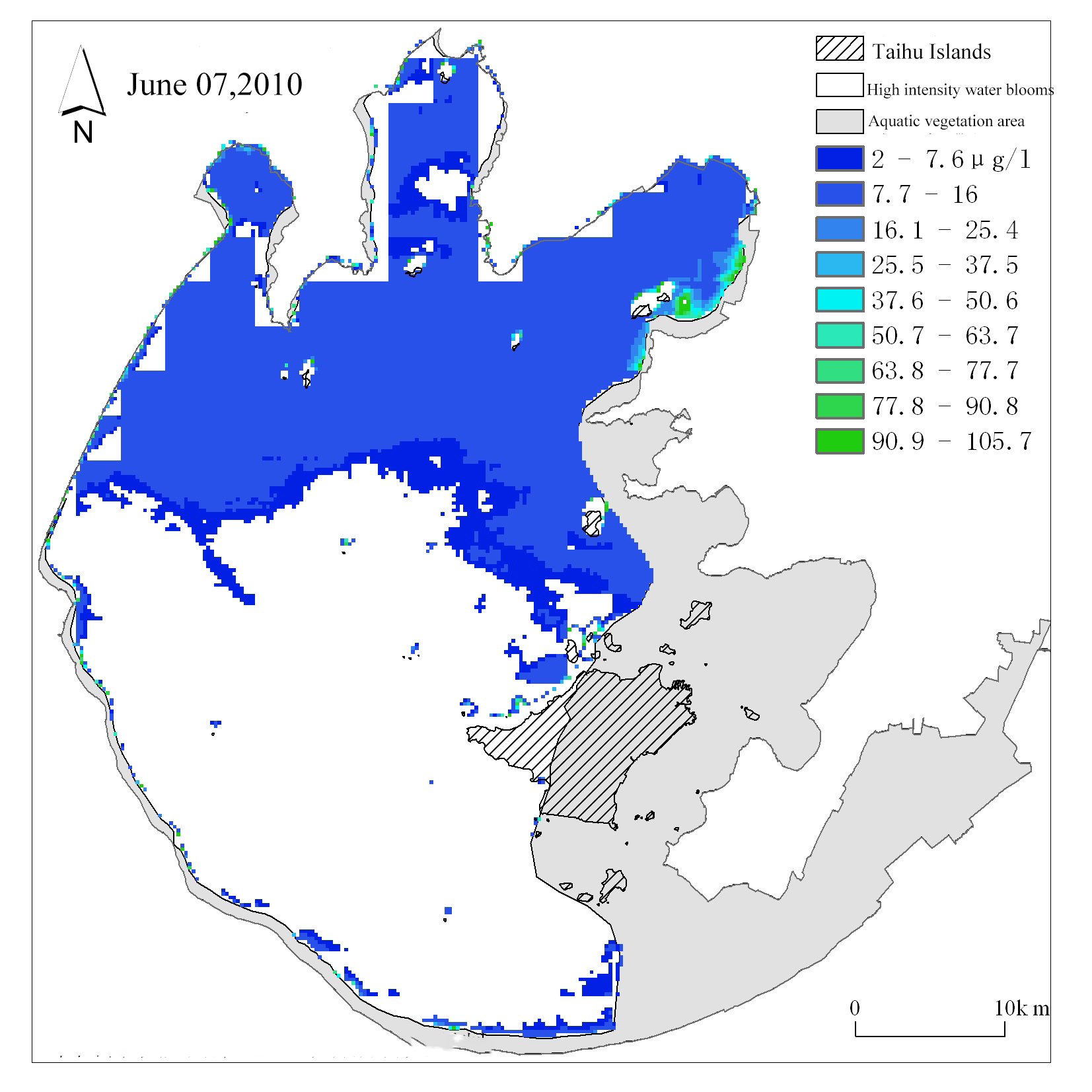

Supplement: Supplemental Information 2 — The data were obtained from the remote sensing image data of chlorophyll a concentration from the Lake-Watershed Science SubCenter, National Earth System Science Data Center, National Science & Technology Infrastructure of China, which had inconsistent data scales, data anomalies and different sampling intervals, and the chlorophyll a concentration unit was µg/L. [file peerj-cs-09-1292-s002.zip › 201006070518_taihu_chla.jpg]

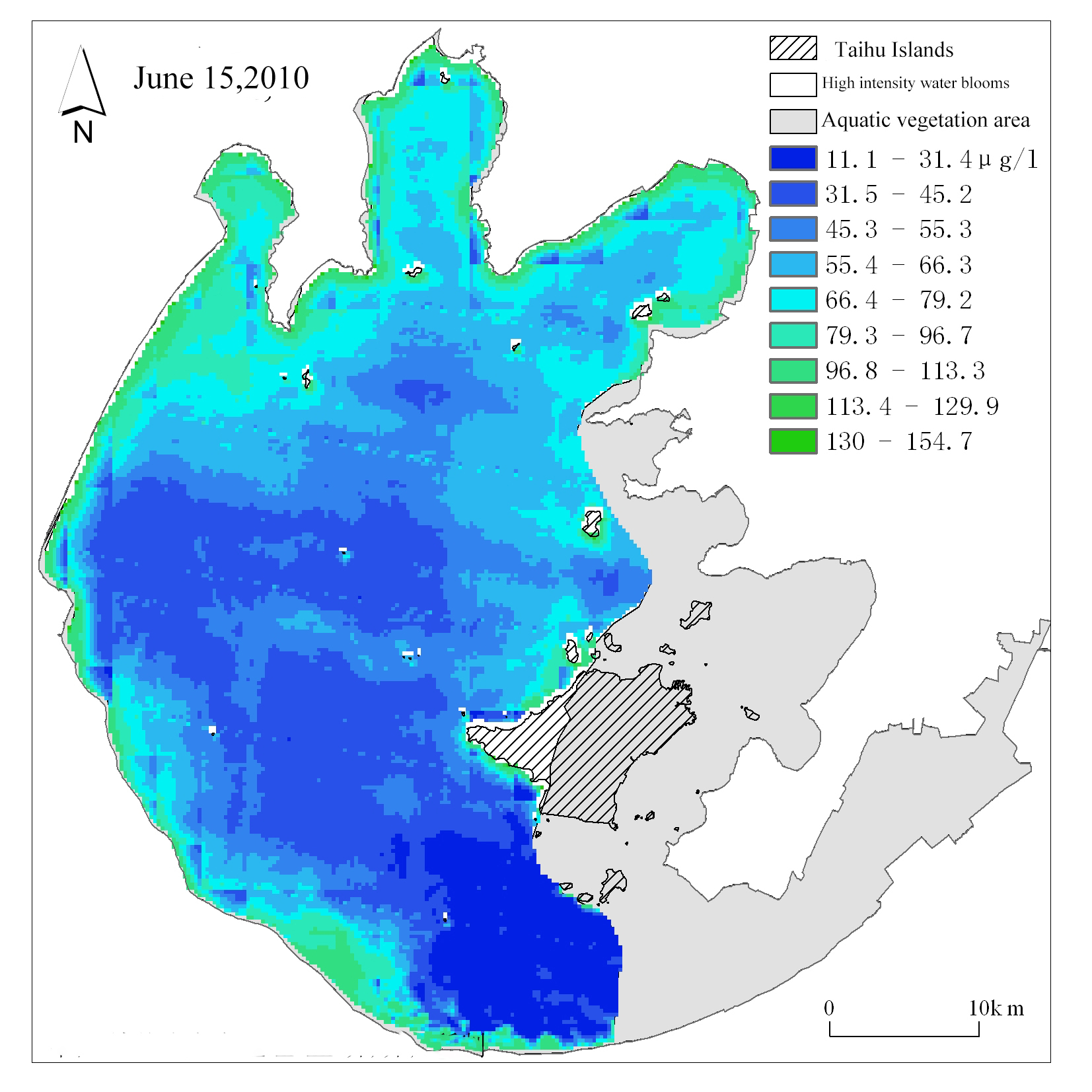

Supplement: Supplemental Information 2 — The data were obtained from the remote sensing image data of chlorophyll a concentration from the Lake-Watershed Science SubCenter, National Earth System Science Data Center, National Science & Technology Infrastructure of China, which had inconsistent data scales, data anomalies and different sampling intervals, and the chlorophyll a concentration unit was µg/L. [file peerj-cs-09-1292-s002.zip › 201006150258_taihu_chla.jpg]

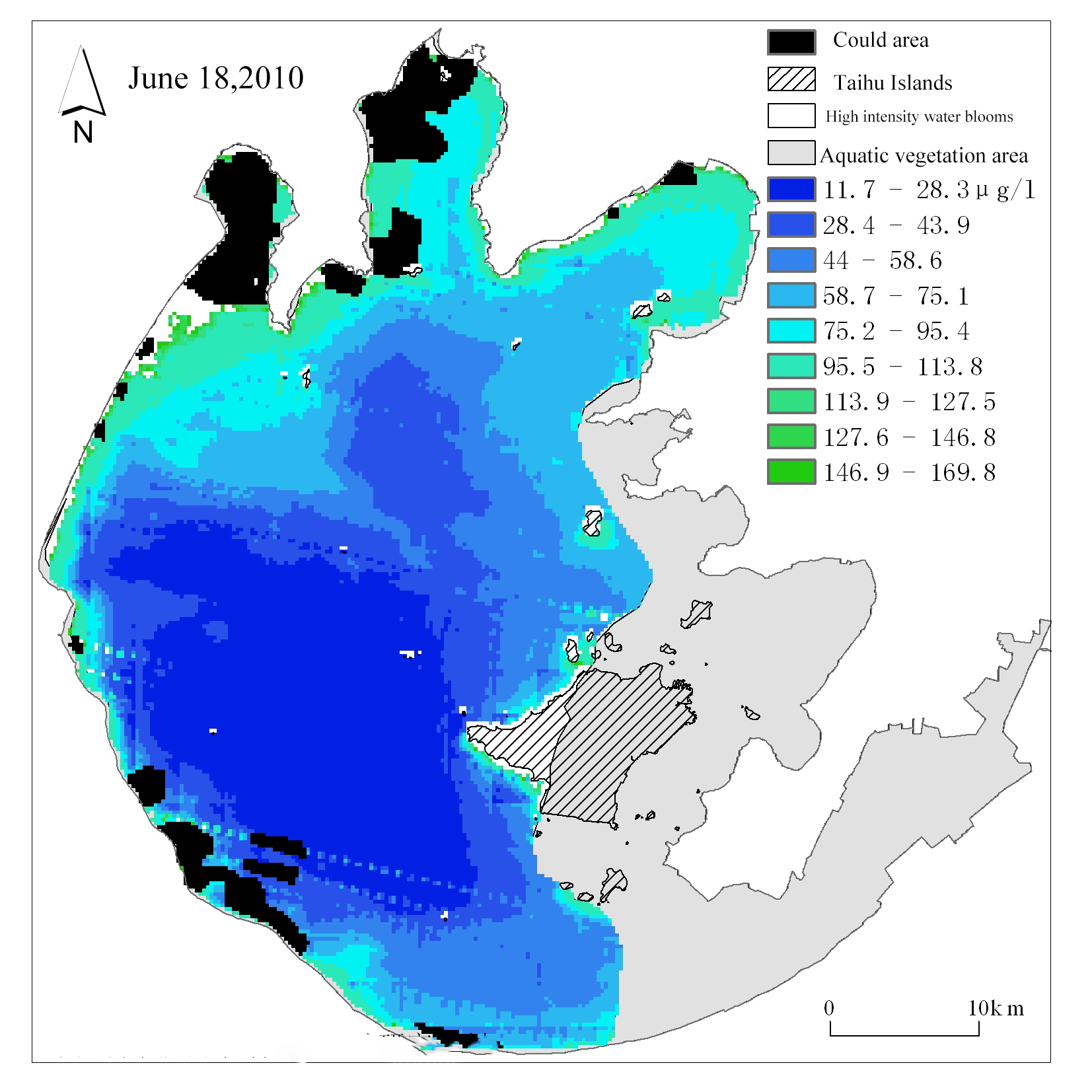

Supplement: Supplemental Information 2 — The data were obtained from the remote sensing image data of chlorophyll a concentration from the Lake-Watershed Science SubCenter, National Earth System Science Data Center, National Science & Technology Infrastructure of China, which had inconsistent data scales, data anomalies and different sampling intervals, and the chlorophyll a concentration unit was µg/L. [file peerj-cs-09-1292-s002.zip › 201006180327_taihu_chla.jpg]

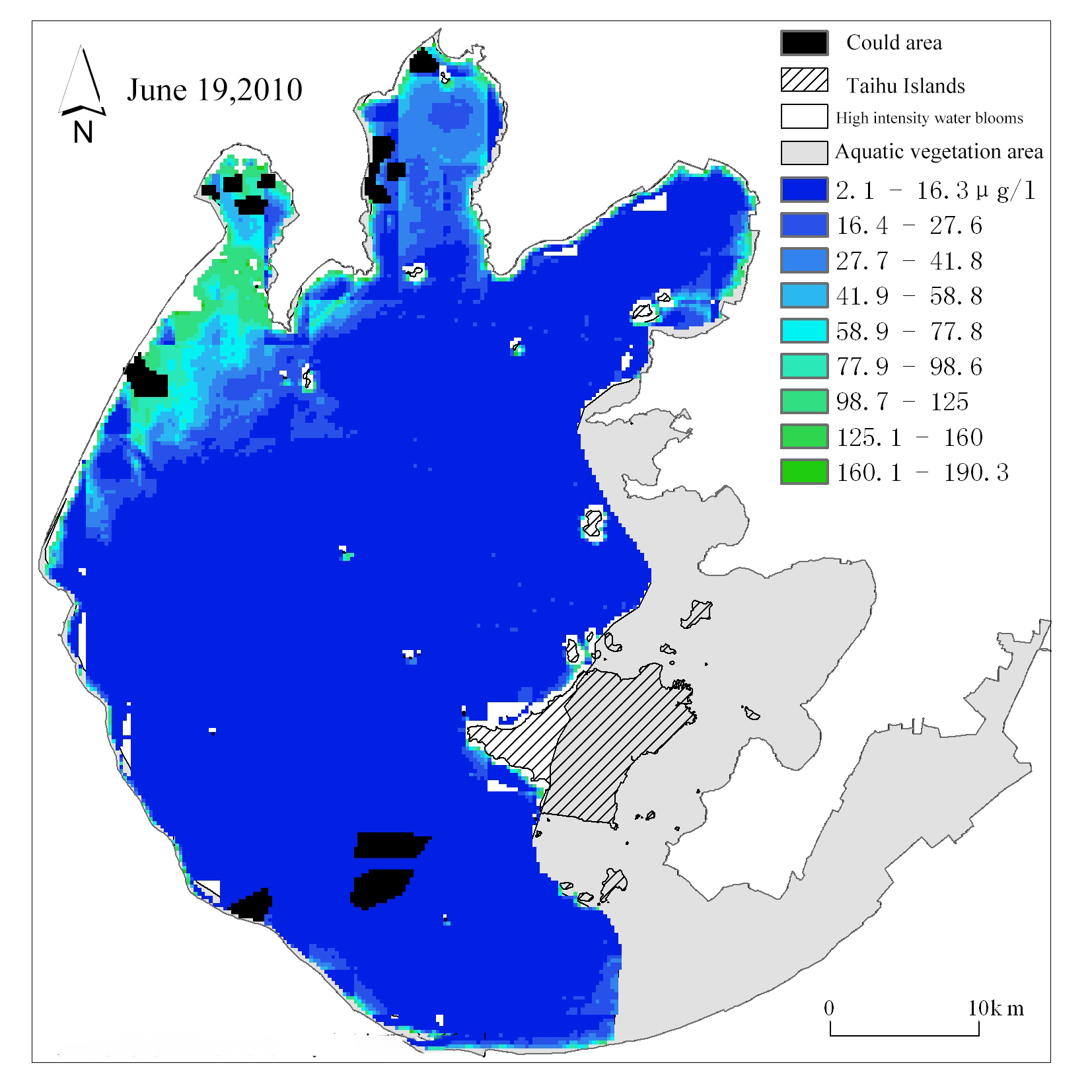

Supplement: Supplemental Information 2 — The data were obtained from the remote sensing image data of chlorophyll a concentration from the Lake-Watershed Science SubCenter, National Earth System Science Data Center, National Science & Technology Infrastructure of China, which had inconsistent data scales, data anomalies and different sampling intervals, and the chlorophyll a concentration unit was µg/L. [file peerj-cs-09-1292-s002.zip › 201006190322_taihu_chla.jpg]

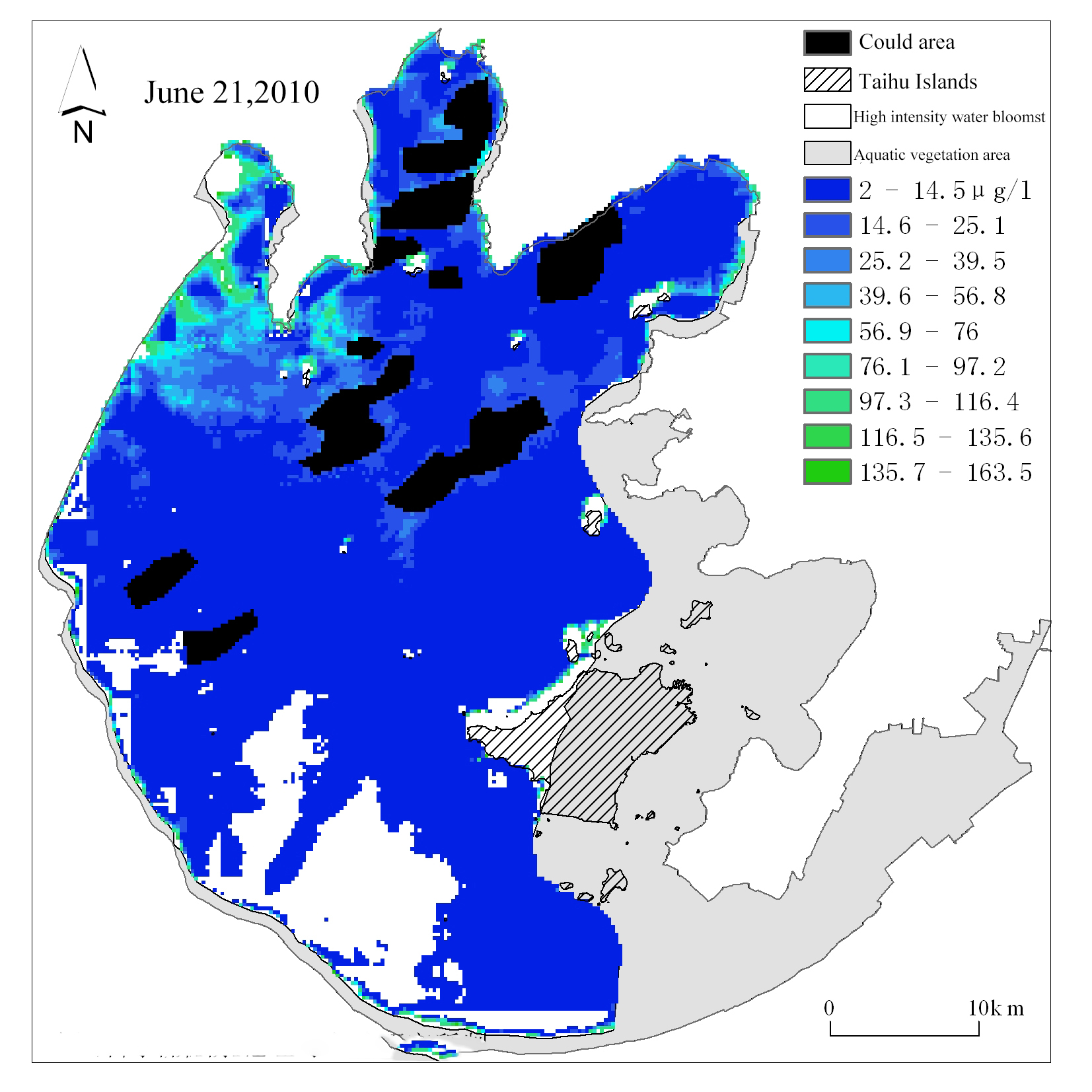

Supplement: Supplemental Information 2 — The data were obtained from the remote sensing image data of chlorophyll a concentration from the Lake-Watershed Science SubCenter, National Earth System Science Data Center, National Science & Technology Infrastructure of China, which had inconsistent data scales, data anomalies and different sampling intervals, and the chlorophyll a concentration unit was µg/L. [file peerj-cs-09-1292-s002.zip › 201006210530_taihu_chla.jpg]

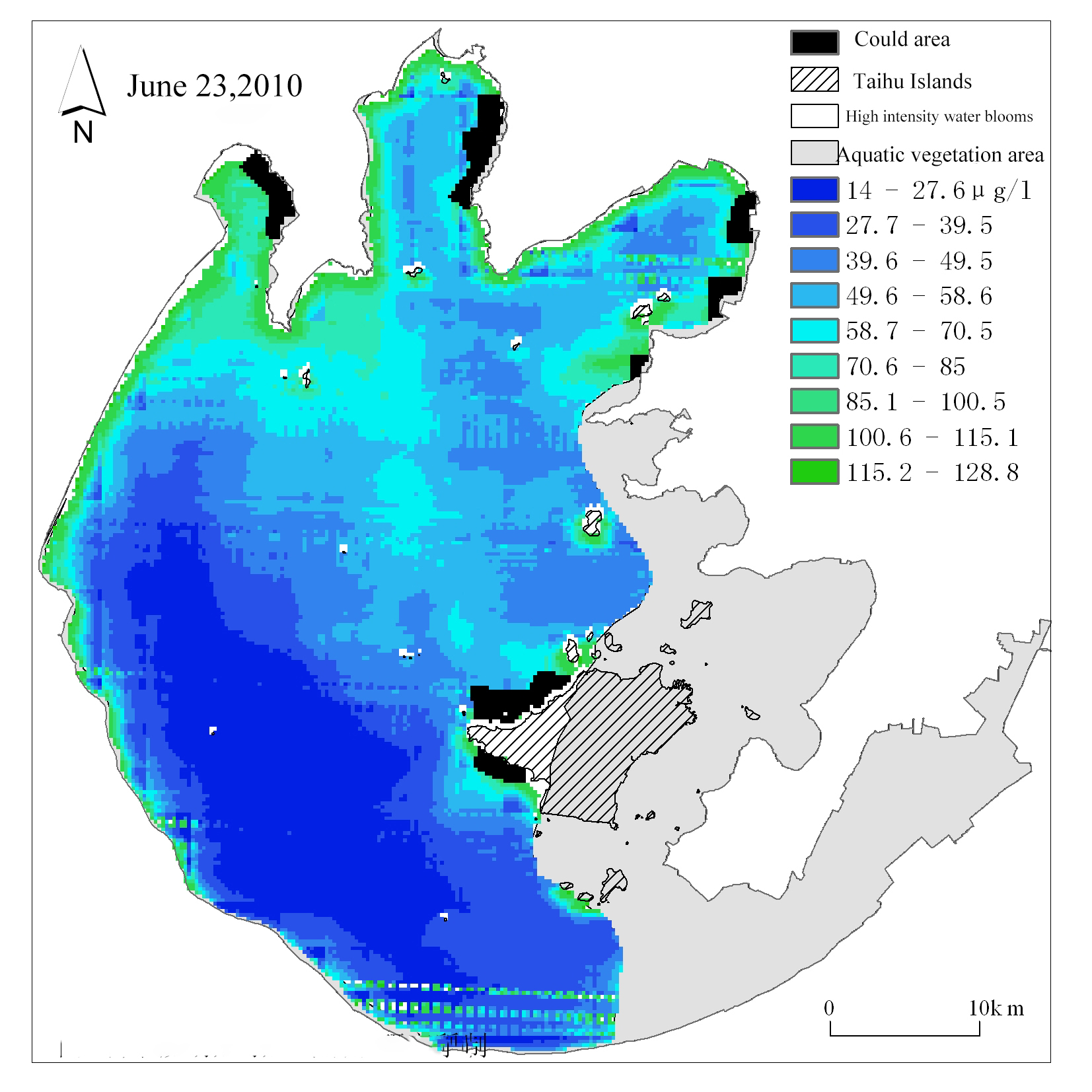

Supplement: Supplemental Information 2 — The data were obtained from the remote sensing image data of chlorophyll a concentration from the Lake-Watershed Science SubCenter, National Earth System Science Data Center, National Science & Technology Infrastructure of China, which had inconsistent data scales, data anomalies and different sampling intervals, and the chlorophyll a concentration unit was µg/L. [file peerj-cs-09-1292-s002.zip › 201006230209_taihu_chla.jpg]

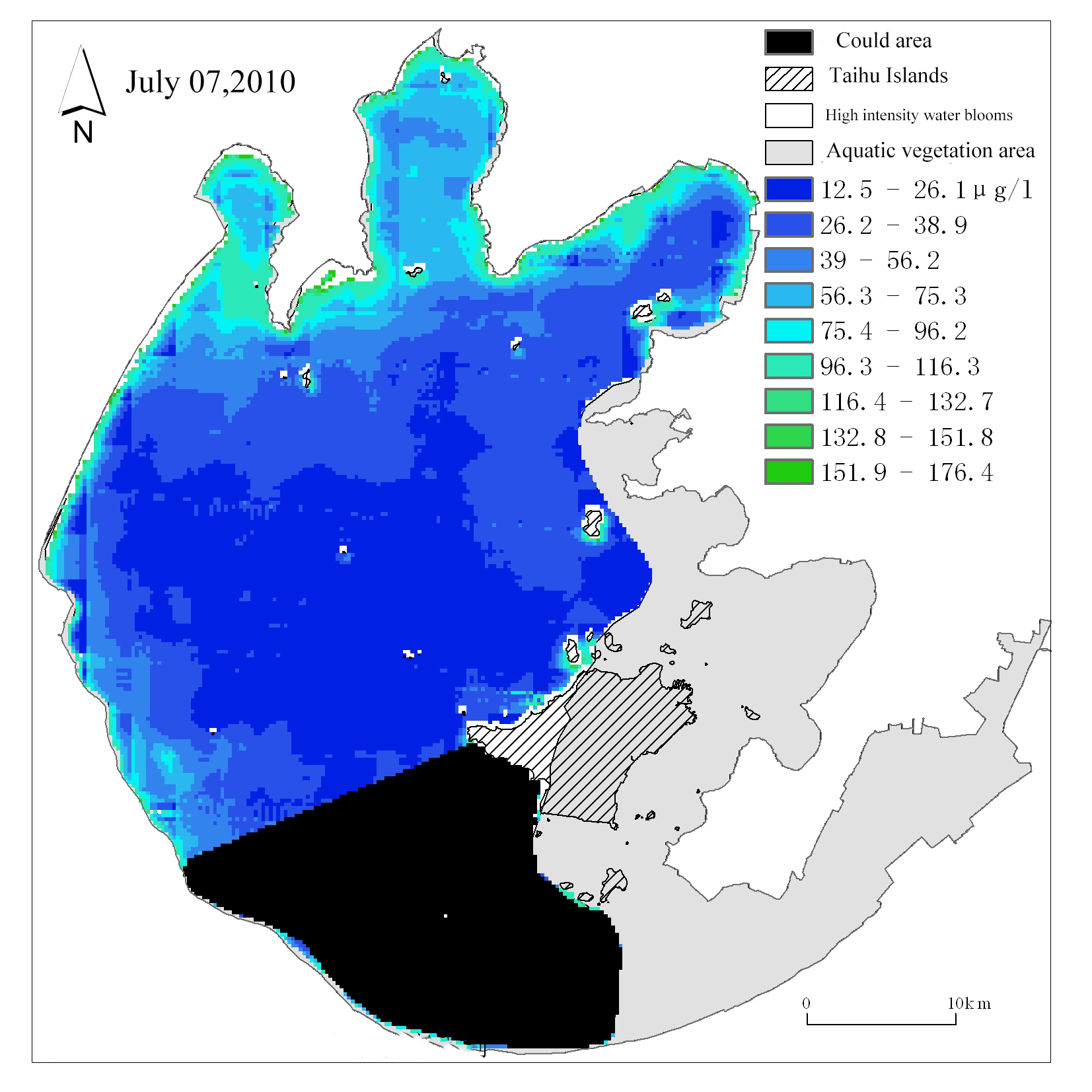

Supplement: Supplemental Information 2 — The data were obtained from the remote sensing image data of chlorophyll a concentration from the Lake-Watershed Science SubCenter, National Earth System Science Data Center, National Science & Technology Infrastructure of China, which had inconsistent data scales, data anomalies and different sampling intervals, and the chlorophyll a concentration unit was µg/L. [file peerj-cs-09-1292-s002.zip › 201007070221_taihu_chla.jpg]

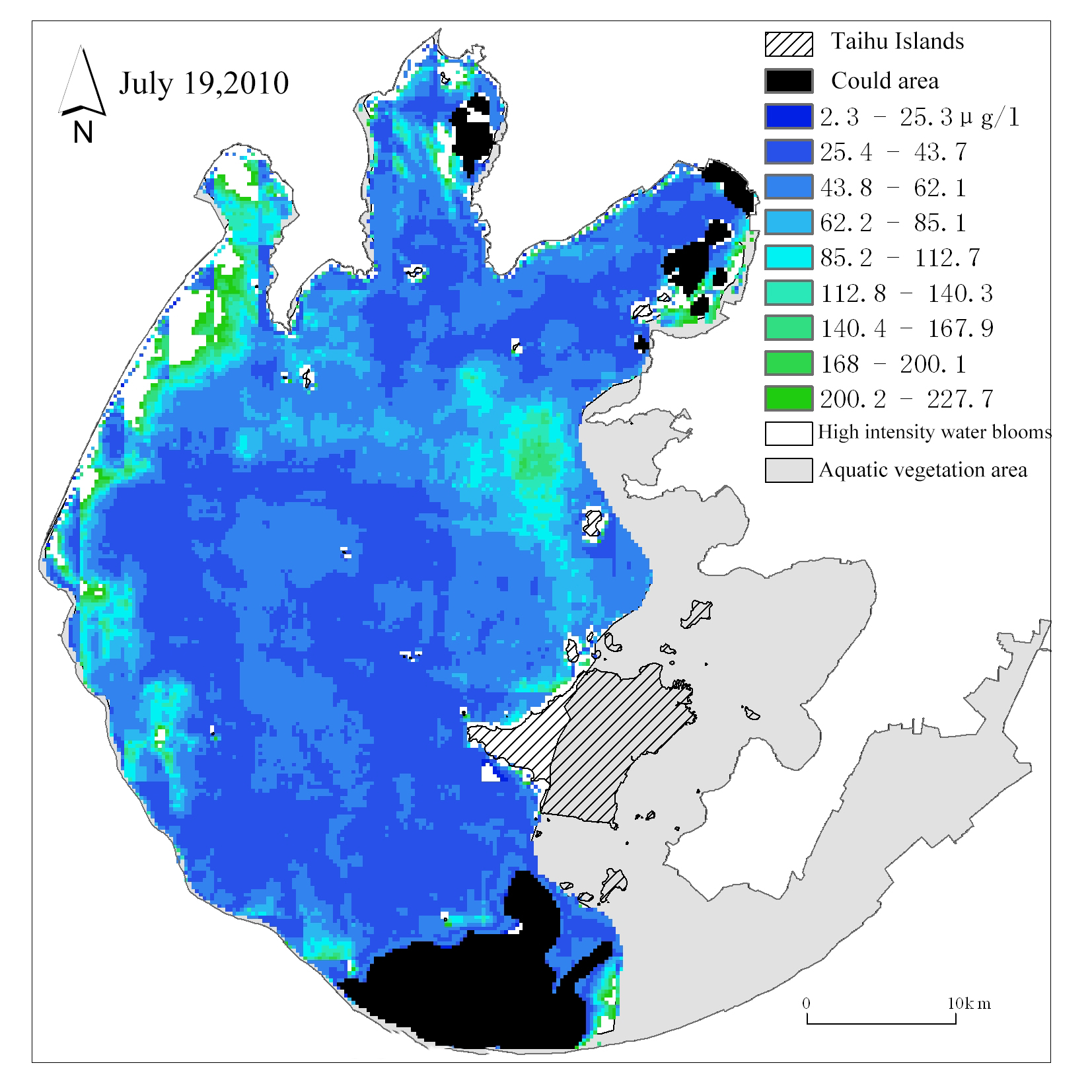

Supplement: Supplemental Information 3 — The data were obtained from the remote sensing image data of chlorophyll a concentration from the Lake-Watershed Science SubCenter, National Earth System Science Data Center, National Science & Technology Infrastructure of China, which had inconsistent data scales, data anomalies and different sampling intervals, and the chlorophyll a concentration unit was µg/L. [file peerj-cs-09-1292-s003.zip › 201007190244_taihu_chla.jpg]

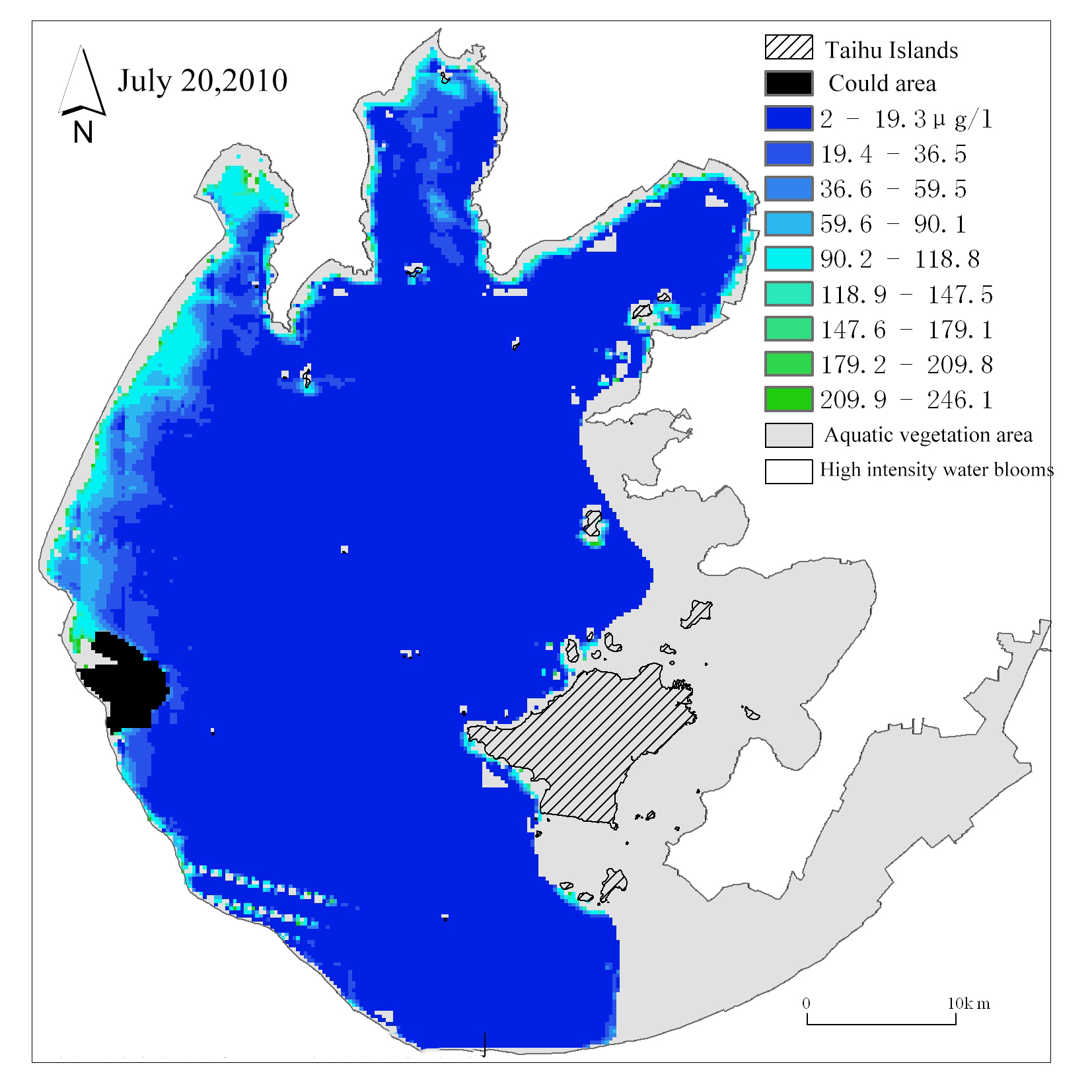

Supplement: Supplemental Information 3 — The data were obtained from the remote sensing image data of chlorophyll a concentration from the Lake-Watershed Science SubCenter, National Earth System Science Data Center, National Science & Technology Infrastructure of China, which had inconsistent data scales, data anomalies and different sampling intervals, and the chlorophyll a concentration unit was µg/L. [file peerj-cs-09-1292-s003.zip › 201007200329_taihu_chla.jpg]

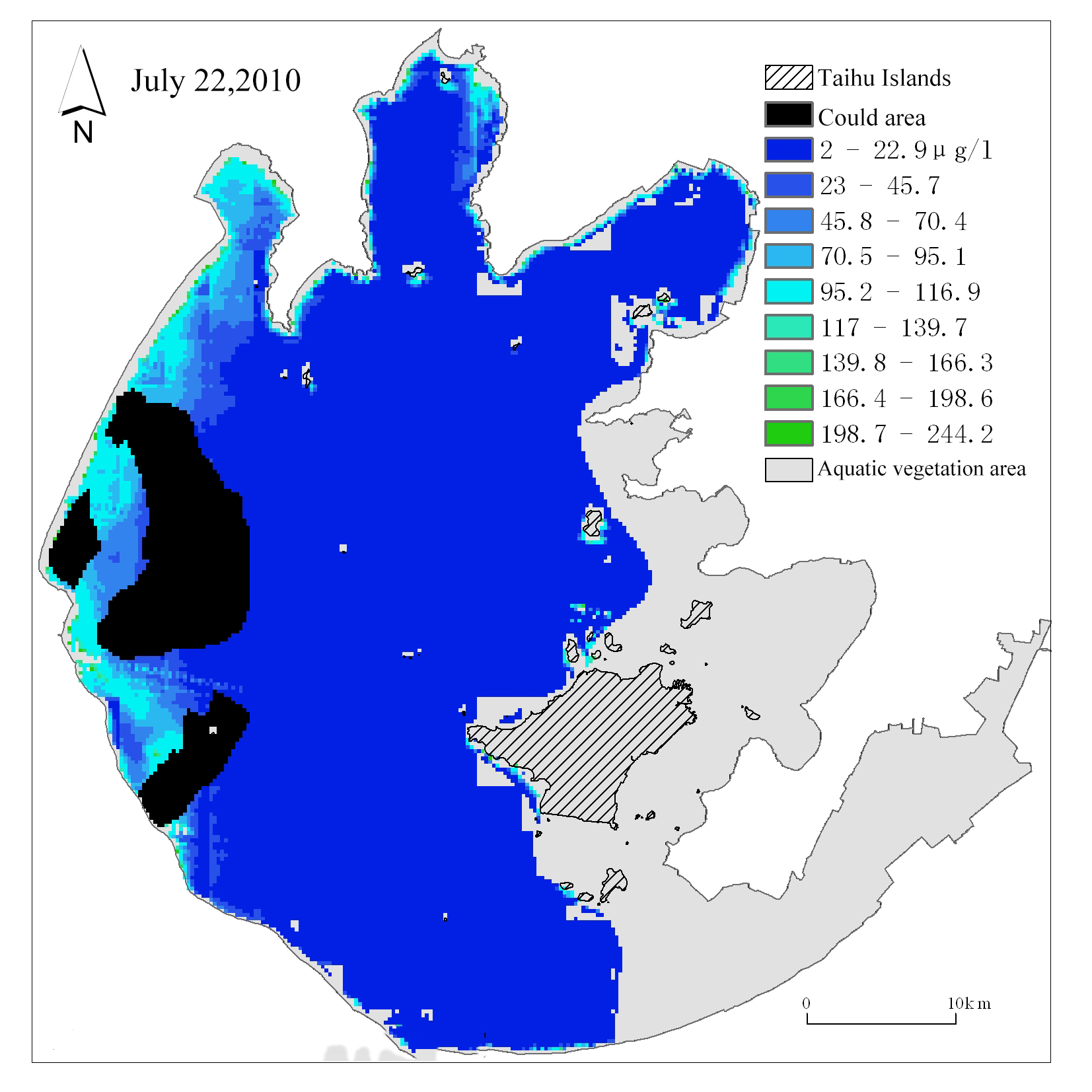

Supplement: Supplemental Information 3 — The data were obtained from the remote sensing image data of chlorophyll a concentration from the Lake-Watershed Science SubCenter, National Earth System Science Data Center, National Science & Technology Infrastructure of China, which had inconsistent data scales, data anomalies and different sampling intervals, and the chlorophyll a concentration unit was µg/L. [file peerj-cs-09-1292-s003.zip › 201007221111_taihu_chl-a.jpg]

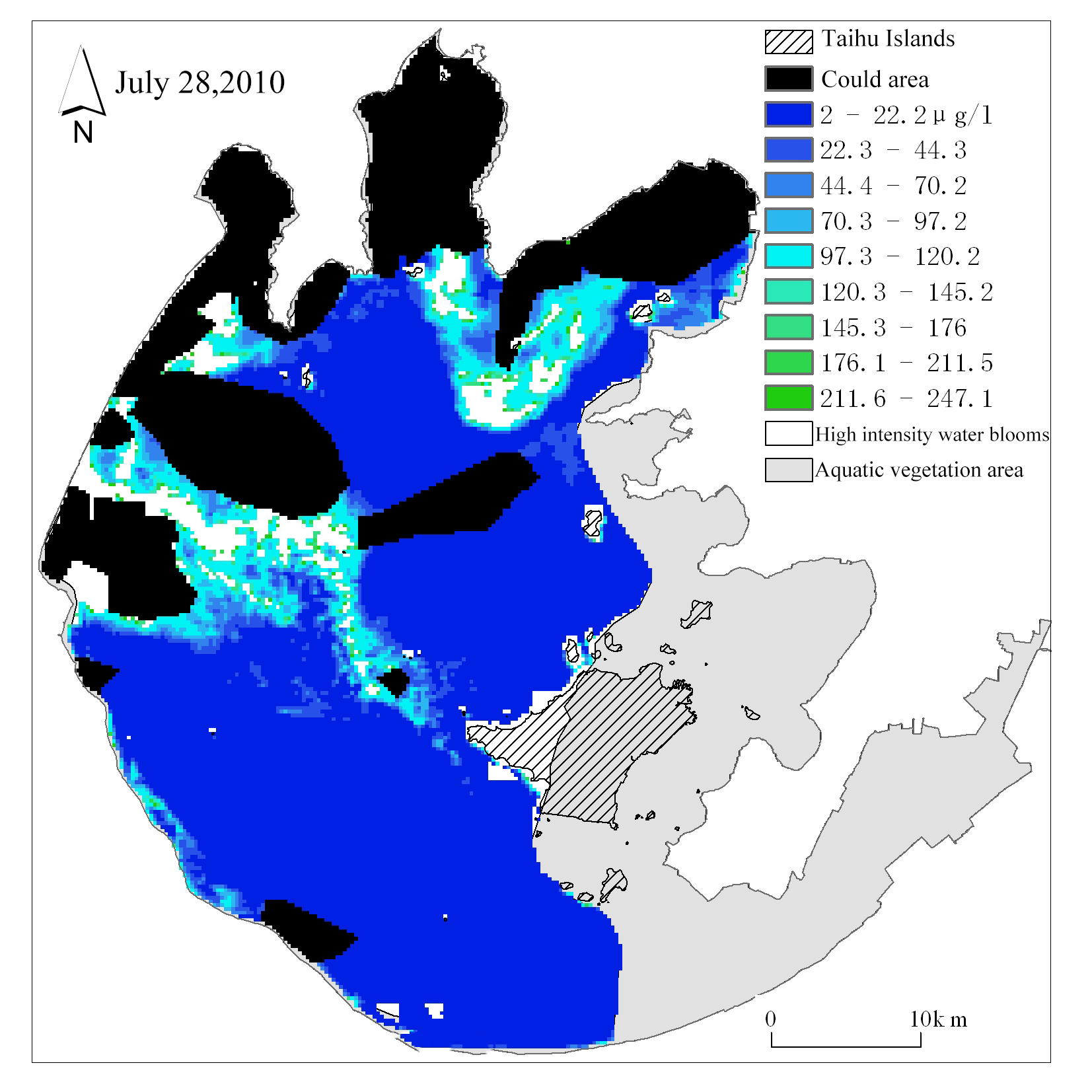

Supplement: Supplemental Information 3 — The data were obtained from the remote sensing image data of chlorophyll a concentration from the Lake-Watershed Science SubCenter, National Earth System Science Data Center, National Science & Technology Infrastructure of China, which had inconsistent data scales, data anomalies and different sampling intervals, and the chlorophyll a concentration unit was µg/L. [file peerj-cs-09-1292-s003.zip › 201007281039_taihu_chl-a.jpg]

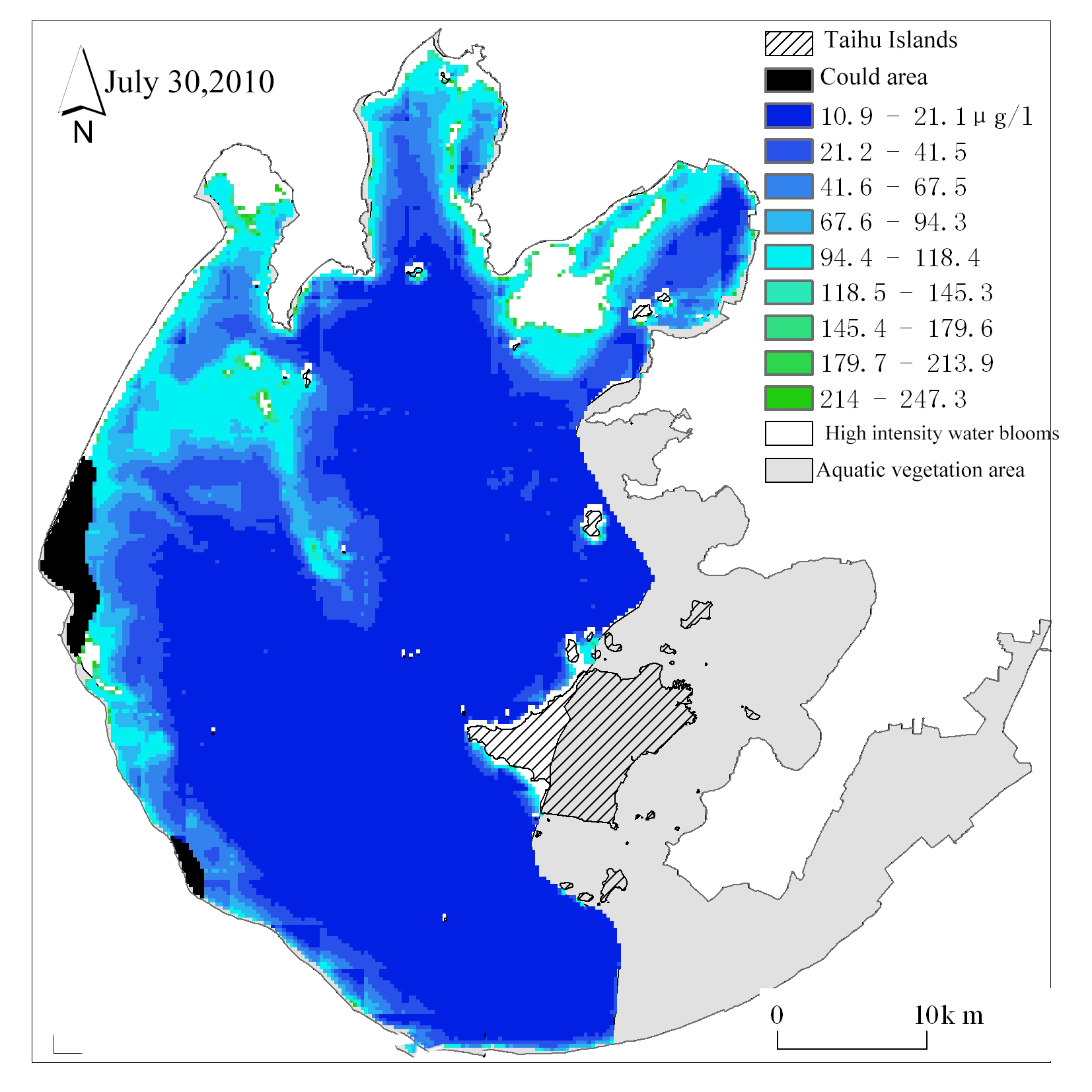

Supplement: Supplemental Information 3 — The data were obtained from the remote sensing image data of chlorophyll a concentration from the Lake-Watershed Science SubCenter, National Earth System Science Data Center, National Science & Technology Infrastructure of China, which had inconsistent data scales, data anomalies and different sampling intervals, and the chlorophyll a concentration unit was µg/L. [file peerj-cs-09-1292-s003.zip › 201007301022_taihu_chl-a.jpg]

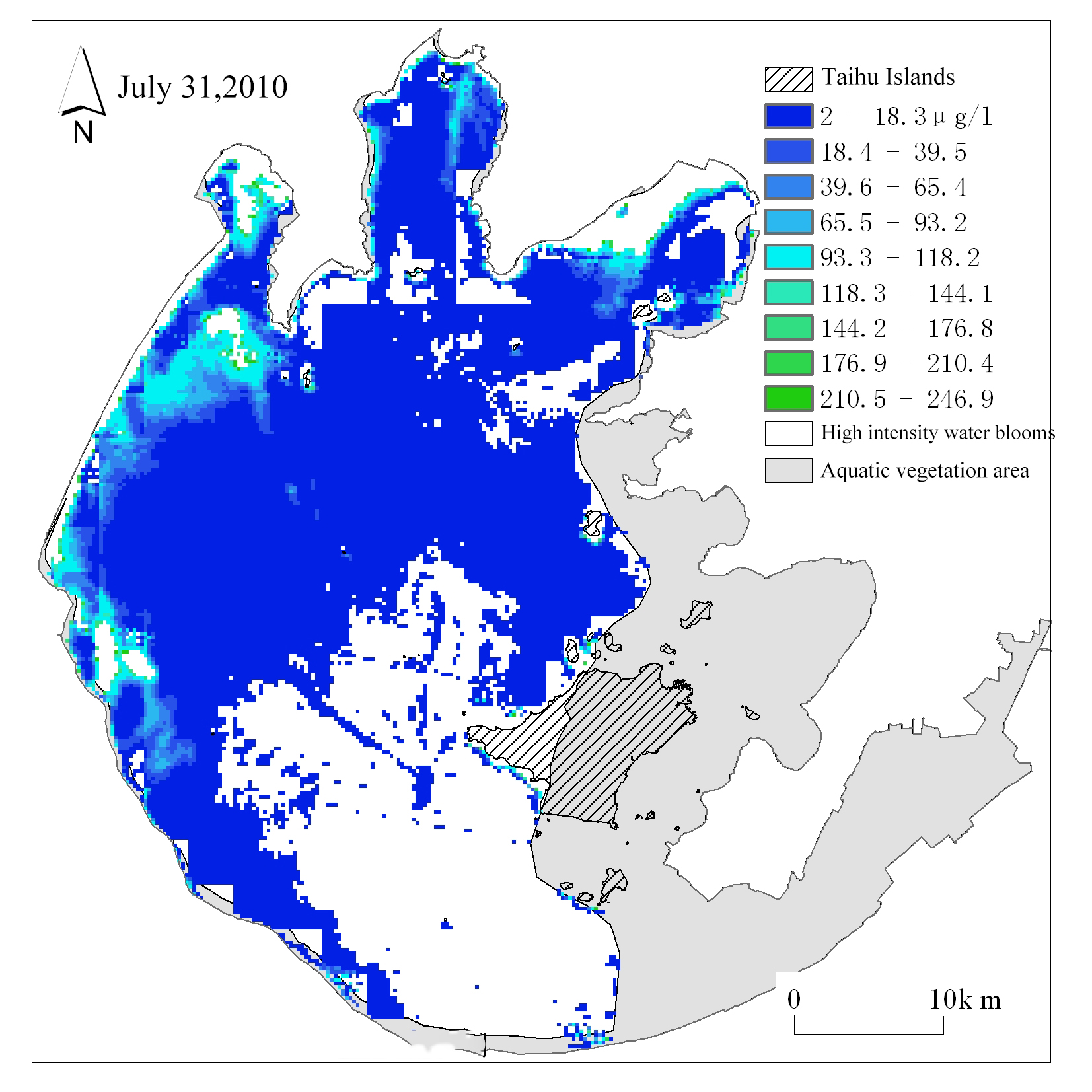

Supplement: Supplemental Information 3 — The data were obtained from the remote sensing image data of chlorophyll a concentration from the Lake-Watershed Science SubCenter, National Earth System Science Data Center, National Science & Technology Infrastructure of China, which had inconsistent data scales, data anomalies and different sampling intervals, and the chlorophyll a concentration unit was µg/L. [file peerj-cs-09-1292-s003.zip › 201007311105_taihu_chla.jpg]

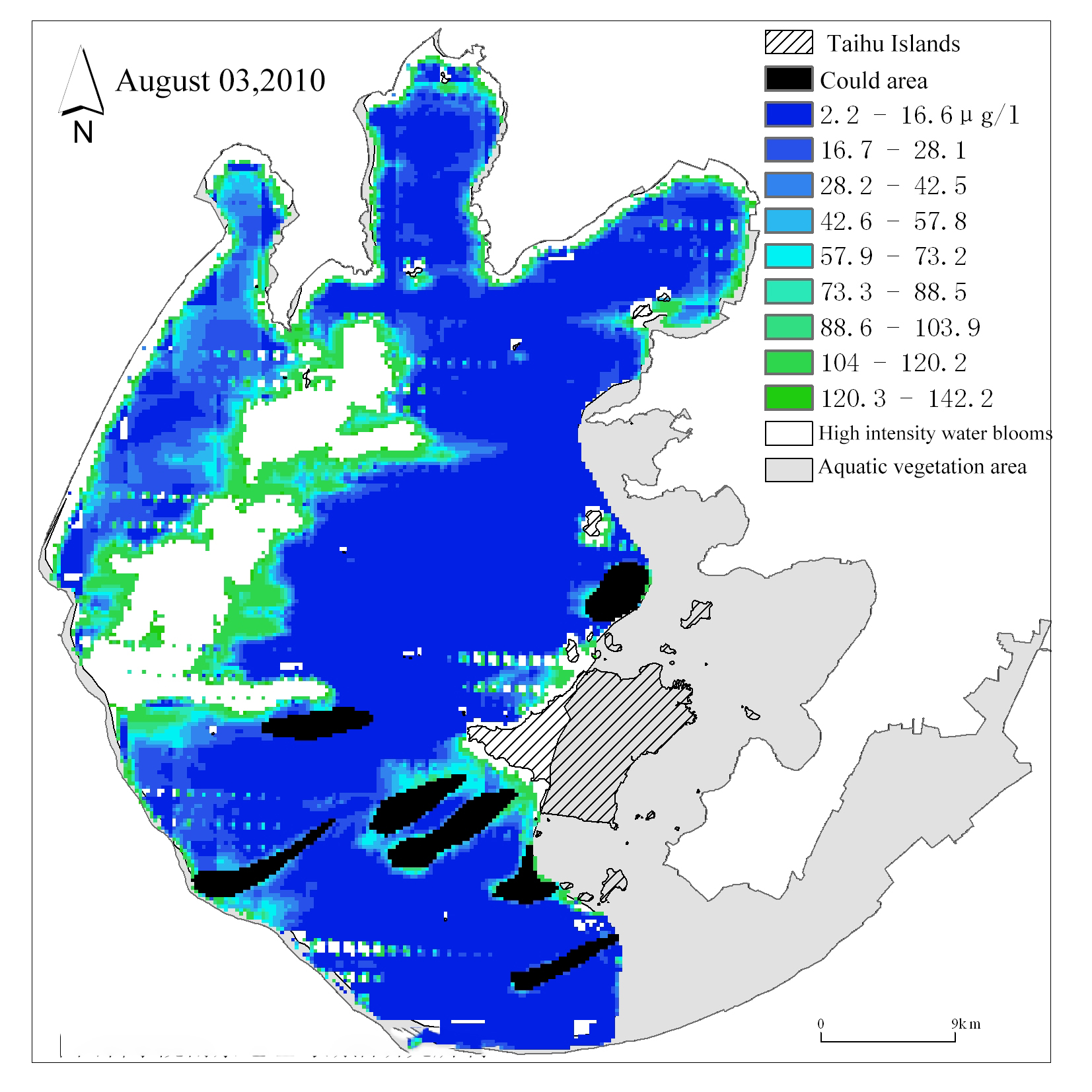

Supplement: Supplemental Information 3 — The data were obtained from the remote sensing image data of chlorophyll a concentration from the Lake-Watershed Science SubCenter, National Earth System Science Data Center, National Science & Technology Infrastructure of China, which had inconsistent data scales, data anomalies and different sampling intervals, and the chlorophyll a concentration unit was µg/L. [file peerj-cs-09-1292-s003.zip › 201008030203_taihu_chla.jpg]

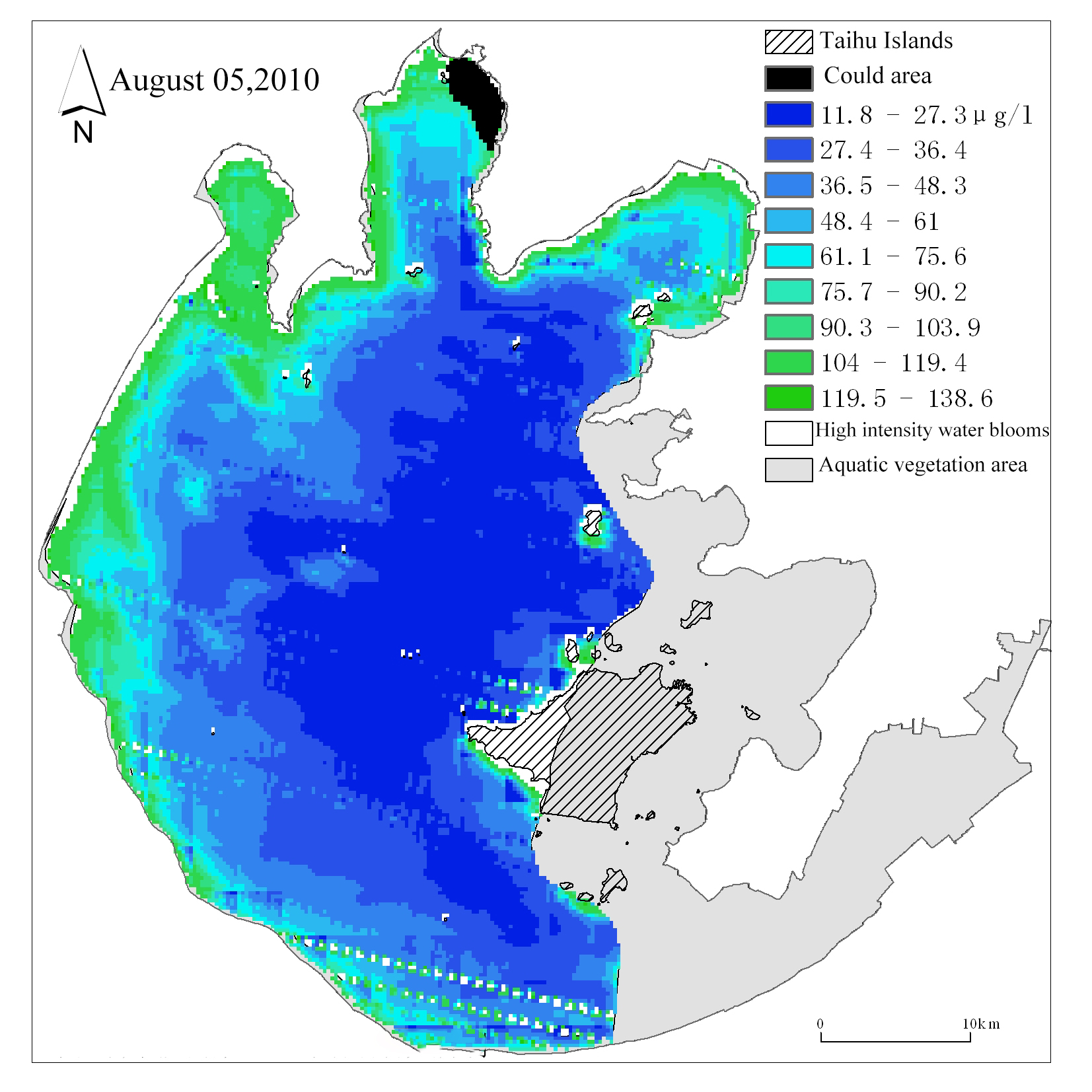

Supplement: Supplemental Information 3 — The data were obtained from the remote sensing image data of chlorophyll a concentration from the Lake-Watershed Science SubCenter, National Earth System Science Data Center, National Science & Technology Infrastructure of China, which had inconsistent data scales, data anomalies and different sampling intervals, and the chlorophyll a concentration unit was µg/L. [file peerj-cs-09-1292-s003.zip › 201008050327_taihu_chla.jpg]

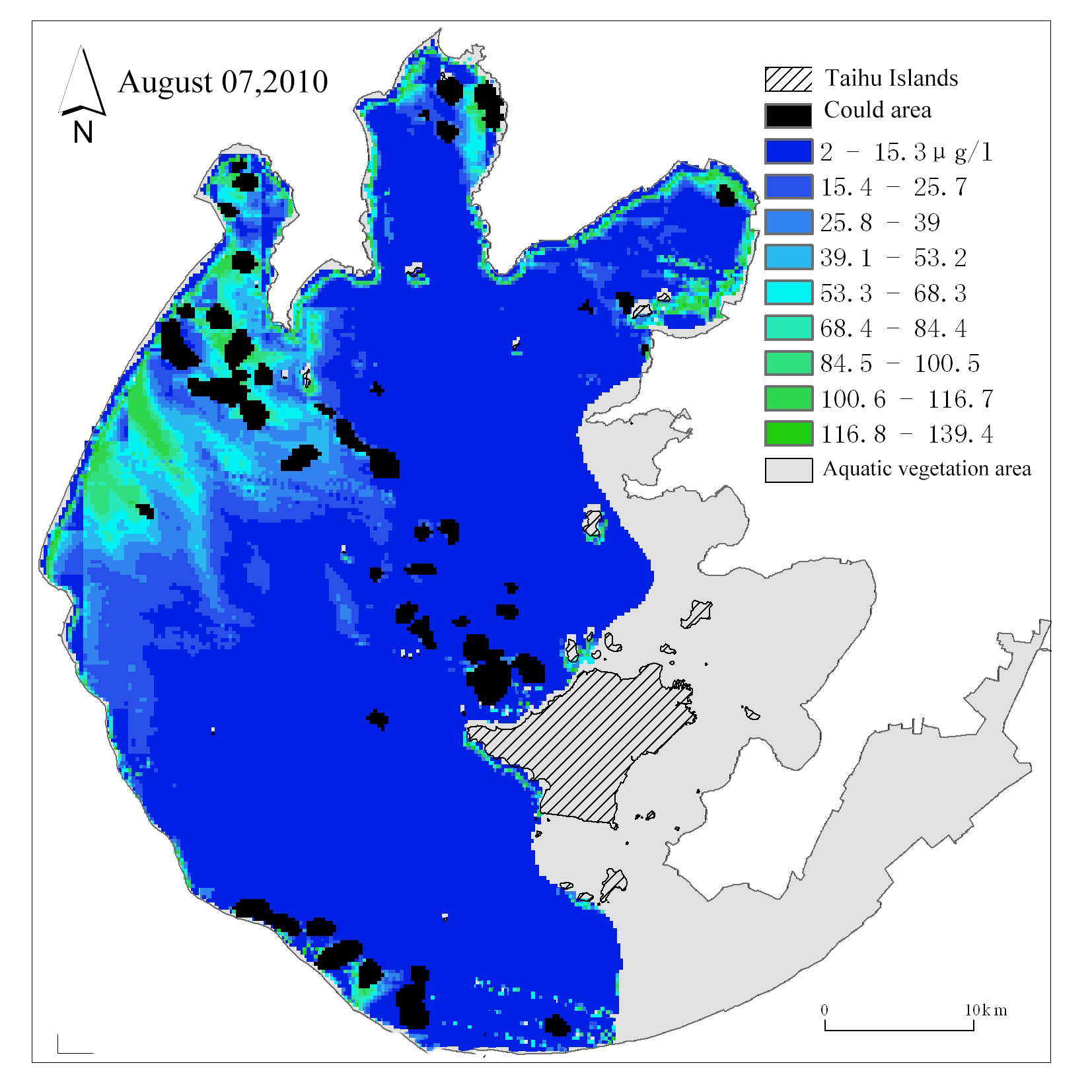

Supplement: Supplemental Information 3 — The data were obtained from the remote sensing image data of chlorophyll a concentration from the Lake-Watershed Science SubCenter, National Earth System Science Data Center, National Science & Technology Infrastructure of China, which had inconsistent data scales, data anomalies and different sampling intervals, and the chlorophyll a concentration unit was µg/L. [file peerj-cs-09-1292-s003.zip › 201008070315_taihu_chla.jpg]

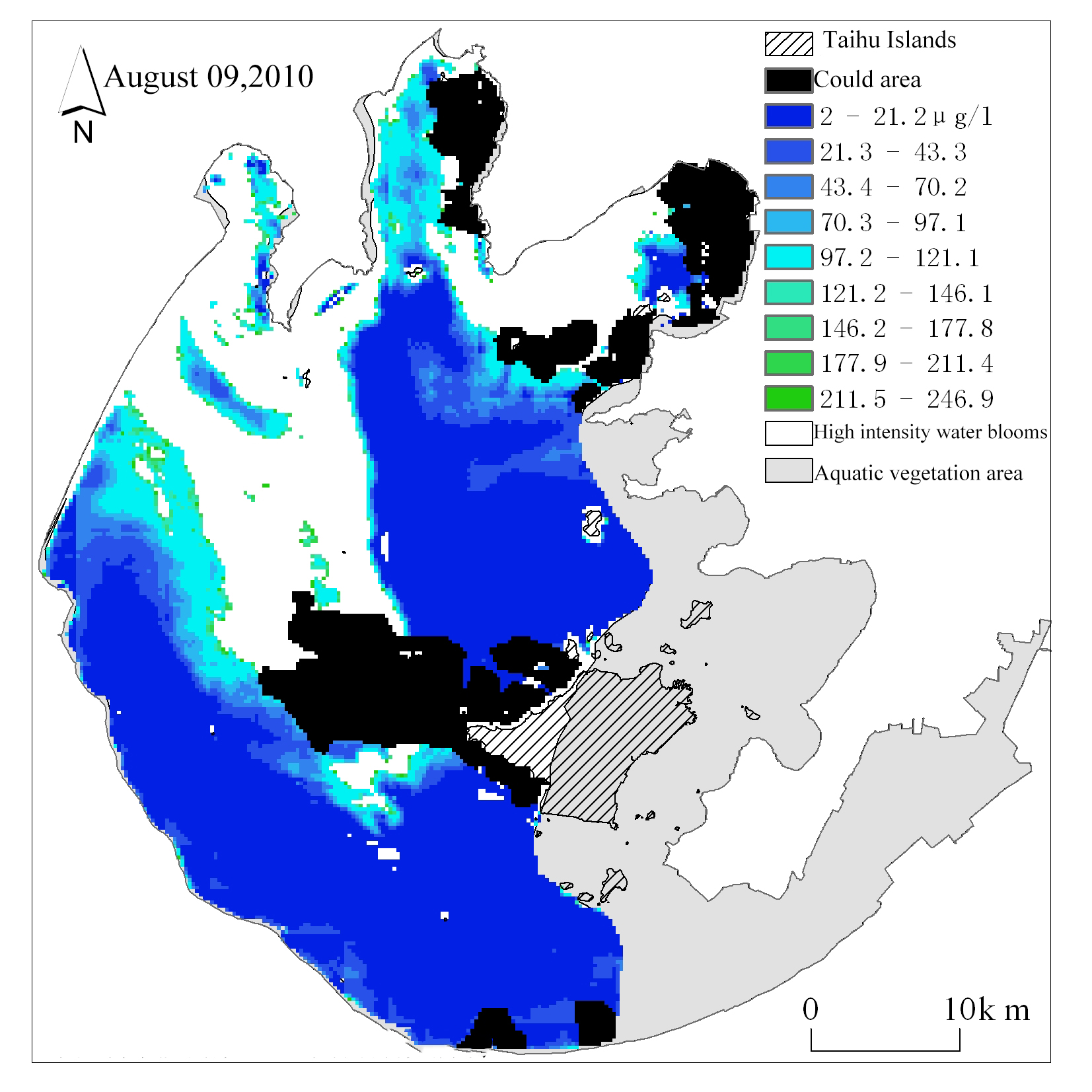

Supplement: Supplemental Information 3 — The data were obtained from the remote sensing image data of chlorophyll a concentration from the Lake-Watershed Science SubCenter, National Earth System Science Data Center, National Science & Technology Infrastructure of China, which had inconsistent data scales, data anomalies and different sampling intervals, and the chlorophyll a concentration unit was µg/L. [file peerj-cs-09-1292-s003.zip › 201008091102_taihu_chla.jpg]

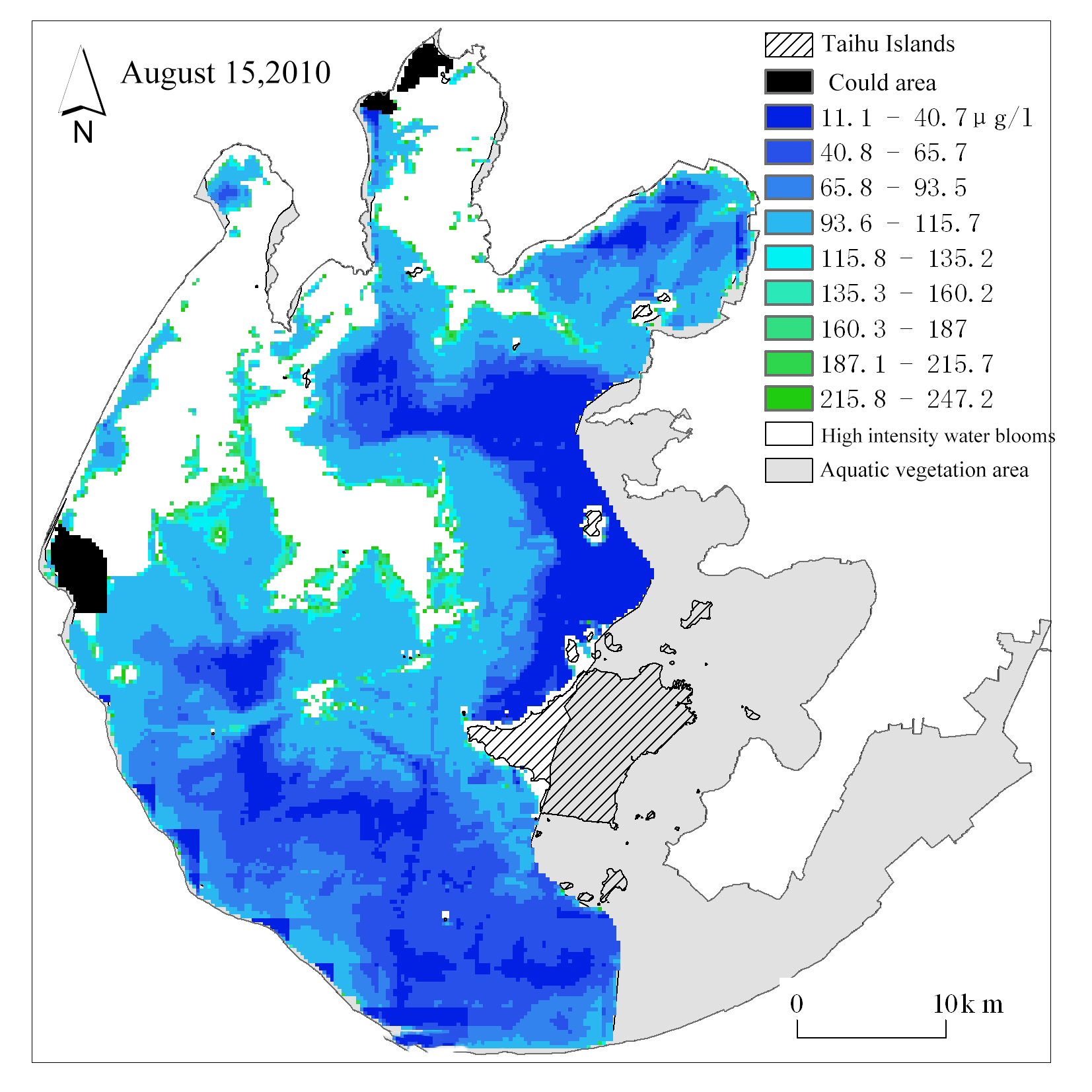

Supplement: Supplemental Information 3 — The data were obtained from the remote sensing image data of chlorophyll a concentration from the Lake-Watershed Science SubCenter, National Earth System Science Data Center, National Science & Technology Infrastructure of China, which had inconsistent data scales, data anomalies and different sampling intervals, and the chlorophyll a concentration unit was µg/L. [file peerj-cs-09-1292-s003.zip › 201008151027_taihu_chl-a.jpg]

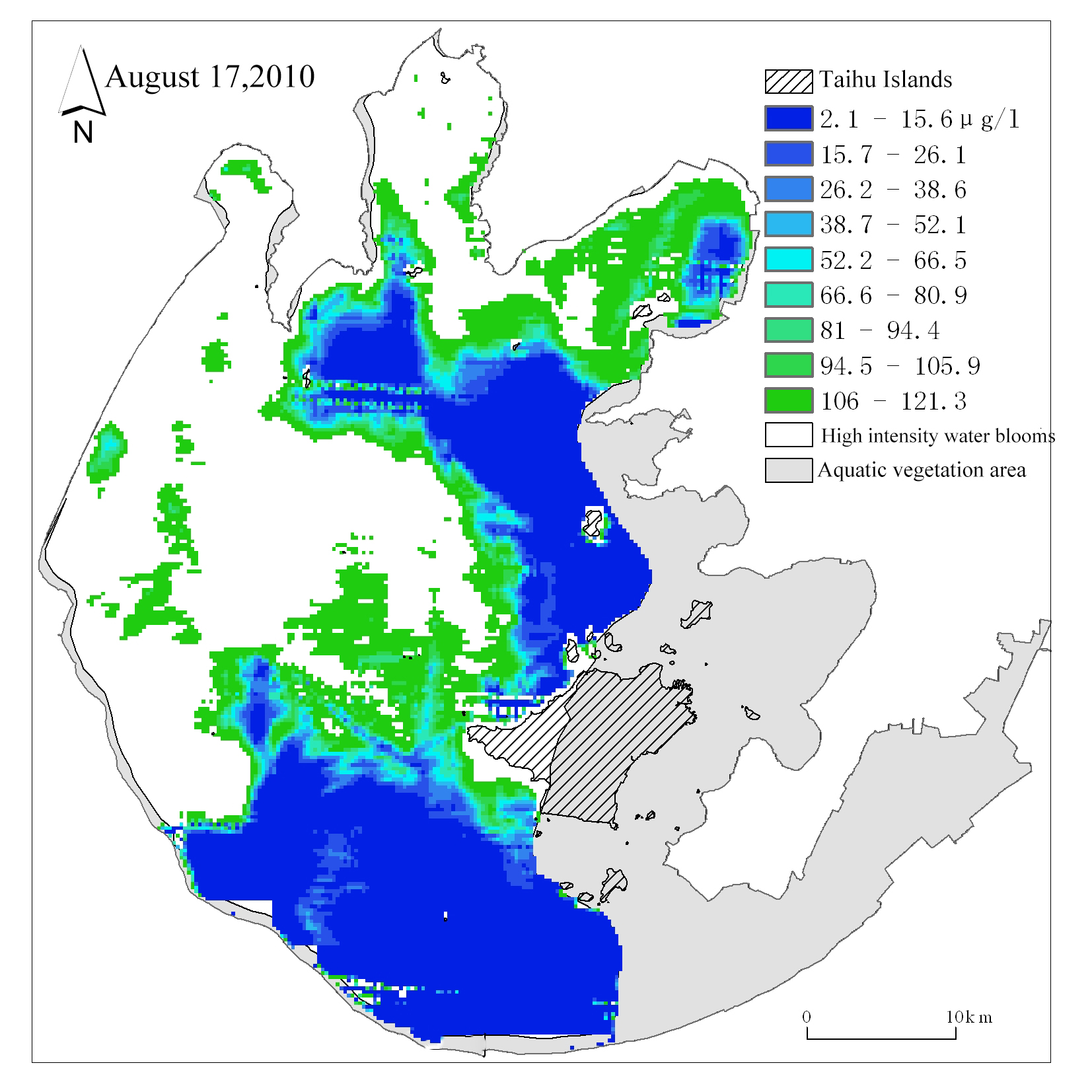

Supplement: Supplemental Information 3 — The data were obtained from the remote sensing image data of chlorophyll a concentration from the Lake-Watershed Science SubCenter, National Earth System Science Data Center, National Science & Technology Infrastructure of China, which had inconsistent data scales, data anomalies and different sampling intervals, and the chlorophyll a concentration unit was µg/L. [file peerj-cs-09-1292-s003.zip › 201008170214_tahihu_chla.jpg]

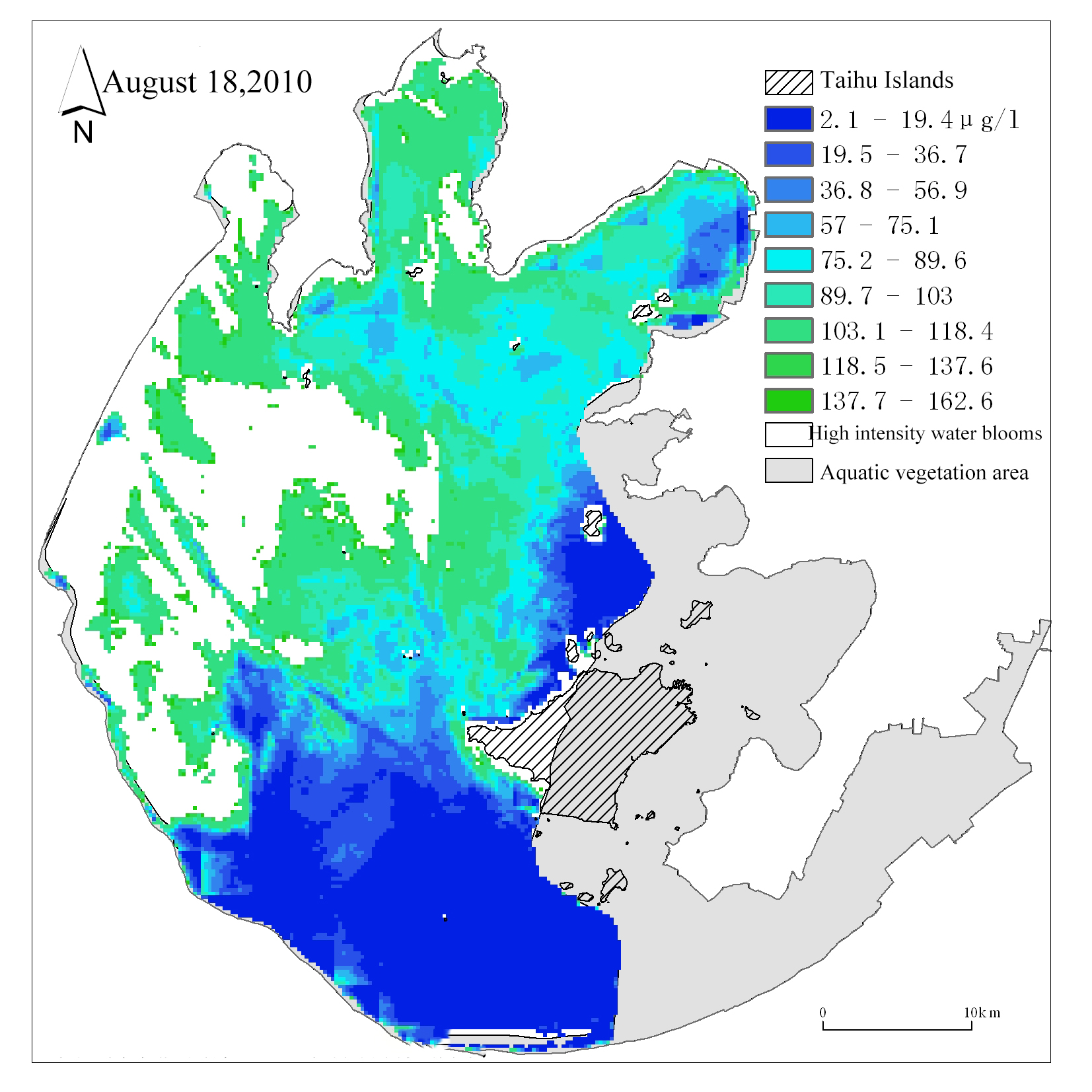

Supplement: Supplemental Information 3 — The data were obtained from the remote sensing image data of chlorophyll a concentration from the Lake-Watershed Science SubCenter, National Earth System Science Data Center, National Science & Technology Infrastructure of China, which had inconsistent data scales, data anomalies and different sampling intervals, and the chlorophyll a concentration unit was µg/L. [file peerj-cs-09-1292-s003.zip › 201008180256_taihu_chla.jpg]

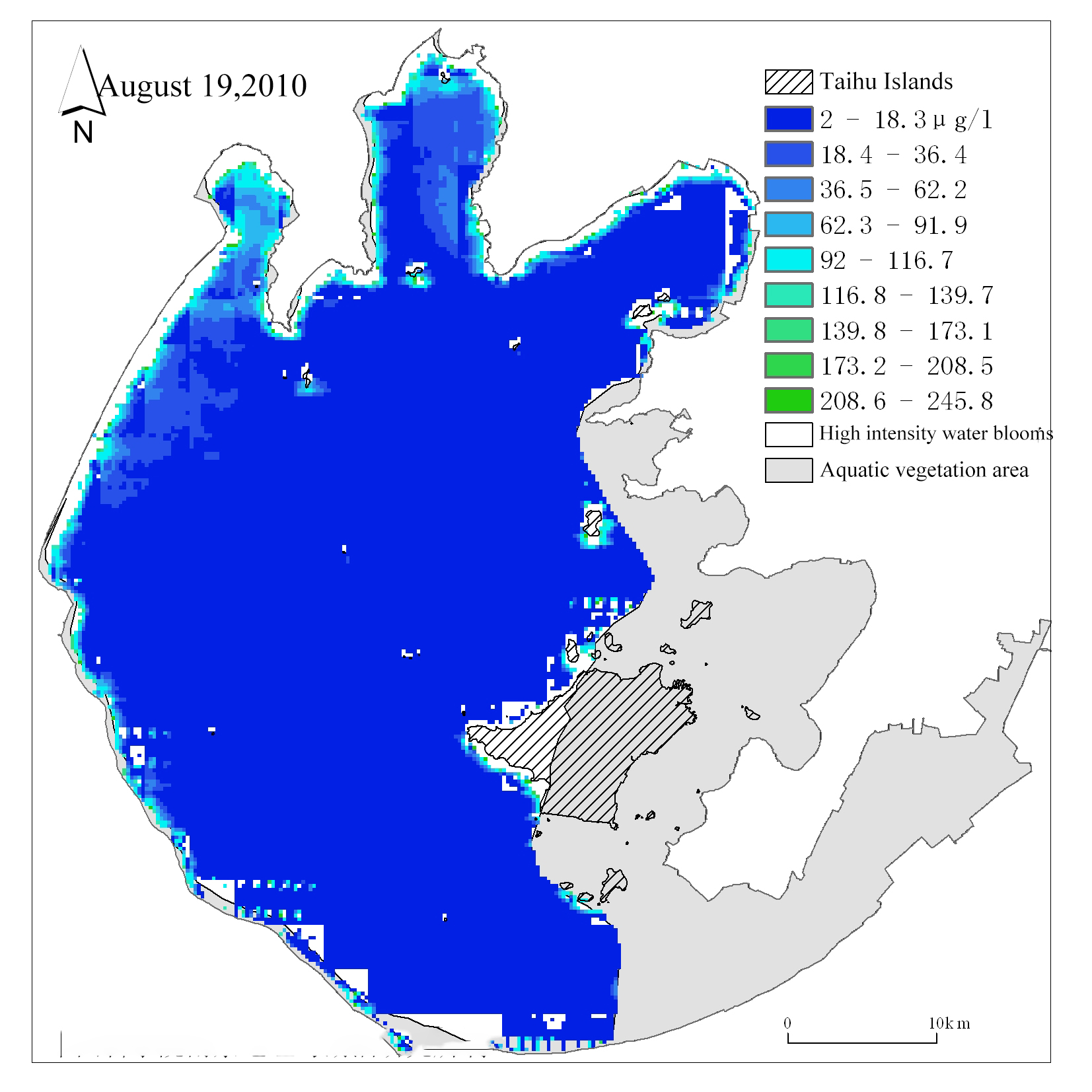

Supplement: Supplemental Information 3 — The data were obtained from the remote sensing image data of chlorophyll a concentration from the Lake-Watershed Science SubCenter, National Earth System Science Data Center, National Science & Technology Infrastructure of China, which had inconsistent data scales, data anomalies and different sampling intervals, and the chlorophyll a concentration unit was µg/L. [file peerj-cs-09-1292-s003.zip › 201008190202_taihu_chla.jpg]

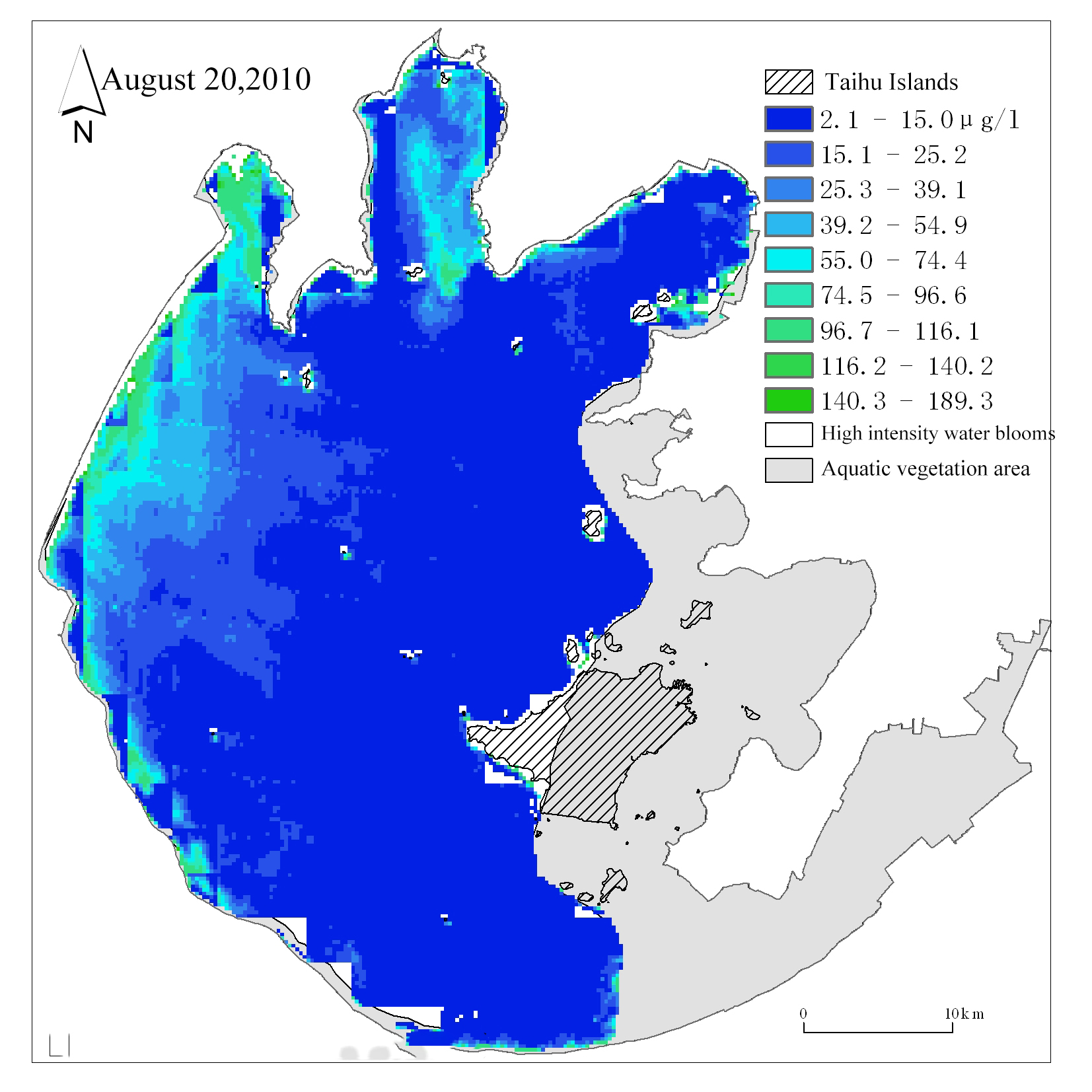

Supplement: Supplemental Information 3 — The data were obtained from the remote sensing image data of chlorophyll a concentration from the Lake-Watershed Science SubCenter, National Earth System Science Data Center, National Science & Technology Infrastructure of China, which had inconsistent data scales, data anomalies and different sampling intervals, and the chlorophyll a concentration unit was µg/L. [file peerj-cs-09-1292-s003.zip › 201008200244_taihu_chla.jpg]

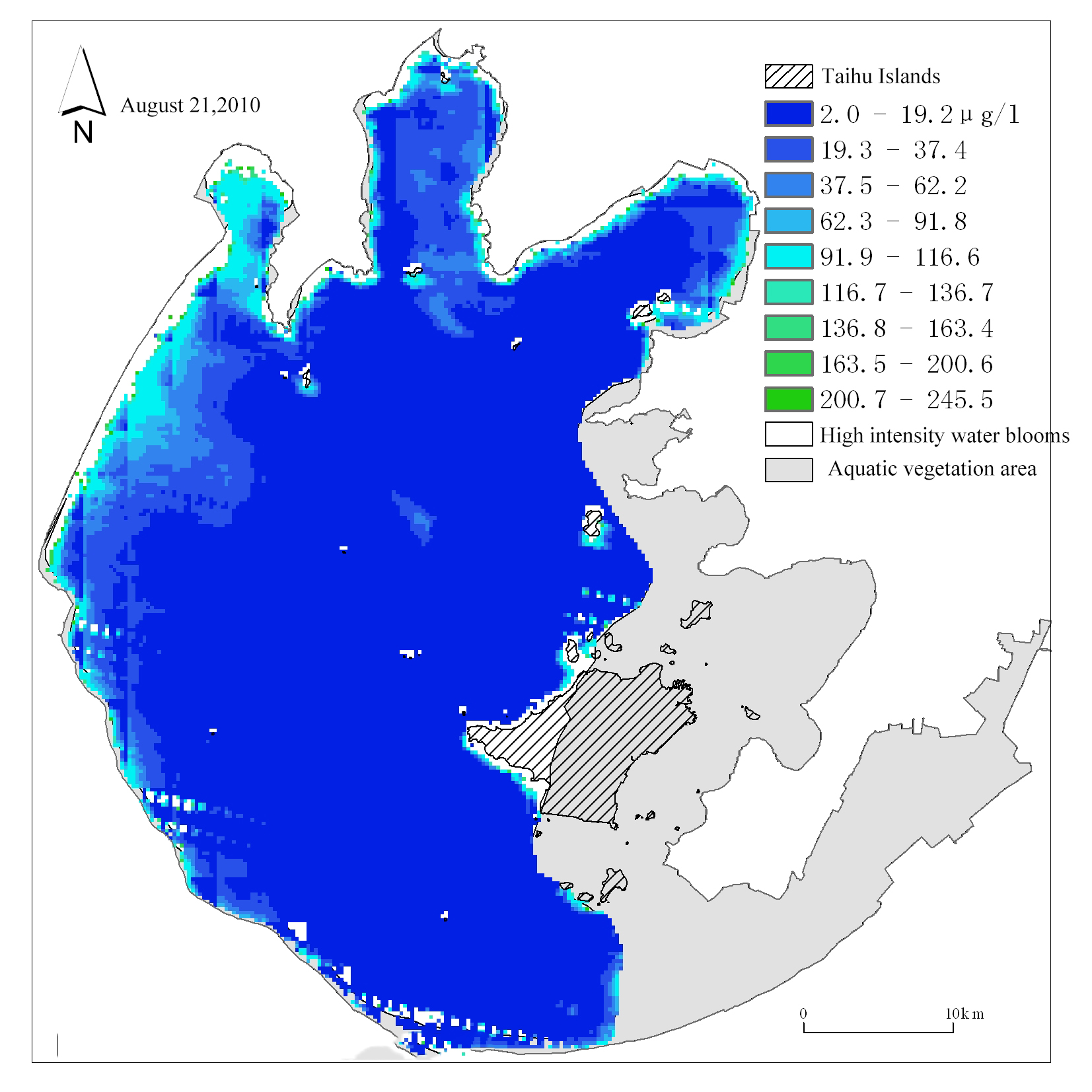

Supplement: Supplemental Information 3 — The data were obtained from the remote sensing image data of chlorophyll a concentration from the Lake-Watershed Science SubCenter, National Earth System Science Data Center, National Science & Technology Infrastructure of China, which had inconsistent data scales, data anomalies and different sampling intervals, and the chlorophyll a concentration unit was µg/L. [file peerj-cs-09-1292-s003.zip › 201008210327_taihu_chla.jpg]

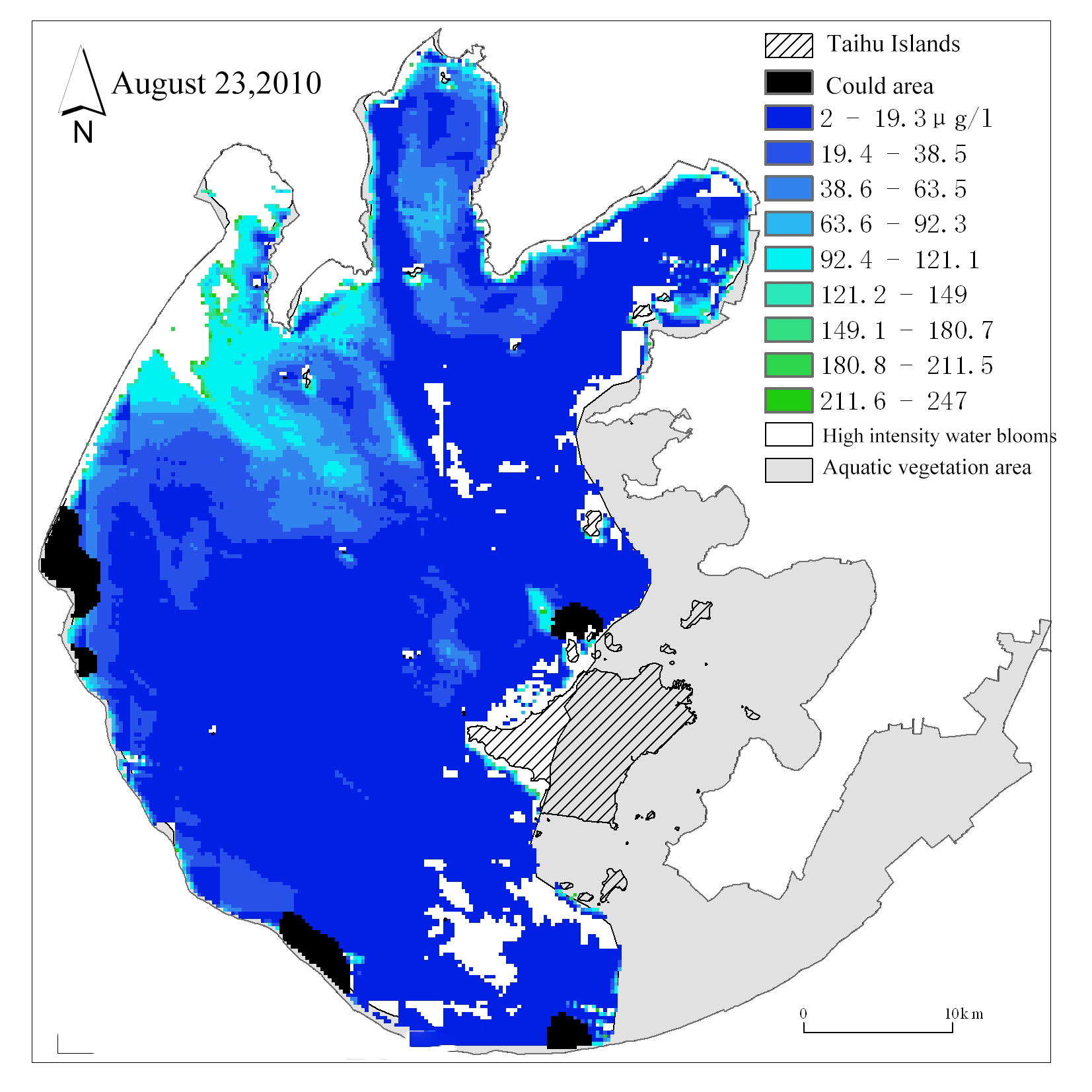

Supplement: Supplemental Information 3 — The data were obtained from the remote sensing image data of chlorophyll a concentration from the Lake-Watershed Science SubCenter, National Earth System Science Data Center, National Science & Technology Infrastructure of China, which had inconsistent data scales, data anomalies and different sampling intervals, and the chlorophyll a concentration unit was µg/L. [file peerj-cs-09-1292-s003.zip › 201008231115_taihu_chla.jpg]

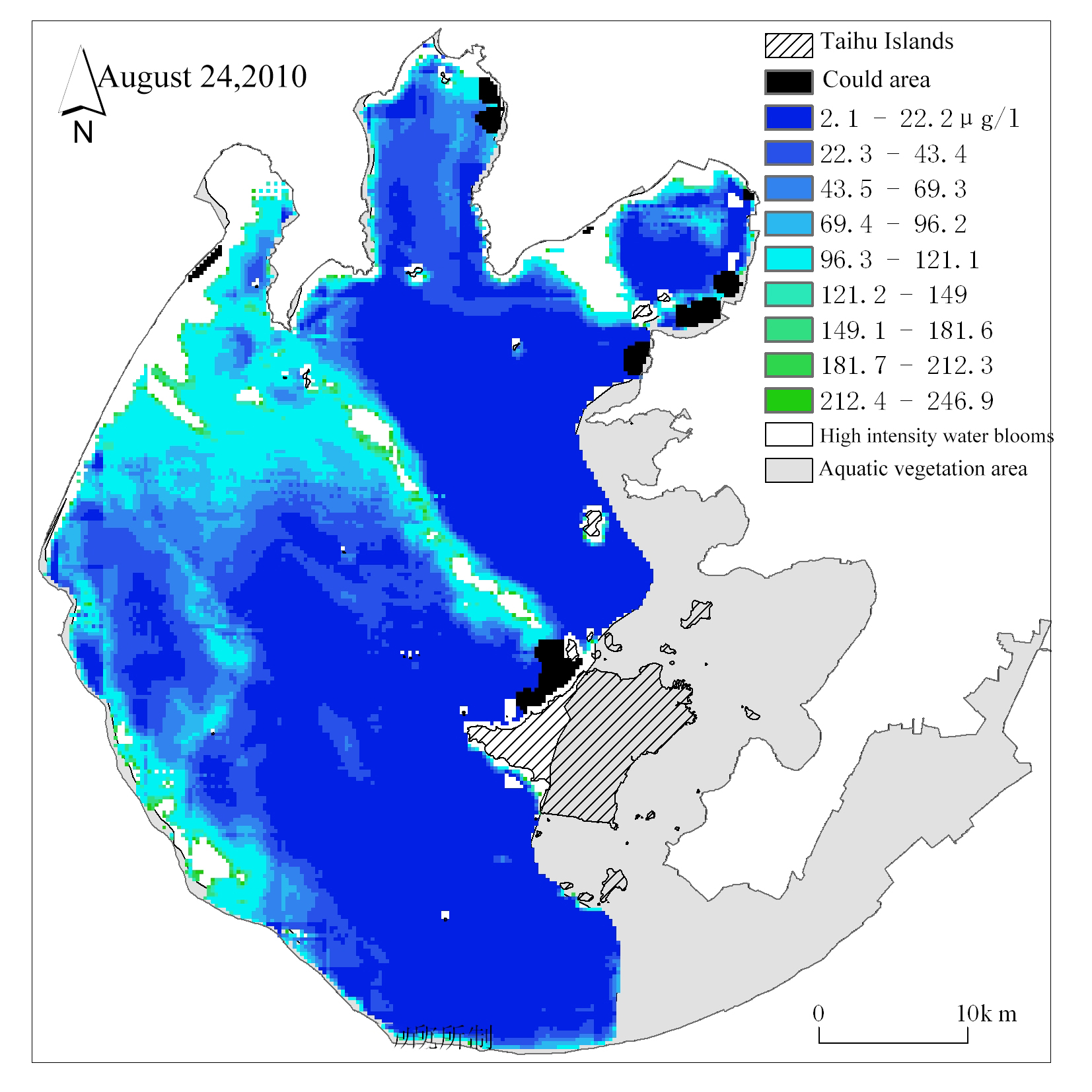

Supplement: Supplemental Information 3 — The data were obtained from the remote sensing image data of chlorophyll a concentration from the Lake-Watershed Science SubCenter, National Earth System Science Data Center, National Science & Technology Infrastructure of China, which had inconsistent data scales, data anomalies and different sampling intervals, and the chlorophyll a concentration unit was µg/L. [file peerj-cs-09-1292-s003.zip › 201008241021_taihu_chla.jpg]

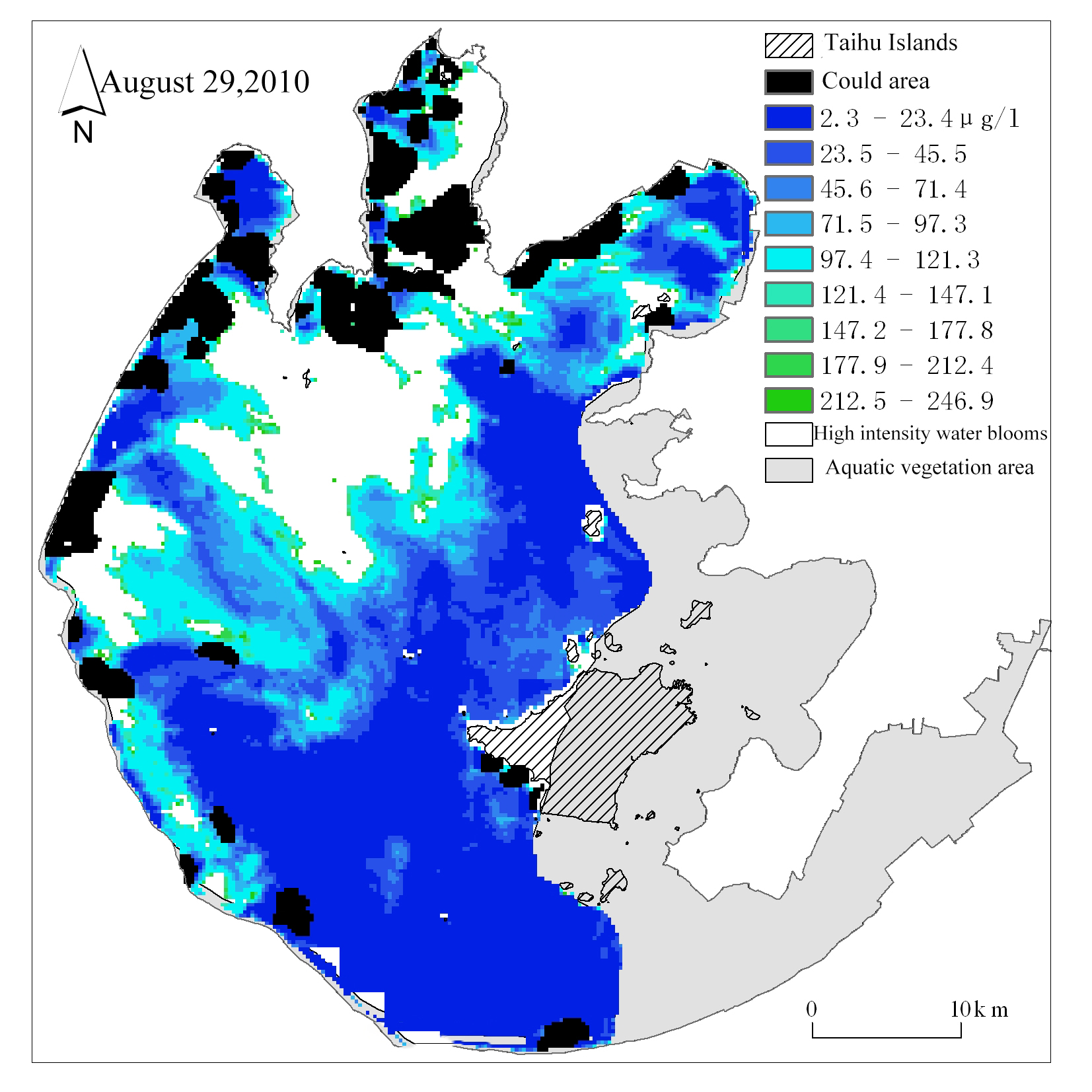

Supplement: Supplemental Information 3 — The data were obtained from the remote sensing image data of chlorophyll a concentration from the Lake-Watershed Science SubCenter, National Earth System Science Data Center, National Science & Technology Infrastructure of China, which had inconsistent data scales, data anomalies and different sampling intervals, and the chlorophyll a concentration unit was µg/L. [file peerj-cs-09-1292-s003.zip › 201008291038_taihu_chla.jpg]

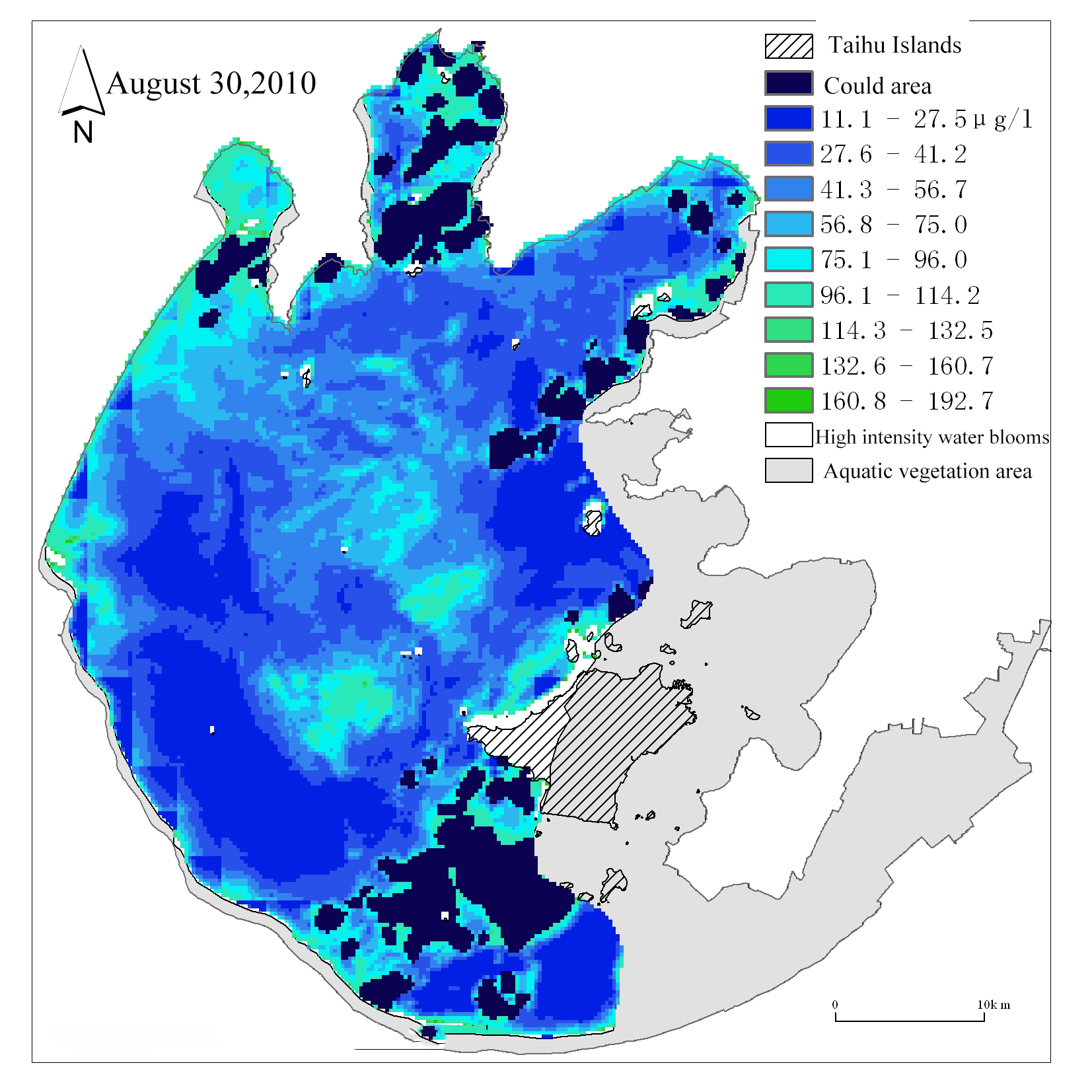

Supplement: Supplemental Information 3 — The data were obtained from the remote sensing image data of chlorophyll a concentration from the Lake-Watershed Science SubCenter, National Earth System Science Data Center, National Science & Technology Infrastructure of China, which had inconsistent data scales, data anomalies and different sampling intervals, and the chlorophyll a concentration unit was µg/L. [file peerj-cs-09-1292-s003.zip › 201008300454_taihu_chla.jpg]

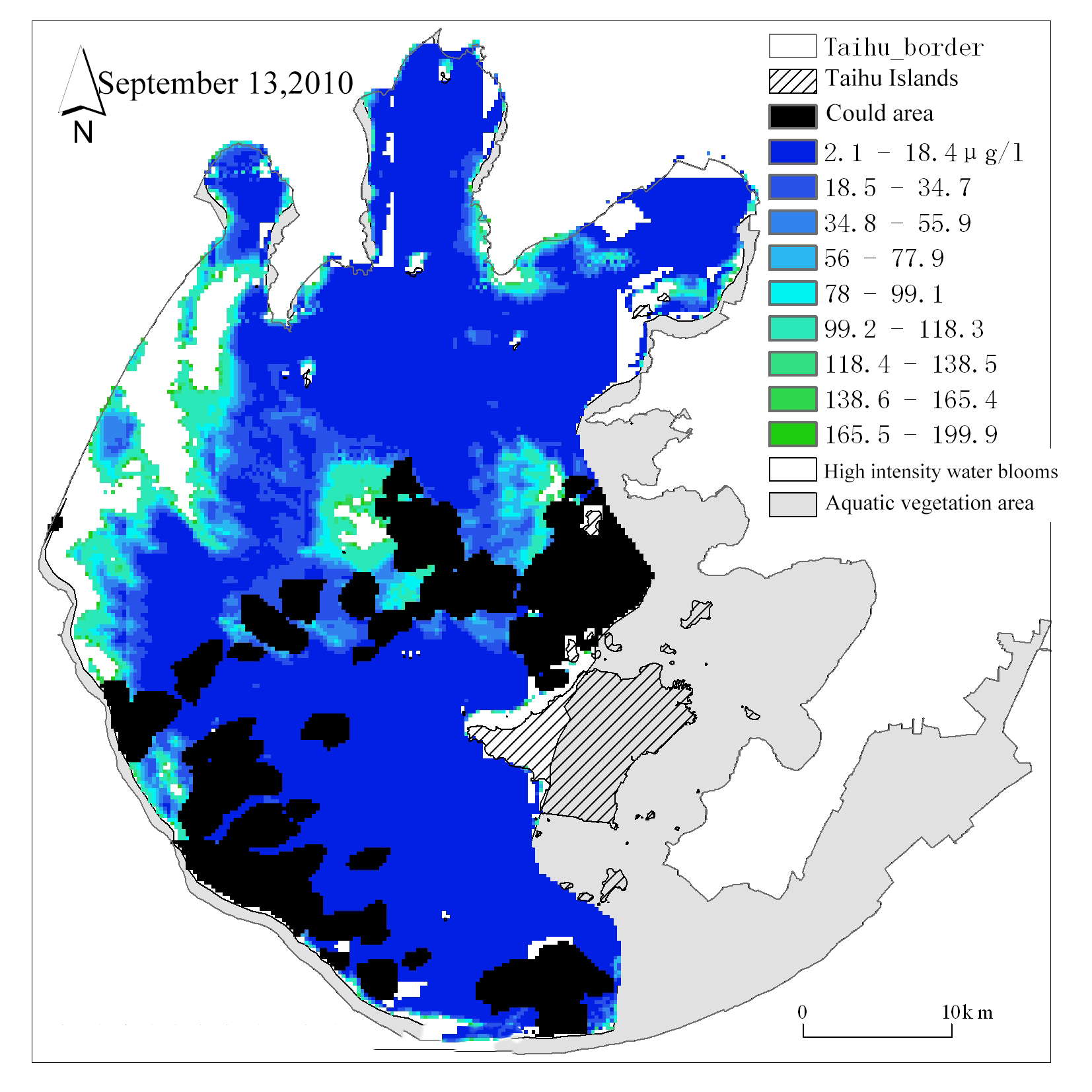

Supplement: Supplemental Information 3 — The data were obtained from the remote sensing image data of chlorophyll a concentration from the Lake-Watershed Science SubCenter, National Earth System Science Data Center, National Science & Technology Infrastructure of China, which had inconsistent data scales, data anomalies and different sampling intervals, and the chlorophyll a concentration unit was µg/L. [file peerj-cs-09-1292-s003.zip › 201009091329_taihu_chla.jpg]

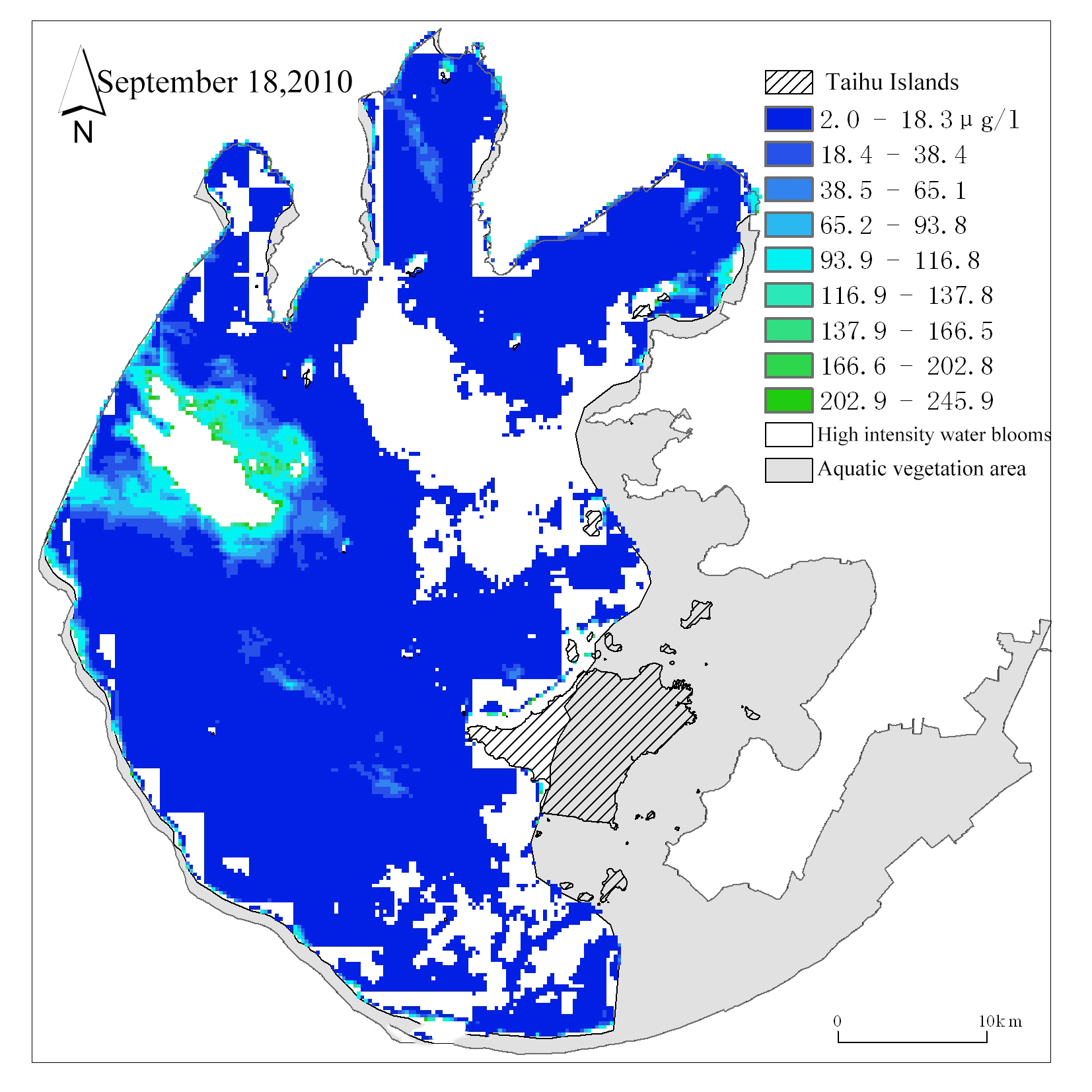

Supplement: Supplemental Information 3 — The data were obtained from the remote sensing image data of chlorophyll a concentration from the Lake-Watershed Science SubCenter, National Earth System Science Data Center, National Science & Technology Infrastructure of China, which had inconsistent data scales, data anomalies and different sampling intervals, and the chlorophyll a concentration unit was µg/L. [file peerj-cs-09-1292-s003.zip › 201009180523_taihu_chla.jpg]

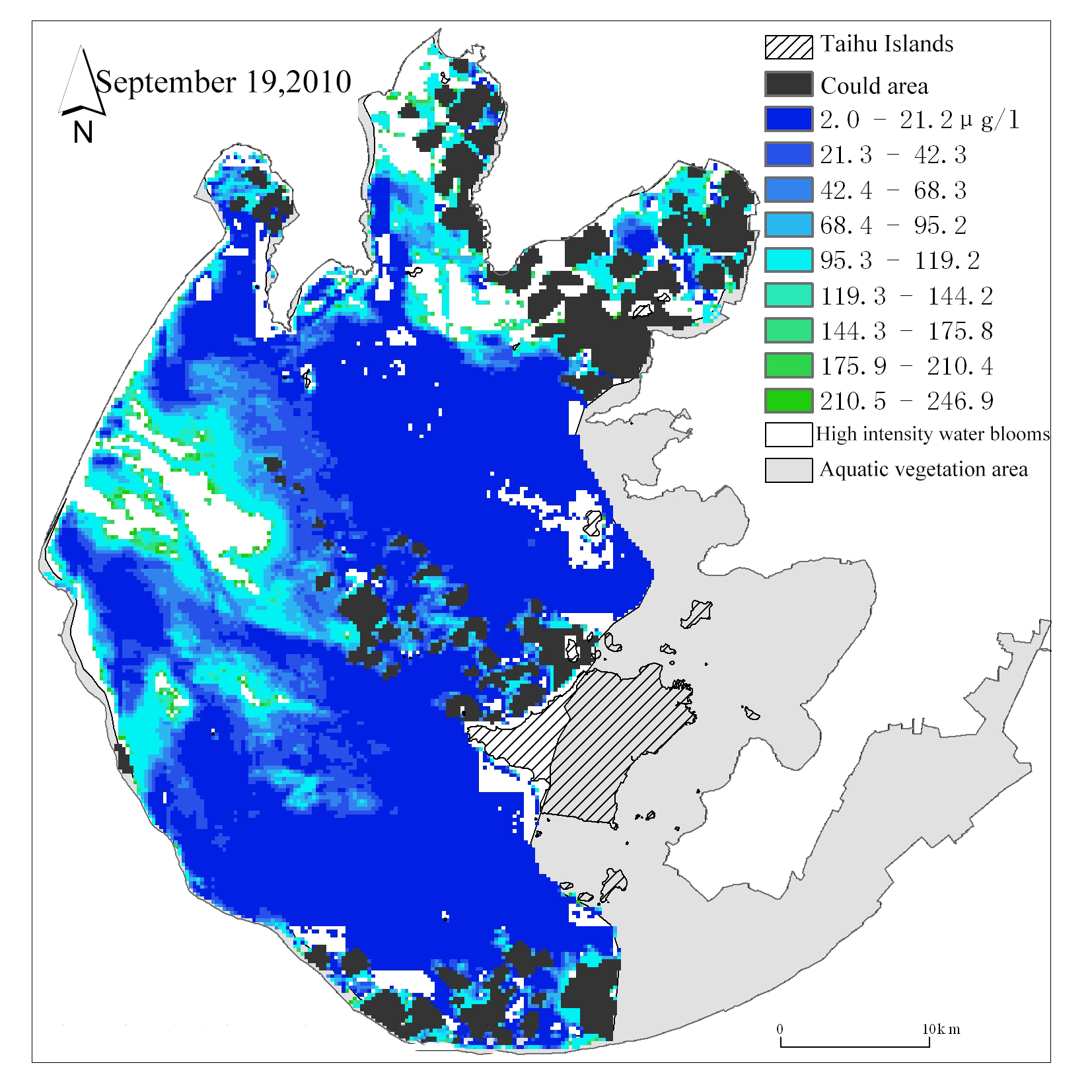

Supplement: Supplemental Information 3 — The data were obtained from the remote sensing image data of chlorophyll a concentration from the Lake-Watershed Science SubCenter, National Earth System Science Data Center, National Science & Technology Infrastructure of China, which had inconsistent data scales, data anomalies and different sampling intervals, and the chlorophyll a concentration unit was µg/L. [file peerj-cs-09-1292-s003.zip › 201009191053_taihu_chla.jpg]

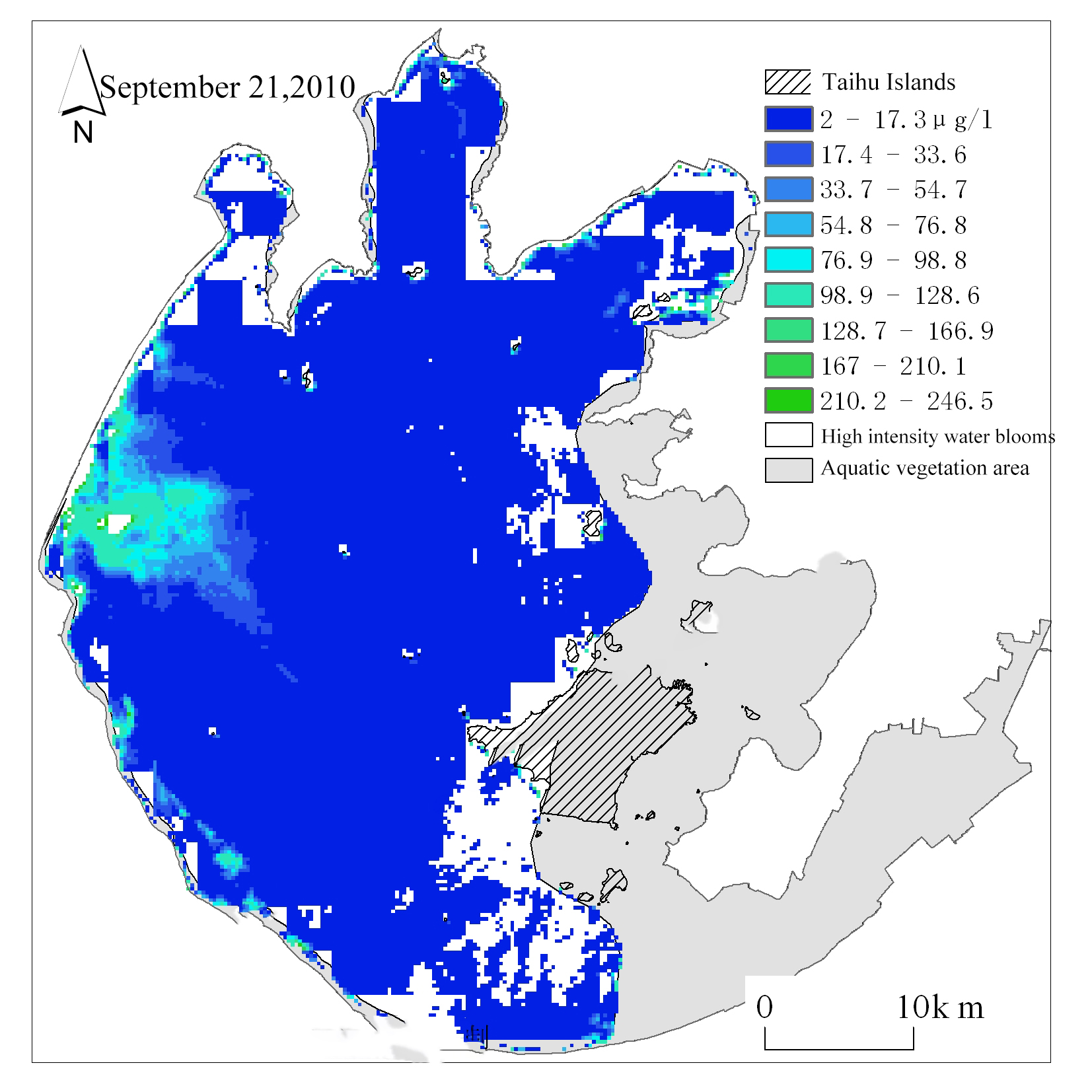

Supplement: Supplemental Information 3 — The data were obtained from the remote sensing image data of chlorophyll a concentration from the Lake-Watershed Science SubCenter, National Earth System Science Data Center, National Science & Technology Infrastructure of China, which had inconsistent data scales, data anomalies and different sampling intervals, and the chlorophyll a concentration unit was µg/L. [file peerj-cs-09-1292-s003.zip › 201009211040_taihu_chl-a.jpg]

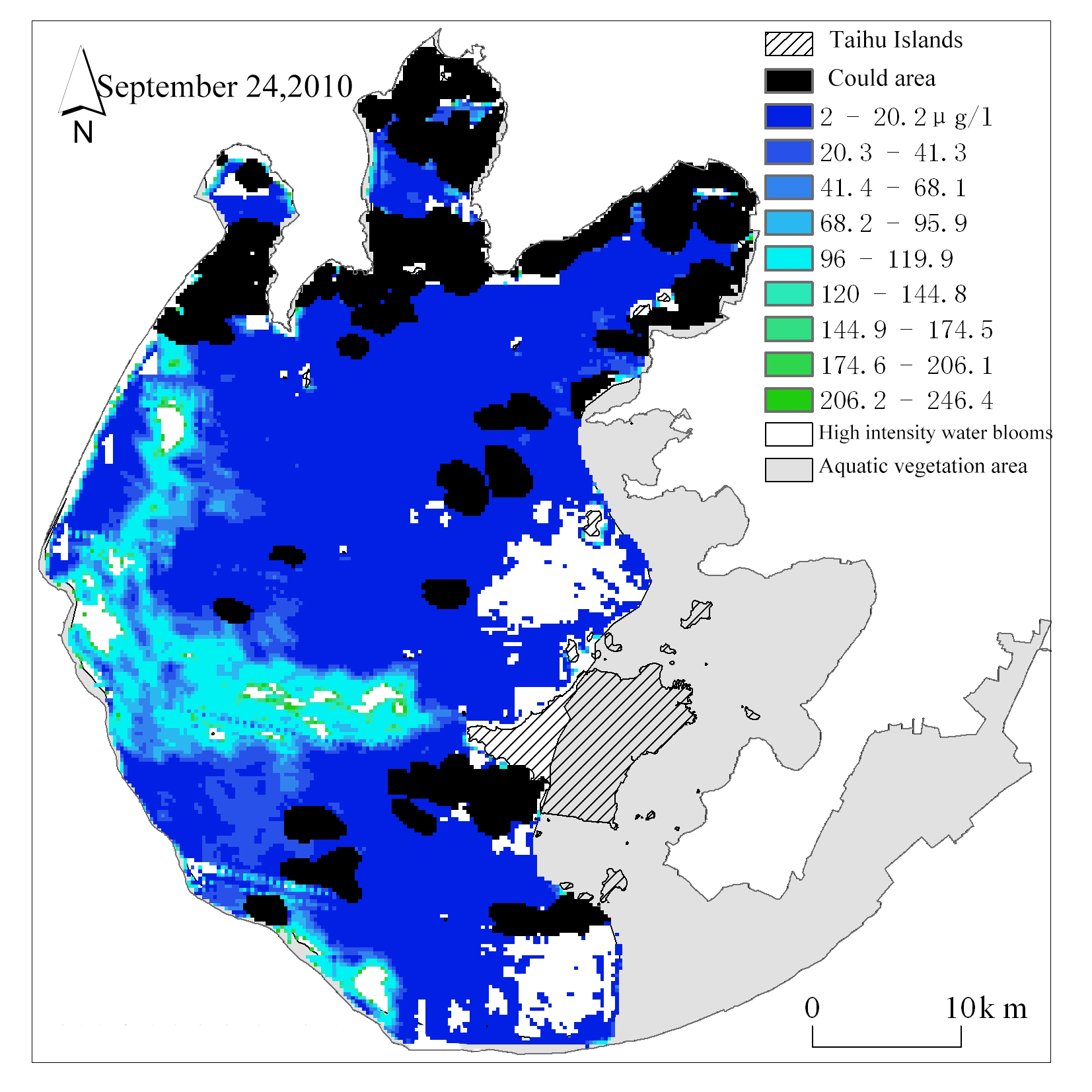

Supplement: Supplemental Information 3 — The data were obtained from the remote sensing image data of chlorophyll a concentration from the Lake-Watershed Science SubCenter, National Earth System Science Data Center, National Science & Technology Infrastructure of China, which had inconsistent data scales, data anomalies and different sampling intervals, and the chlorophyll a concentration unit was µg/L. [file peerj-cs-09-1292-s003.zip › 201009241115_taihu_chla.jpg]

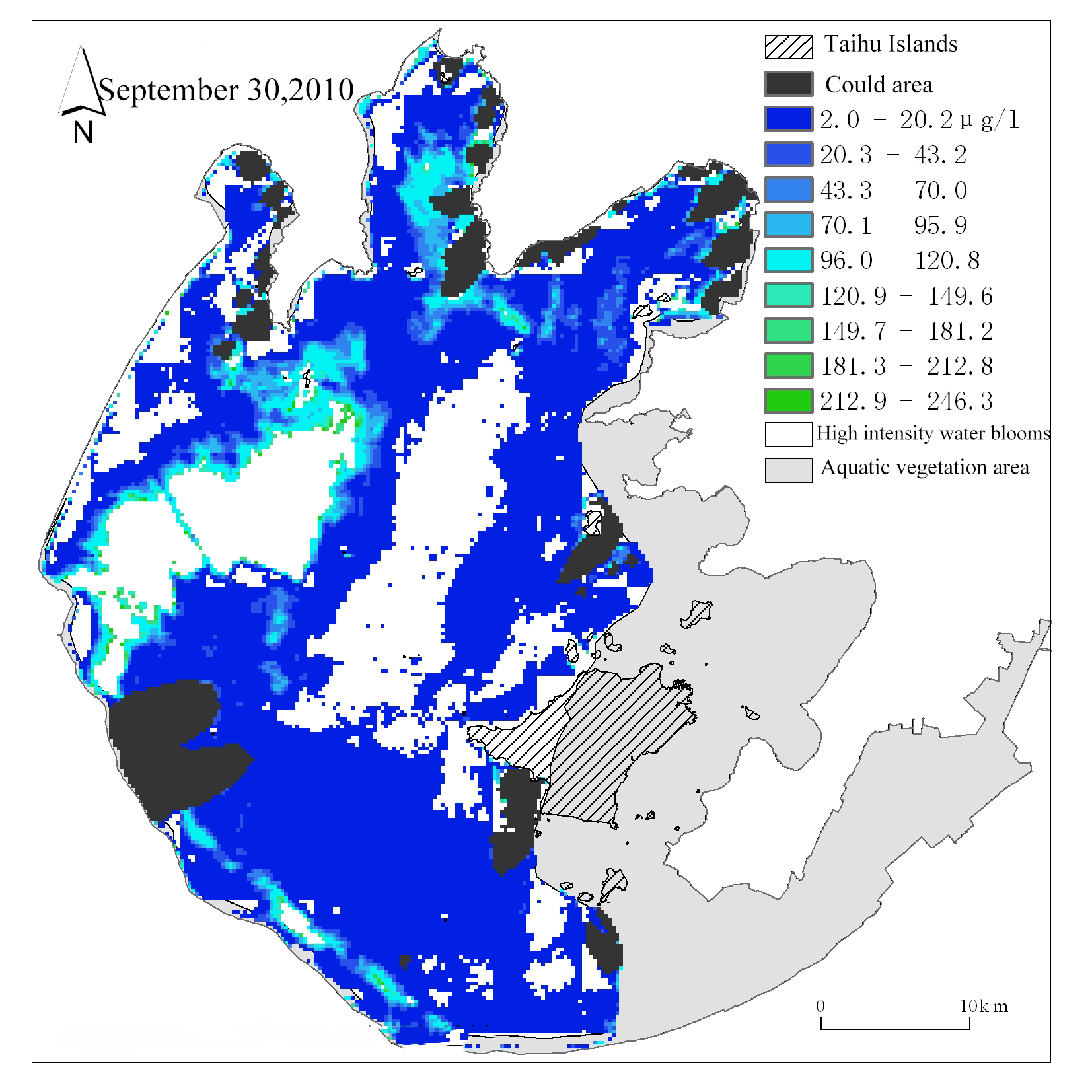

Supplement: Supplemental Information 3 — The data were obtained from the remote sensing image data of chlorophyll a concentration from the Lake-Watershed Science SubCenter, National Earth System Science Data Center, National Science & Technology Infrastructure of China, which had inconsistent data scales, data anomalies and different sampling intervals, and the chlorophyll a concentration unit was µg/L. [file peerj-cs-09-1292-s003.zip › 201009300238_taihu_chla.jpg]

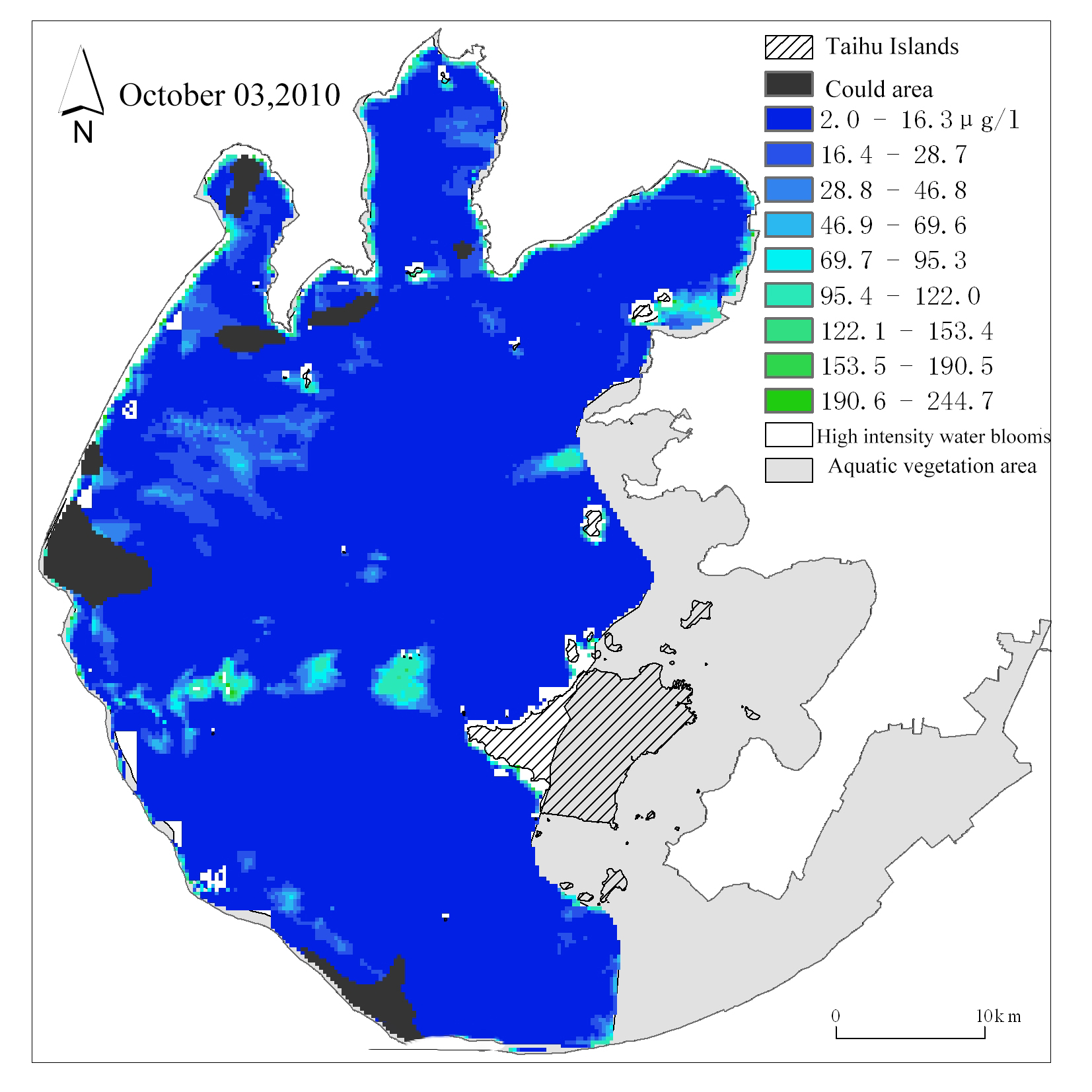

Supplement: Supplemental Information 3 — The data were obtained from the remote sensing image data of chlorophyll a concentration from the Lake-Watershed Science SubCenter, National Earth System Science Data Center, National Science & Technology Infrastructure of China, which had inconsistent data scales, data anomalies and different sampling intervals, and the chlorophyll a concentration unit was µg/L. [file peerj-cs-09-1292-s003.zip › 201010031105_taihu_chla.jpg]

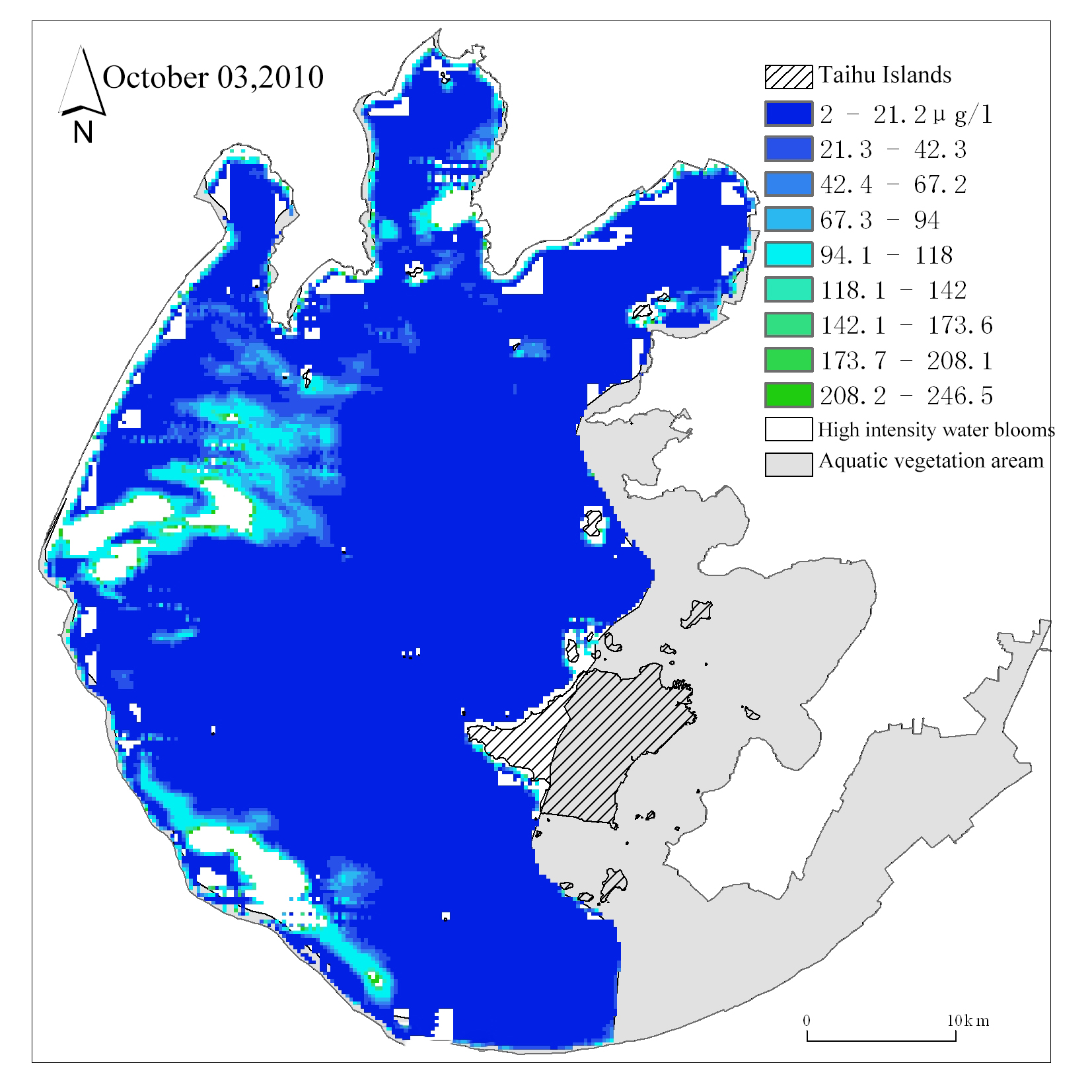

Supplement: Supplemental Information 4 — The data were obtained from the remote sensing image data of chlorophyll a concentration from the Lake-Watershed Science SubCenter, National Earth System Science Data Center, National Science & Technology Infrastructure of China, which had inconsistent data scales, data anomalies and different sampling intervals, and the chlorophyll a concentration unit was µg/L. [file peerj-cs-09-1292-s004.zip › 201010041014_taihu_chla.jpg]

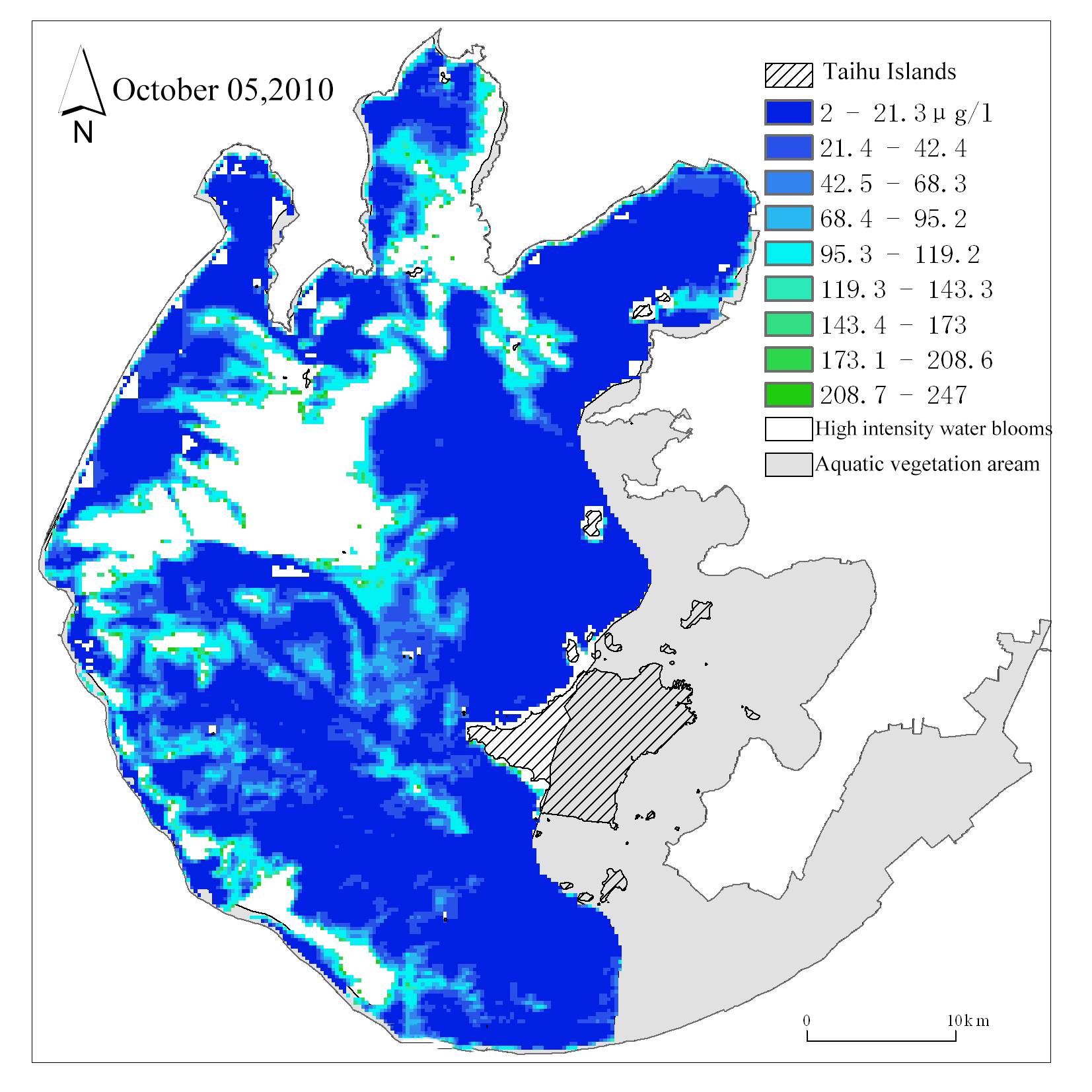

Supplement: Supplemental Information 4 — The data were obtained from the remote sensing image data of chlorophyll a concentration from the Lake-Watershed Science SubCenter, National Earth System Science Data Center, National Science & Technology Infrastructure of China, which had inconsistent data scales, data anomalies and different sampling intervals, and the chlorophyll a concentration unit was µg/L. [file peerj-cs-09-1292-s004.zip › 201010051052_taihu_chla.jpg]

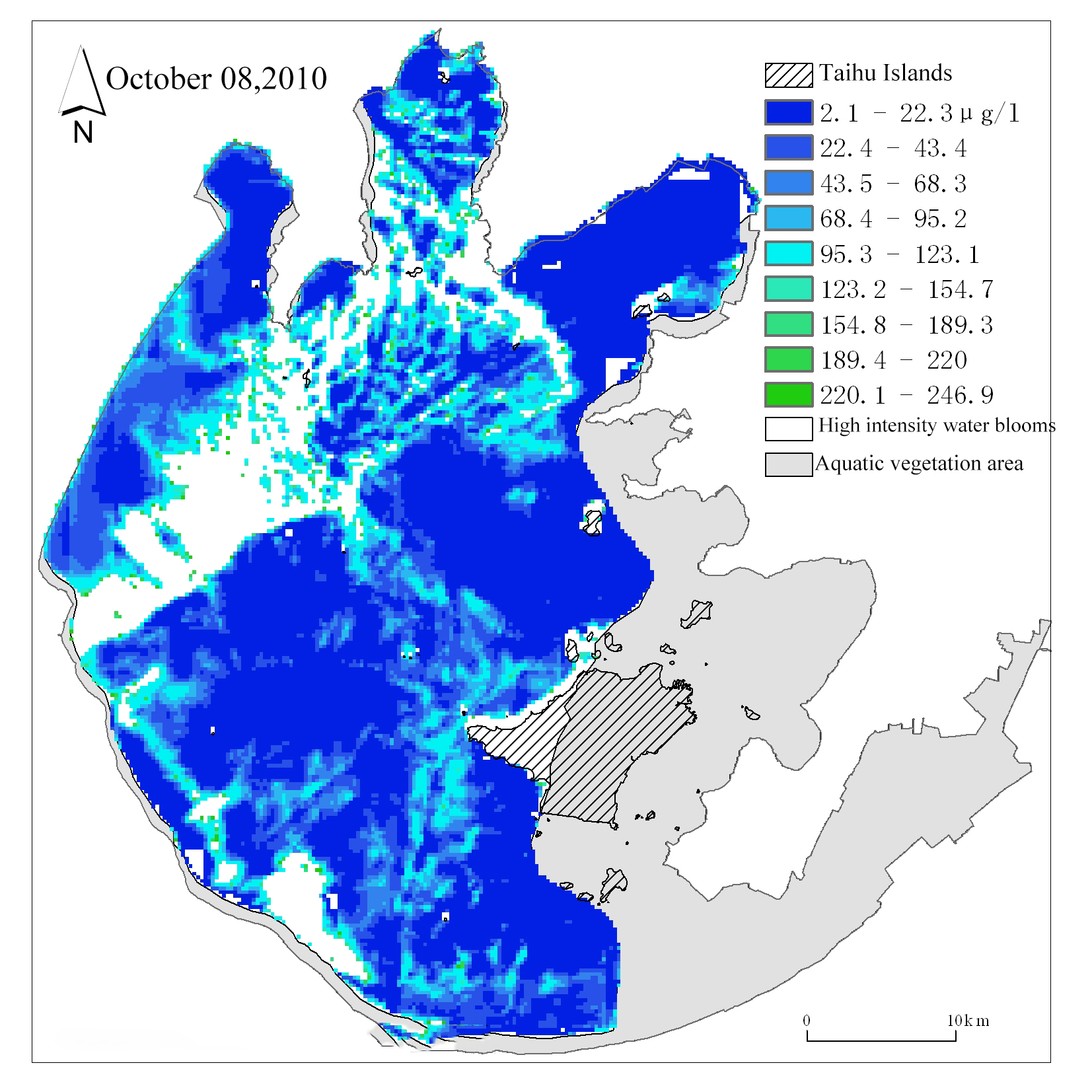

Supplement: Supplemental Information 4 — The data were obtained from the remote sensing image data of chlorophyll a concentration from the Lake-Watershed Science SubCenter, National Earth System Science Data Center, National Science & Technology Infrastructure of China, which had inconsistent data scales, data anomalies and different sampling intervals, and the chlorophyll a concentration unit was µg/L. [file peerj-cs-09-1292-s004.zip › 201010081259_taihu_chla.jpg]

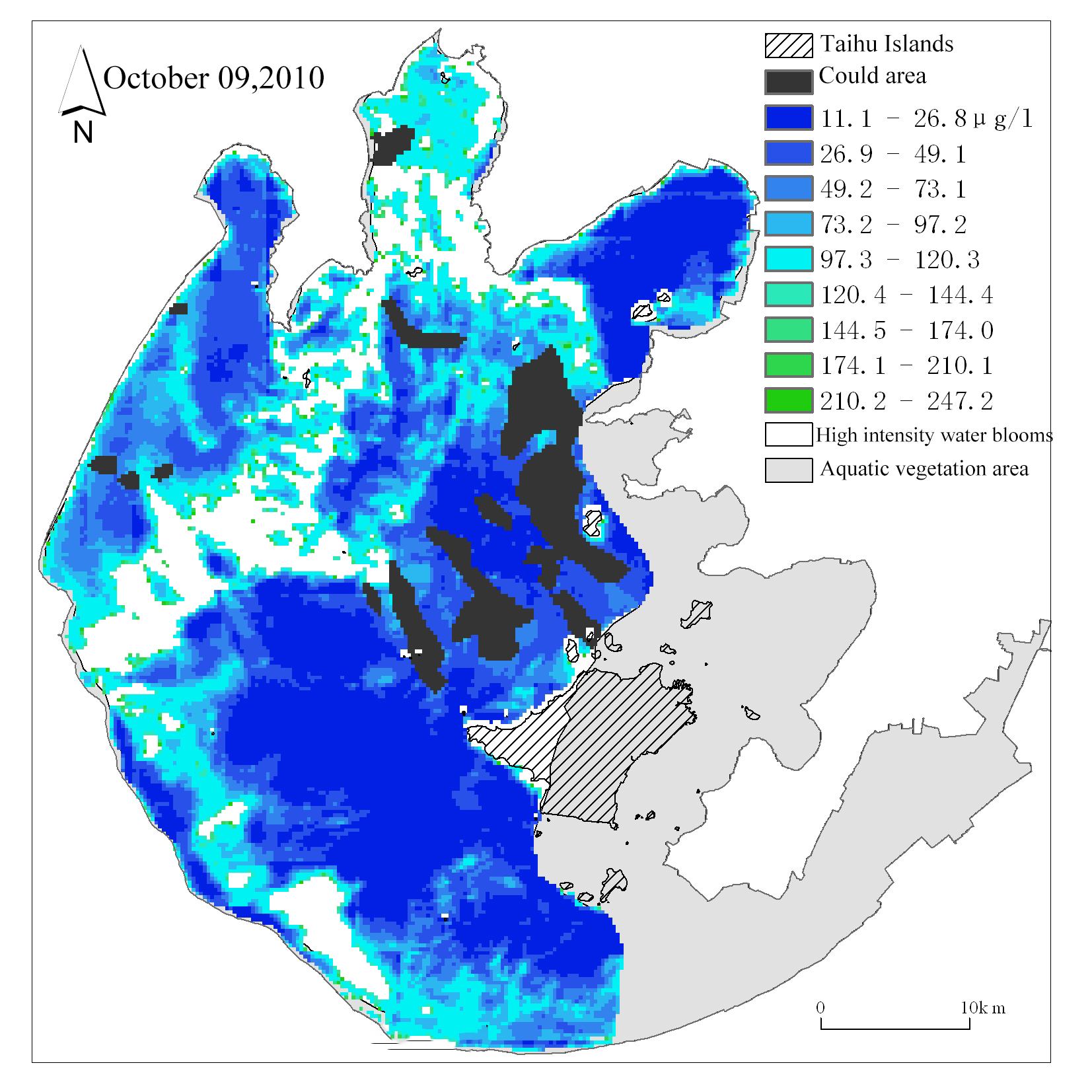

Supplement: Supplemental Information 4 — The data were obtained from the remote sensing image data of chlorophyll a concentration from the Lake-Watershed Science SubCenter, National Earth System Science Data Center, National Science & Technology Infrastructure of China, which had inconsistent data scales, data anomalies and different sampling intervals, and the chlorophyll a concentration unit was µg/L. [file peerj-cs-09-1292-s004.zip › 201010090232_taihu_chla.jpg]

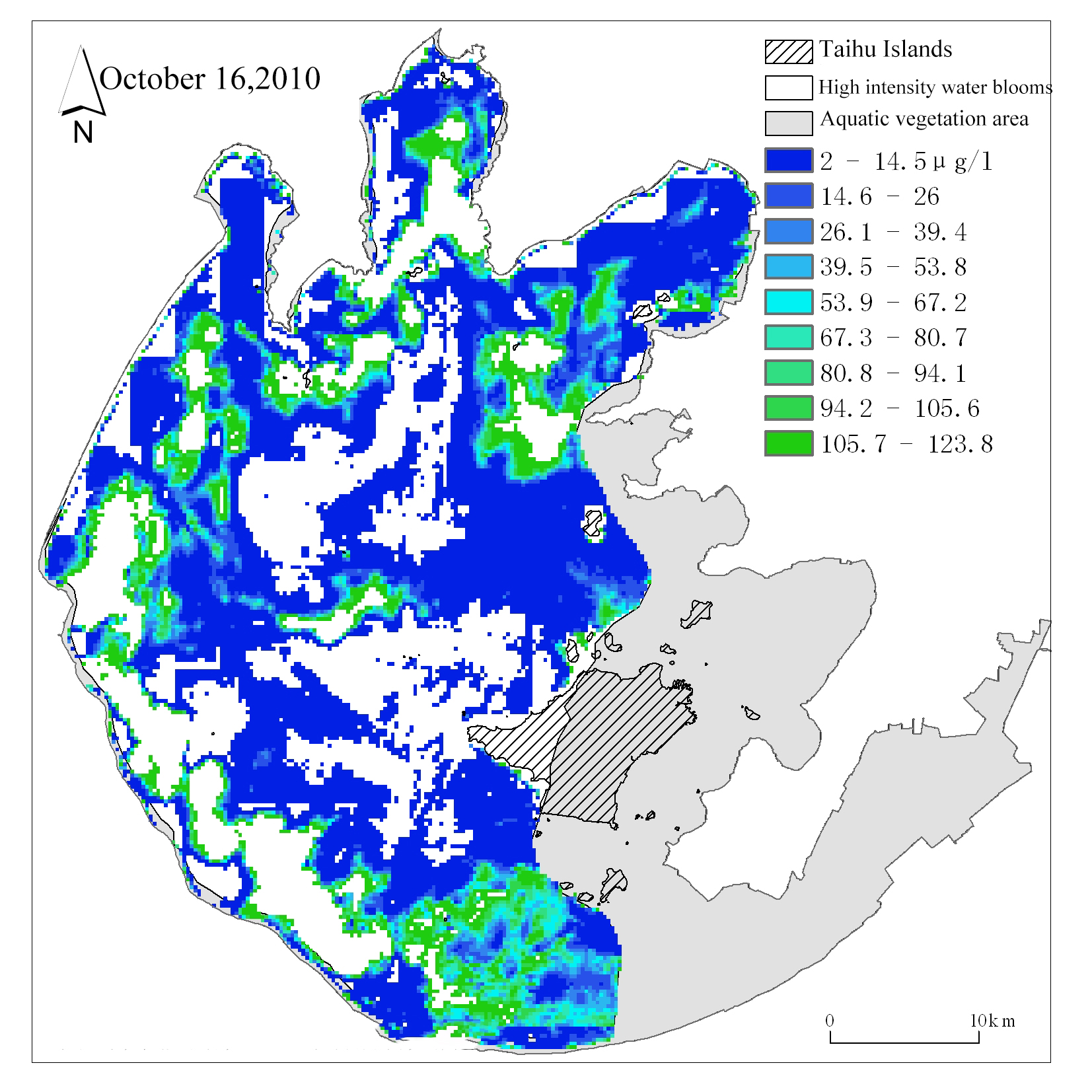

Supplement: Supplemental Information 4 — The data were obtained from the remote sensing image data of chlorophyll a concentration from the Lake-Watershed Science SubCenter, National Earth System Science Data Center, National Science & Technology Infrastructure of China, which had inconsistent data scales, data anomalies and different sampling intervals, and the chlorophyll a concentration unit was µg/L. [file peerj-cs-09-1292-s004.zip › 201010160238_taihu_chla.jpg]

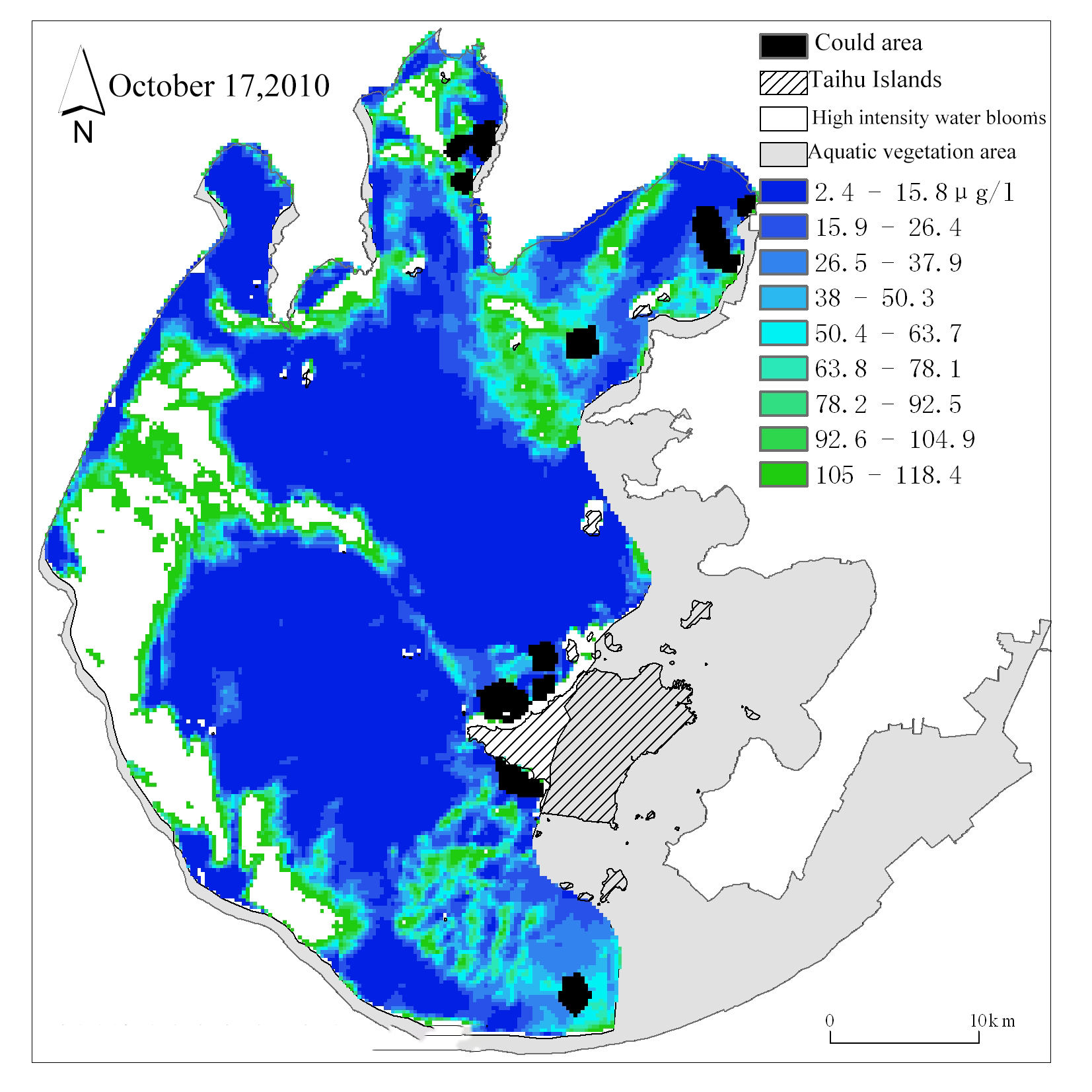

Supplement: Supplemental Information 4 — The data were obtained from the remote sensing image data of chlorophyll a concentration from the Lake-Watershed Science SubCenter, National Earth System Science Data Center, National Science & Technology Infrastructure of China, which had inconsistent data scales, data anomalies and different sampling intervals, and the chlorophyll a concentration unit was µg/L. [file peerj-cs-09-1292-s004.zip › 201010170454_taihu_chla.jpg]

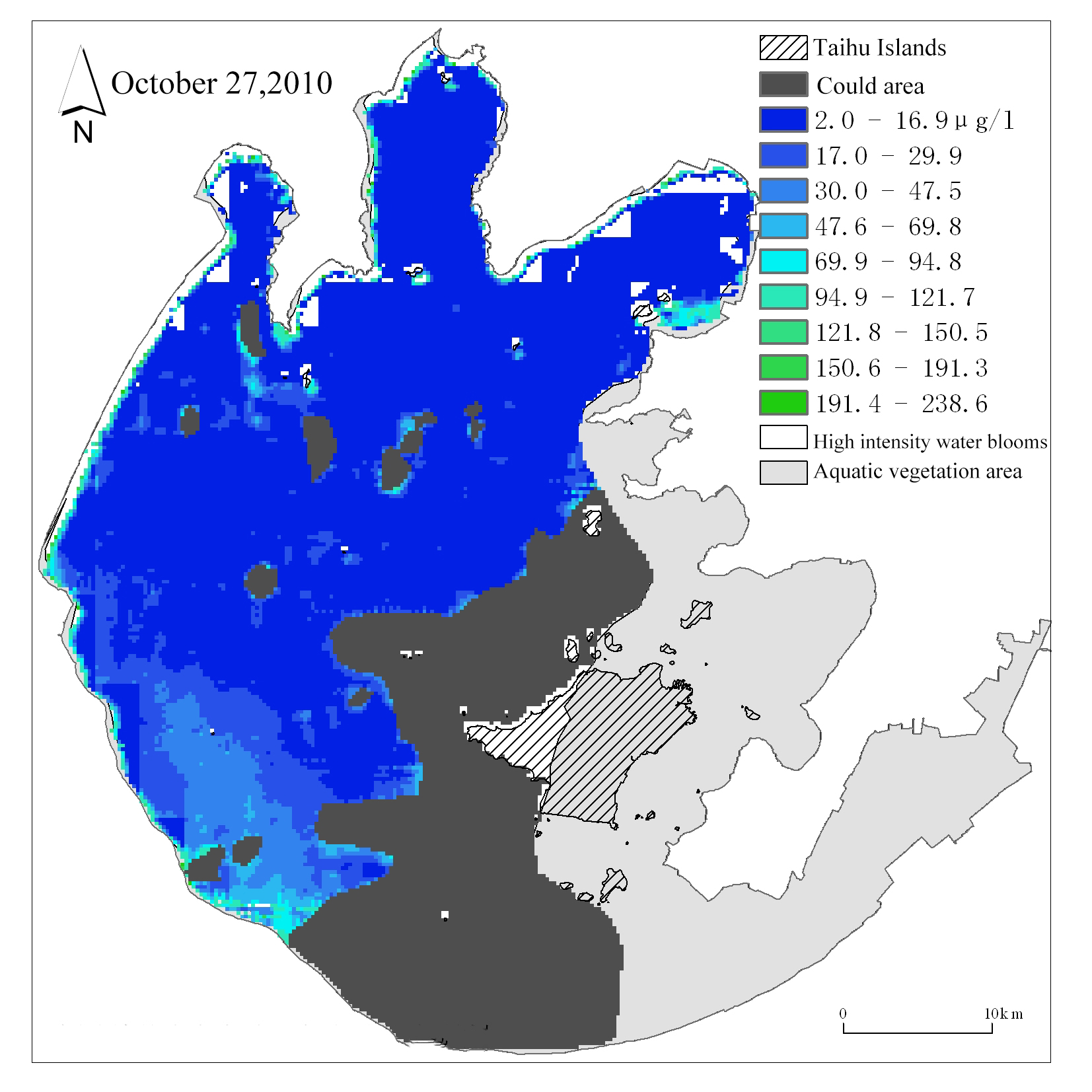

Supplement: Supplemental Information 4 — The data were obtained from the remote sensing image data of chlorophyll a concentration from the Lake-Watershed Science SubCenter, National Earth System Science Data Center, National Science & Technology Infrastructure of China, which had inconsistent data scales, data anomalies and different sampling intervals, and the chlorophyll a concentration unit was µg/L. [file peerj-cs-09-1292-s004.zip › 201010271016_taihu_chla.jpg]

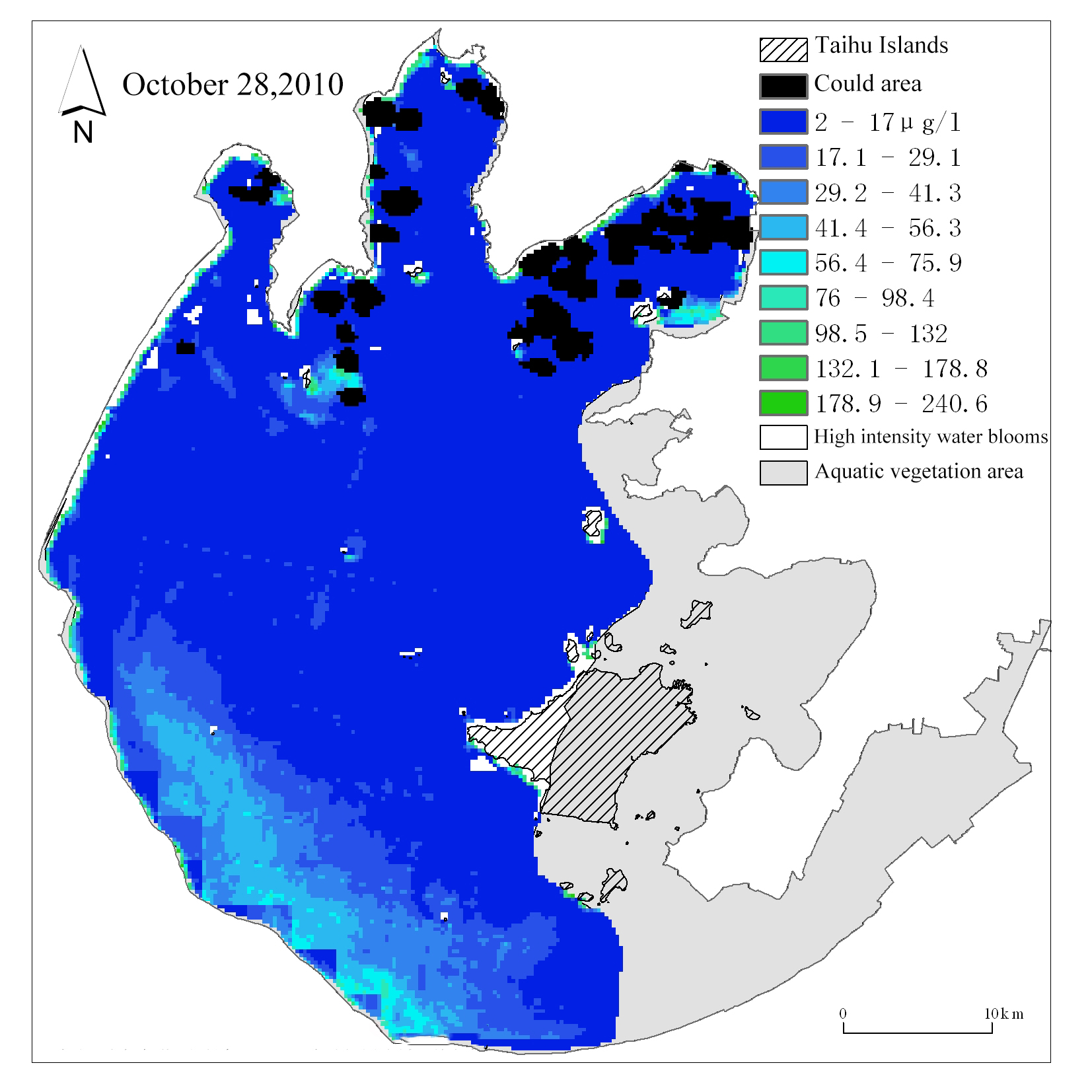

Supplement: Supplemental Information 4 — The data were obtained from the remote sensing image data of chlorophyll a concentration from the Lake-Watershed Science SubCenter, National Earth System Science Data Center, National Science & Technology Infrastructure of China, which had inconsistent data scales, data anomalies and different sampling intervals, and the chlorophyll a concentration unit was µg/L. [file peerj-cs-09-1292-s004.zip › 201010281058_taihu_chla.jpg]

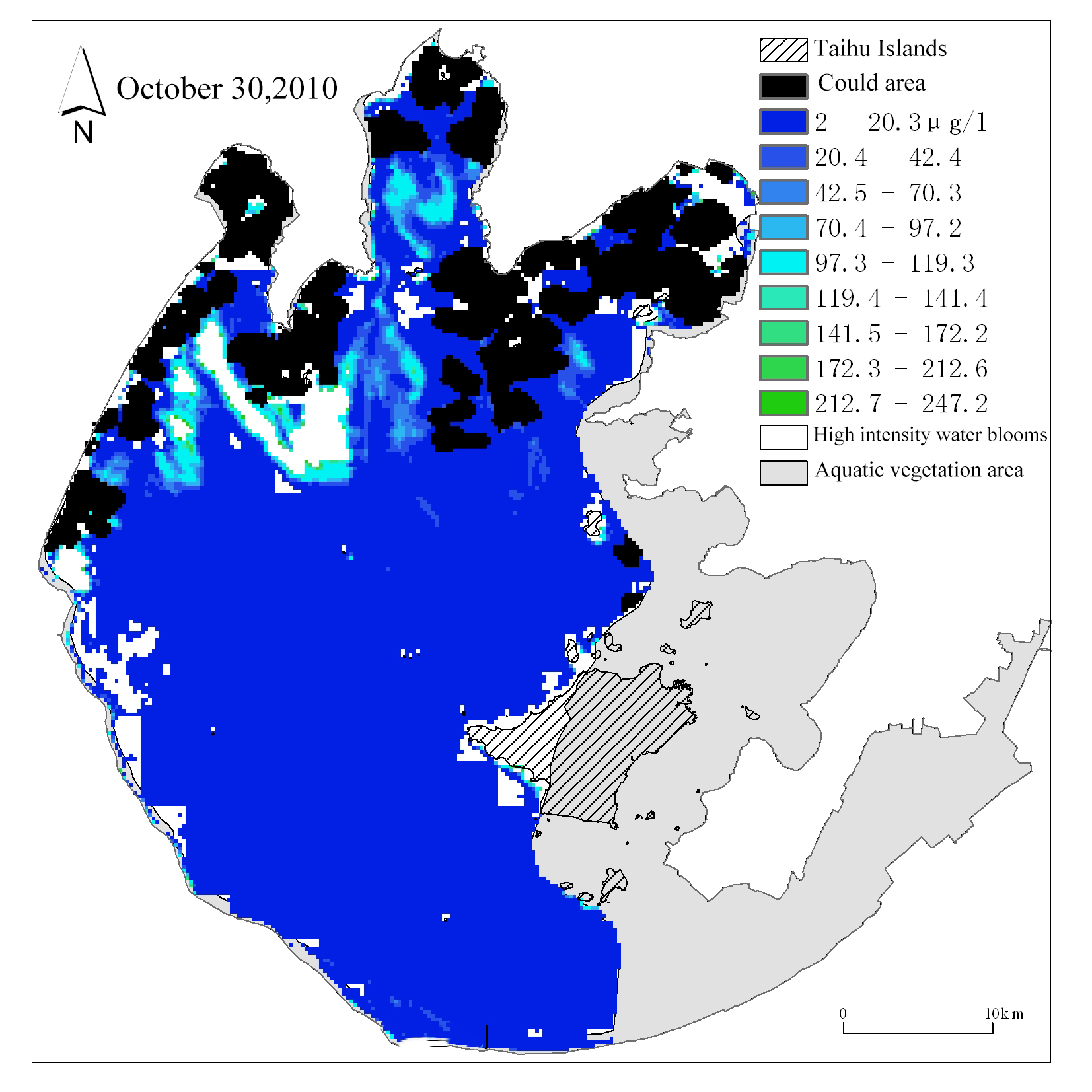

Supplement: Supplemental Information 4 — The data were obtained from the remote sensing image data of chlorophyll a concentration from the Lake-Watershed Science SubCenter, National Earth System Science Data Center, National Science & Technology Infrastructure of China, which had inconsistent data scales, data anomalies and different sampling intervals, and the chlorophyll a concentration unit was µg/L. [file peerj-cs-09-1292-s004.zip › 201010301050_taihu_chla.jpg]

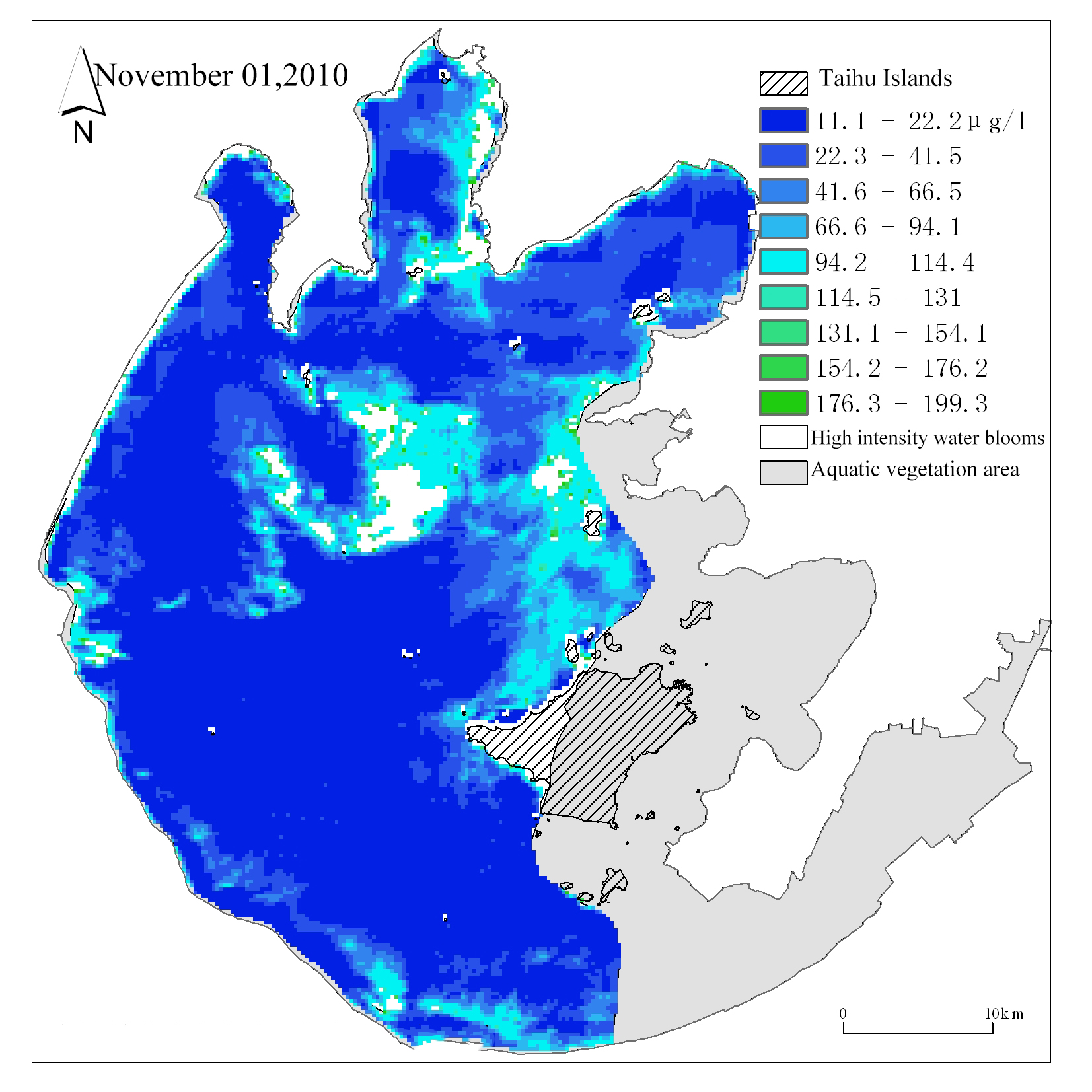

Supplement: Supplemental Information 4 — The data were obtained from the remote sensing image data of chlorophyll a concentration from the Lake-Watershed Science SubCenter, National Earth System Science Data Center, National Science & Technology Infrastructure of China, which had inconsistent data scales, data anomalies and different sampling intervals, and the chlorophyll a concentration unit was µg/L. [file peerj-cs-09-1292-s004.zip › 201011011034_taihu_chla.jpg]

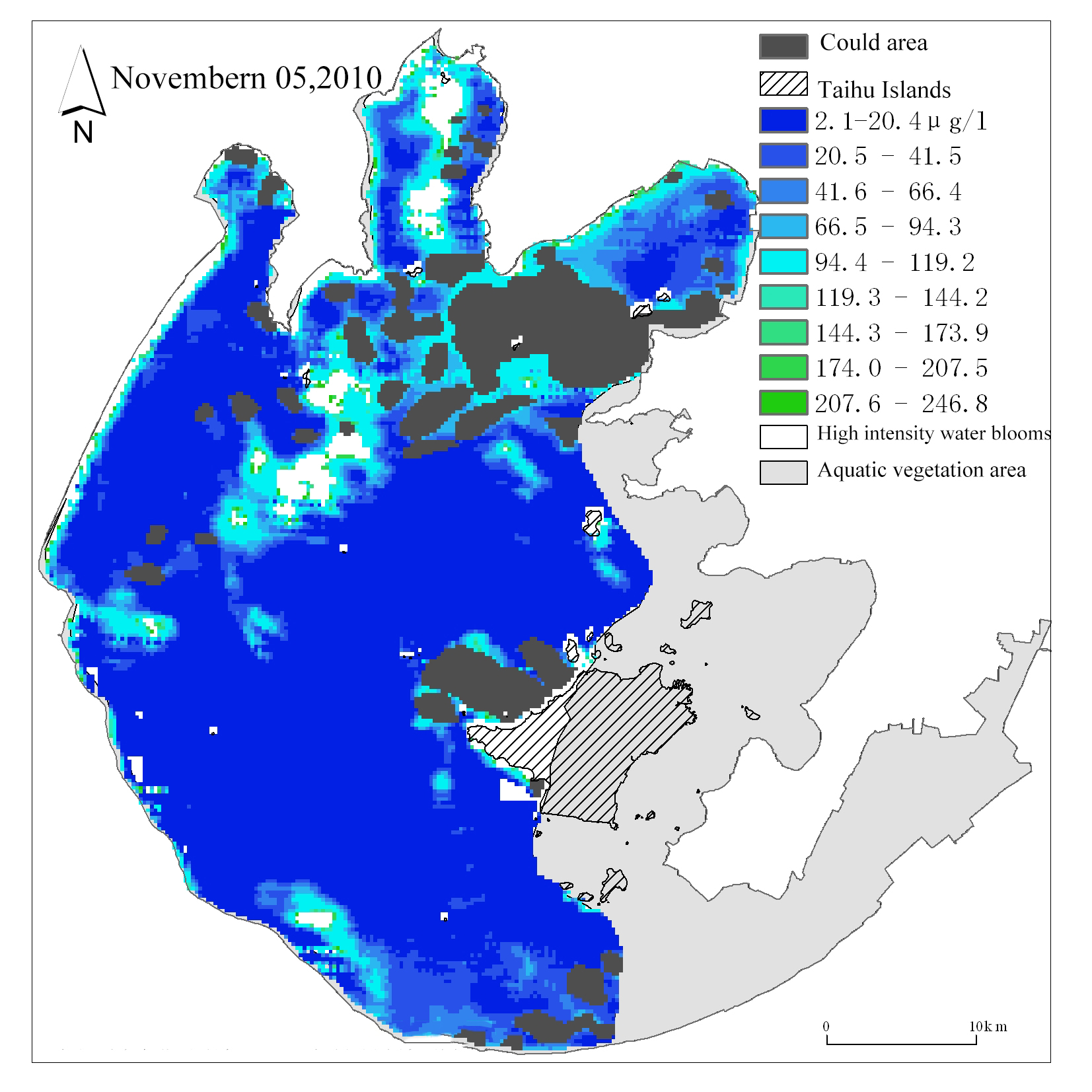

Supplement: Supplemental Information 4 — The data were obtained from the remote sensing image data of chlorophyll a concentration from the Lake-Watershed Science SubCenter, National Earth System Science Data Center, National Science & Technology Infrastructure of China, which had inconsistent data scales, data anomalies and different sampling intervals, and the chlorophyll a concentration unit was µg/L. [file peerj-cs-09-1292-s004.zip › 201011051010_taihu_chla.jpg]

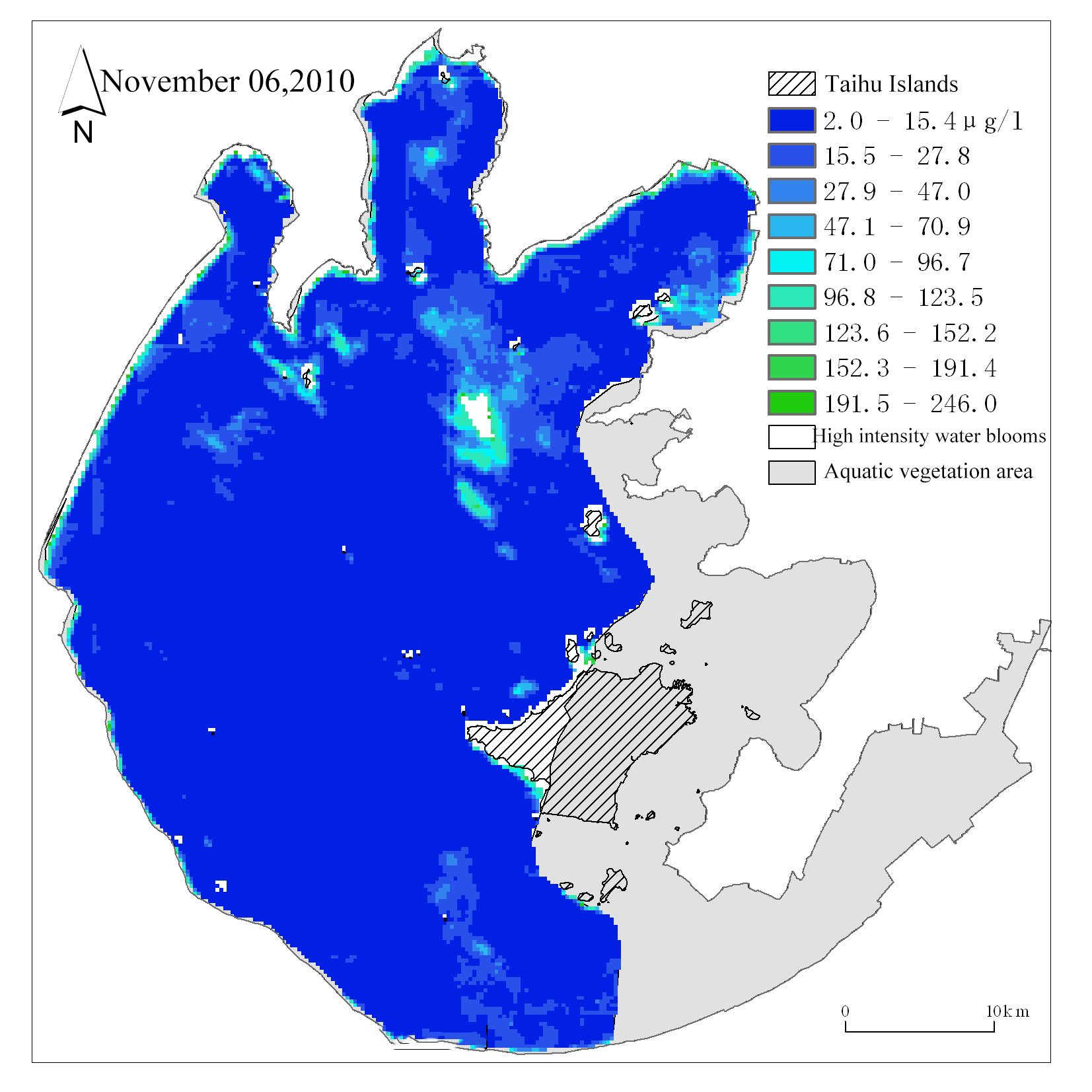

Supplement: Supplemental Information 4 — The data were obtained from the remote sensing image data of chlorophyll a concentration from the Lake-Watershed Science SubCenter, National Earth System Science Data Center, National Science & Technology Infrastructure of China, which had inconsistent data scales, data anomalies and different sampling intervals, and the chlorophyll a concentration unit was µg/L. [file peerj-cs-09-1292-s004.zip › 201011061052_taihu_chla.jpg]

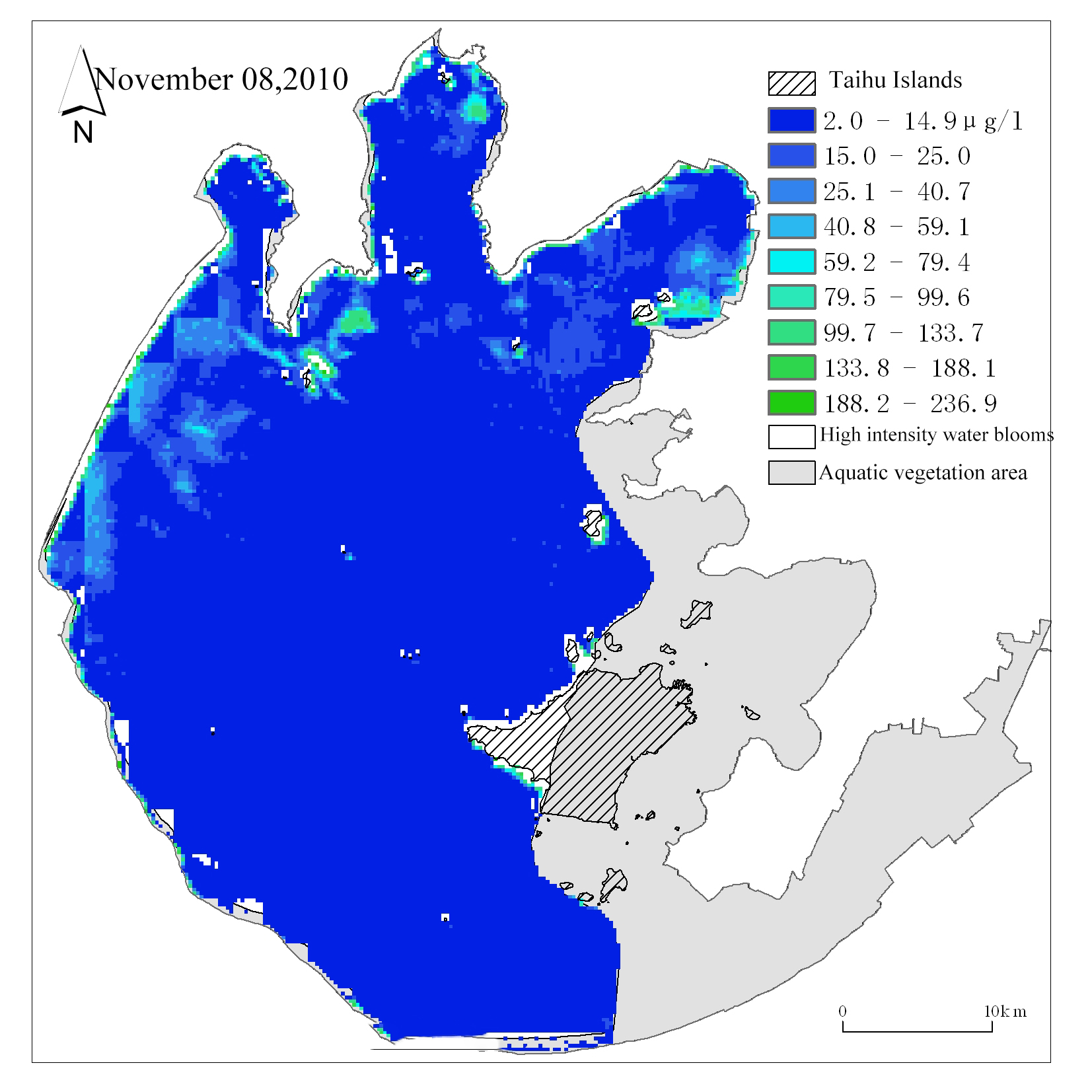

Supplement: Supplemental Information 4 — The data were obtained from the remote sensing image data of chlorophyll a concentration from the Lake-Watershed Science SubCenter, National Earth System Science Data Center, National Science & Technology Infrastructure of China, which had inconsistent data scales, data anomalies and different sampling intervals, and the chlorophyll a concentration unit was µg/L. [file peerj-cs-09-1292-s004.zip › 201011081040_taihu_chla.jpg]

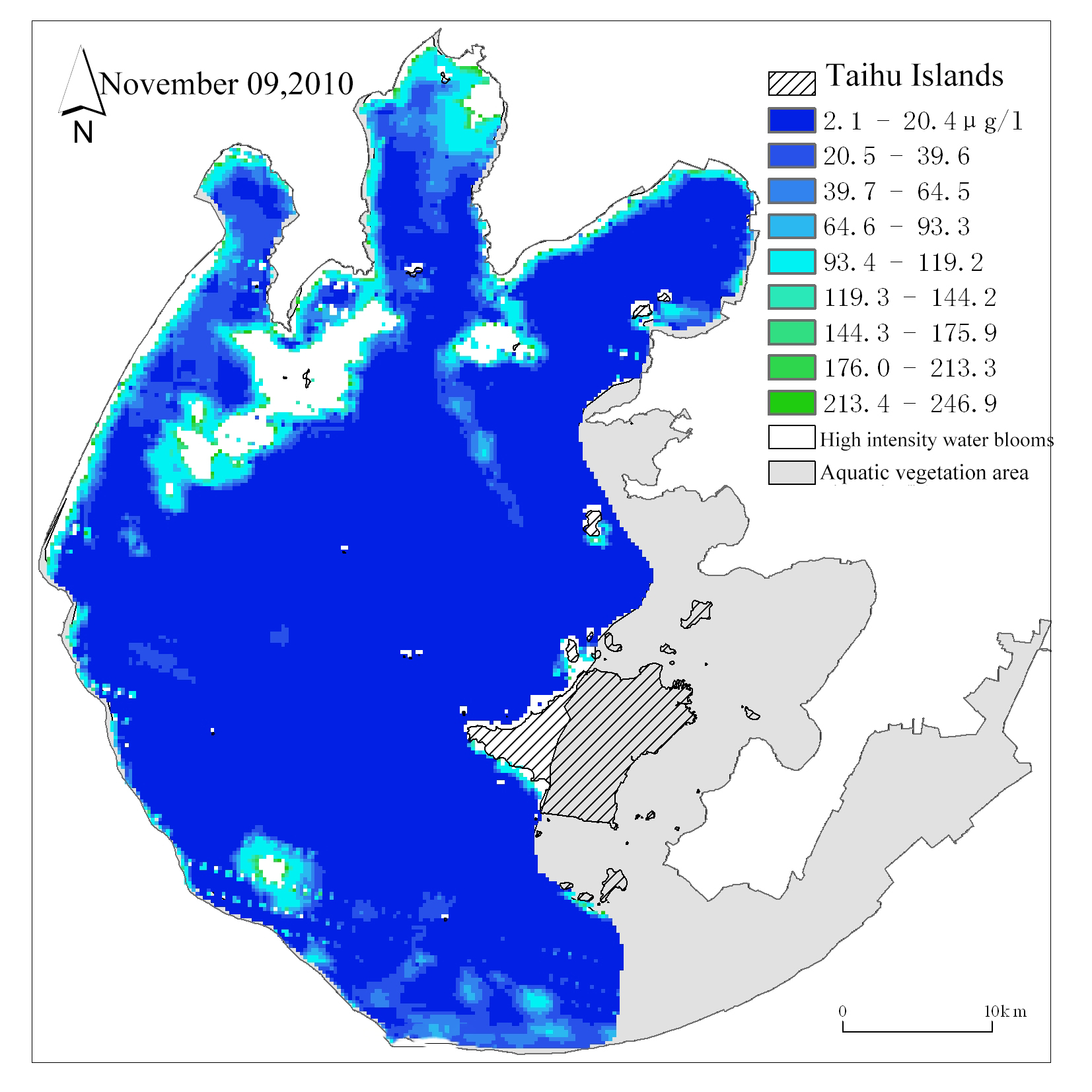

Supplement: Supplemental Information 4 — The data were obtained from the remote sensing image data of chlorophyll a concentration from the Lake-Watershed Science SubCenter, National Earth System Science Data Center, National Science & Technology Infrastructure of China, which had inconsistent data scales, data anomalies and different sampling intervals, and the chlorophyll a concentration unit was µg/L. [file peerj-cs-09-1292-s004.zip › 201011091123_taihu_chla.jpg]

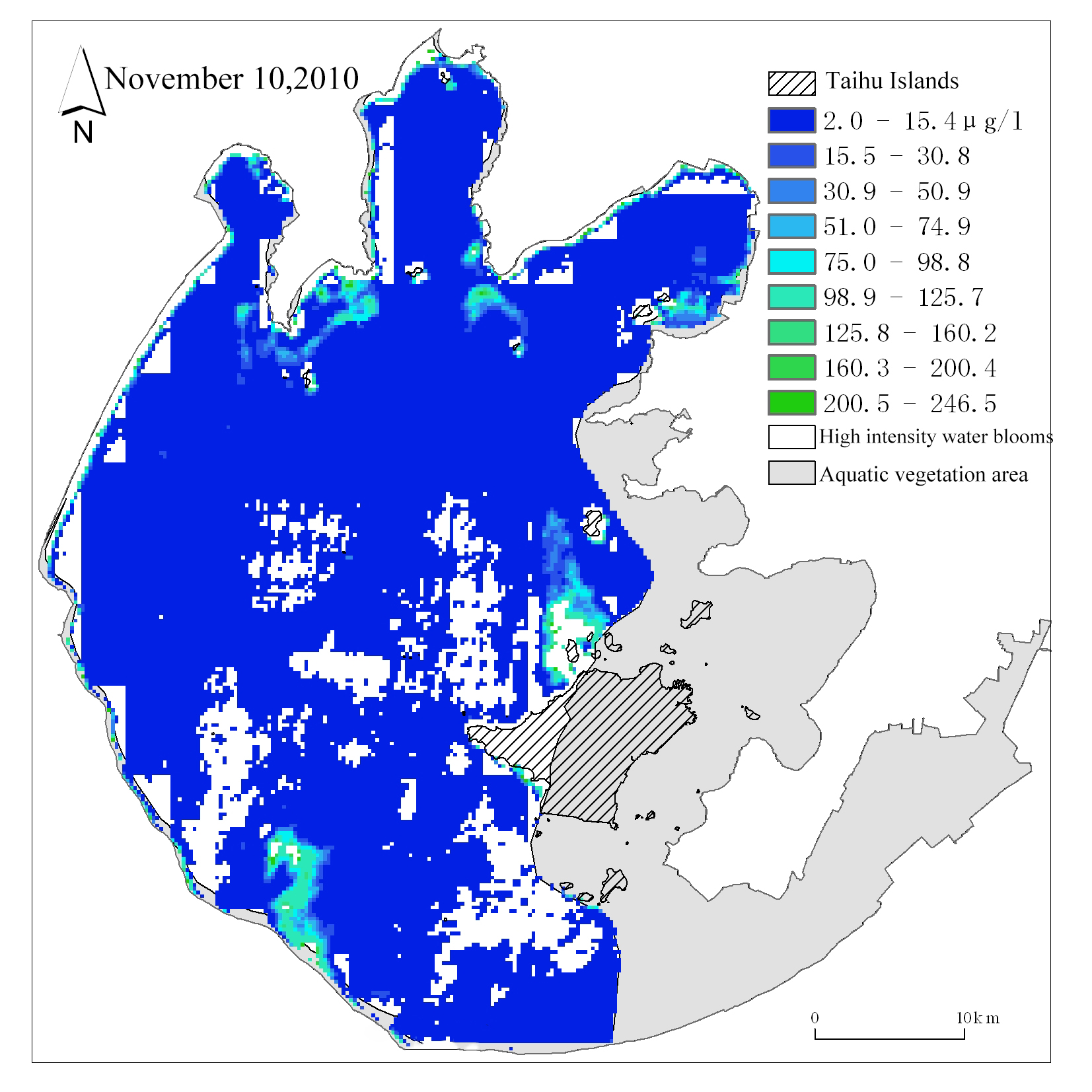

Supplement: Supplemental Information 4 — The data were obtained from the remote sensing image data of chlorophyll a concentration from the Lake-Watershed Science SubCenter, National Earth System Science Data Center, National Science & Technology Infrastructure of China, which had inconsistent data scales, data anomalies and different sampling intervals, and the chlorophyll a concentration unit was µg/L. [file peerj-cs-09-1292-s004.zip › 201011101028_taihu_chla.jpg]

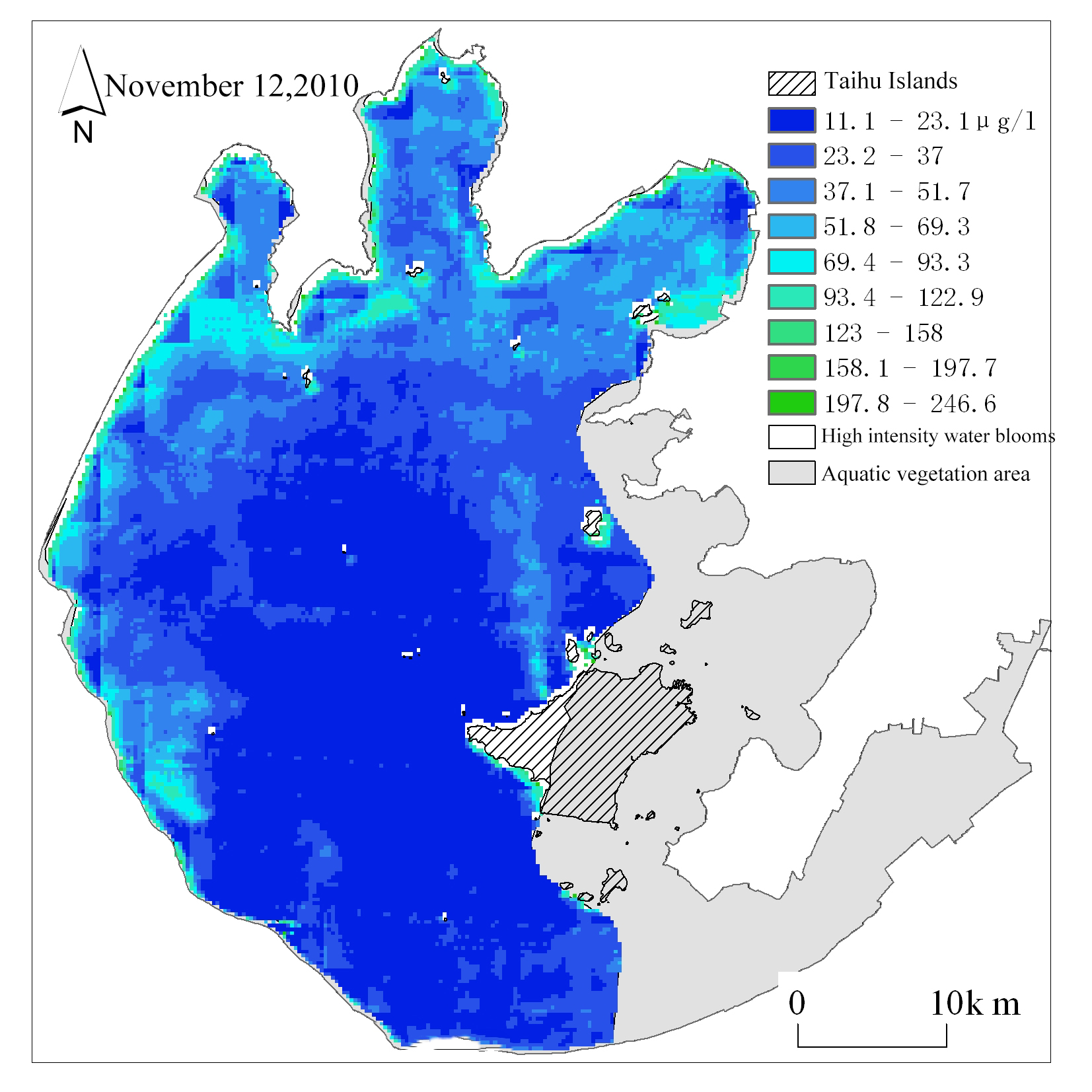

Supplement: Supplemental Information 4 — The data were obtained from the remote sensing image data of chlorophyll a concentration from the Lake-Watershed Science SubCenter, National Earth System Science Data Center, National Science & Technology Infrastructure of China, which had inconsistent data scales, data anomalies and different sampling intervals, and the chlorophyll a concentration unit was µg/L. [file peerj-cs-09-1292-s004.zip › 201011121016_taihu_chla.jpg]

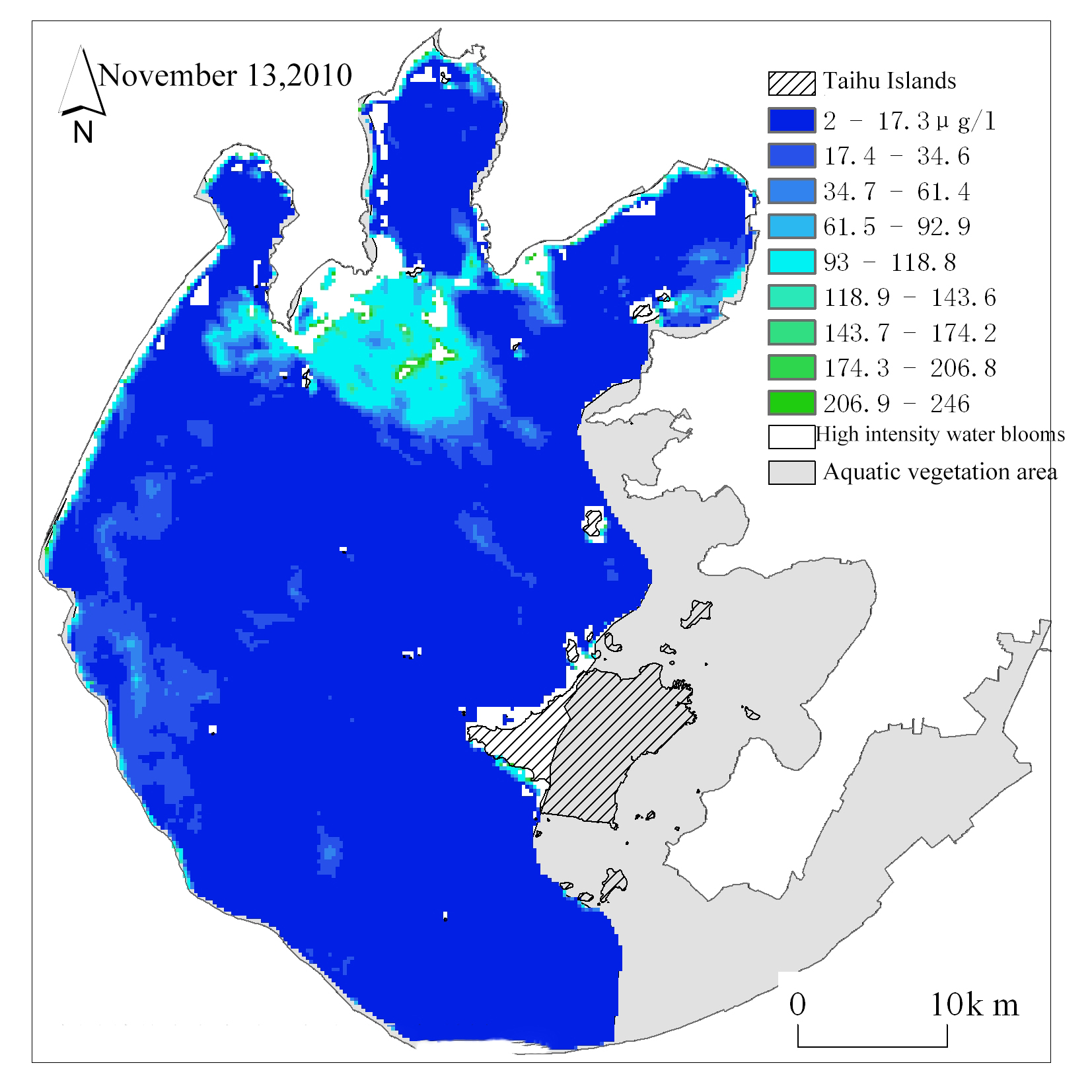

Supplement: Supplemental Information 4 — The data were obtained from the remote sensing image data of chlorophyll a concentration from the Lake-Watershed Science SubCenter, National Earth System Science Data Center, National Science & Technology Infrastructure of China, which had inconsistent data scales, data anomalies and different sampling intervals, and the chlorophyll a concentration unit was µg/L. [file peerj-cs-09-1292-s004.zip › 201011131102_taihu_chla.jpg]

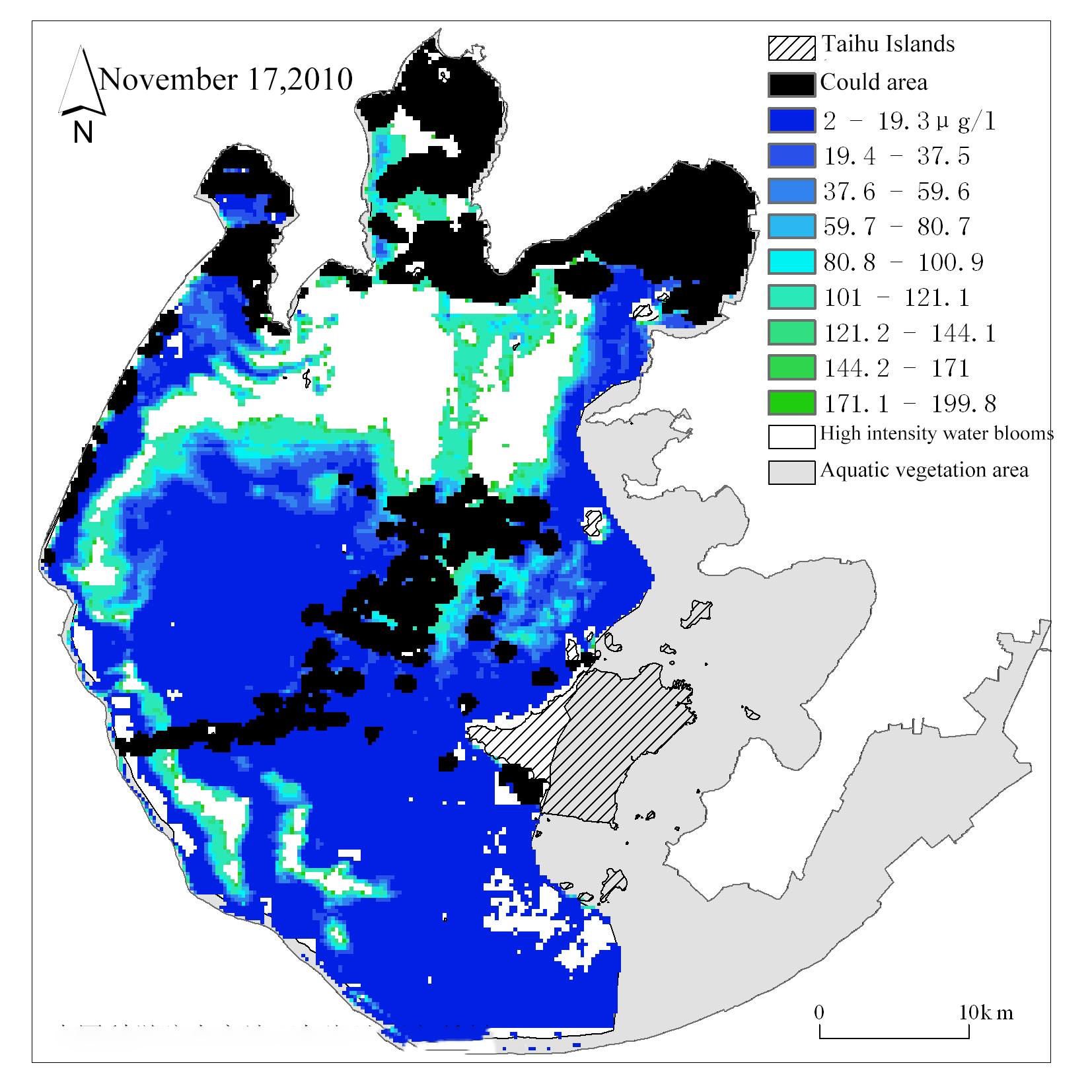

Supplement: Supplemental Information 4 — The data were obtained from the remote sensing image data of chlorophyll a concentration from the Lake-Watershed Science SubCenter, National Earth System Science Data Center, National Science & Technology Infrastructure of China, which had inconsistent data scales, data anomalies and different sampling intervals, and the chlorophyll a concentration unit was µg/L. [file peerj-cs-09-1292-s004.zip › 201011171040_taihu_chla.jpg]

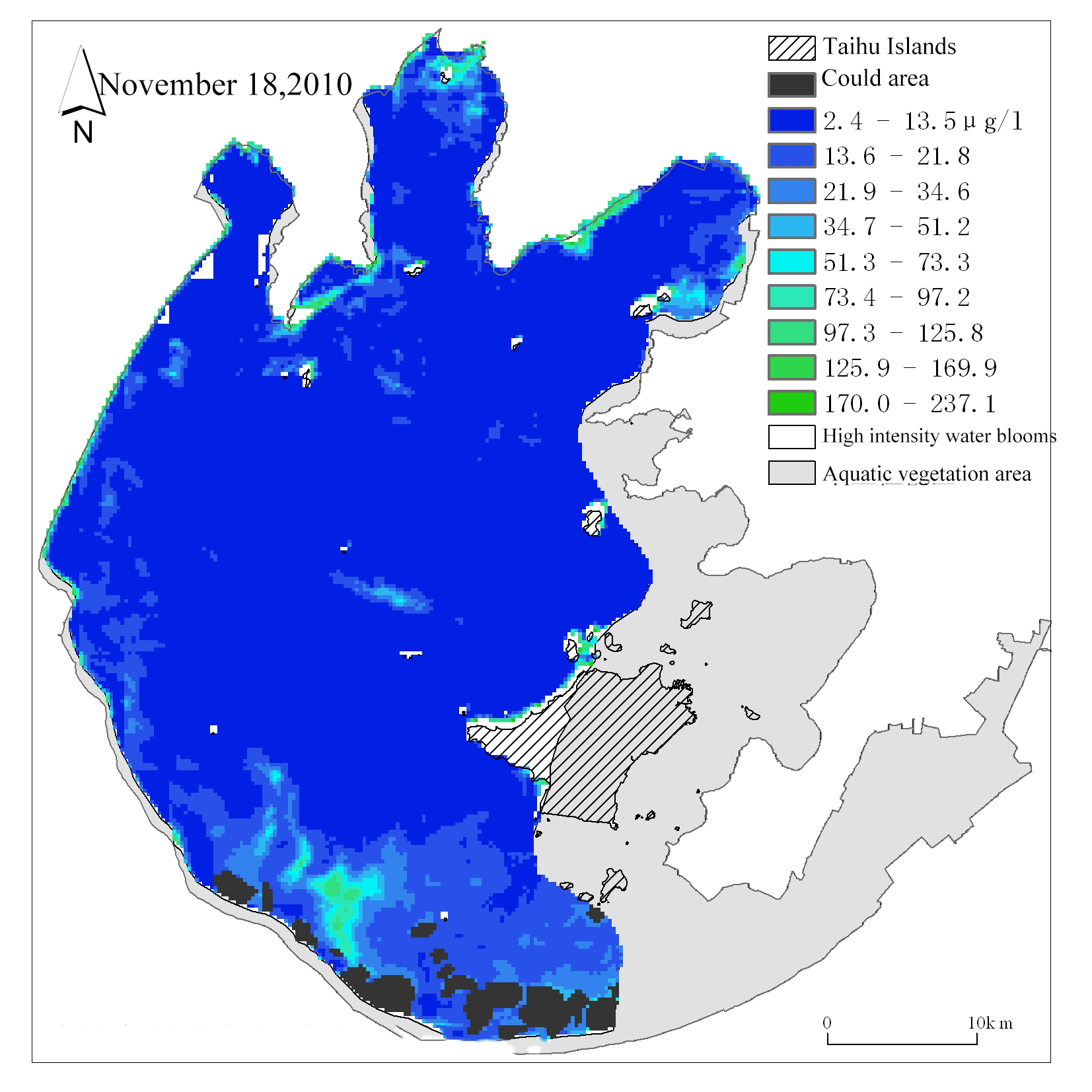

Supplement: Supplemental Information 4 — The data were obtained from the remote sensing image data of chlorophyll a concentration from the Lake-Watershed Science SubCenter, National Earth System Science Data Center, National Science & Technology Infrastructure of China, which had inconsistent data scales, data anomalies and different sampling intervals, and the chlorophyll a concentration unit was µg/L. [file peerj-cs-09-1292-s004.zip › 201011181258_taihu_chla.jpg]

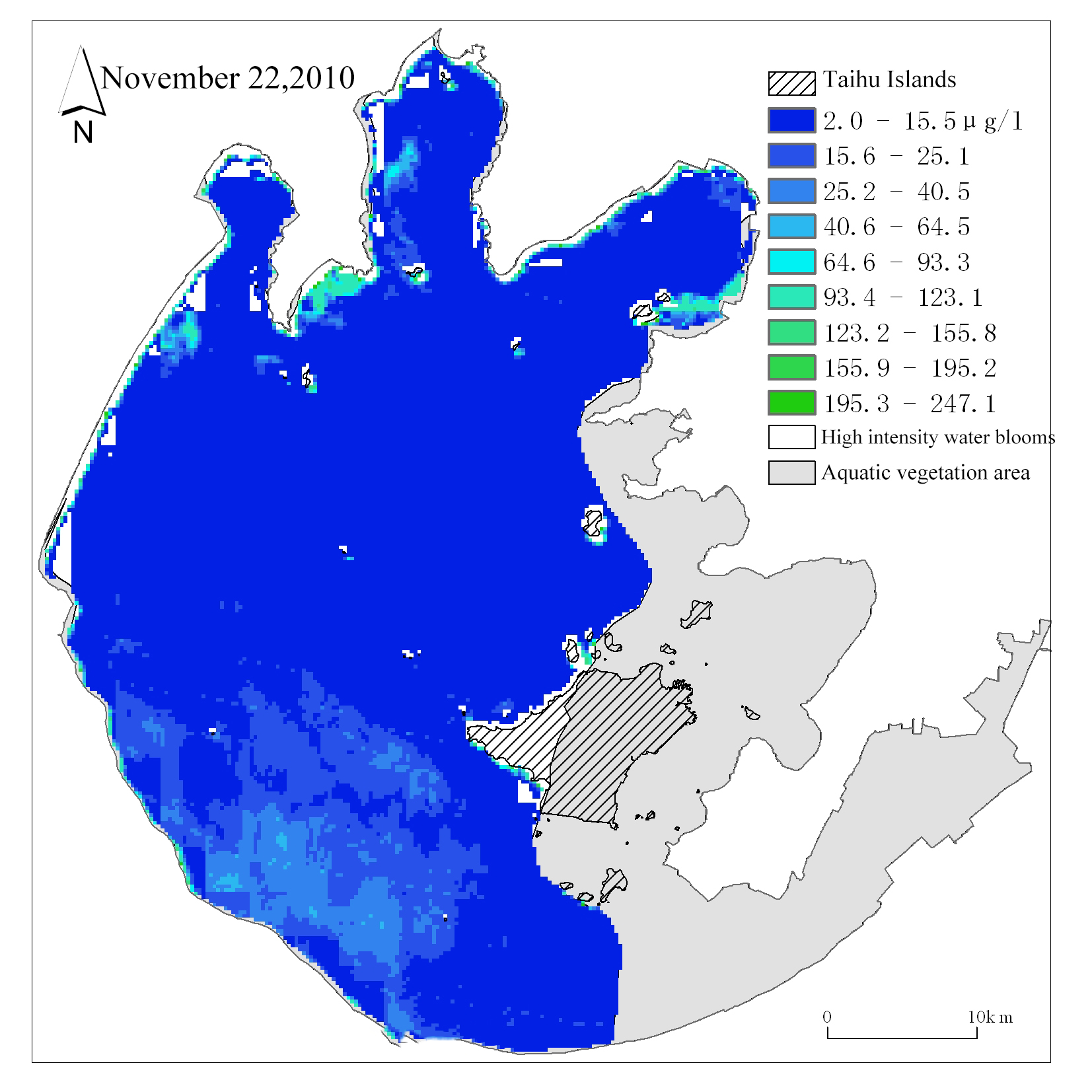

Supplement: Supplemental Information 4 — The data were obtained from the remote sensing image data of chlorophyll a concentration from the Lake-Watershed Science SubCenter, National Earth System Science Data Center, National Science & Technology Infrastructure of China, which had inconsistent data scales, data anomalies and different sampling intervals, and the chlorophyll a concentration unit was µg/L. [file peerj-cs-09-1292-s004.zip › 201011220256_taihu_chla.jpg]

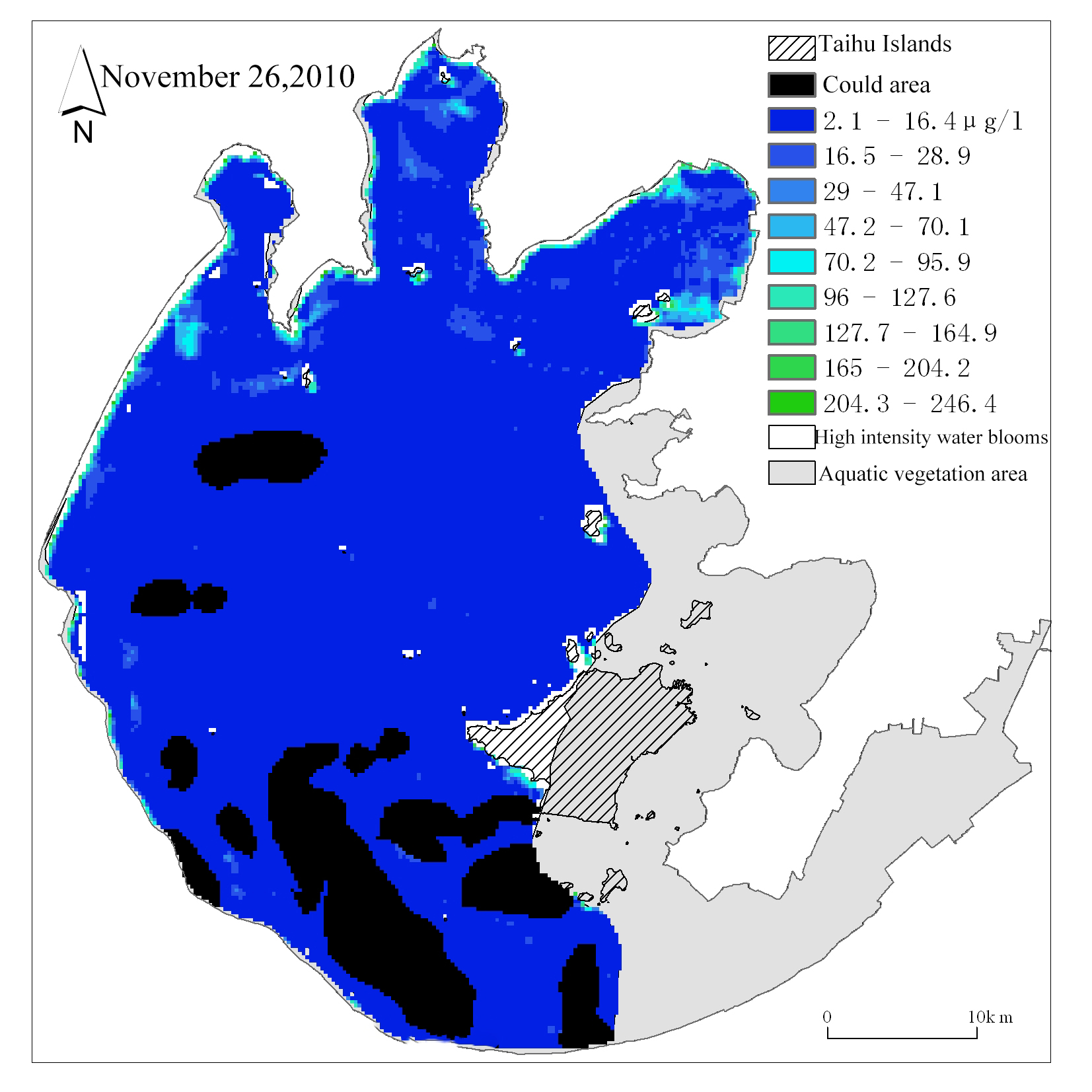

Supplement: Supplemental Information 4 — The data were obtained from the remote sensing image data of chlorophyll a concentration from the Lake-Watershed Science SubCenter, National Earth System Science Data Center, National Science & Technology Infrastructure of China, which had inconsistent data scales, data anomalies and different sampling intervals, and the chlorophyll a concentration unit was µg/L. [file peerj-cs-09-1292-s004.zip › 201011261028_taihu_chl-a.jpg]

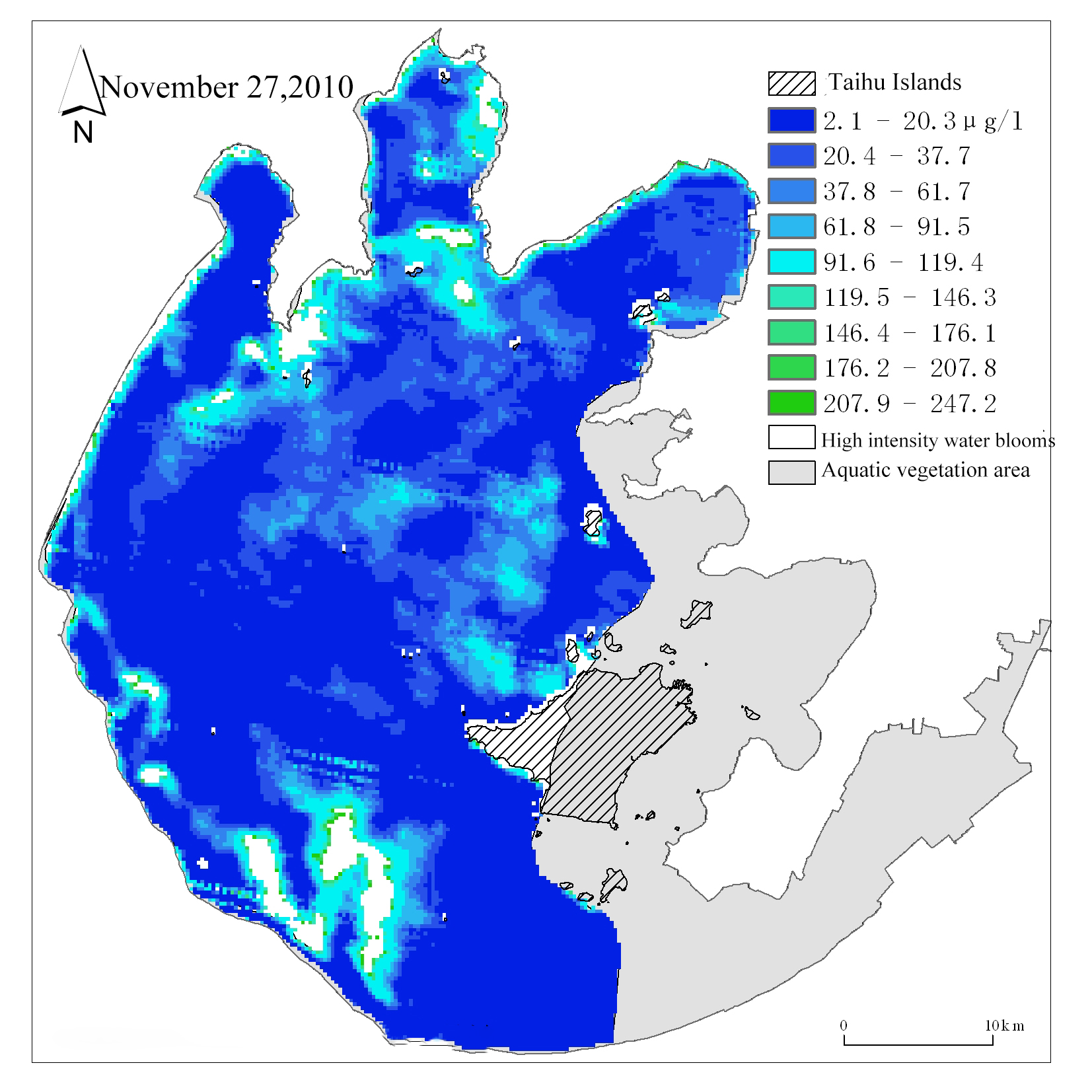

Supplement: Supplemental Information 4 — The data were obtained from the remote sensing image data of chlorophyll a concentration from the Lake-Watershed Science SubCenter, National Earth System Science Data Center, National Science & Technology Infrastructure of China, which had inconsistent data scales, data anomalies and different sampling intervals, and the chlorophyll a concentration unit was µg/L. [file peerj-cs-09-1292-s004.zip › 201011271115_taihu_chl-a.jpg]

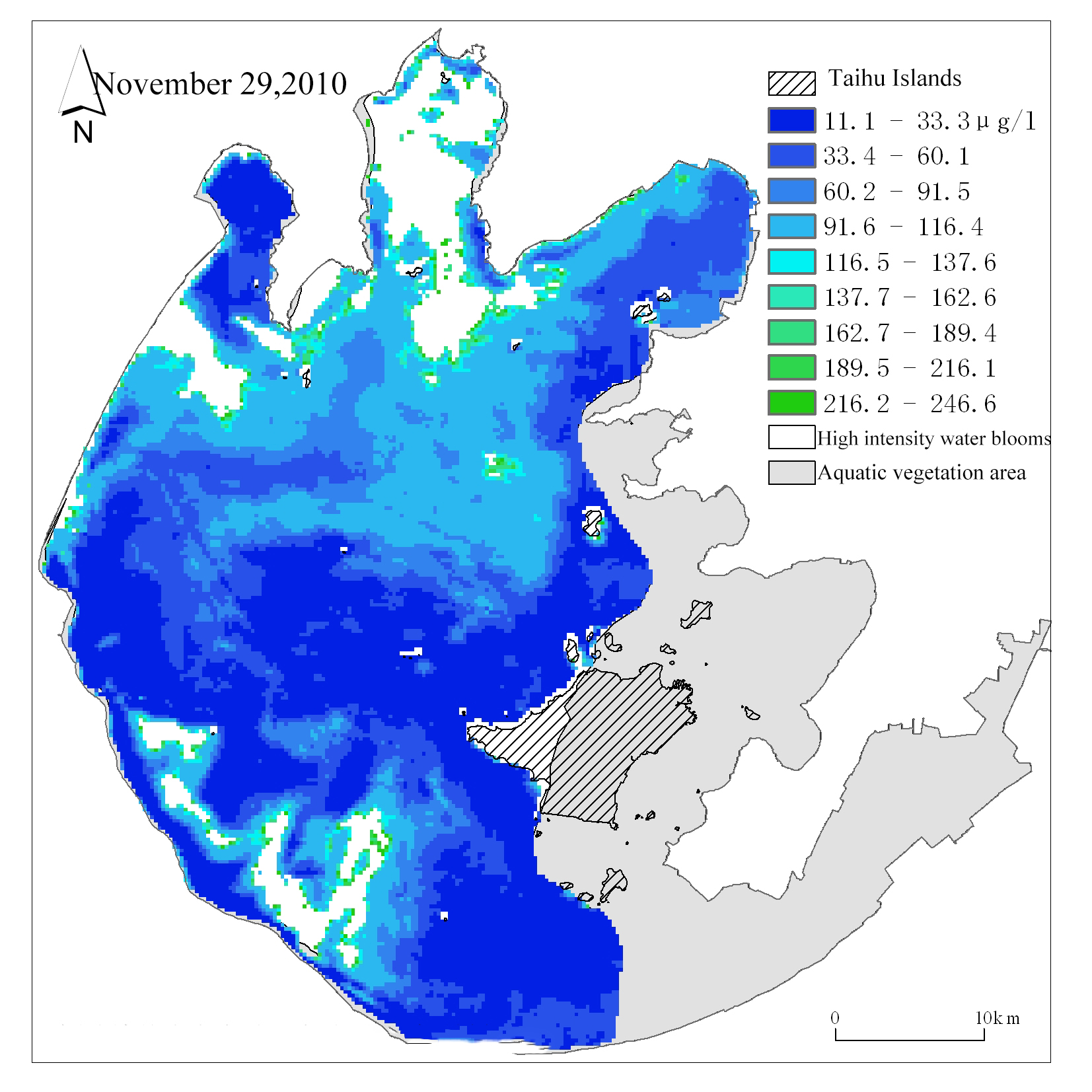

Supplement: Supplemental Information 4 — The data were obtained from the remote sensing image data of chlorophyll a concentration from the Lake-Watershed Science SubCenter, National Earth System Science Data Center, National Science & Technology Infrastructure of China, which had inconsistent data scales, data anomalies and different sampling intervals, and the chlorophyll a concentration unit was µg/L. [file peerj-cs-09-1292-s004.zip › 201011291059_taihu_chla.jpg]

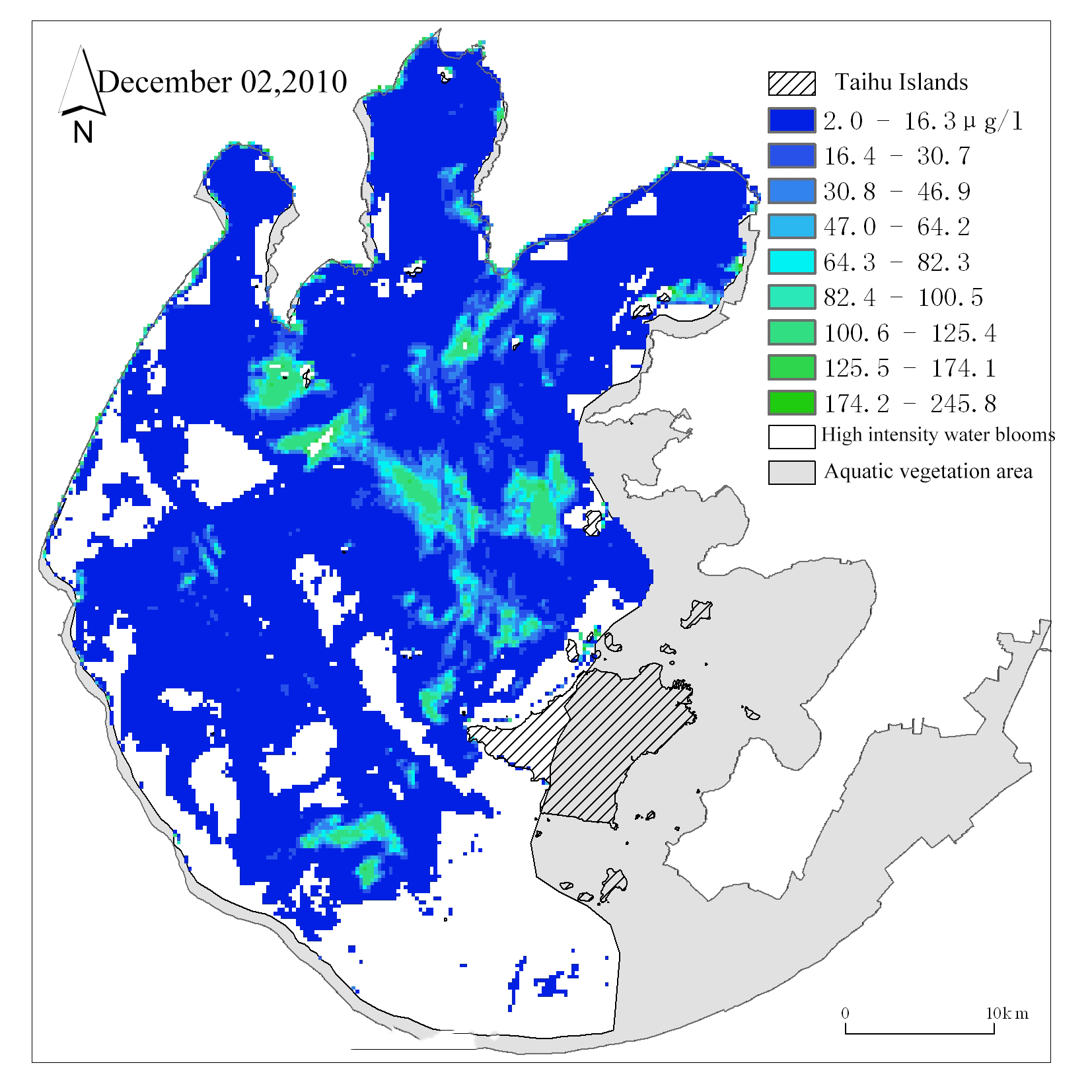

Supplement: Supplemental Information 4 — The data were obtained from the remote sensing image data of chlorophyll a concentration from the Lake-Watershed Science SubCenter, National Earth System Science Data Center, National Science & Technology Infrastructure of China, which had inconsistent data scales, data anomalies and different sampling intervals, and the chlorophyll a concentration unit was µg/L. [file peerj-cs-09-1292-s004.zip › 201012021310_taihu_chla.jpg]

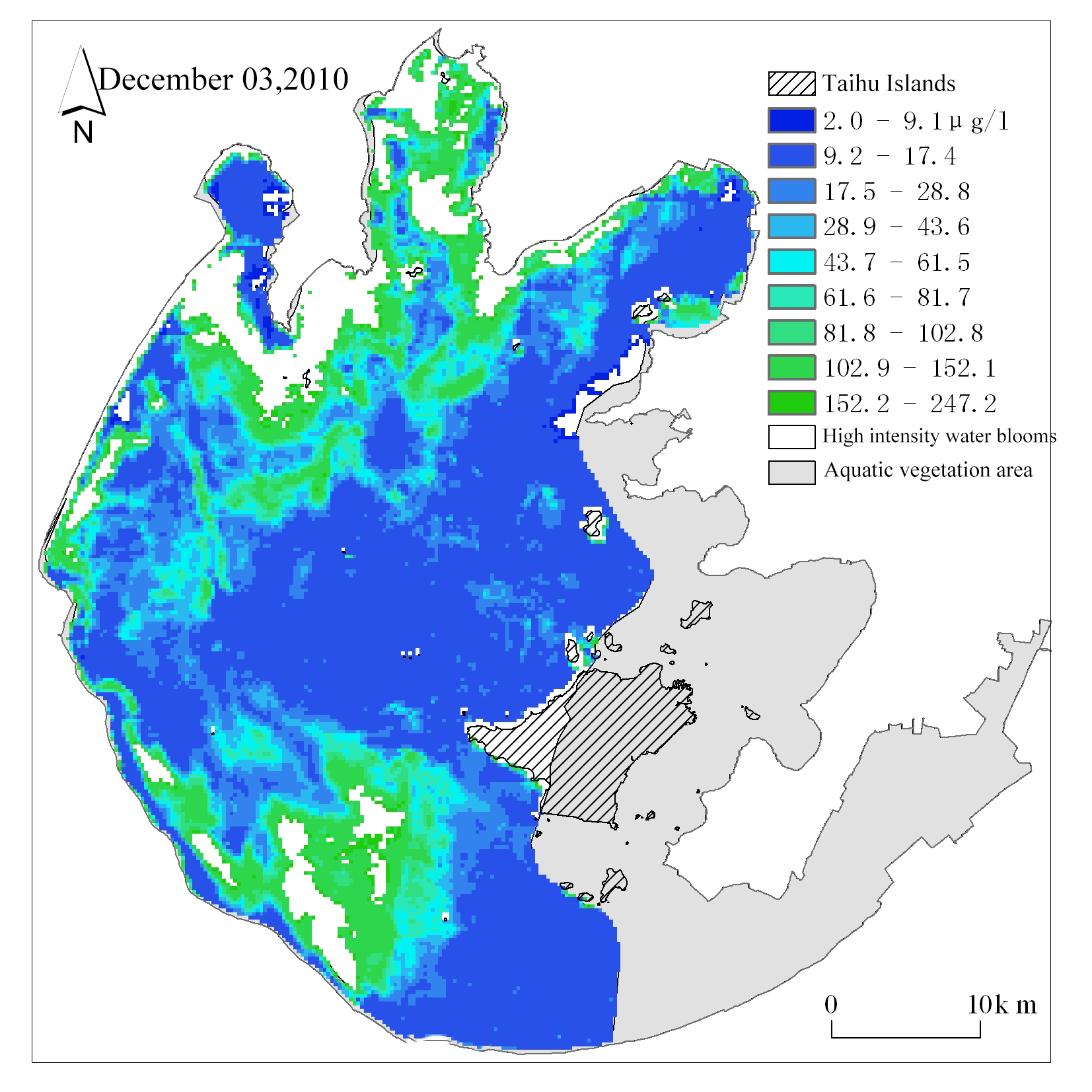

Supplement: Supplemental Information 4 — The data were obtained from the remote sensing image data of chlorophyll a concentration from the Lake-Watershed Science SubCenter, National Earth System Science Data Center, National Science & Technology Infrastructure of China, which had inconsistent data scales, data anomalies and different sampling intervals, and the chlorophyll a concentration unit was µg/L. [file peerj-cs-09-1292-s004.zip › 201012031034_taihu_chla.jpg]

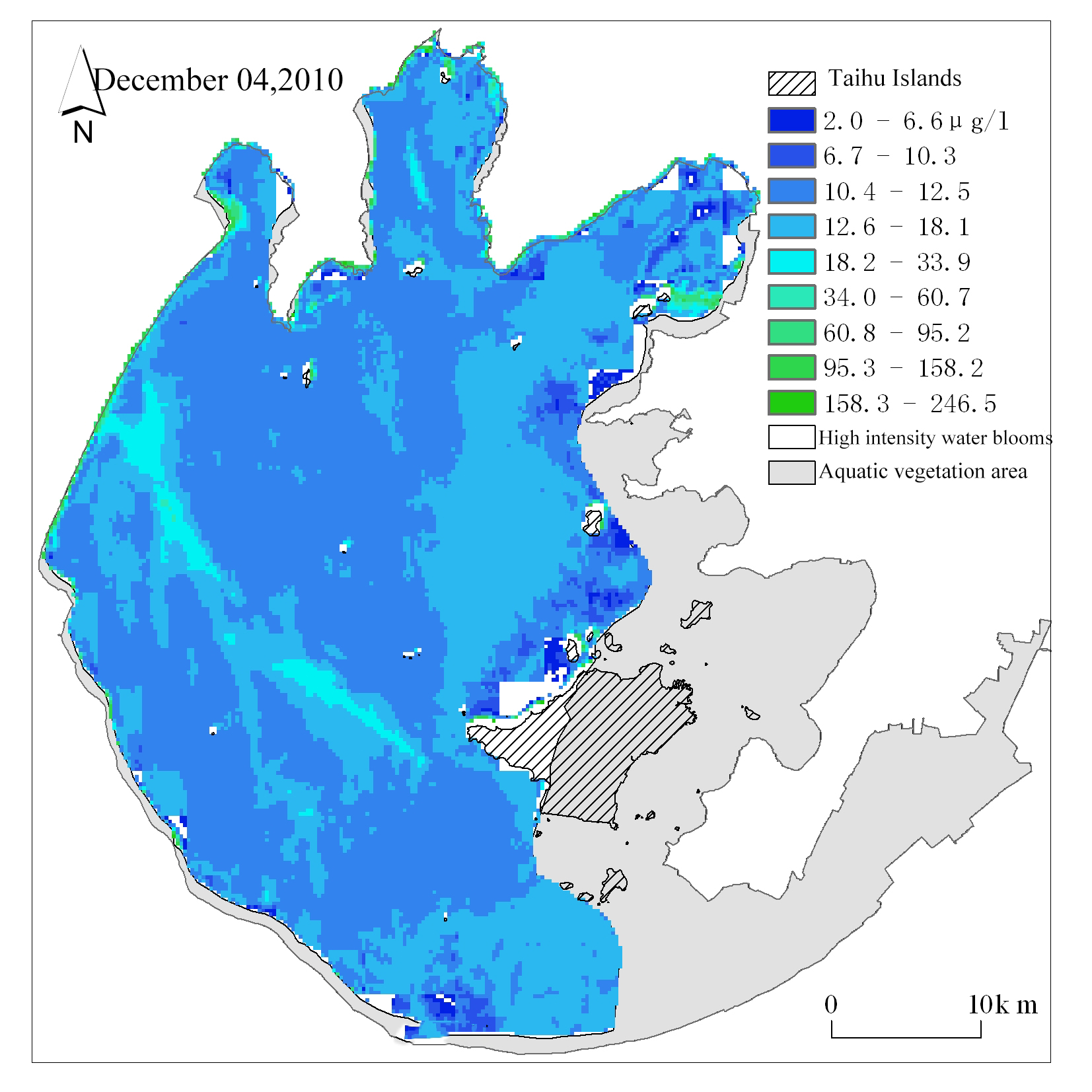

Supplement: Supplemental Information 4 — The data were obtained from the remote sensing image data of chlorophyll a concentration from the Lake-Watershed Science SubCenter, National Earth System Science Data Center, National Science & Technology Infrastructure of China, which had inconsistent data scales, data anomalies and different sampling intervals, and the chlorophyll a concentration unit was µg/L. [file peerj-cs-09-1292-s004.zip › 201012040455_taihu_chla.jpg]

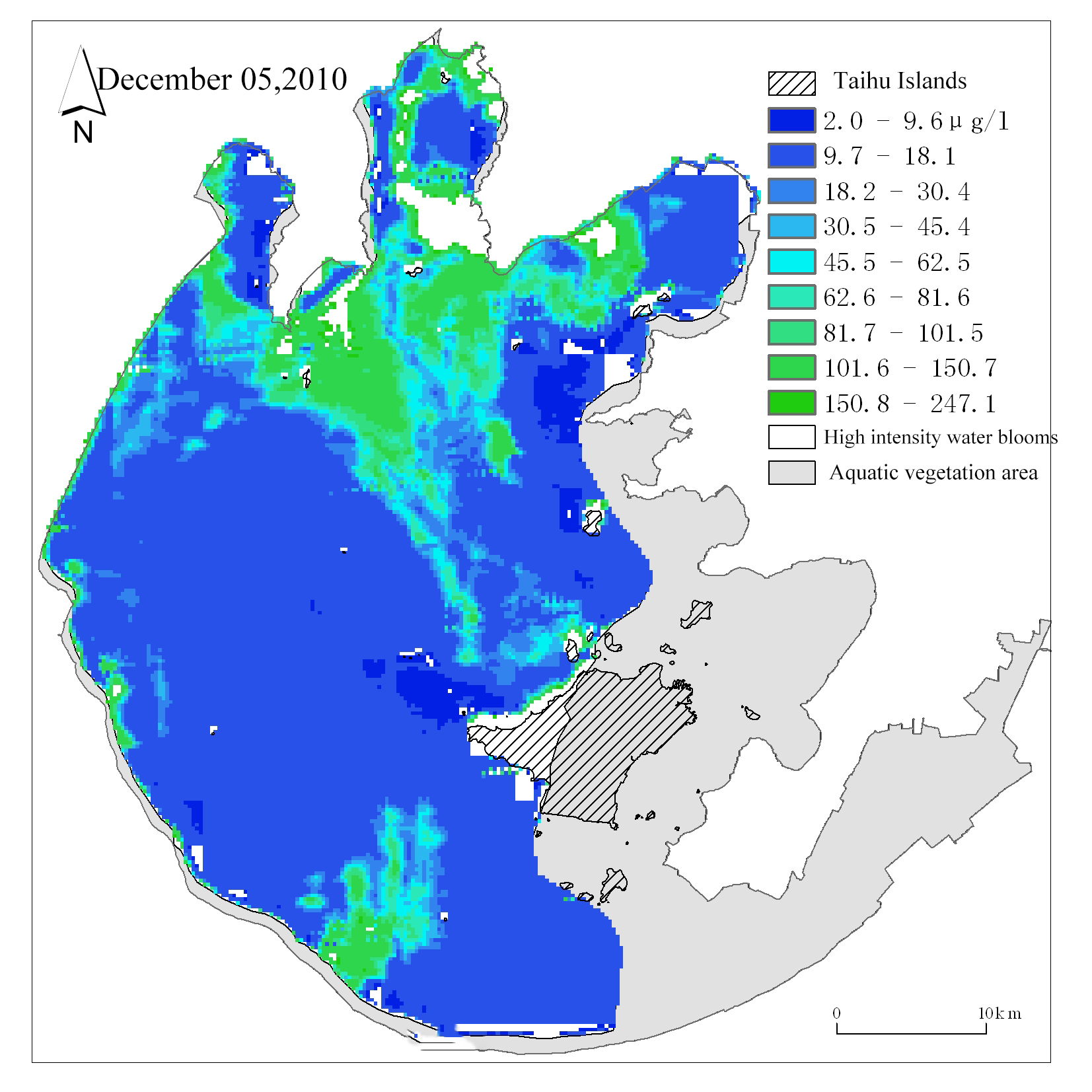

Supplement: Supplemental Information 4 — The data were obtained from the remote sensing image data of chlorophyll a concentration from the Lake-Watershed Science SubCenter, National Earth System Science Data Center, National Science & Technology Infrastructure of China, which had inconsistent data scales, data anomalies and different sampling intervals, and the chlorophyll a concentration unit was µg/L. [file peerj-cs-09-1292-s004.zip › 201012050536_taihu_chla.jpg]

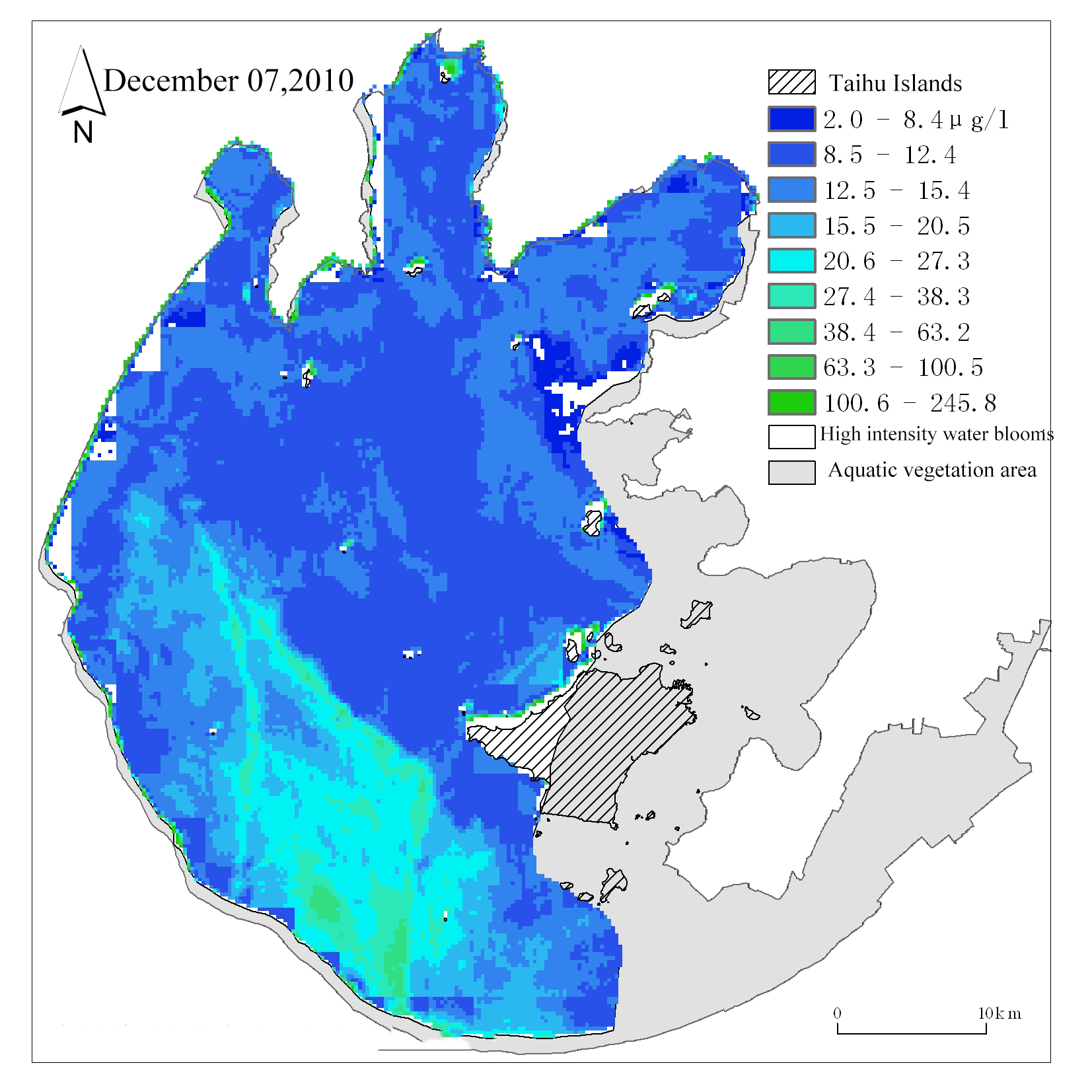

Supplement: Supplemental Information 4 — The data were obtained from the remote sensing image data of chlorophyll a concentration from the Lake-Watershed Science SubCenter, National Earth System Science Data Center, National Science & Technology Infrastructure of China, which had inconsistent data scales, data anomalies and different sampling intervals, and the chlorophyll a concentration unit was µg/L. [file peerj-cs-09-1292-s004.zip › 201012070524_taihu_chla.jpg]

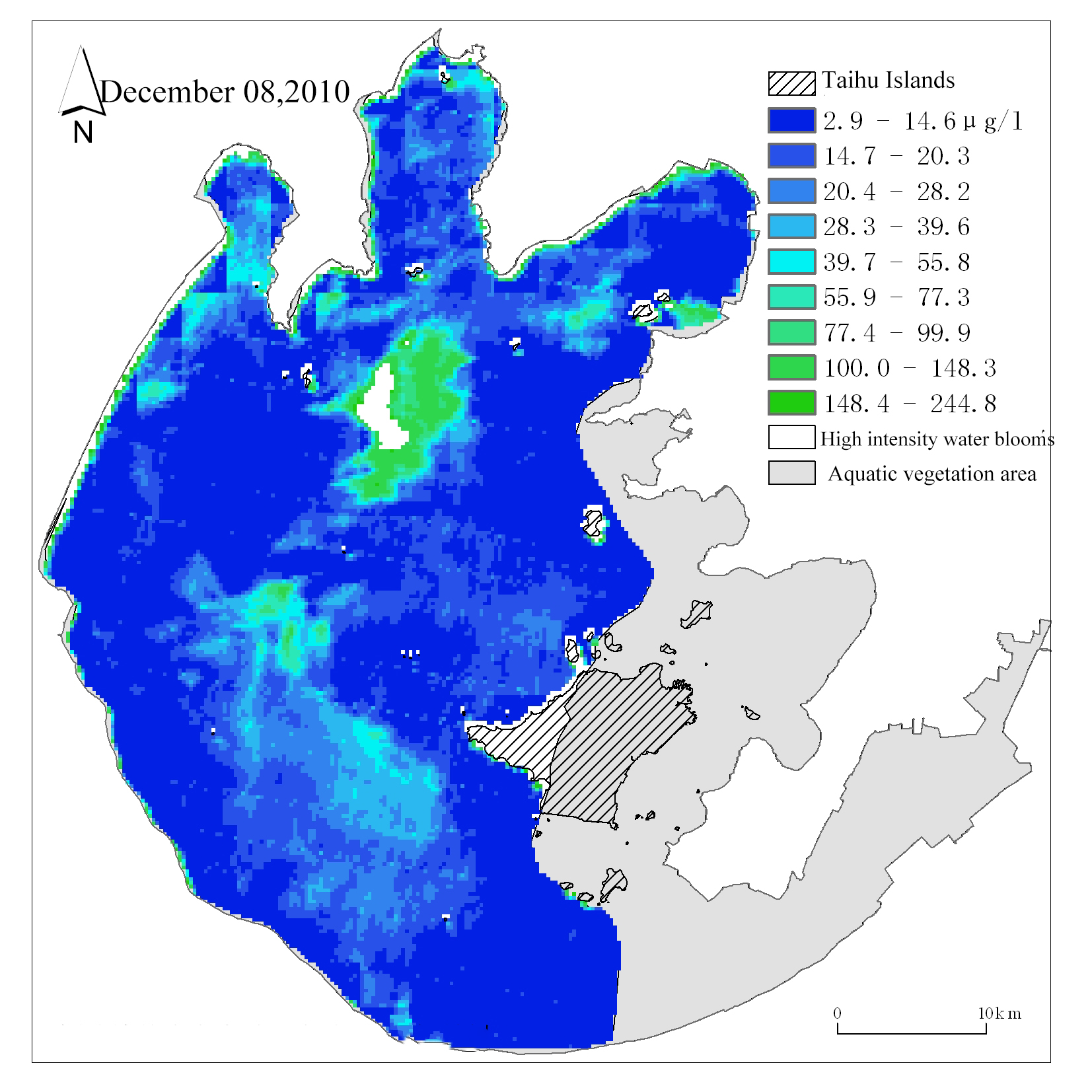

Supplement: Supplemental Information 4 — The data were obtained from the remote sensing image data of chlorophyll a concentration from the Lake-Watershed Science SubCenter, National Earth System Science Data Center, National Science & Technology Infrastructure of China, which had inconsistent data scales, data anomalies and different sampling intervals, and the chlorophyll a concentration unit was µg/L. [file peerj-cs-09-1292-s004.zip › 201012080256_taihu_chla.jpg]

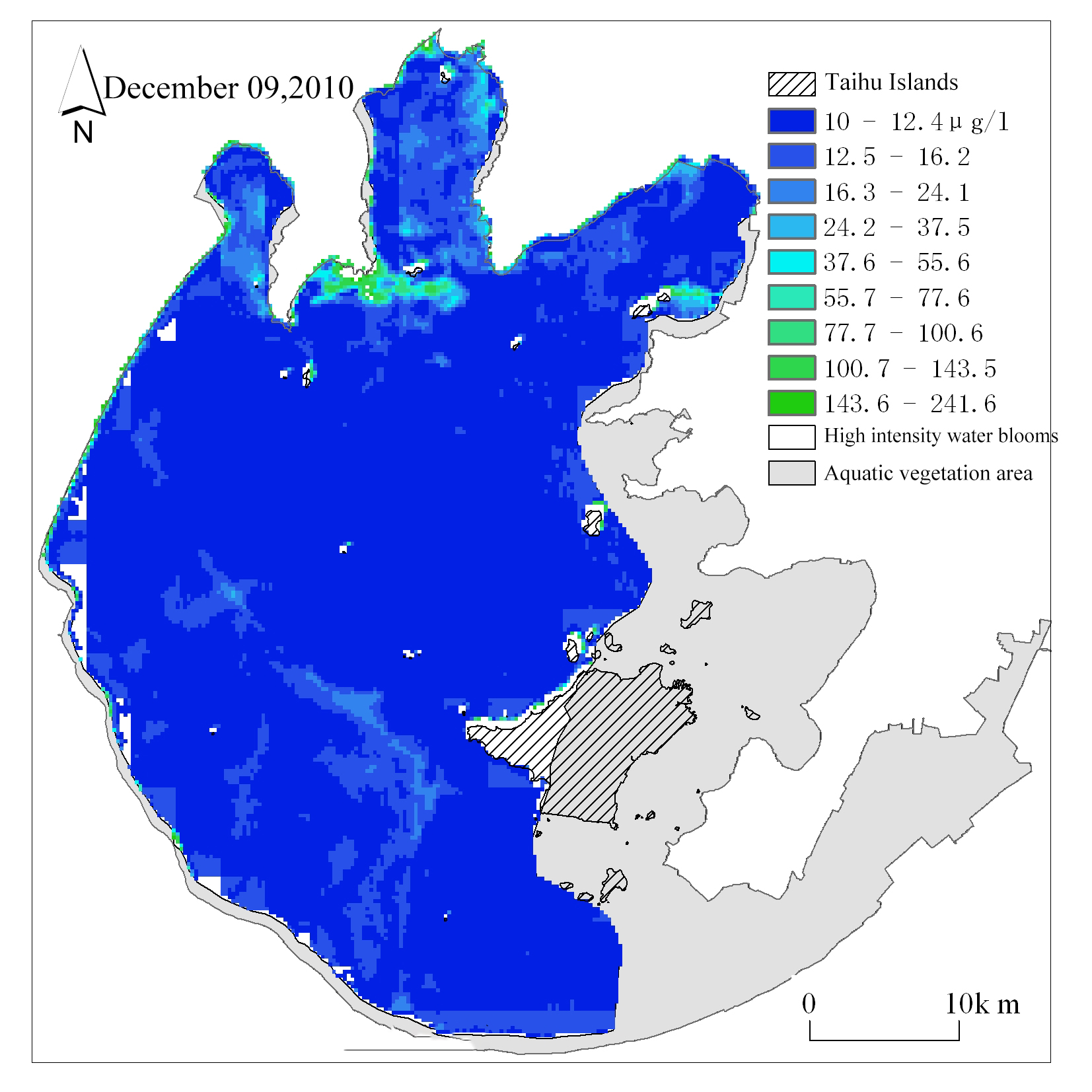

Supplement: Supplemental Information 4 — The data were obtained from the remote sensing image data of chlorophyll a concentration from the Lake-Watershed Science SubCenter, National Earth System Science Data Center, National Science & Technology Infrastructure of China, which had inconsistent data scales, data anomalies and different sampling intervals, and the chlorophyll a concentration unit was µg/L. [file peerj-cs-09-1292-s004.zip › 201012091311_taihu_chla.jpg]

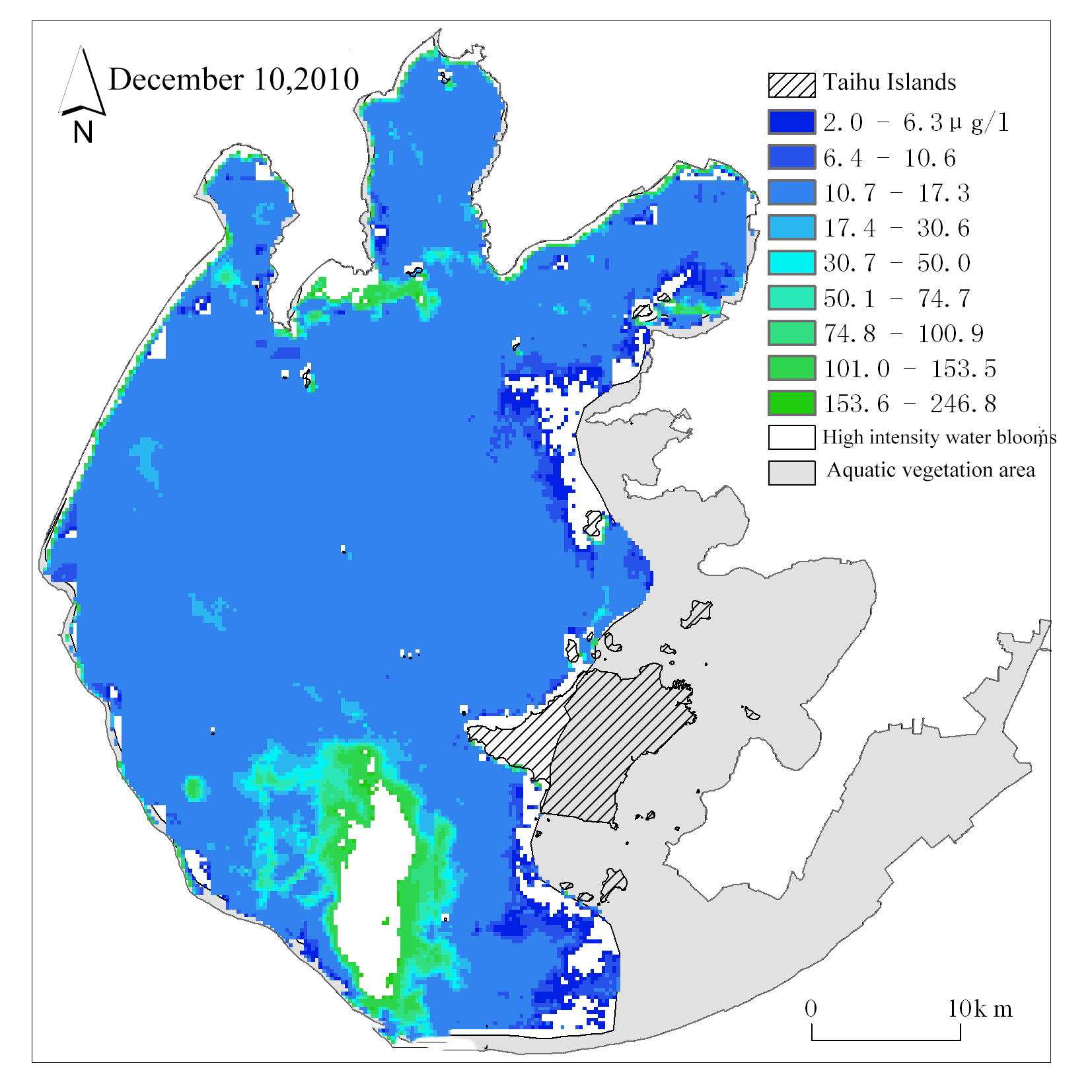

Supplement: Supplemental Information 4 — The data were obtained from the remote sensing image data of chlorophyll a concentration from the Lake-Watershed Science SubCenter, National Earth System Science Data Center, National Science & Technology Infrastructure of China, which had inconsistent data scales, data anomalies and different sampling intervals, and the chlorophyll a concentration unit was µg/L. [file peerj-cs-09-1292-s004.zip › 201012101044_taihu_chla.jpg]

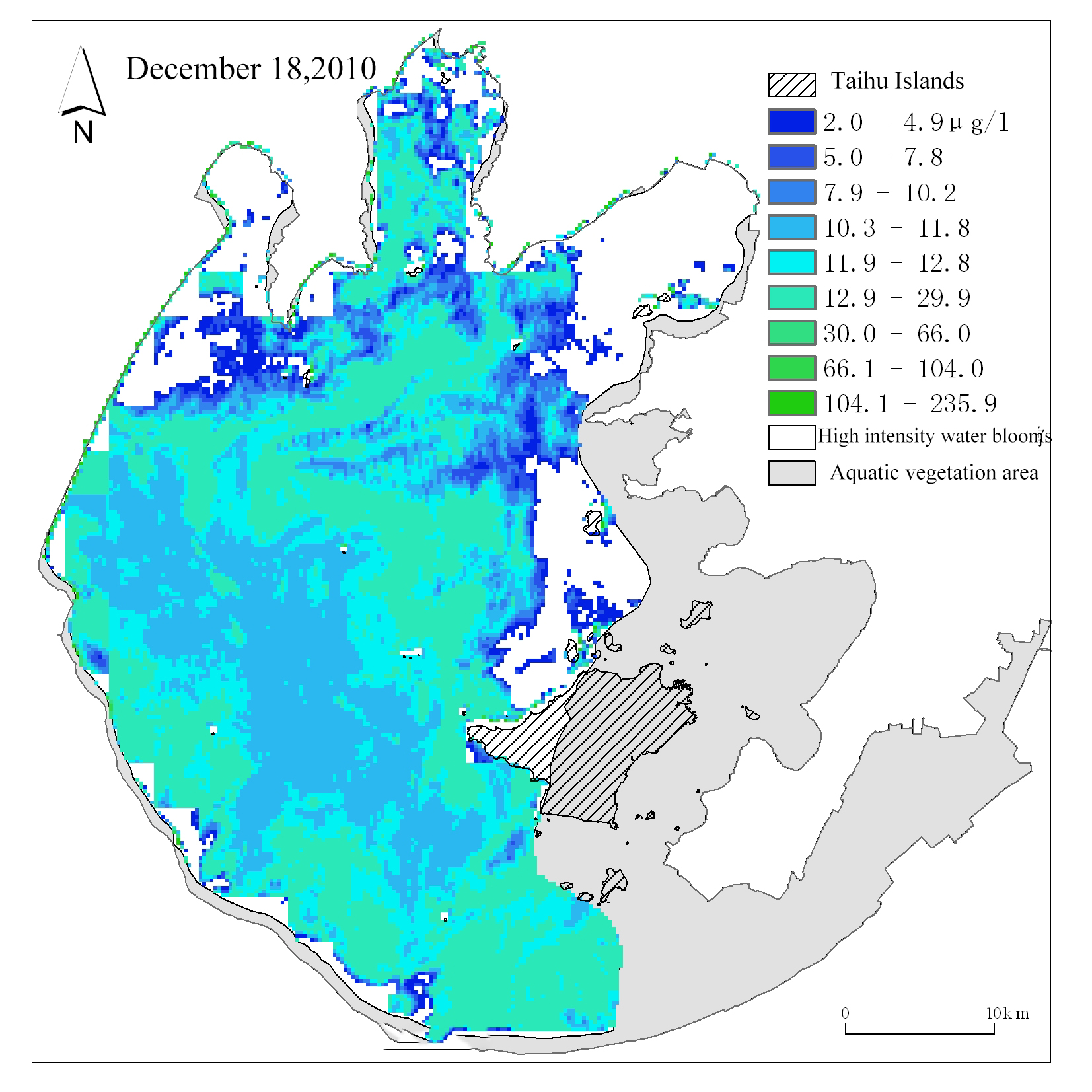

Supplement: Supplemental Information 4 — The data were obtained from the remote sensing image data of chlorophyll a concentration from the Lake-Watershed Science SubCenter, National Earth System Science Data Center, National Science & Technology Infrastructure of China, which had inconsistent data scales, data anomalies and different sampling intervals, and the chlorophyll a concentration unit was µg/L. [file peerj-cs-09-1292-s004.zip › 201012180505_taihu_chla.jpg]

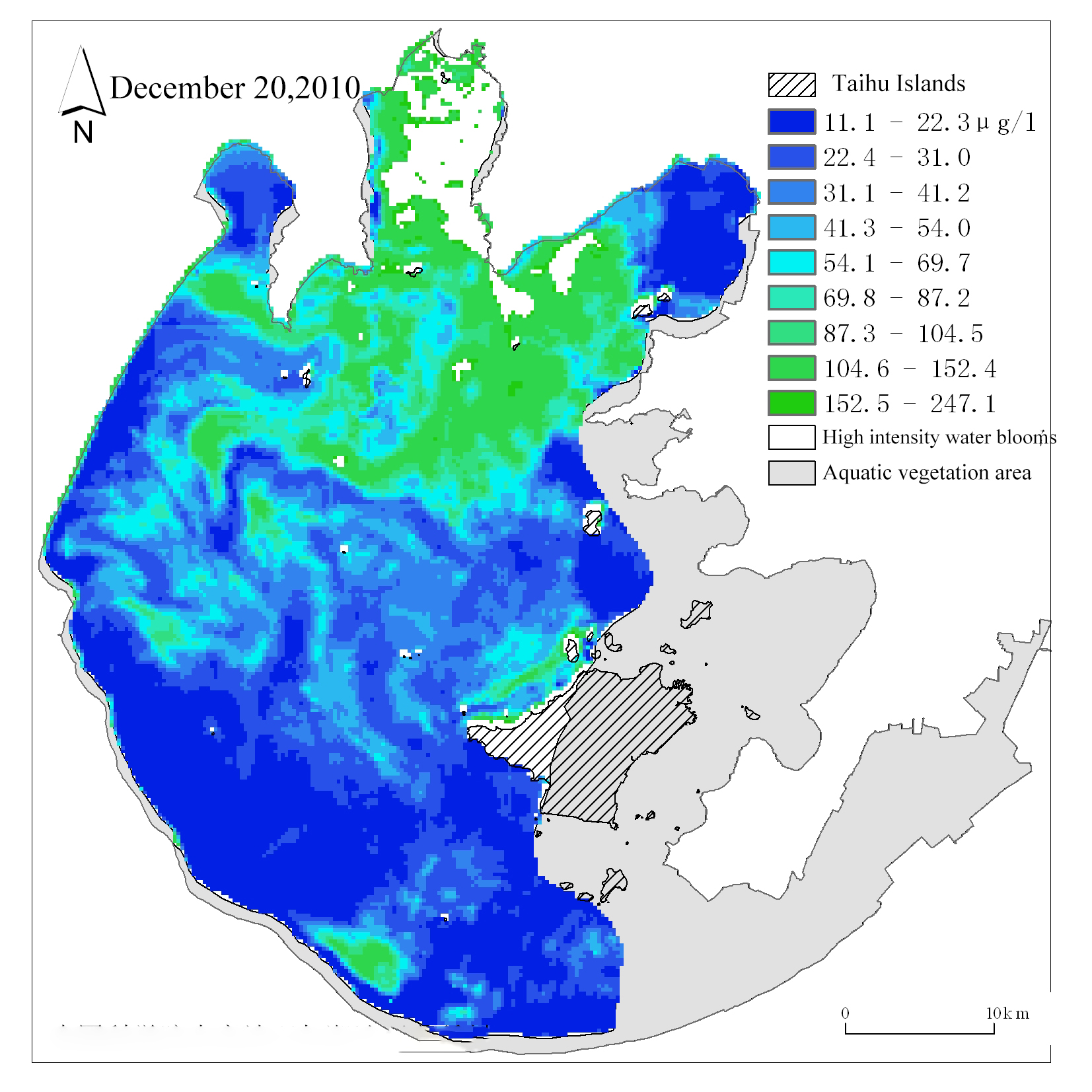

Supplement: Supplemental Information 4 — The data were obtained from the remote sensing image data of chlorophyll a concentration from the Lake-Watershed Science SubCenter, National Earth System Science Data Center, National Science & Technology Infrastructure of China, which had inconsistent data scales, data anomalies and different sampling intervals, and the chlorophyll a concentration unit was µg/L. [file peerj-cs-09-1292-s004.zip › 201012200453_taihu_chla.jpg]

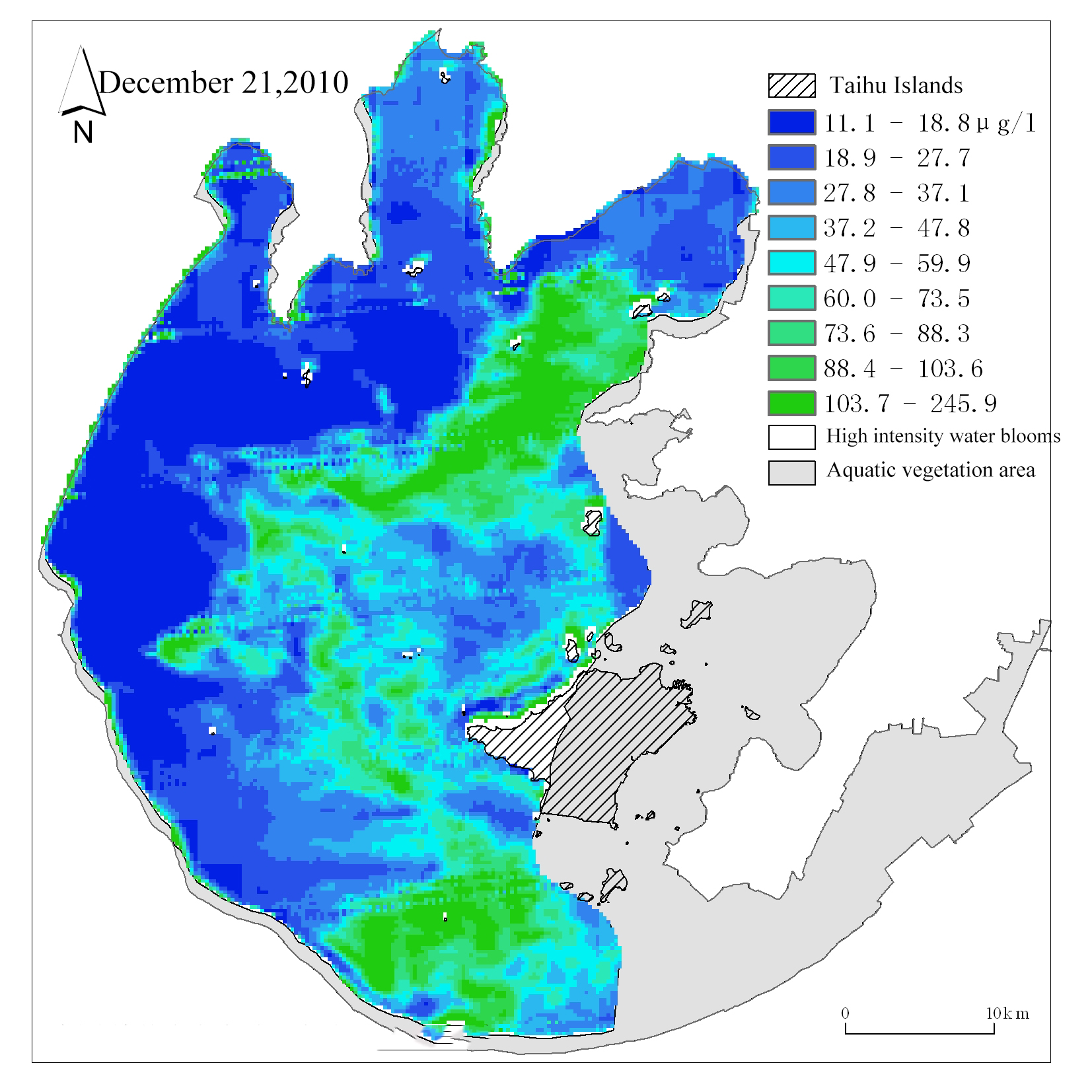

Supplement: Supplemental Information 4 — The data were obtained from the remote sensing image data of chlorophyll a concentration from the Lake-Watershed Science SubCenter, National Earth System Science Data Center, National Science & Technology Infrastructure of China, which had inconsistent data scales, data anomalies and different sampling intervals, and the chlorophyll a concentration unit was µg/L. [file peerj-cs-09-1292-s004.zip › 201012210535_taihu_chla.jpg]

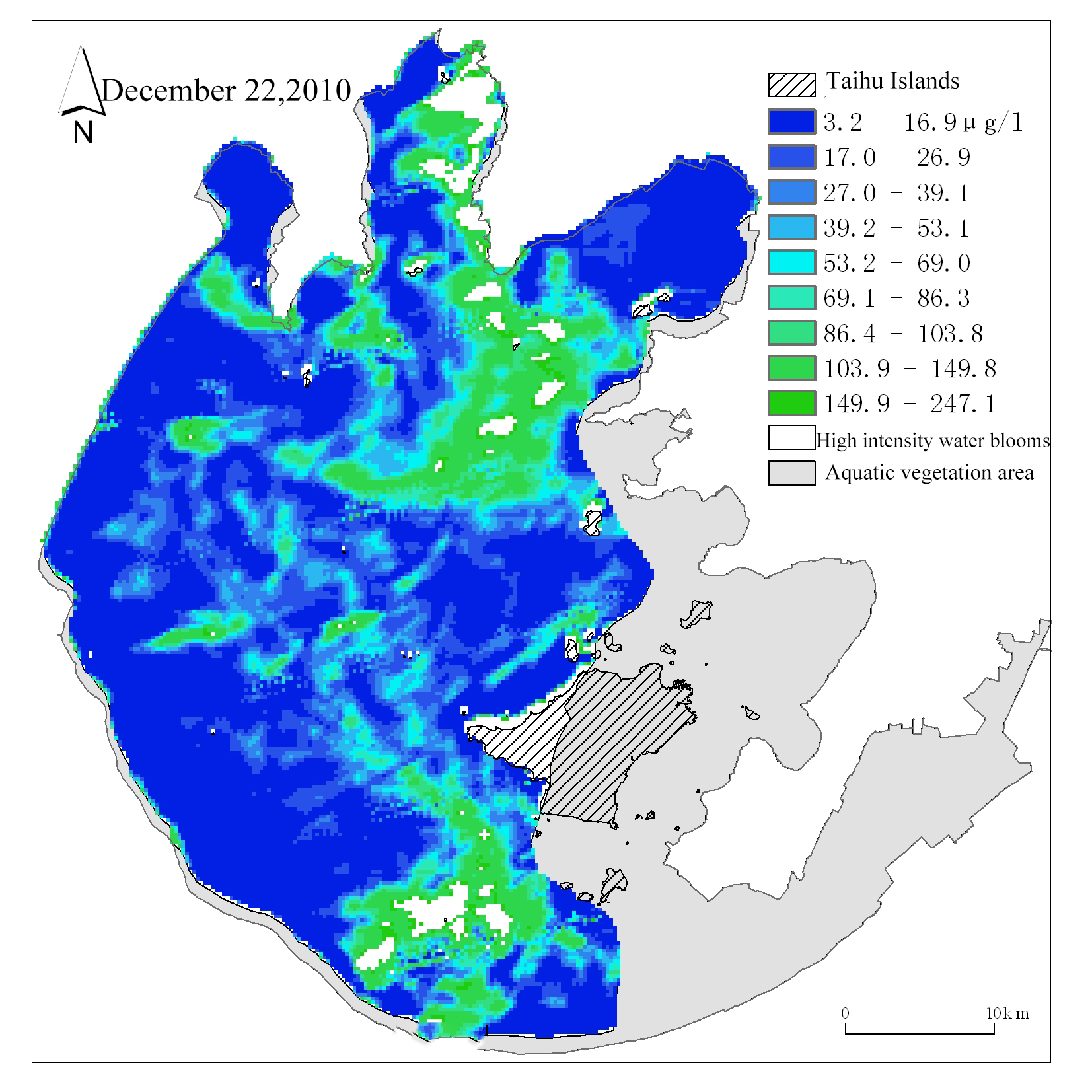

Supplement: Supplemental Information 4 — The data were obtained from the remote sensing image data of chlorophyll a concentration from the Lake-Watershed Science SubCenter, National Earth System Science Data Center, National Science & Technology Infrastructure of China, which had inconsistent data scales, data anomalies and different sampling intervals, and the chlorophyll a concentration unit was µg/L. [file peerj-cs-09-1292-s004.zip › 201012220442_taihu_chla.jpg]

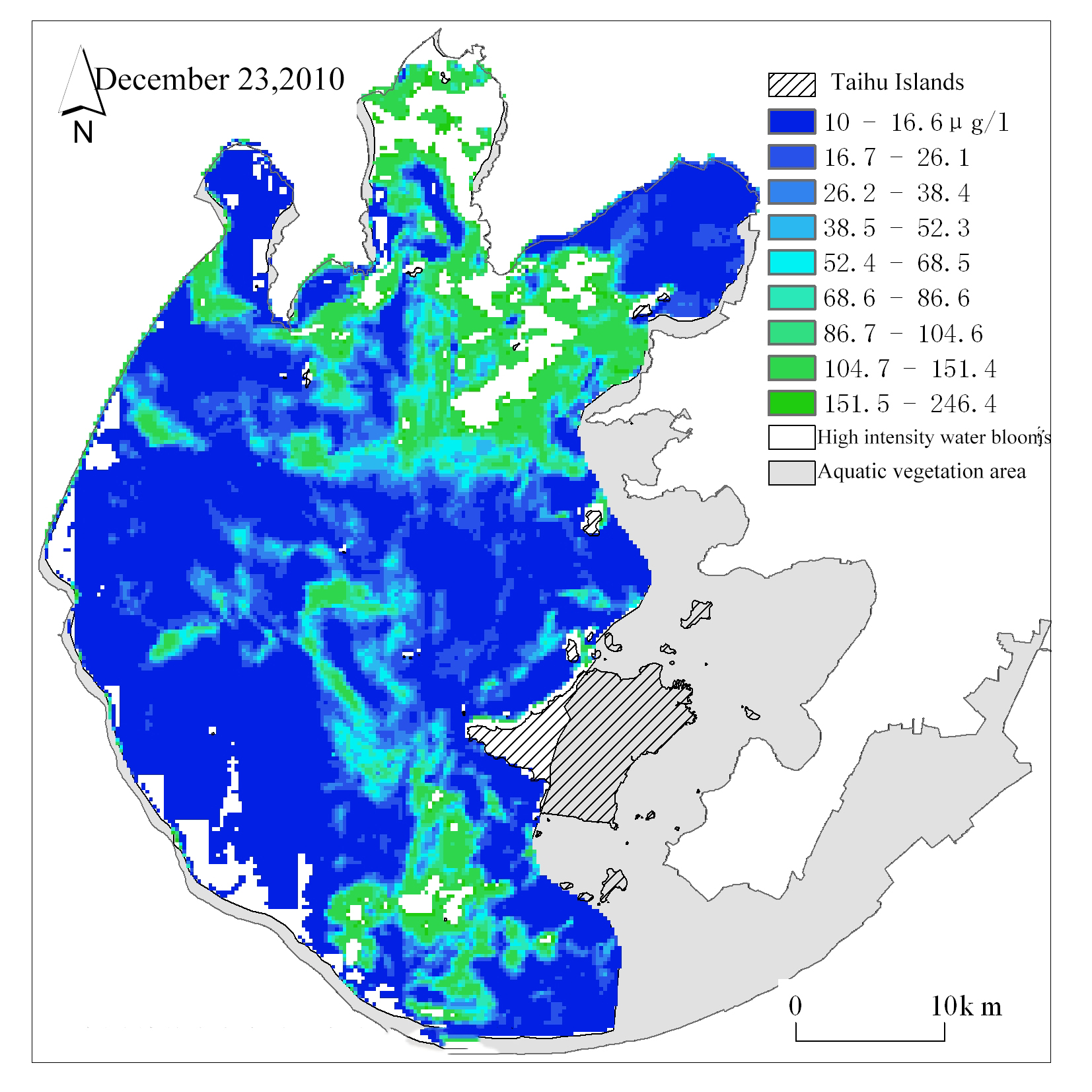

Supplement: Supplemental Information 4 — The data were obtained from the remote sensing image data of chlorophyll a concentration from the Lake-Watershed Science SubCenter, National Earth System Science Data Center, National Science & Technology Infrastructure of China, which had inconsistent data scales, data anomalies and different sampling intervals, and the chlorophyll a concentration unit was µg/L. [file peerj-cs-09-1292-s004.zip › 201012231323_taihu_chla.jpg]

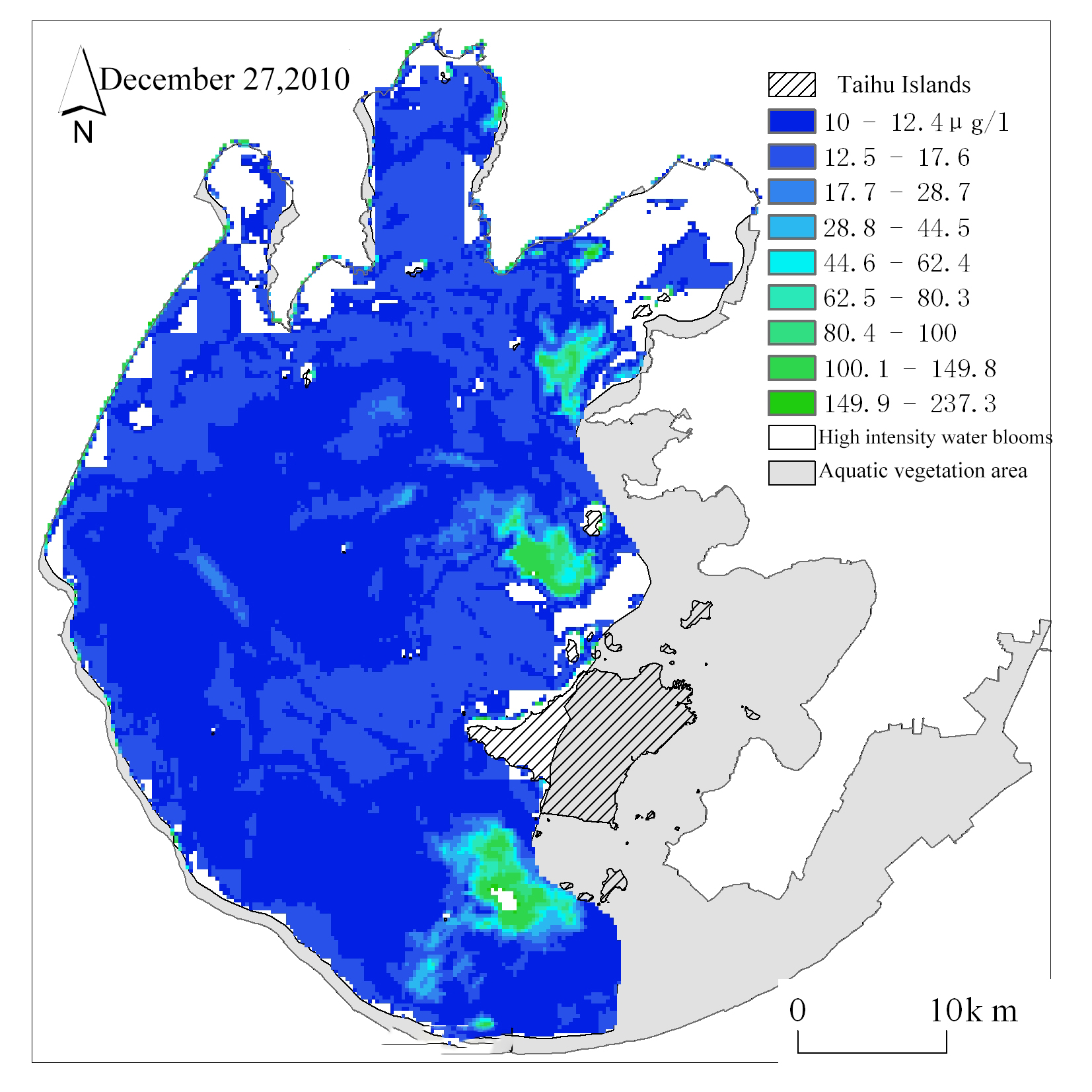

Supplement: Supplemental Information 4 — The data were obtained from the remote sensing image data of chlorophyll a concentration from the Lake-Watershed Science SubCenter, National Earth System Science Data Center, National Science & Technology Infrastructure of China, which had inconsistent data scales, data anomalies and different sampling intervals, and the chlorophyll a concentration unit was µg/L. [file peerj-cs-09-1292-s004.zip › 201012271259_taihu_chla.jpg]

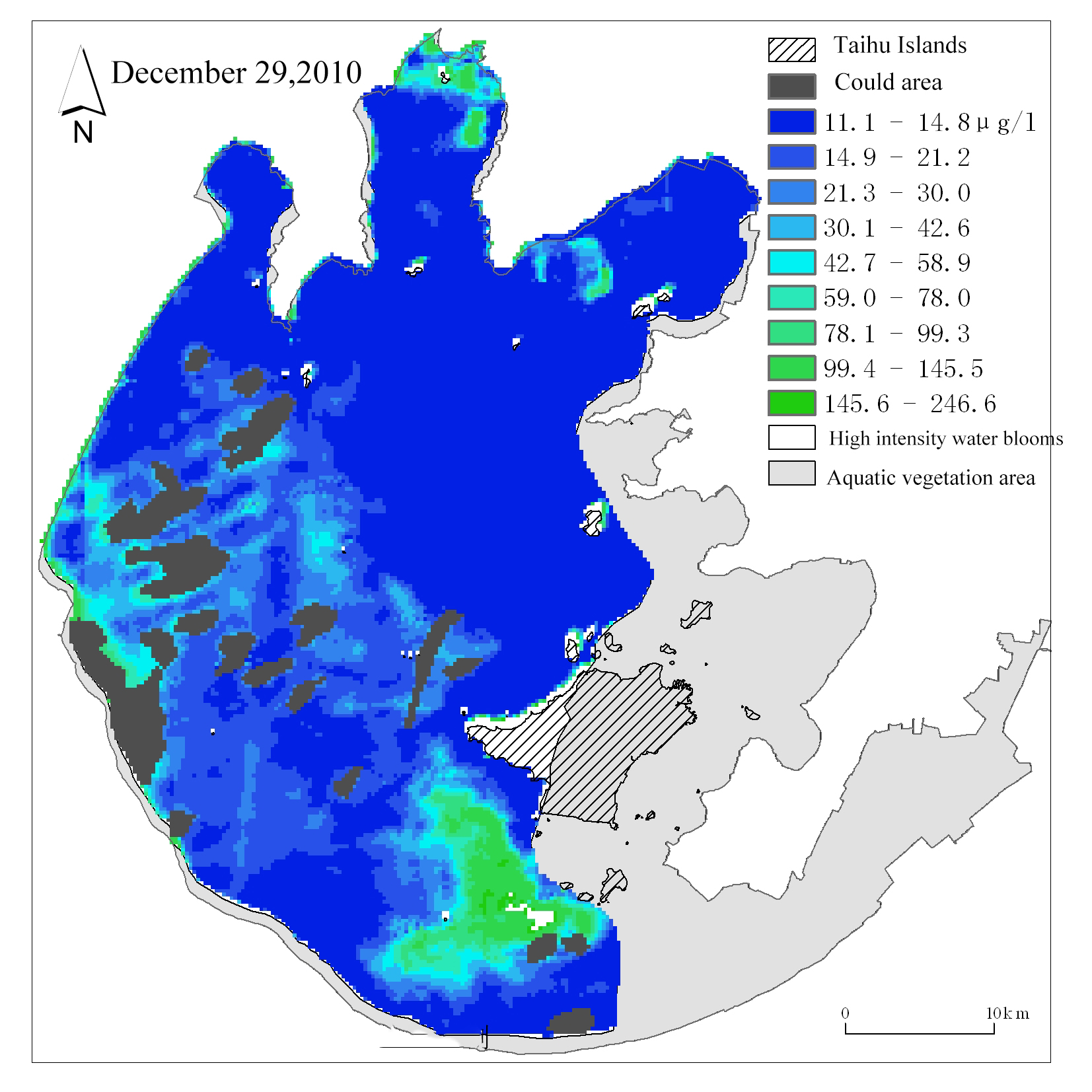

Supplement: Supplemental Information 4 — The data were obtained from the remote sensing image data of chlorophyll a concentration from the Lake-Watershed Science SubCenter, National Earth System Science Data Center, National Science & Technology Infrastructure of China, which had inconsistent data scales, data anomalies and different sampling intervals, and the chlorophyll a concentration unit was µg/L. [file peerj-cs-09-1292-s004.zip › 201012290448_taihu_chla.jpg]
