# Supplementary figures and images for: Remote sensing image analysis and prediction based on improved Pix2Pix model for water environment protection of smart cities (part 2 of 6)
Source: PeerJ Comput Sci. 2023 Apr 26;9:e1292. doi: 10.7717/peerj-cs.1292 (PMC10280440; doi:10.7717/peerj-cs.1292)

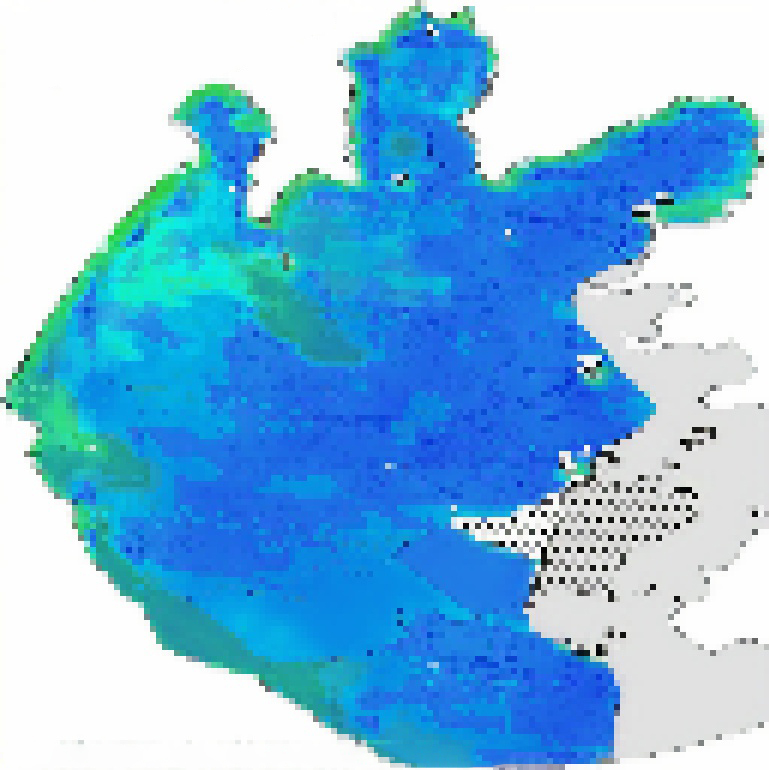

Supplement: Supplemental Information 5 [file peerj-cs-09-1292-s005.zip › batch1/fake/0.jpg]

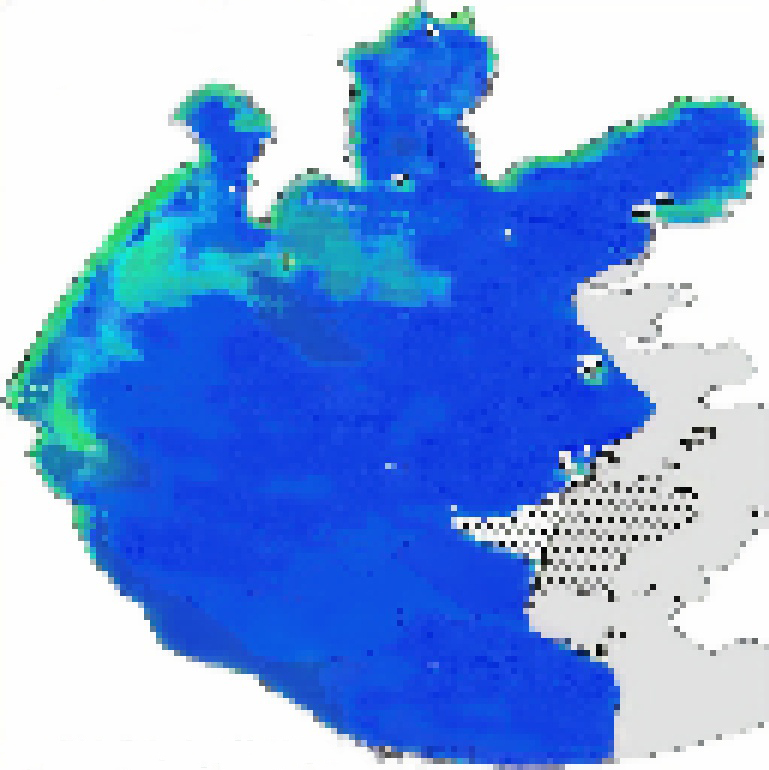

Supplement: Supplemental Information 5 [file peerj-cs-09-1292-s005.zip › batch1/fake/1.jpg]

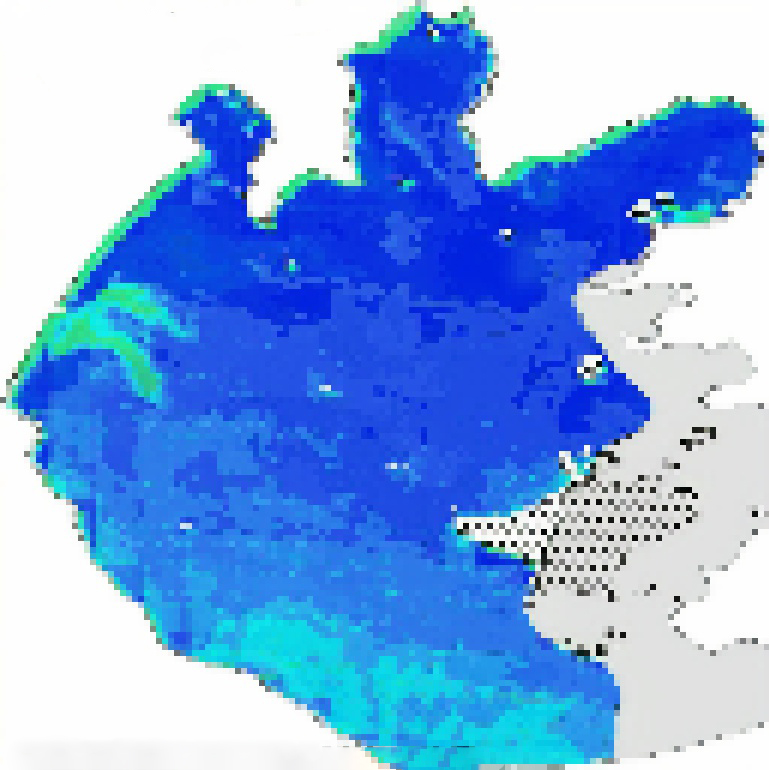

Supplement: Supplemental Information 5 [file peerj-cs-09-1292-s005.zip › batch1/fake/10.jpg]

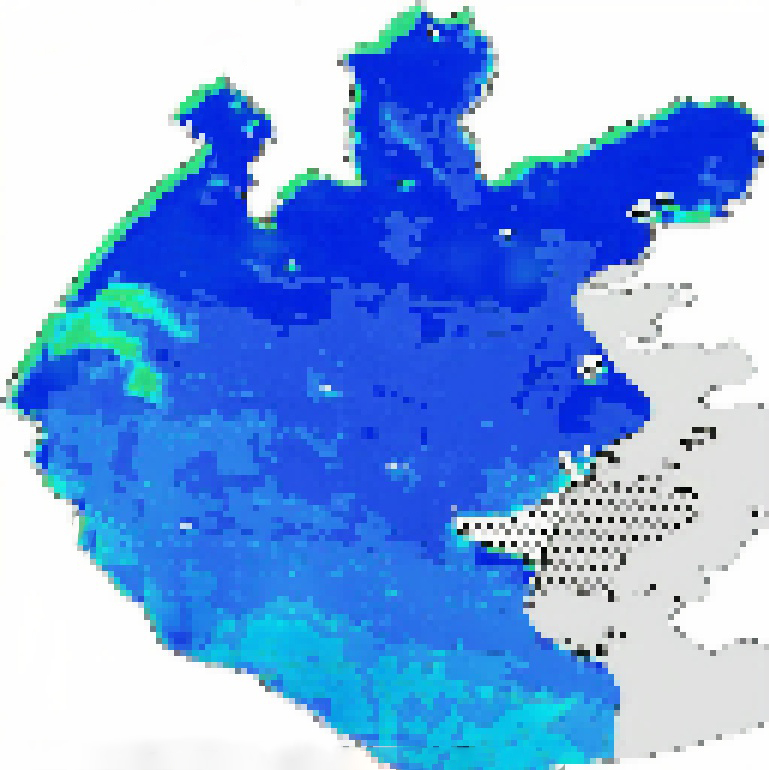

Supplement: Supplemental Information 5 [file peerj-cs-09-1292-s005.zip › batch1/fake/11.jpg]

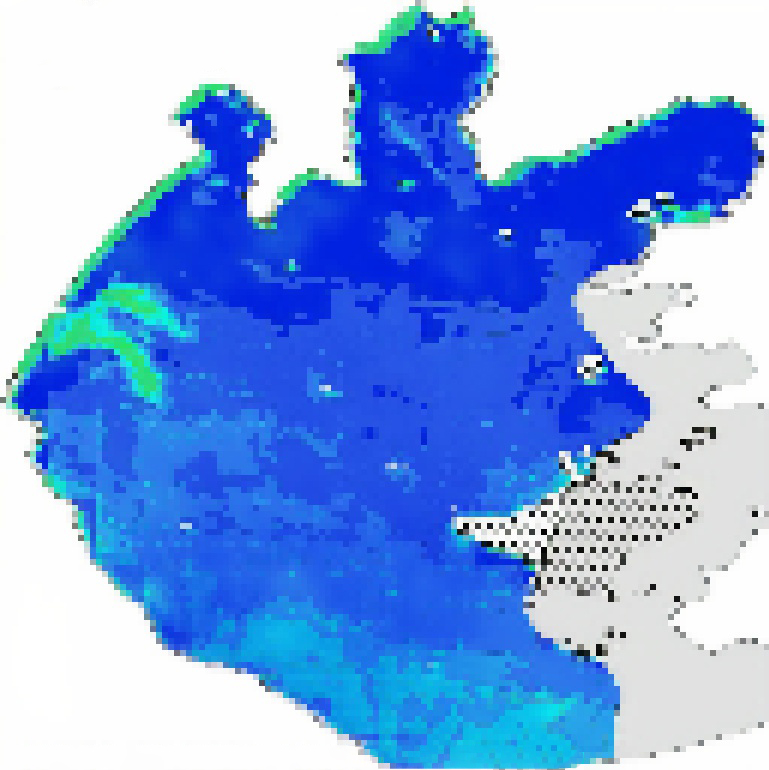

Supplement: Supplemental Information 5 [file peerj-cs-09-1292-s005.zip › batch1/fake/12.jpg]

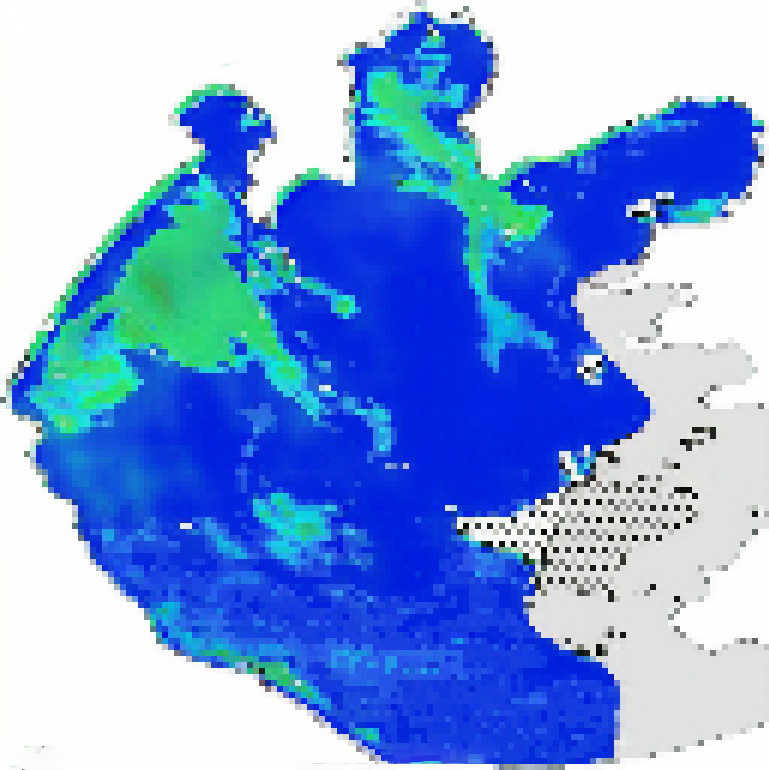

Supplement: Supplemental Information 5 [file peerj-cs-09-1292-s005.zip › batch1/fake/13.jpg]

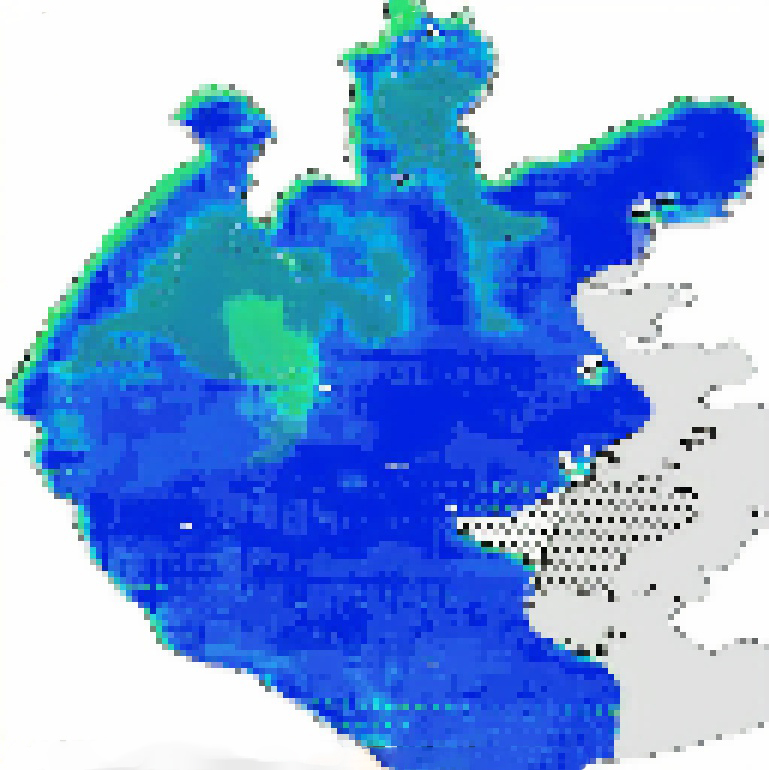

Supplement: Supplemental Information 5 [file peerj-cs-09-1292-s005.zip › batch1/fake/14.jpg]

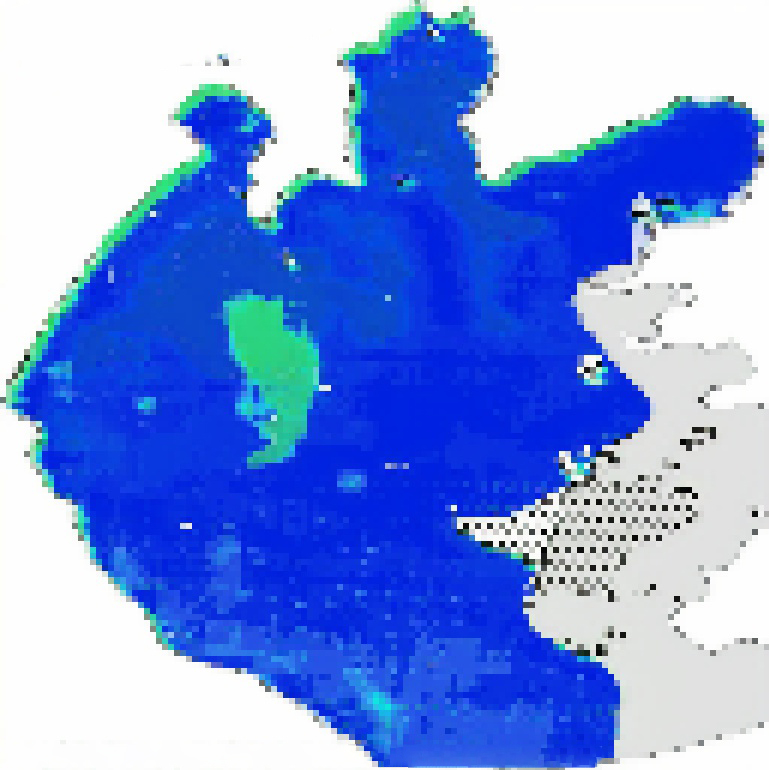

Supplement: Supplemental Information 5 [file peerj-cs-09-1292-s005.zip › batch1/fake/15.jpg]

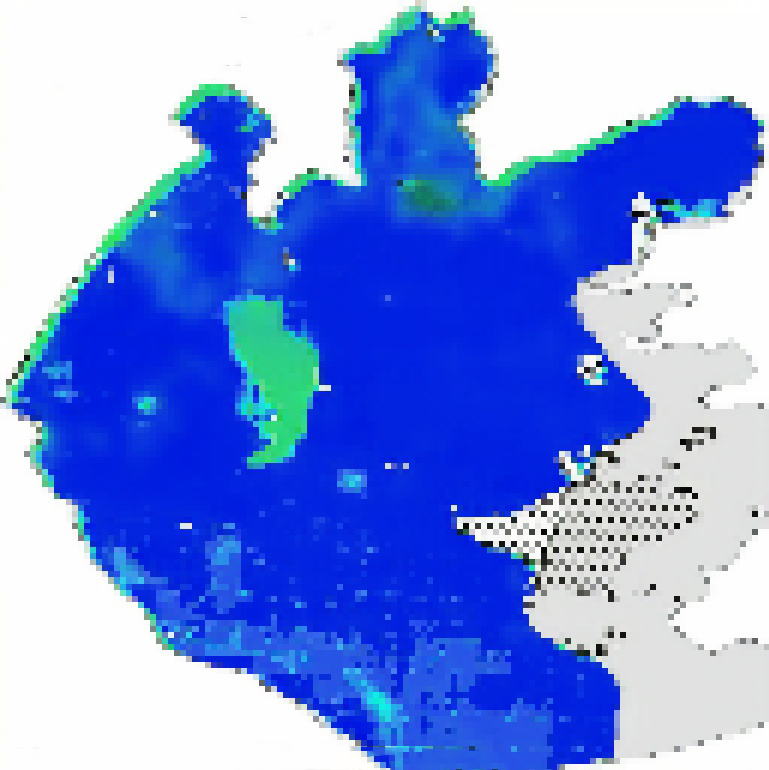

Supplement: Supplemental Information 5 [file peerj-cs-09-1292-s005.zip › batch1/fake/16.jpg]

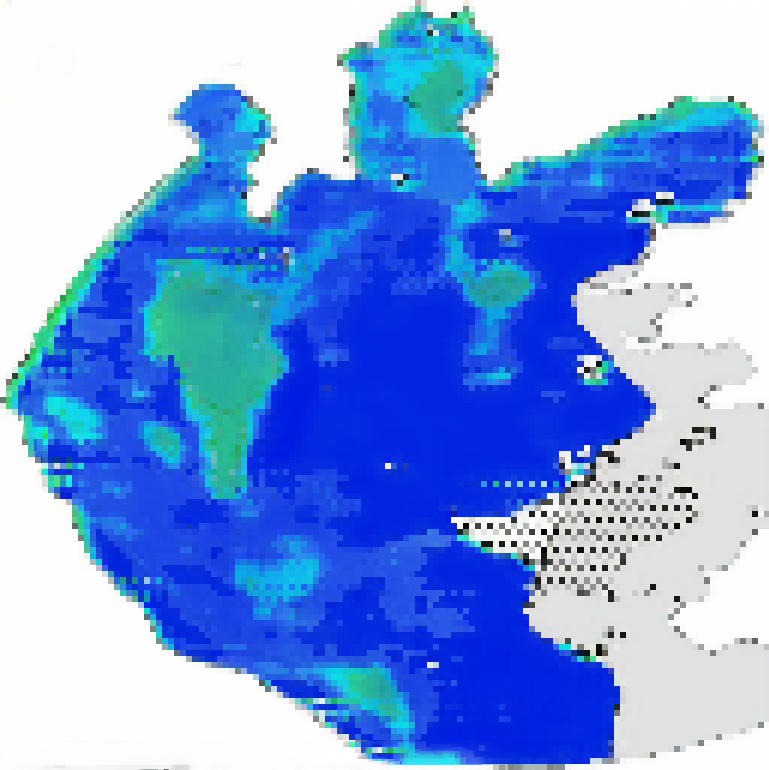

Supplement: Supplemental Information 5 [file peerj-cs-09-1292-s005.zip › batch1/fake/17.jpg]

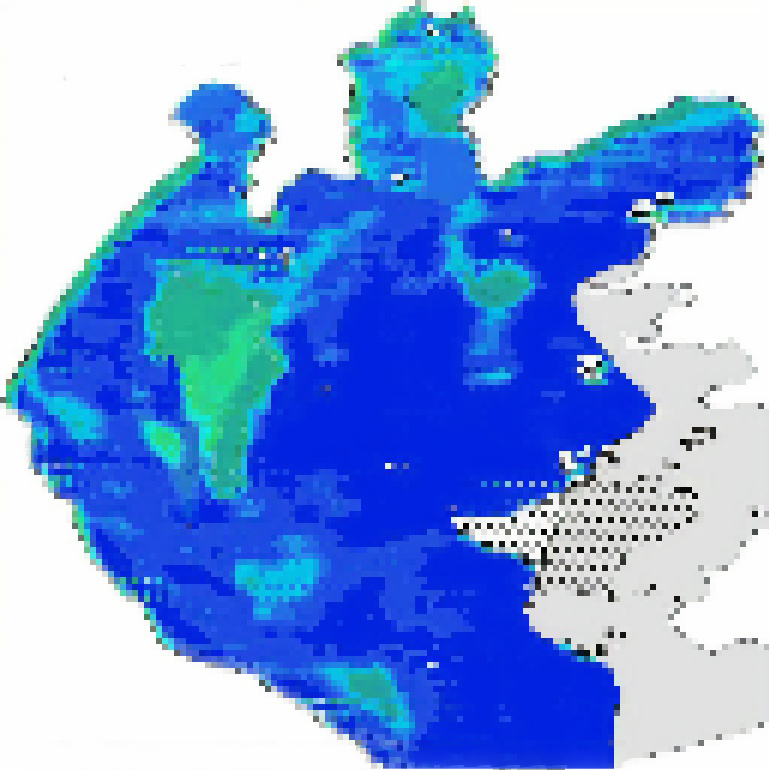

Supplement: Supplemental Information 5 [file peerj-cs-09-1292-s005.zip › batch1/fake/18.jpg]

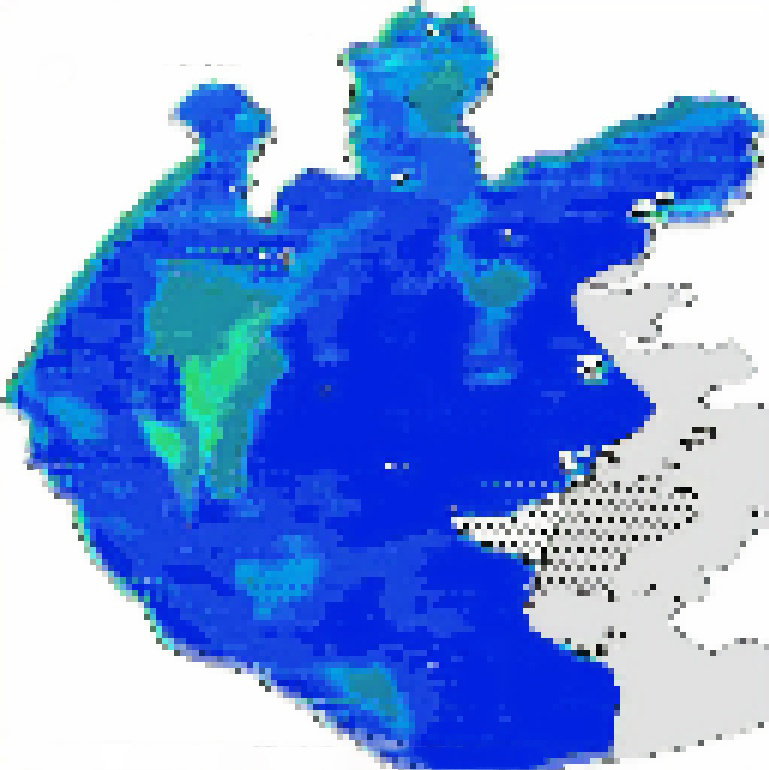

Supplement: Supplemental Information 5 [file peerj-cs-09-1292-s005.zip › batch1/fake/19.jpg]

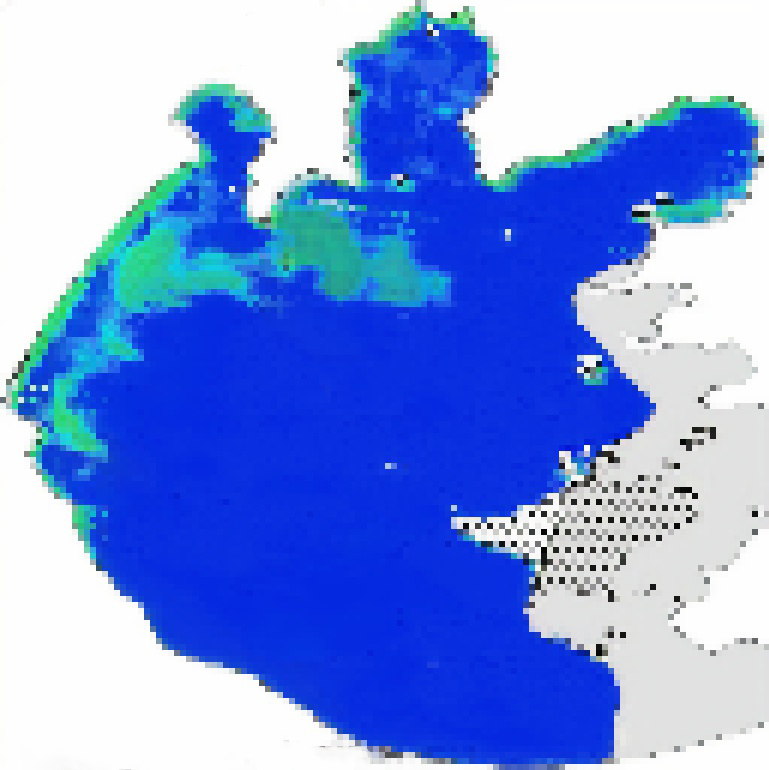

Supplement: Supplemental Information 5 [file peerj-cs-09-1292-s005.zip › batch1/fake/2.jpg]

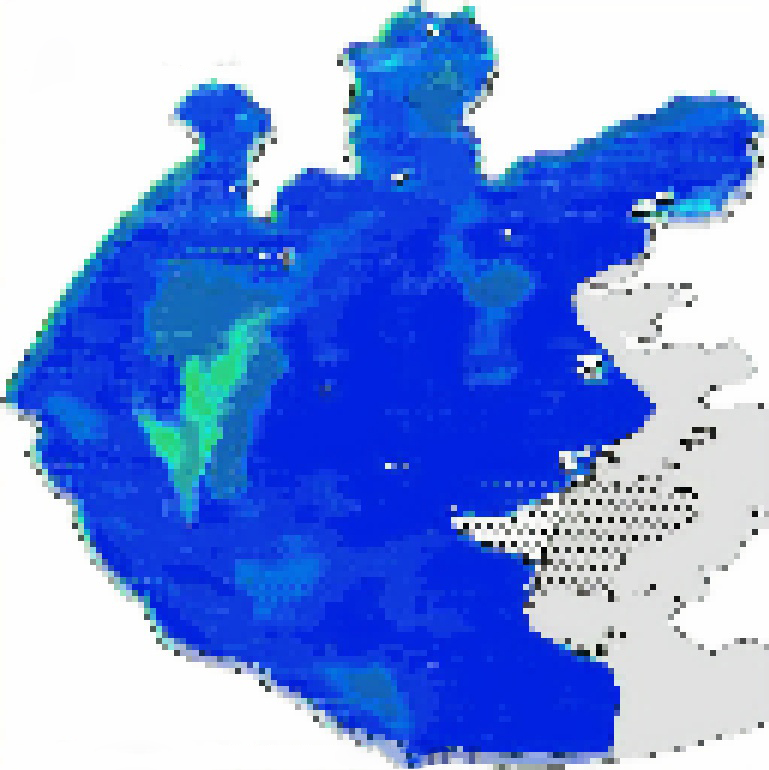

Supplement: Supplemental Information 5 [file peerj-cs-09-1292-s005.zip › batch1/fake/20.jpg]

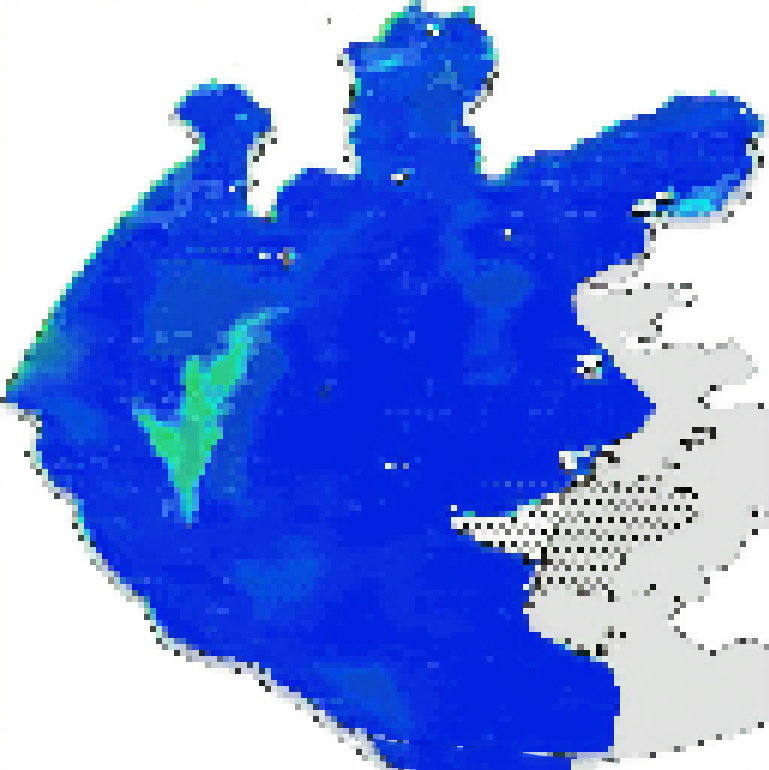

Supplement: Supplemental Information 5 [file peerj-cs-09-1292-s005.zip › batch1/fake/21.jpg]

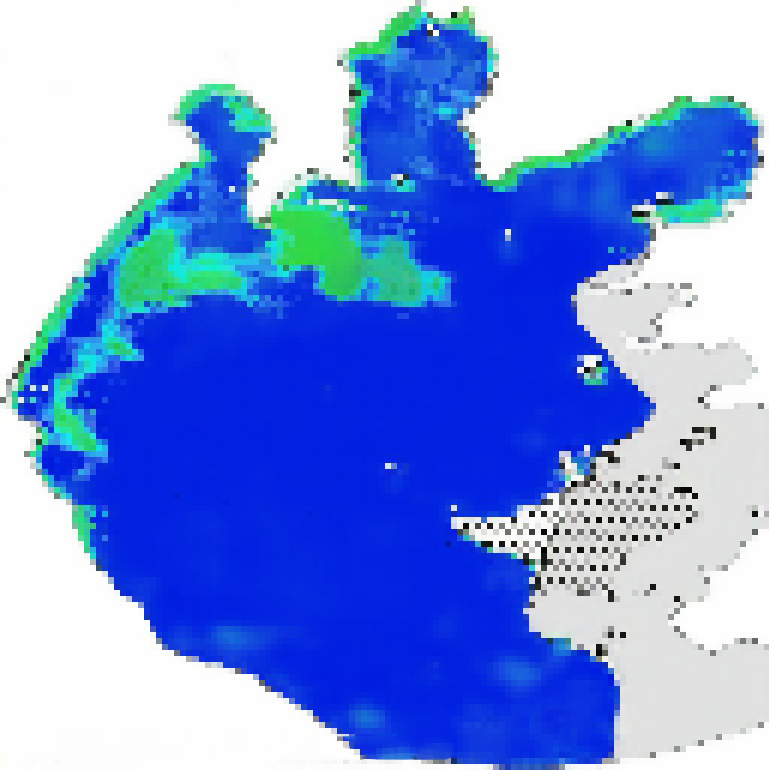

Supplement: Supplemental Information 5 [file peerj-cs-09-1292-s005.zip › batch1/fake/3.jpg]

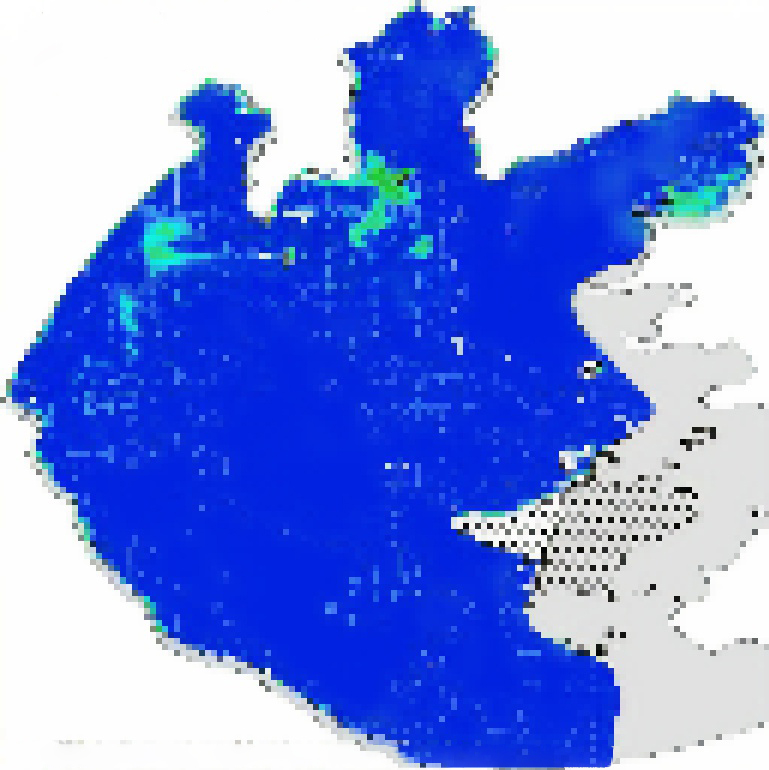

Supplement: Supplemental Information 5 [file peerj-cs-09-1292-s005.zip › batch1/fake/4.jpg]

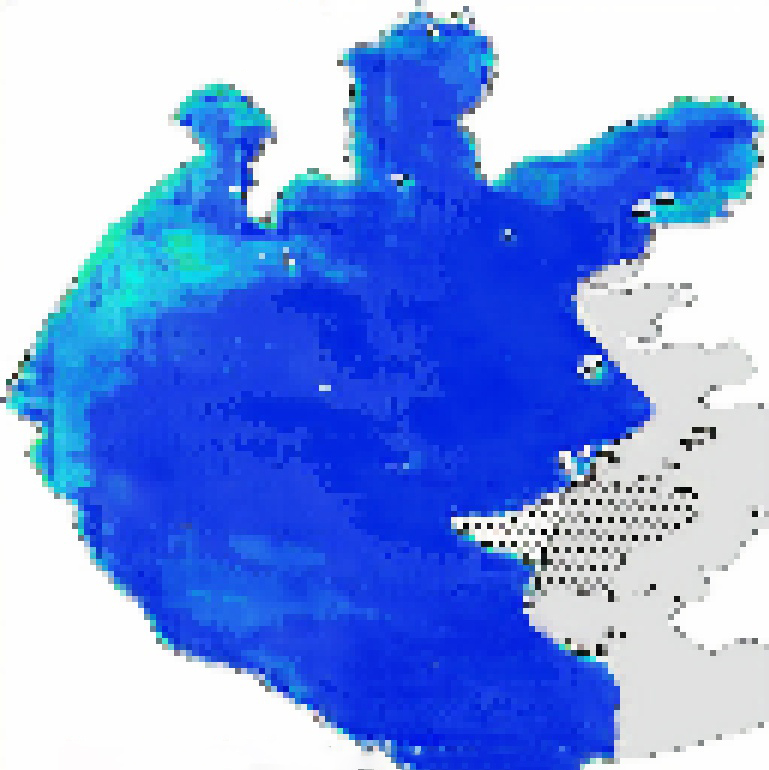

Supplement: Supplemental Information 5 [file peerj-cs-09-1292-s005.zip › batch1/fake/5.jpg]

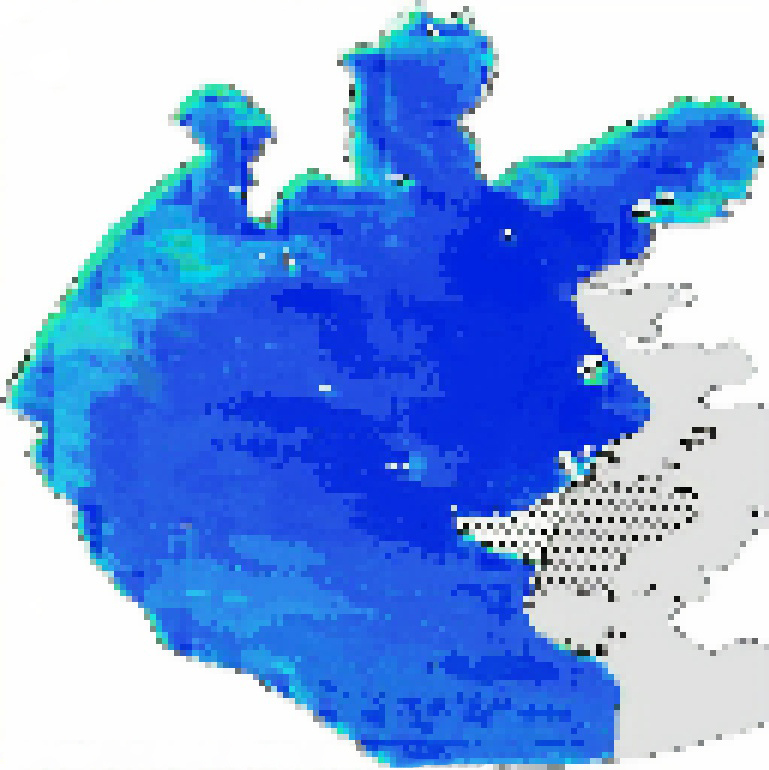

Supplement: Supplemental Information 5 [file peerj-cs-09-1292-s005.zip › batch1/fake/6.jpg]

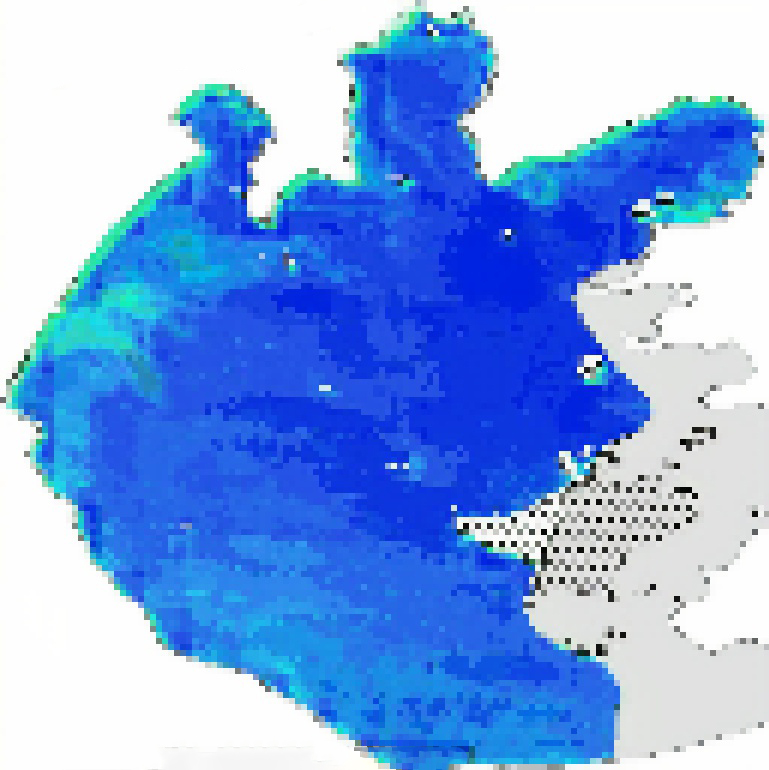

Supplement: Supplemental Information 5 [file peerj-cs-09-1292-s005.zip › batch1/fake/7.jpg]

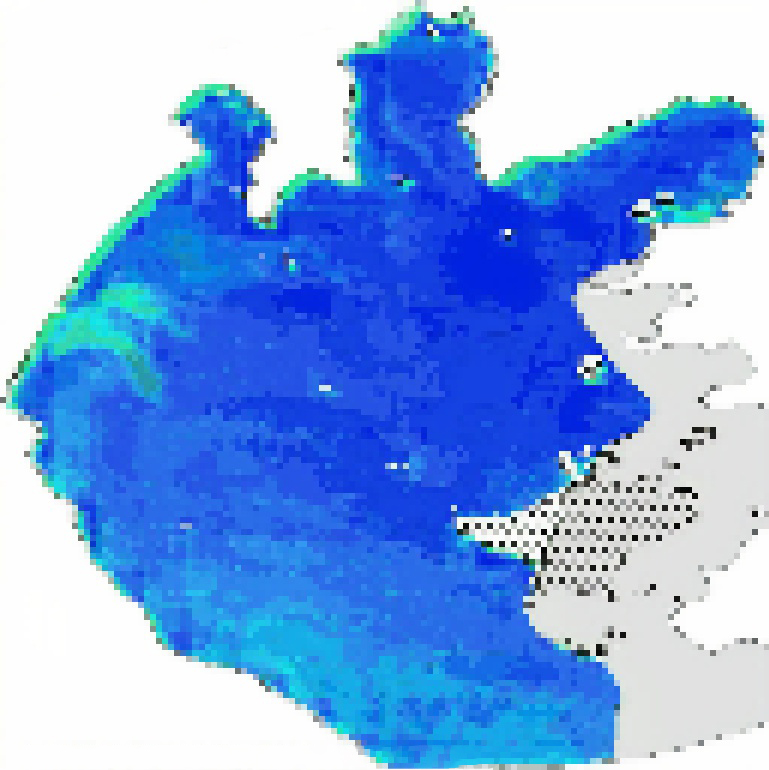

Supplement: Supplemental Information 5 [file peerj-cs-09-1292-s005.zip › batch1/fake/8.jpg]

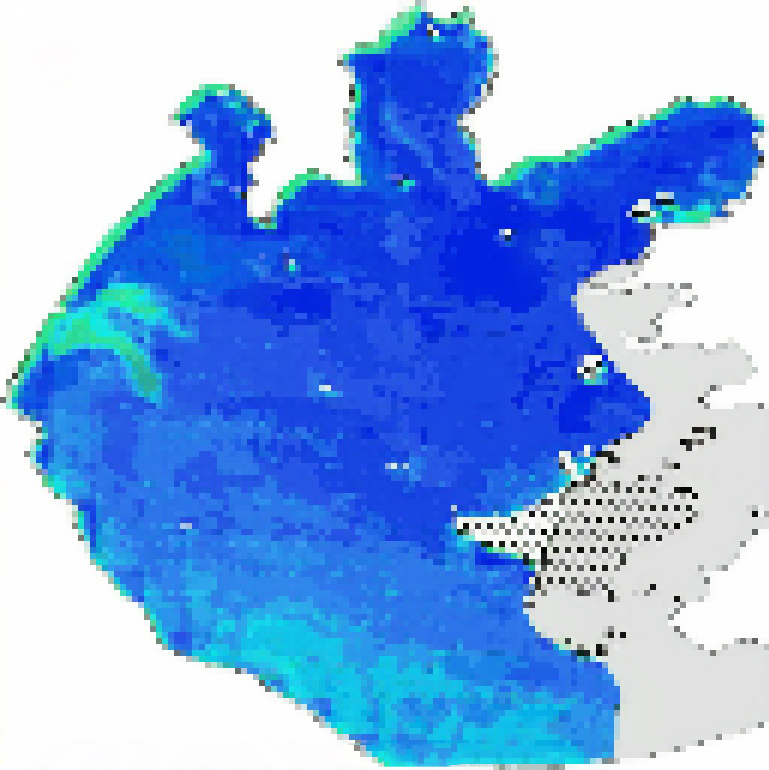

Supplement: Supplemental Information 5 [file peerj-cs-09-1292-s005.zip › batch1/fake/9.jpg]

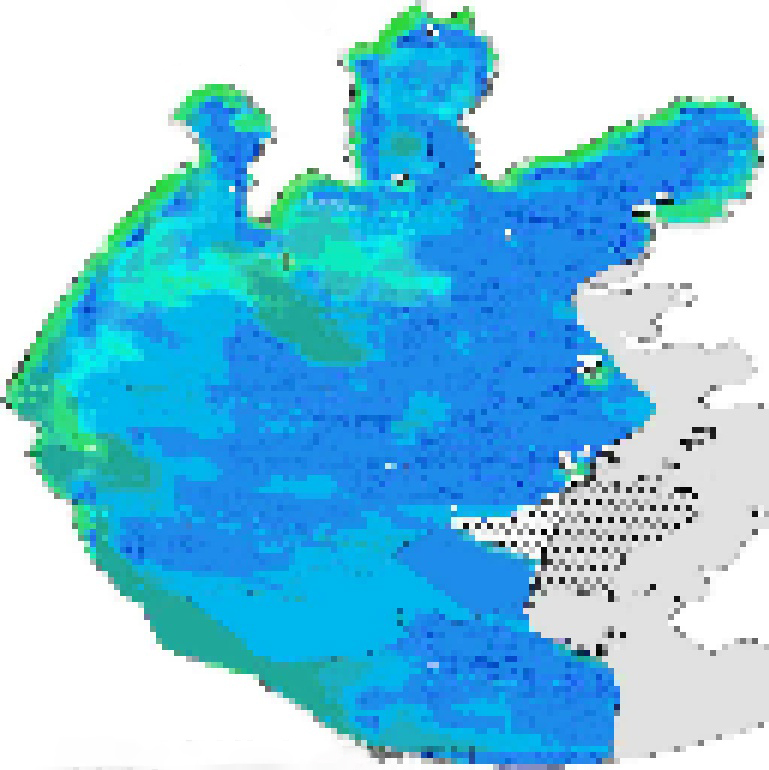

Supplement: Supplemental Information 5 [file peerj-cs-09-1292-s005.zip › batch1/real/0.jpg]

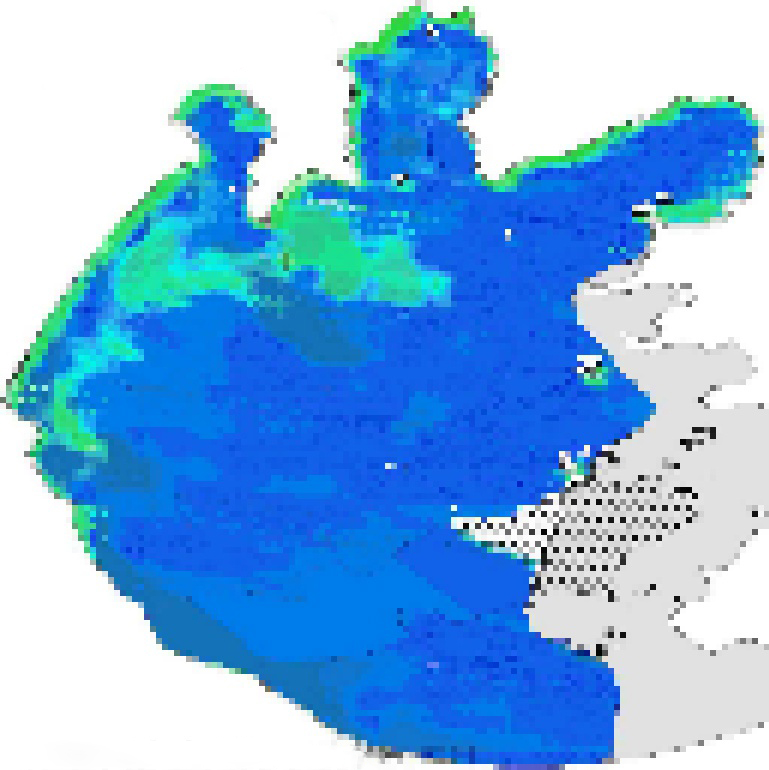

Supplement: Supplemental Information 5 [file peerj-cs-09-1292-s005.zip › batch1/real/1.jpg]

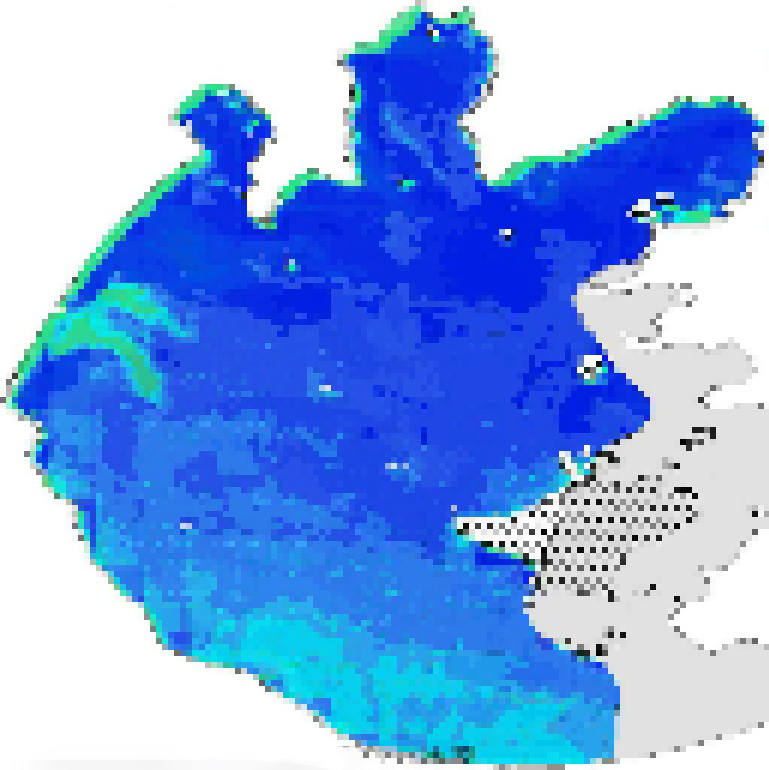

Supplement: Supplemental Information 5 [file peerj-cs-09-1292-s005.zip › batch1/real/10.jpg]

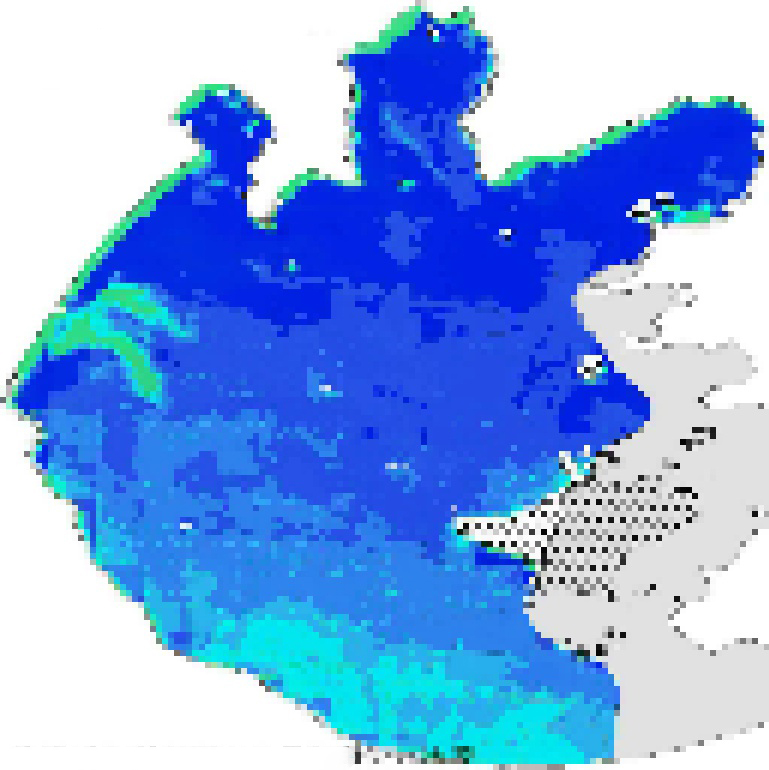

Supplement: Supplemental Information 5 [file peerj-cs-09-1292-s005.zip › batch1/real/11.jpg]

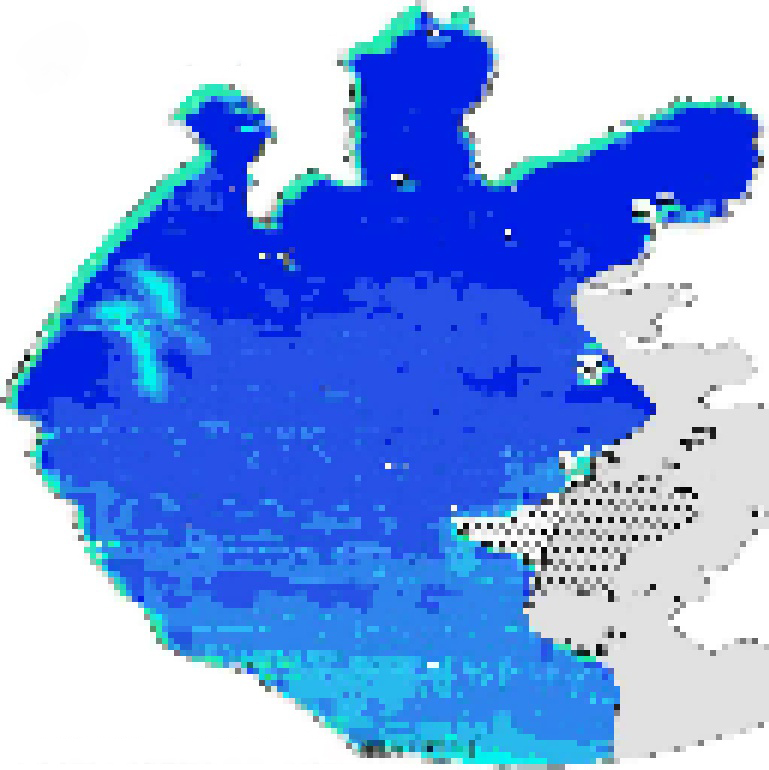

Supplement: Supplemental Information 5 [file peerj-cs-09-1292-s005.zip › batch1/real/12.jpg]

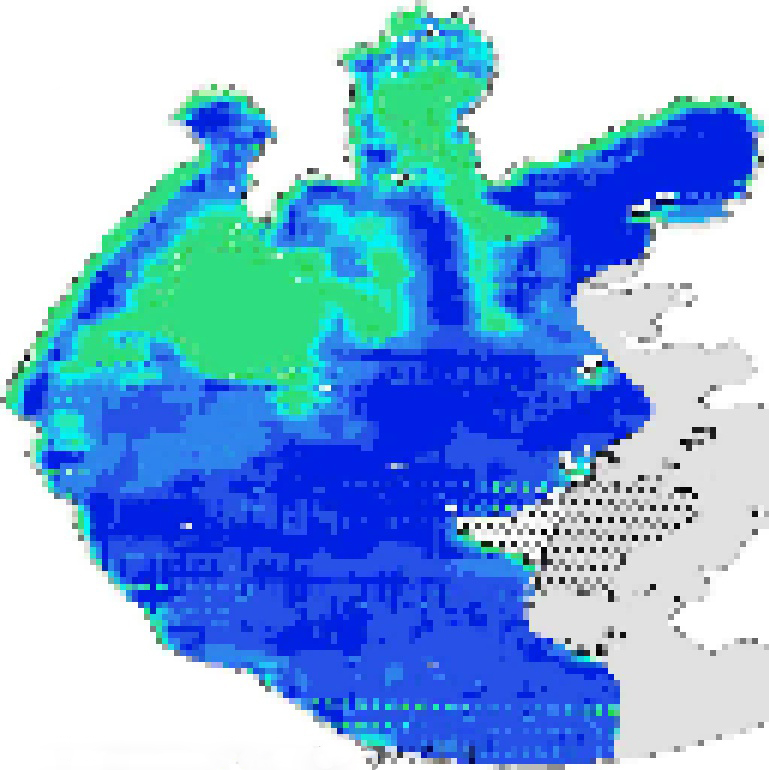

Supplement: Supplemental Information 5 [file peerj-cs-09-1292-s005.zip › batch1/real/13.jpg]

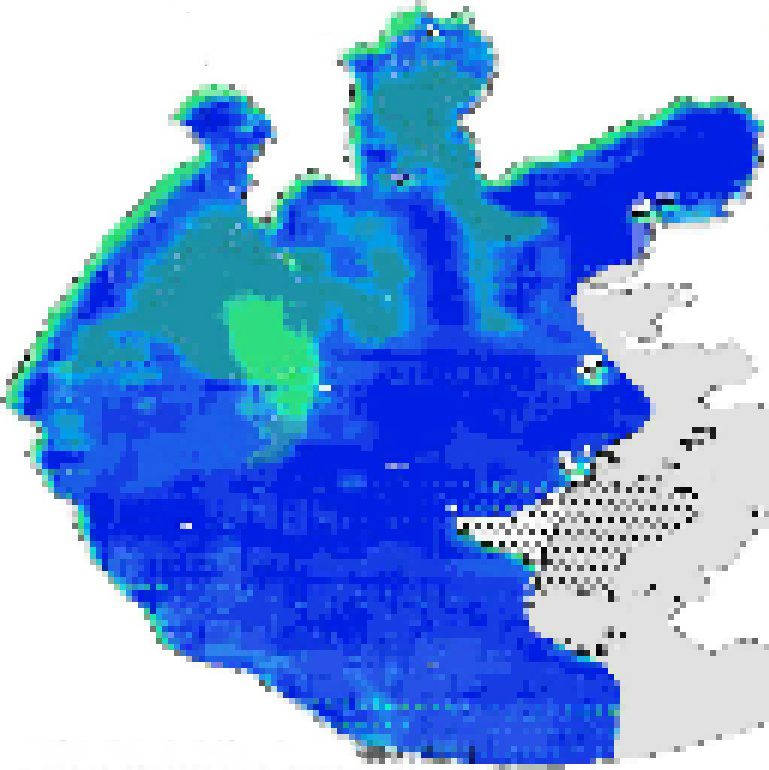

Supplement: Supplemental Information 5 [file peerj-cs-09-1292-s005.zip › batch1/real/14.jpg]

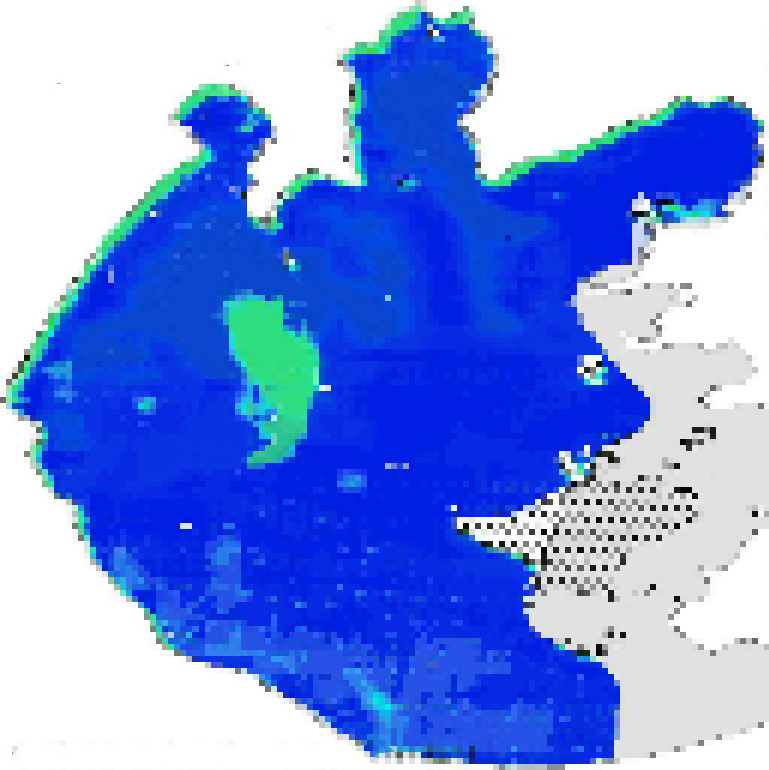

Supplement: Supplemental Information 5 [file peerj-cs-09-1292-s005.zip › batch1/real/15.jpg]

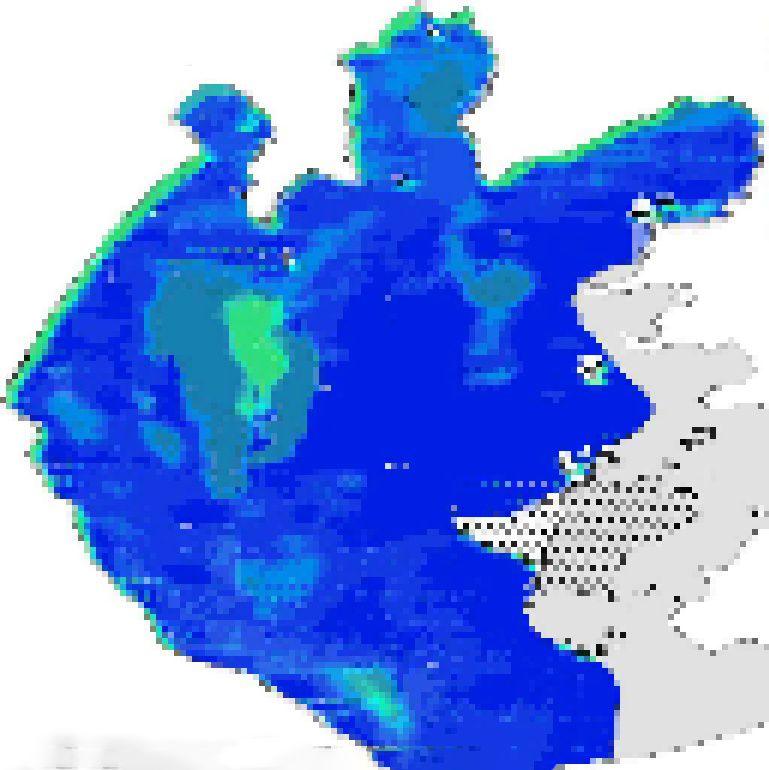

Supplement: Supplemental Information 5 [file peerj-cs-09-1292-s005.zip › batch1/real/16.jpg]

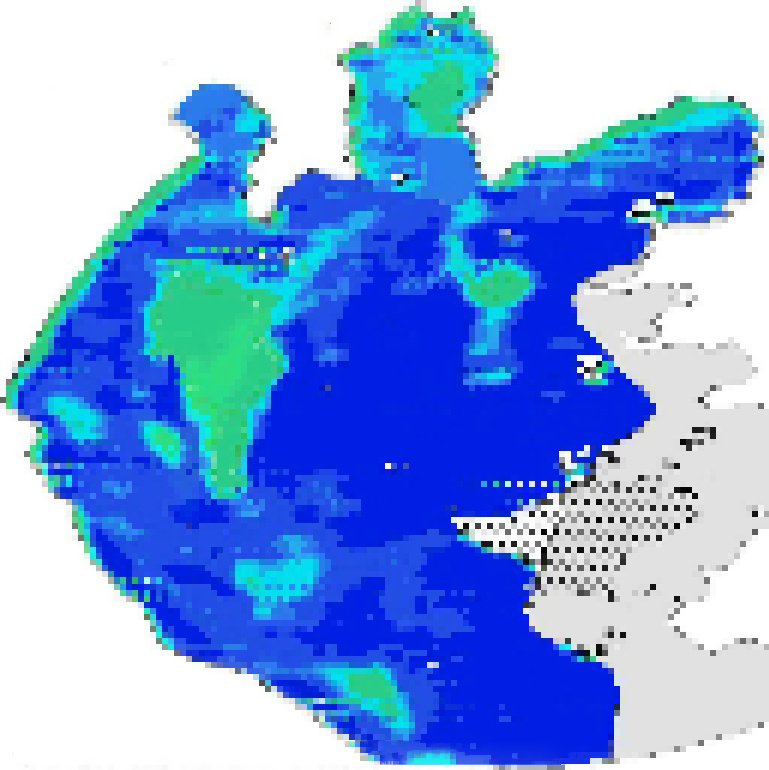

Supplement: Supplemental Information 5 [file peerj-cs-09-1292-s005.zip › batch1/real/17.jpg]

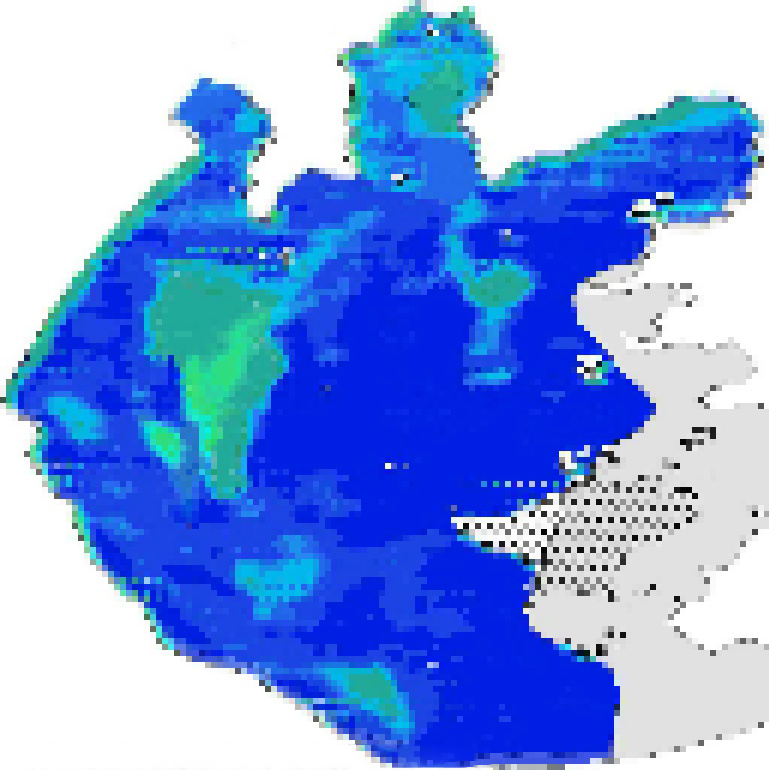

Supplement: Supplemental Information 5 [file peerj-cs-09-1292-s005.zip › batch1/real/18.jpg]

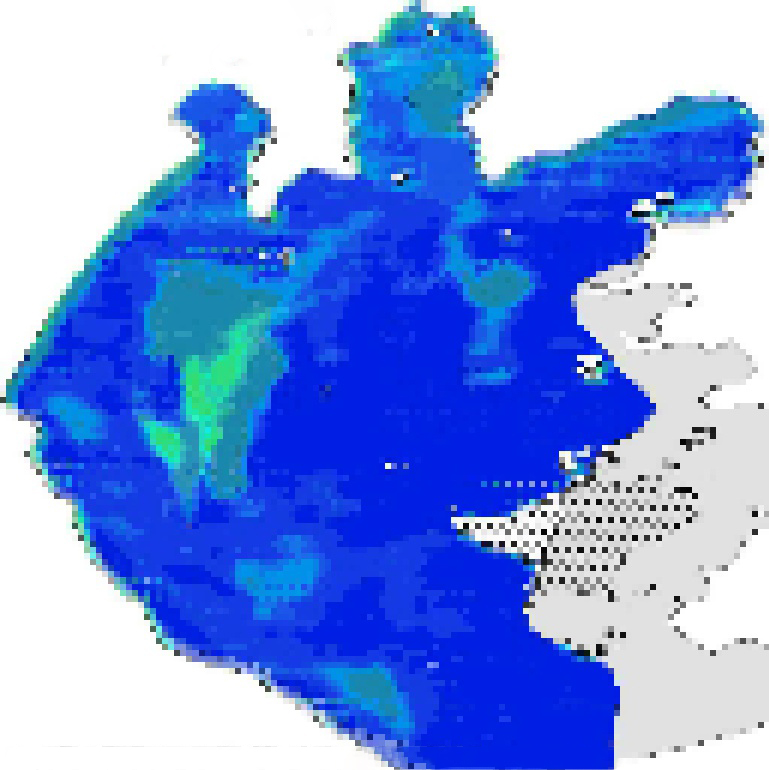

Supplement: Supplemental Information 5 [file peerj-cs-09-1292-s005.zip › batch1/real/19.jpg]

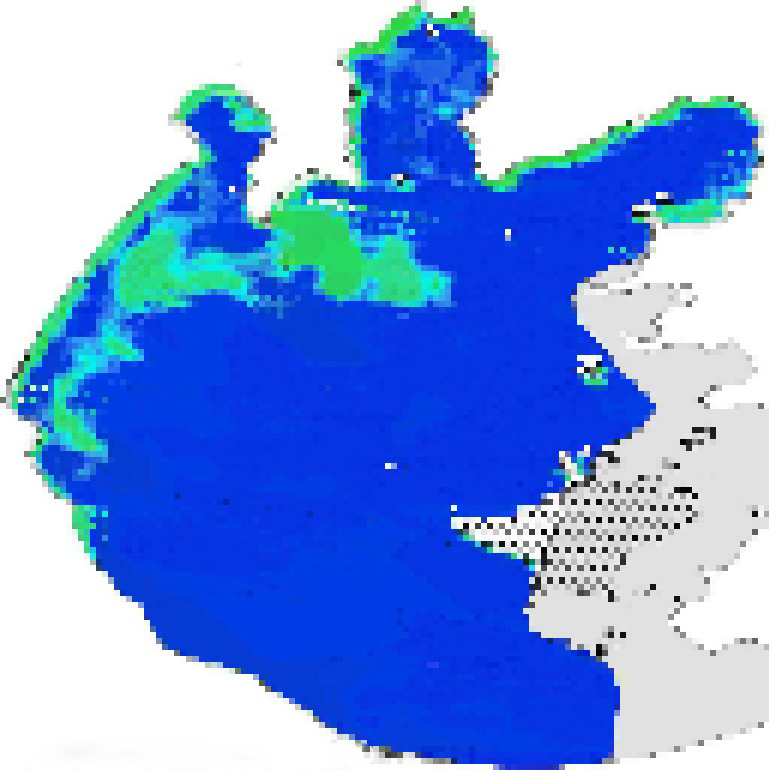

Supplement: Supplemental Information 5 [file peerj-cs-09-1292-s005.zip › batch1/real/2.jpg]

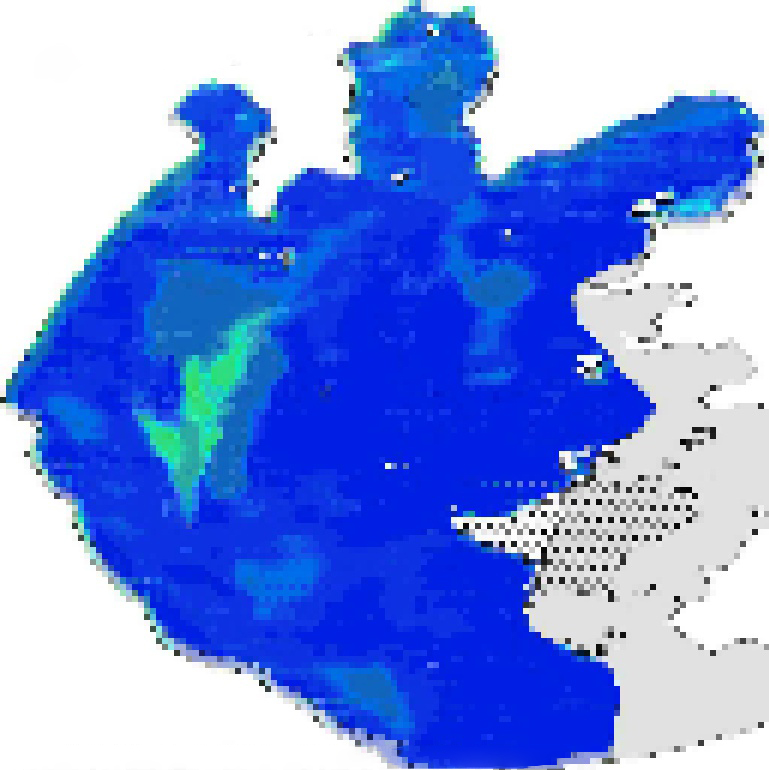

Supplement: Supplemental Information 5 [file peerj-cs-09-1292-s005.zip › batch1/real/20.jpg]

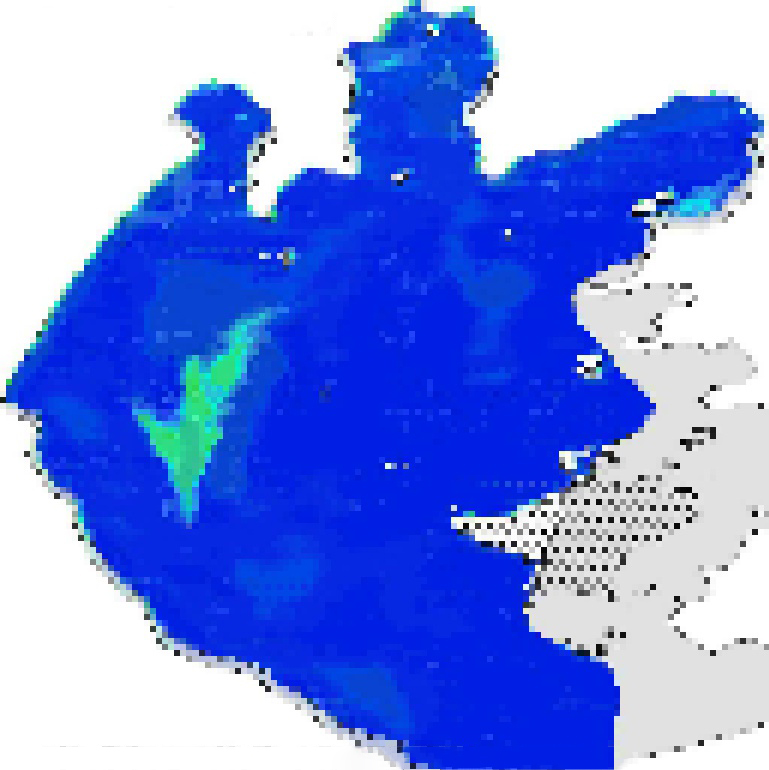

Supplement: Supplemental Information 5 [file peerj-cs-09-1292-s005.zip › batch1/real/21.jpg]

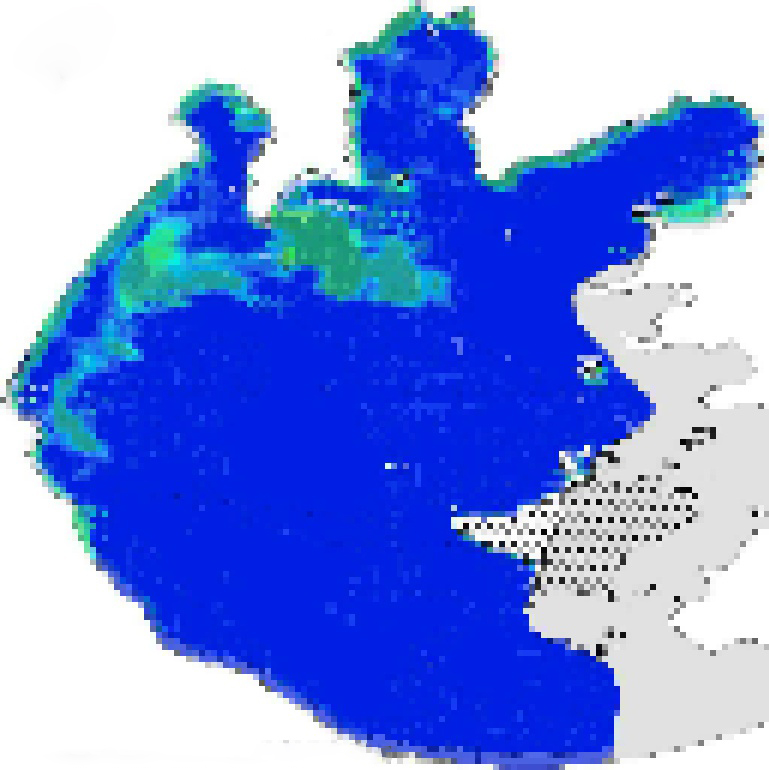

Supplement: Supplemental Information 5 [file peerj-cs-09-1292-s005.zip › batch1/real/3.jpg]

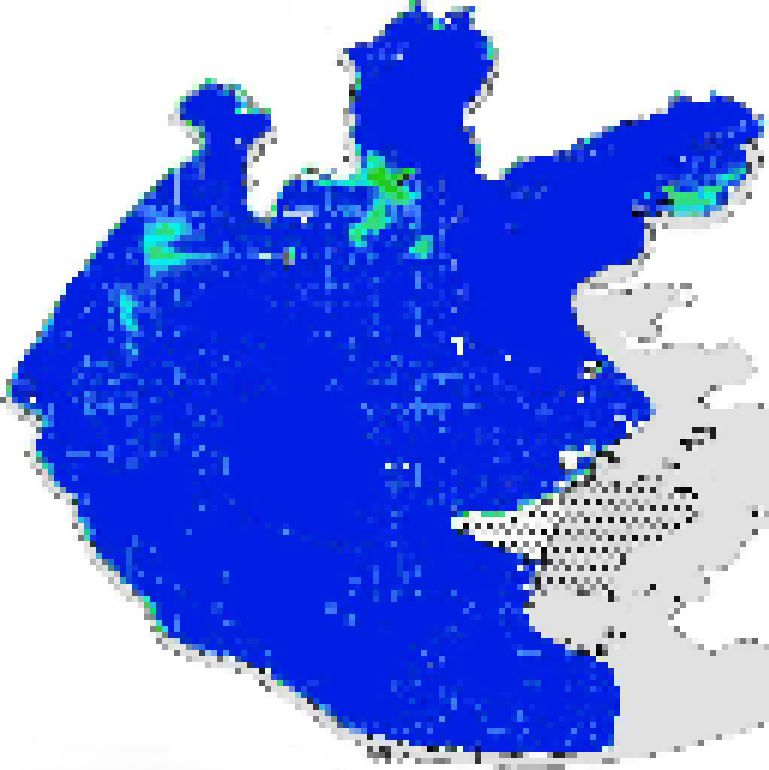

Supplement: Supplemental Information 5 [file peerj-cs-09-1292-s005.zip › batch1/real/4.jpg]

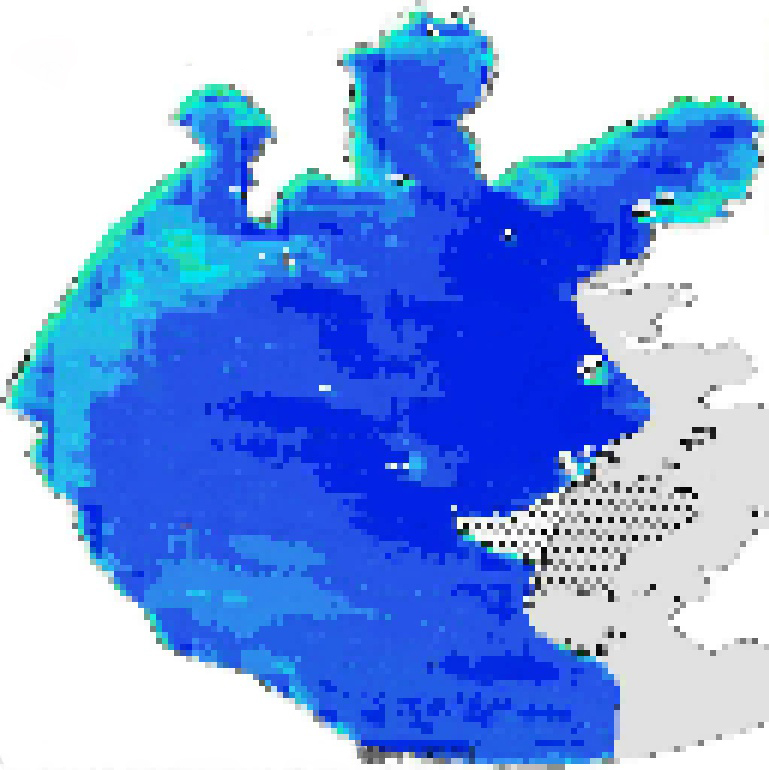

Supplement: Supplemental Information 5 [file peerj-cs-09-1292-s005.zip › batch1/real/5.jpg]

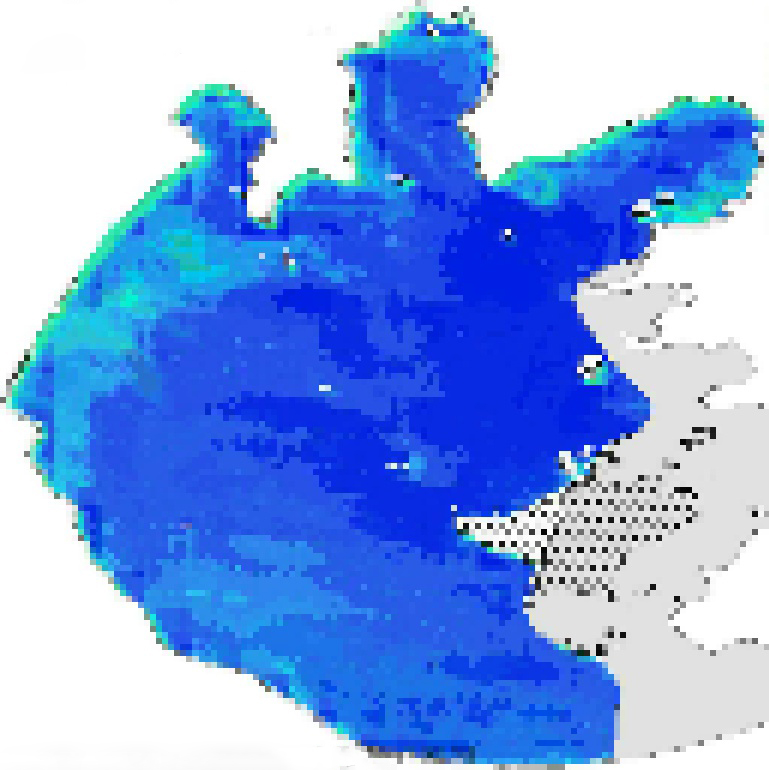

Supplement: Supplemental Information 5 [file peerj-cs-09-1292-s005.zip › batch1/real/6.jpg]

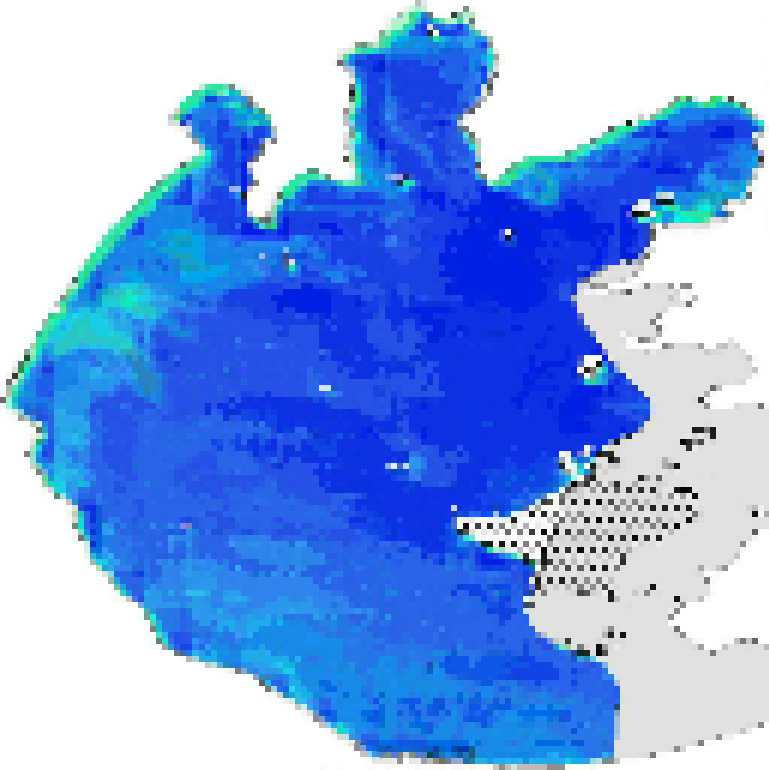

Supplement: Supplemental Information 5 [file peerj-cs-09-1292-s005.zip › batch1/real/7.jpg]

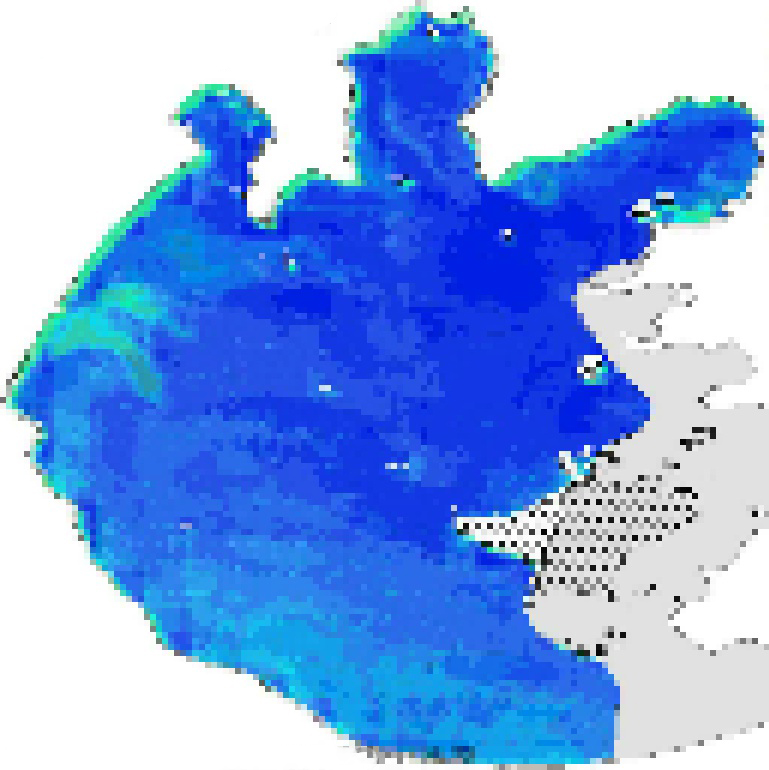

Supplement: Supplemental Information 5 [file peerj-cs-09-1292-s005.zip › batch1/real/8.jpg]

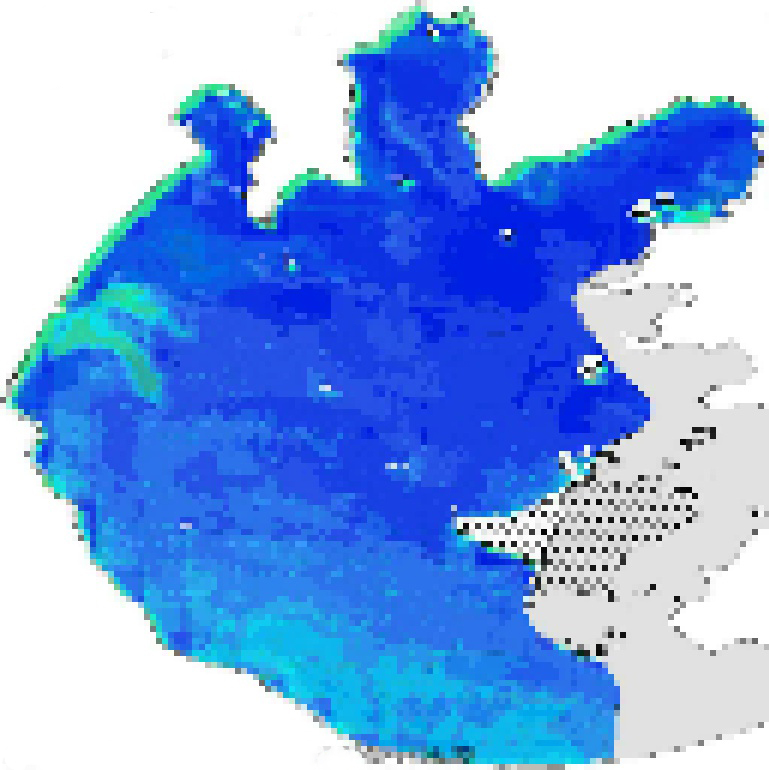

Supplement: Supplemental Information 5 [file peerj-cs-09-1292-s005.zip › batch1/real/9.jpg]

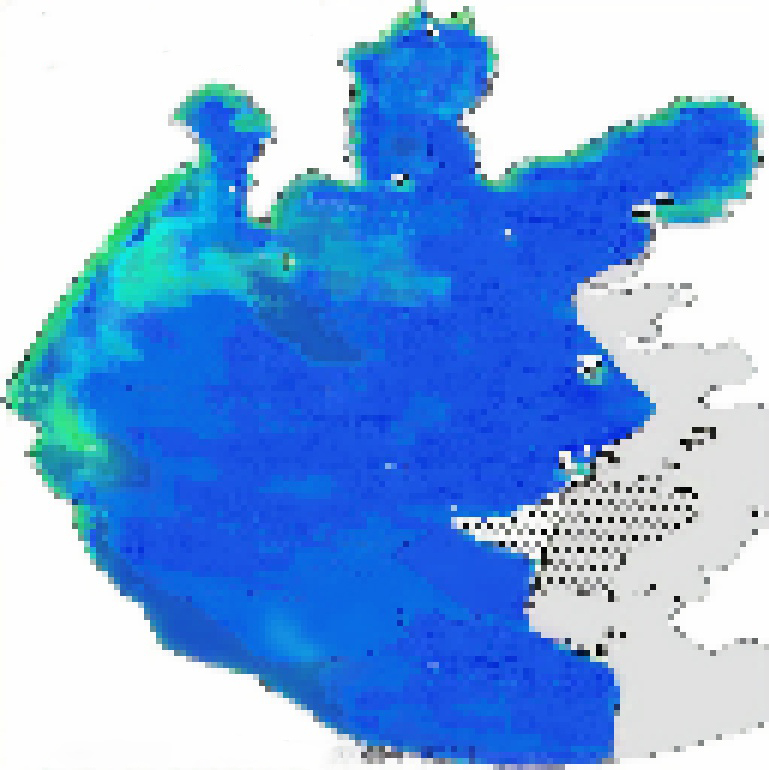

Supplement: Supplemental Information 5 [file peerj-cs-09-1292-s005.zip › batch2/fake/0.jpg]

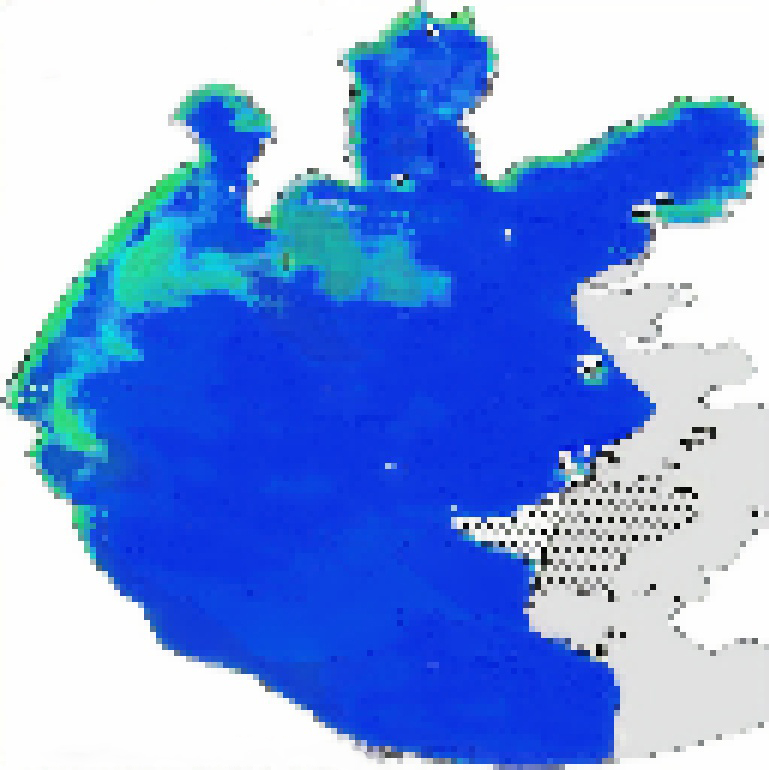

Supplement: Supplemental Information 5 [file peerj-cs-09-1292-s005.zip › batch2/fake/1.jpg]

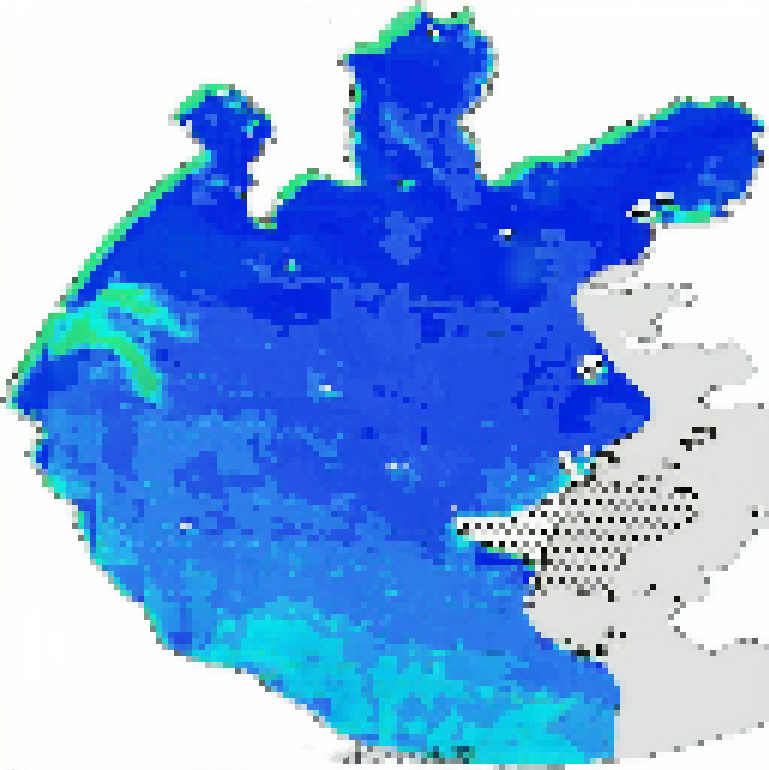

Supplement: Supplemental Information 5 [file peerj-cs-09-1292-s005.zip › batch2/fake/10.jpg]

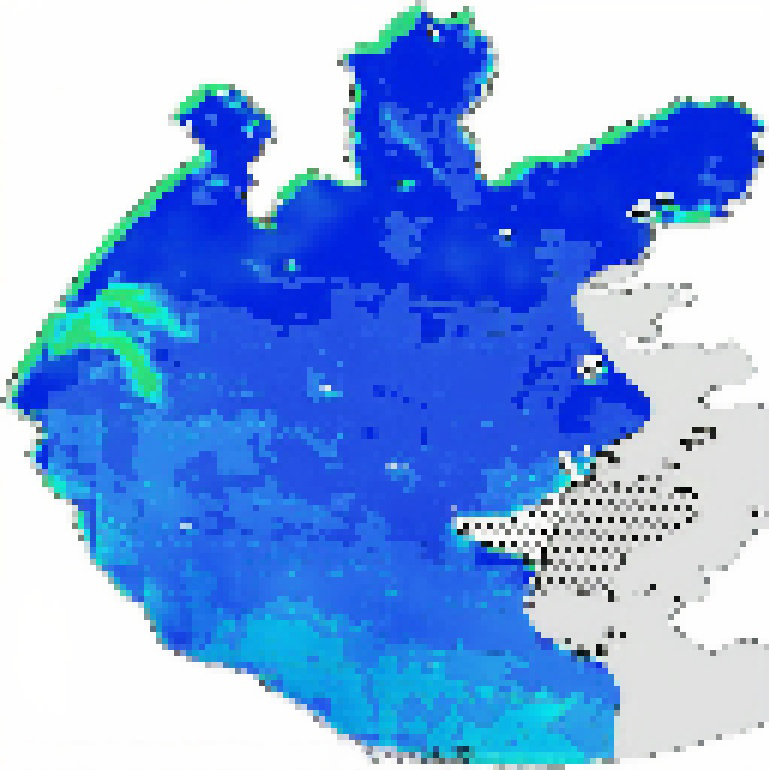

Supplement: Supplemental Information 5 [file peerj-cs-09-1292-s005.zip › batch2/fake/11.jpg]

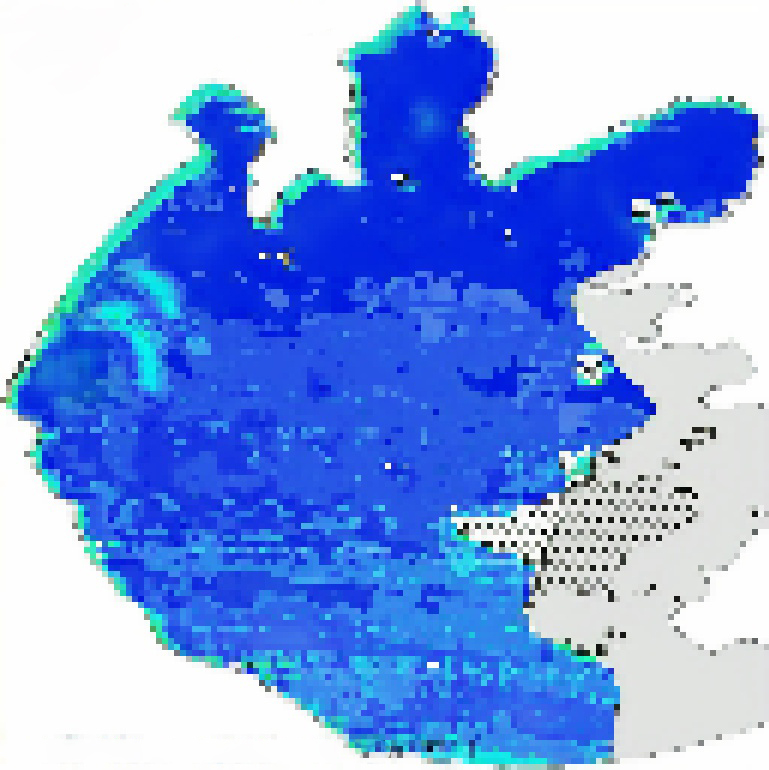

Supplement: Supplemental Information 5 [file peerj-cs-09-1292-s005.zip › batch2/fake/12.jpg]

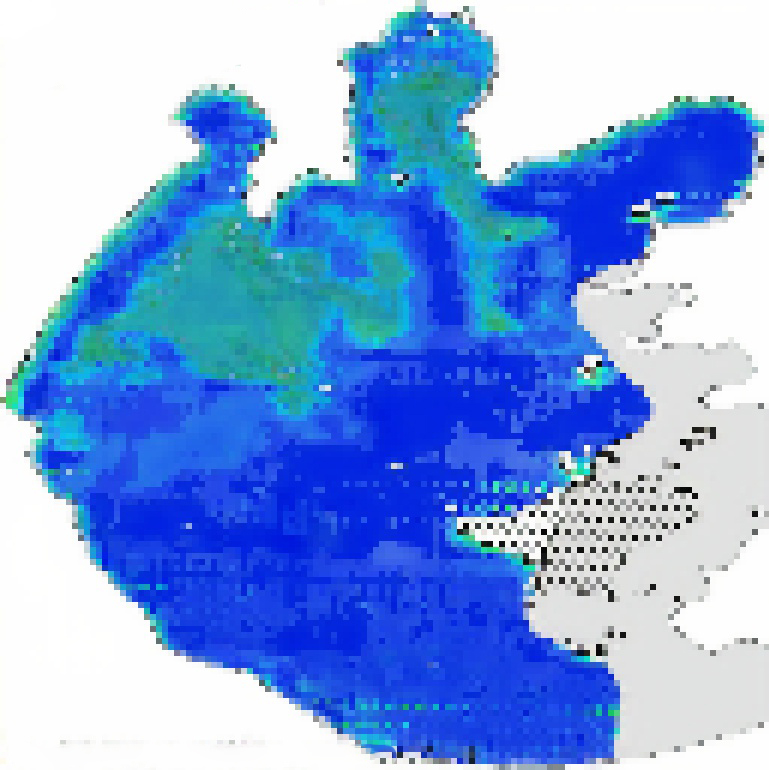

Supplement: Supplemental Information 5 [file peerj-cs-09-1292-s005.zip › batch2/fake/13.jpg]

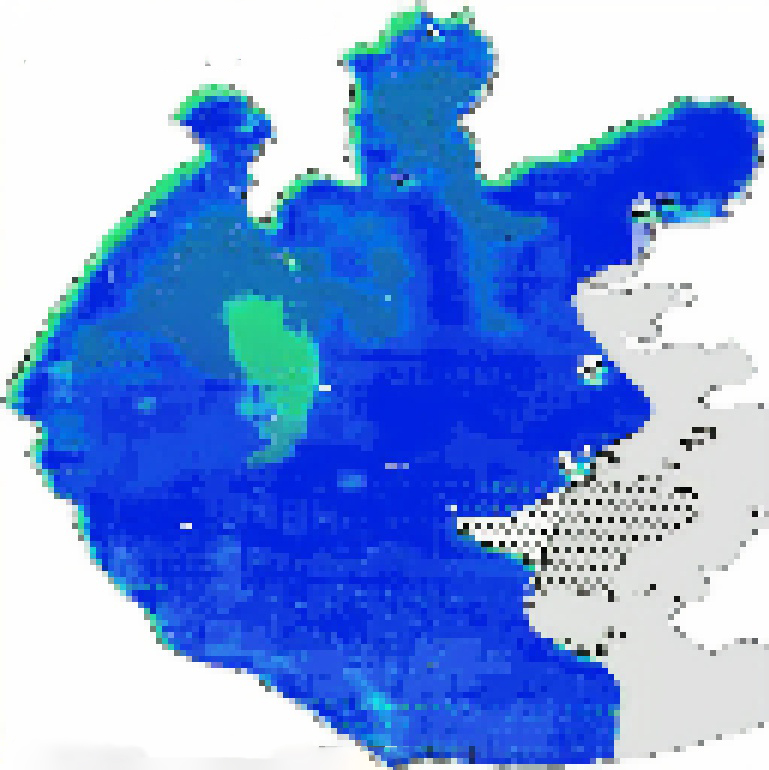

Supplement: Supplemental Information 5 [file peerj-cs-09-1292-s005.zip › batch2/fake/14.jpg]

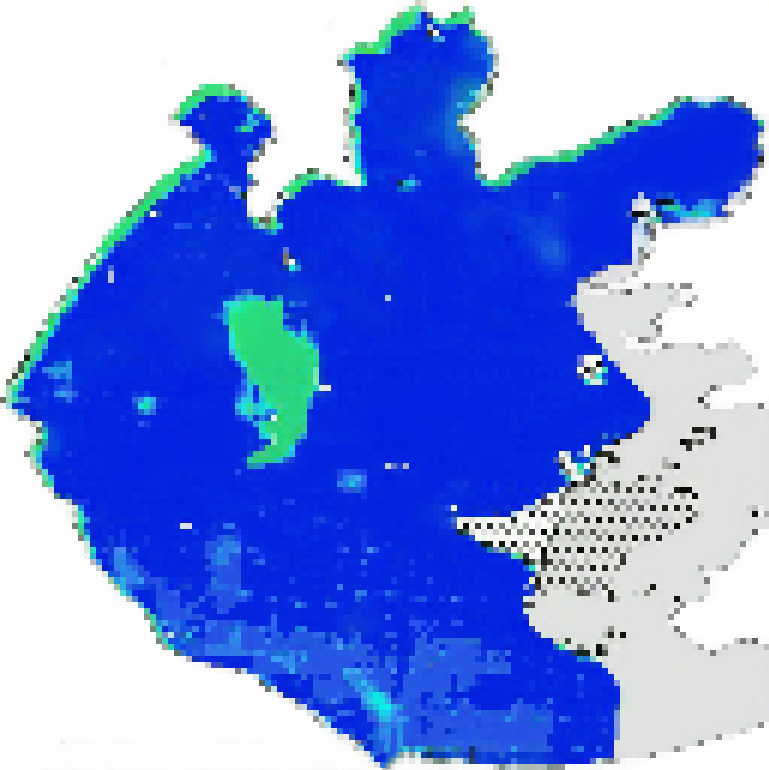

Supplement: Supplemental Information 5 [file peerj-cs-09-1292-s005.zip › batch2/fake/15.jpg]

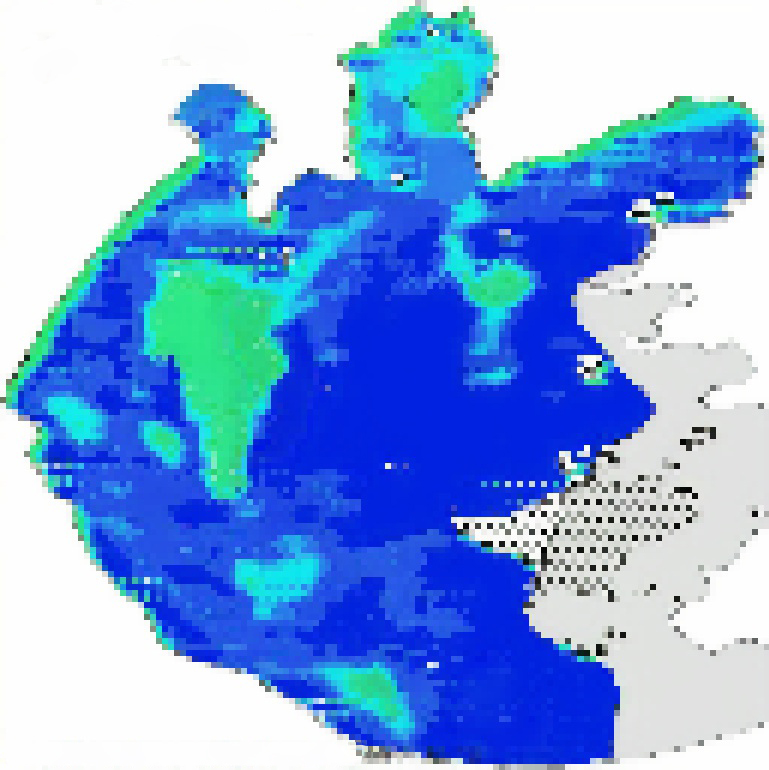

Supplement: Supplemental Information 5 [file peerj-cs-09-1292-s005.zip › batch2/fake/16.jpg]

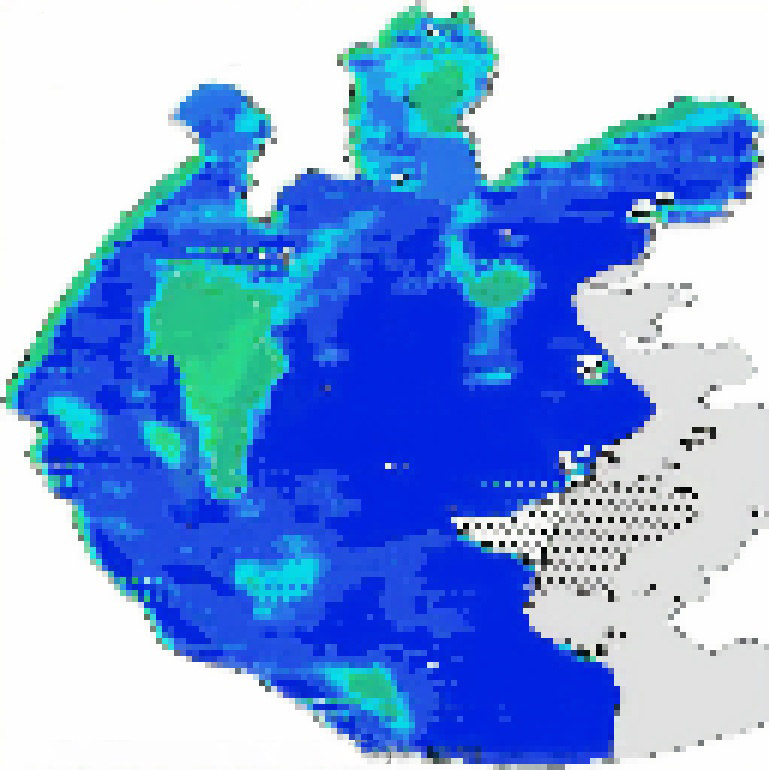

Supplement: Supplemental Information 5 [file peerj-cs-09-1292-s005.zip › batch2/fake/17.jpg]

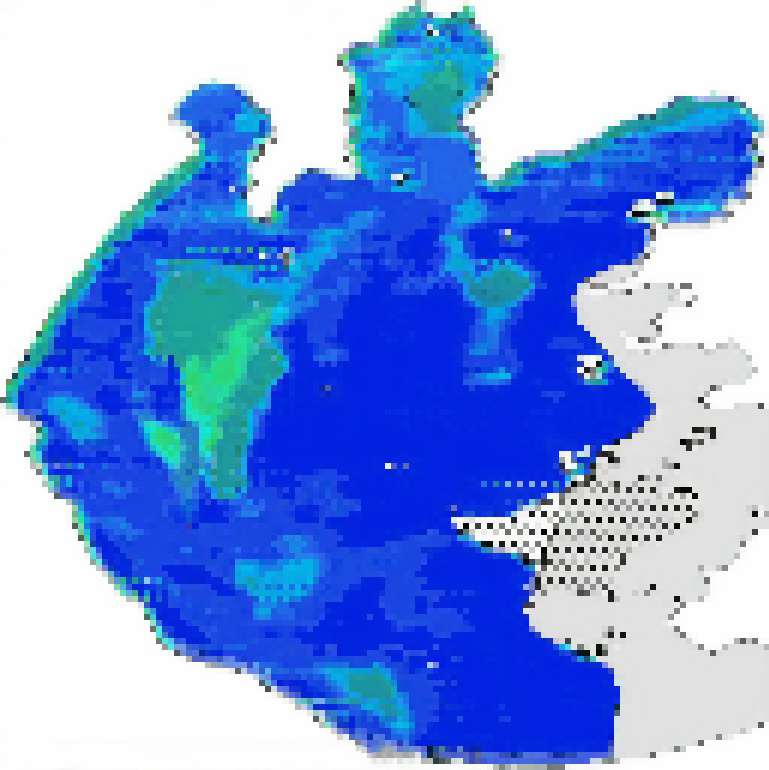

Supplement: Supplemental Information 5 [file peerj-cs-09-1292-s005.zip › batch2/fake/18.jpg]

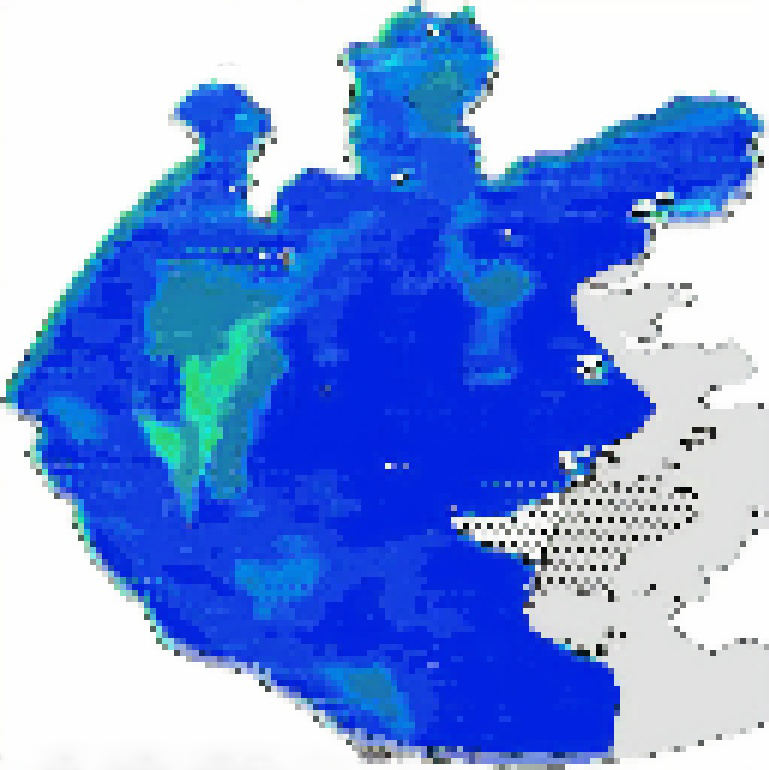

Supplement: Supplemental Information 5 [file peerj-cs-09-1292-s005.zip › batch2/fake/19.jpg]

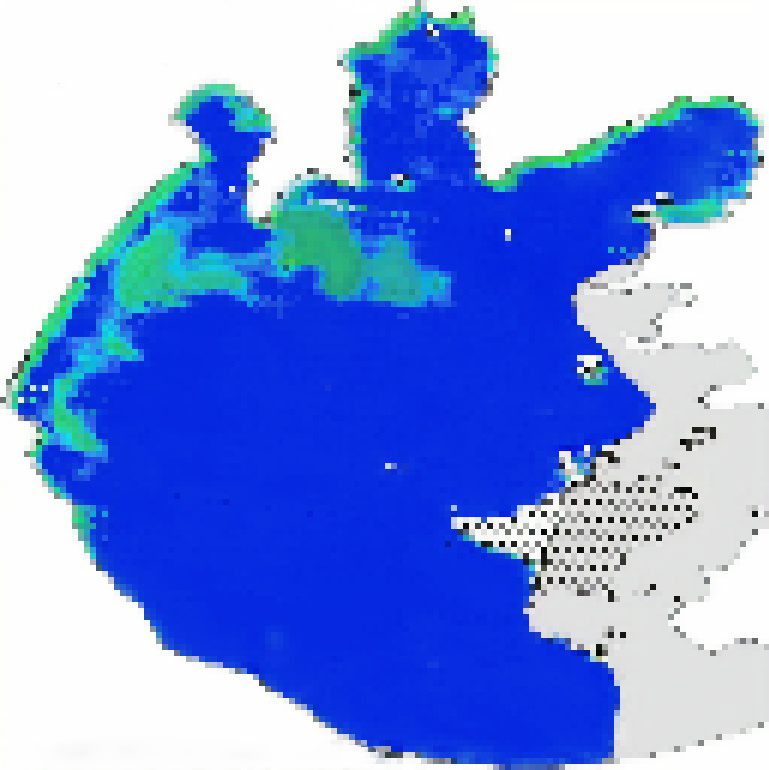

Supplement: Supplemental Information 5 [file peerj-cs-09-1292-s005.zip › batch2/fake/2.jpg]

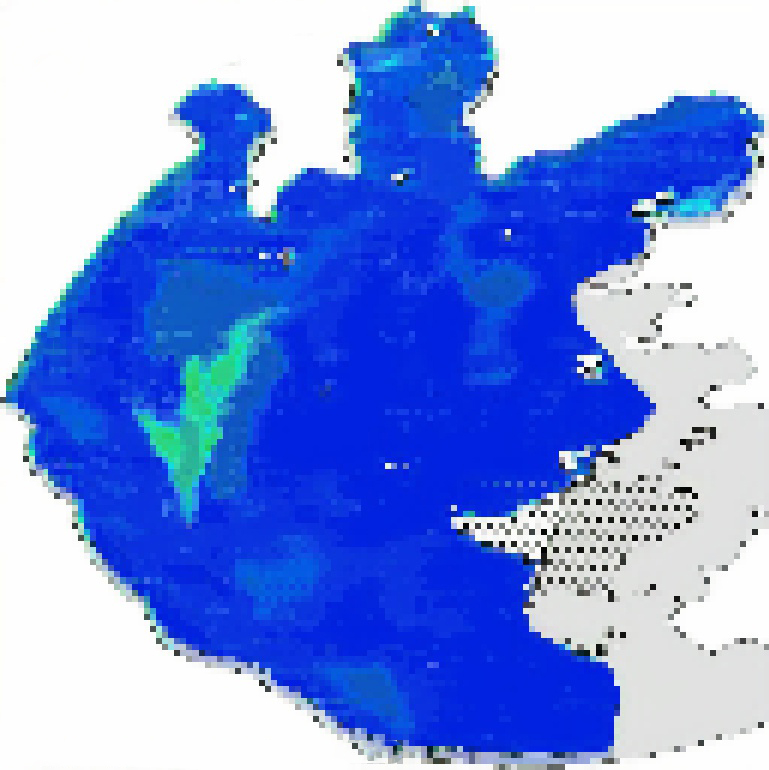

Supplement: Supplemental Information 5 [file peerj-cs-09-1292-s005.zip › batch2/fake/20.jpg]

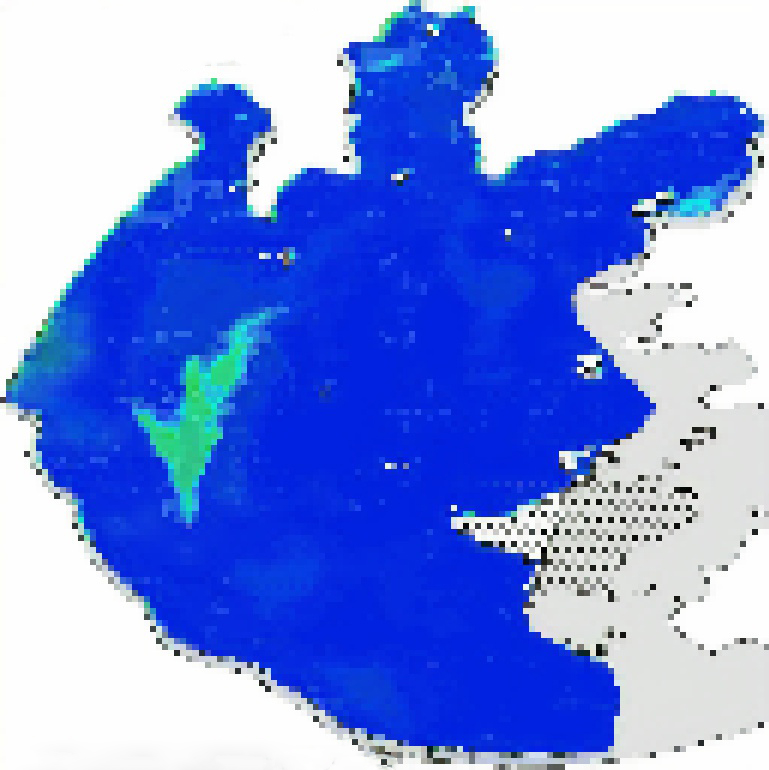

Supplement: Supplemental Information 5 [file peerj-cs-09-1292-s005.zip › batch2/fake/21.jpg]

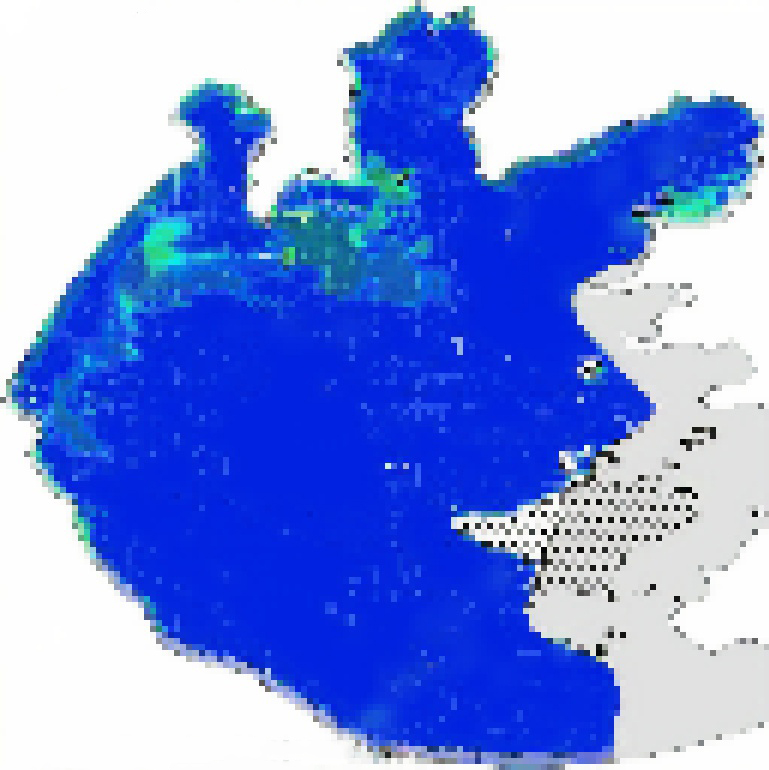

Supplement: Supplemental Information 5 [file peerj-cs-09-1292-s005.zip › batch2/fake/3.jpg]

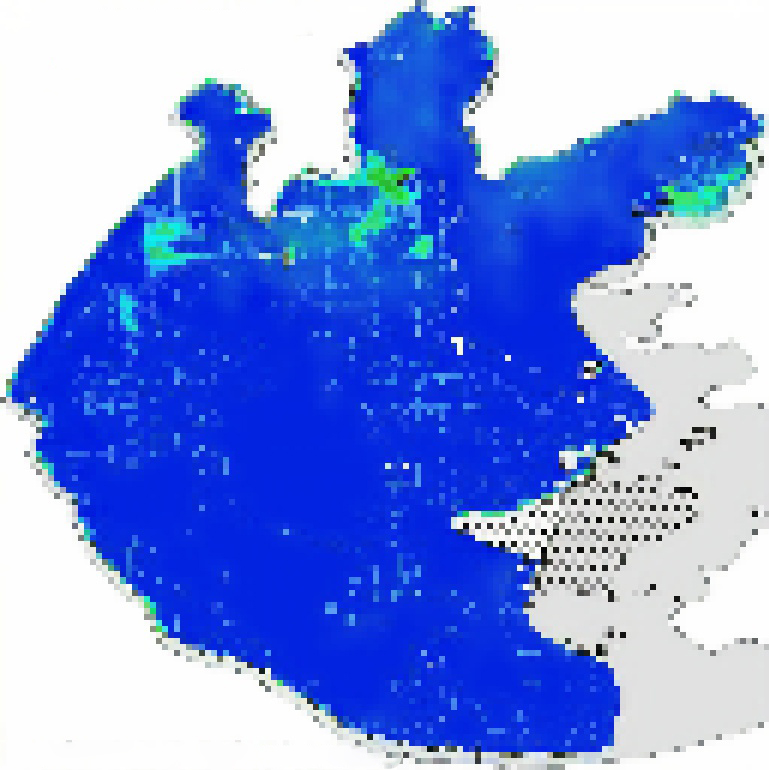

Supplement: Supplemental Information 5 [file peerj-cs-09-1292-s005.zip › batch2/fake/4.jpg]

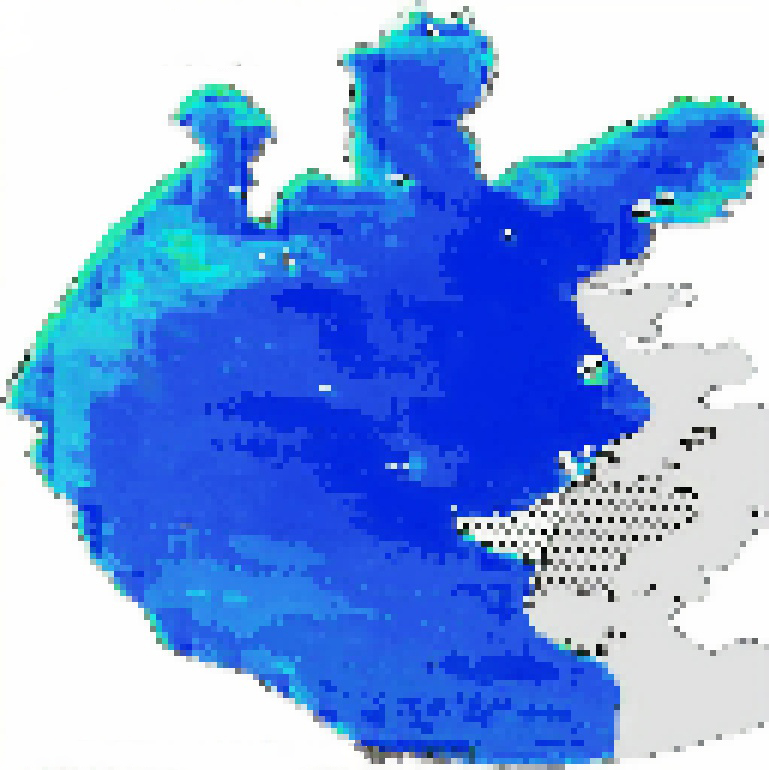

Supplement: Supplemental Information 5 [file peerj-cs-09-1292-s005.zip › batch2/fake/5.jpg]

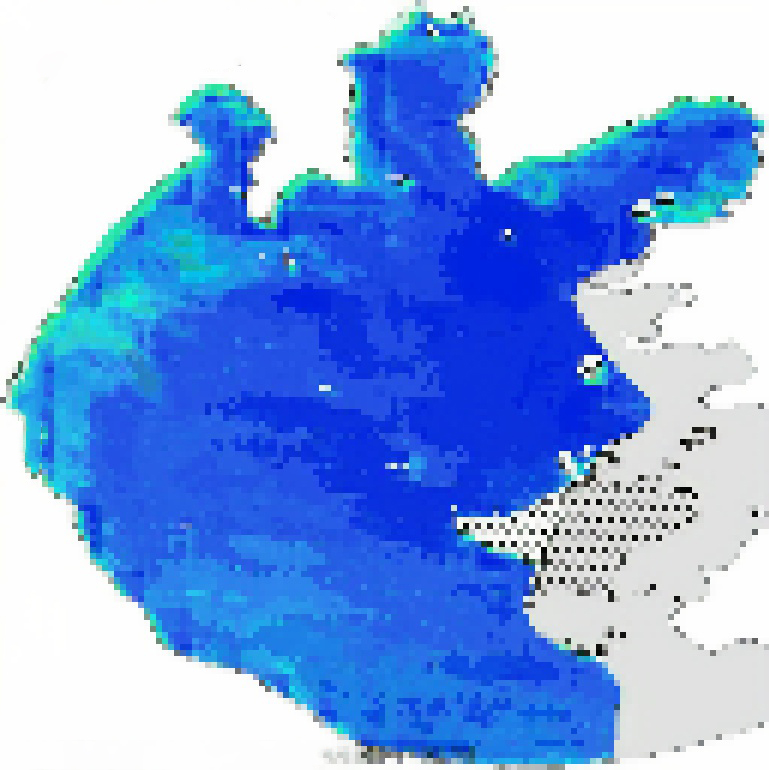

Supplement: Supplemental Information 5 [file peerj-cs-09-1292-s005.zip › batch2/fake/6.jpg]

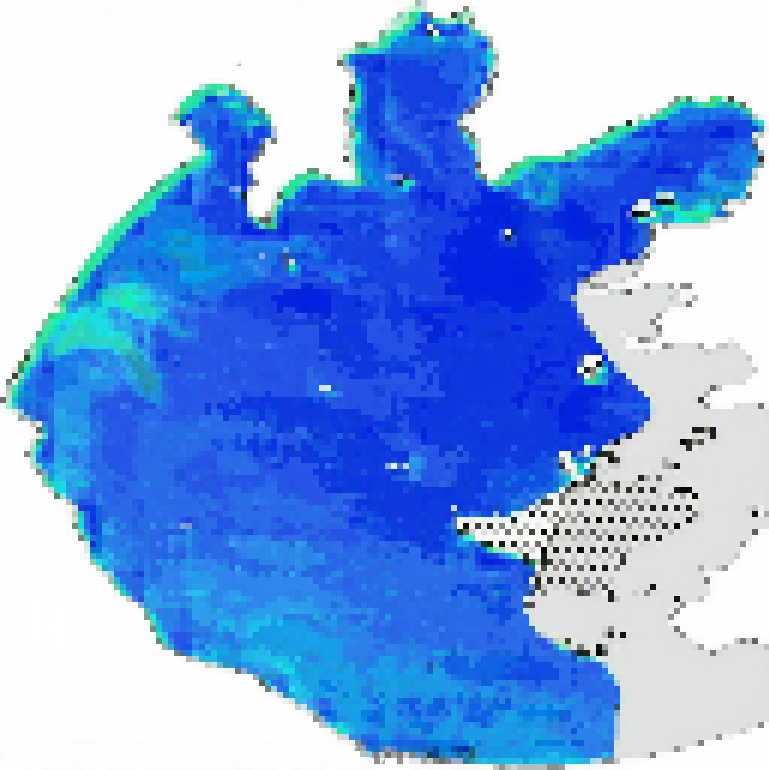

Supplement: Supplemental Information 5 [file peerj-cs-09-1292-s005.zip › batch2/fake/7.jpg]

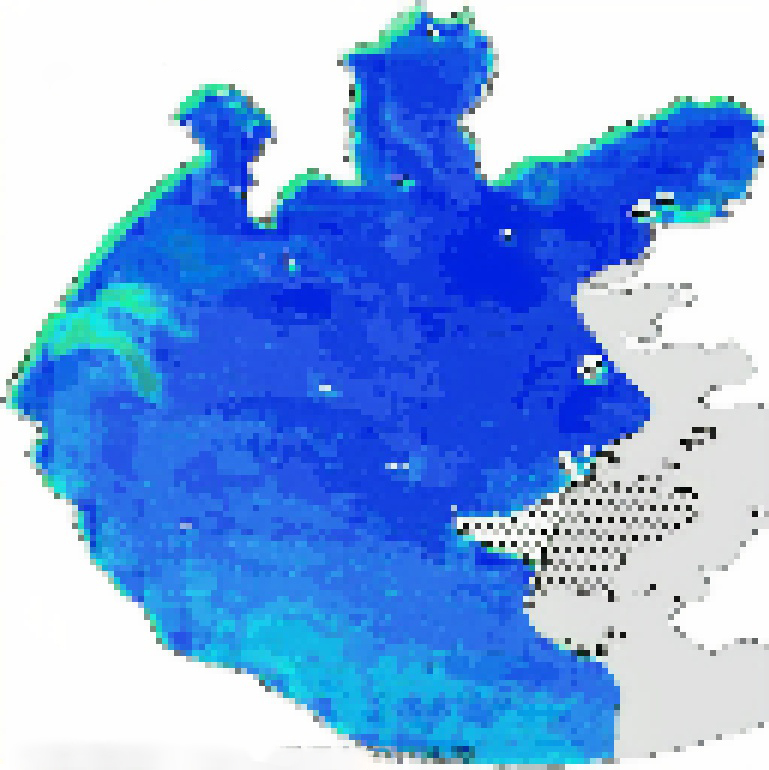

Supplement: Supplemental Information 5 [file peerj-cs-09-1292-s005.zip › batch2/fake/8.jpg]

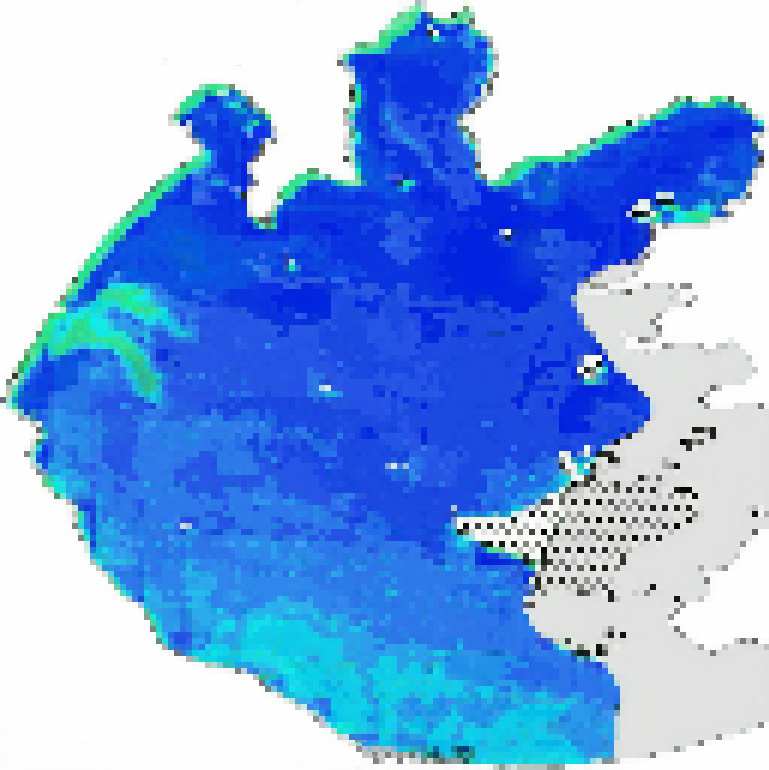

Supplement: Supplemental Information 5 [file peerj-cs-09-1292-s005.zip › batch2/fake/9.jpg]

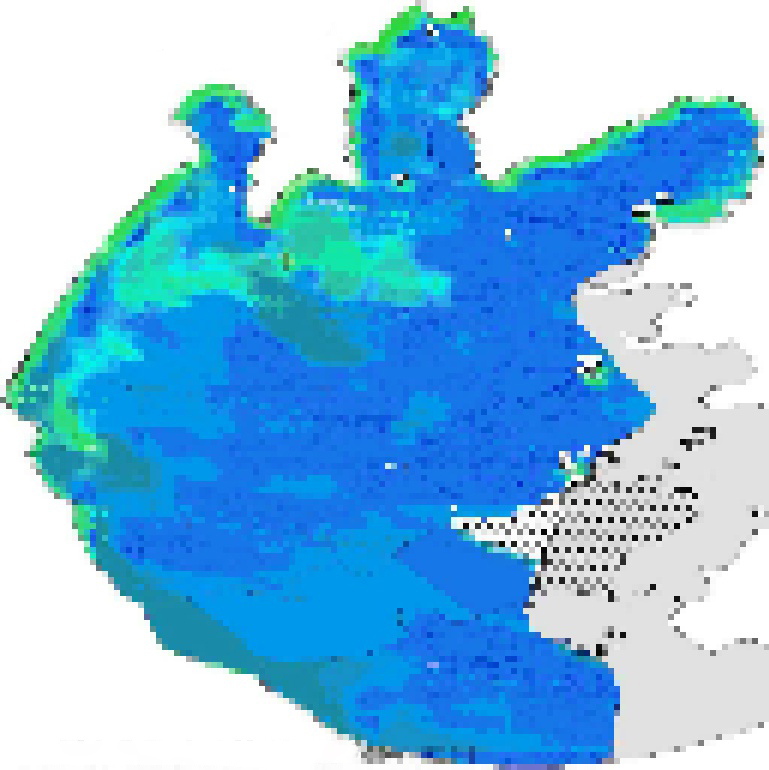

Supplement: Supplemental Information 5 [file peerj-cs-09-1292-s005.zip › batch2/real/0.jpg]

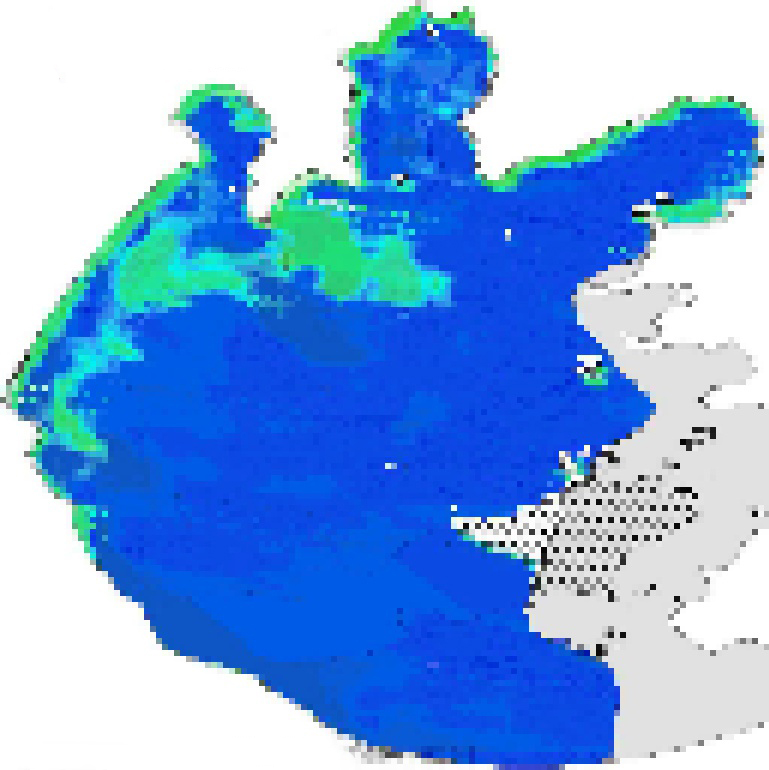

Supplement: Supplemental Information 5 [file peerj-cs-09-1292-s005.zip › batch2/real/1.jpg]

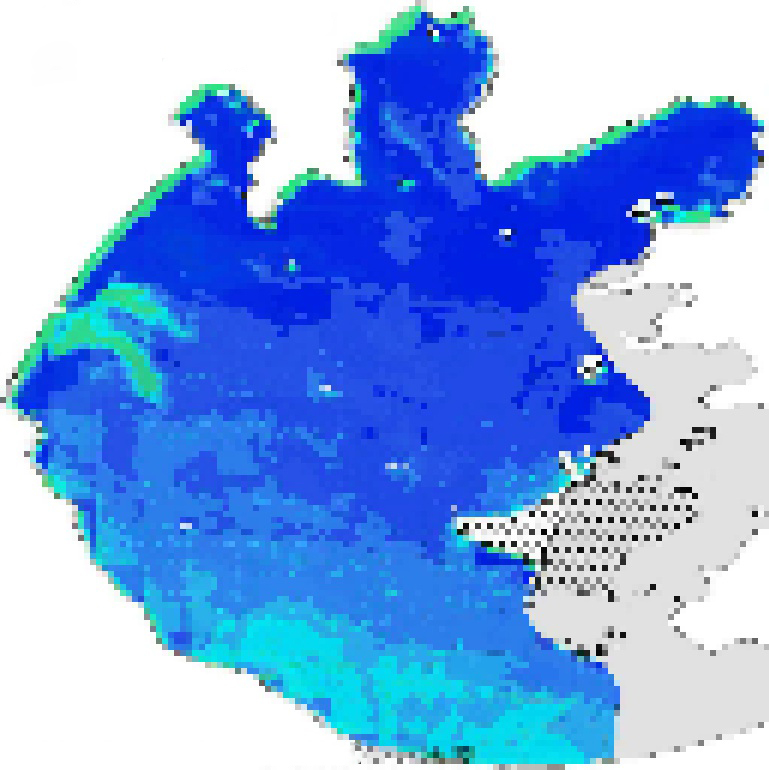

Supplement: Supplemental Information 5 [file peerj-cs-09-1292-s005.zip › batch2/real/10.jpg]

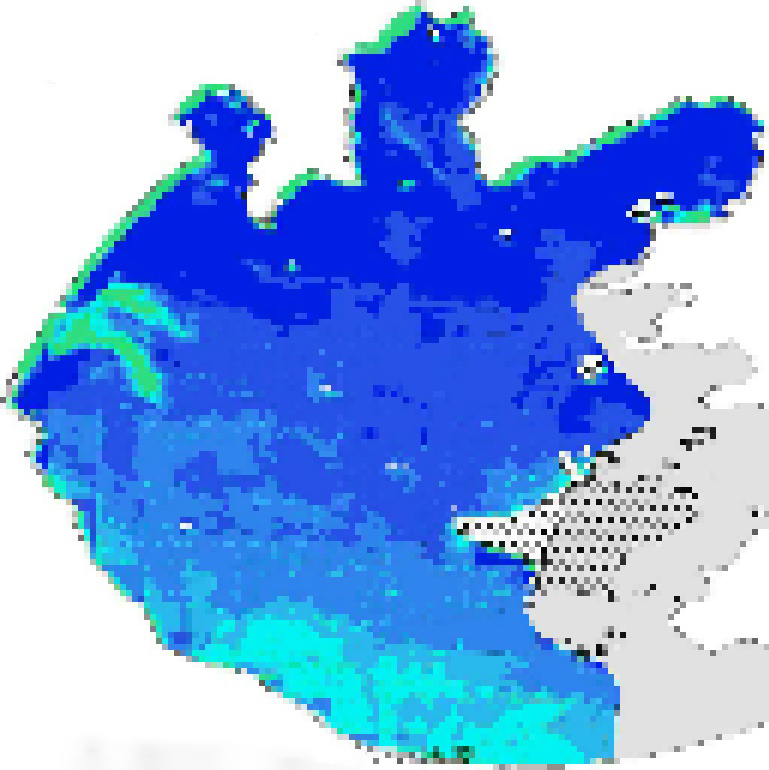

Supplement: Supplemental Information 5 [file peerj-cs-09-1292-s005.zip › batch2/real/11.jpg]

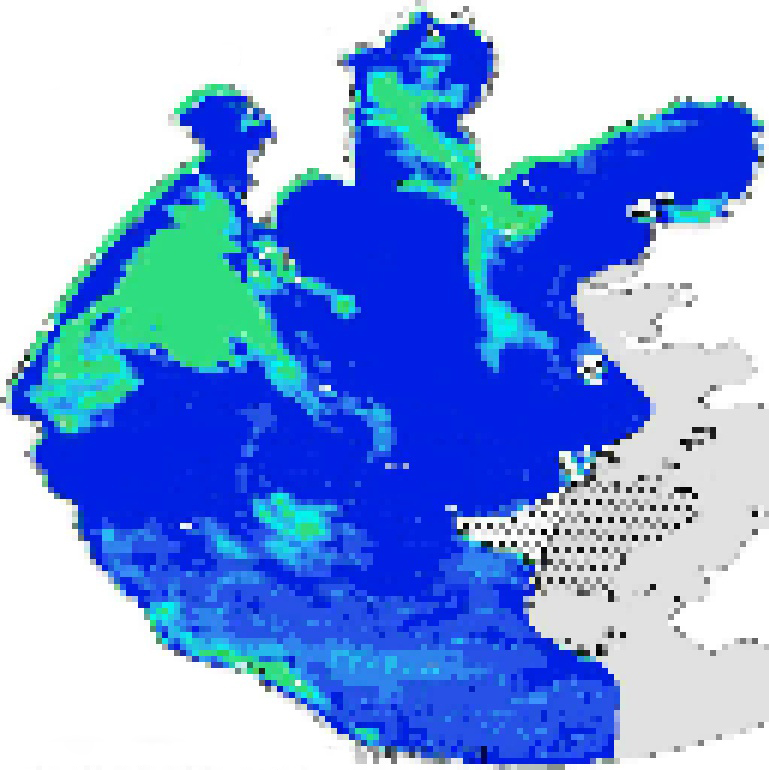

Supplement: Supplemental Information 5 [file peerj-cs-09-1292-s005.zip › batch2/real/12.jpg]

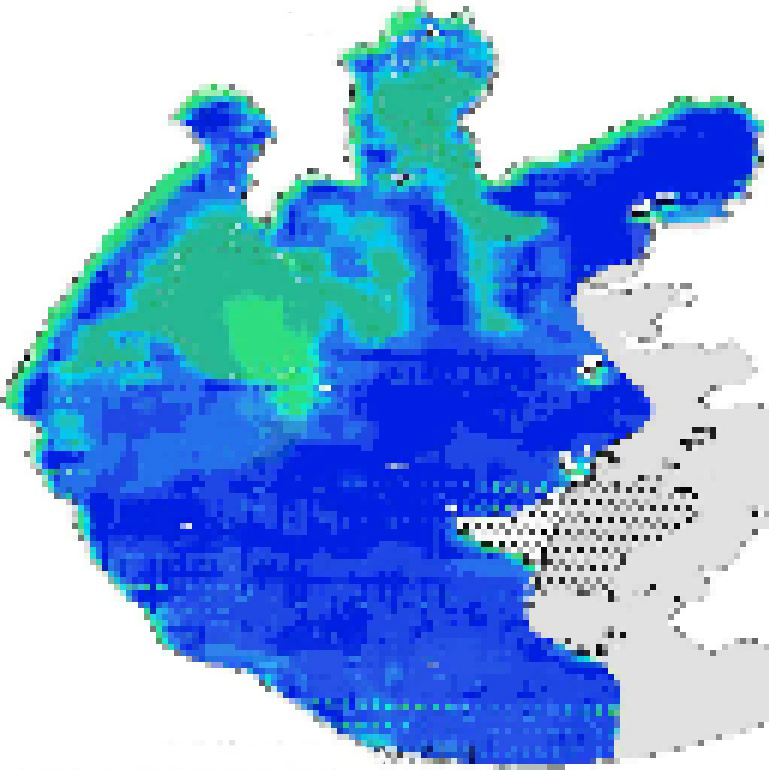

Supplement: Supplemental Information 5 [file peerj-cs-09-1292-s005.zip › batch2/real/13.jpg]

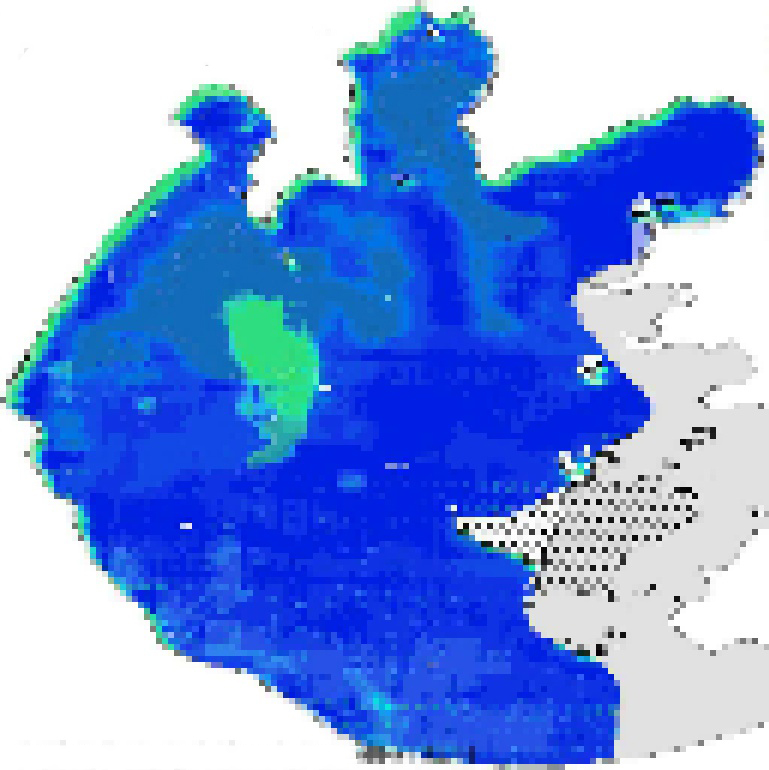

Supplement: Supplemental Information 5 [file peerj-cs-09-1292-s005.zip › batch2/real/14.jpg]

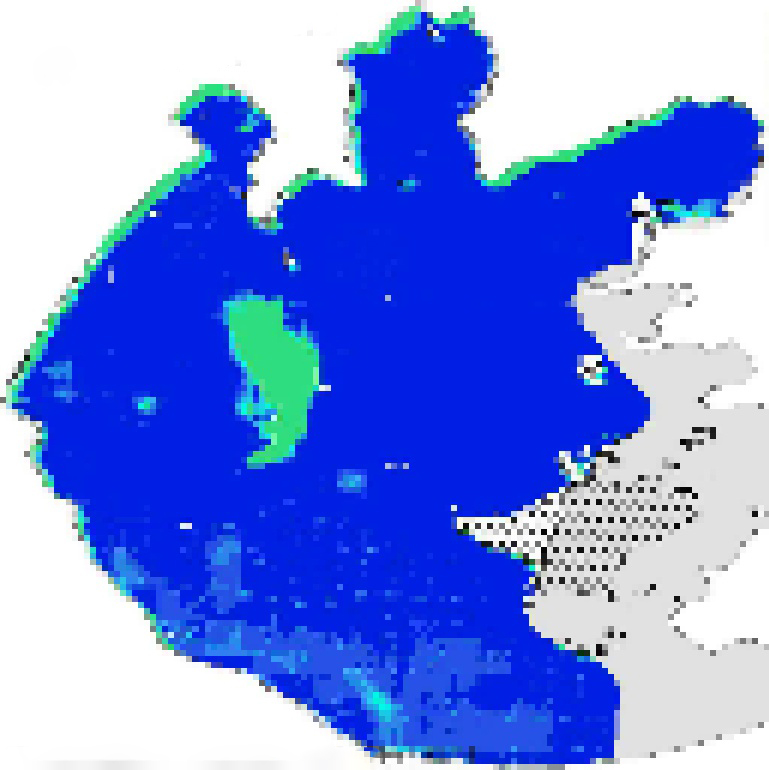

Supplement: Supplemental Information 5 [file peerj-cs-09-1292-s005.zip › batch2/real/15.jpg]

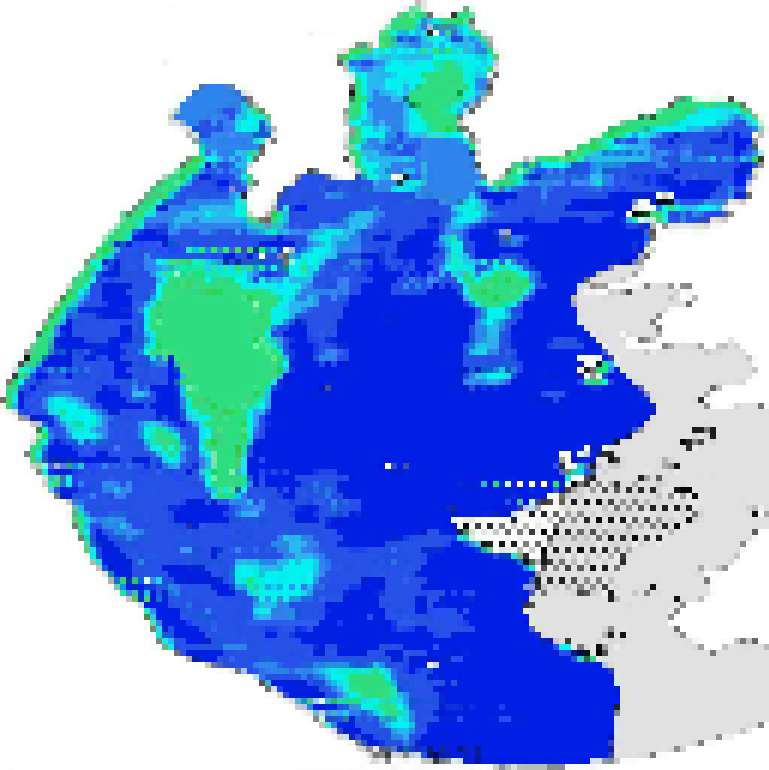

Supplement: Supplemental Information 5 [file peerj-cs-09-1292-s005.zip › batch2/real/16.jpg]

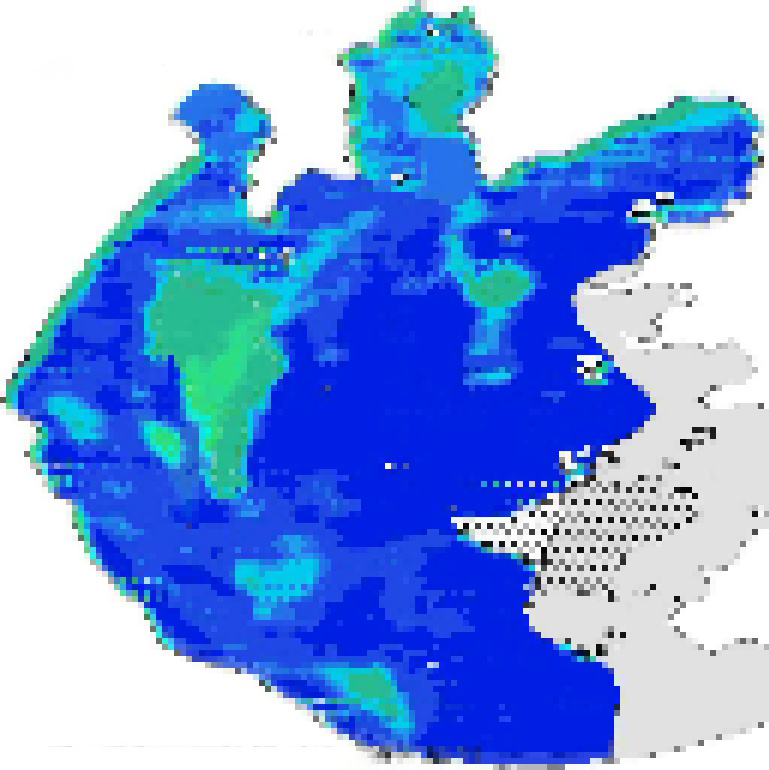

Supplement: Supplemental Information 5 [file peerj-cs-09-1292-s005.zip › batch2/real/17.jpg]

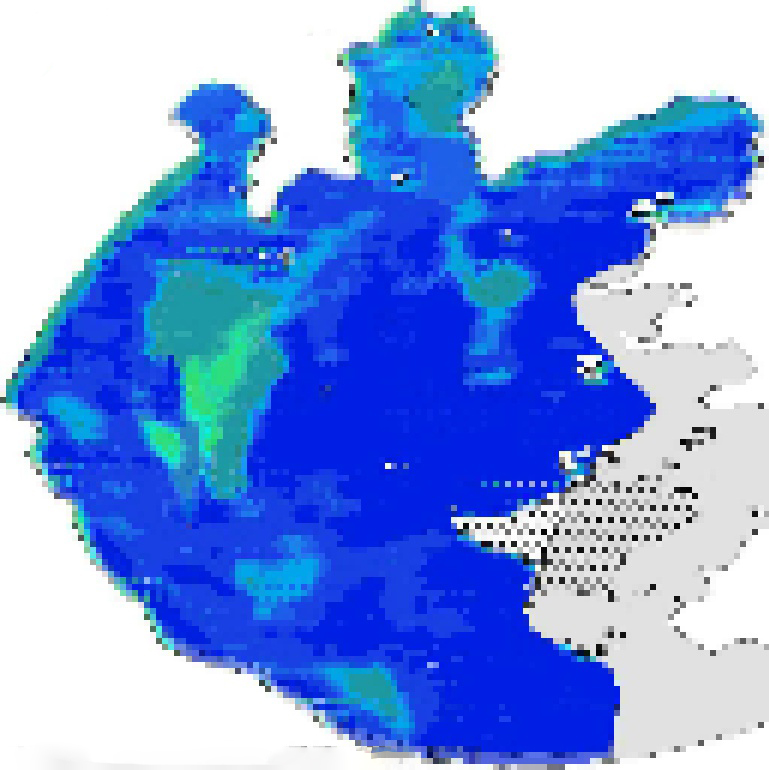

Supplement: Supplemental Information 5 [file peerj-cs-09-1292-s005.zip › batch2/real/18.jpg]

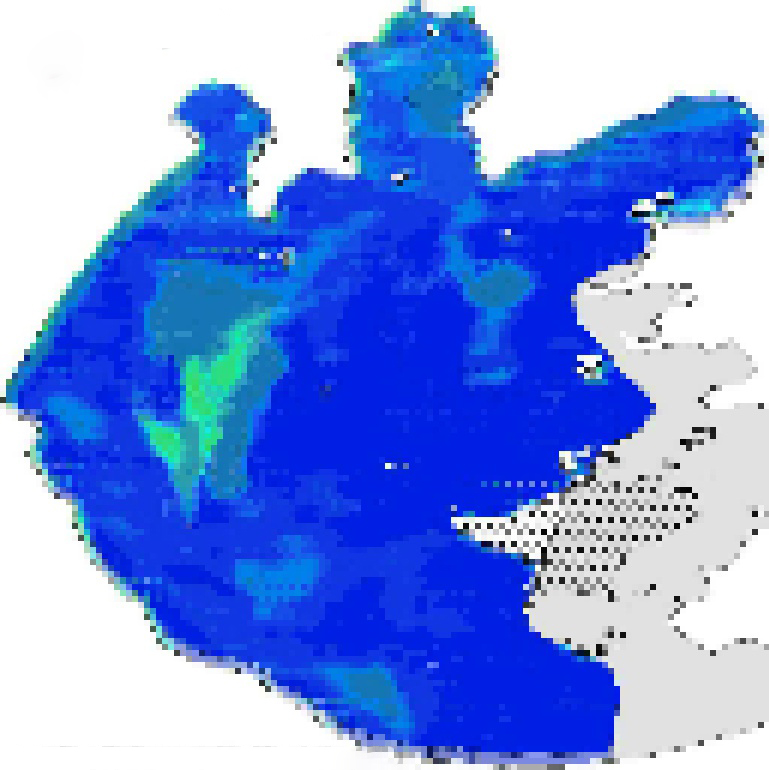

Supplement: Supplemental Information 5 [file peerj-cs-09-1292-s005.zip › batch2/real/19.jpg]

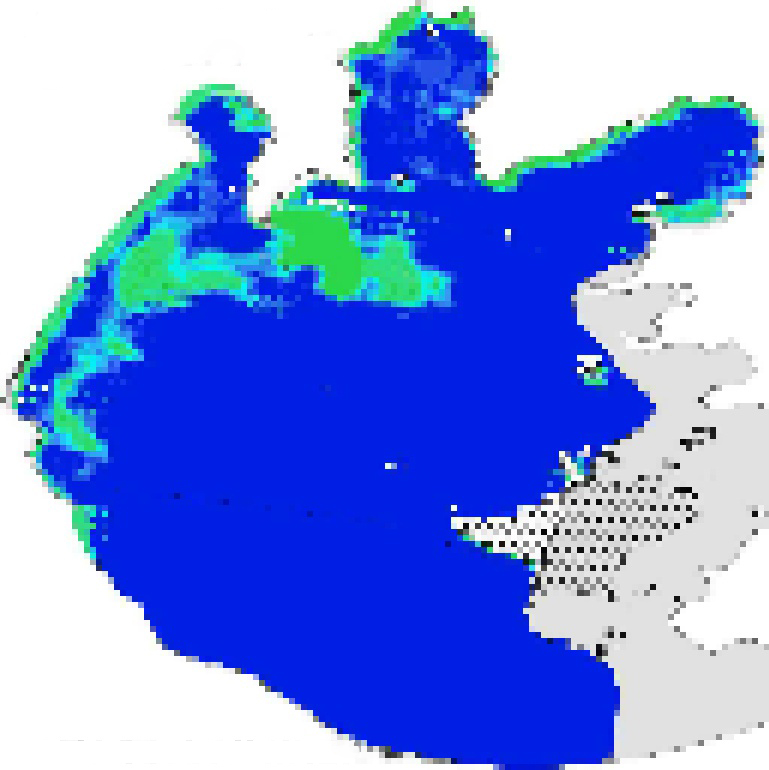

Supplement: Supplemental Information 5 [file peerj-cs-09-1292-s005.zip › batch2/real/2.jpg]

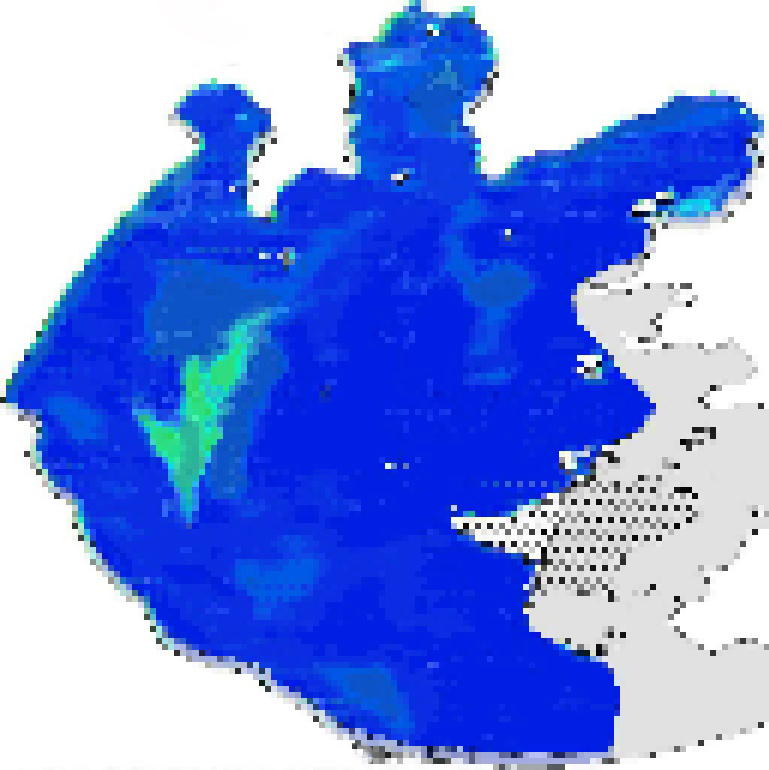

Supplement: Supplemental Information 5 [file peerj-cs-09-1292-s005.zip › batch2/real/20.jpg]

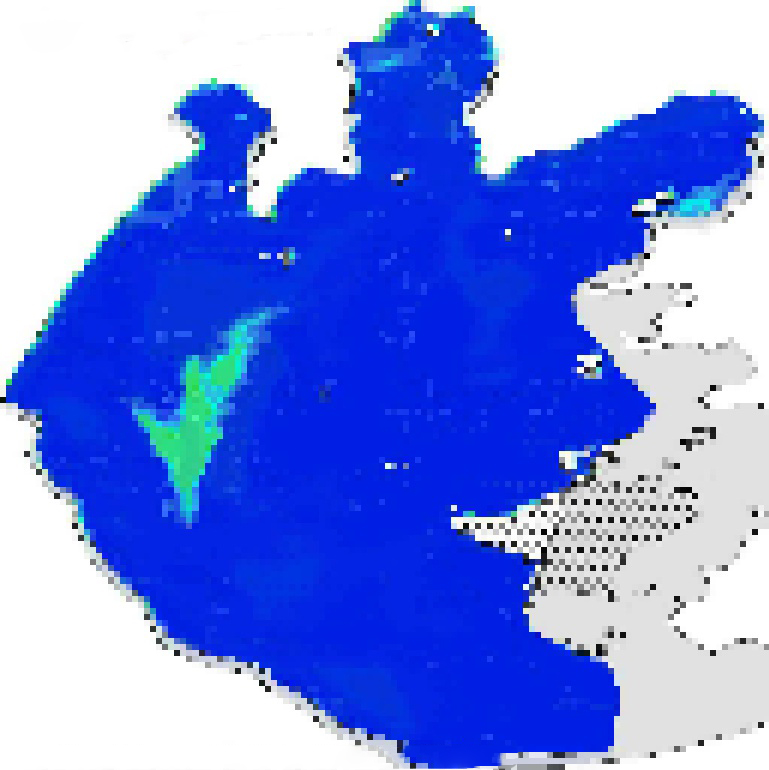

Supplement: Supplemental Information 5 [file peerj-cs-09-1292-s005.zip › batch2/real/21.jpg]

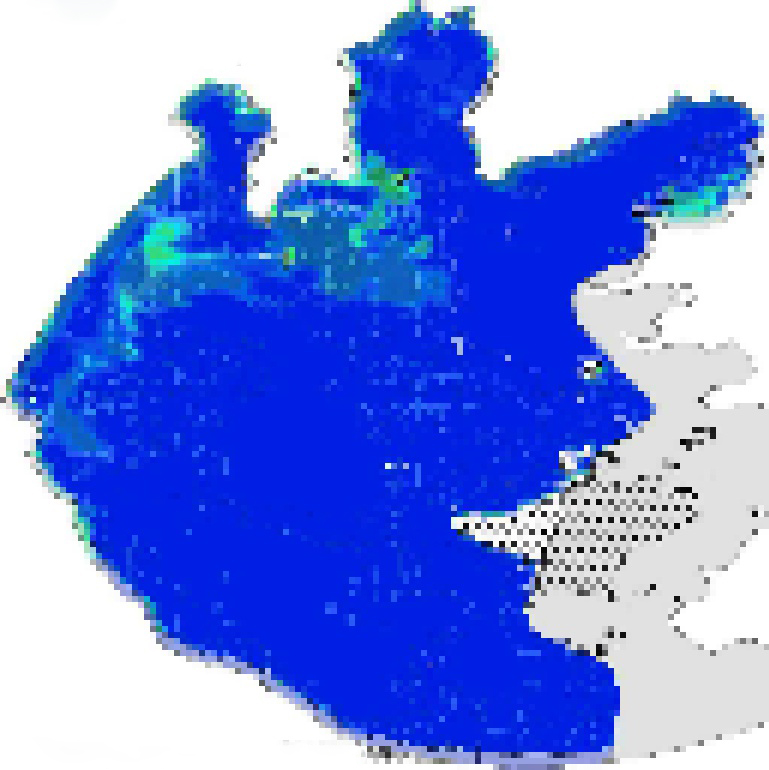

Supplement: Supplemental Information 5 [file peerj-cs-09-1292-s005.zip › batch2/real/3.jpg]

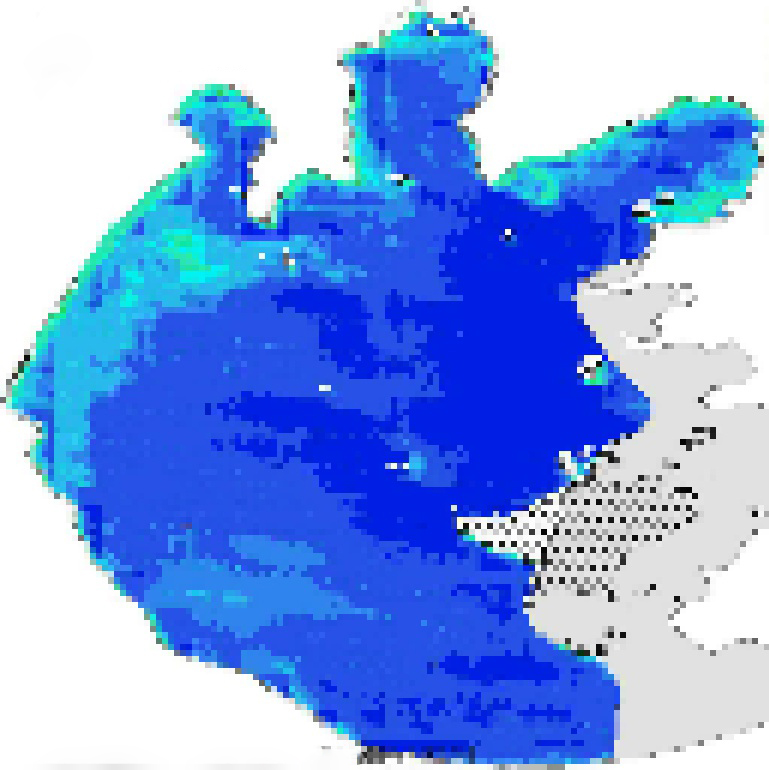

Supplement: Supplemental Information 5 [file peerj-cs-09-1292-s005.zip › batch2/real/4.jpg]

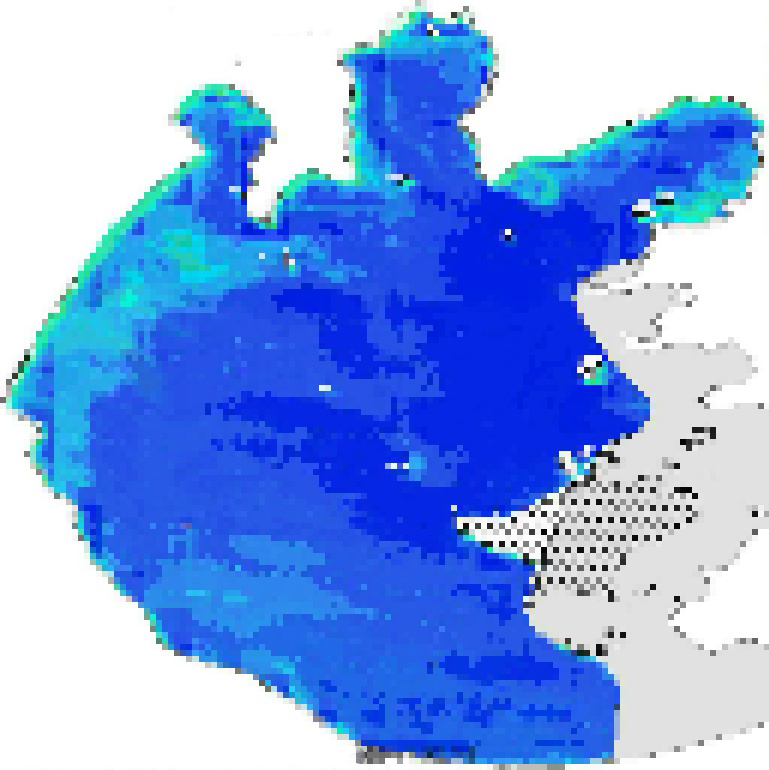

Supplement: Supplemental Information 5 [file peerj-cs-09-1292-s005.zip › batch2/real/5.jpg]

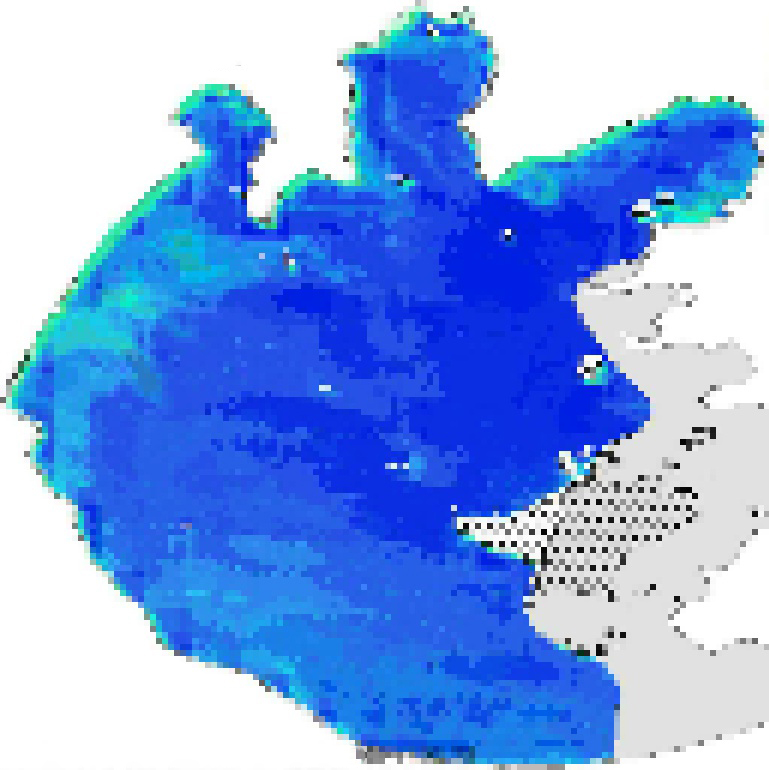

Supplement: Supplemental Information 5 [file peerj-cs-09-1292-s005.zip › batch2/real/6.jpg]

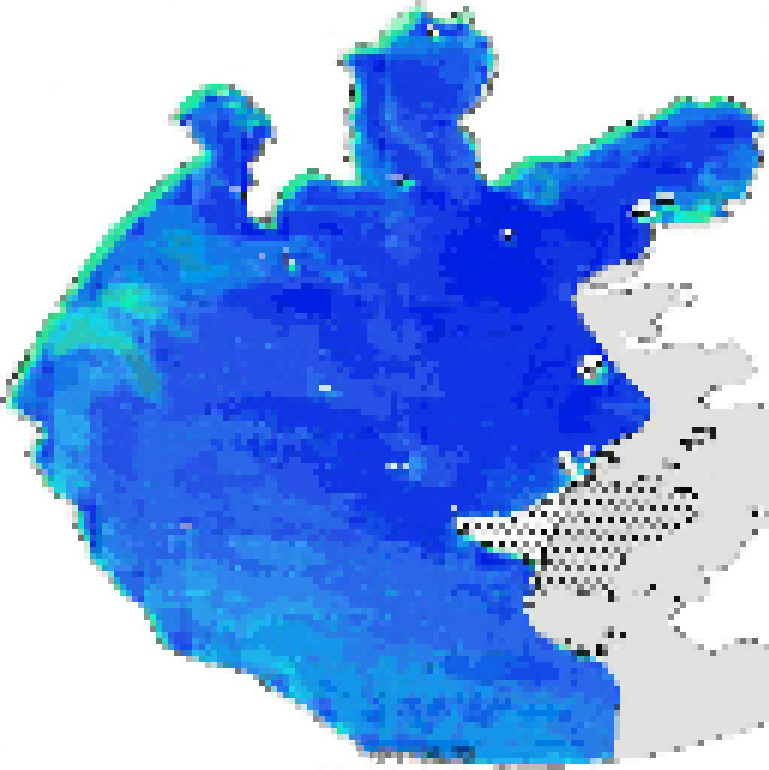

Supplement: Supplemental Information 5 [file peerj-cs-09-1292-s005.zip › batch2/real/7.jpg]

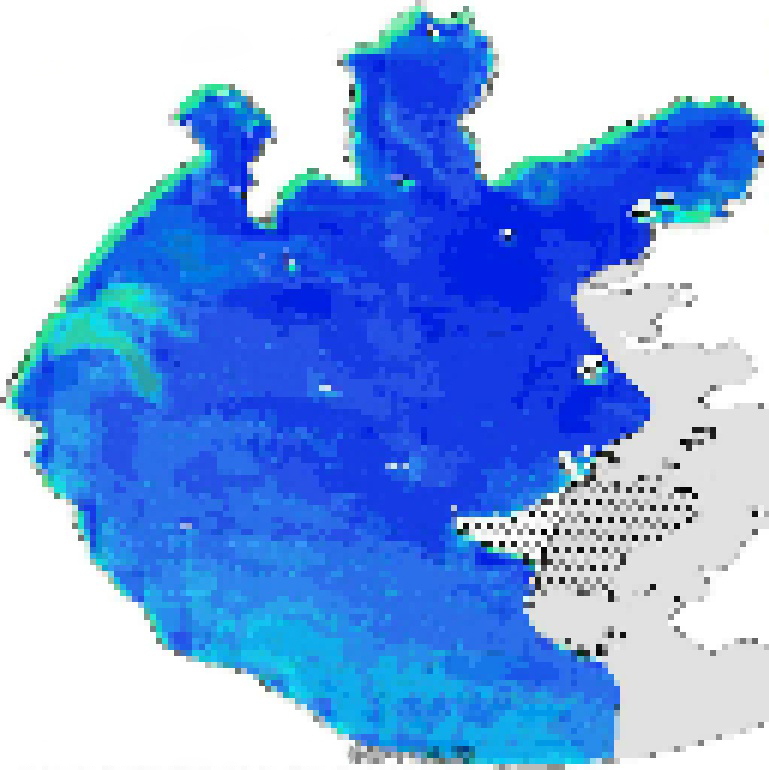

Supplement: Supplemental Information 5 [file peerj-cs-09-1292-s005.zip › batch2/real/8.jpg]

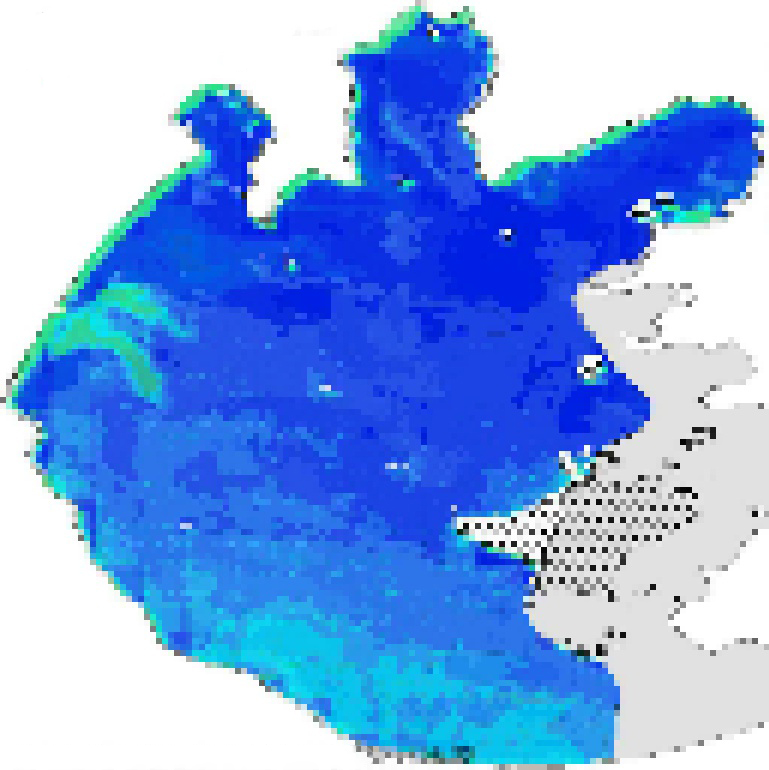

Supplement: Supplemental Information 5 [file peerj-cs-09-1292-s005.zip › batch2/real/9.jpg]

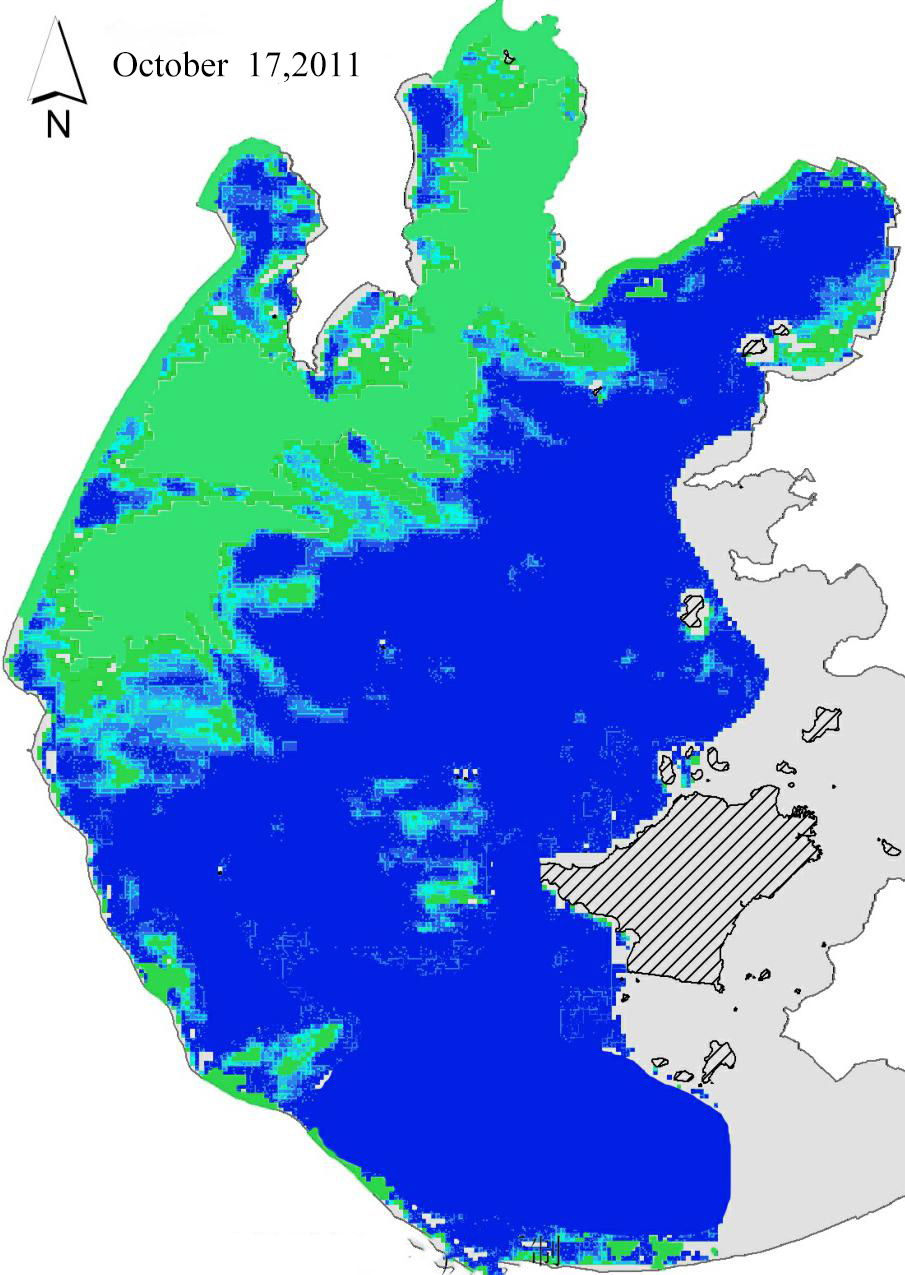

Supplement: Supplemental Information 6 [file peerj-cs-09-1292-s006.zip › 0/20111017_taihu_cla.jpg]

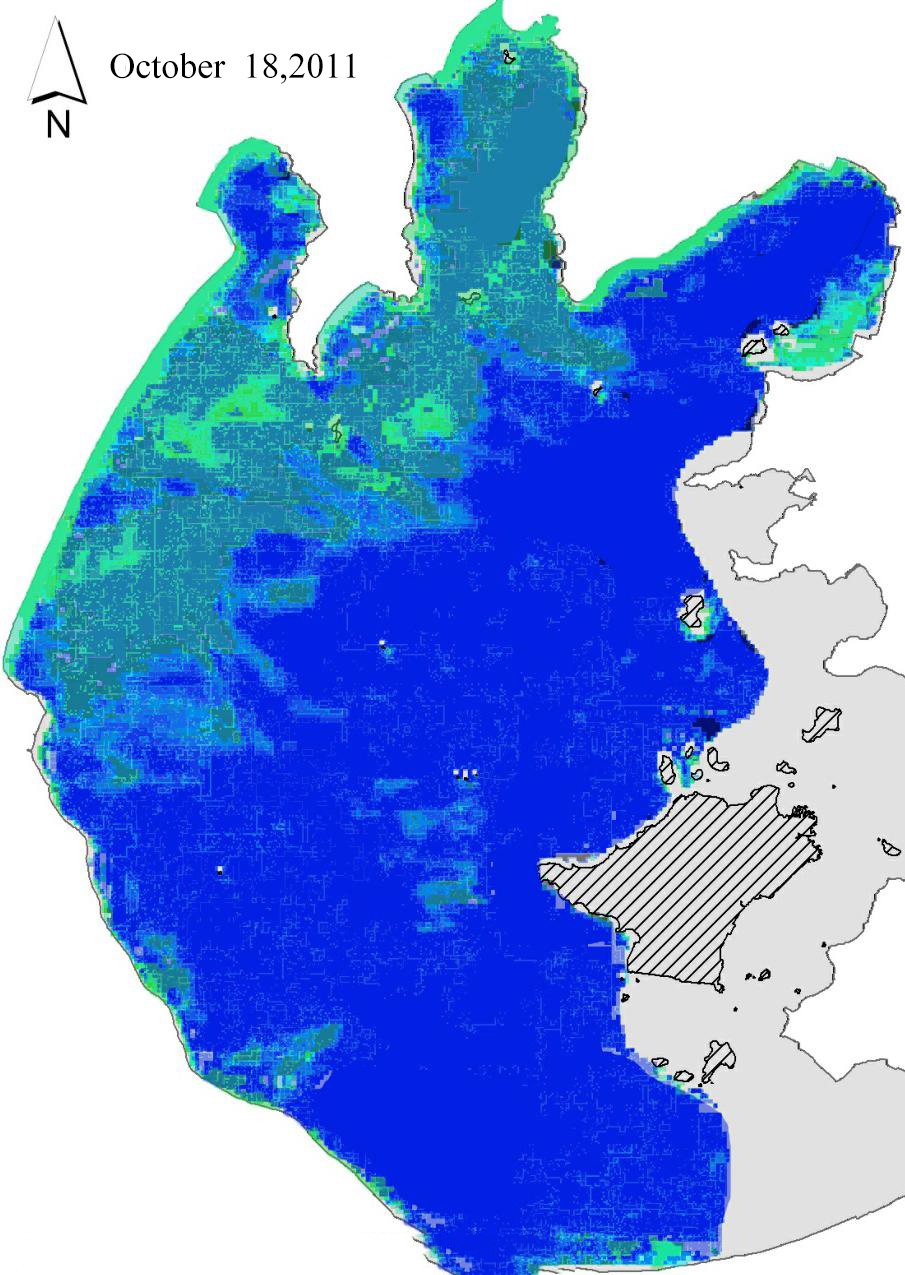

Supplement: Supplemental Information 6 [file peerj-cs-09-1292-s006.zip › 0/20111018_taihu_cla.jpg]

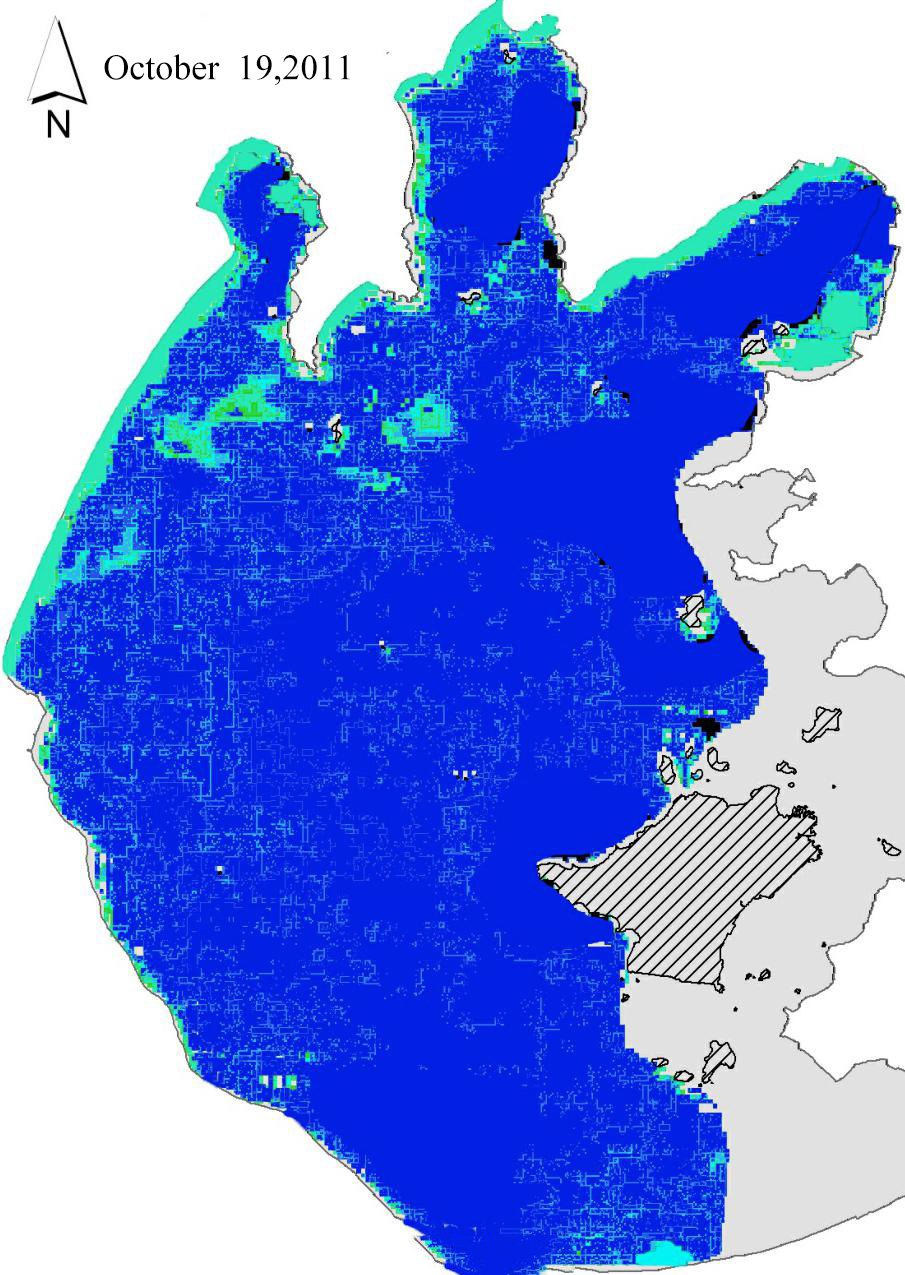

Supplement: Supplemental Information 6 [file peerj-cs-09-1292-s006.zip › 0/20111019_taihu_cla.jpg]

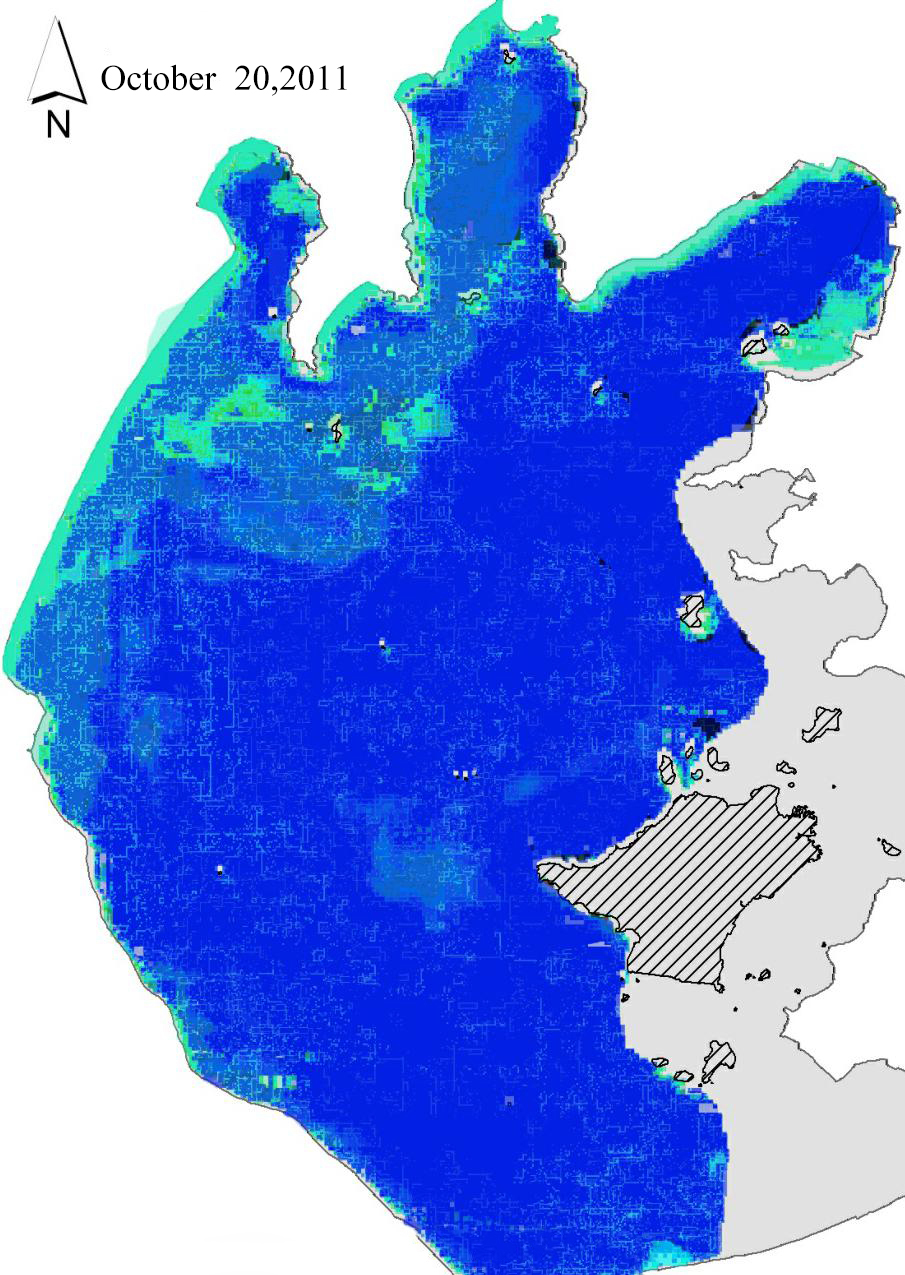

Supplement: Supplemental Information 6 [file peerj-cs-09-1292-s006.zip › 0/20111020_taihu_cla.jpg]

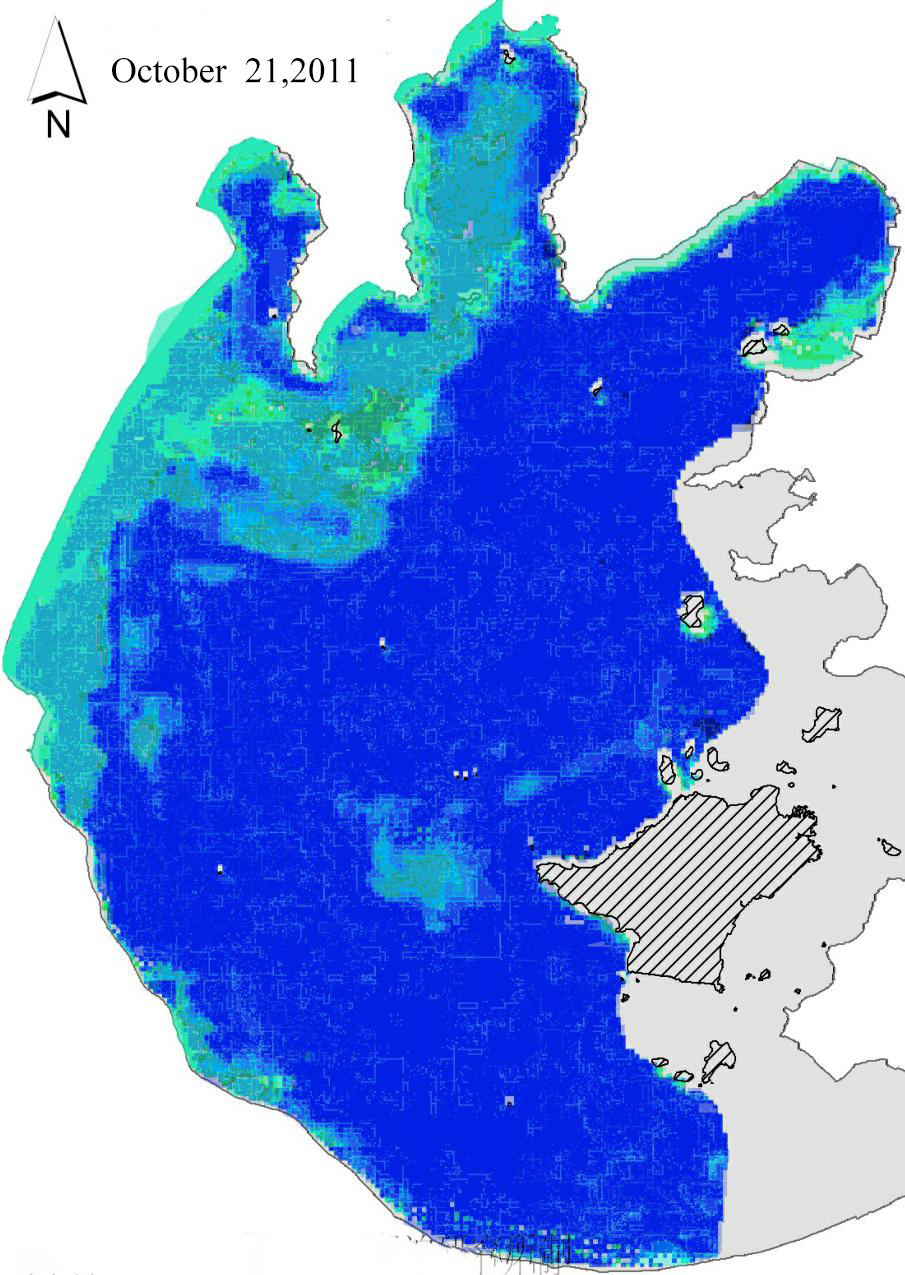

Supplement: Supplemental Information 6 [file peerj-cs-09-1292-s006.zip › 0/20111021_taihu_cla.jpg]

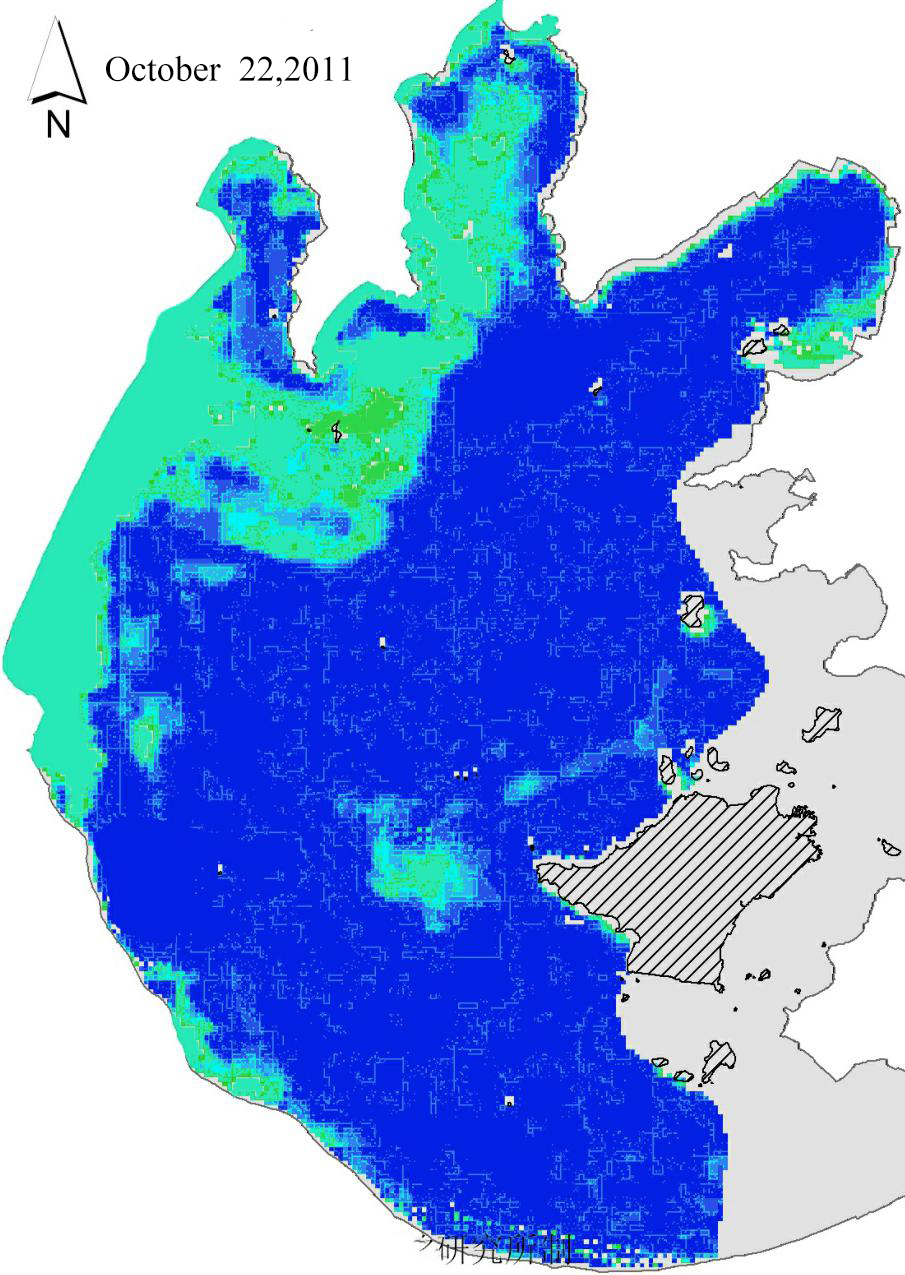

Supplement: Supplemental Information 6 [file peerj-cs-09-1292-s006.zip › 0/20111022_taihu_cla.jpg]

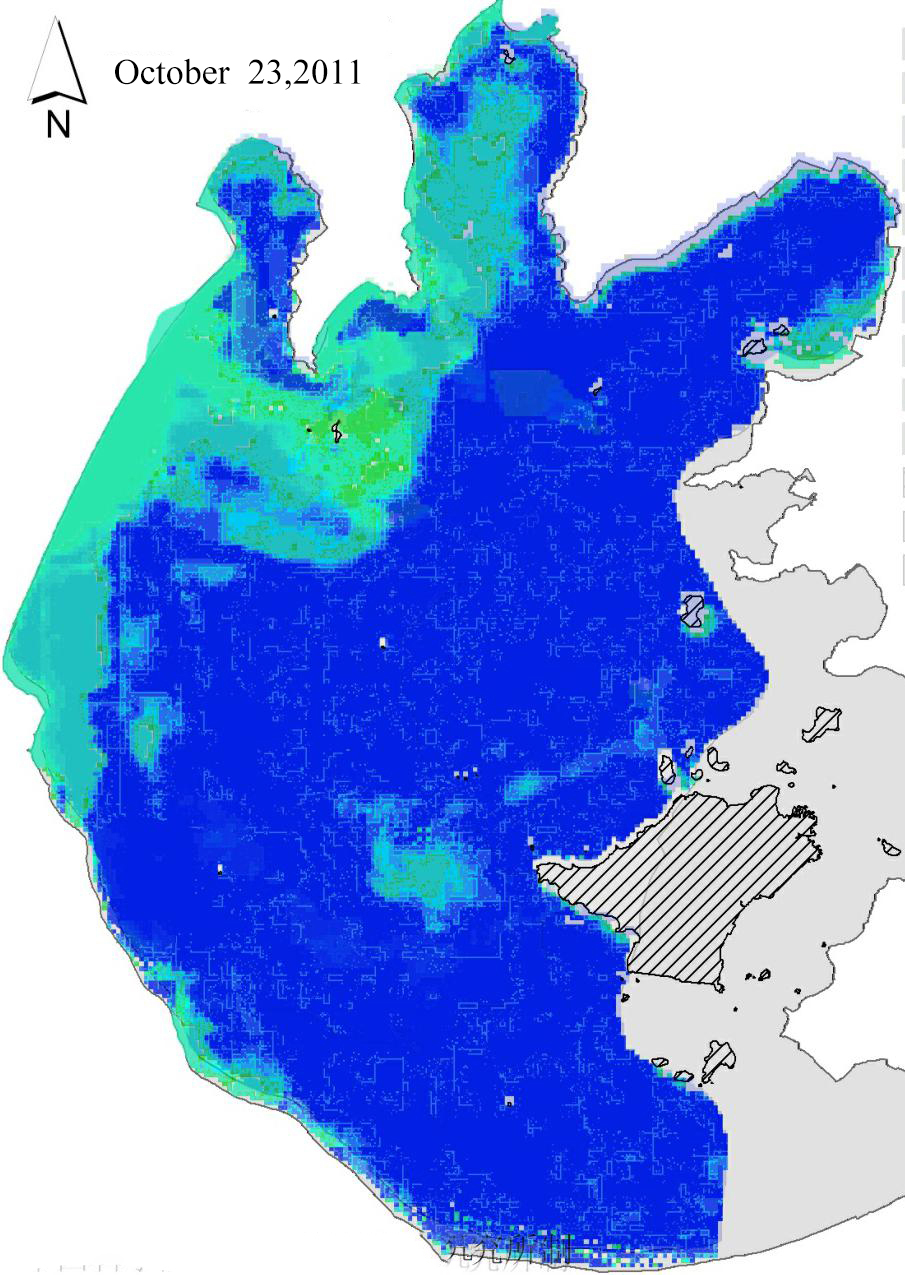

Supplement: Supplemental Information 6 [file peerj-cs-09-1292-s006.zip › 0/20111023_taihu_cla.jpg]

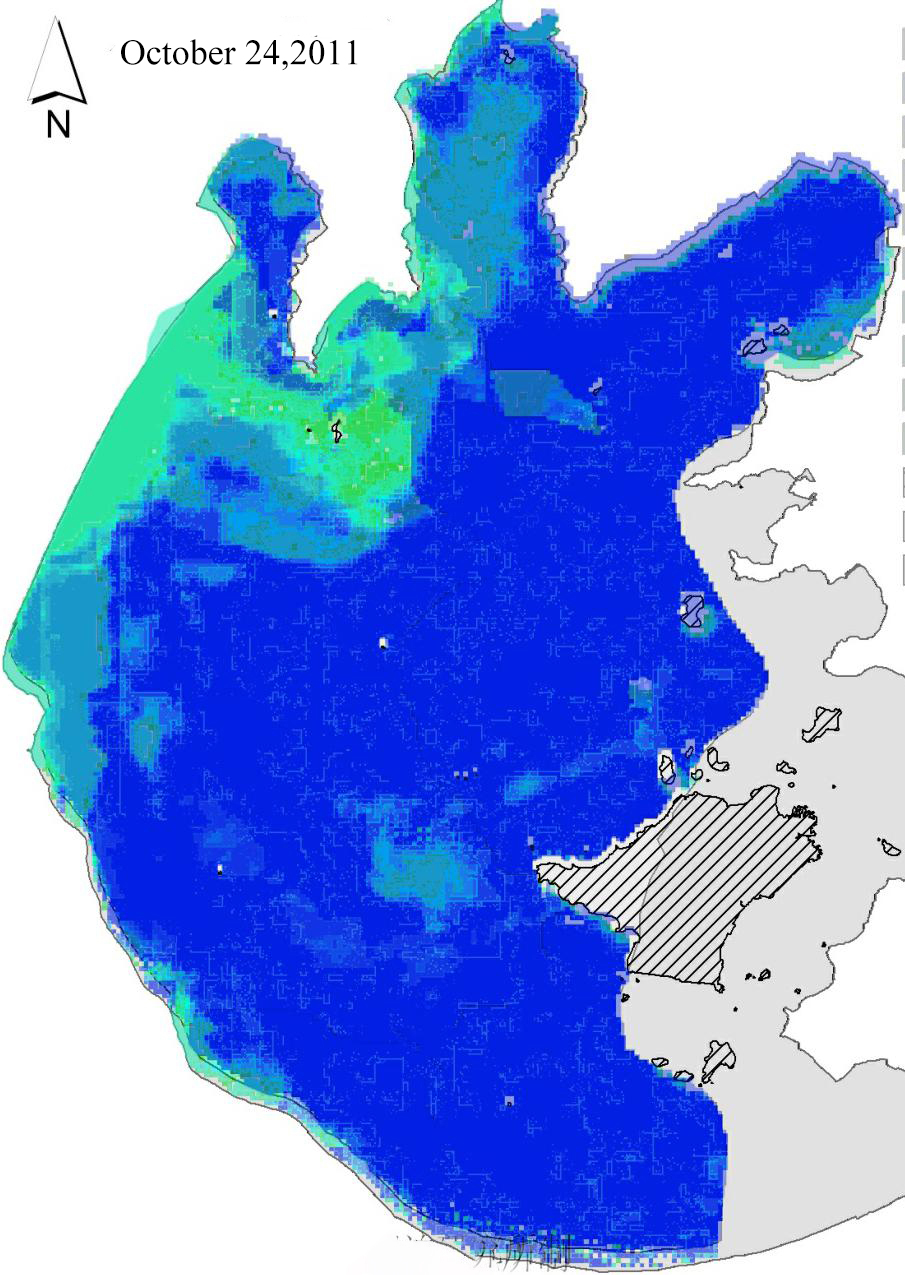

Supplement: Supplemental Information 6 [file peerj-cs-09-1292-s006.zip › 0/20111024_taihu_cla.jpg]

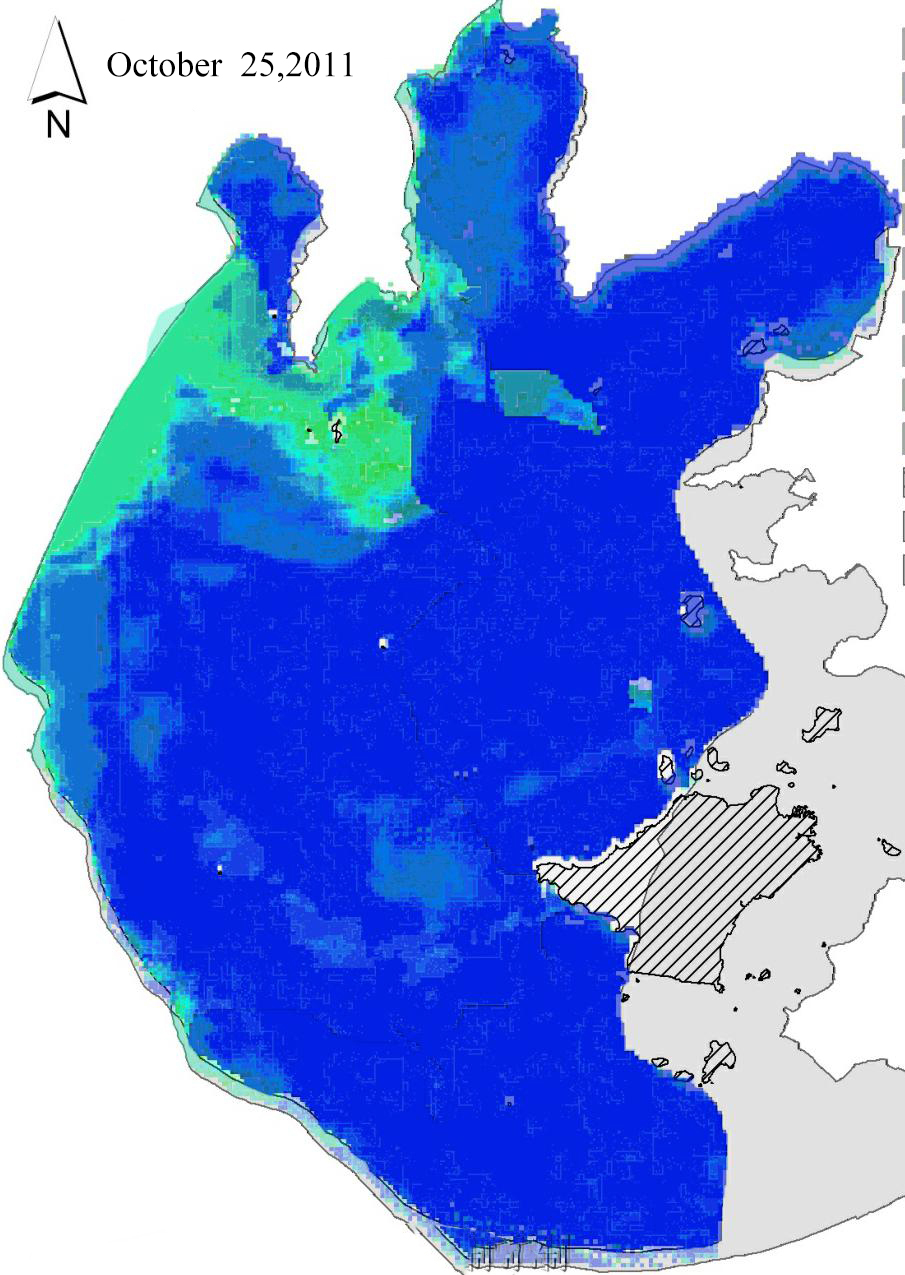

Supplement: Supplemental Information 6 [file peerj-cs-09-1292-s006.zip › 0/20111025_taihu_cla.jpg]

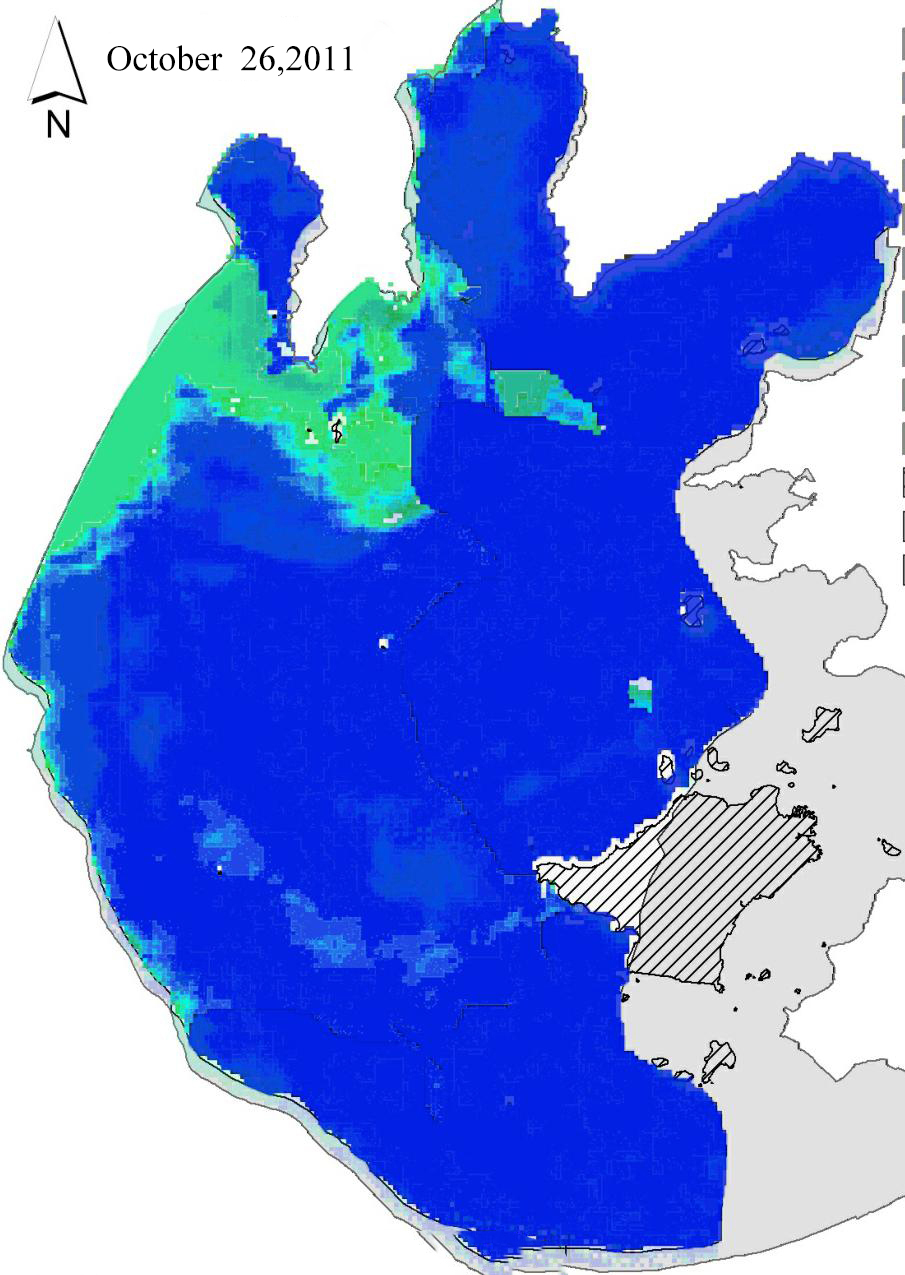

Supplement: Supplemental Information 6 [file peerj-cs-09-1292-s006.zip › 0/20111026_taihu_cla.jpg]

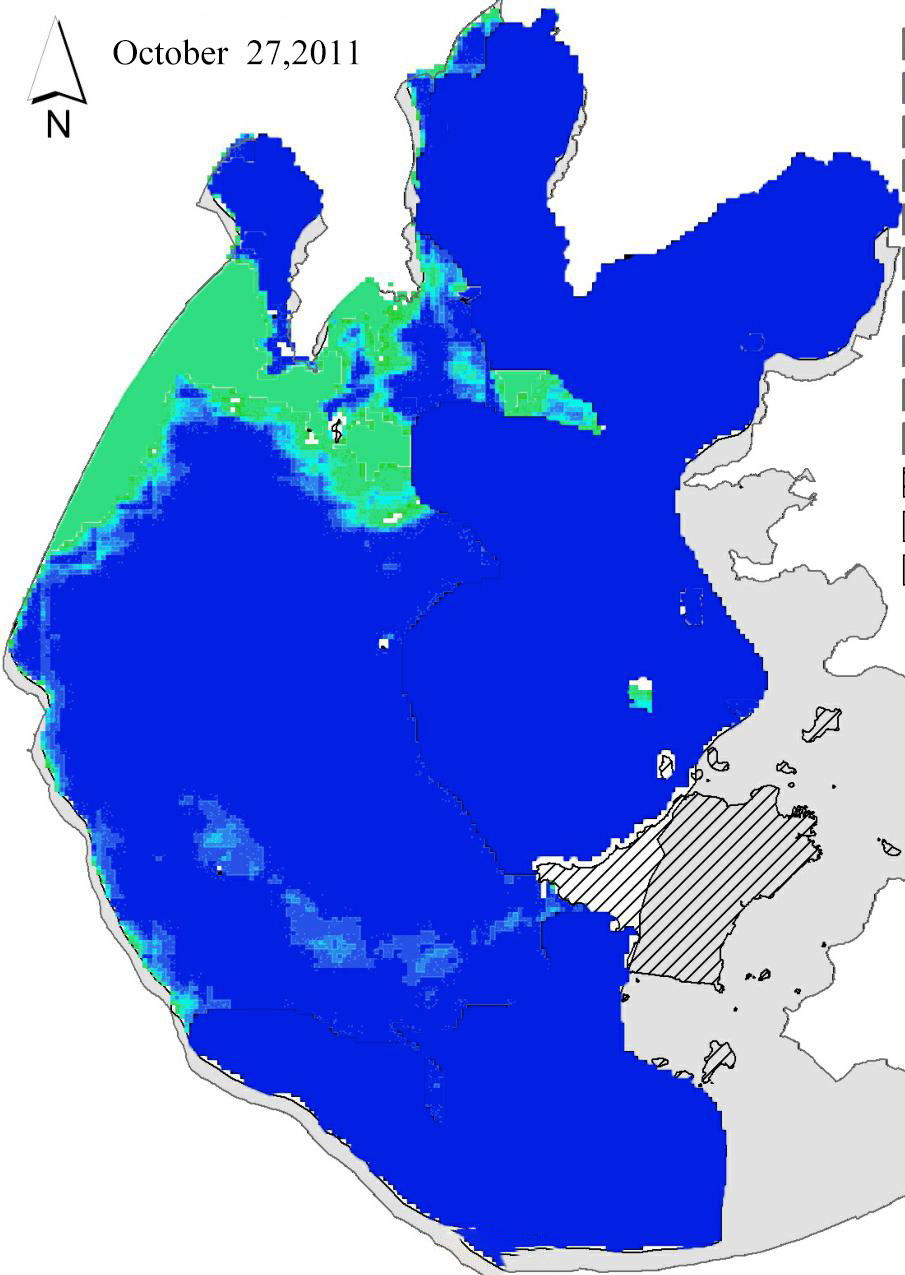

Supplement: Supplemental Information 6 [file peerj-cs-09-1292-s006.zip › 0/20111027_taihu_cla.jpg]

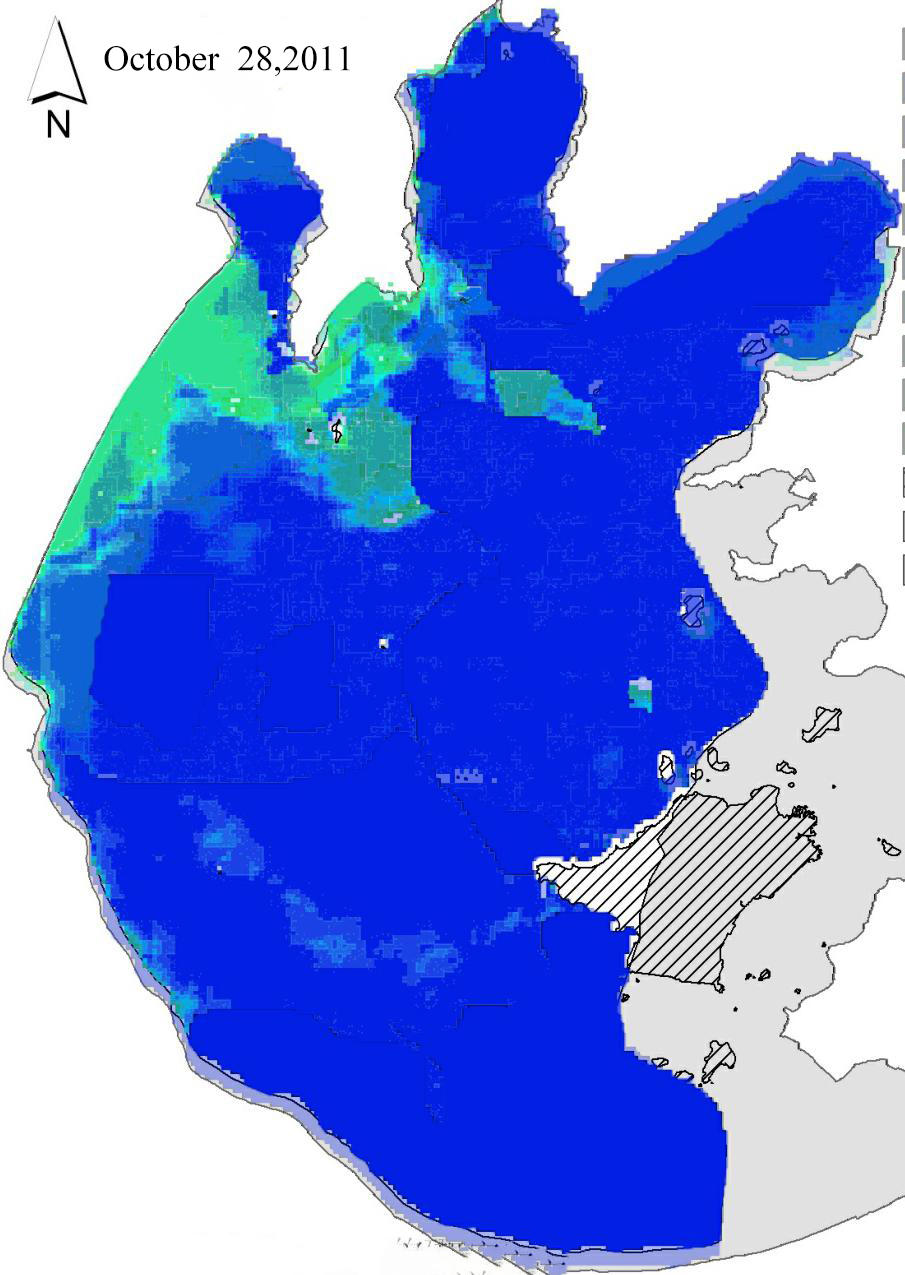

Supplement: Supplemental Information 6 [file peerj-cs-09-1292-s006.zip › 0/20111028_taihu_cla.jpg]
