# Supplementary figures and images for: Remote sensing image analysis and prediction based on improved Pix2Pix model for water environment protection of smart cities (part 3 of 6)
Source: PeerJ Comput Sci. 2023 Apr 26;9:e1292. doi: 10.7717/peerj-cs.1292 (PMC10280440; doi:10.7717/peerj-cs.1292)

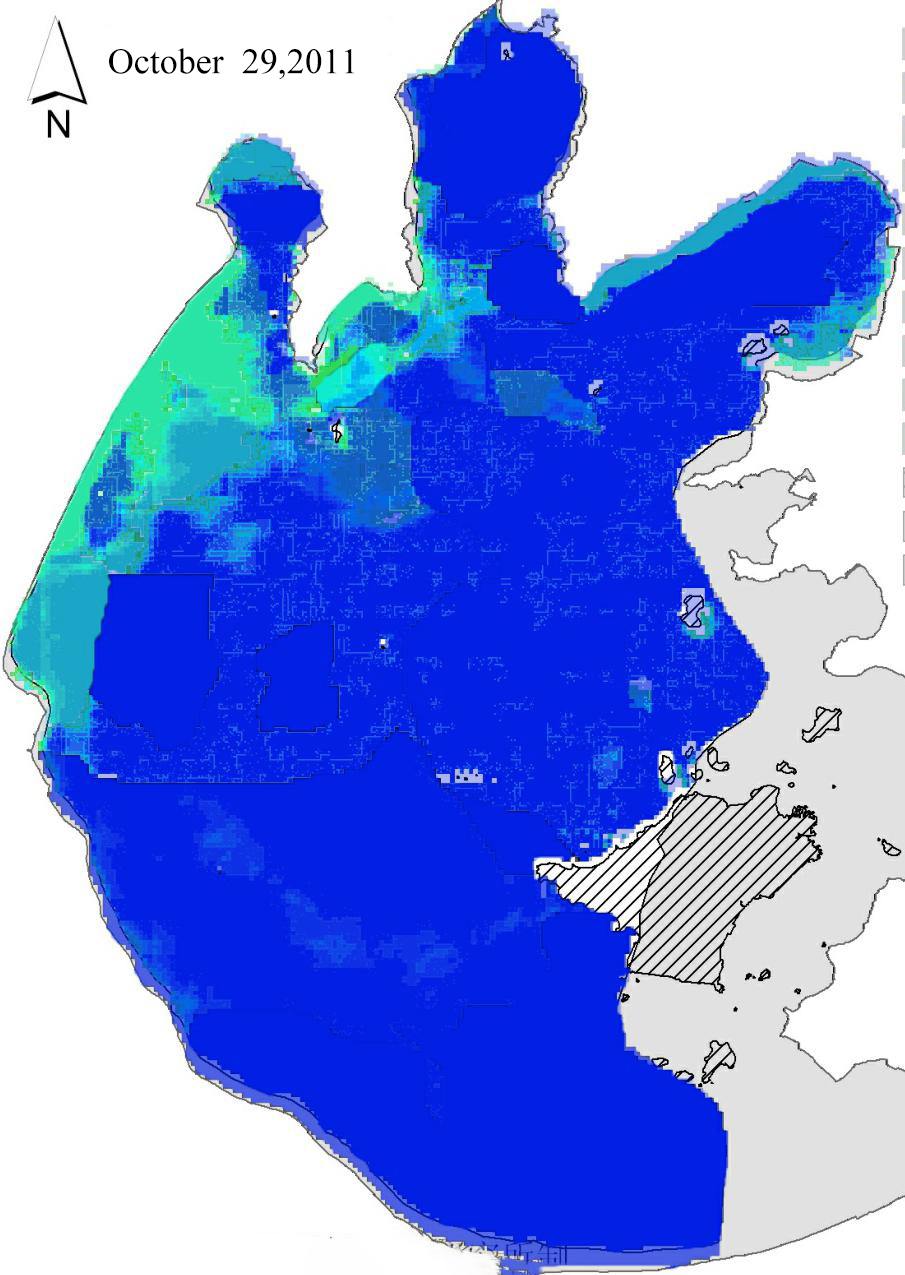

Supplement: Supplemental Information 6 [file peerj-cs-09-1292-s006.zip › 0/20111029_taihu_cla.jpg]

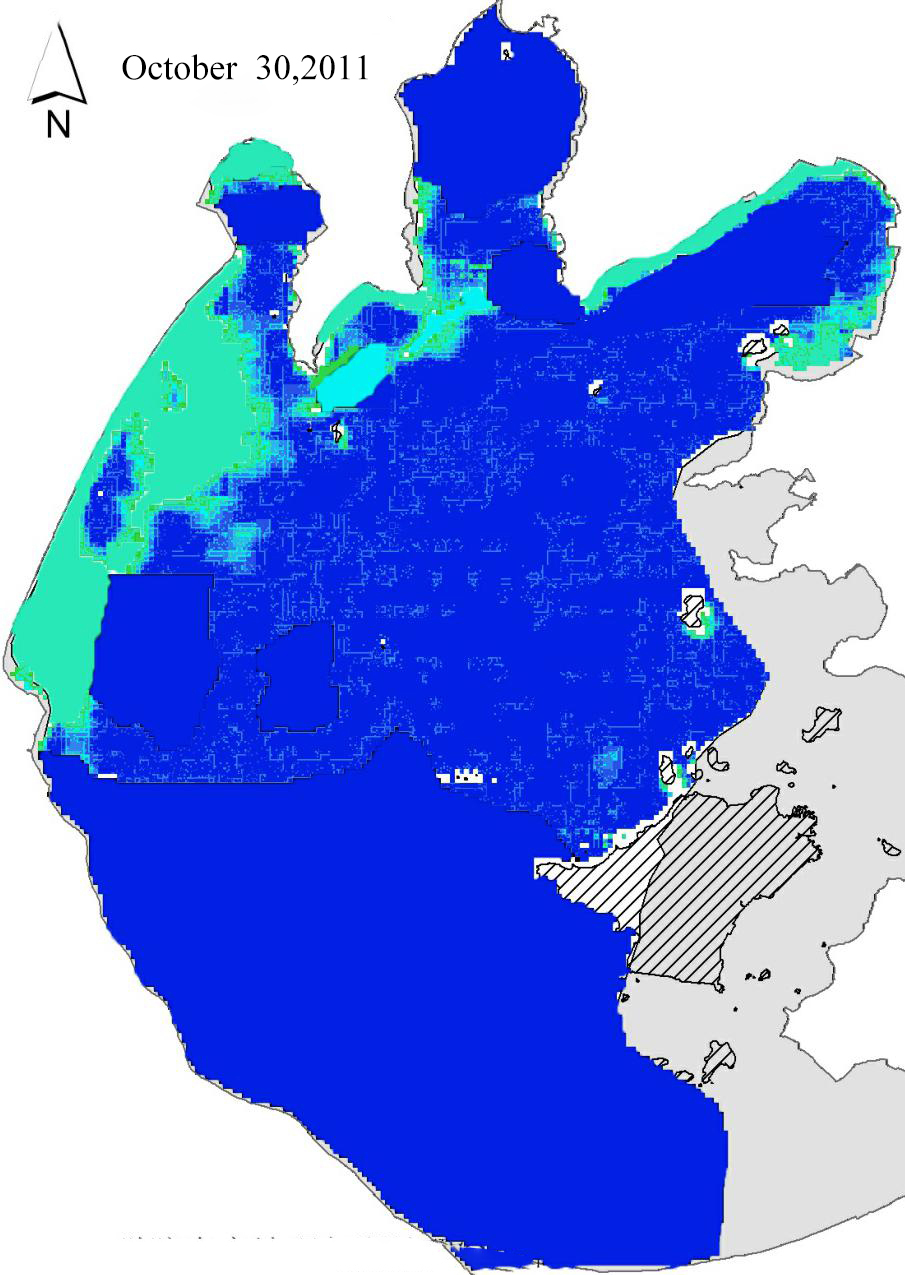

Supplement: Supplemental Information 6 [file peerj-cs-09-1292-s006.zip › 0/20111030_taihu_cla.jpg]

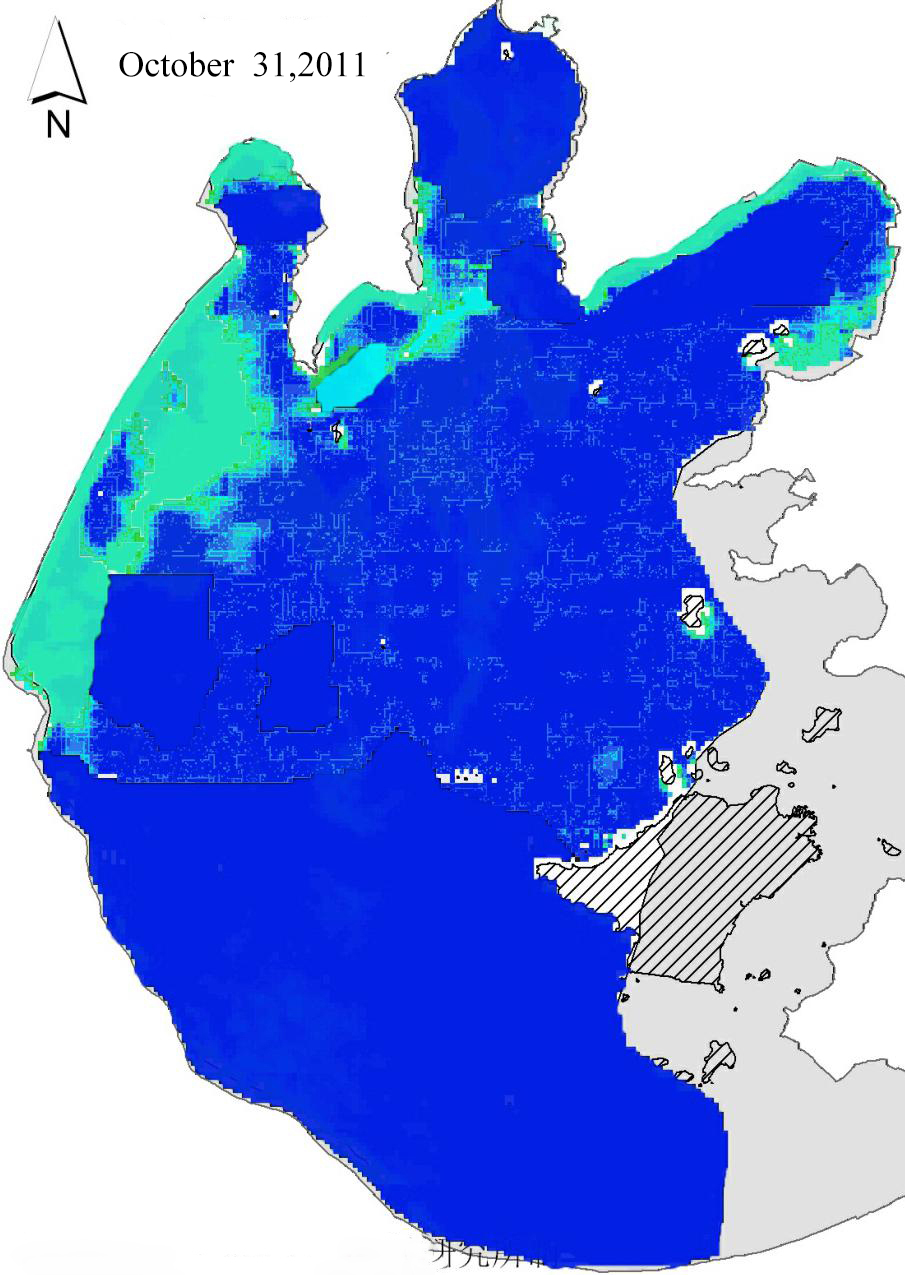

Supplement: Supplemental Information 6 [file peerj-cs-09-1292-s006.zip › 0/20111031_taihu_cla.jpg]

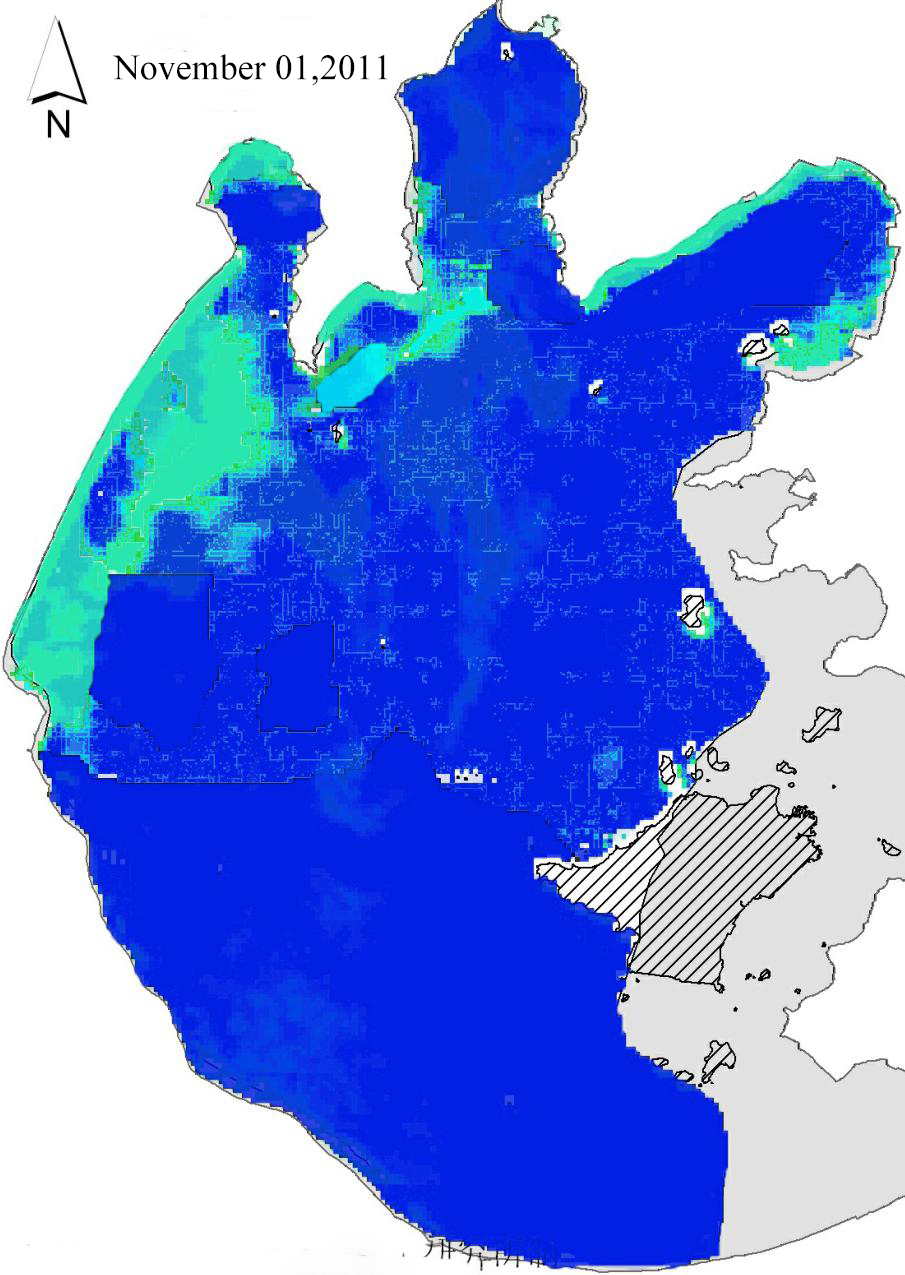

Supplement: Supplemental Information 6 [file peerj-cs-09-1292-s006.zip › 0/20111101_taihu_cla.jpg]

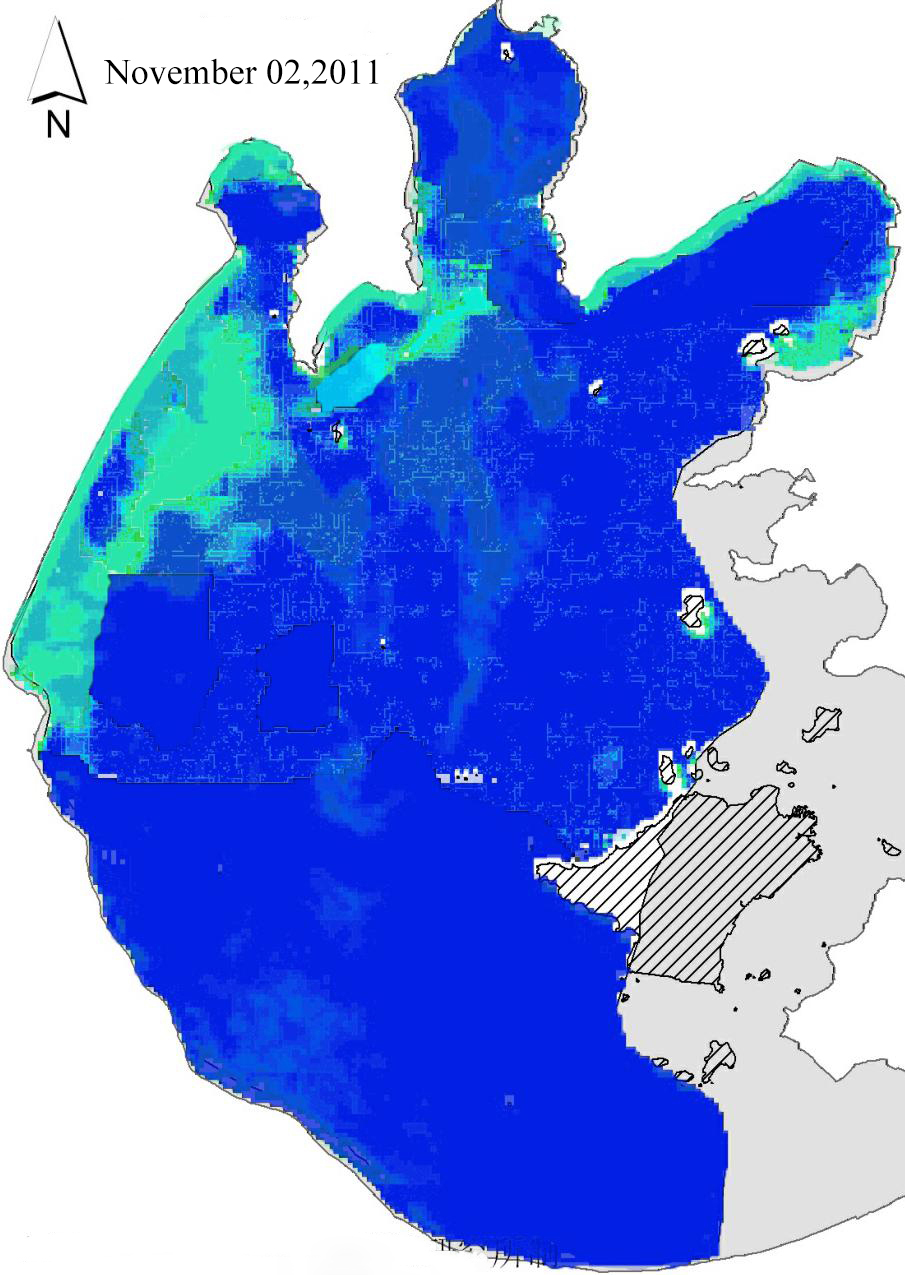

Supplement: Supplemental Information 6 [file peerj-cs-09-1292-s006.zip › 0/20111102_taihu_cla.jpg]

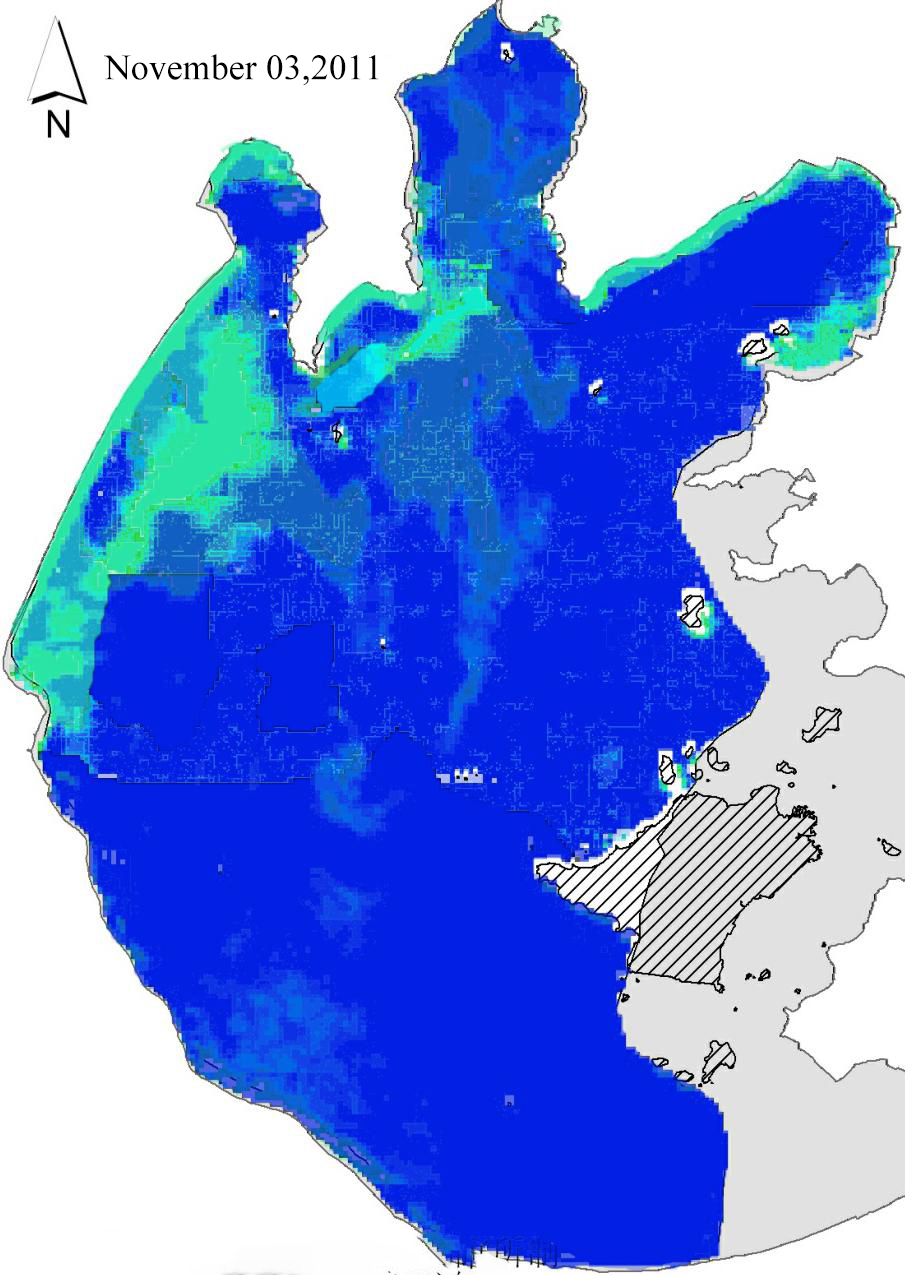

Supplement: Supplemental Information 6 [file peerj-cs-09-1292-s006.zip › 0/20111103_taihu_cla.jpg]

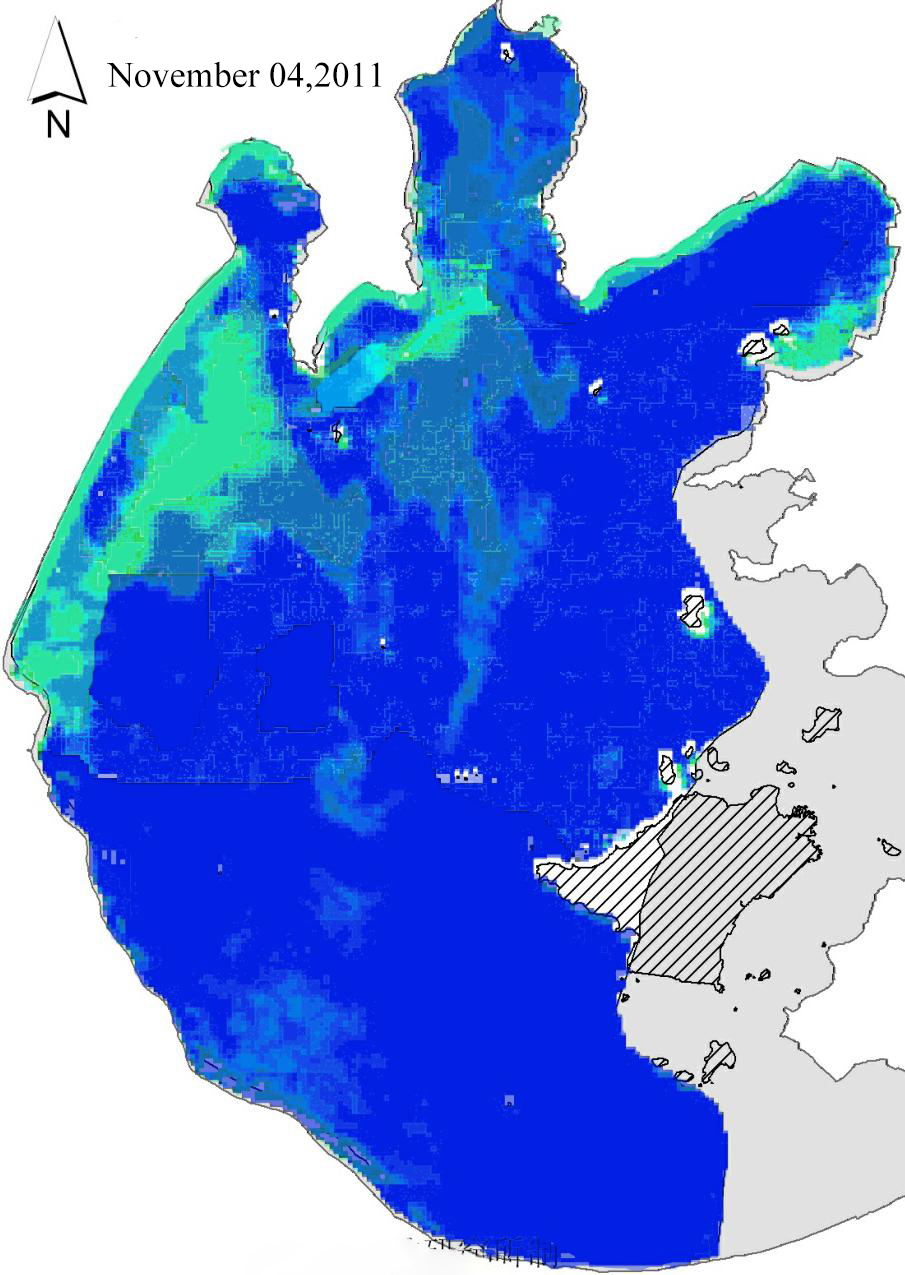

Supplement: Supplemental Information 6 [file peerj-cs-09-1292-s006.zip › 0/20111104_taihu_cla.jpg]

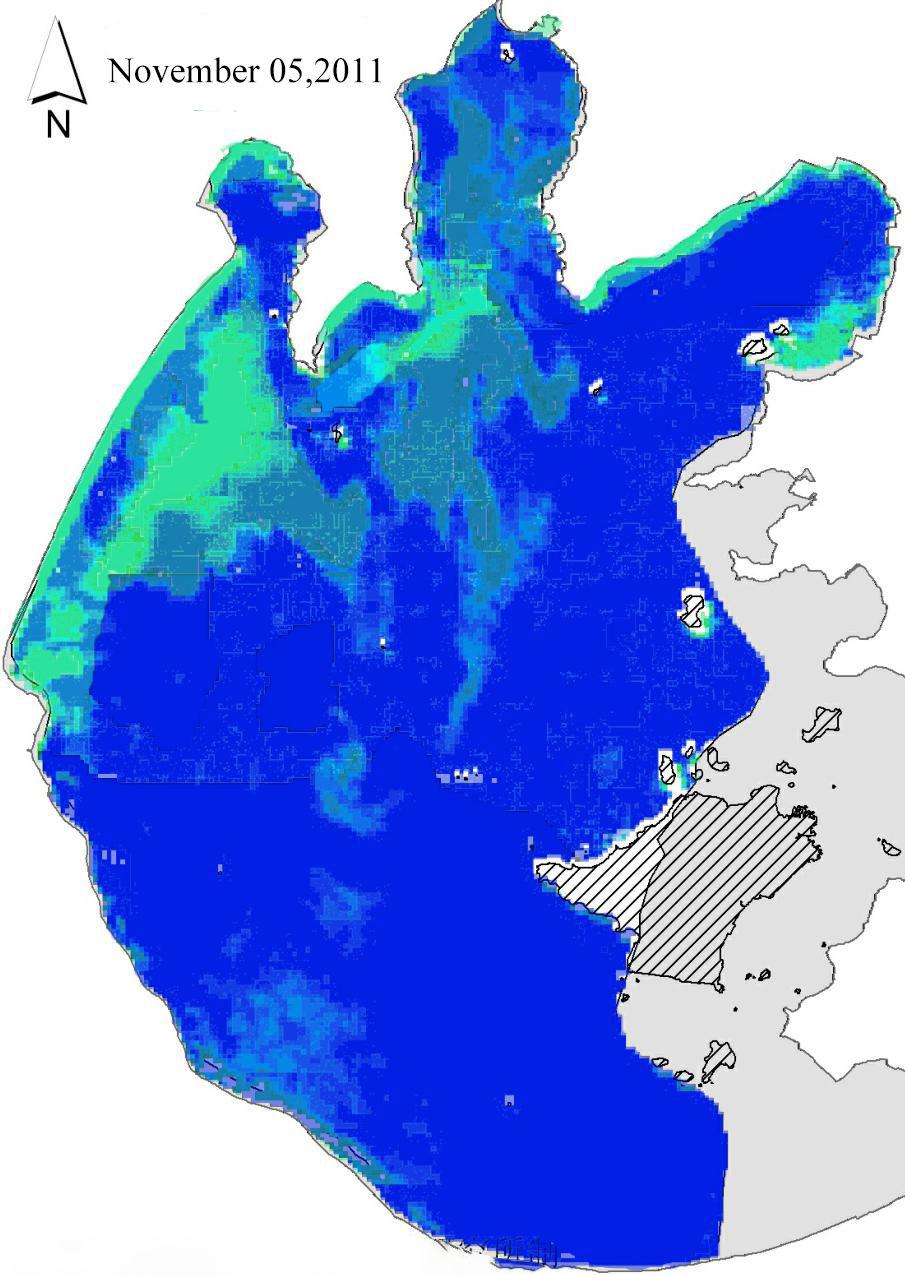

Supplement: Supplemental Information 6 [file peerj-cs-09-1292-s006.zip › 0/20111105_taihu_cla.jpg]

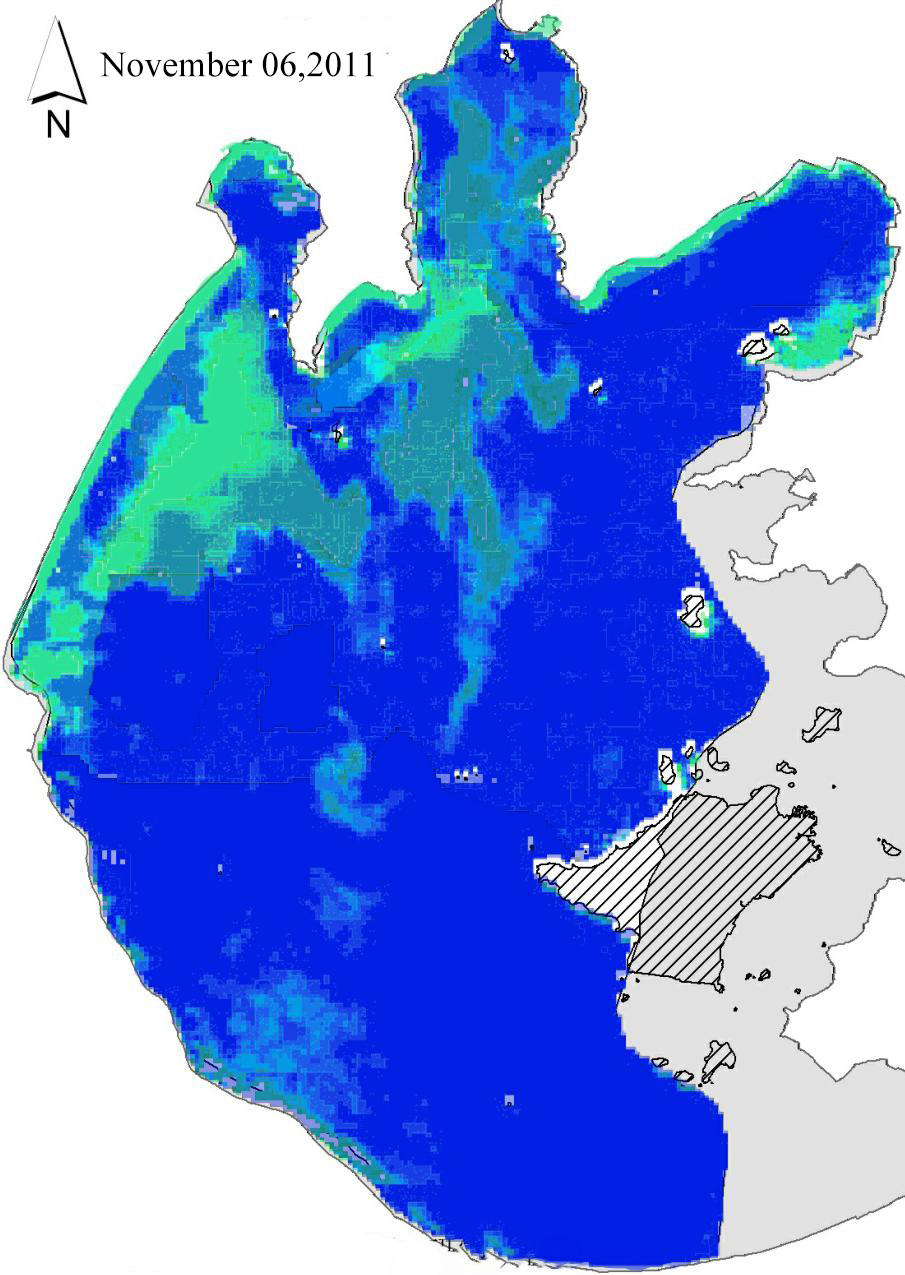

Supplement: Supplemental Information 6 [file peerj-cs-09-1292-s006.zip › 0/20111106_taihu_cla.jpg]

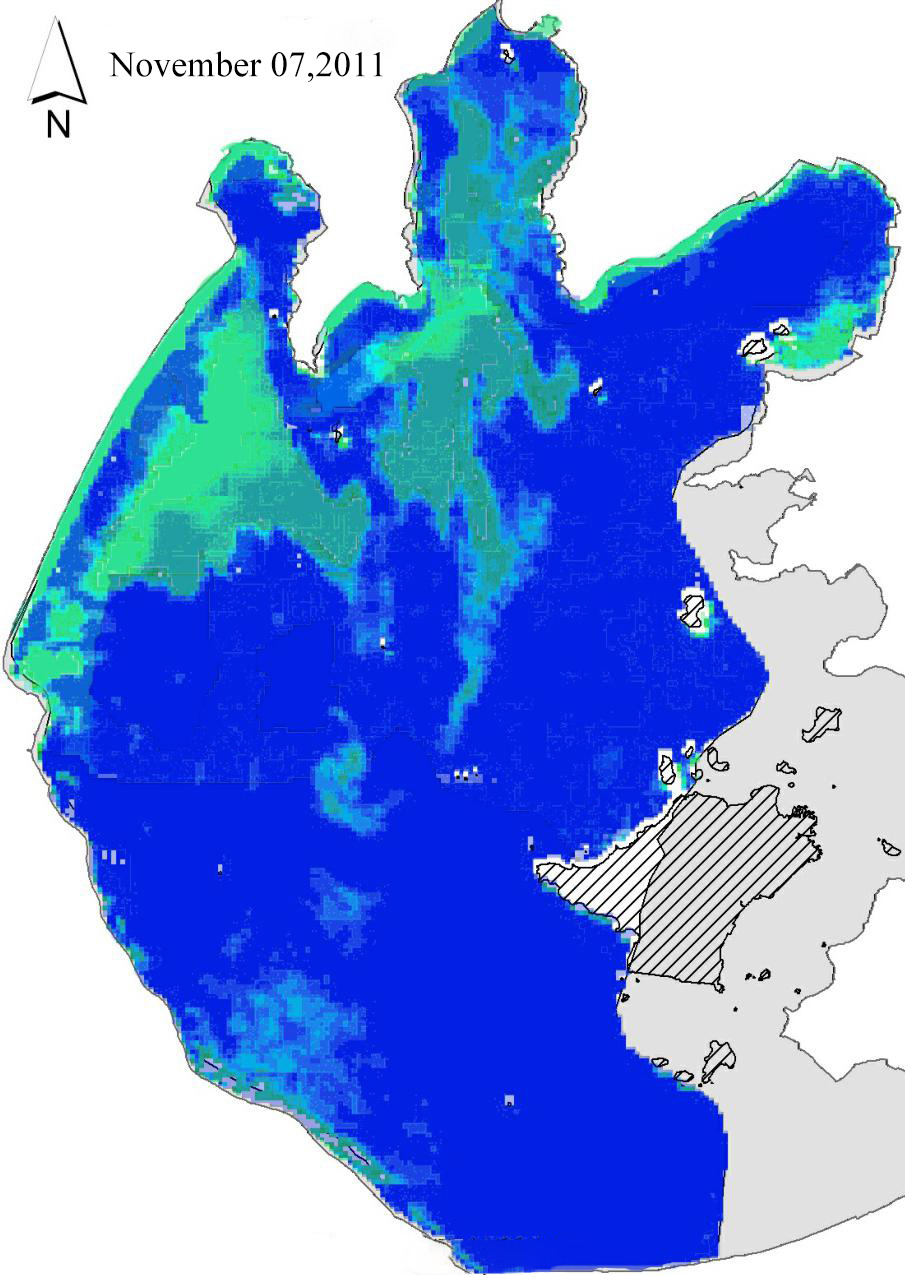

Supplement: Supplemental Information 6 [file peerj-cs-09-1292-s006.zip › 0/20111107_taihu_cla.jpg]

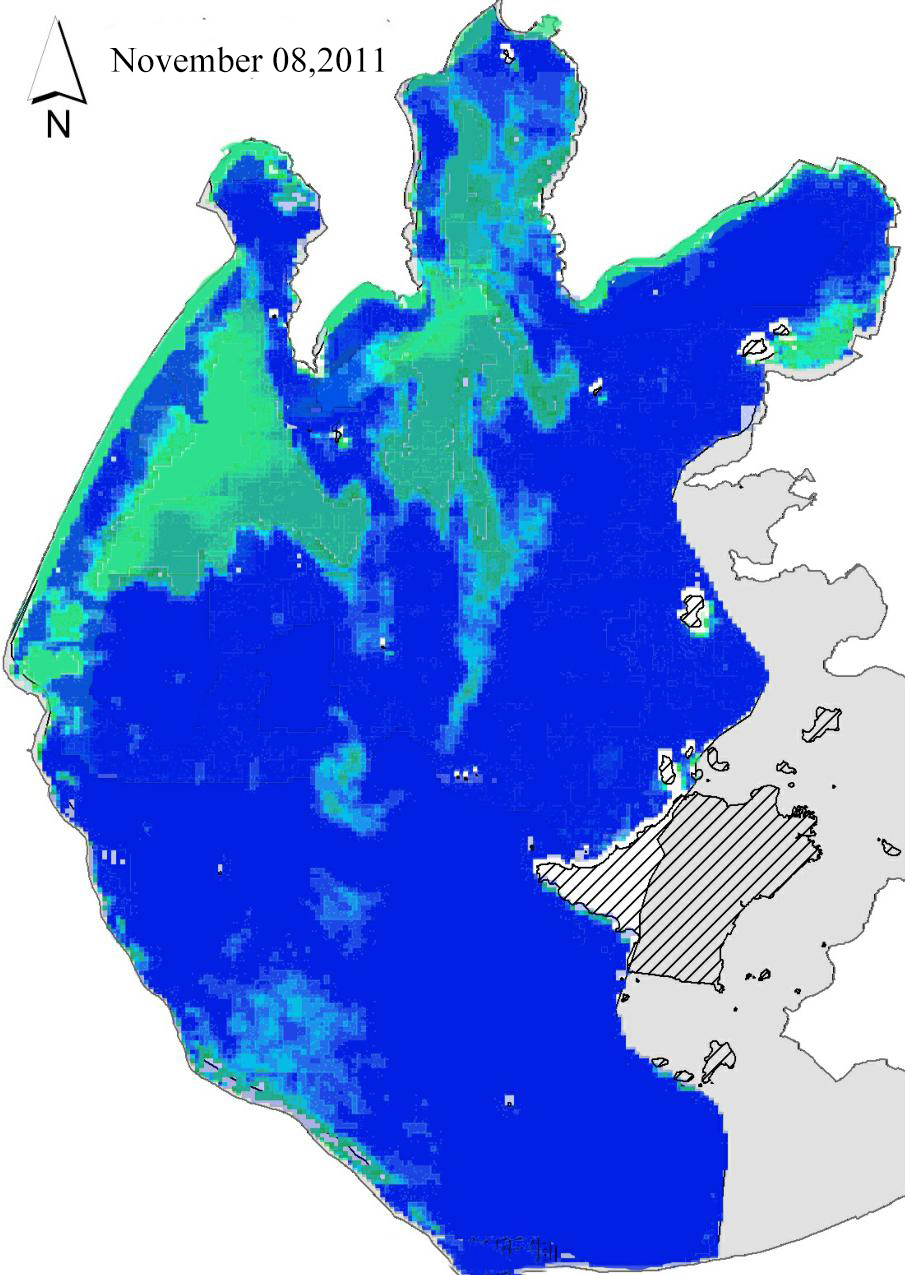

Supplement: Supplemental Information 6 [file peerj-cs-09-1292-s006.zip › 0/20111108_taihu_cla.jpg]

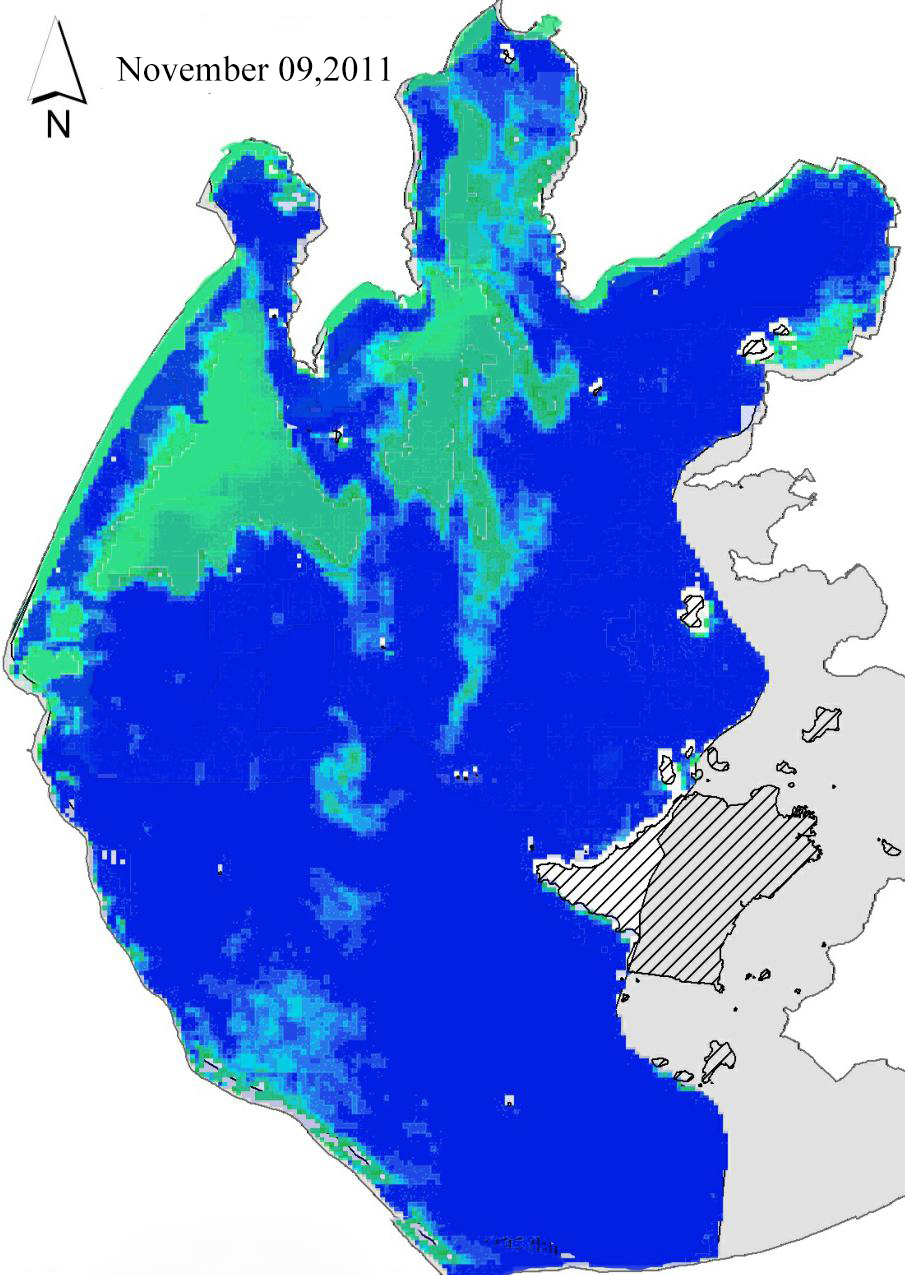

Supplement: Supplemental Information 6 [file peerj-cs-09-1292-s006.zip › 0/20111109_taihu_cla.jpg]

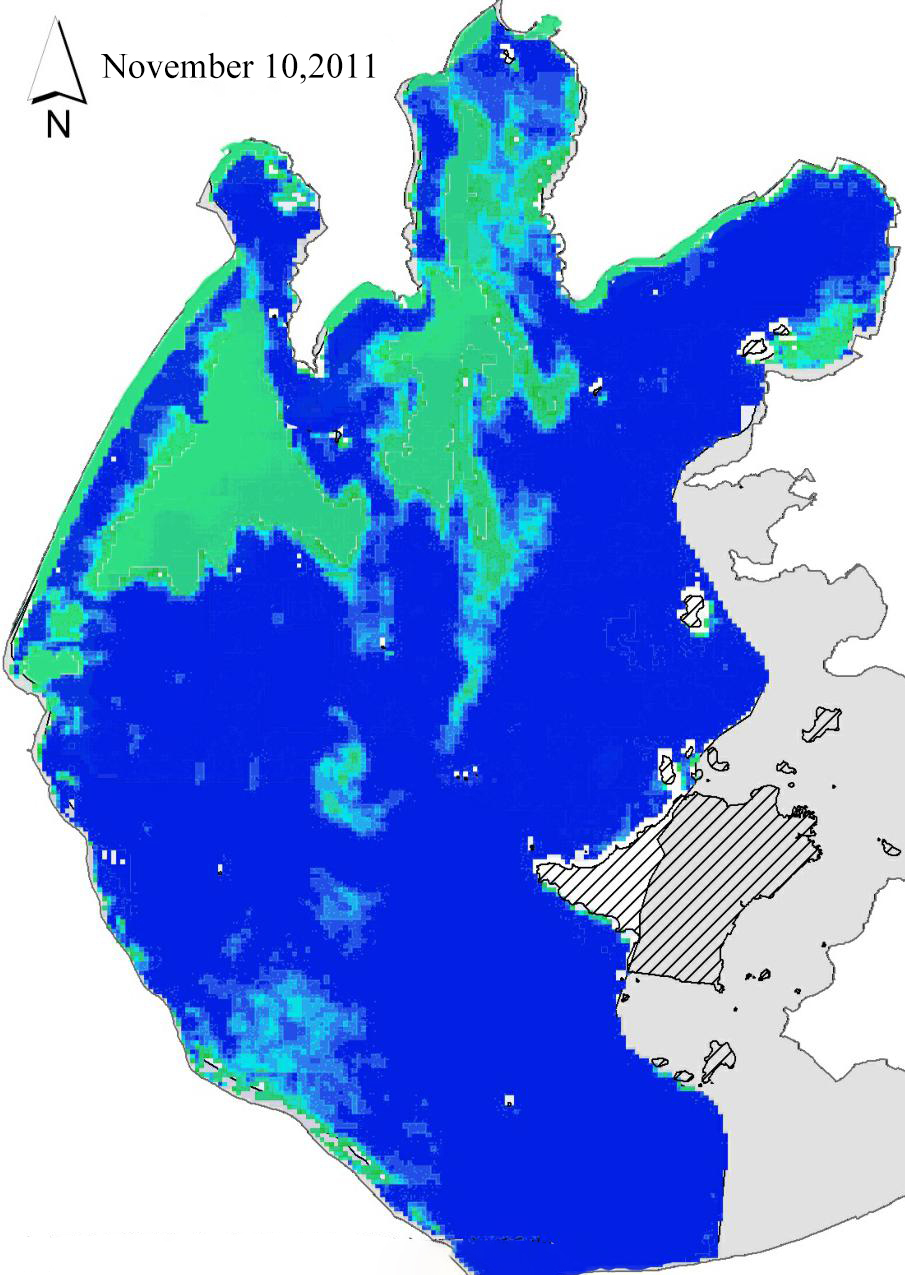

Supplement: Supplemental Information 6 [file peerj-cs-09-1292-s006.zip › 0/20111110_taihu_cla.jpg]

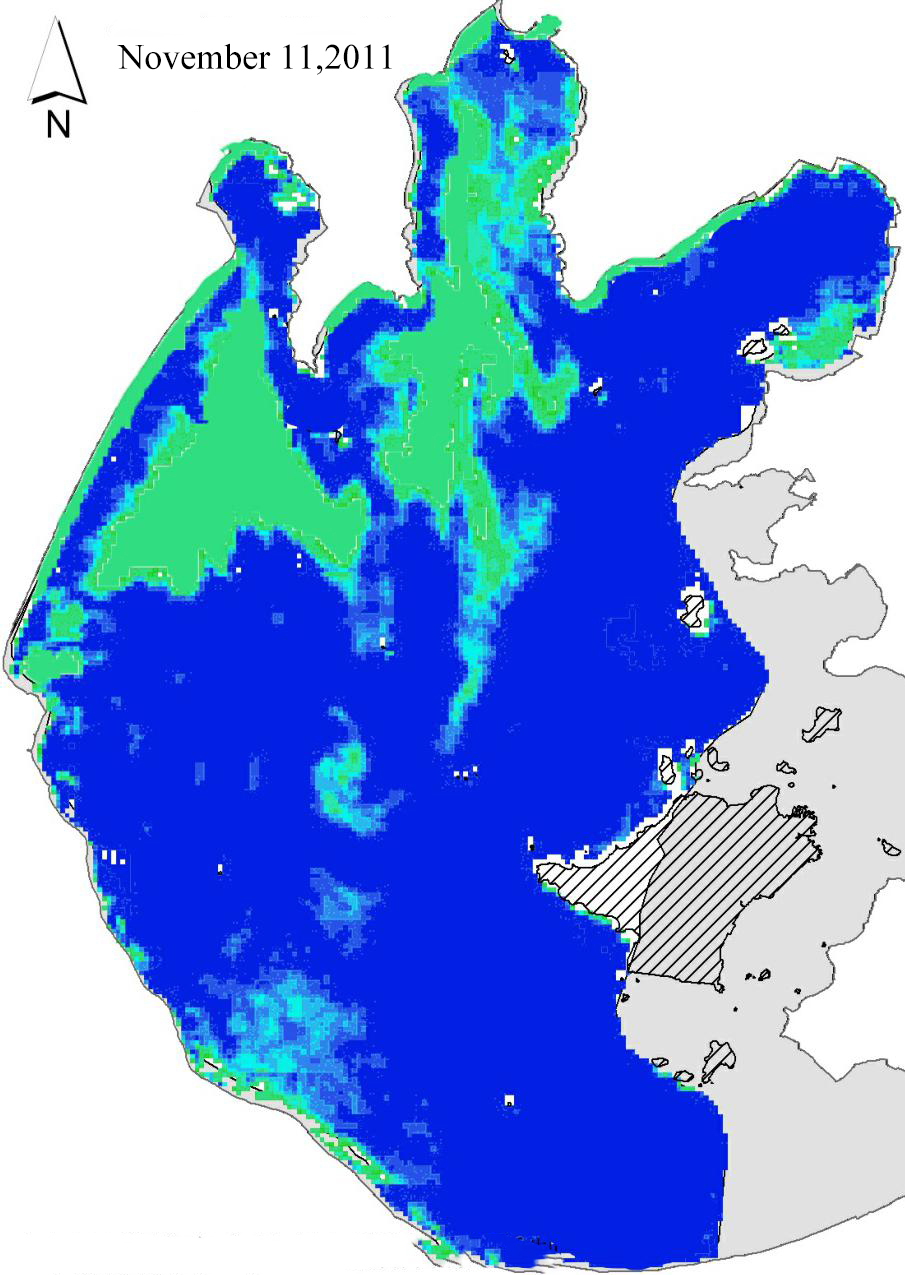

Supplement: Supplemental Information 6 [file peerj-cs-09-1292-s006.zip › 0/20111111_taihu_chla.jpg]

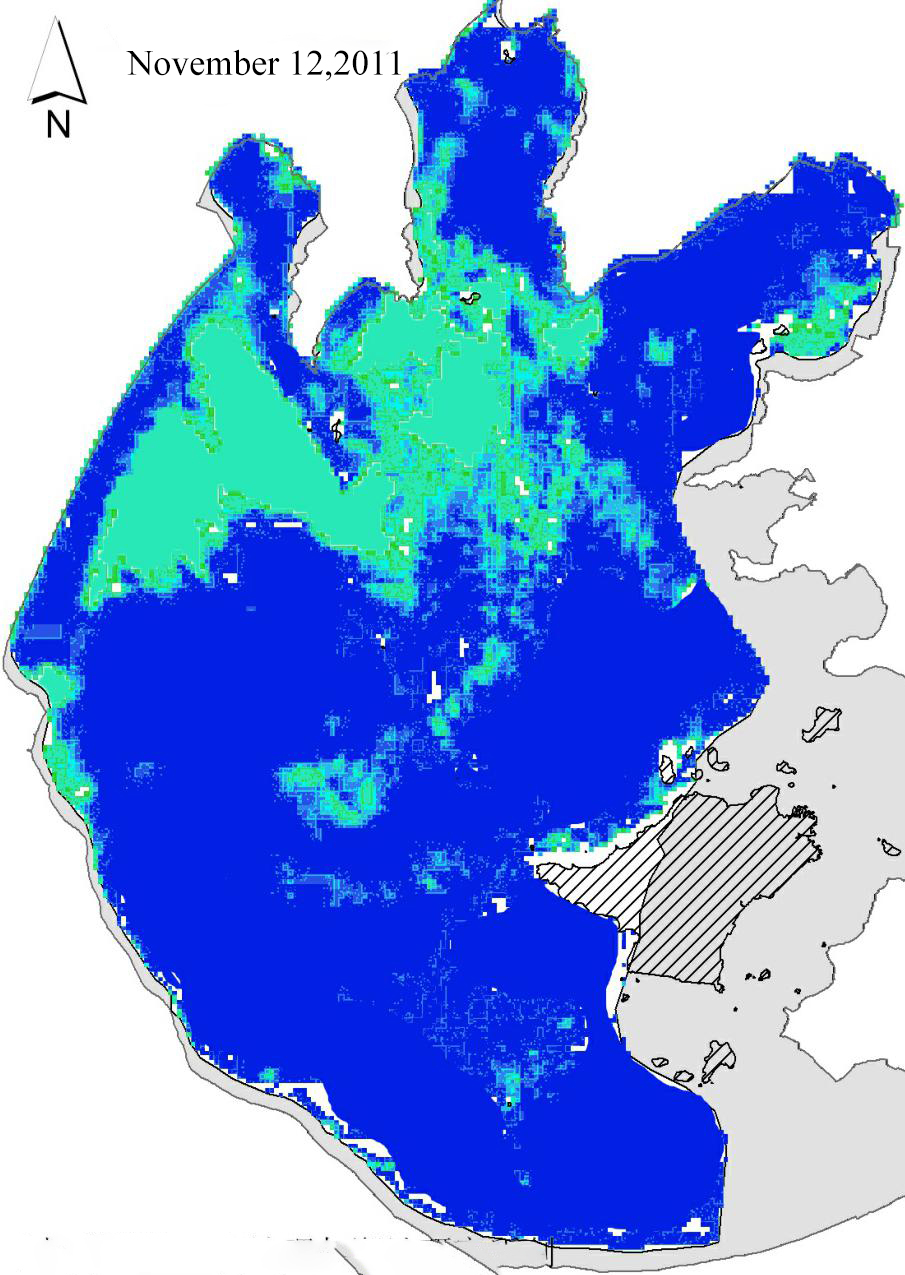

Supplement: Supplemental Information 6 [file peerj-cs-09-1292-s006.zip › 0/20111112_taihu_cla.jpg]

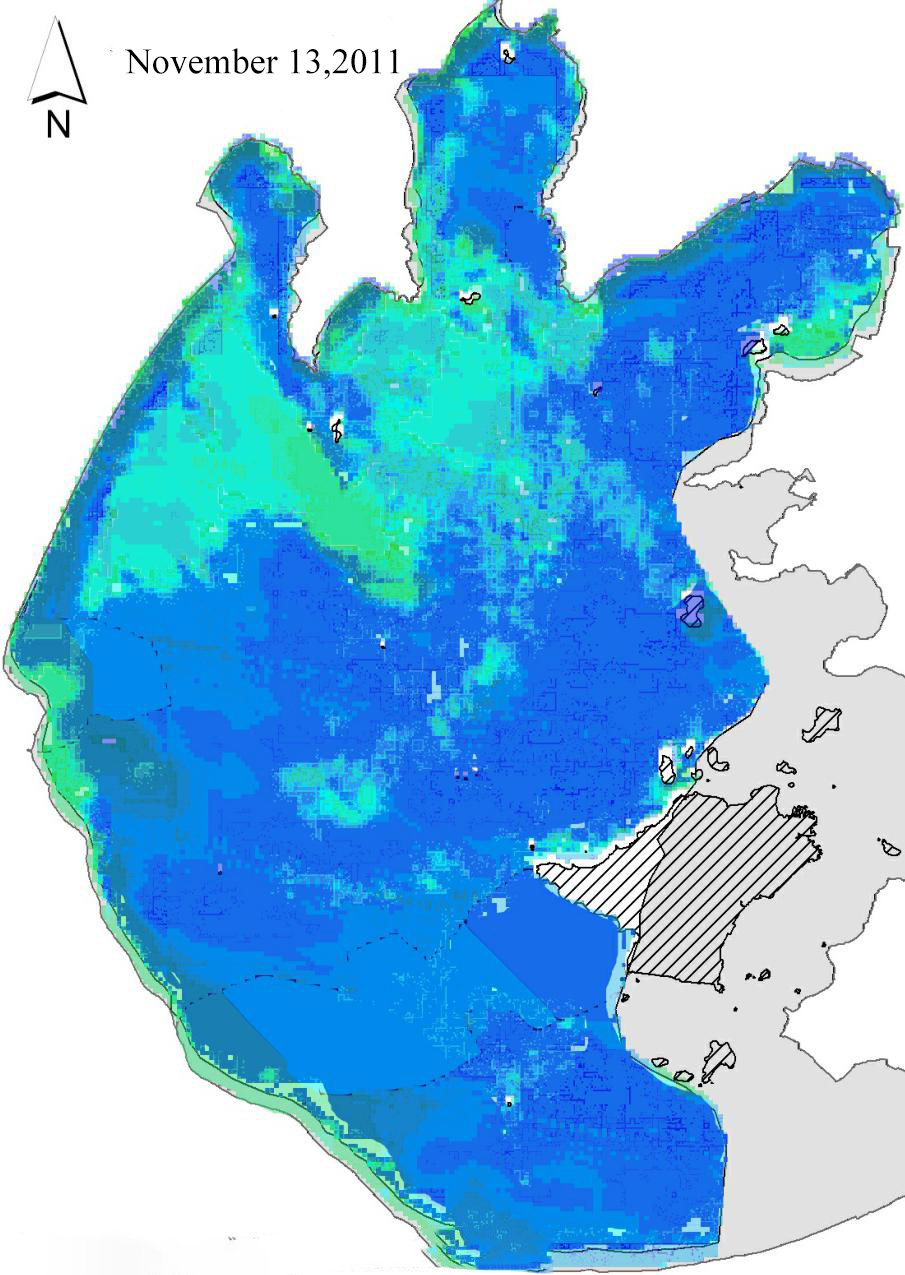

Supplement: Supplemental Information 6 [file peerj-cs-09-1292-s006.zip › 0/20111113_taihu_cla.jpg]

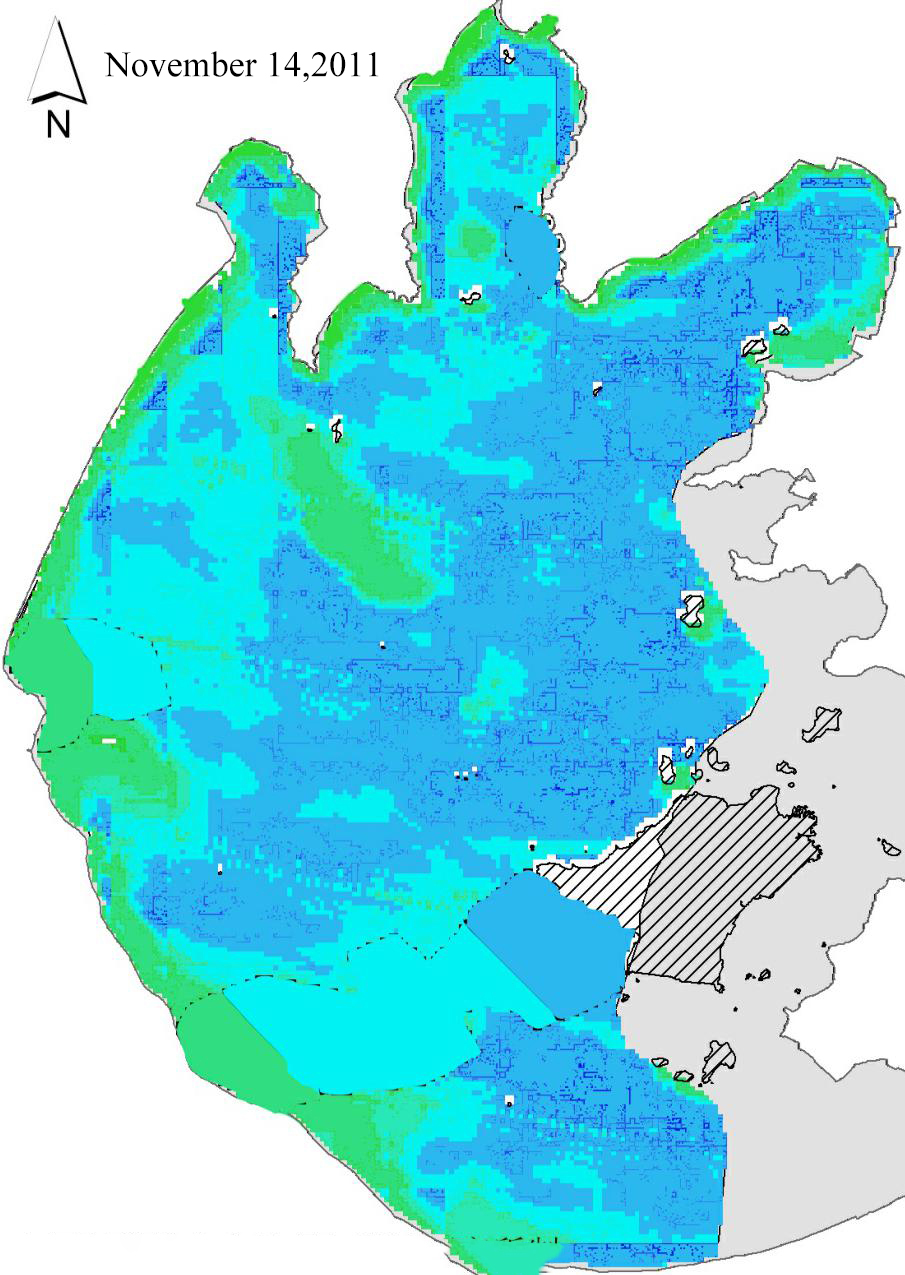

Supplement: Supplemental Information 6 [file peerj-cs-09-1292-s006.zip › 0/20111114_taihu_cla.jpg]

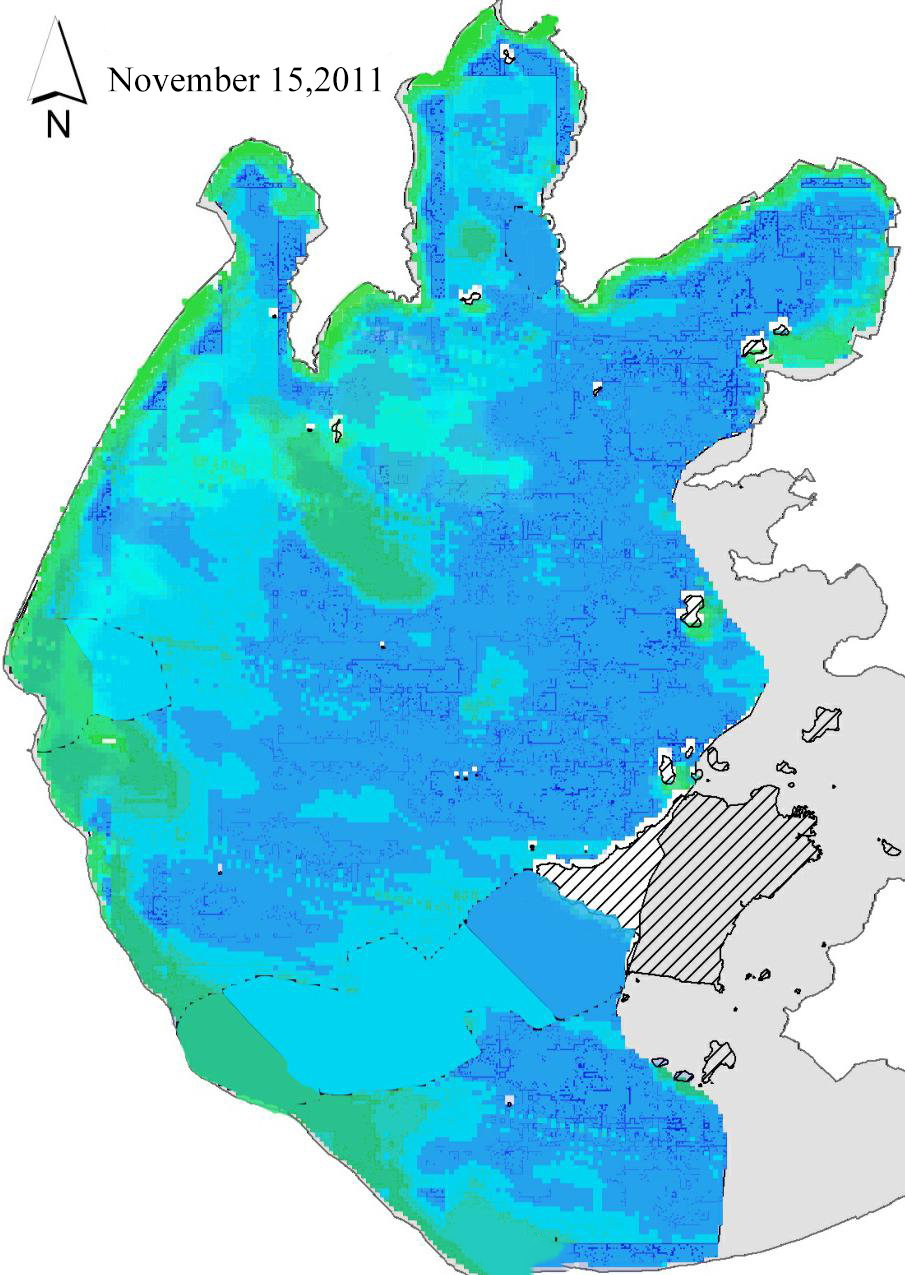

Supplement: Supplemental Information 6 [file peerj-cs-09-1292-s006.zip › 0/20111115_taihu_cla.jpg]

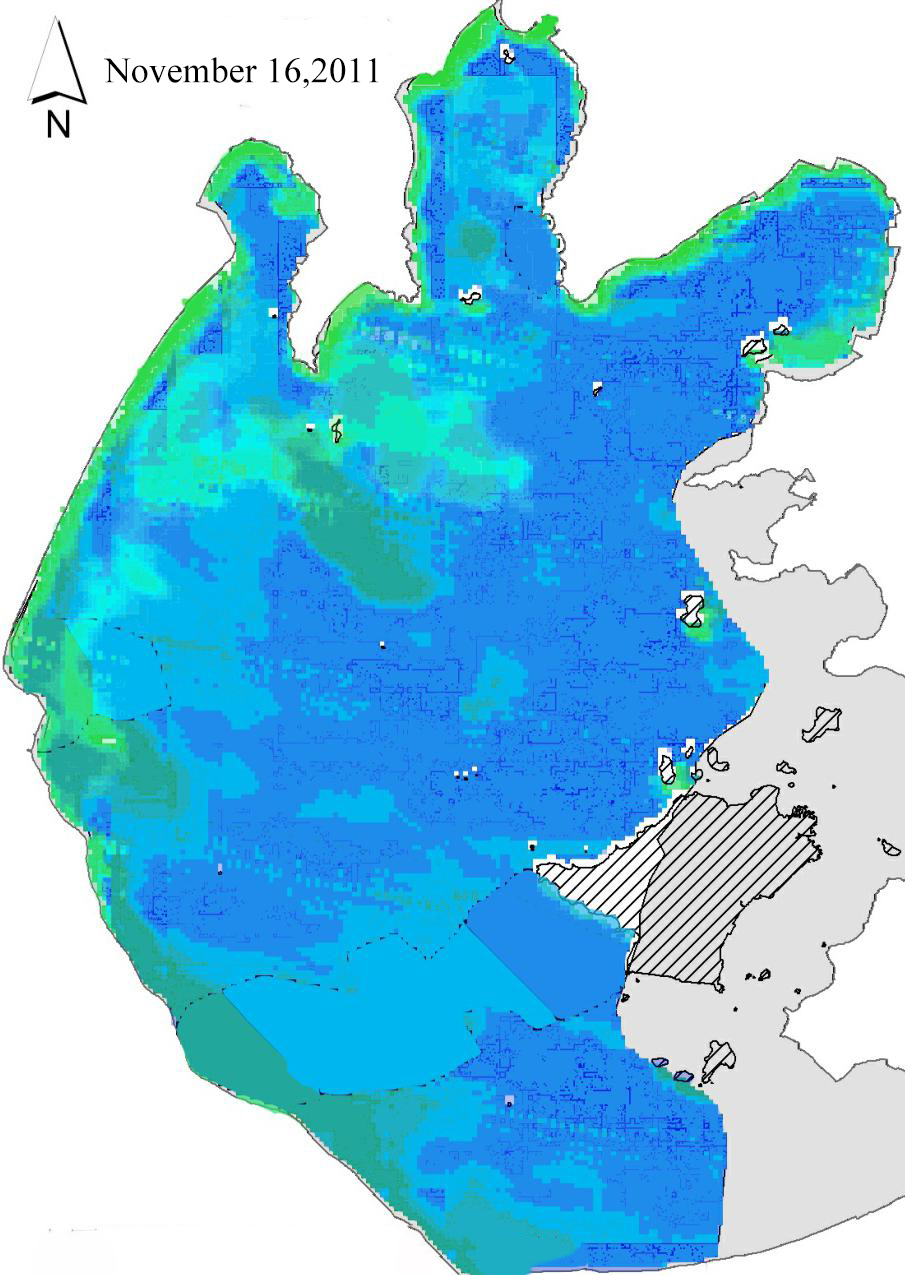

Supplement: Supplemental Information 6 [file peerj-cs-09-1292-s006.zip › 0/20111116_taihu_cla.jpg]

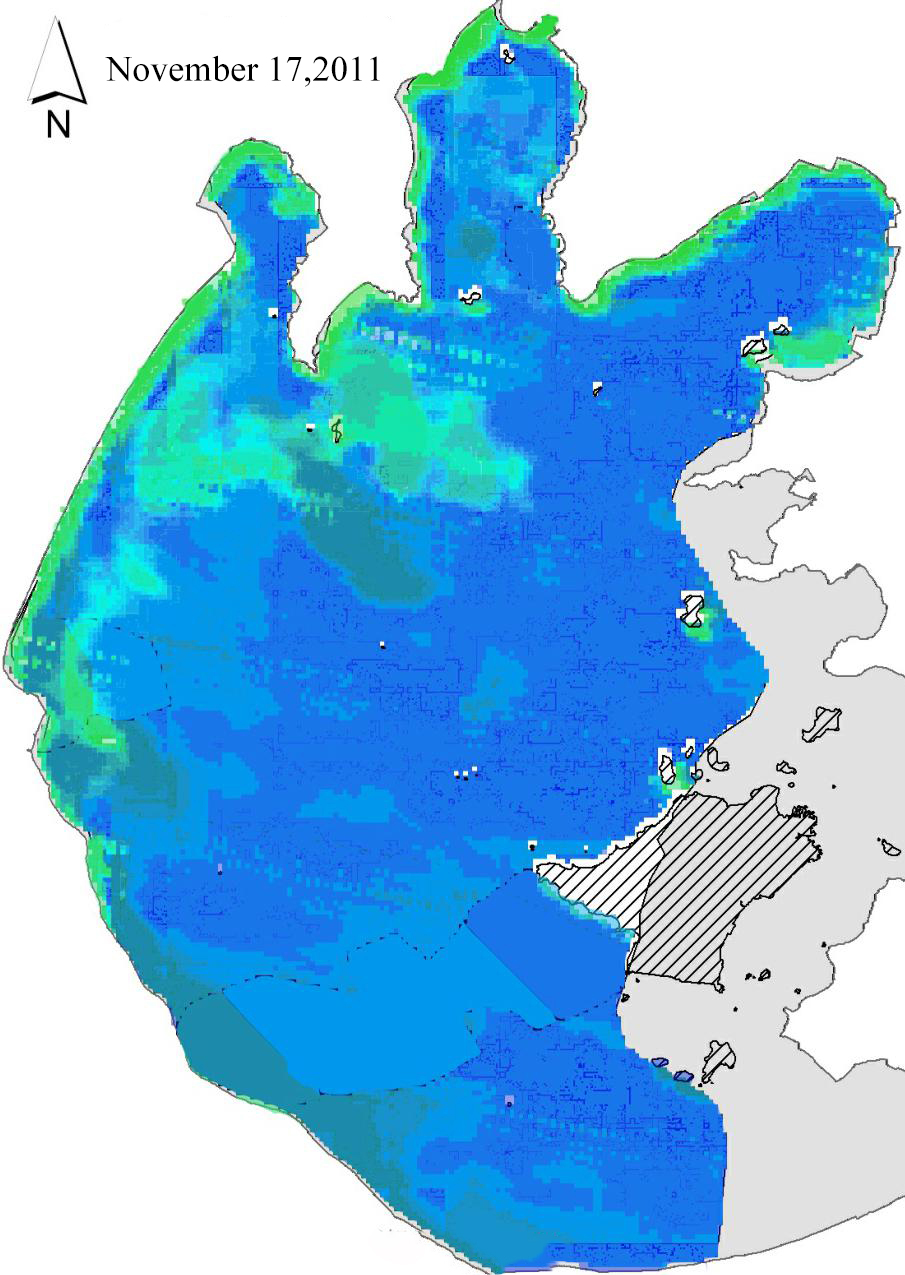

Supplement: Supplemental Information 6 [file peerj-cs-09-1292-s006.zip › 0/20111117_taihu_cla.jpg]

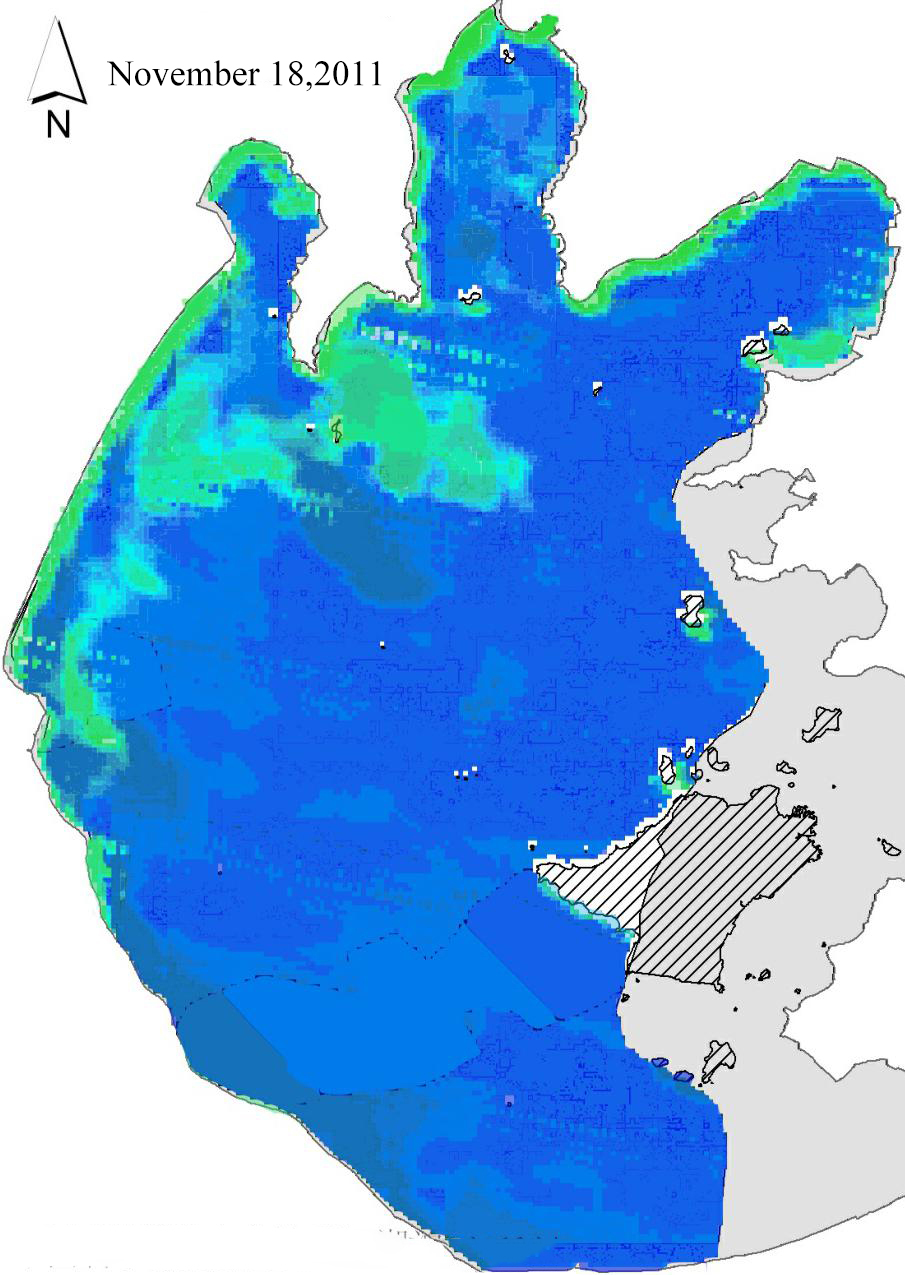

Supplement: Supplemental Information 6 [file peerj-cs-09-1292-s006.zip › 0/20111118_taihu_cla.jpg]

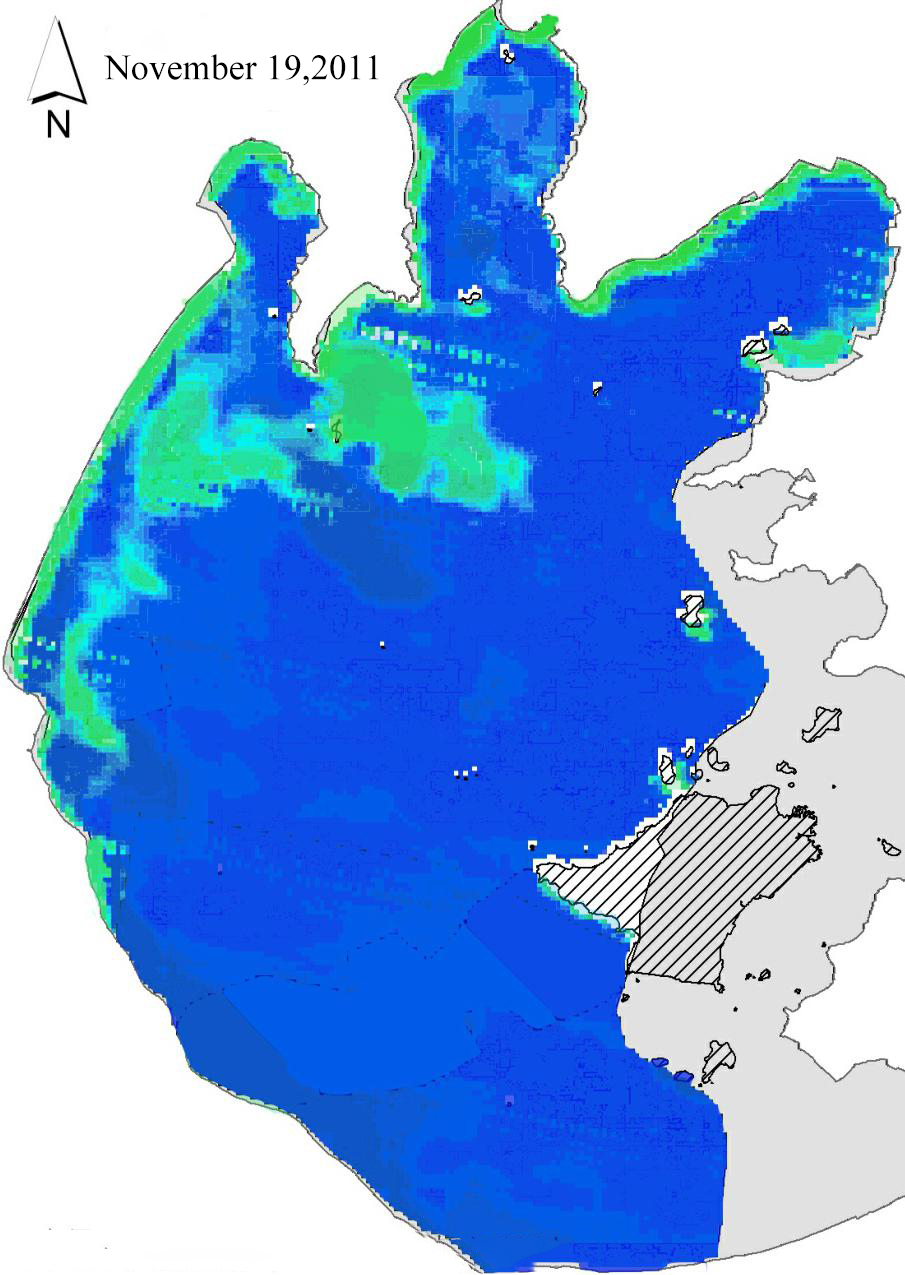

Supplement: Supplemental Information 6 [file peerj-cs-09-1292-s006.zip › 0/20111119_taihu_cla.jpg]

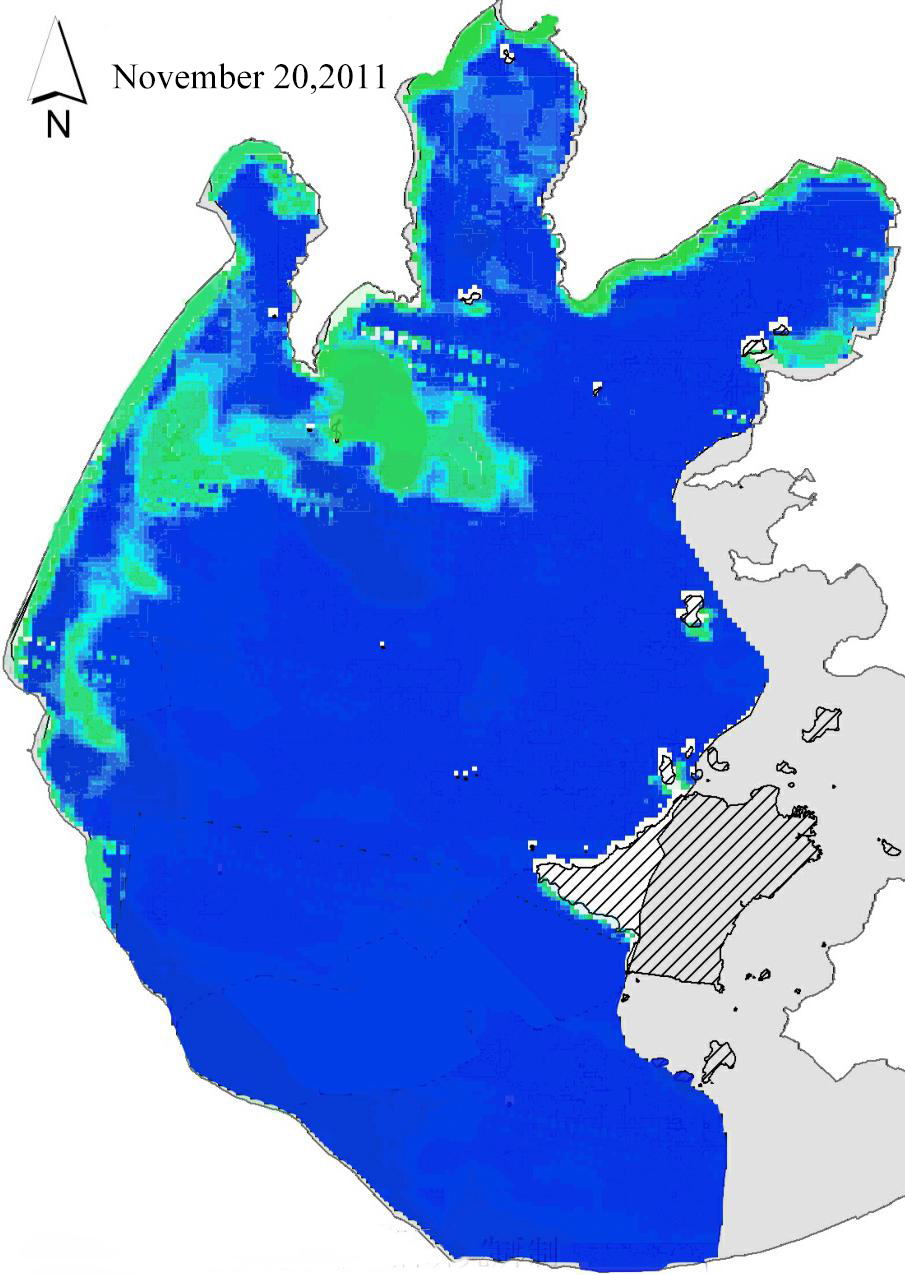

Supplement: Supplemental Information 6 [file peerj-cs-09-1292-s006.zip › 0/20111120_taihu_cla.jpg]

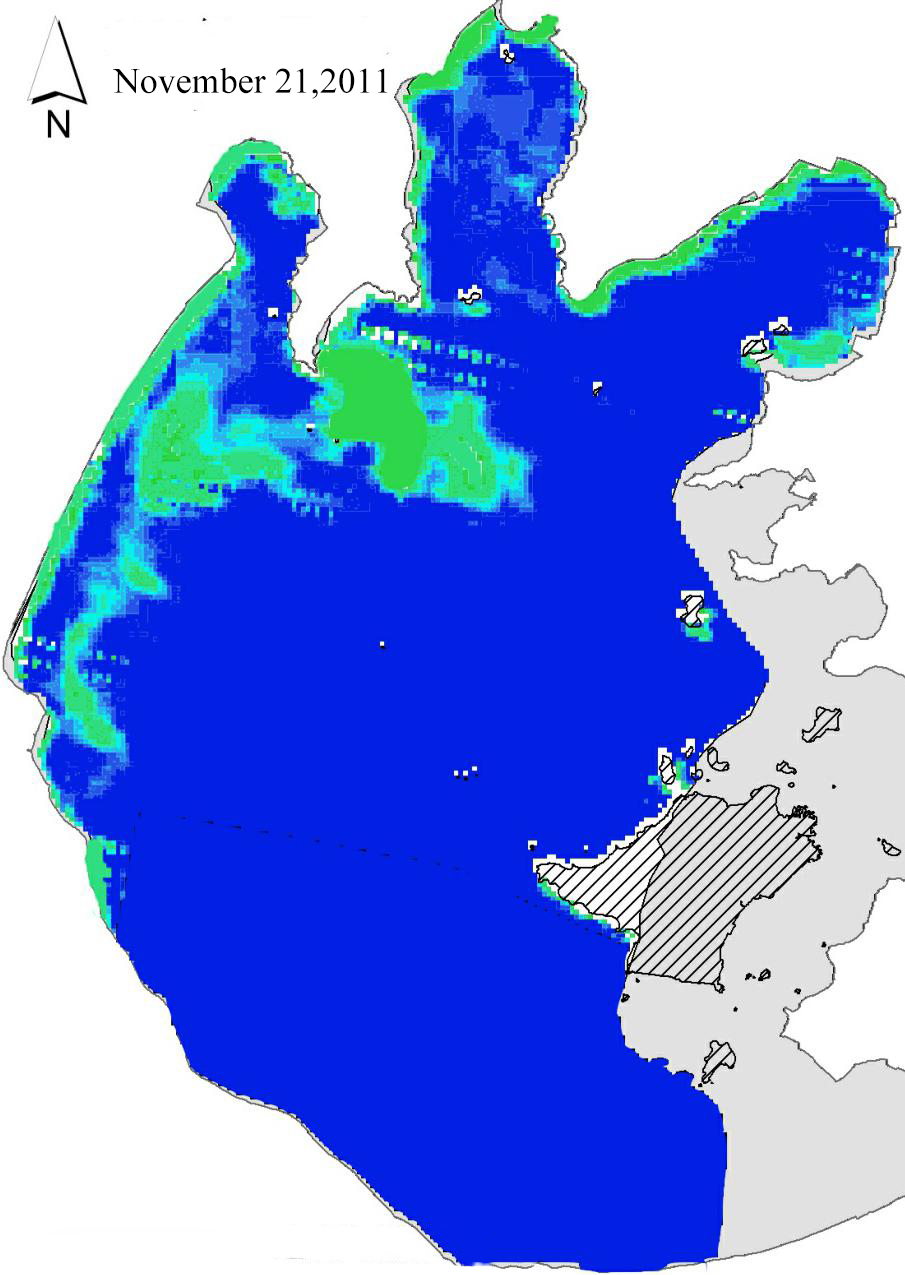

Supplement: Supplemental Information 6 [file peerj-cs-09-1292-s006.zip › 0/20111121_taihu_cla.jpg]

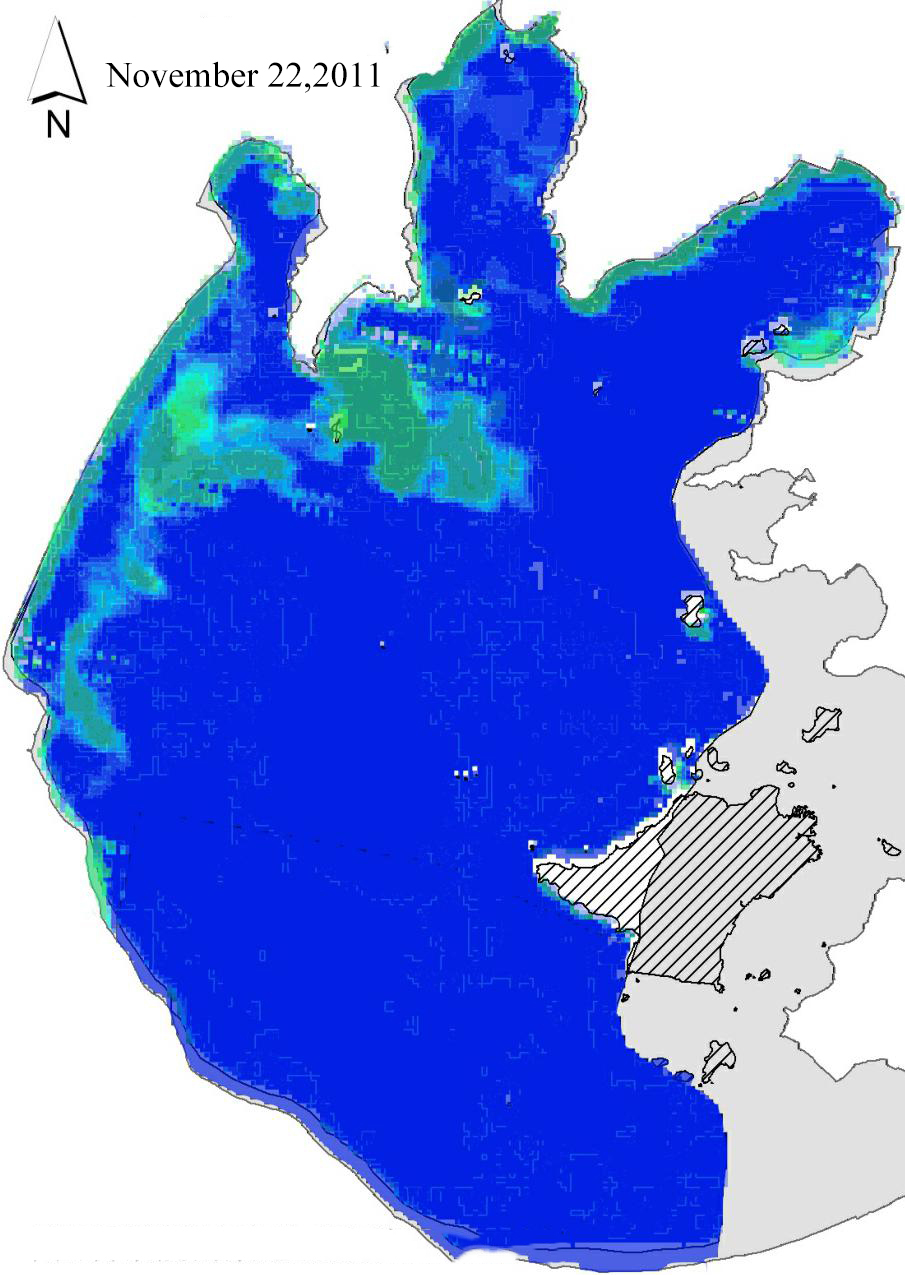

Supplement: Supplemental Information 6 [file peerj-cs-09-1292-s006.zip › 0/20111122_taihu_cla.jpg]

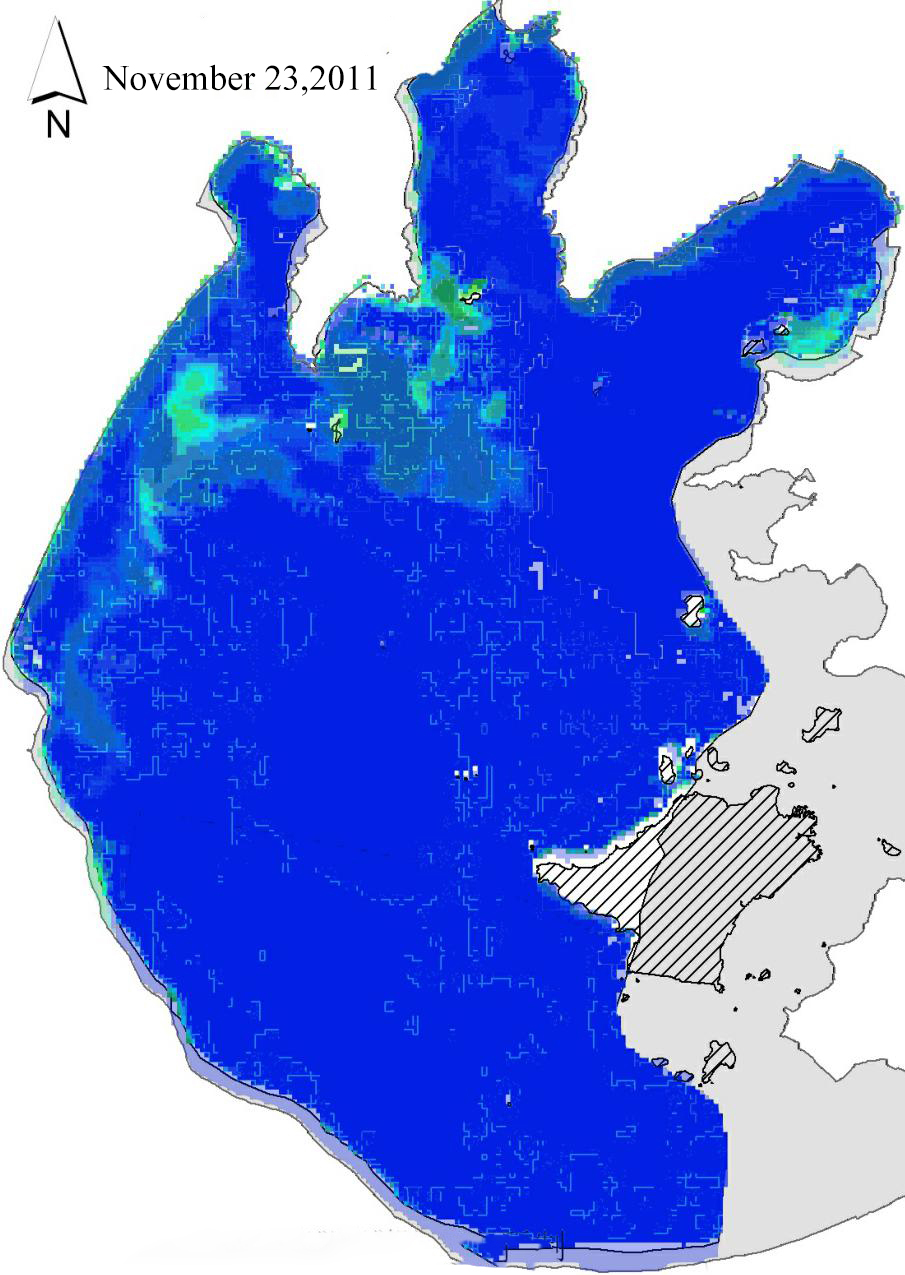

Supplement: Supplemental Information 6 [file peerj-cs-09-1292-s006.zip › 0/20111123_taihu_cla.jpg]

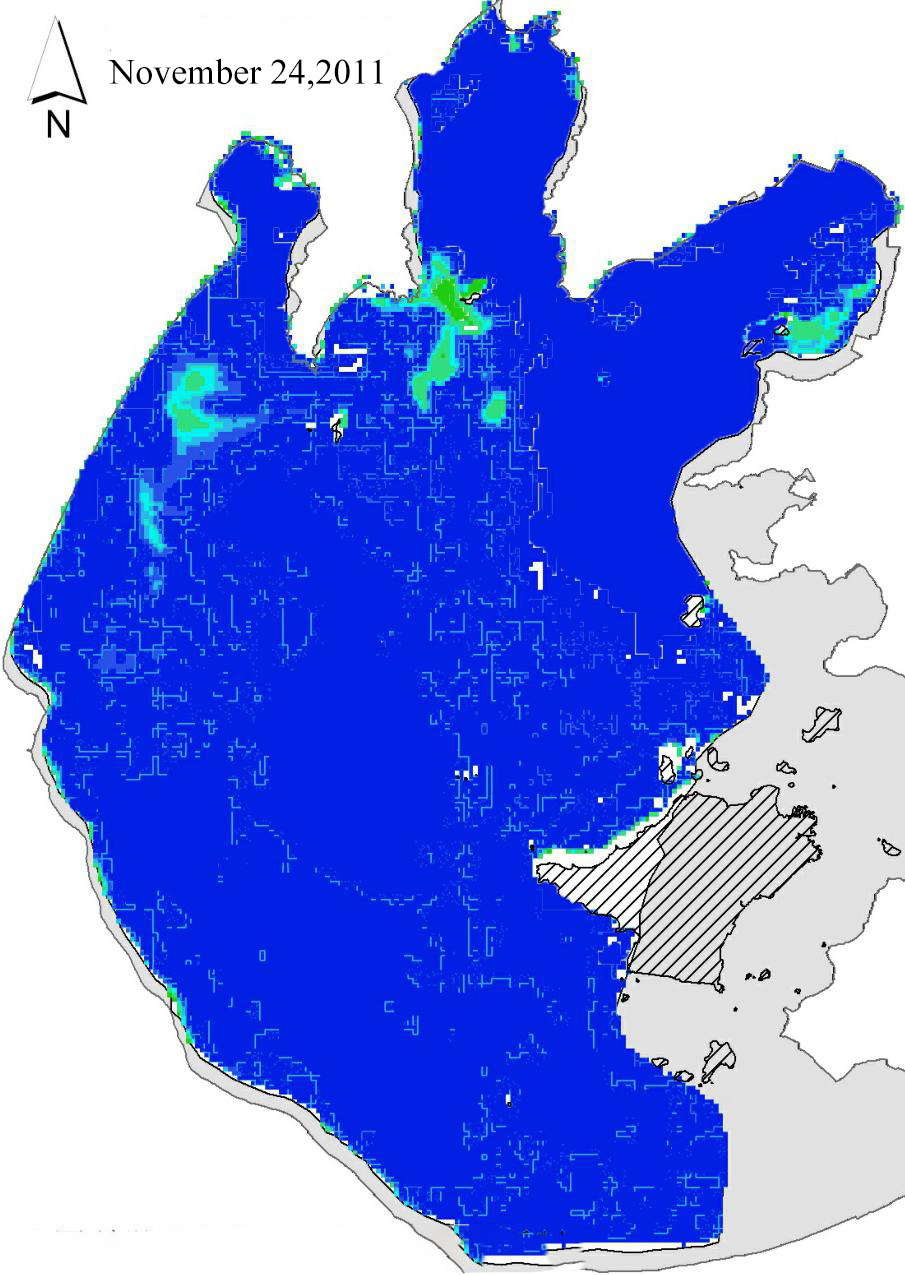

Supplement: Supplemental Information 6 [file peerj-cs-09-1292-s006.zip › 0/20111124_taihu_chla.jpg]

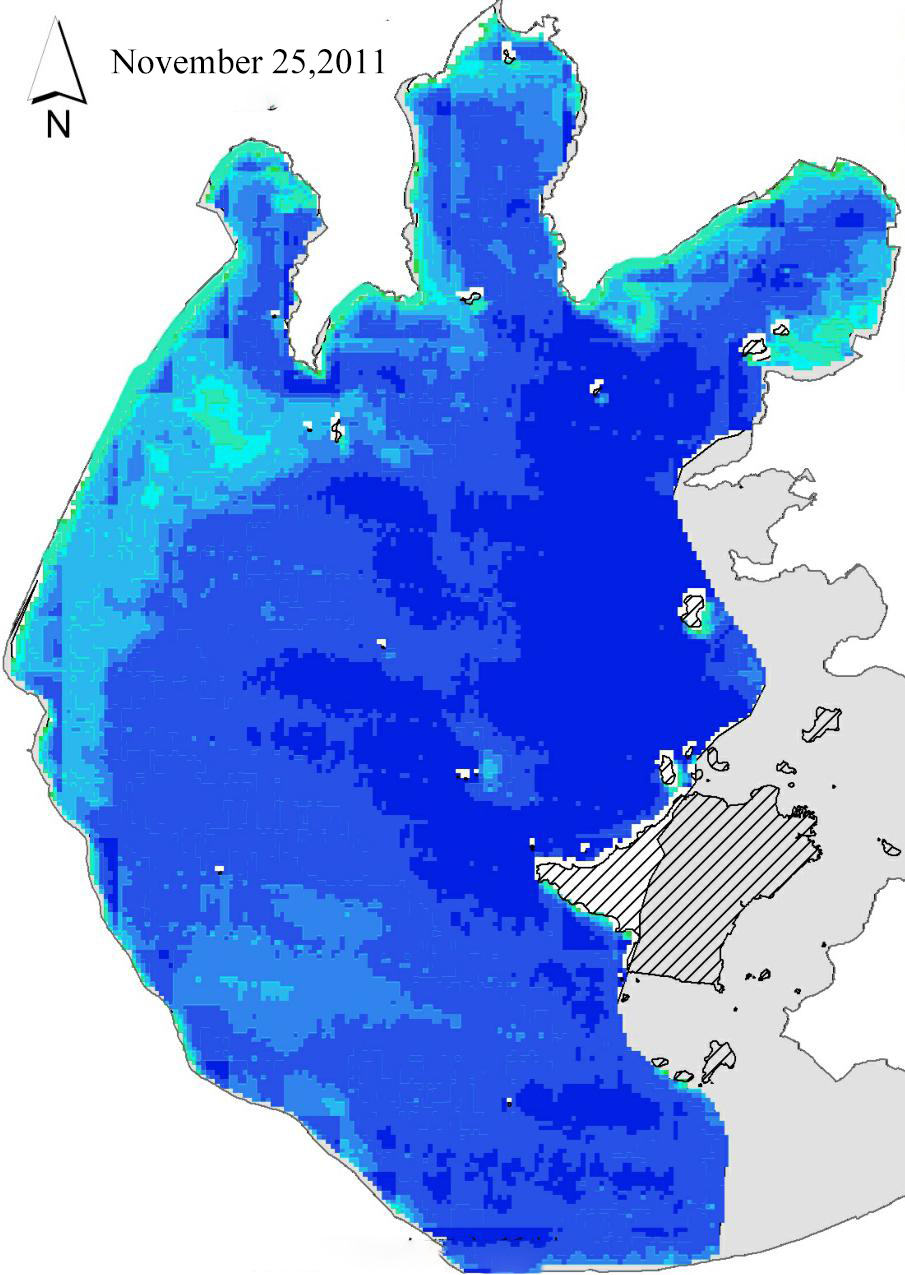

Supplement: Supplemental Information 6 [file peerj-cs-09-1292-s006.zip › 0/20111125_taihu_cla.jpg]

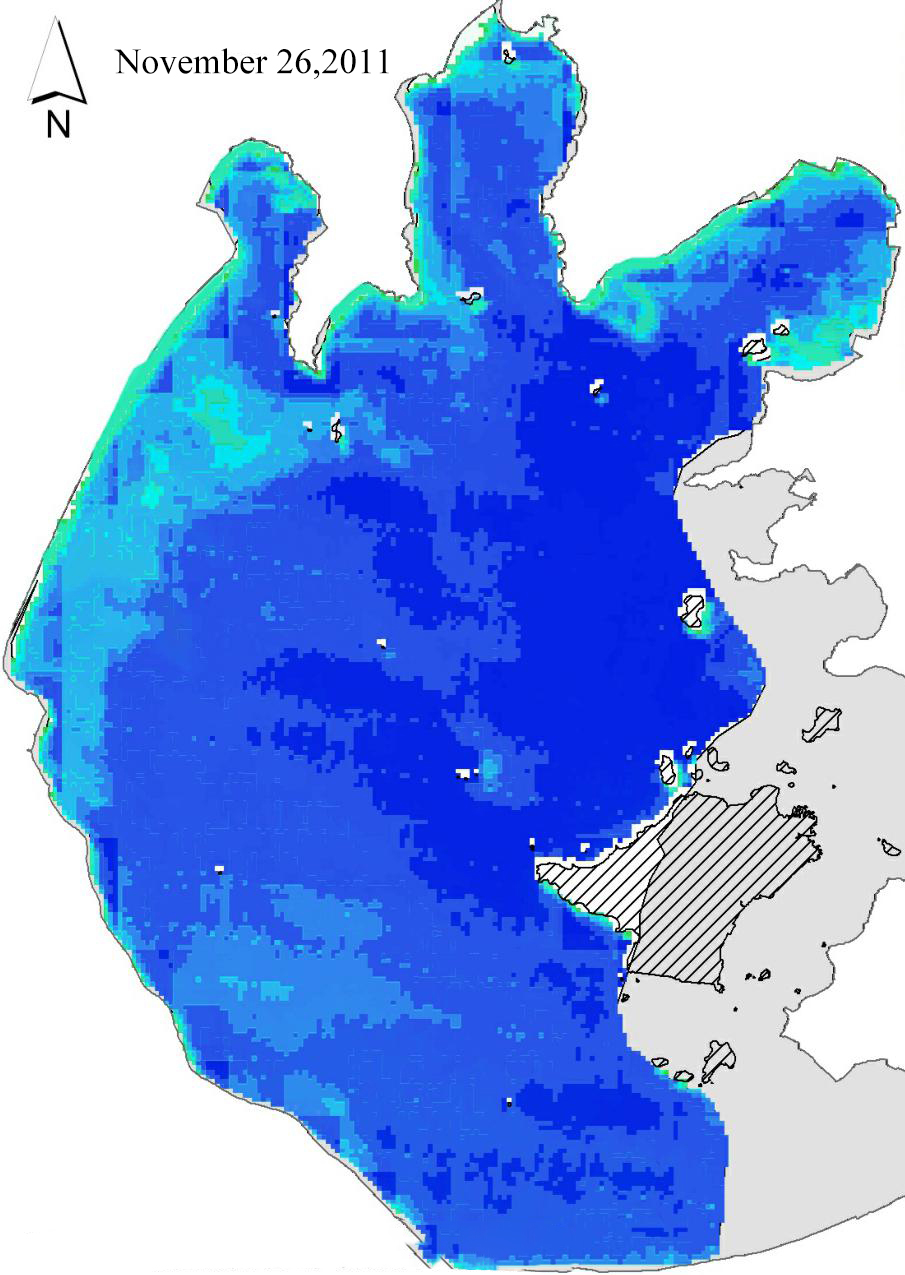

Supplement: Supplemental Information 6 [file peerj-cs-09-1292-s006.zip › 0/20111126_taihu_cla.jpg]

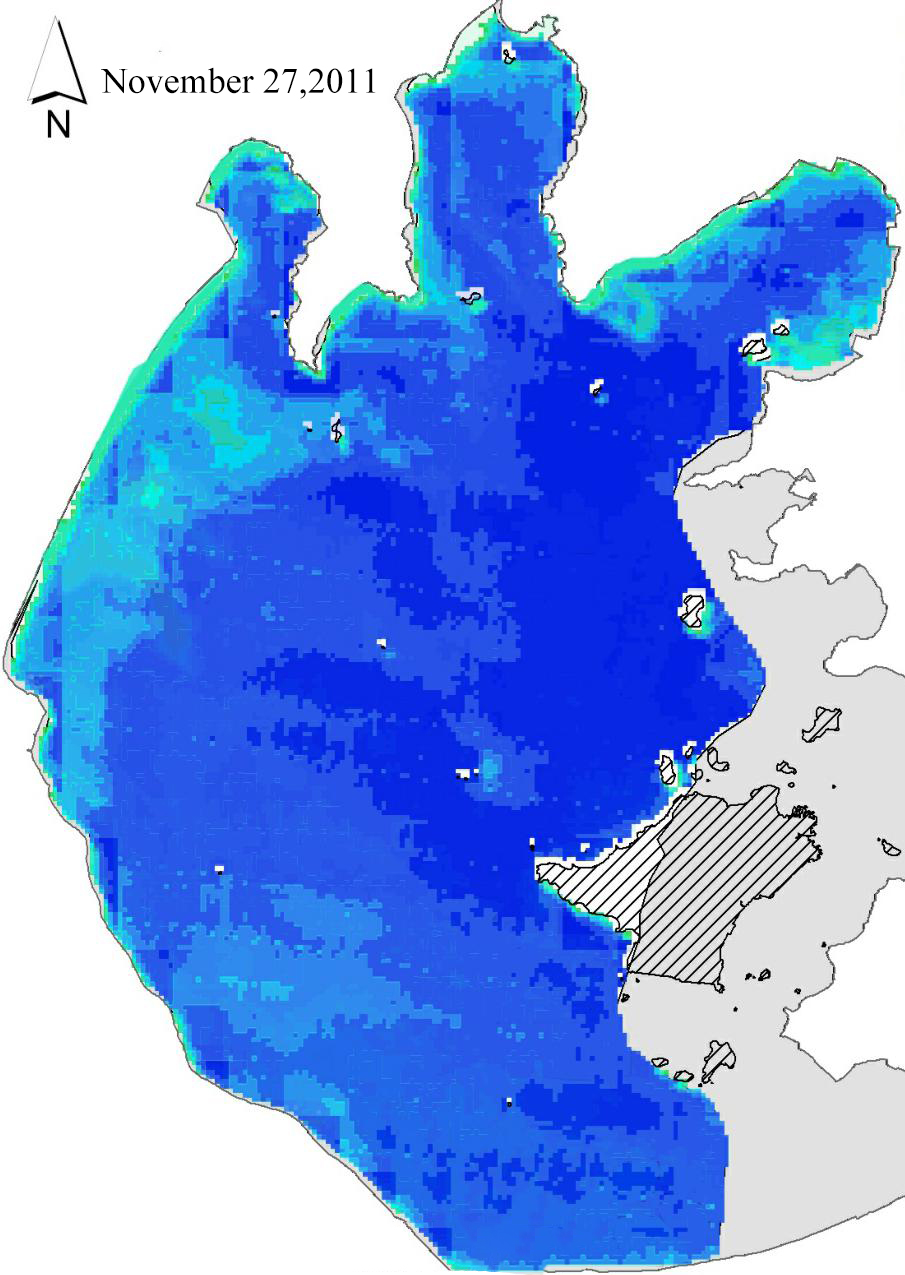

Supplement: Supplemental Information 6 [file peerj-cs-09-1292-s006.zip › 0/20111127_taihu_cla.jpg]

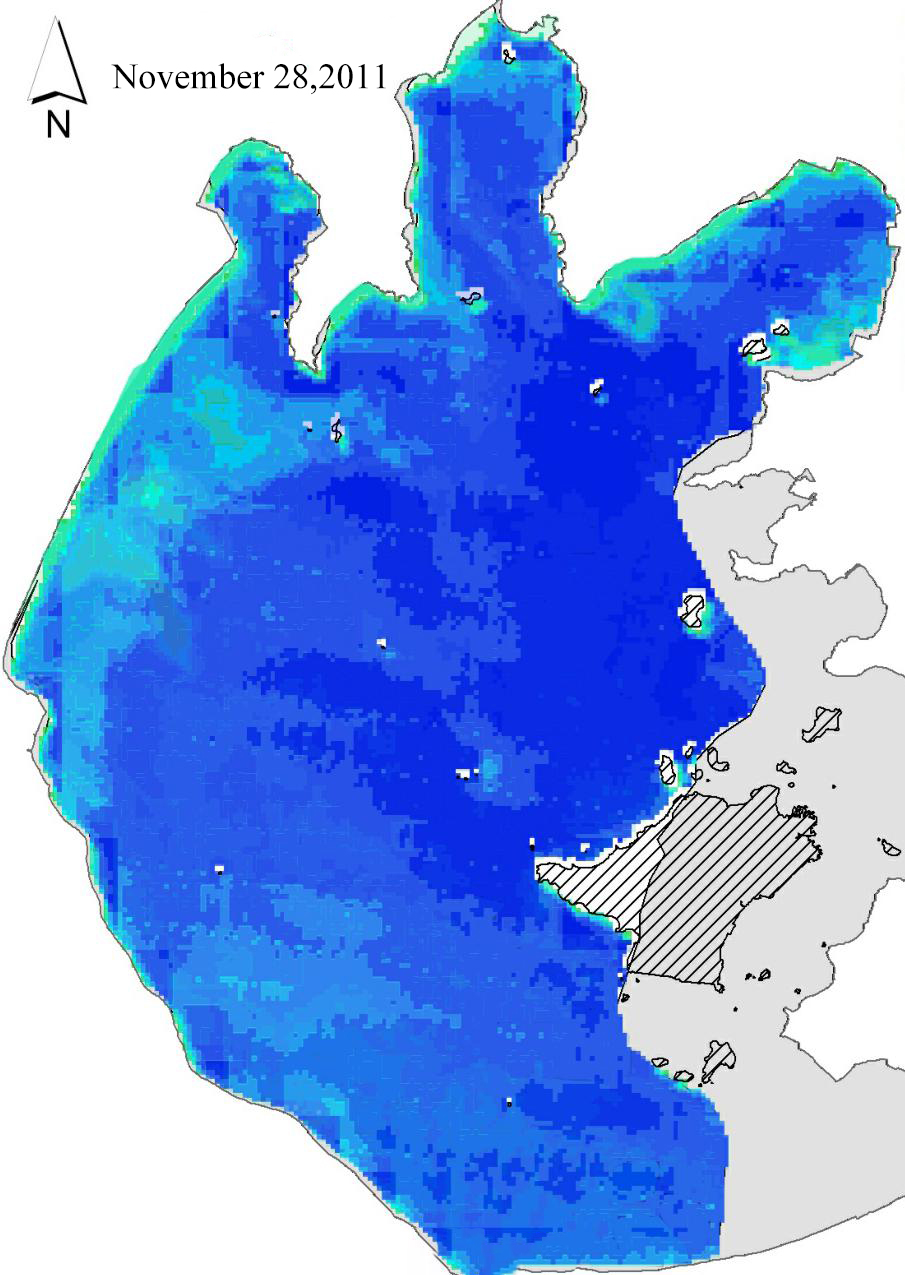

Supplement: Supplemental Information 6 [file peerj-cs-09-1292-s006.zip › 0/20111128_taihu_cla.jpg]

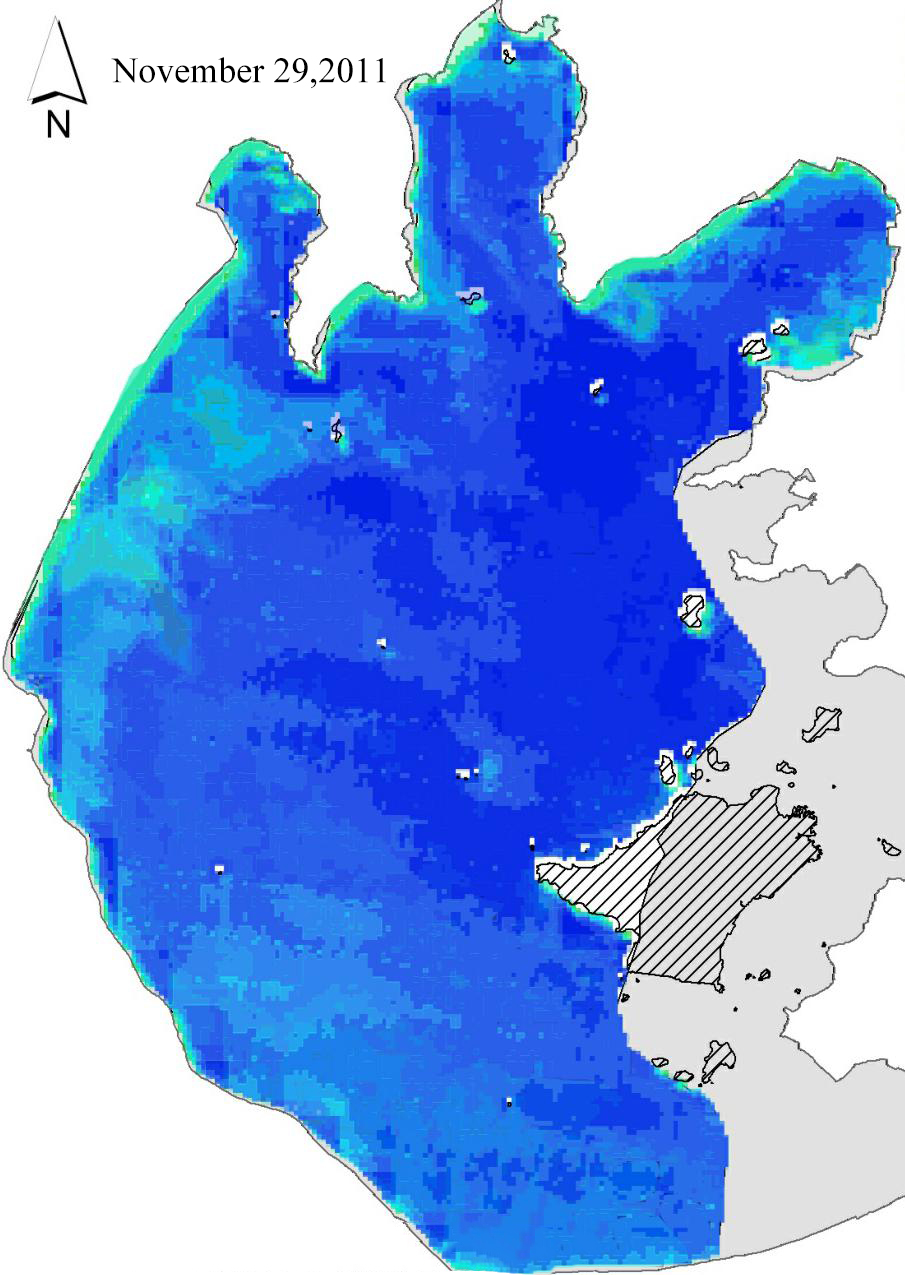

Supplement: Supplemental Information 6 [file peerj-cs-09-1292-s006.zip › 0/20111129_taihu_cla.jpg]

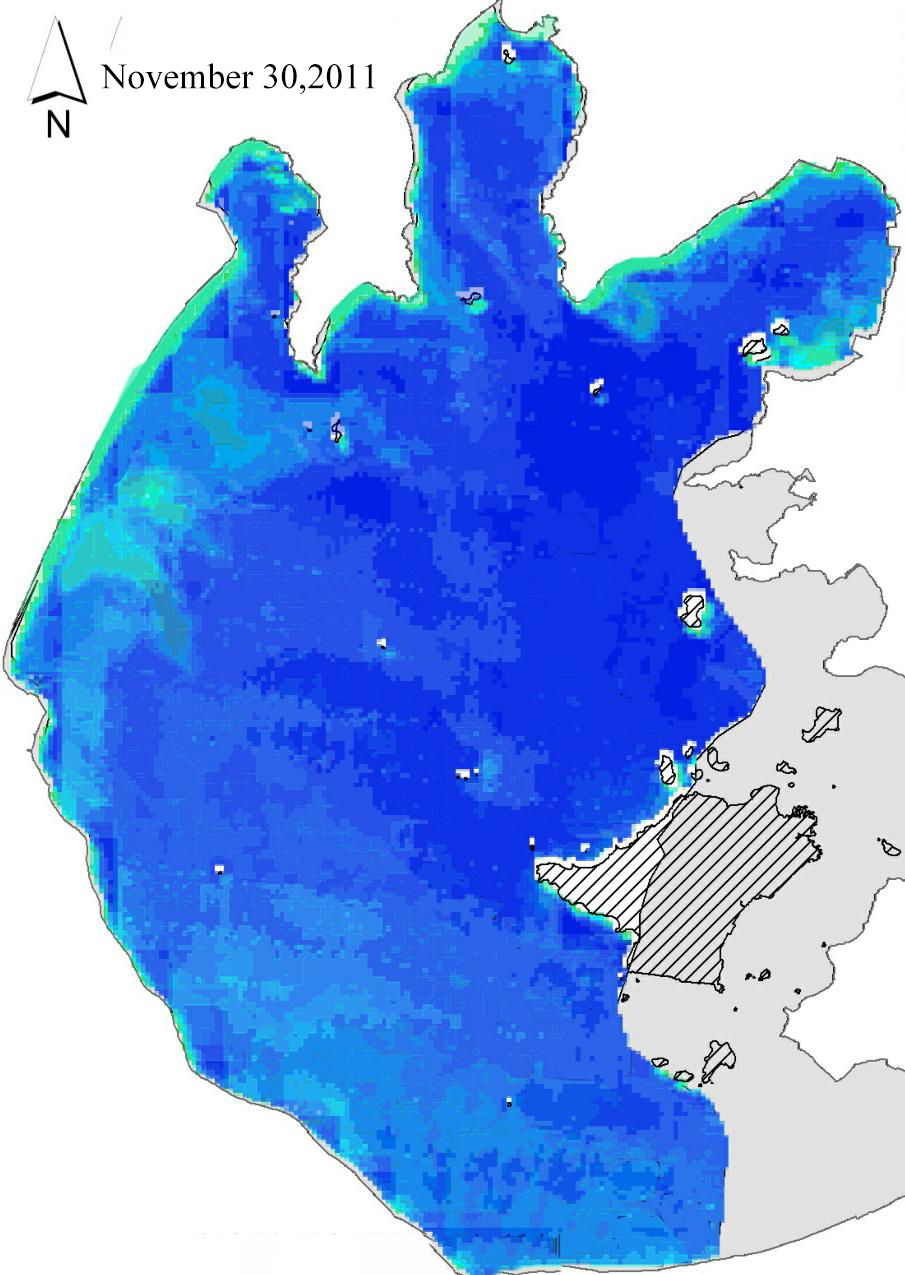

Supplement: Supplemental Information 6 [file peerj-cs-09-1292-s006.zip › 0/20111130_taihu_cla.jpg]

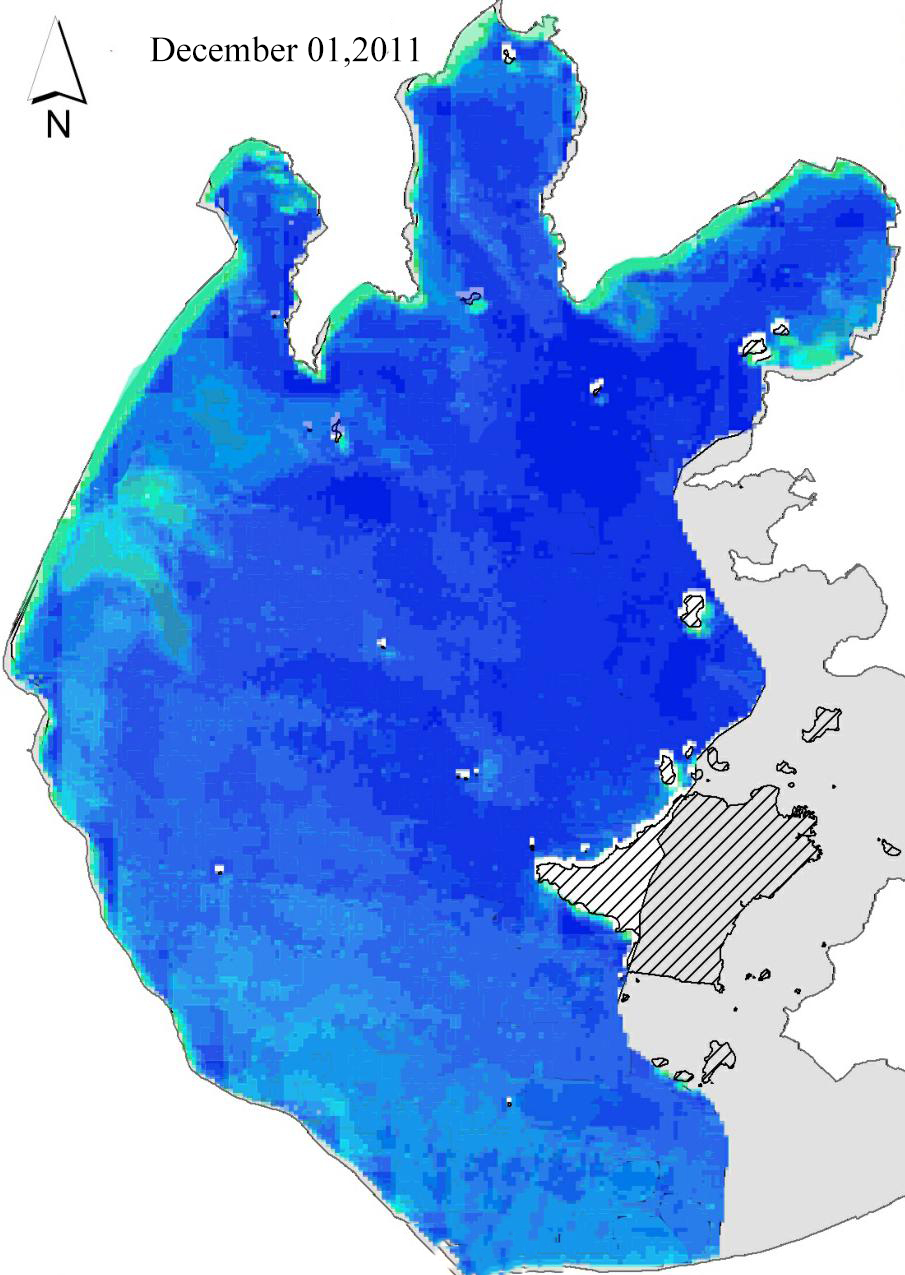

Supplement: Supplemental Information 6 [file peerj-cs-09-1292-s006.zip › 0/20111201_taihu_cla.jpg]

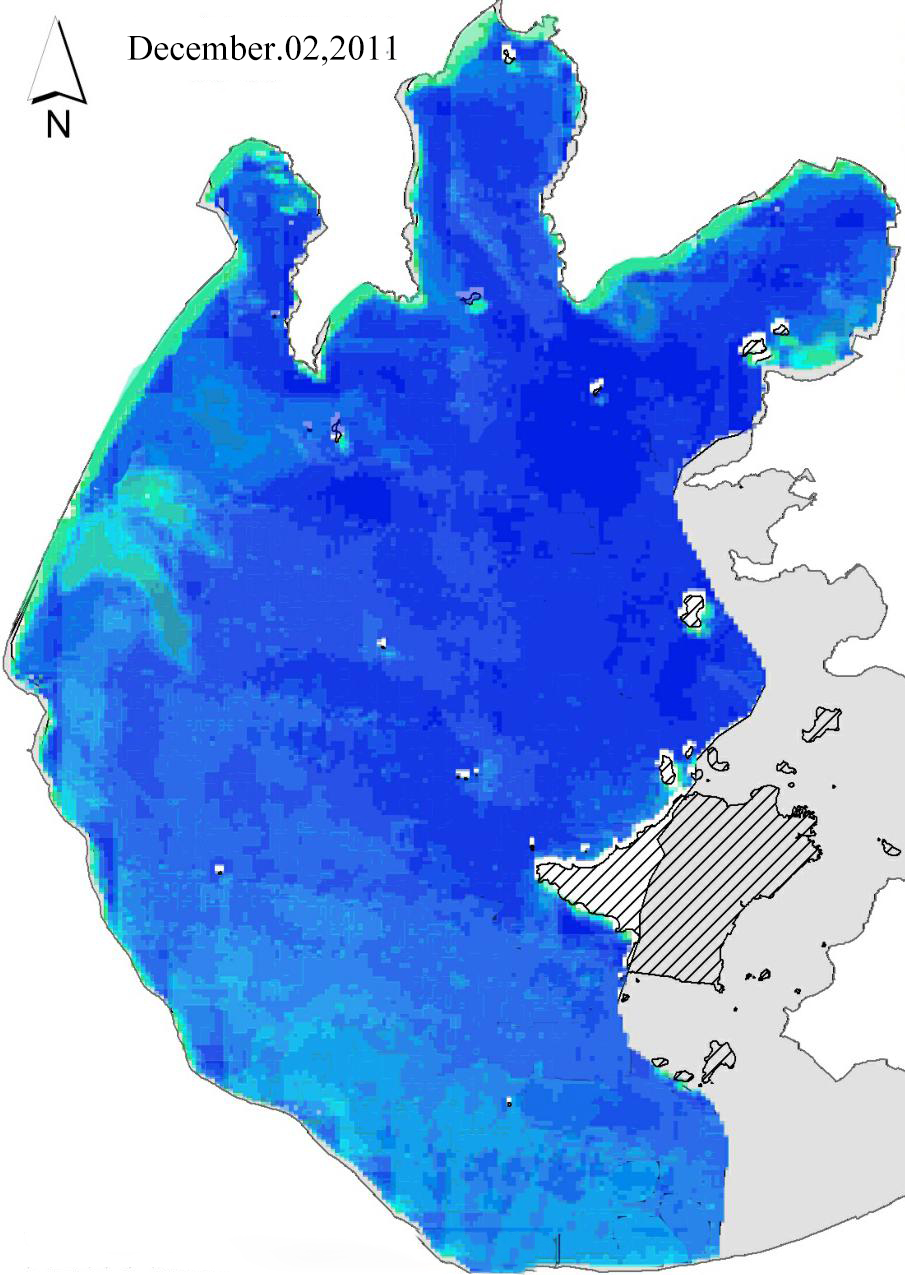

Supplement: Supplemental Information 6 [file peerj-cs-09-1292-s006.zip › 0/20111202_taihu_cla.jpg]

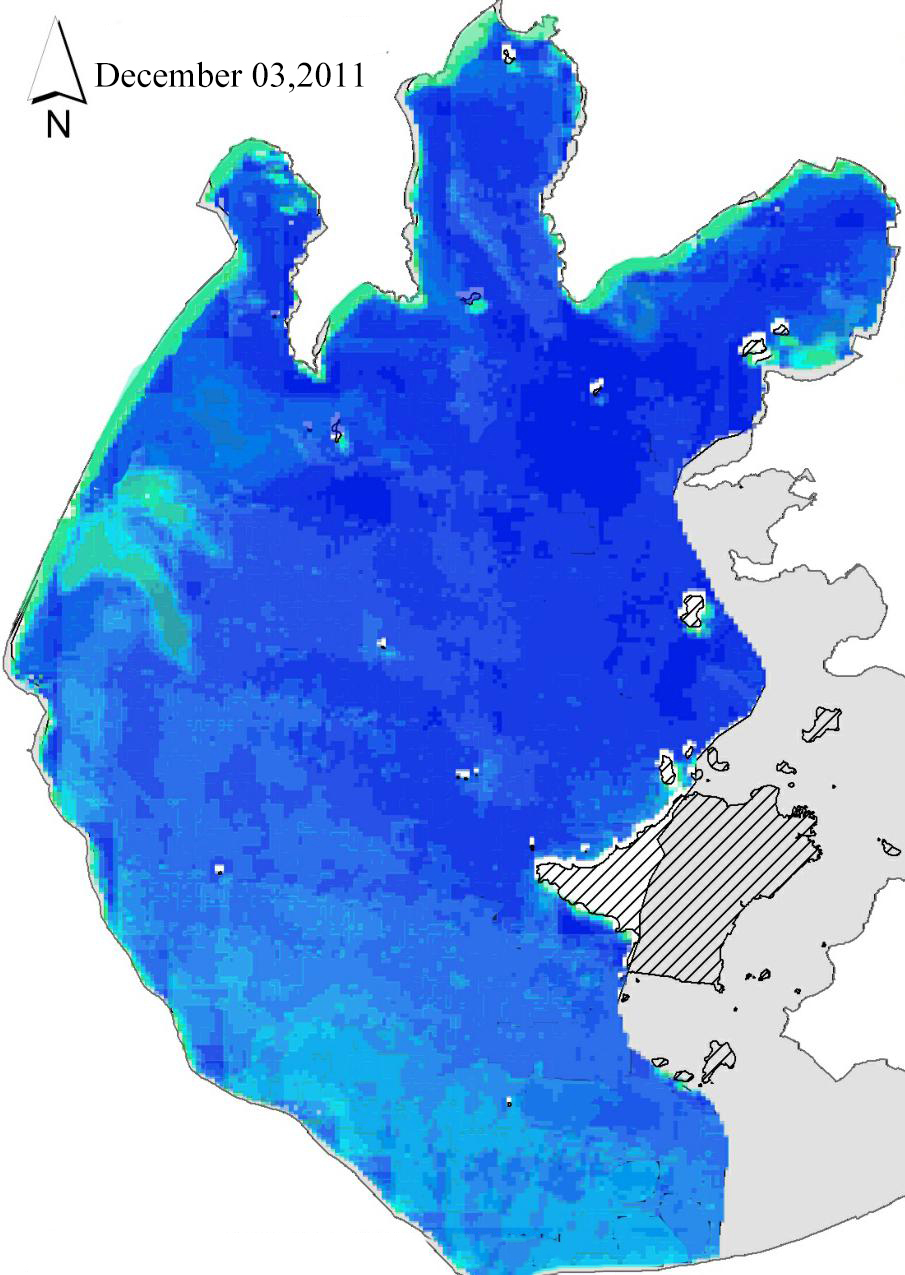

Supplement: Supplemental Information 6 [file peerj-cs-09-1292-s006.zip › 0/20111203_taihu_cla.jpg]

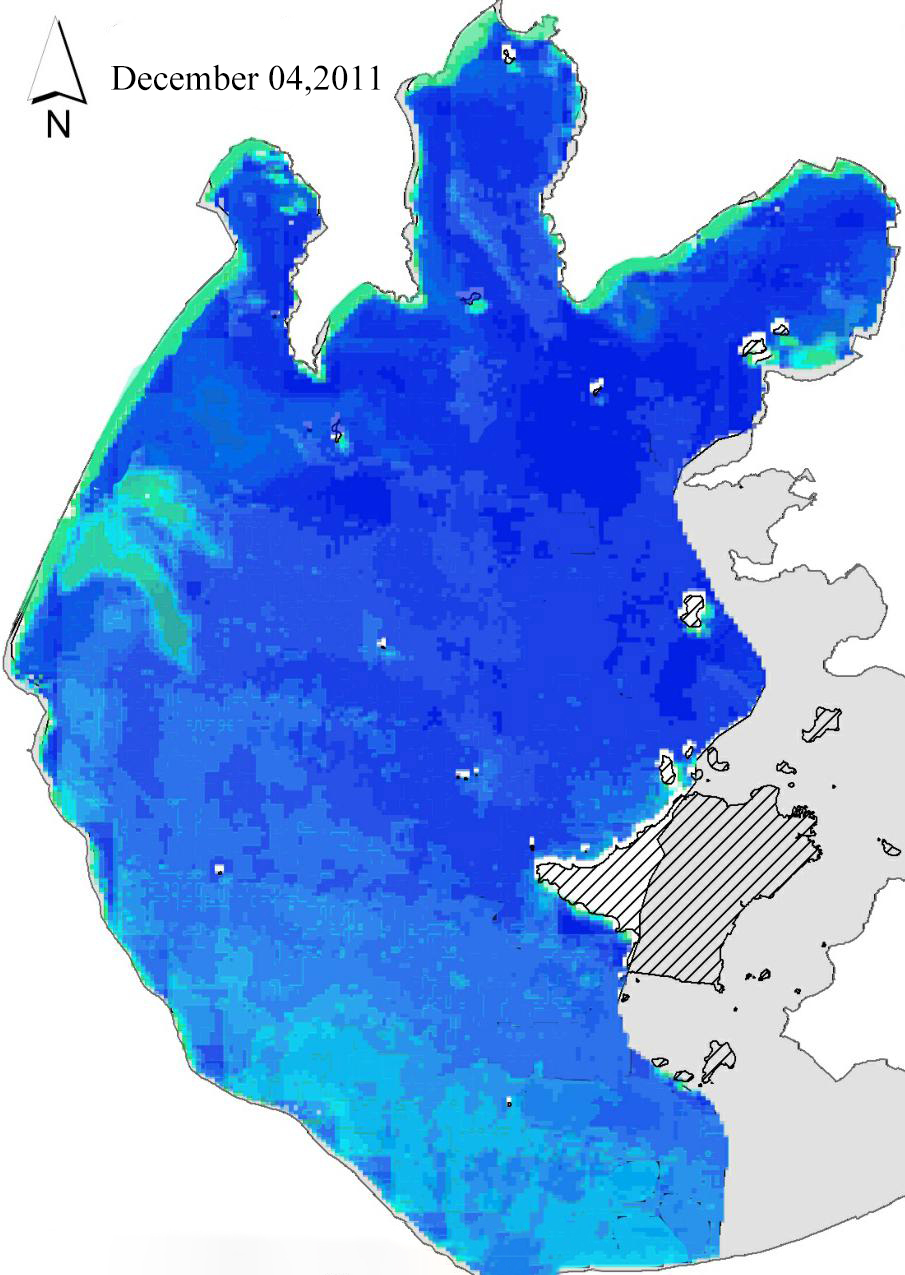

Supplement: Supplemental Information 6 [file peerj-cs-09-1292-s006.zip › 0/20111204_taihu_cla.jpg]

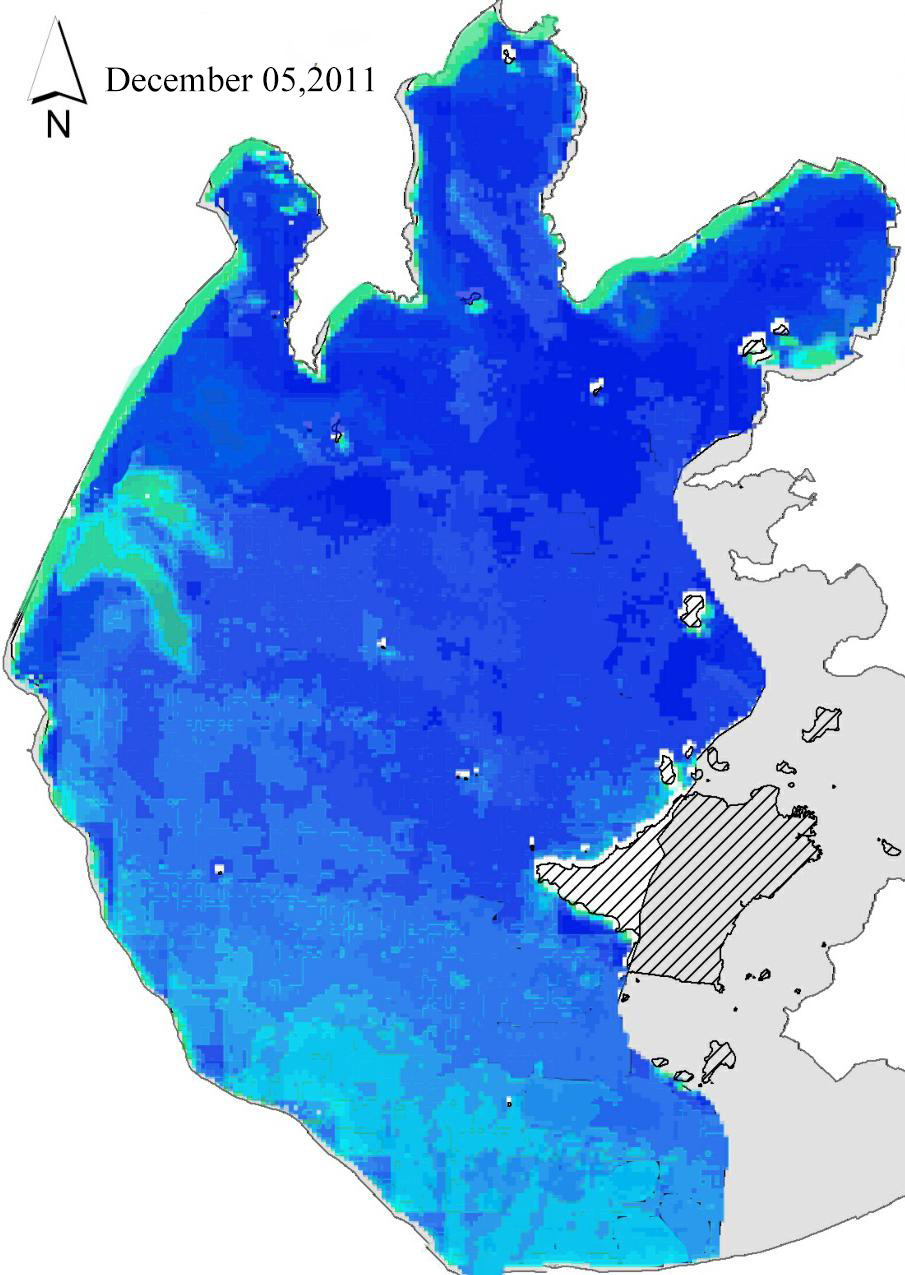

Supplement: Supplemental Information 6 [file peerj-cs-09-1292-s006.zip › 0/20111205_taihu_cla.jpg]

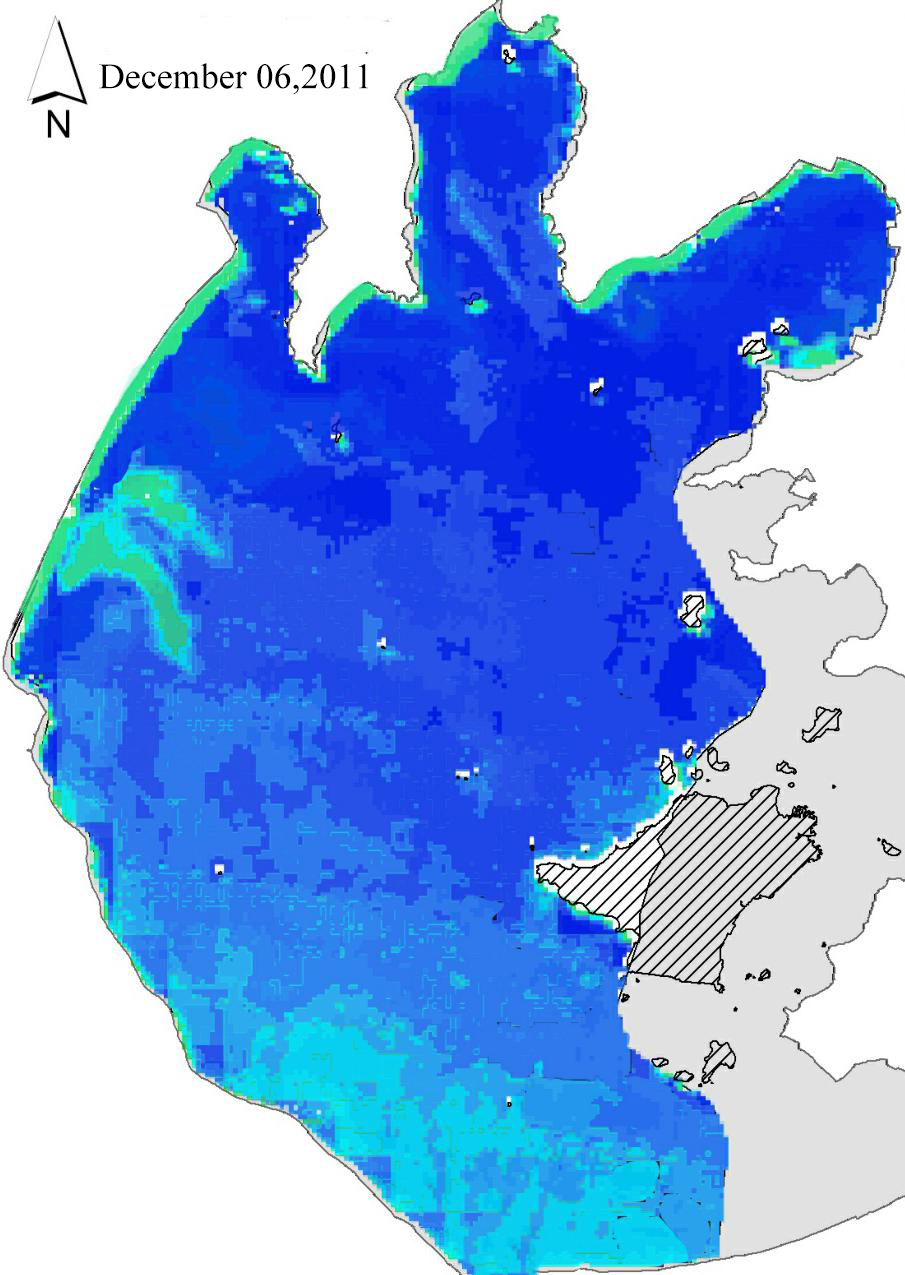

Supplement: Supplemental Information 6 [file peerj-cs-09-1292-s006.zip › 0/20111206_taihu_cla.jpg]

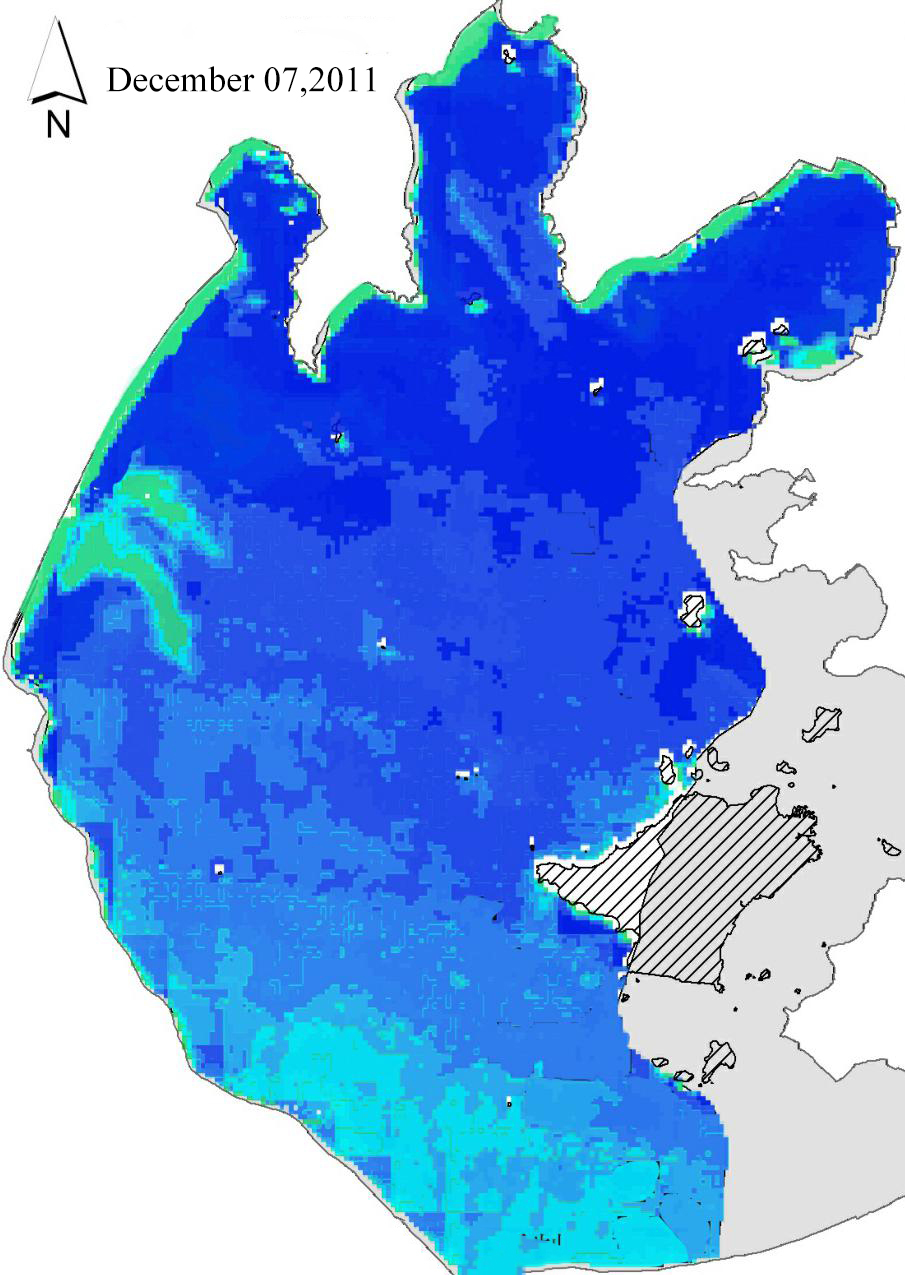

Supplement: Supplemental Information 6 [file peerj-cs-09-1292-s006.zip › 0/20111207_taihu_cla.jpg]

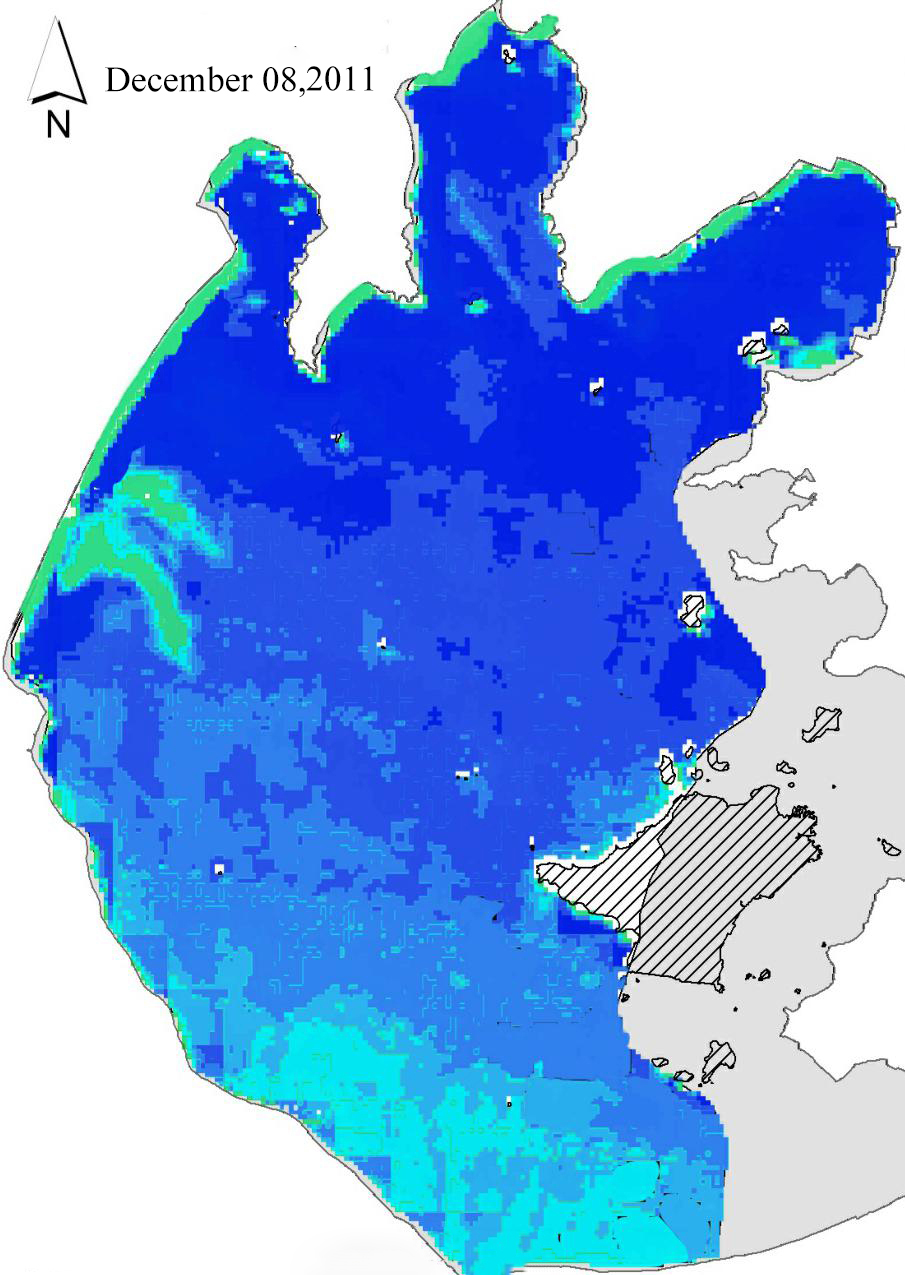

Supplement: Supplemental Information 6 [file peerj-cs-09-1292-s006.zip › 0/20111208_taihu_cla.jpg]

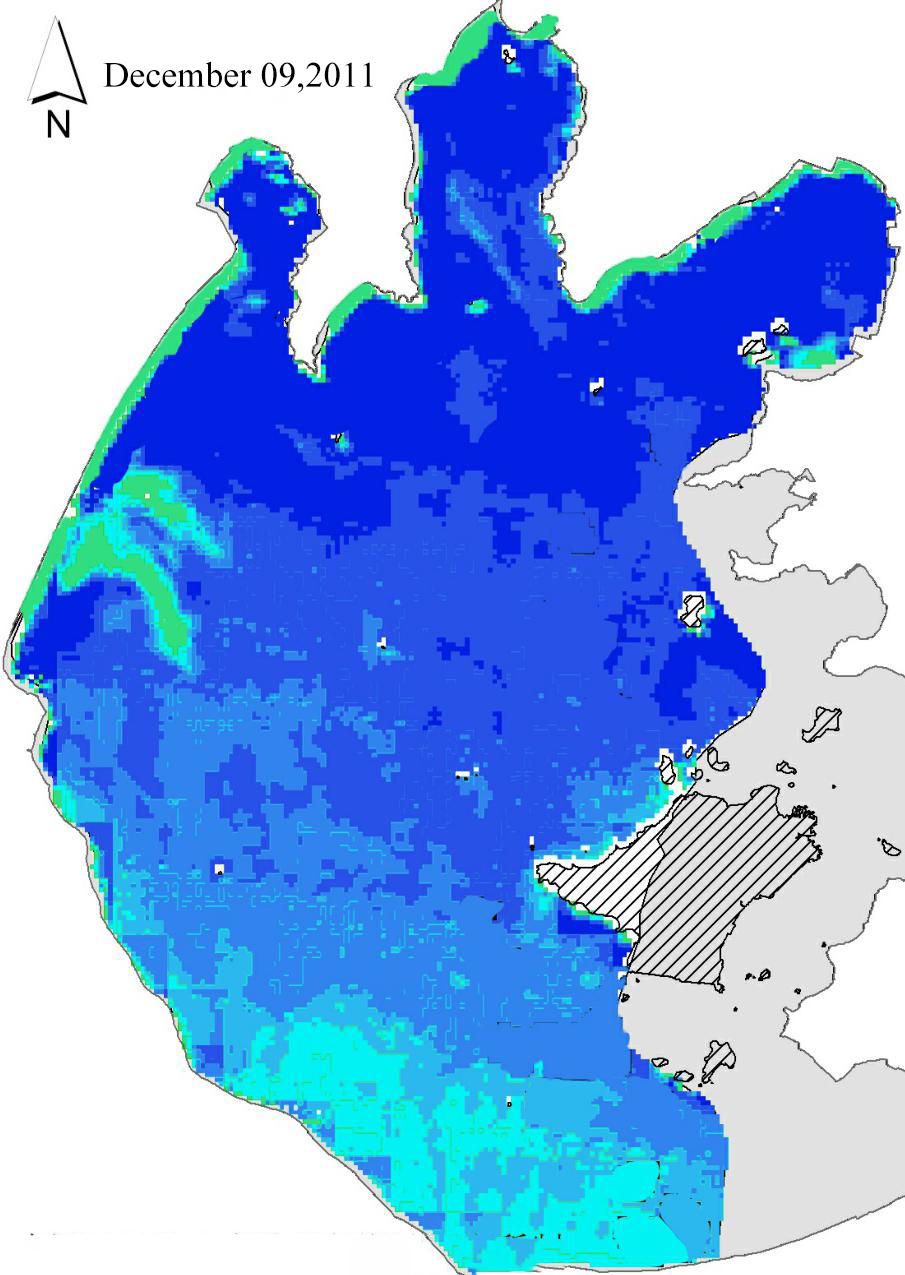

Supplement: Supplemental Information 6 [file peerj-cs-09-1292-s006.zip › 0/20111209_taihu_chla.jpg]

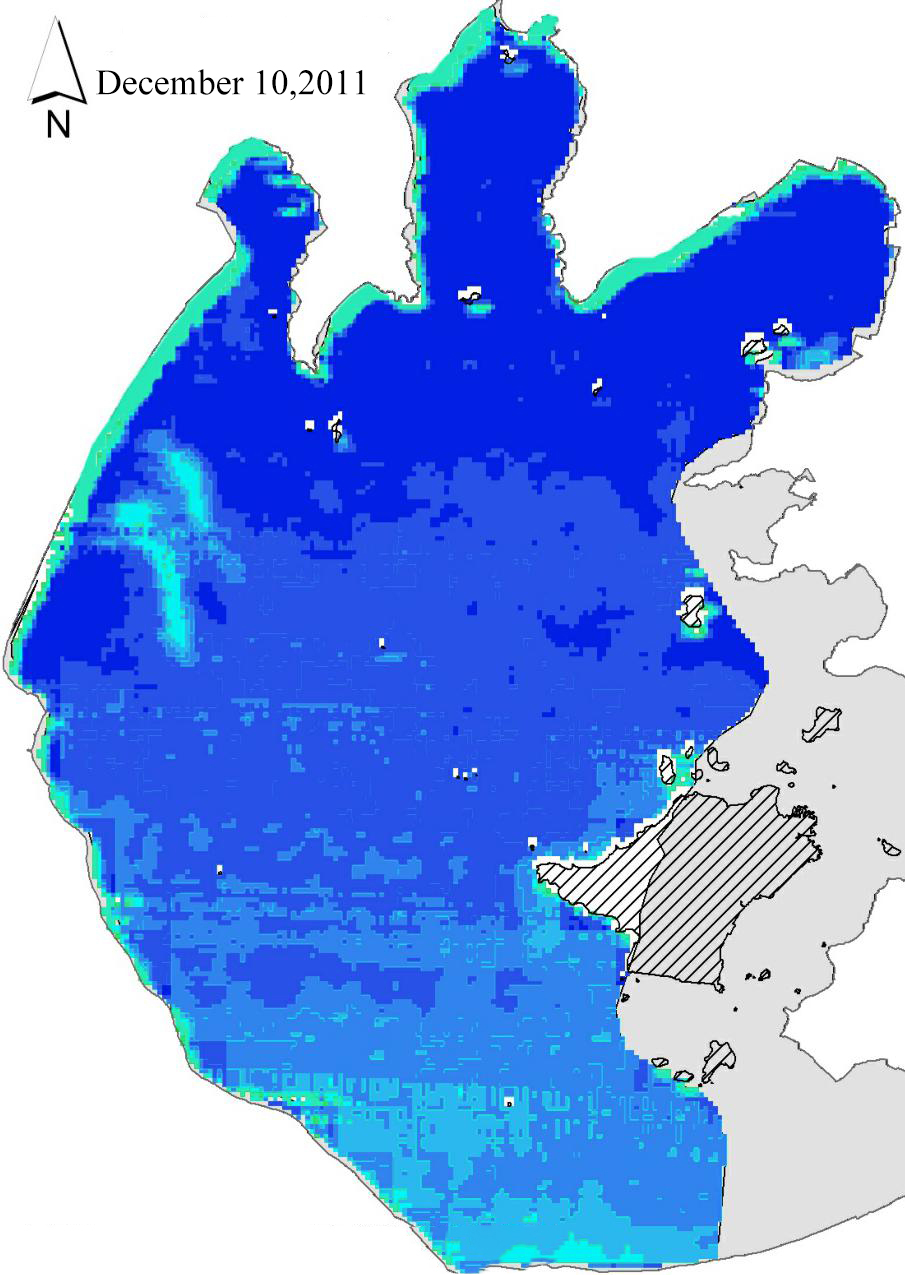

Supplement: Supplemental Information 6 [file peerj-cs-09-1292-s006.zip › 0/20111210_taihu_chla.jpg]

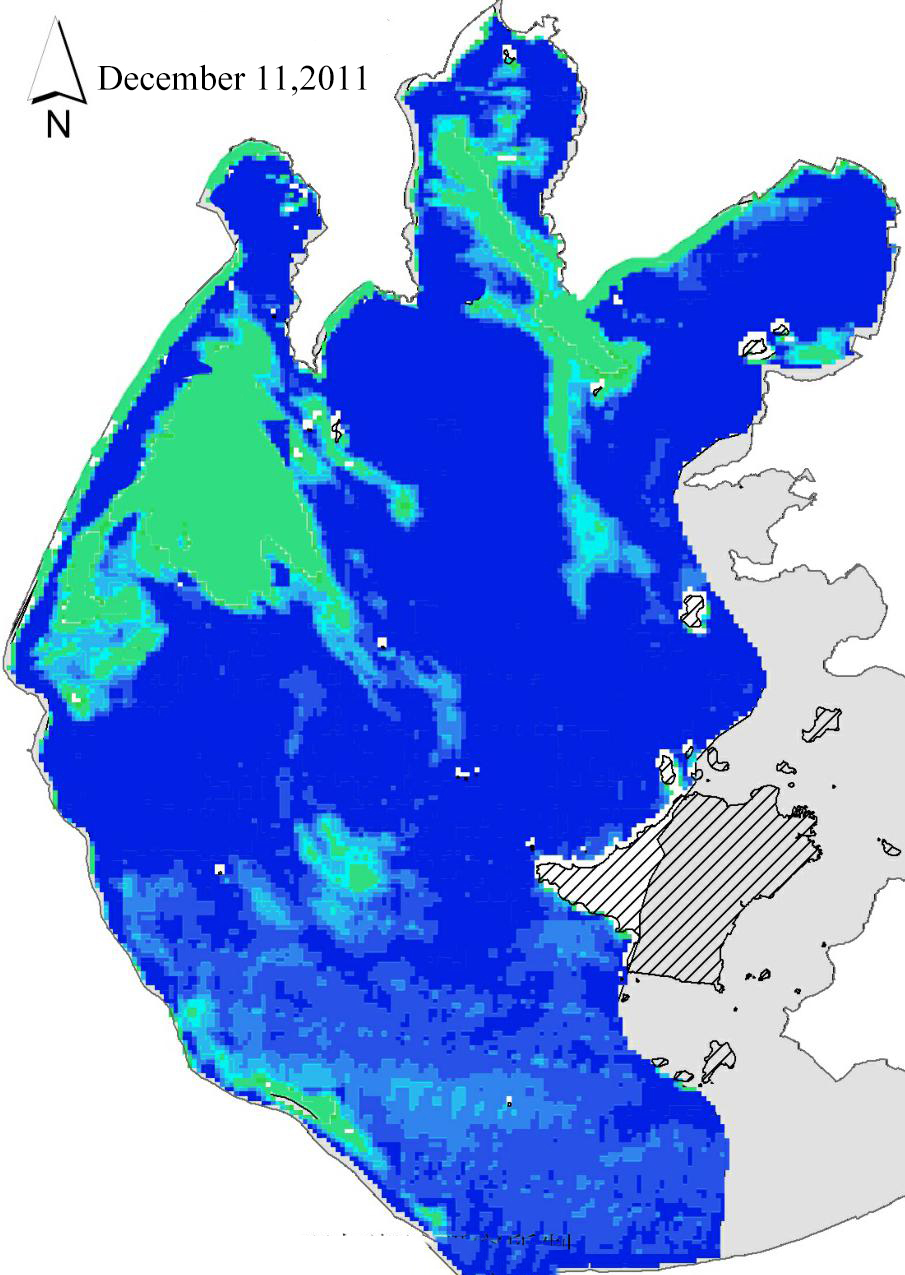

Supplement: Supplemental Information 6 [file peerj-cs-09-1292-s006.zip › 0/20111211_taihu_chla.jpg]

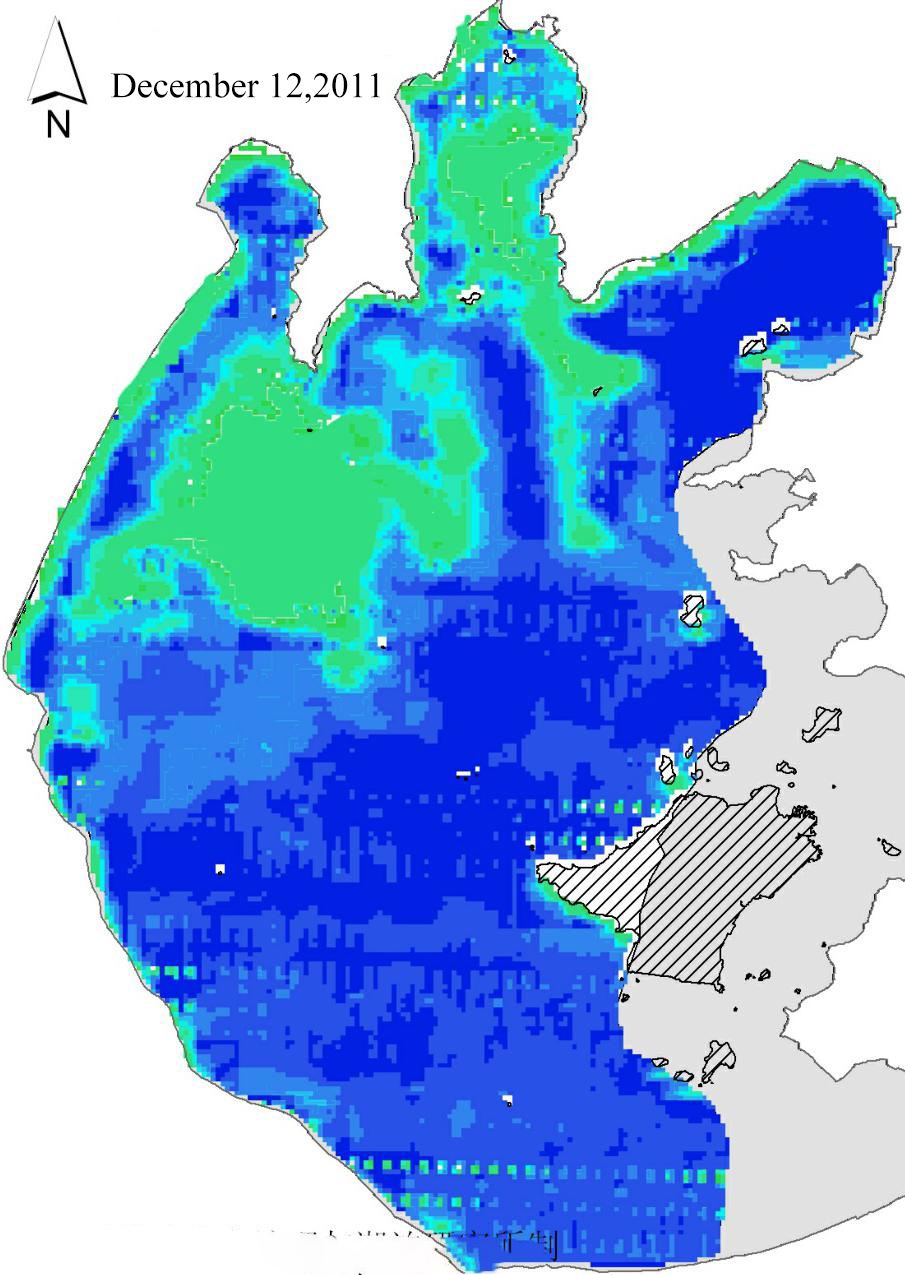

Supplement: Supplemental Information 6 [file peerj-cs-09-1292-s006.zip › 0/20111212_taihu_cla.jpg]

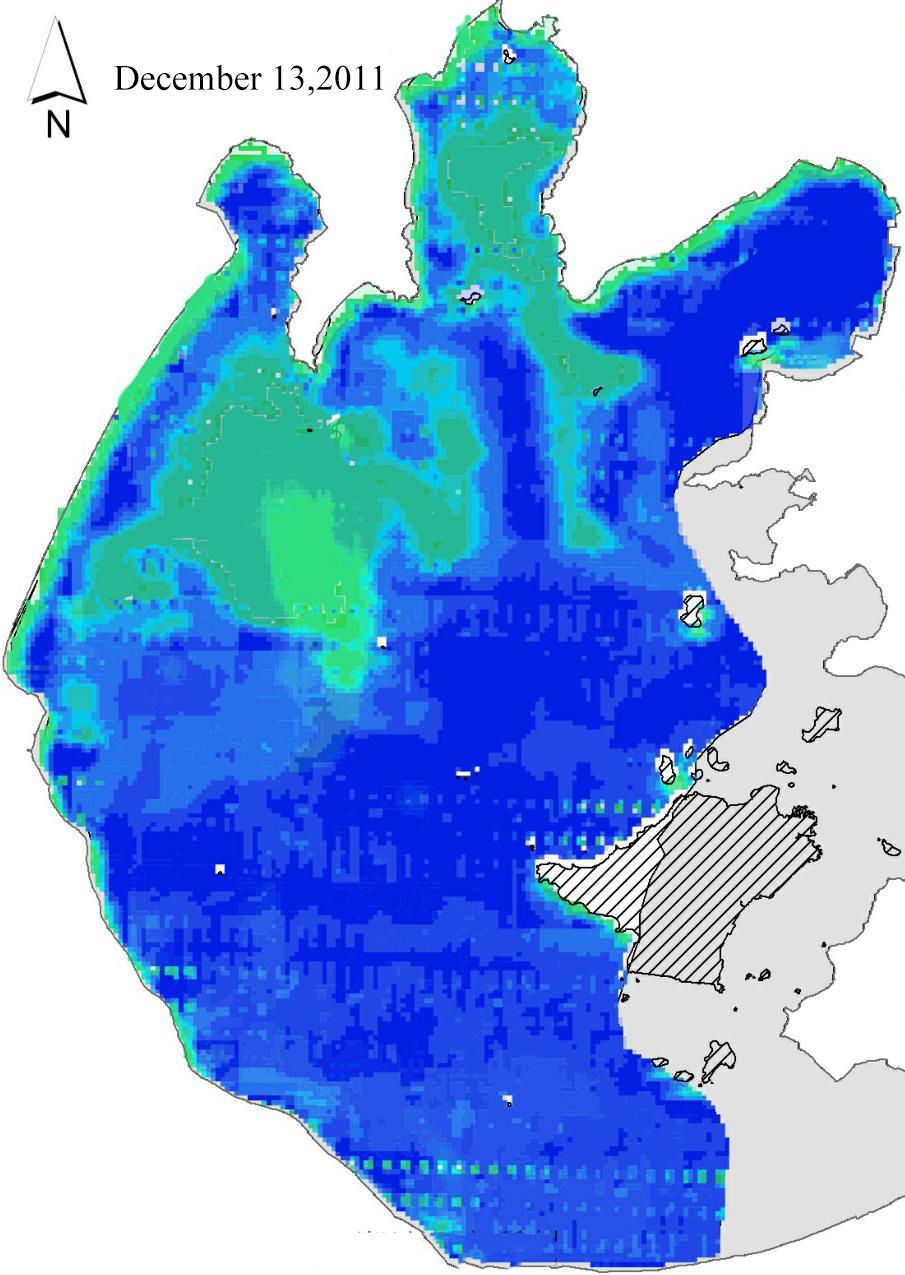

Supplement: Supplemental Information 6 [file peerj-cs-09-1292-s006.zip › 0/20111213_taihu_cla.jpg]

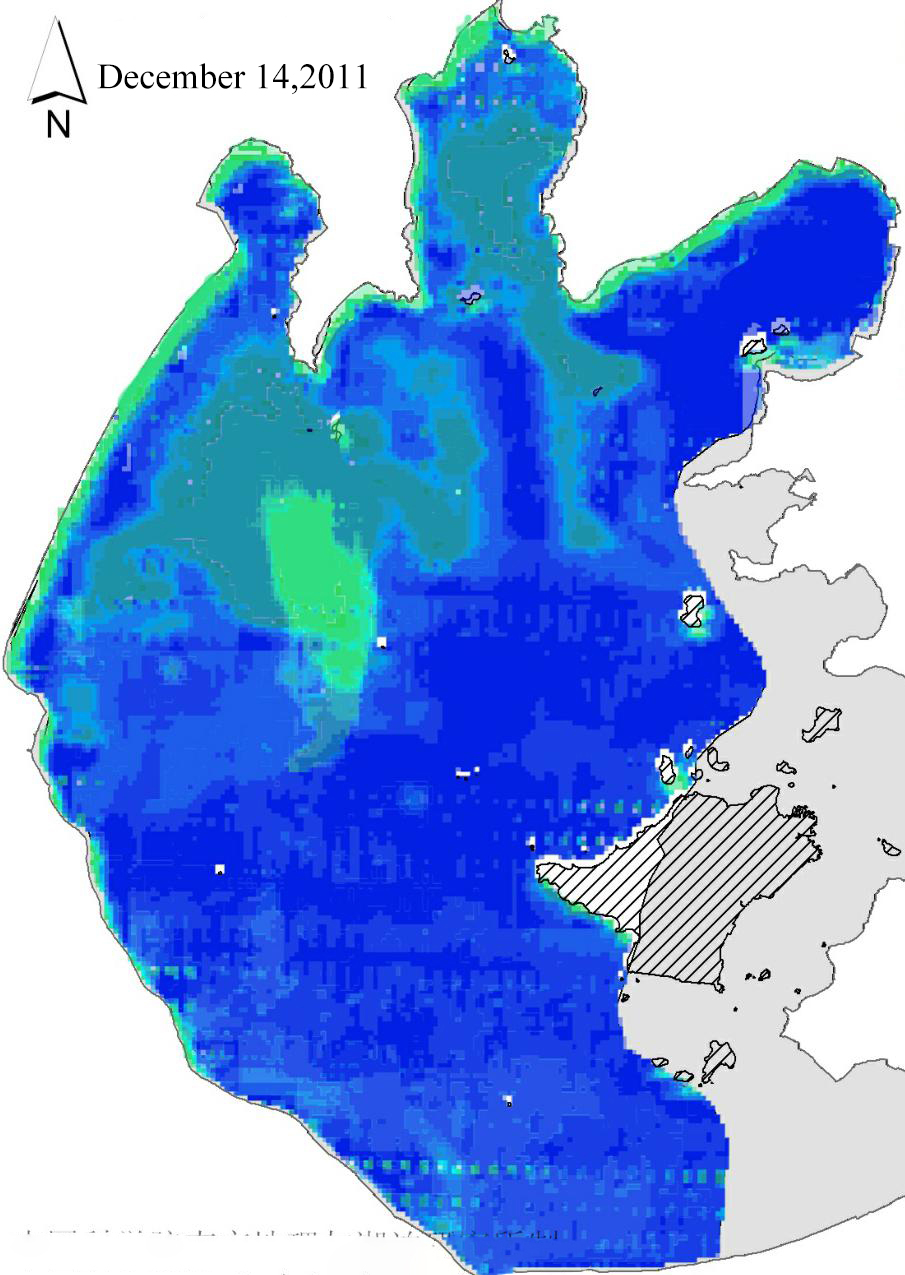

Supplement: Supplemental Information 6 [file peerj-cs-09-1292-s006.zip › 0/20111214_taihu_cla.jpg]

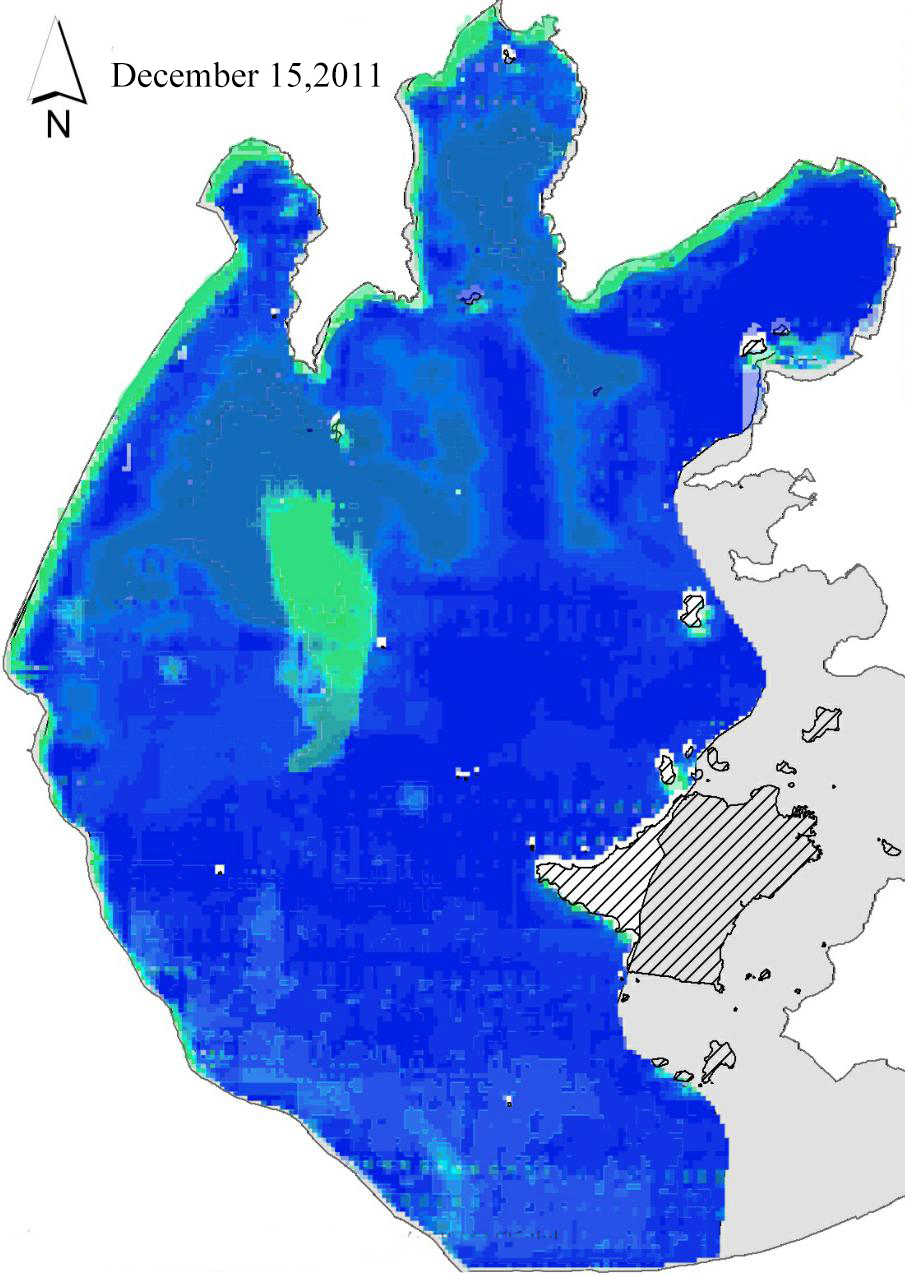

Supplement: Supplemental Information 6 [file peerj-cs-09-1292-s006.zip › 0/20111215_taihu_cla.jpg]

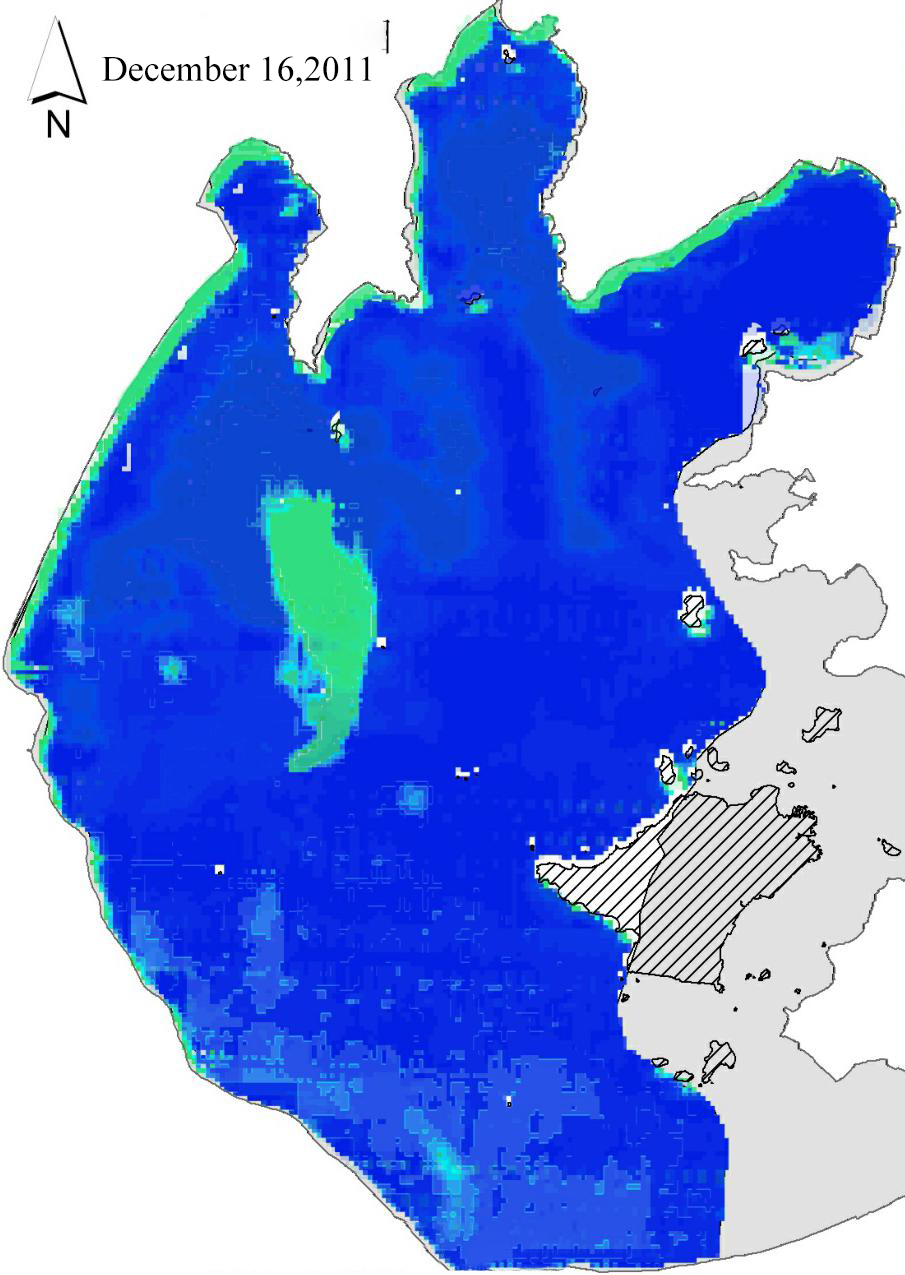

Supplement: Supplemental Information 6 [file peerj-cs-09-1292-s006.zip › 0/20111216_taihu_cla.jpg]

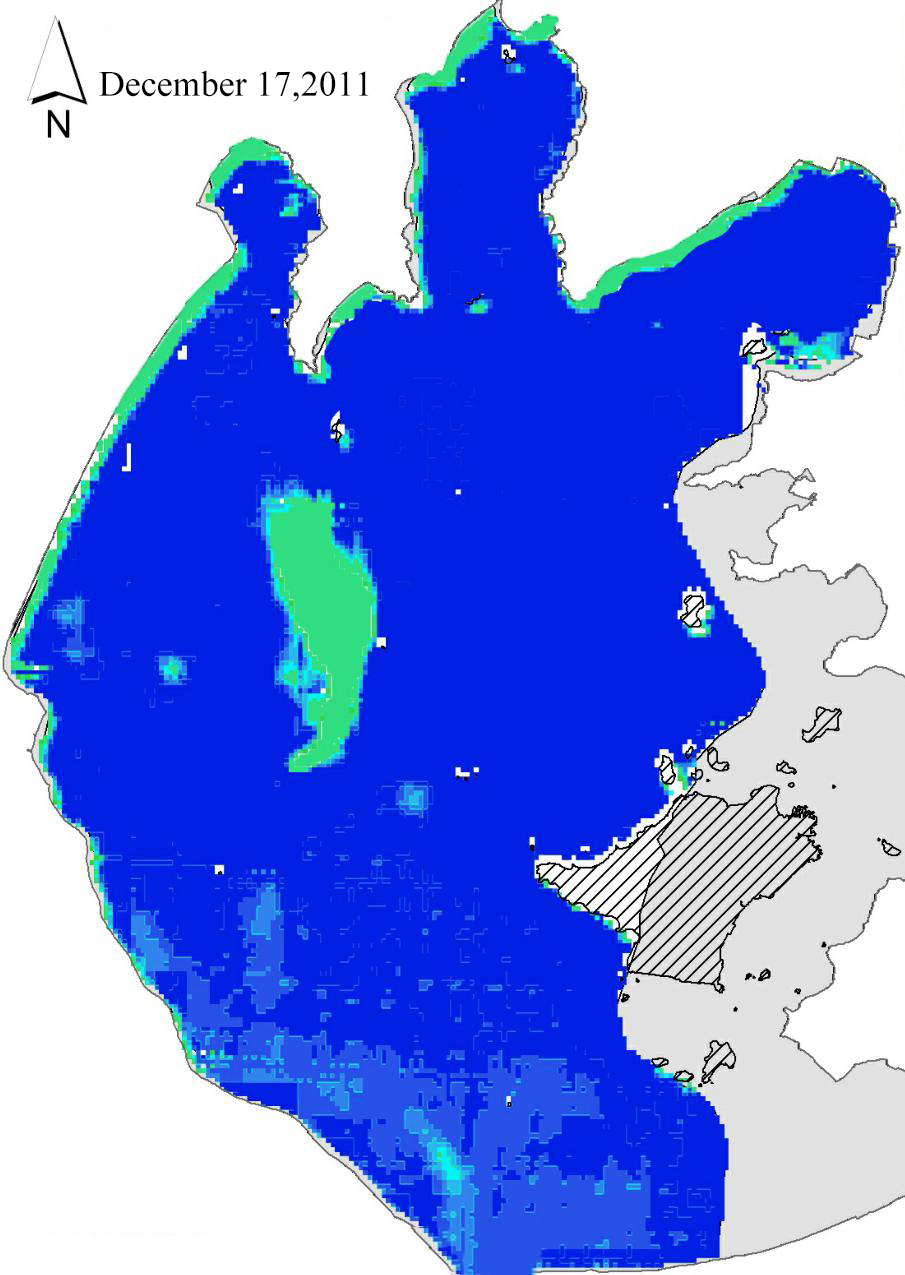

Supplement: Supplemental Information 6 [file peerj-cs-09-1292-s006.zip › 0/20111217_taihu_cla.jpg]

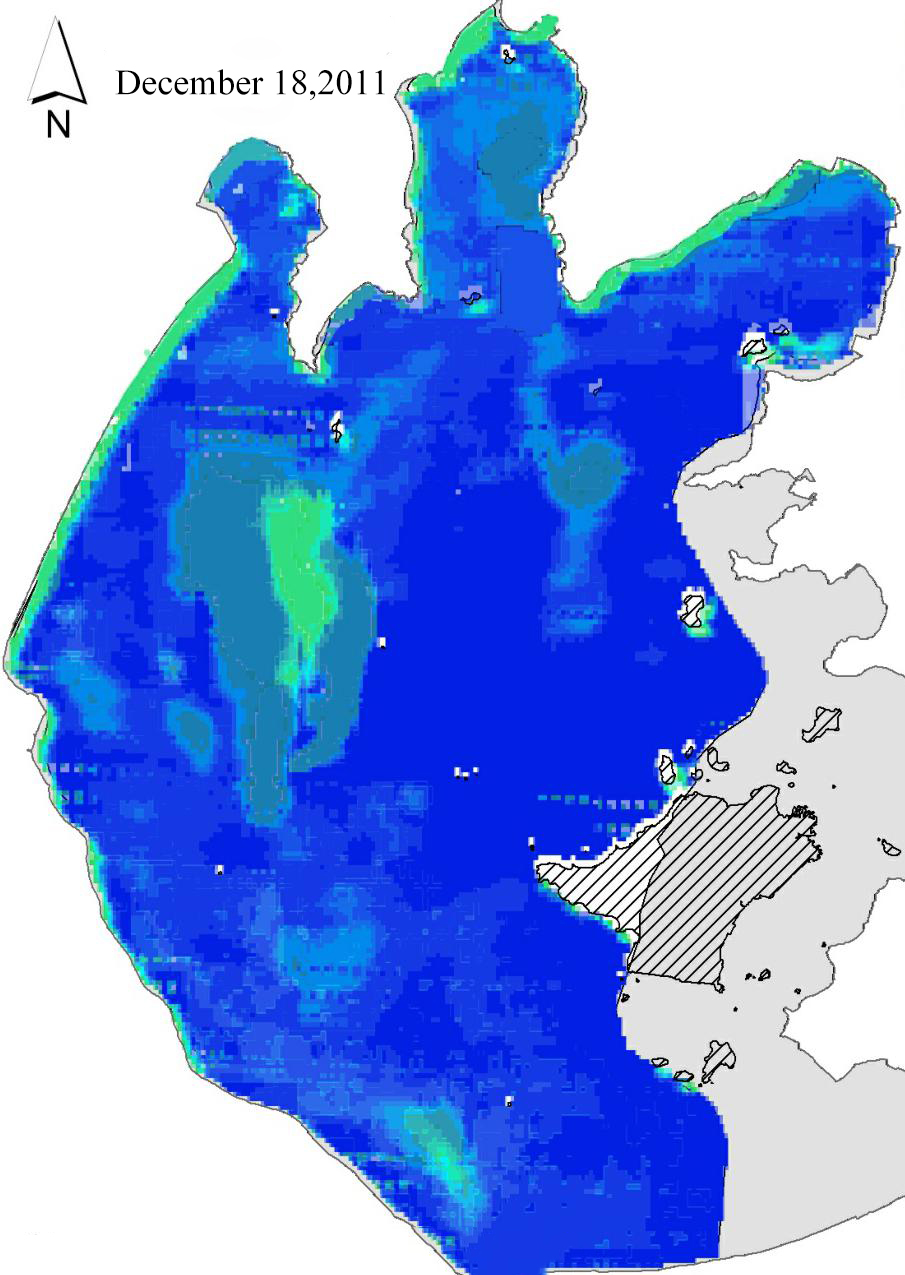

Supplement: Supplemental Information 6 [file peerj-cs-09-1292-s006.zip › 0/20111218_taihu_cla.jpg]

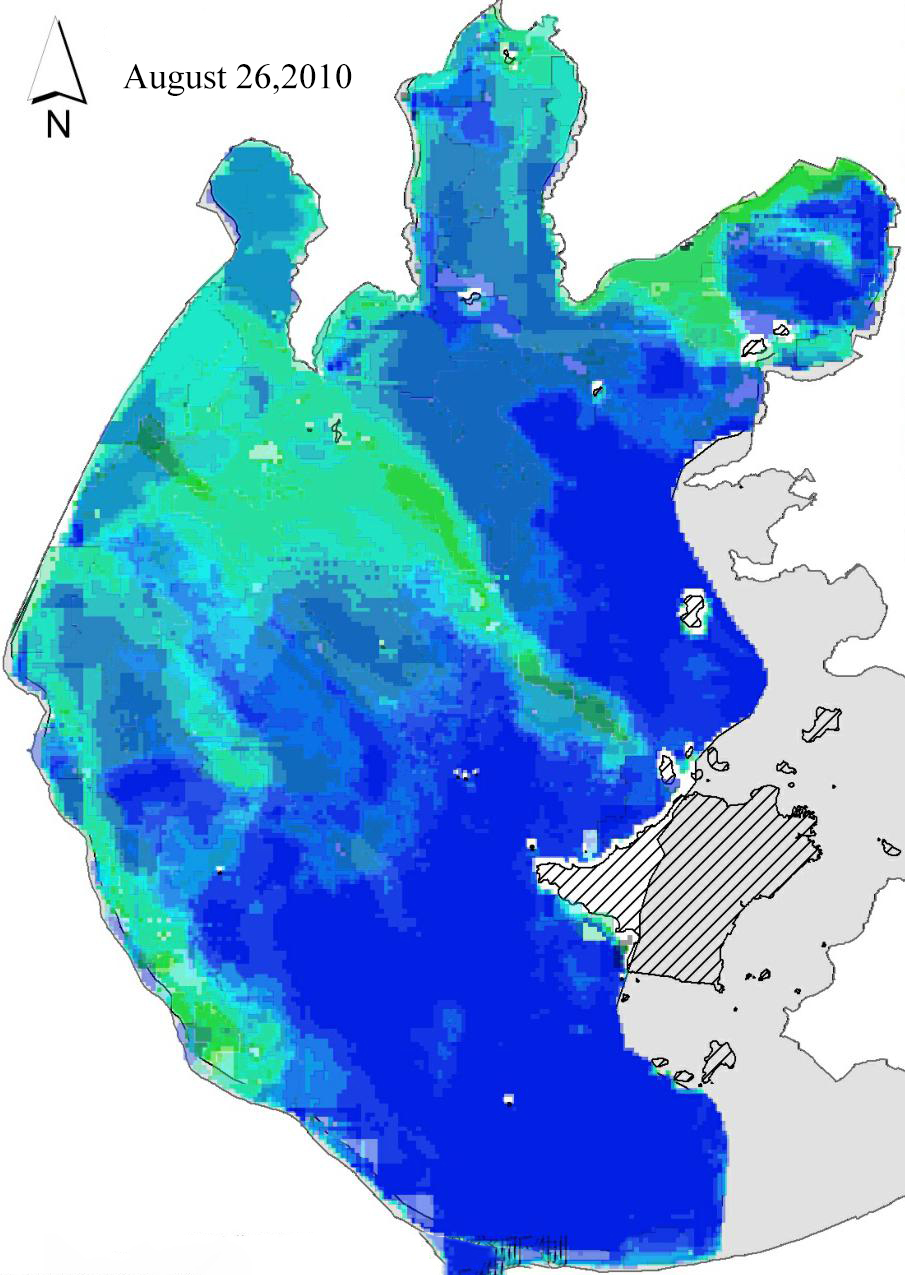

Supplement: Supplemental Information 7 — The data are remote sensing images of chlorophyll a concentration after data scale unification, remote sensing image repair, and time series filling. Remote sensing images of 30 consecutive moments were used as input to the 3D-GAN model. [file peerj-cs-09-1292-s007.zip › 201008260245.jpg]

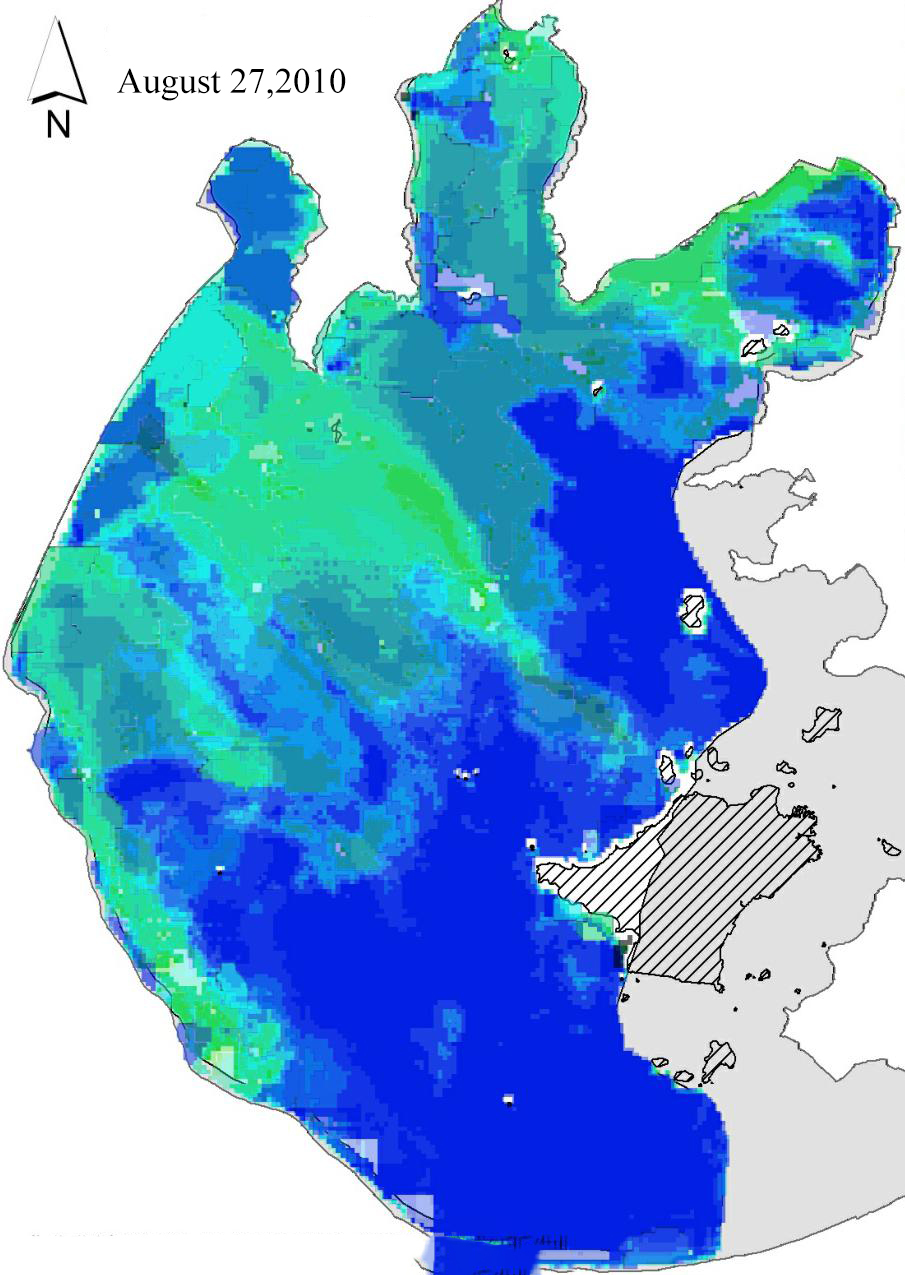

Supplement: Supplemental Information 7 — The data are remote sensing images of chlorophyll a concentration after data scale unification, remote sensing image repair, and time series filling. Remote sensing images of 30 consecutive moments were used as input to the 3D-GAN model. [file peerj-cs-09-1292-s007.zip › 201008270245.jpg]

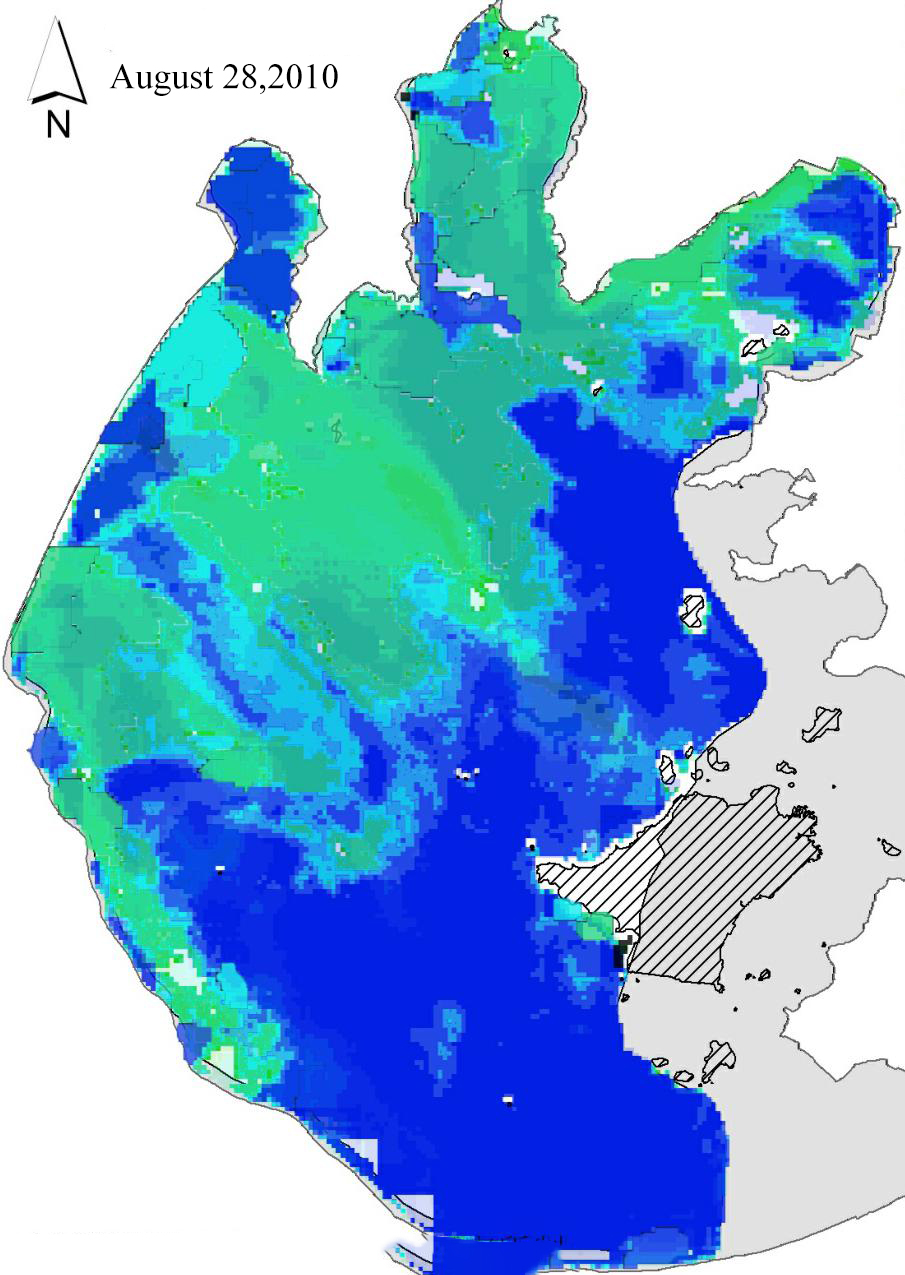

Supplement: Supplemental Information 7 — The data are remote sensing images of chlorophyll a concentration after data scale unification, remote sensing image repair, and time series filling. Remote sensing images of 30 consecutive moments were used as input to the 3D-GAN model. [file peerj-cs-09-1292-s007.zip › 201008280245.jpg]

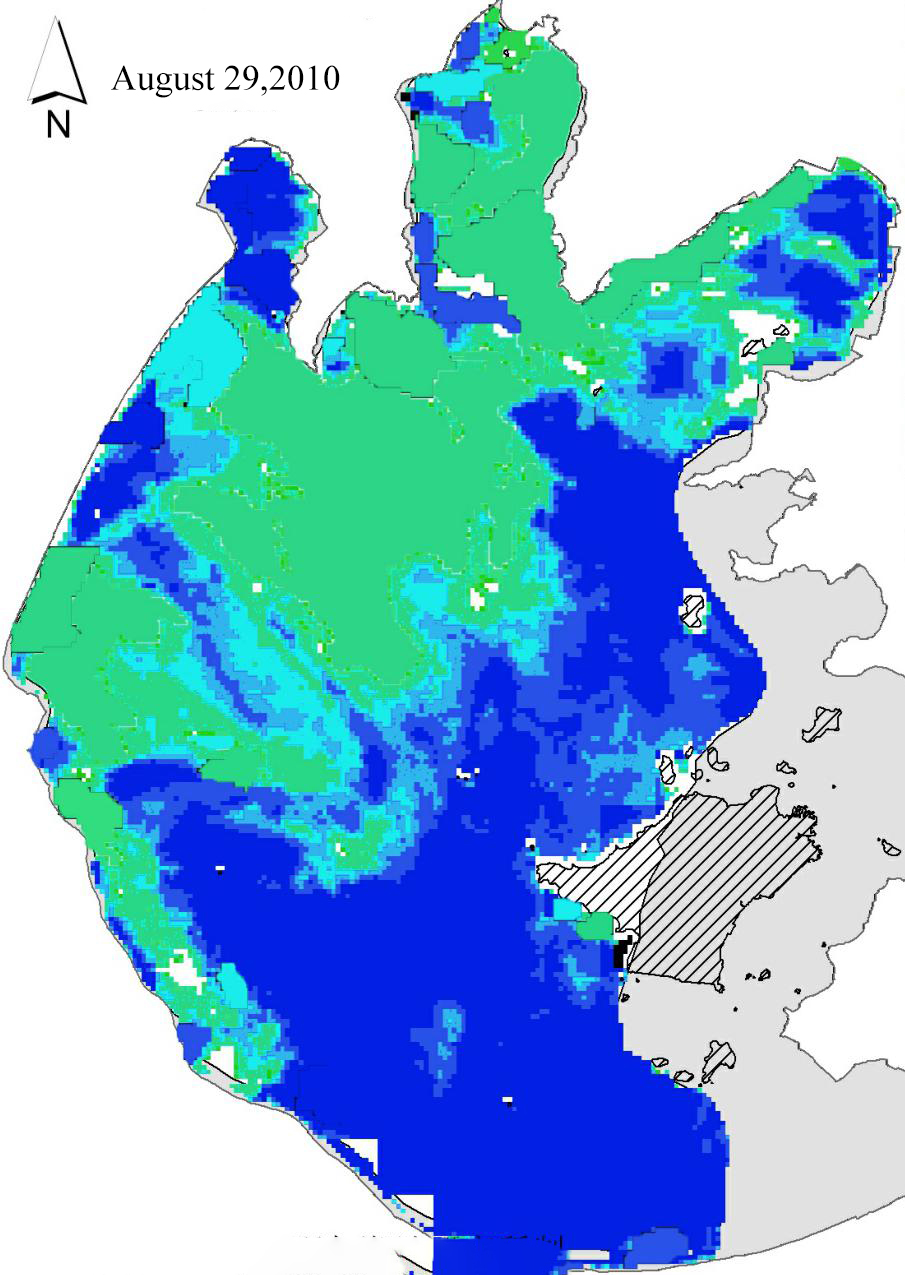

Supplement: Supplemental Information 7 — The data are remote sensing images of chlorophyll a concentration after data scale unification, remote sensing image repair, and time series filling. Remote sensing images of 30 consecutive moments were used as input to the 3D-GAN model. [file peerj-cs-09-1292-s007.zip › 201008290245.jpg]

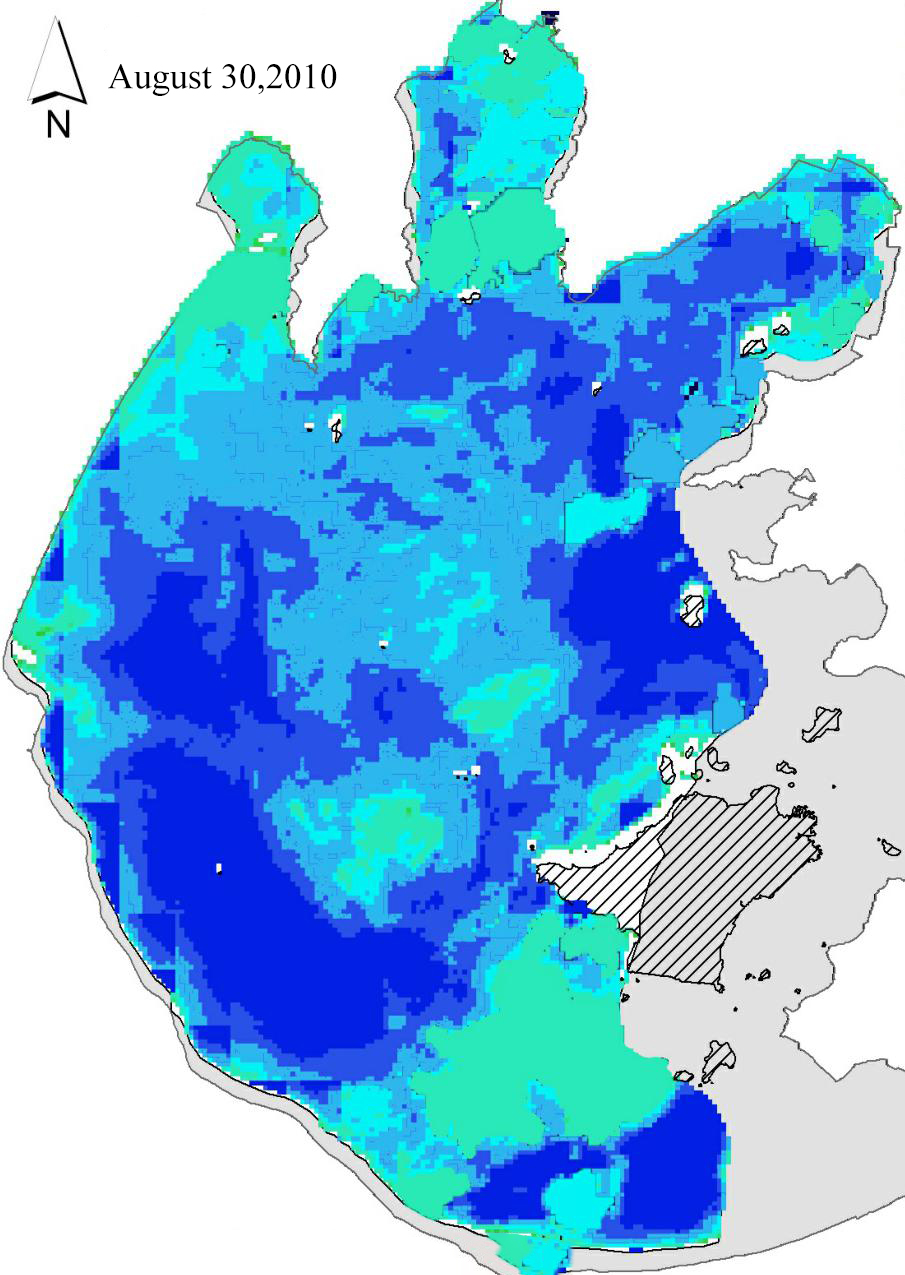

Supplement: Supplemental Information 7 — The data are remote sensing images of chlorophyll a concentration after data scale unification, remote sensing image repair, and time series filling. Remote sensing images of 30 consecutive moments were used as input to the 3D-GAN model. [file peerj-cs-09-1292-s007.zip › 201008300245.jpg]

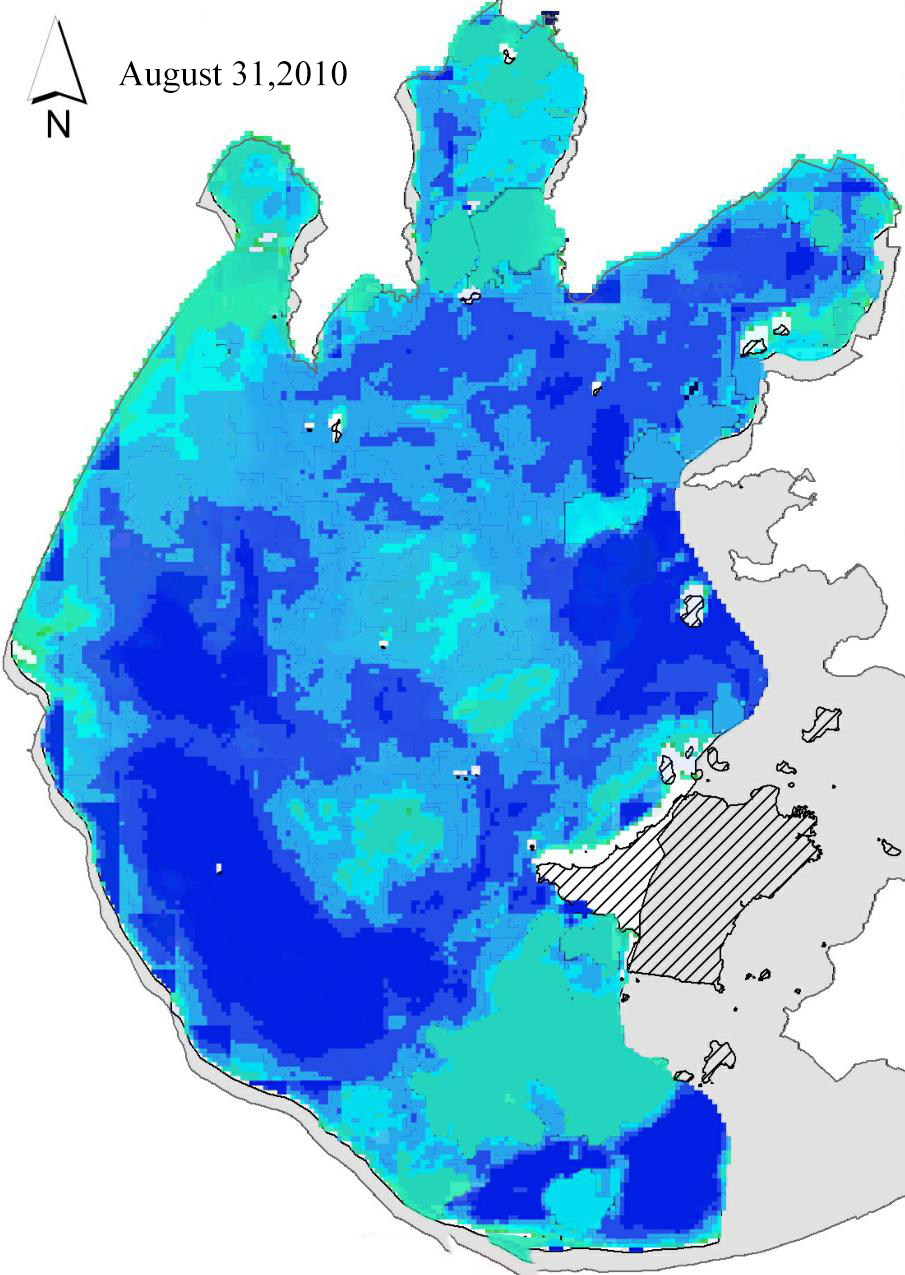

Supplement: Supplemental Information 7 — The data are remote sensing images of chlorophyll a concentration after data scale unification, remote sensing image repair, and time series filling. Remote sensing images of 30 consecutive moments were used as input to the 3D-GAN model. [file peerj-cs-09-1292-s007.zip › 201008310245.jpg]

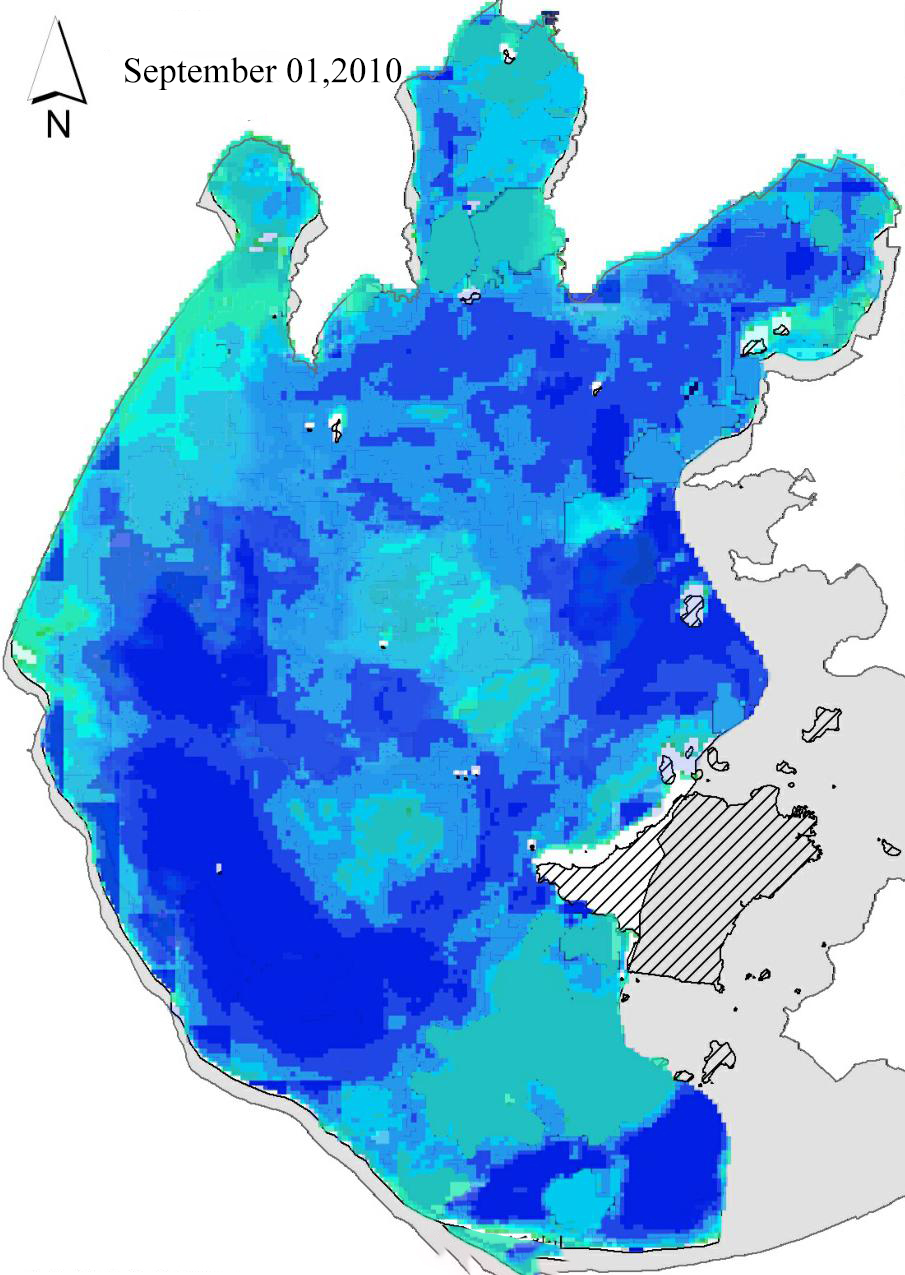

Supplement: Supplemental Information 7 — The data are remote sensing images of chlorophyll a concentration after data scale unification, remote sensing image repair, and time series filling. Remote sensing images of 30 consecutive moments were used as input to the 3D-GAN model. [file peerj-cs-09-1292-s007.zip › 201009010245.jpg]

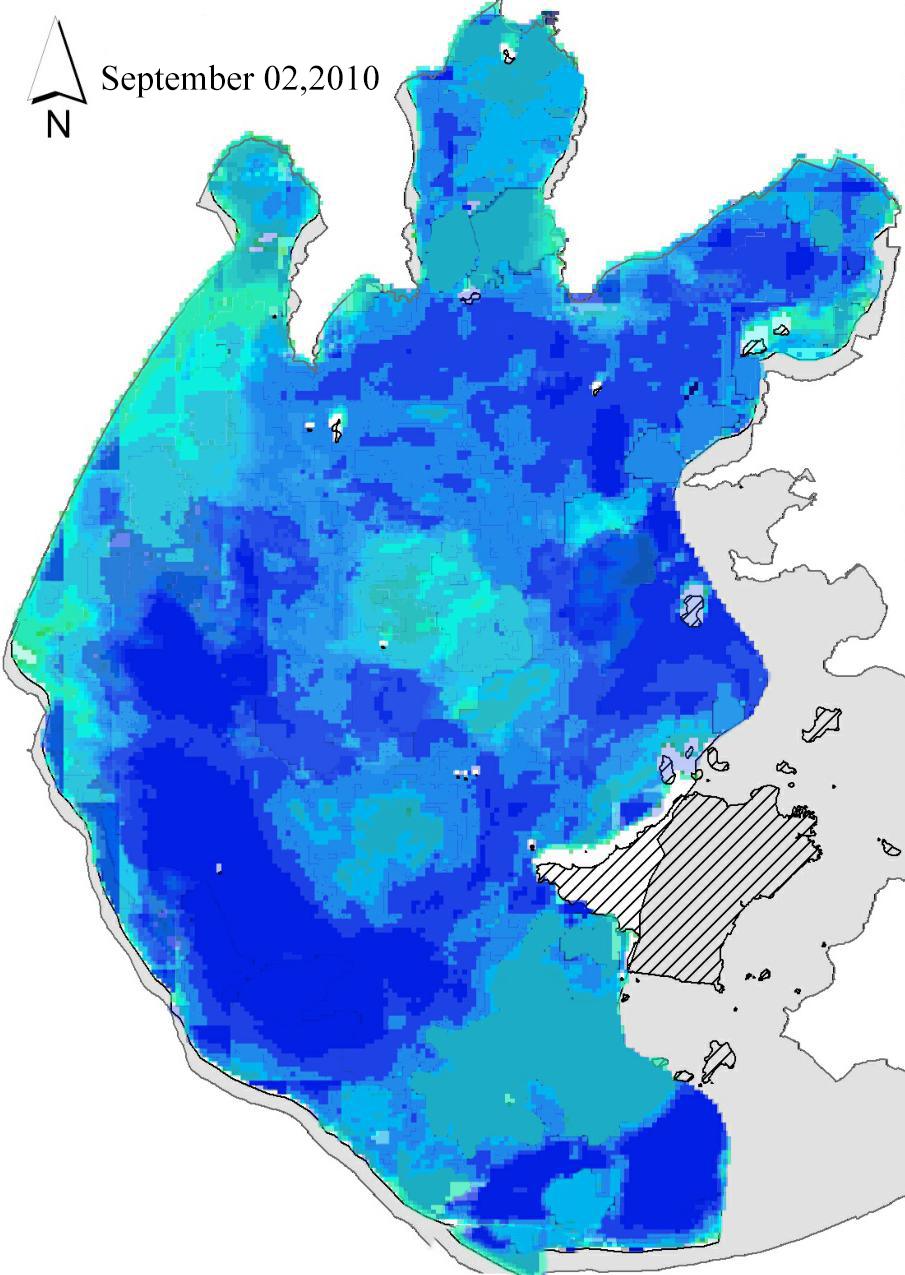

Supplement: Supplemental Information 7 — The data are remote sensing images of chlorophyll a concentration after data scale unification, remote sensing image repair, and time series filling. Remote sensing images of 30 consecutive moments were used as input to the 3D-GAN model. [file peerj-cs-09-1292-s007.zip › 201009020245.jpg]

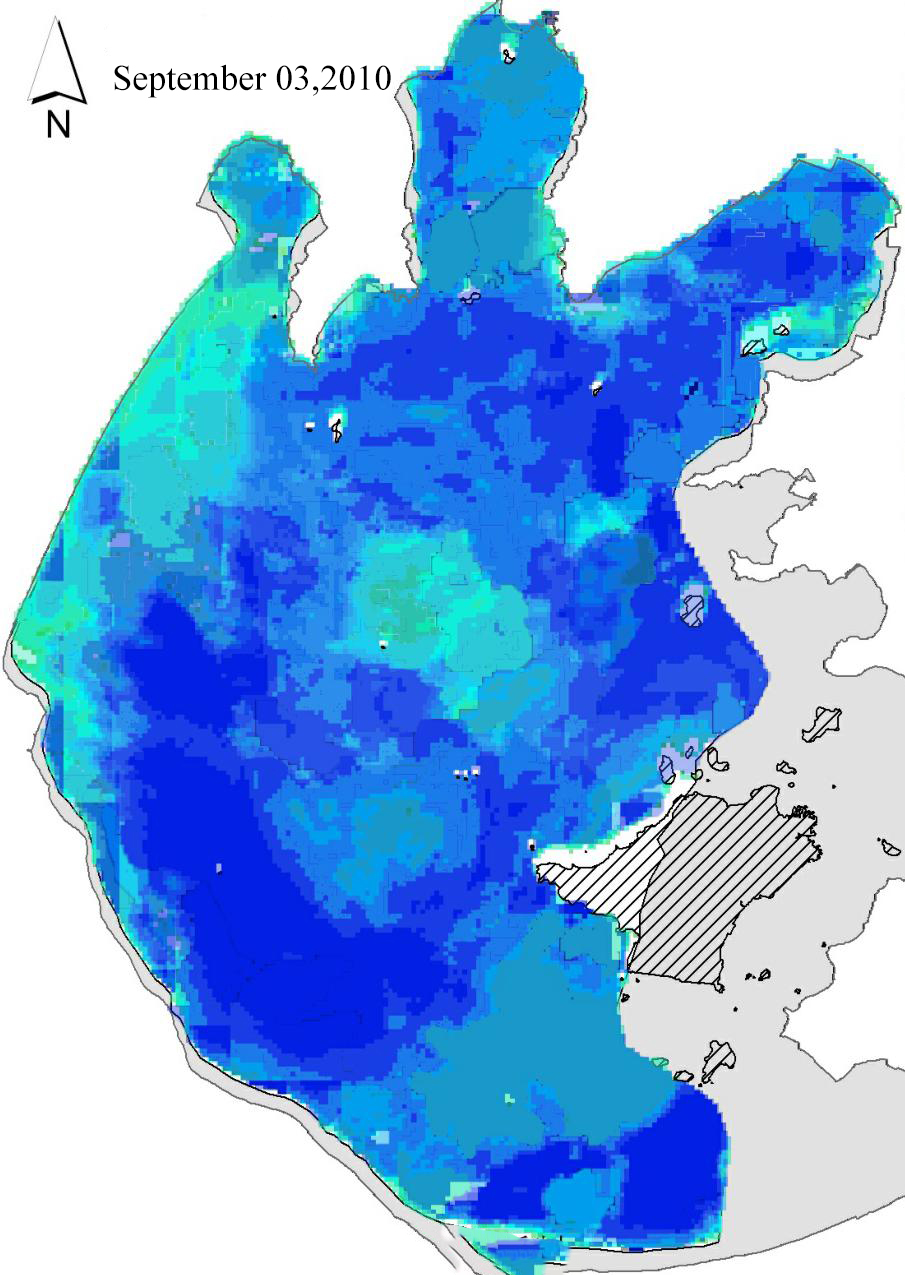

Supplement: Supplemental Information 7 — The data are remote sensing images of chlorophyll a concentration after data scale unification, remote sensing image repair, and time series filling. Remote sensing images of 30 consecutive moments were used as input to the 3D-GAN model. [file peerj-cs-09-1292-s007.zip › 201009030245.jpg]

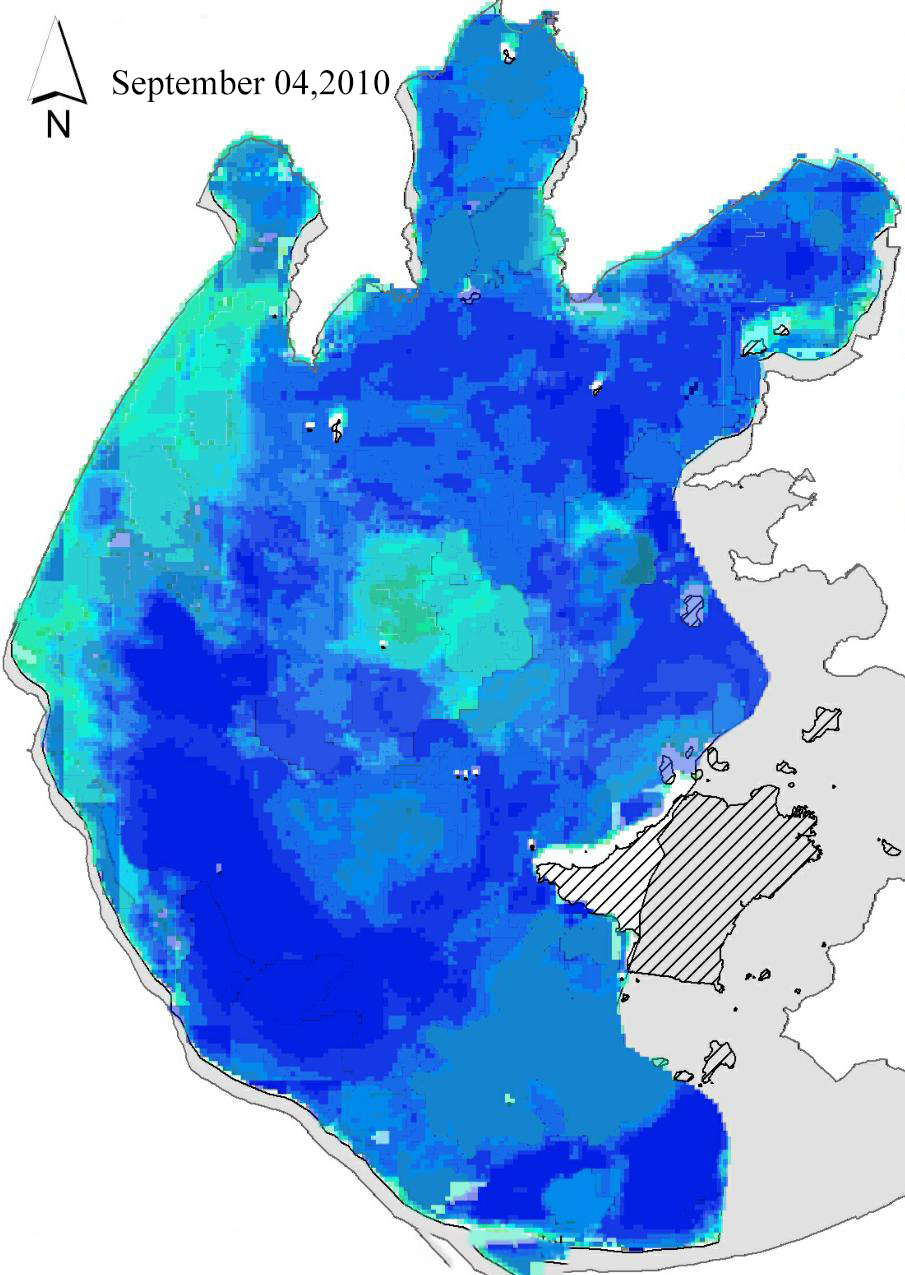

Supplement: Supplemental Information 7 — The data are remote sensing images of chlorophyll a concentration after data scale unification, remote sensing image repair, and time series filling. Remote sensing images of 30 consecutive moments were used as input to the 3D-GAN model. [file peerj-cs-09-1292-s007.zip › 201009040245.jpg]

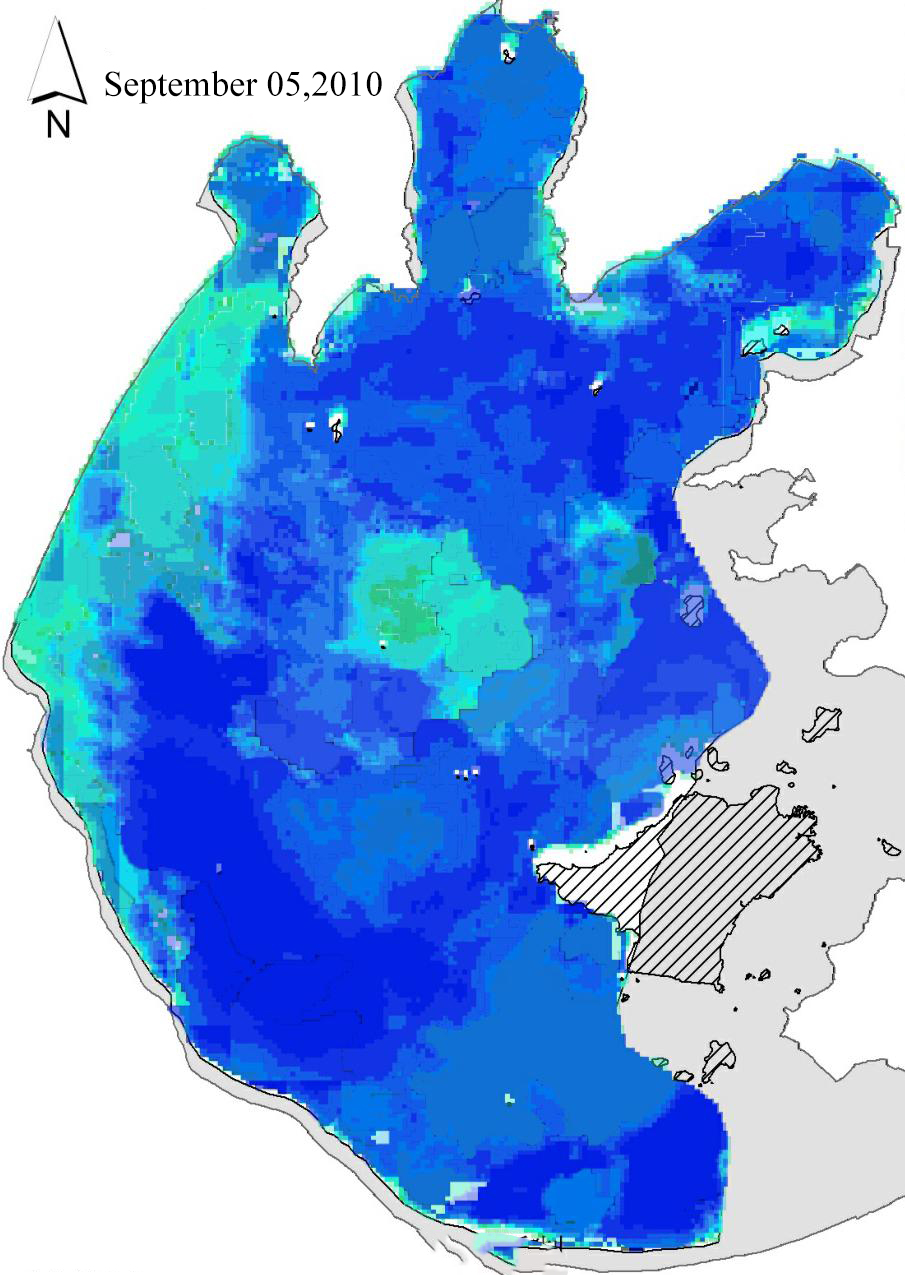

Supplement: Supplemental Information 7 — The data are remote sensing images of chlorophyll a concentration after data scale unification, remote sensing image repair, and time series filling. Remote sensing images of 30 consecutive moments were used as input to the 3D-GAN model. [file peerj-cs-09-1292-s007.zip › 201009050245.jpg]

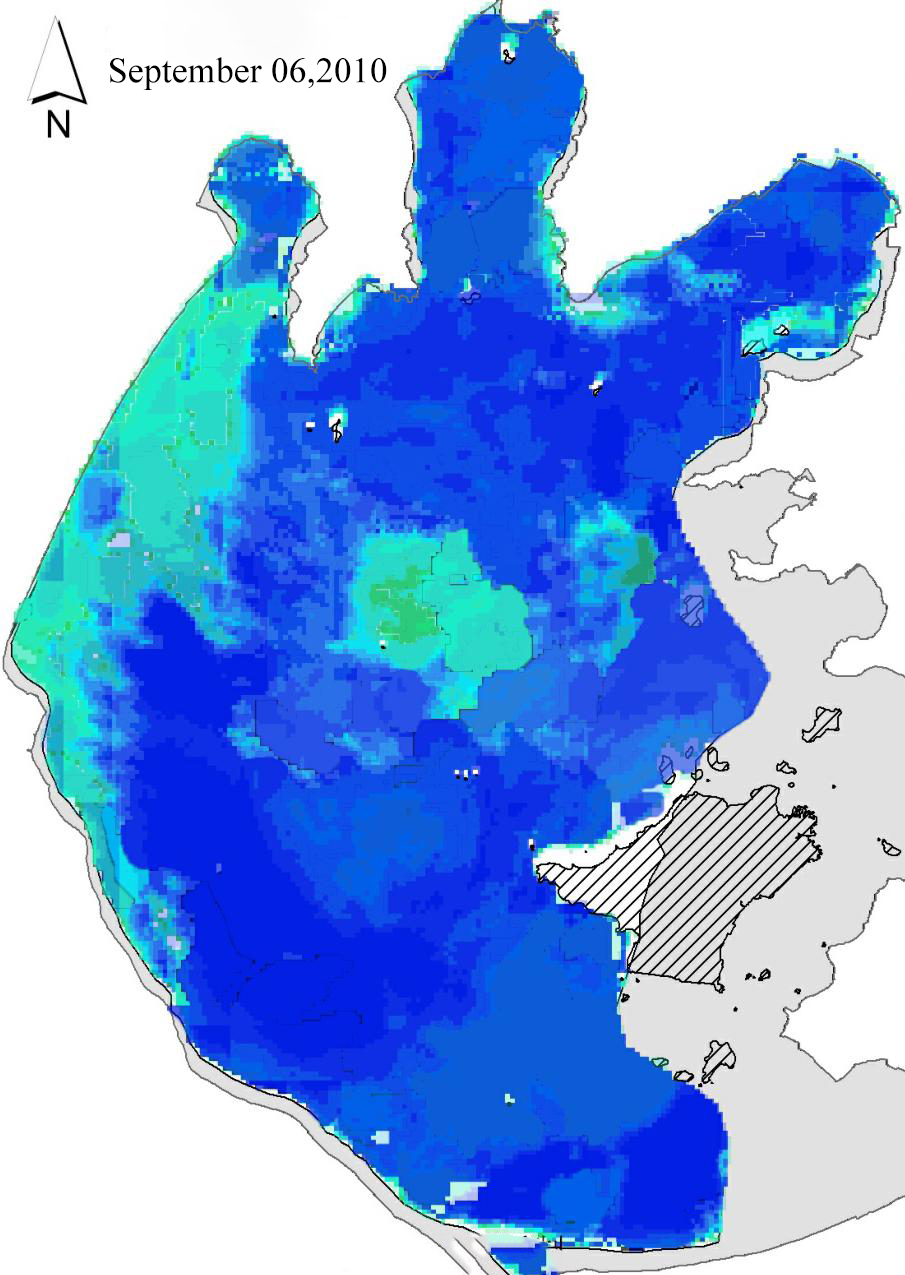

Supplement: Supplemental Information 7 — The data are remote sensing images of chlorophyll a concentration after data scale unification, remote sensing image repair, and time series filling. Remote sensing images of 30 consecutive moments were used as input to the 3D-GAN model. [file peerj-cs-09-1292-s007.zip › 201009060245.jpg]

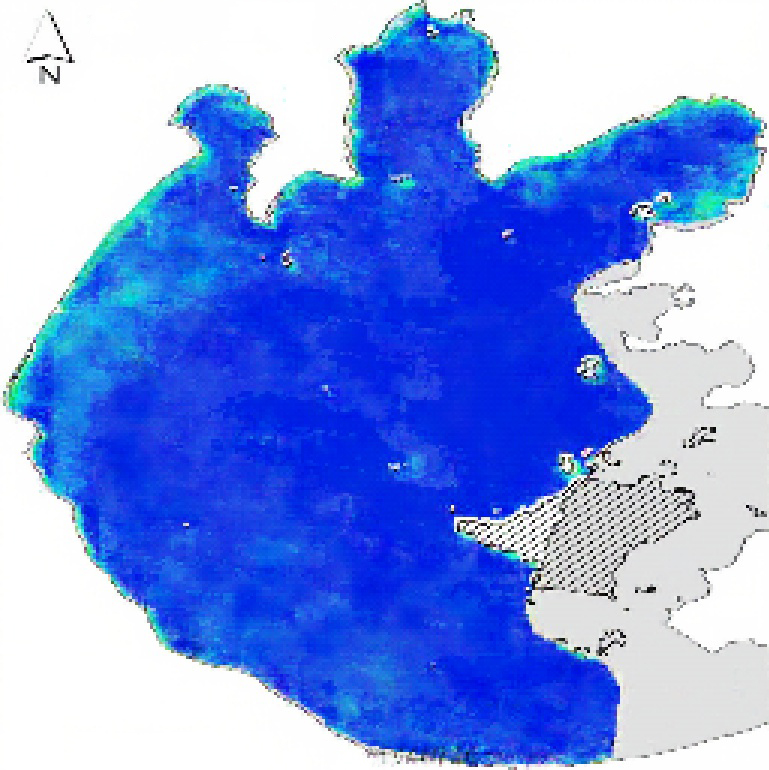

Supplement: Supplemental Information 8 — The data are remote sensing images of chlorophyll a concentration after data scale unification, remote sensing image repair, and time series filling. Remote sensing images of 30 consecutive moments were used as input to the 3D-GAN model. [file peerj-cs-09-1292-s008.zip › 412.jpg]

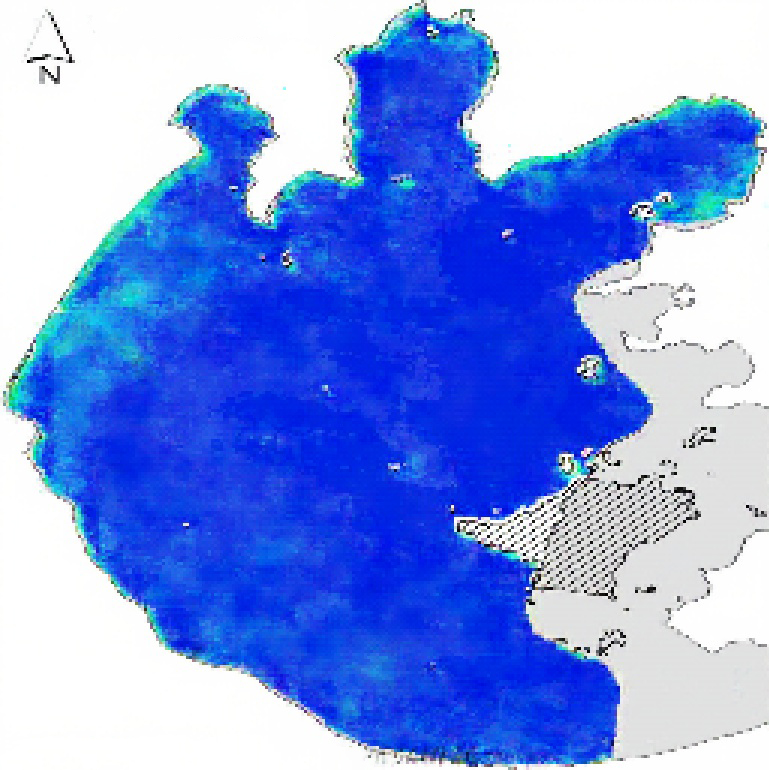

Supplement: Supplemental Information 8 — The data are remote sensing images of chlorophyll a concentration after data scale unification, remote sensing image repair, and time series filling. Remote sensing images of 30 consecutive moments were used as input to the 3D-GAN model. [file peerj-cs-09-1292-s008.zip › 413.jpg]

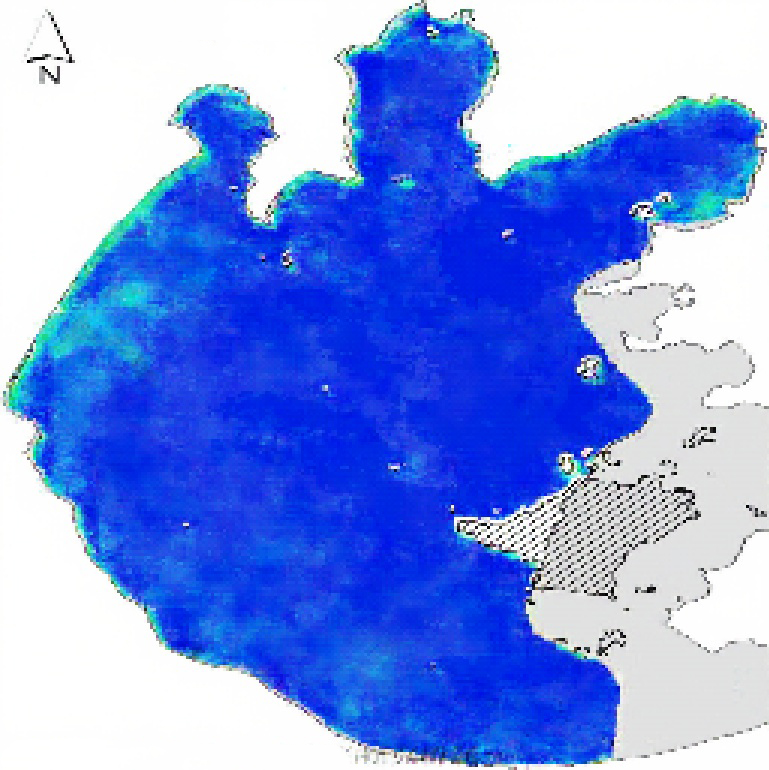

Supplement: Supplemental Information 8 — The data are remote sensing images of chlorophyll a concentration after data scale unification, remote sensing image repair, and time series filling. Remote sensing images of 30 consecutive moments were used as input to the 3D-GAN model. [file peerj-cs-09-1292-s008.zip › 414.jpg]

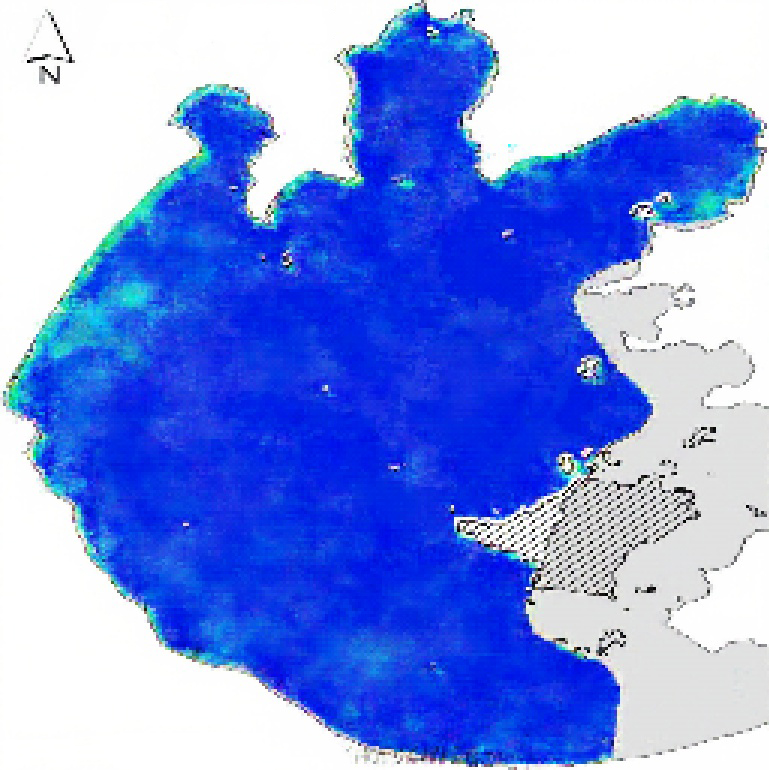

Supplement: Supplemental Information 8 — The data are remote sensing images of chlorophyll a concentration after data scale unification, remote sensing image repair, and time series filling. Remote sensing images of 30 consecutive moments were used as input to the 3D-GAN model. [file peerj-cs-09-1292-s008.zip › 415.jpg]

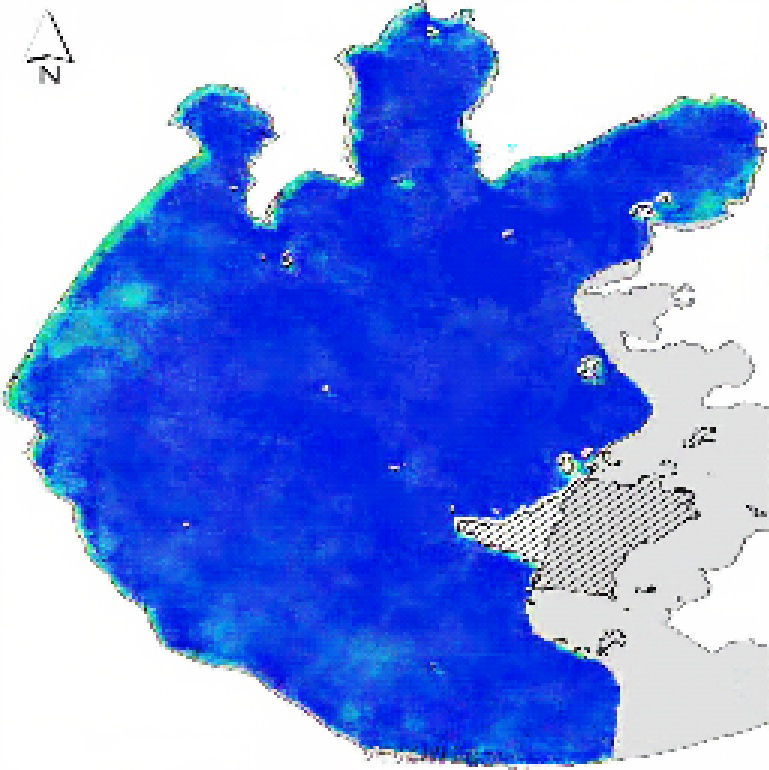

Supplement: Supplemental Information 8 — The data are remote sensing images of chlorophyll a concentration after data scale unification, remote sensing image repair, and time series filling. Remote sensing images of 30 consecutive moments were used as input to the 3D-GAN model. [file peerj-cs-09-1292-s008.zip › 416.jpg]

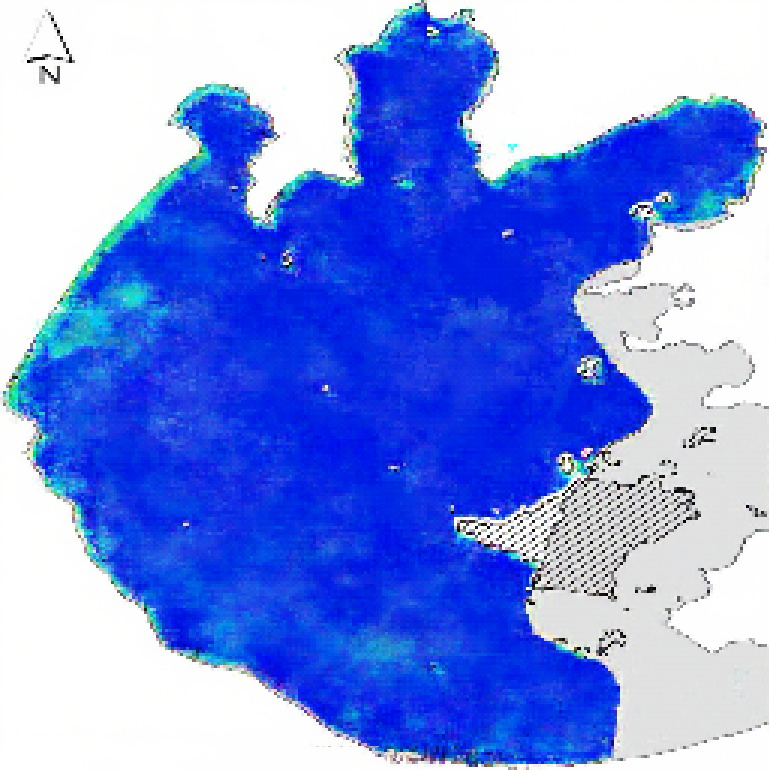

Supplement: Supplemental Information 8 — The data are remote sensing images of chlorophyll a concentration after data scale unification, remote sensing image repair, and time series filling. Remote sensing images of 30 consecutive moments were used as input to the 3D-GAN model. [file peerj-cs-09-1292-s008.zip › 417.jpg]

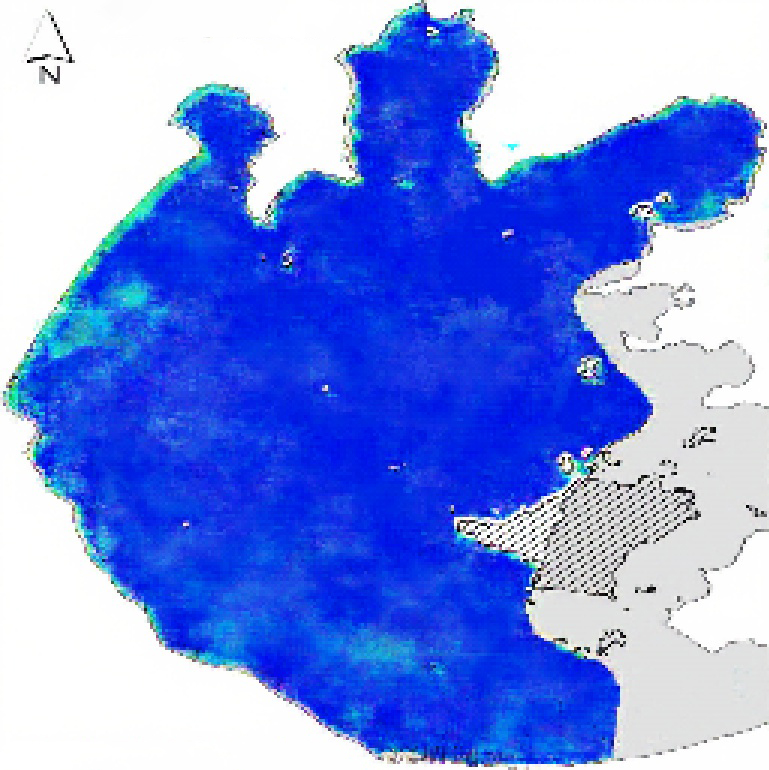

Supplement: Supplemental Information 8 — The data are remote sensing images of chlorophyll a concentration after data scale unification, remote sensing image repair, and time series filling. Remote sensing images of 30 consecutive moments were used as input to the 3D-GAN model. [file peerj-cs-09-1292-s008.zip › 418.jpg]

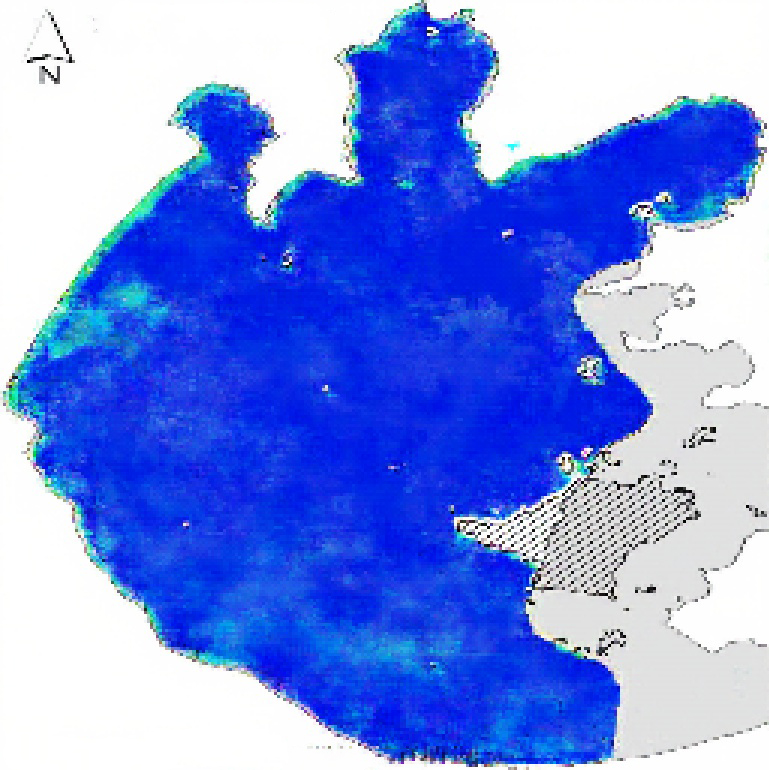

Supplement: Supplemental Information 8 — The data are remote sensing images of chlorophyll a concentration after data scale unification, remote sensing image repair, and time series filling. Remote sensing images of 30 consecutive moments were used as input to the 3D-GAN model. [file peerj-cs-09-1292-s008.zip › 419.jpg]

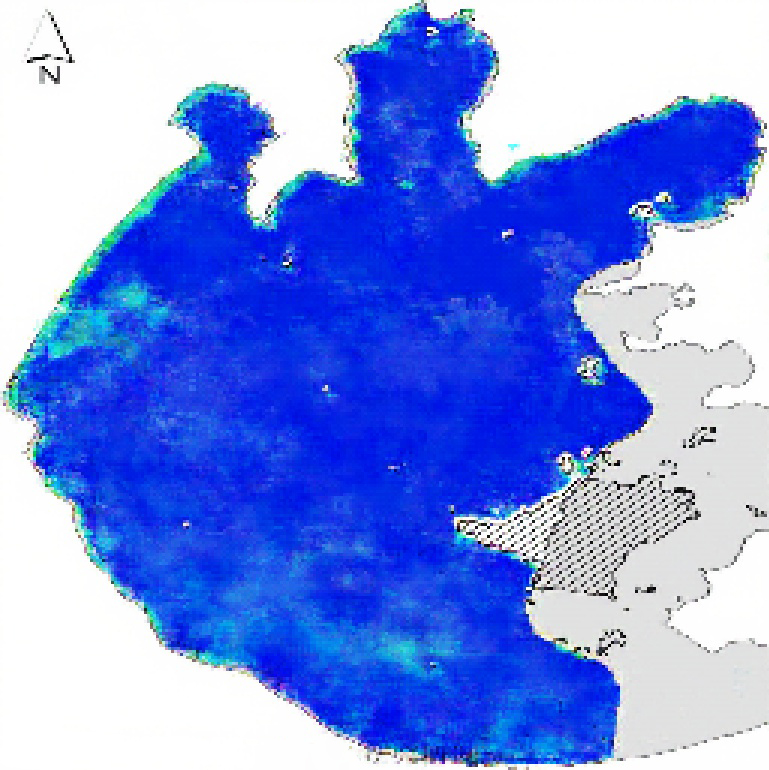

Supplement: Supplemental Information 8 — The data are remote sensing images of chlorophyll a concentration after data scale unification, remote sensing image repair, and time series filling. Remote sensing images of 30 consecutive moments were used as input to the 3D-GAN model. [file peerj-cs-09-1292-s008.zip › 420.jpg]

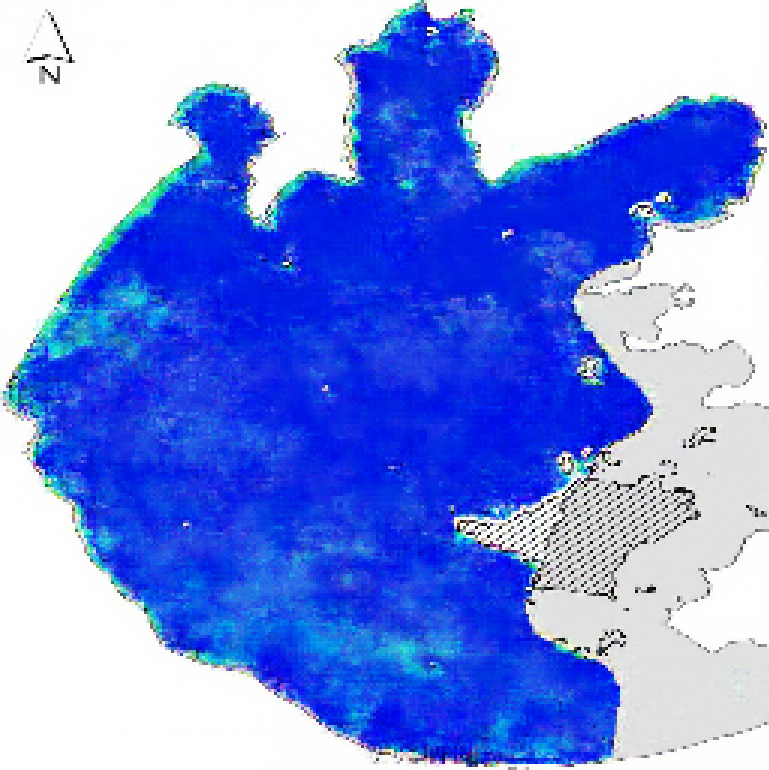

Supplement: Supplemental Information 8 — The data are remote sensing images of chlorophyll a concentration after data scale unification, remote sensing image repair, and time series filling. Remote sensing images of 30 consecutive moments were used as input to the 3D-GAN model. [file peerj-cs-09-1292-s008.zip › 421.jpg]

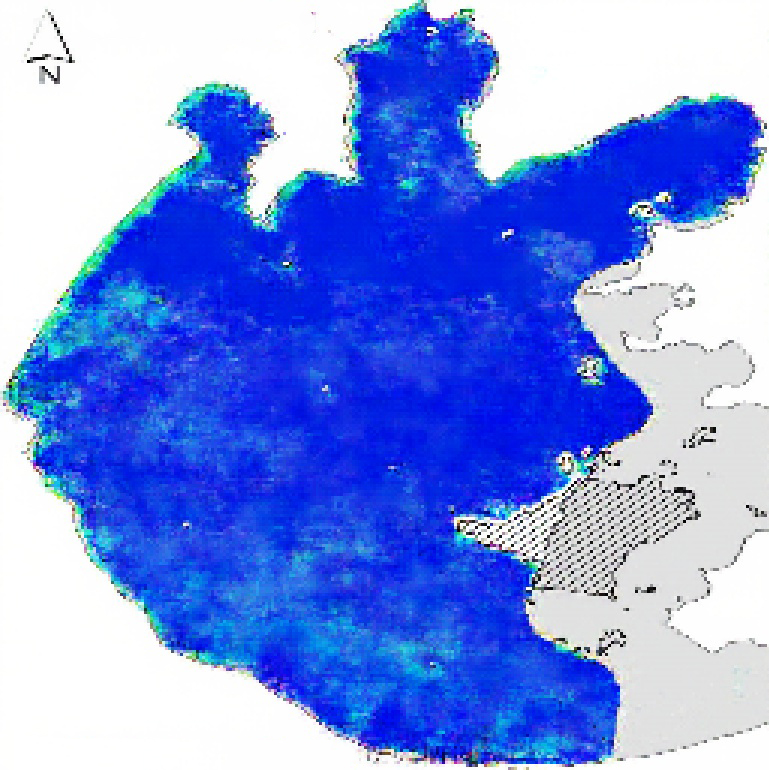

Supplement: Supplemental Information 8 — The data are remote sensing images of chlorophyll a concentration after data scale unification, remote sensing image repair, and time series filling. Remote sensing images of 30 consecutive moments were used as input to the 3D-GAN model. [file peerj-cs-09-1292-s008.zip › 422.jpg]

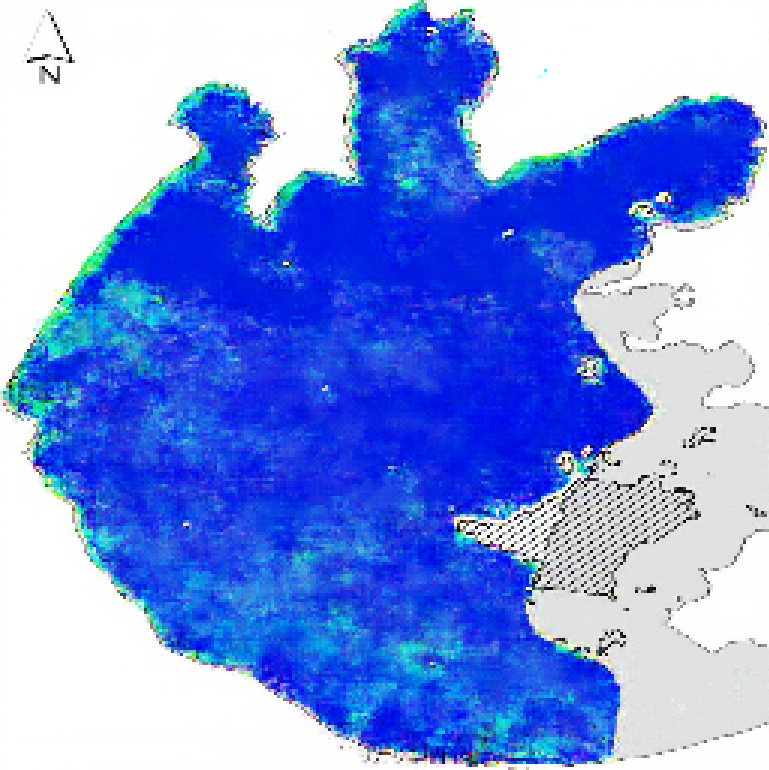

Supplement: Supplemental Information 8 — The data are remote sensing images of chlorophyll a concentration after data scale unification, remote sensing image repair, and time series filling. Remote sensing images of 30 consecutive moments were used as input to the 3D-GAN model. [file peerj-cs-09-1292-s008.zip › 423.jpg]

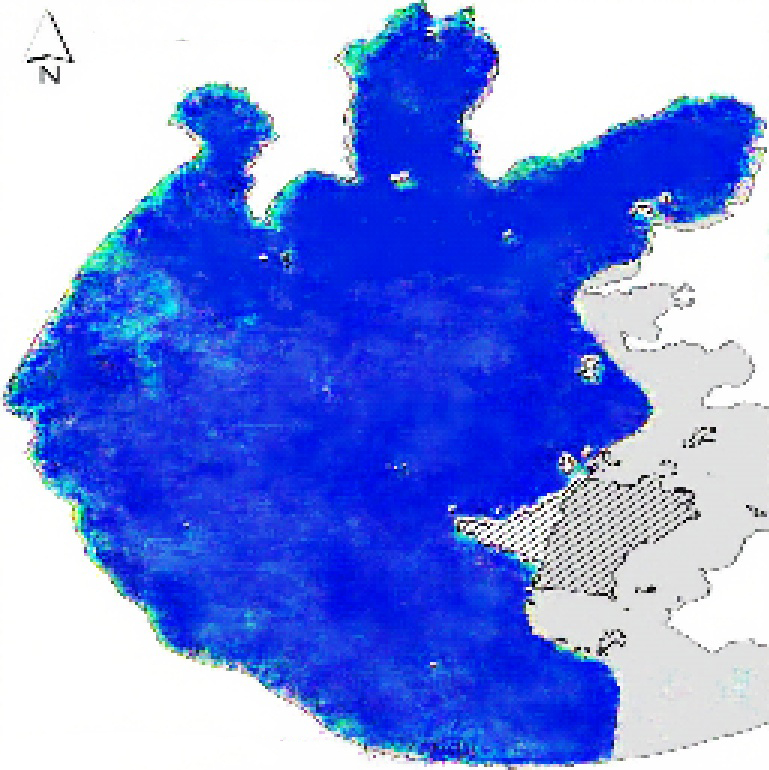

Supplement: Supplemental Information 8 — The data are remote sensing images of chlorophyll a concentration after data scale unification, remote sensing image repair, and time series filling. Remote sensing images of 30 consecutive moments were used as input to the 3D-GAN model. [file peerj-cs-09-1292-s008.zip › 424.jpg]

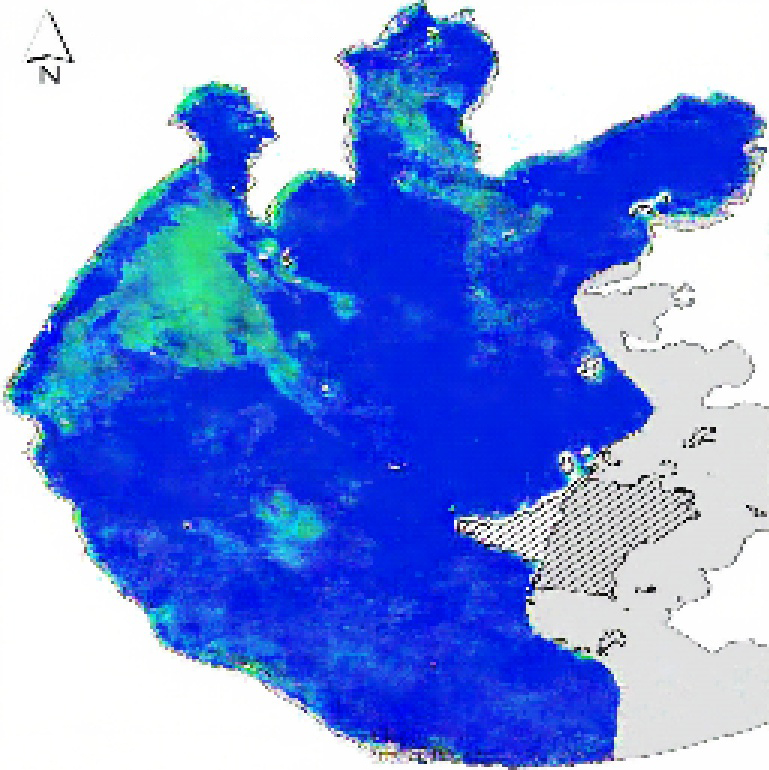

Supplement: Supplemental Information 8 — The data are remote sensing images of chlorophyll a concentration after data scale unification, remote sensing image repair, and time series filling. Remote sensing images of 30 consecutive moments were used as input to the 3D-GAN model. [file peerj-cs-09-1292-s008.zip › 425.jpg]

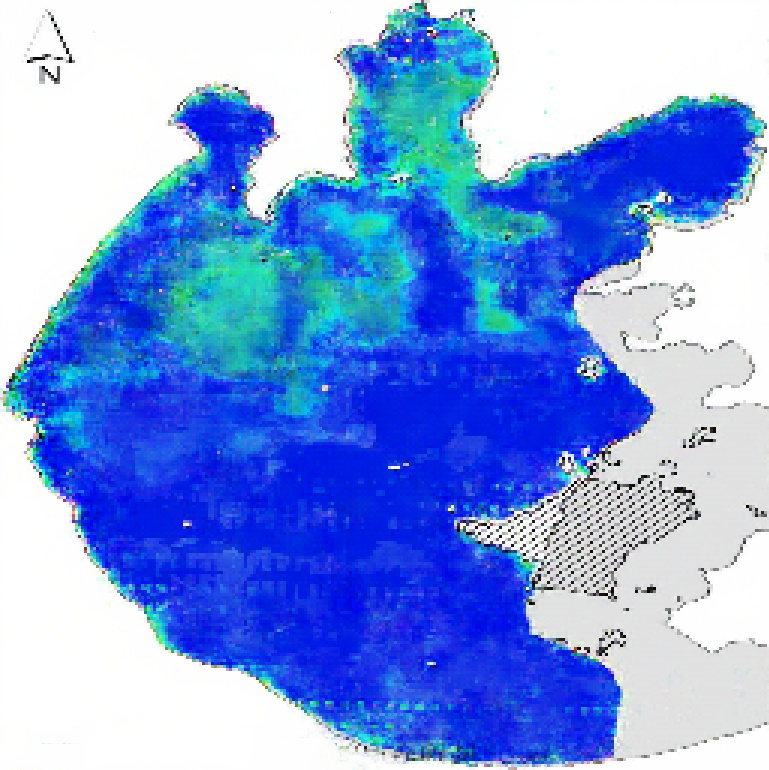

Supplement: Supplemental Information 8 — The data are remote sensing images of chlorophyll a concentration after data scale unification, remote sensing image repair, and time series filling. Remote sensing images of 30 consecutive moments were used as input to the 3D-GAN model. [file peerj-cs-09-1292-s008.zip › 426.jpg]

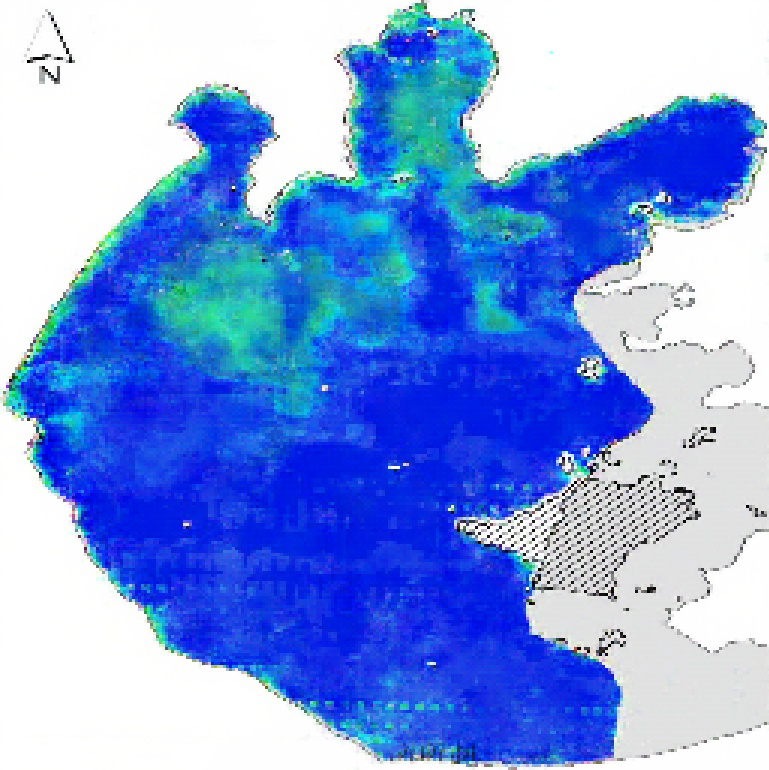

Supplement: Supplemental Information 8 — The data are remote sensing images of chlorophyll a concentration after data scale unification, remote sensing image repair, and time series filling. Remote sensing images of 30 consecutive moments were used as input to the 3D-GAN model. [file peerj-cs-09-1292-s008.zip › 427.jpg]

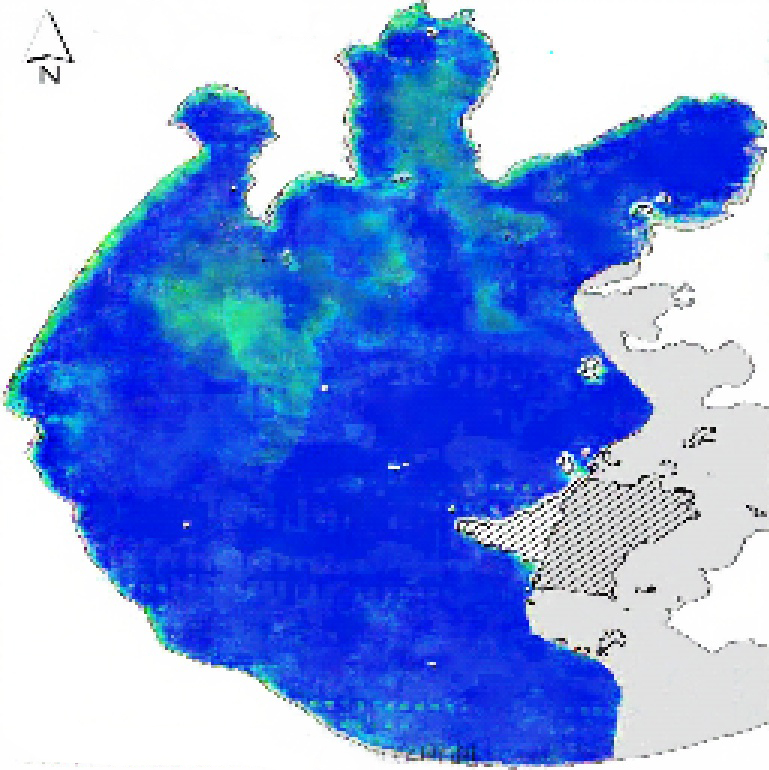

Supplement: Supplemental Information 8 — The data are remote sensing images of chlorophyll a concentration after data scale unification, remote sensing image repair, and time series filling. Remote sensing images of 30 consecutive moments were used as input to the 3D-GAN model. [file peerj-cs-09-1292-s008.zip › 428.jpg]

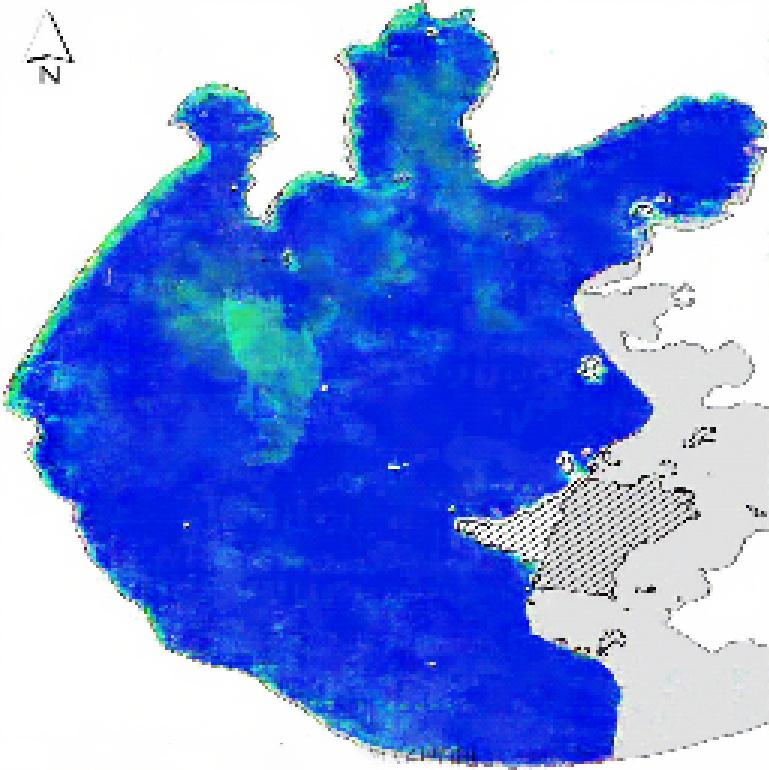

Supplement: Supplemental Information 8 — The data are remote sensing images of chlorophyll a concentration after data scale unification, remote sensing image repair, and time series filling. Remote sensing images of 30 consecutive moments were used as input to the 3D-GAN model. [file peerj-cs-09-1292-s008.zip › 429.jpg]

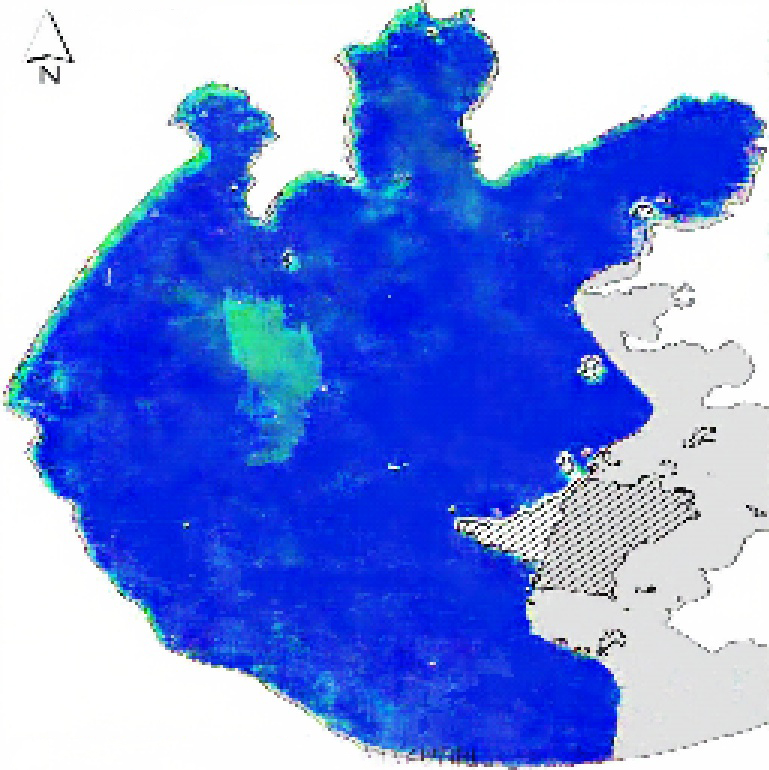

Supplement: Supplemental Information 8 — The data are remote sensing images of chlorophyll a concentration after data scale unification, remote sensing image repair, and time series filling. Remote sensing images of 30 consecutive moments were used as input to the 3D-GAN model. [file peerj-cs-09-1292-s008.zip › 430.jpg]

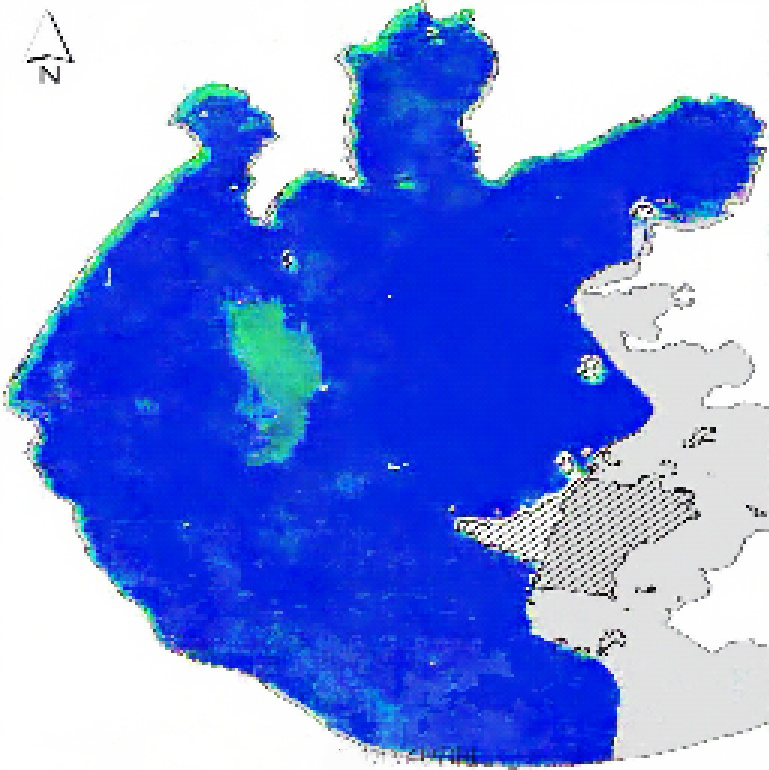

Supplement: Supplemental Information 8 — The data are remote sensing images of chlorophyll a concentration after data scale unification, remote sensing image repair, and time series filling. Remote sensing images of 30 consecutive moments were used as input to the 3D-GAN model. [file peerj-cs-09-1292-s008.zip › 431.jpg]

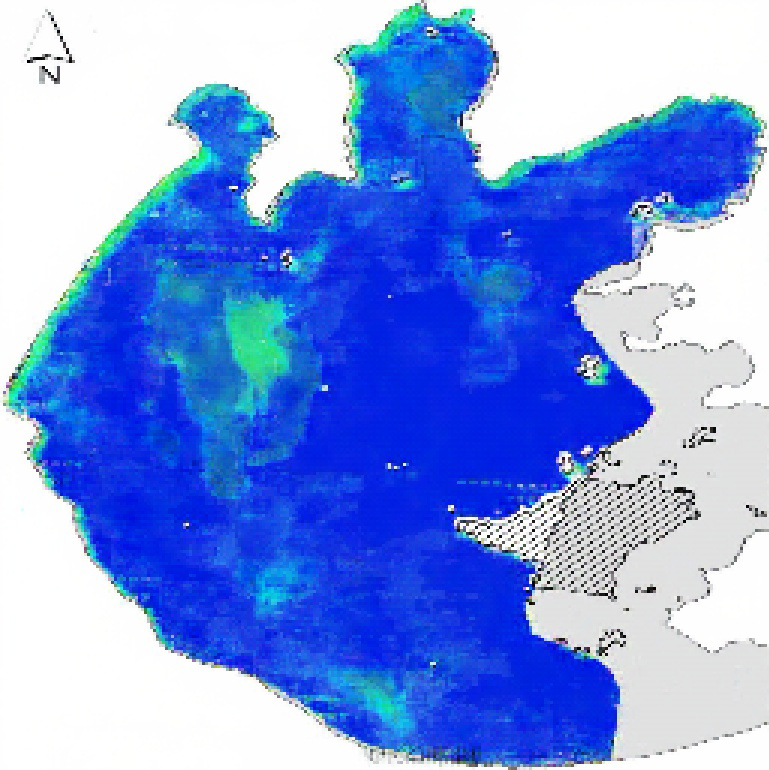

Supplement: Supplemental Information 8 — The data are remote sensing images of chlorophyll a concentration after data scale unification, remote sensing image repair, and time series filling. Remote sensing images of 30 consecutive moments were used as input to the 3D-GAN model. [file peerj-cs-09-1292-s008.zip › 432.jpg]

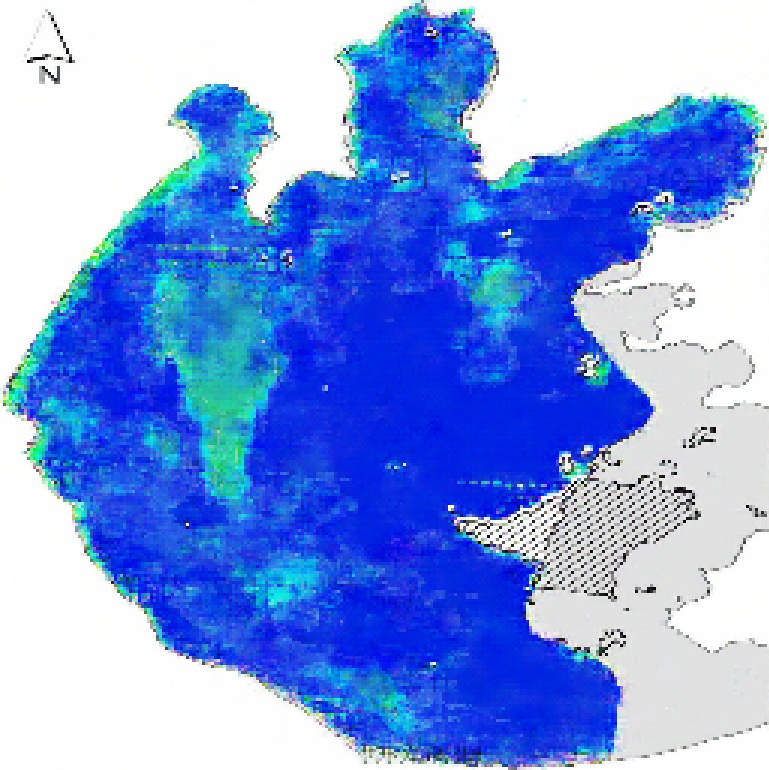

Supplement: Supplemental Information 8 — The data are remote sensing images of chlorophyll a concentration after data scale unification, remote sensing image repair, and time series filling. Remote sensing images of 30 consecutive moments were used as input to the 3D-GAN model. [file peerj-cs-09-1292-s008.zip › 433.jpg]

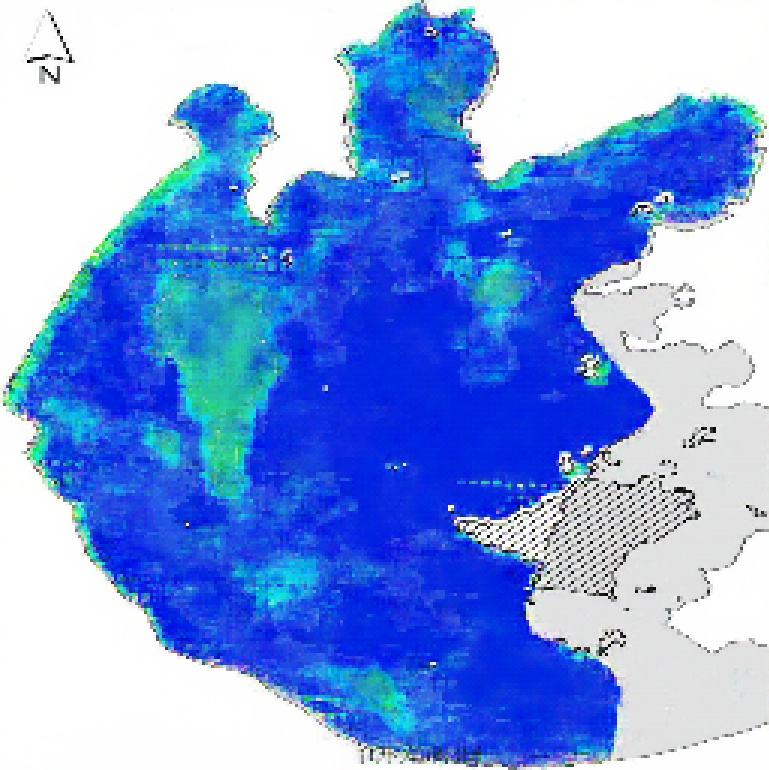

Supplement: Supplemental Information 8 — The data are remote sensing images of chlorophyll a concentration after data scale unification, remote sensing image repair, and time series filling. Remote sensing images of 30 consecutive moments were used as input to the 3D-GAN model. [file peerj-cs-09-1292-s008.zip › 434.jpg]

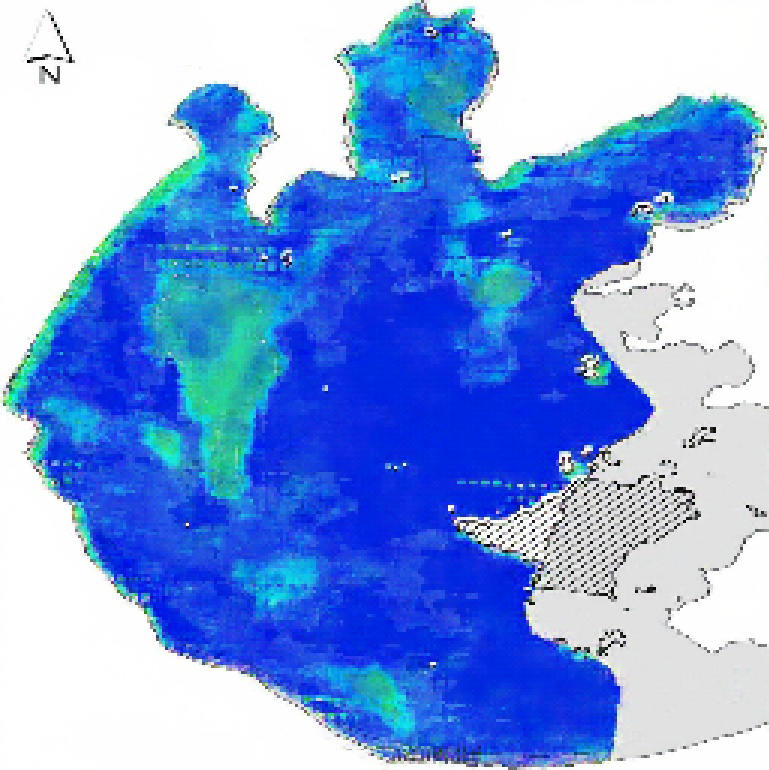

Supplement: Supplemental Information 8 — The data are remote sensing images of chlorophyll a concentration after data scale unification, remote sensing image repair, and time series filling. Remote sensing images of 30 consecutive moments were used as input to the 3D-GAN model. [file peerj-cs-09-1292-s008.zip › 435.jpg]

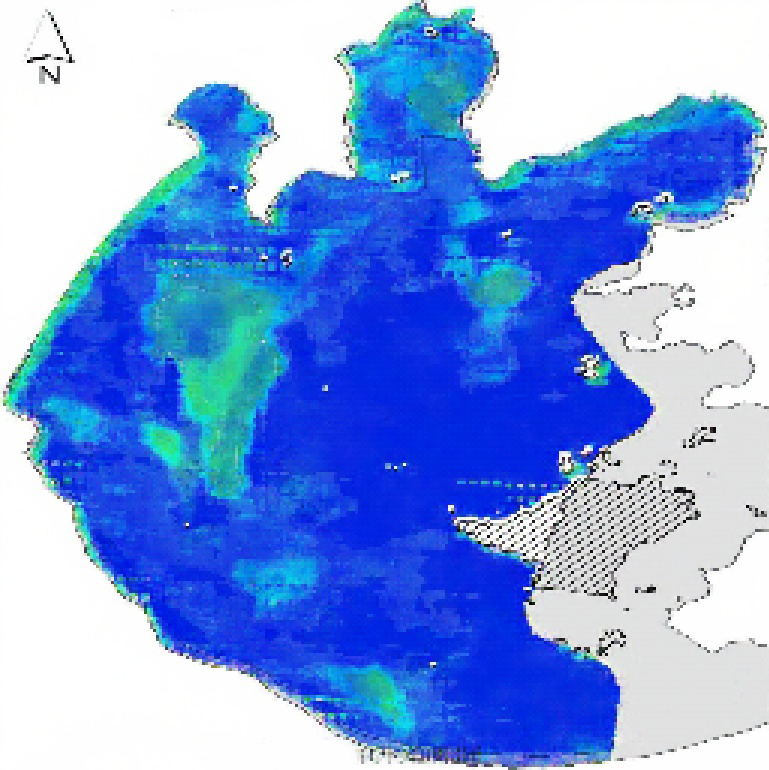

Supplement: Supplemental Information 8 — The data are remote sensing images of chlorophyll a concentration after data scale unification, remote sensing image repair, and time series filling. Remote sensing images of 30 consecutive moments were used as input to the 3D-GAN model. [file peerj-cs-09-1292-s008.zip › 436.jpg]

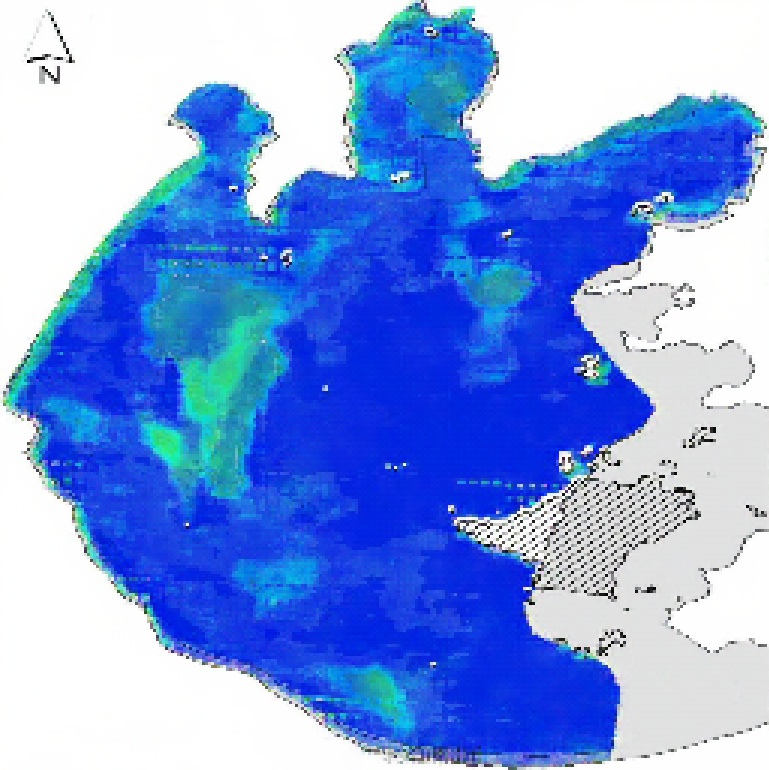

Supplement: Supplemental Information 8 — The data are remote sensing images of chlorophyll a concentration after data scale unification, remote sensing image repair, and time series filling. Remote sensing images of 30 consecutive moments were used as input to the 3D-GAN model. [file peerj-cs-09-1292-s008.zip › 437.jpg]

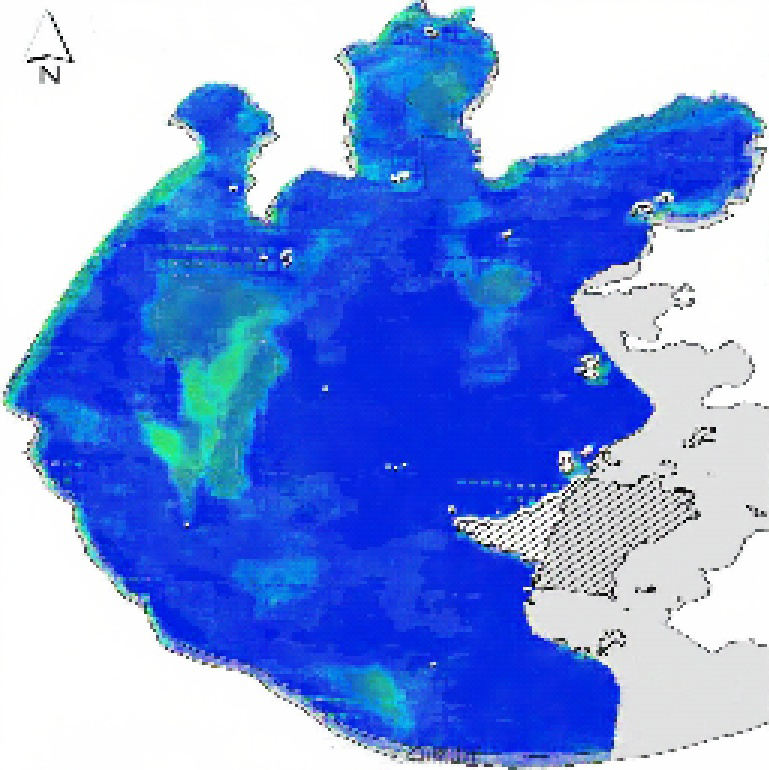

Supplement: Supplemental Information 8 — The data are remote sensing images of chlorophyll a concentration after data scale unification, remote sensing image repair, and time series filling. Remote sensing images of 30 consecutive moments were used as input to the 3D-GAN model. [file peerj-cs-09-1292-s008.zip › 438.jpg]

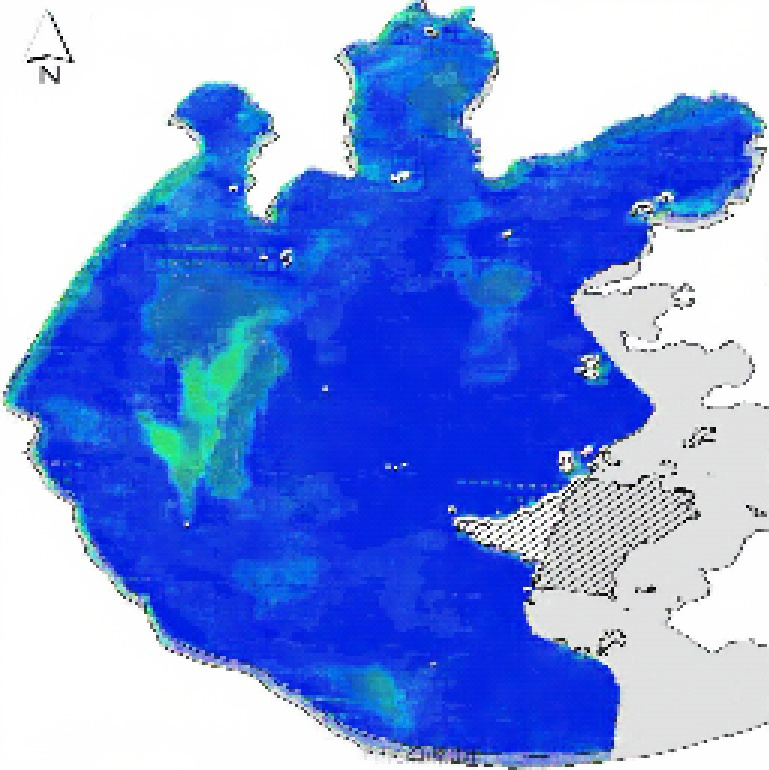

Supplement: Supplemental Information 8 — The data are remote sensing images of chlorophyll a concentration after data scale unification, remote sensing image repair, and time series filling. Remote sensing images of 30 consecutive moments were used as input to the 3D-GAN model. [file peerj-cs-09-1292-s008.zip › 439.jpg]

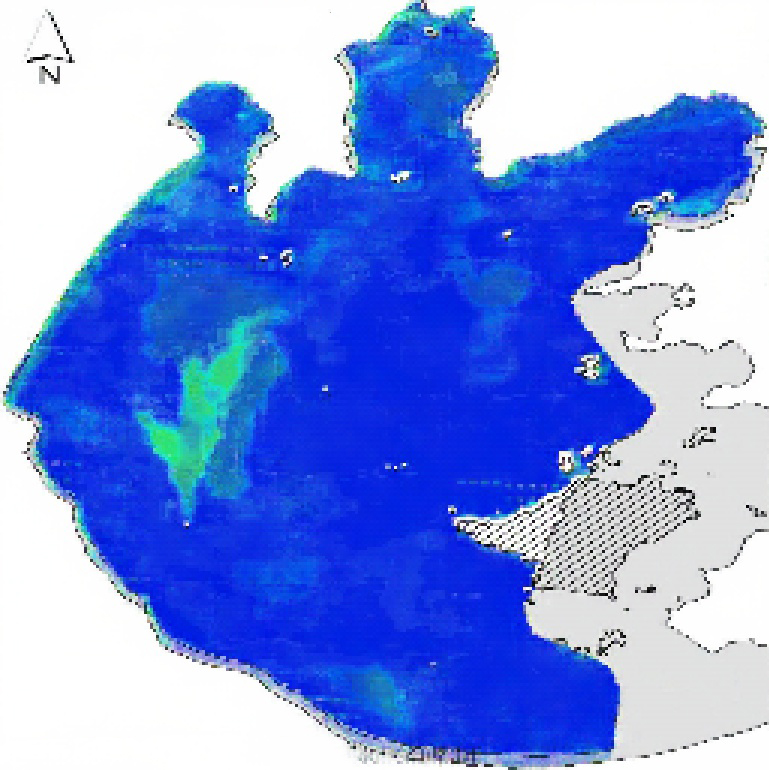

Supplement: Supplemental Information 8 — The data are remote sensing images of chlorophyll a concentration after data scale unification, remote sensing image repair, and time series filling. Remote sensing images of 30 consecutive moments were used as input to the 3D-GAN model. [file peerj-cs-09-1292-s008.zip › 440.jpg]

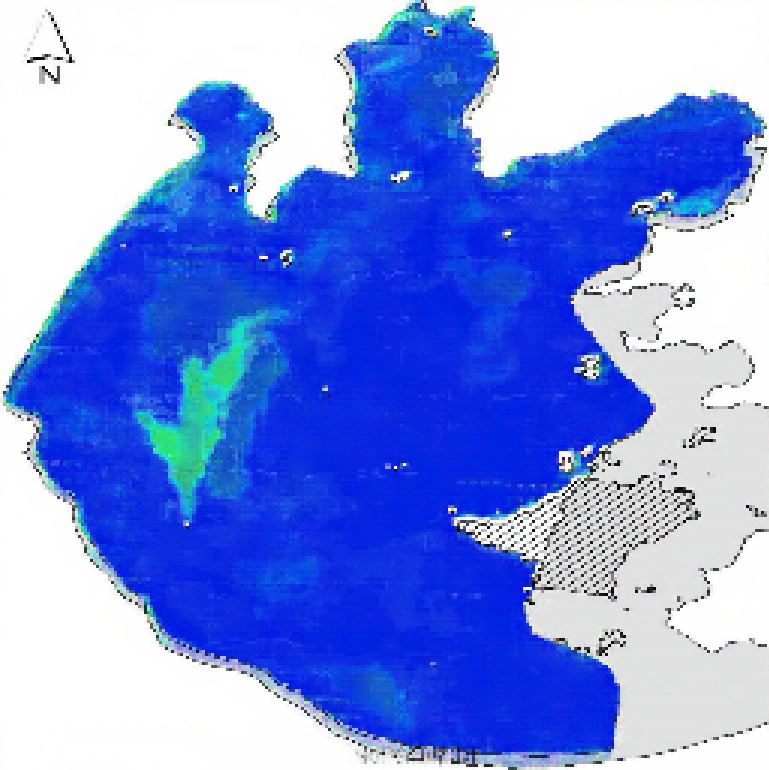

Supplement: Supplemental Information 8 — The data are remote sensing images of chlorophyll a concentration after data scale unification, remote sensing image repair, and time series filling. Remote sensing images of 30 consecutive moments were used as input to the 3D-GAN model. [file peerj-cs-09-1292-s008.zip › 441.jpg]

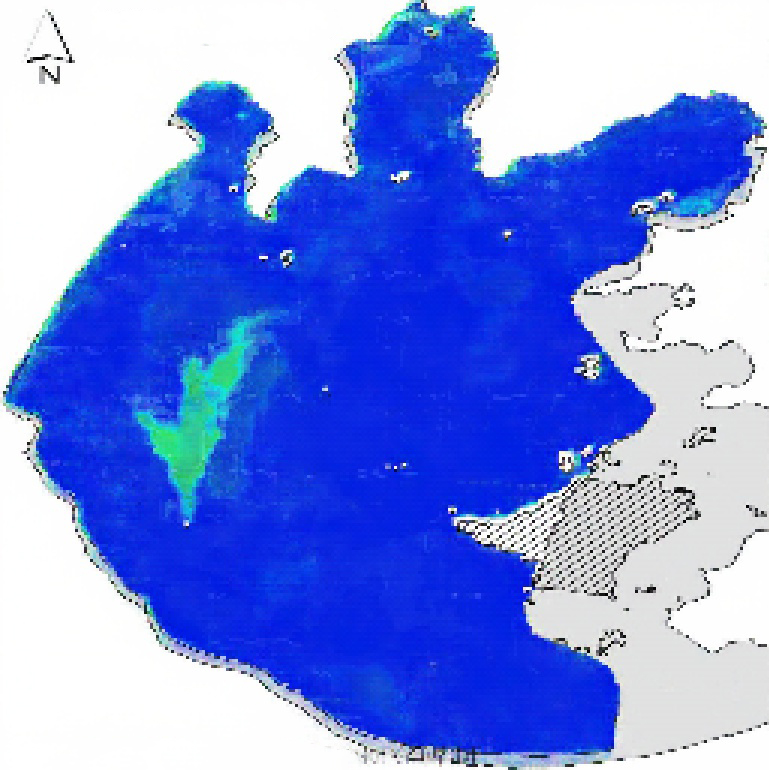

Supplement: Supplemental Information 8 — The data are remote sensing images of chlorophyll a concentration after data scale unification, remote sensing image repair, and time series filling. Remote sensing images of 30 consecutive moments were used as input to the 3D-GAN model. [file peerj-cs-09-1292-s008.zip › 442.jpg]

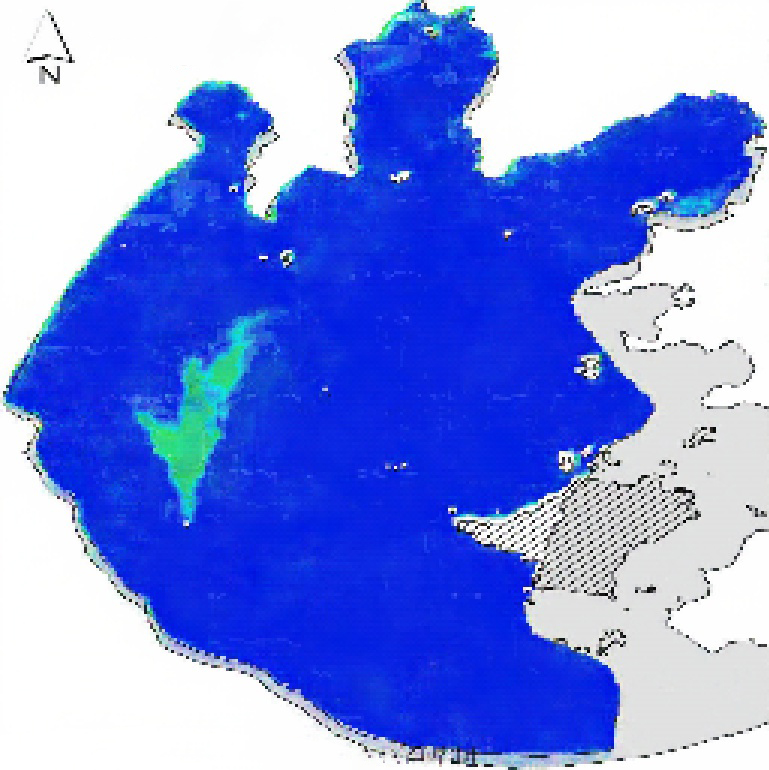

Supplement: Supplemental Information 8 — The data are remote sensing images of chlorophyll a concentration after data scale unification, remote sensing image repair, and time series filling. Remote sensing images of 30 consecutive moments were used as input to the 3D-GAN model. [file peerj-cs-09-1292-s008.zip › 443.jpg]

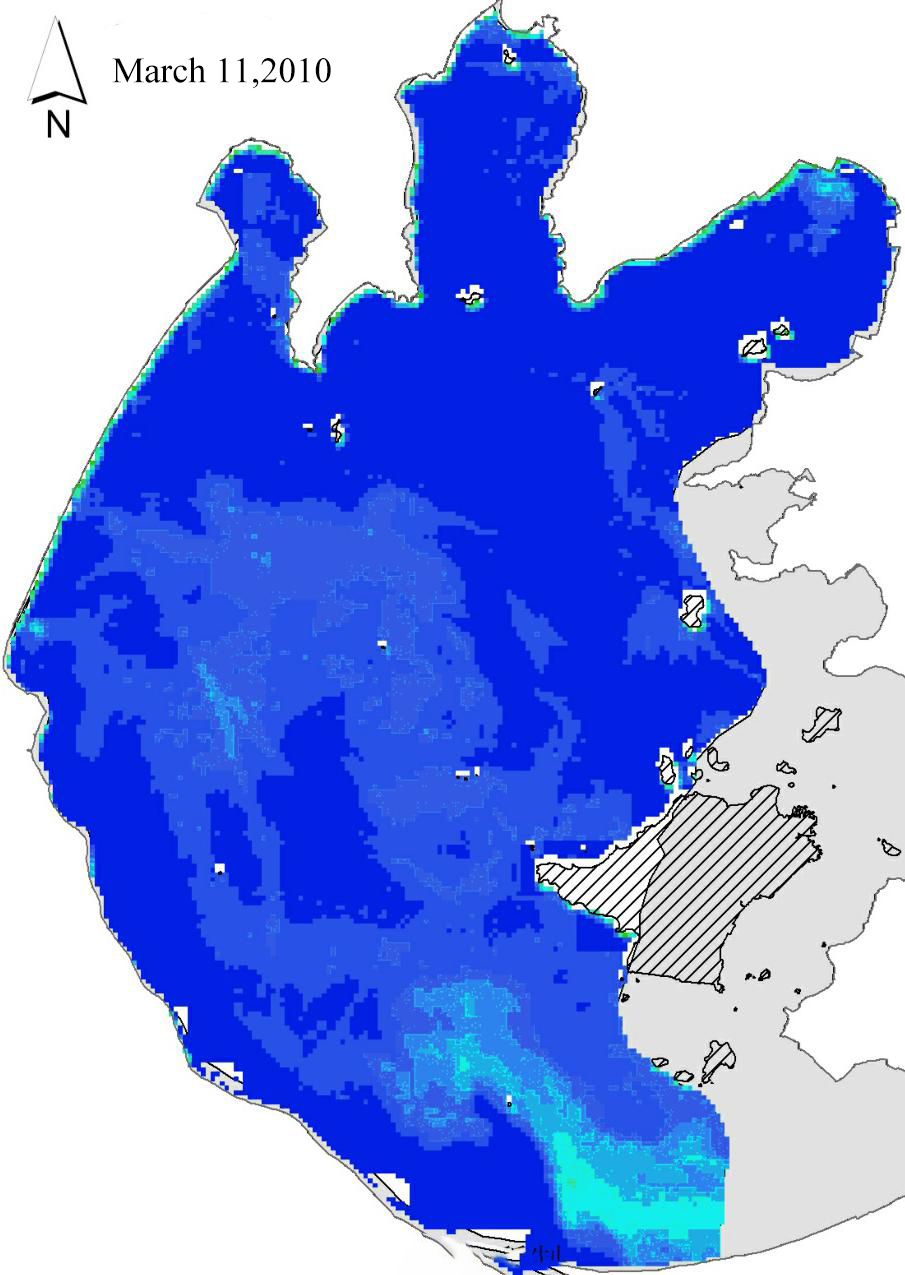

Supplement: Supplemental Information 9 — The data are remote sensing images of chlorophyll a concentration after data scale unification, remote sensing image repair, and time series filling. Remote sensing images of 30 consecutive moments were used as input to the 3D-GAN model. [file peerj-cs-09-1292-s009.zip › 201003110245.jpg]

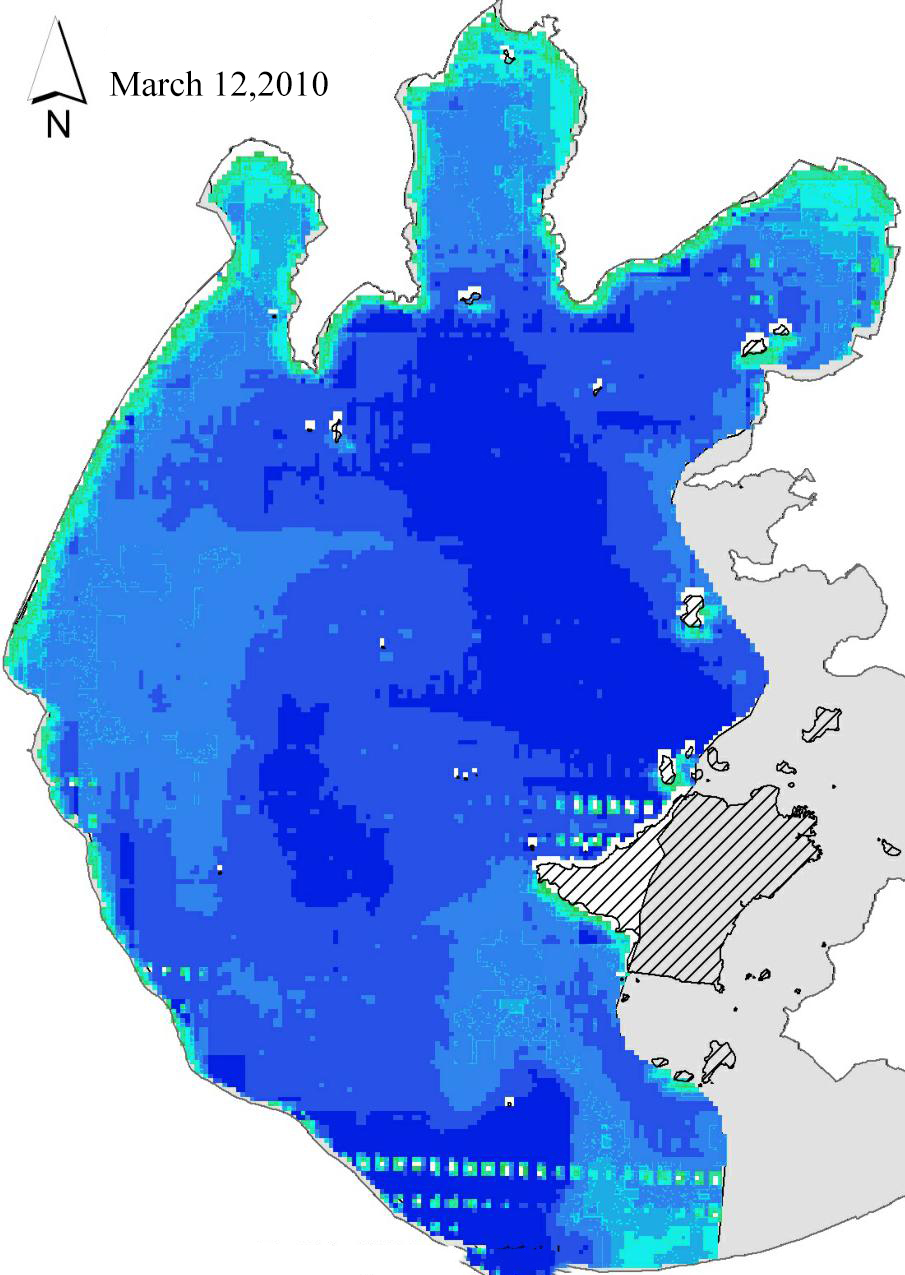

Supplement: Supplemental Information 9 — The data are remote sensing images of chlorophyll a concentration after data scale unification, remote sensing image repair, and time series filling. Remote sensing images of 30 consecutive moments were used as input to the 3D-GAN model. [file peerj-cs-09-1292-s009.zip › 201003120245.jpg]

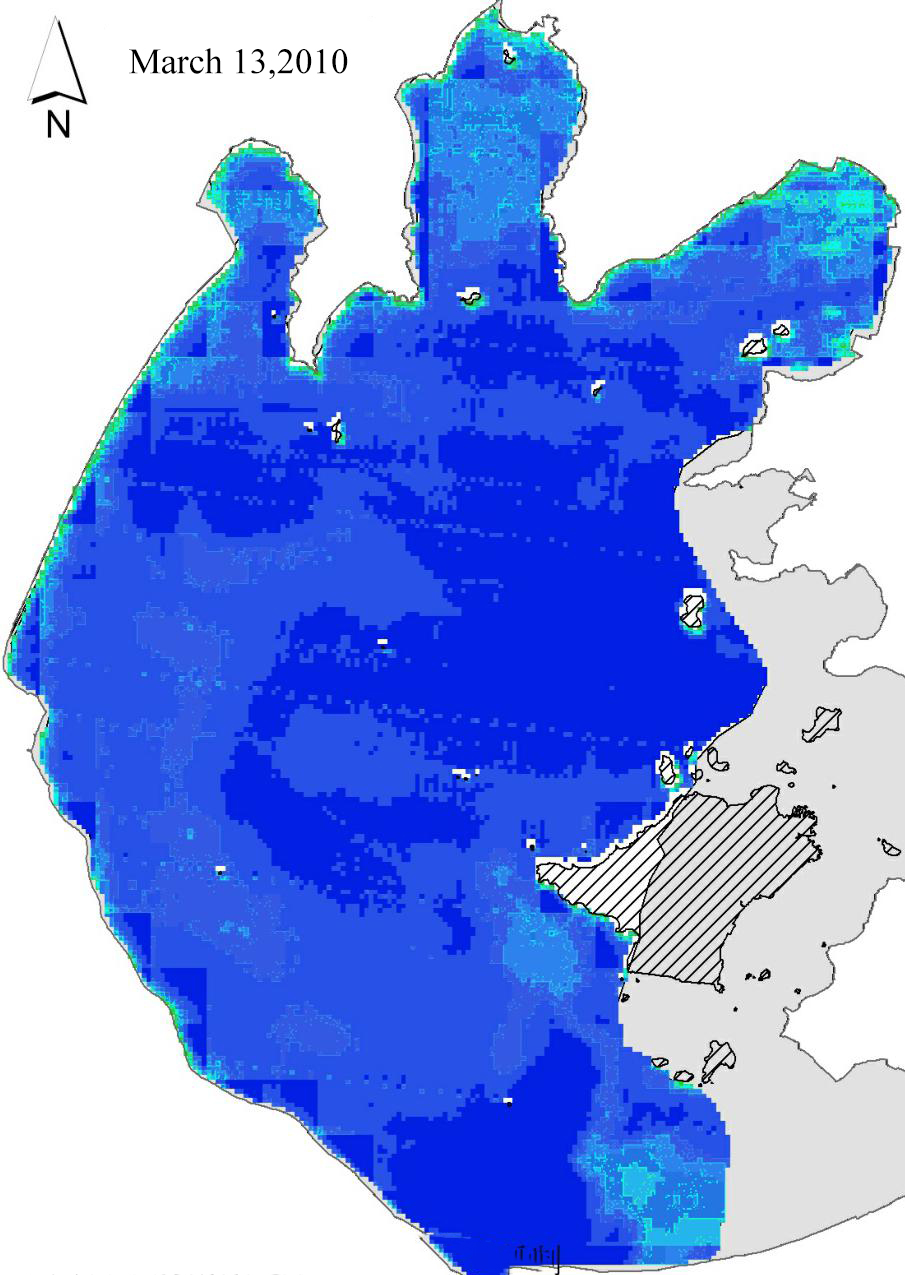

Supplement: Supplemental Information 9 — The data are remote sensing images of chlorophyll a concentration after data scale unification, remote sensing image repair, and time series filling. Remote sensing images of 30 consecutive moments were used as input to the 3D-GAN model. [file peerj-cs-09-1292-s009.zip › 201003130245.jpg]

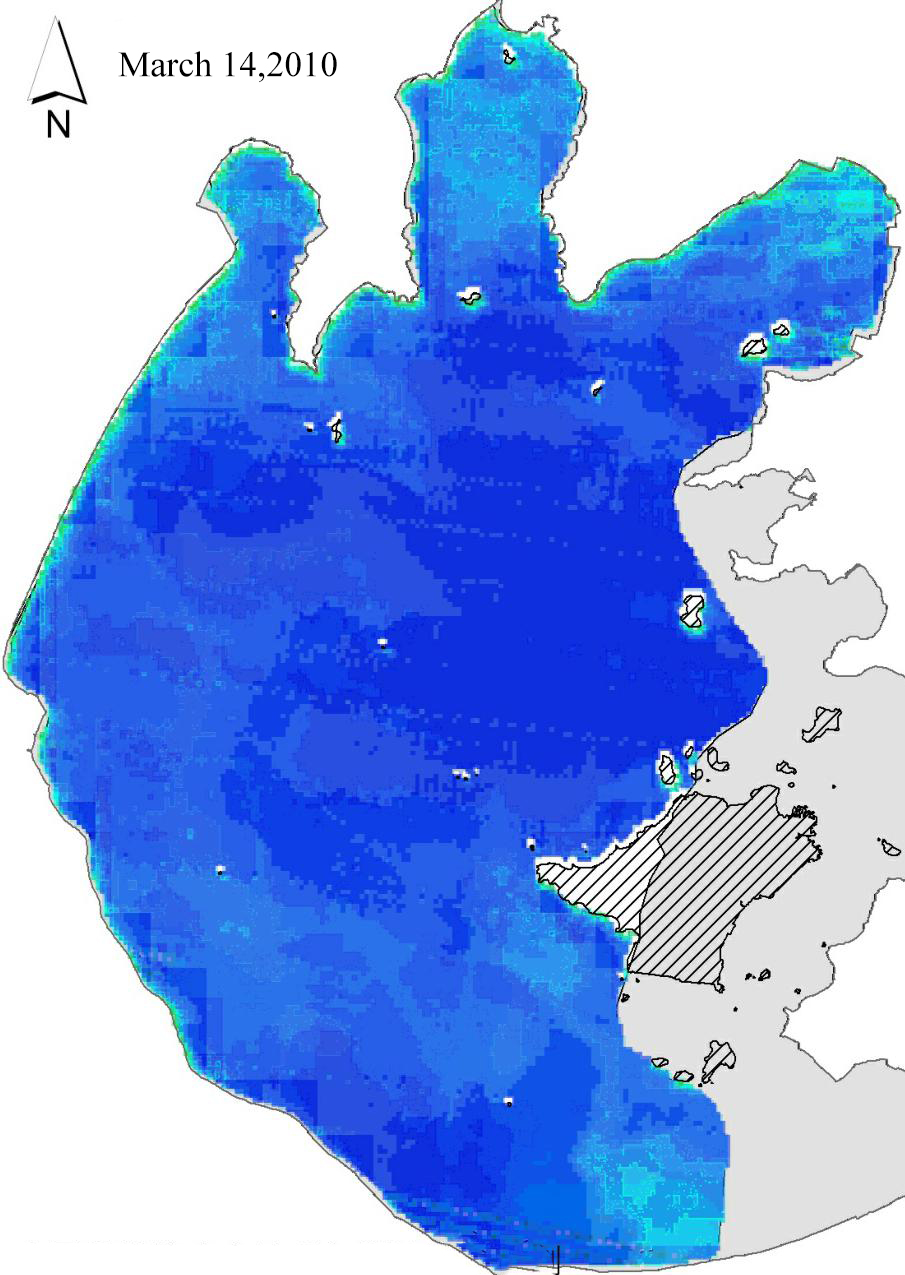

Supplement: Supplemental Information 9 — The data are remote sensing images of chlorophyll a concentration after data scale unification, remote sensing image repair, and time series filling. Remote sensing images of 30 consecutive moments were used as input to the 3D-GAN model. [file peerj-cs-09-1292-s009.zip › 201003140245.jpg]

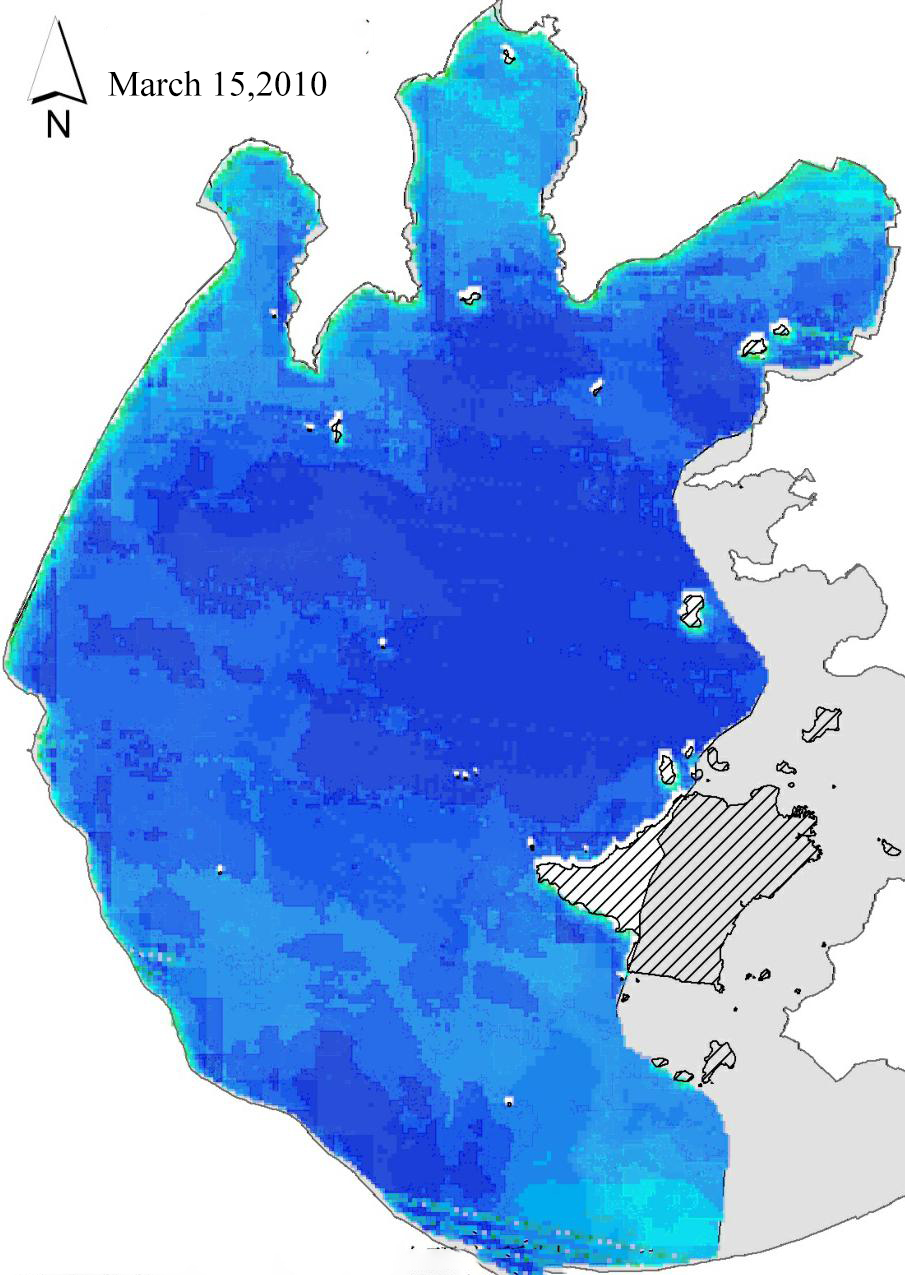

Supplement: Supplemental Information 9 — The data are remote sensing images of chlorophyll a concentration after data scale unification, remote sensing image repair, and time series filling. Remote sensing images of 30 consecutive moments were used as input to the 3D-GAN model. [file peerj-cs-09-1292-s009.zip › 201003150245.jpg]
