# Supplementary figures and images for: Remote sensing image analysis and prediction based on improved Pix2Pix model for water environment protection of smart cities (part 4 of 6)
Source: PeerJ Comput Sci. 2023 Apr 26;9:e1292. doi: 10.7717/peerj-cs.1292 (PMC10280440; doi:10.7717/peerj-cs.1292)

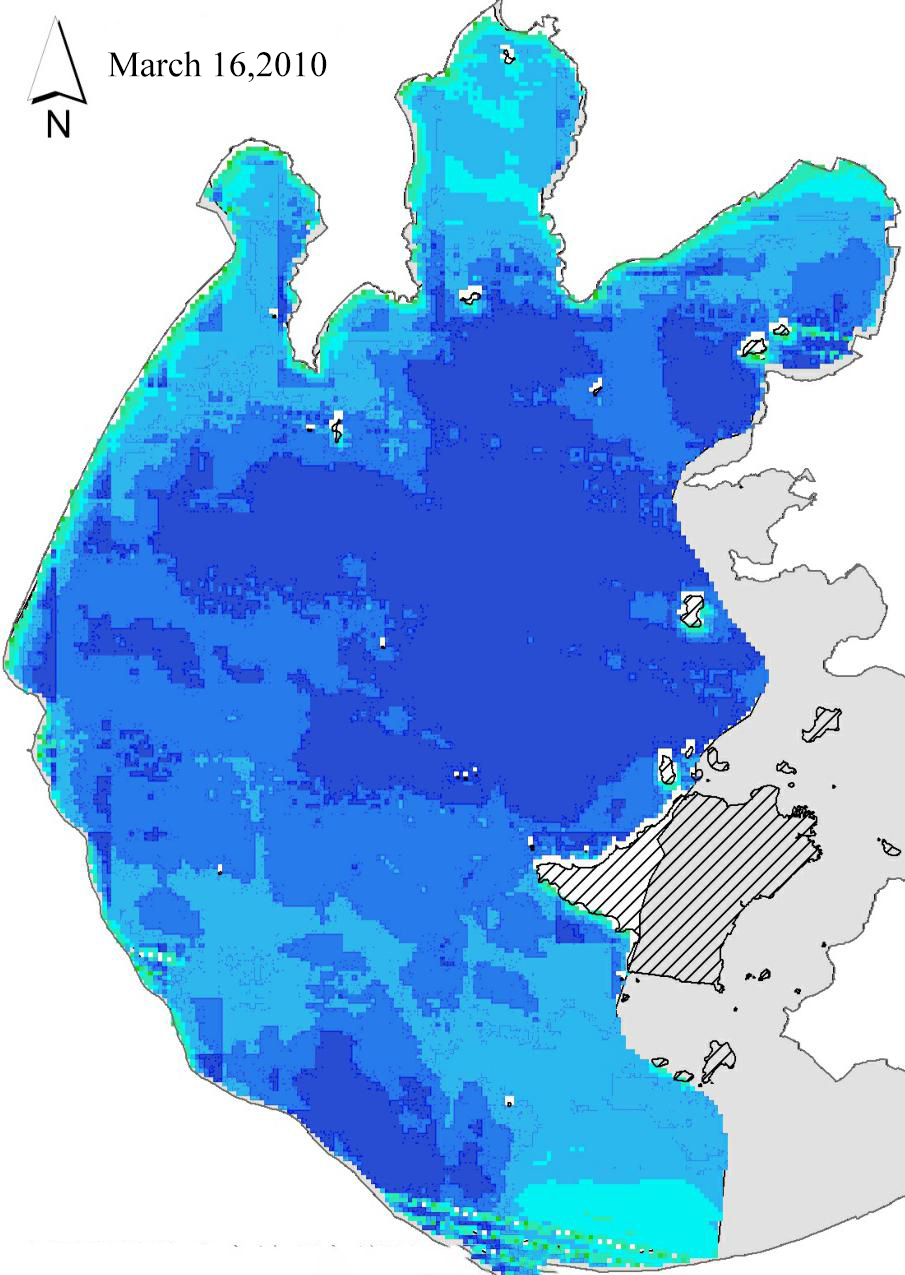

Supplement: Supplemental Information 9 — The data are remote sensing images of chlorophyll a concentration after data scale unification, remote sensing image repair, and time series filling. Remote sensing images of 30 consecutive moments were used as input to the 3D-GAN model. [file peerj-cs-09-1292-s009.zip › 201003160245.jpg]

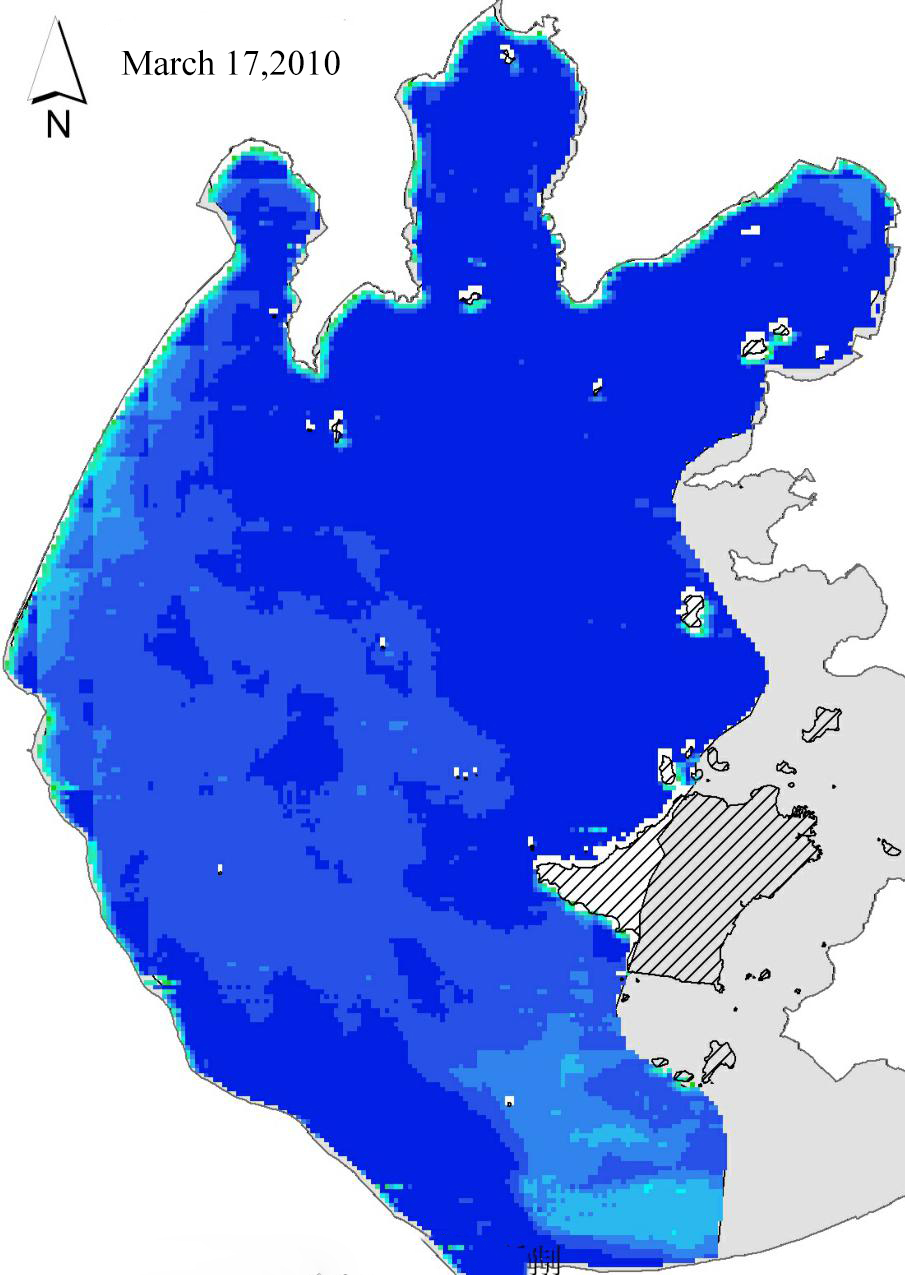

Supplement: Supplemental Information 9 — The data are remote sensing images of chlorophyll a concentration after data scale unification, remote sensing image repair, and time series filling. Remote sensing images of 30 consecutive moments were used as input to the 3D-GAN model. [file peerj-cs-09-1292-s009.zip › 201003170245.jpg]

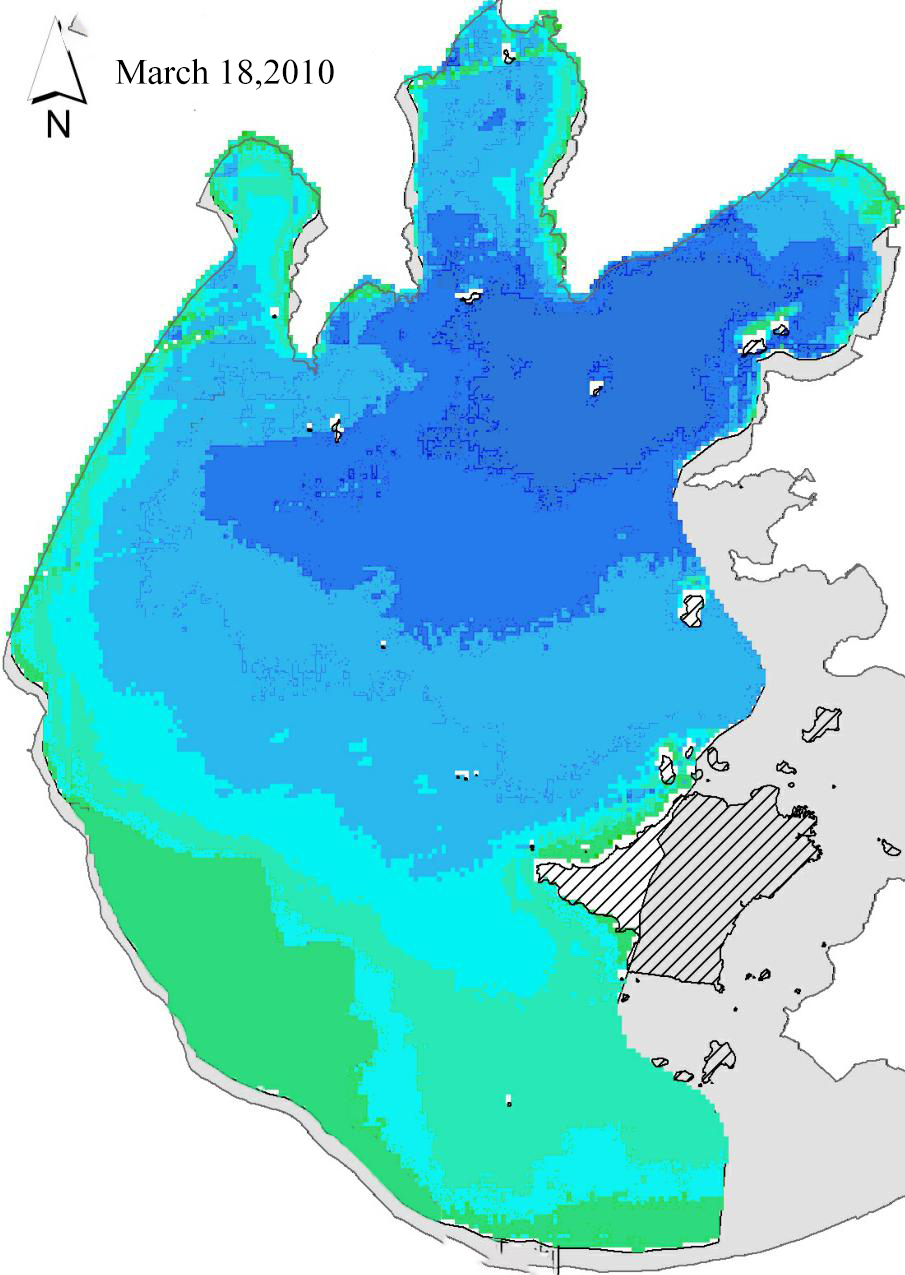

Supplement: Supplemental Information 9 — The data are remote sensing images of chlorophyll a concentration after data scale unification, remote sensing image repair, and time series filling. Remote sensing images of 30 consecutive moments were used as input to the 3D-GAN model. [file peerj-cs-09-1292-s009.zip › 201003180245.jpg]

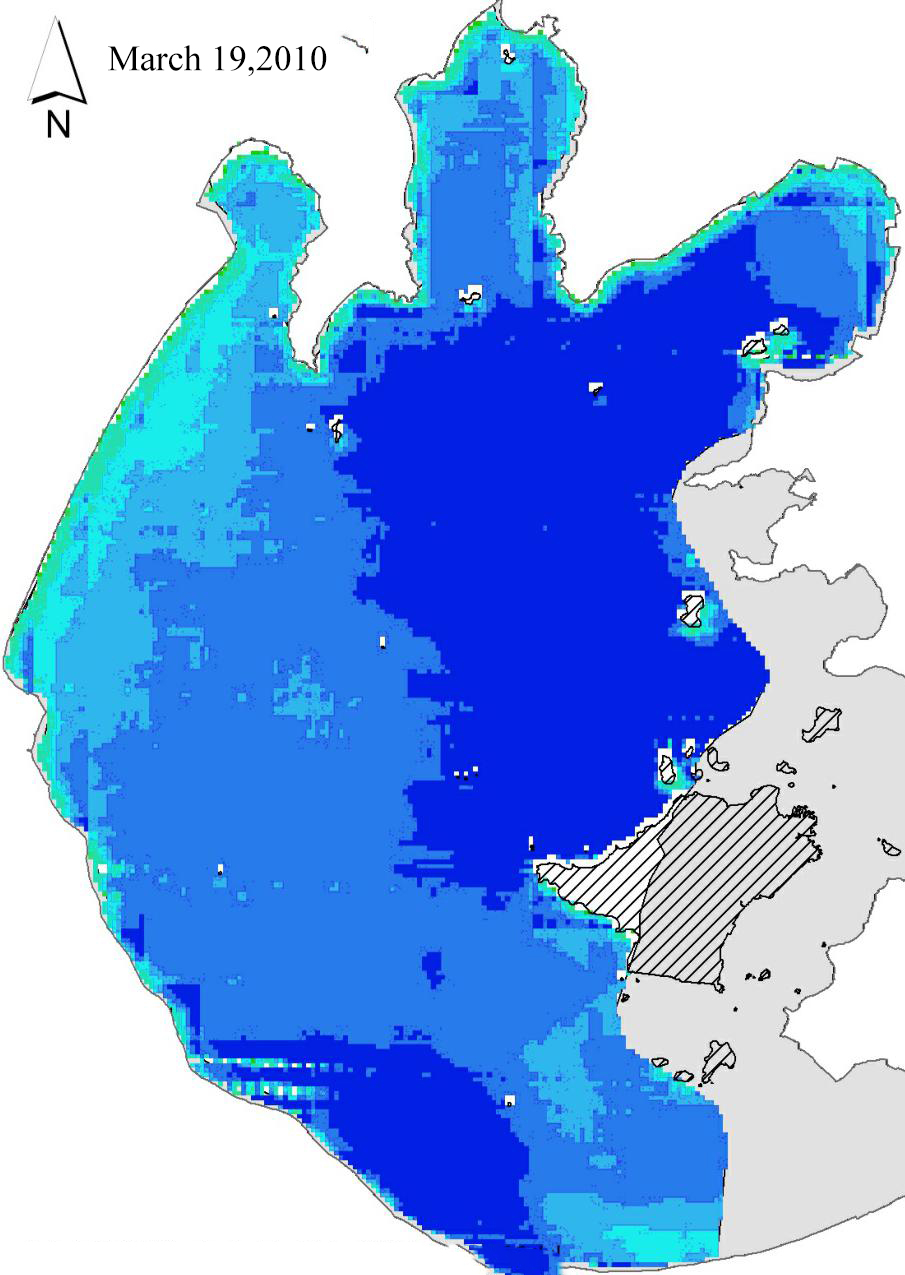

Supplement: Supplemental Information 9 — The data are remote sensing images of chlorophyll a concentration after data scale unification, remote sensing image repair, and time series filling. Remote sensing images of 30 consecutive moments were used as input to the 3D-GAN model. [file peerj-cs-09-1292-s009.zip › 201003190245.jpg]

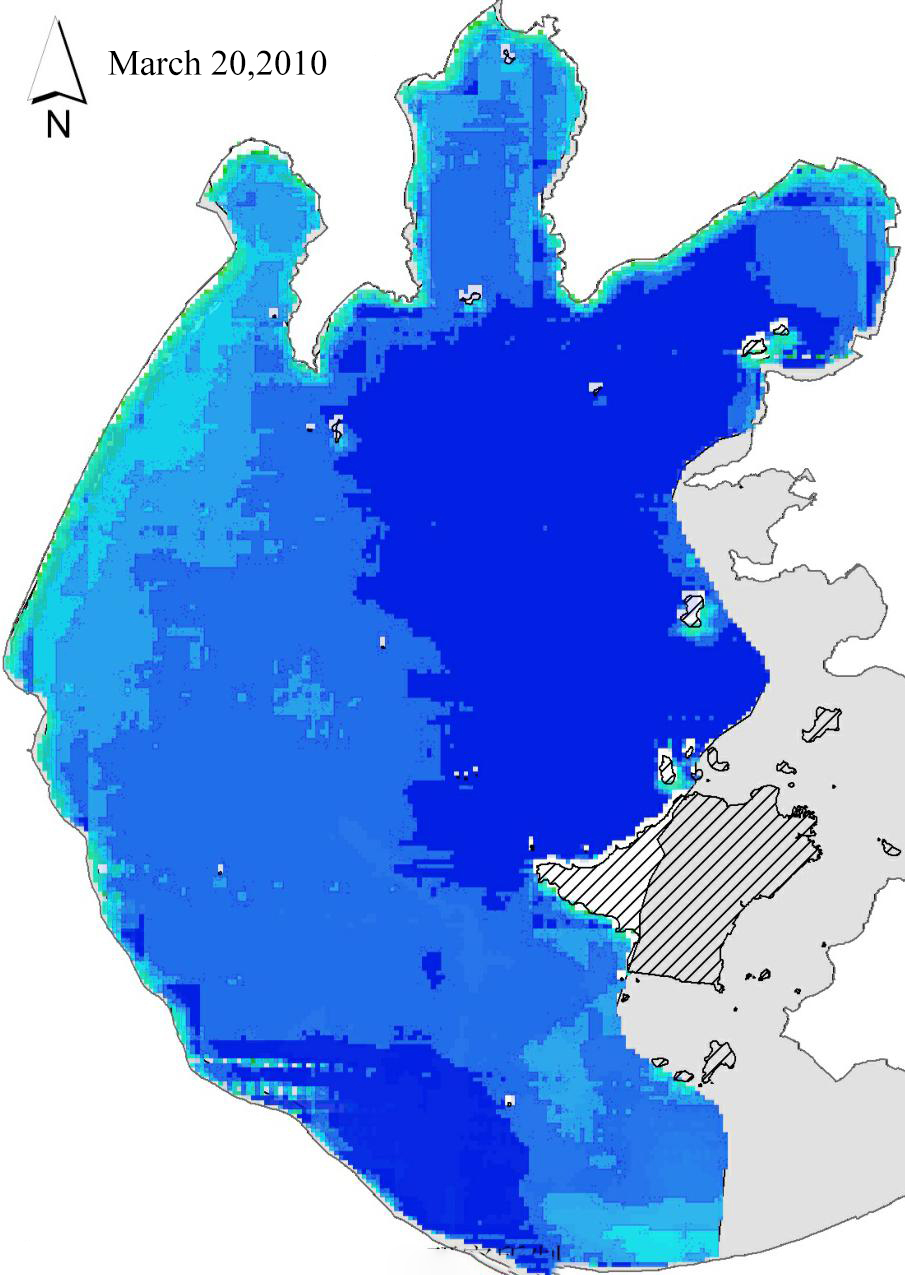

Supplement: Supplemental Information 9 — The data are remote sensing images of chlorophyll a concentration after data scale unification, remote sensing image repair, and time series filling. Remote sensing images of 30 consecutive moments were used as input to the 3D-GAN model. [file peerj-cs-09-1292-s009.zip › 201003200245.jpg]

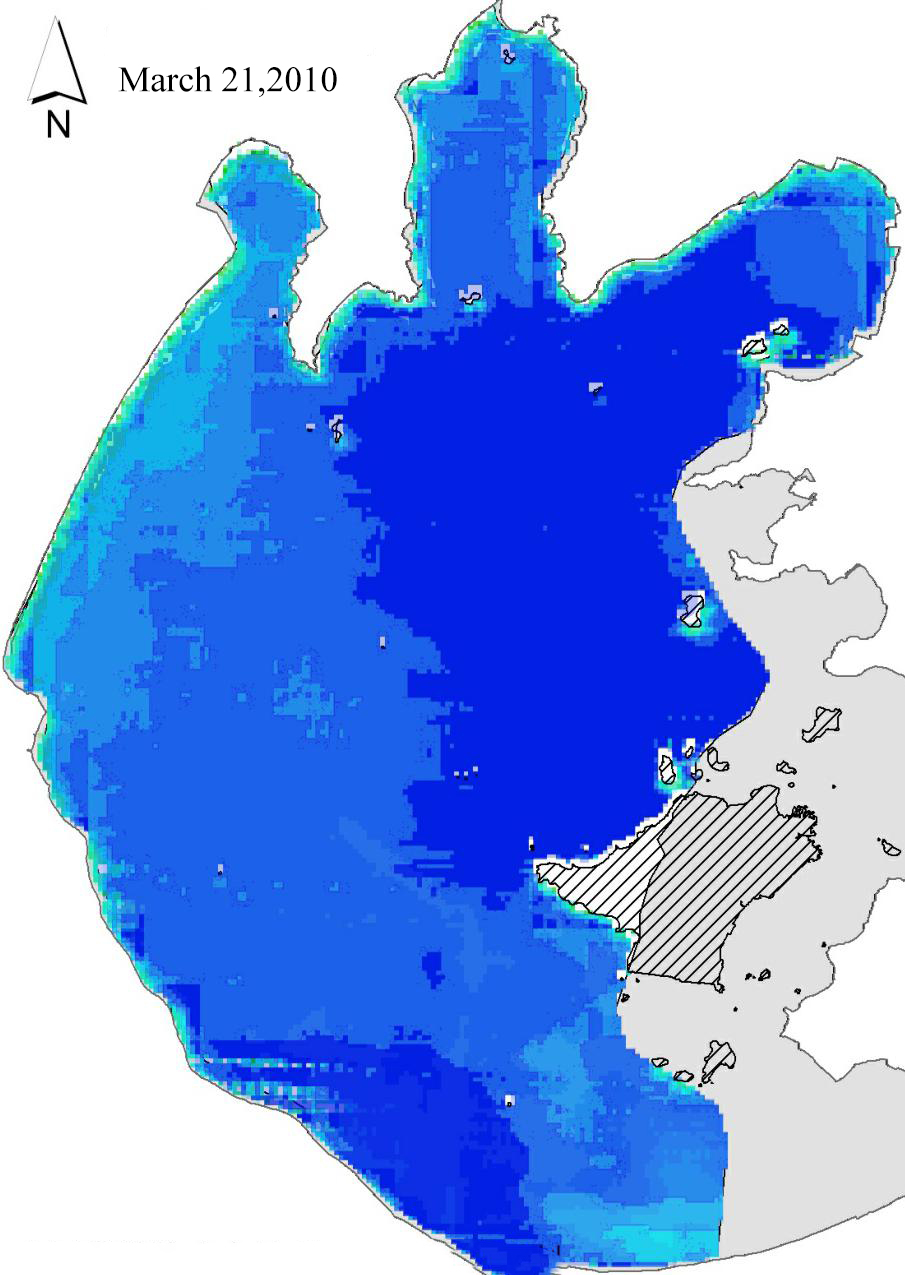

Supplement: Supplemental Information 9 — The data are remote sensing images of chlorophyll a concentration after data scale unification, remote sensing image repair, and time series filling. Remote sensing images of 30 consecutive moments were used as input to the 3D-GAN model. [file peerj-cs-09-1292-s009.zip › 201003210245.jpg]

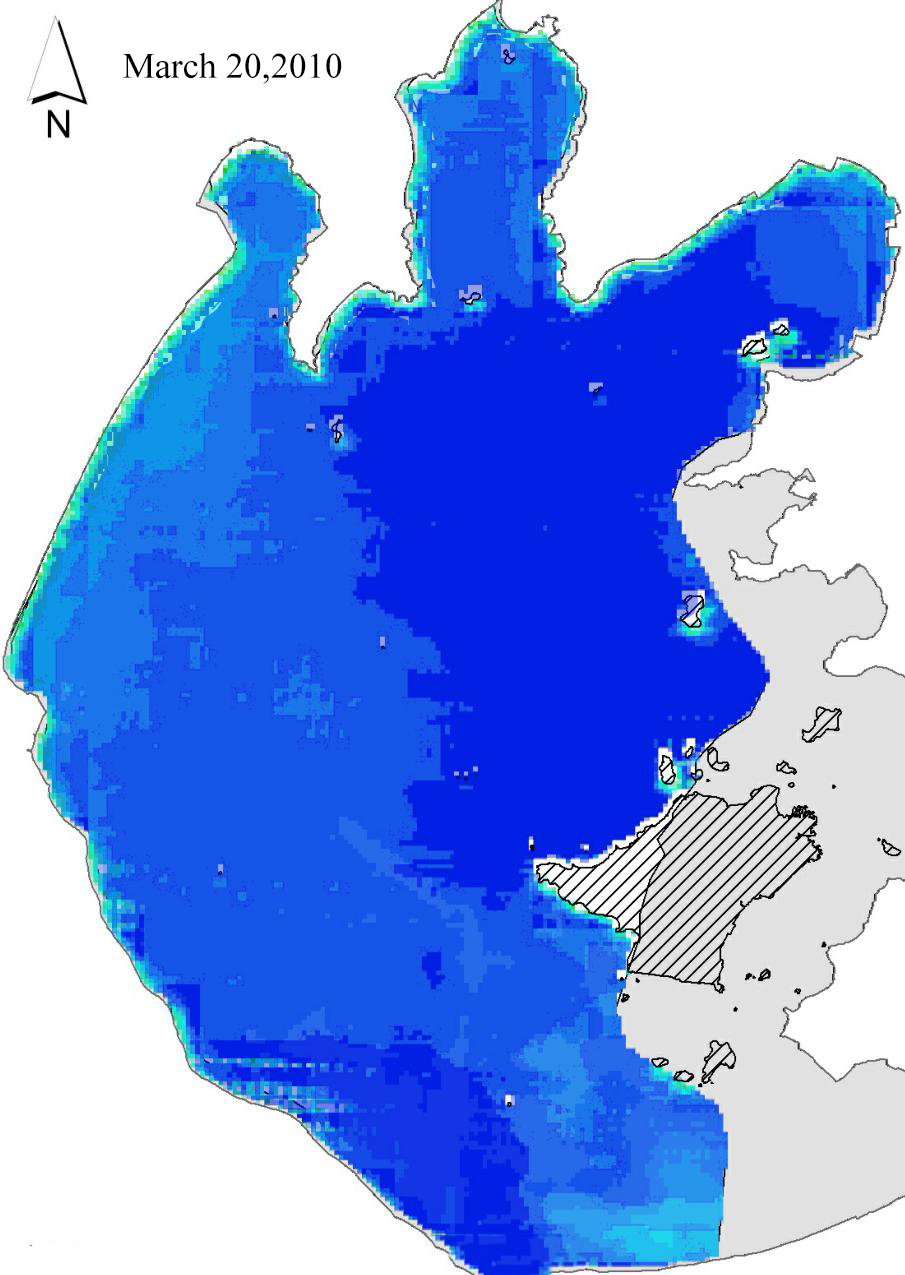

Supplement: Supplemental Information 9 — The data are remote sensing images of chlorophyll a concentration after data scale unification, remote sensing image repair, and time series filling. Remote sensing images of 30 consecutive moments were used as input to the 3D-GAN model. [file peerj-cs-09-1292-s009.zip › 201003220245.jpg]

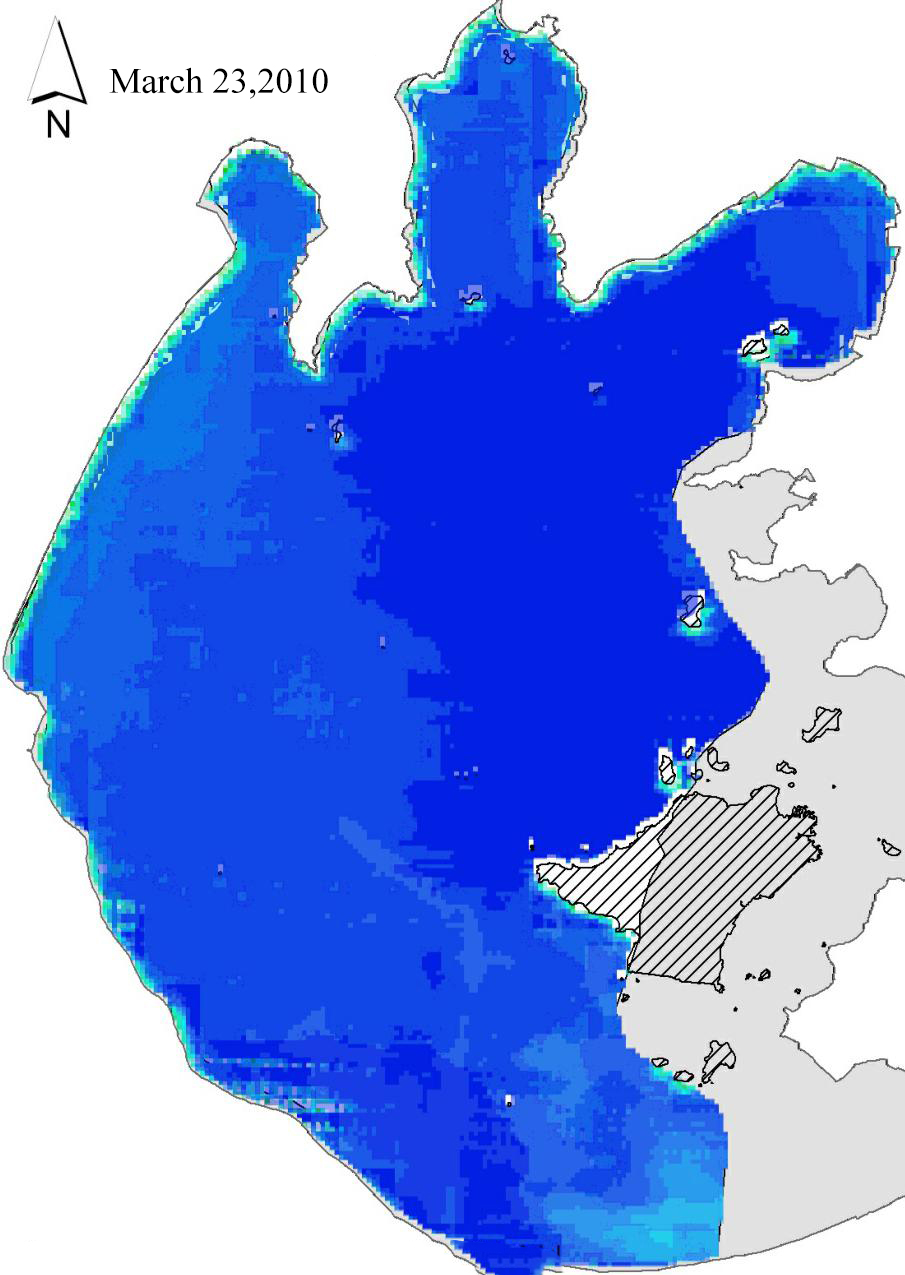

Supplement: Supplemental Information 9 — The data are remote sensing images of chlorophyll a concentration after data scale unification, remote sensing image repair, and time series filling. Remote sensing images of 30 consecutive moments were used as input to the 3D-GAN model. [file peerj-cs-09-1292-s009.zip › 201003230245.jpg]

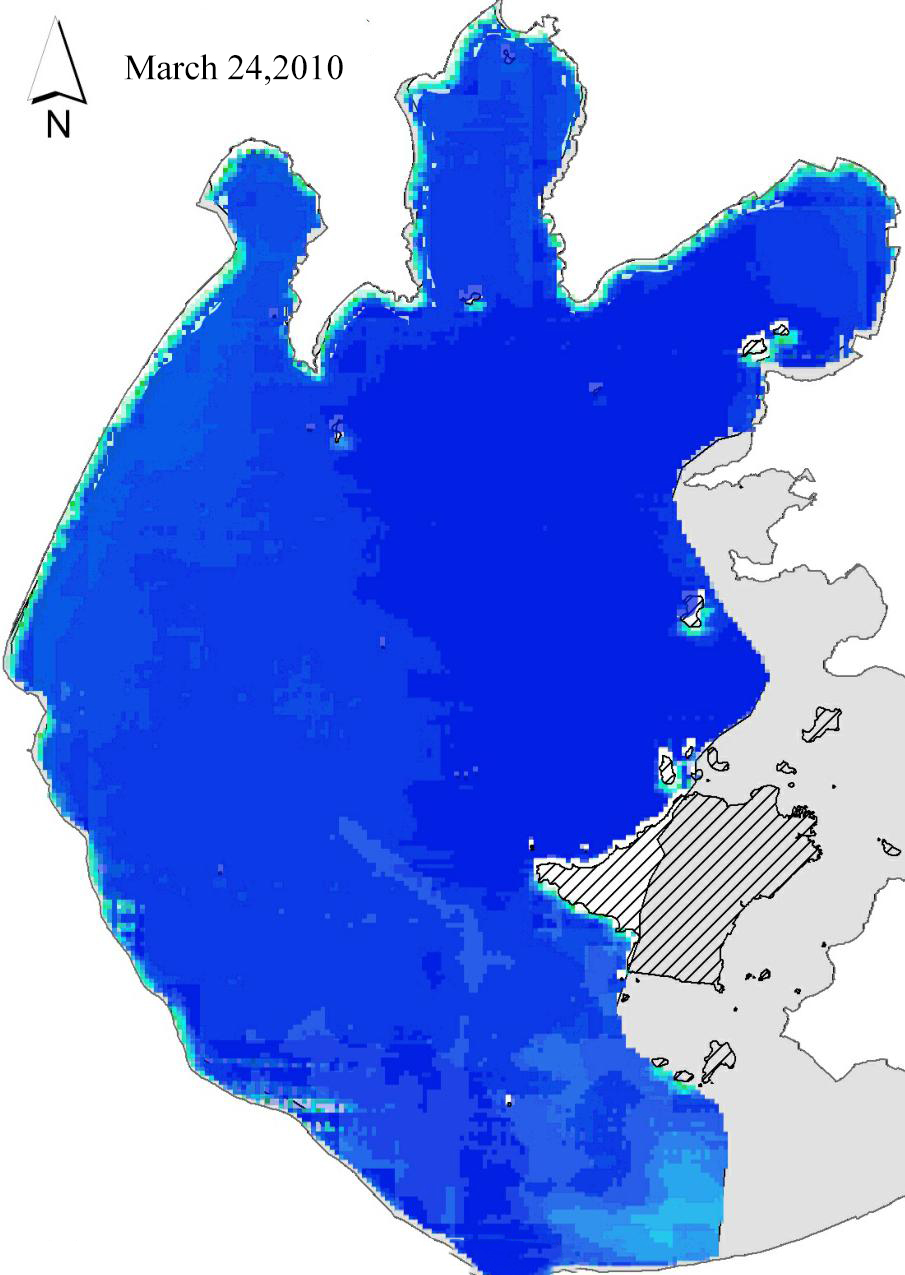

Supplement: Supplemental Information 9 — The data are remote sensing images of chlorophyll a concentration after data scale unification, remote sensing image repair, and time series filling. Remote sensing images of 30 consecutive moments were used as input to the 3D-GAN model. [file peerj-cs-09-1292-s009.zip › 201003240245.jpg]

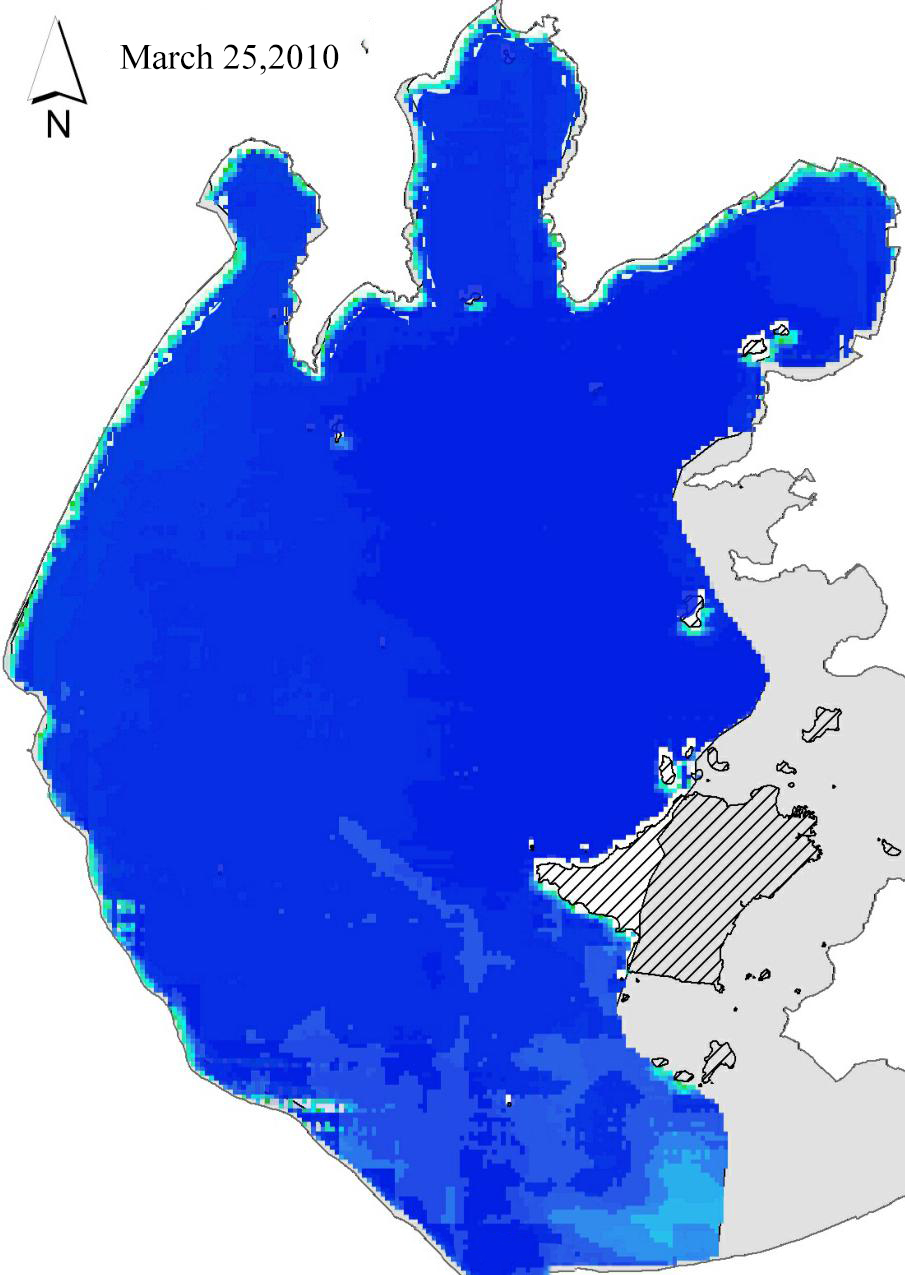

Supplement: Supplemental Information 9 — The data are remote sensing images of chlorophyll a concentration after data scale unification, remote sensing image repair, and time series filling. Remote sensing images of 30 consecutive moments were used as input to the 3D-GAN model. [file peerj-cs-09-1292-s009.zip › 201003250245.jpg]

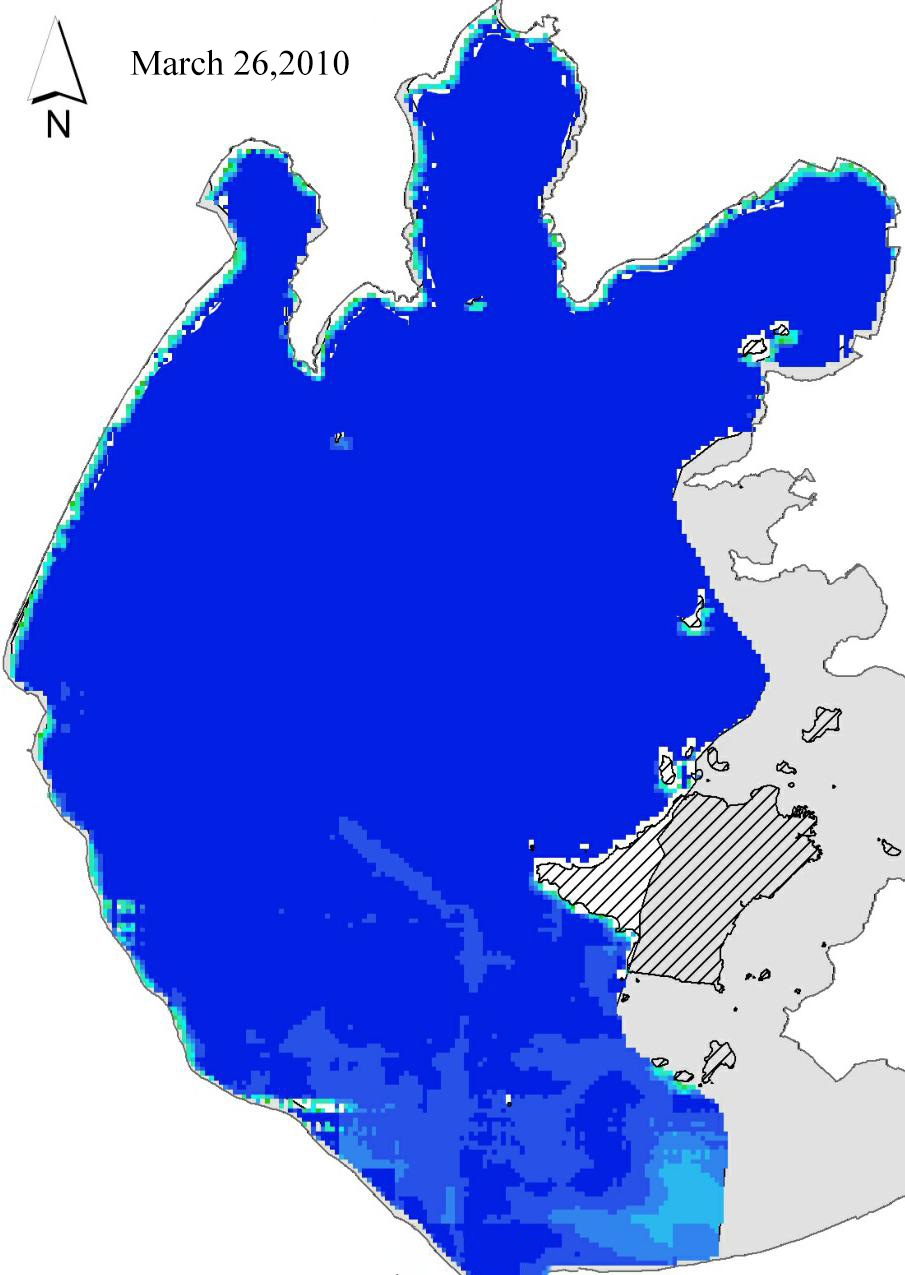

Supplement: Supplemental Information 9 — The data are remote sensing images of chlorophyll a concentration after data scale unification, remote sensing image repair, and time series filling. Remote sensing images of 30 consecutive moments were used as input to the 3D-GAN model. [file peerj-cs-09-1292-s009.zip › 201003260245.jpg]

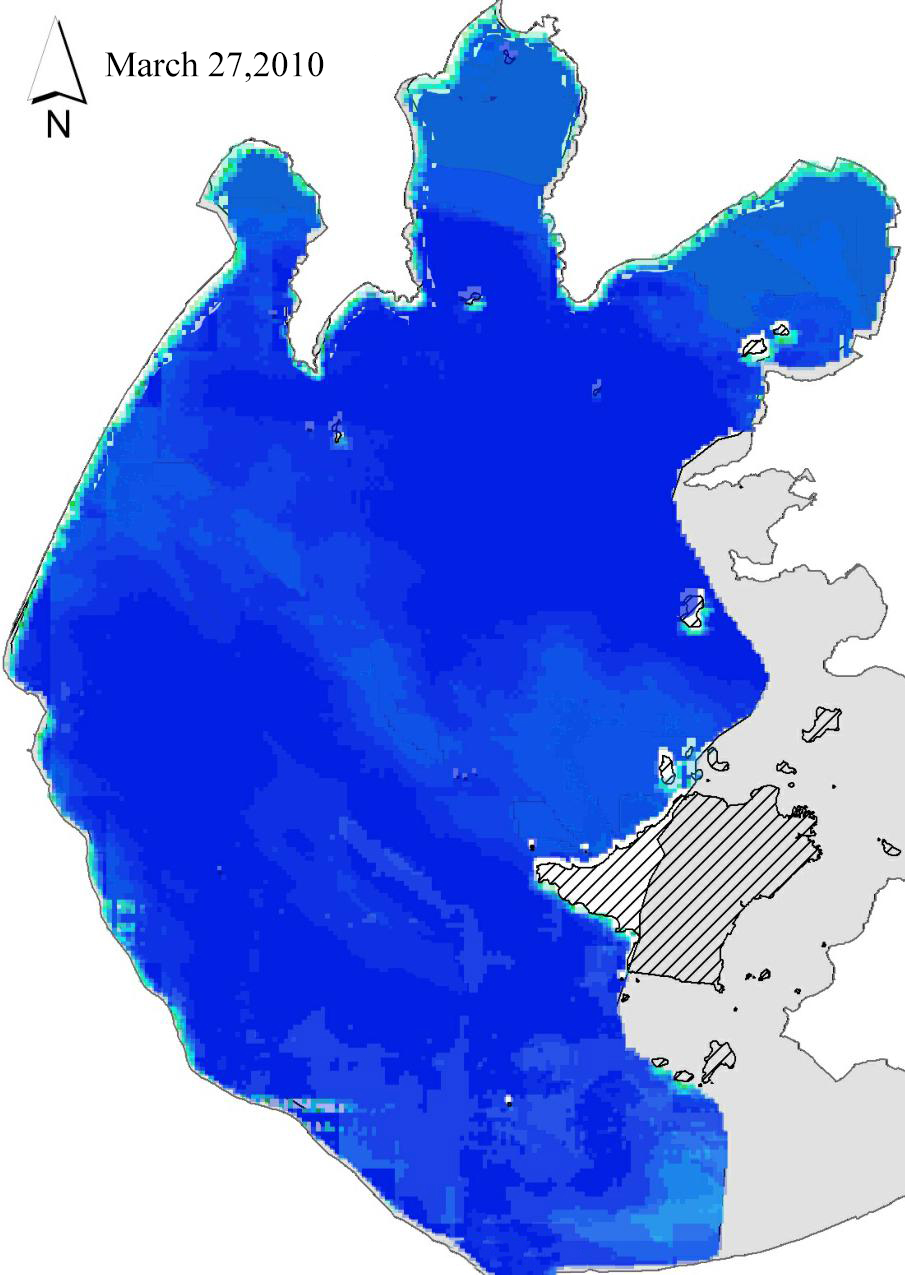

Supplement: Supplemental Information 9 — The data are remote sensing images of chlorophyll a concentration after data scale unification, remote sensing image repair, and time series filling. Remote sensing images of 30 consecutive moments were used as input to the 3D-GAN model. [file peerj-cs-09-1292-s009.zip › 201003270245.jpg]

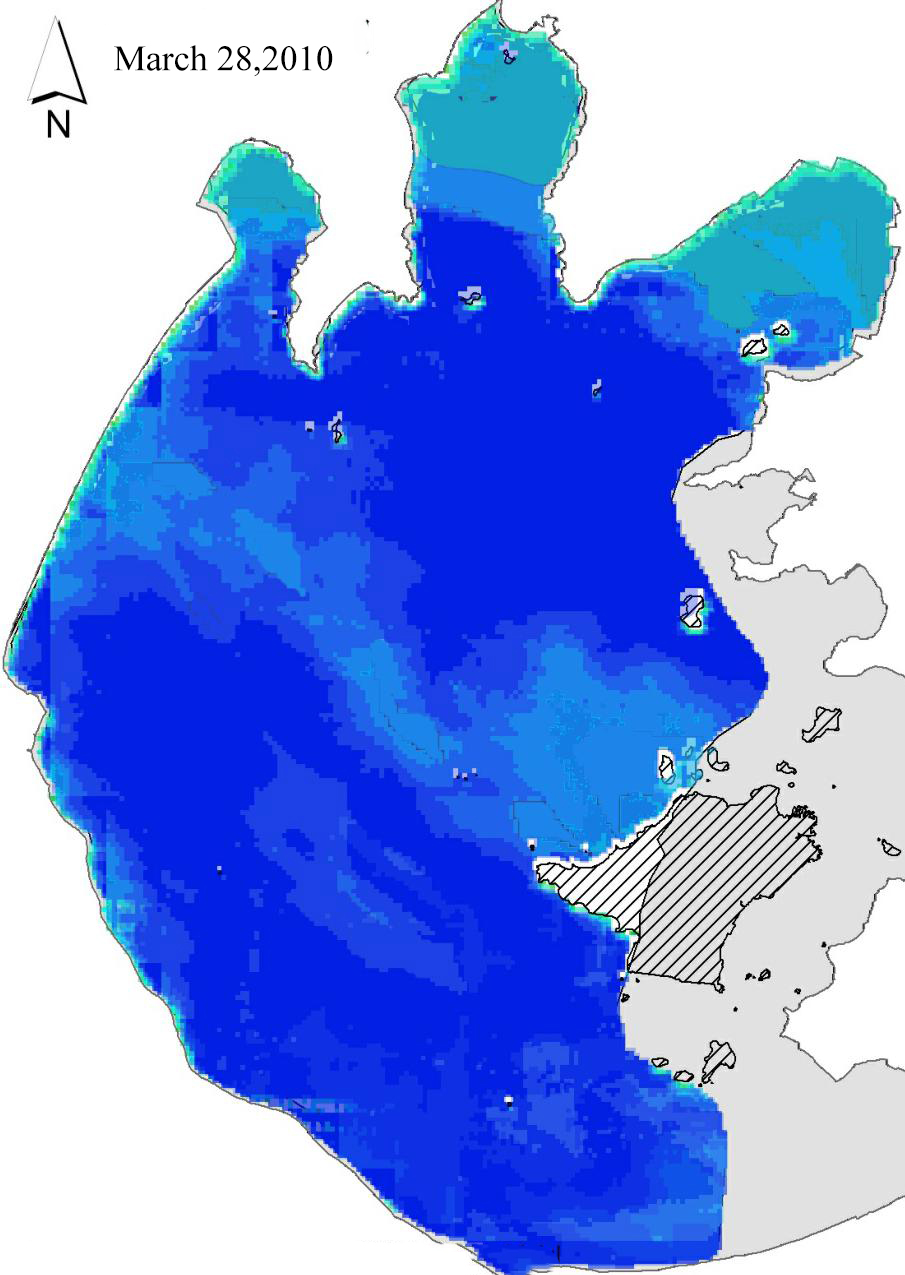

Supplement: Supplemental Information 9 — The data are remote sensing images of chlorophyll a concentration after data scale unification, remote sensing image repair, and time series filling. Remote sensing images of 30 consecutive moments were used as input to the 3D-GAN model. [file peerj-cs-09-1292-s009.zip › 201003280245.jpg]

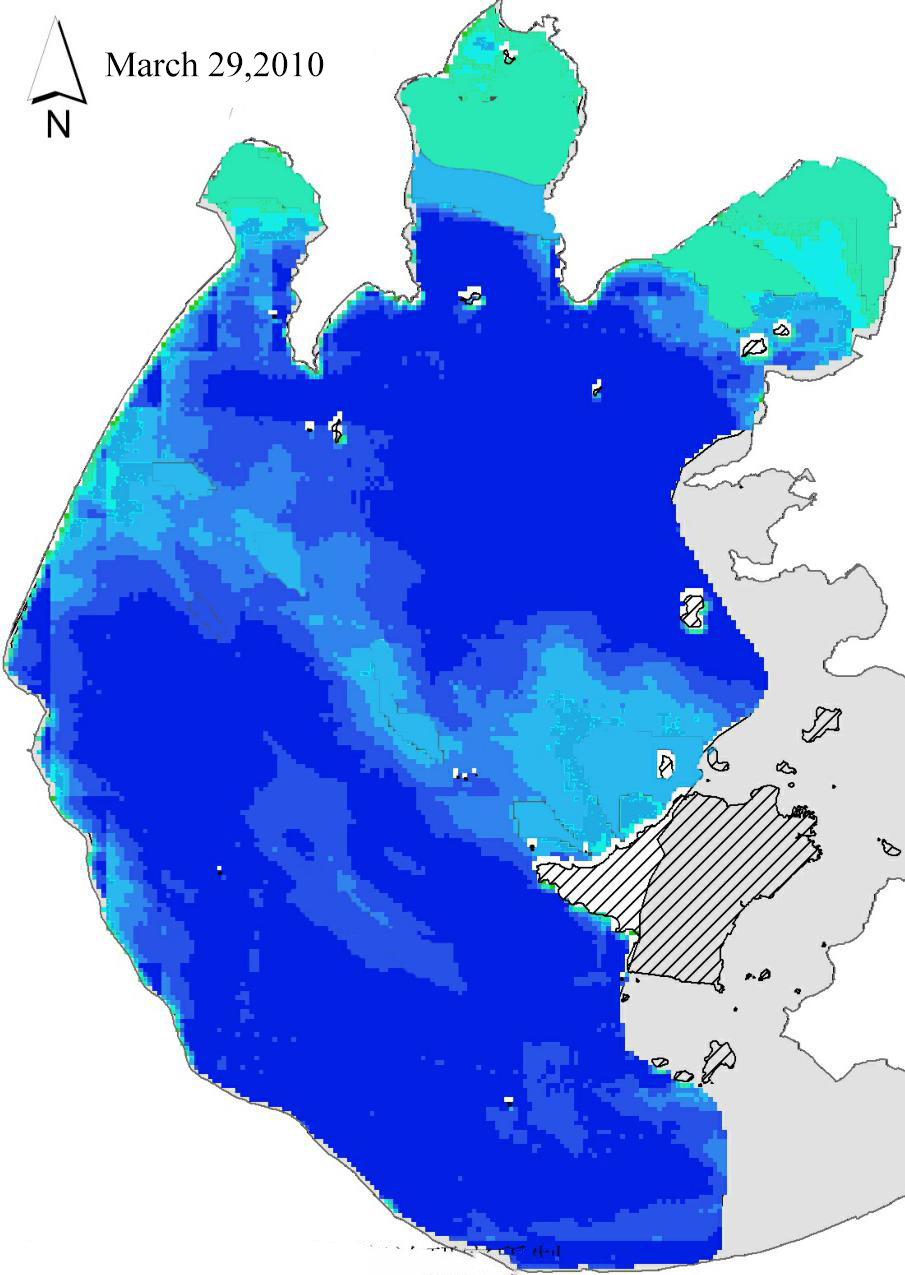

Supplement: Supplemental Information 9 — The data are remote sensing images of chlorophyll a concentration after data scale unification, remote sensing image repair, and time series filling. Remote sensing images of 30 consecutive moments were used as input to the 3D-GAN model. [file peerj-cs-09-1292-s009.zip › 201003290245.jpg]

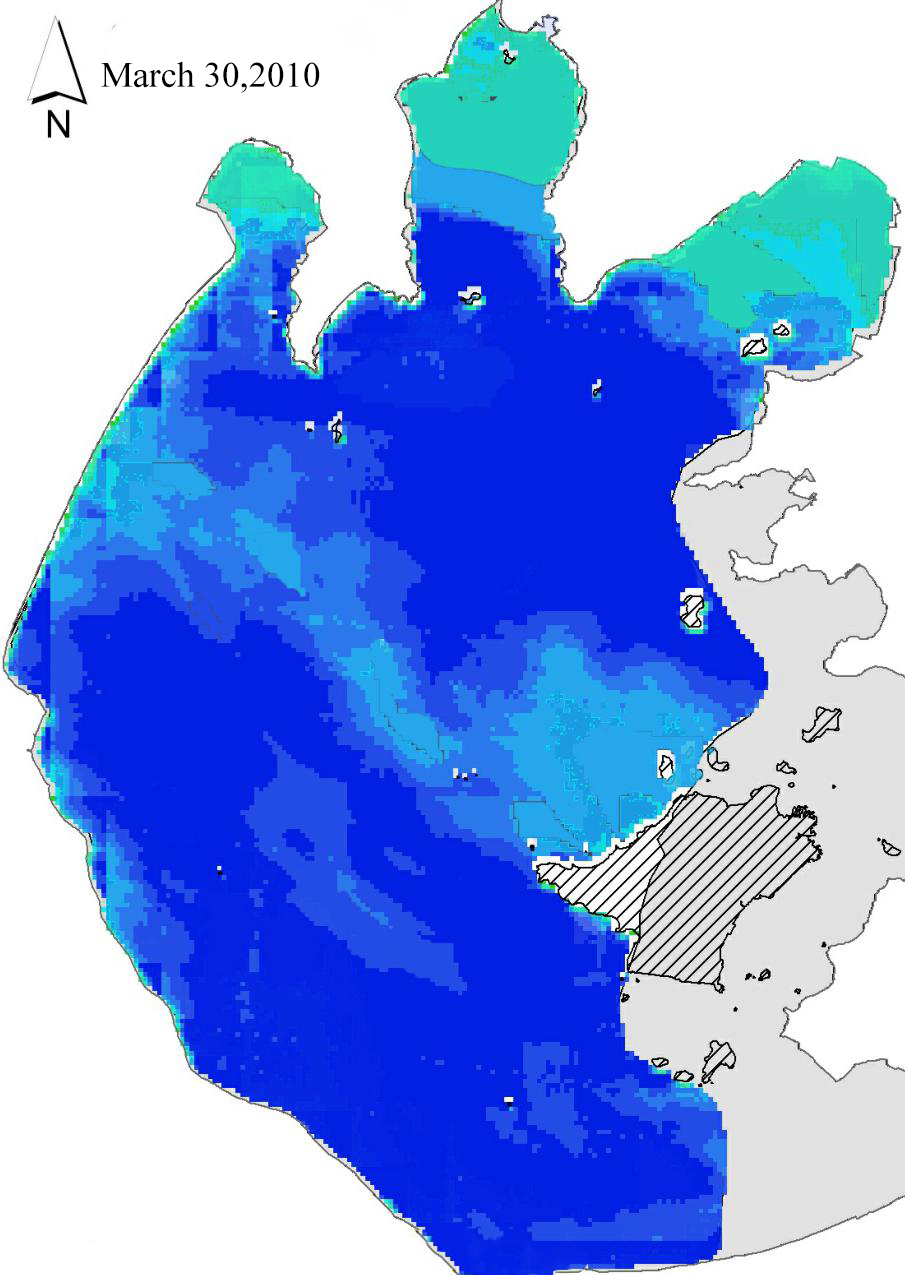

Supplement: Supplemental Information 9 — The data are remote sensing images of chlorophyll a concentration after data scale unification, remote sensing image repair, and time series filling. Remote sensing images of 30 consecutive moments were used as input to the 3D-GAN model. [file peerj-cs-09-1292-s009.zip › 201003300245.jpg]

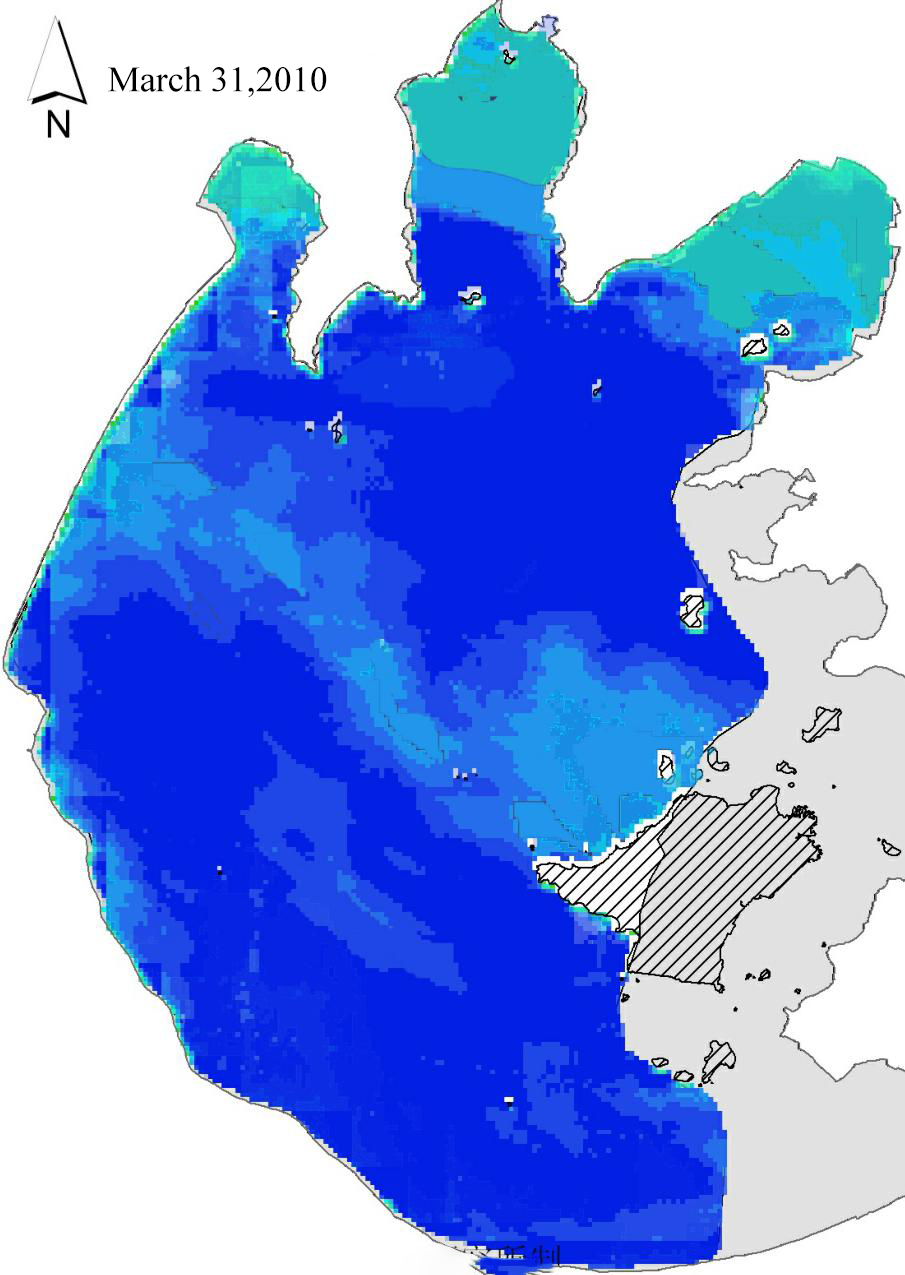

Supplement: Supplemental Information 9 — The data are remote sensing images of chlorophyll a concentration after data scale unification, remote sensing image repair, and time series filling. Remote sensing images of 30 consecutive moments were used as input to the 3D-GAN model. [file peerj-cs-09-1292-s009.zip › 201003310245.jpg]

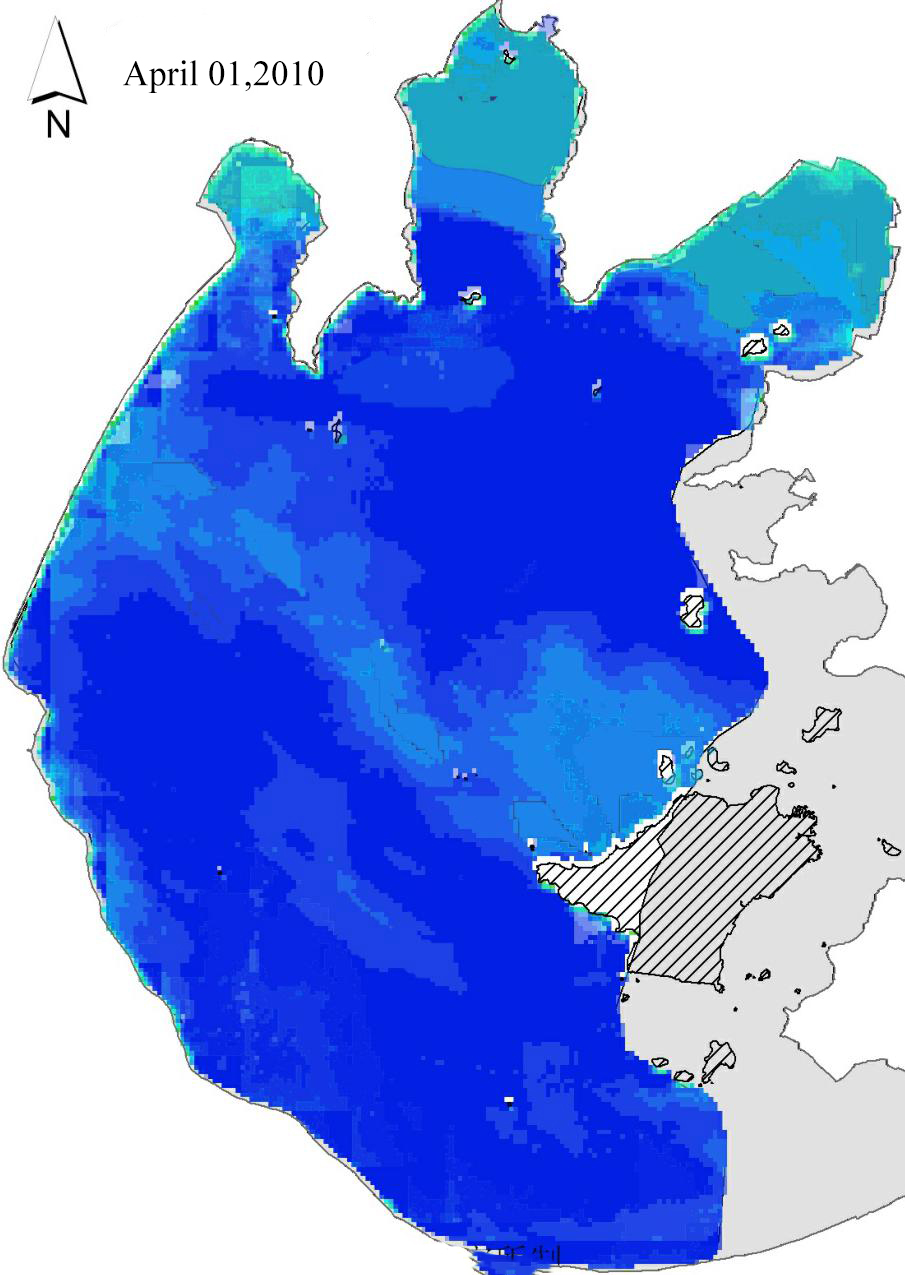

Supplement: Supplemental Information 9 — The data are remote sensing images of chlorophyll a concentration after data scale unification, remote sensing image repair, and time series filling. Remote sensing images of 30 consecutive moments were used as input to the 3D-GAN model. [file peerj-cs-09-1292-s009.zip › 201004010245.jpg]

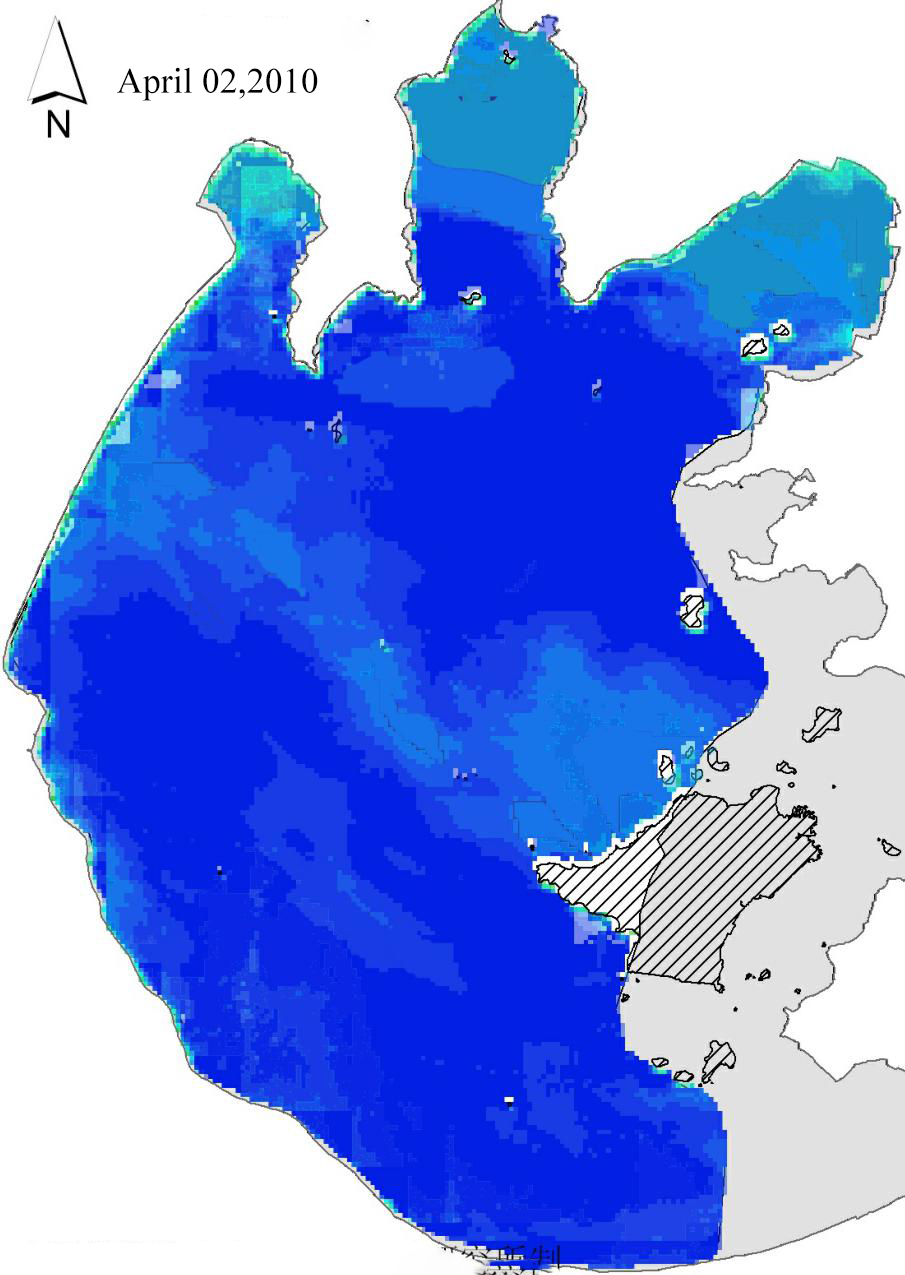

Supplement: Supplemental Information 9 — The data are remote sensing images of chlorophyll a concentration after data scale unification, remote sensing image repair, and time series filling. Remote sensing images of 30 consecutive moments were used as input to the 3D-GAN model. [file peerj-cs-09-1292-s009.zip › 201004020245.jpg]

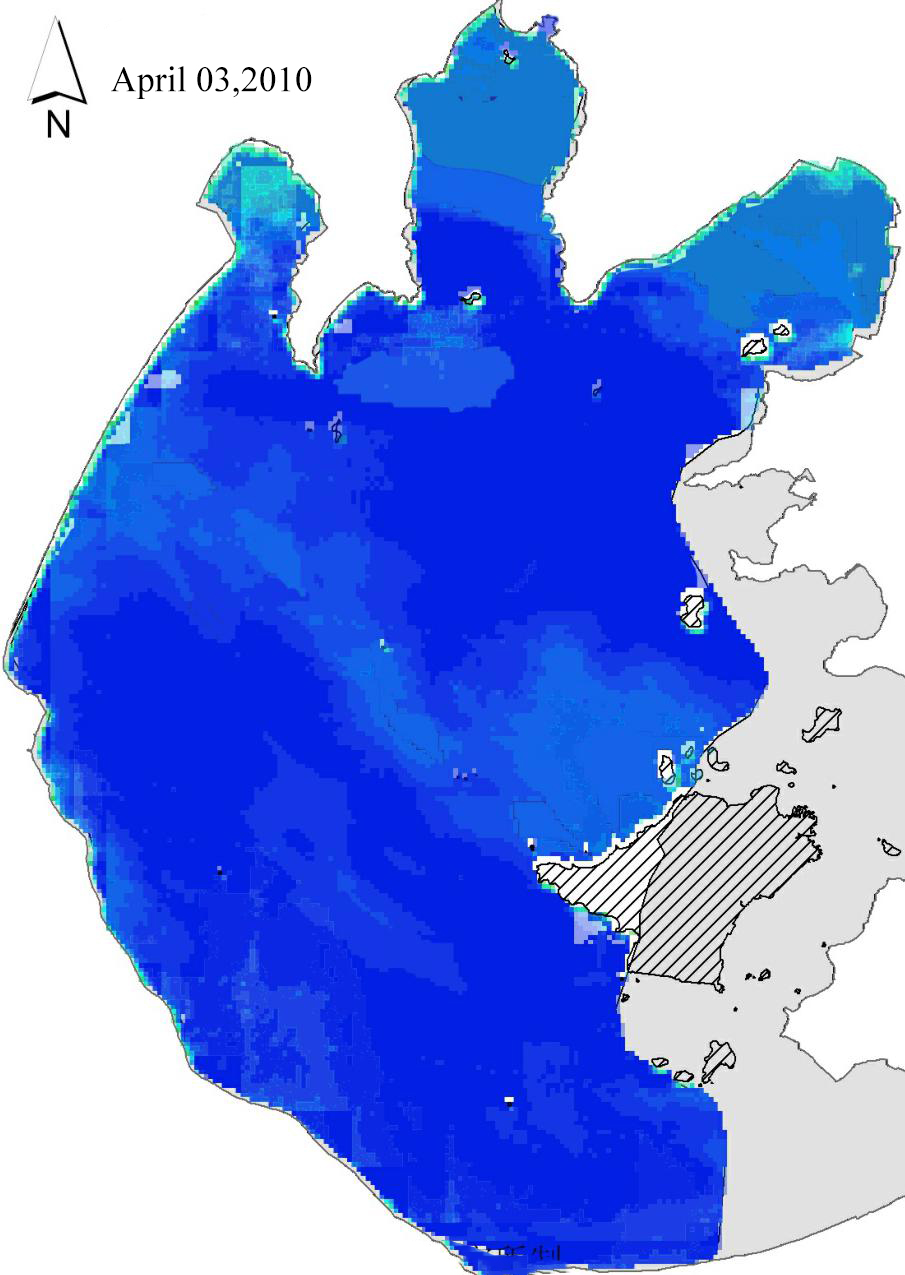

Supplement: Supplemental Information 9 — The data are remote sensing images of chlorophyll a concentration after data scale unification, remote sensing image repair, and time series filling. Remote sensing images of 30 consecutive moments were used as input to the 3D-GAN model. [file peerj-cs-09-1292-s009.zip › 201004030245.jpg]

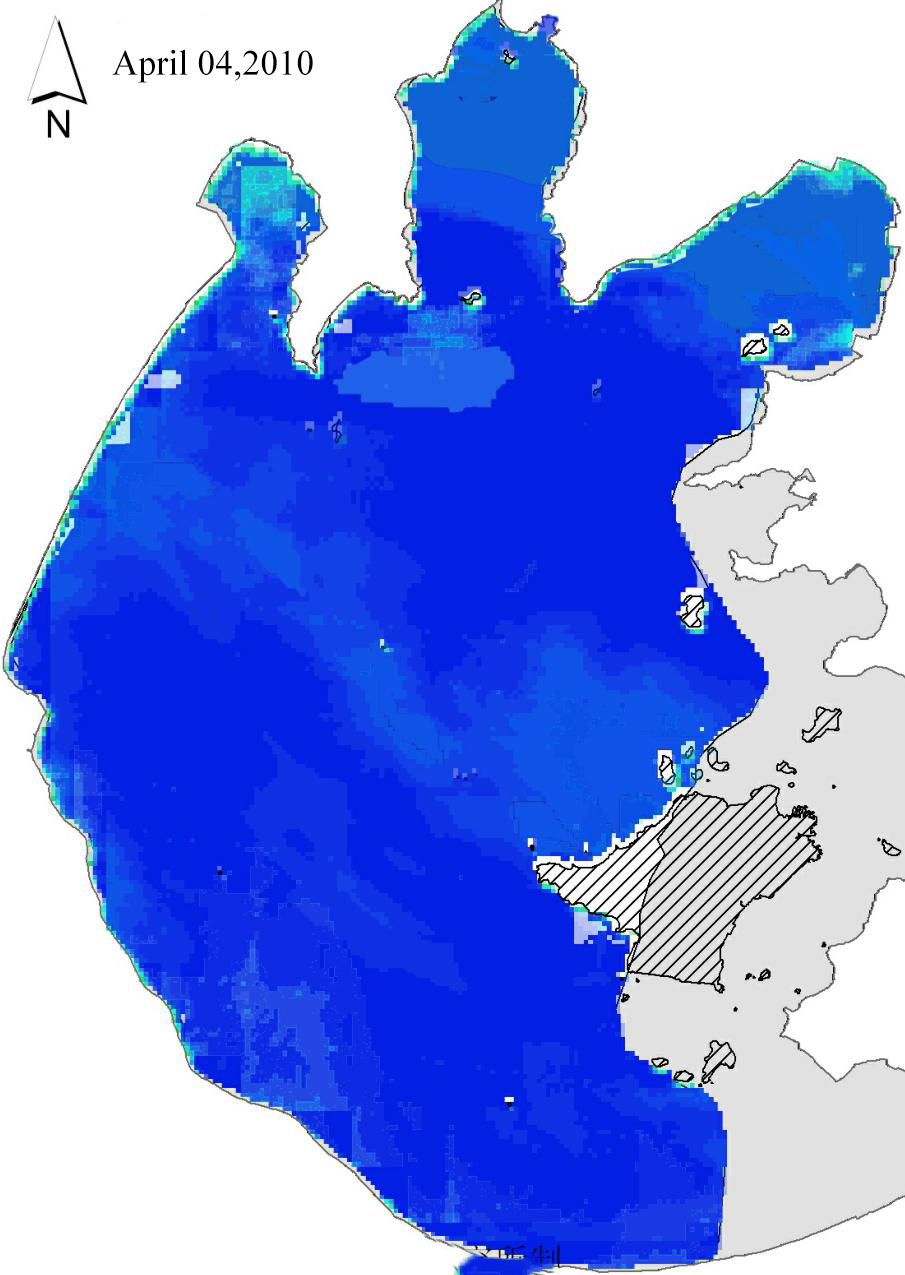

Supplement: Supplemental Information 9 — The data are remote sensing images of chlorophyll a concentration after data scale unification, remote sensing image repair, and time series filling. Remote sensing images of 30 consecutive moments were used as input to the 3D-GAN model. [file peerj-cs-09-1292-s009.zip › 201004040245.jpg]

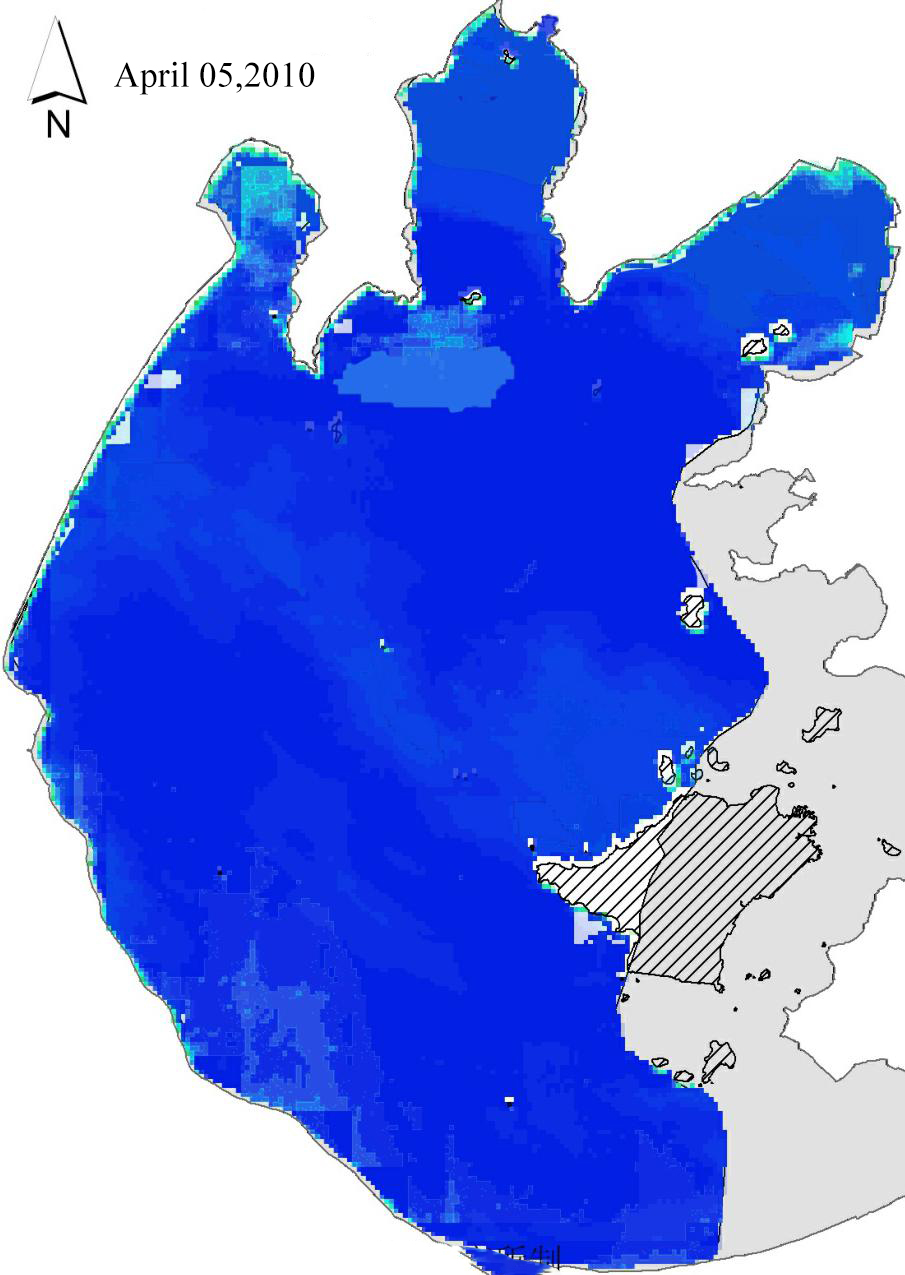

Supplement: Supplemental Information 9 — The data are remote sensing images of chlorophyll a concentration after data scale unification, remote sensing image repair, and time series filling. Remote sensing images of 30 consecutive moments were used as input to the 3D-GAN model. [file peerj-cs-09-1292-s009.zip › 201004050245.jpg]

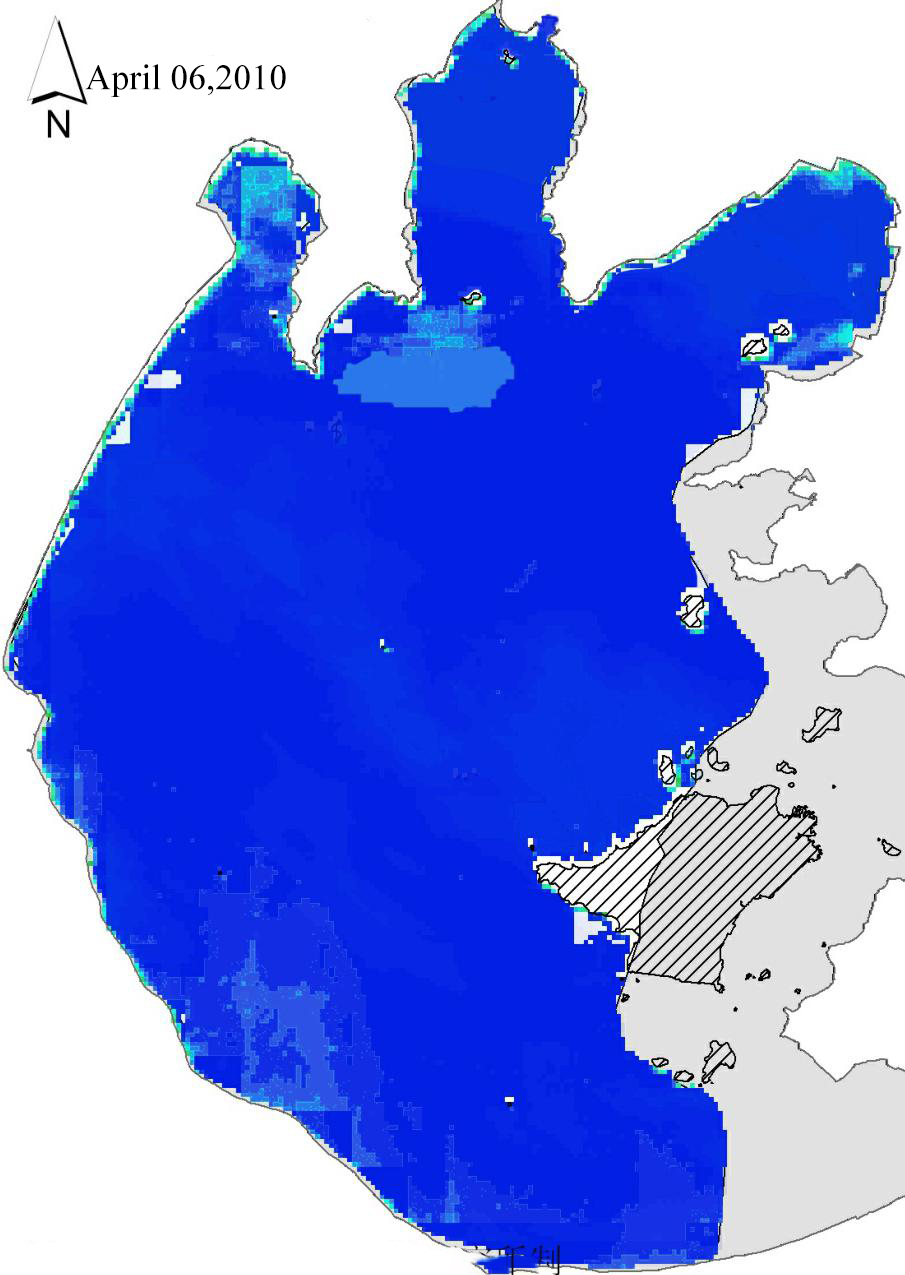

Supplement: Supplemental Information 9 — The data are remote sensing images of chlorophyll a concentration after data scale unification, remote sensing image repair, and time series filling. Remote sensing images of 30 consecutive moments were used as input to the 3D-GAN model. [file peerj-cs-09-1292-s009.zip › 201004060245.jpg]

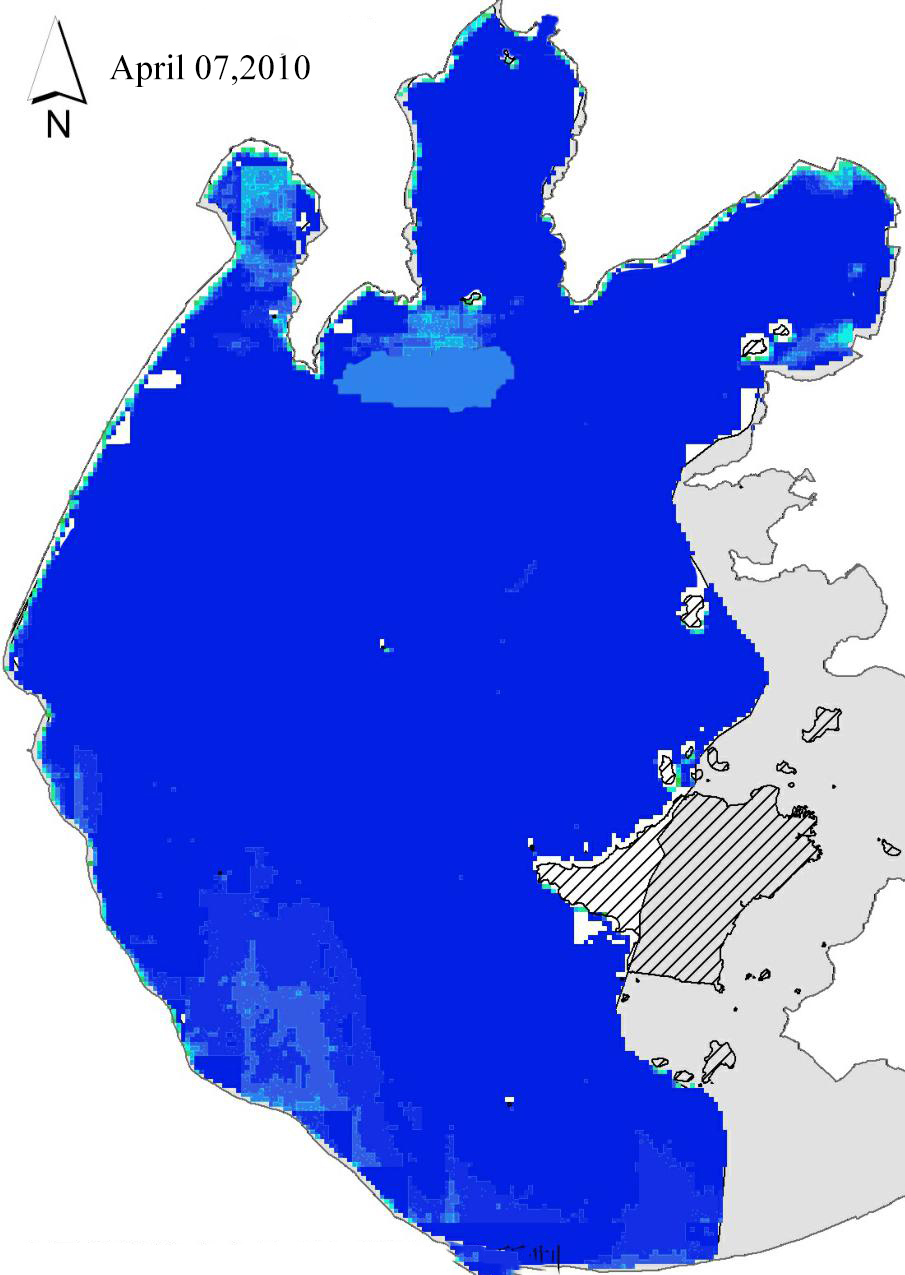

Supplement: Supplemental Information 9 — The data are remote sensing images of chlorophyll a concentration after data scale unification, remote sensing image repair, and time series filling. Remote sensing images of 30 consecutive moments were used as input to the 3D-GAN model. [file peerj-cs-09-1292-s009.zip › 201004070245.jpg]

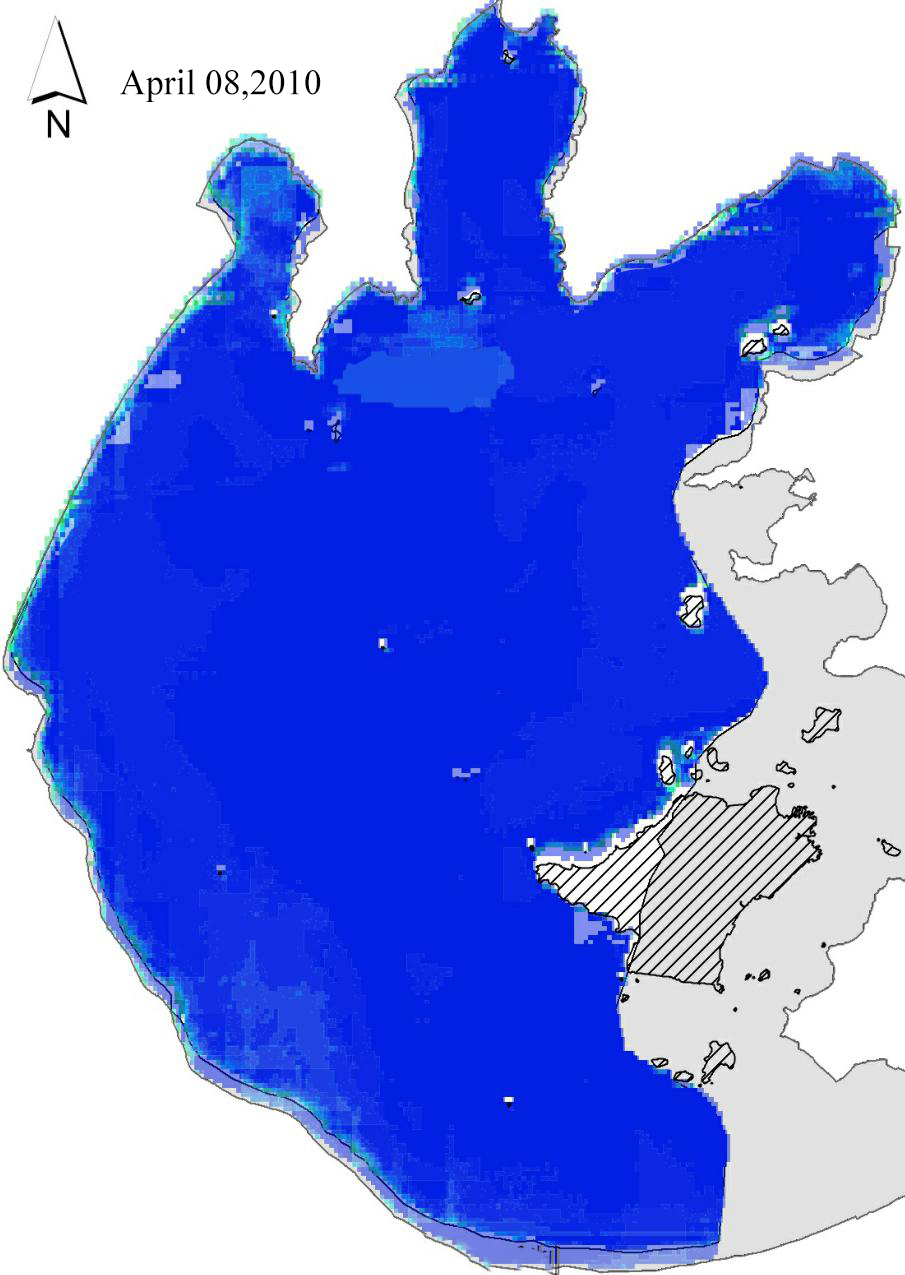

Supplement: Supplemental Information 9 — The data are remote sensing images of chlorophyll a concentration after data scale unification, remote sensing image repair, and time series filling. Remote sensing images of 30 consecutive moments were used as input to the 3D-GAN model. [file peerj-cs-09-1292-s009.zip › 201004080245.jpg]

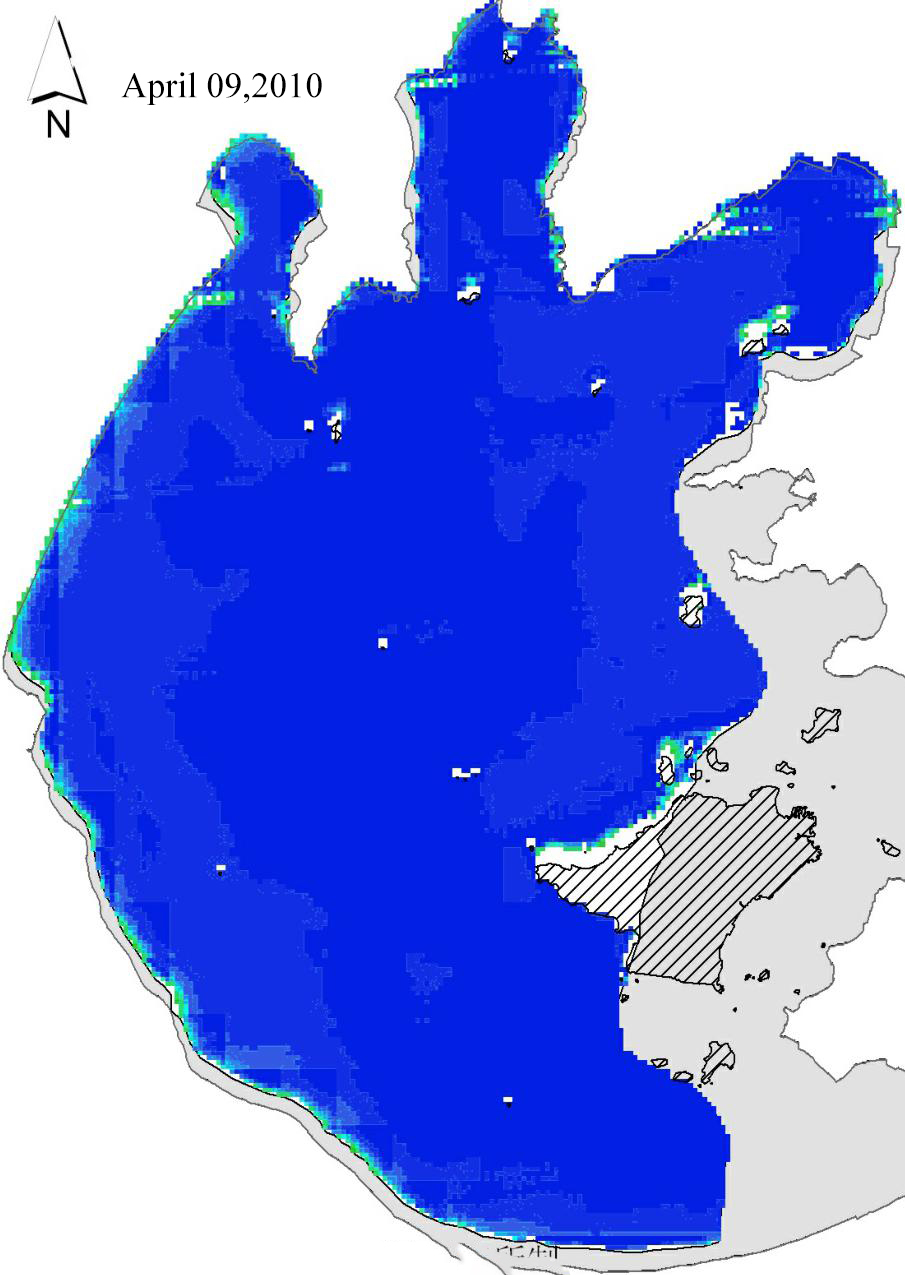

Supplement: Supplemental Information 9 — The data are remote sensing images of chlorophyll a concentration after data scale unification, remote sensing image repair, and time series filling. Remote sensing images of 30 consecutive moments were used as input to the 3D-GAN model. [file peerj-cs-09-1292-s009.zip › 201004090245.jpg]

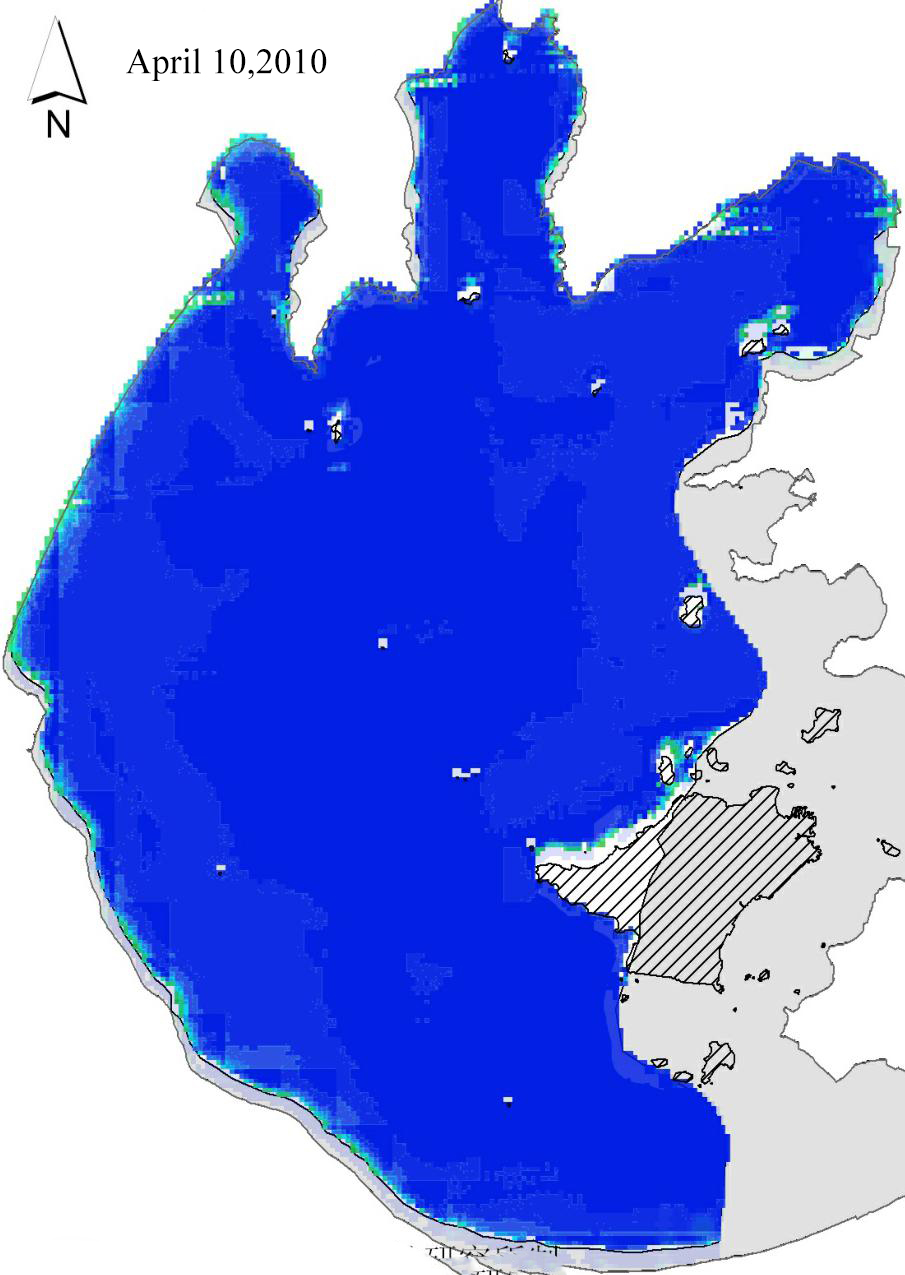

Supplement: Supplemental Information 9 — The data are remote sensing images of chlorophyll a concentration after data scale unification, remote sensing image repair, and time series filling. Remote sensing images of 30 consecutive moments were used as input to the 3D-GAN model. [file peerj-cs-09-1292-s009.zip › 201004100245.jpg]

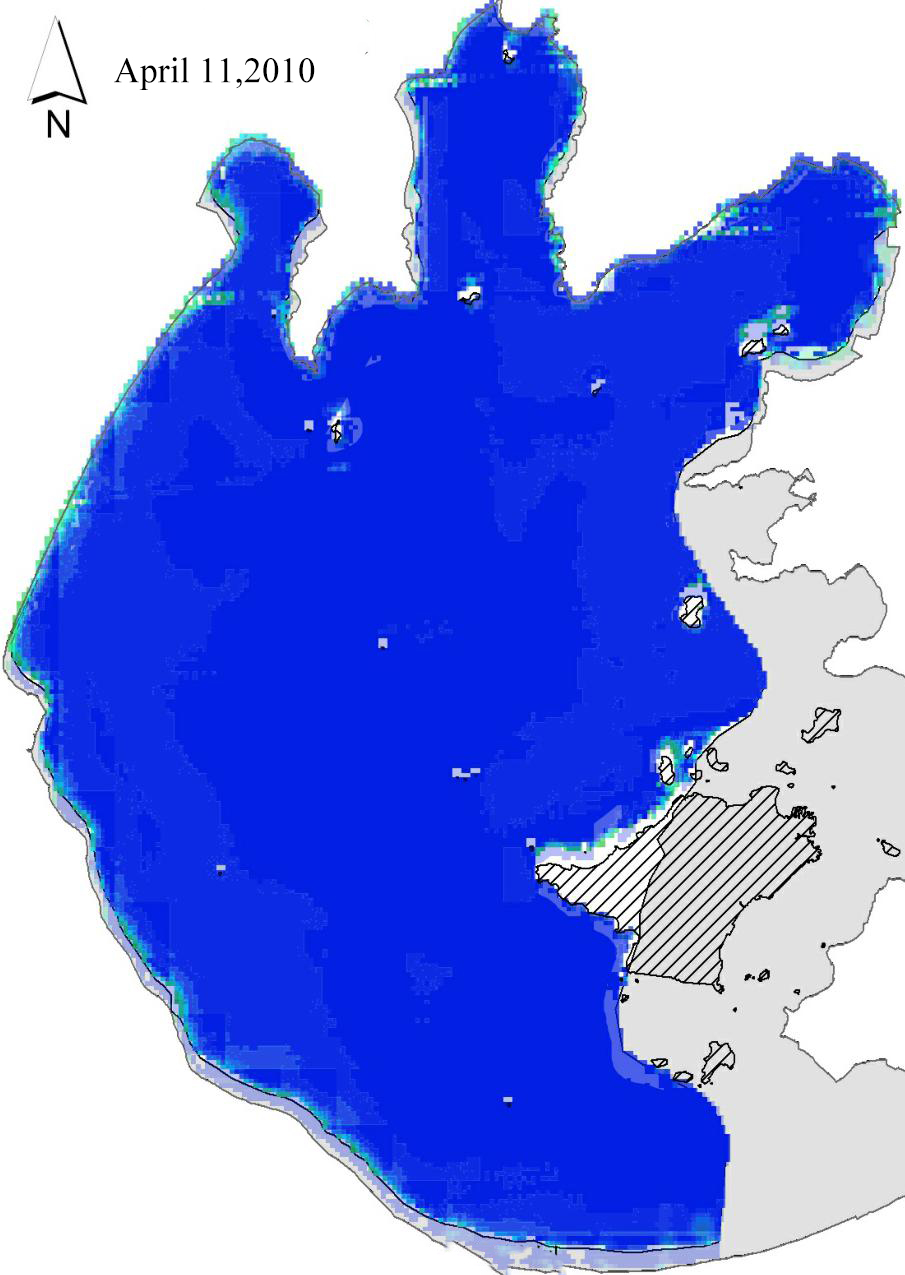

Supplement: Supplemental Information 9 — The data are remote sensing images of chlorophyll a concentration after data scale unification, remote sensing image repair, and time series filling. Remote sensing images of 30 consecutive moments were used as input to the 3D-GAN model. [file peerj-cs-09-1292-s009.zip › 201004110245.jpg]

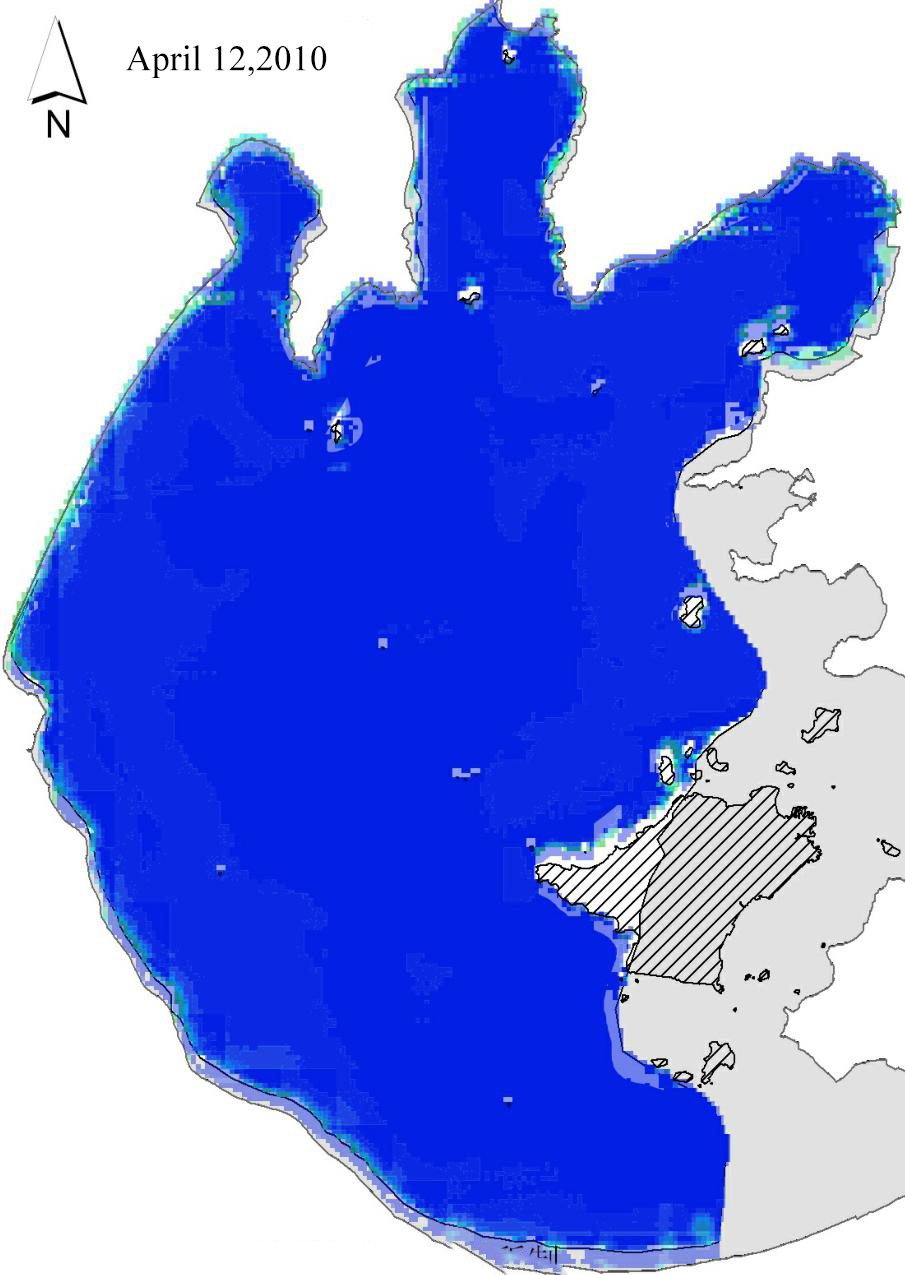

Supplement: Supplemental Information 9 — The data are remote sensing images of chlorophyll a concentration after data scale unification, remote sensing image repair, and time series filling. Remote sensing images of 30 consecutive moments were used as input to the 3D-GAN model. [file peerj-cs-09-1292-s009.zip › 201004120245.jpg]

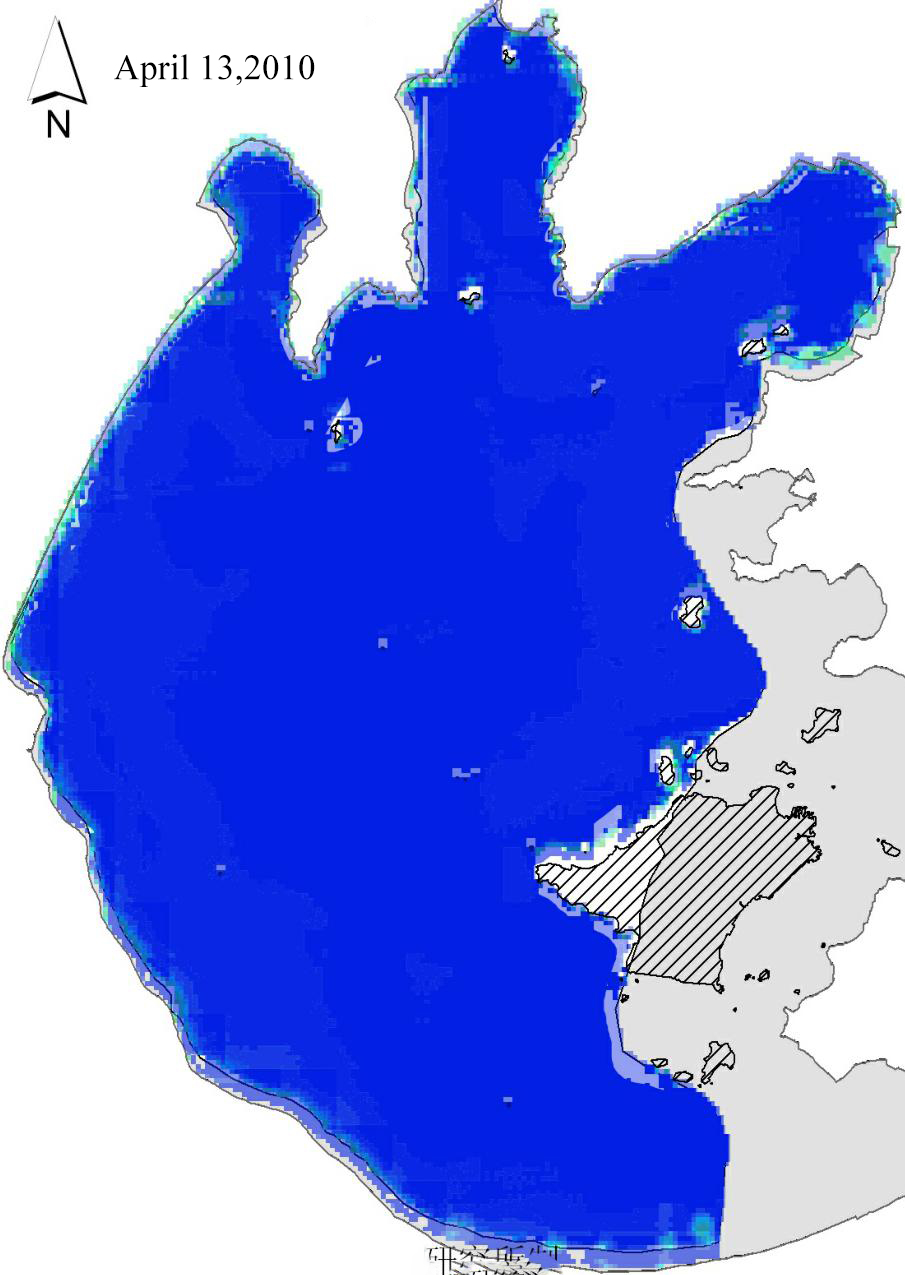

Supplement: Supplemental Information 9 — The data are remote sensing images of chlorophyll a concentration after data scale unification, remote sensing image repair, and time series filling. Remote sensing images of 30 consecutive moments were used as input to the 3D-GAN model. [file peerj-cs-09-1292-s009.zip › 201004130245.jpg]

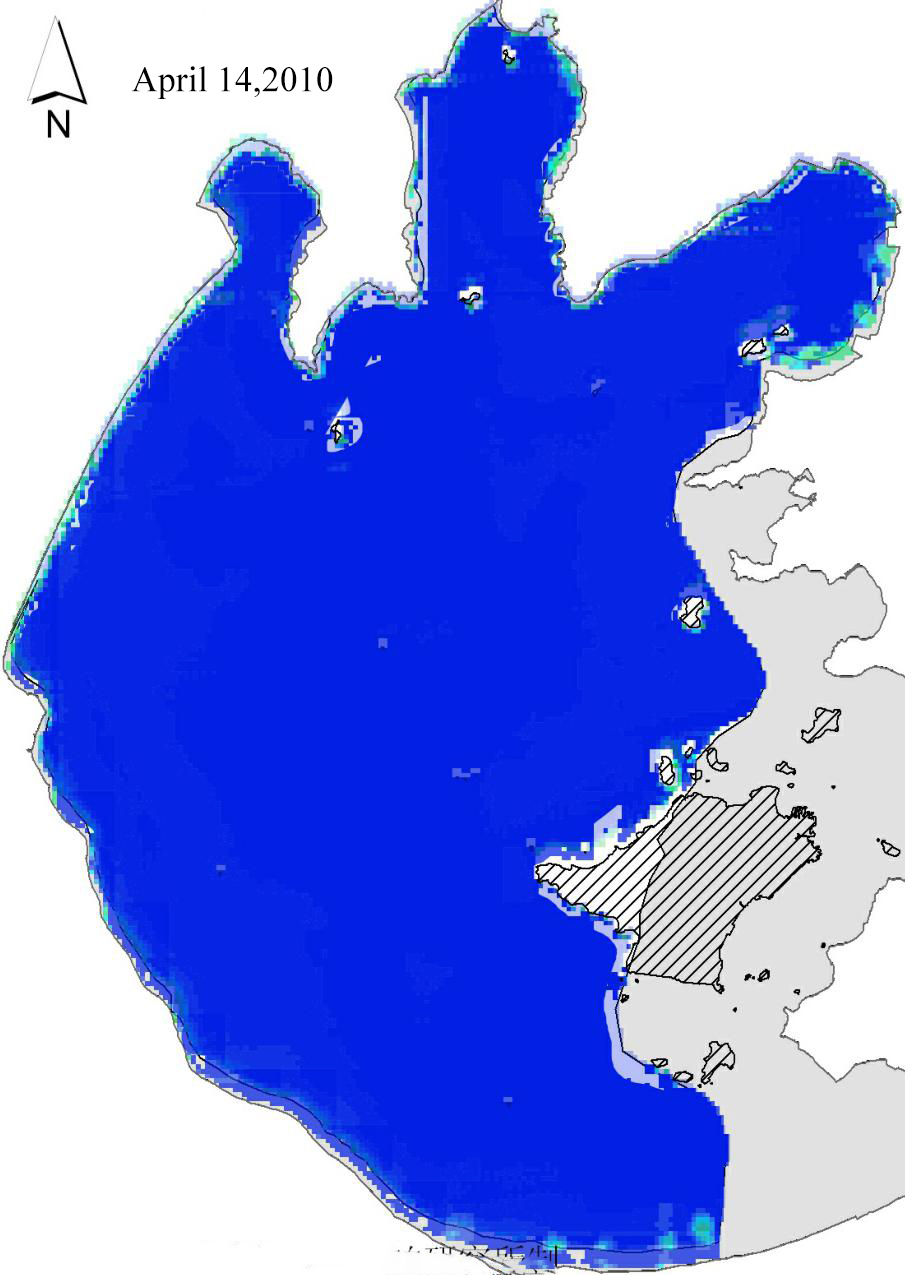

Supplement: Supplemental Information 9 — The data are remote sensing images of chlorophyll a concentration after data scale unification, remote sensing image repair, and time series filling. Remote sensing images of 30 consecutive moments were used as input to the 3D-GAN model. [file peerj-cs-09-1292-s009.zip › 201004140245.jpg]

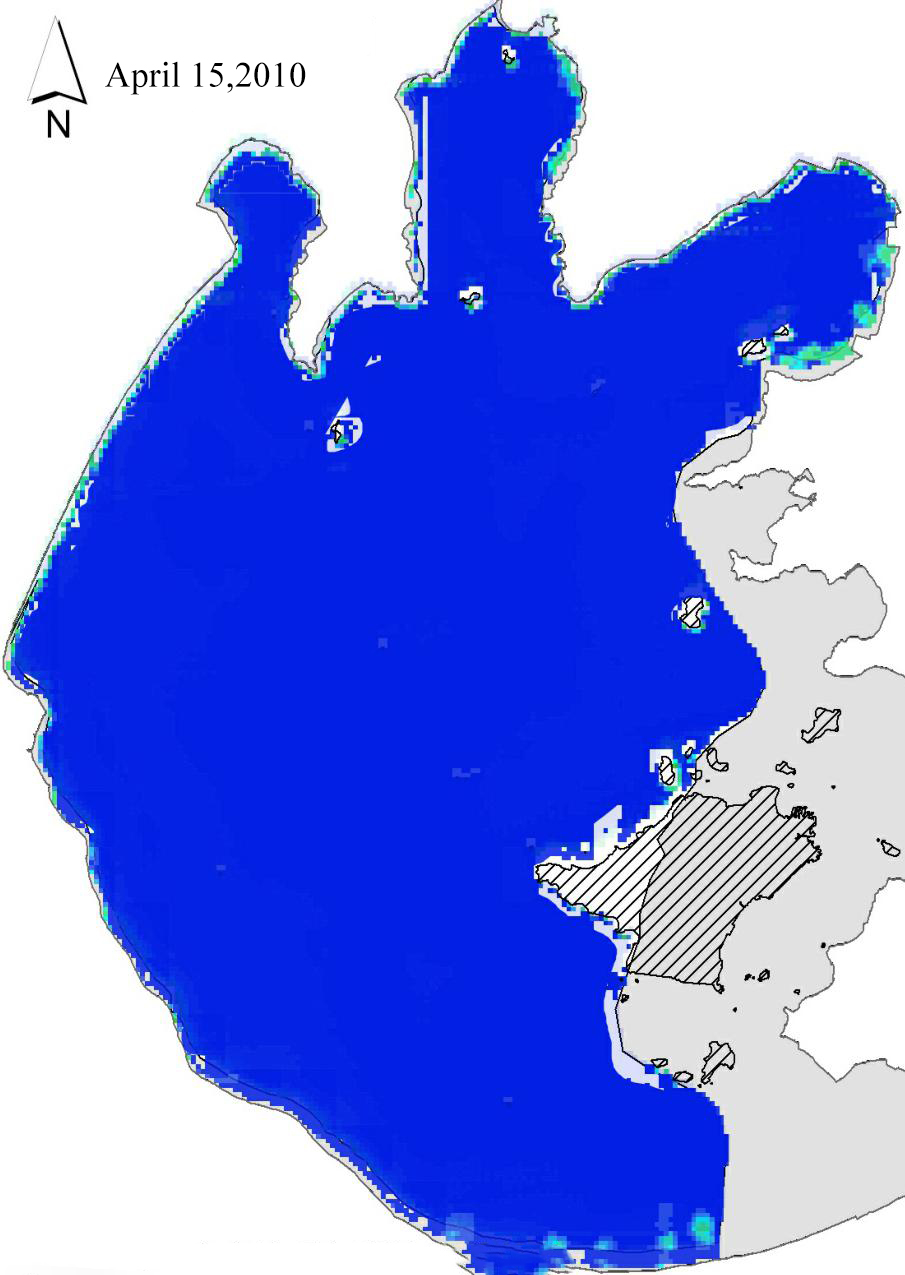

Supplement: Supplemental Information 9 — The data are remote sensing images of chlorophyll a concentration after data scale unification, remote sensing image repair, and time series filling. Remote sensing images of 30 consecutive moments were used as input to the 3D-GAN model. [file peerj-cs-09-1292-s009.zip › 201004150245.jpg]

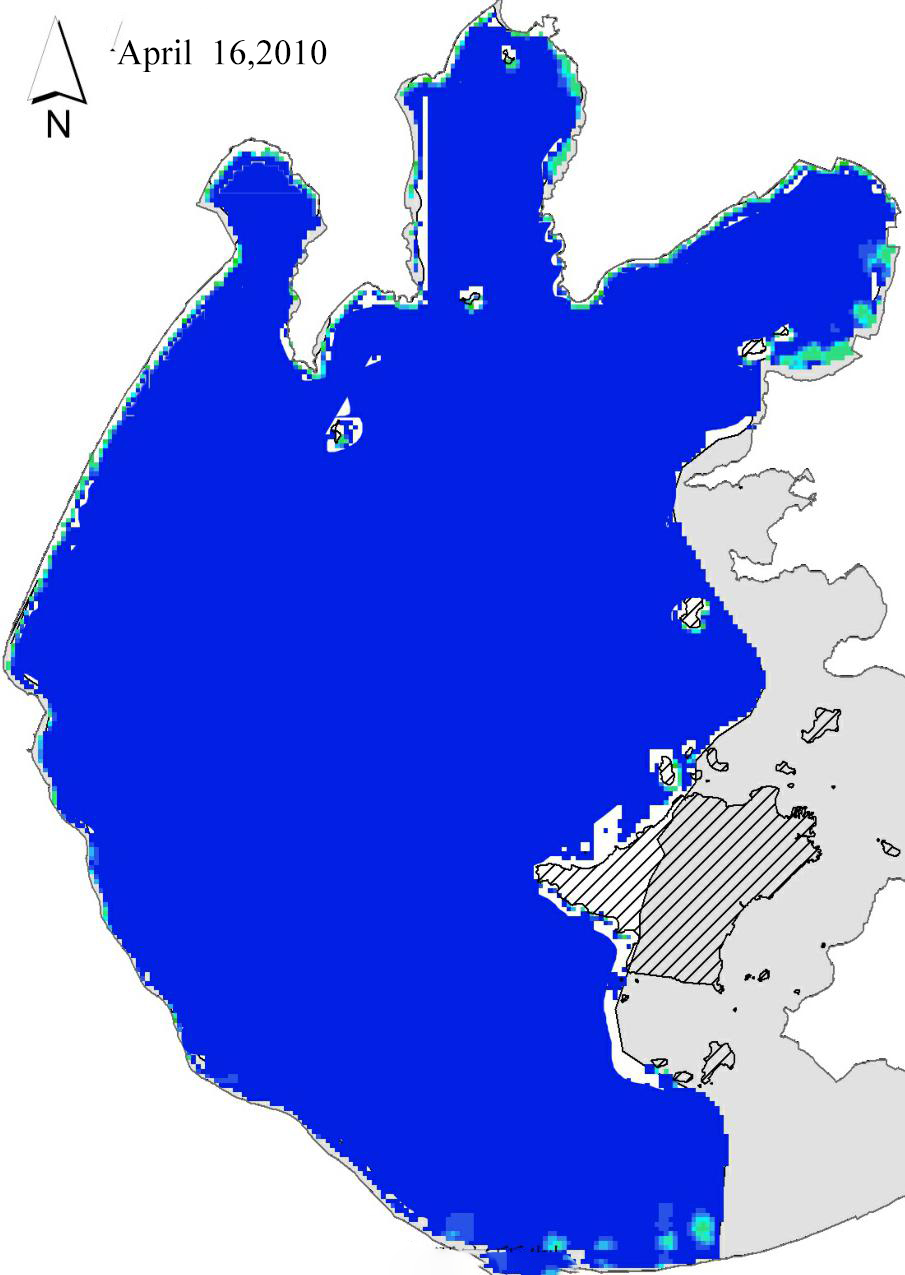

Supplement: Supplemental Information 9 — The data are remote sensing images of chlorophyll a concentration after data scale unification, remote sensing image repair, and time series filling. Remote sensing images of 30 consecutive moments were used as input to the 3D-GAN model. [file peerj-cs-09-1292-s009.zip › 201004160245.jpg]

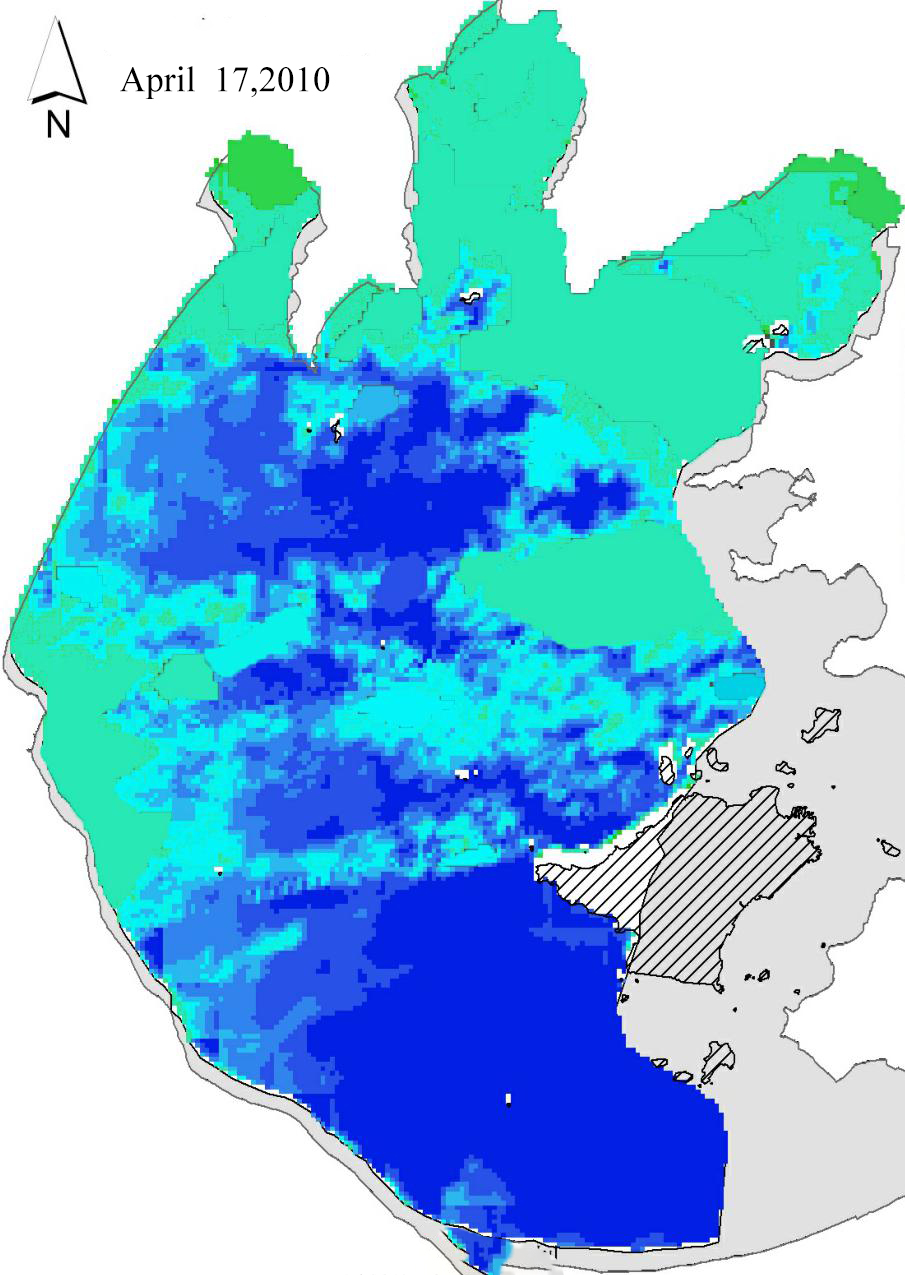

Supplement: Supplemental Information 9 — The data are remote sensing images of chlorophyll a concentration after data scale unification, remote sensing image repair, and time series filling. Remote sensing images of 30 consecutive moments were used as input to the 3D-GAN model. [file peerj-cs-09-1292-s009.zip › 201004170245.jpg]

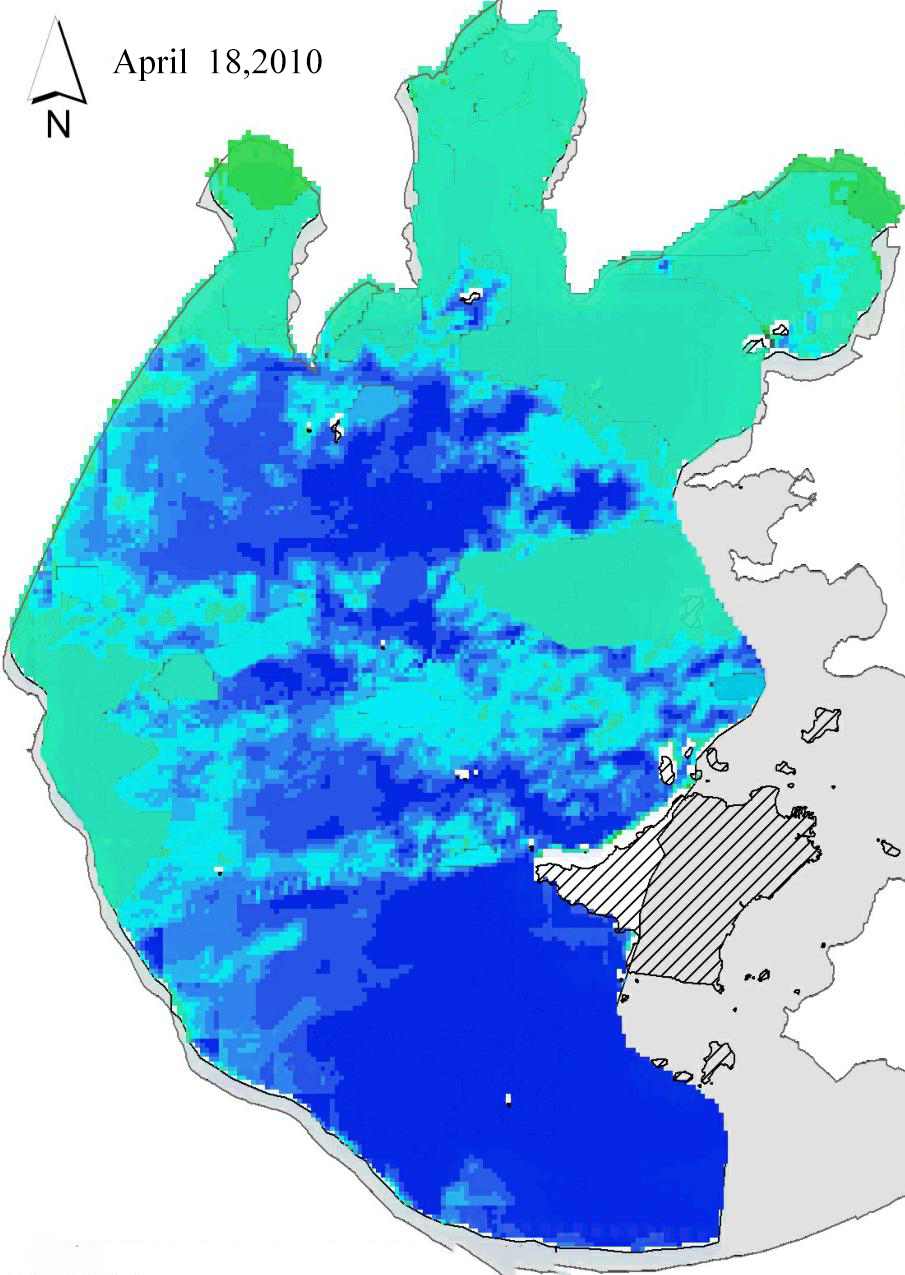

Supplement: Supplemental Information 9 — The data are remote sensing images of chlorophyll a concentration after data scale unification, remote sensing image repair, and time series filling. Remote sensing images of 30 consecutive moments were used as input to the 3D-GAN model. [file peerj-cs-09-1292-s009.zip › 201004180245.jpg]

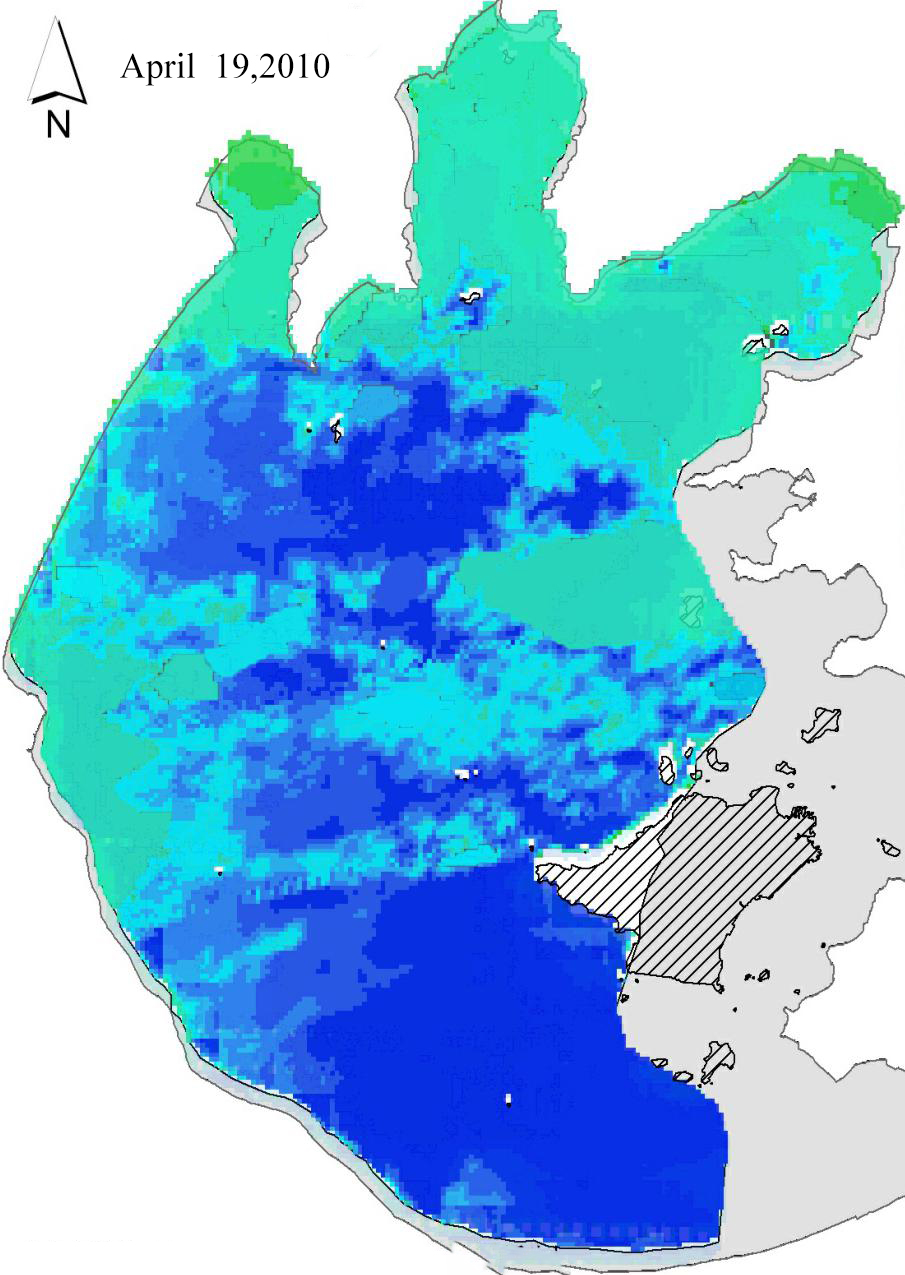

Supplement: Supplemental Information 9 — The data are remote sensing images of chlorophyll a concentration after data scale unification, remote sensing image repair, and time series filling. Remote sensing images of 30 consecutive moments were used as input to the 3D-GAN model. [file peerj-cs-09-1292-s009.zip › 201004190245.jpg]

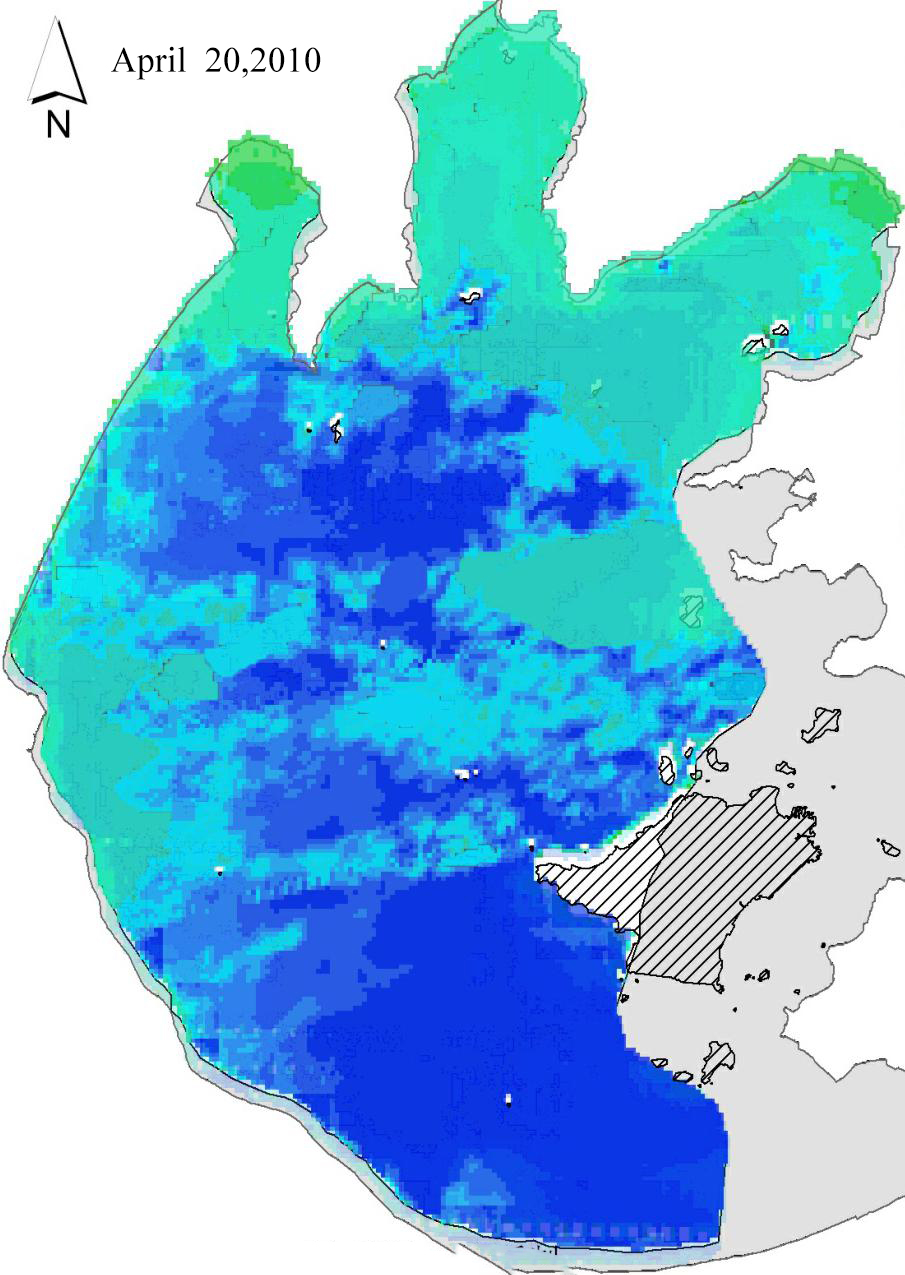

Supplement: Supplemental Information 9 — The data are remote sensing images of chlorophyll a concentration after data scale unification, remote sensing image repair, and time series filling. Remote sensing images of 30 consecutive moments were used as input to the 3D-GAN model. [file peerj-cs-09-1292-s009.zip › 201004200245.jpg]

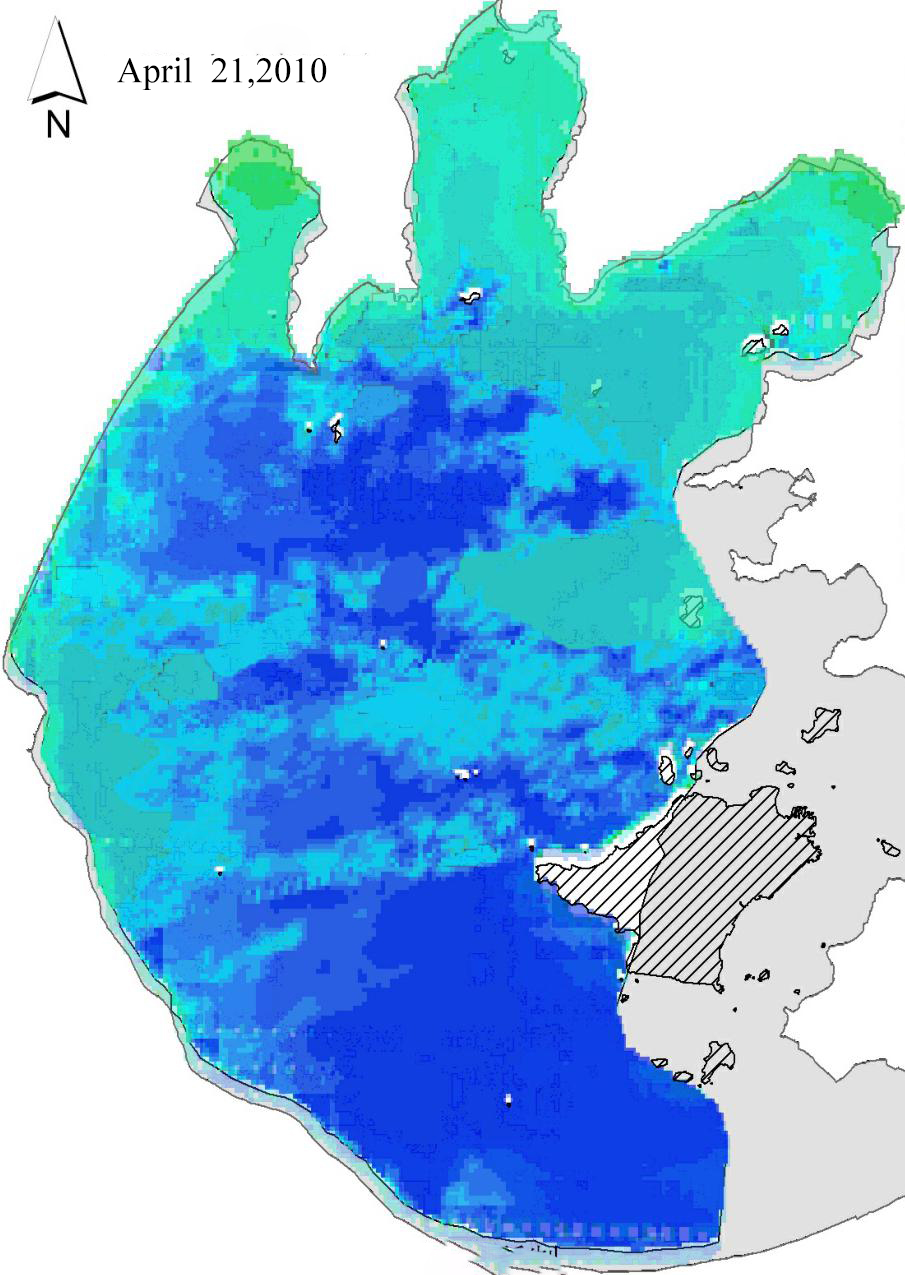

Supplement: Supplemental Information 9 — The data are remote sensing images of chlorophyll a concentration after data scale unification, remote sensing image repair, and time series filling. Remote sensing images of 30 consecutive moments were used as input to the 3D-GAN model. [file peerj-cs-09-1292-s009.zip › 201004210245.jpg]

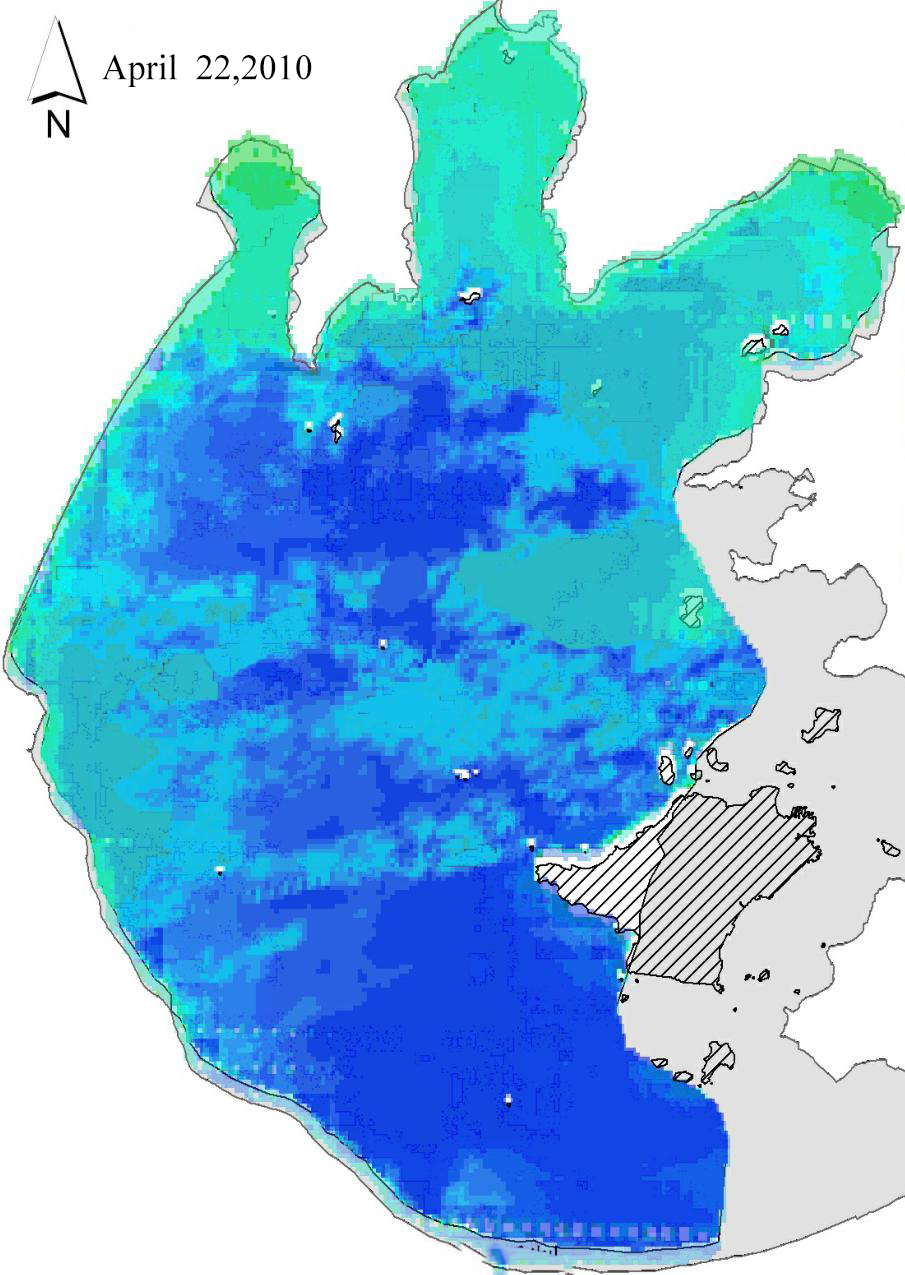

Supplement: Supplemental Information 9 — The data are remote sensing images of chlorophyll a concentration after data scale unification, remote sensing image repair, and time series filling. Remote sensing images of 30 consecutive moments were used as input to the 3D-GAN model. [file peerj-cs-09-1292-s009.zip › 201004220245.jpg]

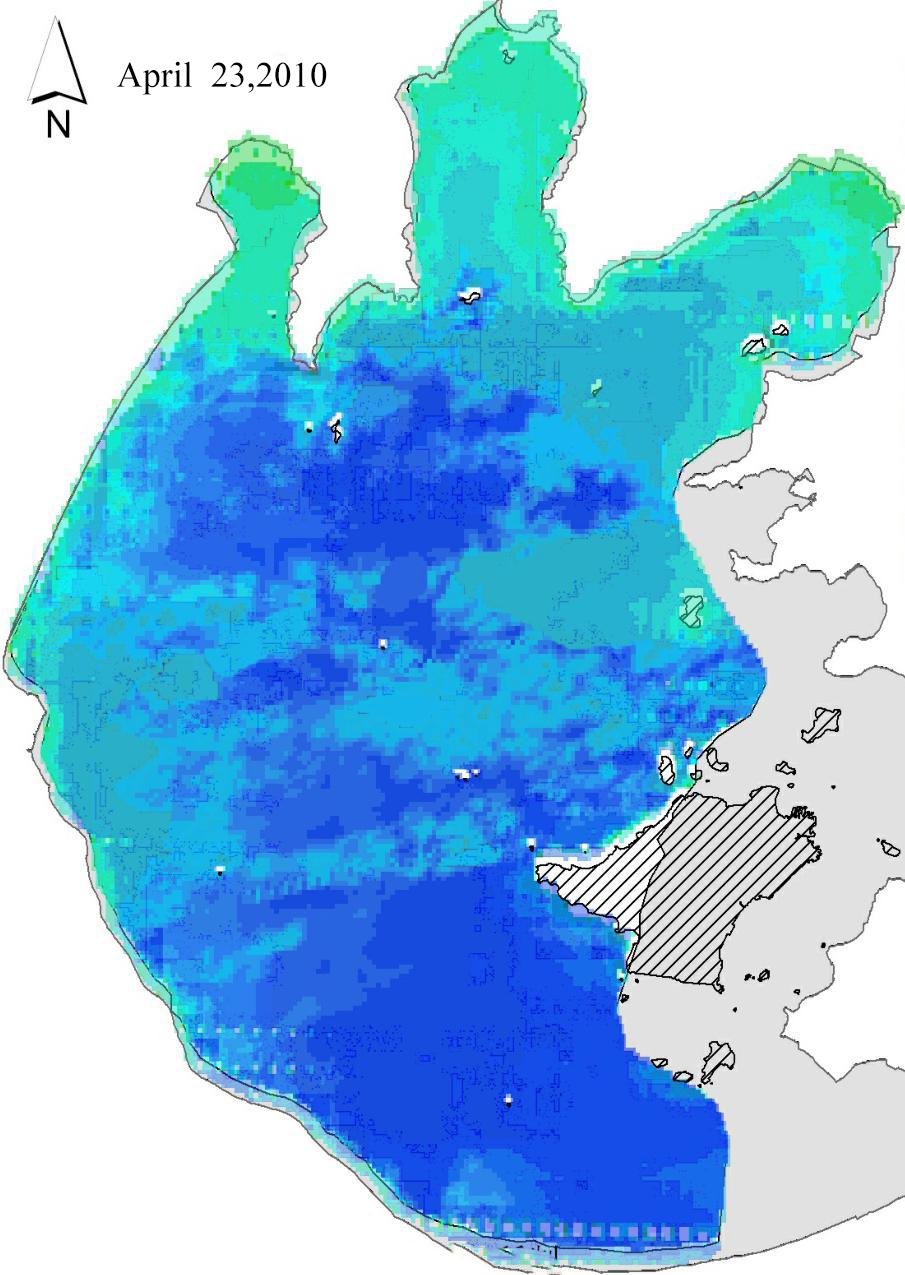

Supplement: Supplemental Information 9 — The data are remote sensing images of chlorophyll a concentration after data scale unification, remote sensing image repair, and time series filling. Remote sensing images of 30 consecutive moments were used as input to the 3D-GAN model. [file peerj-cs-09-1292-s009.zip › 201004230245.jpg]

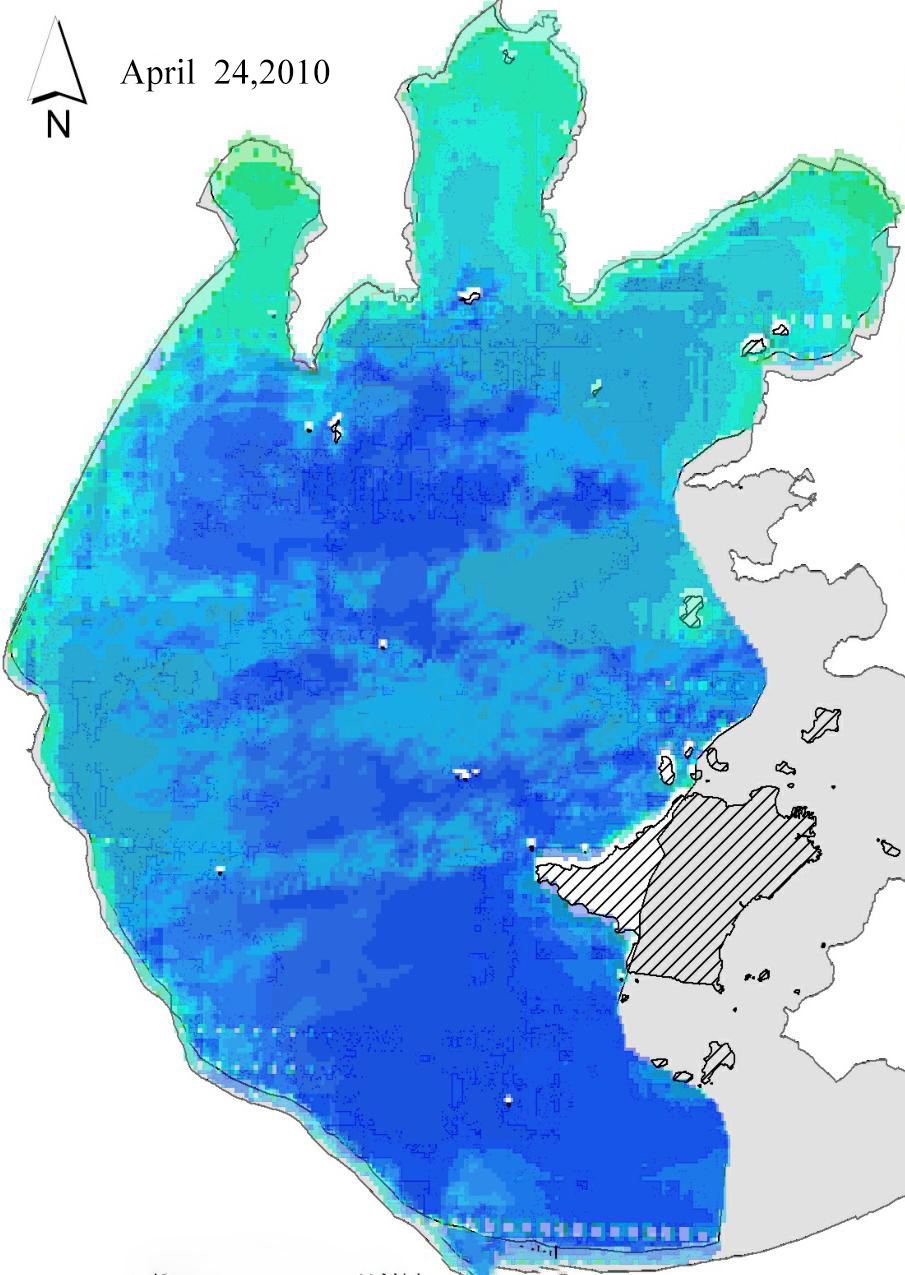

Supplement: Supplemental Information 9 — The data are remote sensing images of chlorophyll a concentration after data scale unification, remote sensing image repair, and time series filling. Remote sensing images of 30 consecutive moments were used as input to the 3D-GAN model. [file peerj-cs-09-1292-s009.zip › 201004240245.jpg]

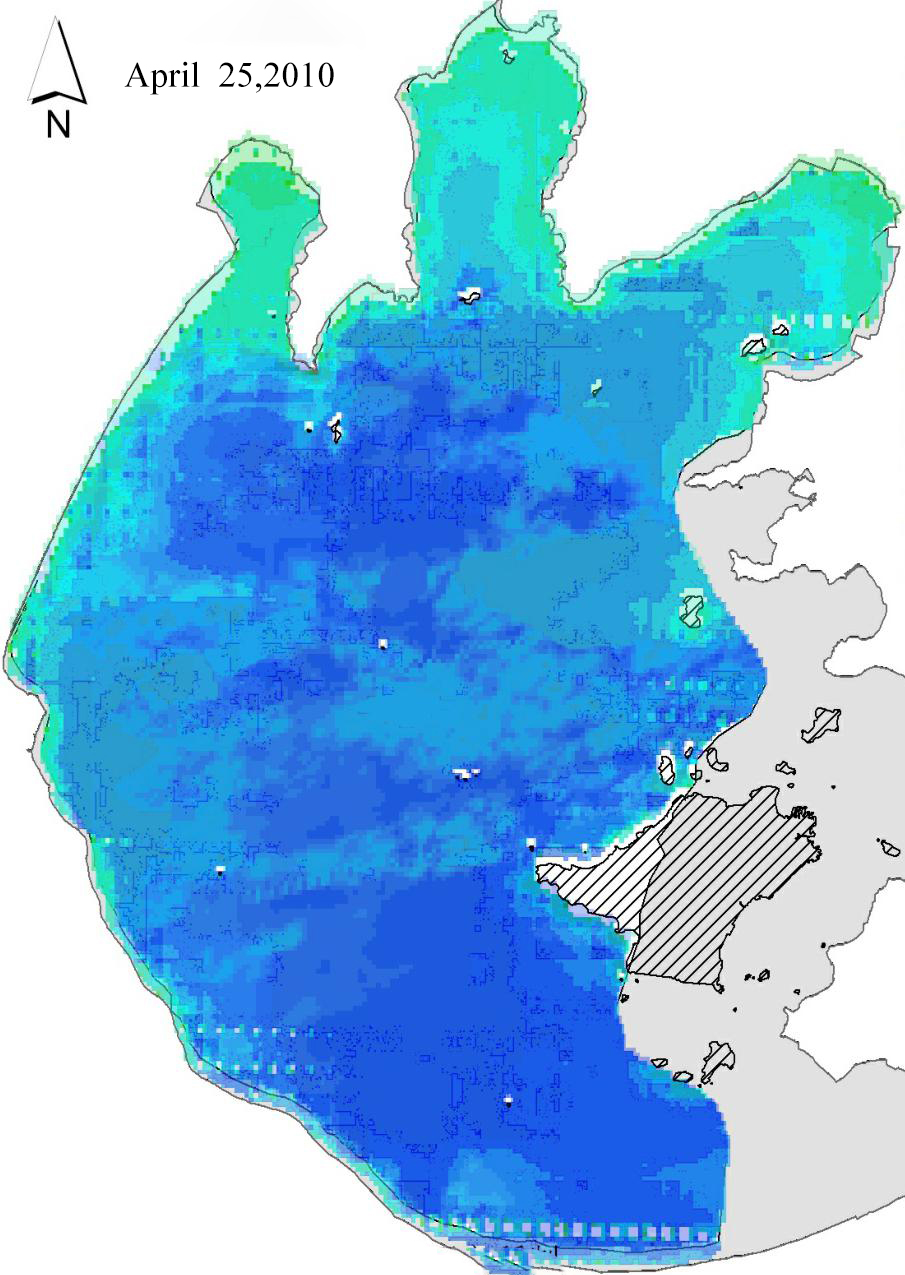

Supplement: Supplemental Information 9 — The data are remote sensing images of chlorophyll a concentration after data scale unification, remote sensing image repair, and time series filling. Remote sensing images of 30 consecutive moments were used as input to the 3D-GAN model. [file peerj-cs-09-1292-s009.zip › 201004250245.jpg]

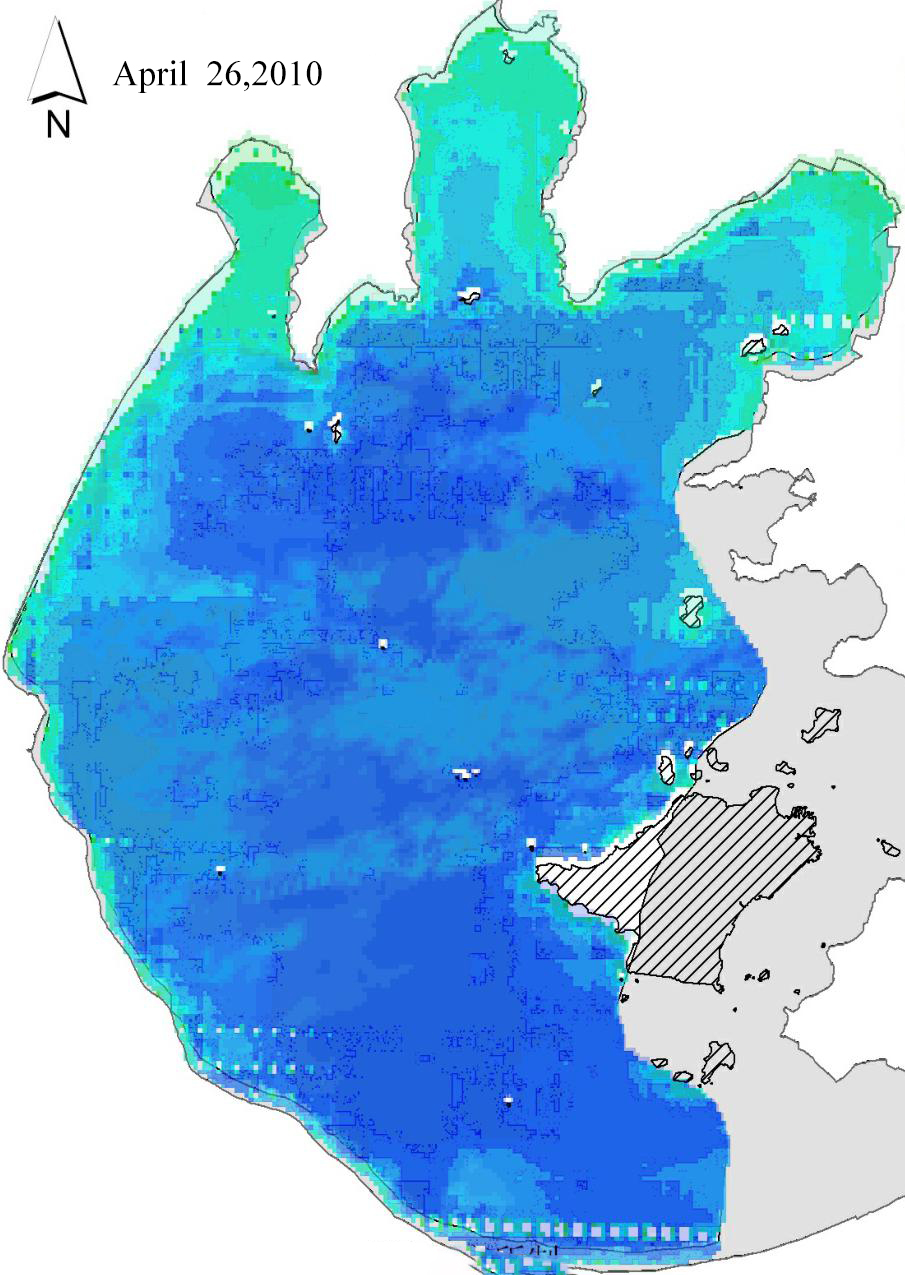

Supplement: Supplemental Information 9 — The data are remote sensing images of chlorophyll a concentration after data scale unification, remote sensing image repair, and time series filling. Remote sensing images of 30 consecutive moments were used as input to the 3D-GAN model. [file peerj-cs-09-1292-s009.zip › 201004260245.jpg]

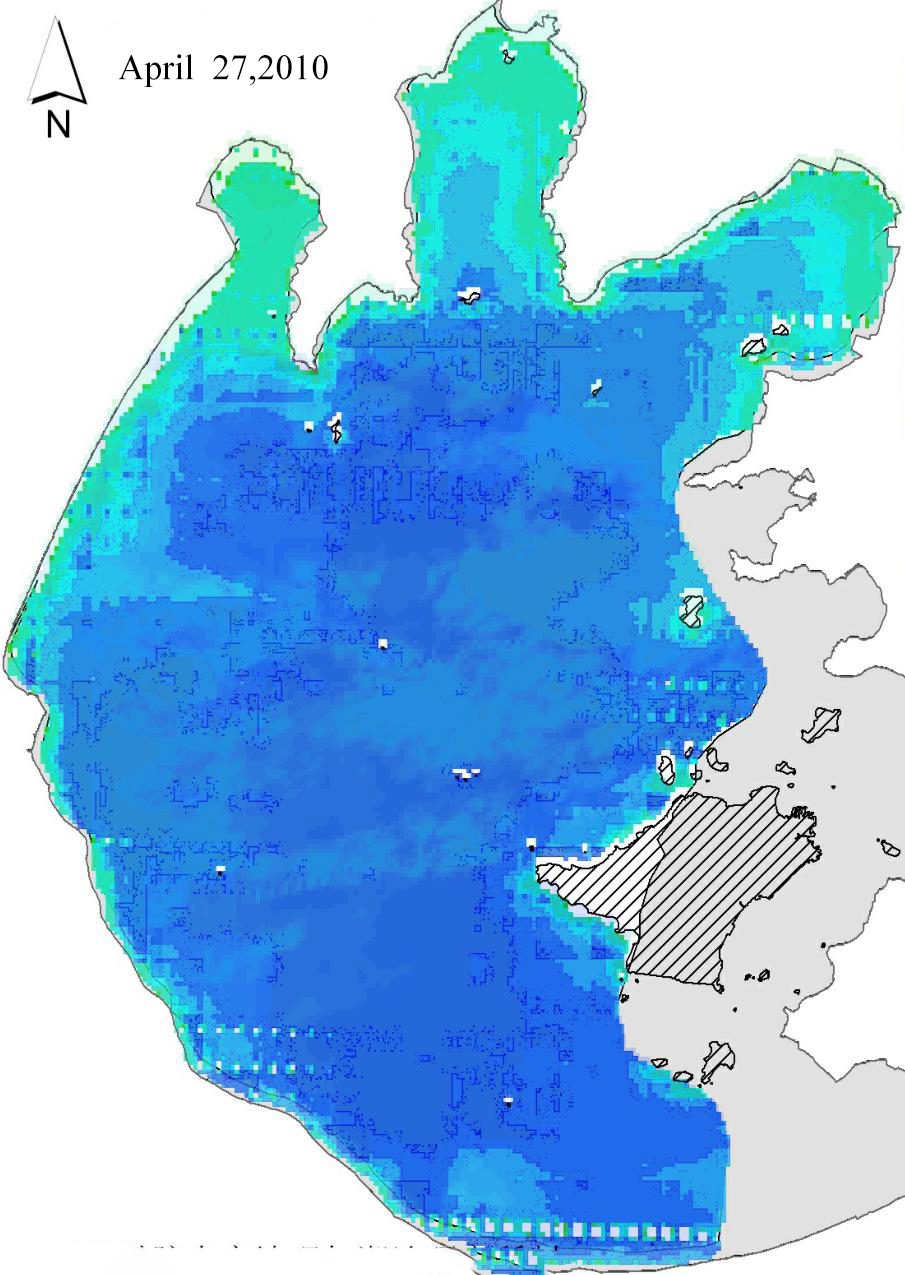

Supplement: Supplemental Information 9 — The data are remote sensing images of chlorophyll a concentration after data scale unification, remote sensing image repair, and time series filling. Remote sensing images of 30 consecutive moments were used as input to the 3D-GAN model. [file peerj-cs-09-1292-s009.zip › 201004270245.jpg]

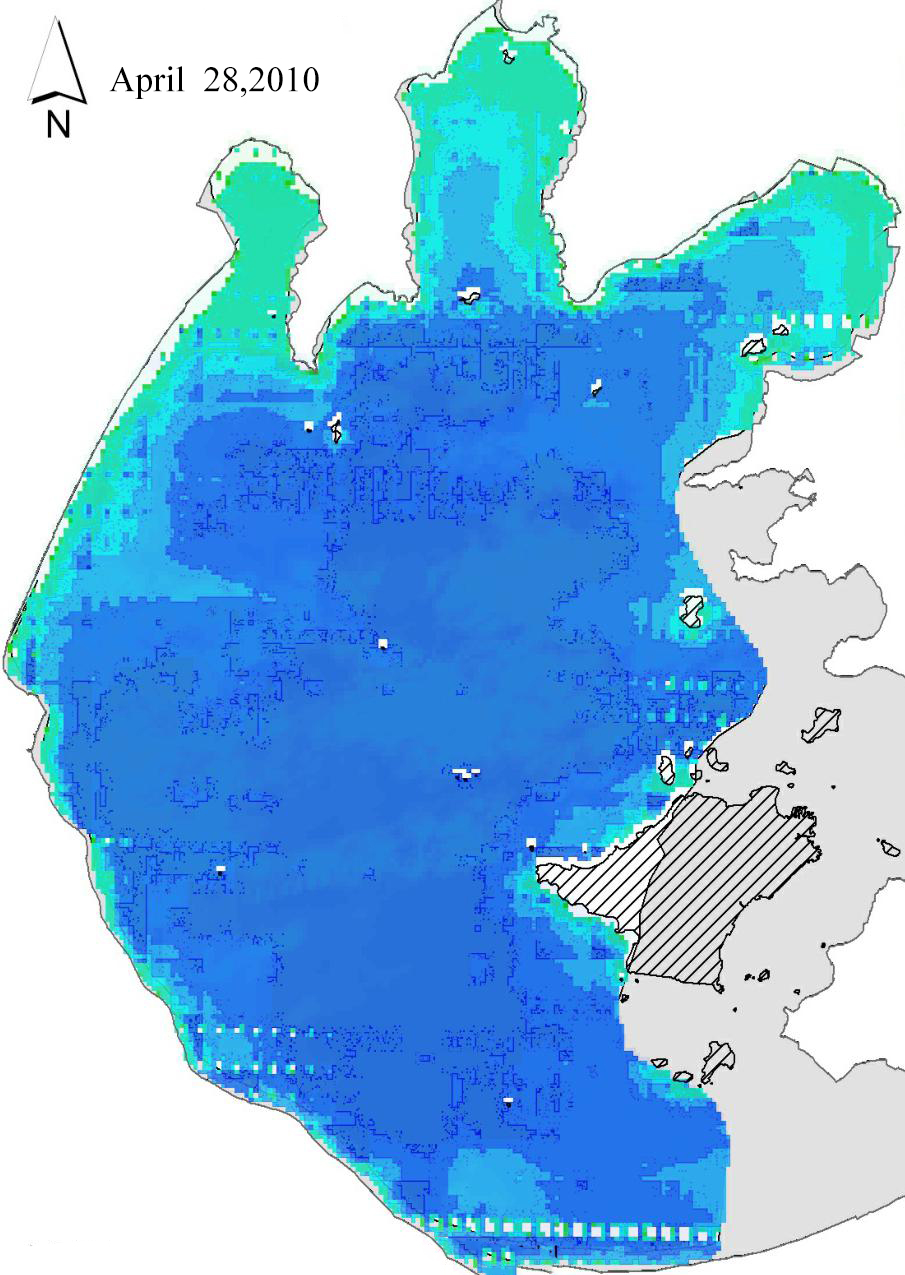

Supplement: Supplemental Information 9 — The data are remote sensing images of chlorophyll a concentration after data scale unification, remote sensing image repair, and time series filling. Remote sensing images of 30 consecutive moments were used as input to the 3D-GAN model. [file peerj-cs-09-1292-s009.zip › 201004280245.jpg]

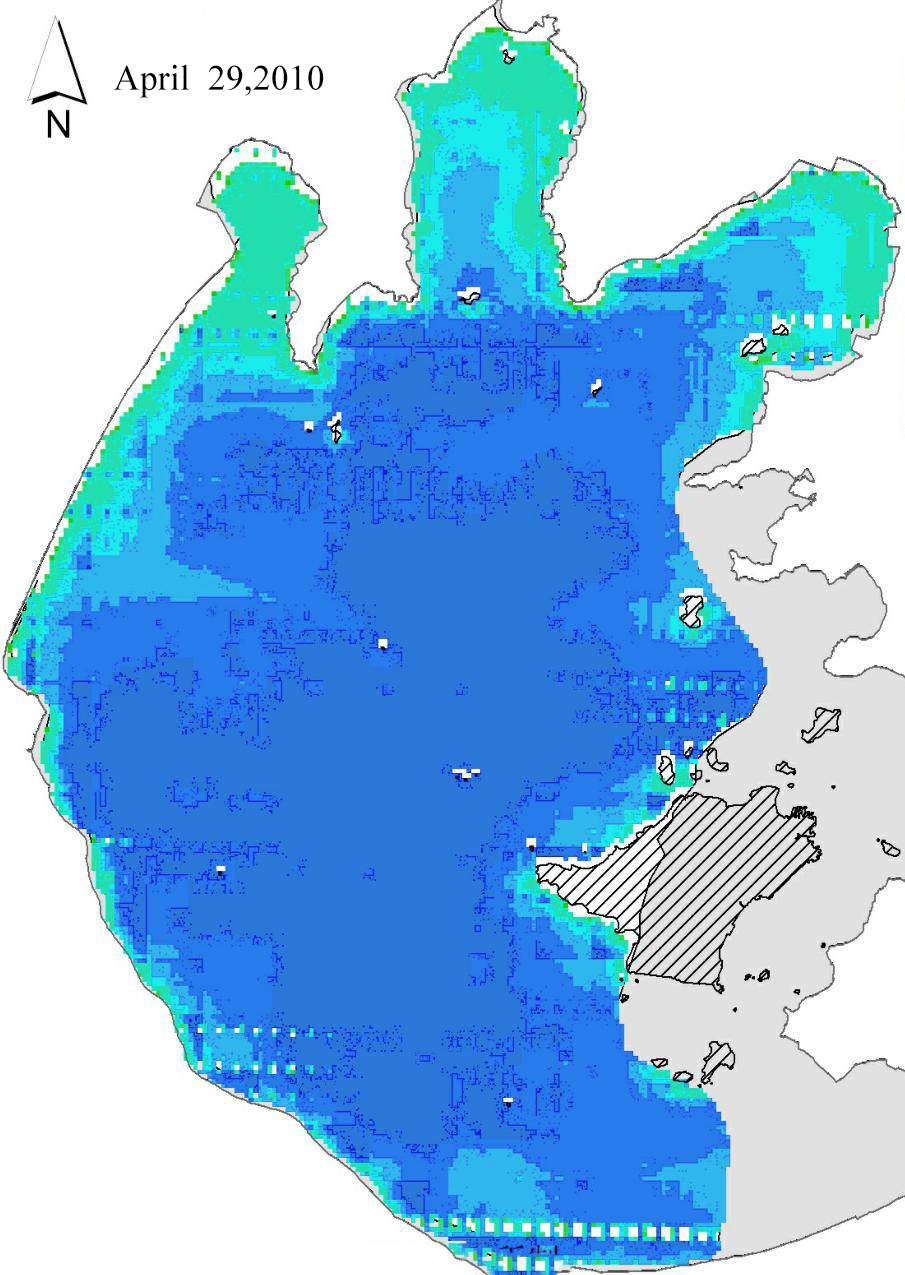

Supplement: Supplemental Information 9 — The data are remote sensing images of chlorophyll a concentration after data scale unification, remote sensing image repair, and time series filling. Remote sensing images of 30 consecutive moments were used as input to the 3D-GAN model. [file peerj-cs-09-1292-s009.zip › 201004290245.jpg]

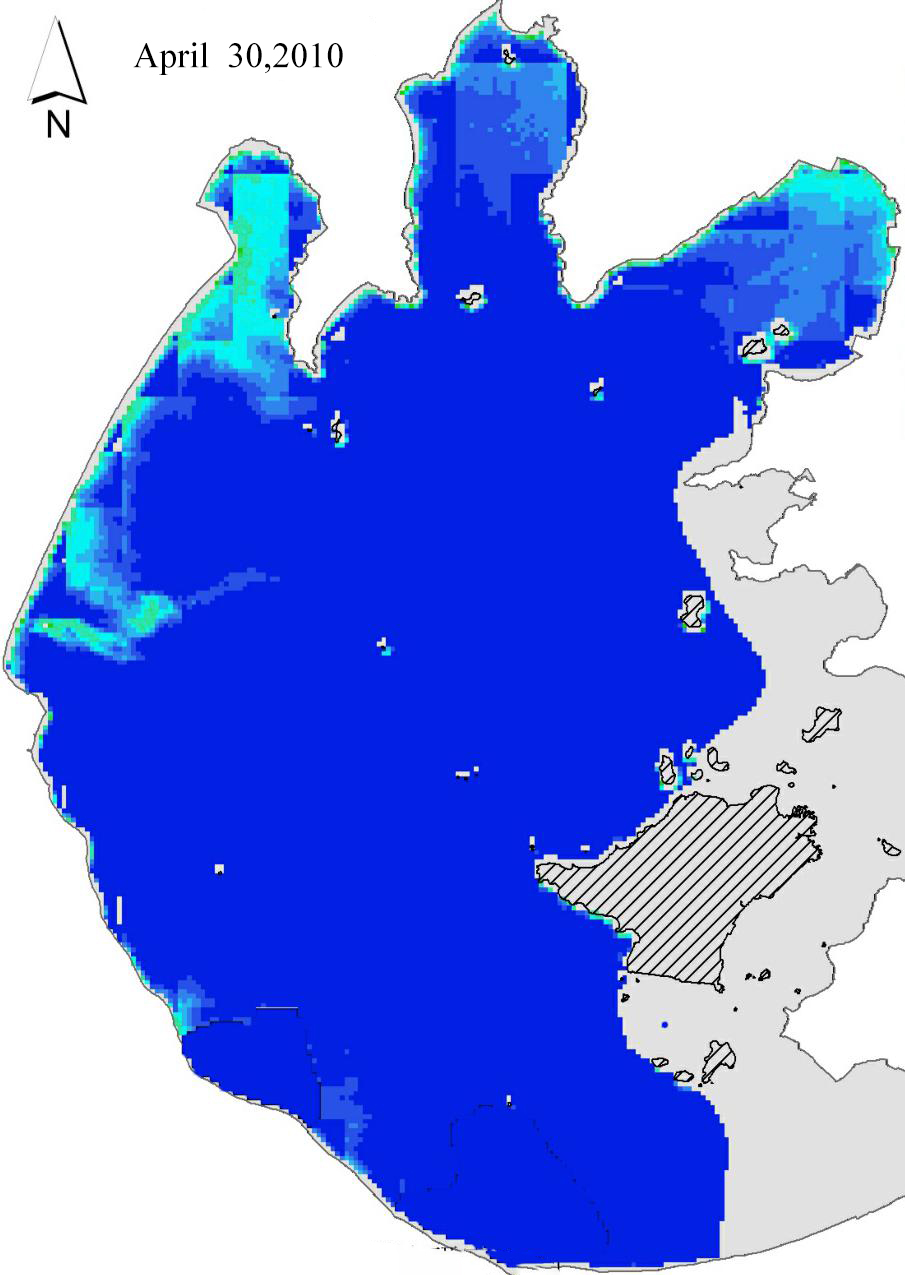

Supplement: Supplemental Information 9 — The data are remote sensing images of chlorophyll a concentration after data scale unification, remote sensing image repair, and time series filling. Remote sensing images of 30 consecutive moments were used as input to the 3D-GAN model. [file peerj-cs-09-1292-s009.zip › 201004300245.jpg]

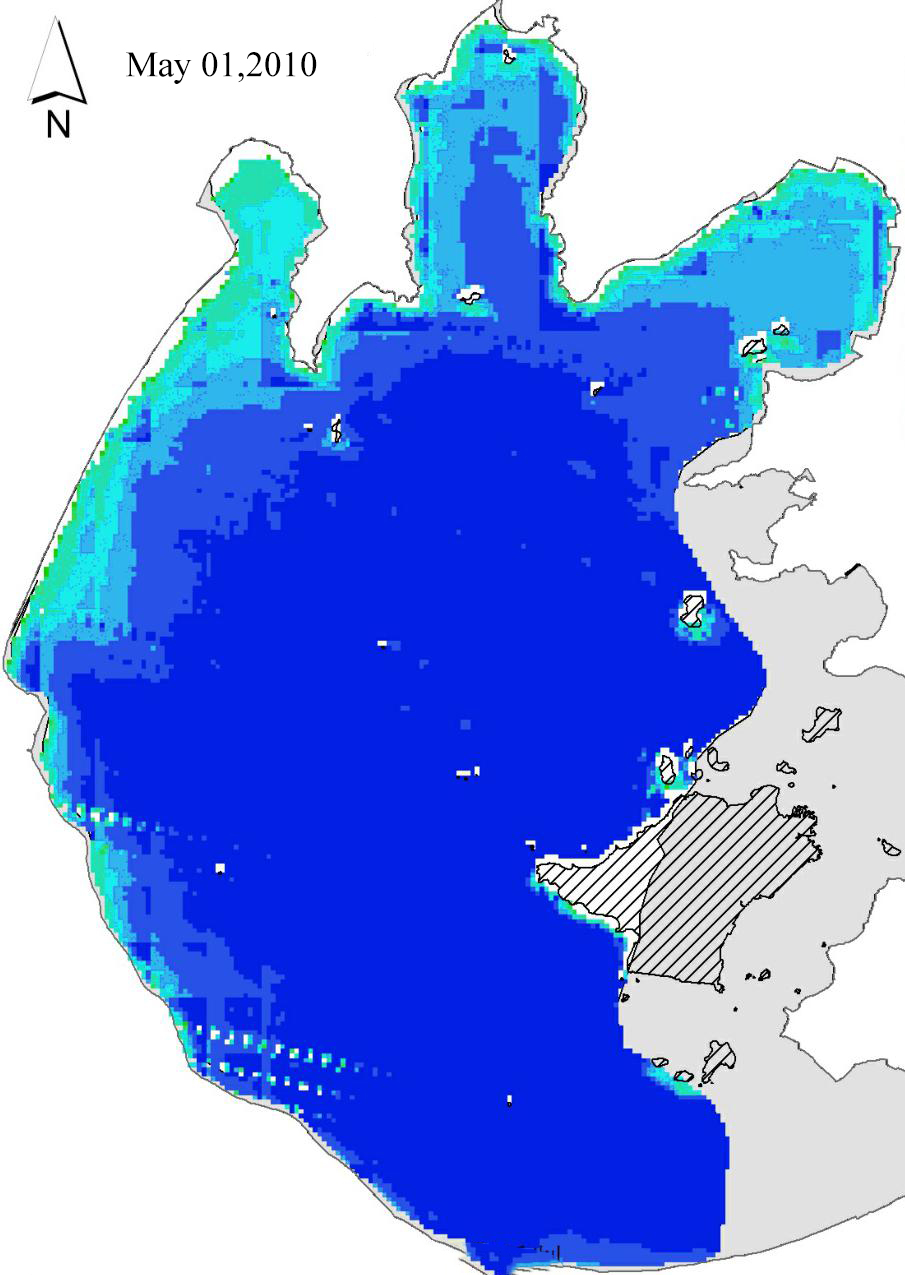

Supplement: Supplemental Information 9 — The data are remote sensing images of chlorophyll a concentration after data scale unification, remote sensing image repair, and time series filling. Remote sensing images of 30 consecutive moments were used as input to the 3D-GAN model. [file peerj-cs-09-1292-s009.zip › 201005010245.jpg]

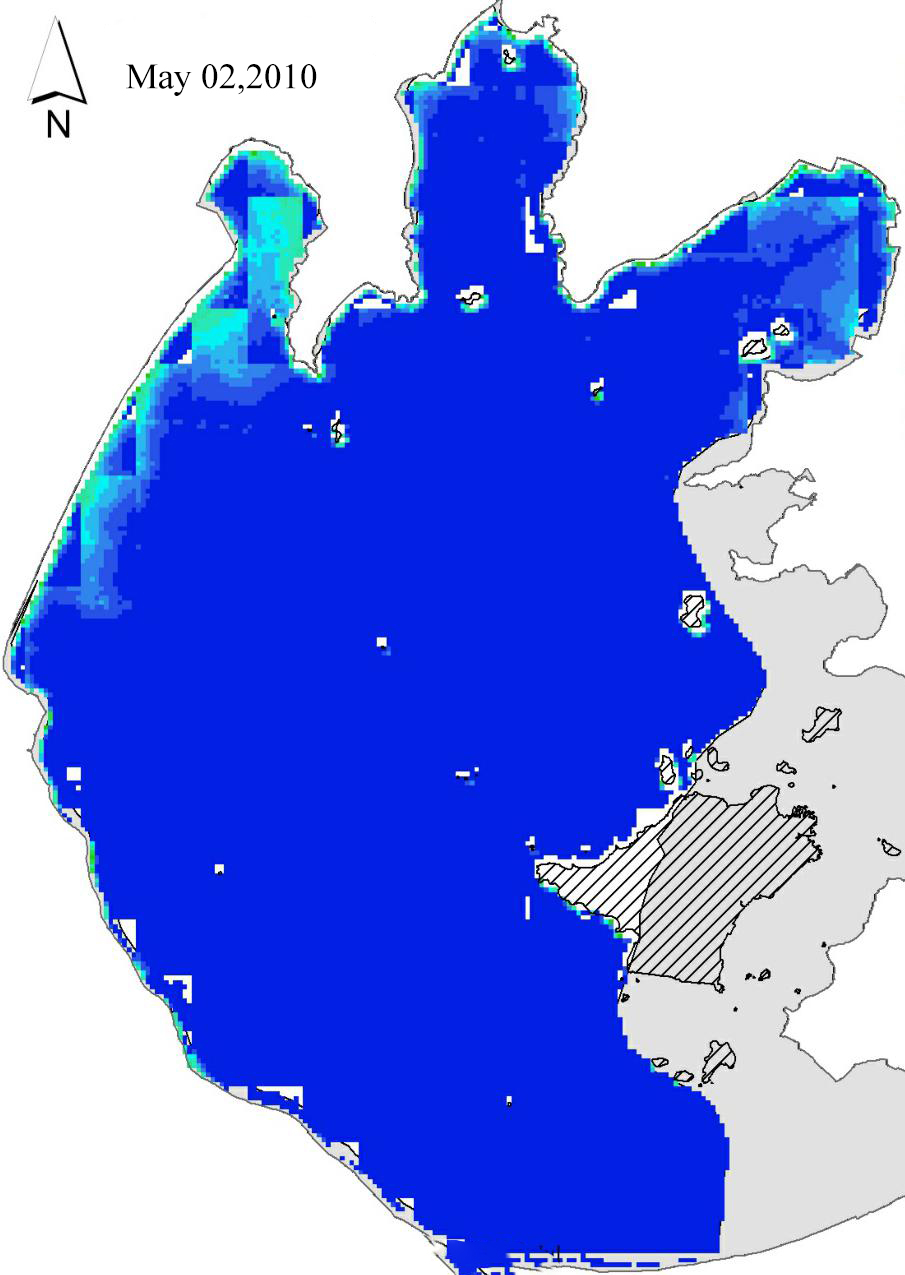

Supplement: Supplemental Information 9 — The data are remote sensing images of chlorophyll a concentration after data scale unification, remote sensing image repair, and time series filling. Remote sensing images of 30 consecutive moments were used as input to the 3D-GAN model. [file peerj-cs-09-1292-s009.zip › 201005020245.jpg]

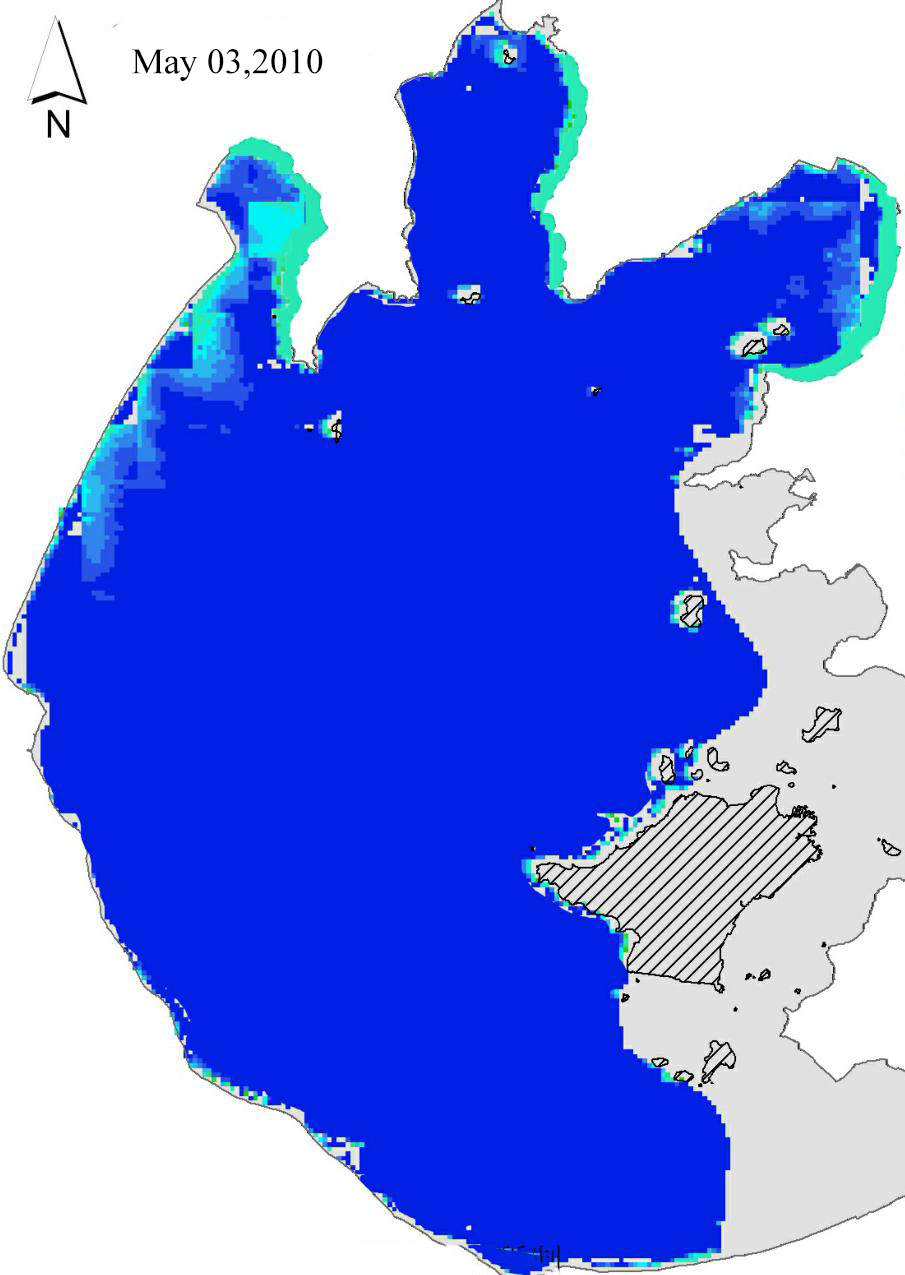

Supplement: Supplemental Information 9 — The data are remote sensing images of chlorophyll a concentration after data scale unification, remote sensing image repair, and time series filling. Remote sensing images of 30 consecutive moments were used as input to the 3D-GAN model. [file peerj-cs-09-1292-s009.zip › 201005030245.jpg]

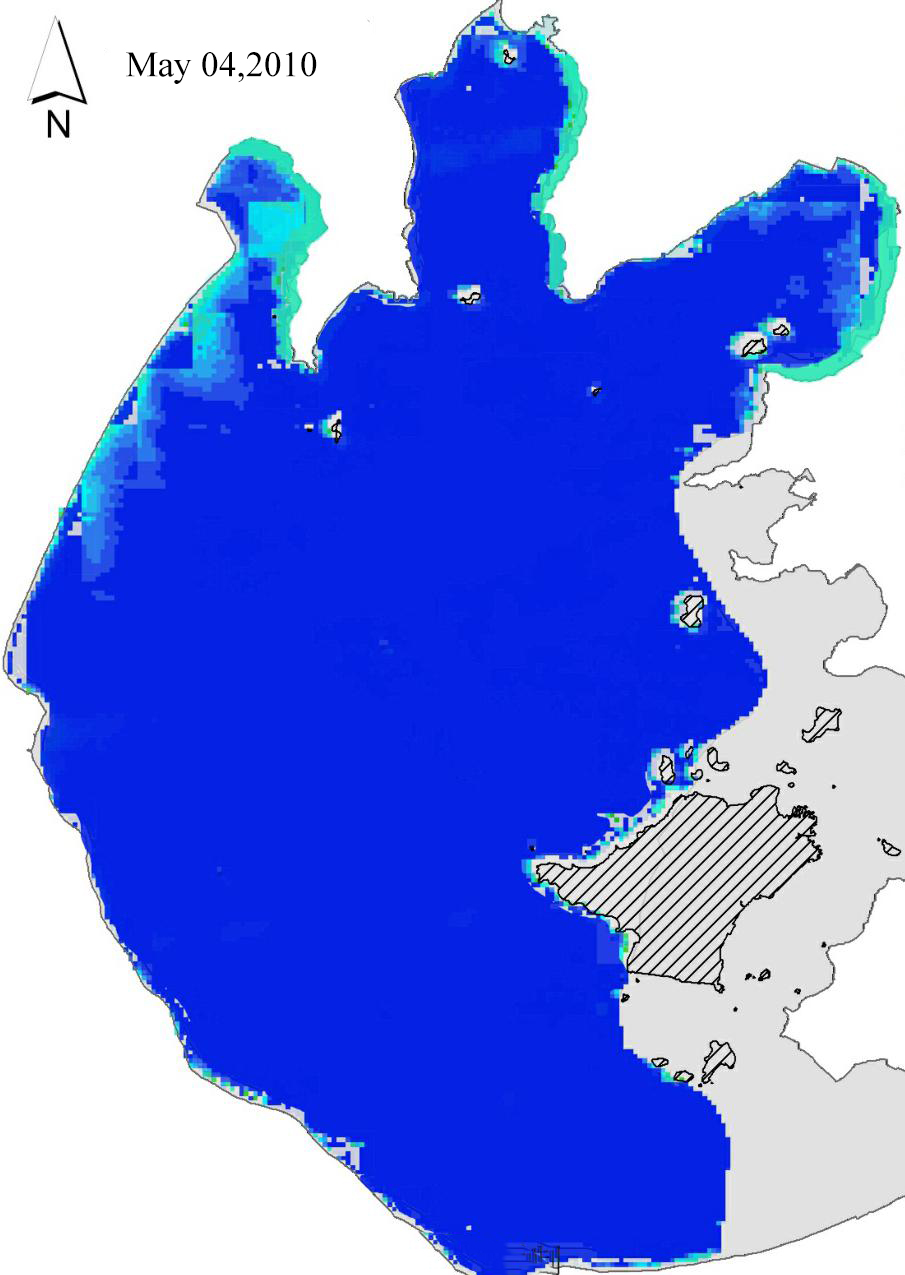

Supplement: Supplemental Information 9 — The data are remote sensing images of chlorophyll a concentration after data scale unification, remote sensing image repair, and time series filling. Remote sensing images of 30 consecutive moments were used as input to the 3D-GAN model. [file peerj-cs-09-1292-s009.zip › 201005040245.jpg]

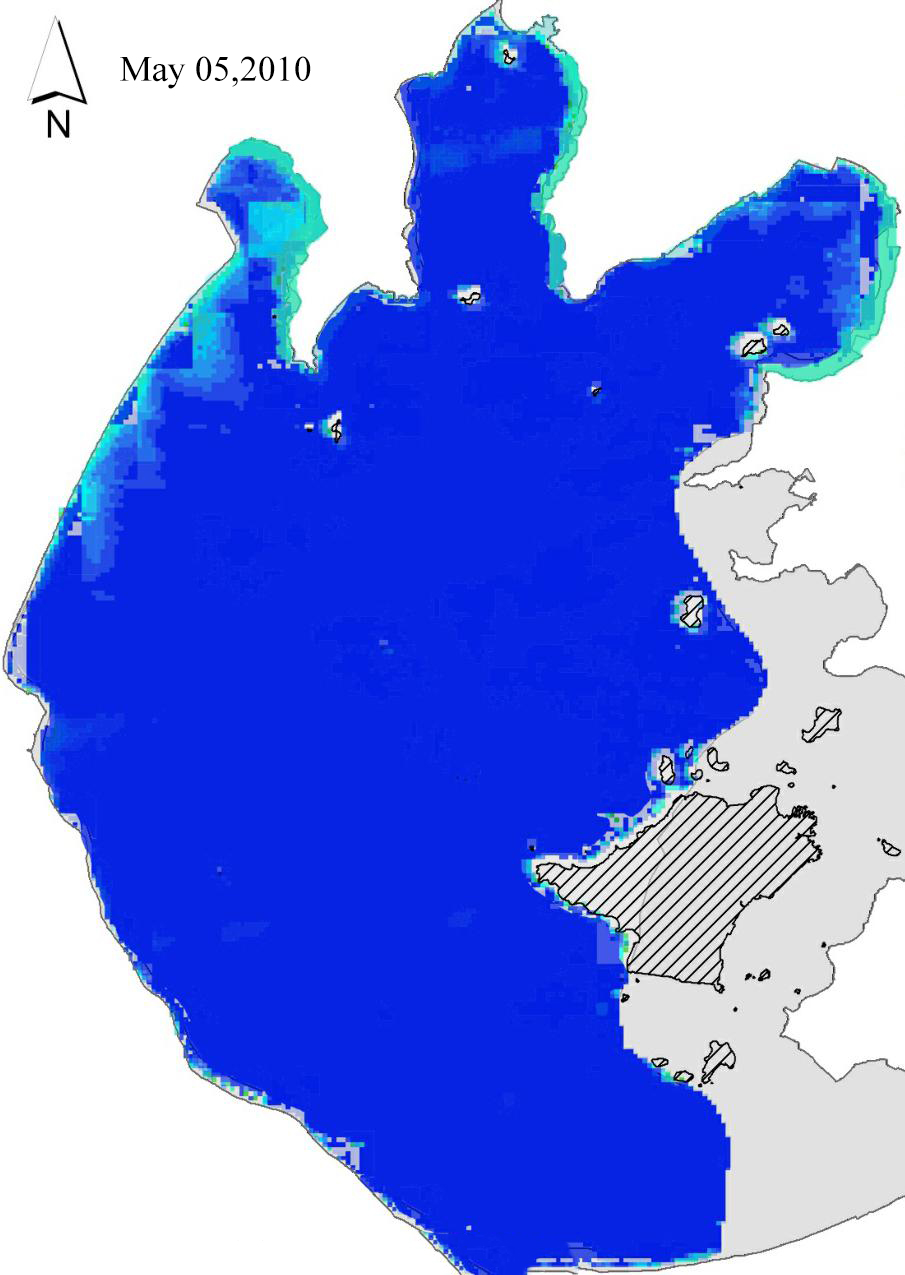

Supplement: Supplemental Information 9 — The data are remote sensing images of chlorophyll a concentration after data scale unification, remote sensing image repair, and time series filling. Remote sensing images of 30 consecutive moments were used as input to the 3D-GAN model. [file peerj-cs-09-1292-s009.zip › 201005050245.jpg]

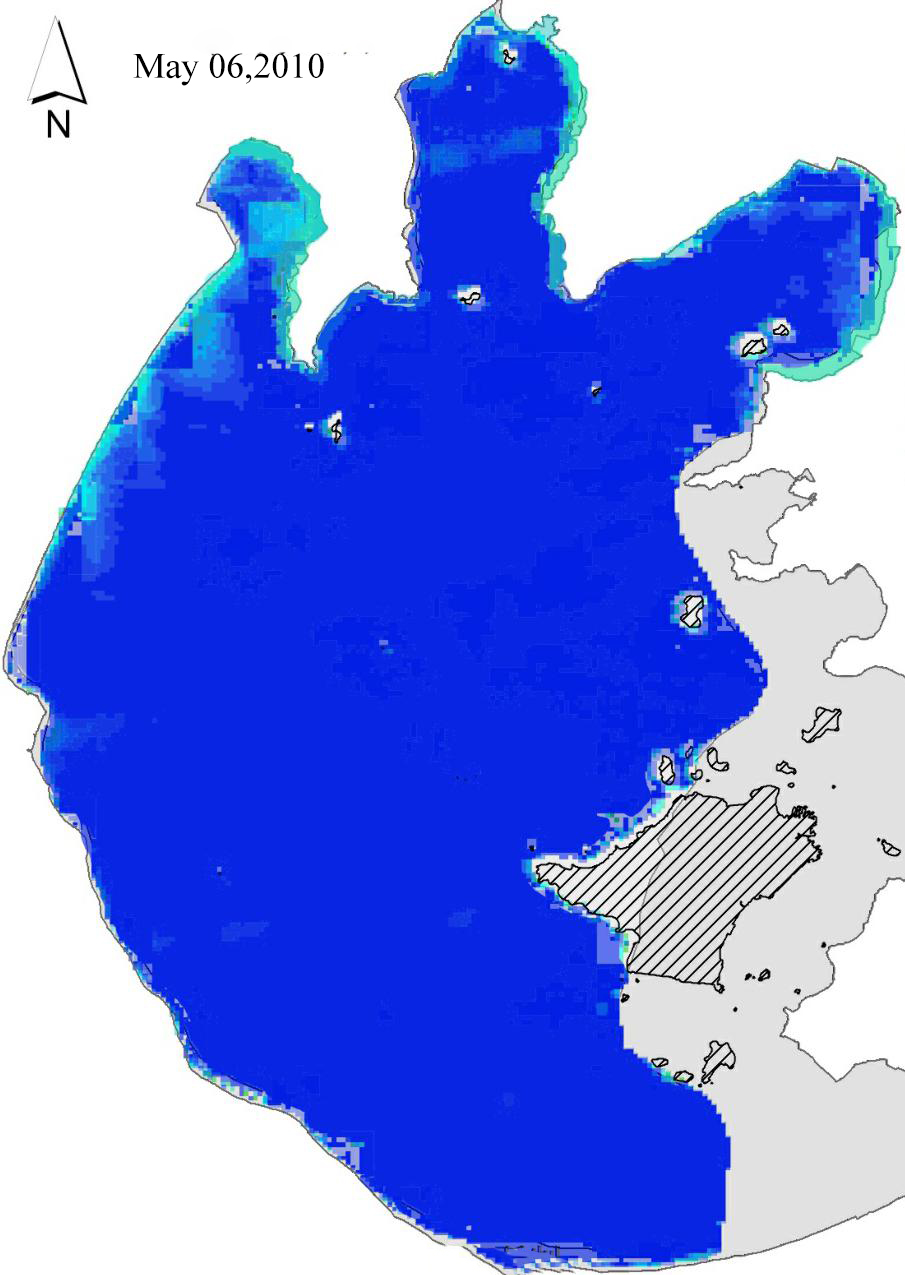

Supplement: Supplemental Information 9 — The data are remote sensing images of chlorophyll a concentration after data scale unification, remote sensing image repair, and time series filling. Remote sensing images of 30 consecutive moments were used as input to the 3D-GAN model. [file peerj-cs-09-1292-s009.zip › 201005060245.jpg]

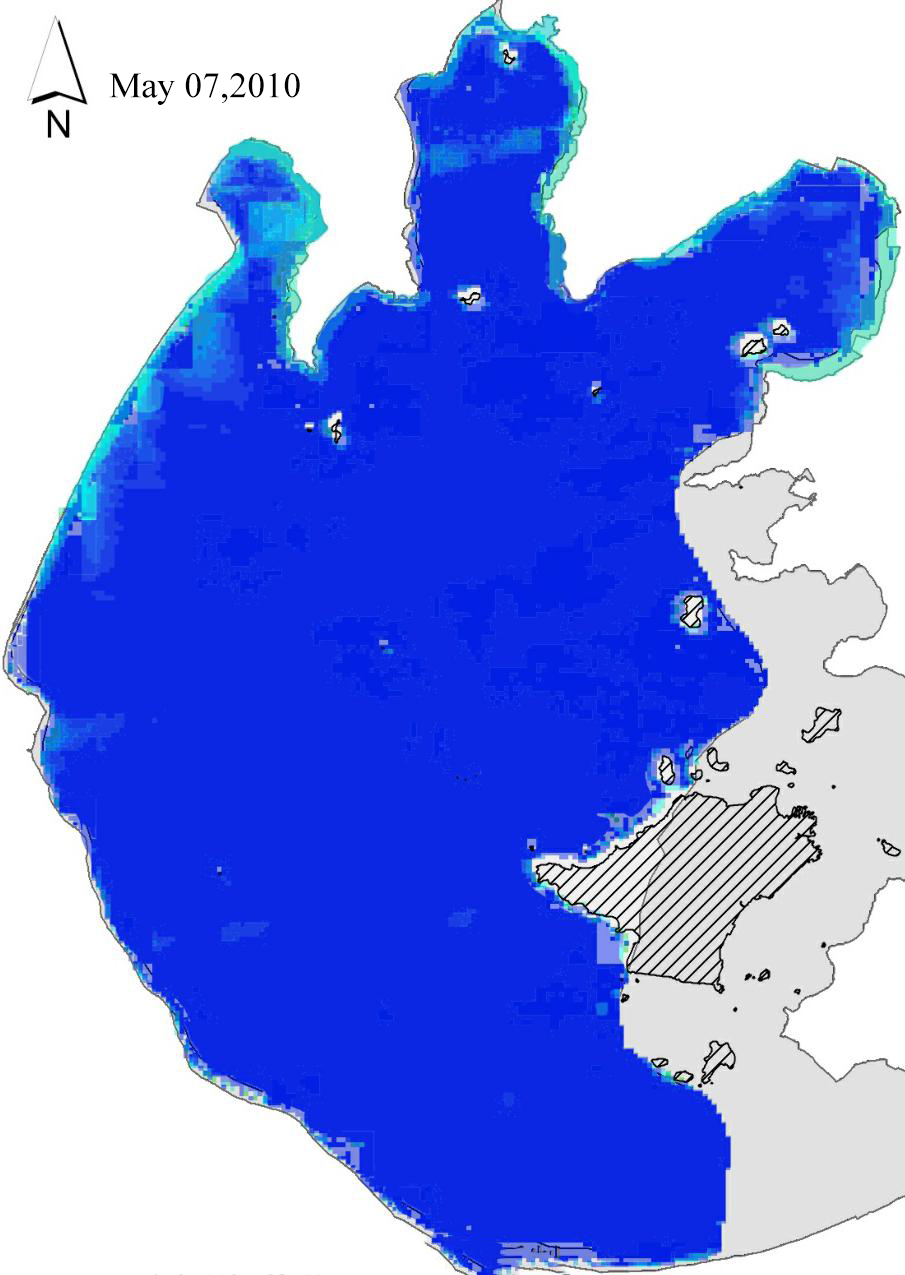

Supplement: Supplemental Information 9 — The data are remote sensing images of chlorophyll a concentration after data scale unification, remote sensing image repair, and time series filling. Remote sensing images of 30 consecutive moments were used as input to the 3D-GAN model. [file peerj-cs-09-1292-s009.zip › 201005070245.jpg]

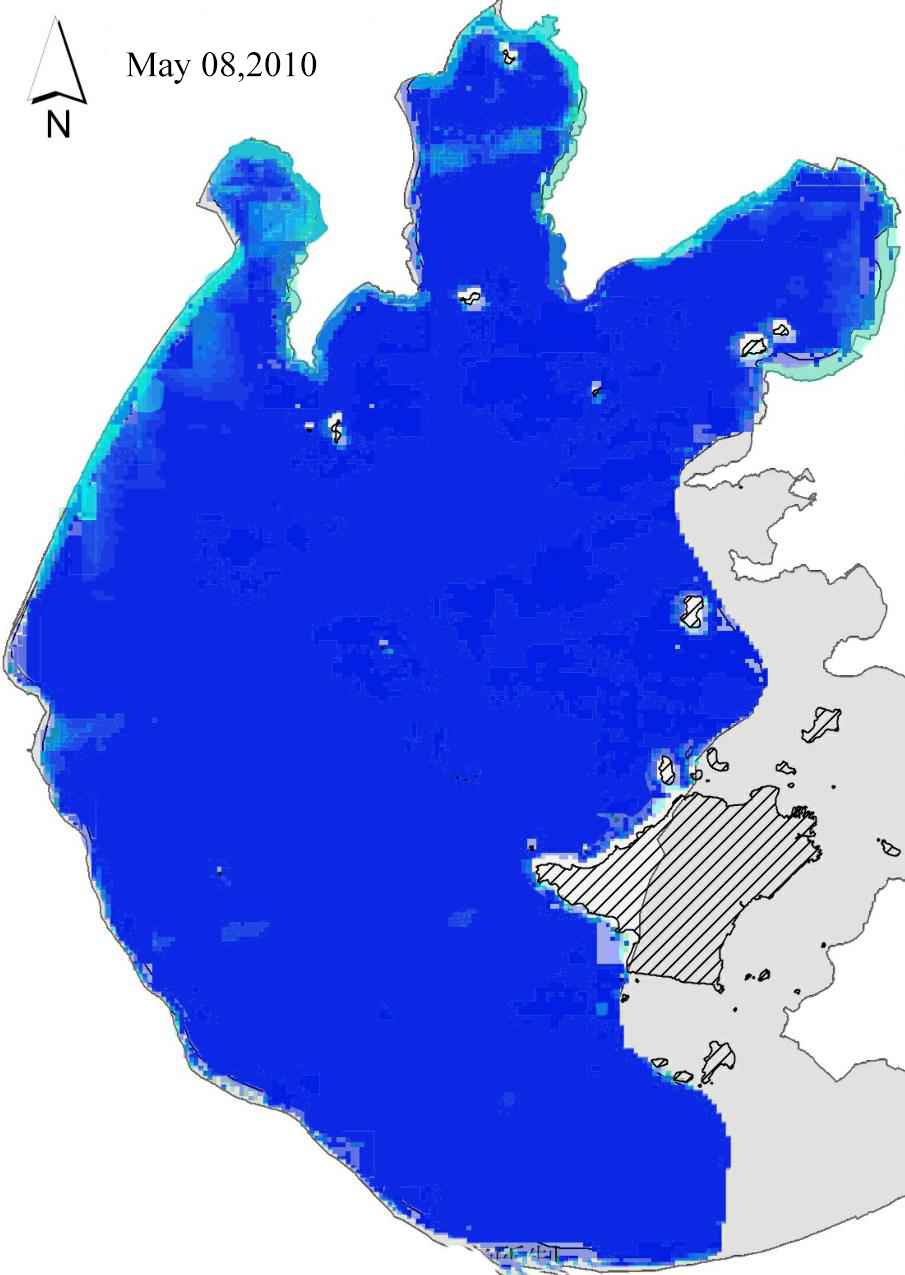

Supplement: Supplemental Information 9 — The data are remote sensing images of chlorophyll a concentration after data scale unification, remote sensing image repair, and time series filling. Remote sensing images of 30 consecutive moments were used as input to the 3D-GAN model. [file peerj-cs-09-1292-s009.zip › 201005080245.jpg]

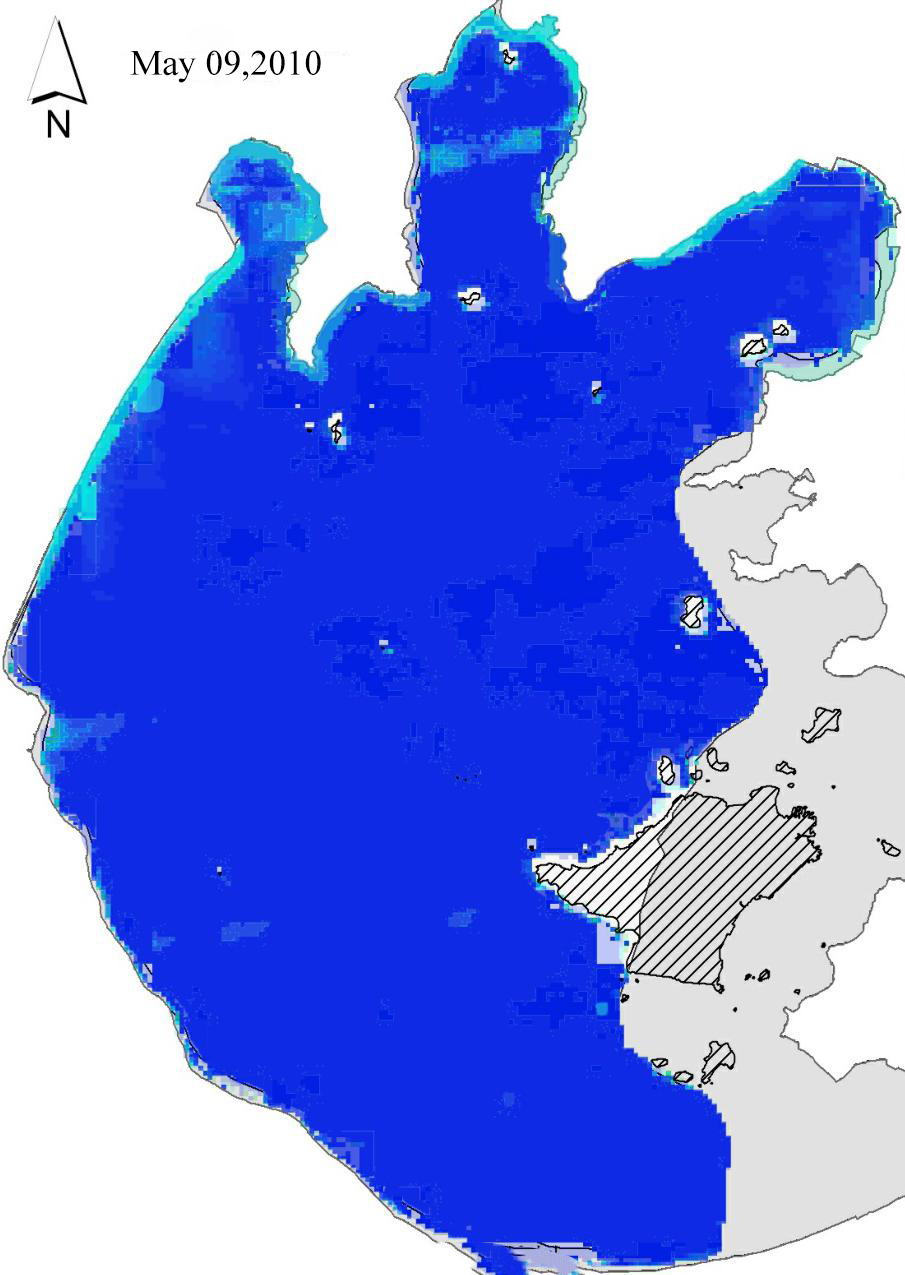

Supplement: Supplemental Information 9 — The data are remote sensing images of chlorophyll a concentration after data scale unification, remote sensing image repair, and time series filling. Remote sensing images of 30 consecutive moments were used as input to the 3D-GAN model. [file peerj-cs-09-1292-s009.zip › 201005090245.jpg]

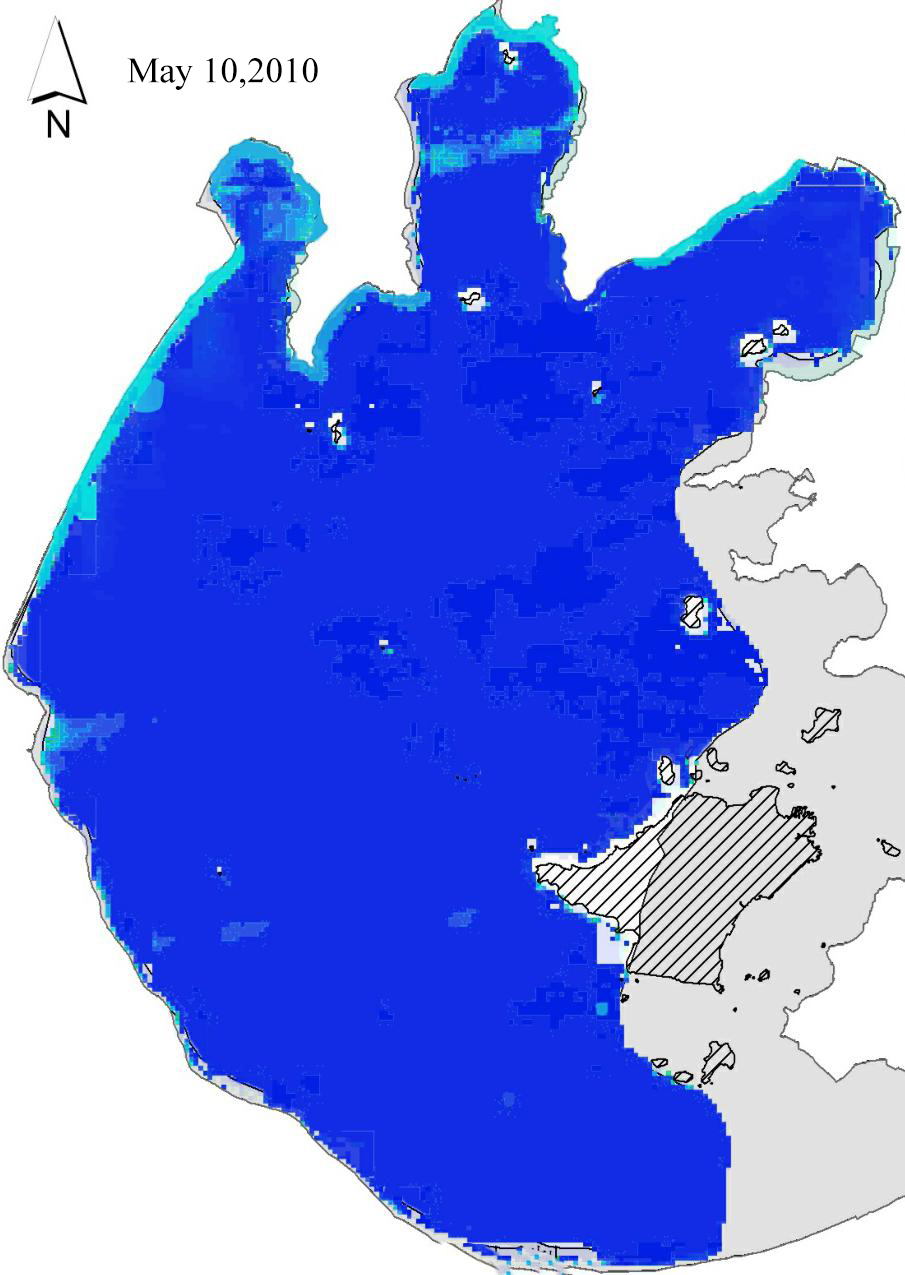

Supplement: Supplemental Information 9 — The data are remote sensing images of chlorophyll a concentration after data scale unification, remote sensing image repair, and time series filling. Remote sensing images of 30 consecutive moments were used as input to the 3D-GAN model. [file peerj-cs-09-1292-s009.zip › 201005100245.jpg]

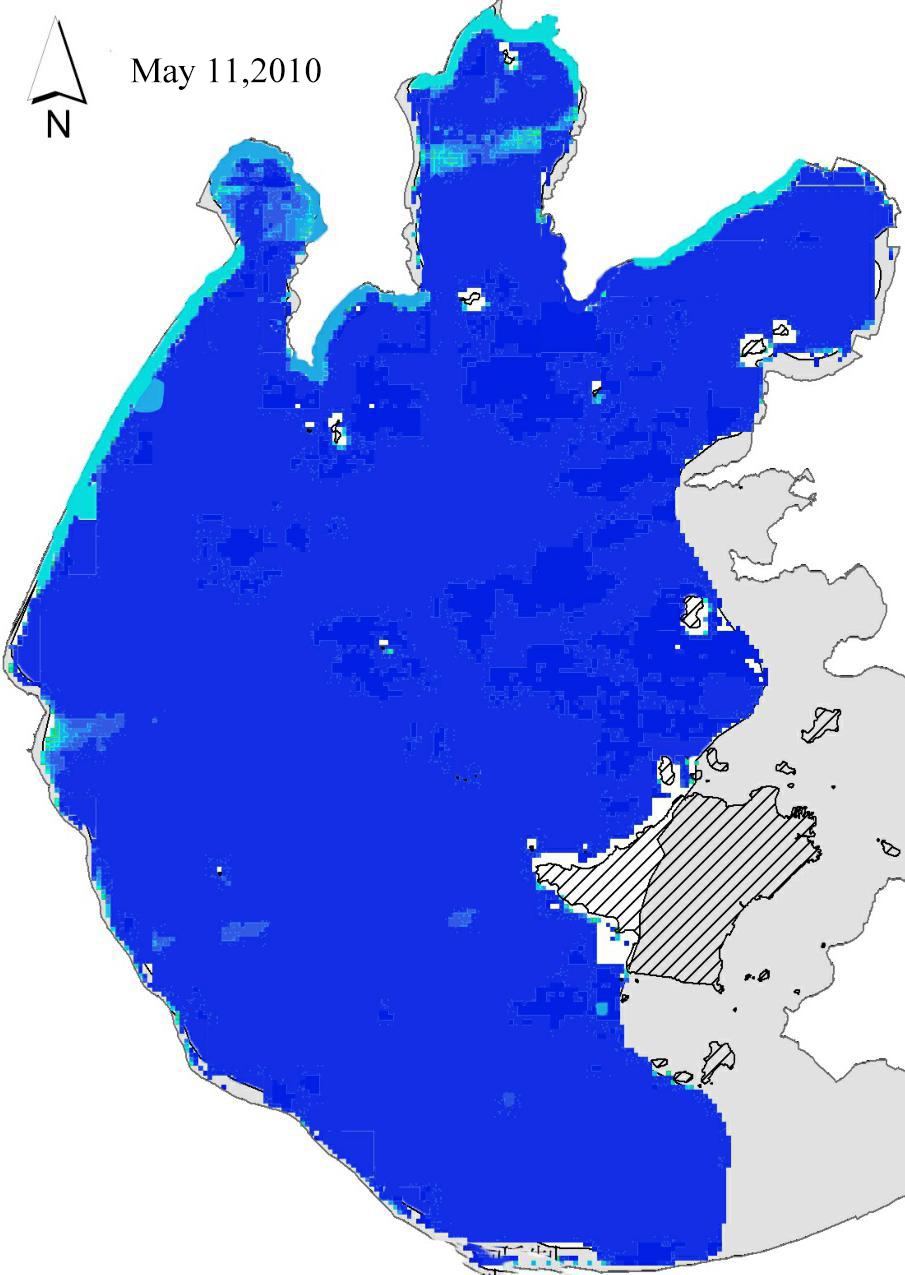

Supplement: Supplemental Information 9 — The data are remote sensing images of chlorophyll a concentration after data scale unification, remote sensing image repair, and time series filling. Remote sensing images of 30 consecutive moments were used as input to the 3D-GAN model. [file peerj-cs-09-1292-s009.zip › 201005110245.jpg]

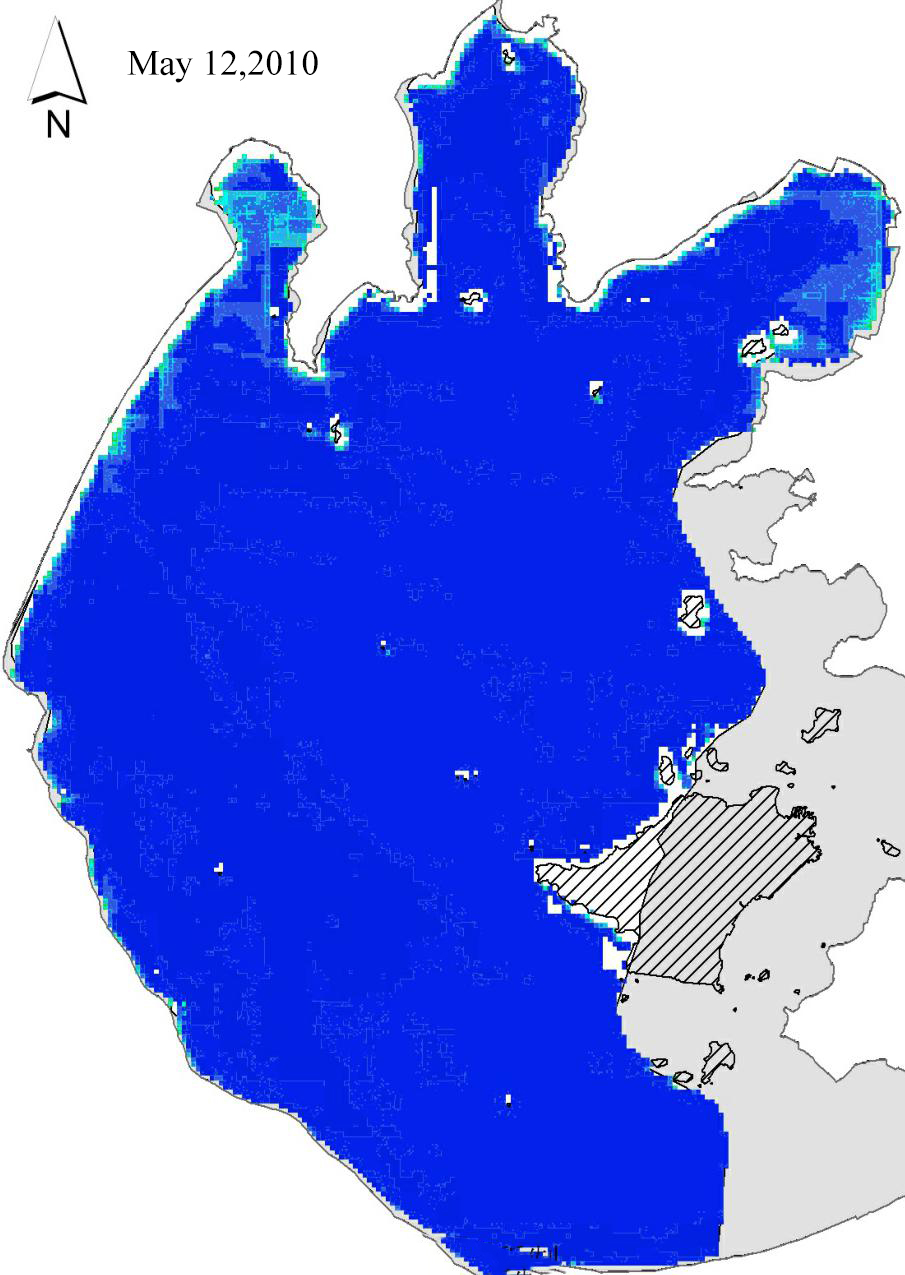

Supplement: Supplemental Information 9 — The data are remote sensing images of chlorophyll a concentration after data scale unification, remote sensing image repair, and time series filling. Remote sensing images of 30 consecutive moments were used as input to the 3D-GAN model. [file peerj-cs-09-1292-s009.zip › 201005120245.jpg]

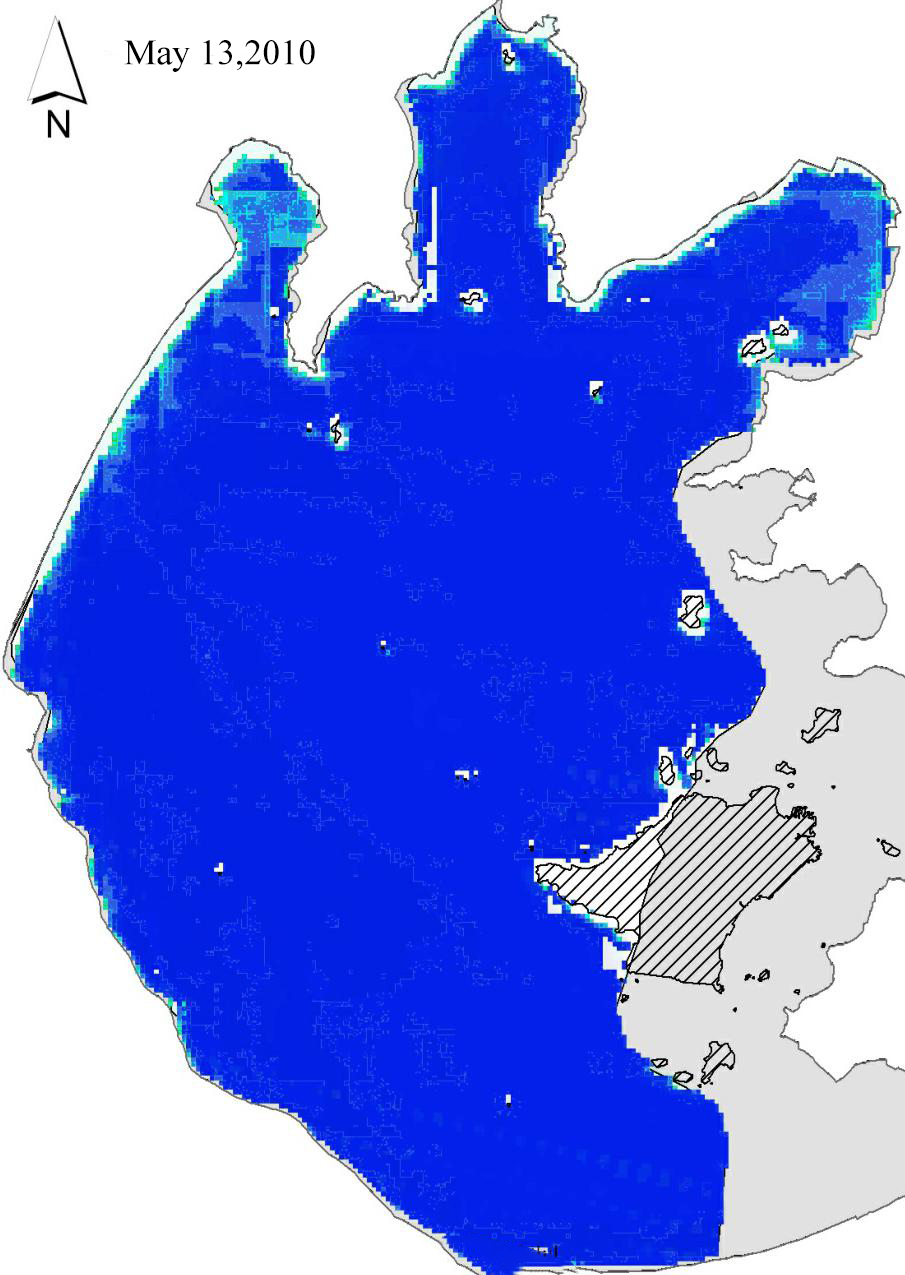

Supplement: Supplemental Information 9 — The data are remote sensing images of chlorophyll a concentration after data scale unification, remote sensing image repair, and time series filling. Remote sensing images of 30 consecutive moments were used as input to the 3D-GAN model. [file peerj-cs-09-1292-s009.zip › 201005130245.jpg]

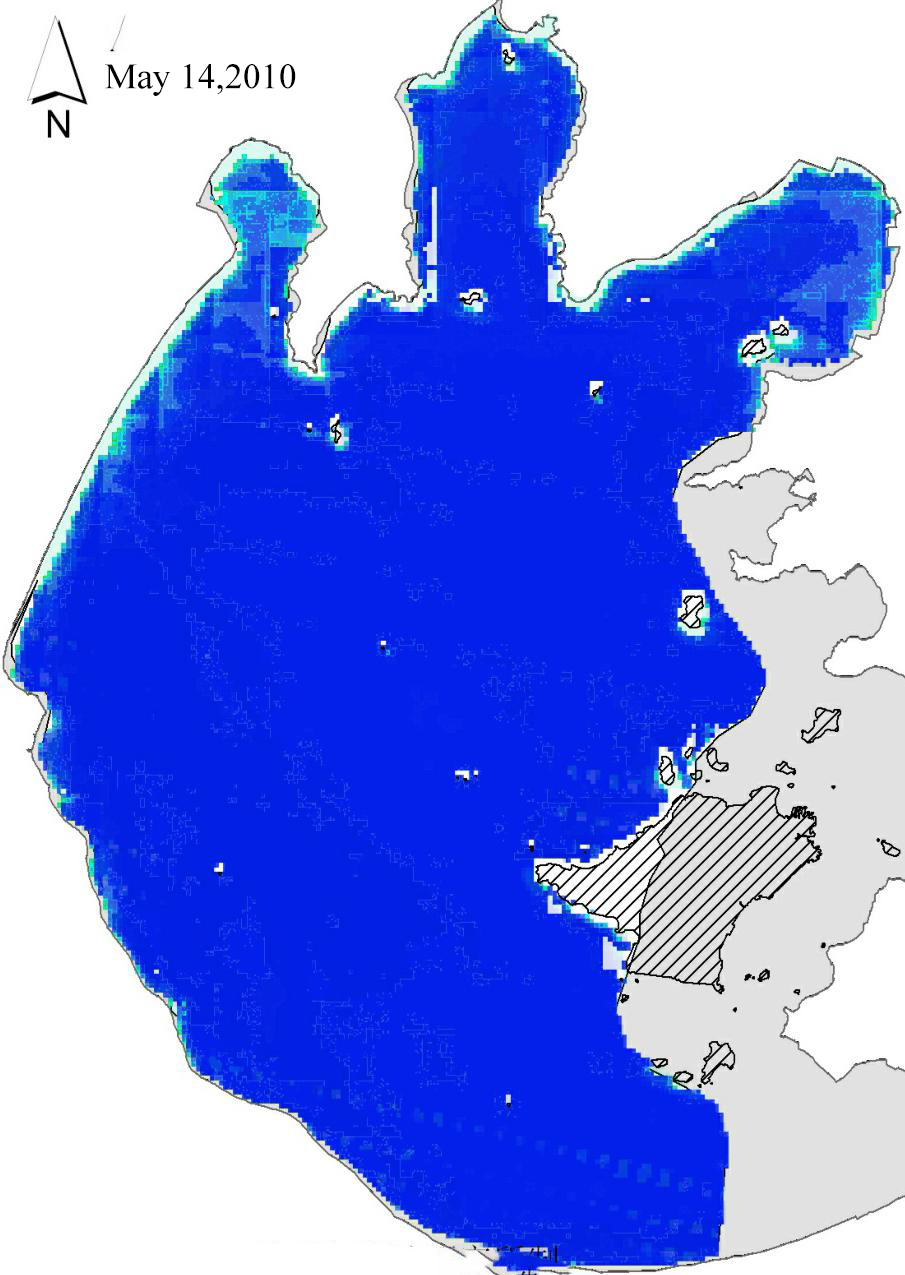

Supplement: Supplemental Information 9 — The data are remote sensing images of chlorophyll a concentration after data scale unification, remote sensing image repair, and time series filling. Remote sensing images of 30 consecutive moments were used as input to the 3D-GAN model. [file peerj-cs-09-1292-s009.zip › 201005140245.jpg]

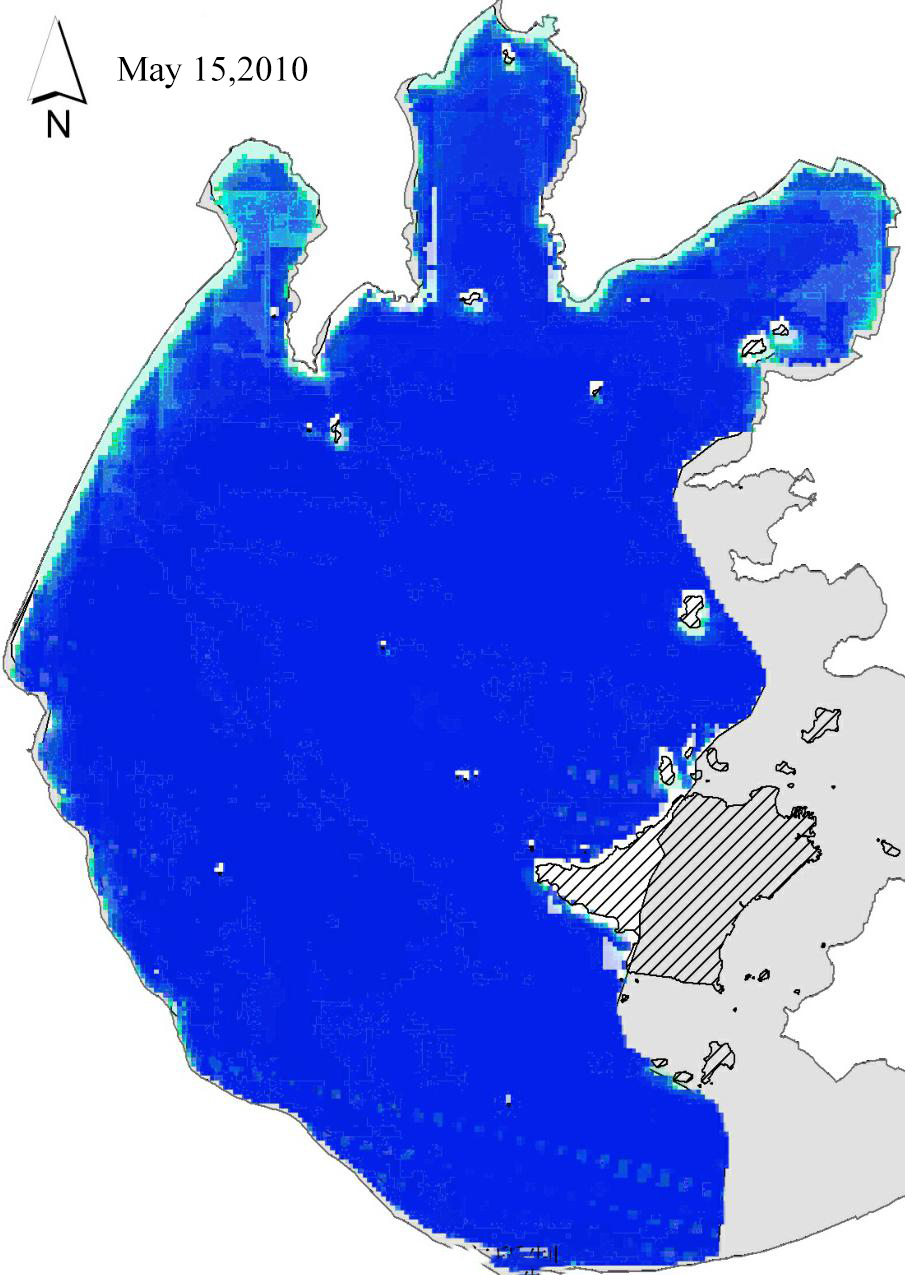

Supplement: Supplemental Information 9 — The data are remote sensing images of chlorophyll a concentration after data scale unification, remote sensing image repair, and time series filling. Remote sensing images of 30 consecutive moments were used as input to the 3D-GAN model. [file peerj-cs-09-1292-s009.zip › 201005150245.jpg]

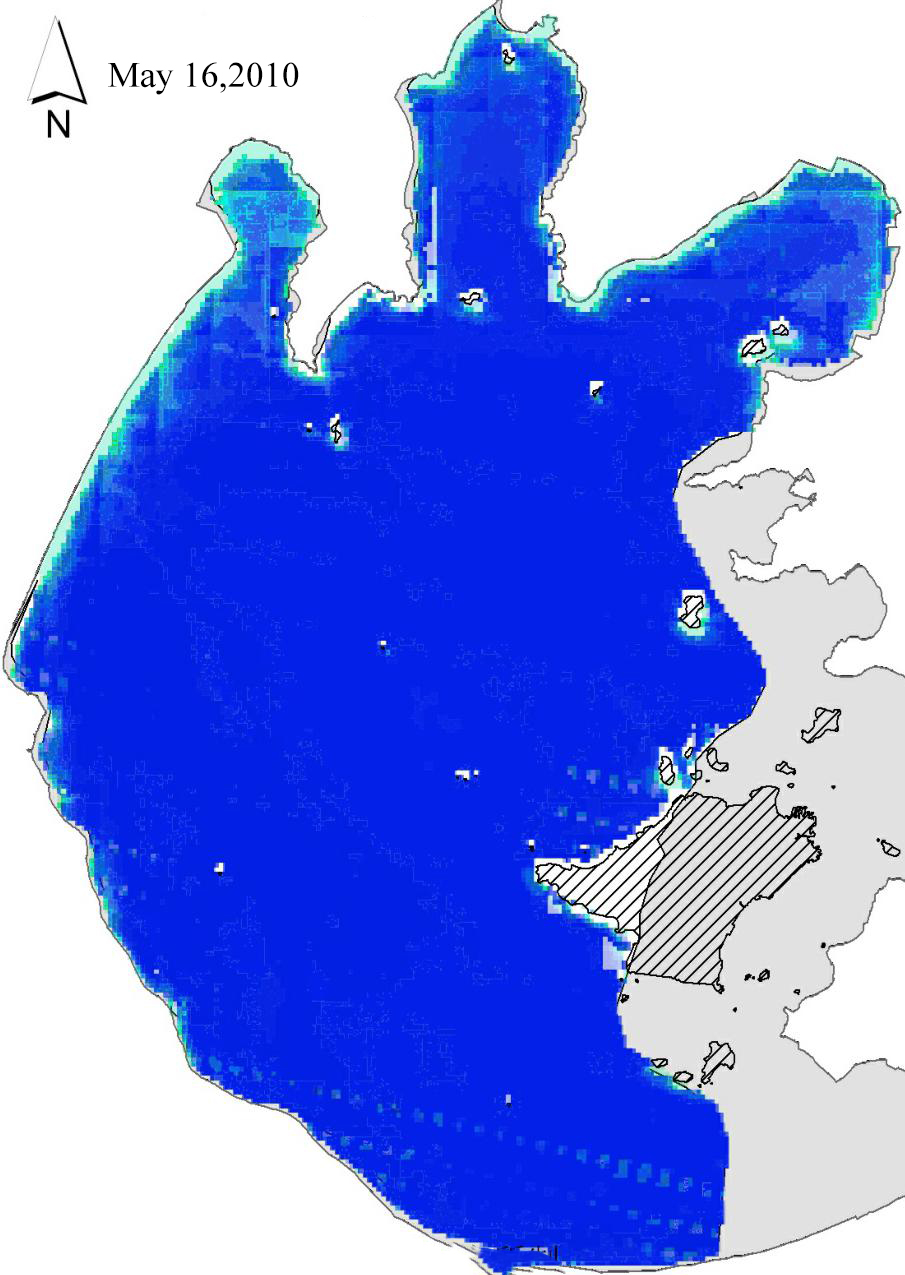

Supplement: Supplemental Information 9 — The data are remote sensing images of chlorophyll a concentration after data scale unification, remote sensing image repair, and time series filling. Remote sensing images of 30 consecutive moments were used as input to the 3D-GAN model. [file peerj-cs-09-1292-s009.zip › 201005160245.jpg]

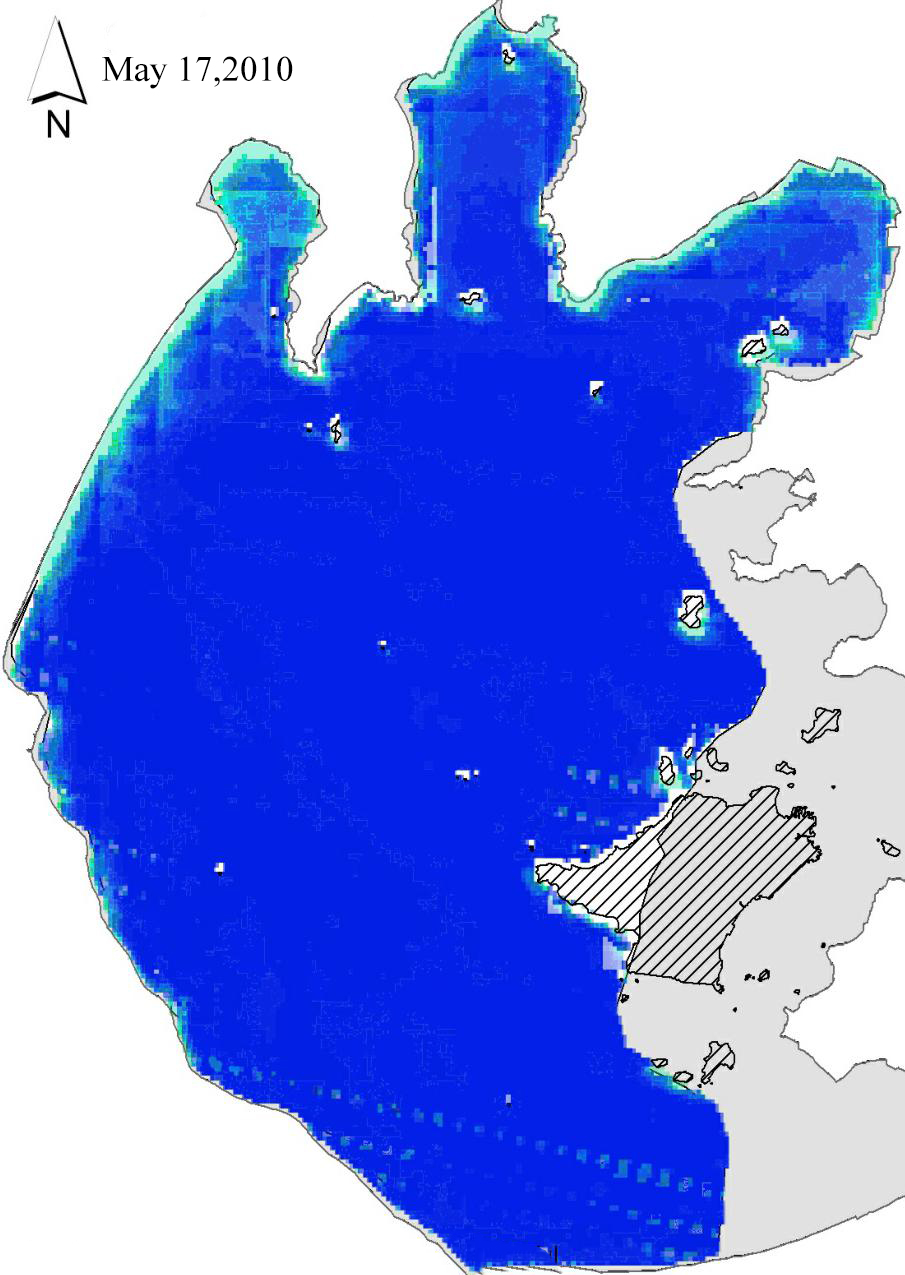

Supplement: Supplemental Information 9 — The data are remote sensing images of chlorophyll a concentration after data scale unification, remote sensing image repair, and time series filling. Remote sensing images of 30 consecutive moments were used as input to the 3D-GAN model. [file peerj-cs-09-1292-s009.zip › 201005170245.jpg]

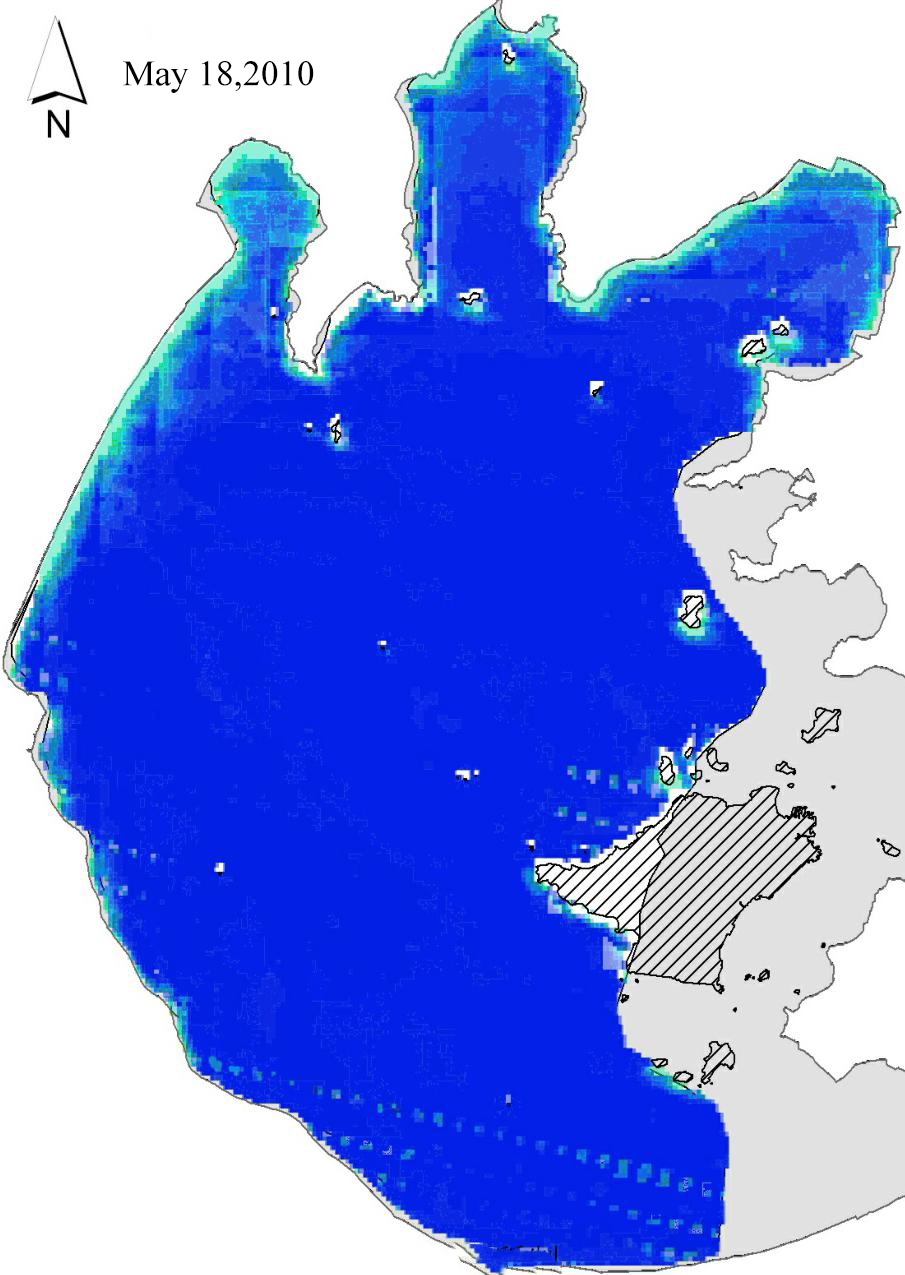

Supplement: Supplemental Information 9 — The data are remote sensing images of chlorophyll a concentration after data scale unification, remote sensing image repair, and time series filling. Remote sensing images of 30 consecutive moments were used as input to the 3D-GAN model. [file peerj-cs-09-1292-s009.zip › 201005180245.jpg]

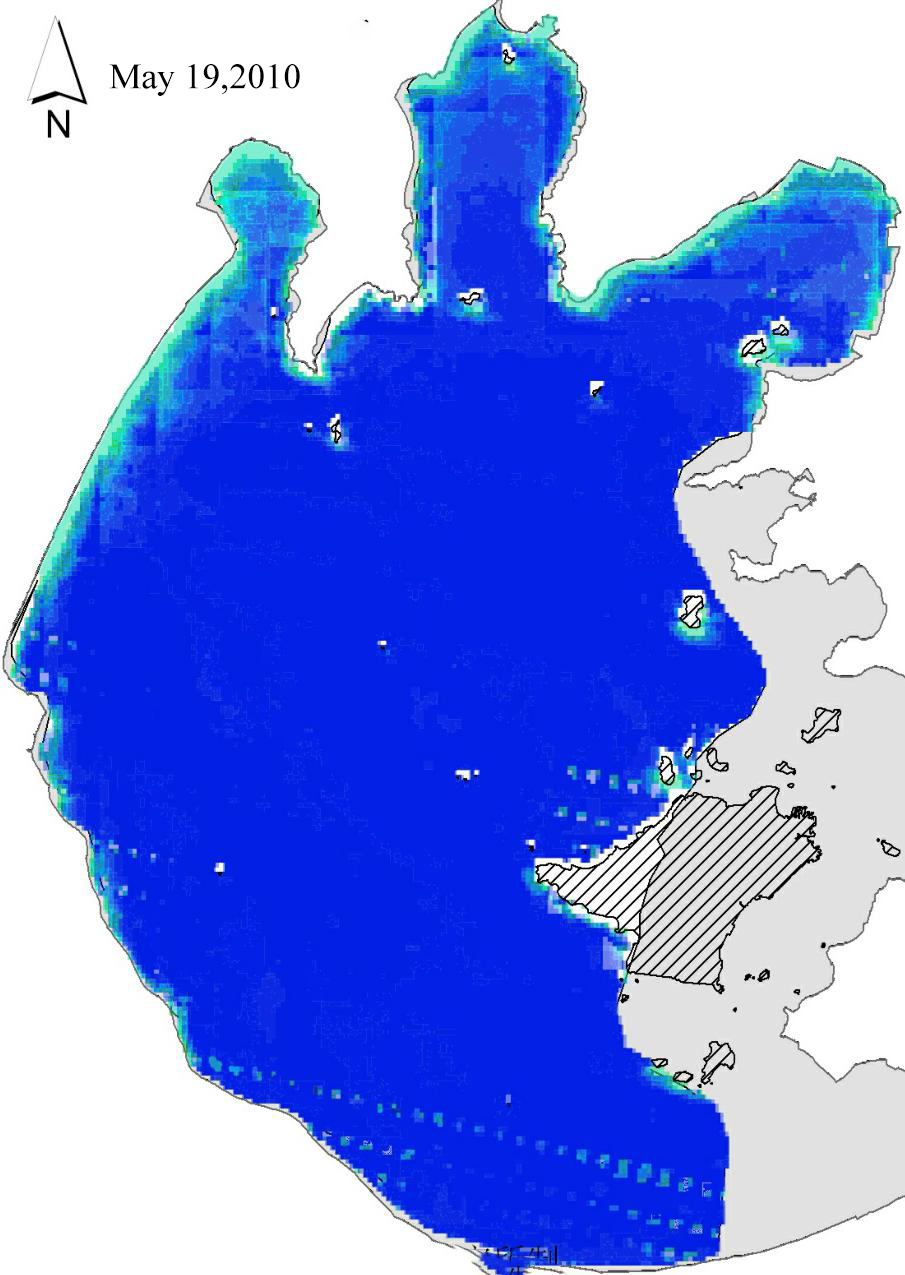

Supplement: Supplemental Information 9 — The data are remote sensing images of chlorophyll a concentration after data scale unification, remote sensing image repair, and time series filling. Remote sensing images of 30 consecutive moments were used as input to the 3D-GAN model. [file peerj-cs-09-1292-s009.zip › 201005190245.jpg]

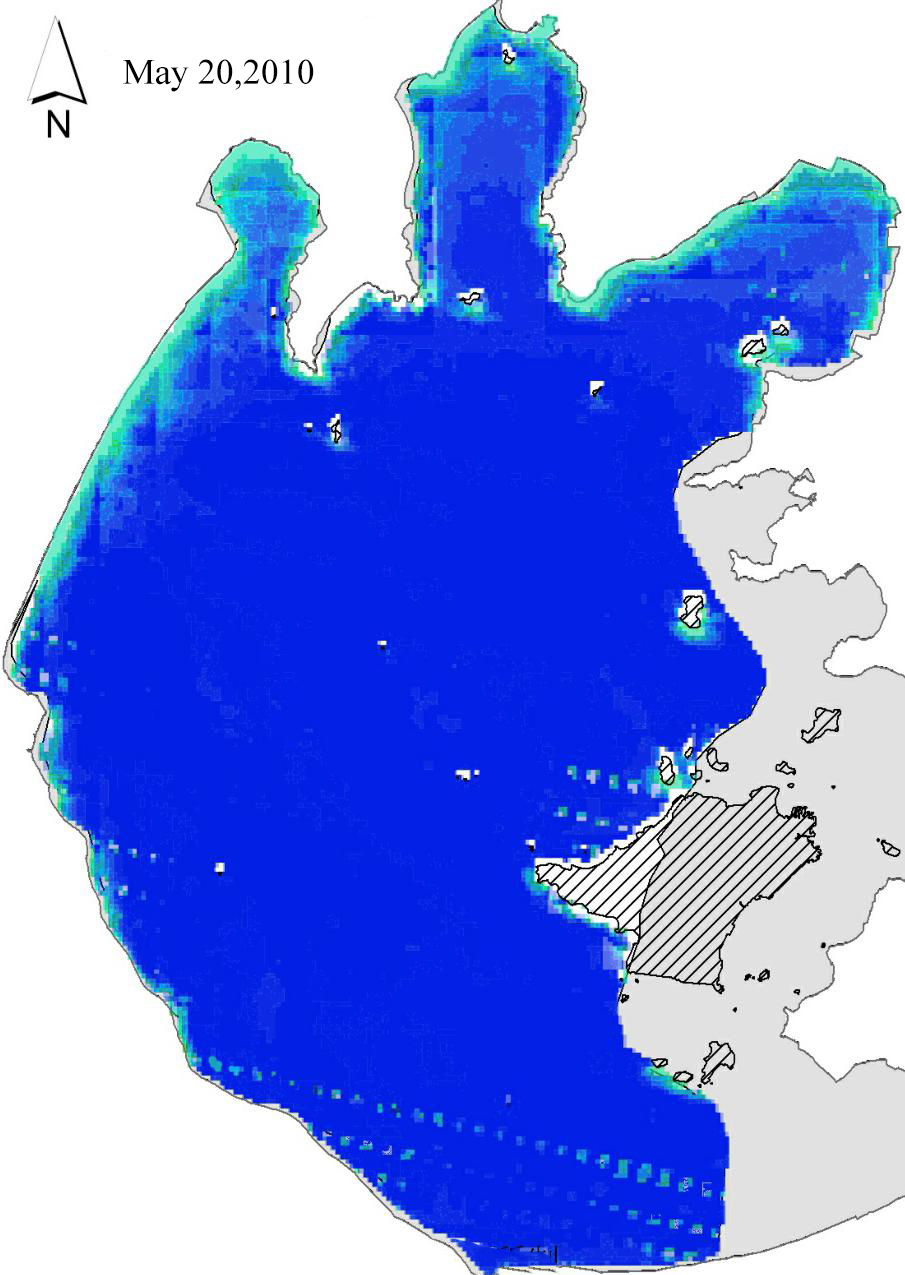

Supplement: Supplemental Information 9 — The data are remote sensing images of chlorophyll a concentration after data scale unification, remote sensing image repair, and time series filling. Remote sensing images of 30 consecutive moments were used as input to the 3D-GAN model. [file peerj-cs-09-1292-s009.zip › 201005200245.jpg]

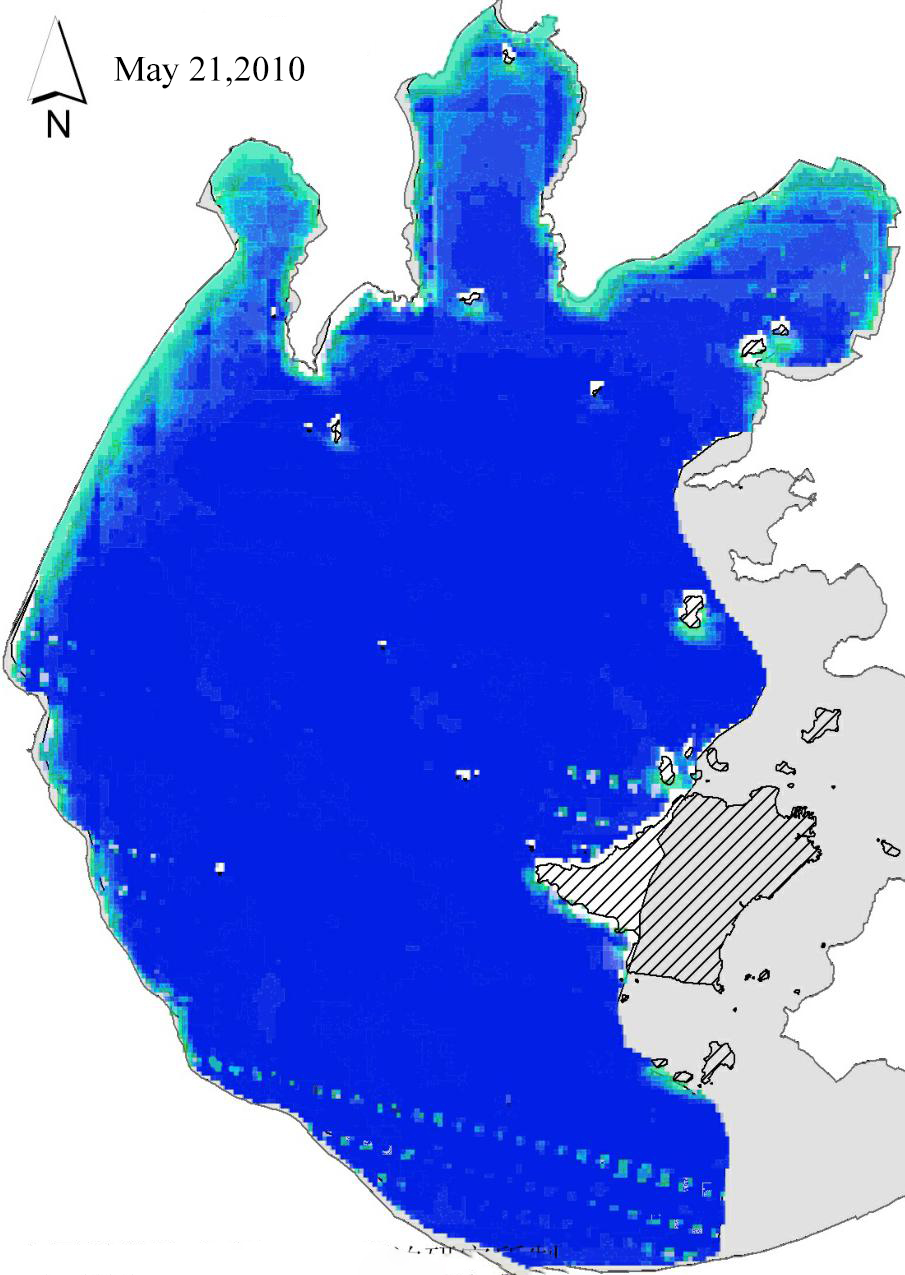

Supplement: Supplemental Information 9 — The data are remote sensing images of chlorophyll a concentration after data scale unification, remote sensing image repair, and time series filling. Remote sensing images of 30 consecutive moments were used as input to the 3D-GAN model. [file peerj-cs-09-1292-s009.zip › 201005210245.jpg]

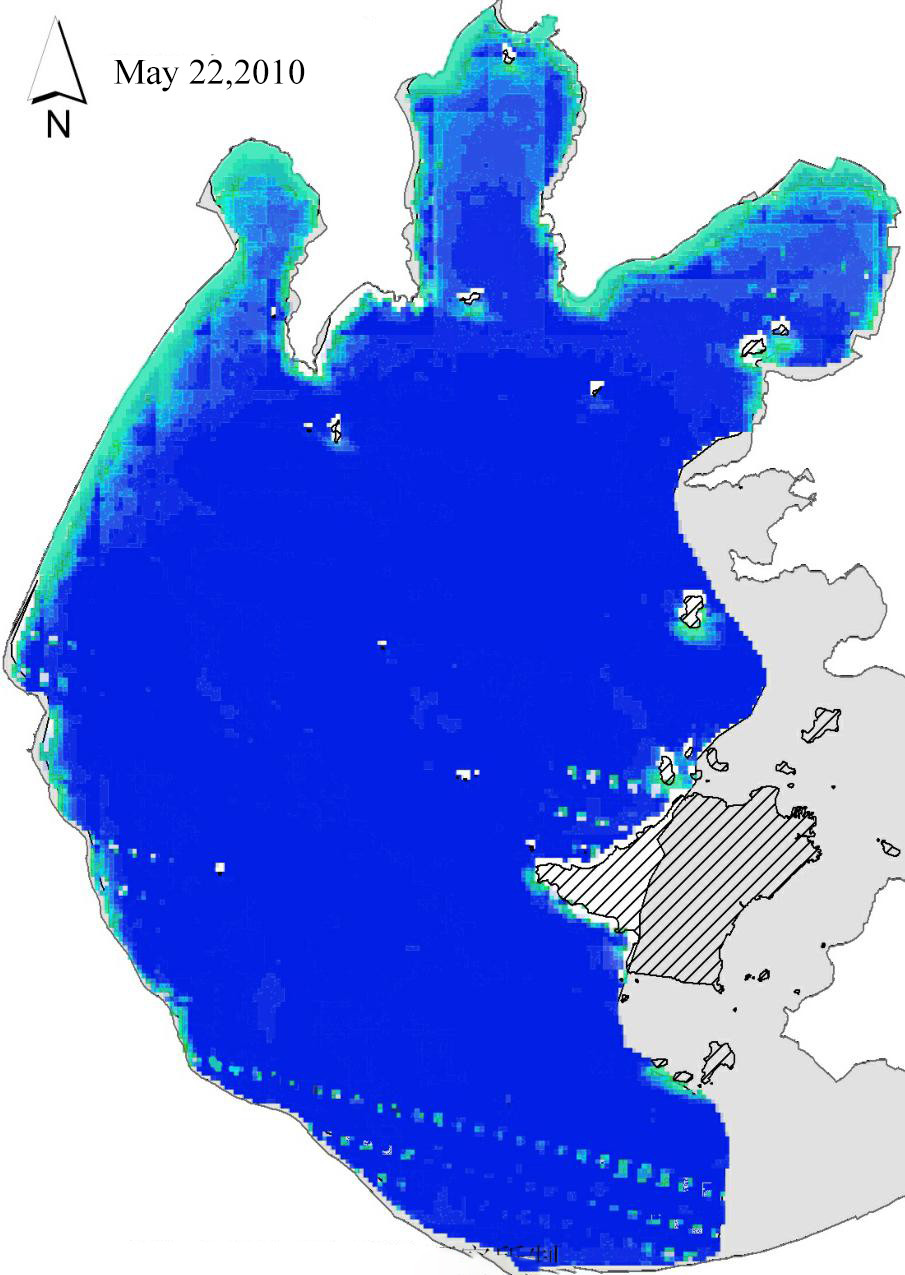

Supplement: Supplemental Information 9 — The data are remote sensing images of chlorophyll a concentration after data scale unification, remote sensing image repair, and time series filling. Remote sensing images of 30 consecutive moments were used as input to the 3D-GAN model. [file peerj-cs-09-1292-s009.zip › 201005220245.jpg]

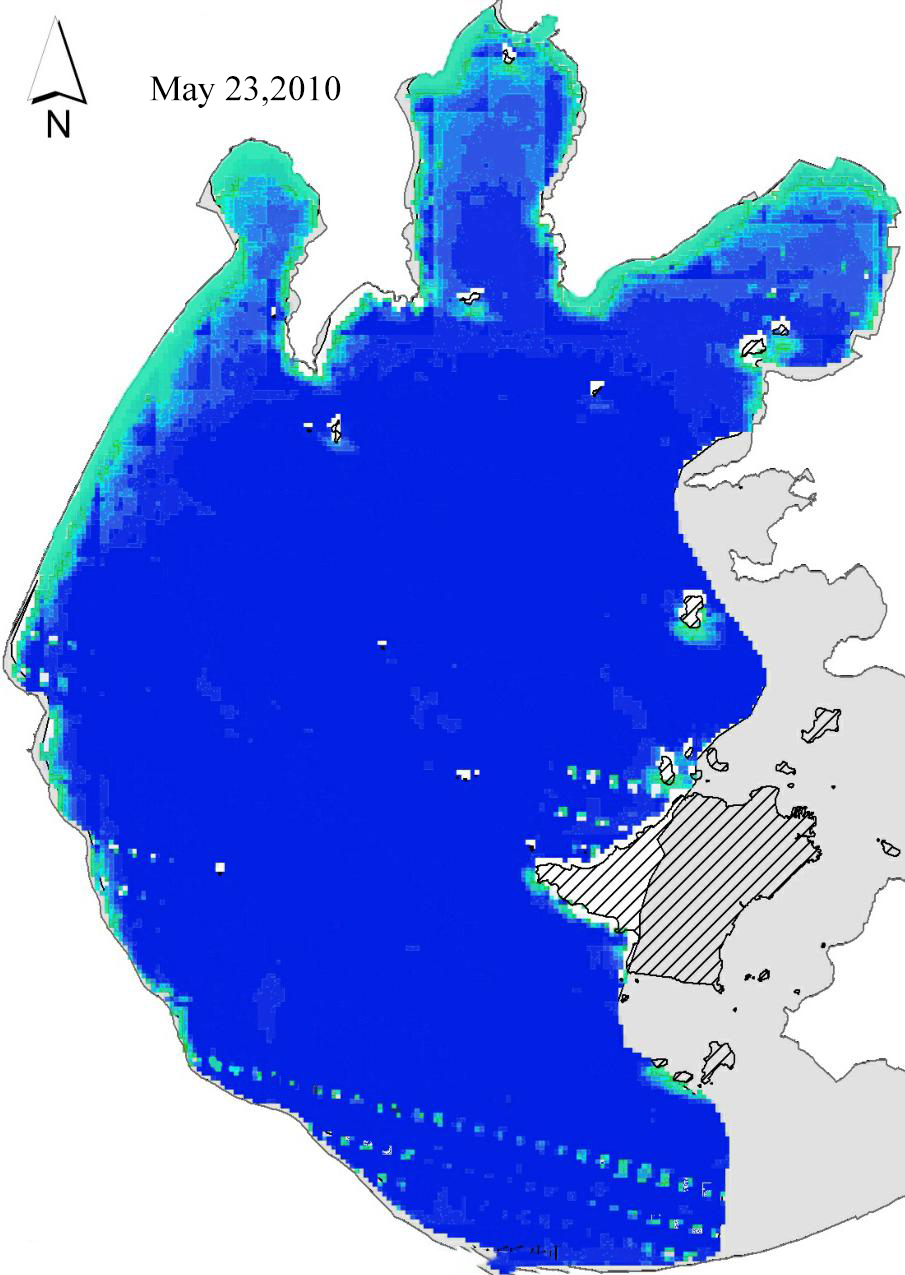

Supplement: Supplemental Information 9 — The data are remote sensing images of chlorophyll a concentration after data scale unification, remote sensing image repair, and time series filling. Remote sensing images of 30 consecutive moments were used as input to the 3D-GAN model. [file peerj-cs-09-1292-s009.zip › 201005230245.jpg]

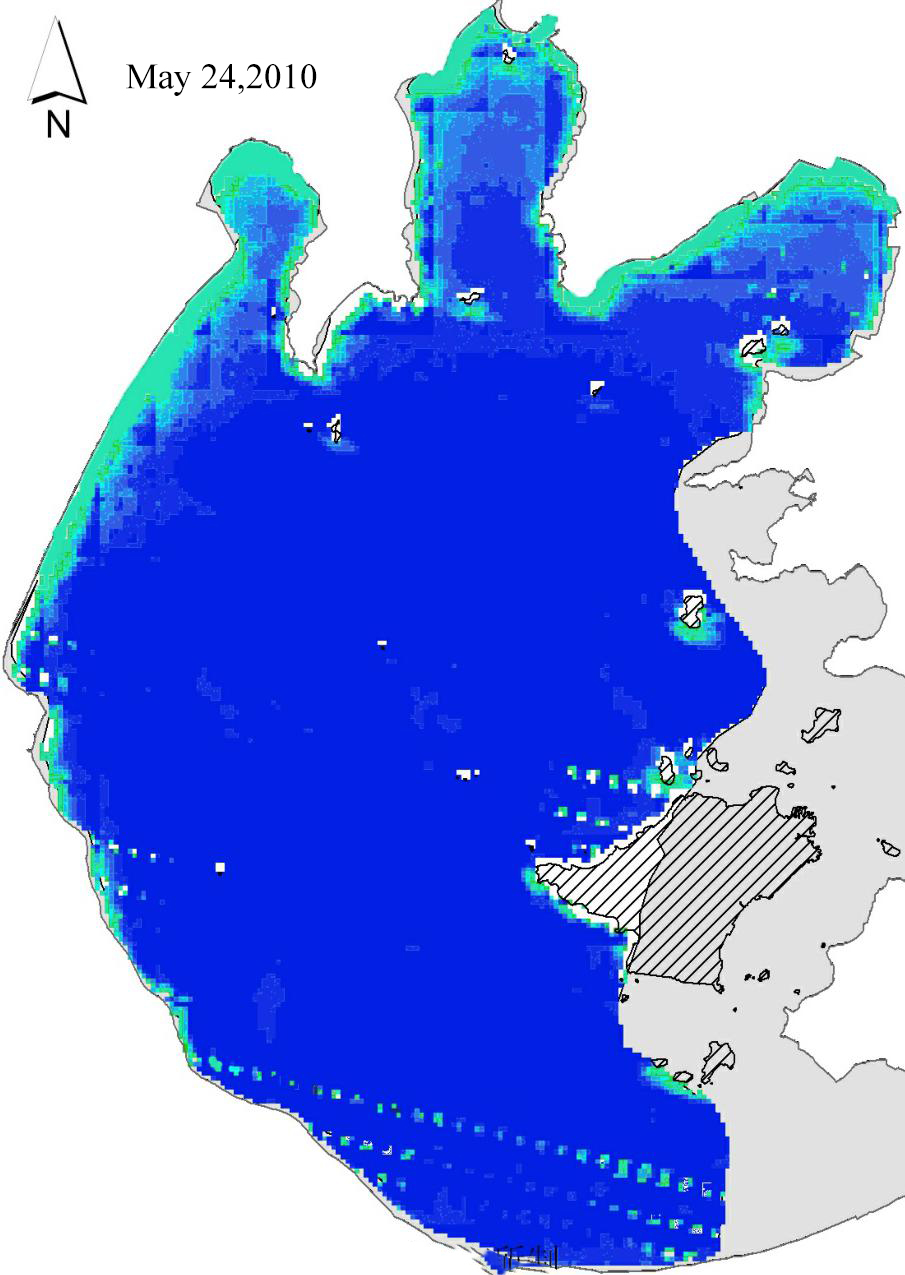

Supplement: Supplemental Information 9 — The data are remote sensing images of chlorophyll a concentration after data scale unification, remote sensing image repair, and time series filling. Remote sensing images of 30 consecutive moments were used as input to the 3D-GAN model. [file peerj-cs-09-1292-s009.zip › 201005240245.jpg]

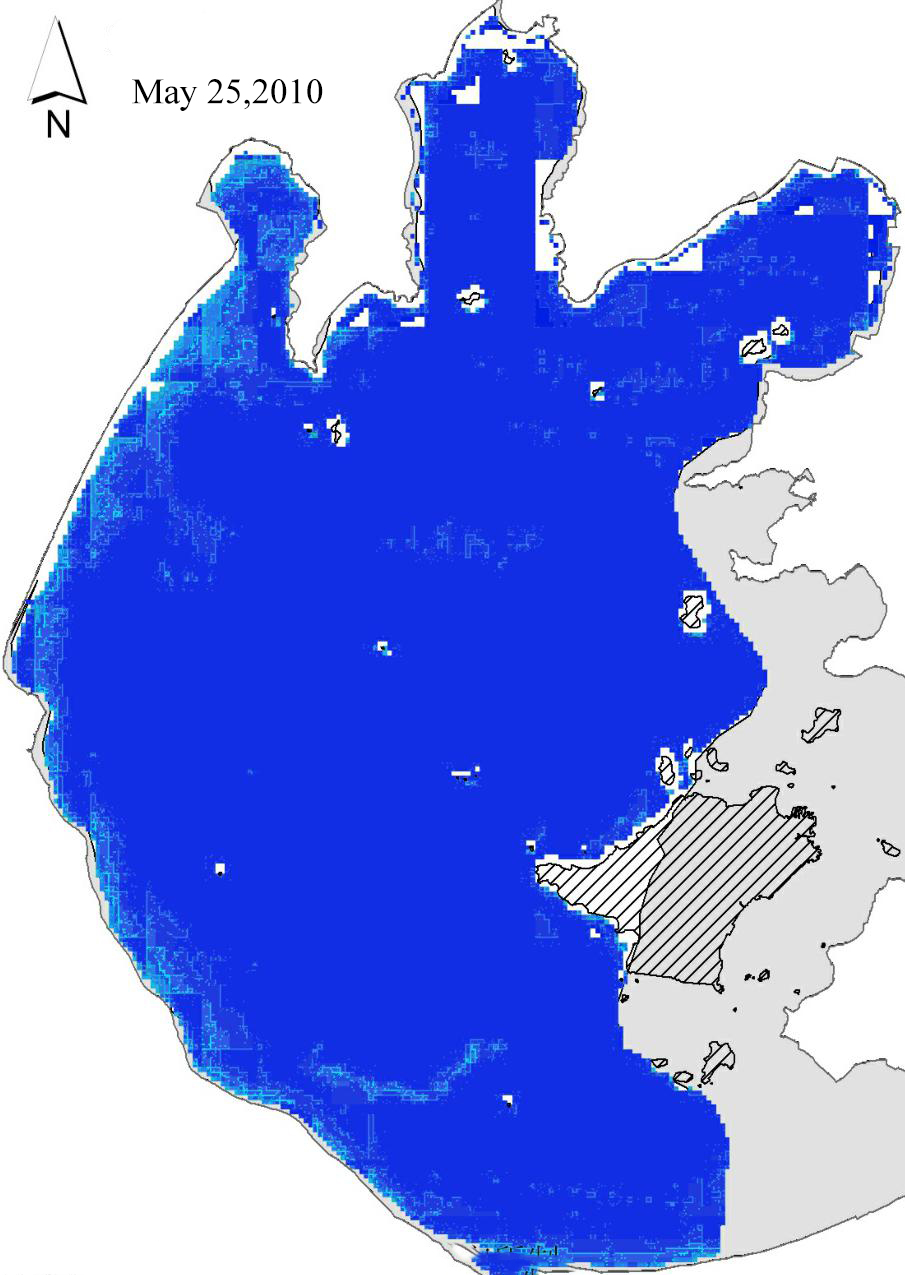

Supplement: Supplemental Information 9 — The data are remote sensing images of chlorophyll a concentration after data scale unification, remote sensing image repair, and time series filling. Remote sensing images of 30 consecutive moments were used as input to the 3D-GAN model. [file peerj-cs-09-1292-s009.zip › 201005250245.jpg]

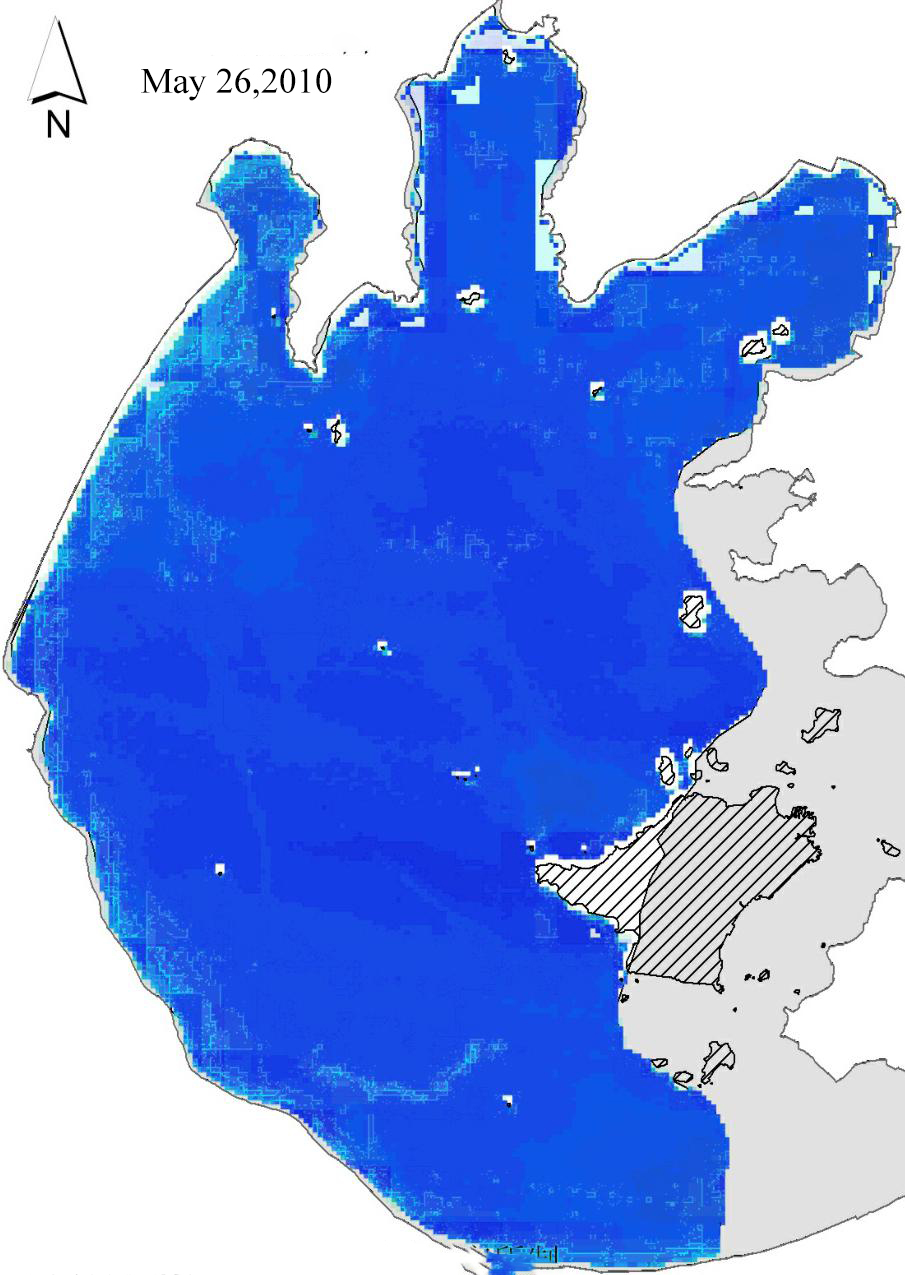

Supplement: Supplemental Information 9 — The data are remote sensing images of chlorophyll a concentration after data scale unification, remote sensing image repair, and time series filling. Remote sensing images of 30 consecutive moments were used as input to the 3D-GAN model. [file peerj-cs-09-1292-s009.zip › 201005260245.jpg]

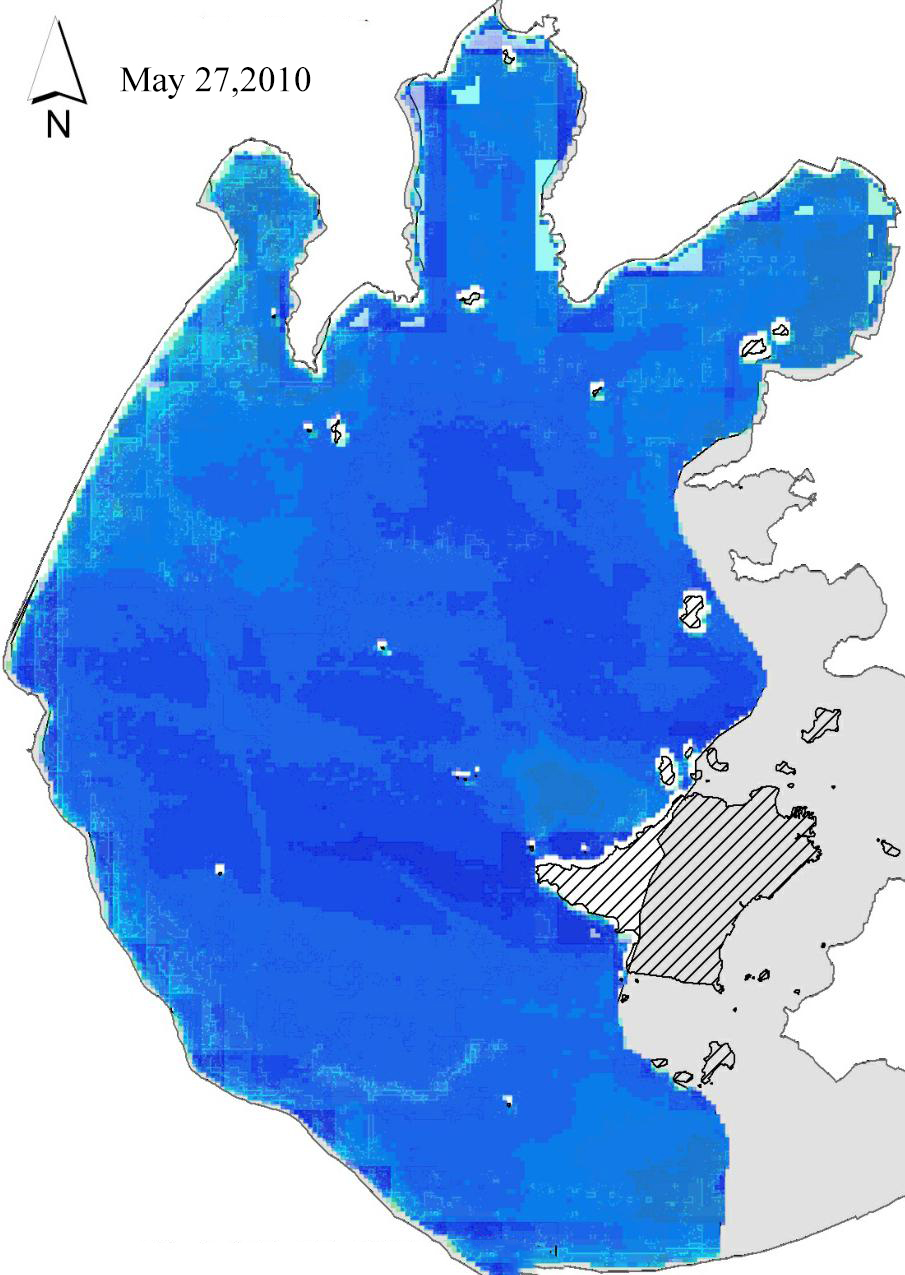

Supplement: Supplemental Information 9 — The data are remote sensing images of chlorophyll a concentration after data scale unification, remote sensing image repair, and time series filling. Remote sensing images of 30 consecutive moments were used as input to the 3D-GAN model. [file peerj-cs-09-1292-s009.zip › 201005270245.jpg]

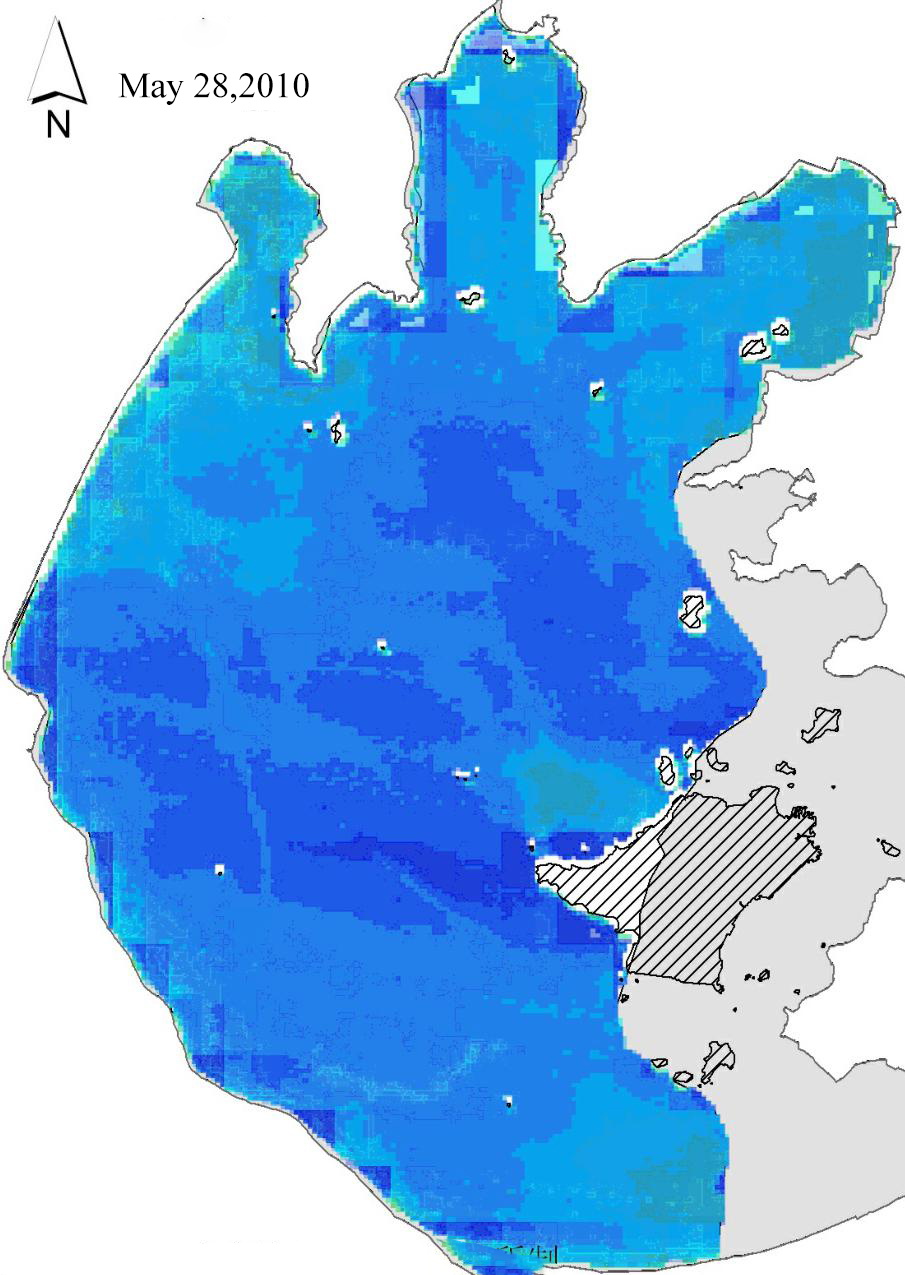

Supplement: Supplemental Information 9 — The data are remote sensing images of chlorophyll a concentration after data scale unification, remote sensing image repair, and time series filling. Remote sensing images of 30 consecutive moments were used as input to the 3D-GAN model. [file peerj-cs-09-1292-s009.zip › 201005280245.jpg]

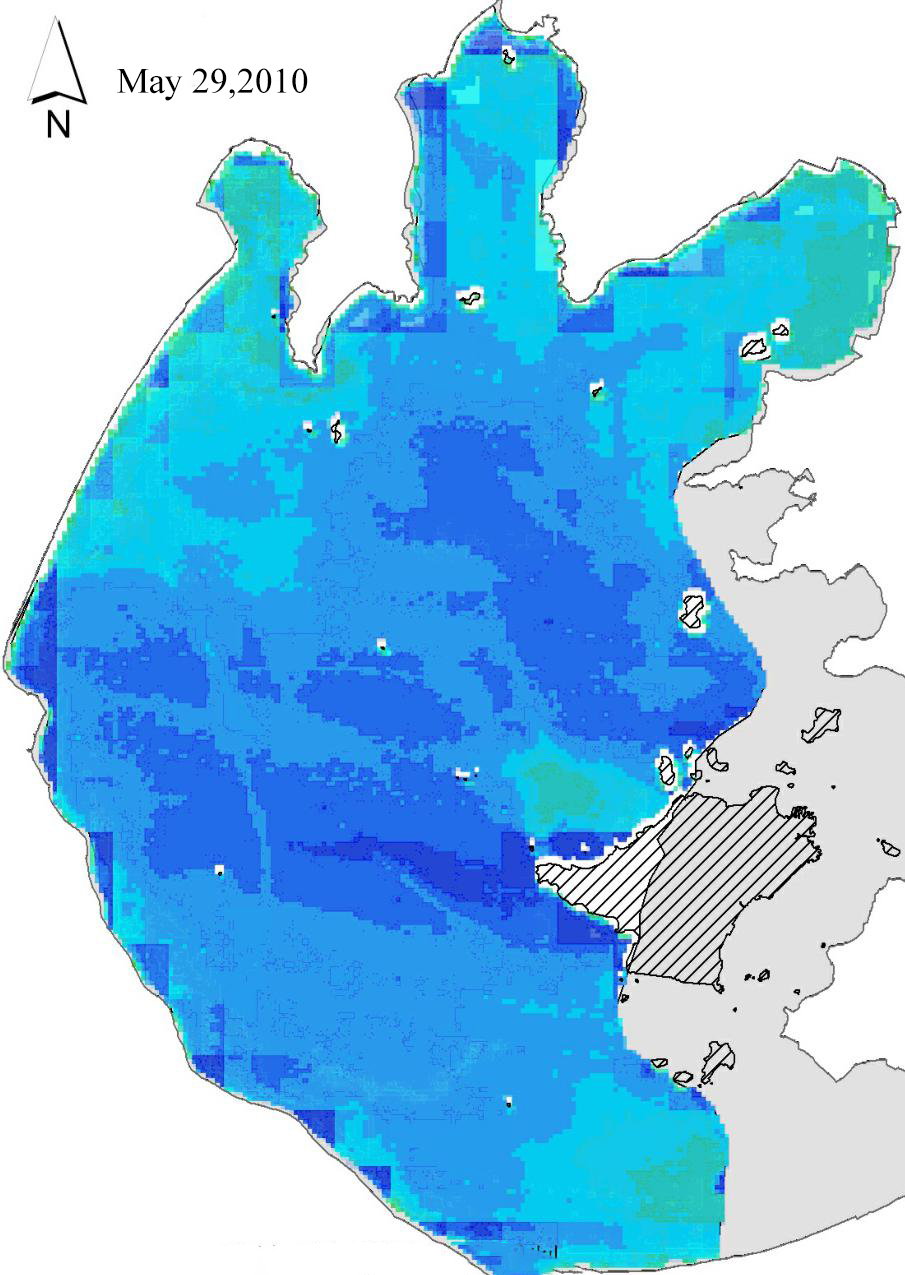

Supplement: Supplemental Information 9 — The data are remote sensing images of chlorophyll a concentration after data scale unification, remote sensing image repair, and time series filling. Remote sensing images of 30 consecutive moments were used as input to the 3D-GAN model. [file peerj-cs-09-1292-s009.zip › 201005290245.jpg]

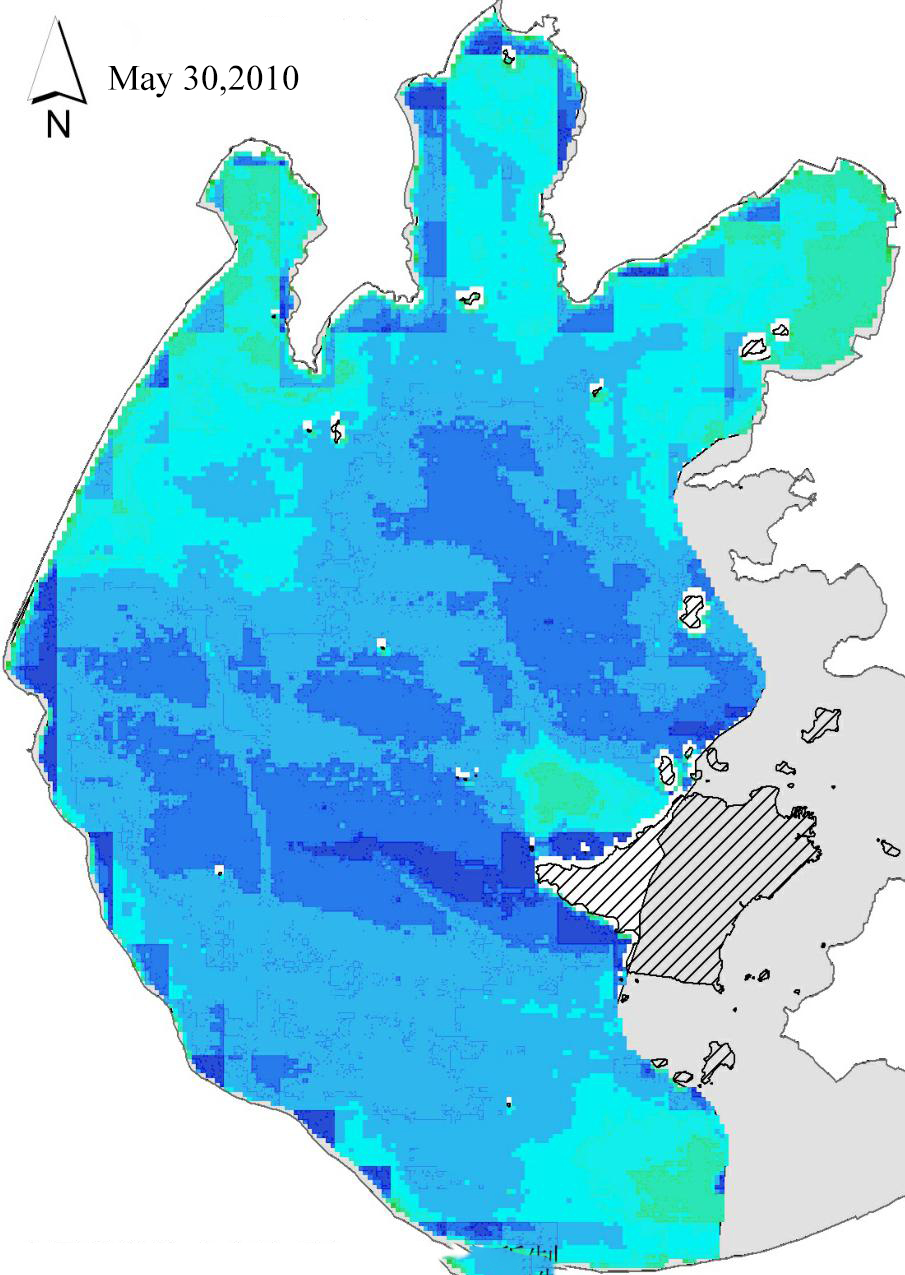

Supplement: Supplemental Information 9 — The data are remote sensing images of chlorophyll a concentration after data scale unification, remote sensing image repair, and time series filling. Remote sensing images of 30 consecutive moments were used as input to the 3D-GAN model. [file peerj-cs-09-1292-s009.zip › 201005300245.jpg]

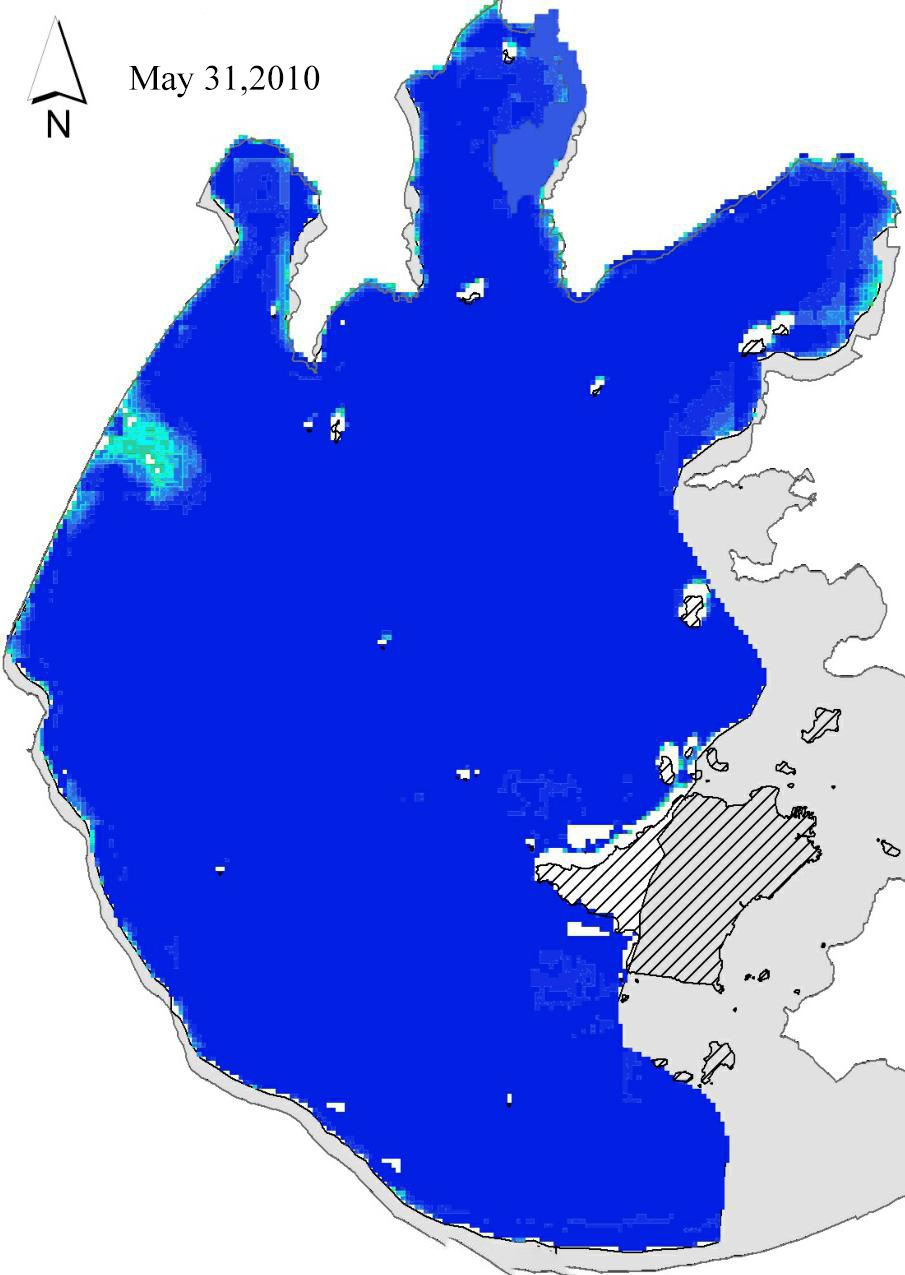

Supplement: Supplemental Information 9 — The data are remote sensing images of chlorophyll a concentration after data scale unification, remote sensing image repair, and time series filling. Remote sensing images of 30 consecutive moments were used as input to the 3D-GAN model. [file peerj-cs-09-1292-s009.zip › 201005310245.jpg]

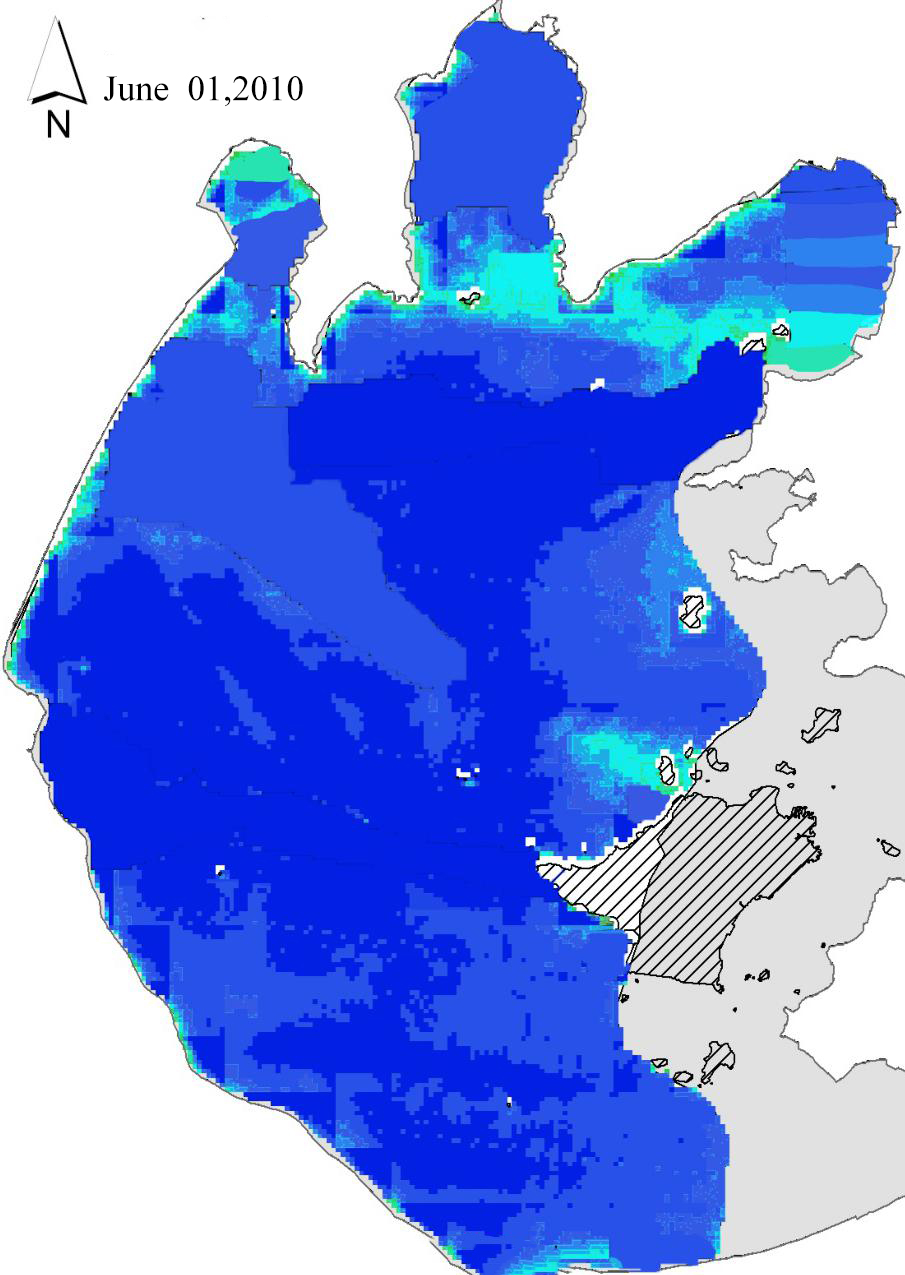

Supplement: Supplemental Information 9 — The data are remote sensing images of chlorophyll a concentration after data scale unification, remote sensing image repair, and time series filling. Remote sensing images of 30 consecutive moments were used as input to the 3D-GAN model. [file peerj-cs-09-1292-s009.zip › 201006010245.jpg]

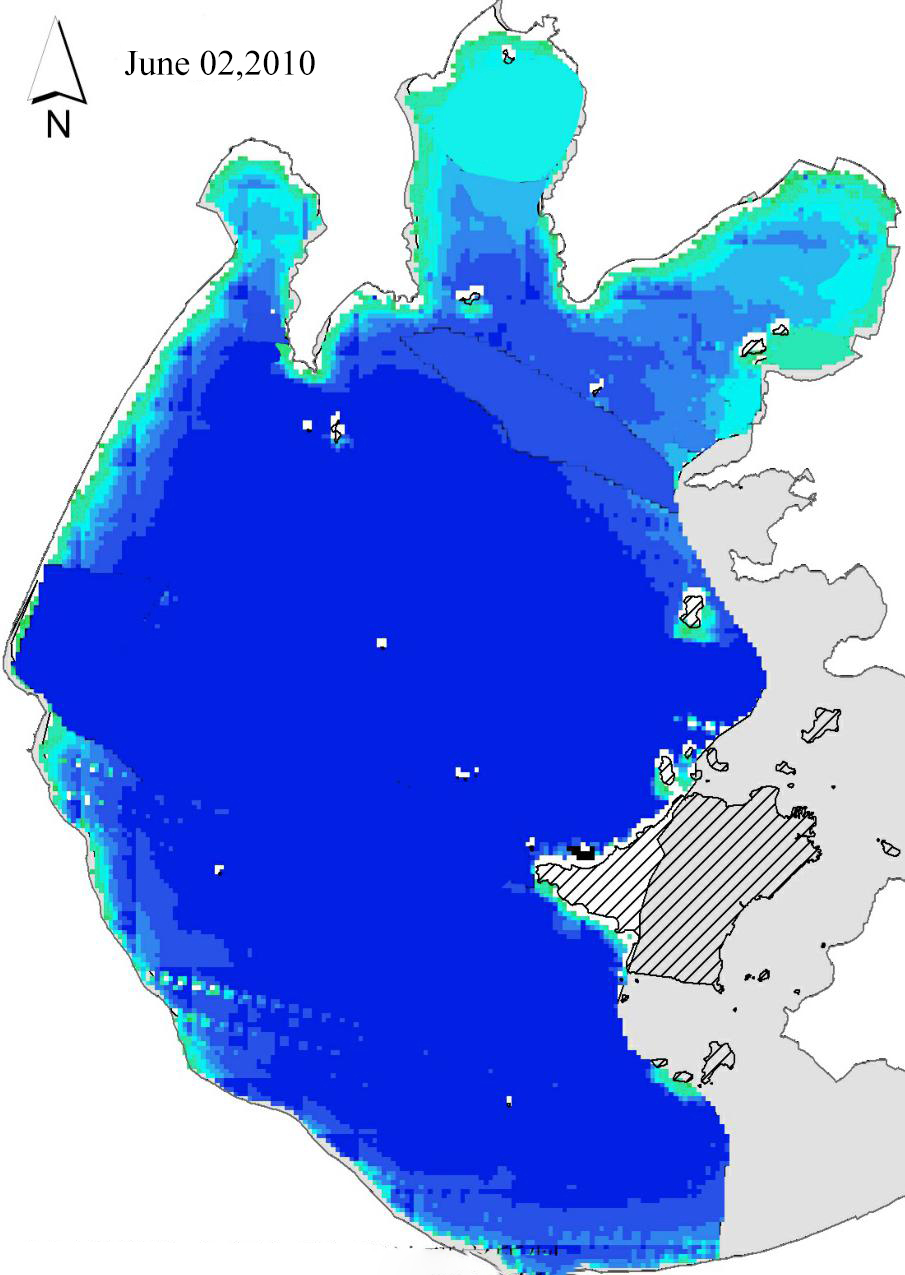

Supplement: Supplemental Information 9 — The data are remote sensing images of chlorophyll a concentration after data scale unification, remote sensing image repair, and time series filling. Remote sensing images of 30 consecutive moments were used as input to the 3D-GAN model. [file peerj-cs-09-1292-s009.zip › 201006020245.jpg]

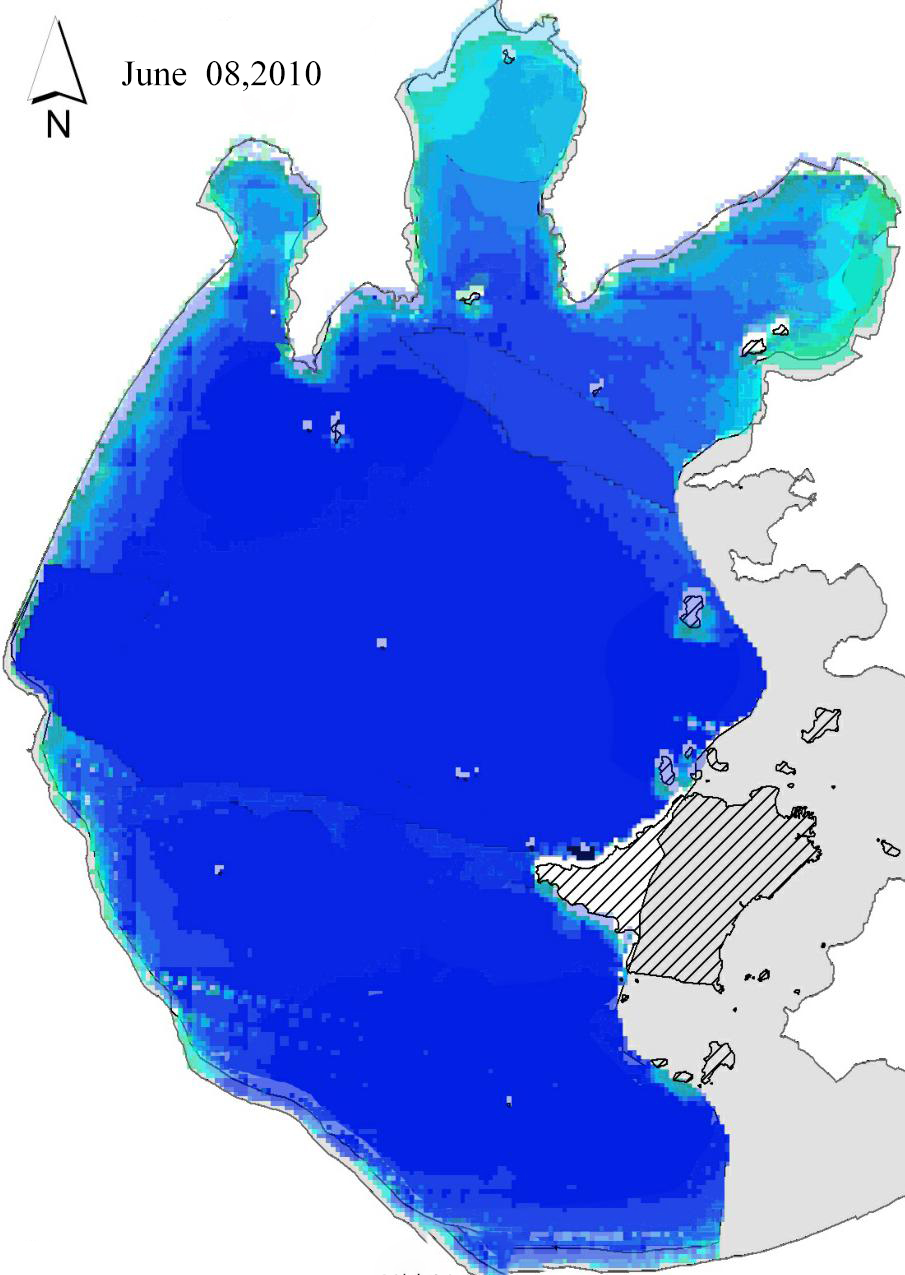

Supplement: Supplemental Information 10 — The data are remote sensing images of chlorophyll a concentration after data scale unification, remote sensing image repair, and time series filling. Remote sensing images of 30 consecutive moments were used as input to the 3D-GAN model. [file peerj-cs-09-1292-s010.zip › 201006030245.jpg]

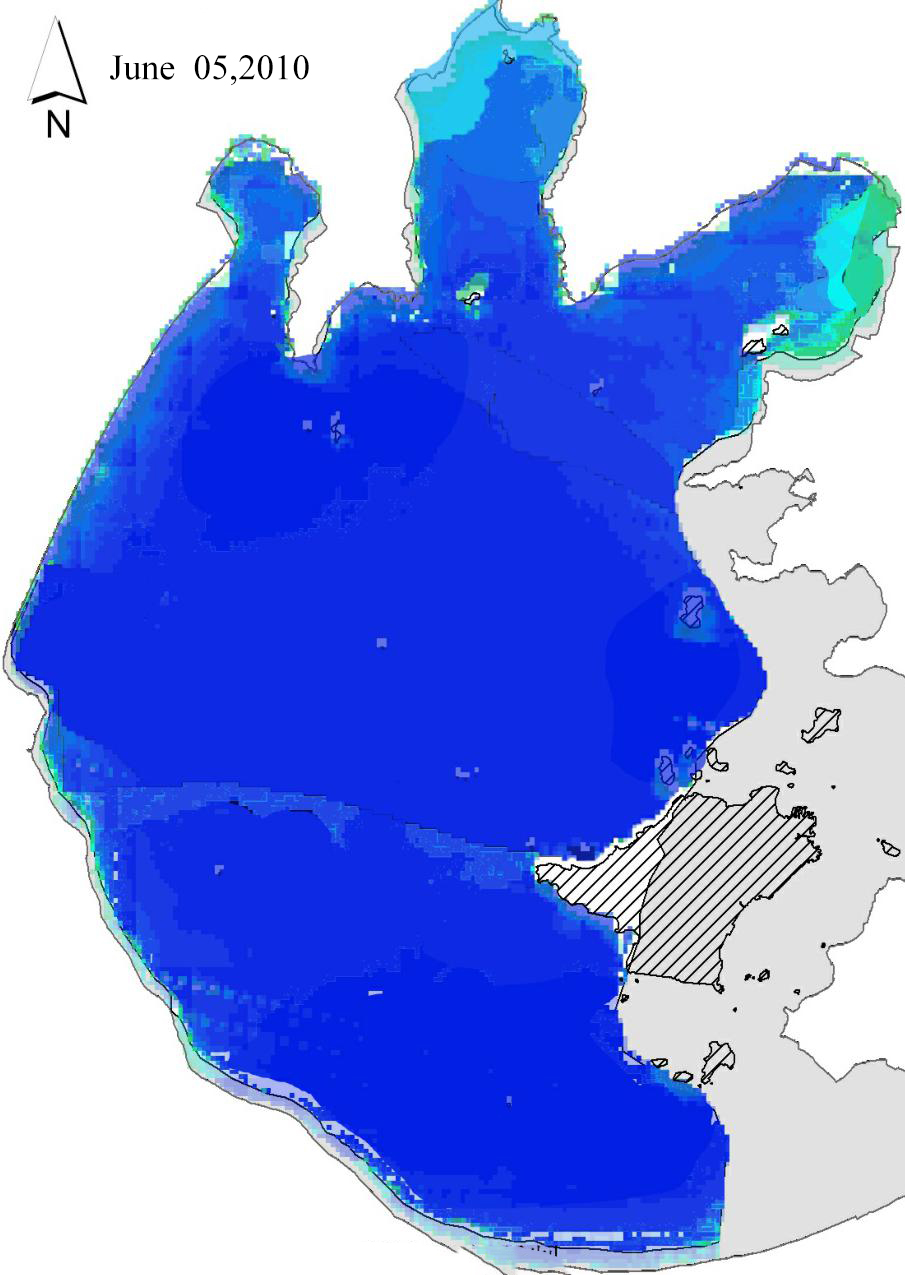

Supplement: Supplemental Information 10 — The data are remote sensing images of chlorophyll a concentration after data scale unification, remote sensing image repair, and time series filling. Remote sensing images of 30 consecutive moments were used as input to the 3D-GAN model. [file peerj-cs-09-1292-s010.zip › 201006040245.jpg]

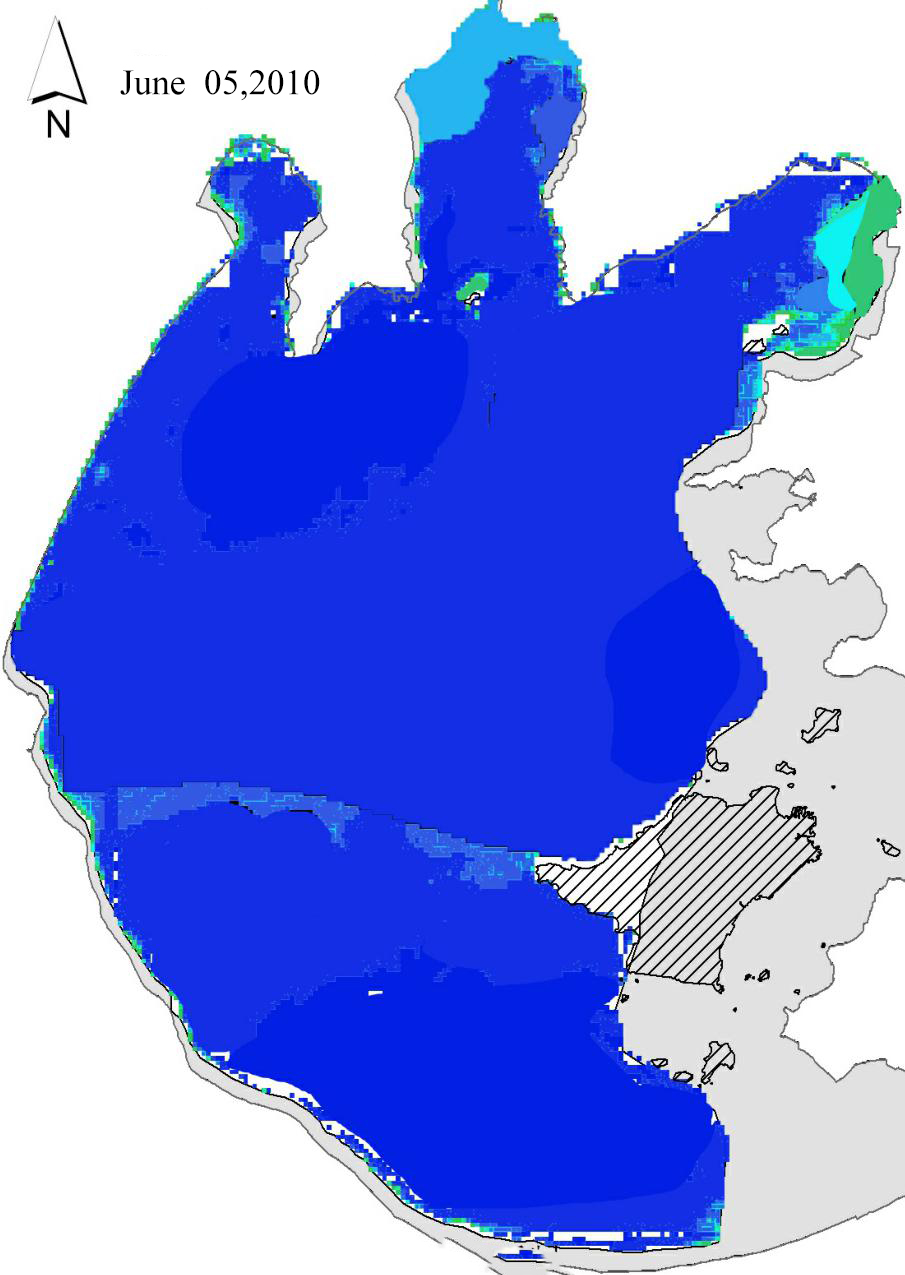

Supplement: Supplemental Information 10 — The data are remote sensing images of chlorophyll a concentration after data scale unification, remote sensing image repair, and time series filling. Remote sensing images of 30 consecutive moments were used as input to the 3D-GAN model. [file peerj-cs-09-1292-s010.zip › 201006050245.jpg]

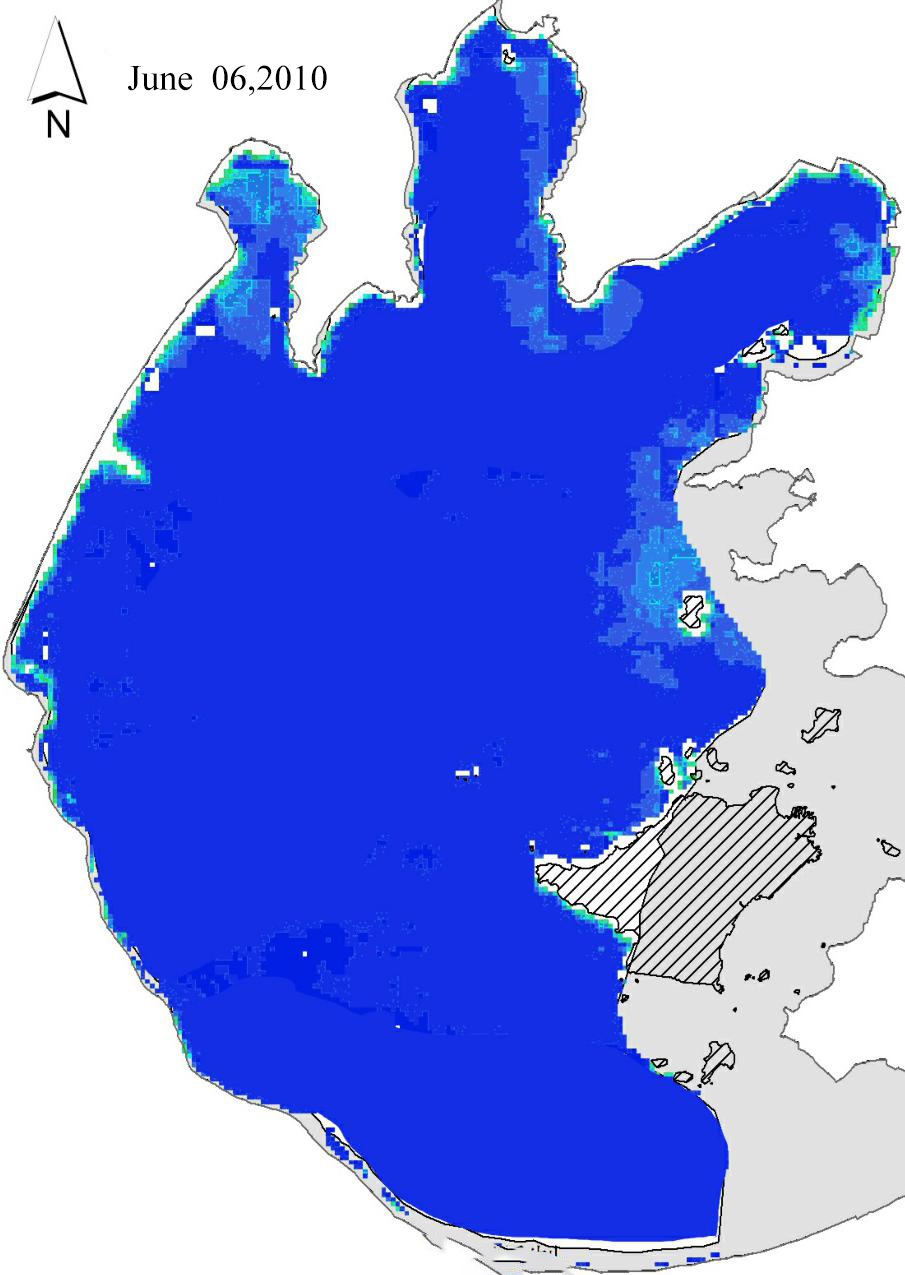

Supplement: Supplemental Information 10 — The data are remote sensing images of chlorophyll a concentration after data scale unification, remote sensing image repair, and time series filling. Remote sensing images of 30 consecutive moments were used as input to the 3D-GAN model. [file peerj-cs-09-1292-s010.zip › 201006060245.jpg]

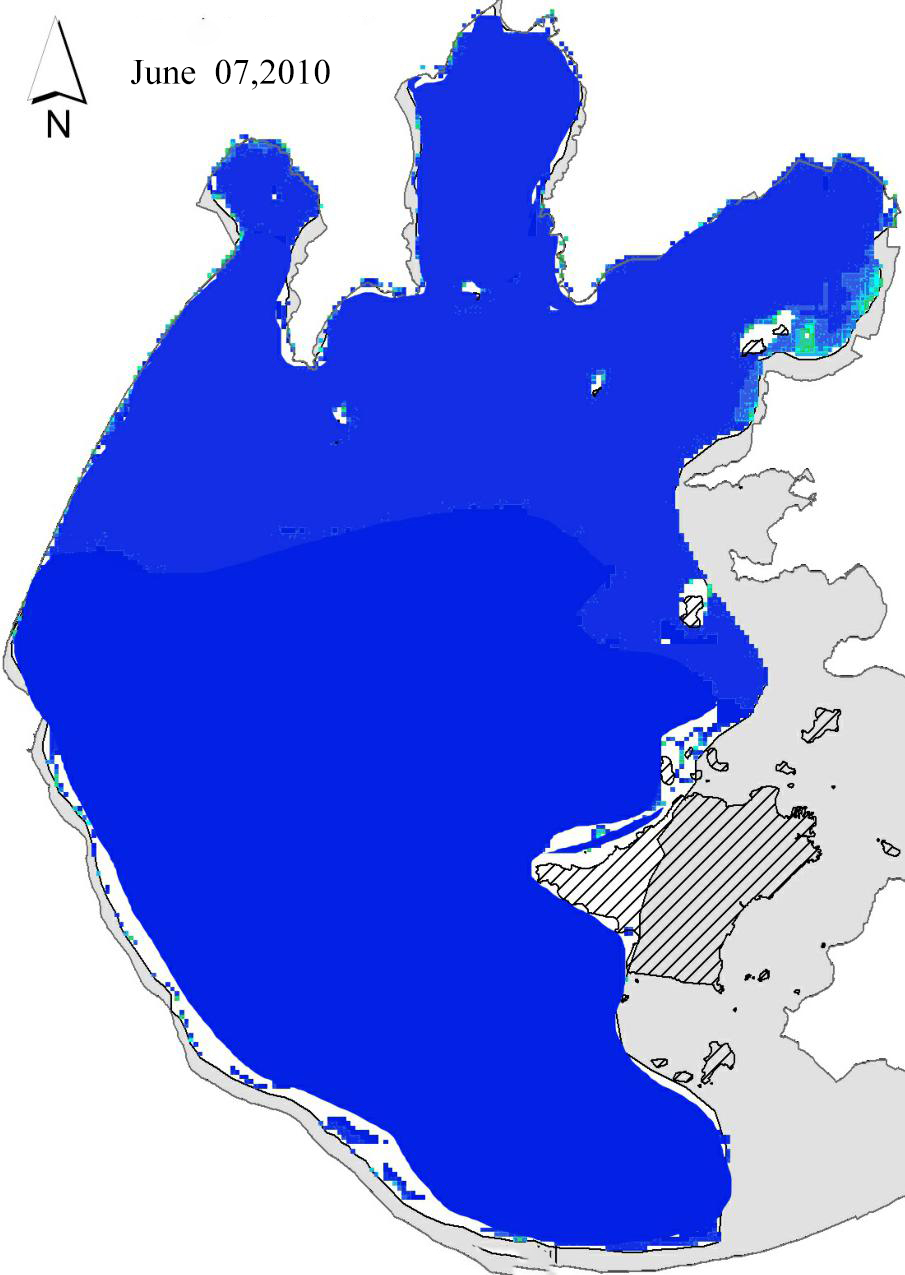

Supplement: Supplemental Information 10 — The data are remote sensing images of chlorophyll a concentration after data scale unification, remote sensing image repair, and time series filling. Remote sensing images of 30 consecutive moments were used as input to the 3D-GAN model. [file peerj-cs-09-1292-s010.zip › 201006070245.jpg]

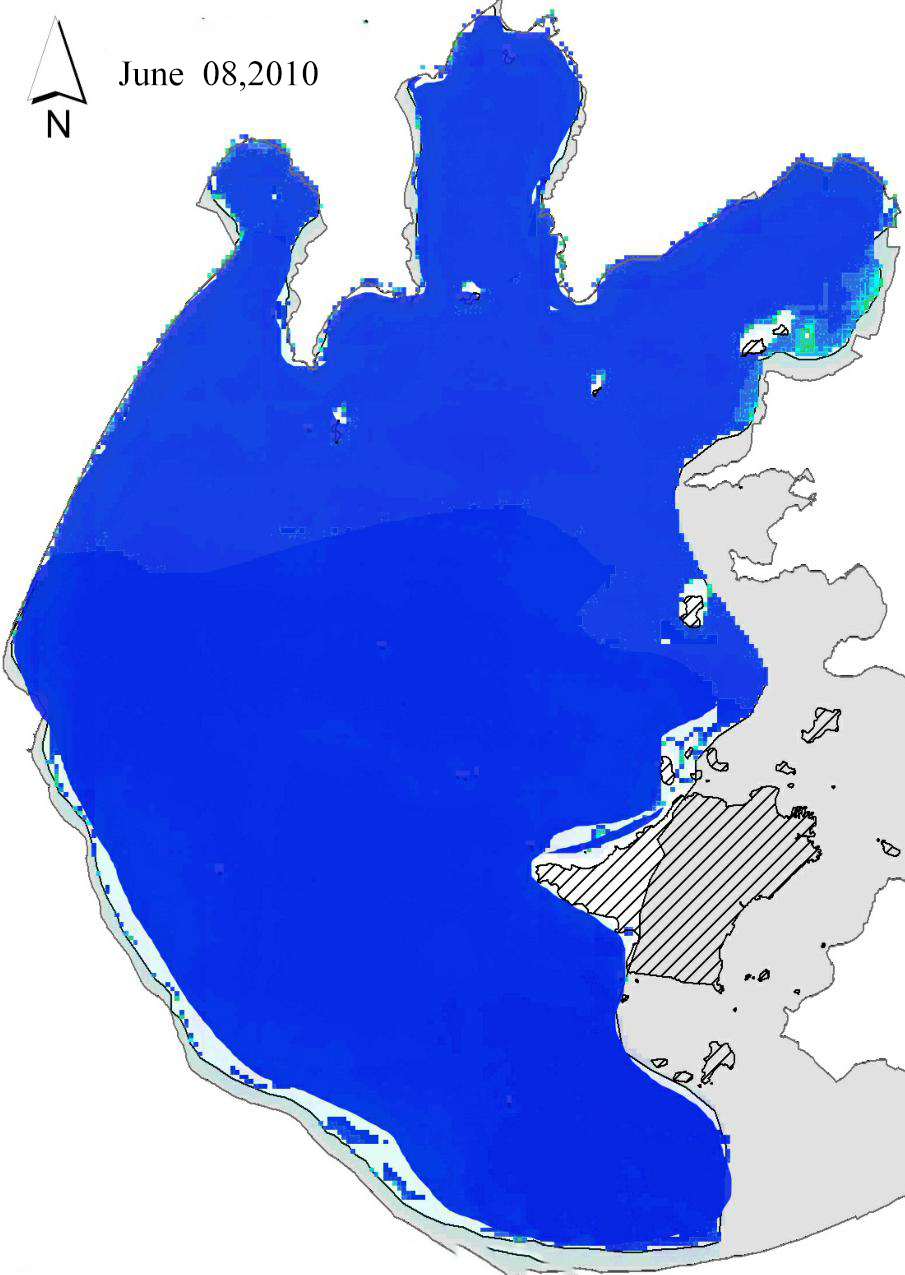

Supplement: Supplemental Information 10 — The data are remote sensing images of chlorophyll a concentration after data scale unification, remote sensing image repair, and time series filling. Remote sensing images of 30 consecutive moments were used as input to the 3D-GAN model. [file peerj-cs-09-1292-s010.zip › 201006080245.jpg]

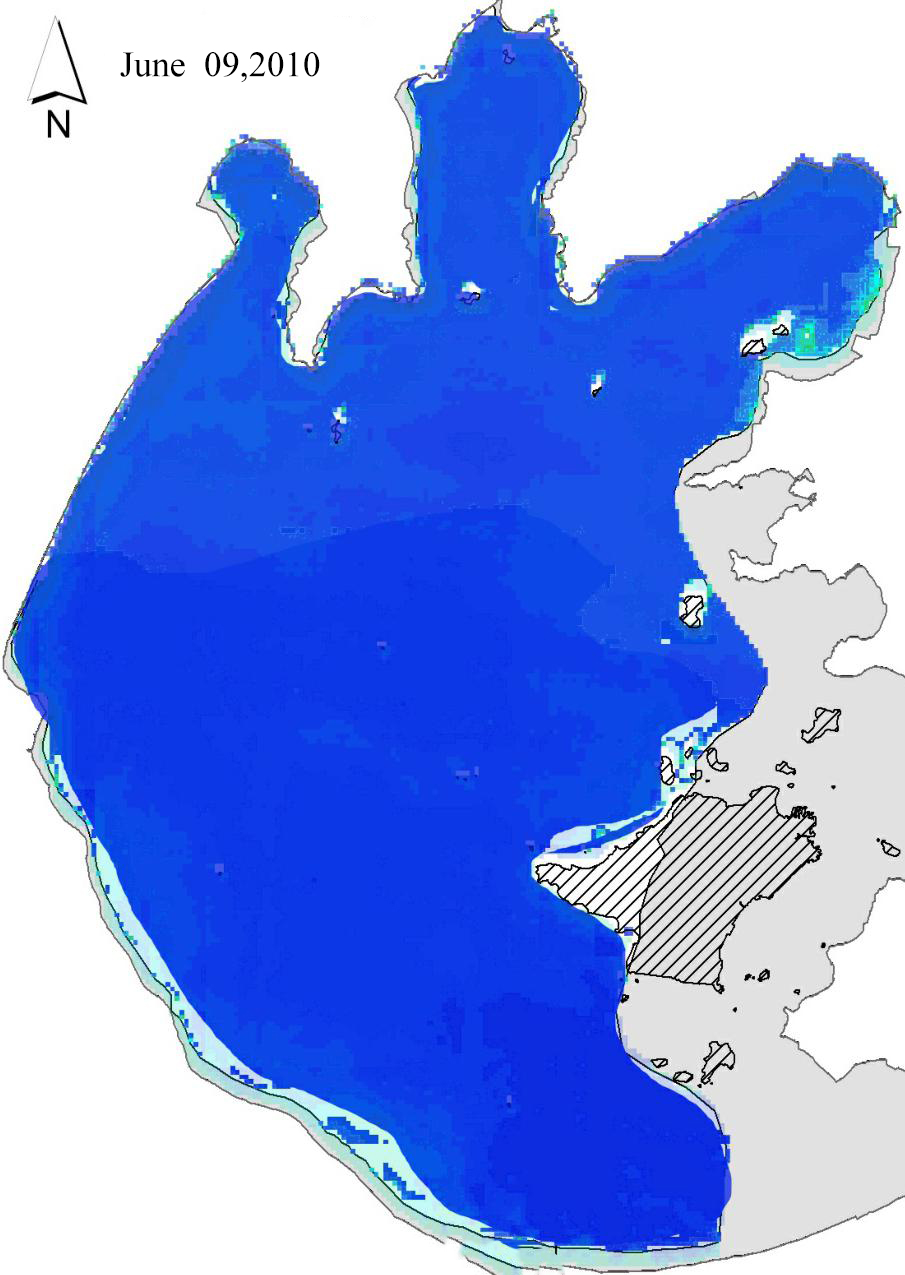

Supplement: Supplemental Information 10 — The data are remote sensing images of chlorophyll a concentration after data scale unification, remote sensing image repair, and time series filling. Remote sensing images of 30 consecutive moments were used as input to the 3D-GAN model. [file peerj-cs-09-1292-s010.zip › 201006090245.jpg]

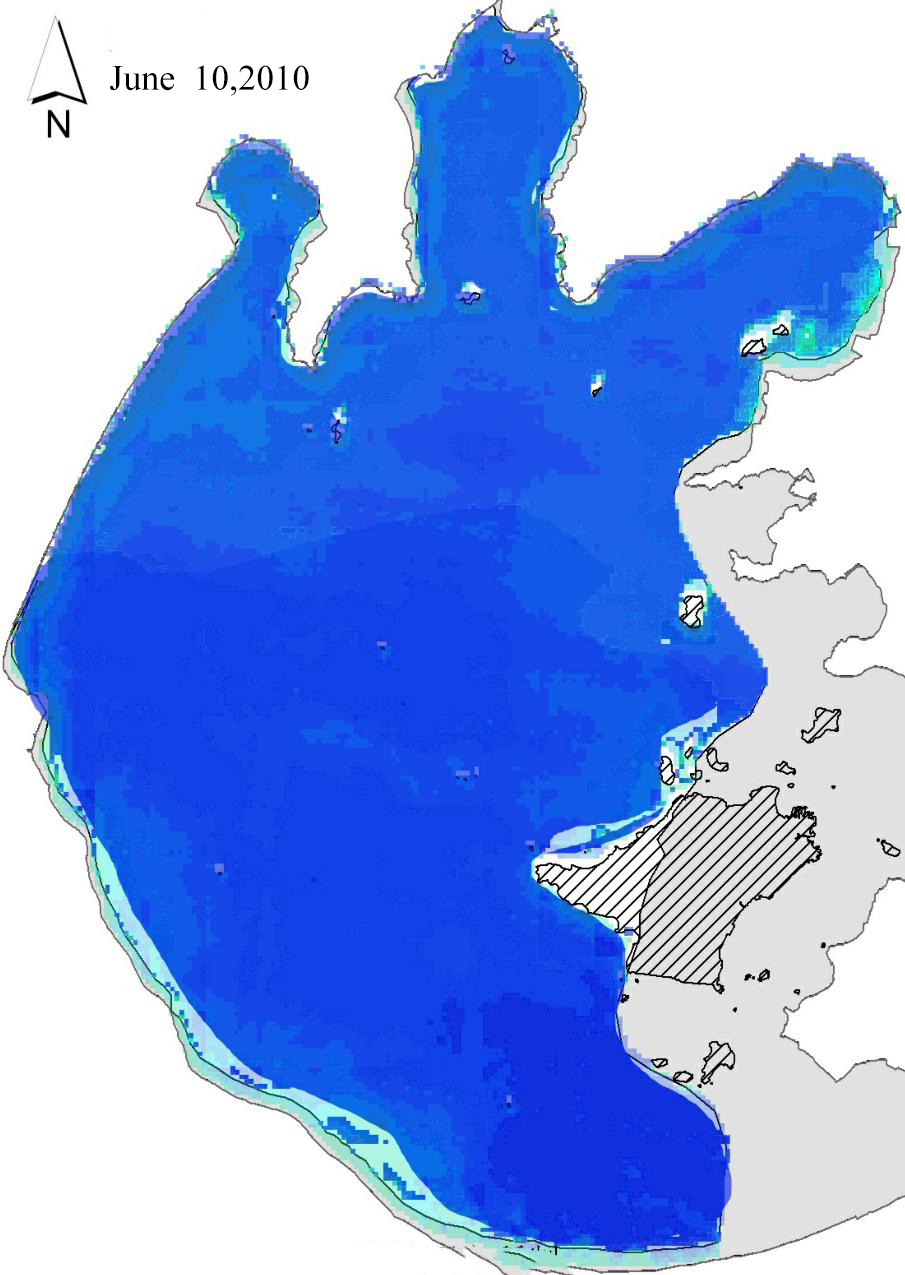

Supplement: Supplemental Information 10 — The data are remote sensing images of chlorophyll a concentration after data scale unification, remote sensing image repair, and time series filling. Remote sensing images of 30 consecutive moments were used as input to the 3D-GAN model. [file peerj-cs-09-1292-s010.zip › 201006100245.jpg]

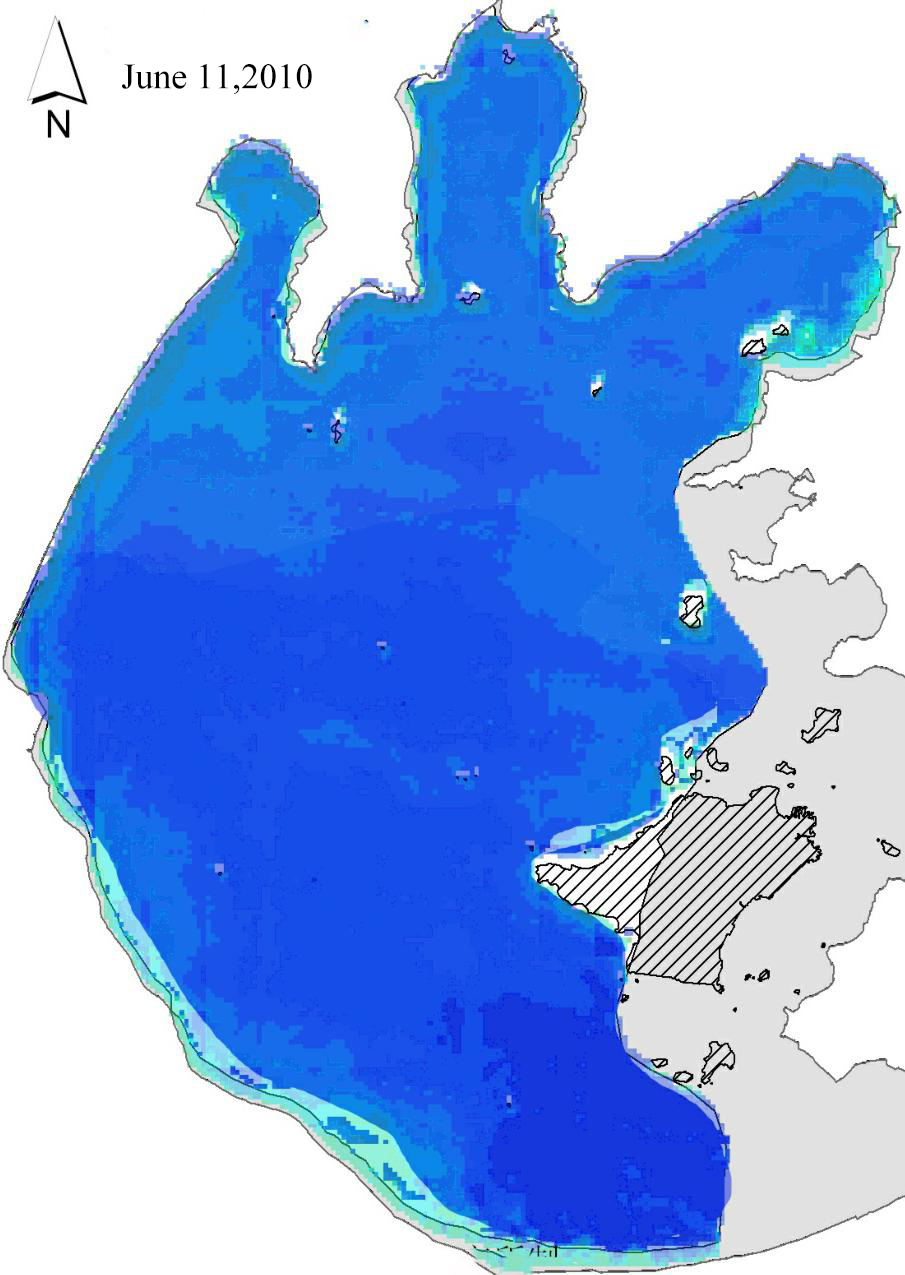

Supplement: Supplemental Information 10 — The data are remote sensing images of chlorophyll a concentration after data scale unification, remote sensing image repair, and time series filling. Remote sensing images of 30 consecutive moments were used as input to the 3D-GAN model. [file peerj-cs-09-1292-s010.zip › 201006110245.jpg]

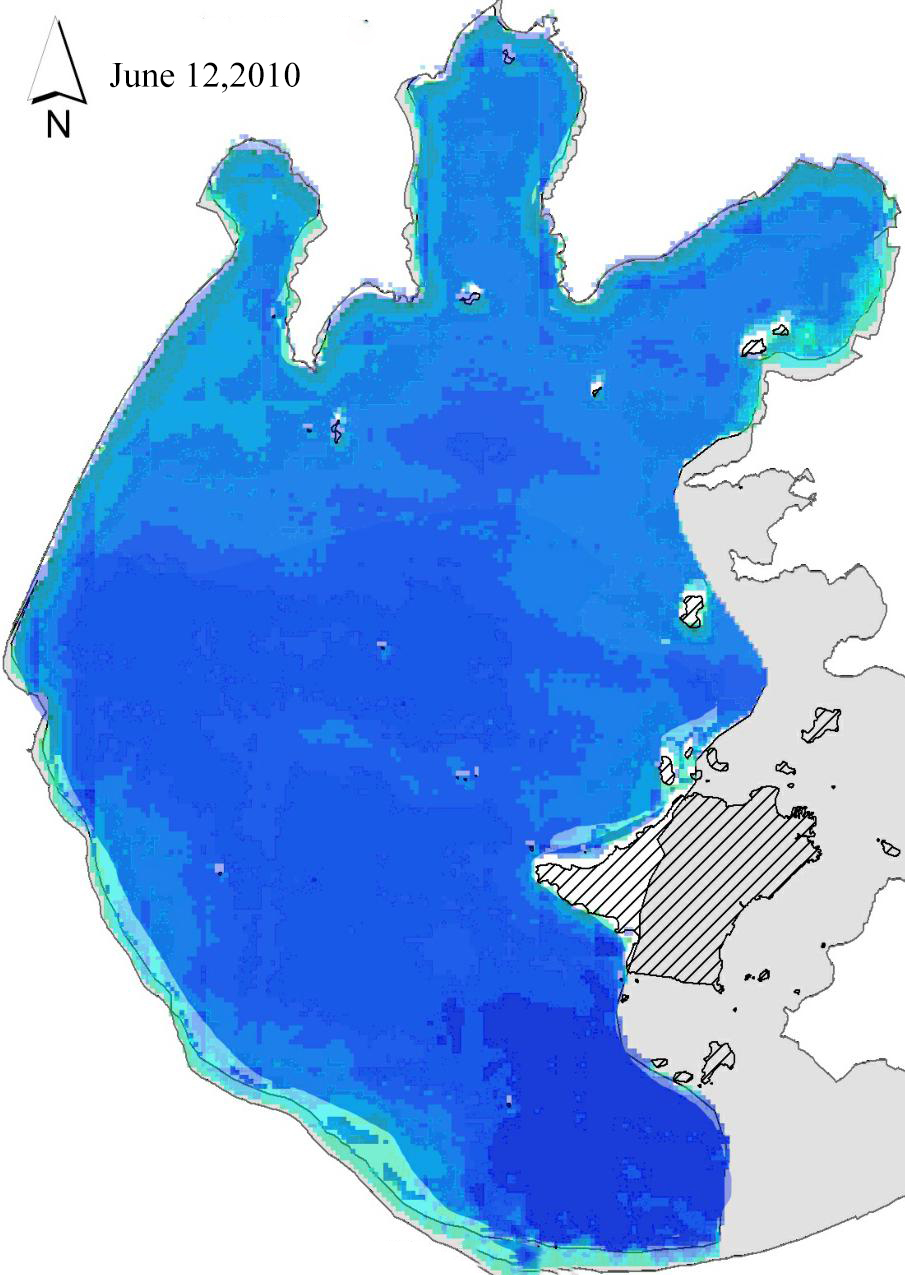

Supplement: Supplemental Information 10 — The data are remote sensing images of chlorophyll a concentration after data scale unification, remote sensing image repair, and time series filling. Remote sensing images of 30 consecutive moments were used as input to the 3D-GAN model. [file peerj-cs-09-1292-s010.zip › 201006120245.jpg]

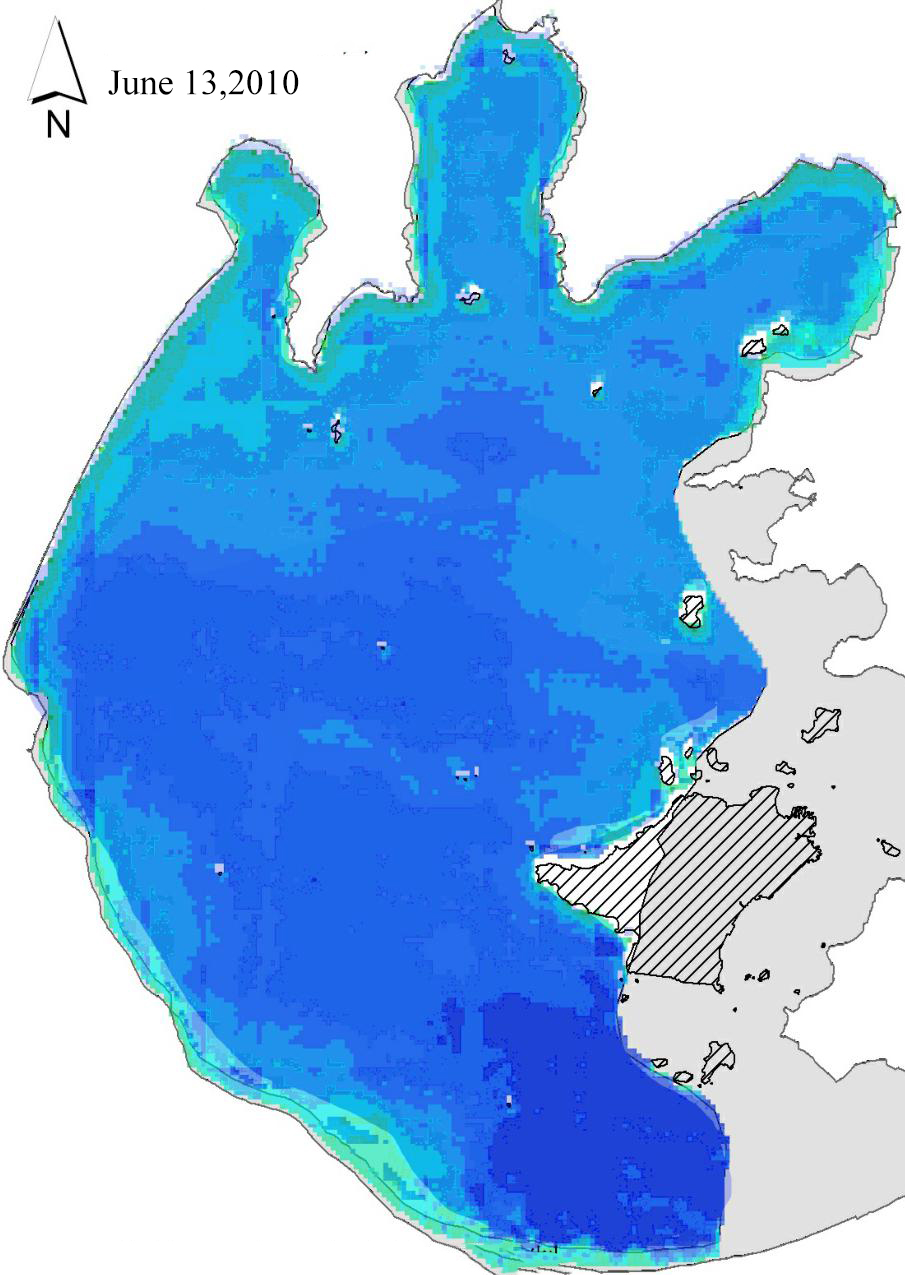

Supplement: Supplemental Information 10 — The data are remote sensing images of chlorophyll a concentration after data scale unification, remote sensing image repair, and time series filling. Remote sensing images of 30 consecutive moments were used as input to the 3D-GAN model. [file peerj-cs-09-1292-s010.zip › 201006130245.jpg]

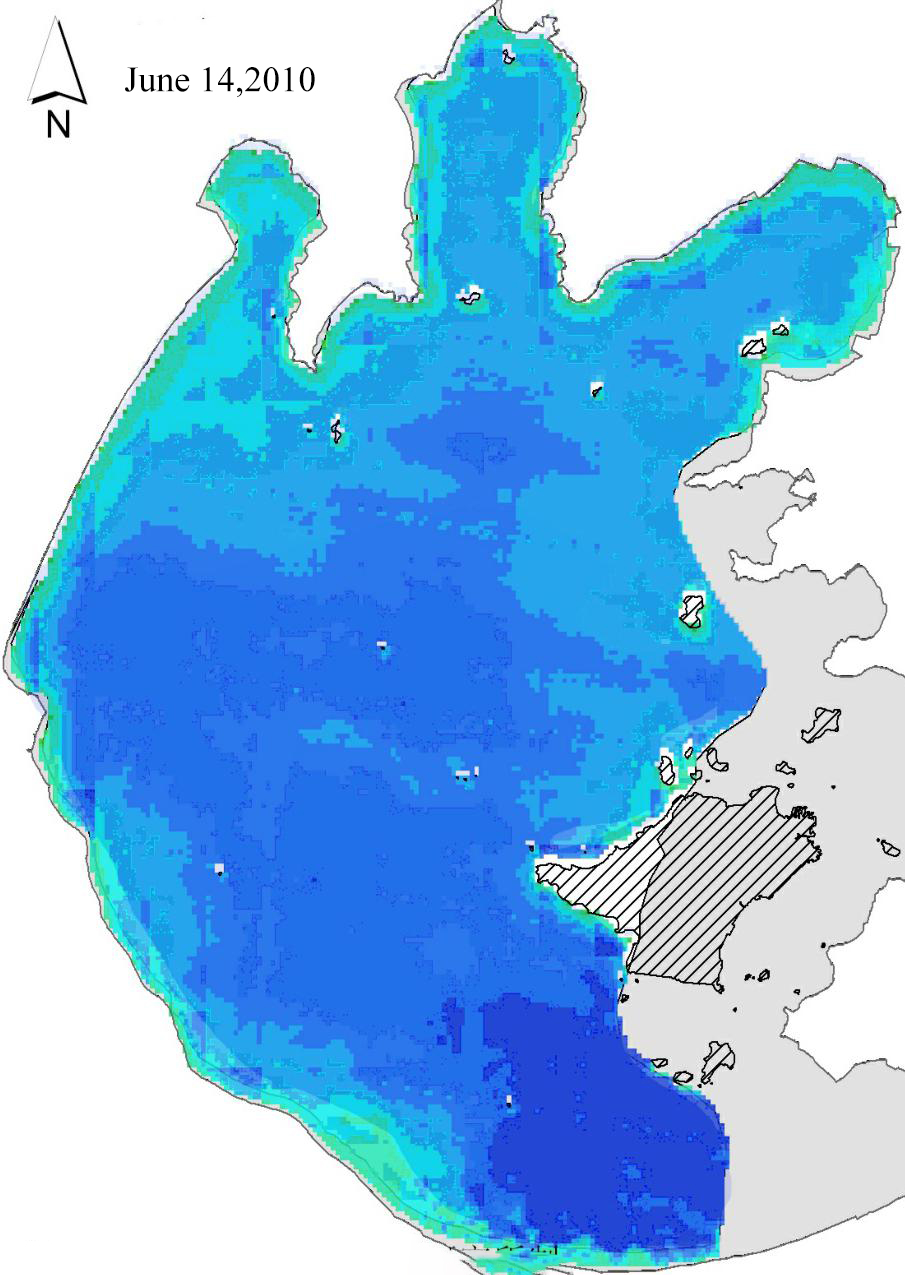

Supplement: Supplemental Information 10 — The data are remote sensing images of chlorophyll a concentration after data scale unification, remote sensing image repair, and time series filling. Remote sensing images of 30 consecutive moments were used as input to the 3D-GAN model. [file peerj-cs-09-1292-s010.zip › 201006140245.jpg]

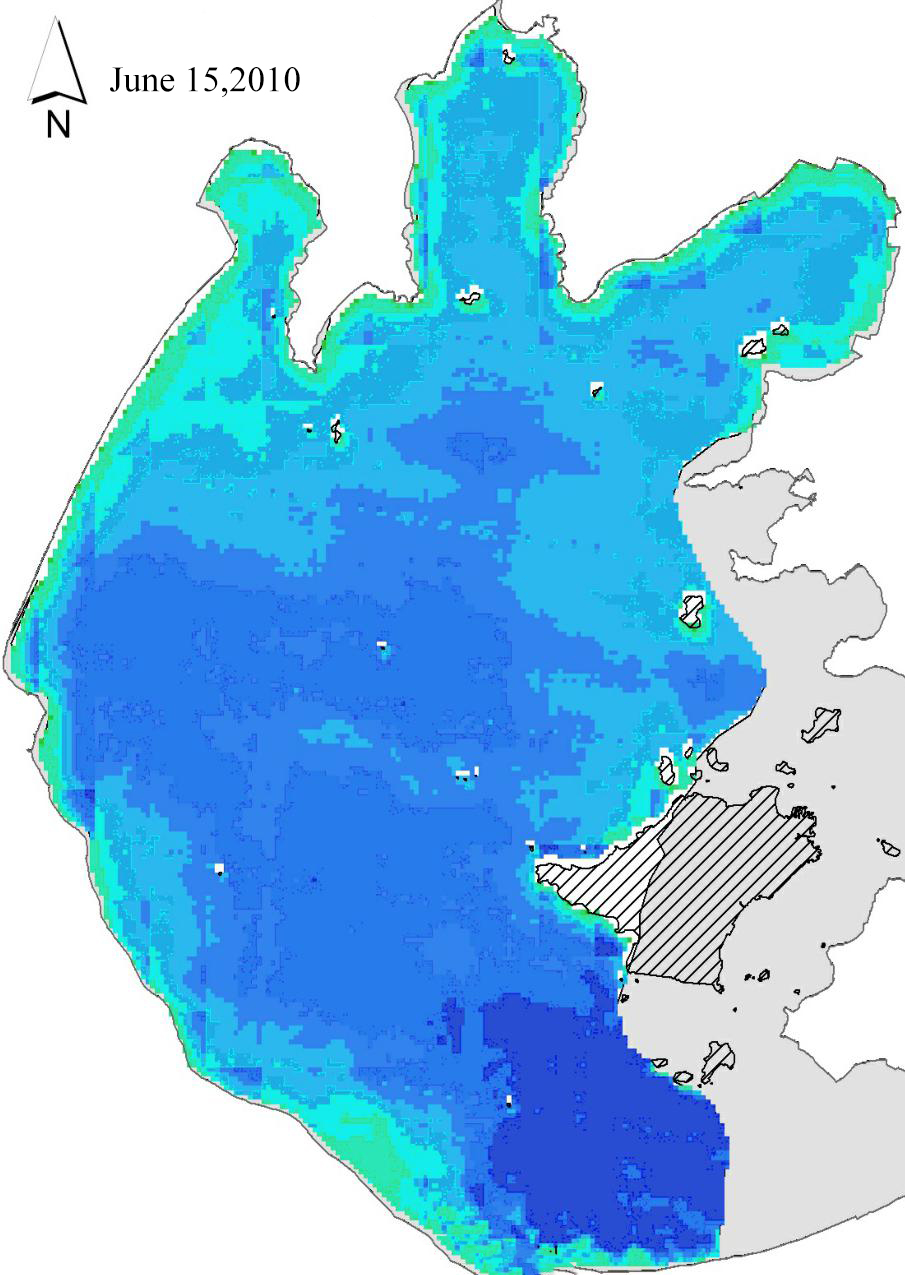

Supplement: Supplemental Information 10 — The data are remote sensing images of chlorophyll a concentration after data scale unification, remote sensing image repair, and time series filling. Remote sensing images of 30 consecutive moments were used as input to the 3D-GAN model. [file peerj-cs-09-1292-s010.zip › 201006150245.jpg]

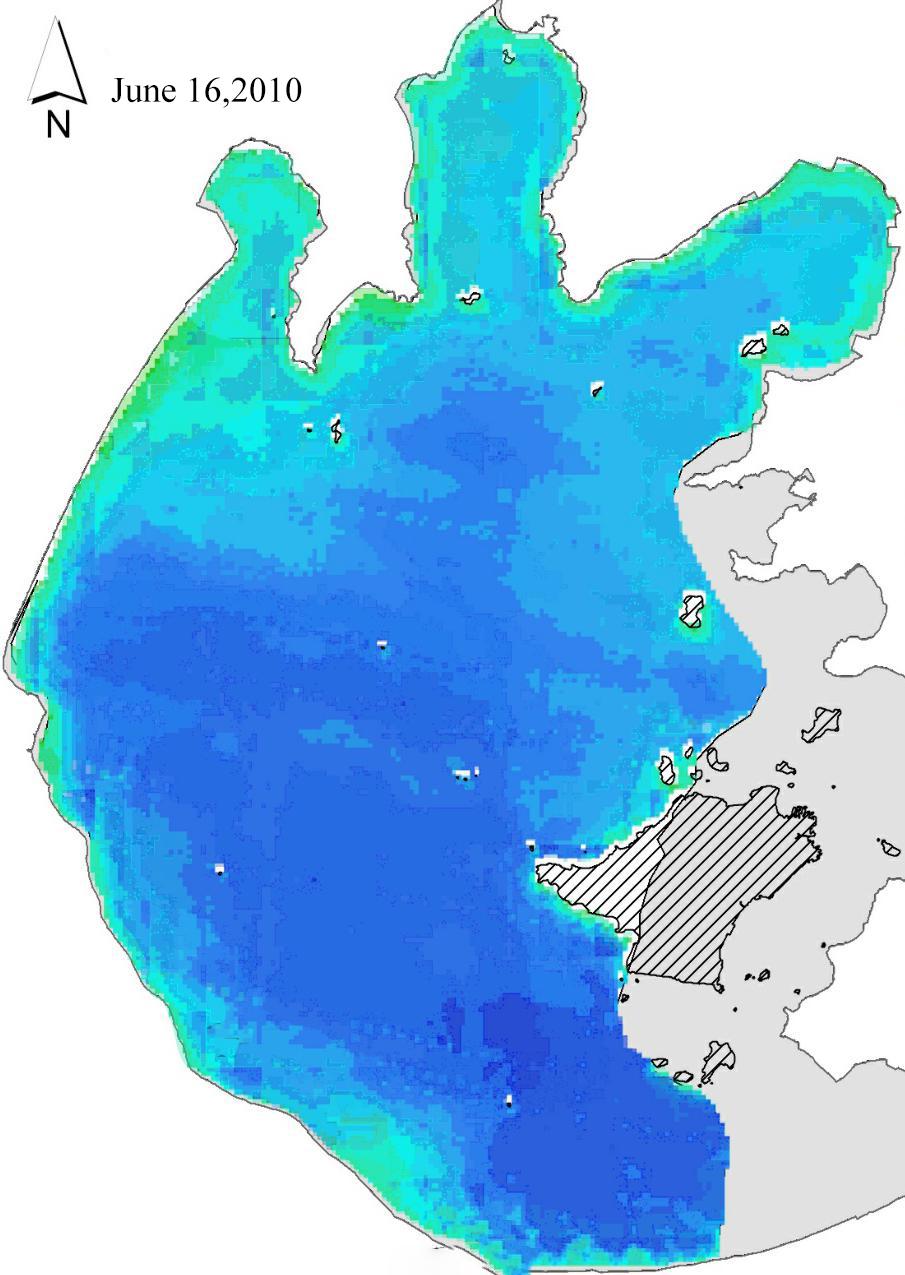

Supplement: Supplemental Information 10 — The data are remote sensing images of chlorophyll a concentration after data scale unification, remote sensing image repair, and time series filling. Remote sensing images of 30 consecutive moments were used as input to the 3D-GAN model. [file peerj-cs-09-1292-s010.zip › 201006160245.jpg]

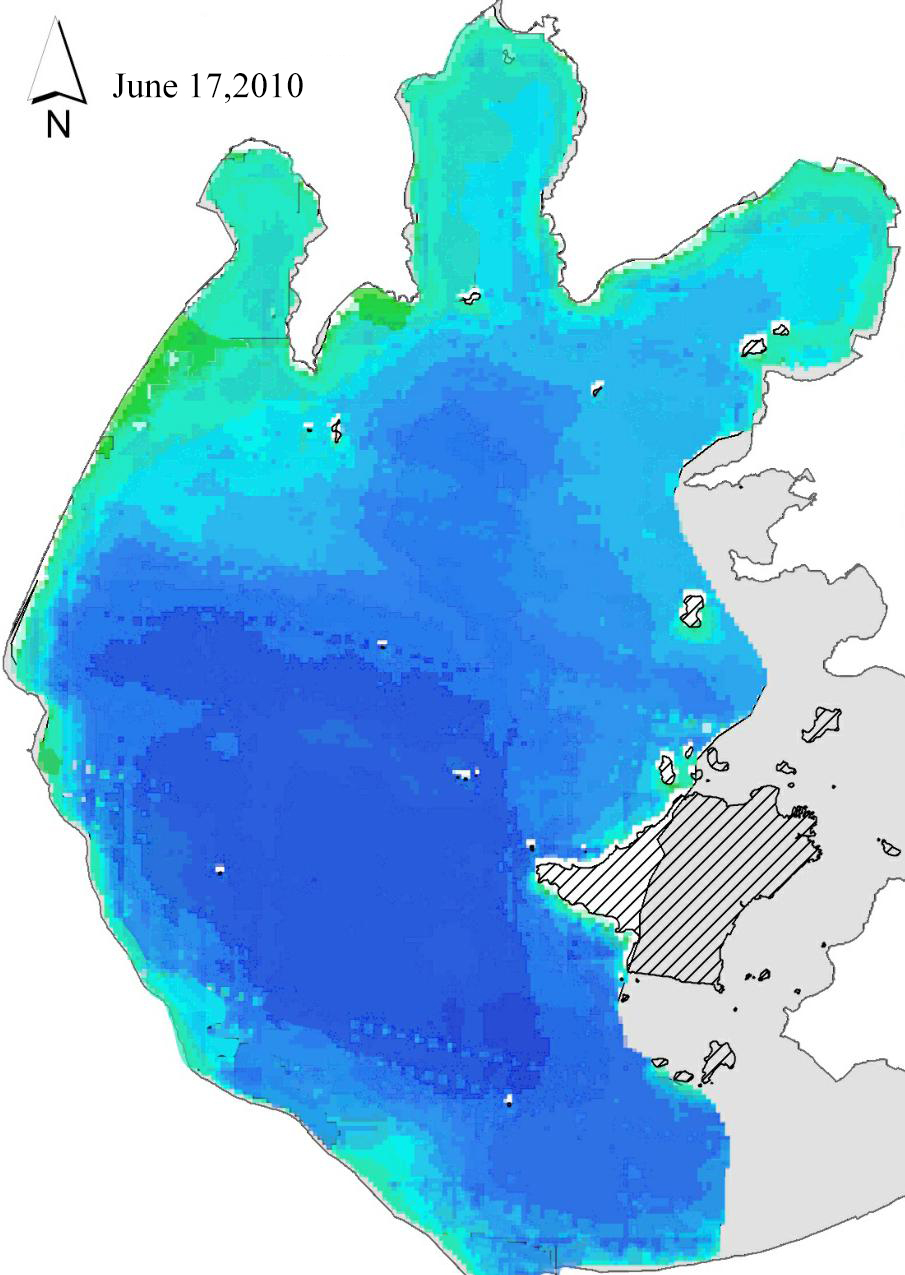

Supplement: Supplemental Information 10 — The data are remote sensing images of chlorophyll a concentration after data scale unification, remote sensing image repair, and time series filling. Remote sensing images of 30 consecutive moments were used as input to the 3D-GAN model. [file peerj-cs-09-1292-s010.zip › 201006170245.jpg]

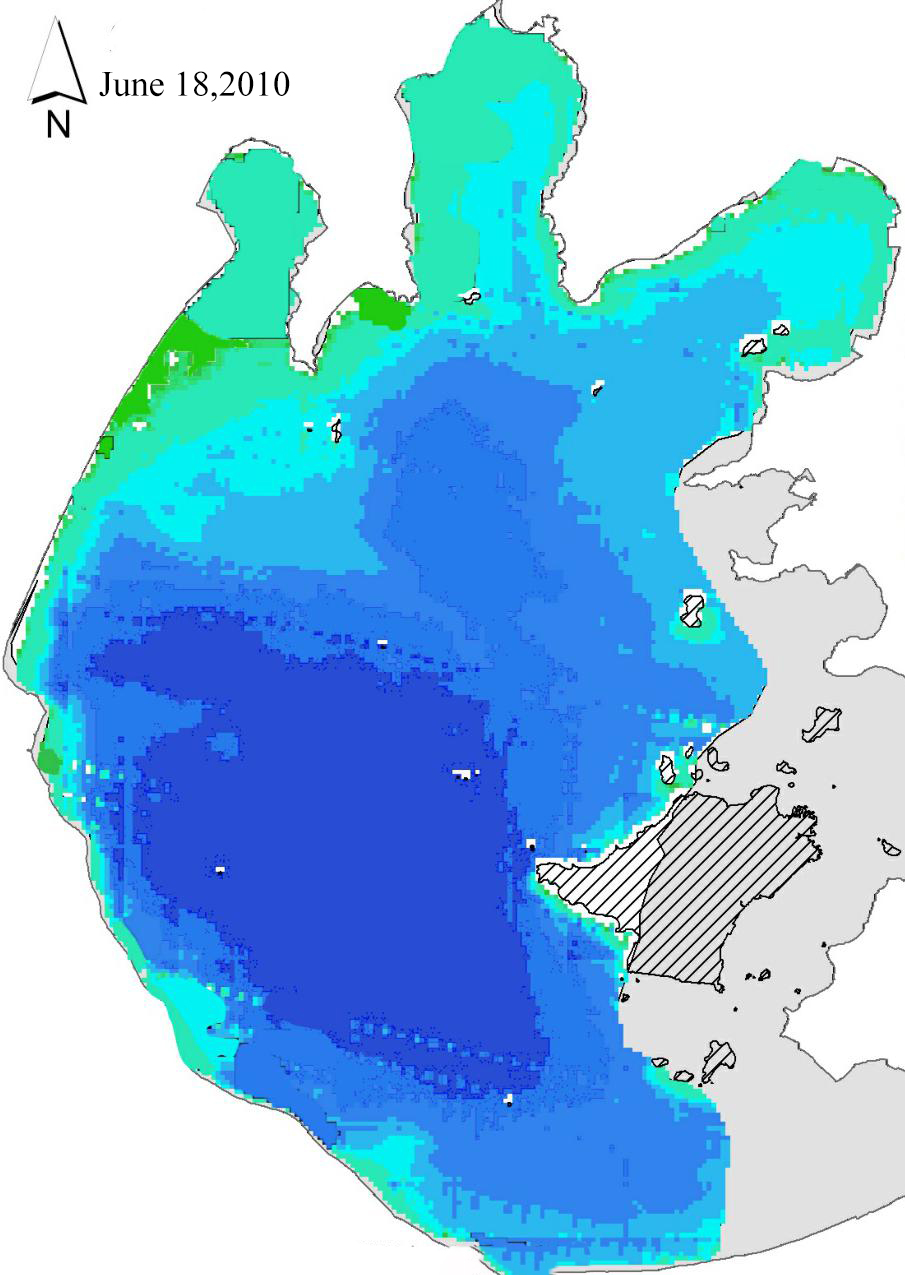

Supplement: Supplemental Information 10 — The data are remote sensing images of chlorophyll a concentration after data scale unification, remote sensing image repair, and time series filling. Remote sensing images of 30 consecutive moments were used as input to the 3D-GAN model. [file peerj-cs-09-1292-s010.zip › 201006180245.jpg]

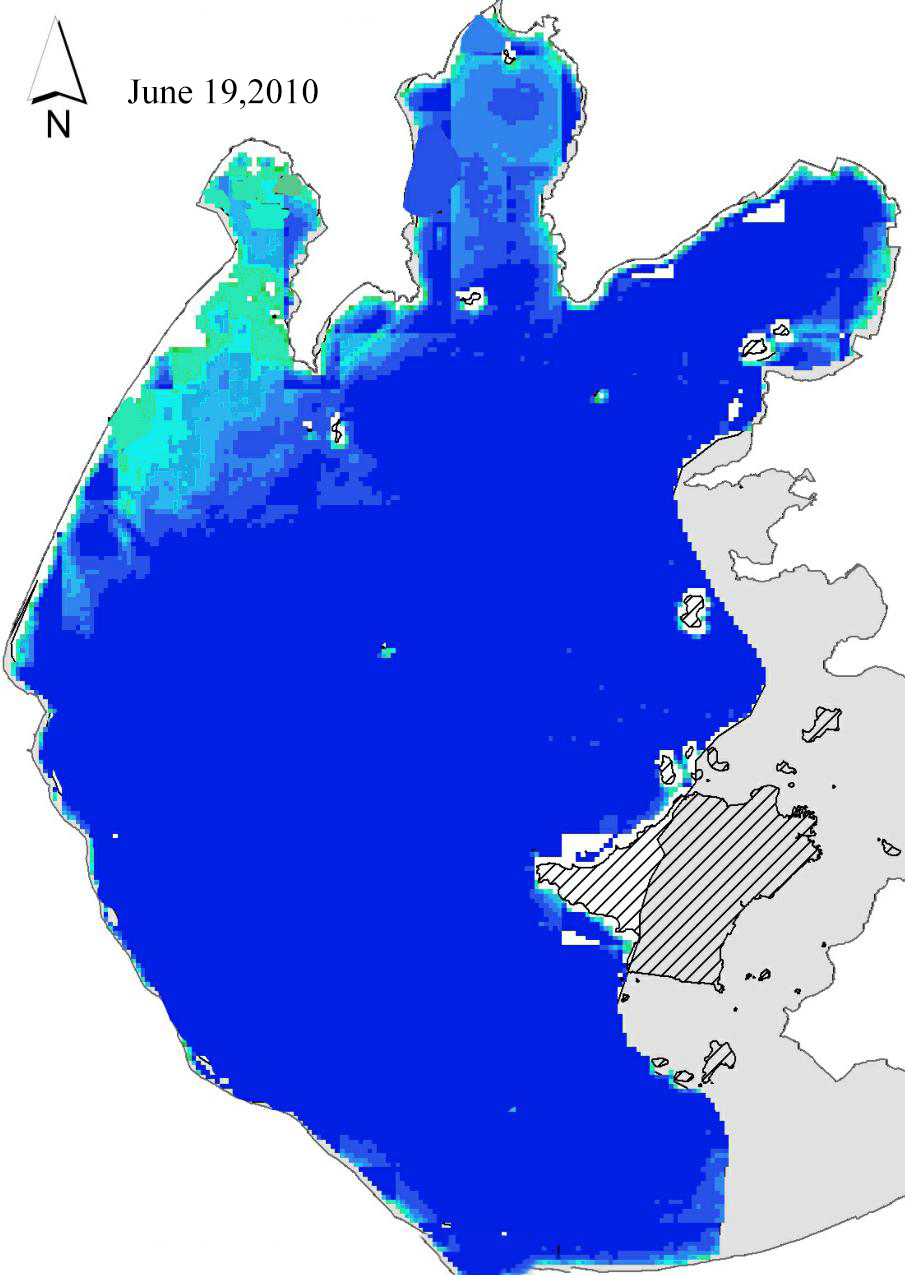

Supplement: Supplemental Information 10 — The data are remote sensing images of chlorophyll a concentration after data scale unification, remote sensing image repair, and time series filling. Remote sensing images of 30 consecutive moments were used as input to the 3D-GAN model. [file peerj-cs-09-1292-s010.zip › 201006190245.jpg]

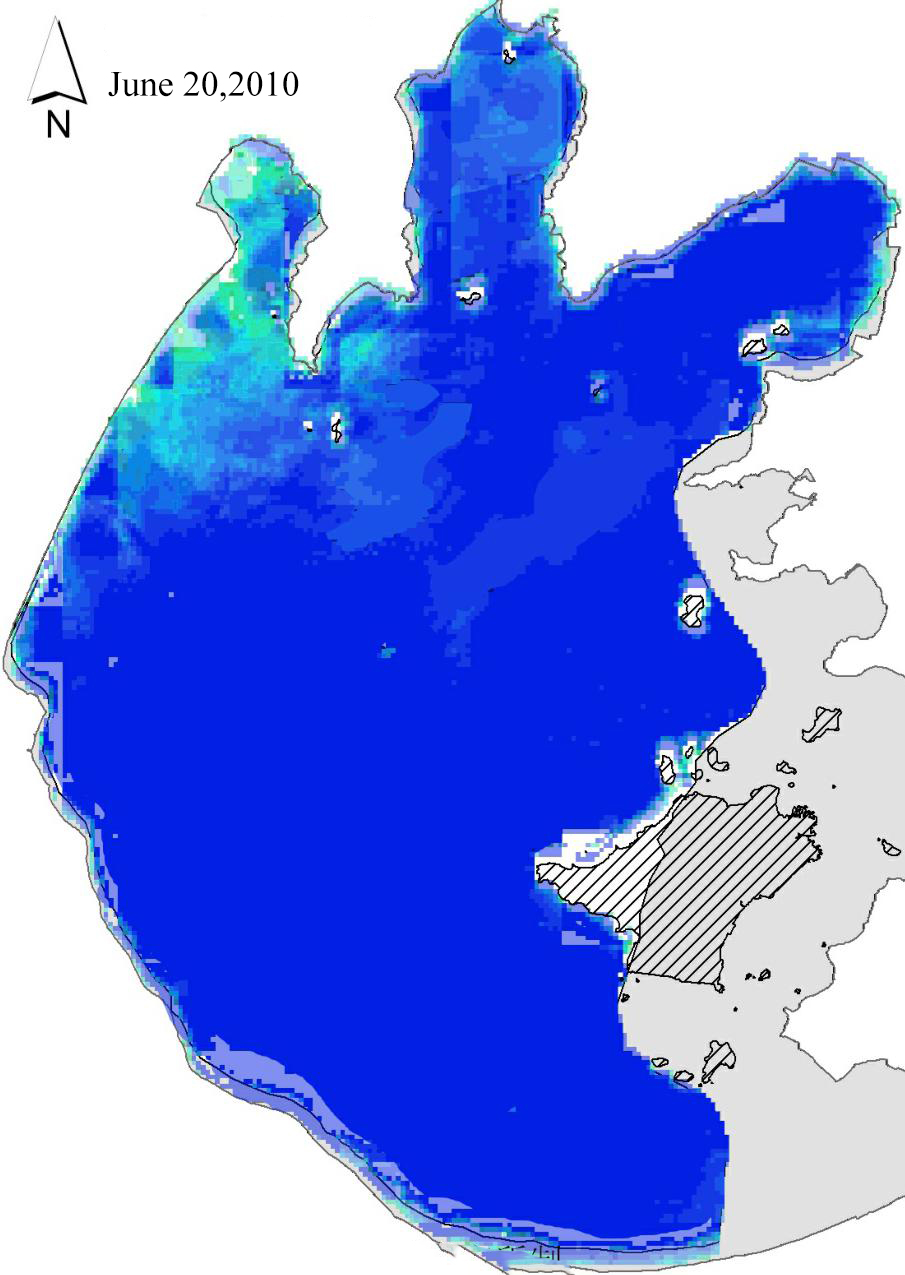

Supplement: Supplemental Information 10 — The data are remote sensing images of chlorophyll a concentration after data scale unification, remote sensing image repair, and time series filling. Remote sensing images of 30 consecutive moments were used as input to the 3D-GAN model. [file peerj-cs-09-1292-s010.zip › 201006200245.jpg]

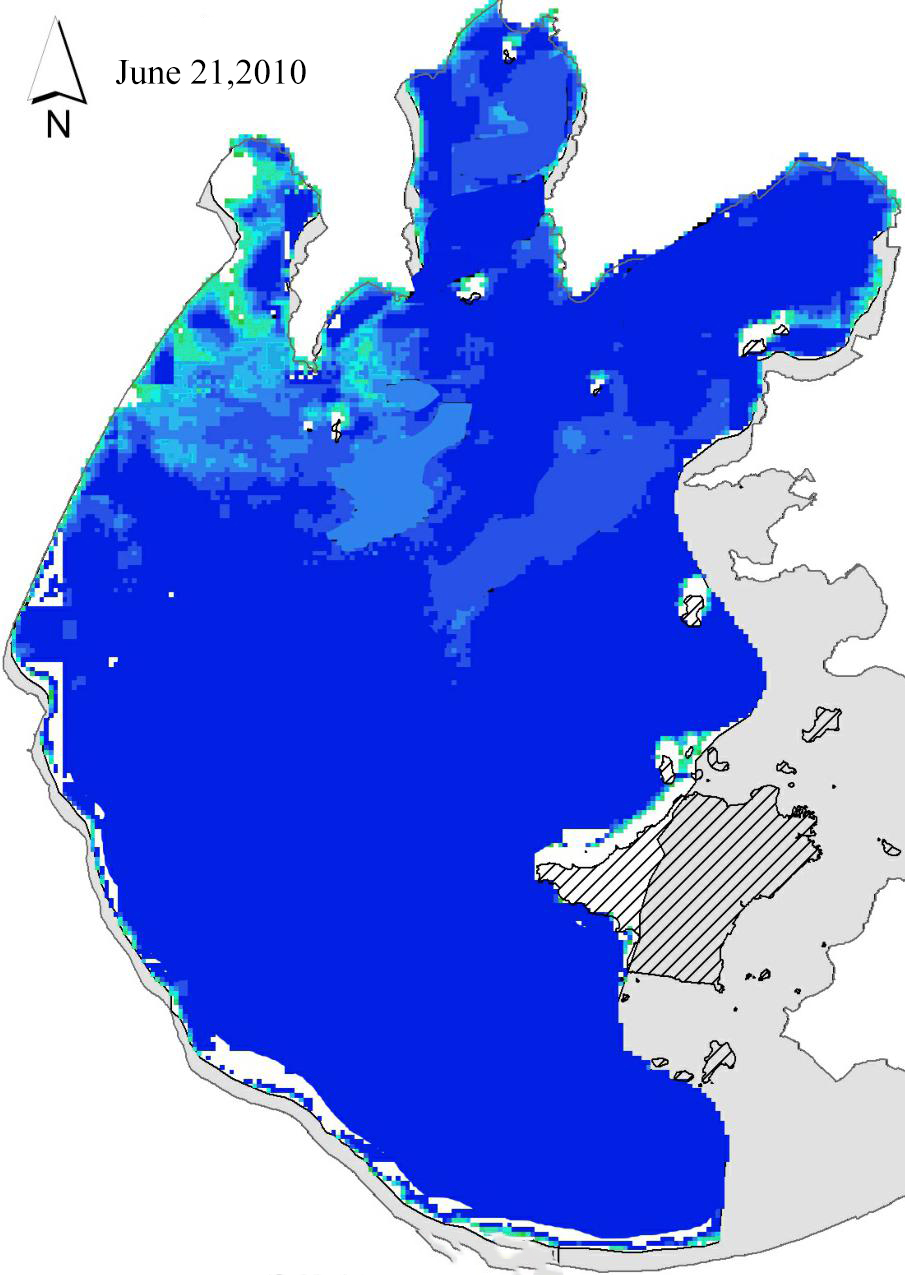

Supplement: Supplemental Information 10 — The data are remote sensing images of chlorophyll a concentration after data scale unification, remote sensing image repair, and time series filling. Remote sensing images of 30 consecutive moments were used as input to the 3D-GAN model. [file peerj-cs-09-1292-s010.zip › 201006210245.jpg]

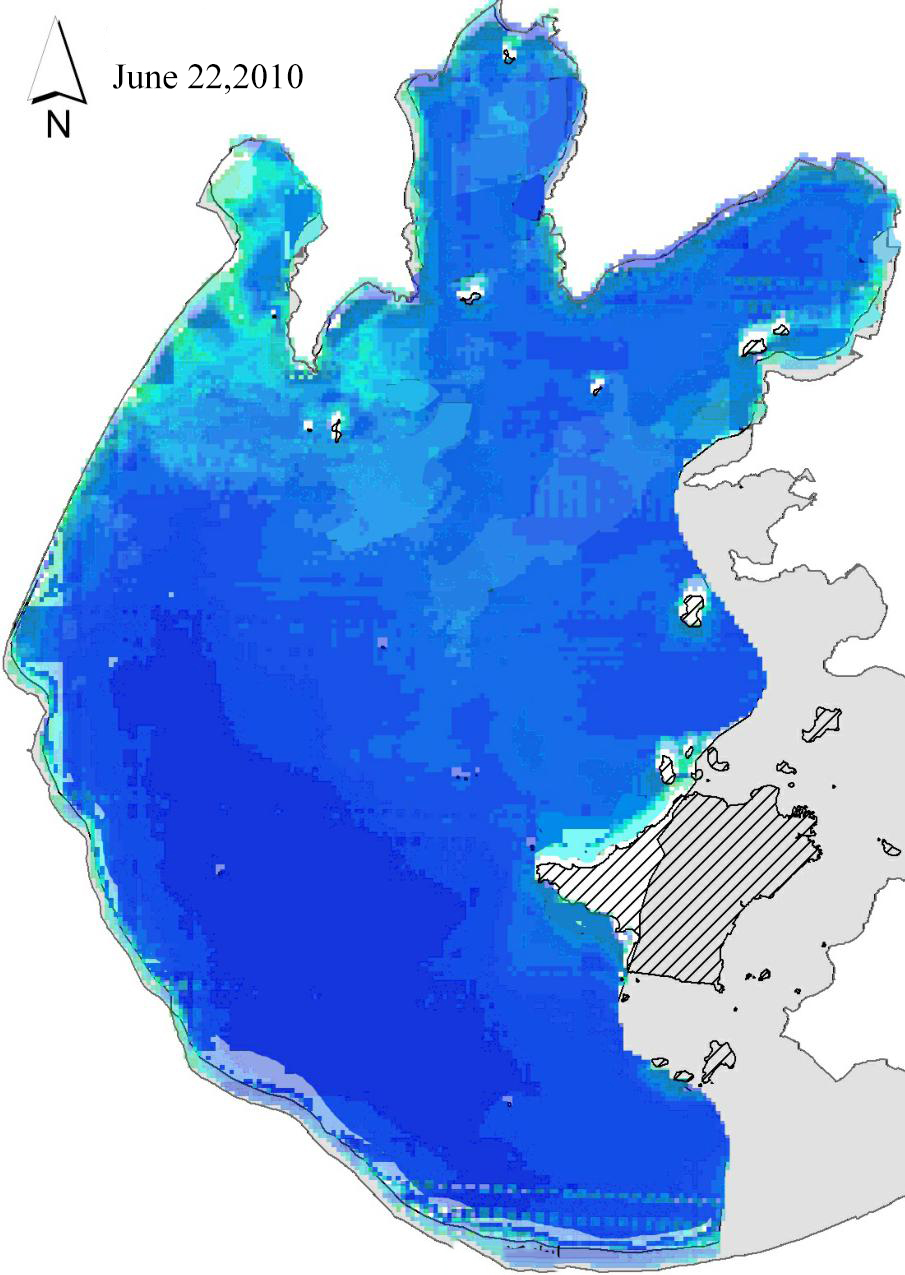

Supplement: Supplemental Information 10 — The data are remote sensing images of chlorophyll a concentration after data scale unification, remote sensing image repair, and time series filling. Remote sensing images of 30 consecutive moments were used as input to the 3D-GAN model. [file peerj-cs-09-1292-s010.zip › 201006220245.jpg]

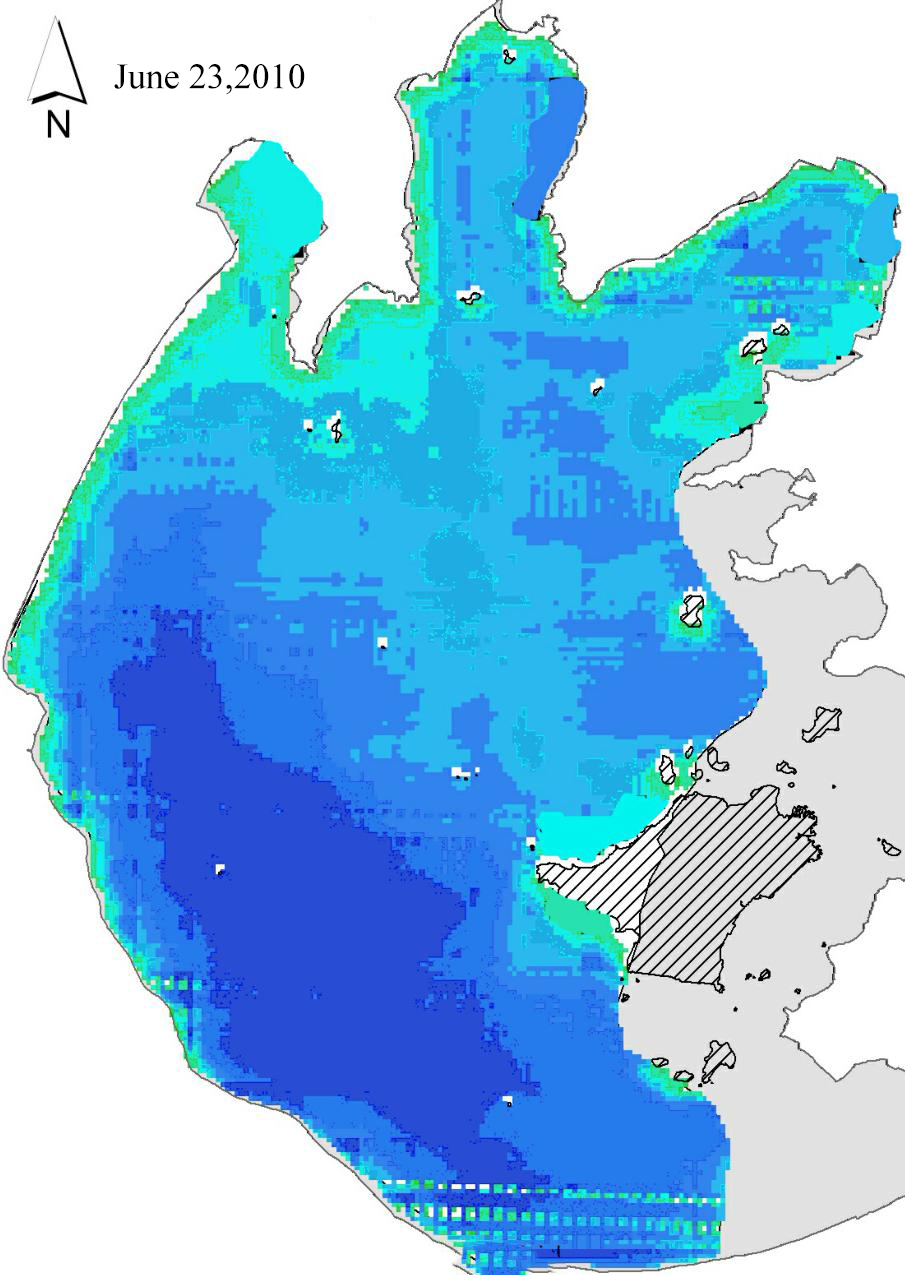

Supplement: Supplemental Information 10 — The data are remote sensing images of chlorophyll a concentration after data scale unification, remote sensing image repair, and time series filling. Remote sensing images of 30 consecutive moments were used as input to the 3D-GAN model. [file peerj-cs-09-1292-s010.zip › 201006230245.jpg]
