# Supplementary figures and images for: Remote sensing image analysis and prediction based on improved Pix2Pix model for water environment protection of smart cities (part 5 of 6)
Source: PeerJ Comput Sci. 2023 Apr 26;9:e1292. doi: 10.7717/peerj-cs.1292 (PMC10280440; doi:10.7717/peerj-cs.1292)

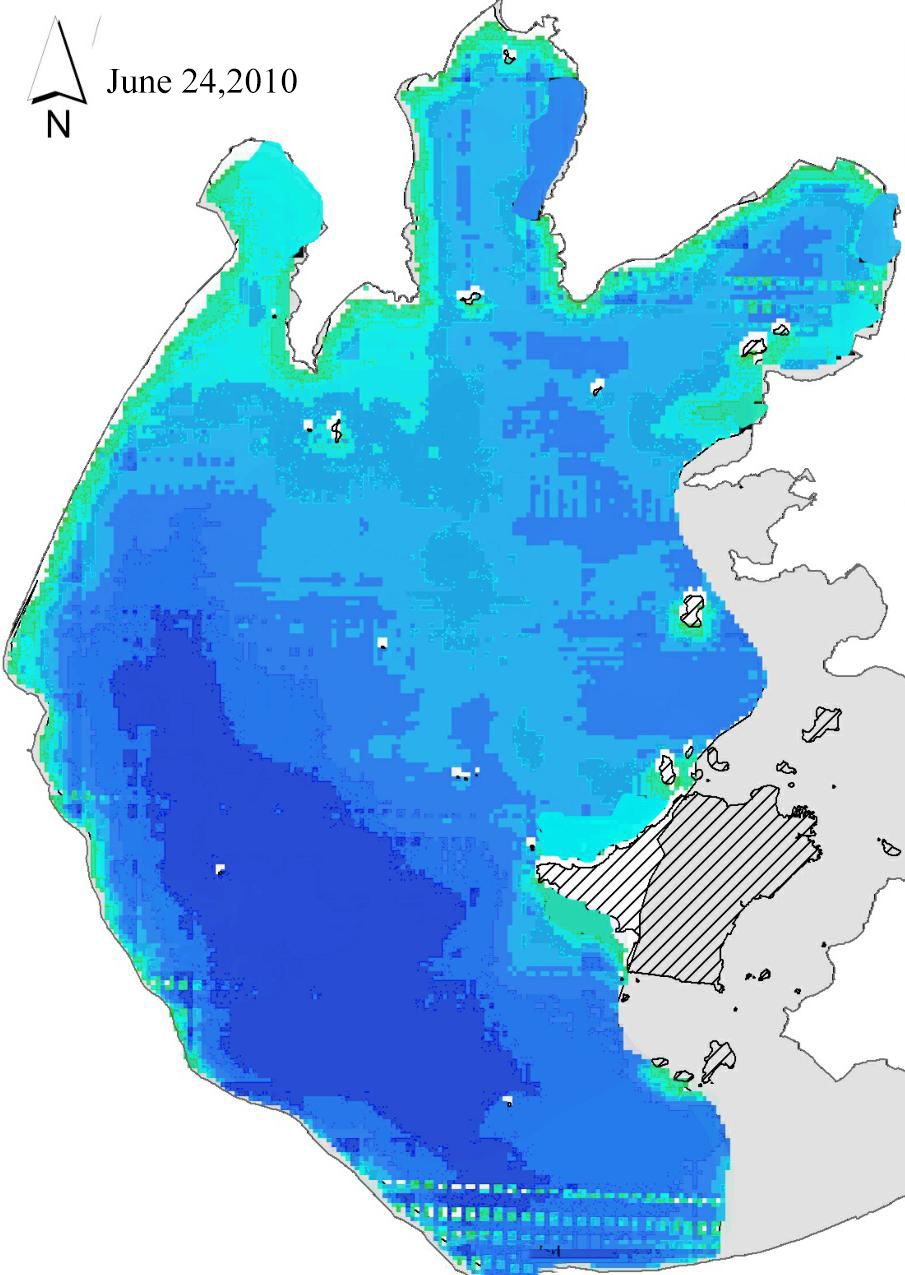

Supplement: Supplemental Information 10 — The data are remote sensing images of chlorophyll a concentration after data scale unification, remote sensing image repair, and time series filling. Remote sensing images of 30 consecutive moments were used as input to the 3D-GAN model. [file peerj-cs-09-1292-s010.zip › 201006240245.jpg]

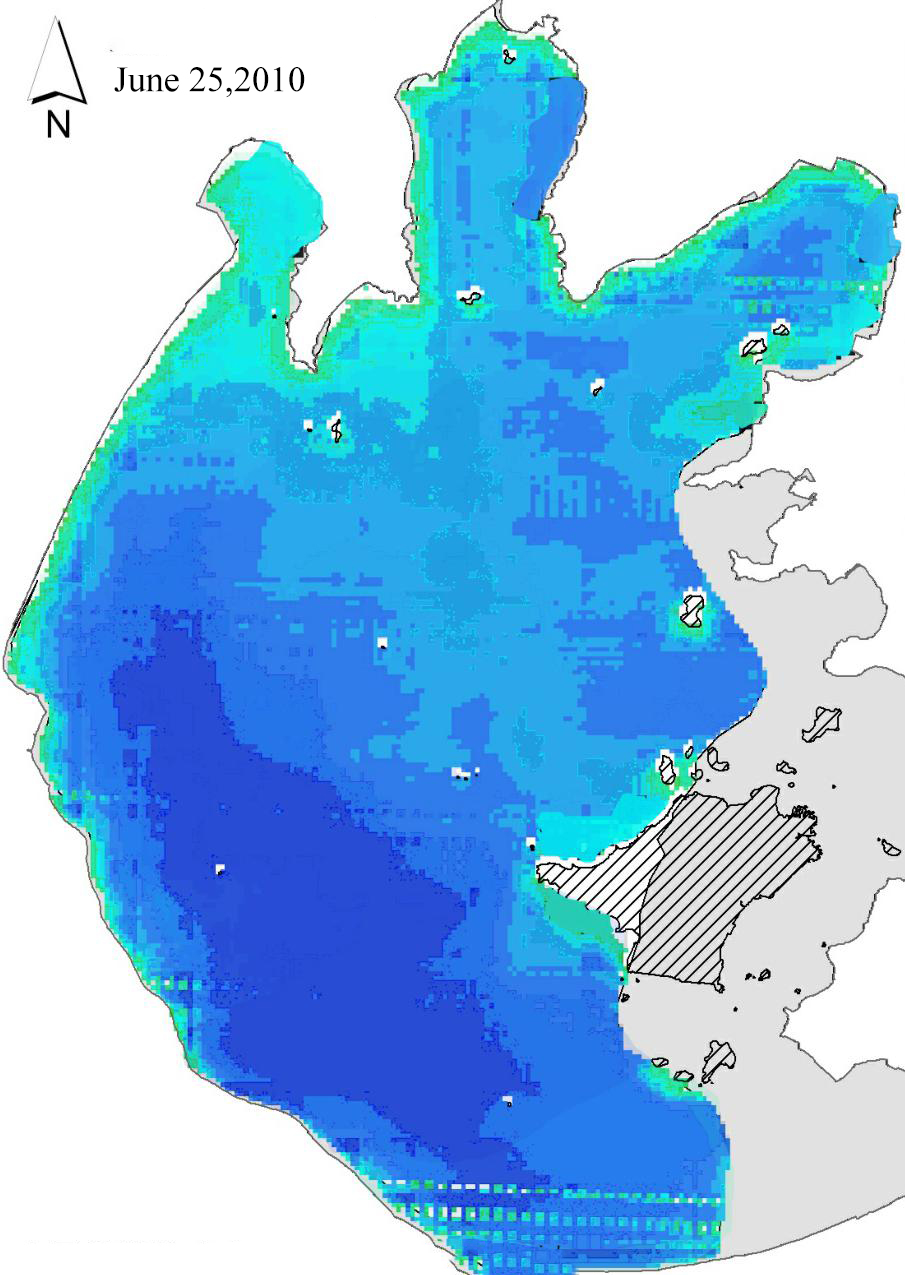

Supplement: Supplemental Information 10 — The data are remote sensing images of chlorophyll a concentration after data scale unification, remote sensing image repair, and time series filling. Remote sensing images of 30 consecutive moments were used as input to the 3D-GAN model. [file peerj-cs-09-1292-s010.zip › 201006250245.jpg]

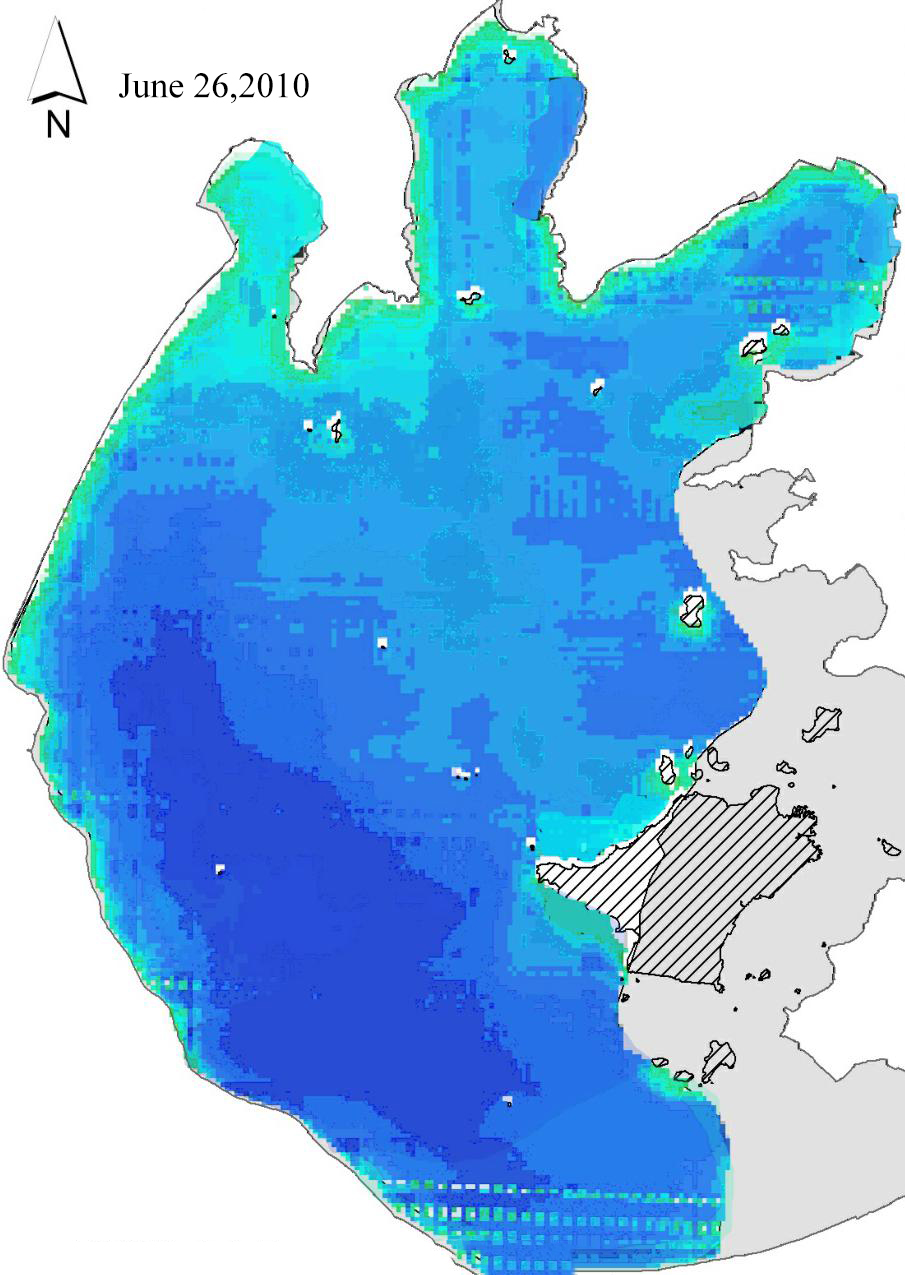

Supplement: Supplemental Information 10 — The data are remote sensing images of chlorophyll a concentration after data scale unification, remote sensing image repair, and time series filling. Remote sensing images of 30 consecutive moments were used as input to the 3D-GAN model. [file peerj-cs-09-1292-s010.zip › 201006260245.jpg]

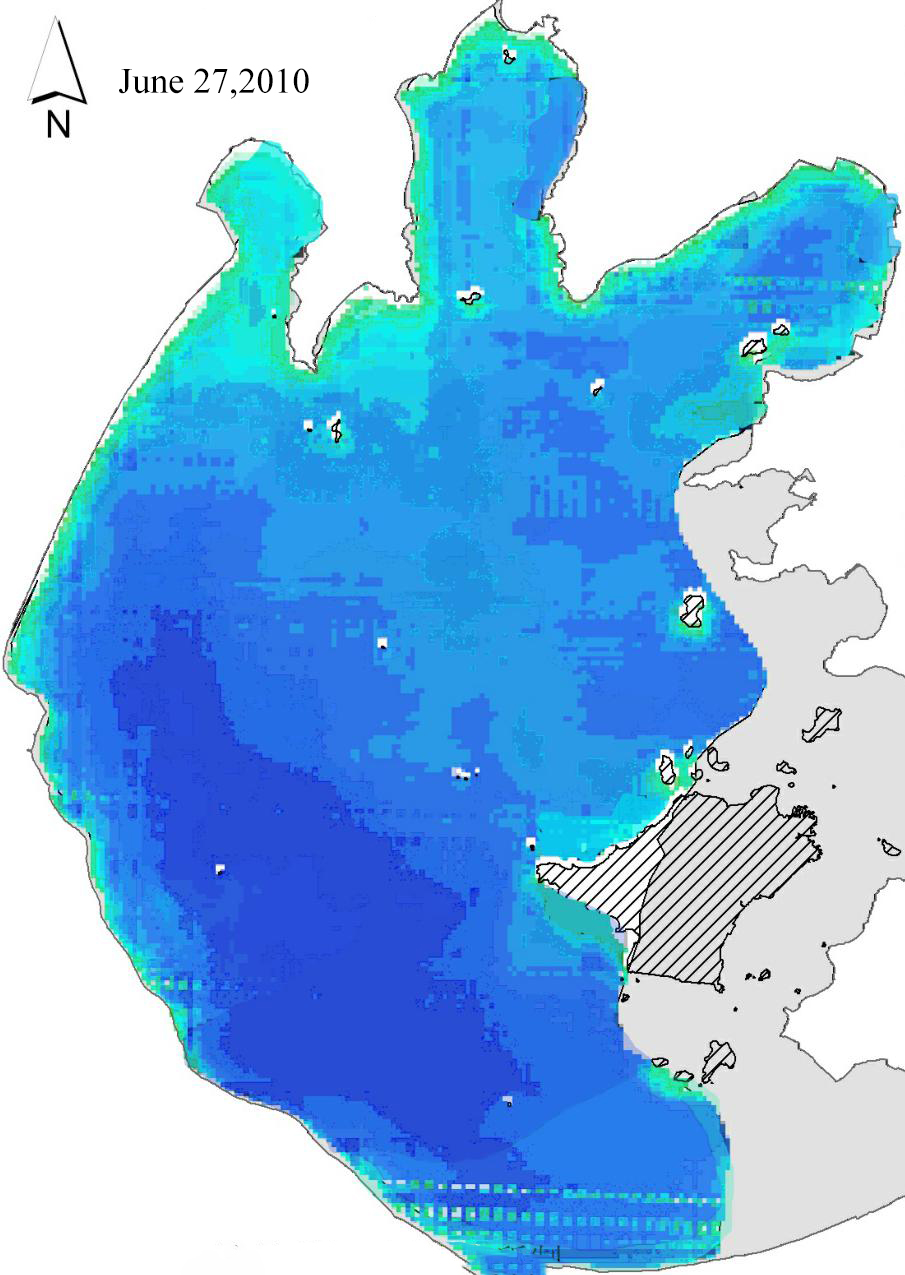

Supplement: Supplemental Information 10 — The data are remote sensing images of chlorophyll a concentration after data scale unification, remote sensing image repair, and time series filling. Remote sensing images of 30 consecutive moments were used as input to the 3D-GAN model. [file peerj-cs-09-1292-s010.zip › 201006270245.jpg]

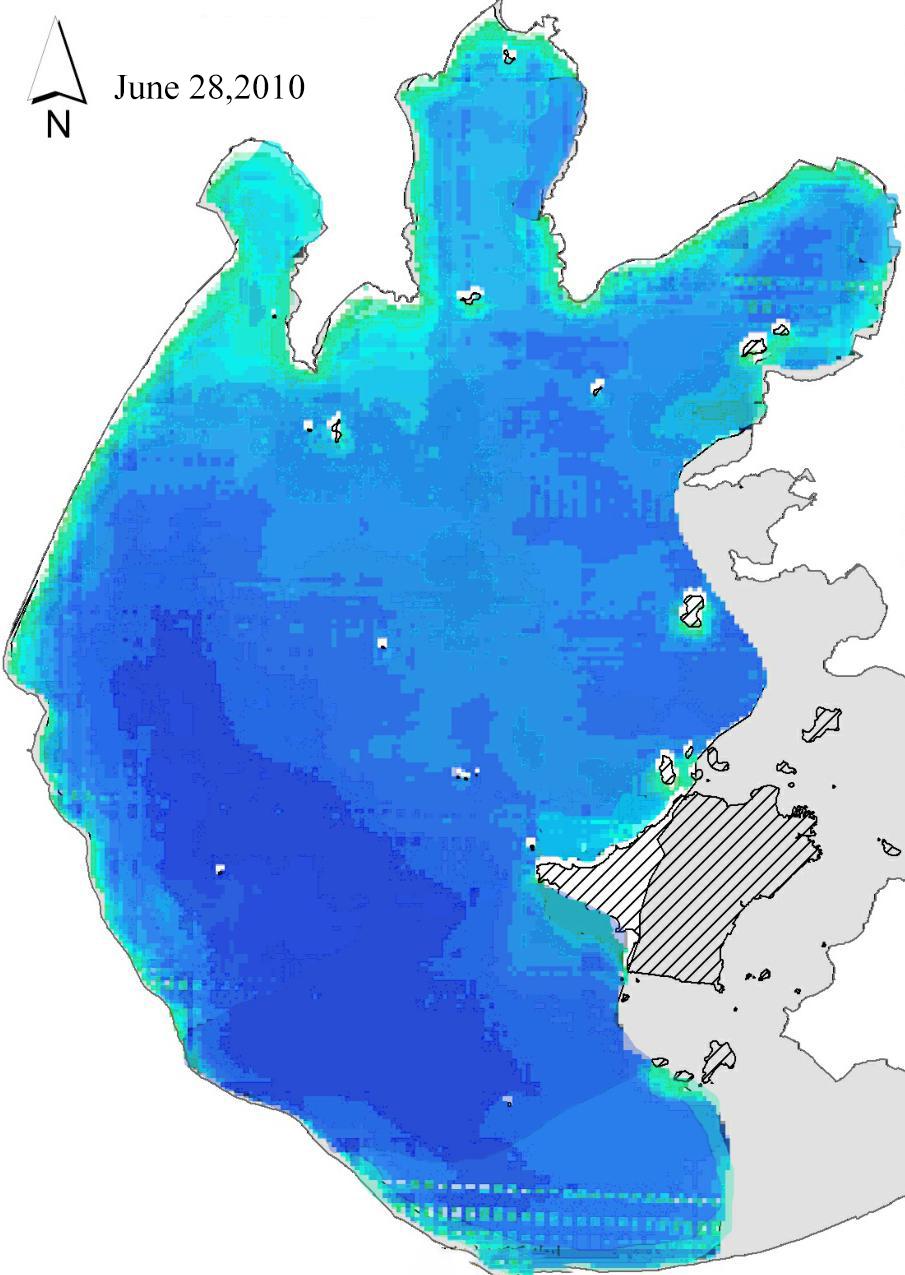

Supplement: Supplemental Information 10 — The data are remote sensing images of chlorophyll a concentration after data scale unification, remote sensing image repair, and time series filling. Remote sensing images of 30 consecutive moments were used as input to the 3D-GAN model. [file peerj-cs-09-1292-s010.zip › 201006280245.jpg]

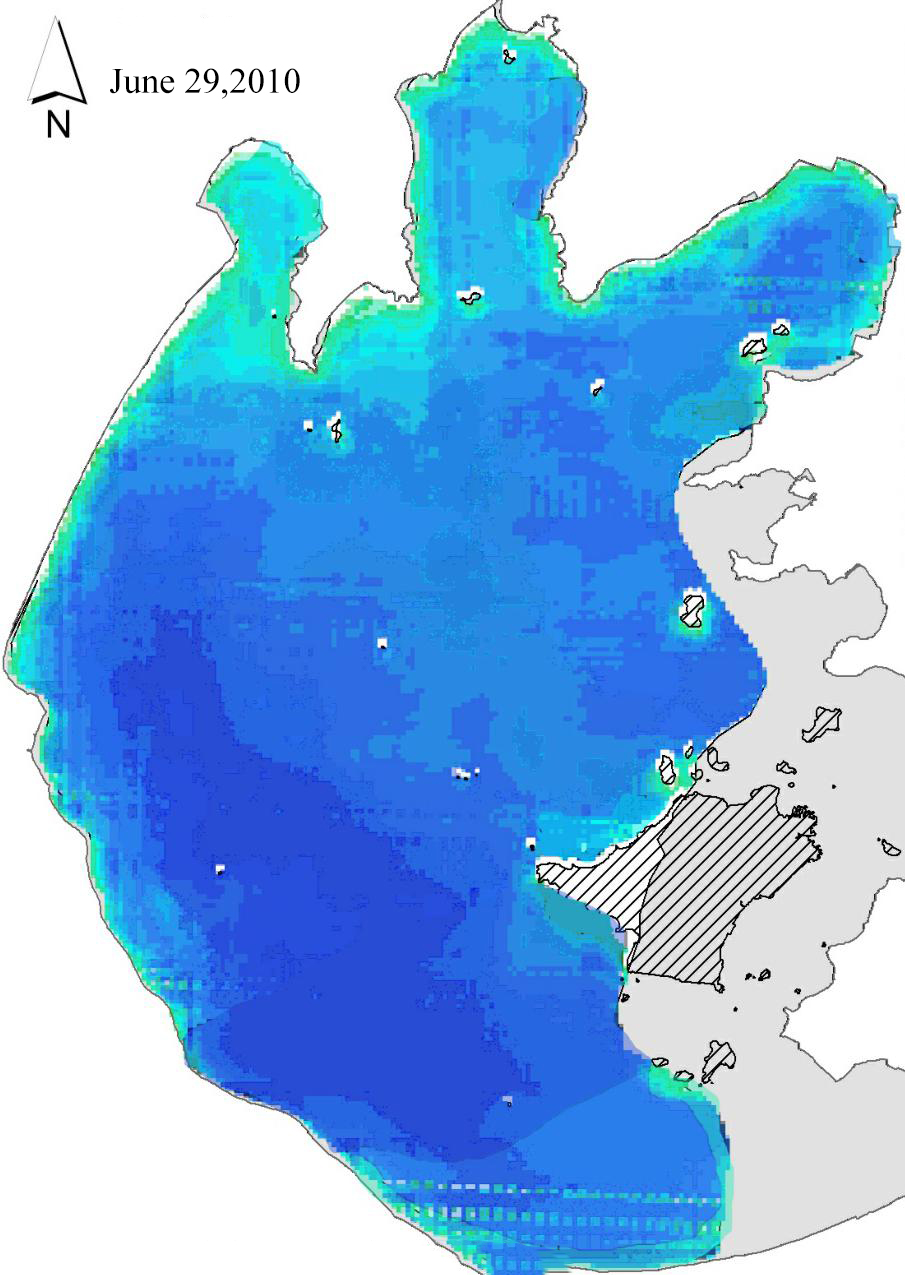

Supplement: Supplemental Information 10 — The data are remote sensing images of chlorophyll a concentration after data scale unification, remote sensing image repair, and time series filling. Remote sensing images of 30 consecutive moments were used as input to the 3D-GAN model. [file peerj-cs-09-1292-s010.zip › 201006290245.jpg]

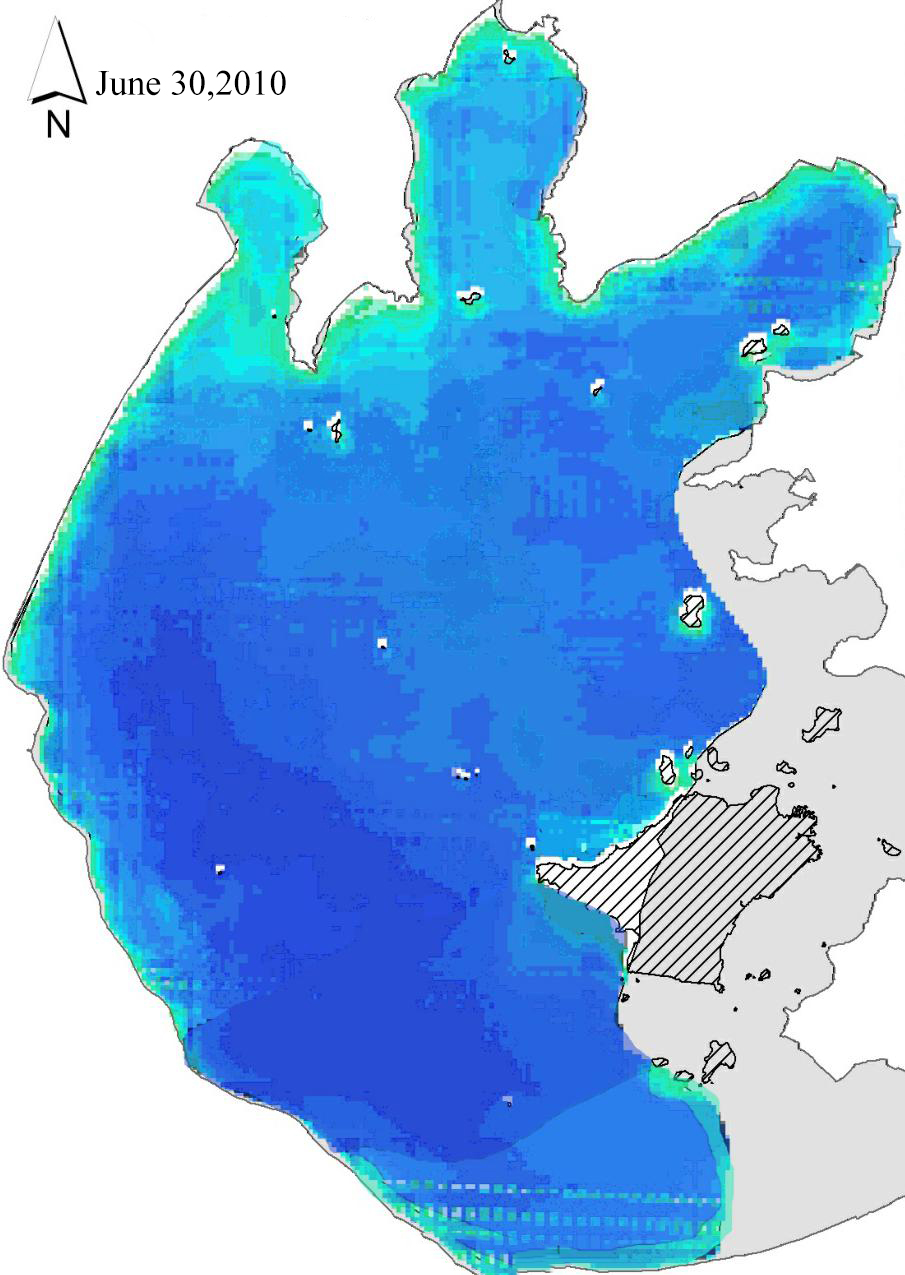

Supplement: Supplemental Information 10 — The data are remote sensing images of chlorophyll a concentration after data scale unification, remote sensing image repair, and time series filling. Remote sensing images of 30 consecutive moments were used as input to the 3D-GAN model. [file peerj-cs-09-1292-s010.zip › 201006300245.jpg]

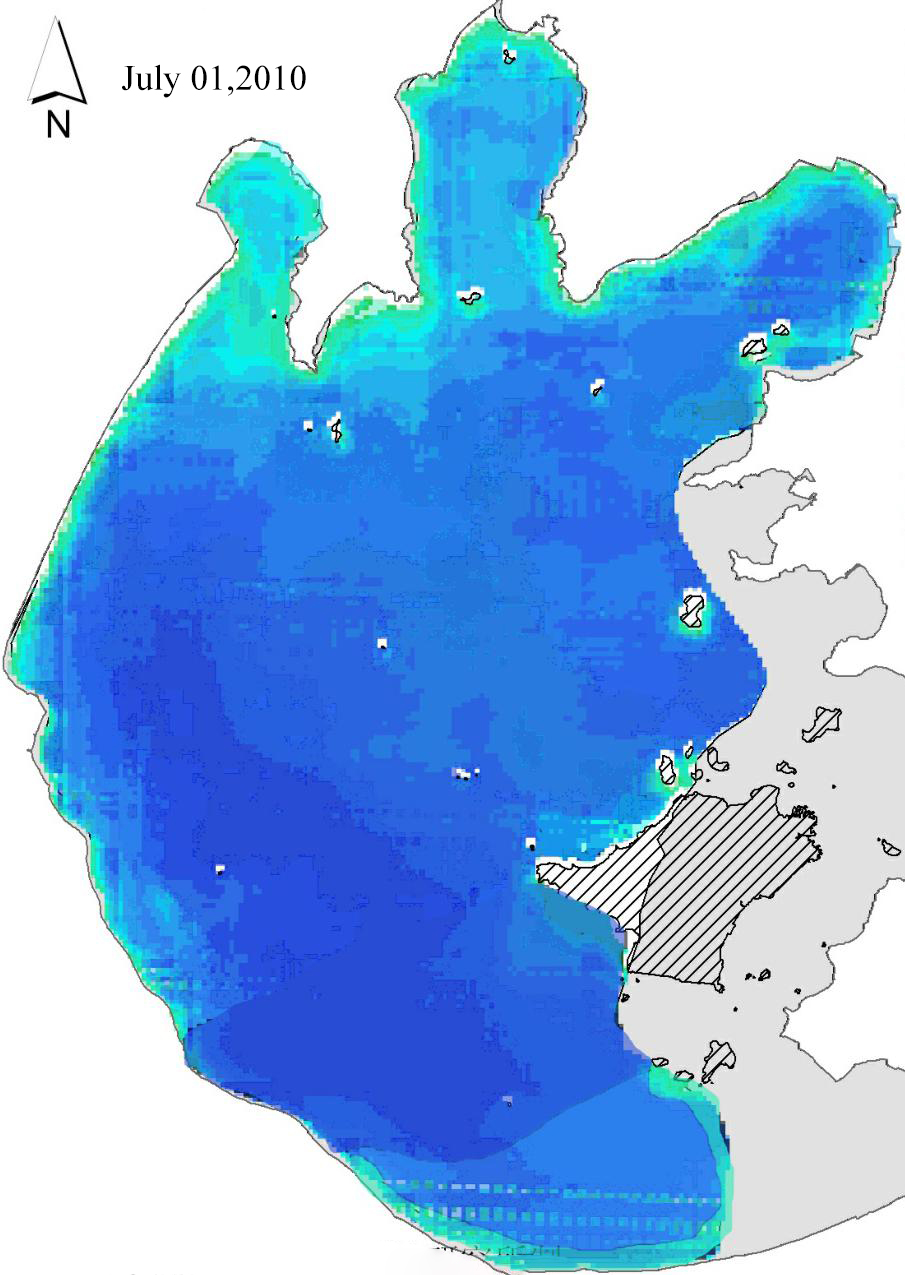

Supplement: Supplemental Information 10 — The data are remote sensing images of chlorophyll a concentration after data scale unification, remote sensing image repair, and time series filling. Remote sensing images of 30 consecutive moments were used as input to the 3D-GAN model. [file peerj-cs-09-1292-s010.zip › 201007010245.jpg]

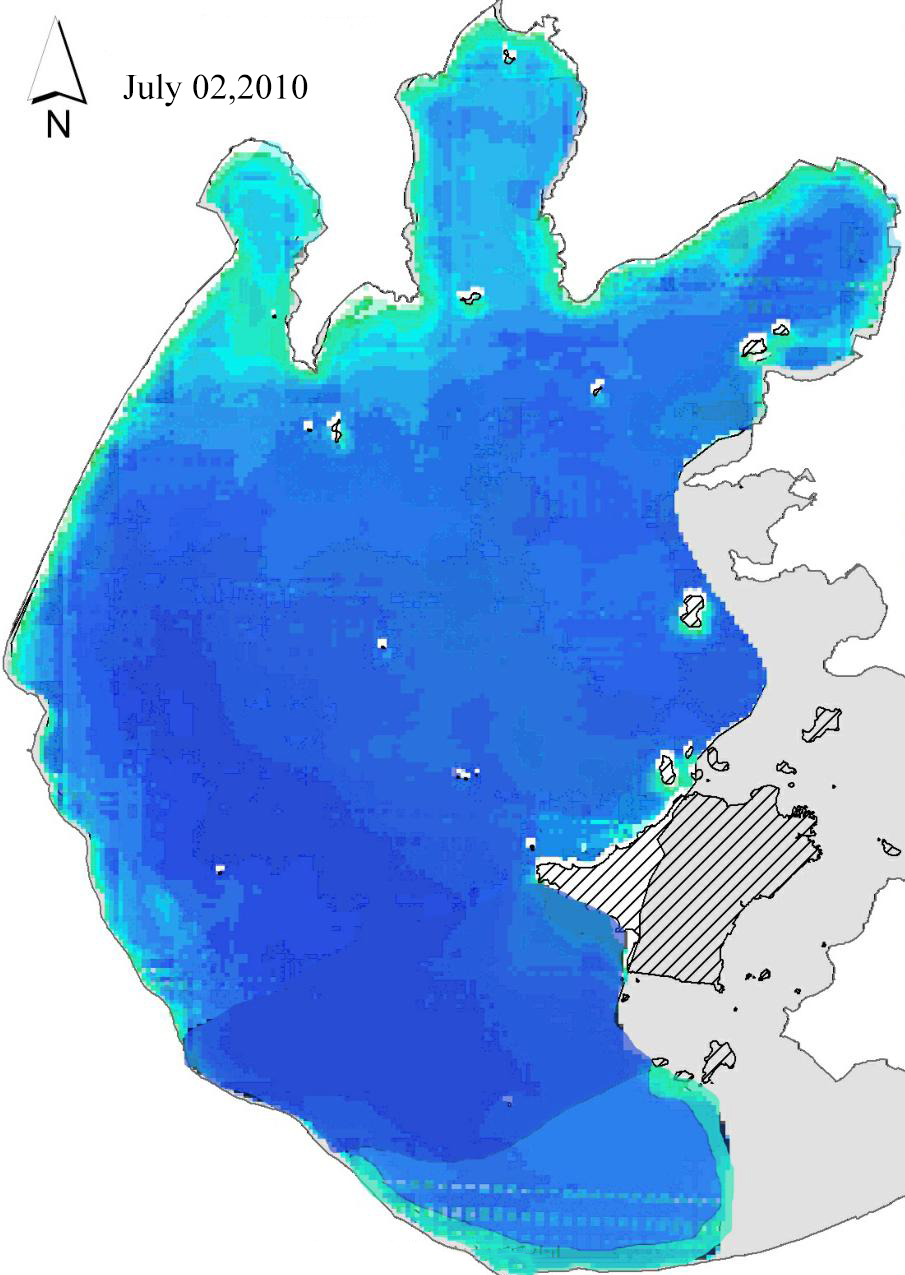

Supplement: Supplemental Information 10 — The data are remote sensing images of chlorophyll a concentration after data scale unification, remote sensing image repair, and time series filling. Remote sensing images of 30 consecutive moments were used as input to the 3D-GAN model. [file peerj-cs-09-1292-s010.zip › 201007020245.jpg]

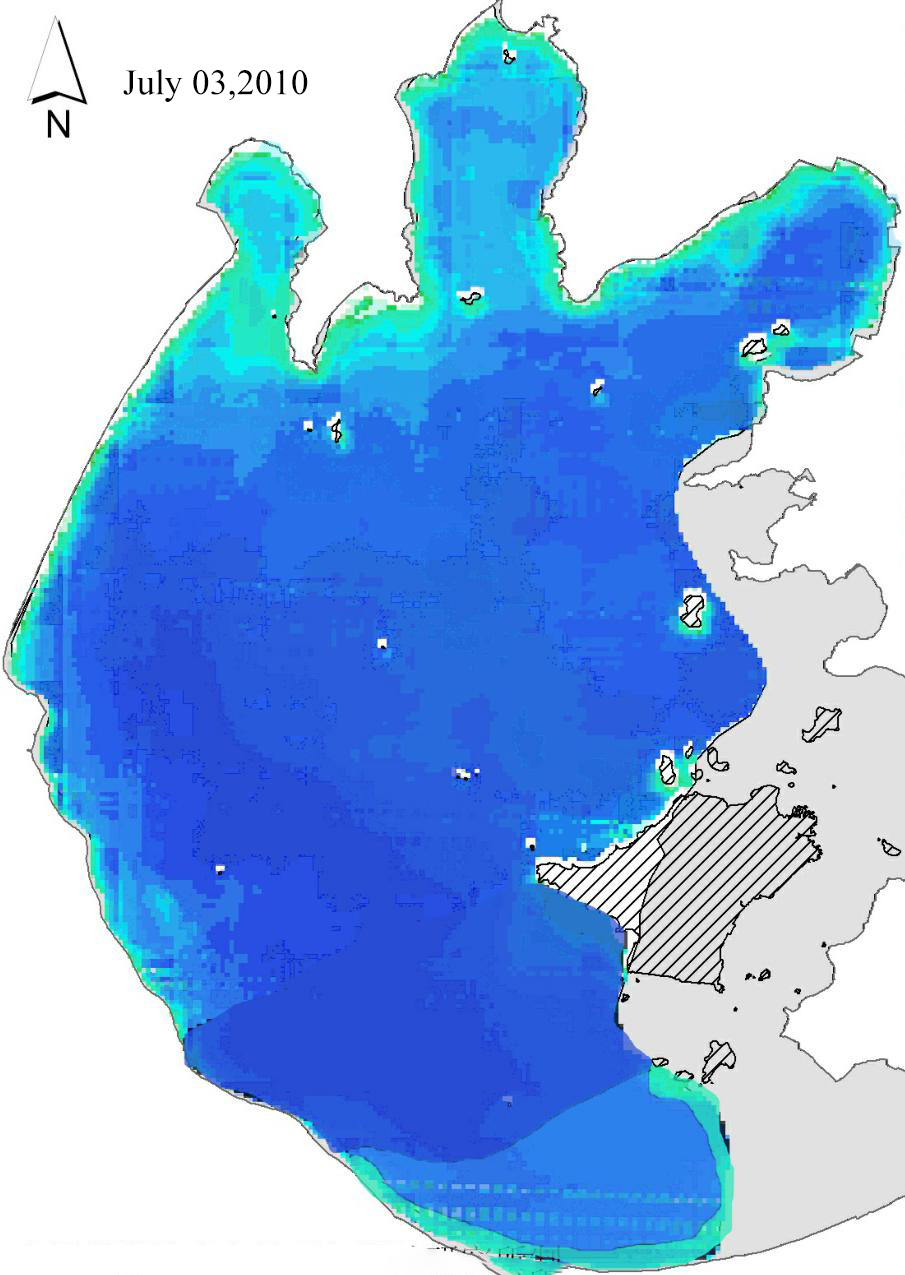

Supplement: Supplemental Information 10 — The data are remote sensing images of chlorophyll a concentration after data scale unification, remote sensing image repair, and time series filling. Remote sensing images of 30 consecutive moments were used as input to the 3D-GAN model. [file peerj-cs-09-1292-s010.zip › 201007030245.jpg]

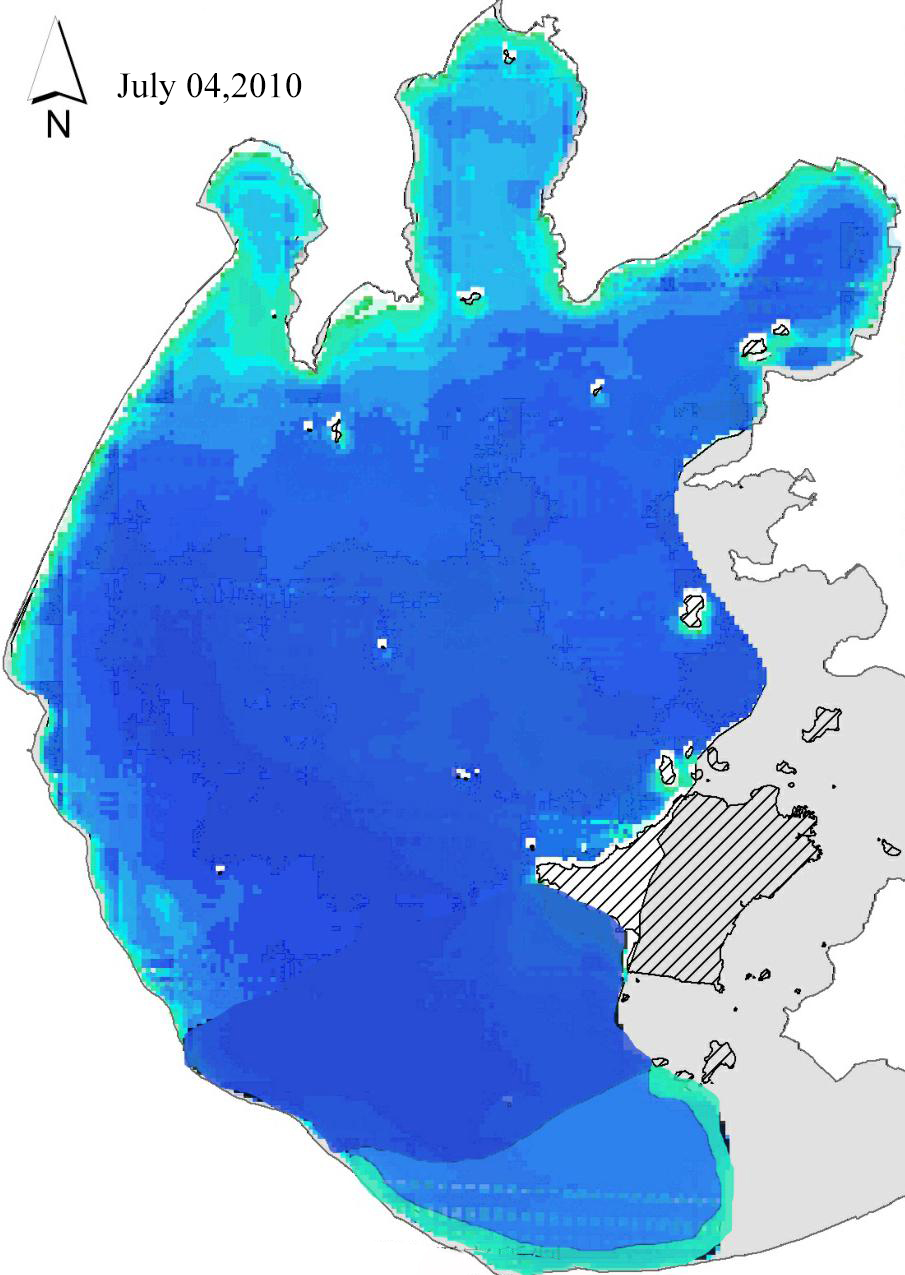

Supplement: Supplemental Information 10 — The data are remote sensing images of chlorophyll a concentration after data scale unification, remote sensing image repair, and time series filling. Remote sensing images of 30 consecutive moments were used as input to the 3D-GAN model. [file peerj-cs-09-1292-s010.zip › 201007040245.jpg]

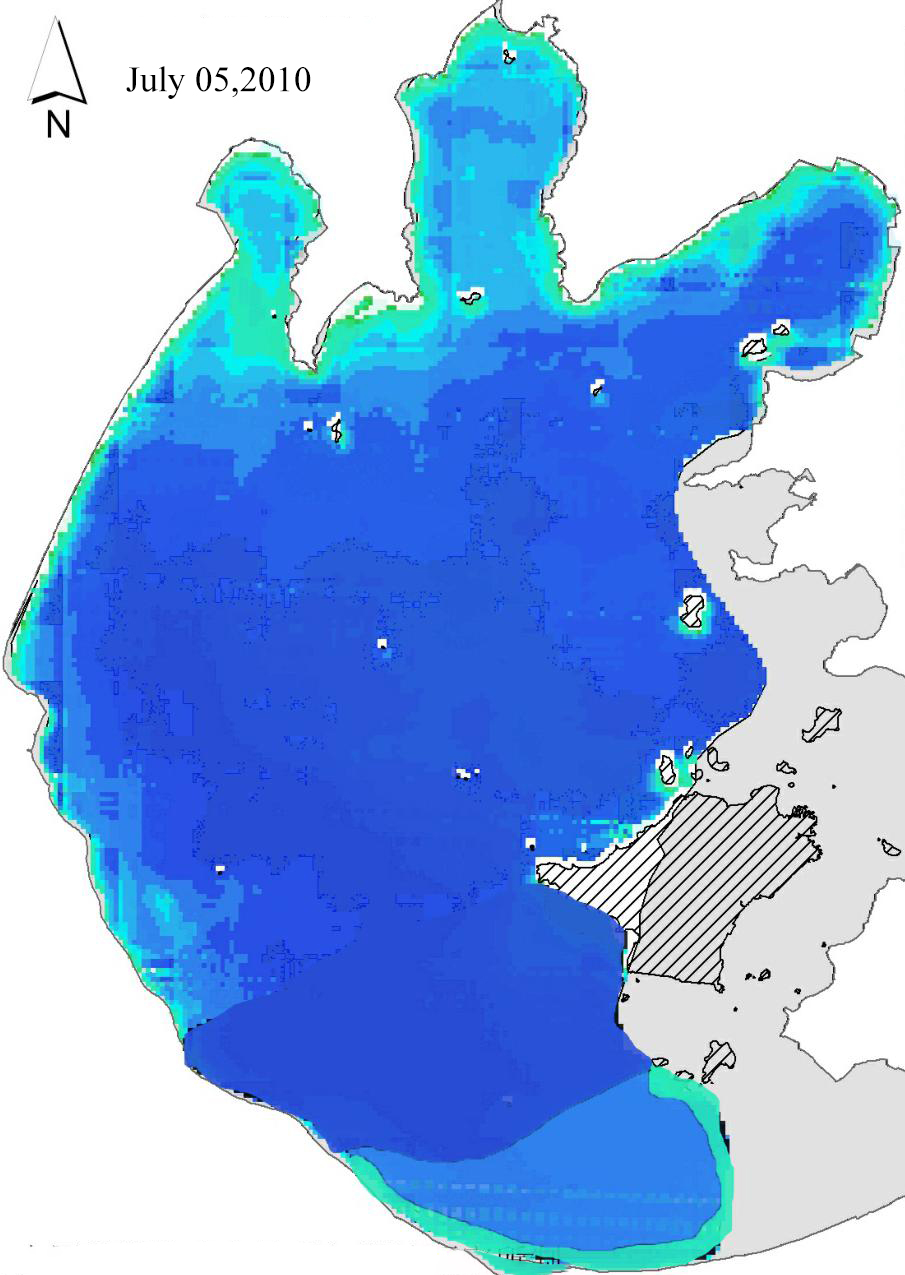

Supplement: Supplemental Information 10 — The data are remote sensing images of chlorophyll a concentration after data scale unification, remote sensing image repair, and time series filling. Remote sensing images of 30 consecutive moments were used as input to the 3D-GAN model. [file peerj-cs-09-1292-s010.zip › 201007050245.jpg]

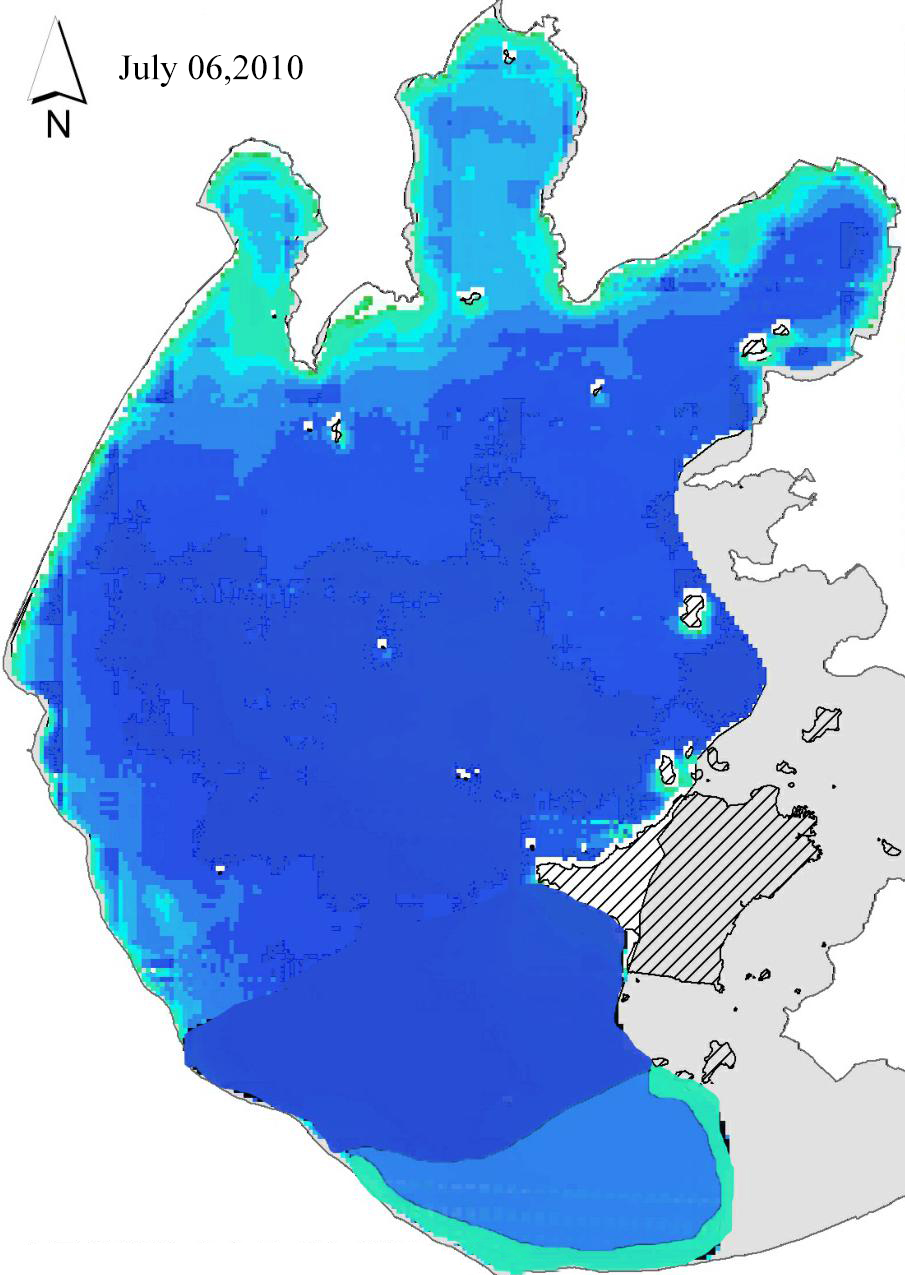

Supplement: Supplemental Information 10 — The data are remote sensing images of chlorophyll a concentration after data scale unification, remote sensing image repair, and time series filling. Remote sensing images of 30 consecutive moments were used as input to the 3D-GAN model. [file peerj-cs-09-1292-s010.zip › 201007060245.jpg]

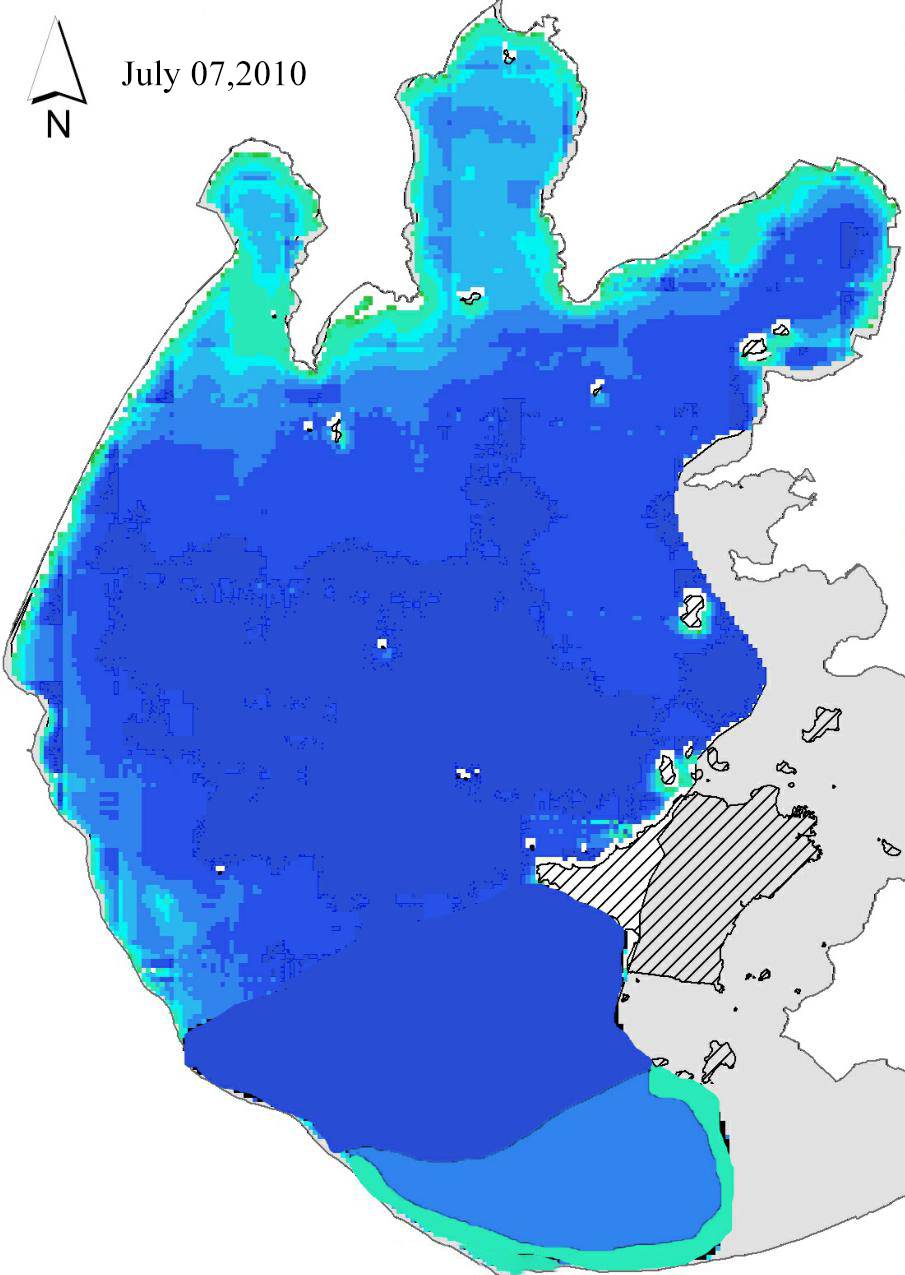

Supplement: Supplemental Information 10 — The data are remote sensing images of chlorophyll a concentration after data scale unification, remote sensing image repair, and time series filling. Remote sensing images of 30 consecutive moments were used as input to the 3D-GAN model. [file peerj-cs-09-1292-s010.zip › 201007070245.jpg]

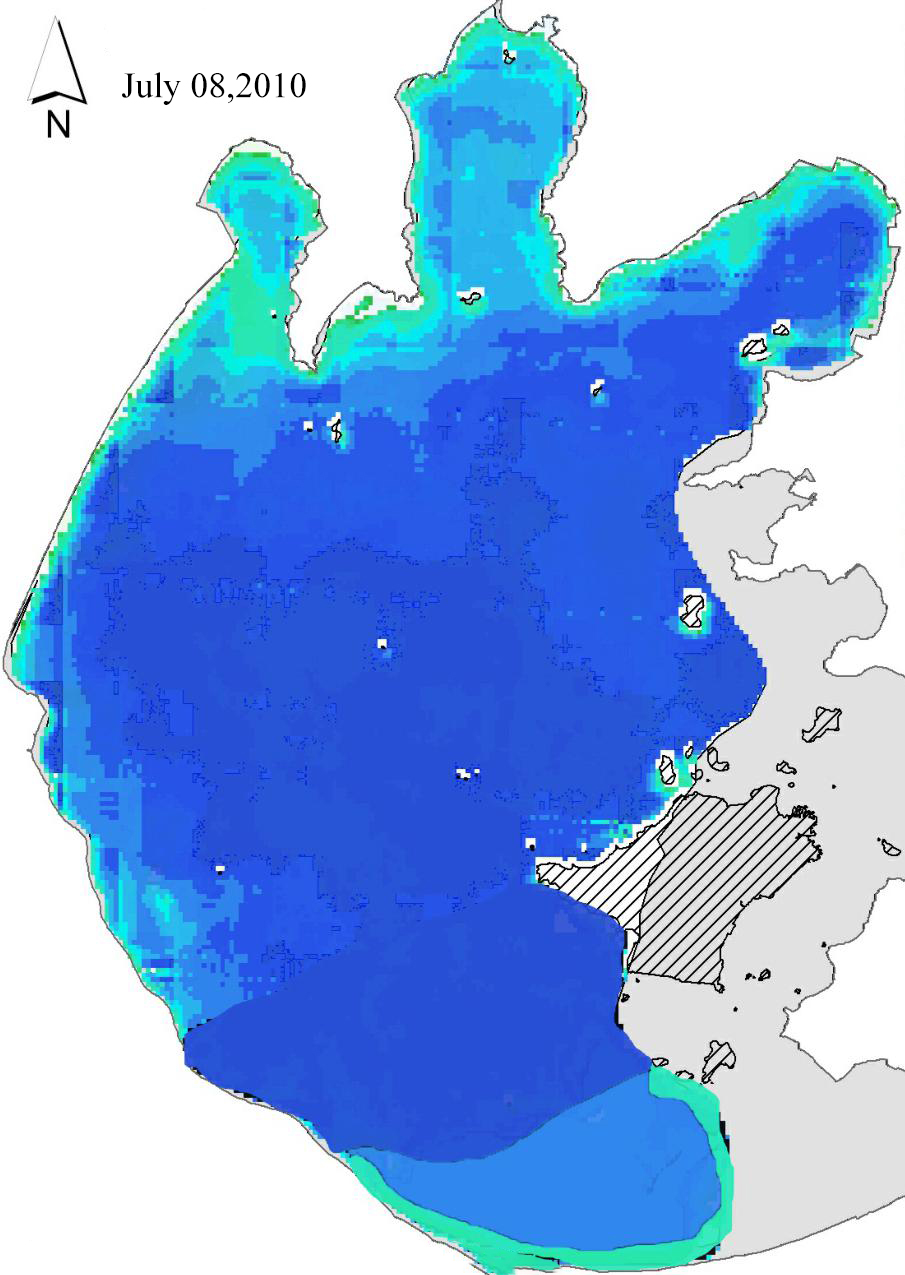

Supplement: Supplemental Information 10 — The data are remote sensing images of chlorophyll a concentration after data scale unification, remote sensing image repair, and time series filling. Remote sensing images of 30 consecutive moments were used as input to the 3D-GAN model. [file peerj-cs-09-1292-s010.zip › 201007080245.jpg]

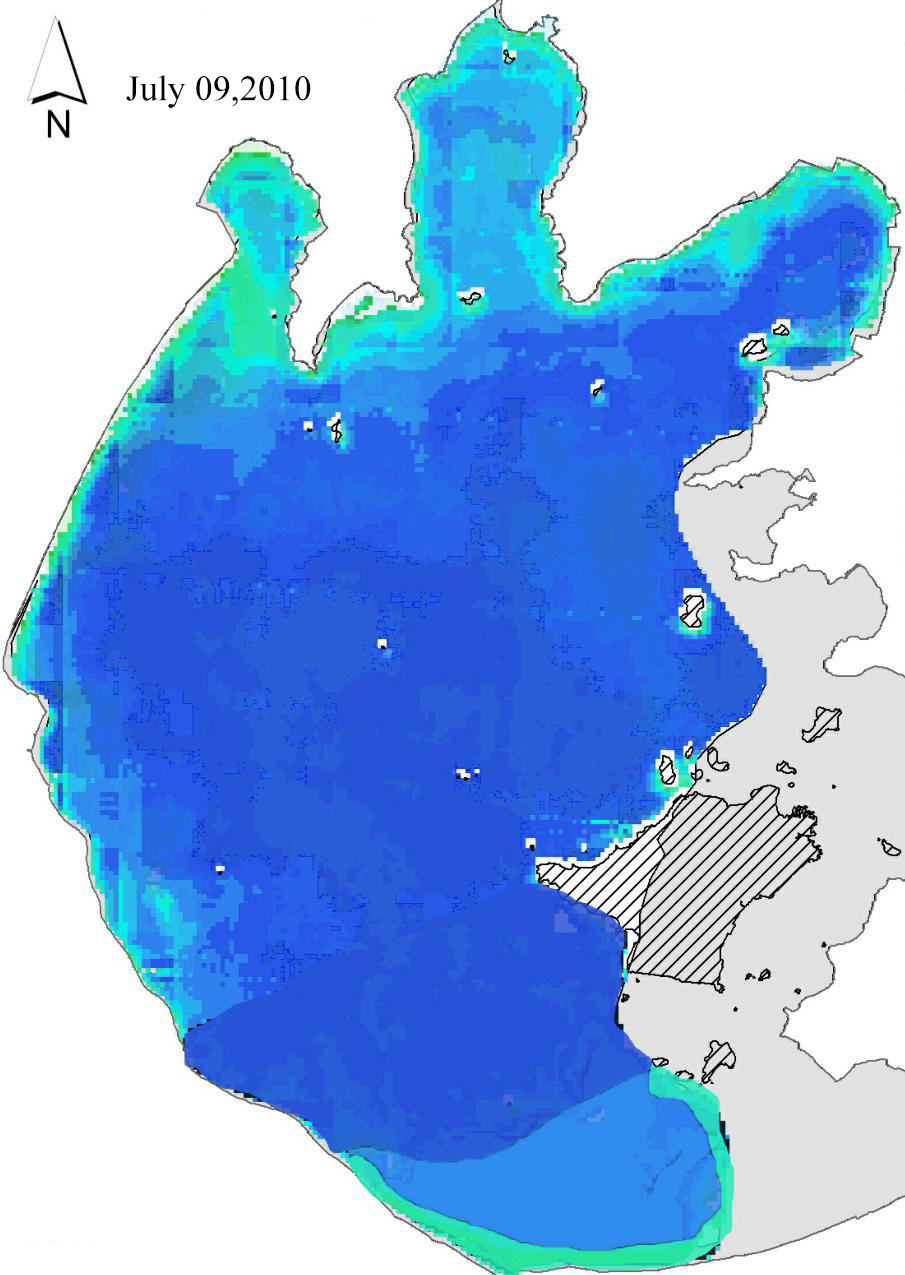

Supplement: Supplemental Information 10 — The data are remote sensing images of chlorophyll a concentration after data scale unification, remote sensing image repair, and time series filling. Remote sensing images of 30 consecutive moments were used as input to the 3D-GAN model. [file peerj-cs-09-1292-s010.zip › 201007090245.jpg]

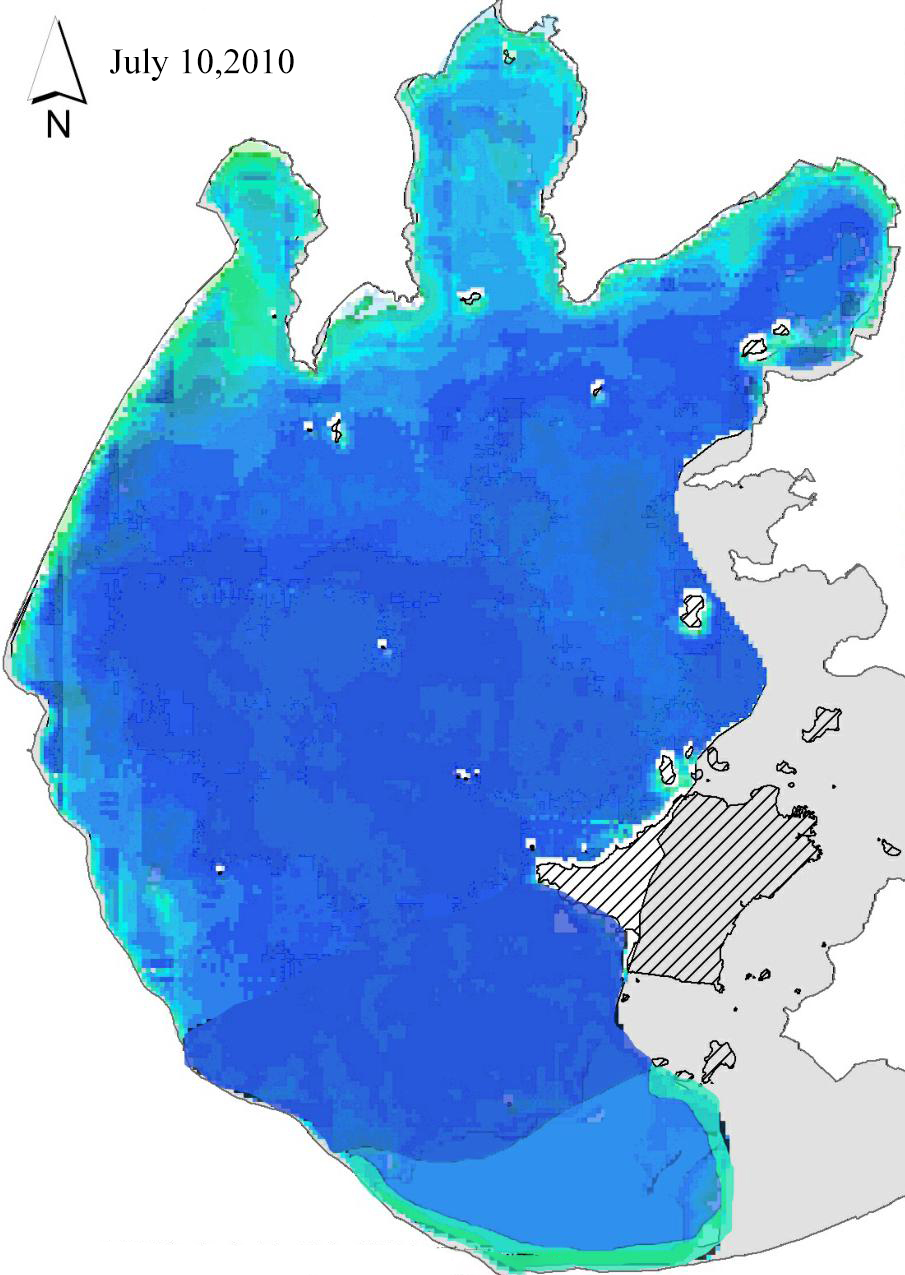

Supplement: Supplemental Information 10 — The data are remote sensing images of chlorophyll a concentration after data scale unification, remote sensing image repair, and time series filling. Remote sensing images of 30 consecutive moments were used as input to the 3D-GAN model. [file peerj-cs-09-1292-s010.zip › 201007100245.jpg]

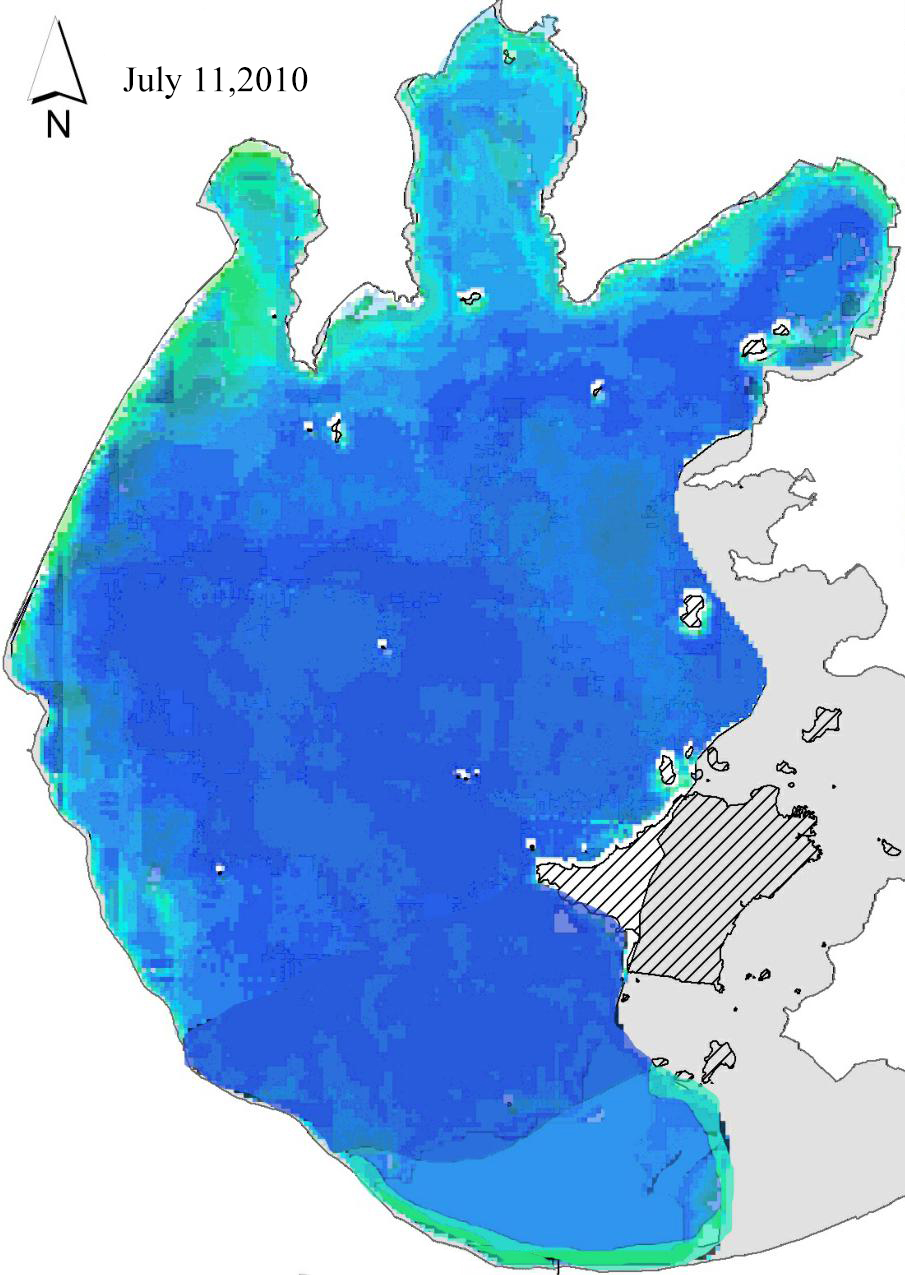

Supplement: Supplemental Information 10 — The data are remote sensing images of chlorophyll a concentration after data scale unification, remote sensing image repair, and time series filling. Remote sensing images of 30 consecutive moments were used as input to the 3D-GAN model. [file peerj-cs-09-1292-s010.zip › 201007110245.jpg]

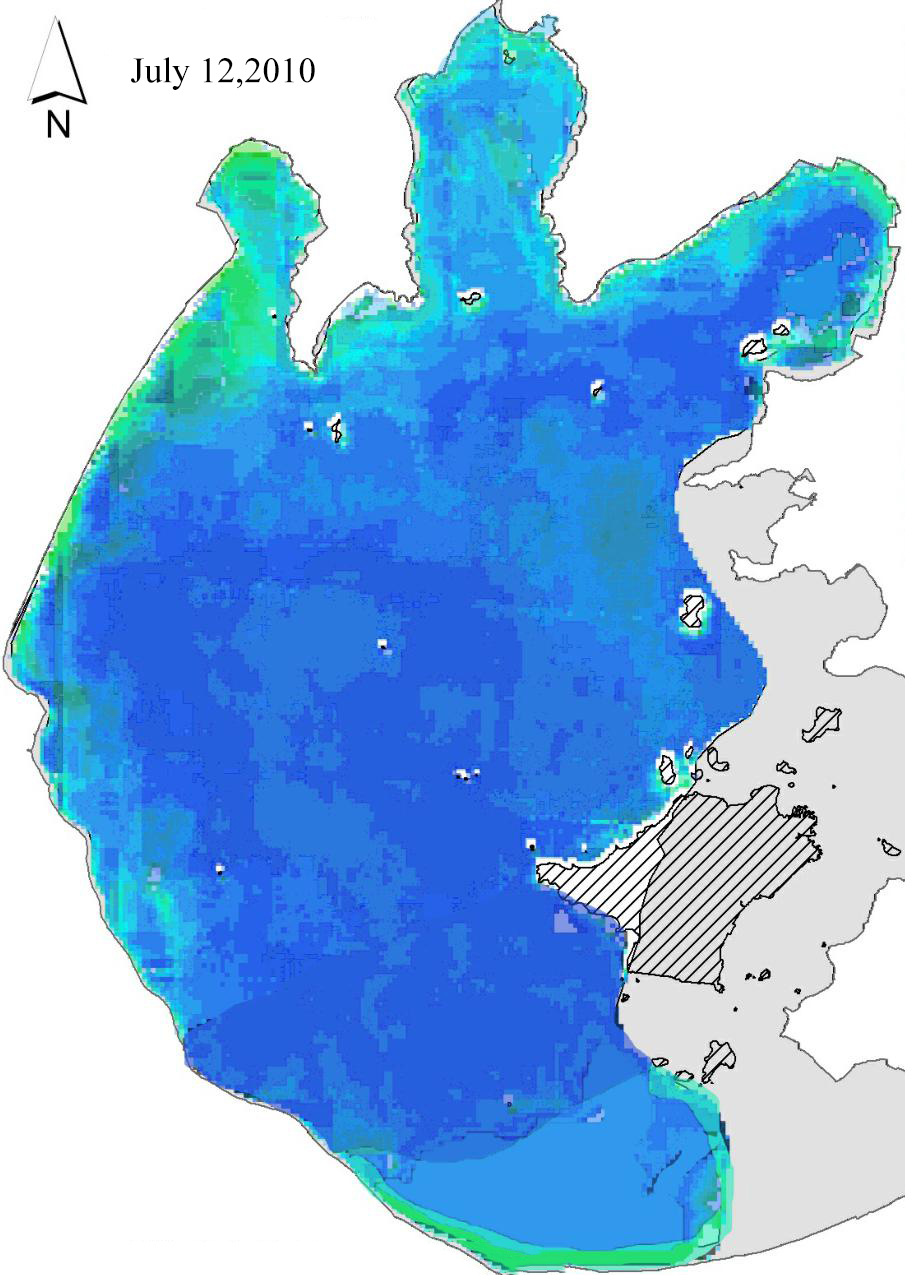

Supplement: Supplemental Information 10 — The data are remote sensing images of chlorophyll a concentration after data scale unification, remote sensing image repair, and time series filling. Remote sensing images of 30 consecutive moments were used as input to the 3D-GAN model. [file peerj-cs-09-1292-s010.zip › 201007120245.jpg]

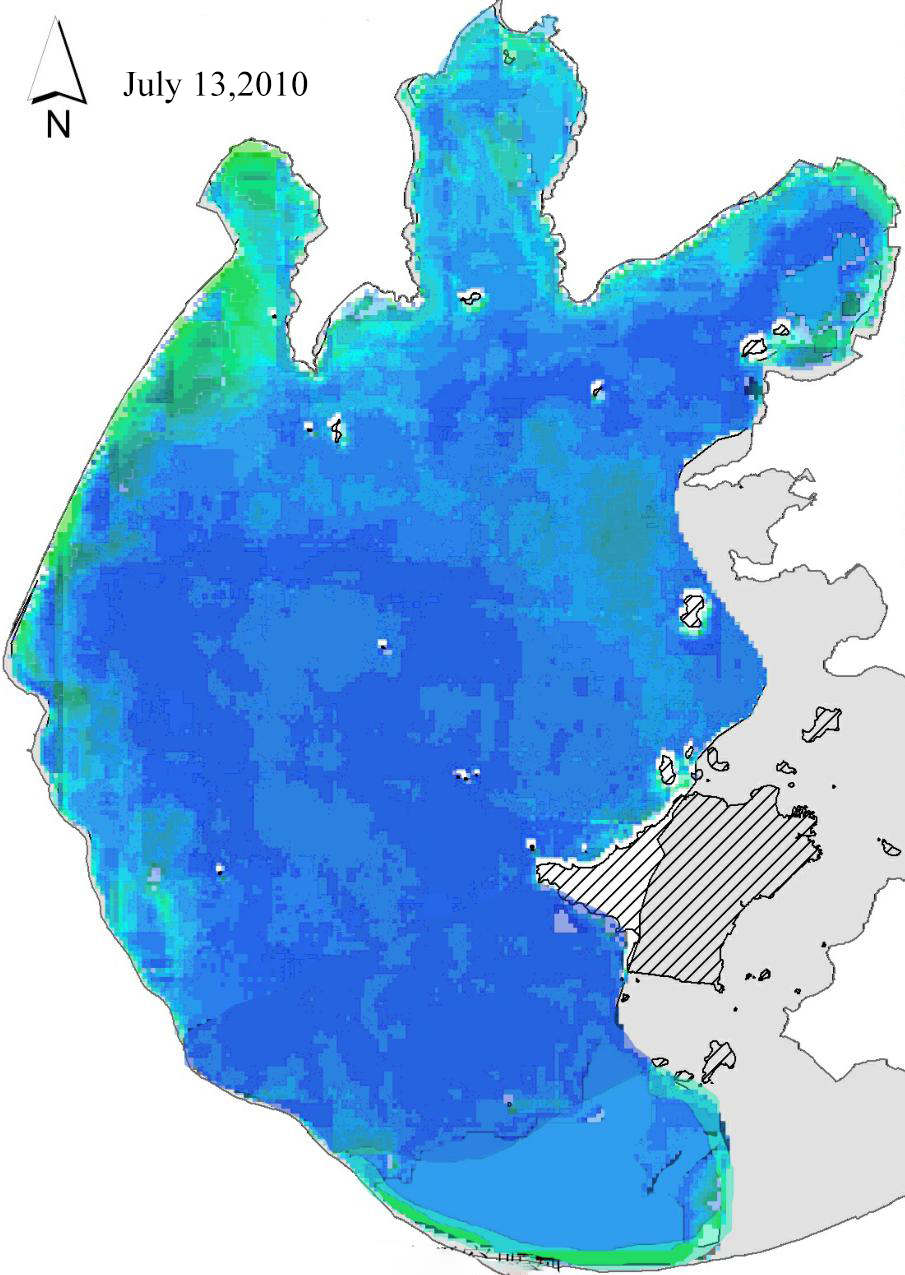

Supplement: Supplemental Information 10 — The data are remote sensing images of chlorophyll a concentration after data scale unification, remote sensing image repair, and time series filling. Remote sensing images of 30 consecutive moments were used as input to the 3D-GAN model. [file peerj-cs-09-1292-s010.zip › 201007130245.jpg]

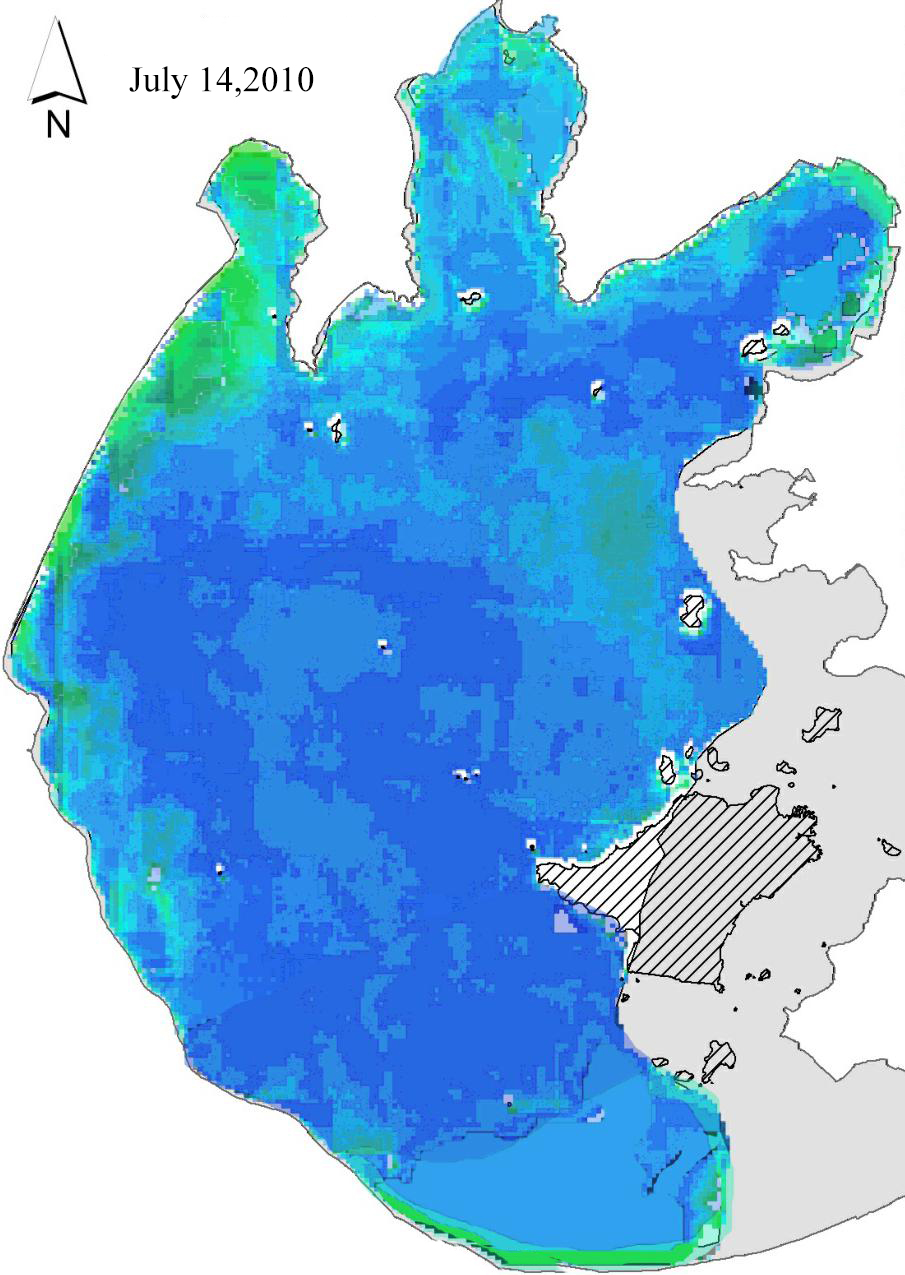

Supplement: Supplemental Information 10 — The data are remote sensing images of chlorophyll a concentration after data scale unification, remote sensing image repair, and time series filling. Remote sensing images of 30 consecutive moments were used as input to the 3D-GAN model. [file peerj-cs-09-1292-s010.zip › 201007140245.jpg]

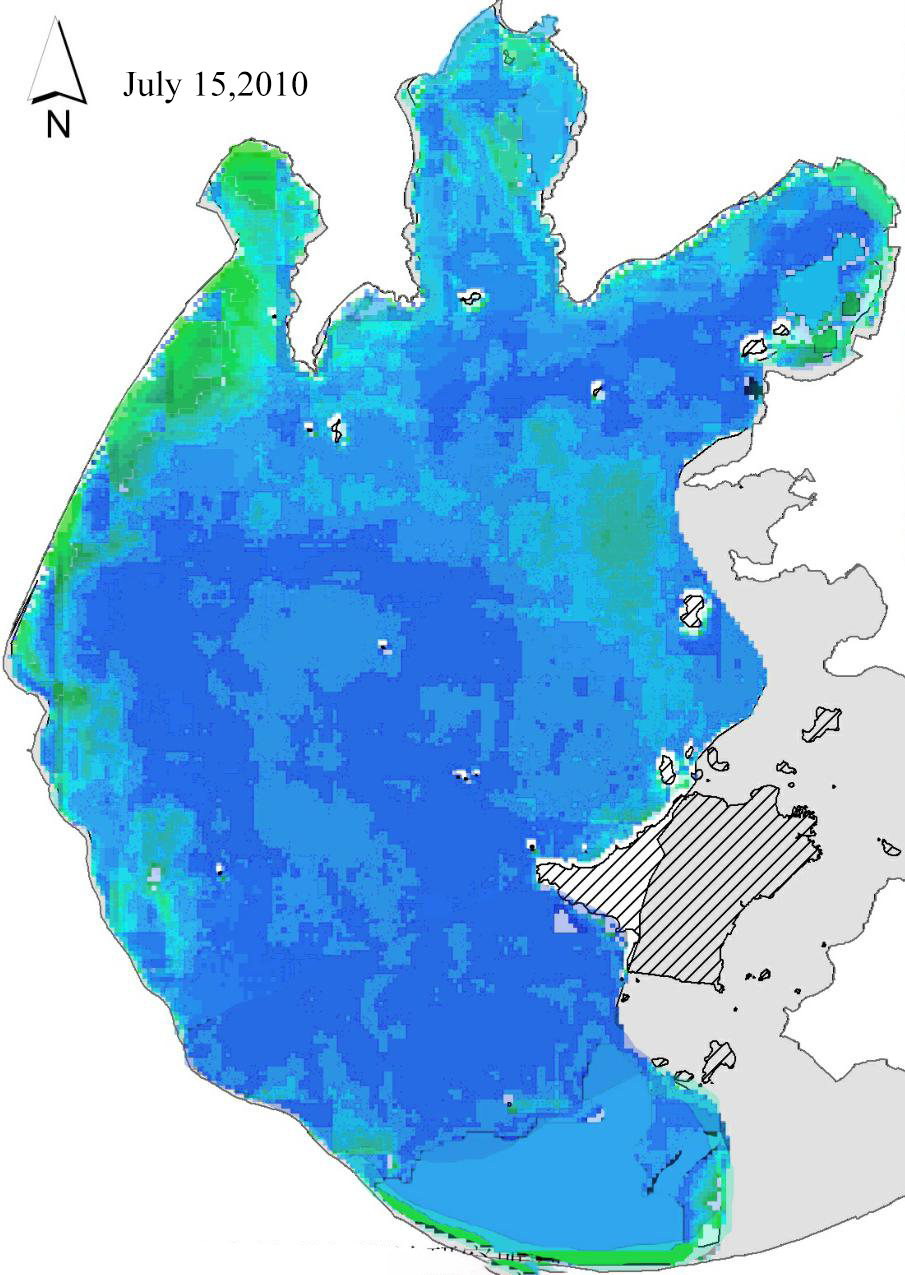

Supplement: Supplemental Information 10 — The data are remote sensing images of chlorophyll a concentration after data scale unification, remote sensing image repair, and time series filling. Remote sensing images of 30 consecutive moments were used as input to the 3D-GAN model. [file peerj-cs-09-1292-s010.zip › 201007150245.jpg]

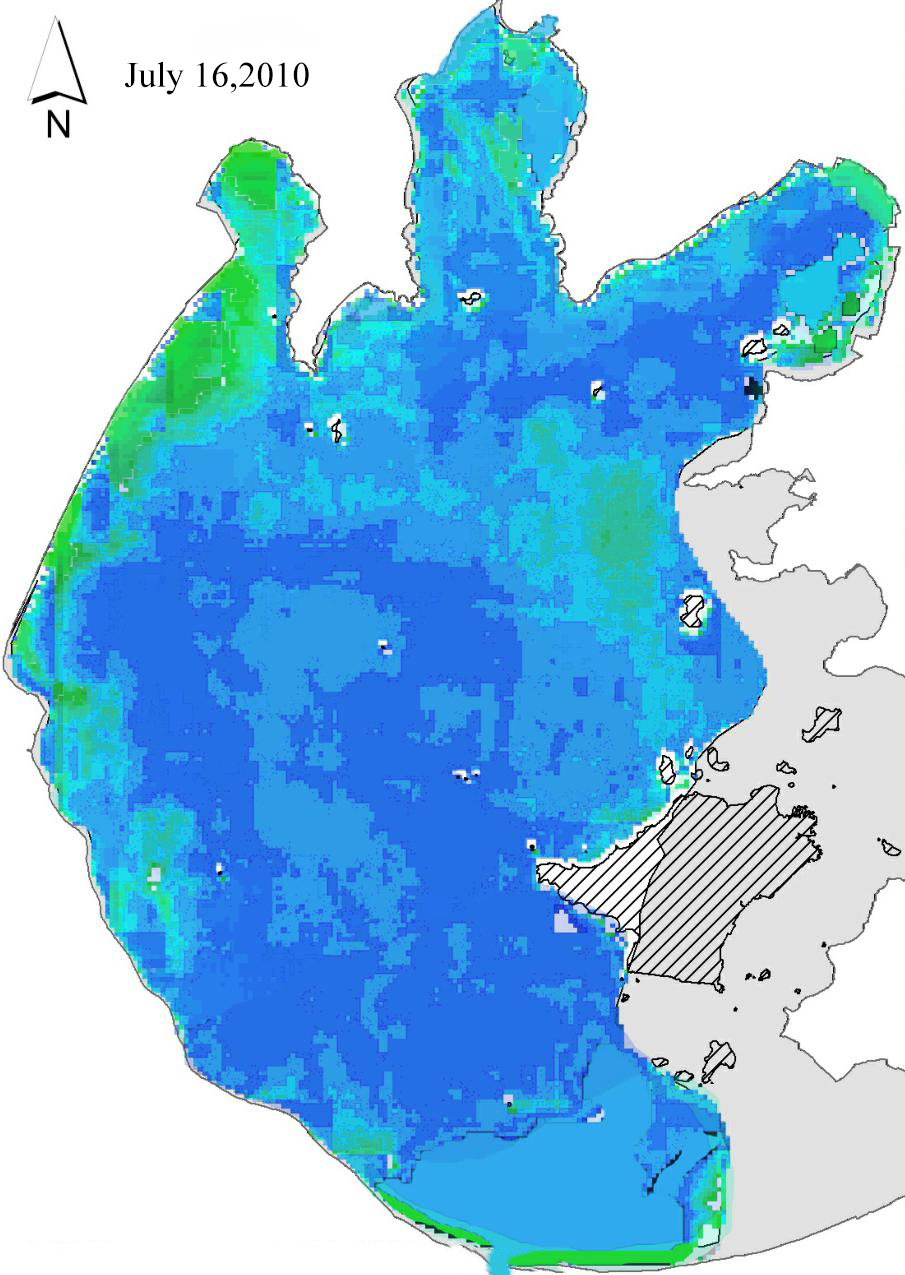

Supplement: Supplemental Information 10 — The data are remote sensing images of chlorophyll a concentration after data scale unification, remote sensing image repair, and time series filling. Remote sensing images of 30 consecutive moments were used as input to the 3D-GAN model. [file peerj-cs-09-1292-s010.zip › 201007160245.jpg]

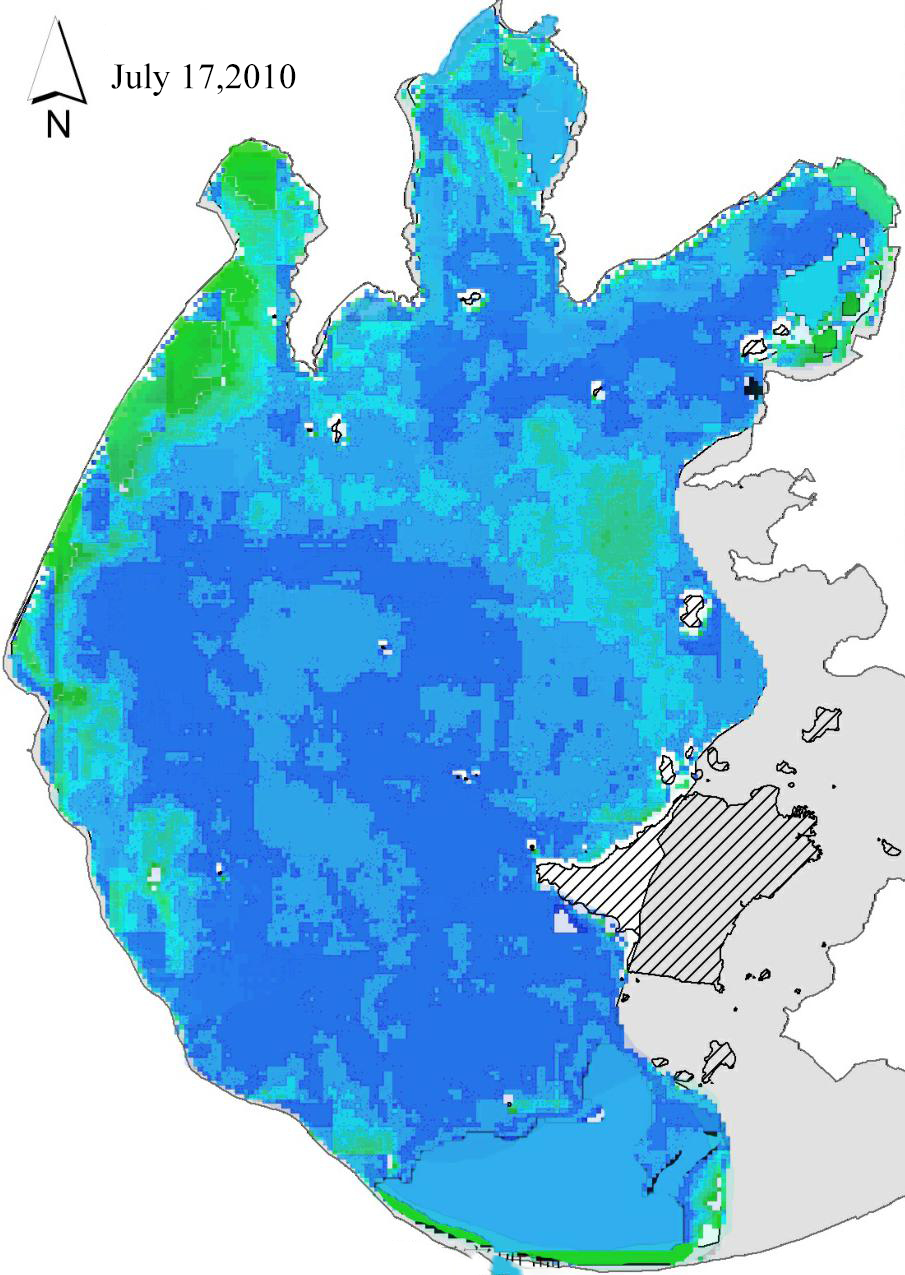

Supplement: Supplemental Information 10 — The data are remote sensing images of chlorophyll a concentration after data scale unification, remote sensing image repair, and time series filling. Remote sensing images of 30 consecutive moments were used as input to the 3D-GAN model. [file peerj-cs-09-1292-s010.zip › 201007170245.jpg]

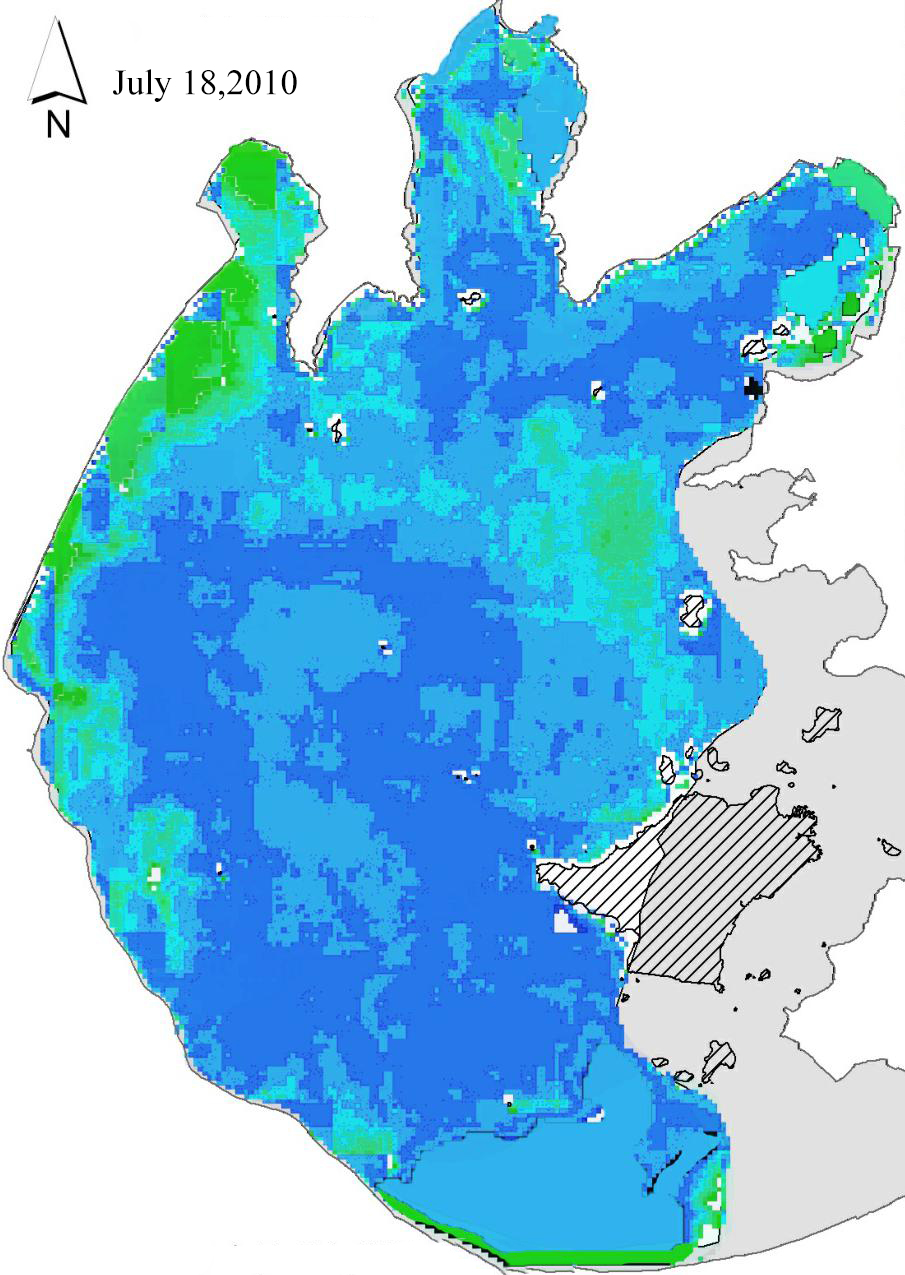

Supplement: Supplemental Information 10 — The data are remote sensing images of chlorophyll a concentration after data scale unification, remote sensing image repair, and time series filling. Remote sensing images of 30 consecutive moments were used as input to the 3D-GAN model. [file peerj-cs-09-1292-s010.zip › 201007180245.jpg]

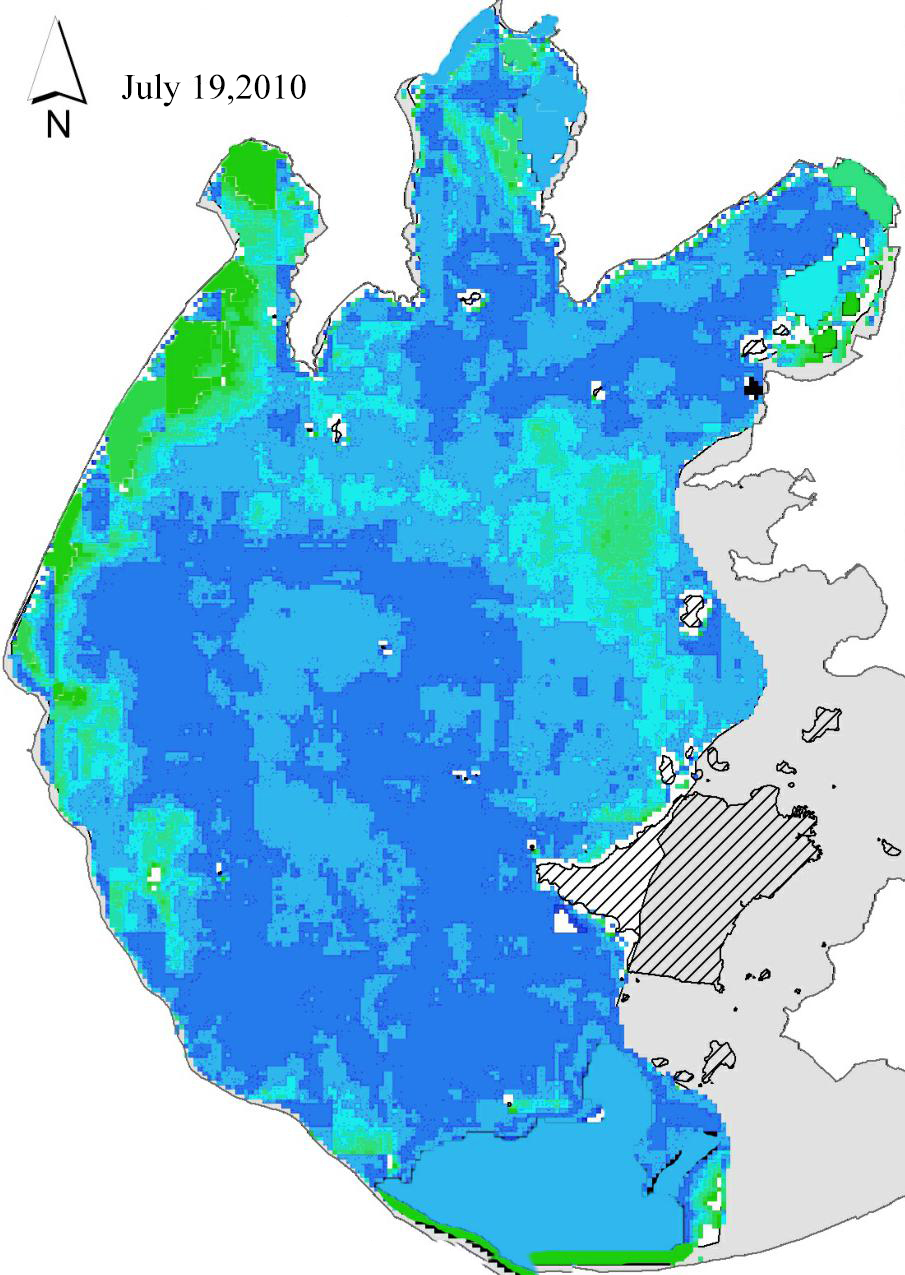

Supplement: Supplemental Information 10 — The data are remote sensing images of chlorophyll a concentration after data scale unification, remote sensing image repair, and time series filling. Remote sensing images of 30 consecutive moments were used as input to the 3D-GAN model. [file peerj-cs-09-1292-s010.zip › 201007190245.jpg]

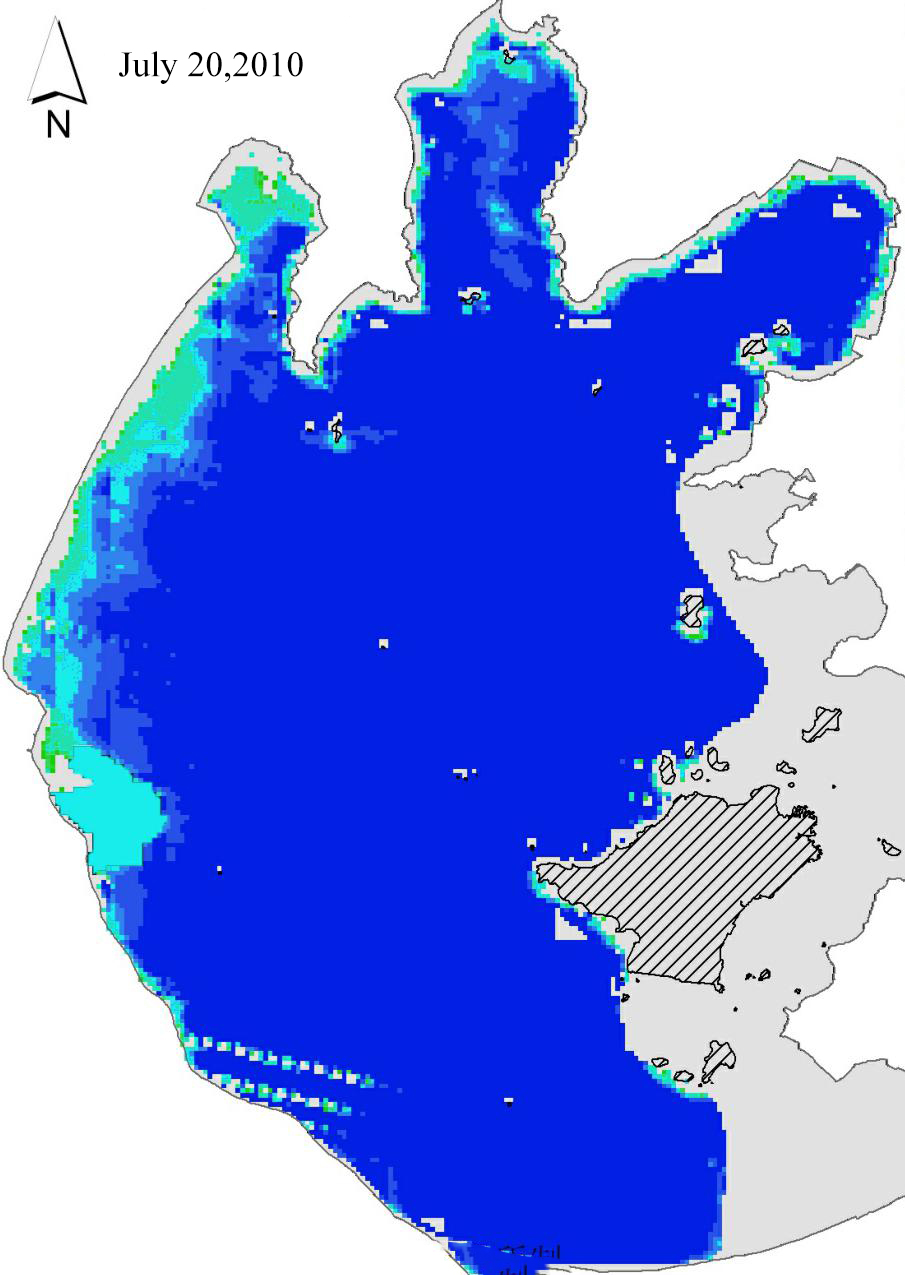

Supplement: Supplemental Information 10 — The data are remote sensing images of chlorophyll a concentration after data scale unification, remote sensing image repair, and time series filling. Remote sensing images of 30 consecutive moments were used as input to the 3D-GAN model. [file peerj-cs-09-1292-s010.zip › 201007200245.jpg]

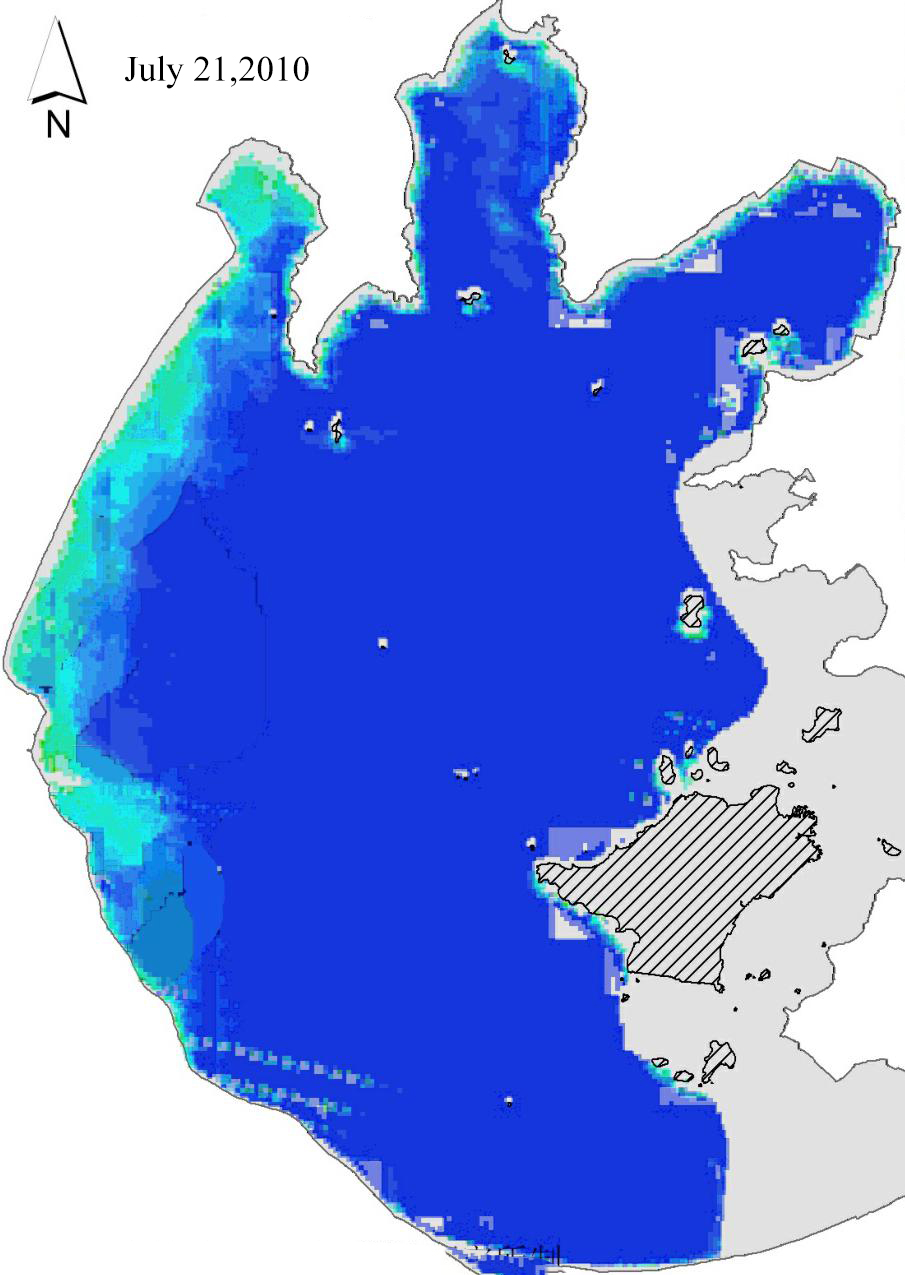

Supplement: Supplemental Information 10 — The data are remote sensing images of chlorophyll a concentration after data scale unification, remote sensing image repair, and time series filling. Remote sensing images of 30 consecutive moments were used as input to the 3D-GAN model. [file peerj-cs-09-1292-s010.zip › 201007210245.jpg]

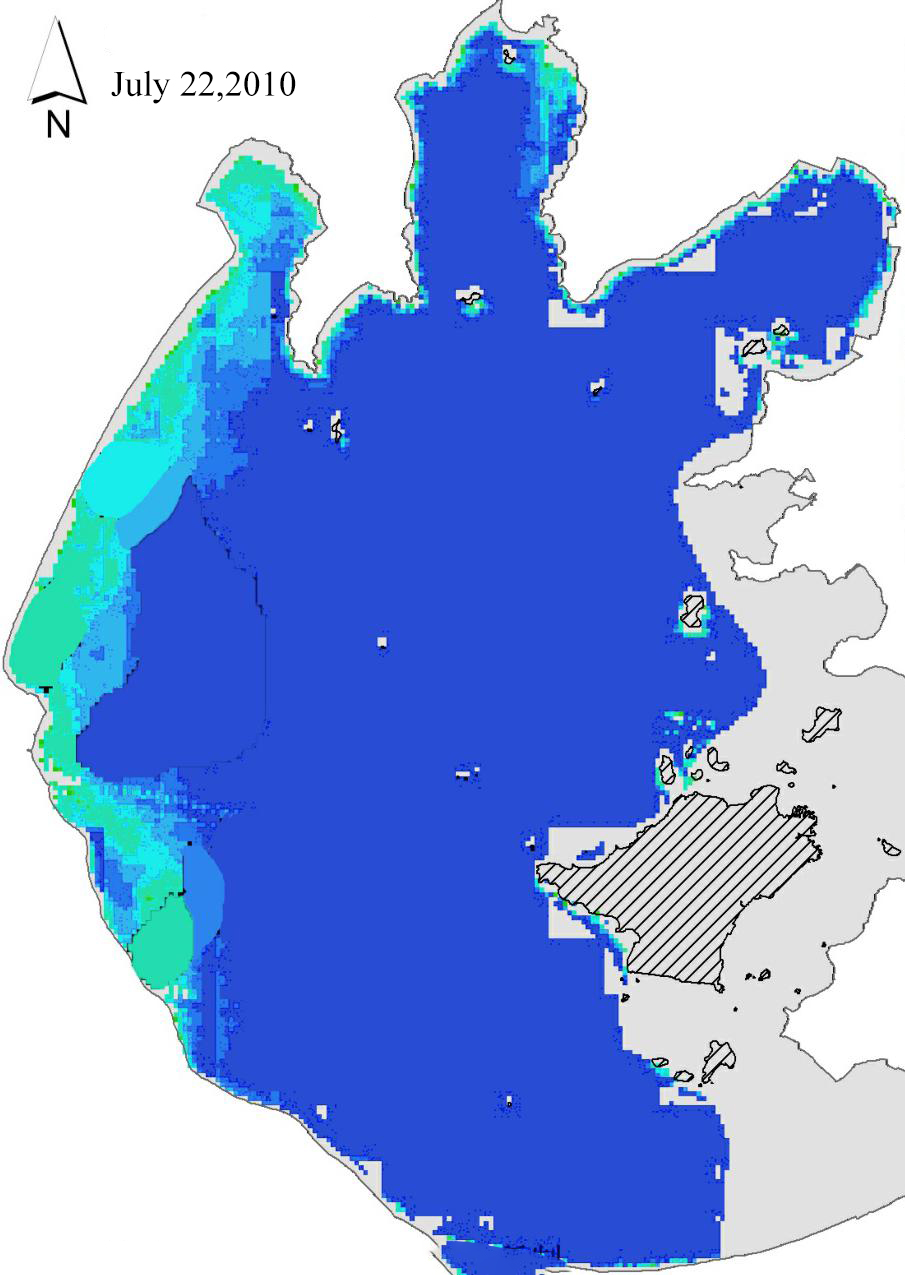

Supplement: Supplemental Information 10 — The data are remote sensing images of chlorophyll a concentration after data scale unification, remote sensing image repair, and time series filling. Remote sensing images of 30 consecutive moments were used as input to the 3D-GAN model. [file peerj-cs-09-1292-s010.zip › 201007220245.jpg]

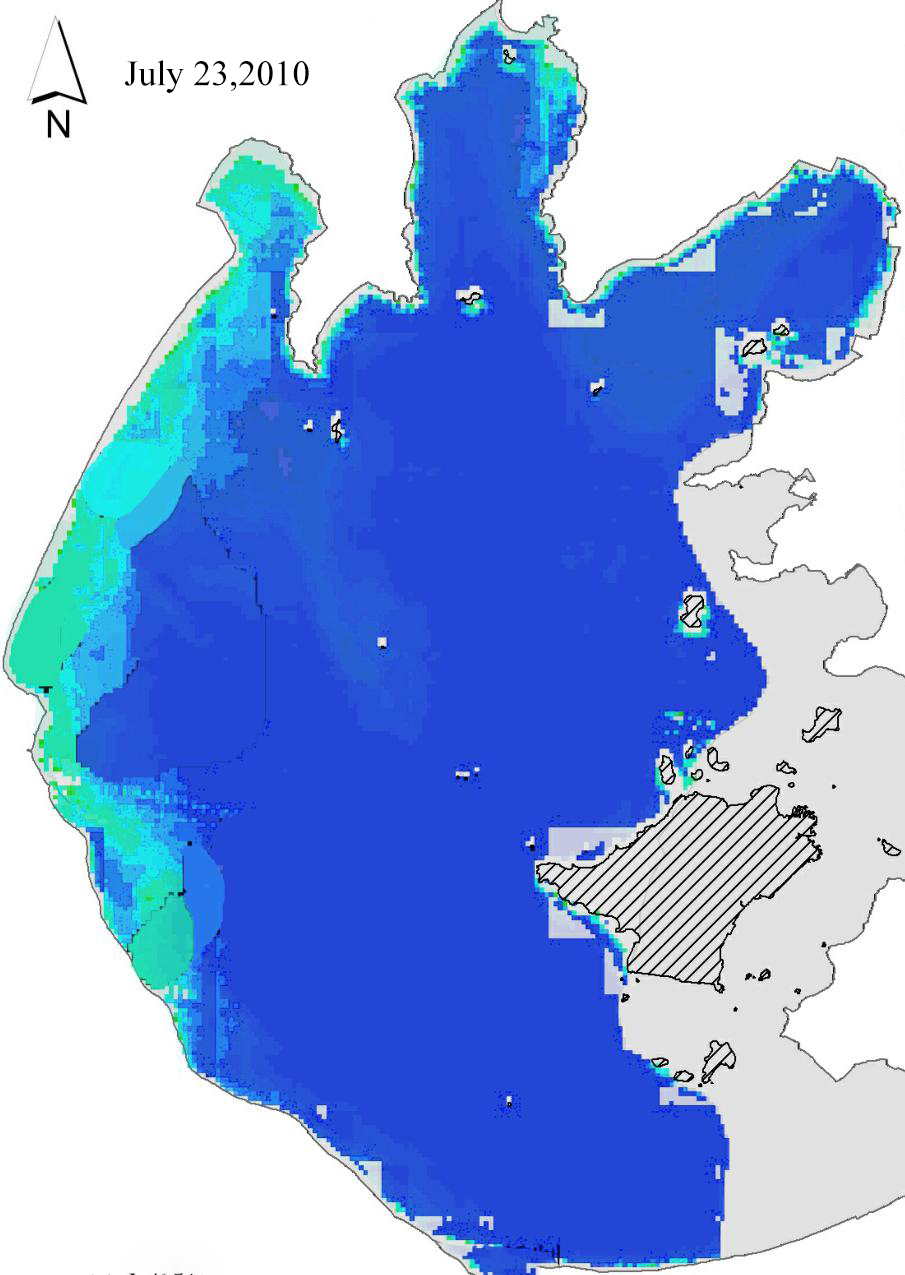

Supplement: Supplemental Information 10 — The data are remote sensing images of chlorophyll a concentration after data scale unification, remote sensing image repair, and time series filling. Remote sensing images of 30 consecutive moments were used as input to the 3D-GAN model. [file peerj-cs-09-1292-s010.zip › 201007230245.jpg]

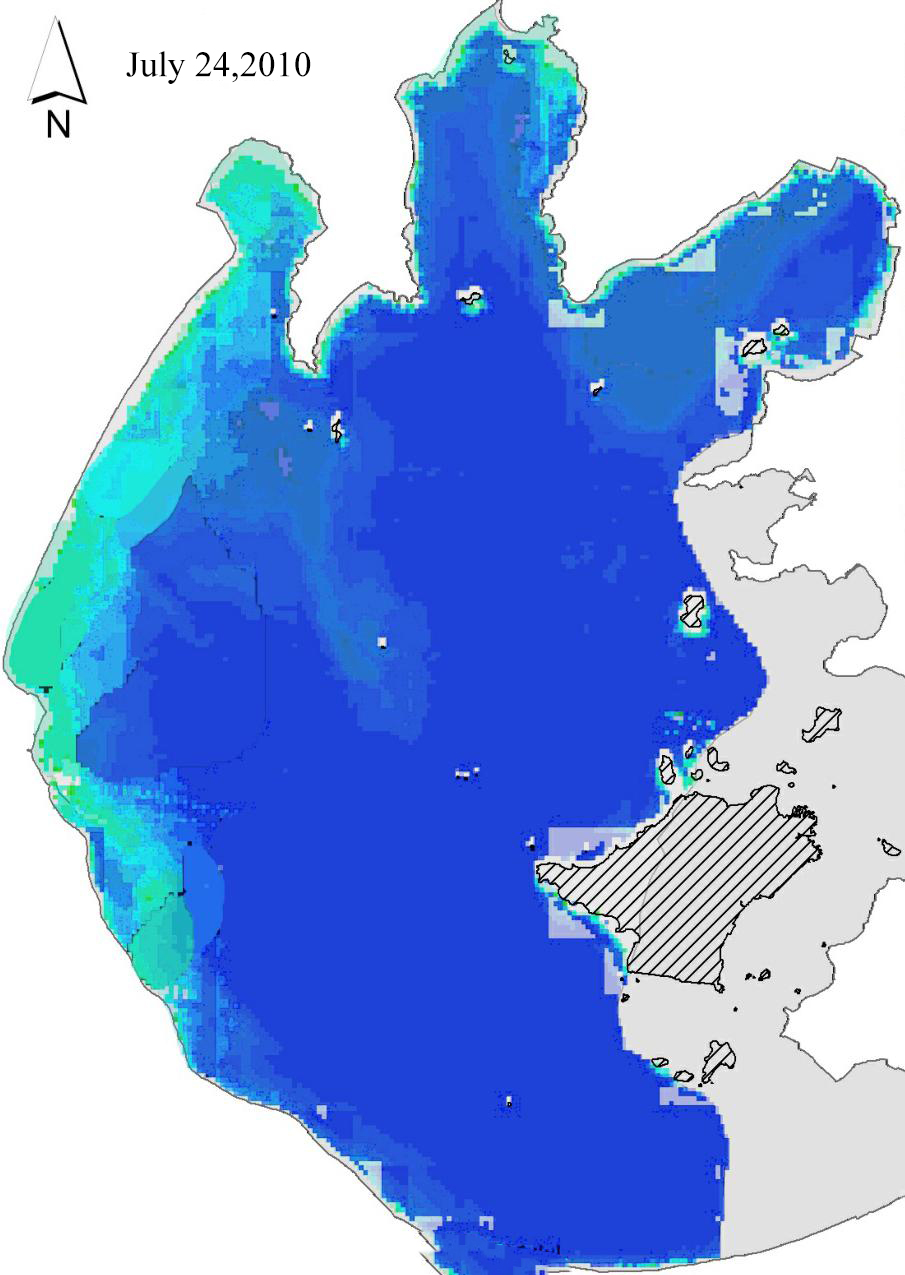

Supplement: Supplemental Information 10 — The data are remote sensing images of chlorophyll a concentration after data scale unification, remote sensing image repair, and time series filling. Remote sensing images of 30 consecutive moments were used as input to the 3D-GAN model. [file peerj-cs-09-1292-s010.zip › 201007240245.jpg]

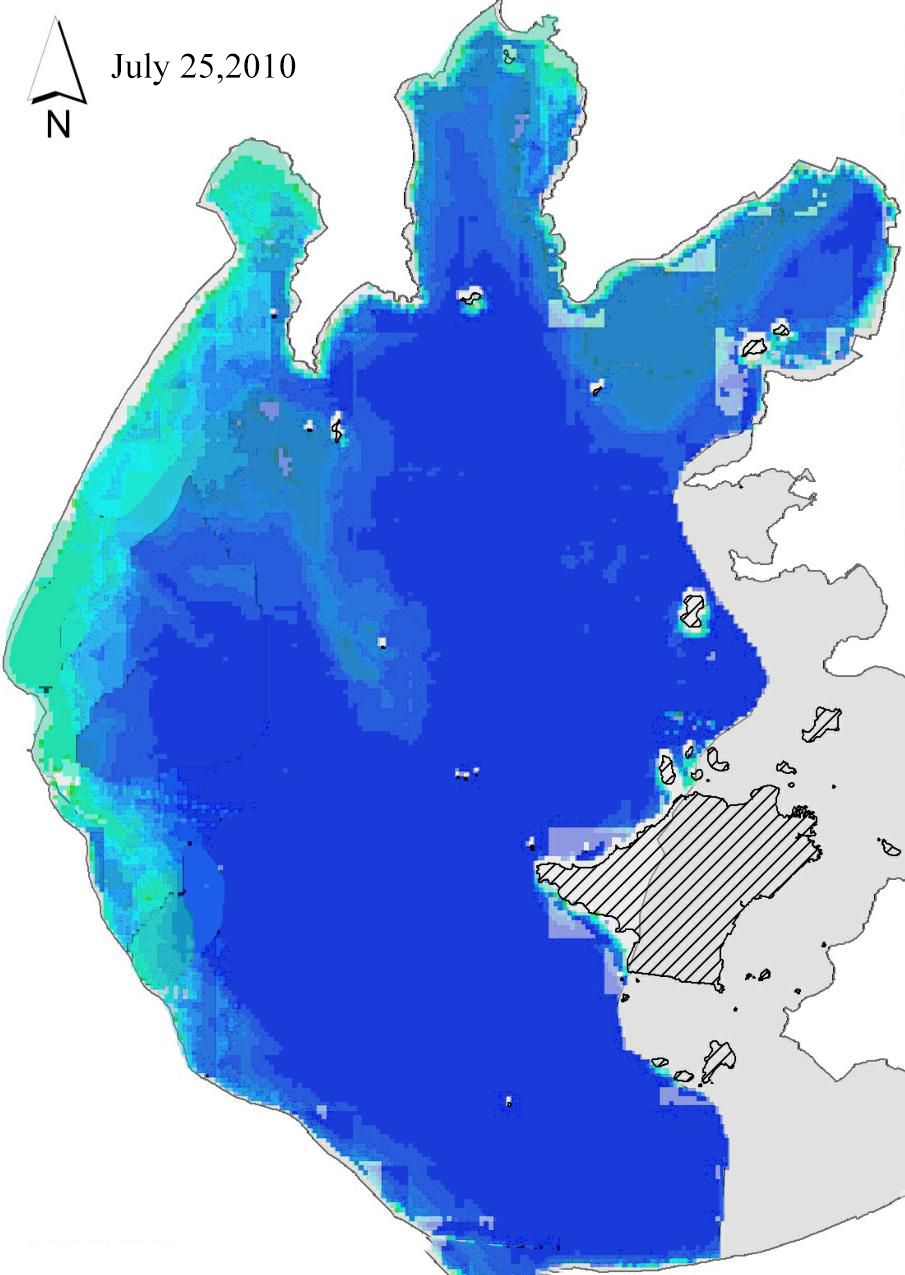

Supplement: Supplemental Information 10 — The data are remote sensing images of chlorophyll a concentration after data scale unification, remote sensing image repair, and time series filling. Remote sensing images of 30 consecutive moments were used as input to the 3D-GAN model. [file peerj-cs-09-1292-s010.zip › 201007250245.jpg]

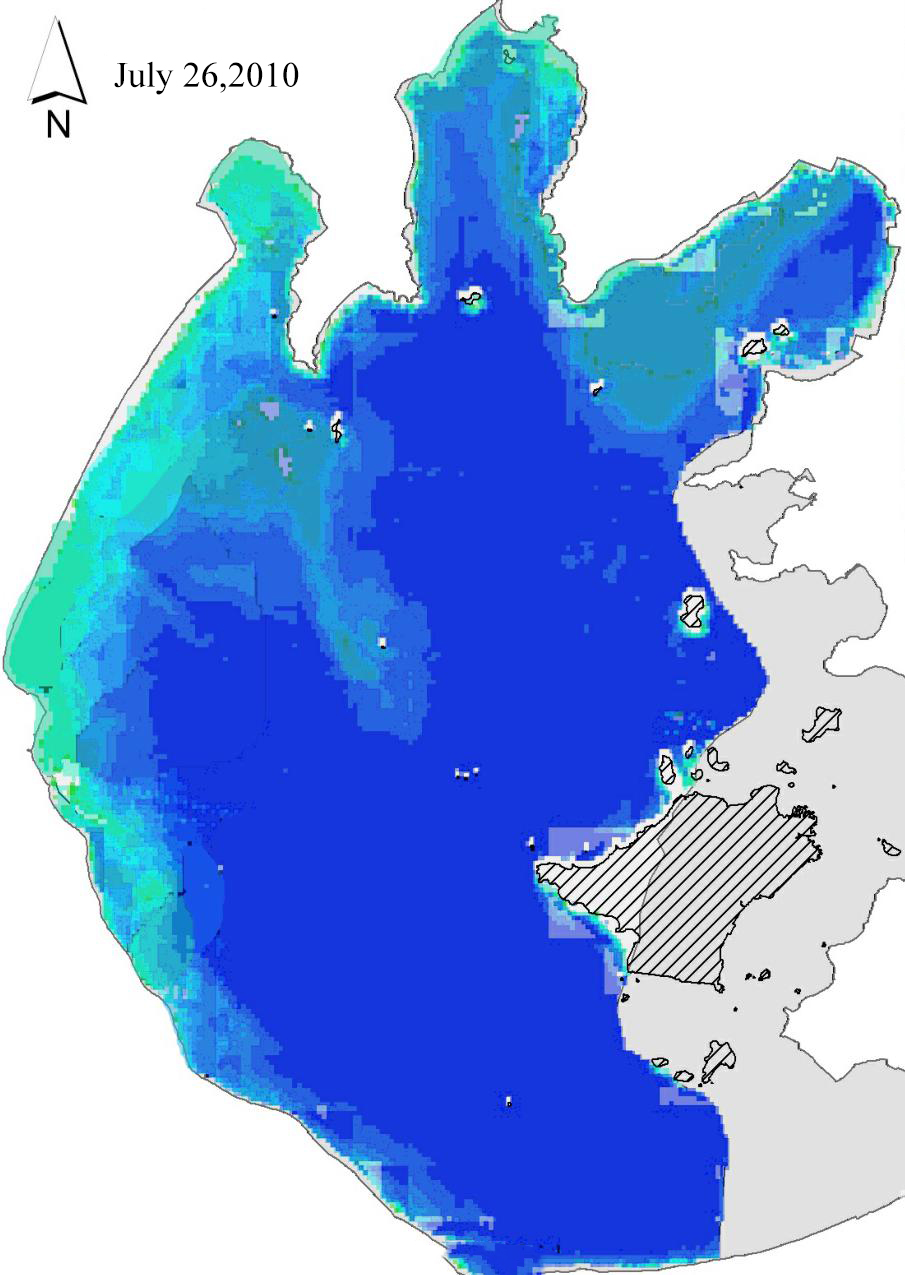

Supplement: Supplemental Information 10 — The data are remote sensing images of chlorophyll a concentration after data scale unification, remote sensing image repair, and time series filling. Remote sensing images of 30 consecutive moments were used as input to the 3D-GAN model. [file peerj-cs-09-1292-s010.zip › 201007260245.jpg]

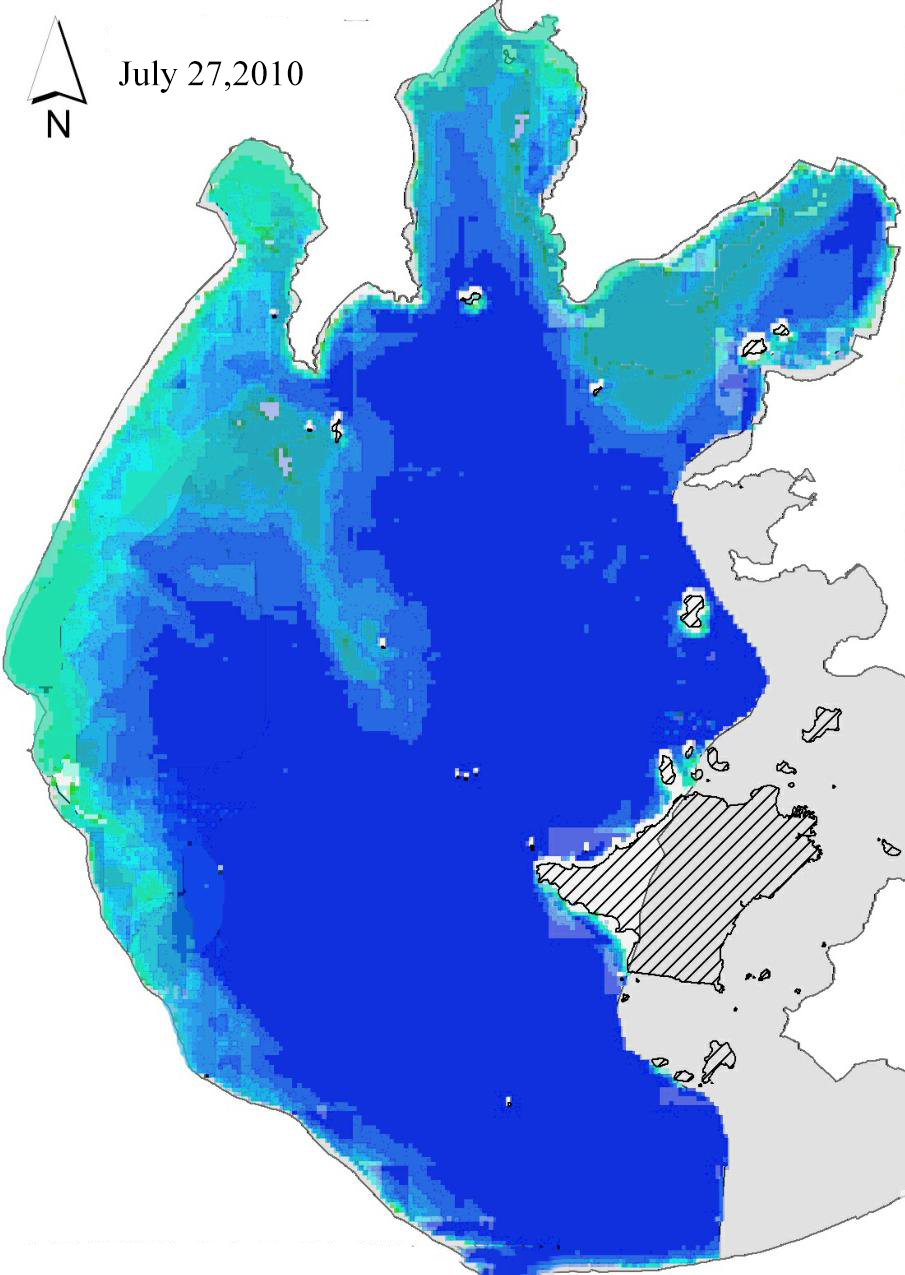

Supplement: Supplemental Information 10 — The data are remote sensing images of chlorophyll a concentration after data scale unification, remote sensing image repair, and time series filling. Remote sensing images of 30 consecutive moments were used as input to the 3D-GAN model. [file peerj-cs-09-1292-s010.zip › 201007270245.jpg]

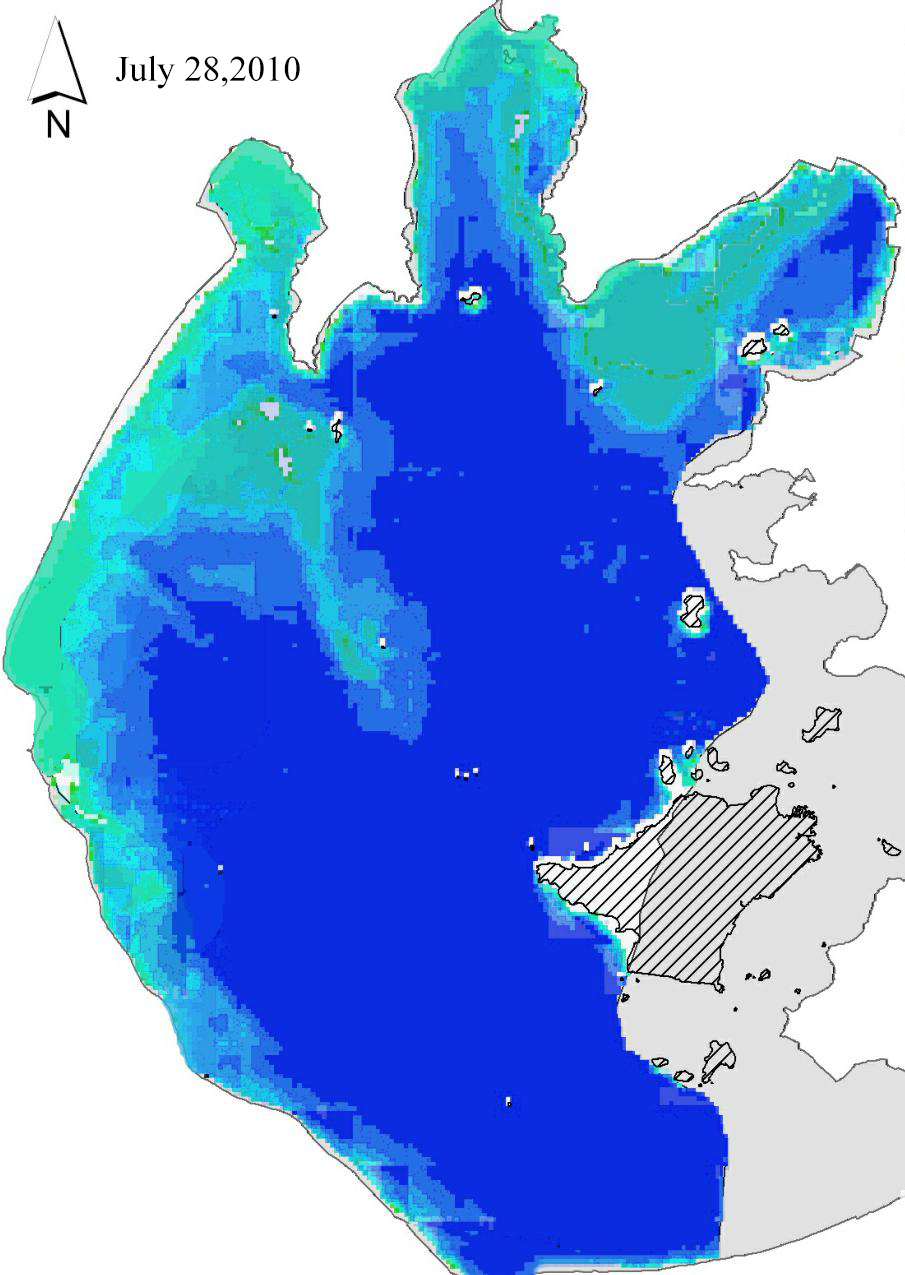

Supplement: Supplemental Information 10 — The data are remote sensing images of chlorophyll a concentration after data scale unification, remote sensing image repair, and time series filling. Remote sensing images of 30 consecutive moments were used as input to the 3D-GAN model. [file peerj-cs-09-1292-s010.zip › 201007280245.jpg]

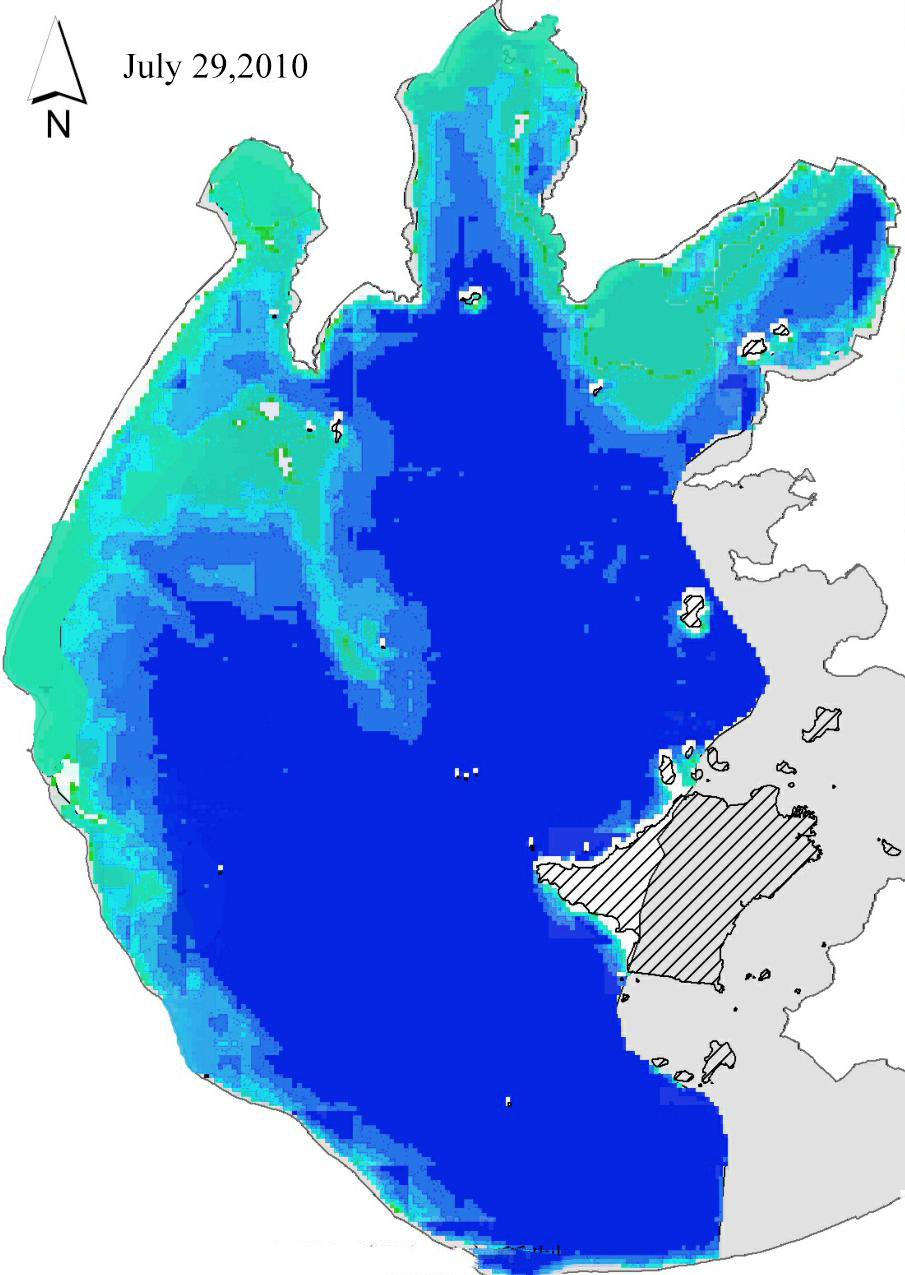

Supplement: Supplemental Information 10 — The data are remote sensing images of chlorophyll a concentration after data scale unification, remote sensing image repair, and time series filling. Remote sensing images of 30 consecutive moments were used as input to the 3D-GAN model. [file peerj-cs-09-1292-s010.zip › 201007290245.jpg]

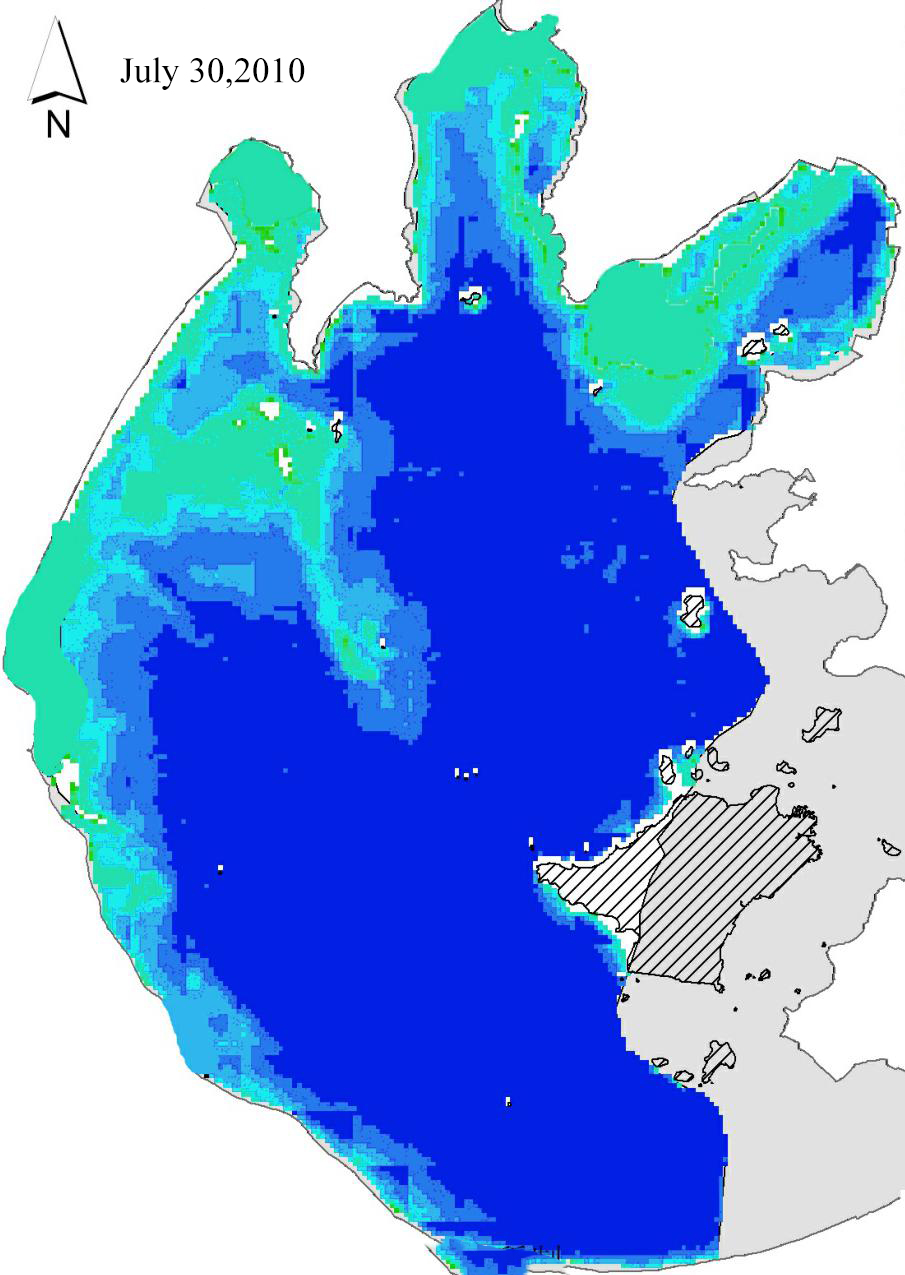

Supplement: Supplemental Information 10 — The data are remote sensing images of chlorophyll a concentration after data scale unification, remote sensing image repair, and time series filling. Remote sensing images of 30 consecutive moments were used as input to the 3D-GAN model. [file peerj-cs-09-1292-s010.zip › 201007300245.jpg]

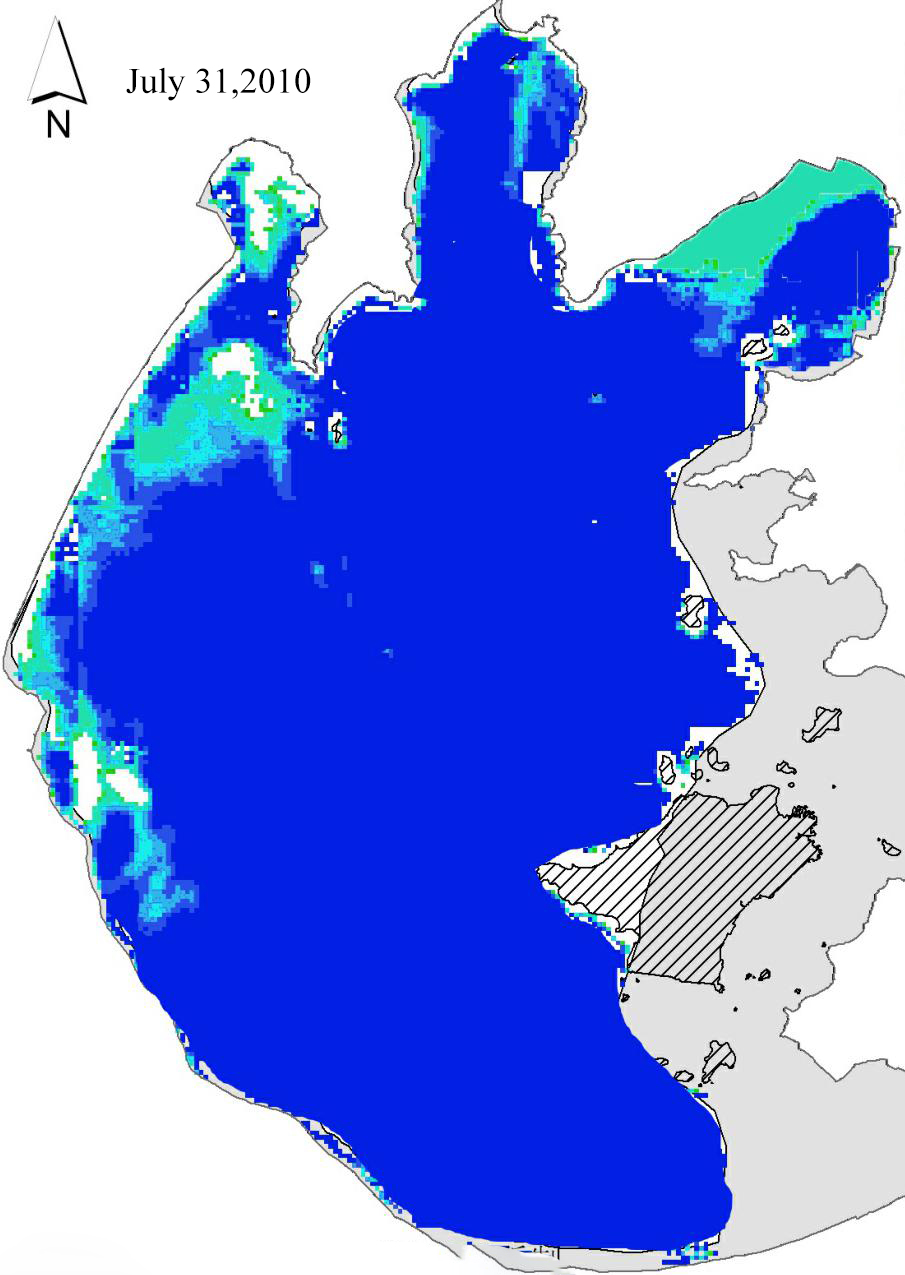

Supplement: Supplemental Information 10 — The data are remote sensing images of chlorophyll a concentration after data scale unification, remote sensing image repair, and time series filling. Remote sensing images of 30 consecutive moments were used as input to the 3D-GAN model. [file peerj-cs-09-1292-s010.zip › 201007310245.jpg]

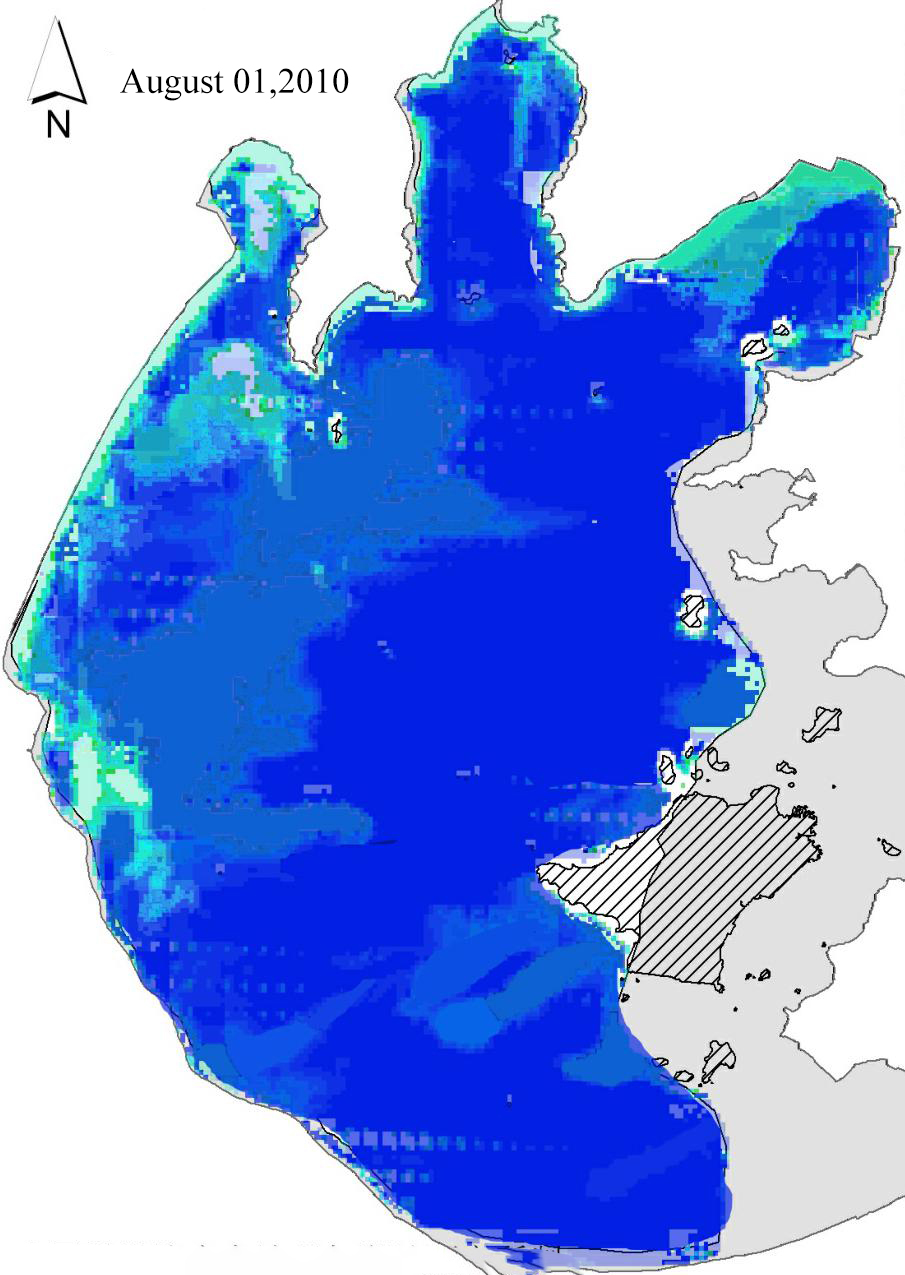

Supplement: Supplemental Information 10 — The data are remote sensing images of chlorophyll a concentration after data scale unification, remote sensing image repair, and time series filling. Remote sensing images of 30 consecutive moments were used as input to the 3D-GAN model. [file peerj-cs-09-1292-s010.zip › 201008010245.jpg]

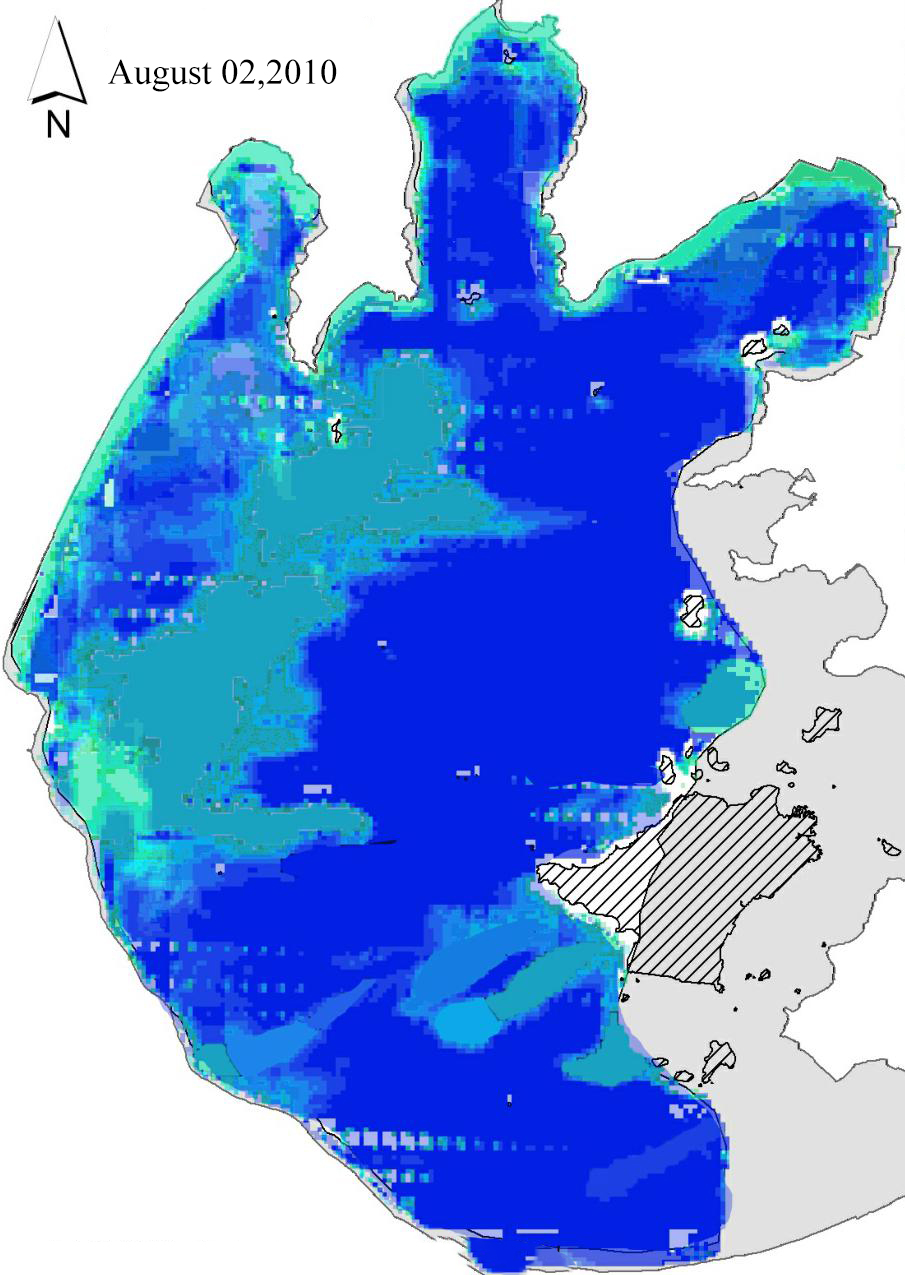

Supplement: Supplemental Information 10 — The data are remote sensing images of chlorophyll a concentration after data scale unification, remote sensing image repair, and time series filling. Remote sensing images of 30 consecutive moments were used as input to the 3D-GAN model. [file peerj-cs-09-1292-s010.zip › 201008020245.jpg]

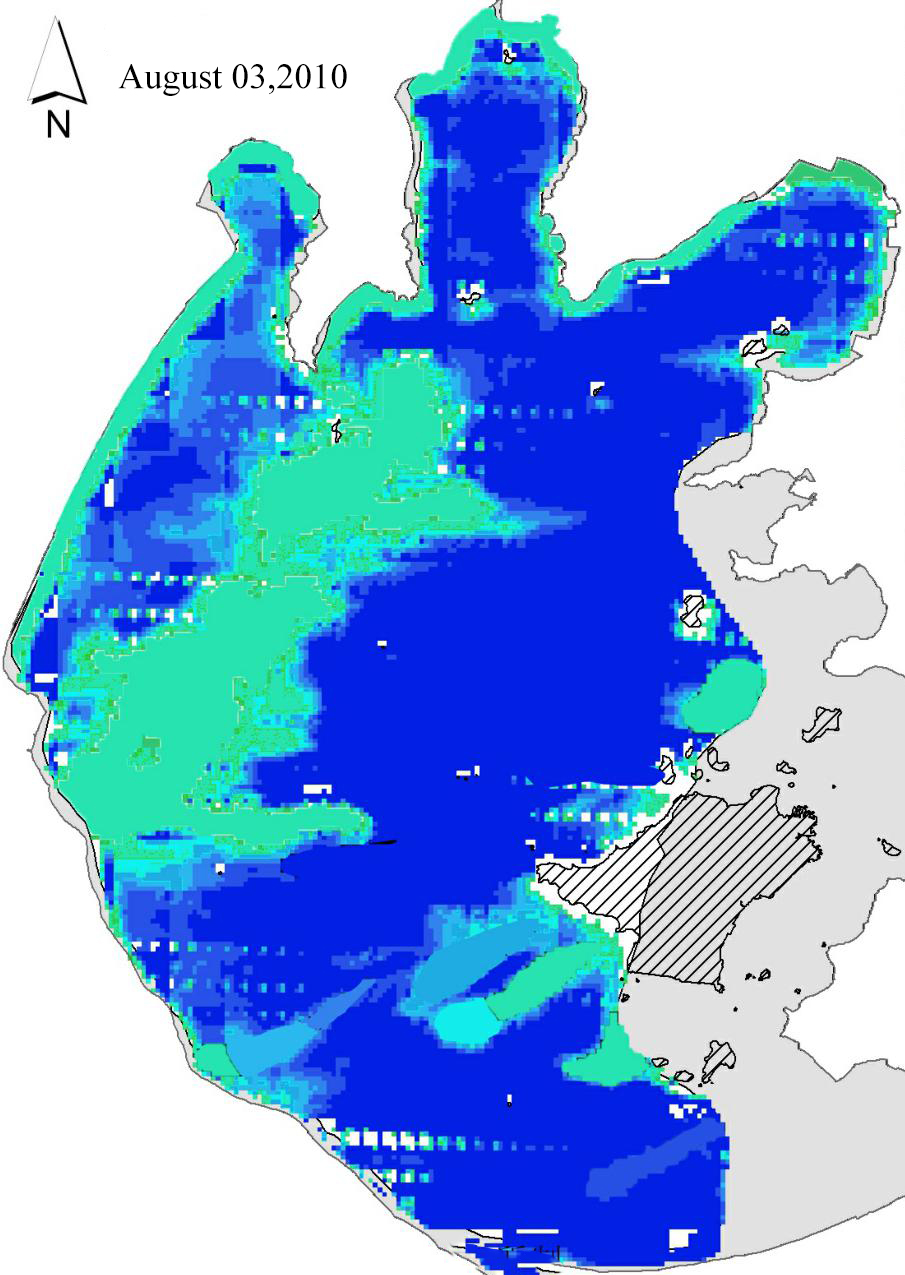

Supplement: Supplemental Information 10 — The data are remote sensing images of chlorophyll a concentration after data scale unification, remote sensing image repair, and time series filling. Remote sensing images of 30 consecutive moments were used as input to the 3D-GAN model. [file peerj-cs-09-1292-s010.zip › 201008030245.jpg]

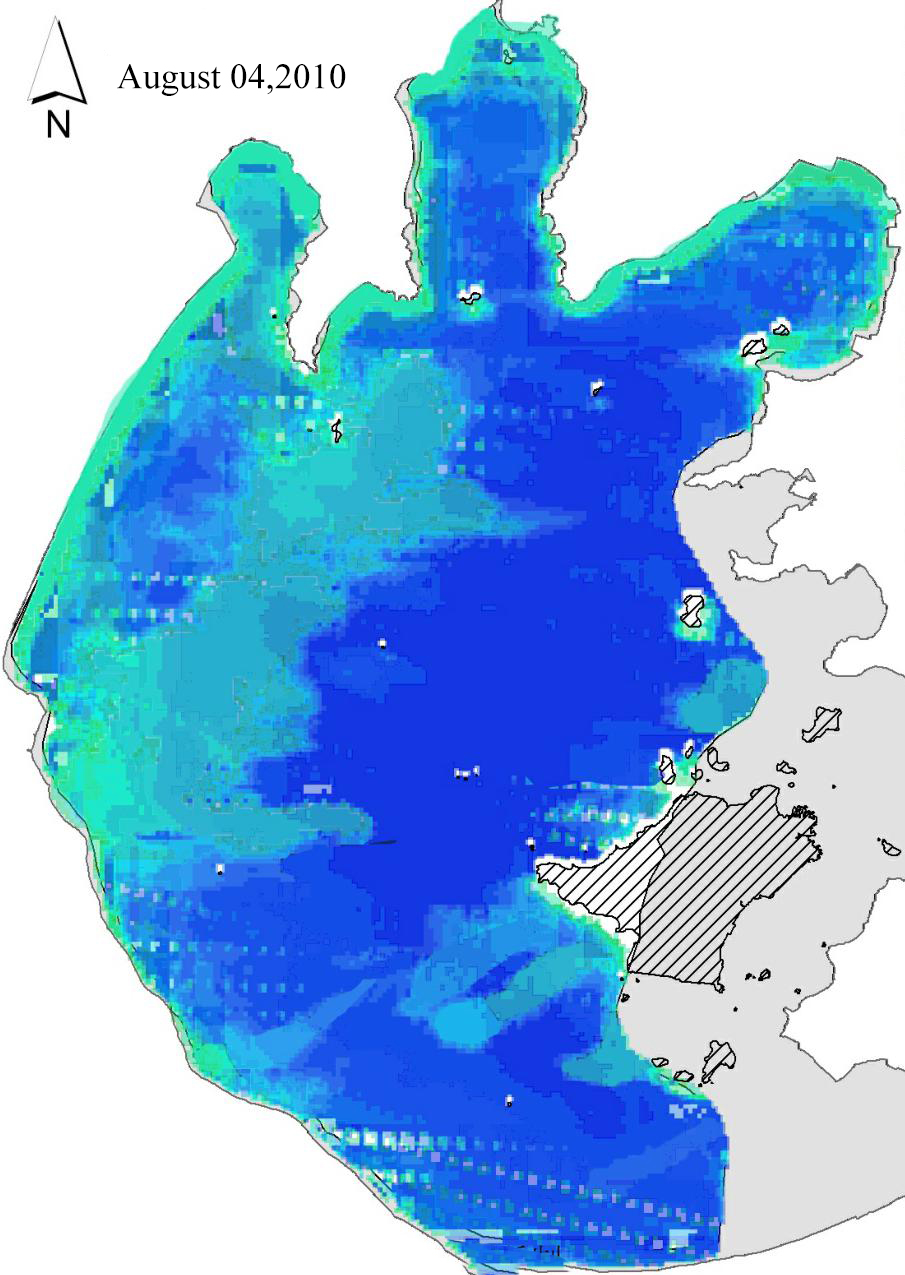

Supplement: Supplemental Information 10 — The data are remote sensing images of chlorophyll a concentration after data scale unification, remote sensing image repair, and time series filling. Remote sensing images of 30 consecutive moments were used as input to the 3D-GAN model. [file peerj-cs-09-1292-s010.zip › 201008040245.jpg]

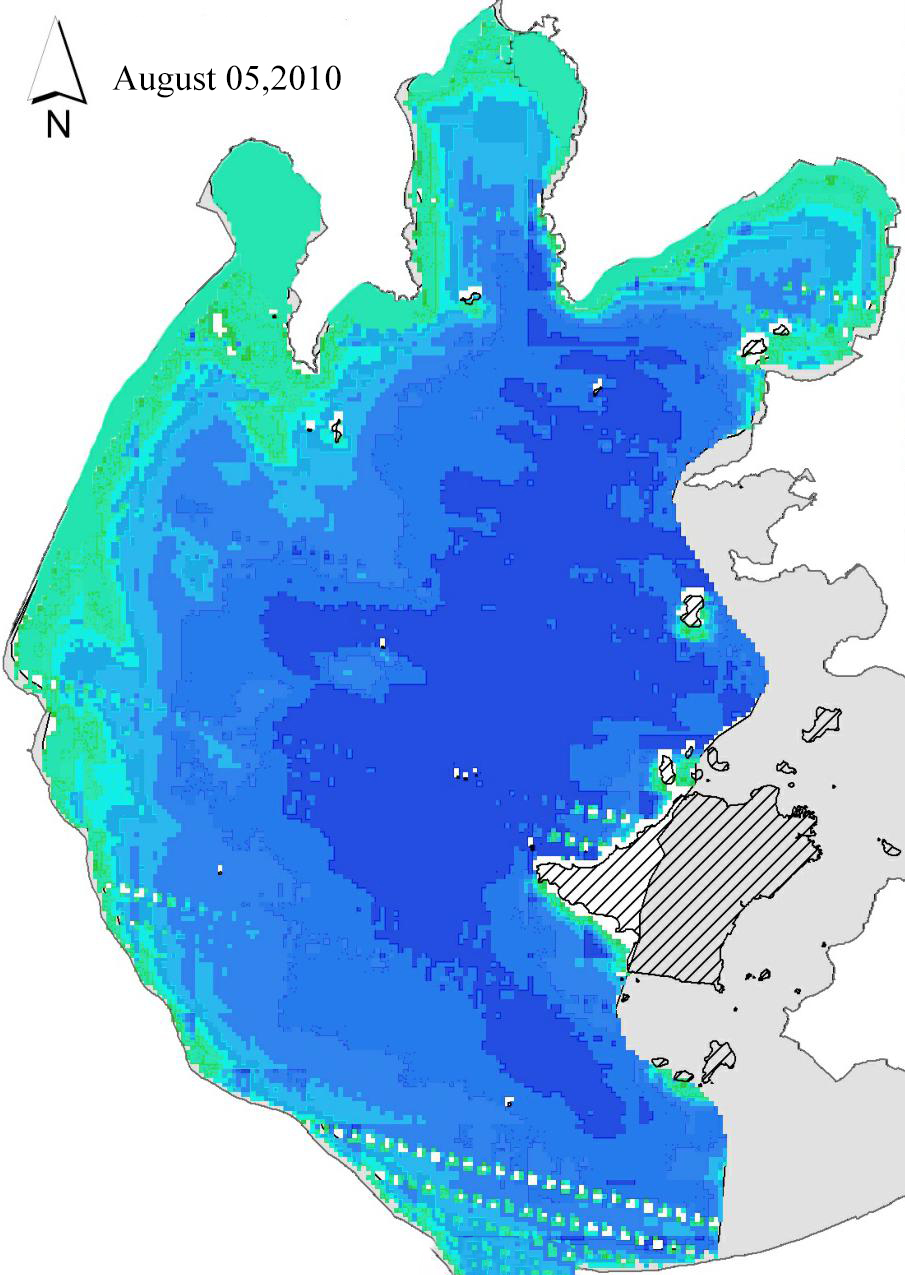

Supplement: Supplemental Information 10 — The data are remote sensing images of chlorophyll a concentration after data scale unification, remote sensing image repair, and time series filling. Remote sensing images of 30 consecutive moments were used as input to the 3D-GAN model. [file peerj-cs-09-1292-s010.zip › 201008050245.jpg]

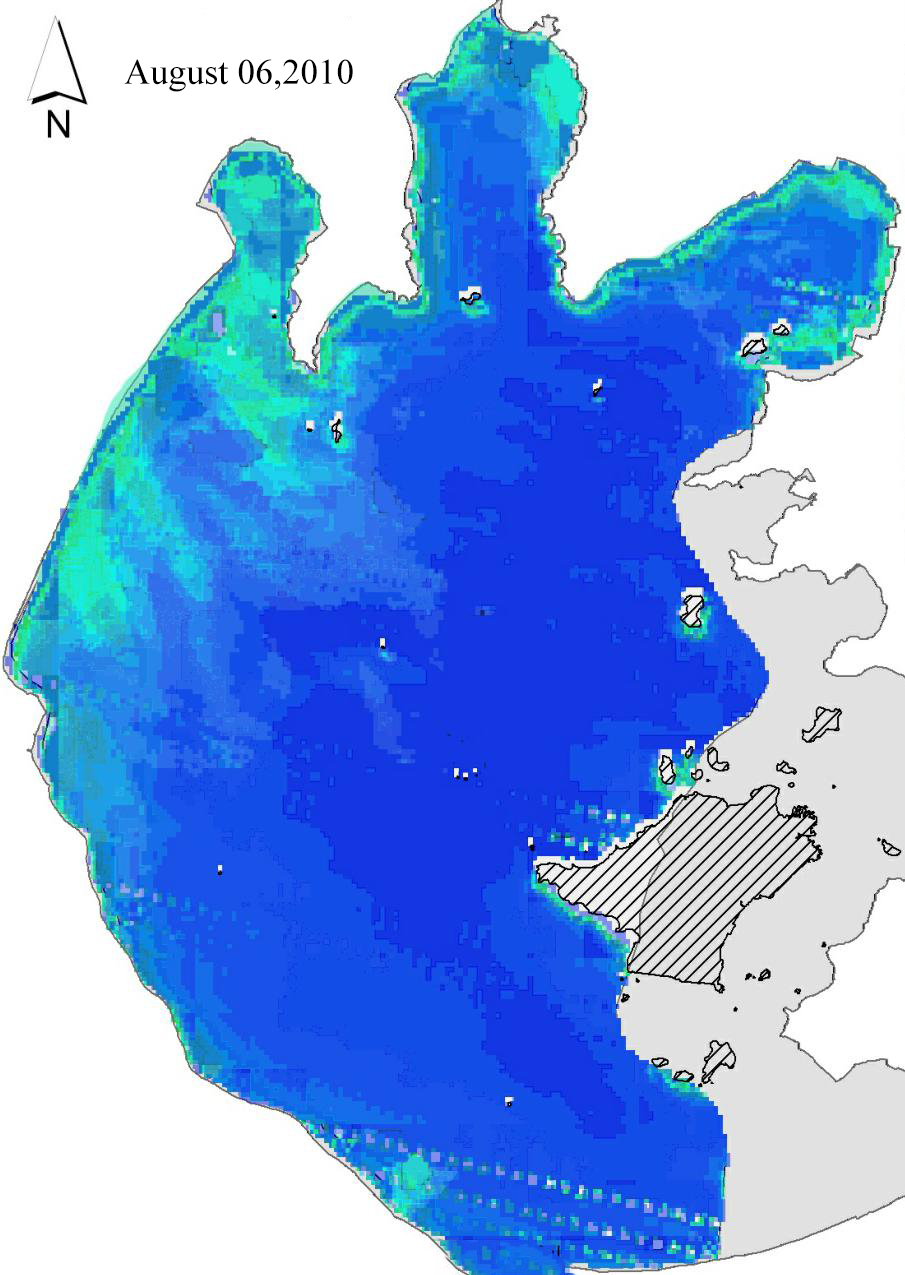

Supplement: Supplemental Information 10 — The data are remote sensing images of chlorophyll a concentration after data scale unification, remote sensing image repair, and time series filling. Remote sensing images of 30 consecutive moments were used as input to the 3D-GAN model. [file peerj-cs-09-1292-s010.zip › 201008060245.jpg]

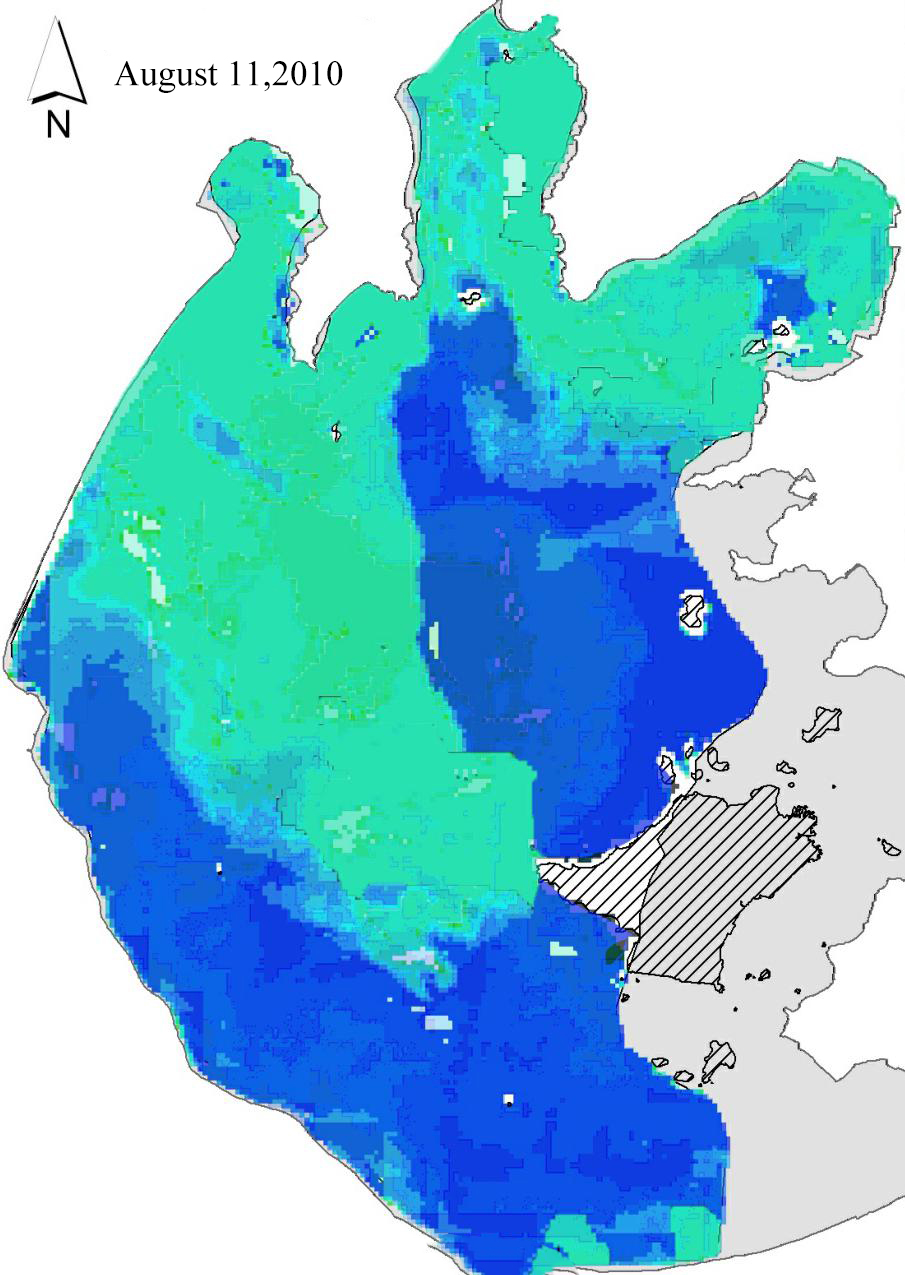

Supplement: Supplemental Information 10 — The data are remote sensing images of chlorophyll a concentration after data scale unification, remote sensing image repair, and time series filling. Remote sensing images of 30 consecutive moments were used as input to the 3D-GAN model. [file peerj-cs-09-1292-s010.zip › 201008110245.jpg]

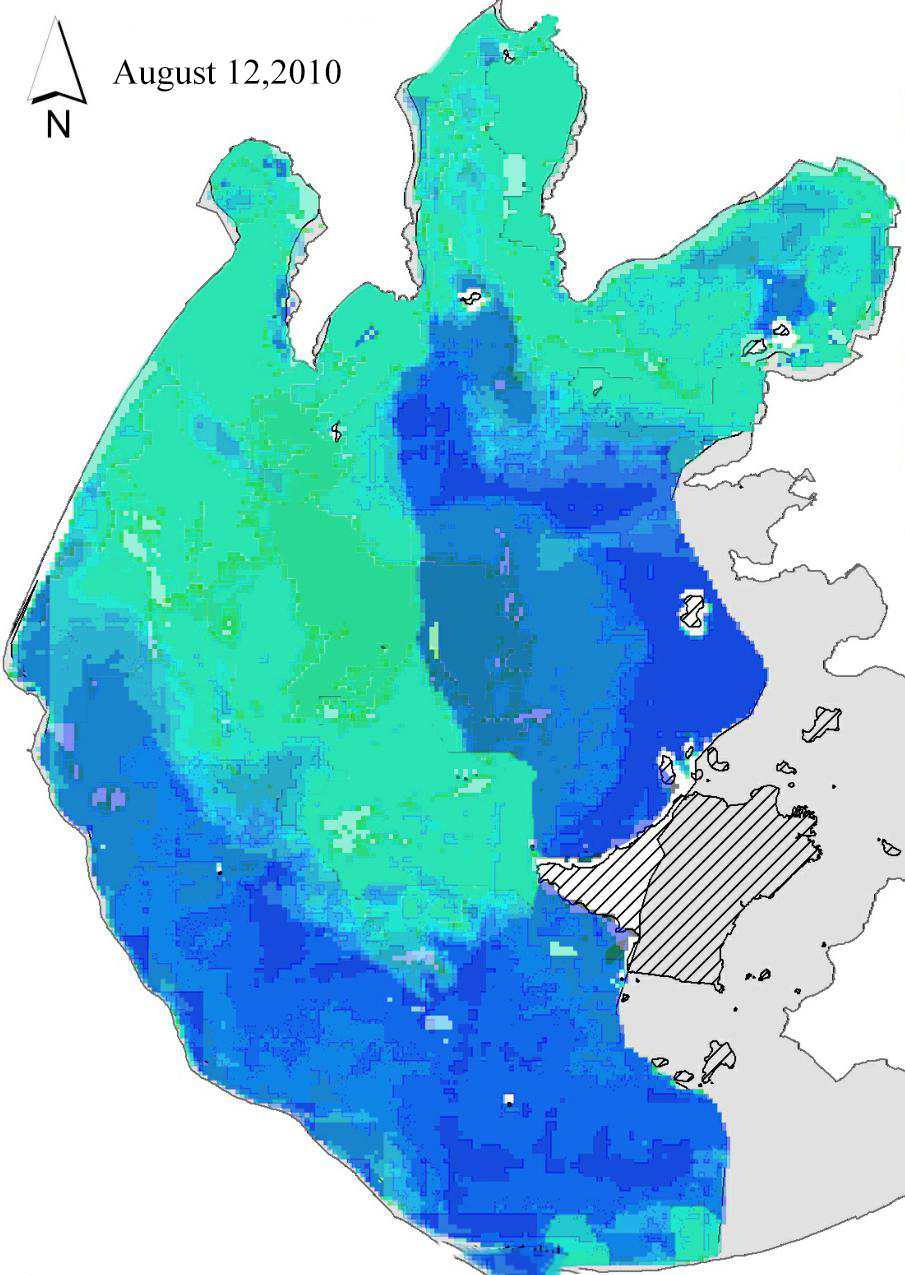

Supplement: Supplemental Information 10 — The data are remote sensing images of chlorophyll a concentration after data scale unification, remote sensing image repair, and time series filling. Remote sensing images of 30 consecutive moments were used as input to the 3D-GAN model. [file peerj-cs-09-1292-s010.zip › 201008120245.jpg]

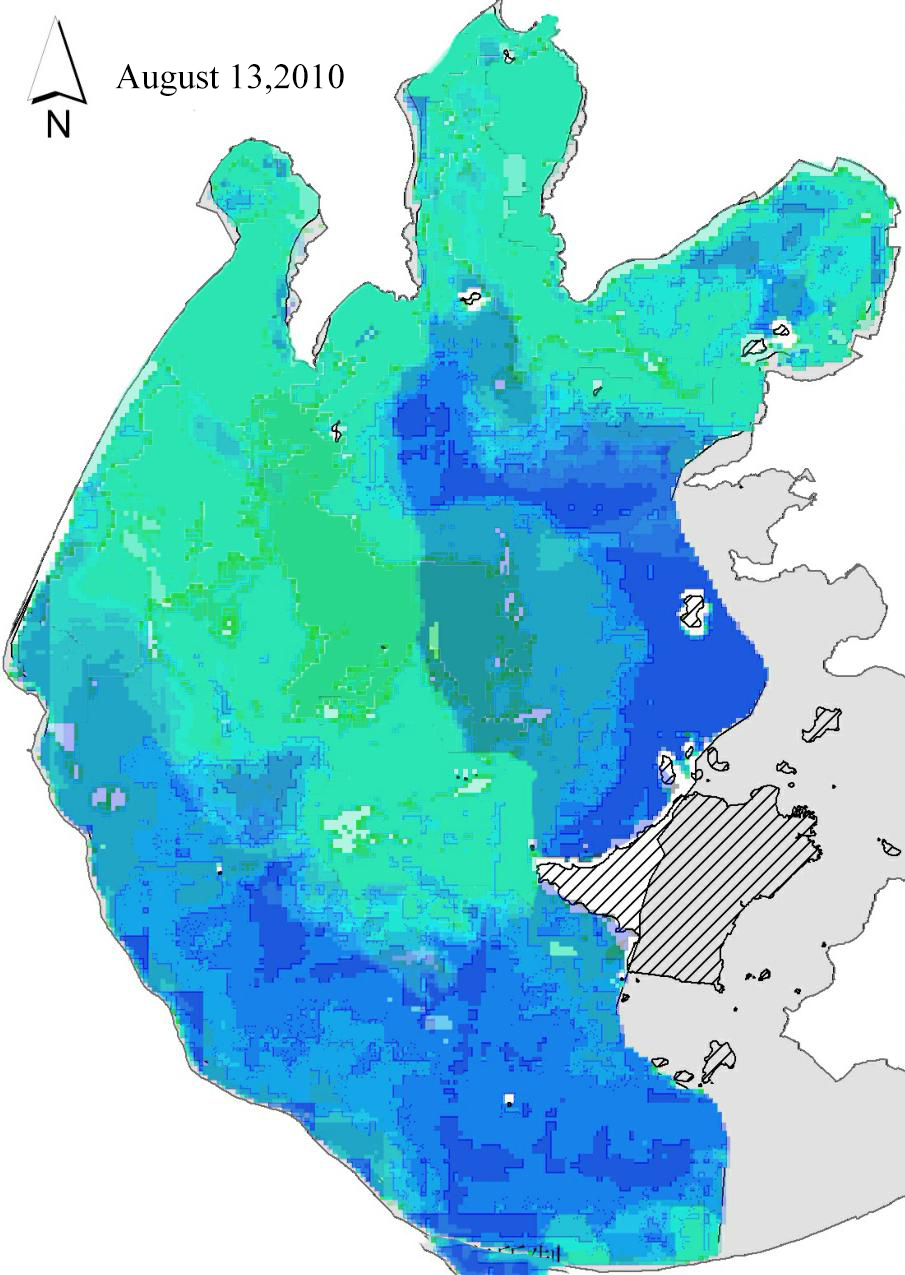

Supplement: Supplemental Information 10 — The data are remote sensing images of chlorophyll a concentration after data scale unification, remote sensing image repair, and time series filling. Remote sensing images of 30 consecutive moments were used as input to the 3D-GAN model. [file peerj-cs-09-1292-s010.zip › 201008130245.jpg]

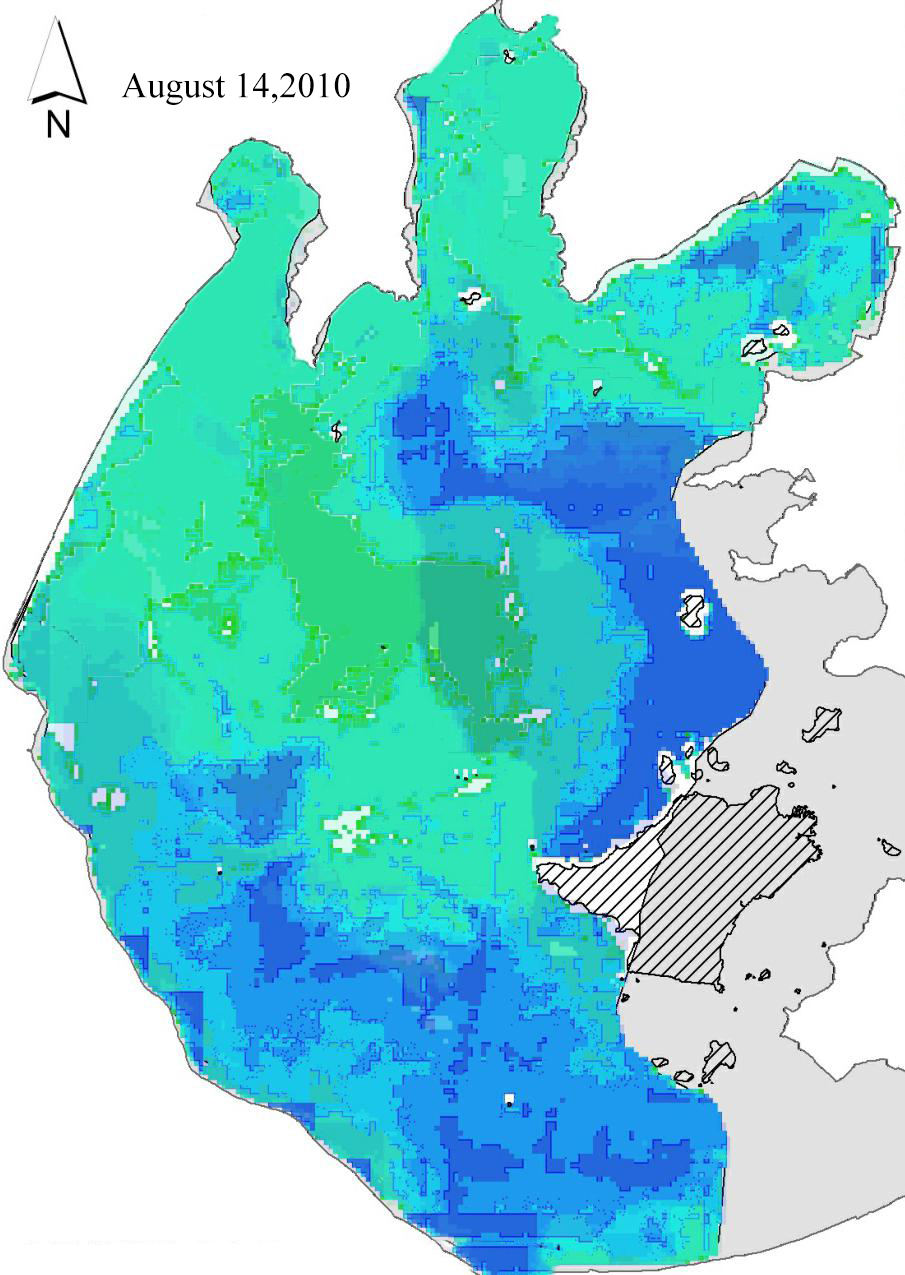

Supplement: Supplemental Information 10 — The data are remote sensing images of chlorophyll a concentration after data scale unification, remote sensing image repair, and time series filling. Remote sensing images of 30 consecutive moments were used as input to the 3D-GAN model. [file peerj-cs-09-1292-s010.zip › 201008140245.jpg]

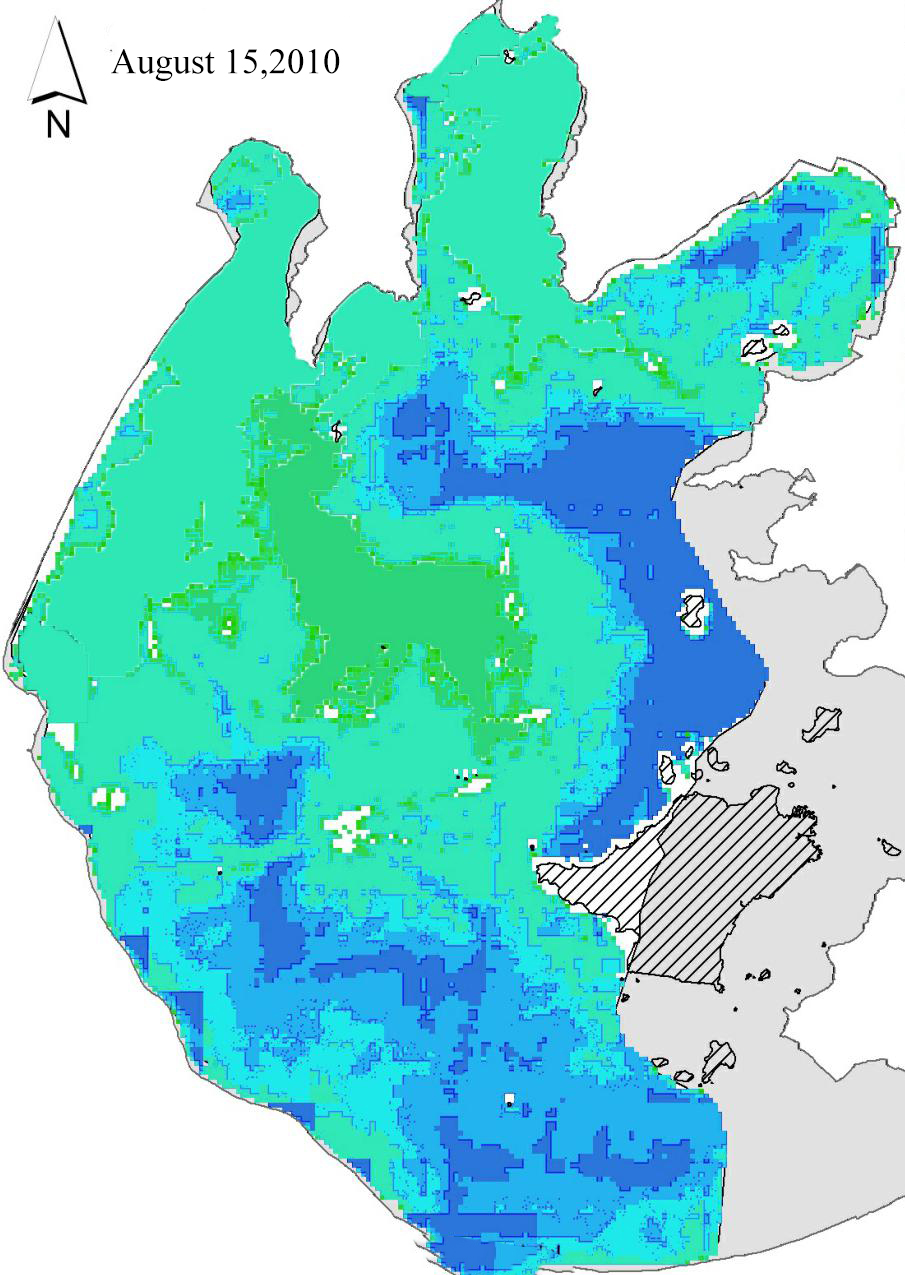

Supplement: Supplemental Information 10 — The data are remote sensing images of chlorophyll a concentration after data scale unification, remote sensing image repair, and time series filling. Remote sensing images of 30 consecutive moments were used as input to the 3D-GAN model. [file peerj-cs-09-1292-s010.zip › 201008150245.jpg]

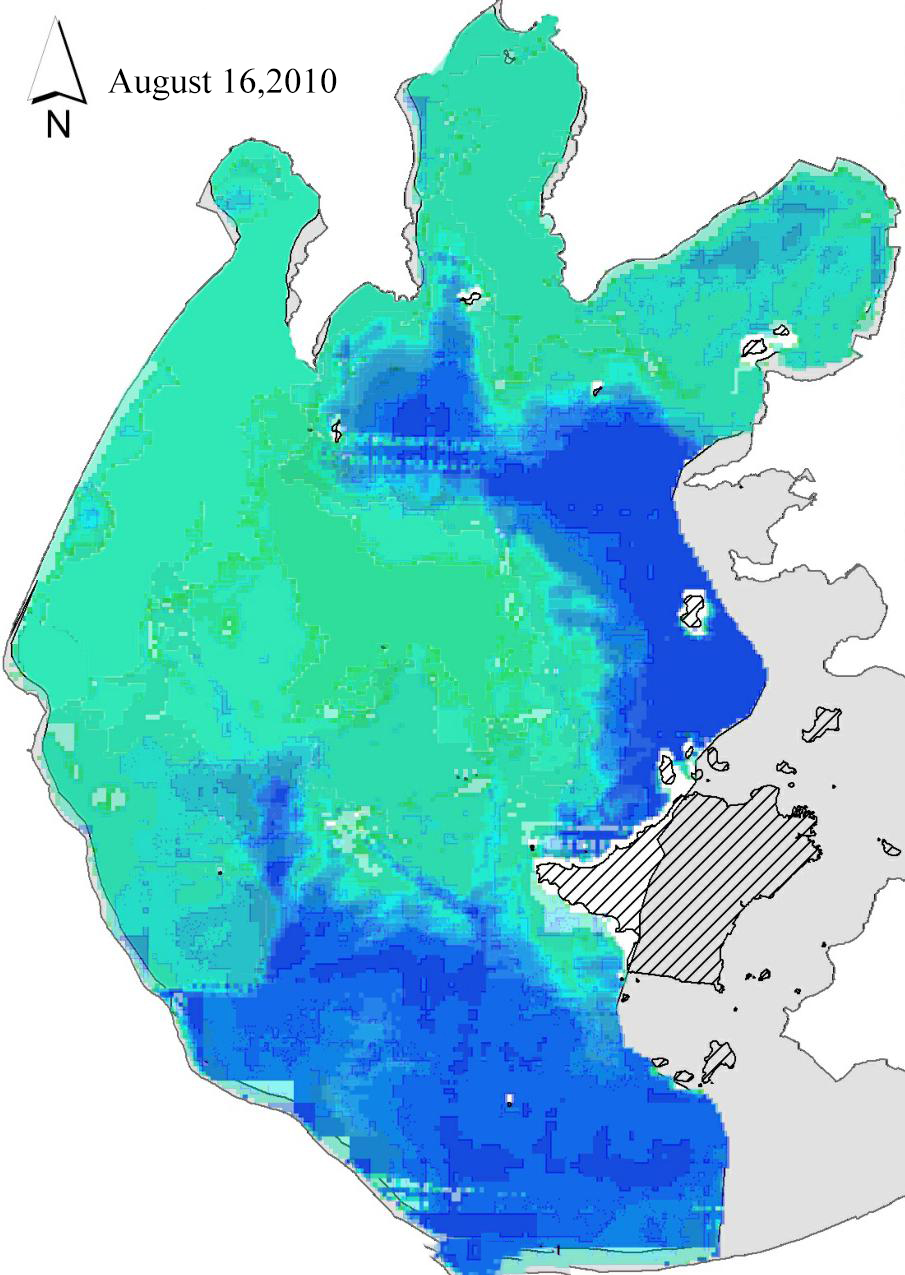

Supplement: Supplemental Information 10 — The data are remote sensing images of chlorophyll a concentration after data scale unification, remote sensing image repair, and time series filling. Remote sensing images of 30 consecutive moments were used as input to the 3D-GAN model. [file peerj-cs-09-1292-s010.zip › 201008160245.jpg]

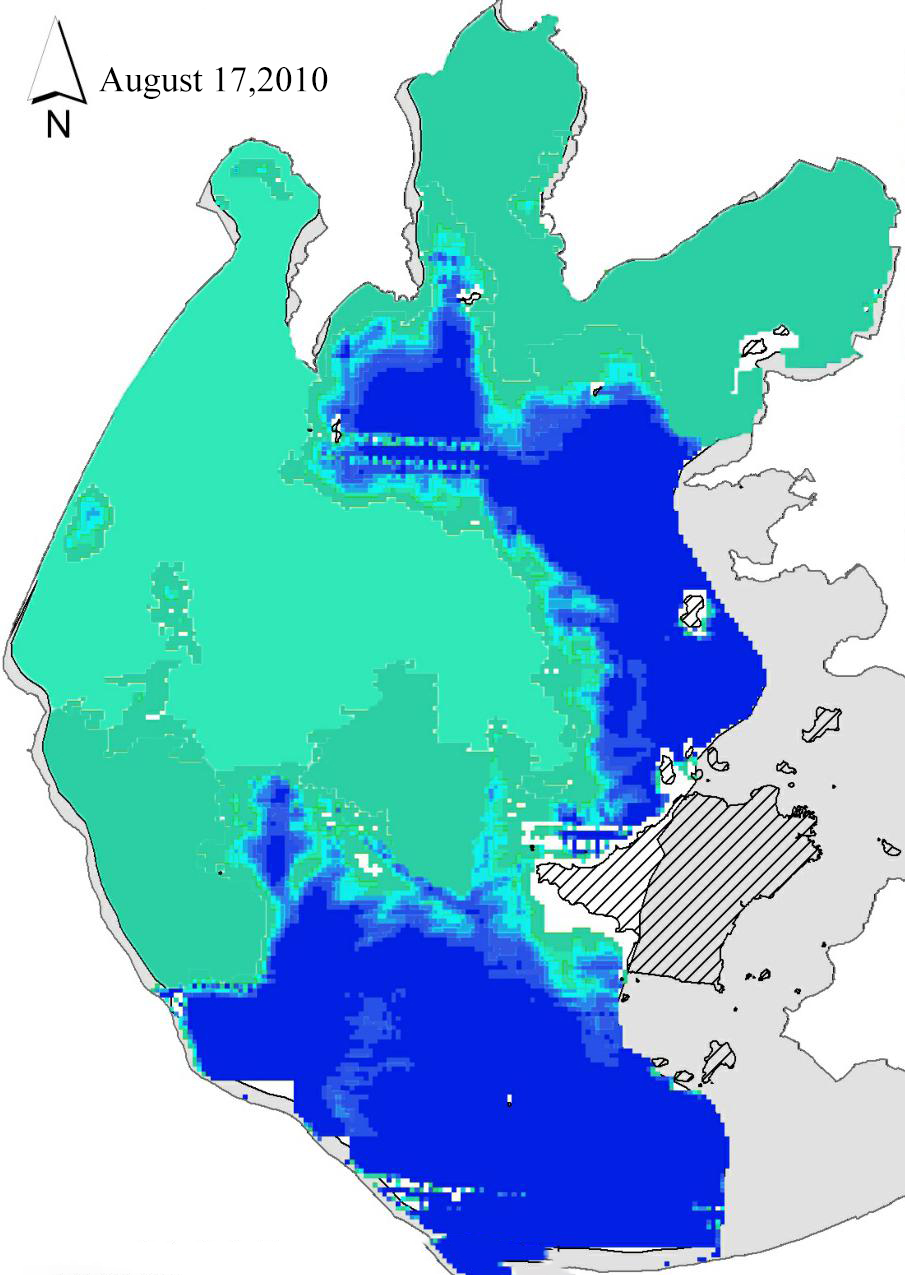

Supplement: Supplemental Information 10 — The data are remote sensing images of chlorophyll a concentration after data scale unification, remote sensing image repair, and time series filling. Remote sensing images of 30 consecutive moments were used as input to the 3D-GAN model. [file peerj-cs-09-1292-s010.zip › 201008170245.jpg]

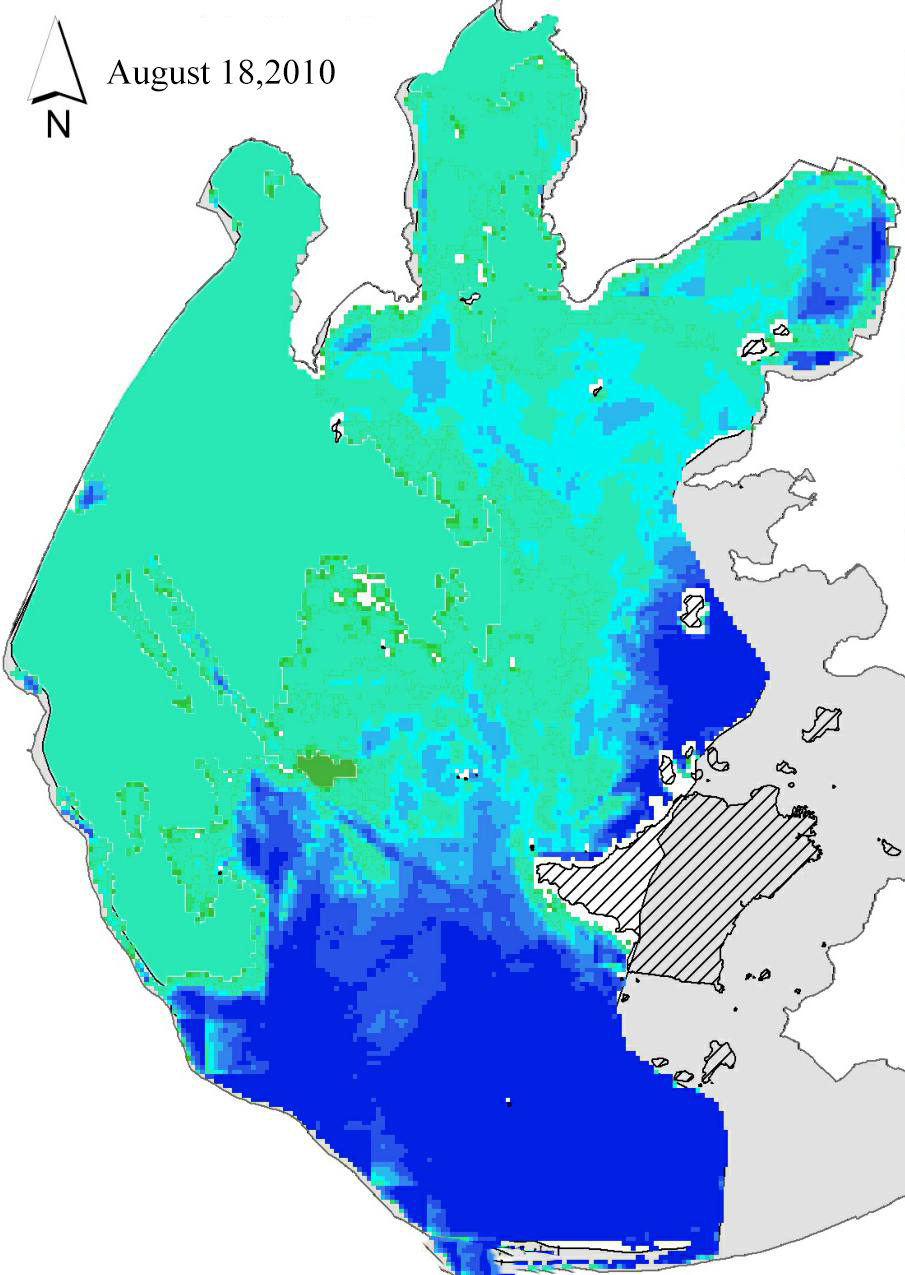

Supplement: Supplemental Information 10 — The data are remote sensing images of chlorophyll a concentration after data scale unification, remote sensing image repair, and time series filling. Remote sensing images of 30 consecutive moments were used as input to the 3D-GAN model. [file peerj-cs-09-1292-s010.zip › 201008180245.jpg]

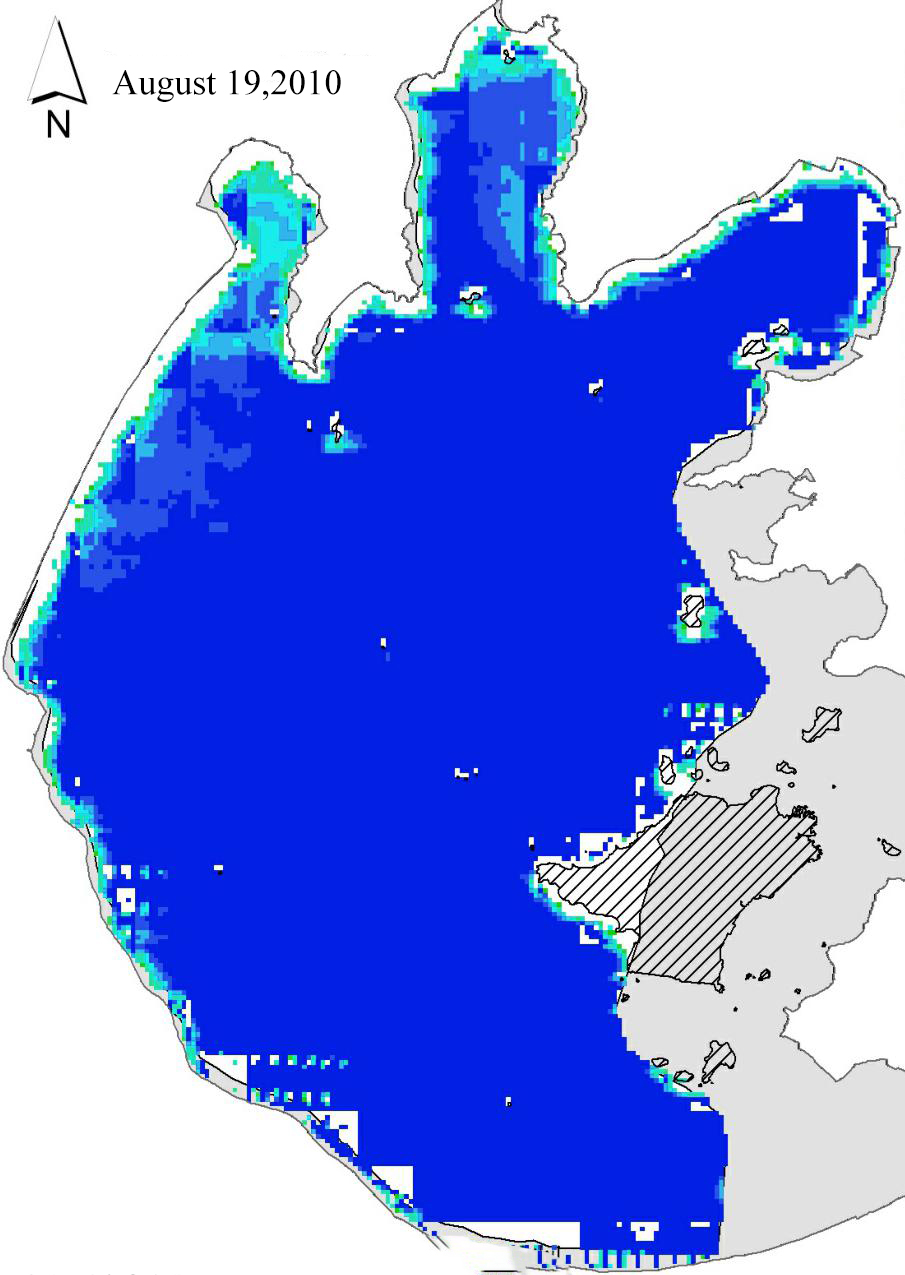

Supplement: Supplemental Information 10 — The data are remote sensing images of chlorophyll a concentration after data scale unification, remote sensing image repair, and time series filling. Remote sensing images of 30 consecutive moments were used as input to the 3D-GAN model. [file peerj-cs-09-1292-s010.zip › 201008190245.jpg]

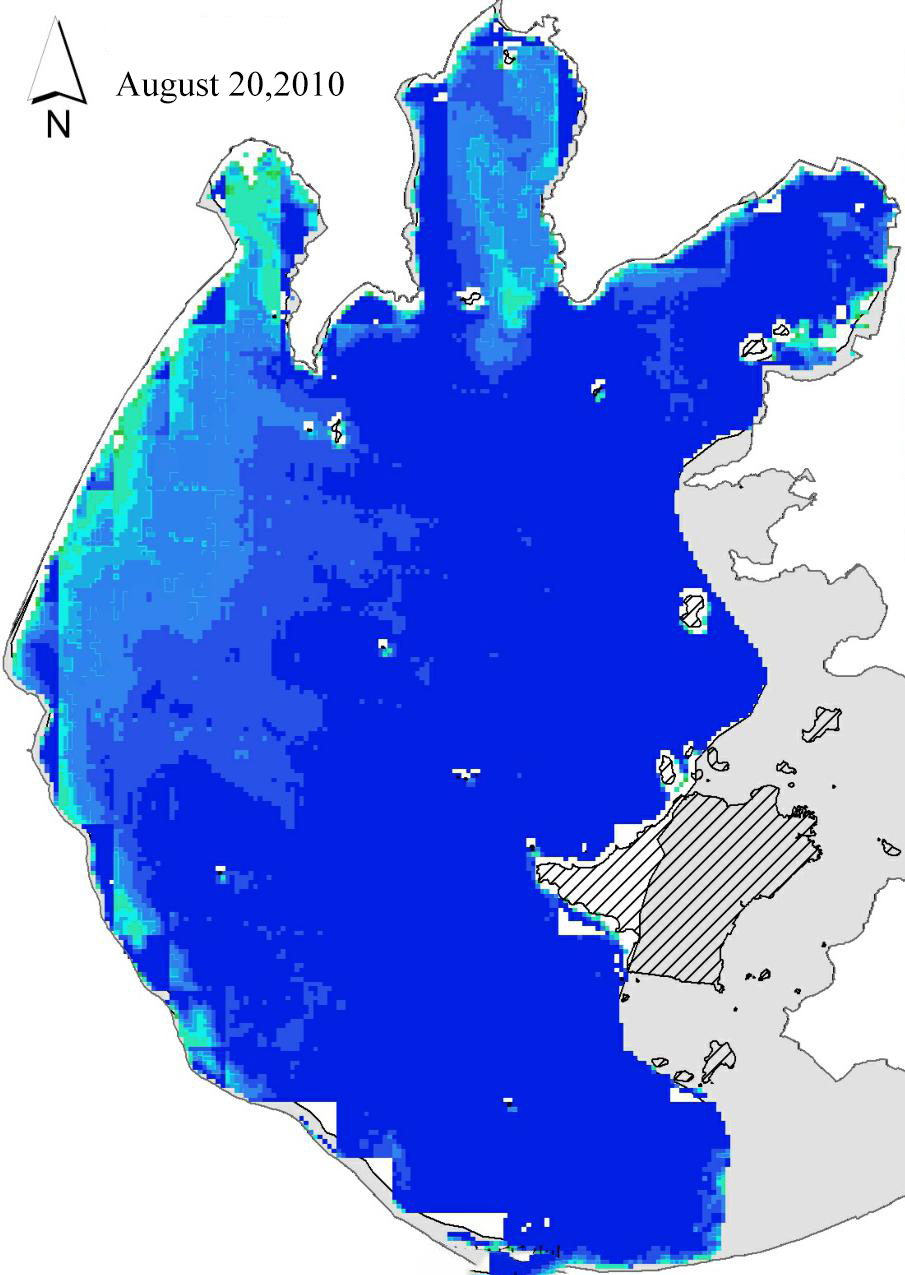

Supplement: Supplemental Information 10 — The data are remote sensing images of chlorophyll a concentration after data scale unification, remote sensing image repair, and time series filling. Remote sensing images of 30 consecutive moments were used as input to the 3D-GAN model. [file peerj-cs-09-1292-s010.zip › 201008200245.jpg]

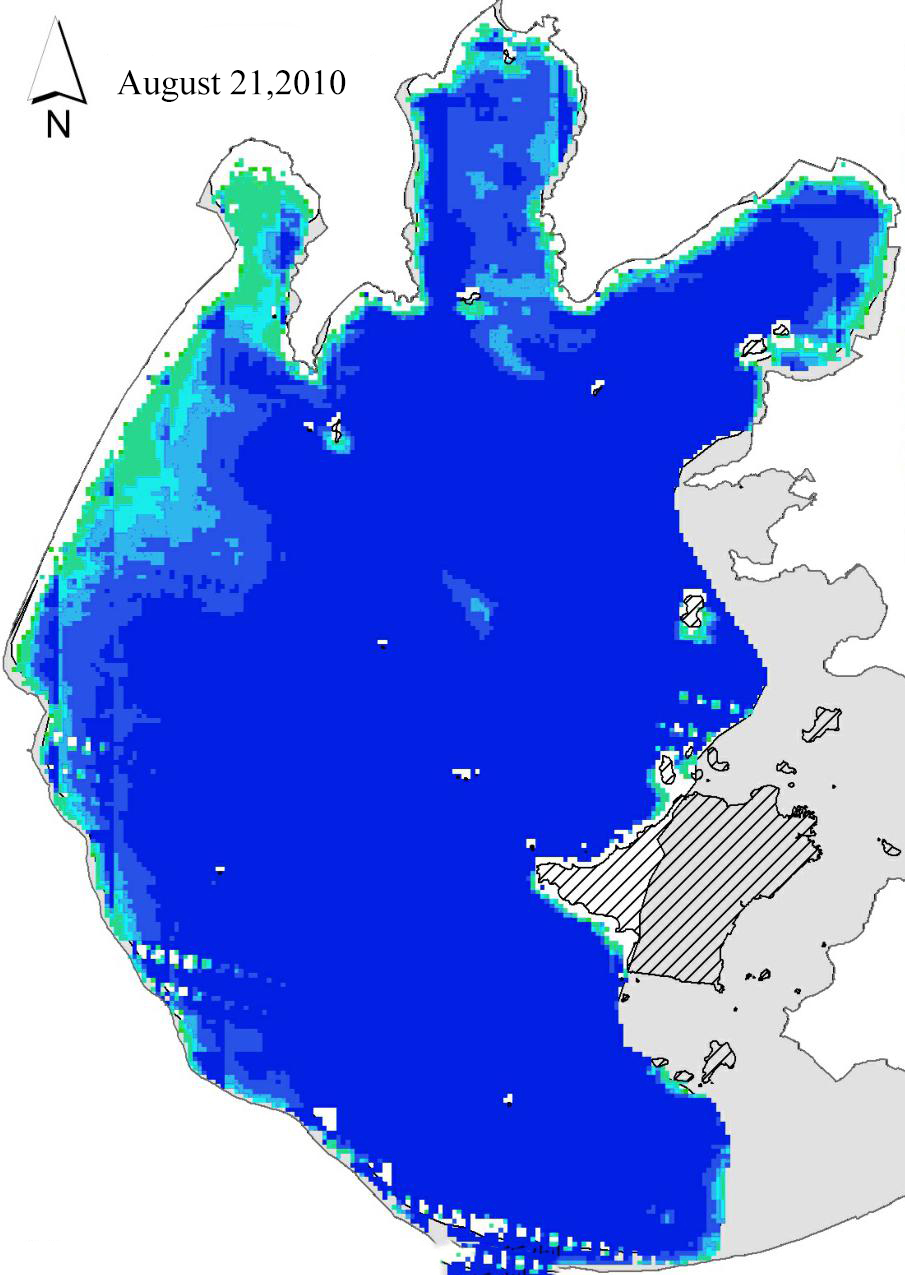

Supplement: Supplemental Information 10 — The data are remote sensing images of chlorophyll a concentration after data scale unification, remote sensing image repair, and time series filling. Remote sensing images of 30 consecutive moments were used as input to the 3D-GAN model. [file peerj-cs-09-1292-s010.zip › 201008210245.jpg]

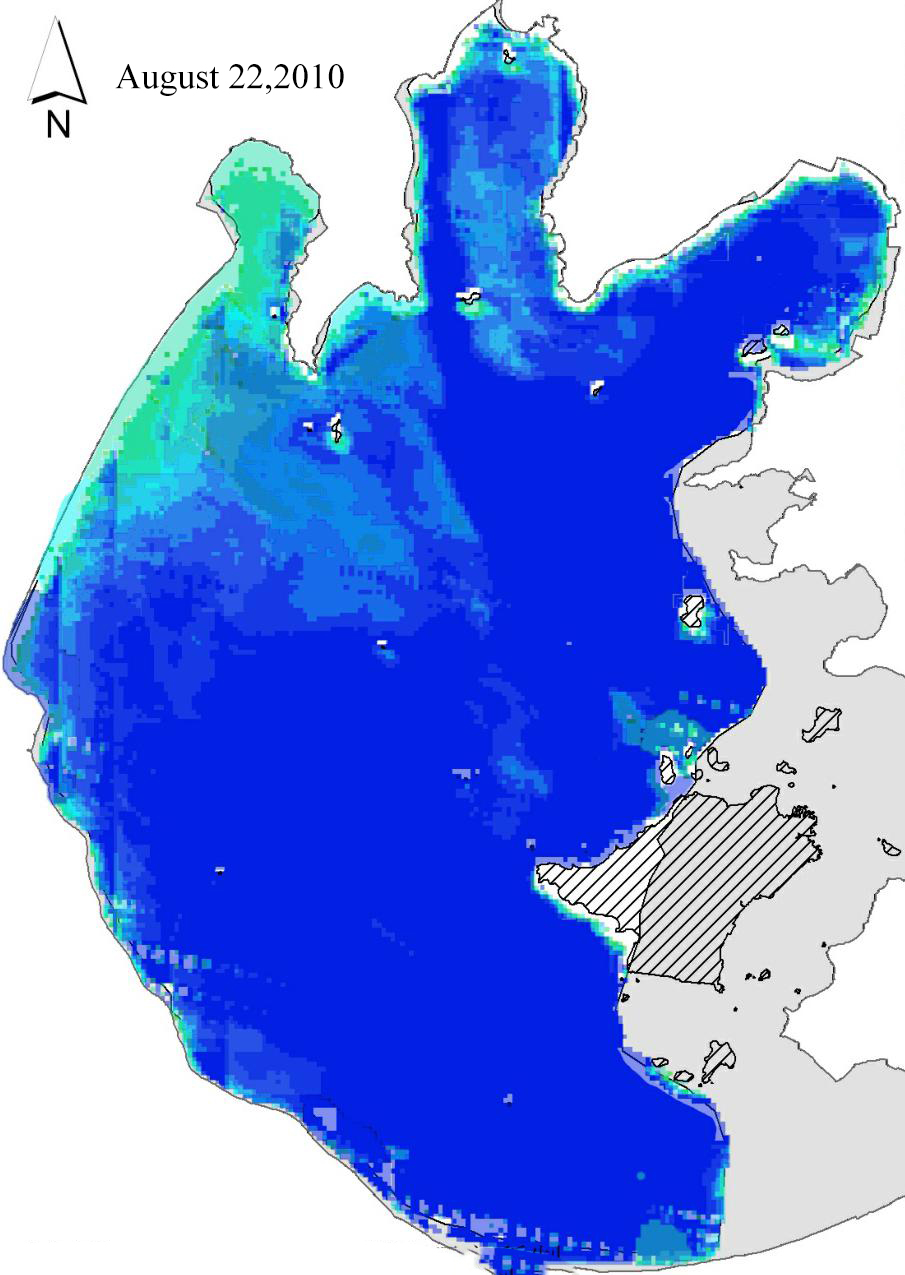

Supplement: Supplemental Information 10 — The data are remote sensing images of chlorophyll a concentration after data scale unification, remote sensing image repair, and time series filling. Remote sensing images of 30 consecutive moments were used as input to the 3D-GAN model. [file peerj-cs-09-1292-s010.zip › 201008220245.jpg]

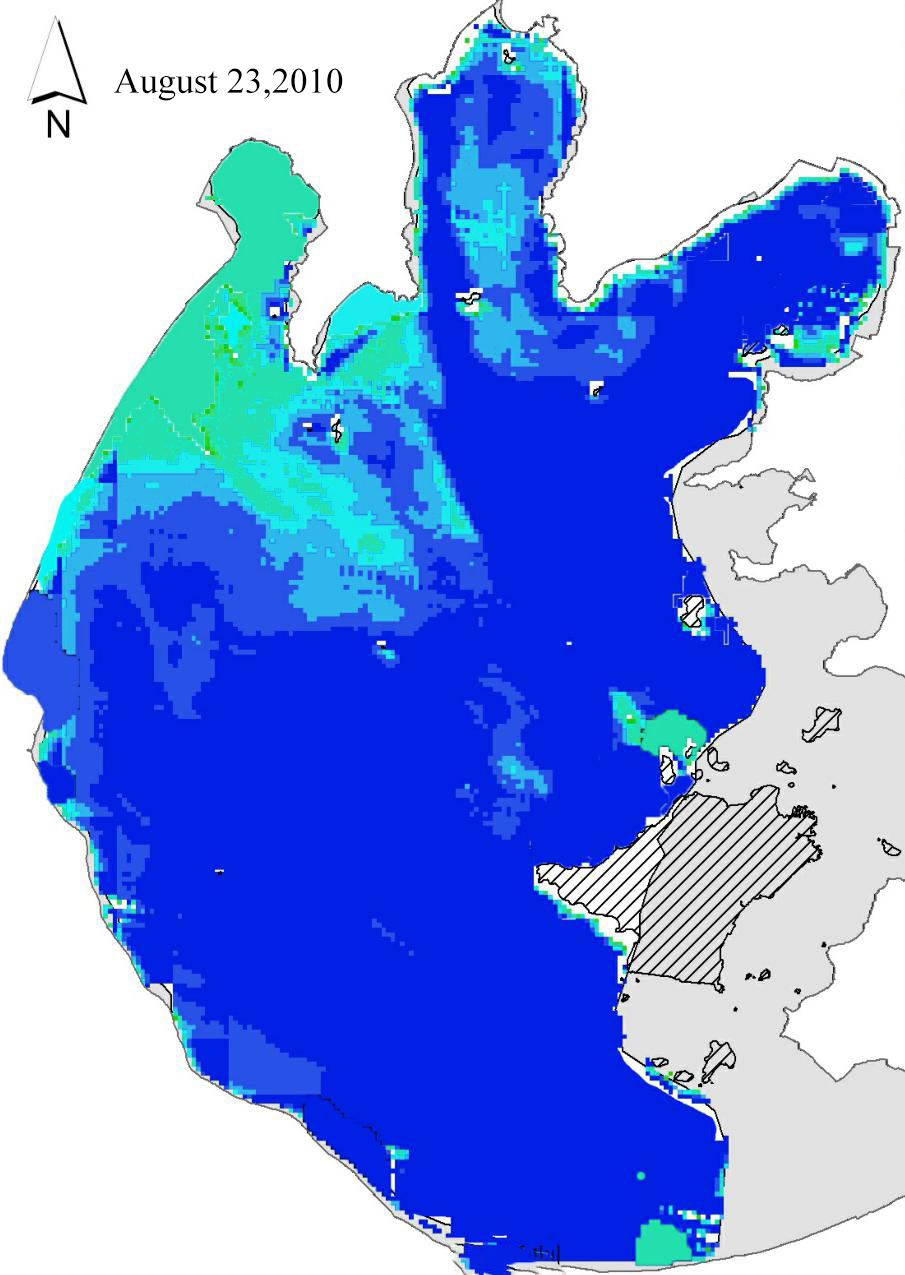

Supplement: Supplemental Information 10 — The data are remote sensing images of chlorophyll a concentration after data scale unification, remote sensing image repair, and time series filling. Remote sensing images of 30 consecutive moments were used as input to the 3D-GAN model. [file peerj-cs-09-1292-s010.zip › 201008230245.jpg]

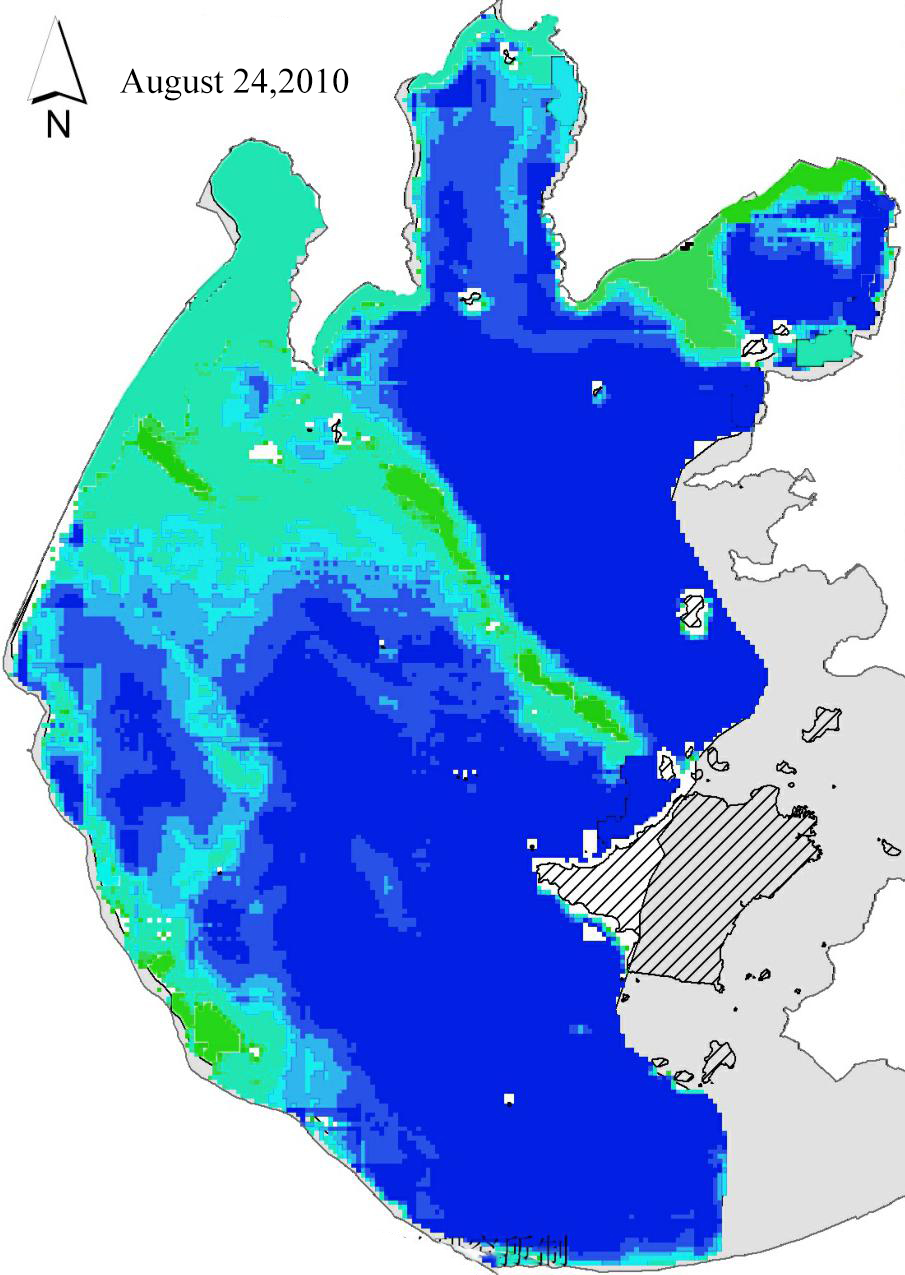

Supplement: Supplemental Information 10 — The data are remote sensing images of chlorophyll a concentration after data scale unification, remote sensing image repair, and time series filling. Remote sensing images of 30 consecutive moments were used as input to the 3D-GAN model. [file peerj-cs-09-1292-s010.zip › 201008240245.jpg]

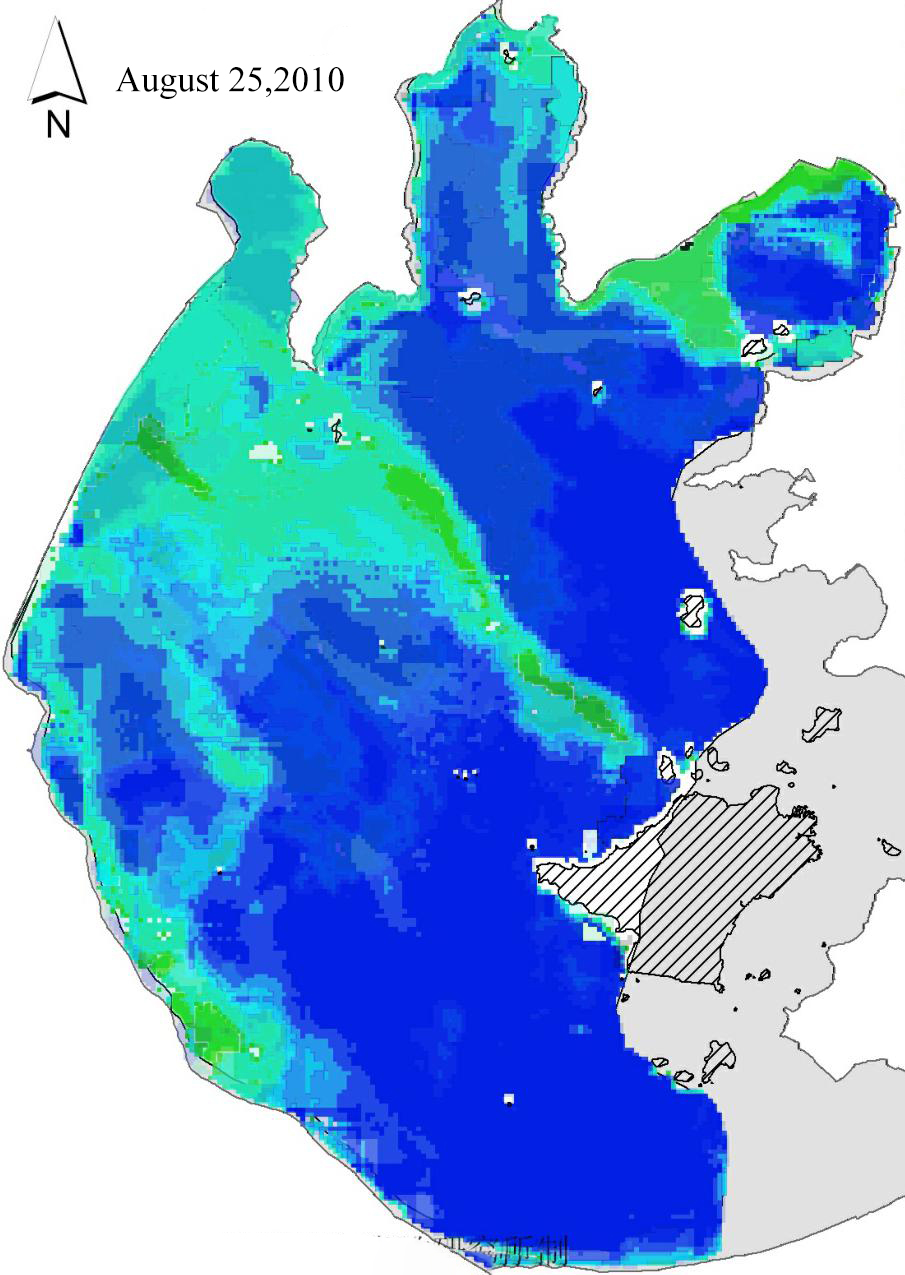

Supplement: Supplemental Information 10 — The data are remote sensing images of chlorophyll a concentration after data scale unification, remote sensing image repair, and time series filling. Remote sensing images of 30 consecutive moments were used as input to the 3D-GAN model. [file peerj-cs-09-1292-s010.zip › 201008250245.jpg]

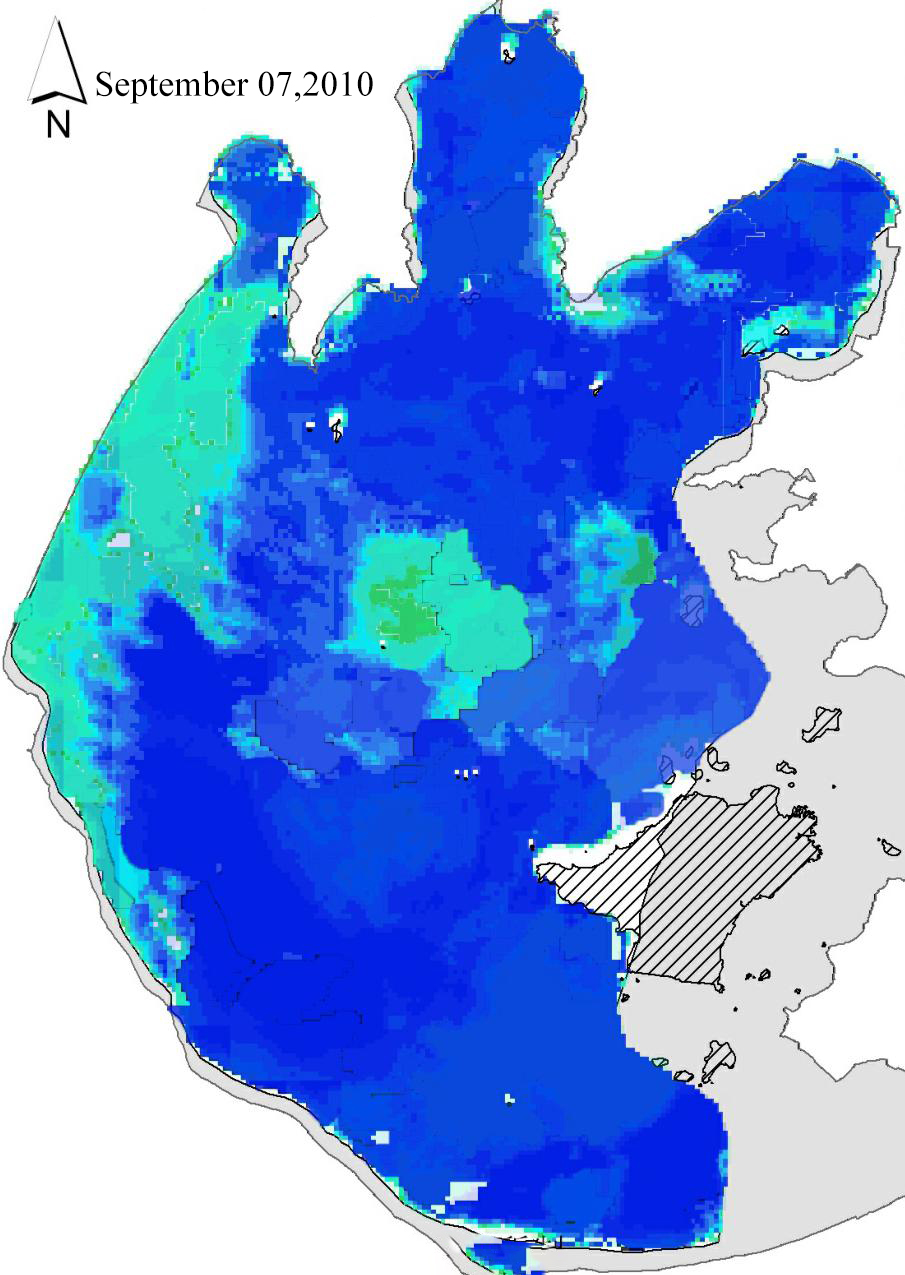

Supplement: Supplemental Information 11 — The data are remote sensing images of chlorophyll a concentration after data scale unification, remote sensing image repair, and time series filling. Remote sensing images of 30 consecutive moments were used as input to the 3D-GAN model. [file peerj-cs-09-1292-s011.zip › 201009070245.jpg]

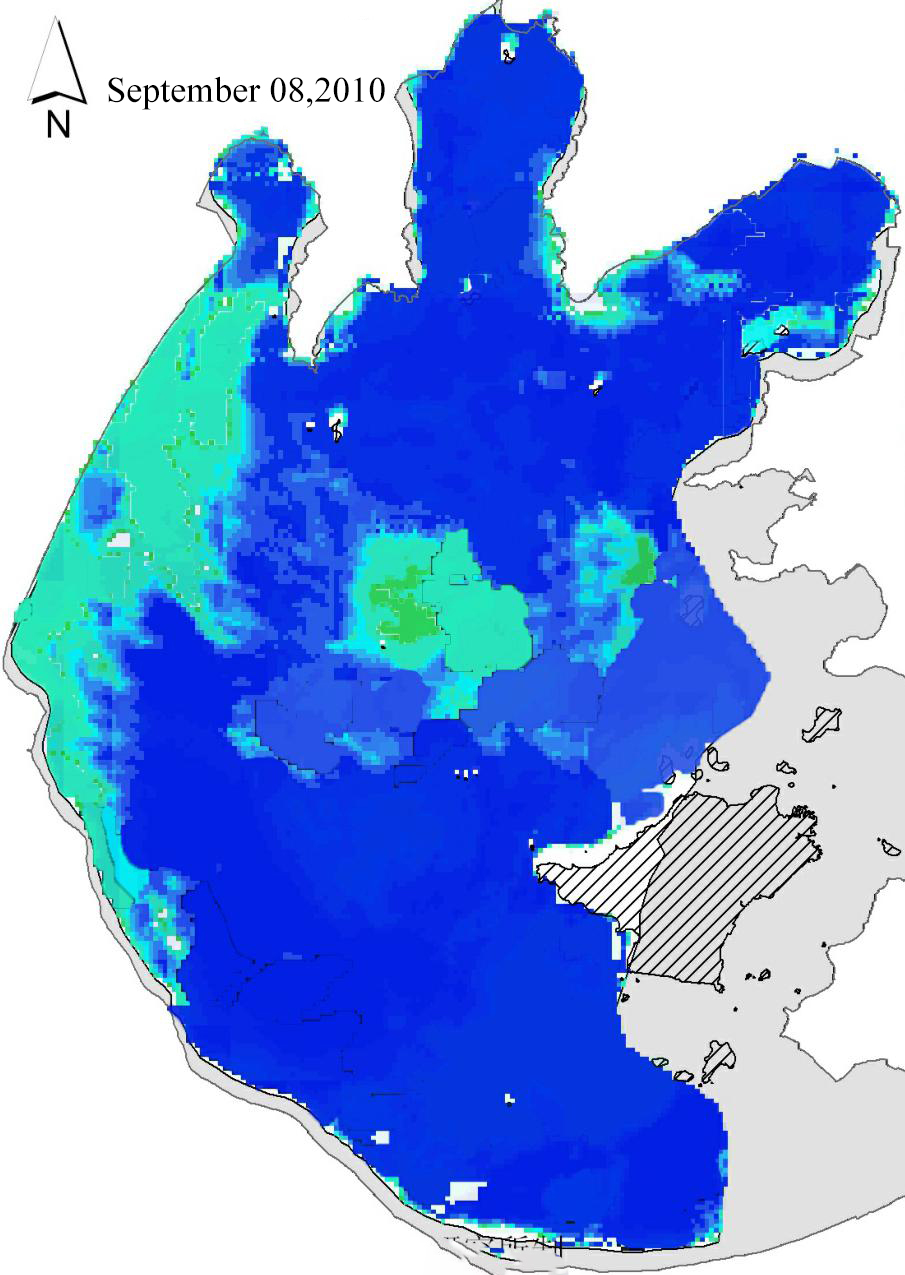

Supplement: Supplemental Information 11 — The data are remote sensing images of chlorophyll a concentration after data scale unification, remote sensing image repair, and time series filling. Remote sensing images of 30 consecutive moments were used as input to the 3D-GAN model. [file peerj-cs-09-1292-s011.zip › 201009080245.jpg]

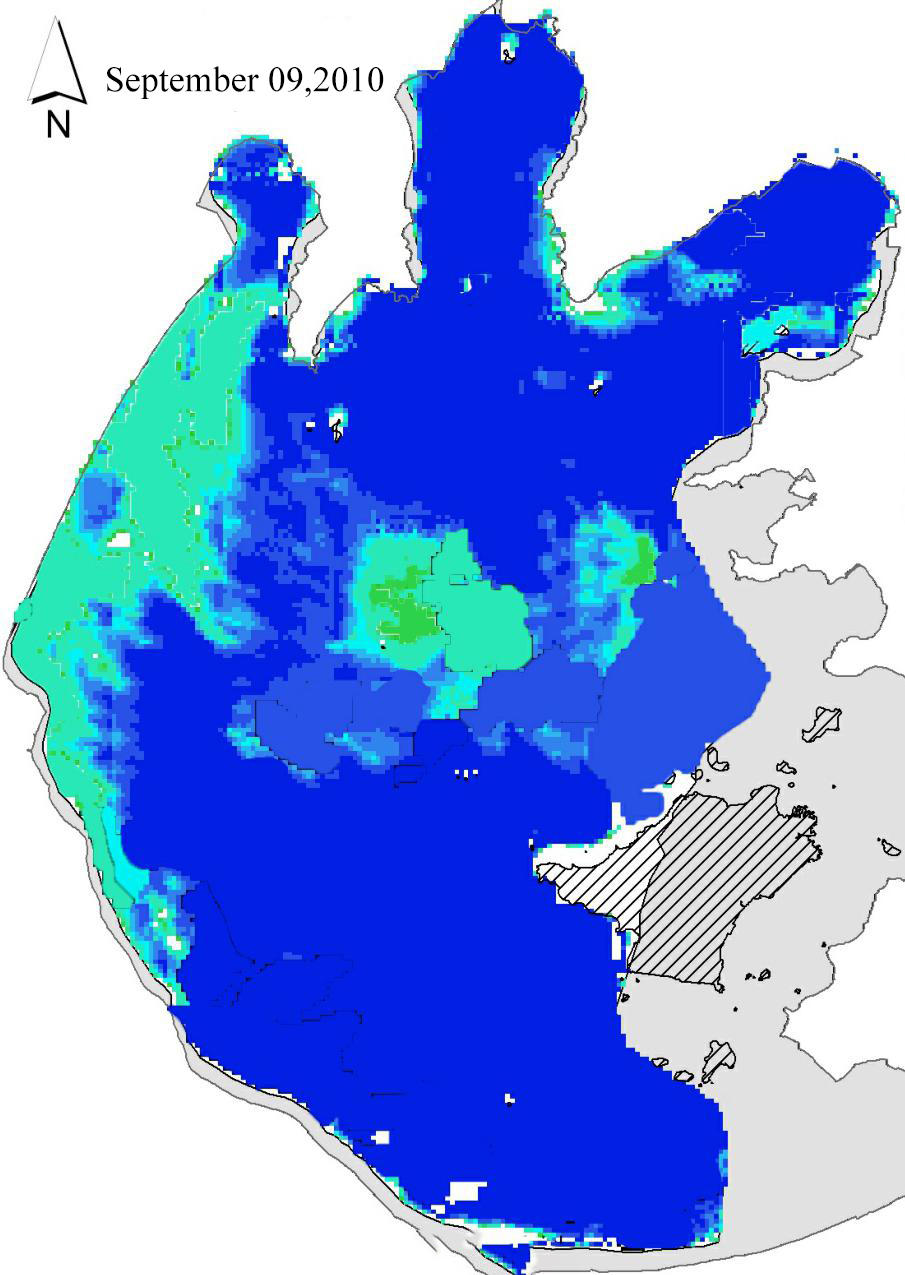

Supplement: Supplemental Information 11 — The data are remote sensing images of chlorophyll a concentration after data scale unification, remote sensing image repair, and time series filling. Remote sensing images of 30 consecutive moments were used as input to the 3D-GAN model. [file peerj-cs-09-1292-s011.zip › 201009090245.jpg]

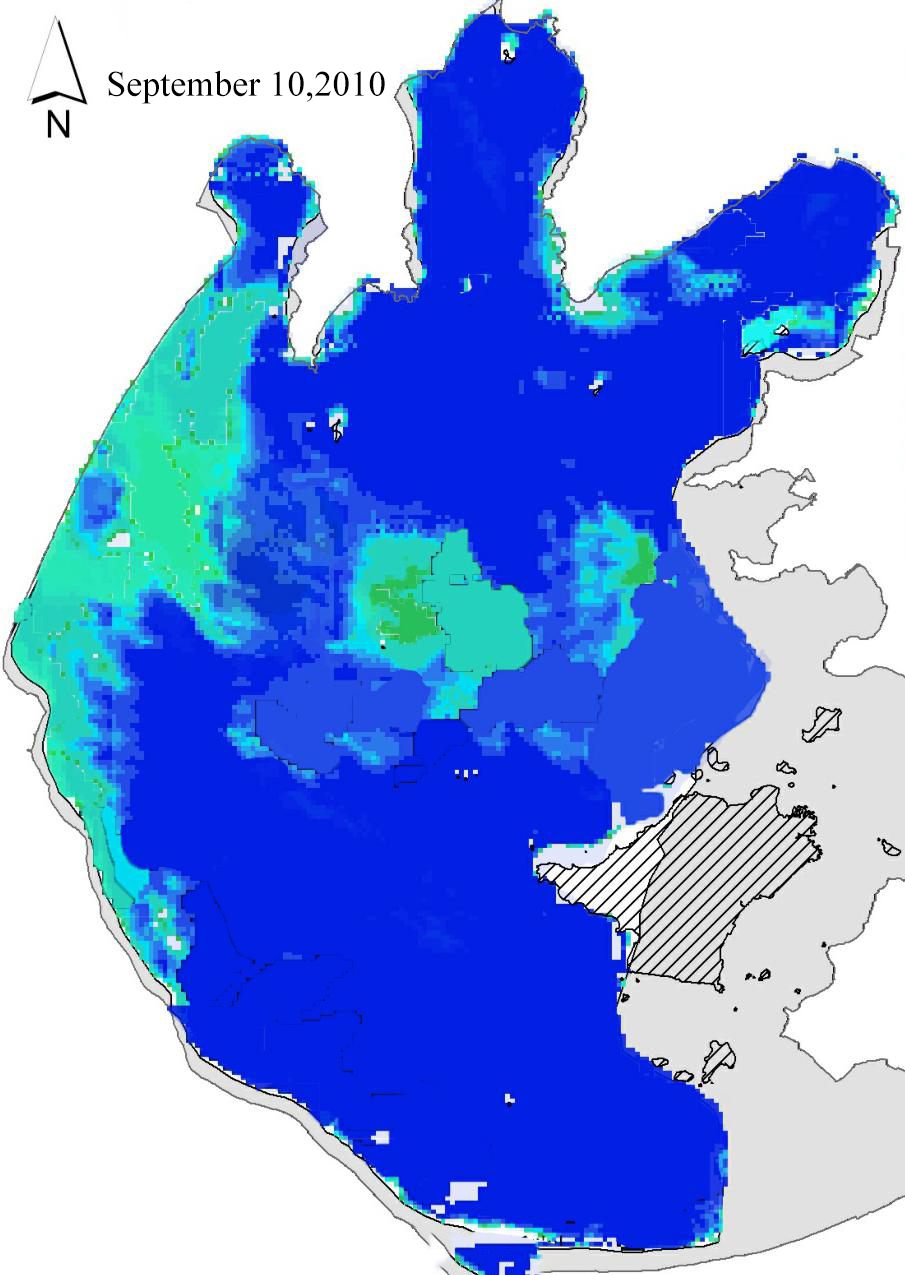

Supplement: Supplemental Information 11 — The data are remote sensing images of chlorophyll a concentration after data scale unification, remote sensing image repair, and time series filling. Remote sensing images of 30 consecutive moments were used as input to the 3D-GAN model. [file peerj-cs-09-1292-s011.zip › 201009100245.jpg]

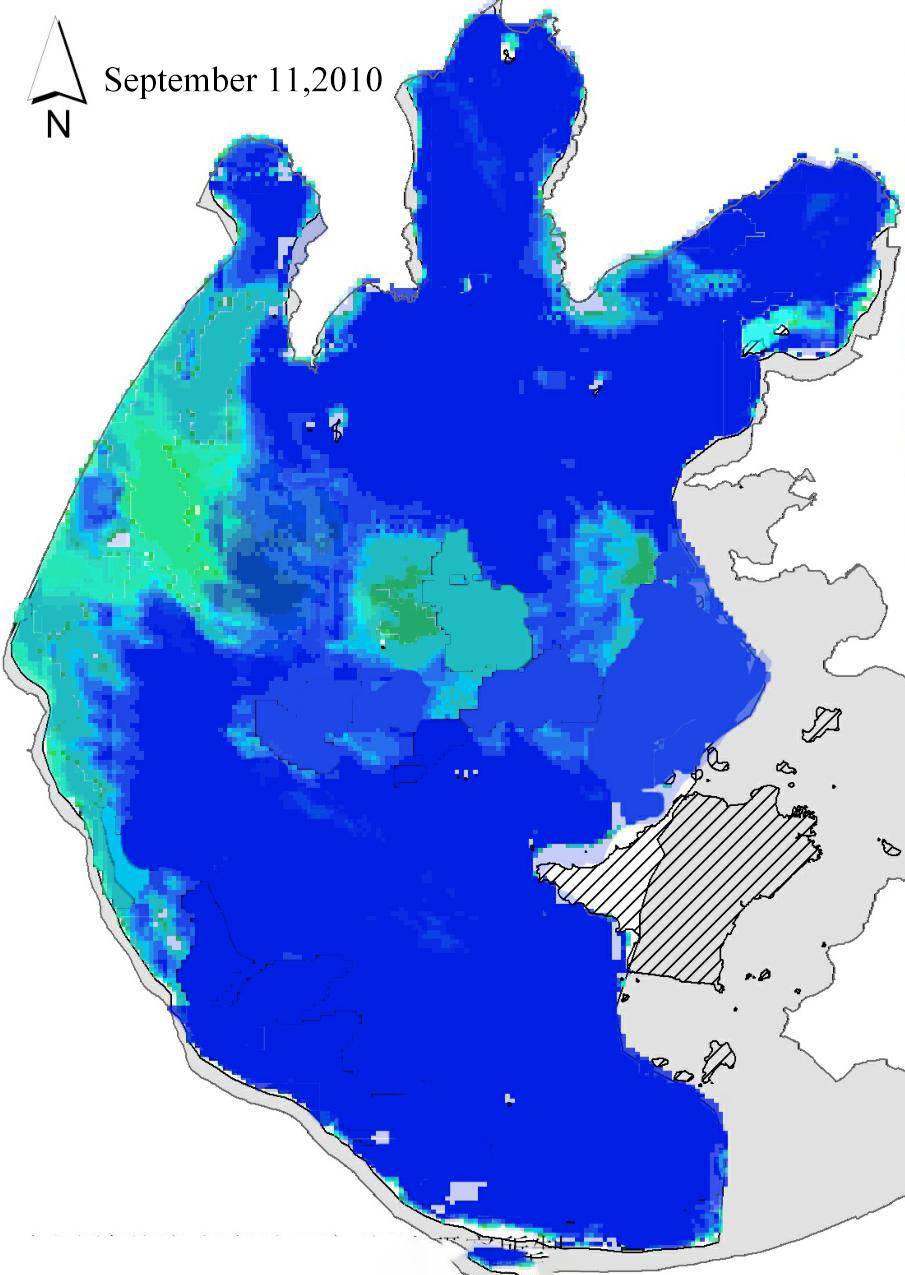

Supplement: Supplemental Information 11 — The data are remote sensing images of chlorophyll a concentration after data scale unification, remote sensing image repair, and time series filling. Remote sensing images of 30 consecutive moments were used as input to the 3D-GAN model. [file peerj-cs-09-1292-s011.zip › 201009110245.jpg]

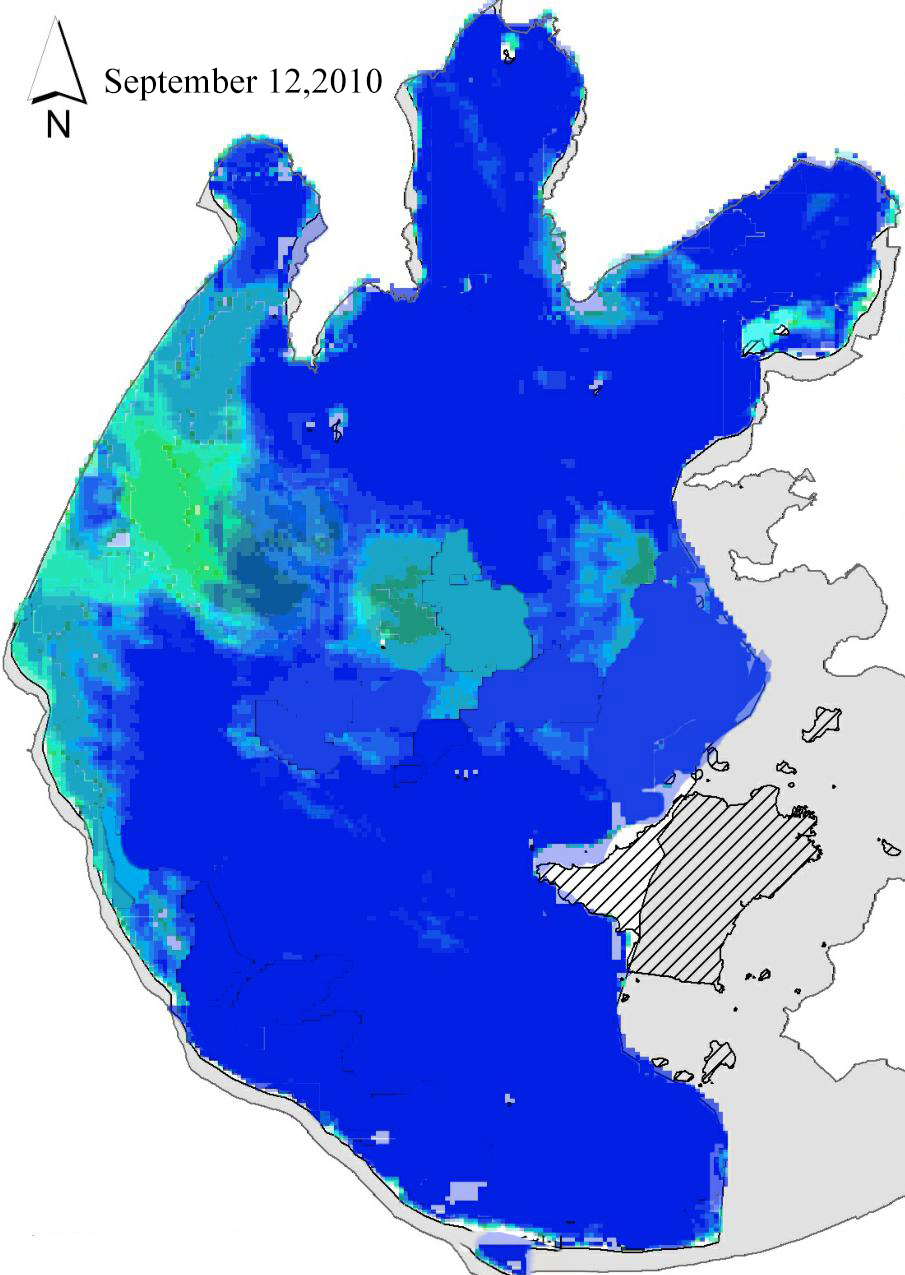

Supplement: Supplemental Information 11 — The data are remote sensing images of chlorophyll a concentration after data scale unification, remote sensing image repair, and time series filling. Remote sensing images of 30 consecutive moments were used as input to the 3D-GAN model. [file peerj-cs-09-1292-s011.zip › 201009120245.jpg]

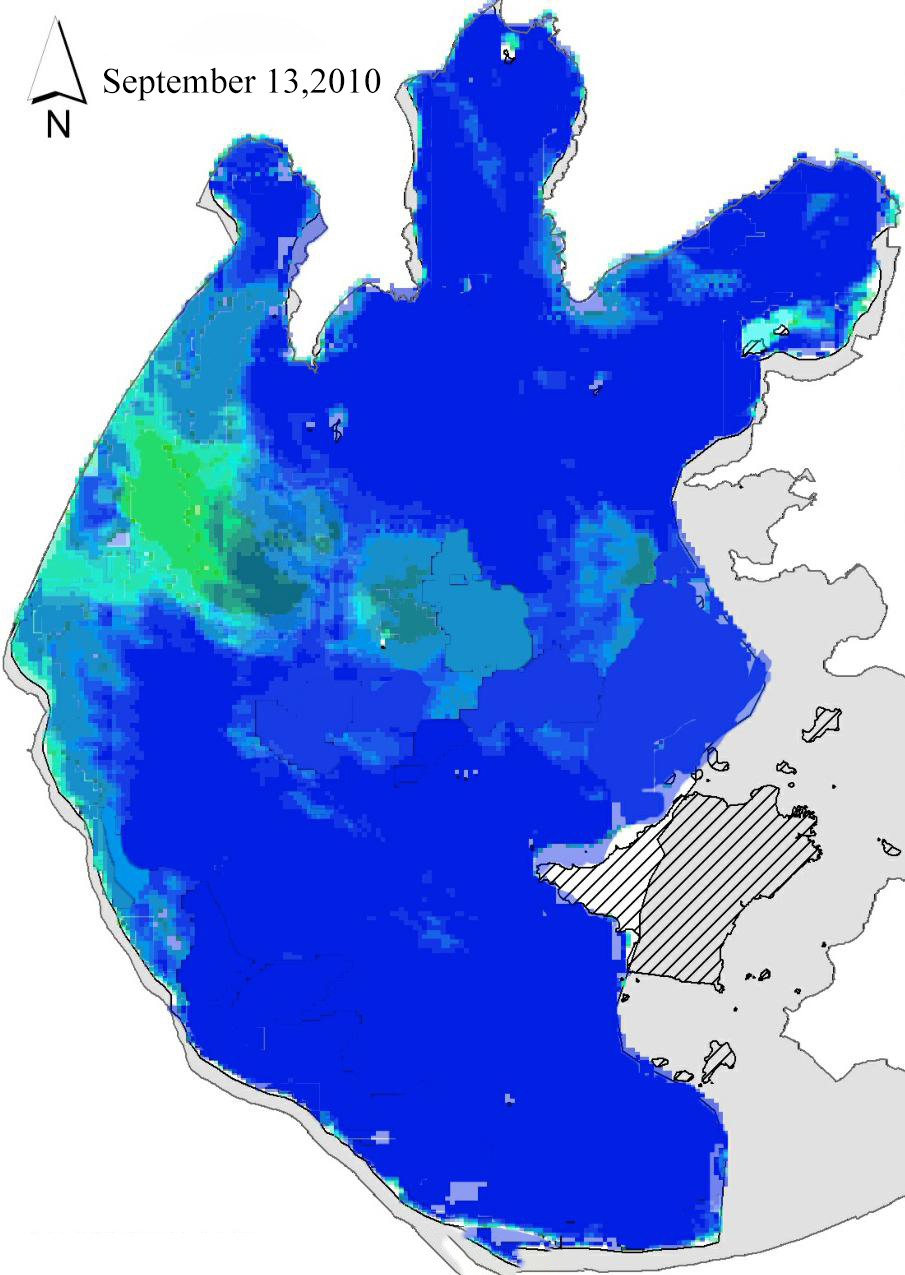

Supplement: Supplemental Information 11 — The data are remote sensing images of chlorophyll a concentration after data scale unification, remote sensing image repair, and time series filling. Remote sensing images of 30 consecutive moments were used as input to the 3D-GAN model. [file peerj-cs-09-1292-s011.zip › 201009130245.jpg]

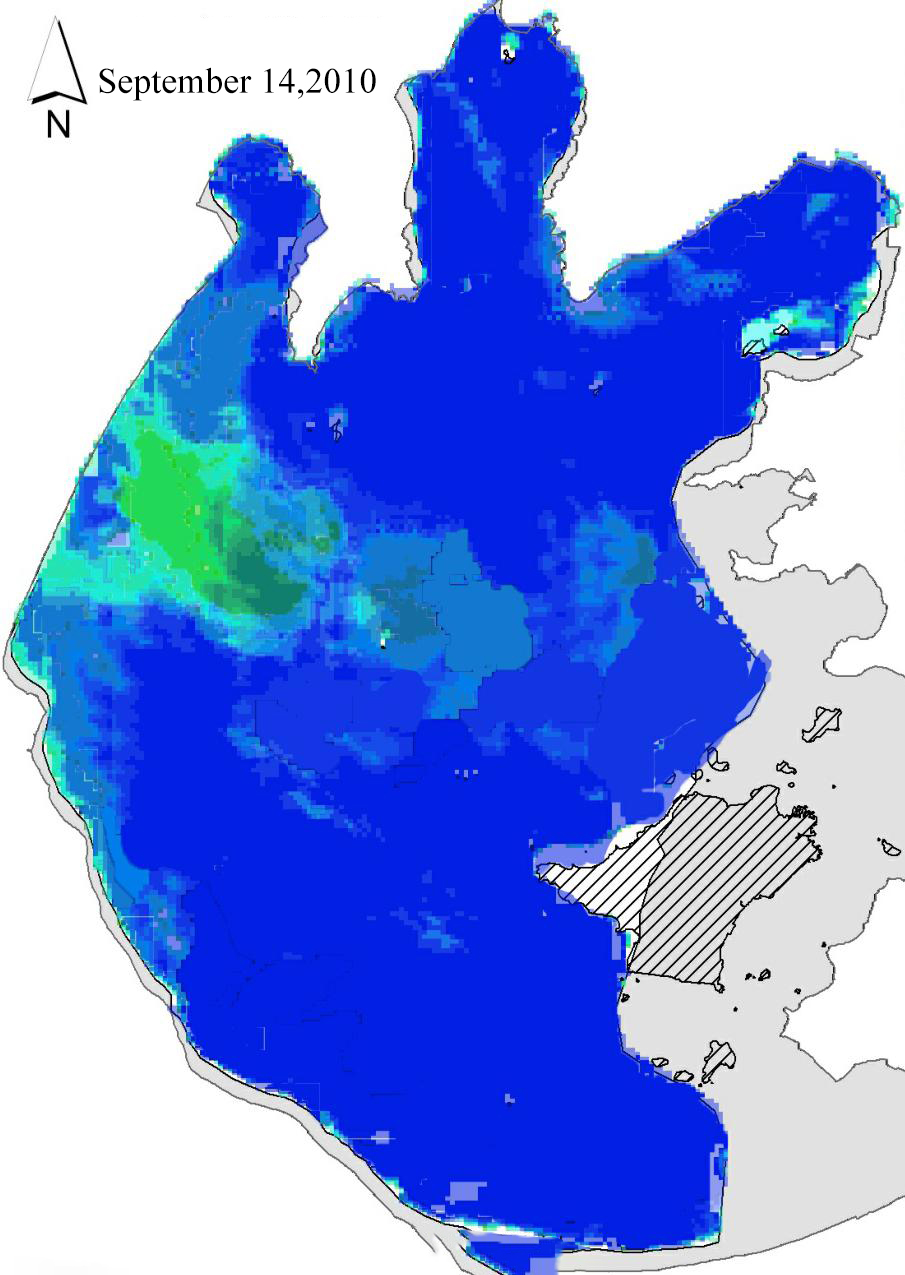

Supplement: Supplemental Information 11 — The data are remote sensing images of chlorophyll a concentration after data scale unification, remote sensing image repair, and time series filling. Remote sensing images of 30 consecutive moments were used as input to the 3D-GAN model. [file peerj-cs-09-1292-s011.zip › 201009140245.jpg]

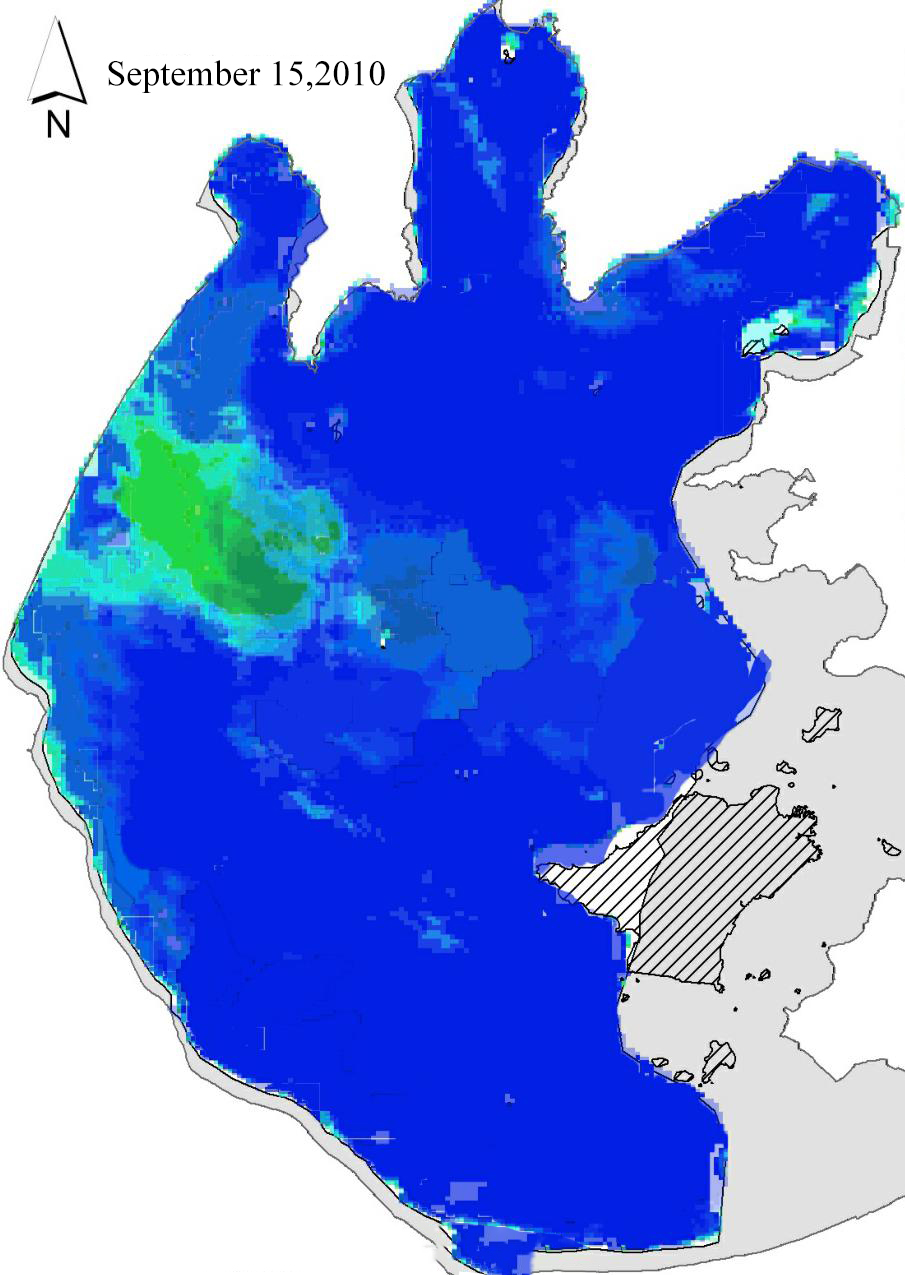

Supplement: Supplemental Information 11 — The data are remote sensing images of chlorophyll a concentration after data scale unification, remote sensing image repair, and time series filling. Remote sensing images of 30 consecutive moments were used as input to the 3D-GAN model. [file peerj-cs-09-1292-s011.zip › 201009150245.jpg]

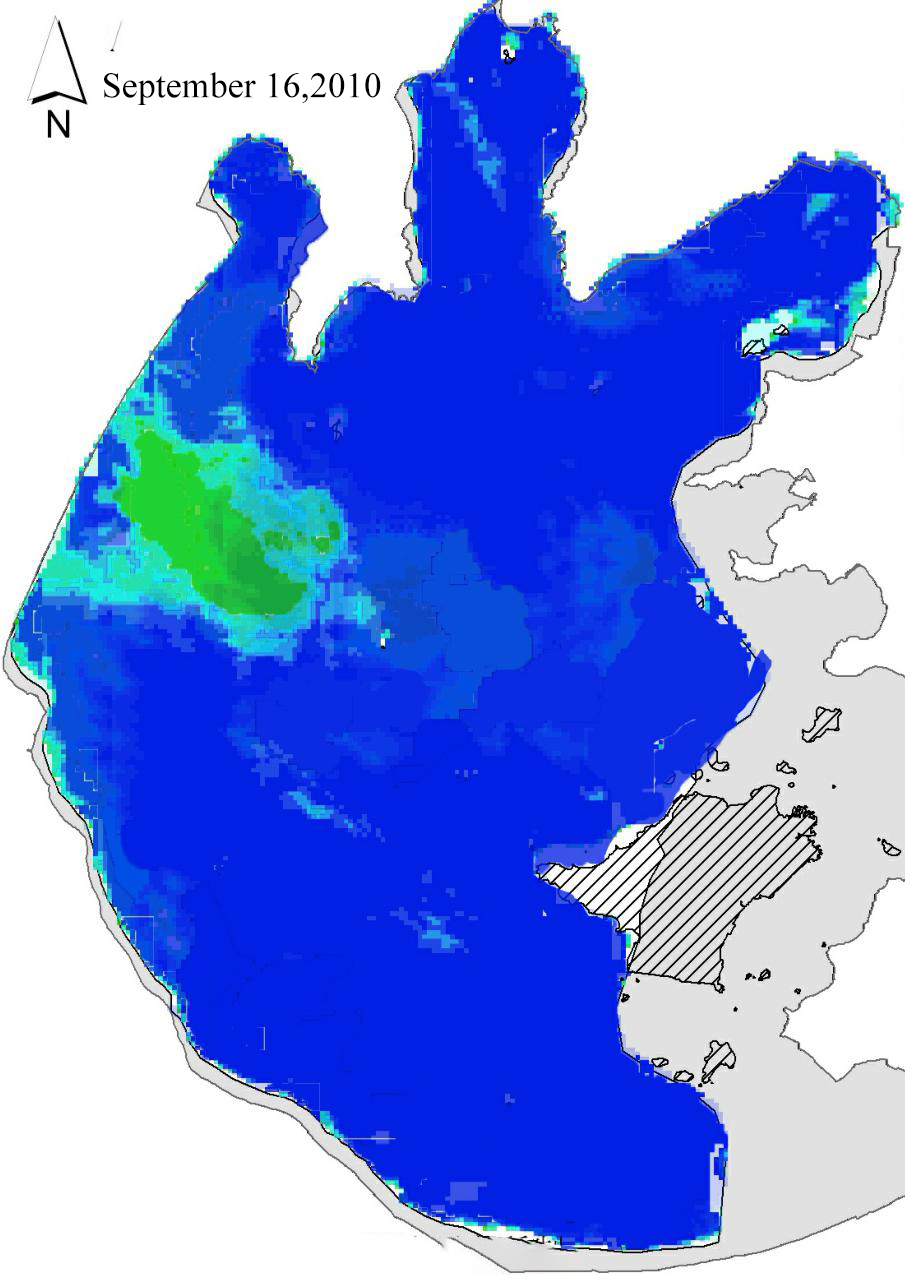

Supplement: Supplemental Information 11 — The data are remote sensing images of chlorophyll a concentration after data scale unification, remote sensing image repair, and time series filling. Remote sensing images of 30 consecutive moments were used as input to the 3D-GAN model. [file peerj-cs-09-1292-s011.zip › 201009160245.jpg]

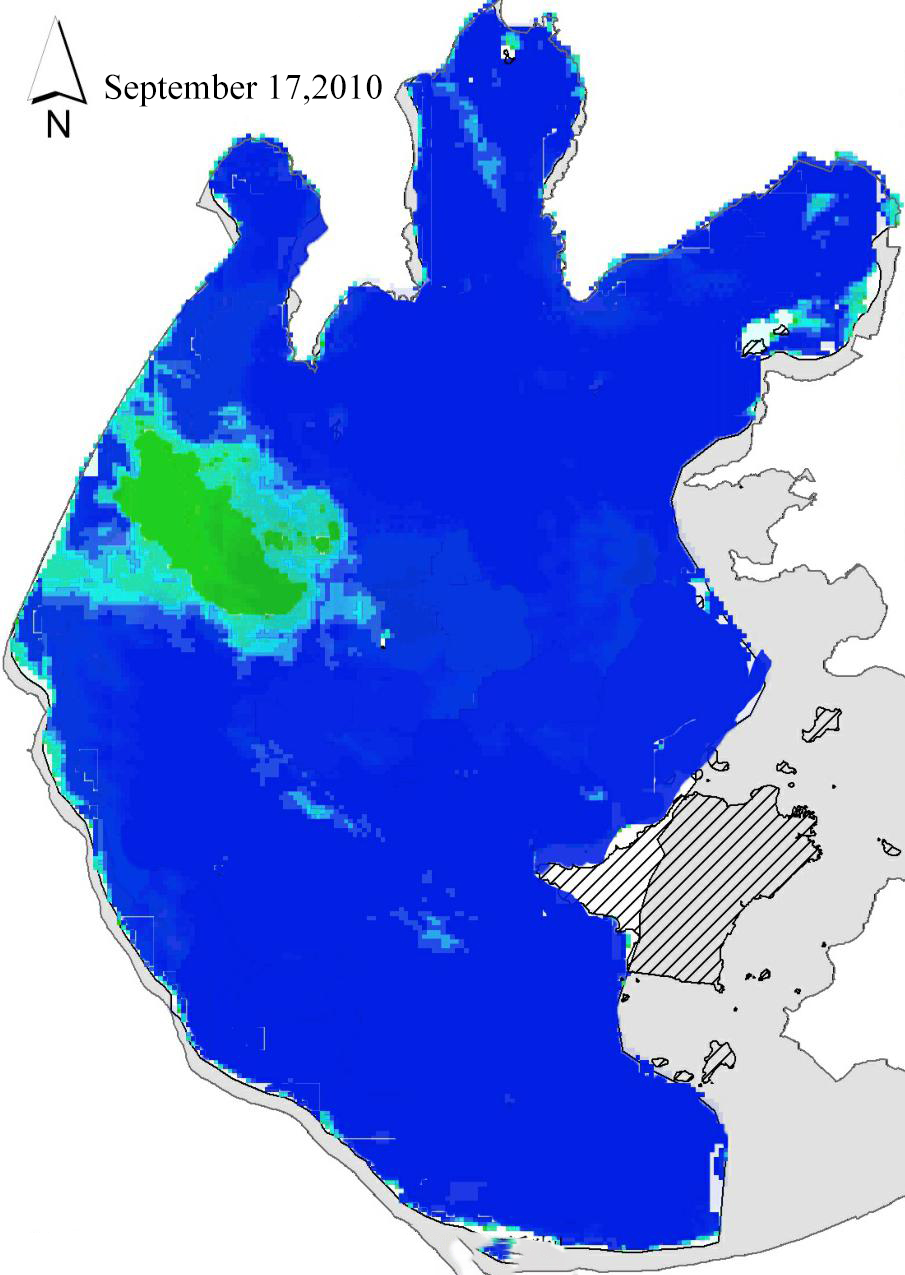

Supplement: Supplemental Information 11 — The data are remote sensing images of chlorophyll a concentration after data scale unification, remote sensing image repair, and time series filling. Remote sensing images of 30 consecutive moments were used as input to the 3D-GAN model. [file peerj-cs-09-1292-s011.zip › 201009170245.jpg]

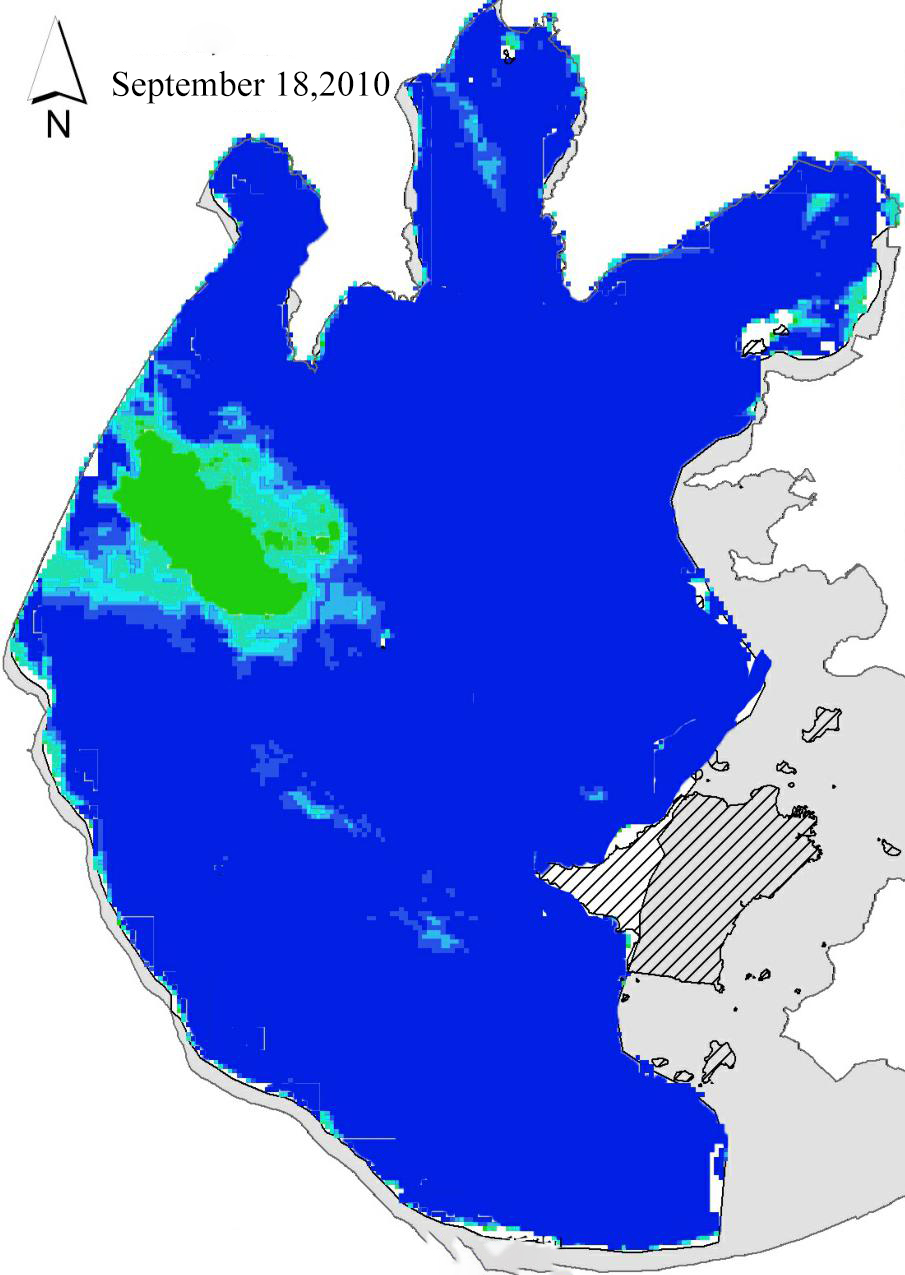

Supplement: Supplemental Information 11 — The data are remote sensing images of chlorophyll a concentration after data scale unification, remote sensing image repair, and time series filling. Remote sensing images of 30 consecutive moments were used as input to the 3D-GAN model. [file peerj-cs-09-1292-s011.zip › 201009180245.jpg]

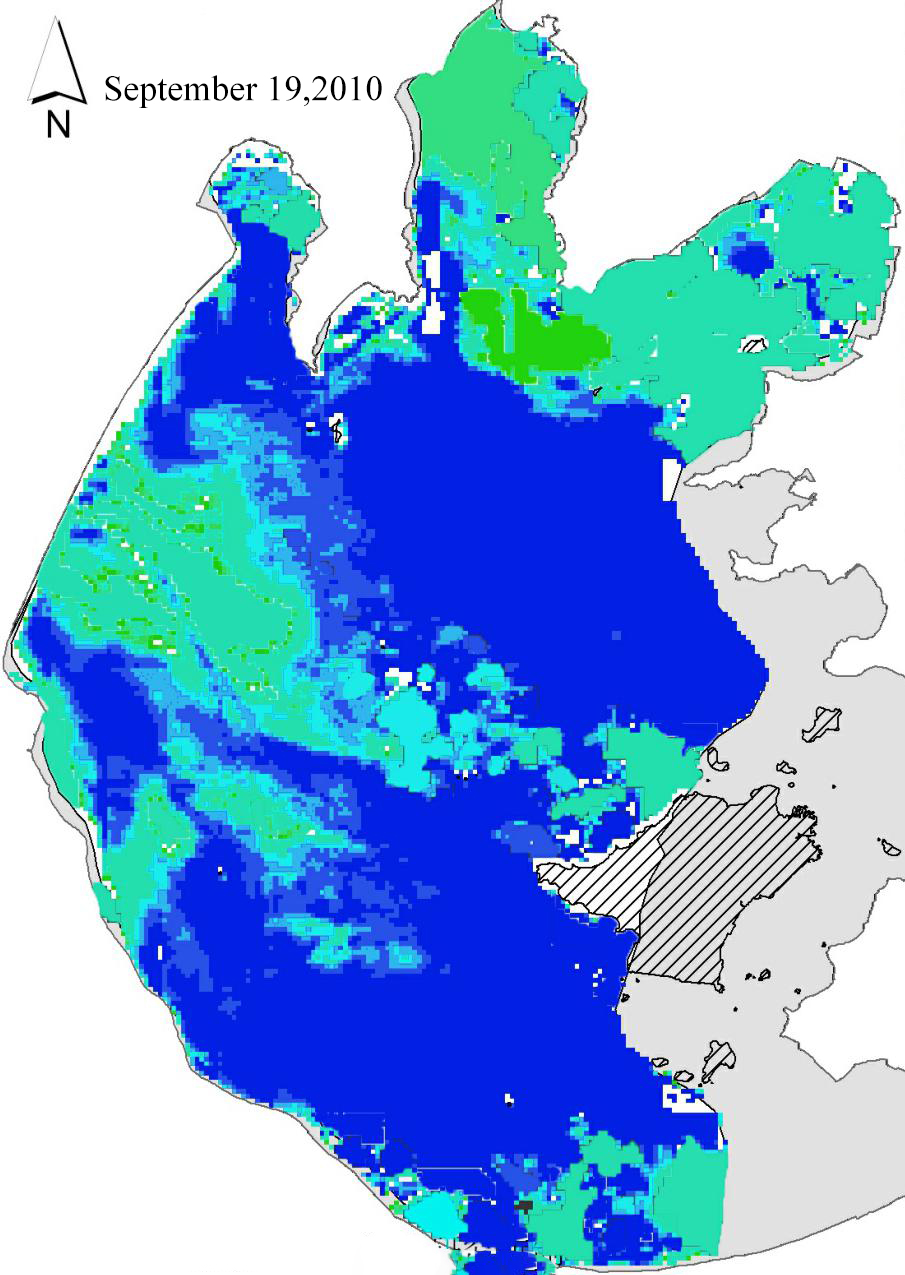

Supplement: Supplemental Information 11 — The data are remote sensing images of chlorophyll a concentration after data scale unification, remote sensing image repair, and time series filling. Remote sensing images of 30 consecutive moments were used as input to the 3D-GAN model. [file peerj-cs-09-1292-s011.zip › 201009190245.jpg]

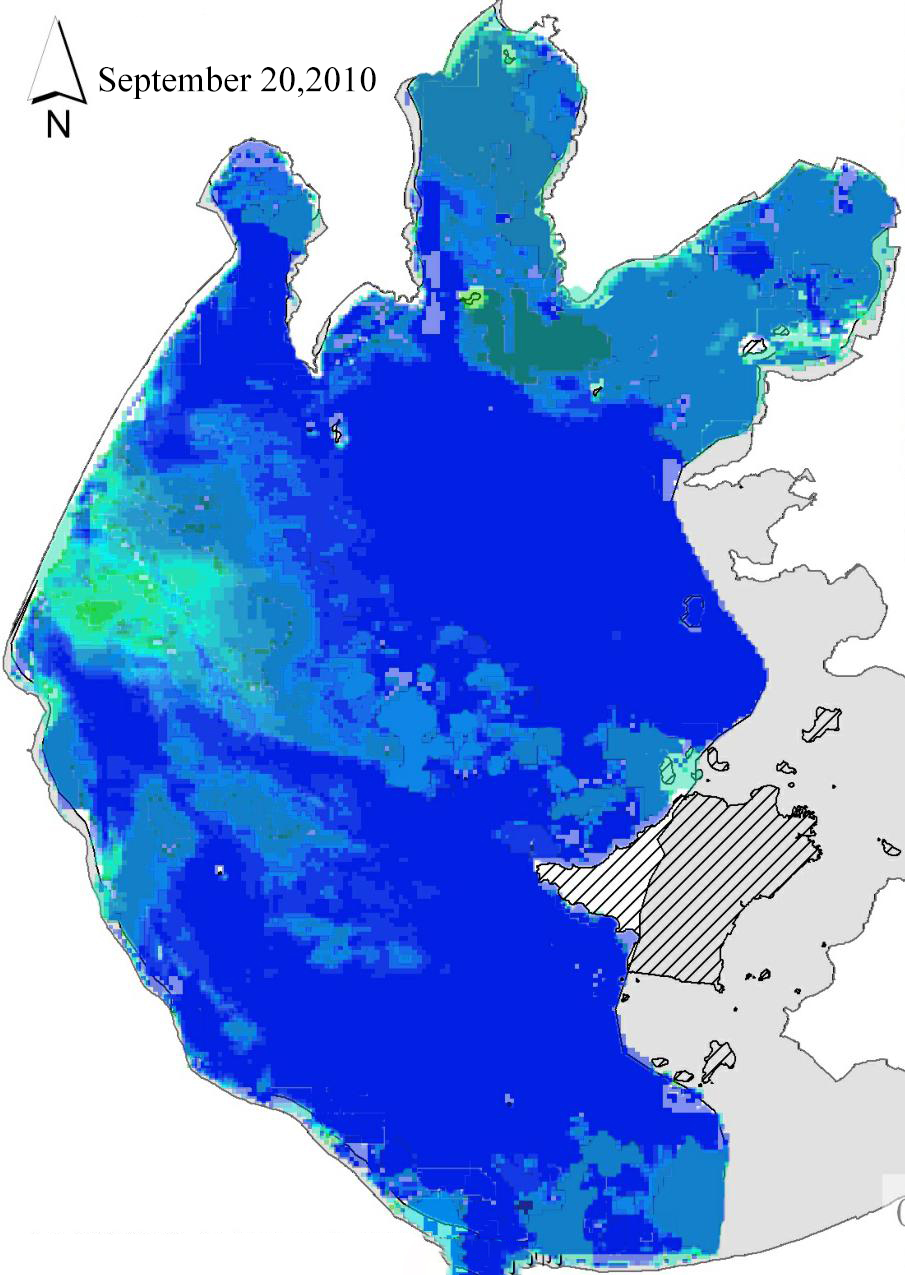

Supplement: Supplemental Information 11 — The data are remote sensing images of chlorophyll a concentration after data scale unification, remote sensing image repair, and time series filling. Remote sensing images of 30 consecutive moments were used as input to the 3D-GAN model. [file peerj-cs-09-1292-s011.zip › 201009200245.jpg]

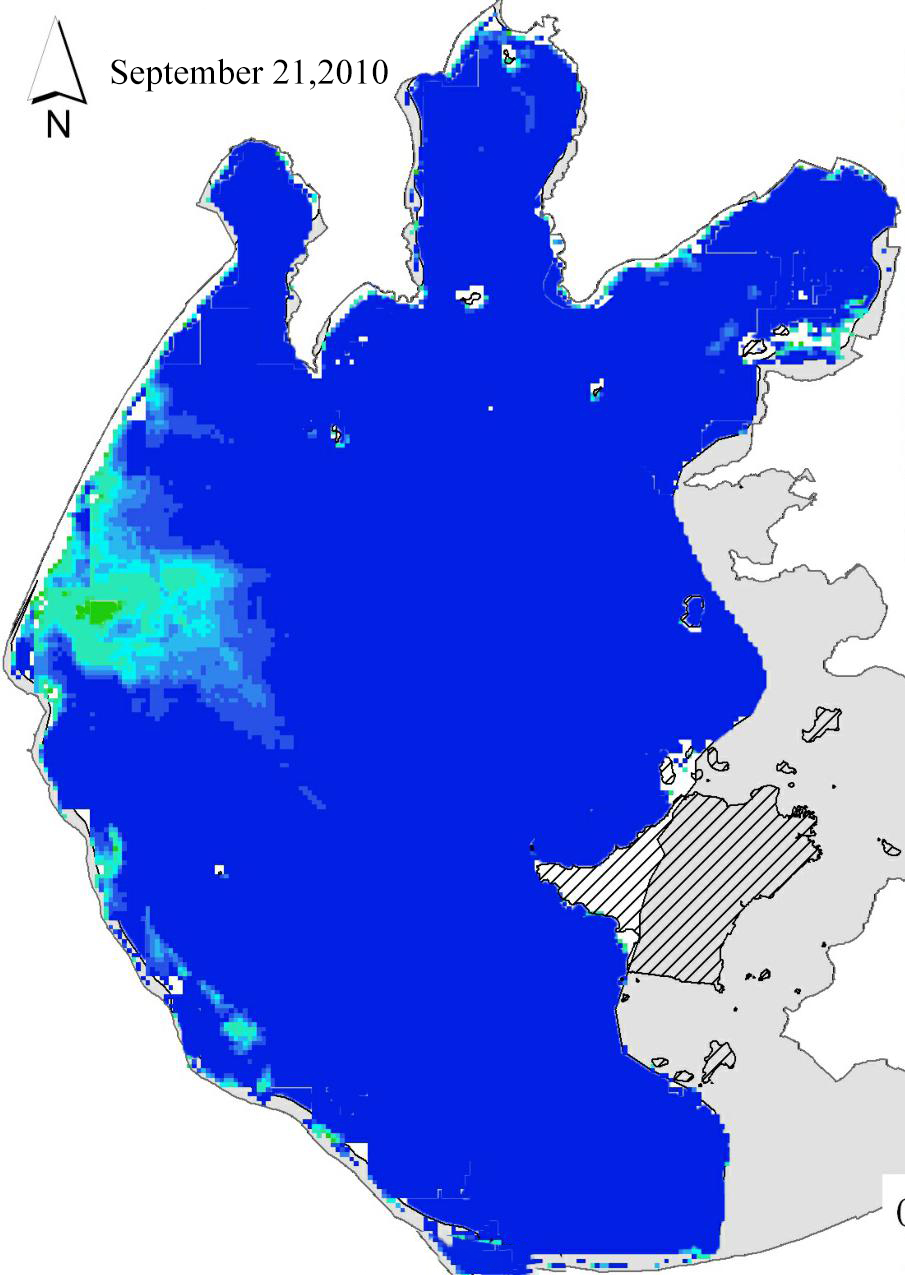

Supplement: Supplemental Information 11 — The data are remote sensing images of chlorophyll a concentration after data scale unification, remote sensing image repair, and time series filling. Remote sensing images of 30 consecutive moments were used as input to the 3D-GAN model. [file peerj-cs-09-1292-s011.zip › 201009210245.jpg]

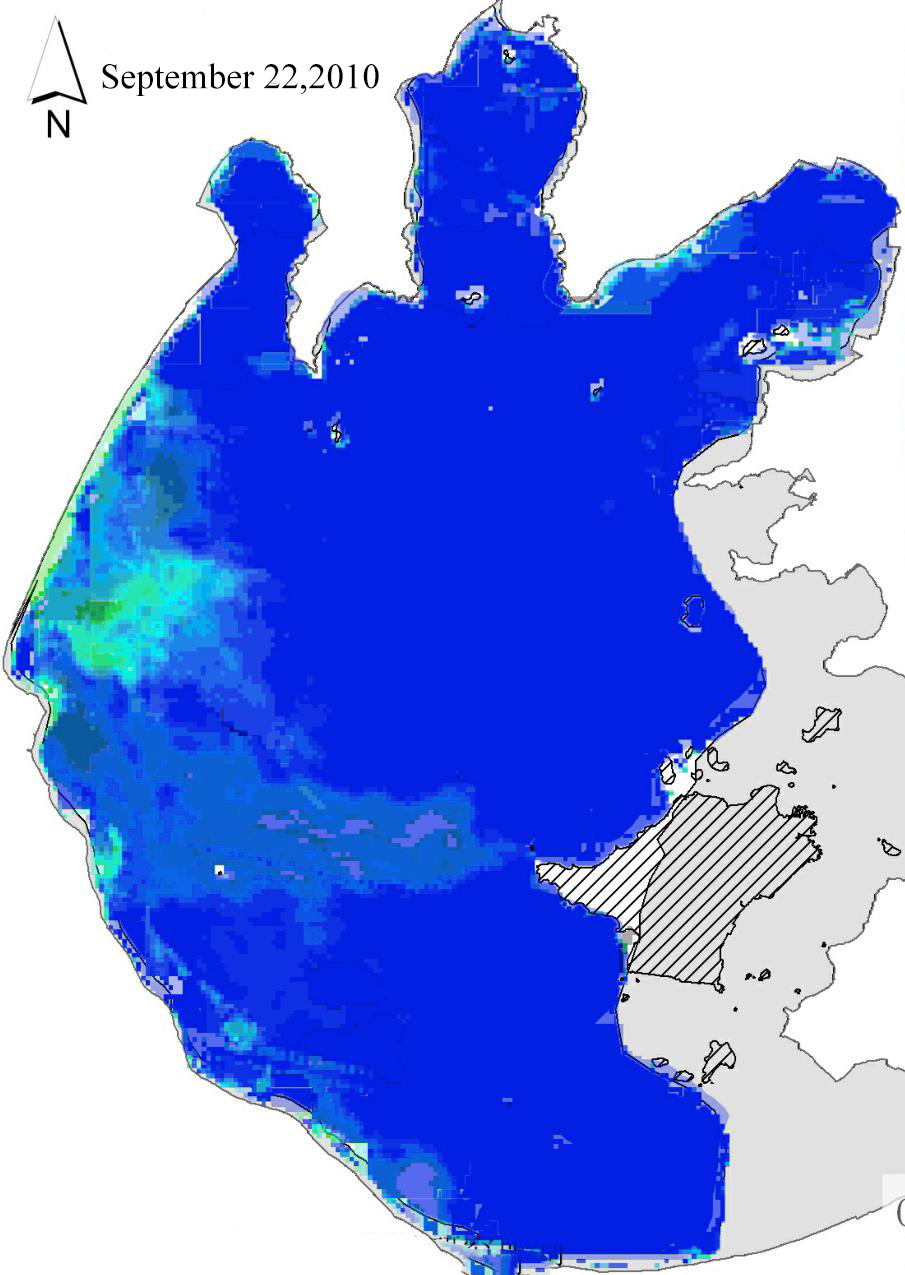

Supplement: Supplemental Information 11 — The data are remote sensing images of chlorophyll a concentration after data scale unification, remote sensing image repair, and time series filling. Remote sensing images of 30 consecutive moments were used as input to the 3D-GAN model. [file peerj-cs-09-1292-s011.zip › 201009220245.jpg]

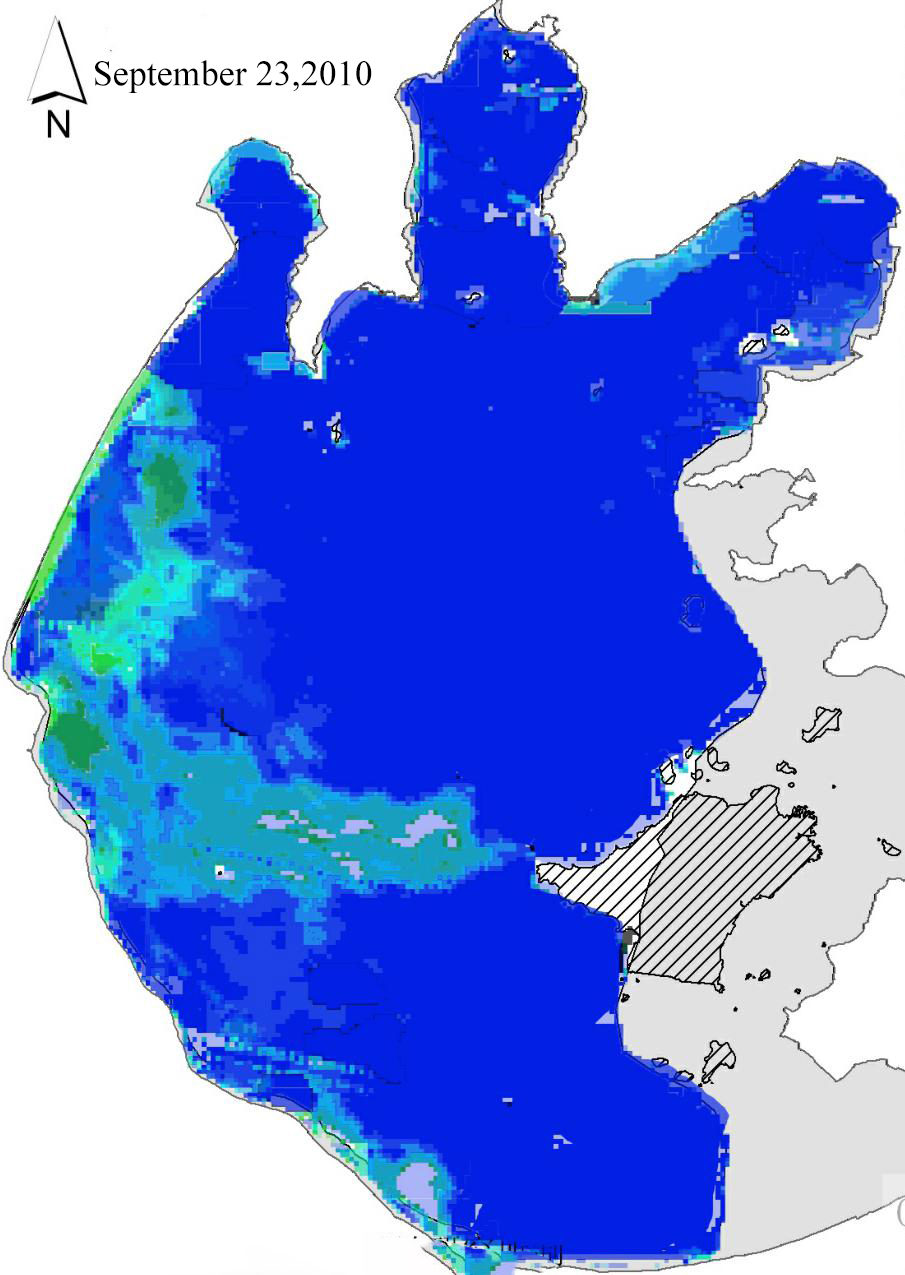

Supplement: Supplemental Information 11 — The data are remote sensing images of chlorophyll a concentration after data scale unification, remote sensing image repair, and time series filling. Remote sensing images of 30 consecutive moments were used as input to the 3D-GAN model. [file peerj-cs-09-1292-s011.zip › 201009230245.jpg]

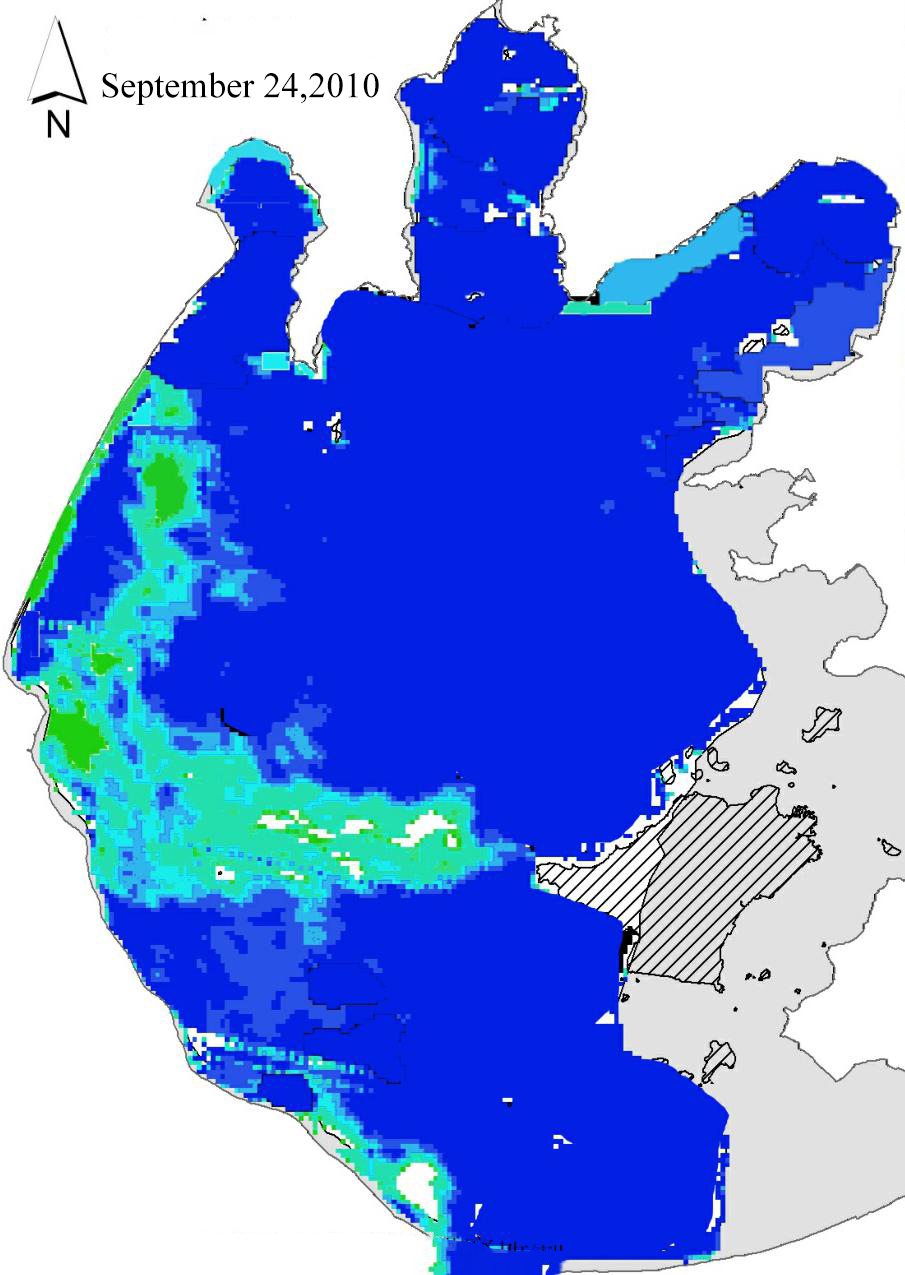

Supplement: Supplemental Information 11 — The data are remote sensing images of chlorophyll a concentration after data scale unification, remote sensing image repair, and time series filling. Remote sensing images of 30 consecutive moments were used as input to the 3D-GAN model. [file peerj-cs-09-1292-s011.zip › 201009240245.jpg]

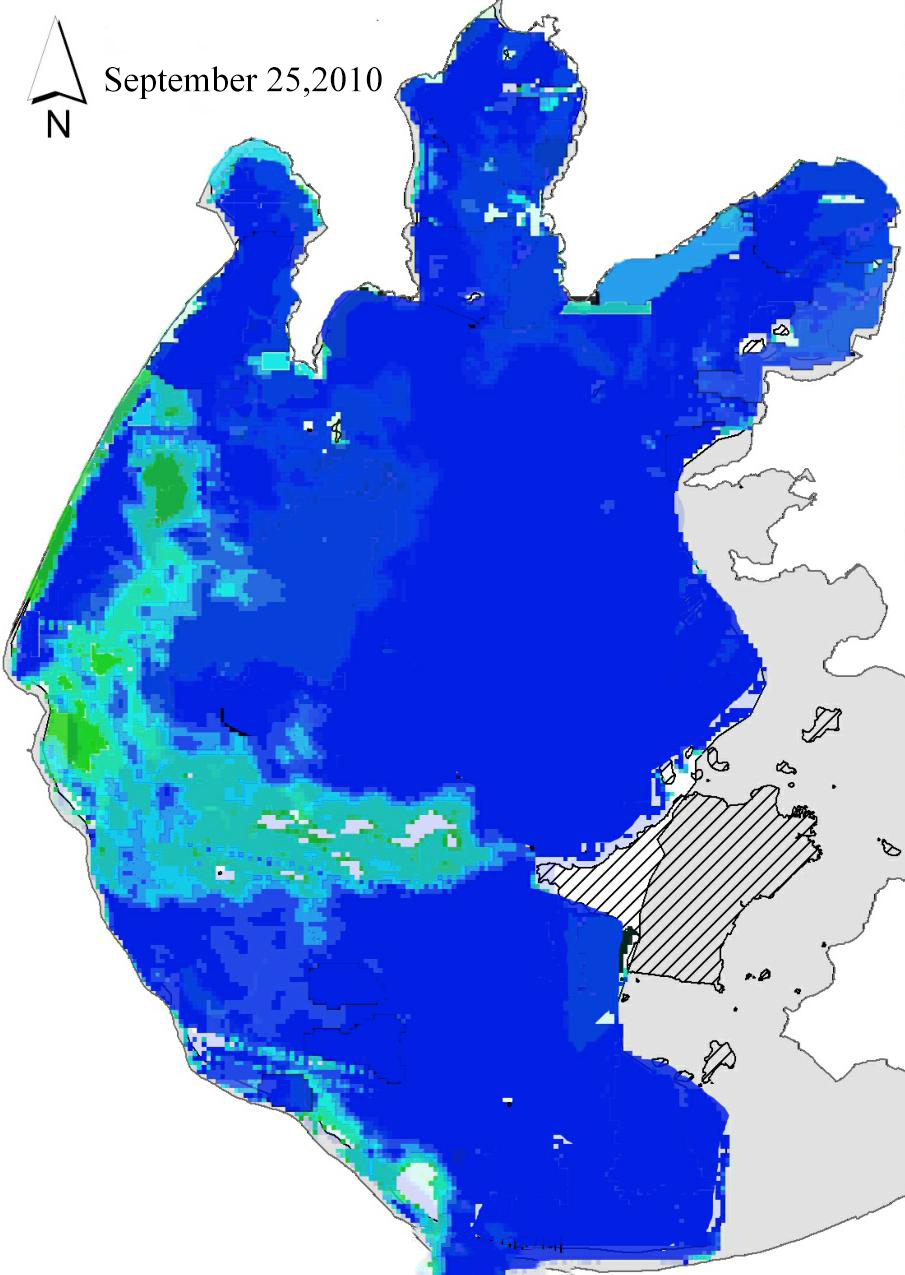

Supplement: Supplemental Information 11 — The data are remote sensing images of chlorophyll a concentration after data scale unification, remote sensing image repair, and time series filling. Remote sensing images of 30 consecutive moments were used as input to the 3D-GAN model. [file peerj-cs-09-1292-s011.zip › 201009250245.jpg]

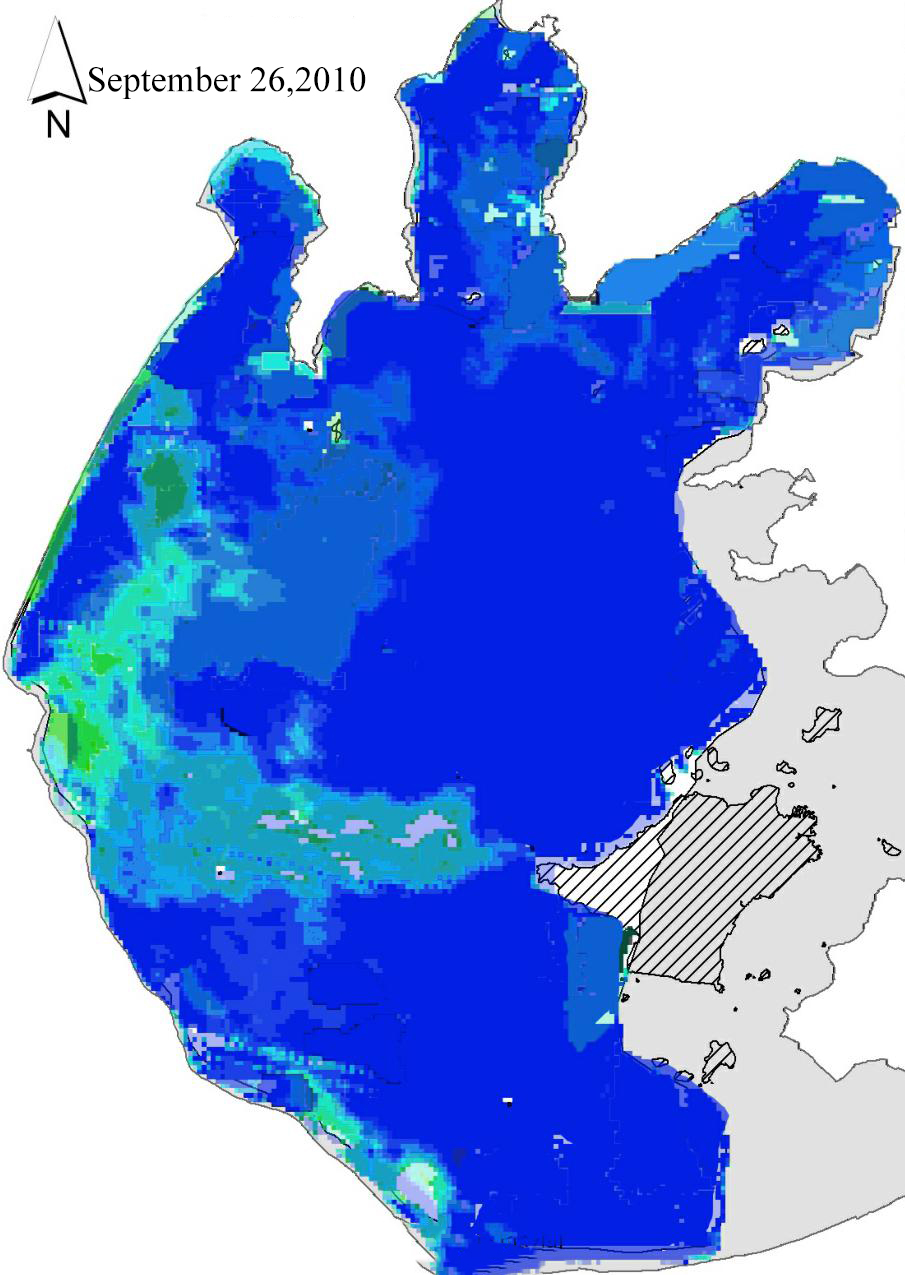

Supplement: Supplemental Information 11 — The data are remote sensing images of chlorophyll a concentration after data scale unification, remote sensing image repair, and time series filling. Remote sensing images of 30 consecutive moments were used as input to the 3D-GAN model. [file peerj-cs-09-1292-s011.zip › 201009260245.jpg]

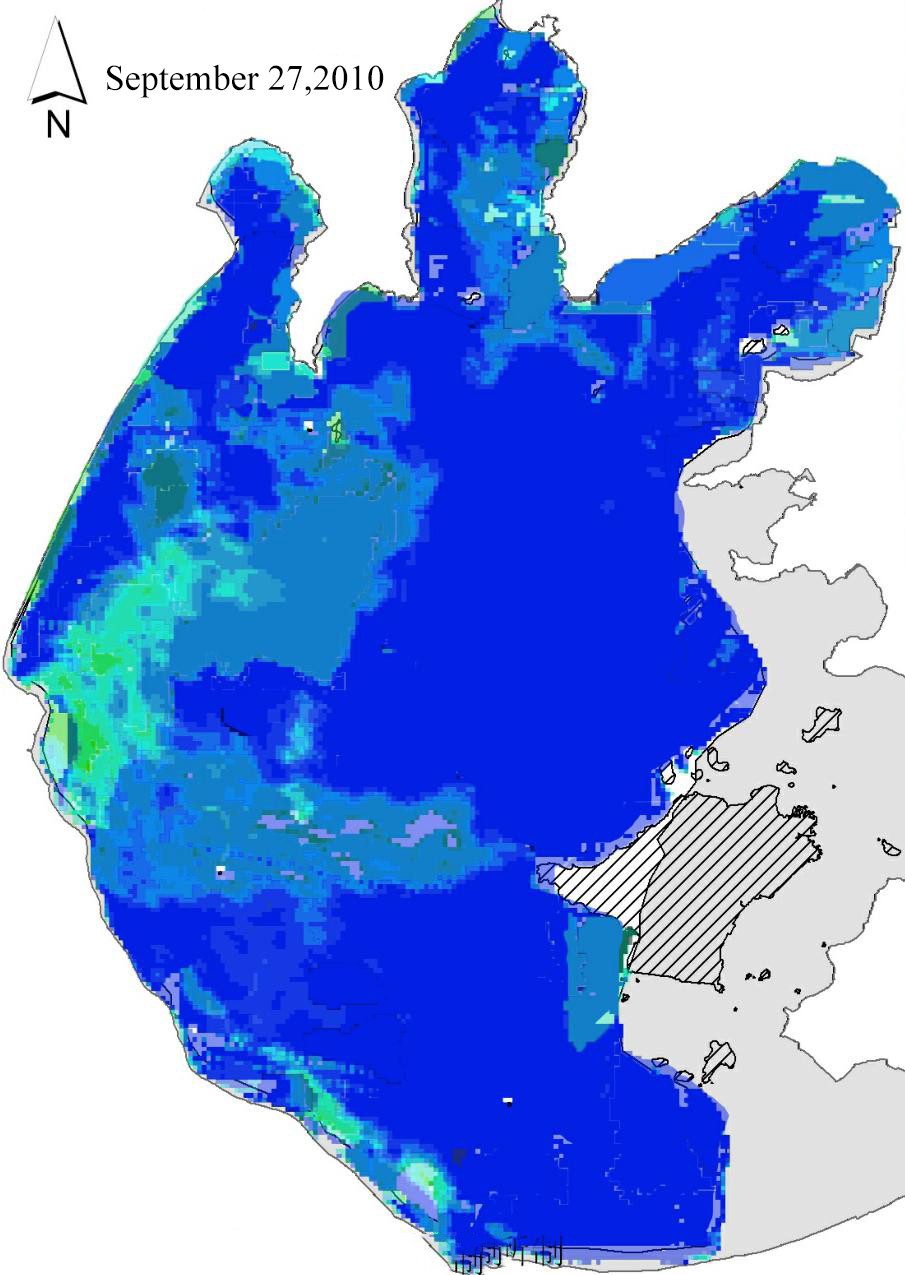

Supplement: Supplemental Information 11 — The data are remote sensing images of chlorophyll a concentration after data scale unification, remote sensing image repair, and time series filling. Remote sensing images of 30 consecutive moments were used as input to the 3D-GAN model. [file peerj-cs-09-1292-s011.zip › 201009270245.jpg]

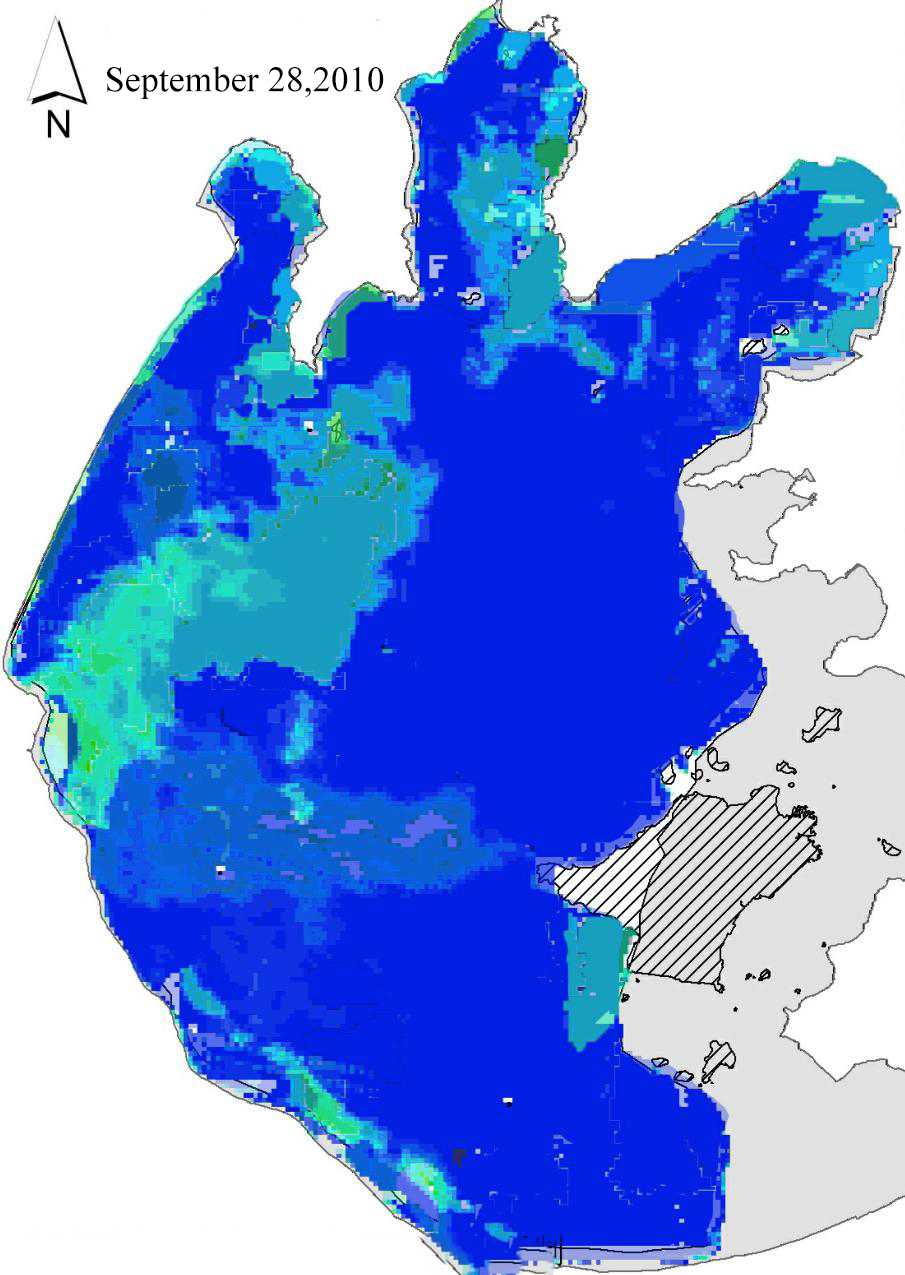

Supplement: Supplemental Information 11 — The data are remote sensing images of chlorophyll a concentration after data scale unification, remote sensing image repair, and time series filling. Remote sensing images of 30 consecutive moments were used as input to the 3D-GAN model. [file peerj-cs-09-1292-s011.zip › 201009280245.jpg]

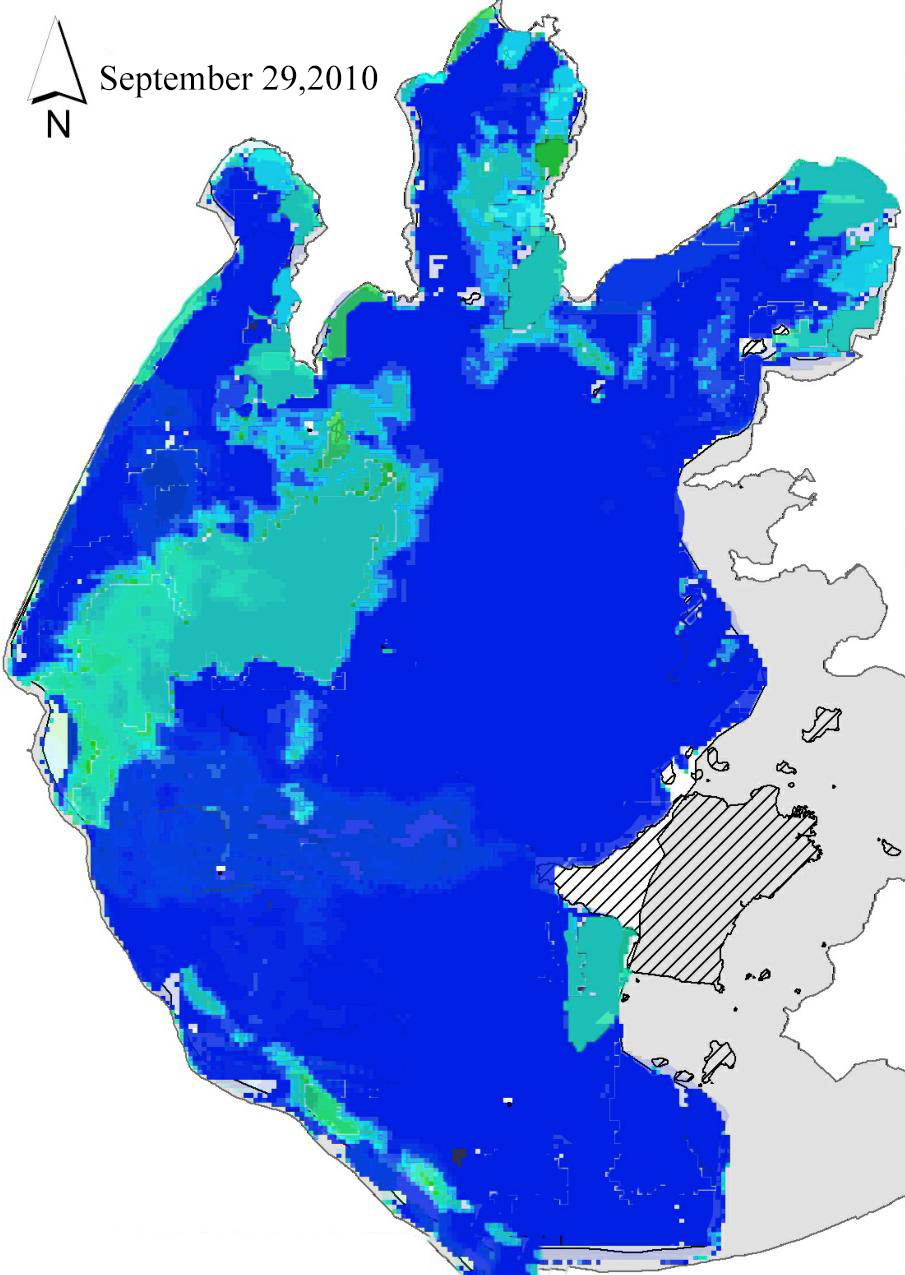

Supplement: Supplemental Information 11 — The data are remote sensing images of chlorophyll a concentration after data scale unification, remote sensing image repair, and time series filling. Remote sensing images of 30 consecutive moments were used as input to the 3D-GAN model. [file peerj-cs-09-1292-s011.zip › 201009290245.jpg]

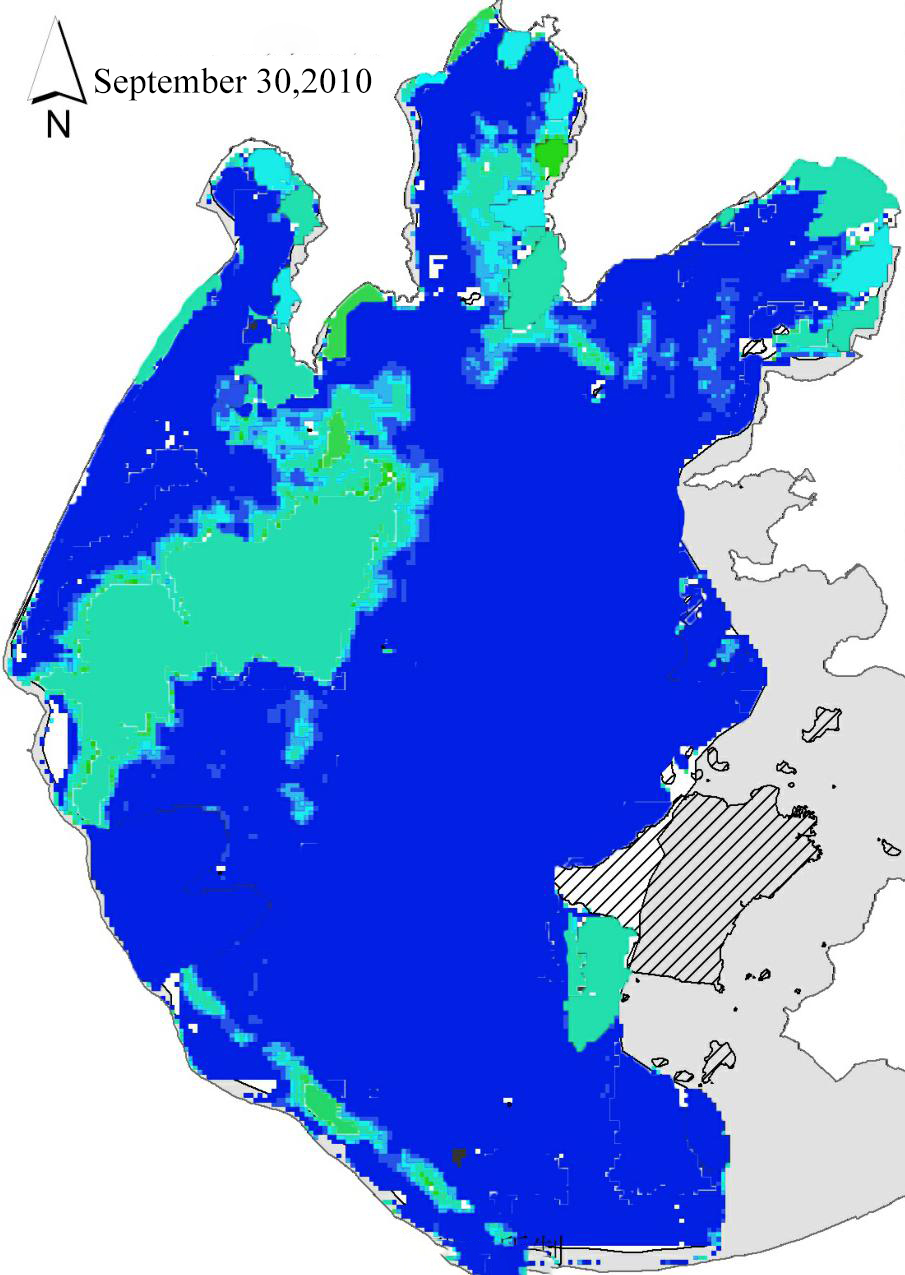

Supplement: Supplemental Information 11 — The data are remote sensing images of chlorophyll a concentration after data scale unification, remote sensing image repair, and time series filling. Remote sensing images of 30 consecutive moments were used as input to the 3D-GAN model. [file peerj-cs-09-1292-s011.zip › 201009300245.jpg]

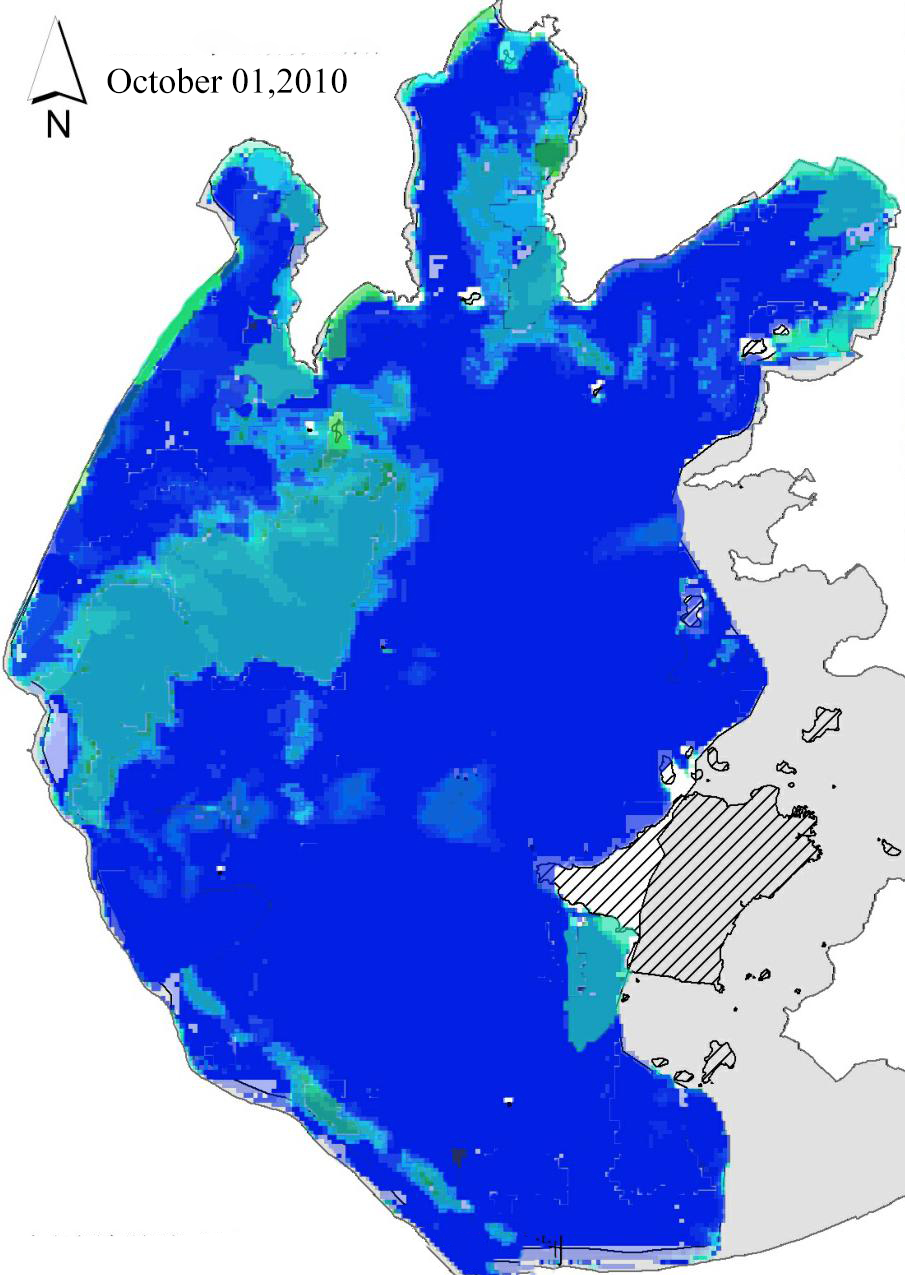

Supplement: Supplemental Information 11 — The data are remote sensing images of chlorophyll a concentration after data scale unification, remote sensing image repair, and time series filling. Remote sensing images of 30 consecutive moments were used as input to the 3D-GAN model. [file peerj-cs-09-1292-s011.zip › 201010010245.jpg]

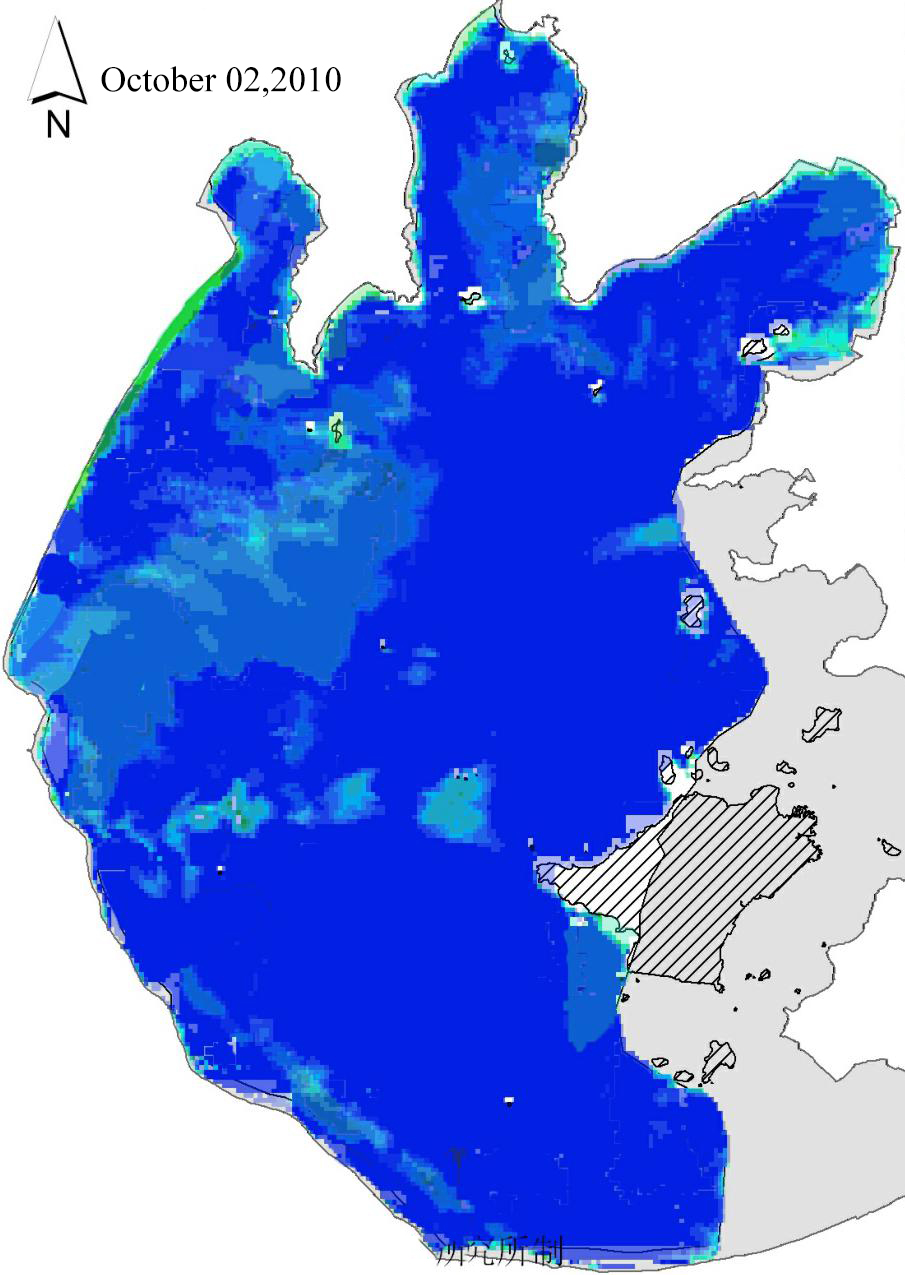

Supplement: Supplemental Information 11 — The data are remote sensing images of chlorophyll a concentration after data scale unification, remote sensing image repair, and time series filling. Remote sensing images of 30 consecutive moments were used as input to the 3D-GAN model. [file peerj-cs-09-1292-s011.zip › 201010020245.jpg]

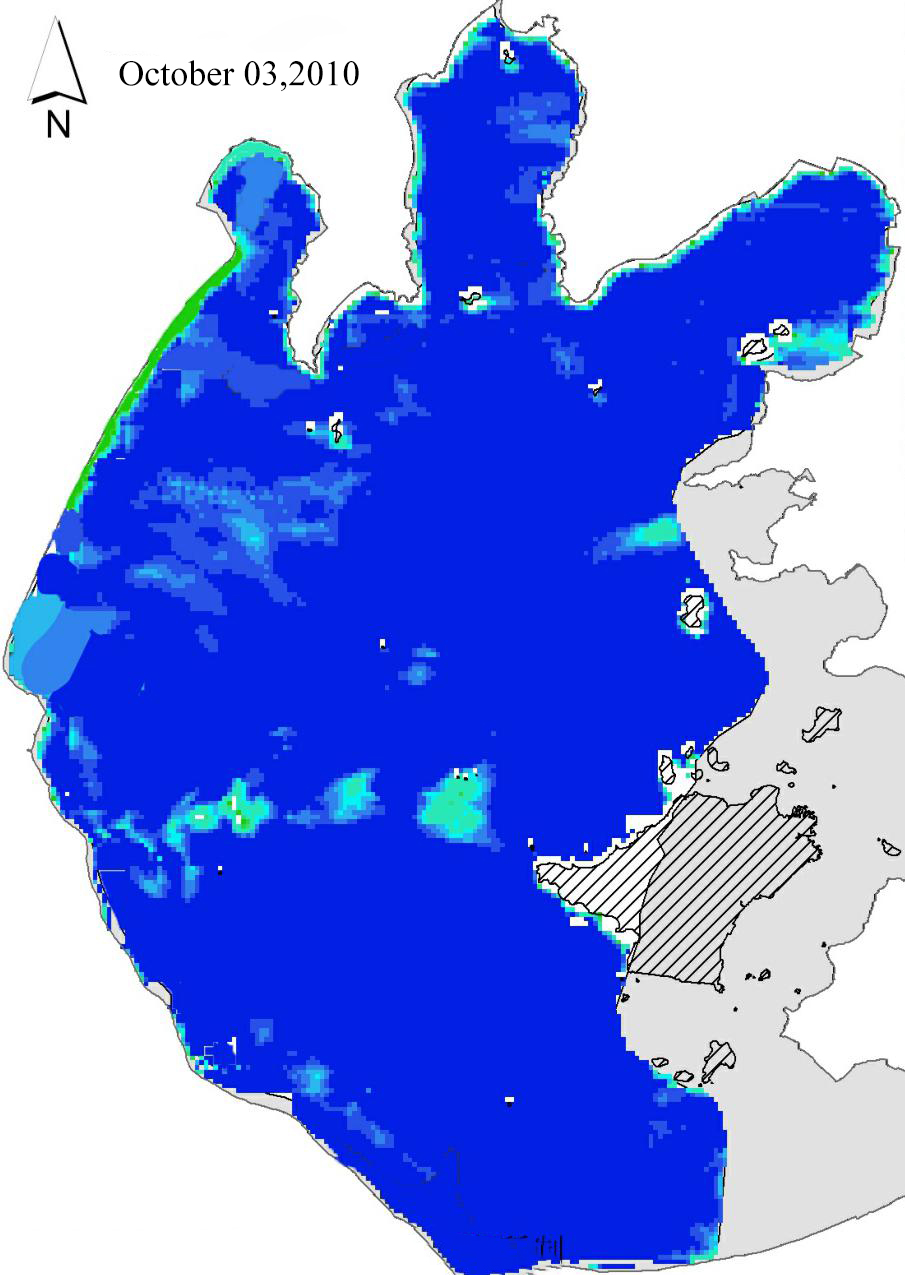

Supplement: Supplemental Information 11 — The data are remote sensing images of chlorophyll a concentration after data scale unification, remote sensing image repair, and time series filling. Remote sensing images of 30 consecutive moments were used as input to the 3D-GAN model. [file peerj-cs-09-1292-s011.zip › 201010030245.jpg]

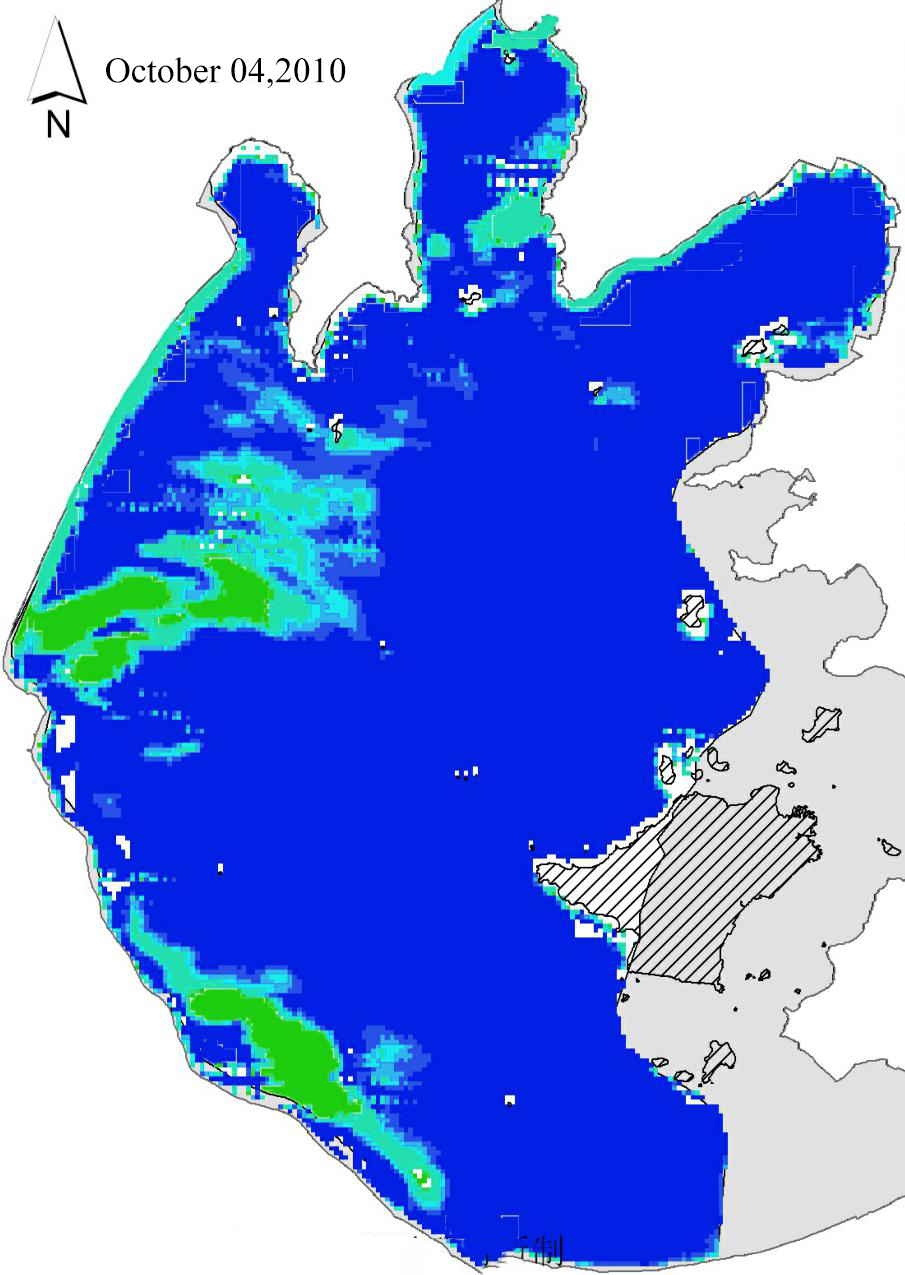

Supplement: Supplemental Information 11 — The data are remote sensing images of chlorophyll a concentration after data scale unification, remote sensing image repair, and time series filling. Remote sensing images of 30 consecutive moments were used as input to the 3D-GAN model. [file peerj-cs-09-1292-s011.zip › 201010040245.jpg]

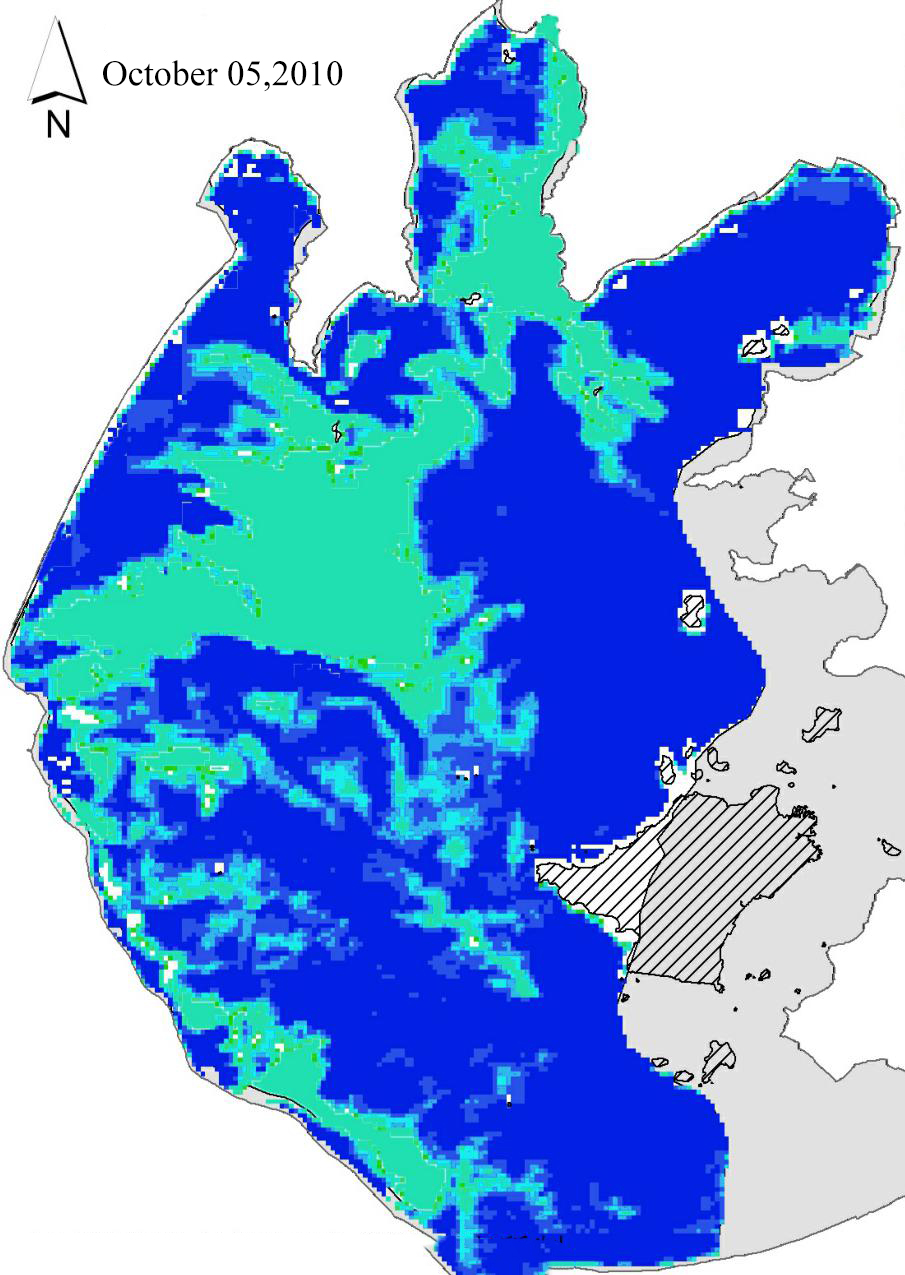

Supplement: Supplemental Information 11 — The data are remote sensing images of chlorophyll a concentration after data scale unification, remote sensing image repair, and time series filling. Remote sensing images of 30 consecutive moments were used as input to the 3D-GAN model. [file peerj-cs-09-1292-s011.zip › 201010050245.jpg]

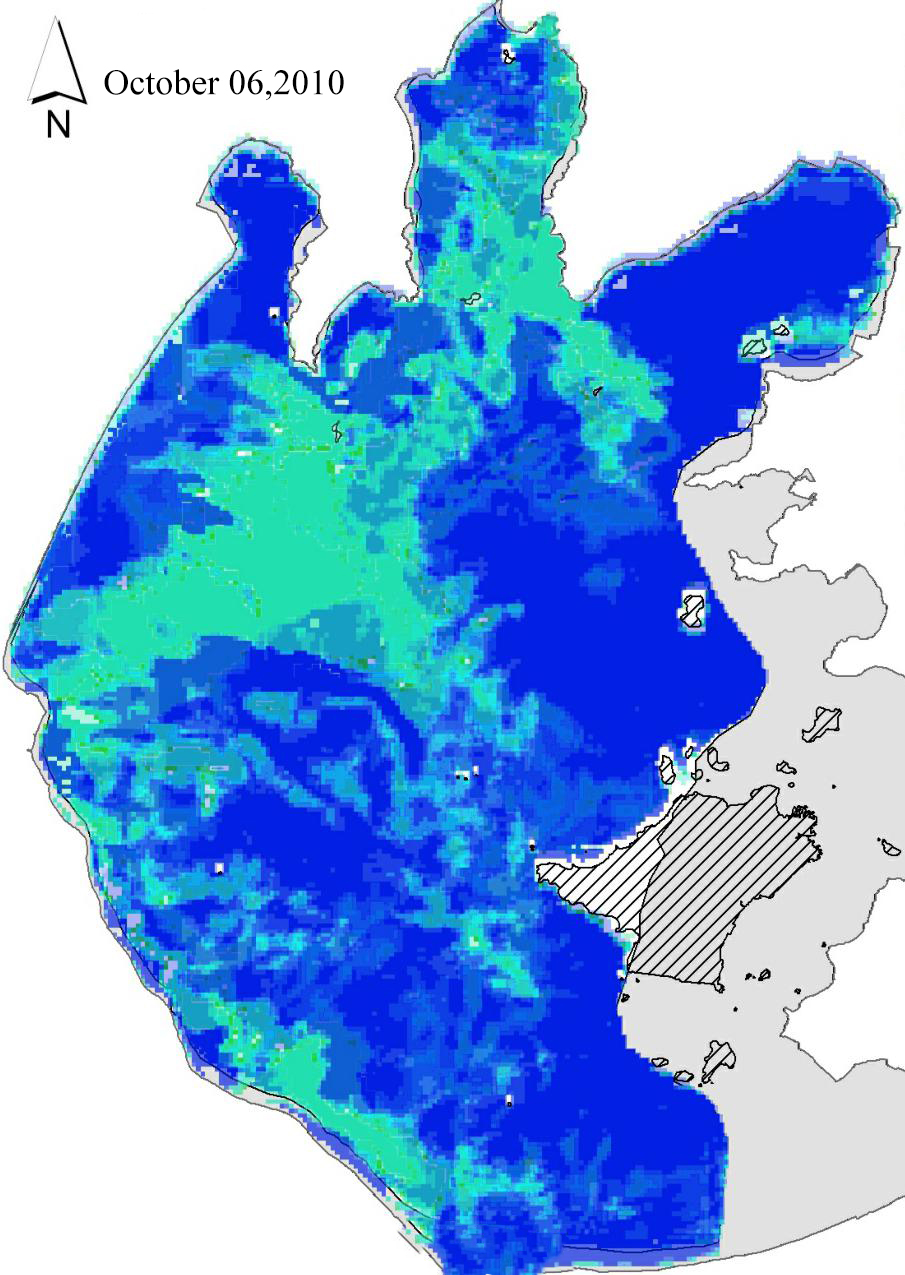

Supplement: Supplemental Information 11 — The data are remote sensing images of chlorophyll a concentration after data scale unification, remote sensing image repair, and time series filling. Remote sensing images of 30 consecutive moments were used as input to the 3D-GAN model. [file peerj-cs-09-1292-s011.zip › 201010060245.jpg]

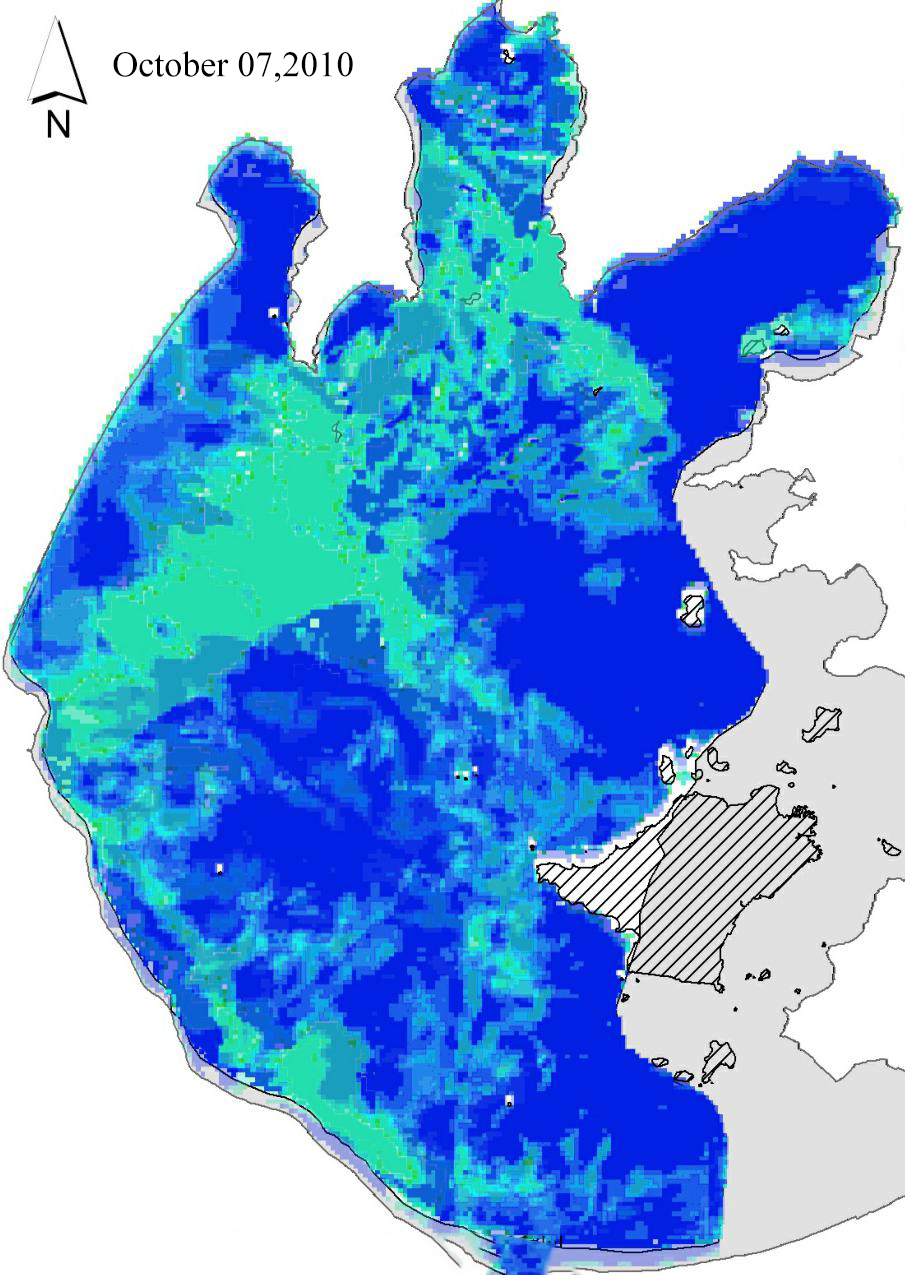

Supplement: Supplemental Information 11 — The data are remote sensing images of chlorophyll a concentration after data scale unification, remote sensing image repair, and time series filling. Remote sensing images of 30 consecutive moments were used as input to the 3D-GAN model. [file peerj-cs-09-1292-s011.zip › 201010070245.jpg]

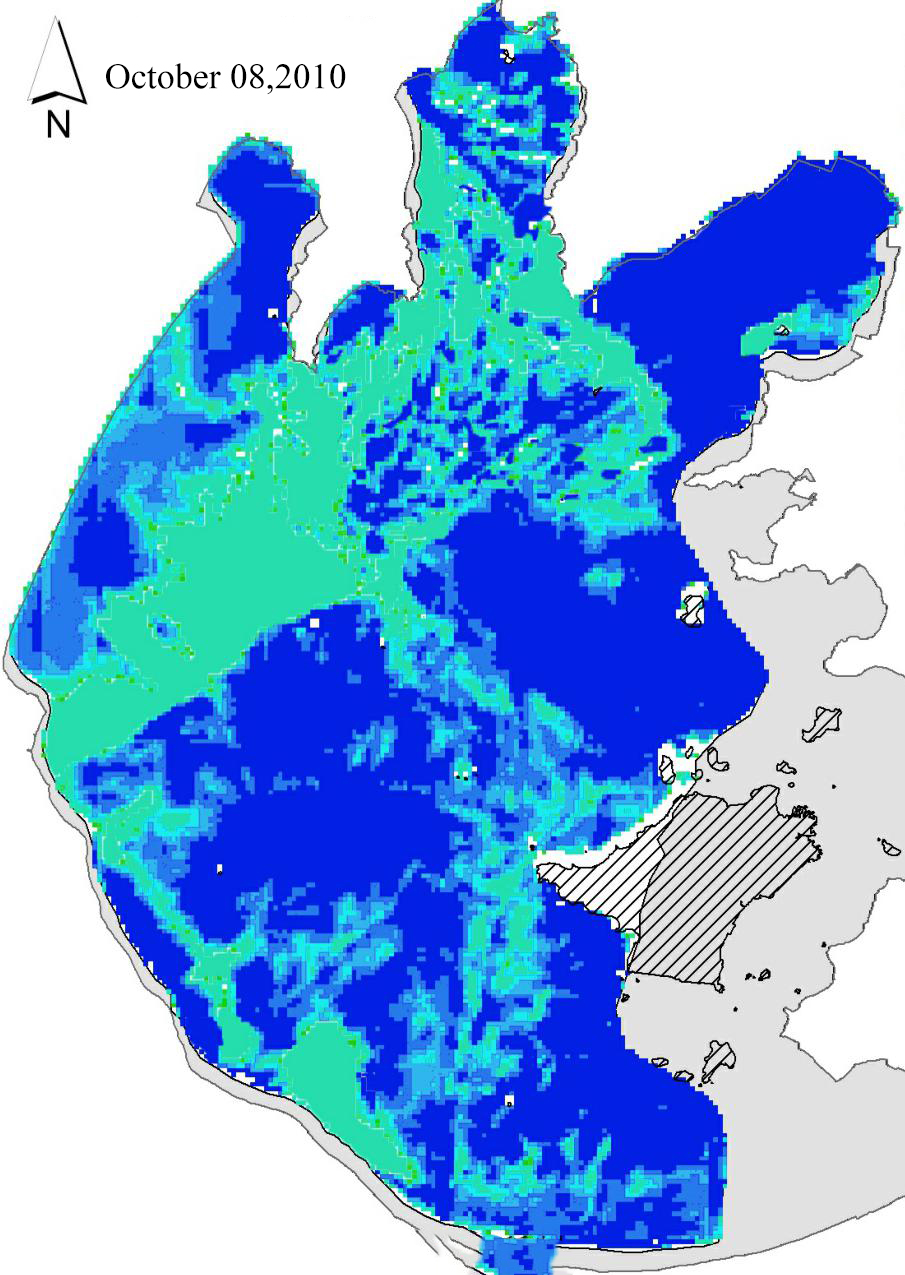

Supplement: Supplemental Information 11 — The data are remote sensing images of chlorophyll a concentration after data scale unification, remote sensing image repair, and time series filling. Remote sensing images of 30 consecutive moments were used as input to the 3D-GAN model. [file peerj-cs-09-1292-s011.zip › 201010080245.jpg]

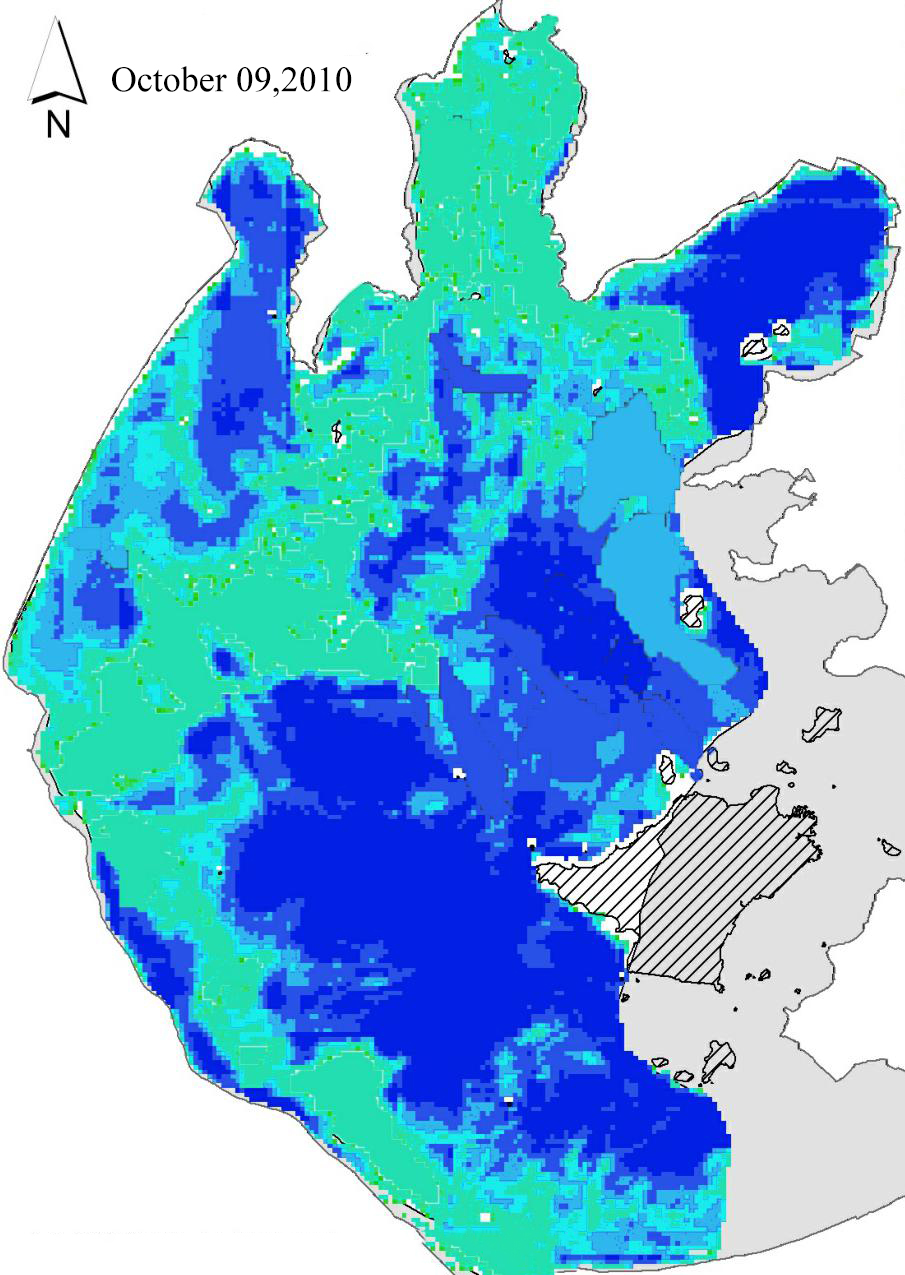

Supplement: Supplemental Information 11 — The data are remote sensing images of chlorophyll a concentration after data scale unification, remote sensing image repair, and time series filling. Remote sensing images of 30 consecutive moments were used as input to the 3D-GAN model. [file peerj-cs-09-1292-s011.zip › 201010090245.jpg]

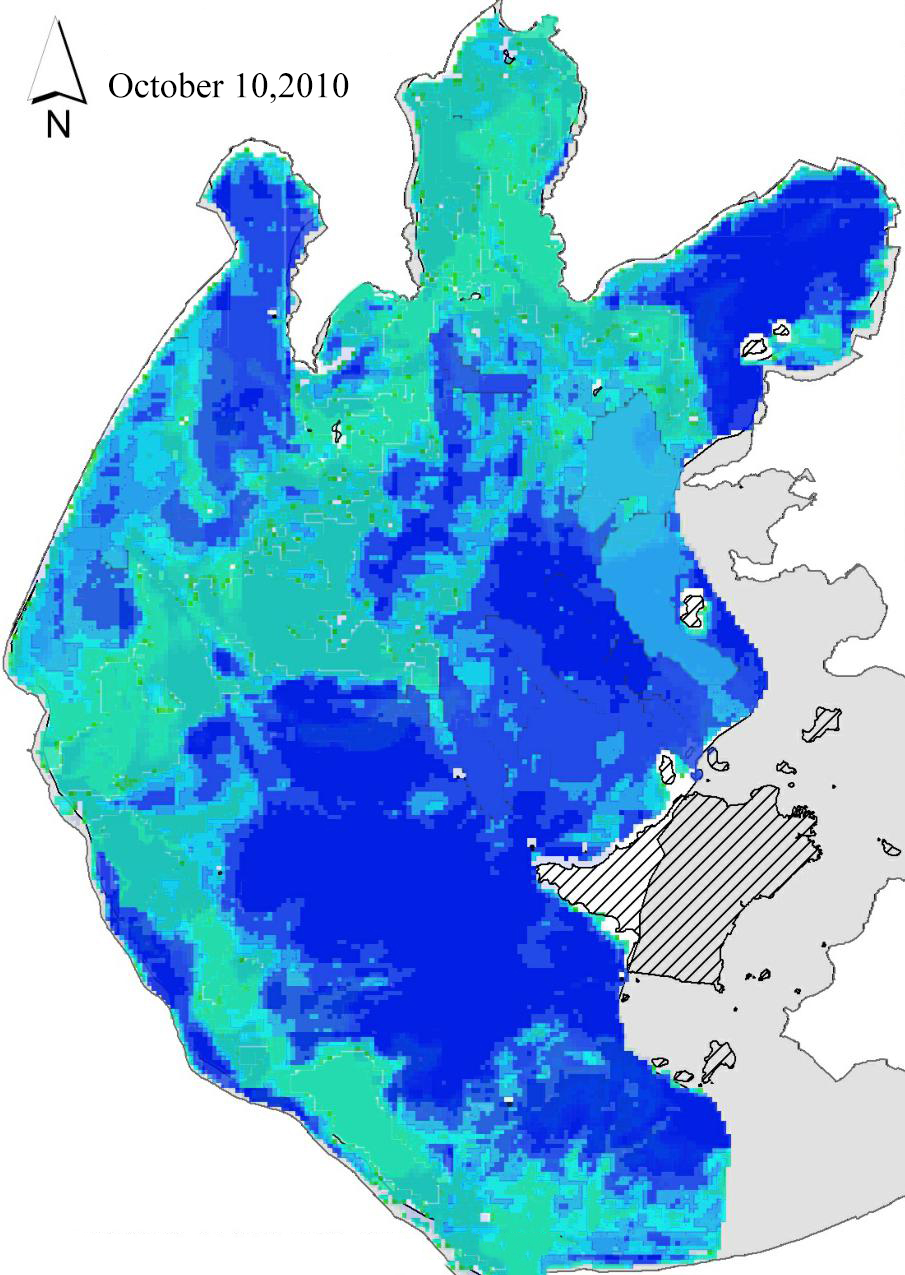

Supplement: Supplemental Information 11 — The data are remote sensing images of chlorophyll a concentration after data scale unification, remote sensing image repair, and time series filling. Remote sensing images of 30 consecutive moments were used as input to the 3D-GAN model. [file peerj-cs-09-1292-s011.zip › 201010100245.jpg]

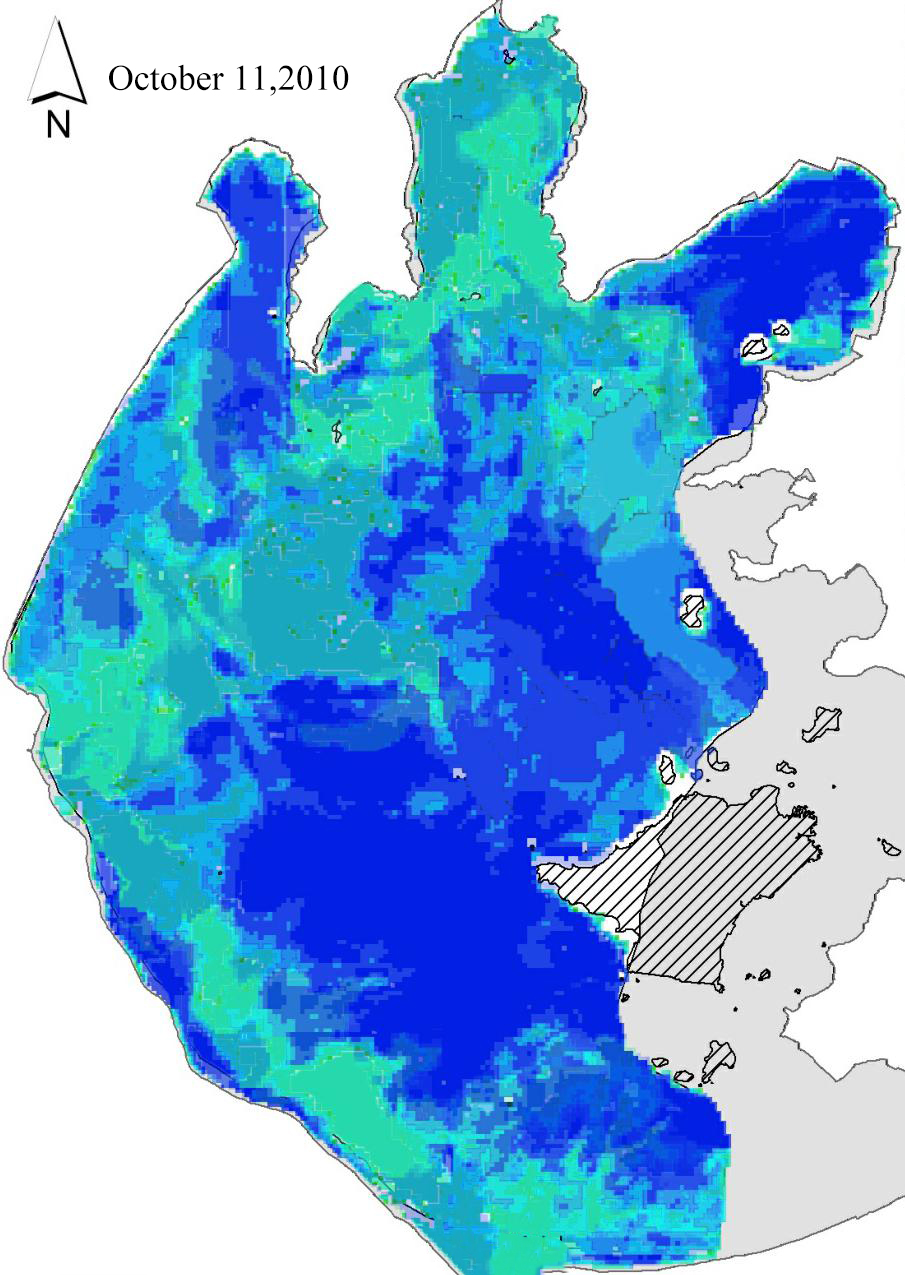

Supplement: Supplemental Information 11 — The data are remote sensing images of chlorophyll a concentration after data scale unification, remote sensing image repair, and time series filling. Remote sensing images of 30 consecutive moments were used as input to the 3D-GAN model. [file peerj-cs-09-1292-s011.zip › 201010110245.jpg]

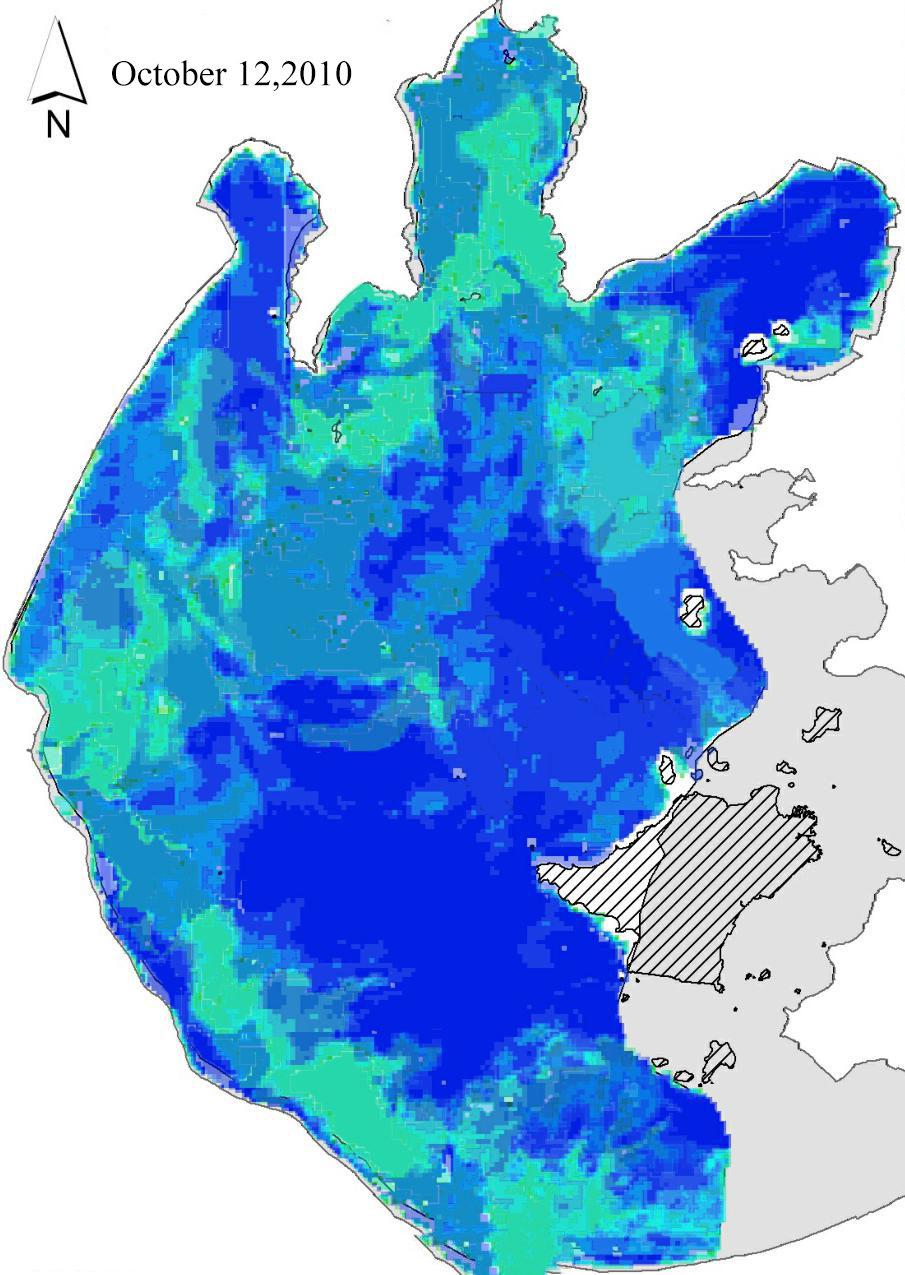

Supplement: Supplemental Information 11 — The data are remote sensing images of chlorophyll a concentration after data scale unification, remote sensing image repair, and time series filling. Remote sensing images of 30 consecutive moments were used as input to the 3D-GAN model. [file peerj-cs-09-1292-s011.zip › 201010120245.jpg]

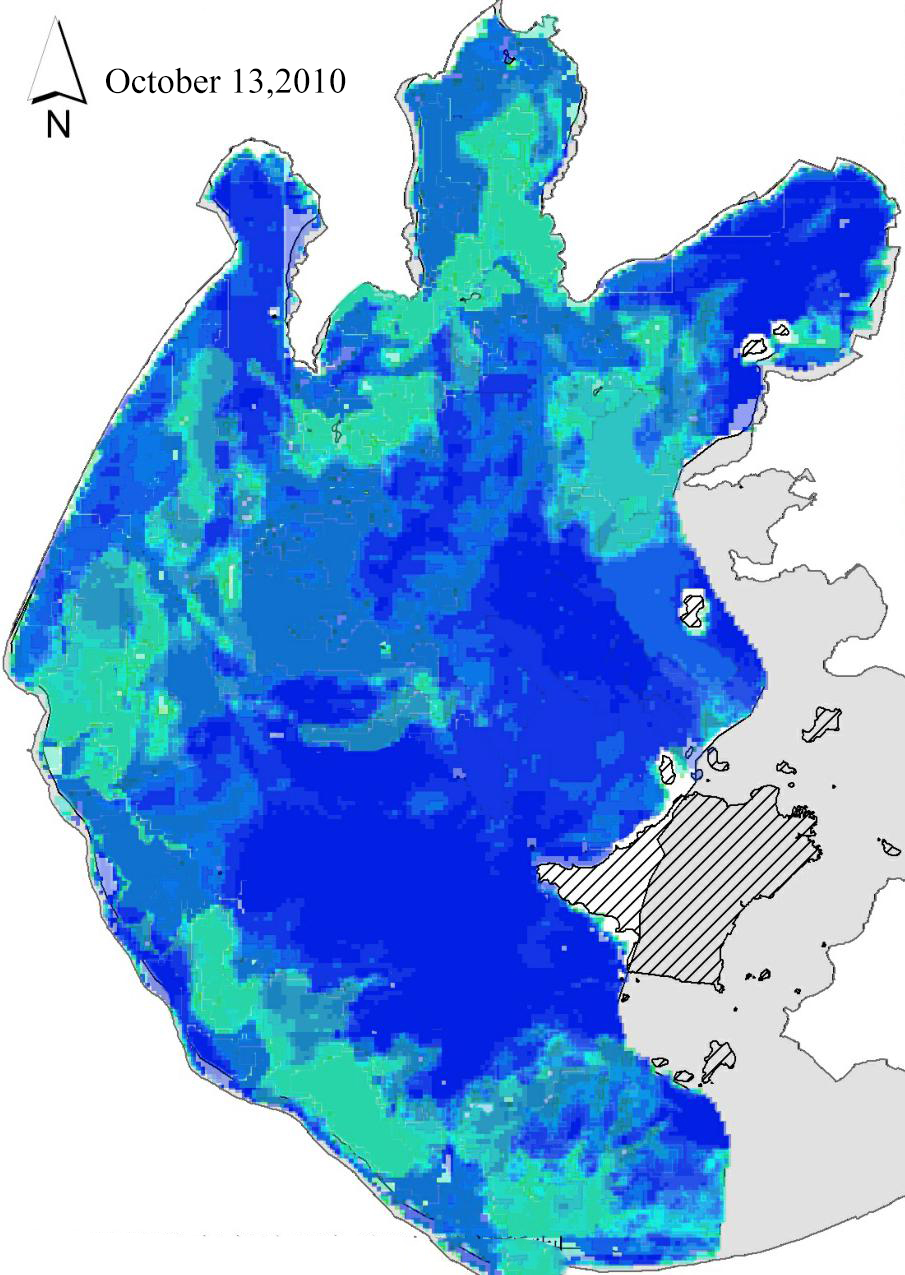

Supplement: Supplemental Information 11 — The data are remote sensing images of chlorophyll a concentration after data scale unification, remote sensing image repair, and time series filling. Remote sensing images of 30 consecutive moments were used as input to the 3D-GAN model. [file peerj-cs-09-1292-s011.zip › 201010130245.jpg]

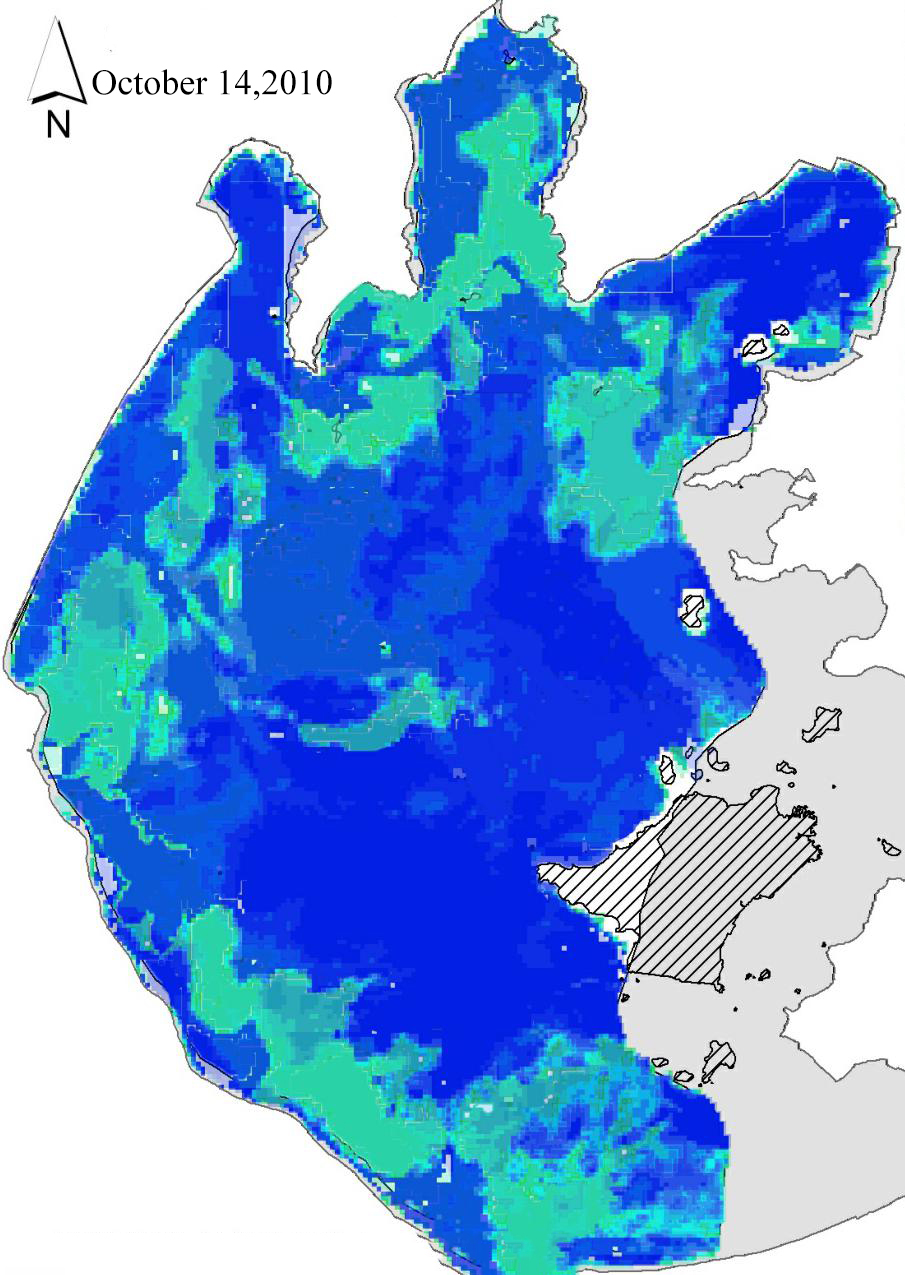

Supplement: Supplemental Information 11 — The data are remote sensing images of chlorophyll a concentration after data scale unification, remote sensing image repair, and time series filling. Remote sensing images of 30 consecutive moments were used as input to the 3D-GAN model. [file peerj-cs-09-1292-s011.zip › 201010140245.jpg]

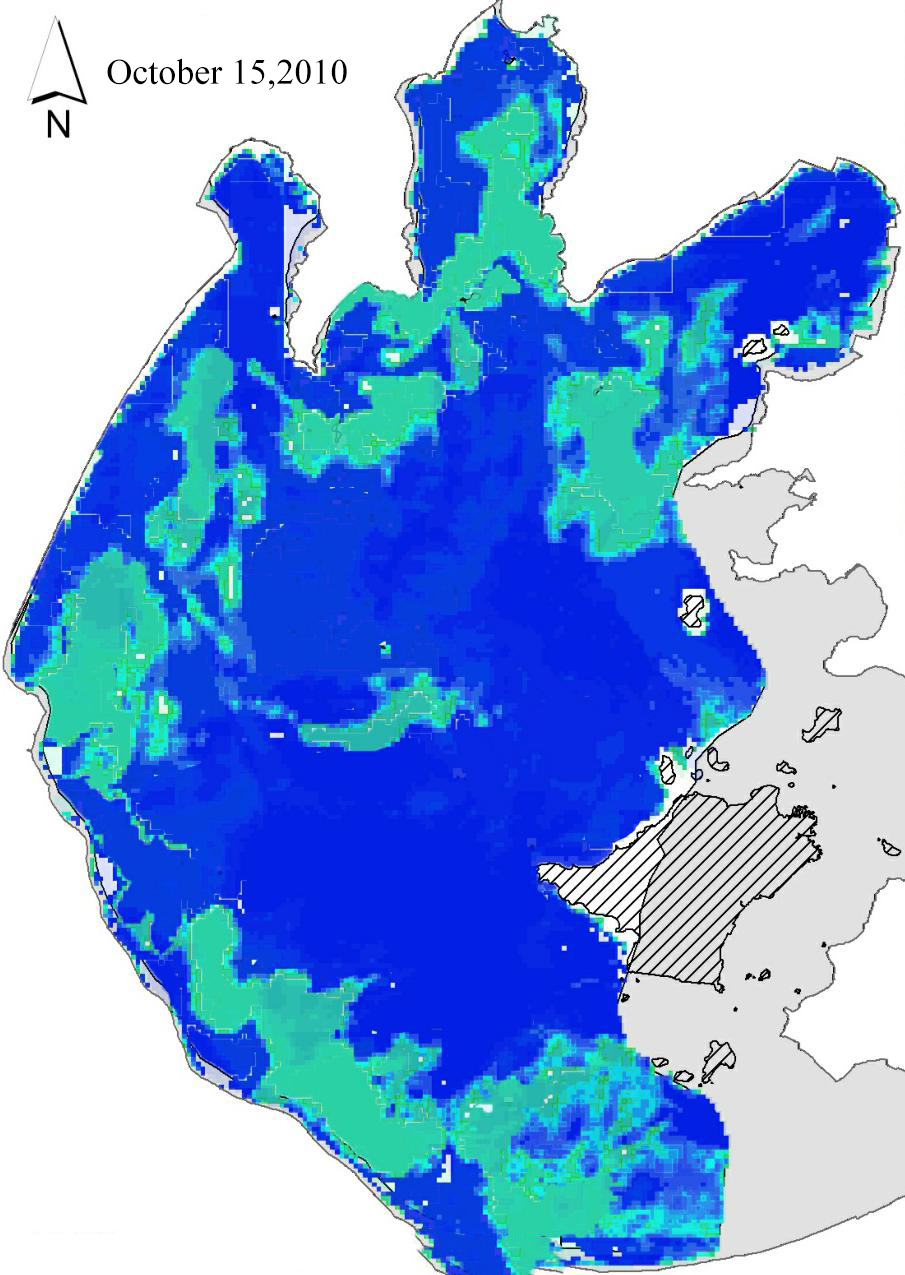

Supplement: Supplemental Information 11 — The data are remote sensing images of chlorophyll a concentration after data scale unification, remote sensing image repair, and time series filling. Remote sensing images of 30 consecutive moments were used as input to the 3D-GAN model. [file peerj-cs-09-1292-s011.zip › 201010150245.jpg]

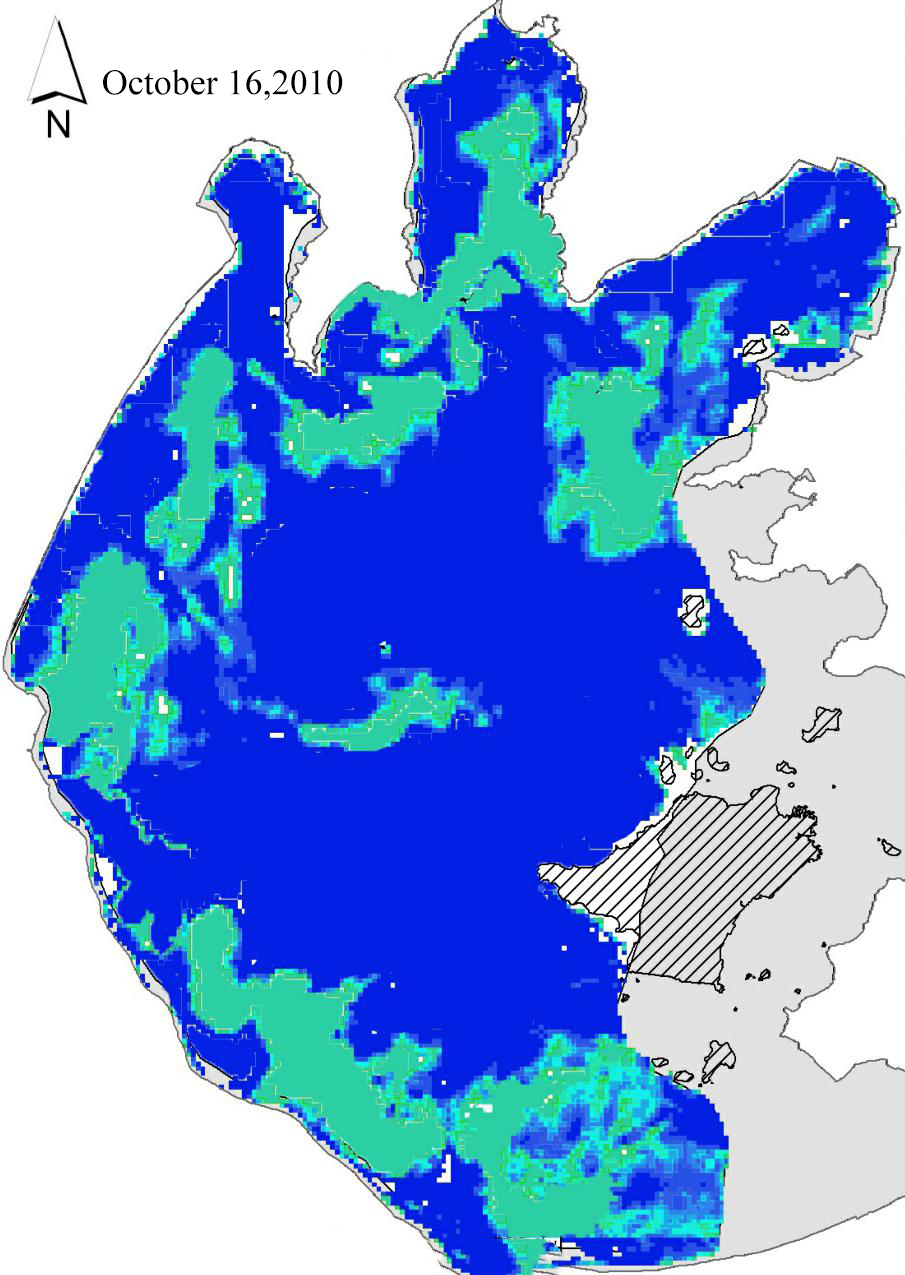

Supplement: Supplemental Information 11 — The data are remote sensing images of chlorophyll a concentration after data scale unification, remote sensing image repair, and time series filling. Remote sensing images of 30 consecutive moments were used as input to the 3D-GAN model. [file peerj-cs-09-1292-s011.zip › 201010160245.jpg]

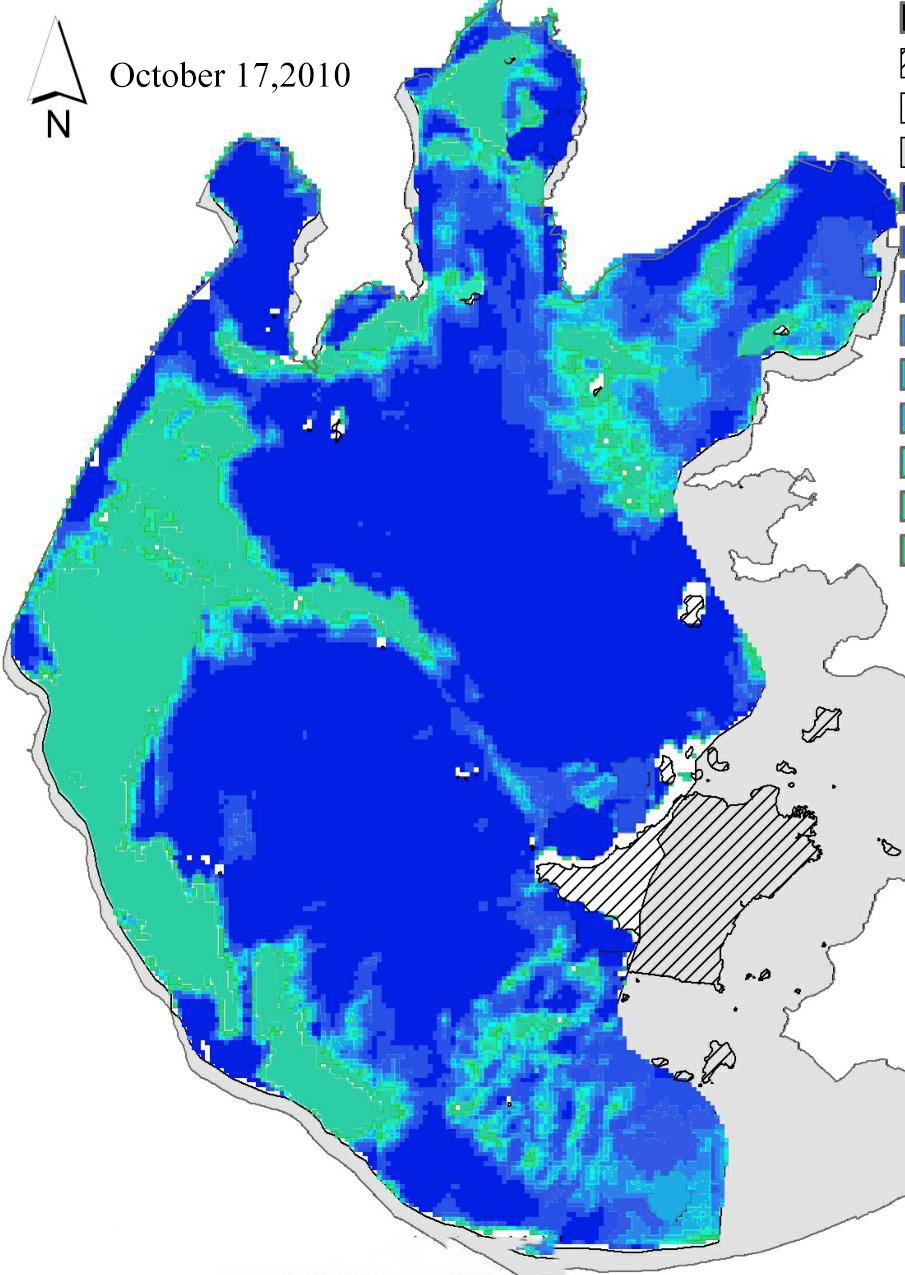

Supplement: Supplemental Information 11 — The data are remote sensing images of chlorophyll a concentration after data scale unification, remote sensing image repair, and time series filling. Remote sensing images of 30 consecutive moments were used as input to the 3D-GAN model. [file peerj-cs-09-1292-s011.zip › 201010170245.jpg]
