# Supplementary figures and images for: Remote sensing image analysis and prediction based on improved Pix2Pix model for water environment protection of smart cities (part 6 of 6)
Source: PeerJ Comput Sci. 2023 Apr 26;9:e1292. doi: 10.7717/peerj-cs.1292 (PMC10280440; doi:10.7717/peerj-cs.1292)

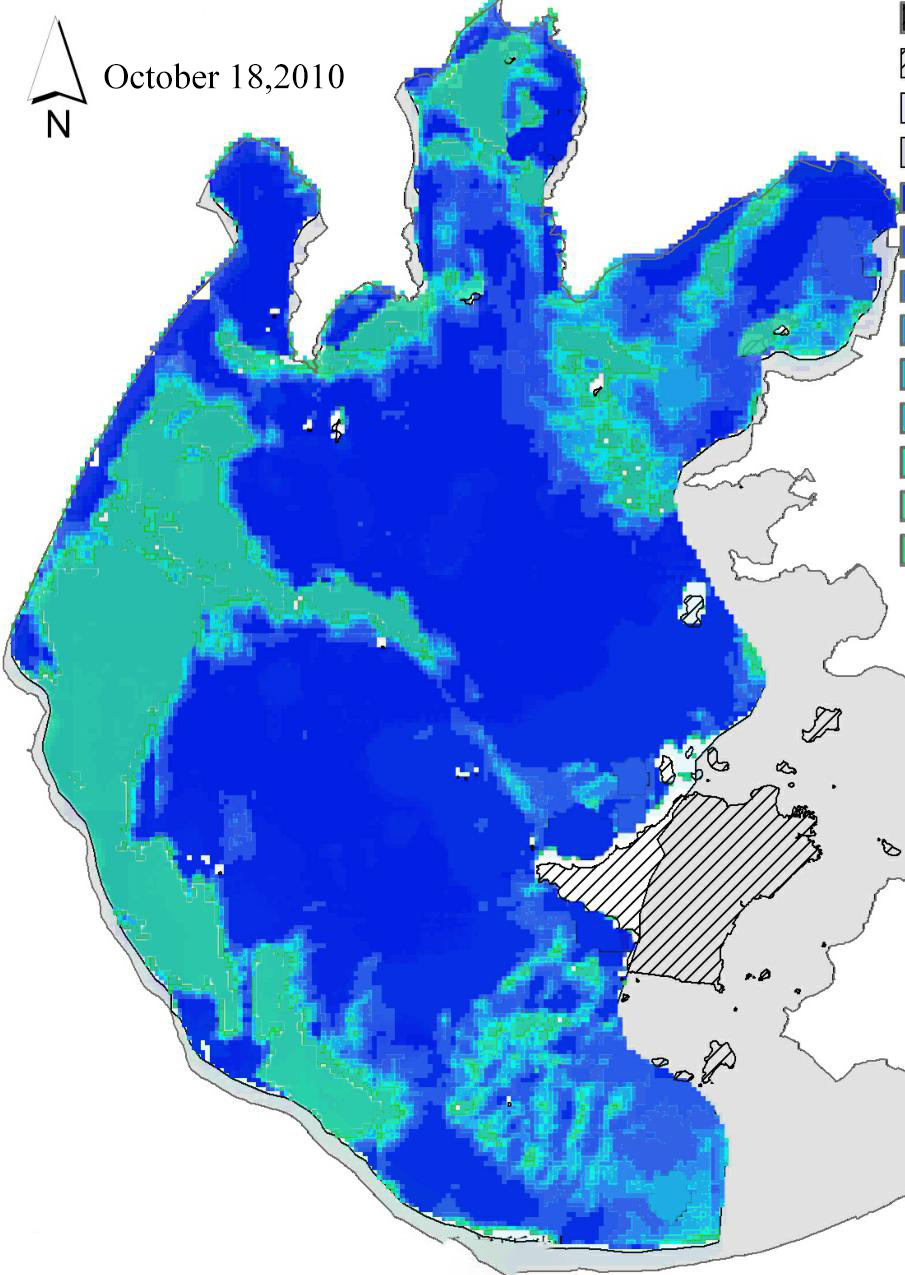

Supplement: Supplemental Information 11 — The data are remote sensing images of chlorophyll a concentration after data scale unification, remote sensing image repair, and time series filling. Remote sensing images of 30 consecutive moments were used as input to the 3D-GAN model. [file peerj-cs-09-1292-s011.zip › 201010180245.jpg]

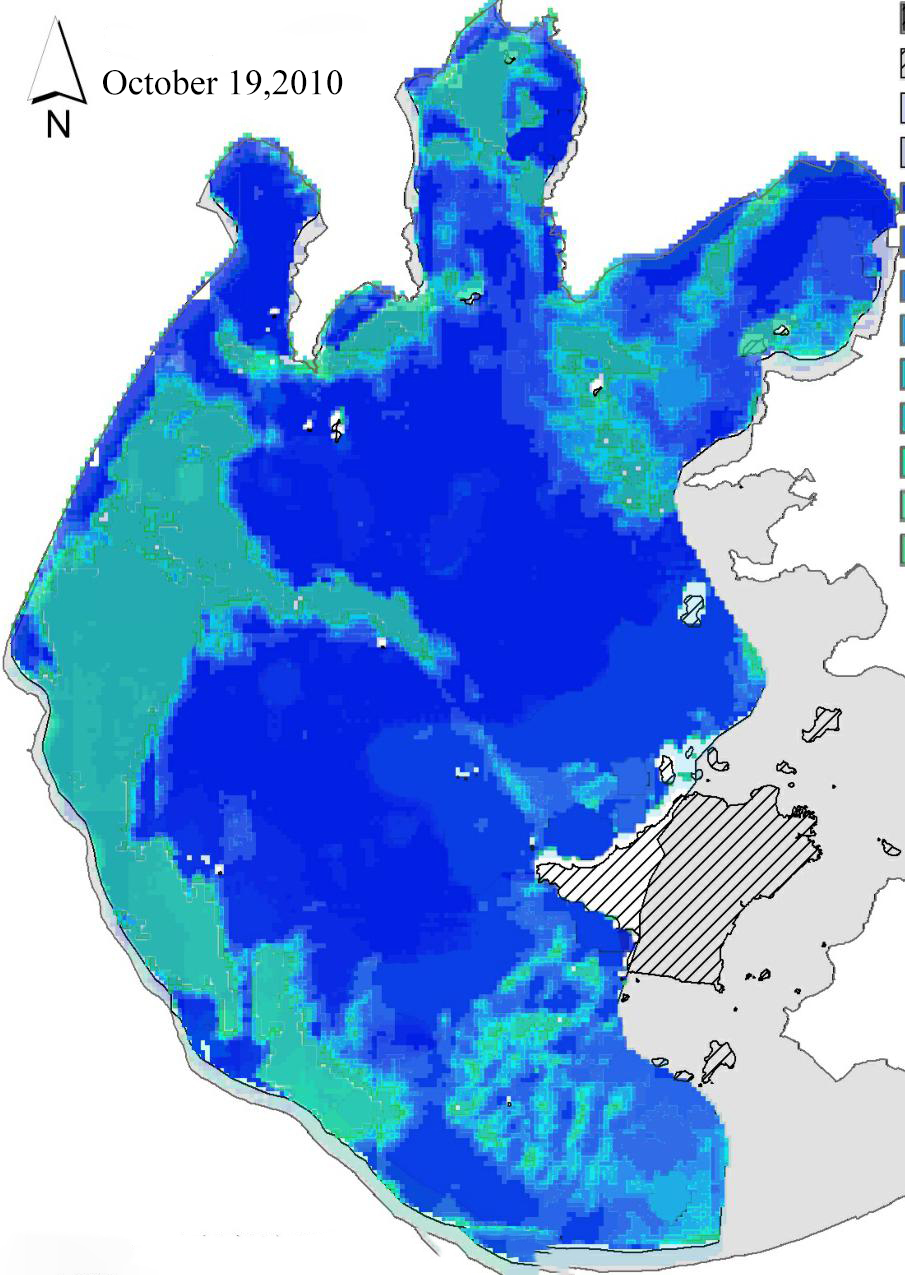

Supplement: Supplemental Information 11 — The data are remote sensing images of chlorophyll a concentration after data scale unification, remote sensing image repair, and time series filling. Remote sensing images of 30 consecutive moments were used as input to the 3D-GAN model. [file peerj-cs-09-1292-s011.zip › 201010190245.jpg]

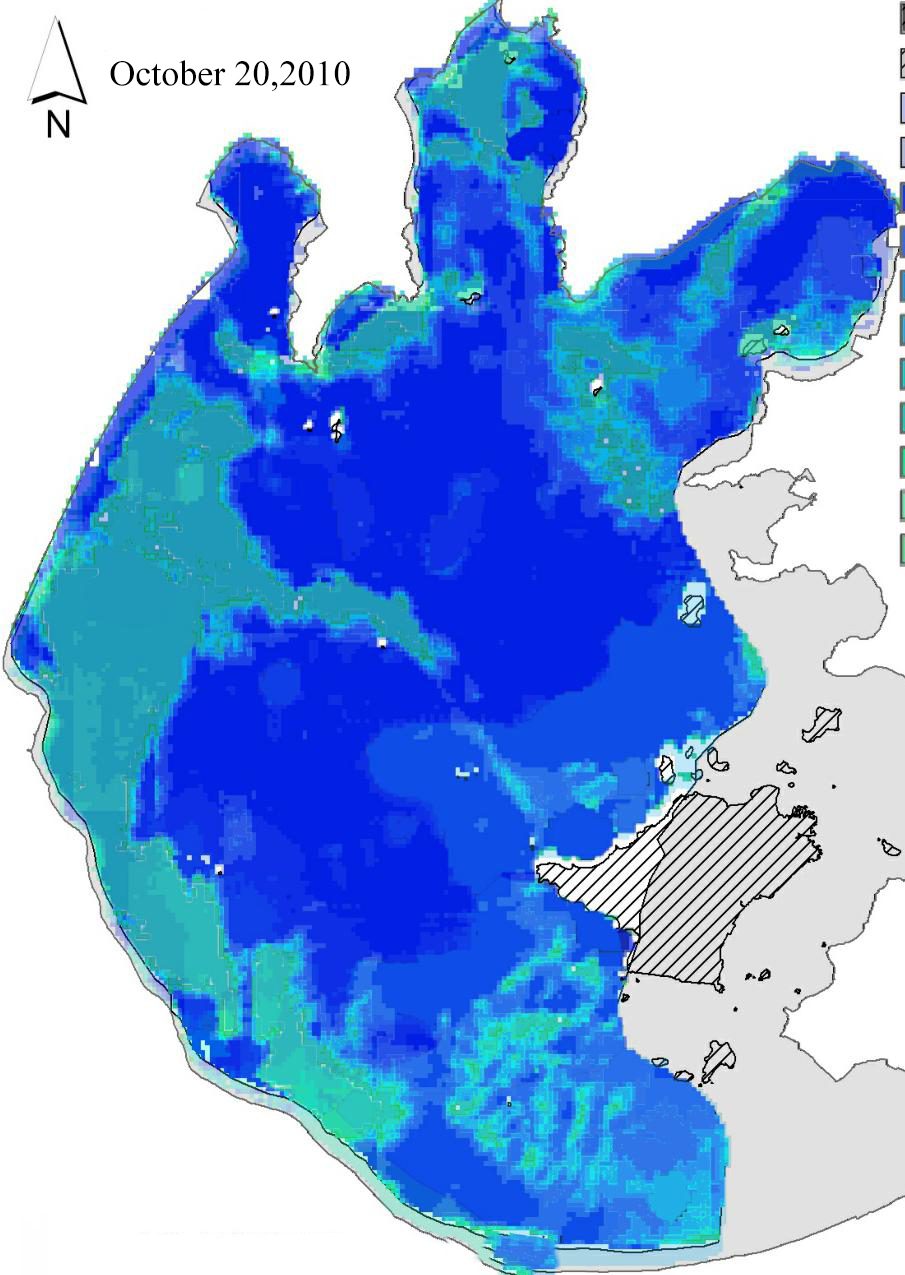

Supplement: Supplemental Information 11 — The data are remote sensing images of chlorophyll a concentration after data scale unification, remote sensing image repair, and time series filling. Remote sensing images of 30 consecutive moments were used as input to the 3D-GAN model. [file peerj-cs-09-1292-s011.zip › 201010200245.jpg]

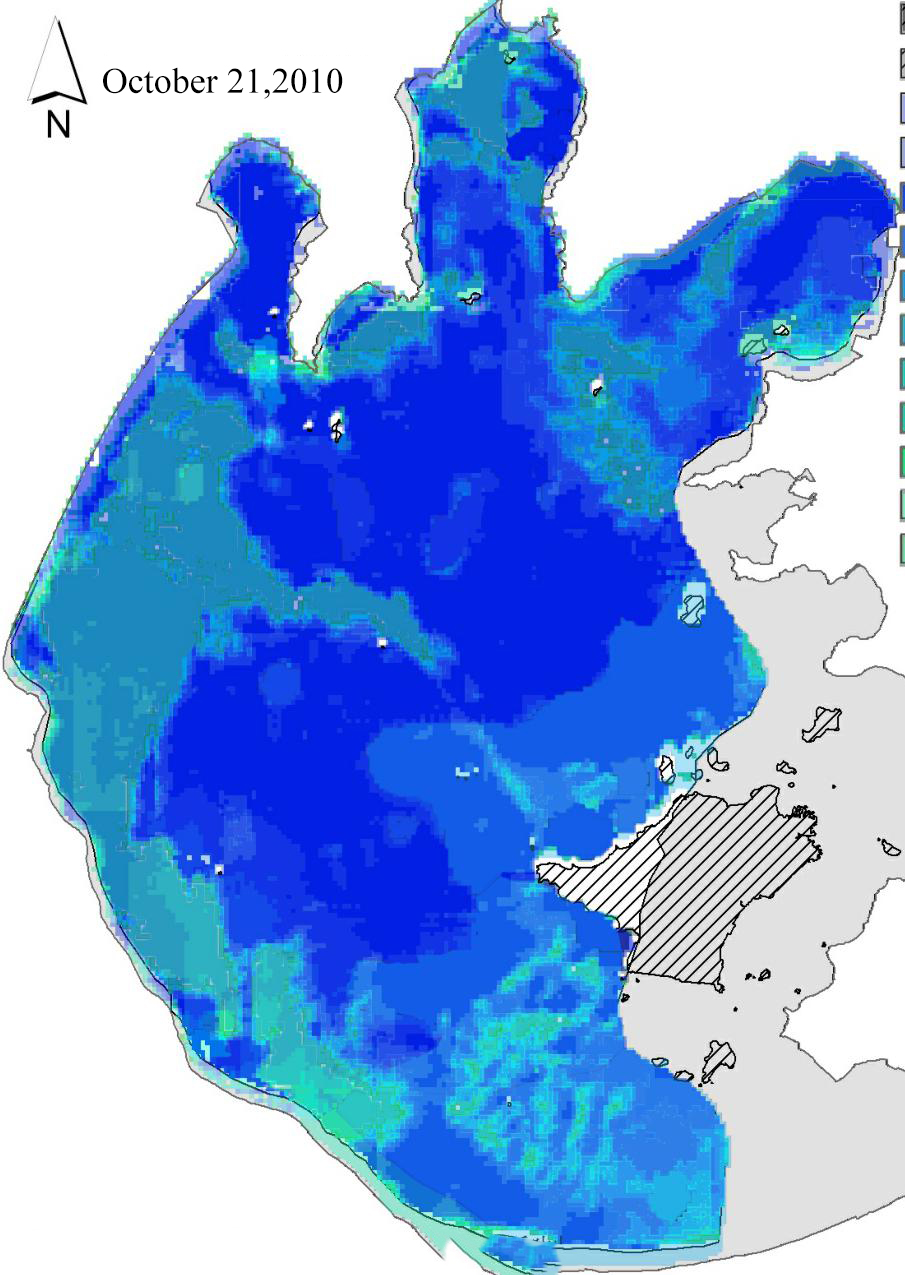

Supplement: Supplemental Information 11 — The data are remote sensing images of chlorophyll a concentration after data scale unification, remote sensing image repair, and time series filling. Remote sensing images of 30 consecutive moments were used as input to the 3D-GAN model. [file peerj-cs-09-1292-s011.zip › 201010210245.jpg]

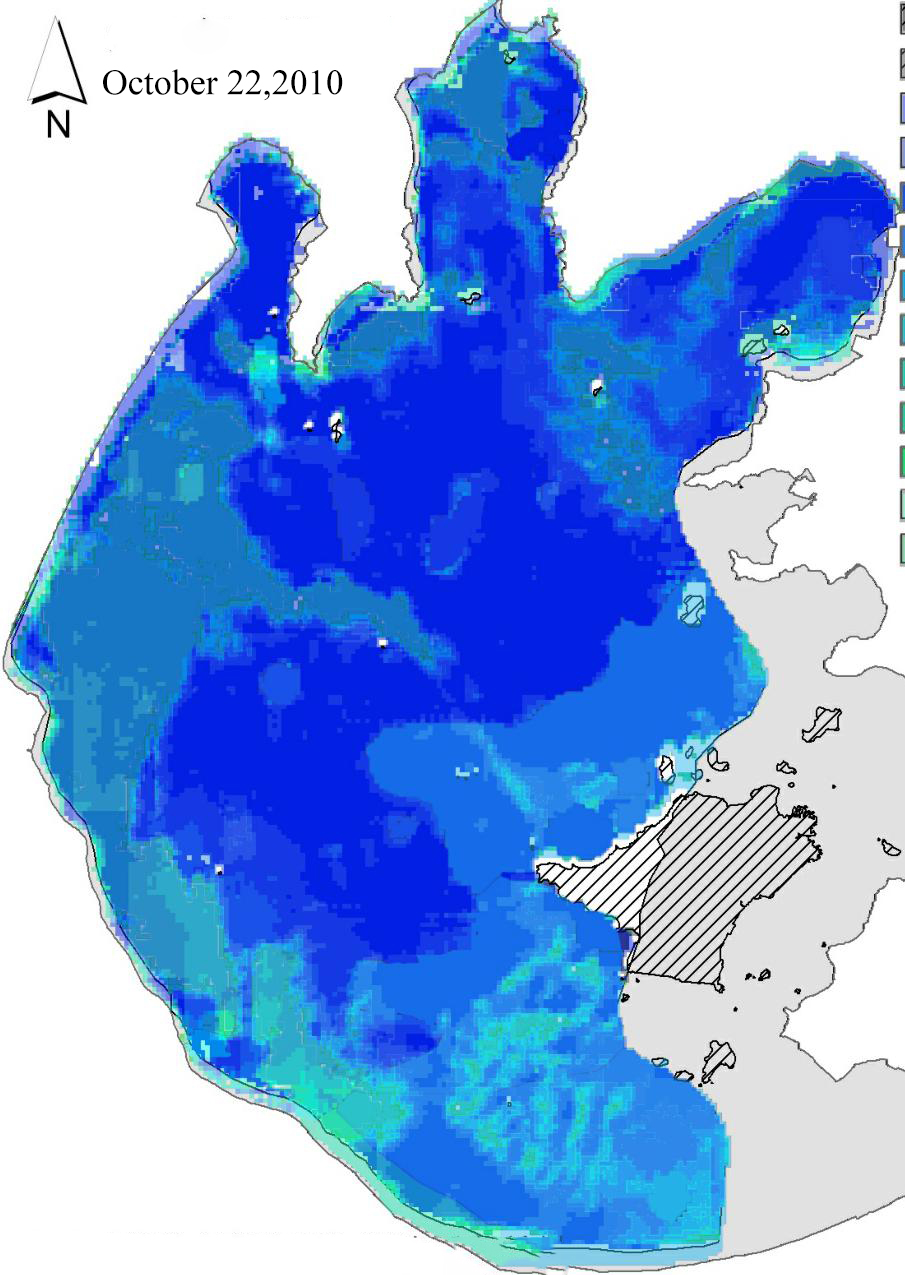

Supplement: Supplemental Information 11 — The data are remote sensing images of chlorophyll a concentration after data scale unification, remote sensing image repair, and time series filling. Remote sensing images of 30 consecutive moments were used as input to the 3D-GAN model. [file peerj-cs-09-1292-s011.zip › 201010220245.jpg]

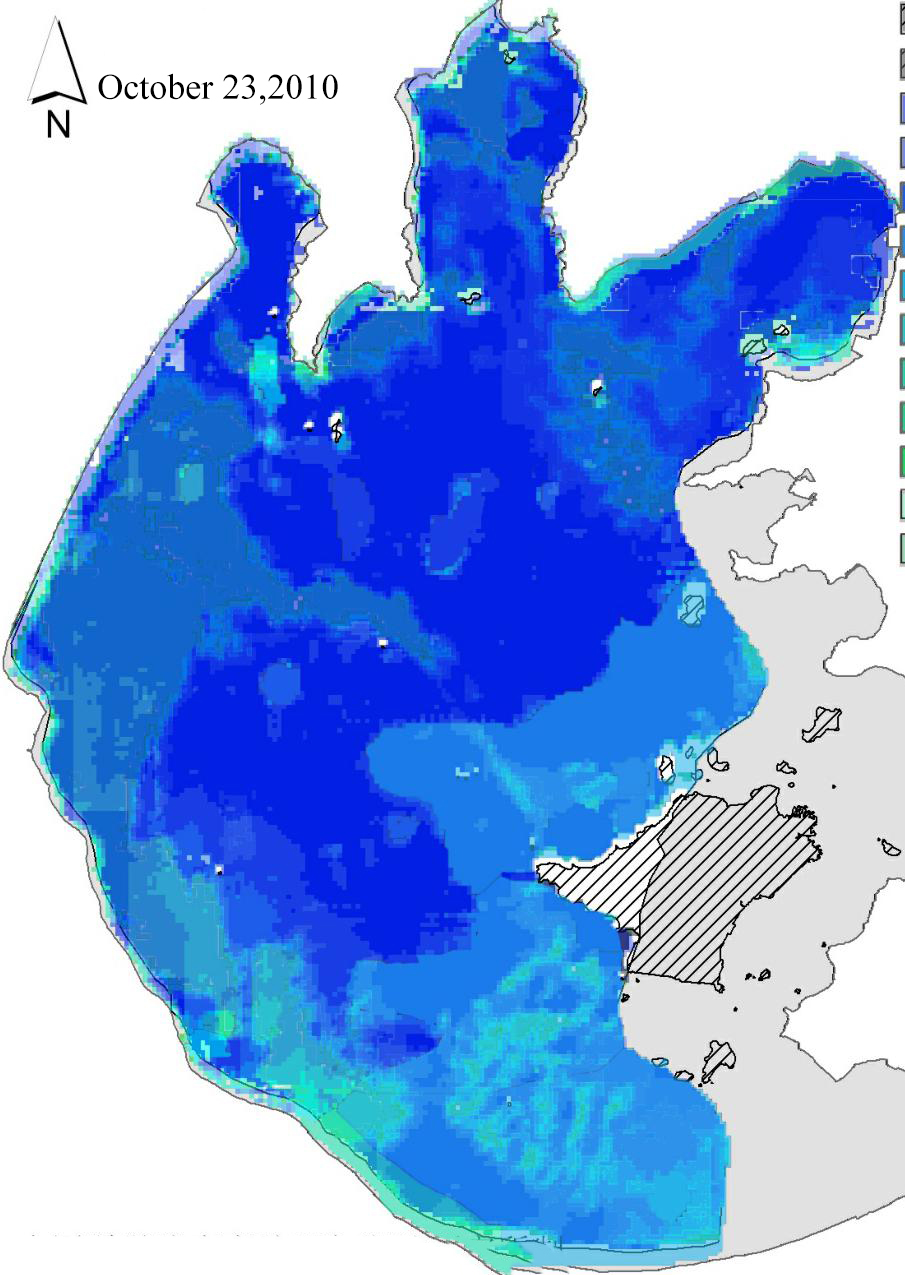

Supplement: Supplemental Information 11 — The data are remote sensing images of chlorophyll a concentration after data scale unification, remote sensing image repair, and time series filling. Remote sensing images of 30 consecutive moments were used as input to the 3D-GAN model. [file peerj-cs-09-1292-s011.zip › 201010230245.jpg]

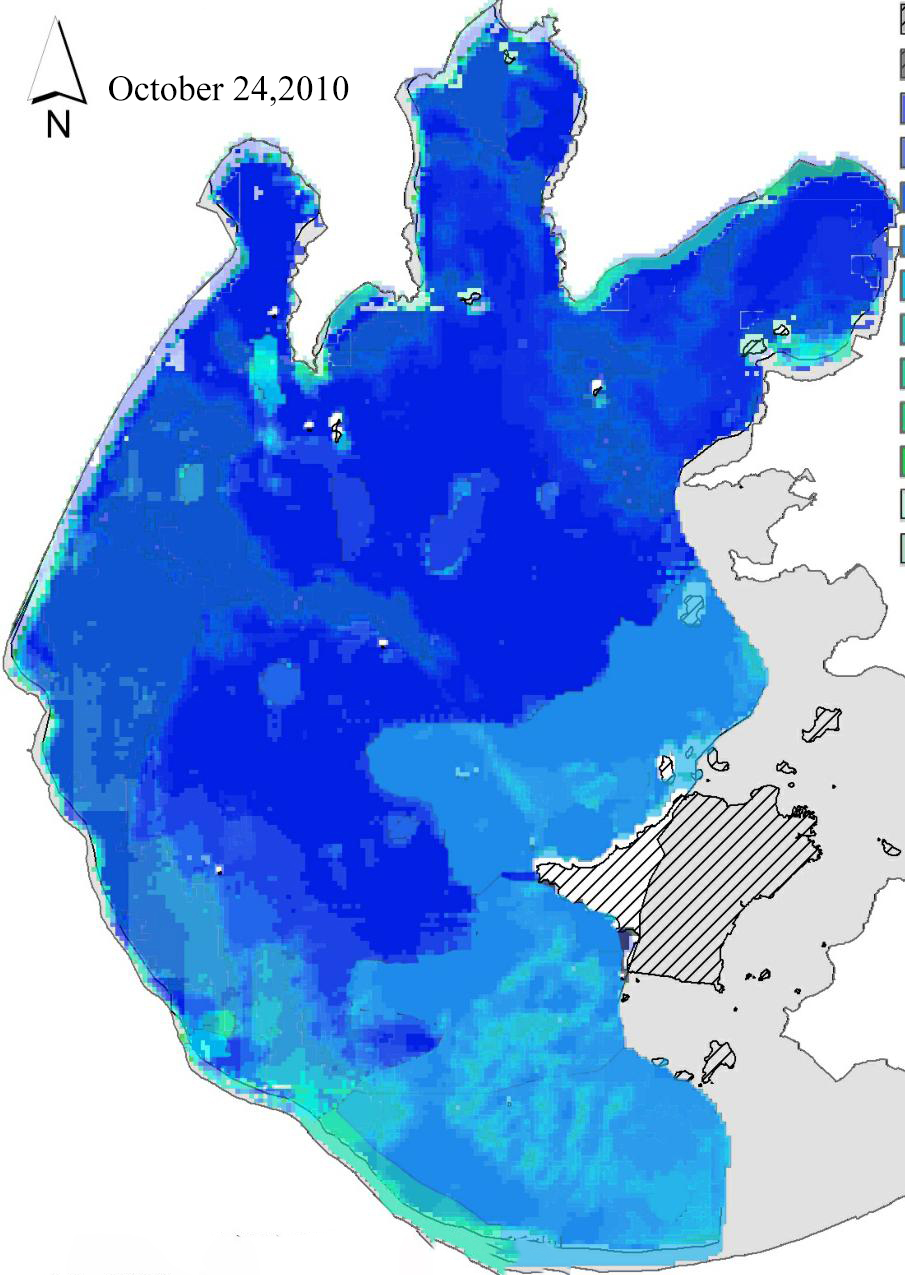

Supplement: Supplemental Information 11 — The data are remote sensing images of chlorophyll a concentration after data scale unification, remote sensing image repair, and time series filling. Remote sensing images of 30 consecutive moments were used as input to the 3D-GAN model. [file peerj-cs-09-1292-s011.zip › 201010240245.jpg]

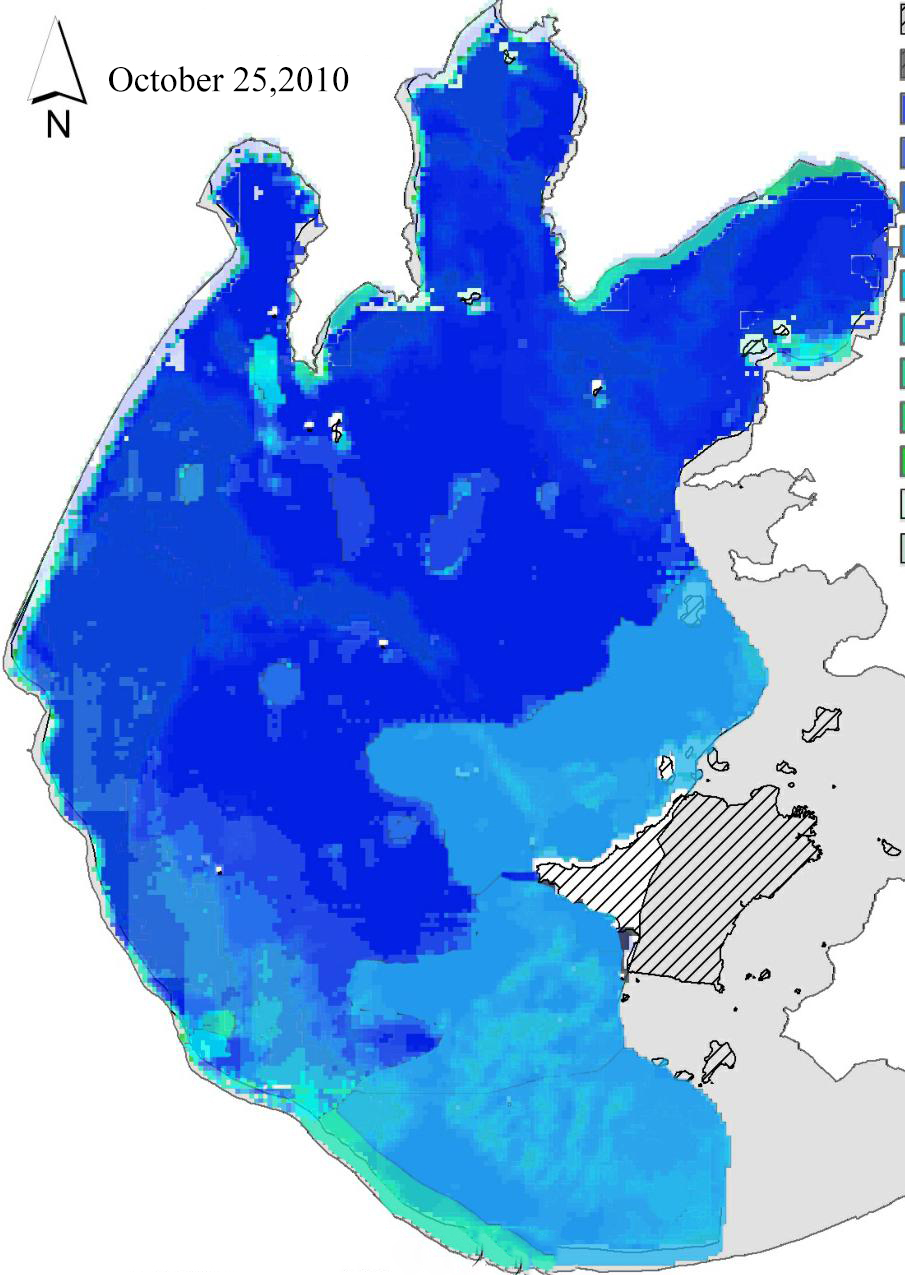

Supplement: Supplemental Information 11 — The data are remote sensing images of chlorophyll a concentration after data scale unification, remote sensing image repair, and time series filling. Remote sensing images of 30 consecutive moments were used as input to the 3D-GAN model. [file peerj-cs-09-1292-s011.zip › 201010250245.jpg]

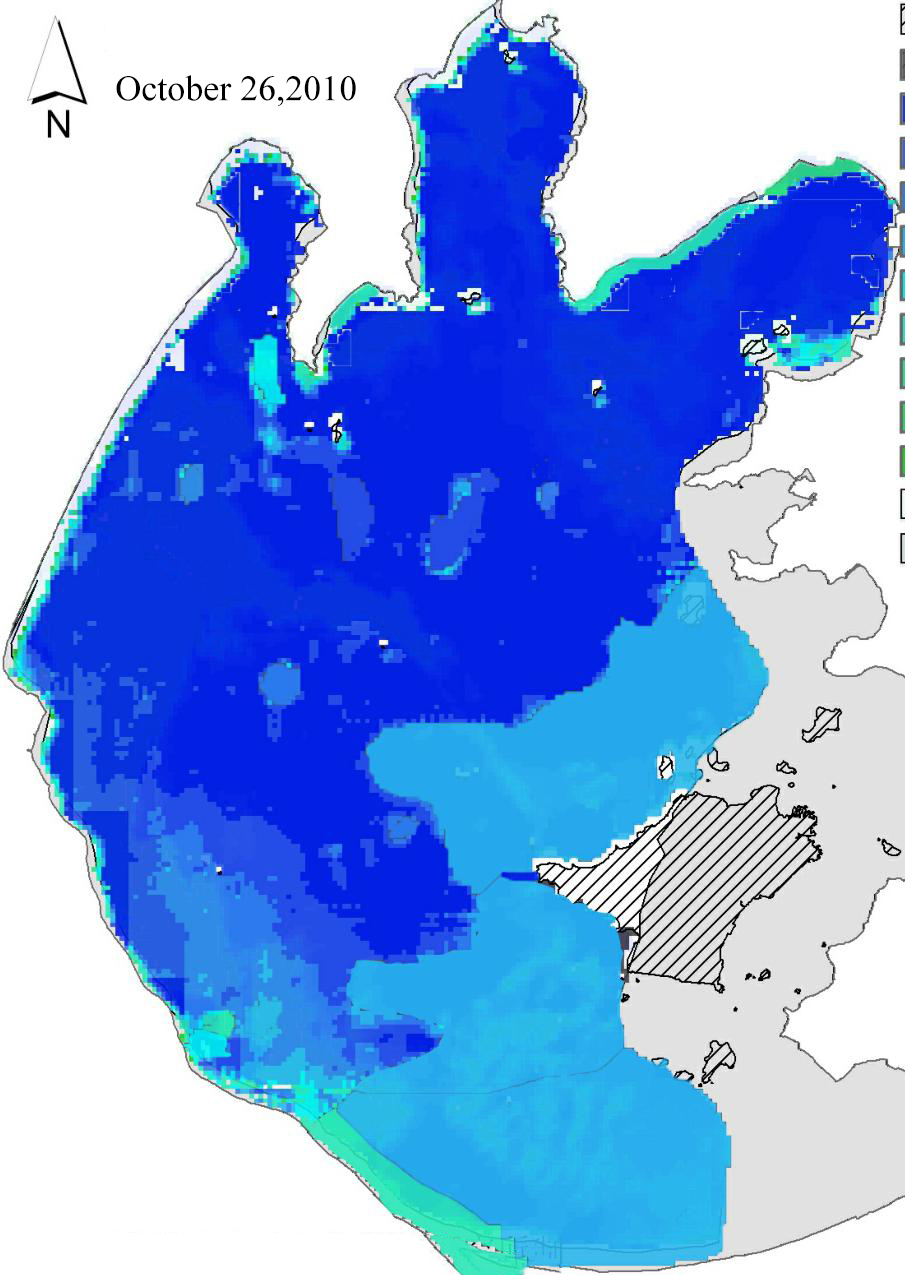

Supplement: Supplemental Information 11 — The data are remote sensing images of chlorophyll a concentration after data scale unification, remote sensing image repair, and time series filling. Remote sensing images of 30 consecutive moments were used as input to the 3D-GAN model. [file peerj-cs-09-1292-s011.zip › 201010260245.jpg]

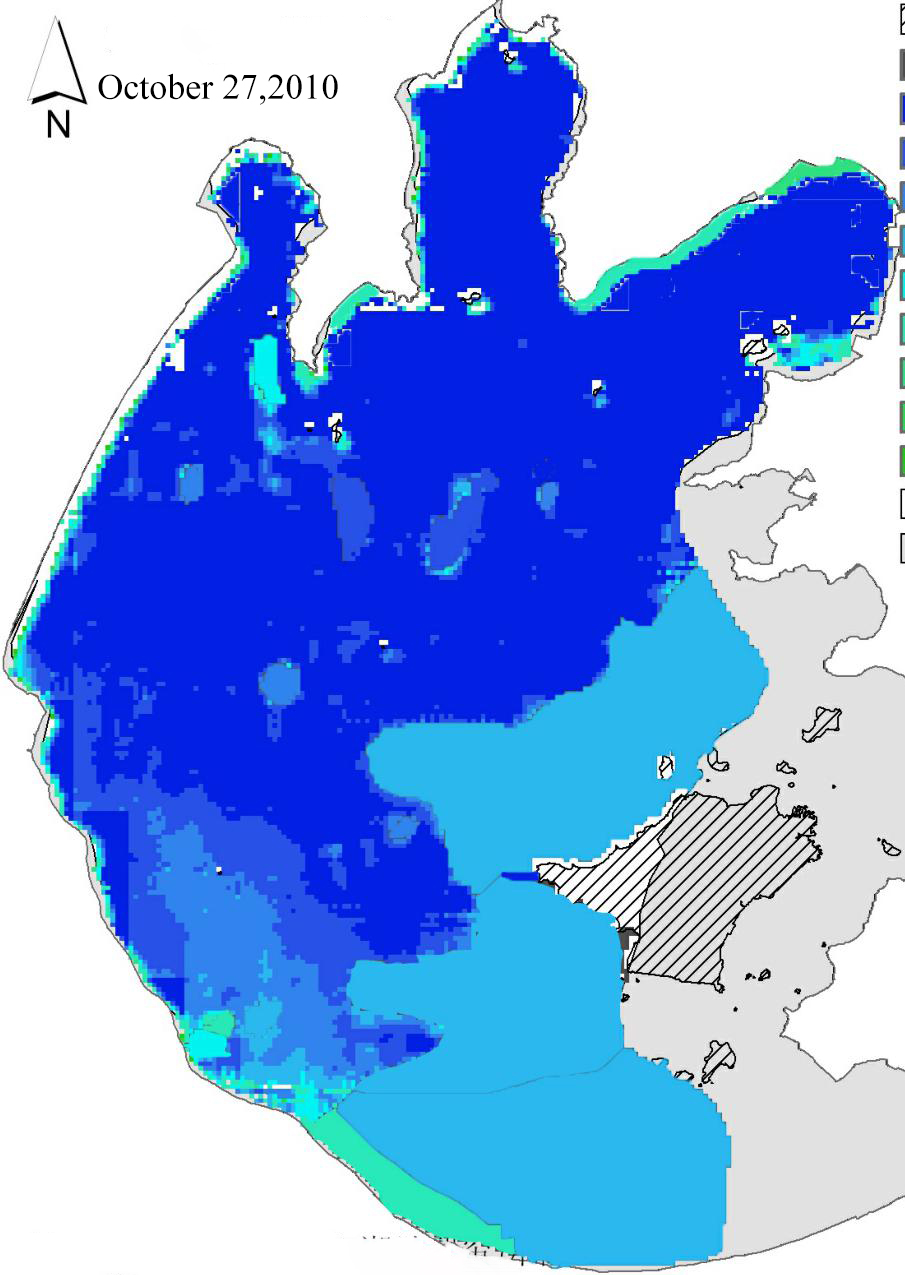

Supplement: Supplemental Information 11 — The data are remote sensing images of chlorophyll a concentration after data scale unification, remote sensing image repair, and time series filling. Remote sensing images of 30 consecutive moments were used as input to the 3D-GAN model. [file peerj-cs-09-1292-s011.zip › 201010270245.jpg]

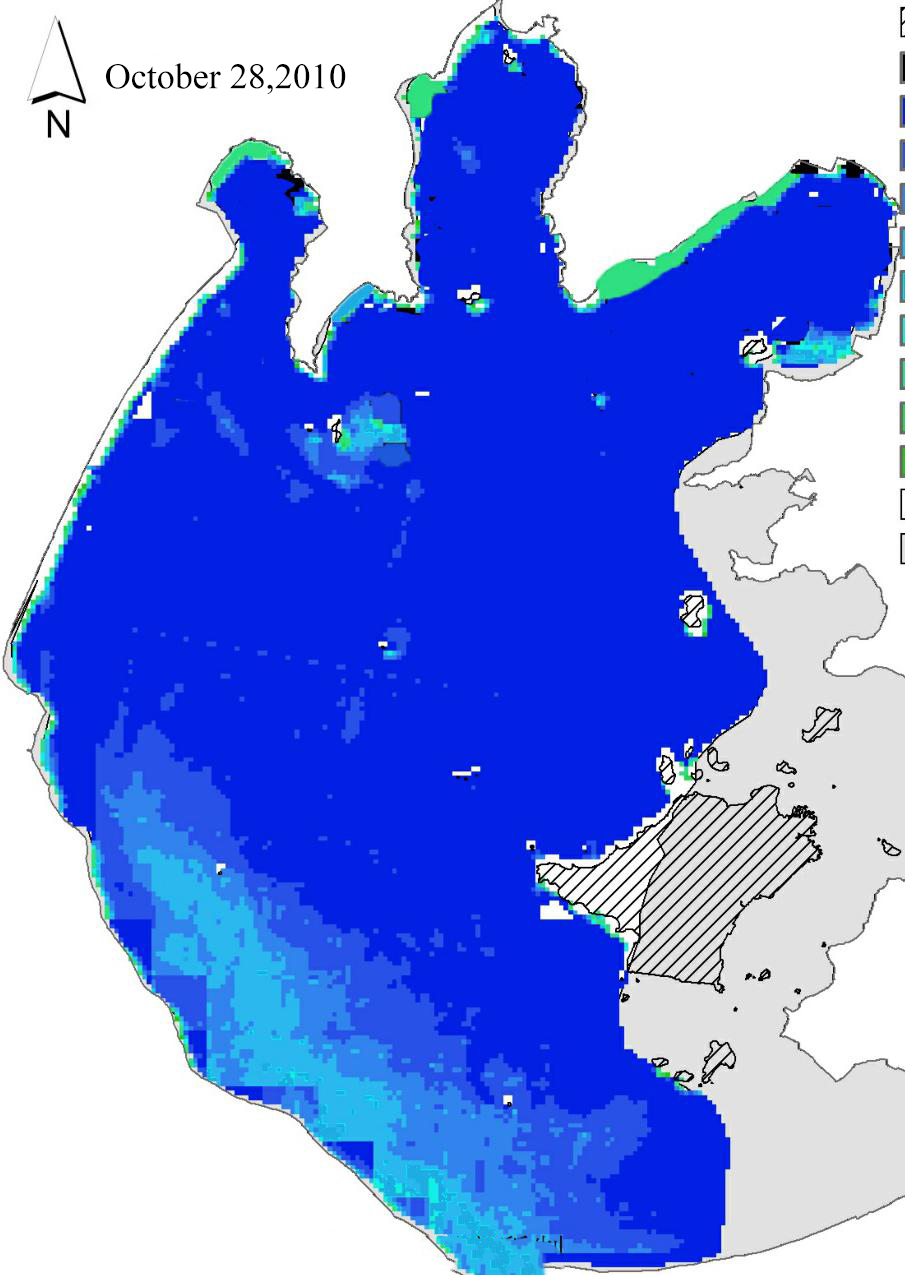

Supplement: Supplemental Information 11 — The data are remote sensing images of chlorophyll a concentration after data scale unification, remote sensing image repair, and time series filling. Remote sensing images of 30 consecutive moments were used as input to the 3D-GAN model. [file peerj-cs-09-1292-s011.zip › 201010280245.jpg]

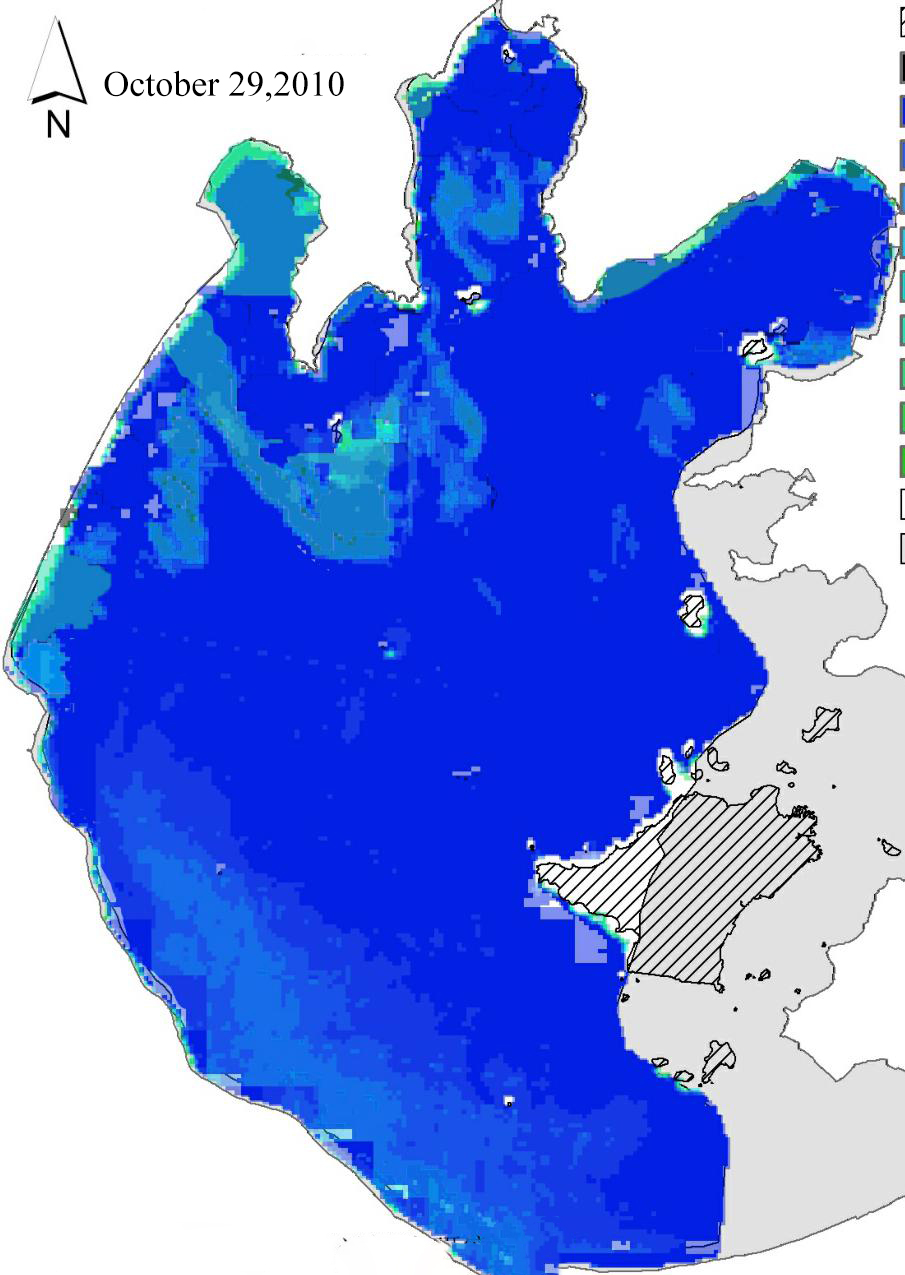

Supplement: Supplemental Information 11 — The data are remote sensing images of chlorophyll a concentration after data scale unification, remote sensing image repair, and time series filling. Remote sensing images of 30 consecutive moments were used as input to the 3D-GAN model. [file peerj-cs-09-1292-s011.zip › 201010290245.jpg]

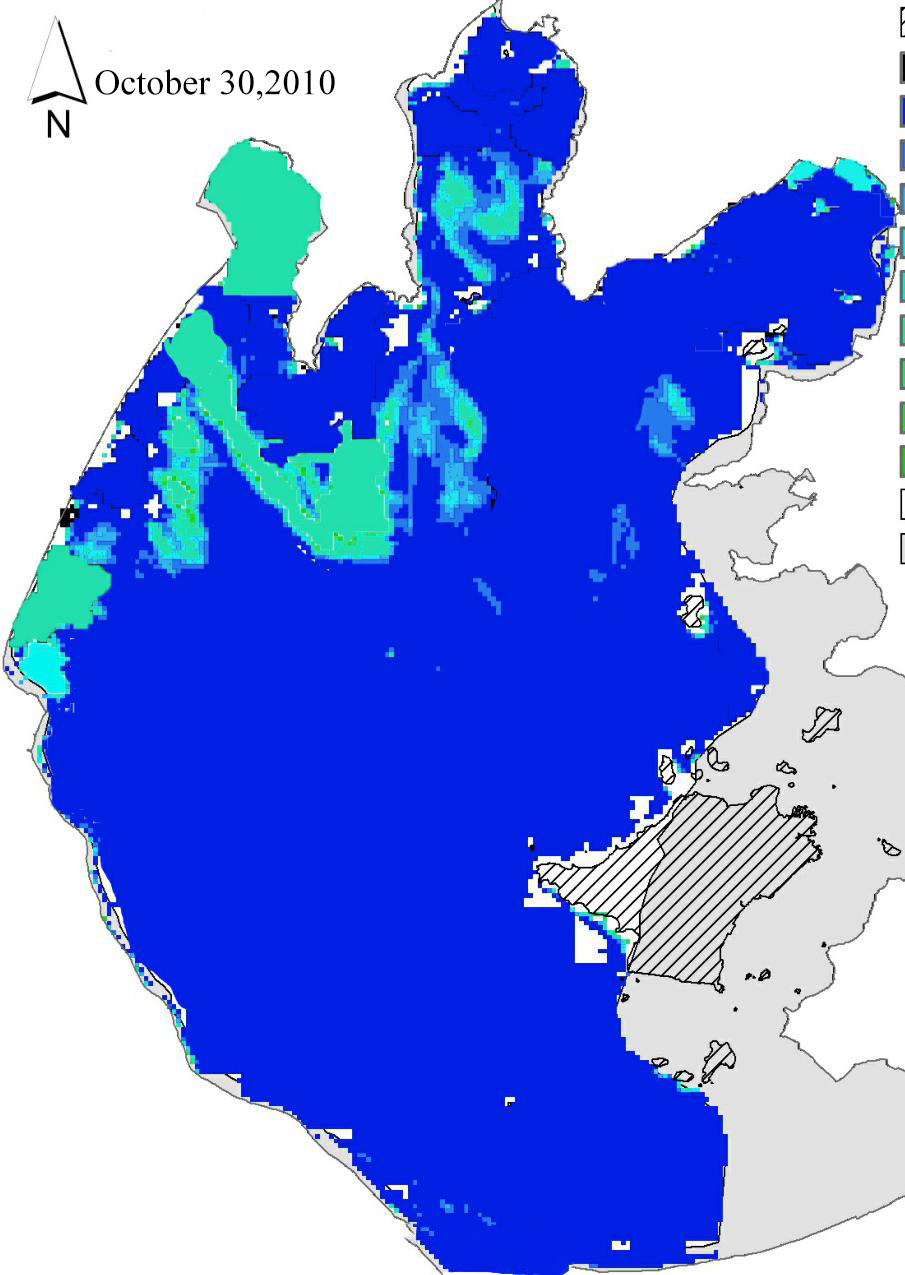

Supplement: Supplemental Information 11 — The data are remote sensing images of chlorophyll a concentration after data scale unification, remote sensing image repair, and time series filling. Remote sensing images of 30 consecutive moments were used as input to the 3D-GAN model. [file peerj-cs-09-1292-s011.zip › 201010300245.jpg]

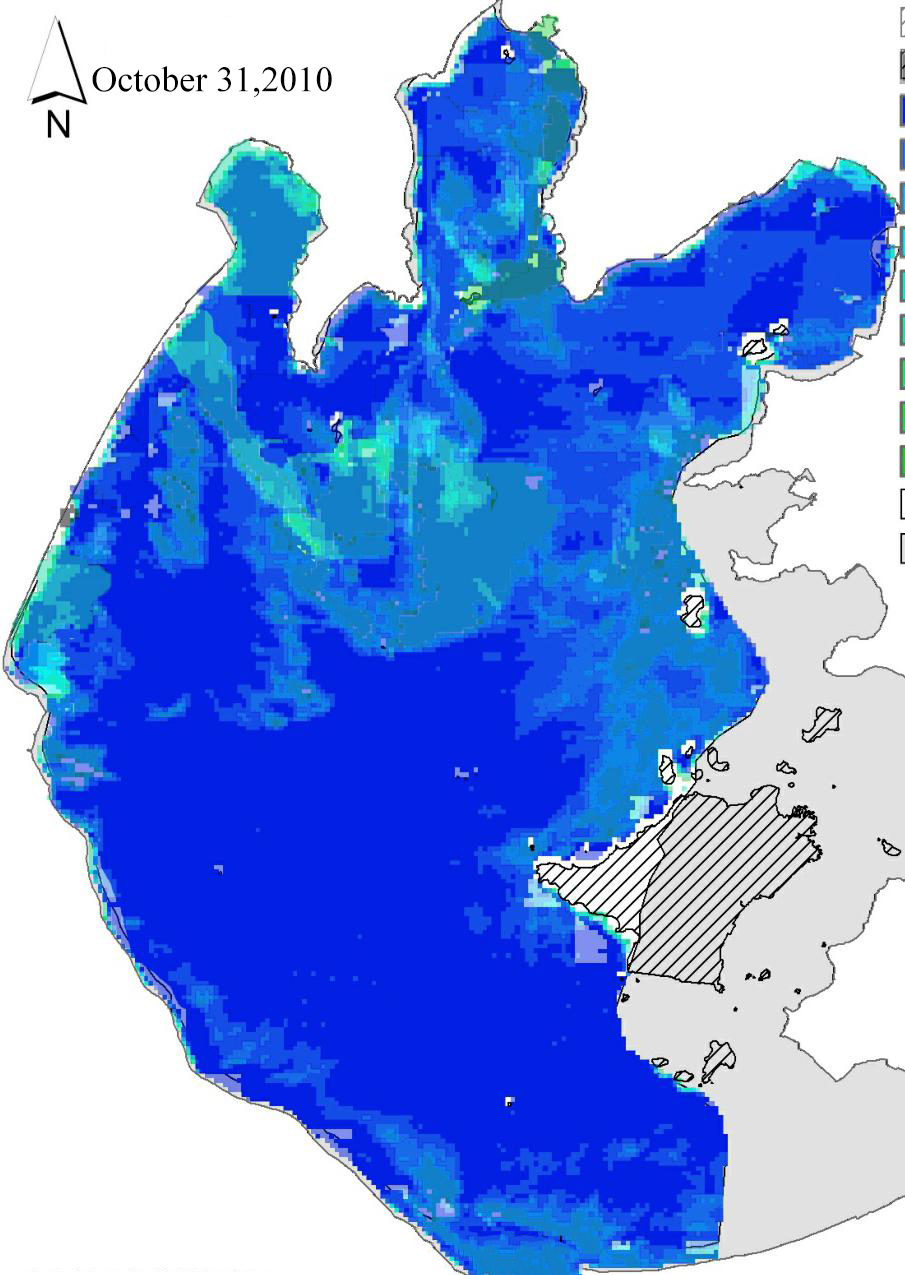

Supplement: Supplemental Information 11 — The data are remote sensing images of chlorophyll a concentration after data scale unification, remote sensing image repair, and time series filling. Remote sensing images of 30 consecutive moments were used as input to the 3D-GAN model. [file peerj-cs-09-1292-s011.zip › 201010310245.jpg]

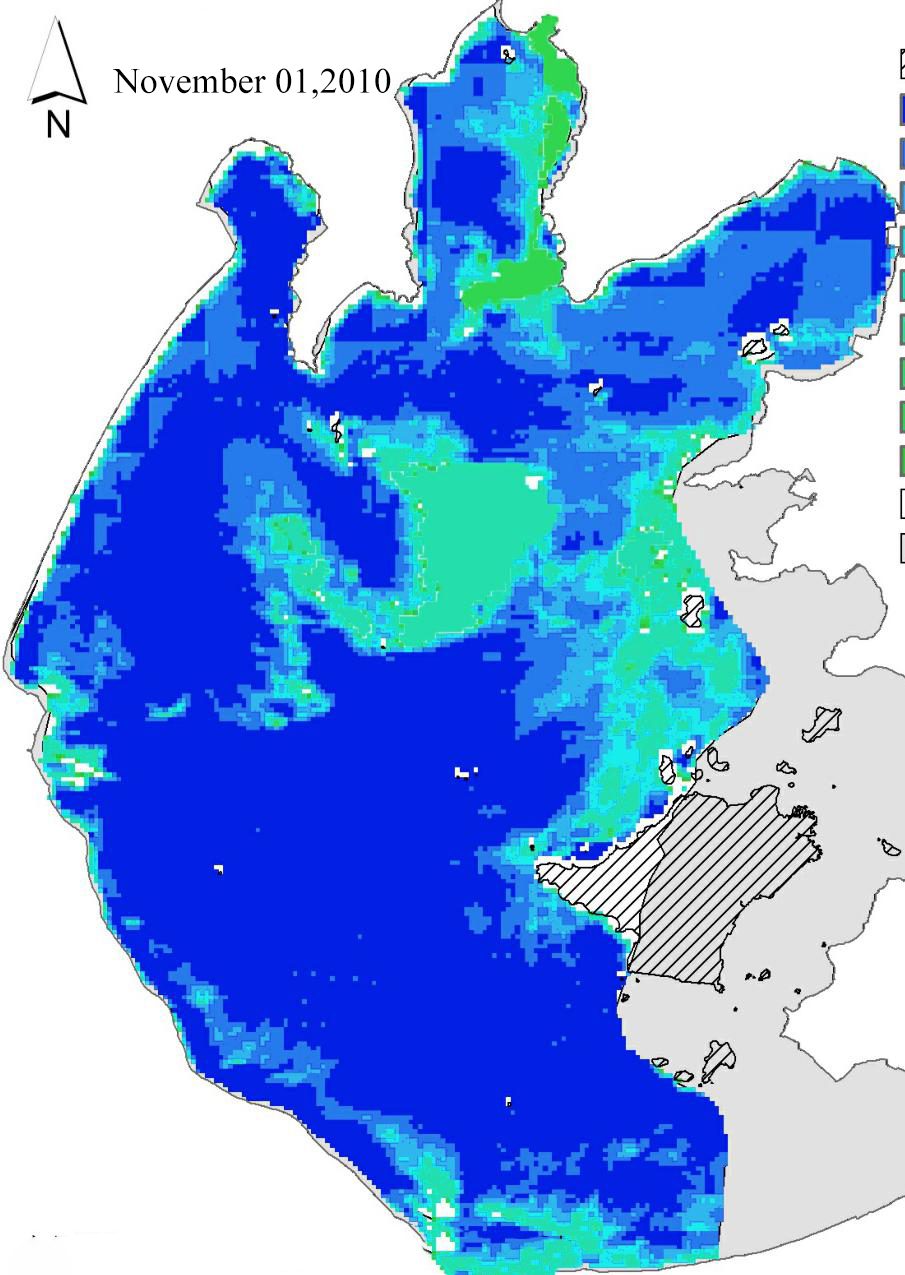

Supplement: Supplemental Information 11 — The data are remote sensing images of chlorophyll a concentration after data scale unification, remote sensing image repair, and time series filling. Remote sensing images of 30 consecutive moments were used as input to the 3D-GAN model. [file peerj-cs-09-1292-s011.zip › 201011010245.jpg]

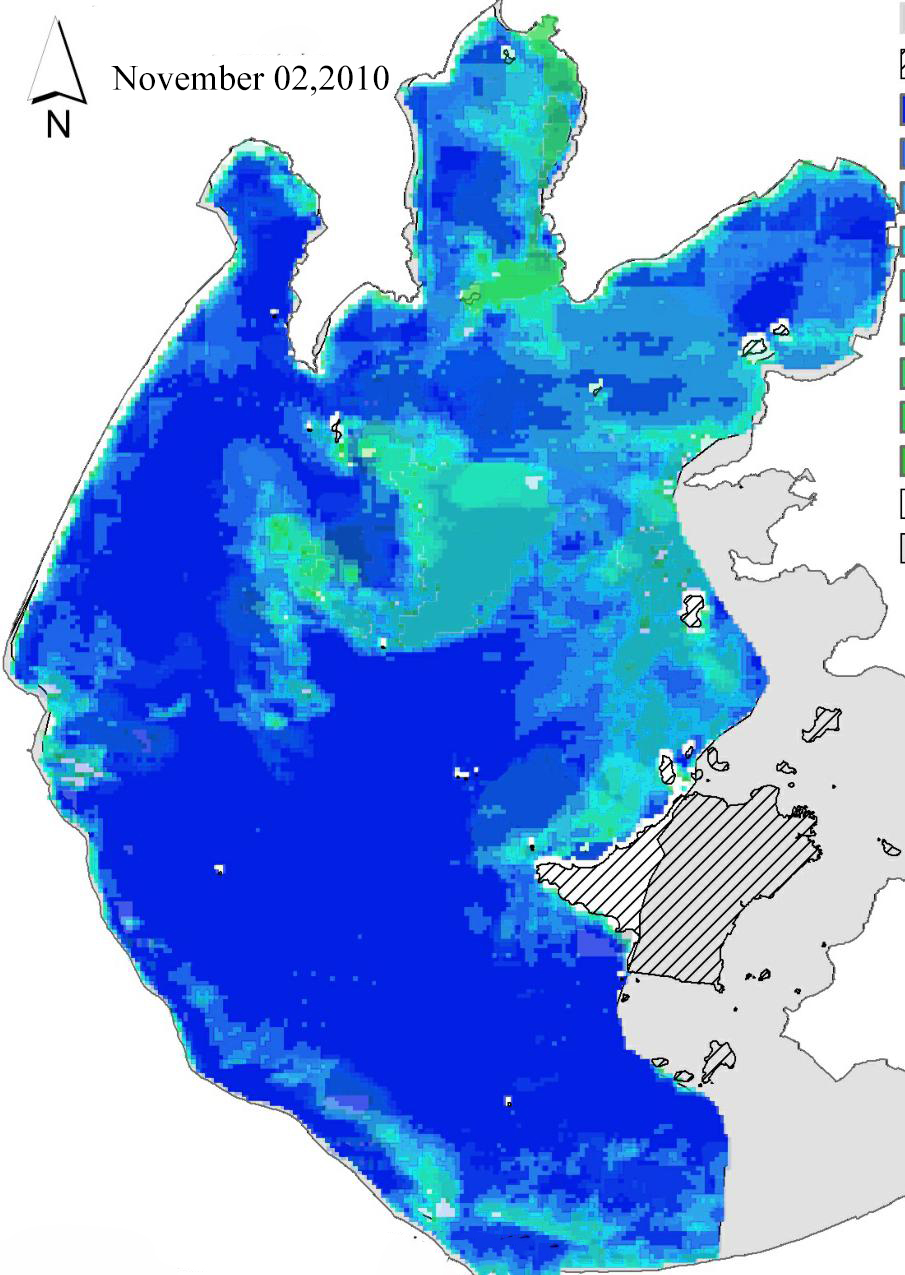

Supplement: Supplemental Information 11 — The data are remote sensing images of chlorophyll a concentration after data scale unification, remote sensing image repair, and time series filling. Remote sensing images of 30 consecutive moments were used as input to the 3D-GAN model. [file peerj-cs-09-1292-s011.zip › 201011020245.jpg]

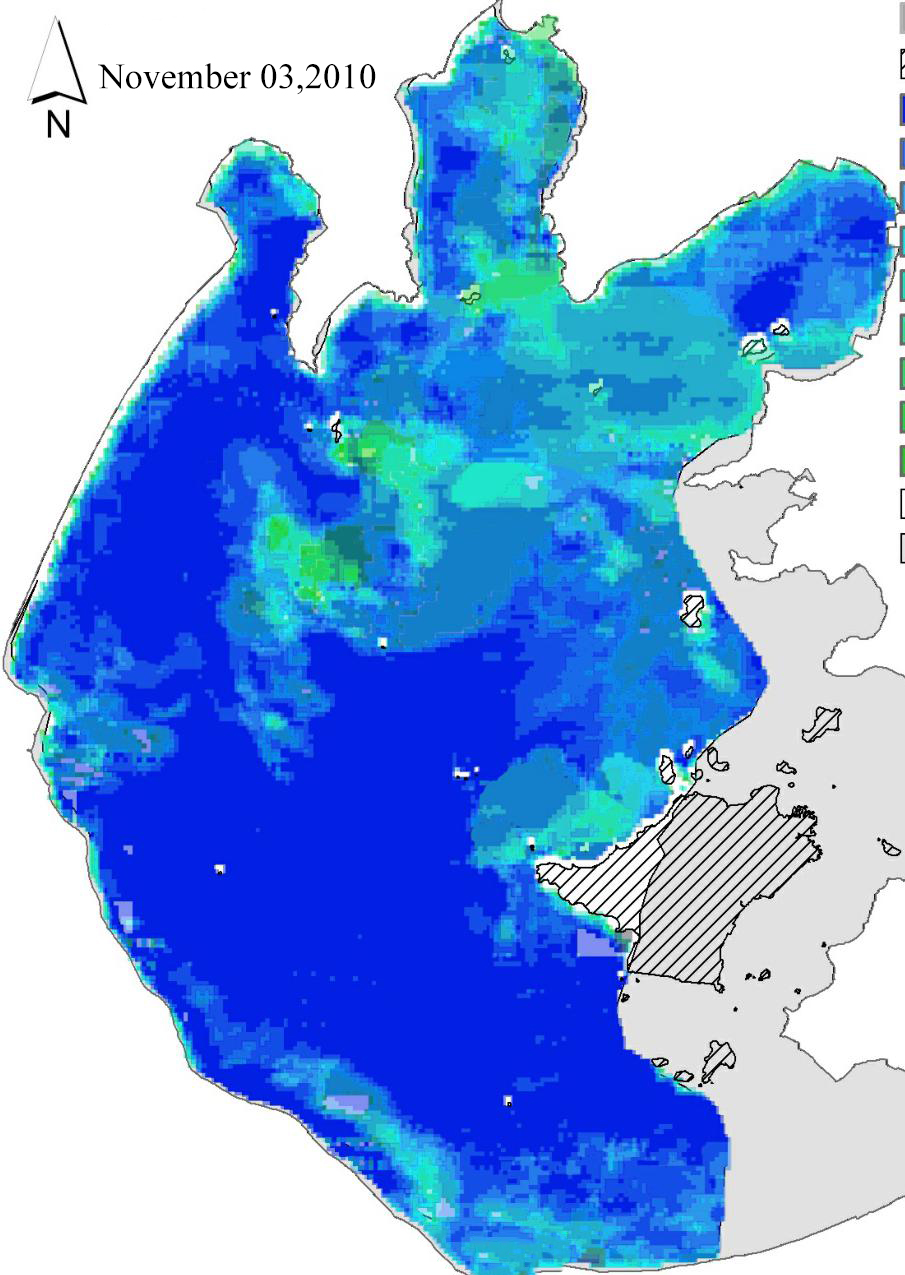

Supplement: Supplemental Information 11 — The data are remote sensing images of chlorophyll a concentration after data scale unification, remote sensing image repair, and time series filling. Remote sensing images of 30 consecutive moments were used as input to the 3D-GAN model. [file peerj-cs-09-1292-s011.zip › 201011030245.jpg]

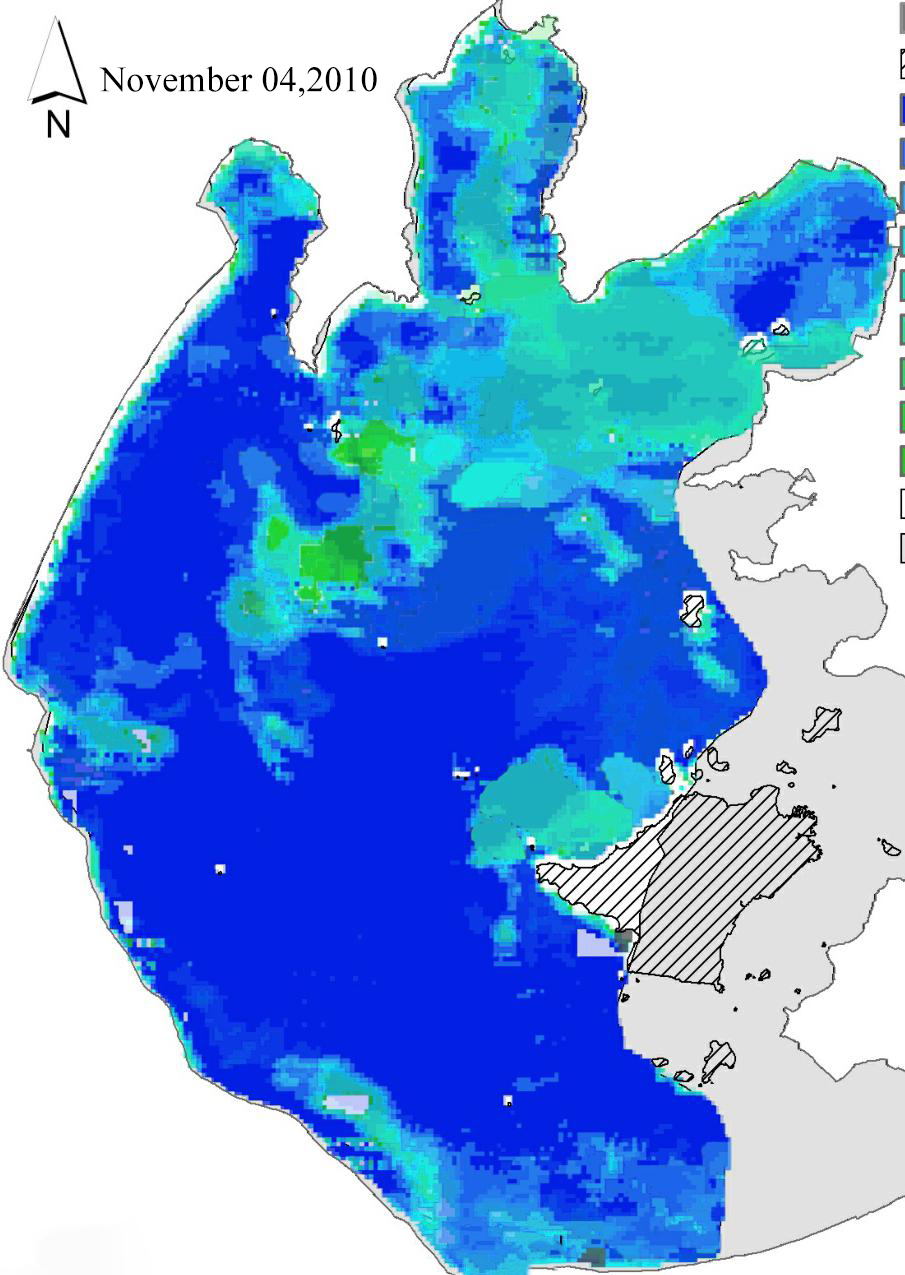

Supplement: Supplemental Information 11 — The data are remote sensing images of chlorophyll a concentration after data scale unification, remote sensing image repair, and time series filling. Remote sensing images of 30 consecutive moments were used as input to the 3D-GAN model. [file peerj-cs-09-1292-s011.zip › 201011040245.jpg]

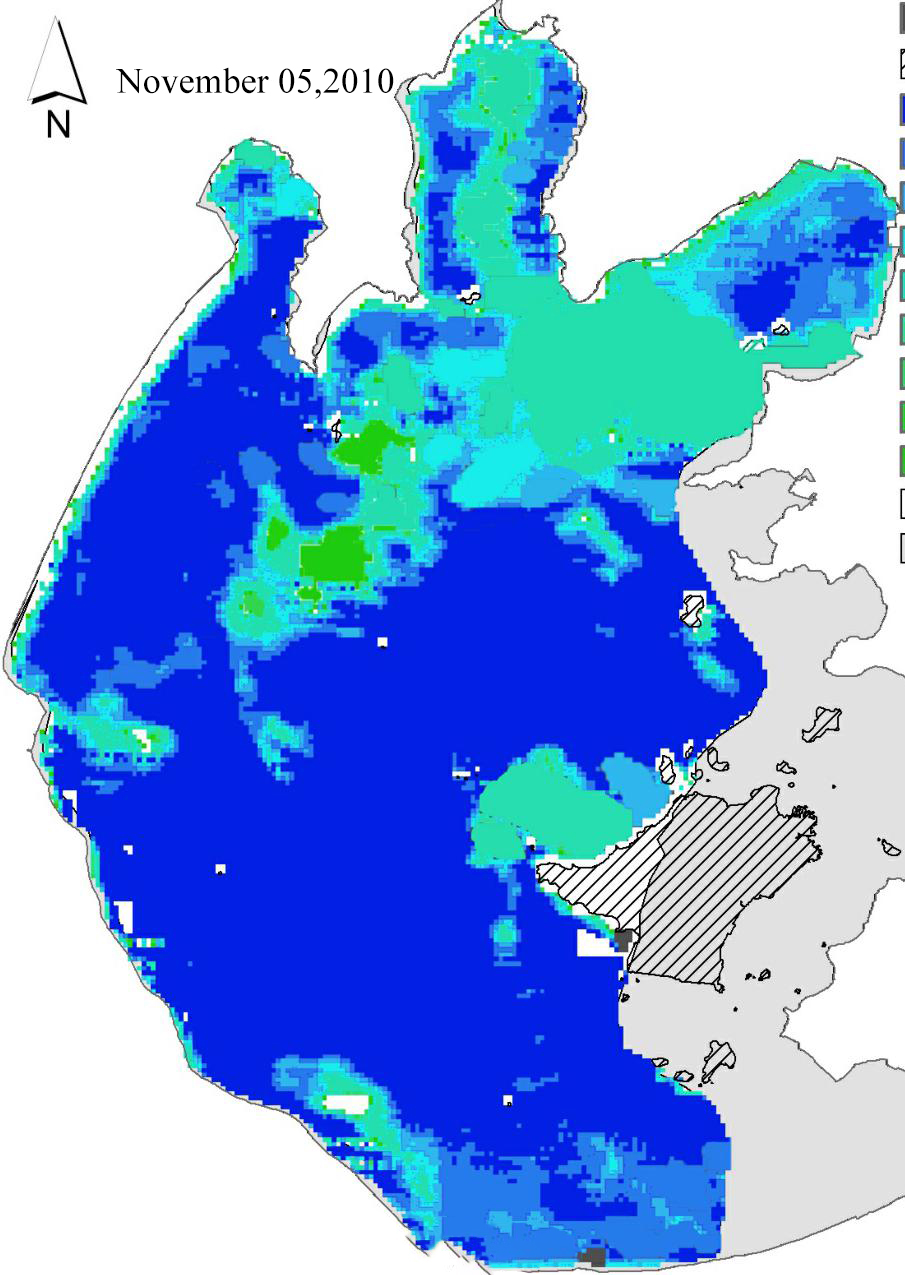

Supplement: Supplemental Information 11 — The data are remote sensing images of chlorophyll a concentration after data scale unification, remote sensing image repair, and time series filling. Remote sensing images of 30 consecutive moments were used as input to the 3D-GAN model. [file peerj-cs-09-1292-s011.zip › 201011050245.jpg]

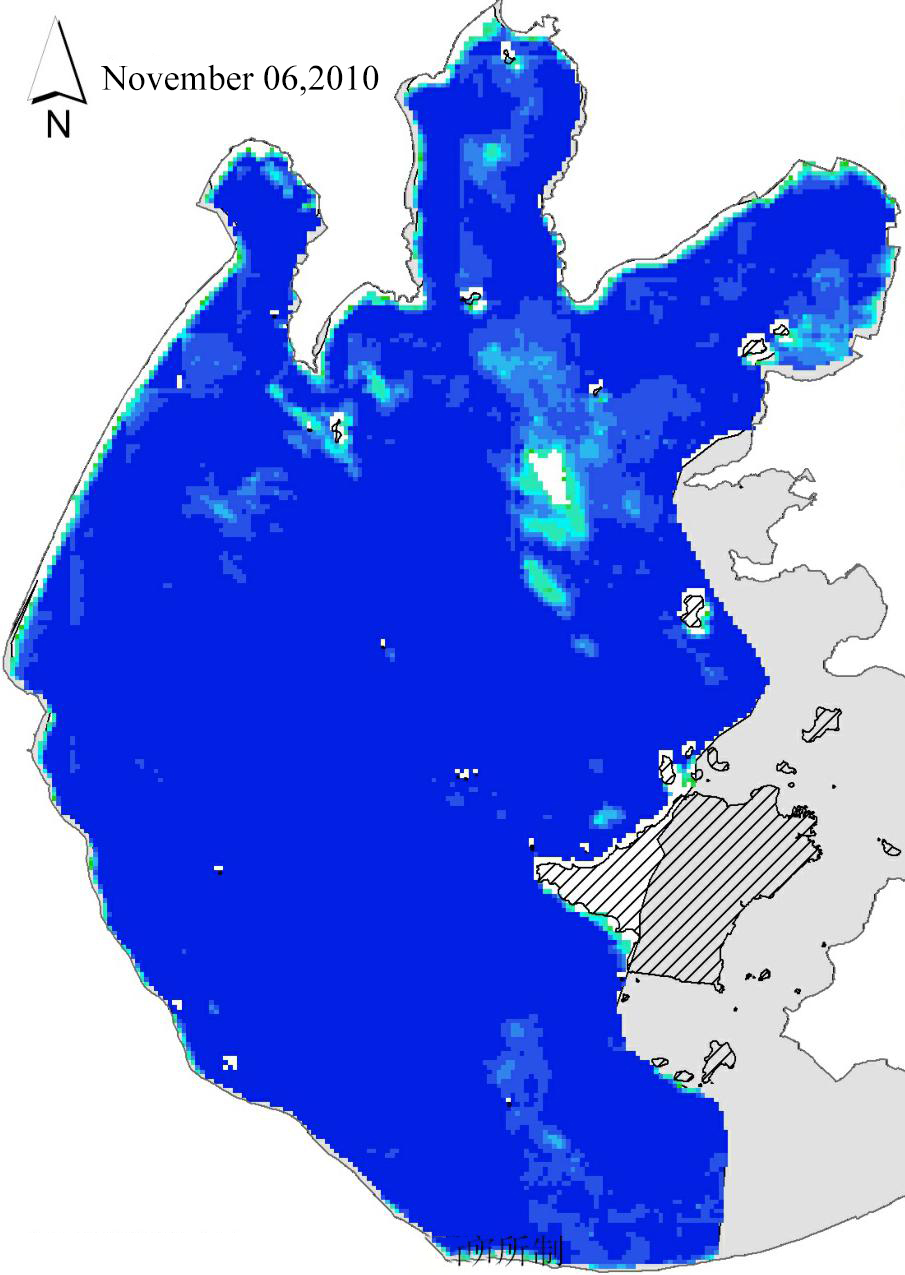

Supplement: Supplemental Information 11 — The data are remote sensing images of chlorophyll a concentration after data scale unification, remote sensing image repair, and time series filling. Remote sensing images of 30 consecutive moments were used as input to the 3D-GAN model. [file peerj-cs-09-1292-s011.zip › 201011060245.jpg]

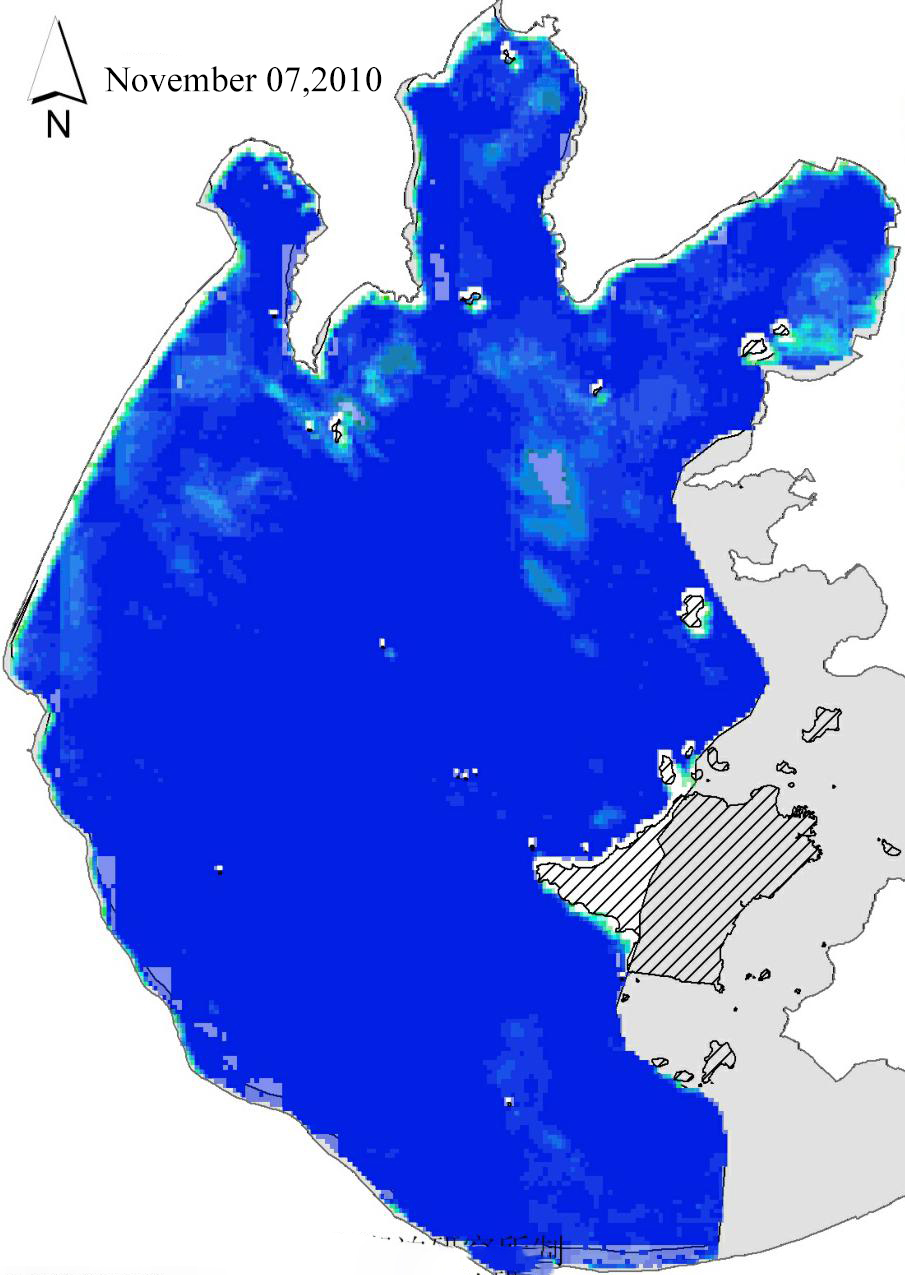

Supplement: Supplemental Information 11 — The data are remote sensing images of chlorophyll a concentration after data scale unification, remote sensing image repair, and time series filling. Remote sensing images of 30 consecutive moments were used as input to the 3D-GAN model. [file peerj-cs-09-1292-s011.zip › 201011070245.jpg]

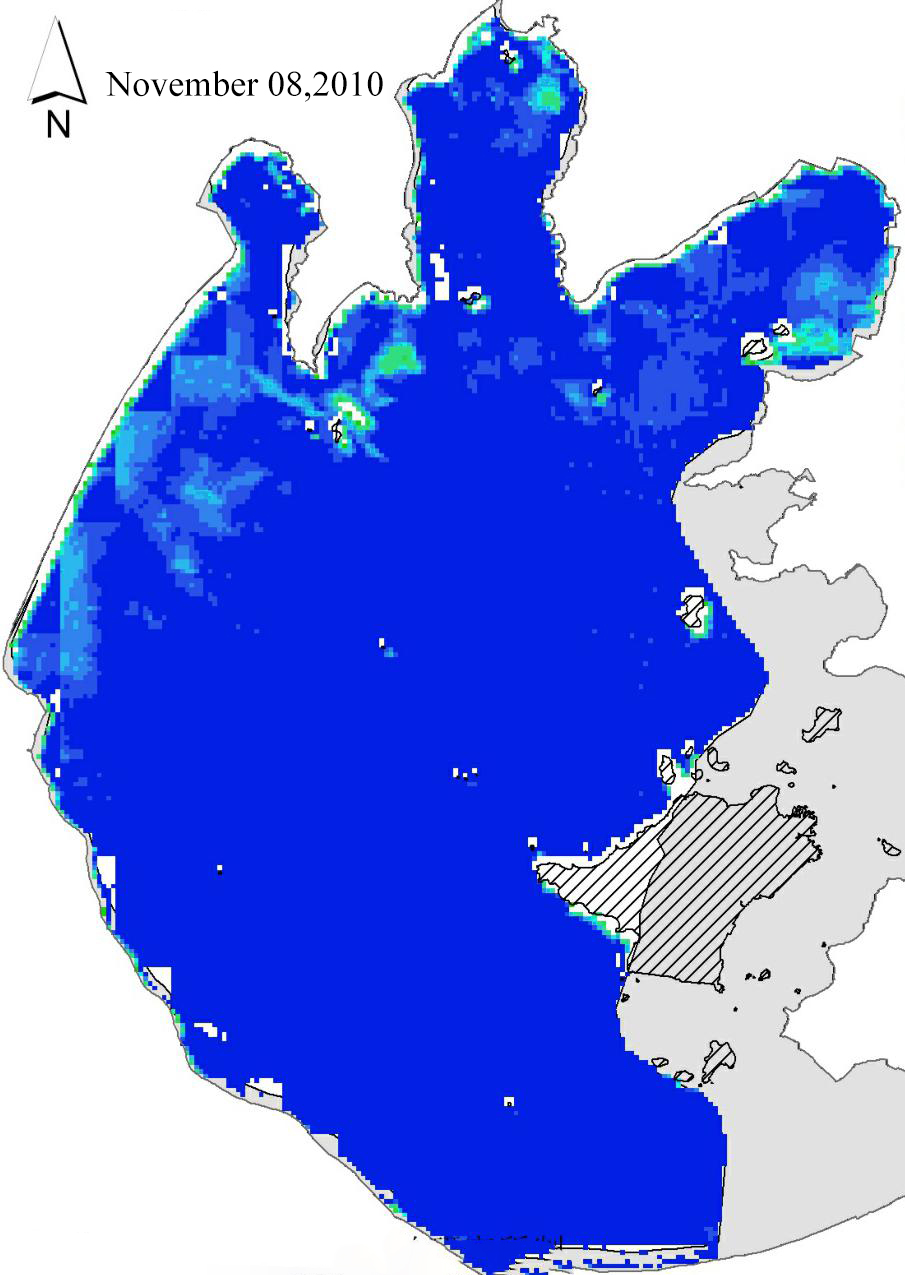

Supplement: Supplemental Information 11 — The data are remote sensing images of chlorophyll a concentration after data scale unification, remote sensing image repair, and time series filling. Remote sensing images of 30 consecutive moments were used as input to the 3D-GAN model. [file peerj-cs-09-1292-s011.zip › 201011080245.jpg]

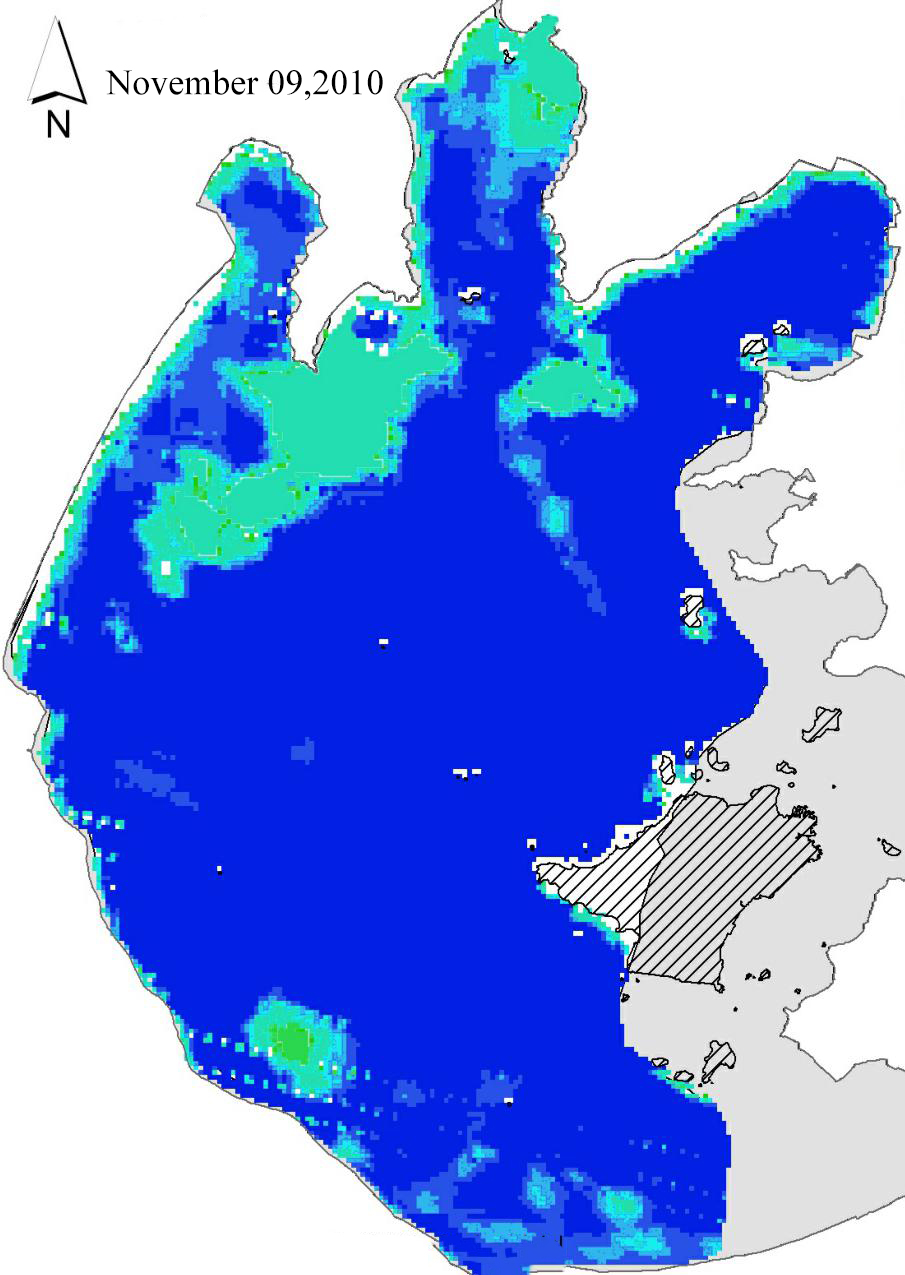

Supplement: Supplemental Information 11 — The data are remote sensing images of chlorophyll a concentration after data scale unification, remote sensing image repair, and time series filling. Remote sensing images of 30 consecutive moments were used as input to the 3D-GAN model. [file peerj-cs-09-1292-s011.zip › 201011090245.jpg]

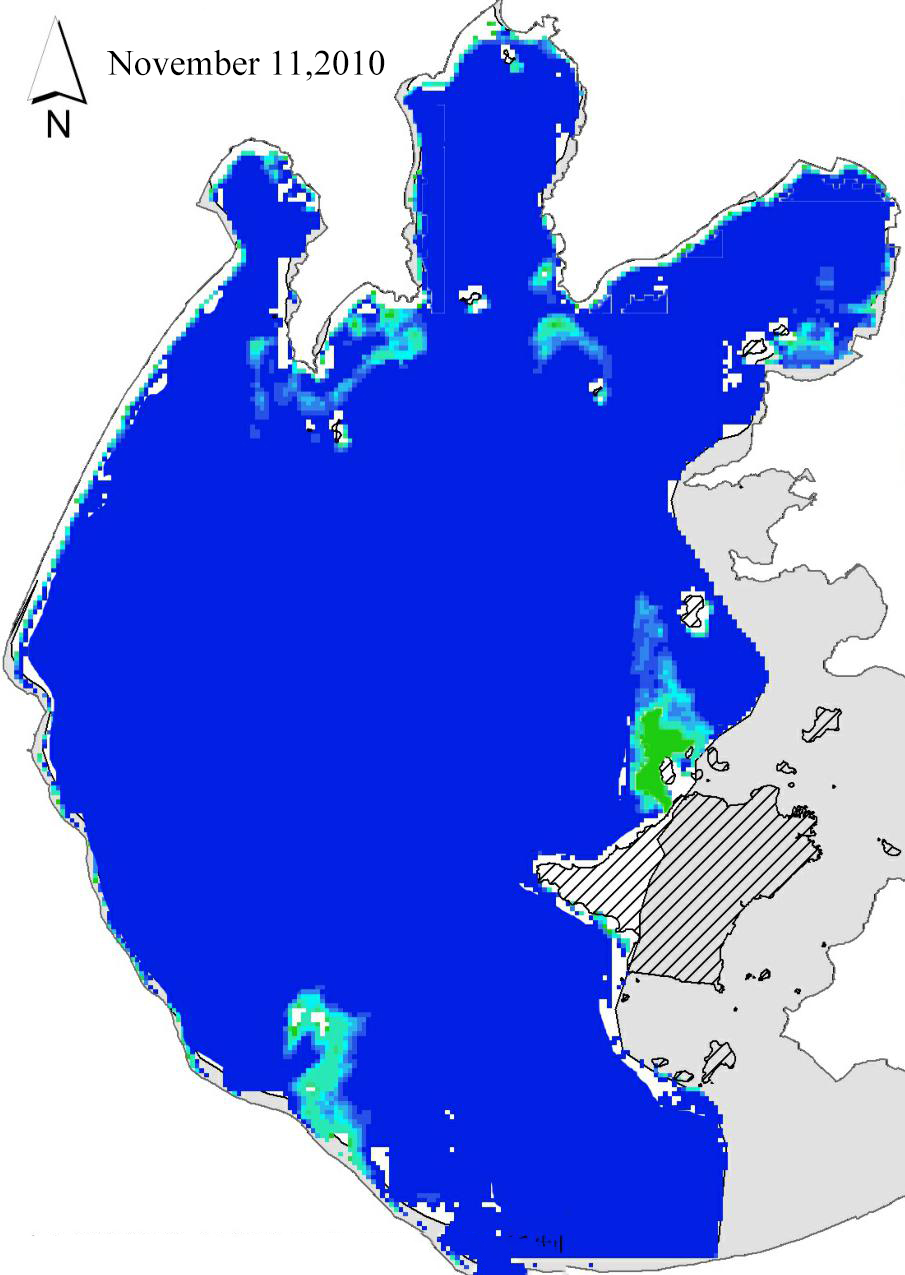

Supplement: Supplemental Information 11 — The data are remote sensing images of chlorophyll a concentration after data scale unification, remote sensing image repair, and time series filling. Remote sensing images of 30 consecutive moments were used as input to the 3D-GAN model. [file peerj-cs-09-1292-s011.zip › 201011100245.jpg]

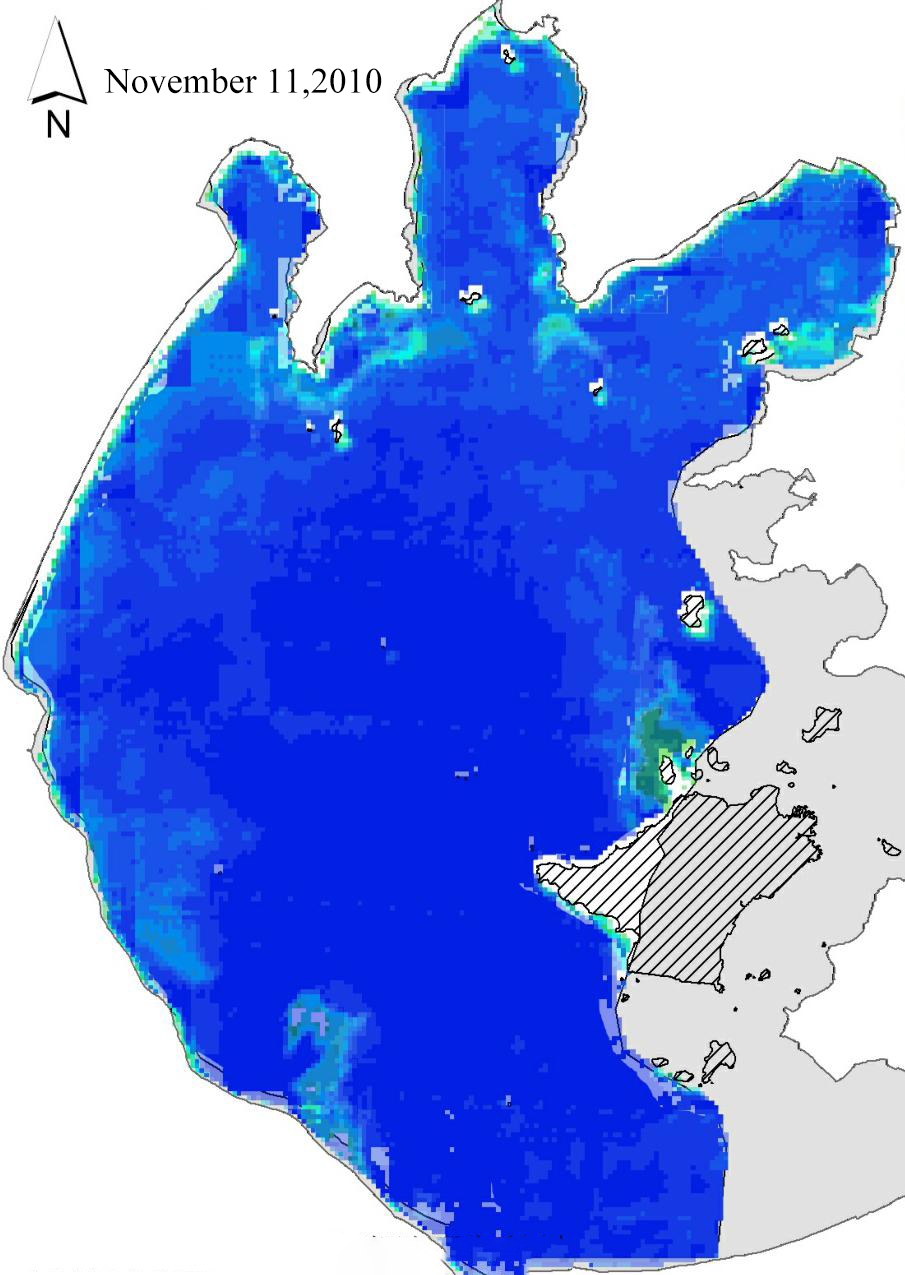

Supplement: Supplemental Information 11 — The data are remote sensing images of chlorophyll a concentration after data scale unification, remote sensing image repair, and time series filling. Remote sensing images of 30 consecutive moments were used as input to the 3D-GAN model. [file peerj-cs-09-1292-s011.zip › 201011110245.jpg]

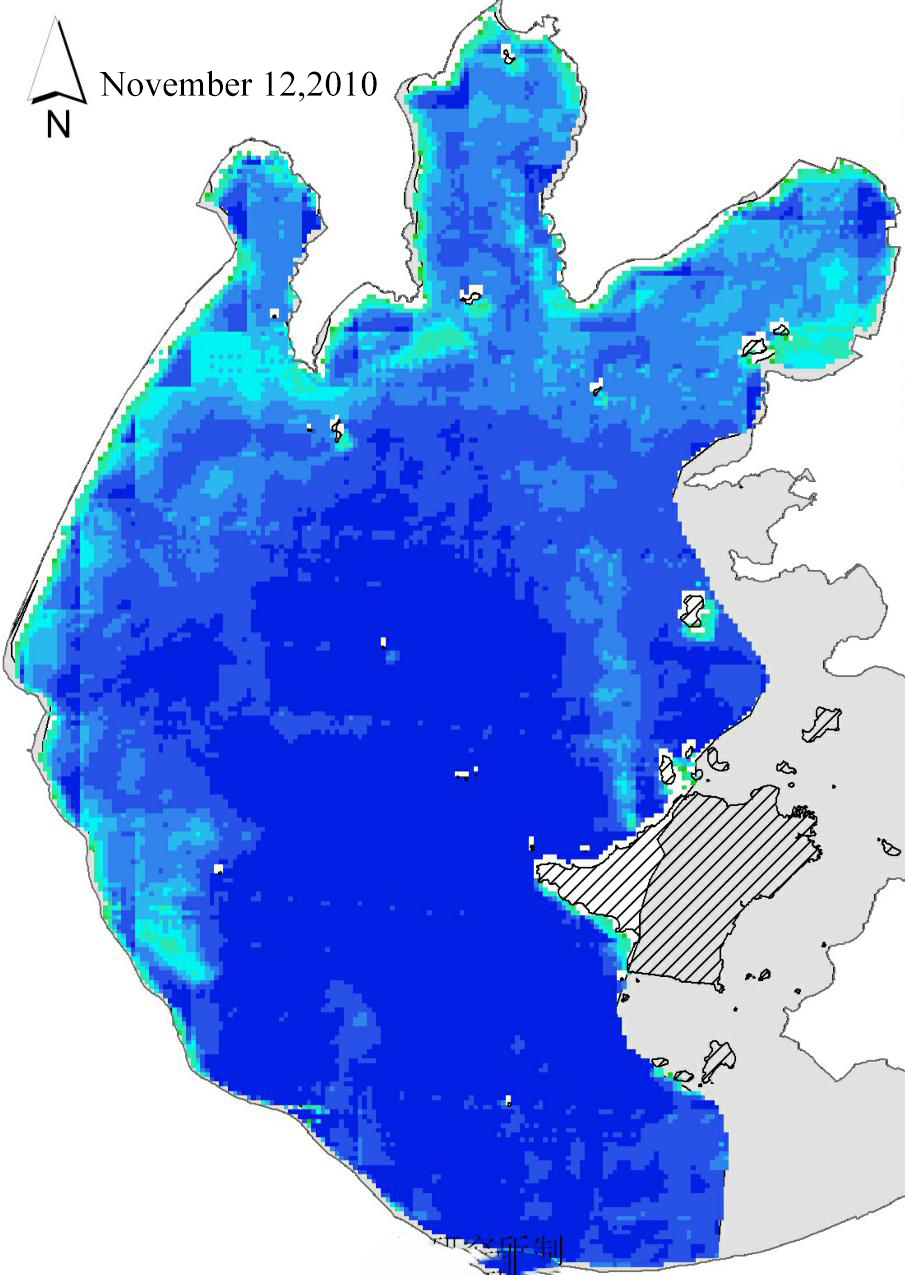

Supplement: Supplemental Information 11 — The data are remote sensing images of chlorophyll a concentration after data scale unification, remote sensing image repair, and time series filling. Remote sensing images of 30 consecutive moments were used as input to the 3D-GAN model. [file peerj-cs-09-1292-s011.zip › 201011120245.jpg]

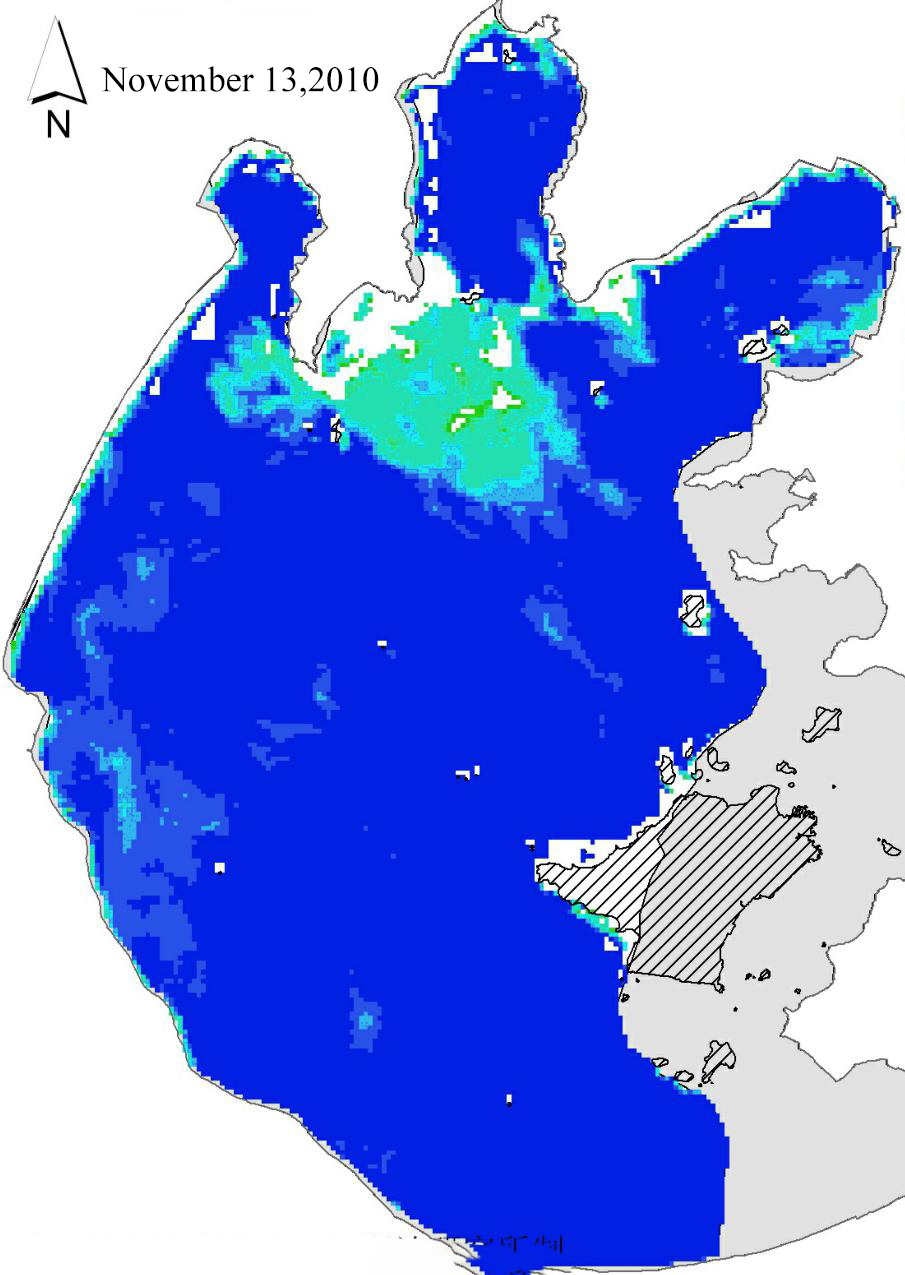

Supplement: Supplemental Information 11 — The data are remote sensing images of chlorophyll a concentration after data scale unification, remote sensing image repair, and time series filling. Remote sensing images of 30 consecutive moments were used as input to the 3D-GAN model. [file peerj-cs-09-1292-s011.zip › 201011130245.jpg]

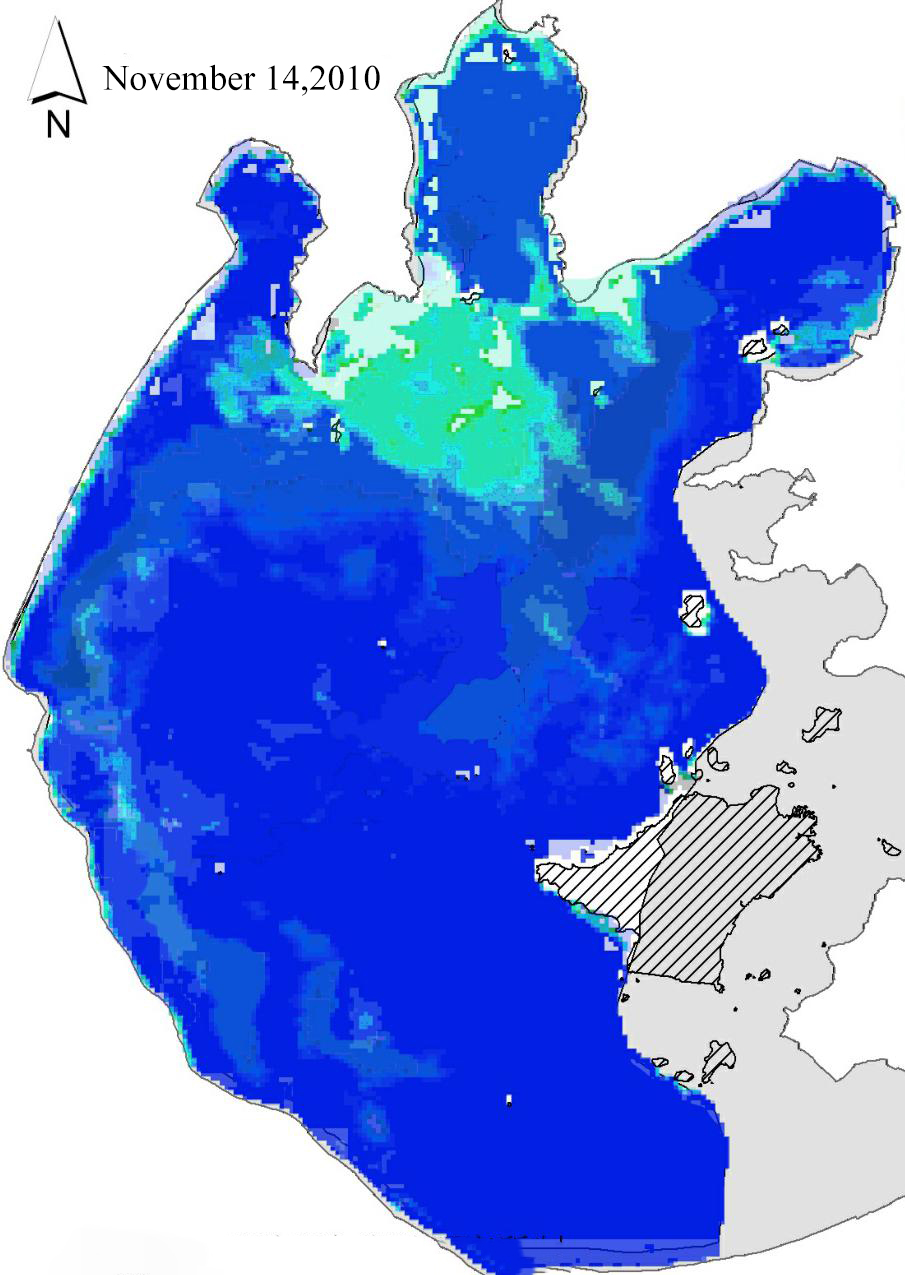

Supplement: Supplemental Information 11 — The data are remote sensing images of chlorophyll a concentration after data scale unification, remote sensing image repair, and time series filling. Remote sensing images of 30 consecutive moments were used as input to the 3D-GAN model. [file peerj-cs-09-1292-s011.zip › 201011140245.jpg]

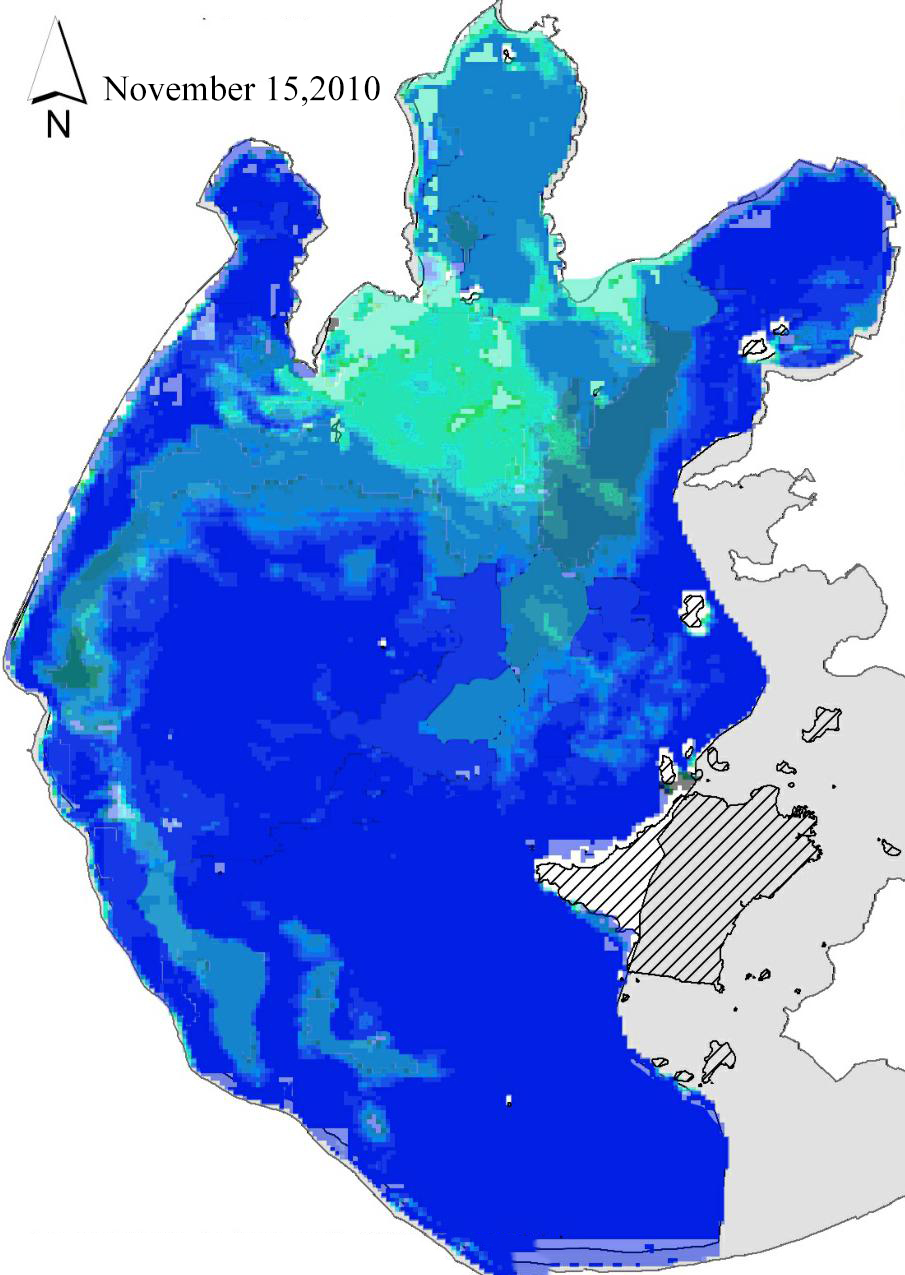

Supplement: Supplemental Information 11 — The data are remote sensing images of chlorophyll a concentration after data scale unification, remote sensing image repair, and time series filling. Remote sensing images of 30 consecutive moments were used as input to the 3D-GAN model. [file peerj-cs-09-1292-s011.zip › 201011150245.jpg]

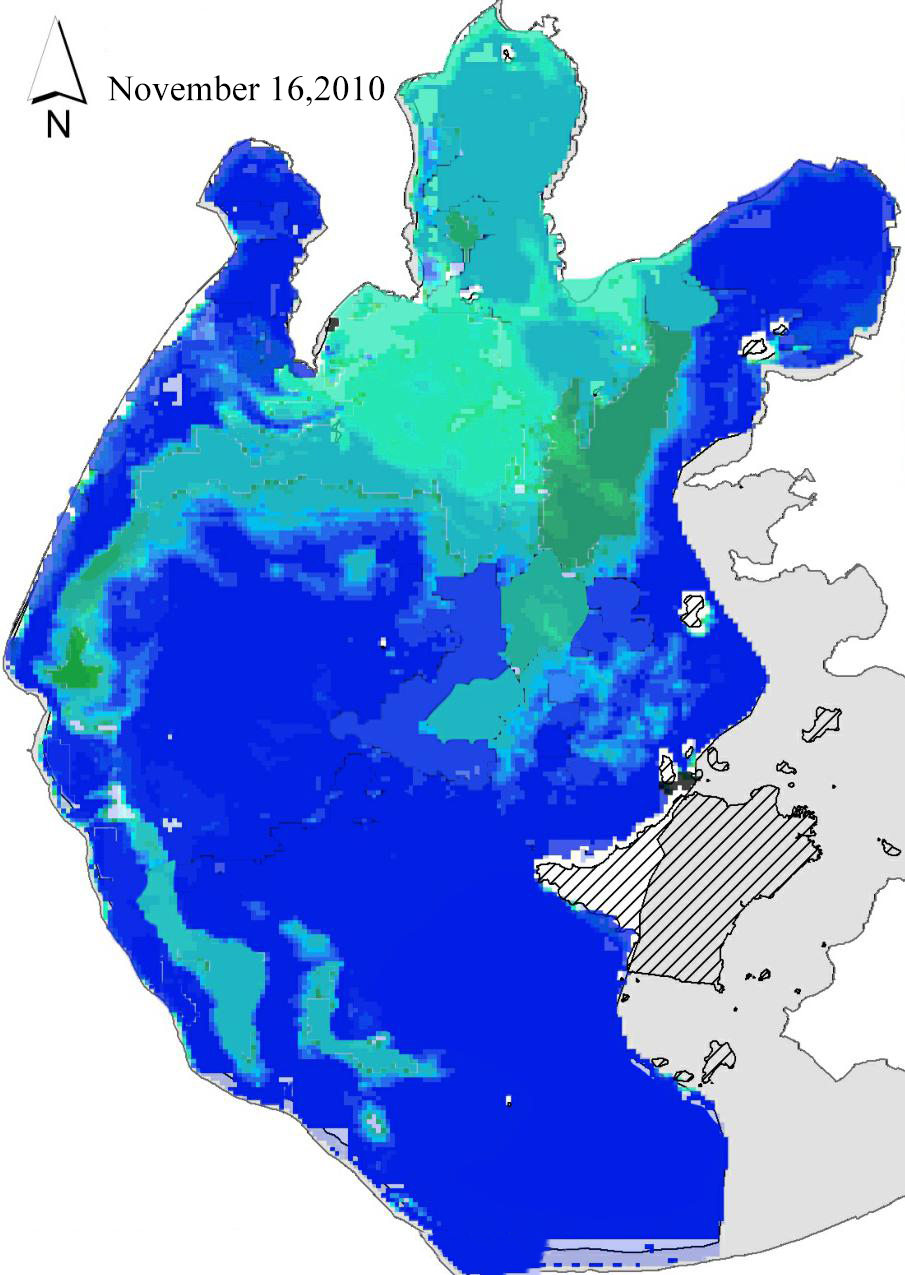

Supplement: Supplemental Information 11 — The data are remote sensing images of chlorophyll a concentration after data scale unification, remote sensing image repair, and time series filling. Remote sensing images of 30 consecutive moments were used as input to the 3D-GAN model. [file peerj-cs-09-1292-s011.zip › 201011160245.jpg]

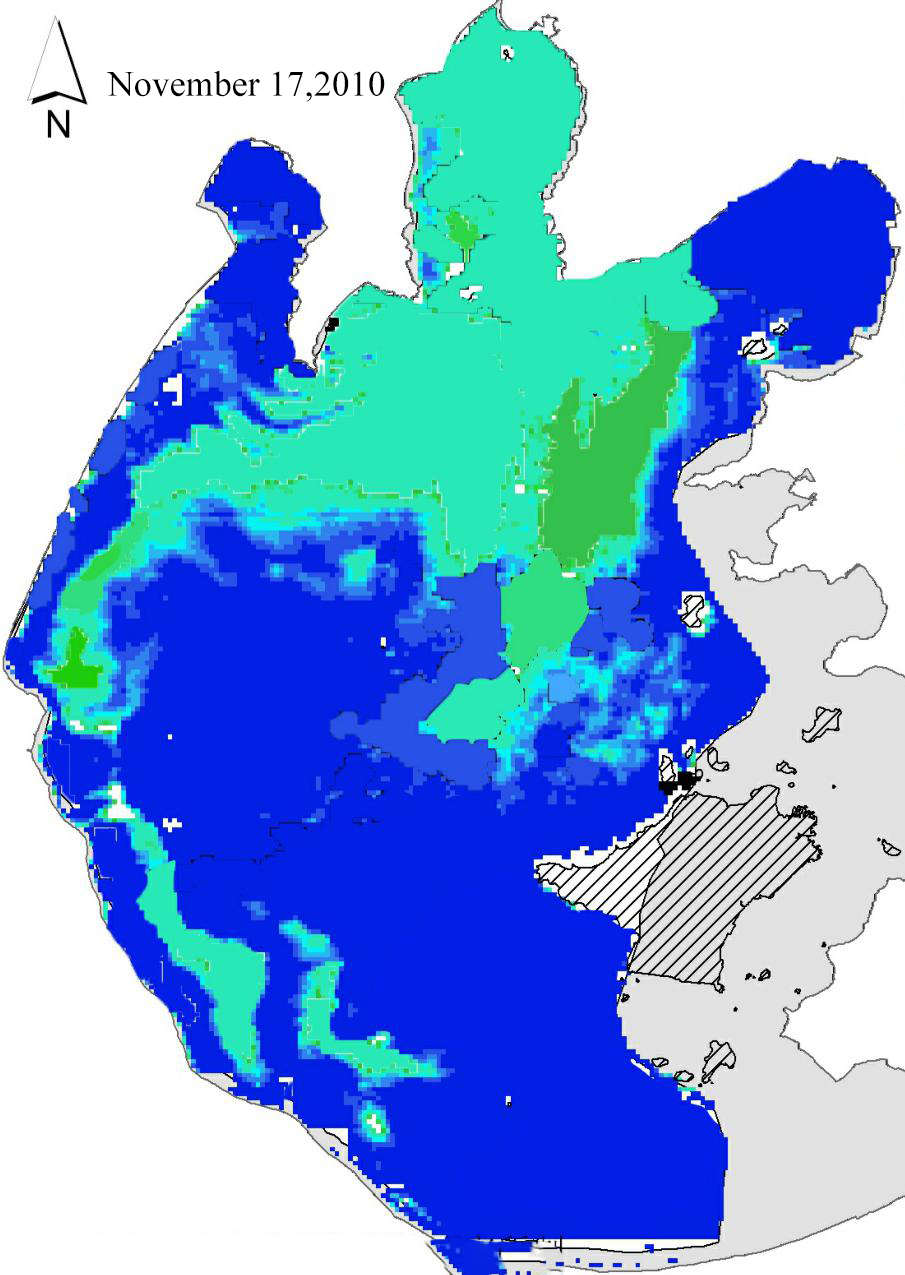

Supplement: Supplemental Information 11 — The data are remote sensing images of chlorophyll a concentration after data scale unification, remote sensing image repair, and time series filling. Remote sensing images of 30 consecutive moments were used as input to the 3D-GAN model. [file peerj-cs-09-1292-s011.zip › 201011170245.jpg]

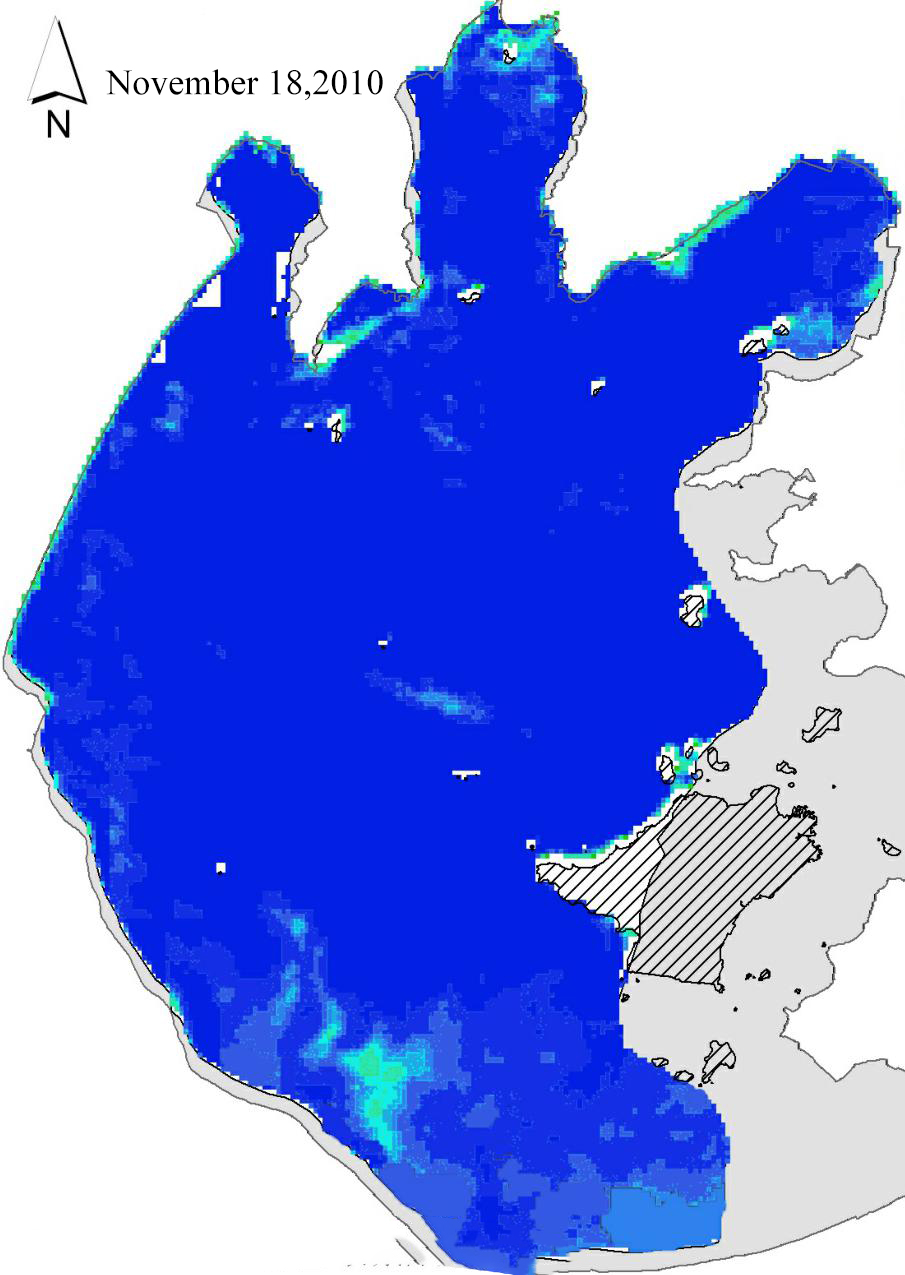

Supplement: Supplemental Information 11 — The data are remote sensing images of chlorophyll a concentration after data scale unification, remote sensing image repair, and time series filling. Remote sensing images of 30 consecutive moments were used as input to the 3D-GAN model. [file peerj-cs-09-1292-s011.zip › 201011180245.jpg]

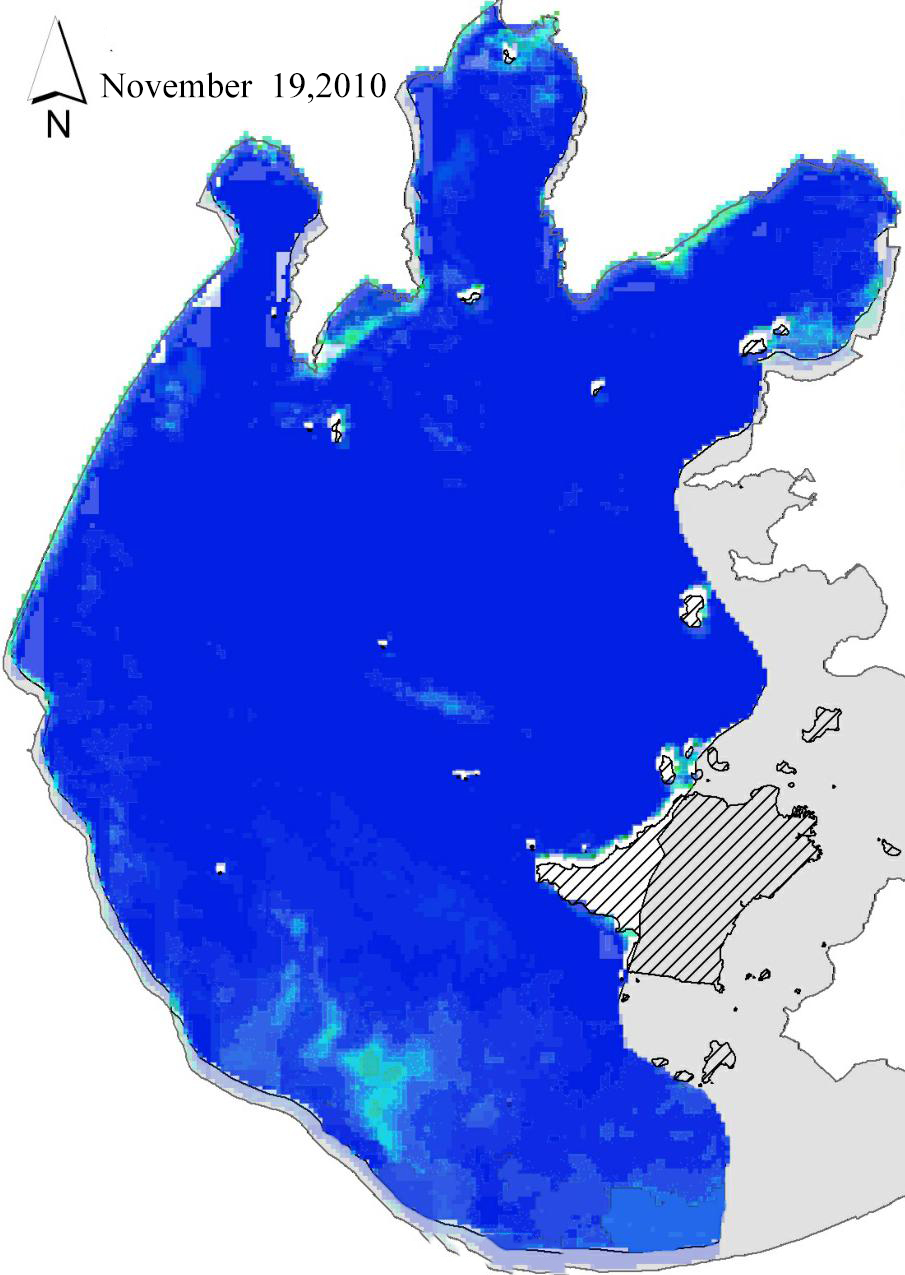

Supplement: Supplemental Information 11 — The data are remote sensing images of chlorophyll a concentration after data scale unification, remote sensing image repair, and time series filling. Remote sensing images of 30 consecutive moments were used as input to the 3D-GAN model. [file peerj-cs-09-1292-s011.zip › 201011190245.jpg]

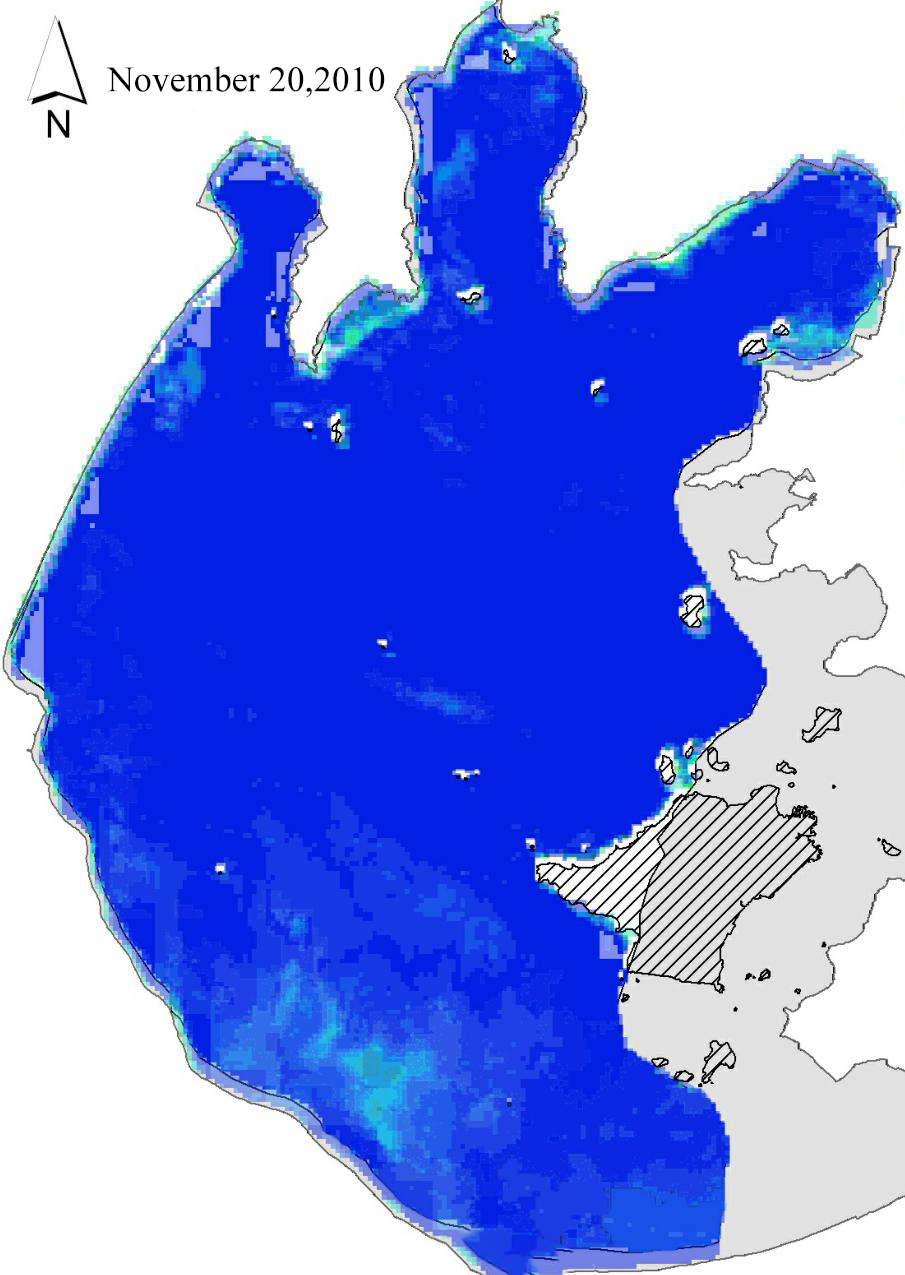

Supplement: Supplemental Information 11 — The data are remote sensing images of chlorophyll a concentration after data scale unification, remote sensing image repair, and time series filling. Remote sensing images of 30 consecutive moments were used as input to the 3D-GAN model. [file peerj-cs-09-1292-s011.zip › 201011200245.jpg]
